# Introducing SPeDE: High-Throughput Dereplication and Accurate Determination of Microbial Diversity from Matrix-Assisted Laser Desorption–Ionization Time of Flight Mass Spectrometry Data

**DOI:** 10.1128/mSystems.00437-19

**Published:** 2019-09-10

**Authors:** Charles Dumolin, Maarten Aerts, Bart Verheyde, Simon Schellaert, Tim Vandamme, Felix Van der Jeugt, Evelien De Canck, Margo Cnockaert, Anneleen D. Wieme, Ilse Cleenwerck, Jindrich Peiren, Peter Dawyndt, Peter Vandamme, Aurélien Carlier

**Affiliations:** a Laboratory of Microbiology, Department of Biochemistry and Microbiology, Faculty of Sciences, Ghent University, Ghent, Belgium; b Computational Biology Laboratory, Department of Applied Mathematics, Computer Science and Statistics, Faculty of Sciences, Ghent University, Ghent, Belgium; c BCCM/LMG Bacteria Collection, Department of Biochemistry and Microbiology, Faculty of Sciences, Ghent University, Ghent, Belgium; California State University, Northridge

**Keywords:** bioinformatics, MALDI-TOF MS, dereplication, microbial ecology

## Abstract

The isolation of microorganisms from microbial community samples often yields a large number of conspecific isolates. Increasing the diversity covered by an isolate collection entails the implementation of methods and protocols to minimize the number of redundant isolates. Matrix-assisted laser desorption–ionization time-of-flight (MALDI-TOF) mass spectrometry methods are ideally suited to this dereplication problem because of their low cost and high throughput. However, the available software tools are cumbersome and rely either on the prior development of reference databases or on global similarity analyses, which are inconvenient and offer low taxonomic resolution. We introduce SPeDE, a user-friendly spectral data analysis tool for the dereplication of MALDI-TOF mass spectra. Rather than relying on global similarity approaches to classify spectra, SPeDE determines the number of unique spectral features by a mix of global and local peak comparisons. This approach allows the identification of a set of nonredundant spectra linked to operational isolation units. We evaluated SPeDE on a data set of 5,228 spectra representing 167 bacterial strains belonging to 132 genera across six phyla and on a data set of 312 spectra of 78 strains measured before and after lyophilization and subculturing. SPeDE was able to dereplicate with high efficiency by identifying redundant spectra while retrieving reference spectra for all strains in a sample. SPeDE can identify distinguishing features between spectra, and its performance exceeds that of established methods in speed and precision. SPeDE is open source under the MIT license and is available from https://github.com/LM-UGent/SPeDE.

**IMPORTANCE** Estimation of the operational isolation units present in a MALDI-TOF mass spectral data set involves an essential dereplication step to identify redundant spectra in a rapid manner and without sacrificing biological resolution. We describe SPeDE, a new algorithm which facilitates culture-dependent clinical or environmental studies. SPeDE enables the rapid analysis and dereplication of isolates, a critical feature when long-term storage of cultures is limited or not feasible. We show that SPeDE can efficiently identify sets of similar spectra at the level of the species or strain, exceeding the taxonomic resolution of other methods. The high-throughput capacity, speed, and low cost of MALDI-TOF mass spectrometry and SPeDE dereplication over traditional gene marker-based sequencing approaches should facilitate adoption of the culturomics approach to bacterial isolation campaigns.

## Abstract (precis)

Estimation of the operational isolation units present in a MALDI-TOF mass spectral data set involves an essential dereplication step to identify redundant spectra in a rapid manner and without sacrificing biological resolution. We describe SPeDE, a new algorithm which facilitates culture-dependent clinical or environmental studies. SPeDE enables the rapid analysis and dereplication of isolates, a critical feature when long-term storage of cultures is limited or not feasible. We show that SPeDE can efficiently identify sets of similar spectra at the level of the species or strain, exceeding the taxonomic resolution of other methods. The high-throughput capacity, speed, and low cost of MALDI-TOF mass spectrometry and SPeDE dereplication over traditional gene marker-based sequencing approaches should facilitate adoption of the culturomics approach to bacterial isolation campaigns.

## INTRODUCTION

The composition and functioning of the microbiome have been linked to the development of diseases and the performance of industrial processes and have recently emerged as key drivers of agricultural yields and plant health (1–5). Describing microbial diversity is key to understanding niche functioning and is now routinely carried out using culture-independent techniques, such as shotgun or amplicon metagenomics. These techniques allow the determination of the relative abundances of operational taxonomic units (OTUs) and the comparison of large cohorts (6). Metagenomic analyses on environmental samples further confirmed the so-called great plate count anomaly, which is the observation that many microbes seen under the microscope escape cultivation under laboratory conditions (7). Uncultivated bacteria can now be identified and genomes can be reconstructed, allowing the formulation of hypotheses regarding microbe-environment, microbe-microbe, and microbe-host interactions. While these systems biology approaches can be powerful, they are limited in scope and the discovery of fundamentally new processes requires cultures (8). Furthermore, minor microbiota components may have a substantial influence and are not easily captured by culture-independent methods (9). Recently, renewed interest in microbial diversity has led to the development of new methods to overcome the shortcomings of conventional cultivation techniques (10, 11).

High-throughput culture-based methods, also called culturomics, aim to recover the diversity of cultivable microorganisms present in a sample (12). In a landmark study, Lagier and colleagues cultured more than 900,000 isolates from the human gut (13). These included over 77% of all prokaryotes previously identified in the human gut, in addition to organisms corresponding to previously unassigned OTUs (13). However, the application of high-throughput cultivation techniques leads to the isolation of multiple conspecific strains or genetically identical clones, inflating the downstream cost of analyses. To control cost, various dereplication methods can be implemented. These typically involve typing and filtering out conspecifics based on 16S rRNA gene sequence or matrix-assisted laser desorption–ionization time-of-flight (MALDI-TOF) mass spectrometry (MS) profiles (14–16). MALDI-TOF MS has proven to be a fast and cost-effective method for dereplication and identification when coupled to a profile database with extensive taxonomic coverage of a sample, with as little as 0.1% of isolates from the gut microbiota further requiring identification via 16S rRNA gene sequencing (17). Furthermore, recent technical and technological advances have enabled MALDI-TOF MS profiling of bacteria at the strain level (18), indicating a taxonomic resolution potentially exceeding that of 16S rRNA sequencing for some taxa. However, commercial MALDI-TOF MS databases are mostly populated with spectra for isolates of clinical or food safety relevance, which makes identification coupled to dereplication impractical for environmental samples containing a high diversity of rarely sampled bacteria (19). Retroactively updating the database with spectra from newly cultured isolates is possible but time-consuming, compromising the throughput of the culturomics approach. In addition, extending commercial databases may involve lengthy clearance procedures by regulatory agencies, such as the U.S. Food and Drug Administration.

Methods based on global similarity measures combined with hierarchical clustering of mass spectra have been developed to uncouple dereplication from identification (15, 20). A shortcoming of this strategy is that the identification of redundant profiles relies either on visual inspection of dendrograms or on the use of a predefined distance cutoff value to delineate clusters of similar spectra (21, 22). Predefined cutoff values do not consider the variability of profiles between taxa and thus need to be adjusted according to the taxonomic composition of the samples. Both shortcomings limit the taxonomic resolution and require user intervention, which contribute to making dereplication analysis time-consuming and subject to reproducibility issues. To our knowledge, no fast and user-friendly tool has been developed to dereplicate large sets of spectral data obtained from culturomics studies without relying on prior identification.

We introduce SPeDE, an algorithm and software implementation enabling high-throughput isolate dereplication using comparison of MALDI-TOF MS profiles. SPeDE discriminates MS spectra through the detection of unique spectral features with adjustable sensitivity. We validated the program with a data set of more than 5,000 spectra obtained from 167 different strains belonging to 132 genera across six phyla, for which we also obtained whole-genome sequences.

## RESULTS

### Rationale and purpose of SPeDE.

We designed SPeDE to identify isolates from recurrent taxa in culture collections in a time- and cost-efficient manner. MALDI-TOF MS spectra are compared in a pairwise manner using local and global measures. SPeDE does not attempt to classify the samples according to a reference database but instead computes the number of exclusive spectral features between pairs of spectra. Pairs of spectra for which all features of one element are shared with the other are considered redundant and grouped in single operational isolation units (OIUs) represented by a reference spectrum. Program parameters affect the sensitivity at which discriminating features are detected, allowing various degrees of taxonomic resolution.

### Optimization of similarity threshold parameters for the identification of nonredundant spectra.

**FIG 1:**
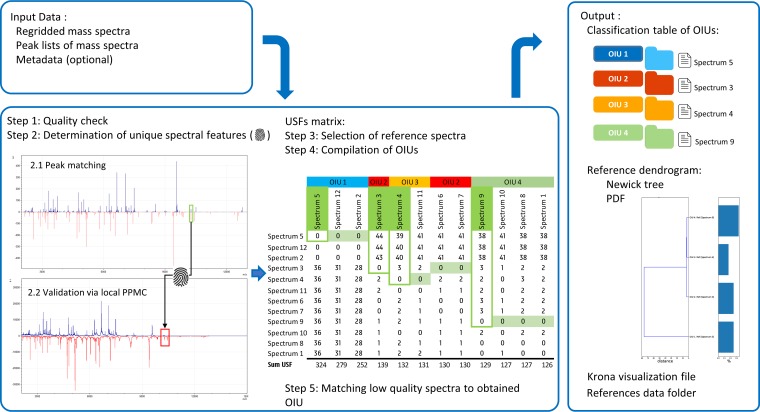
Schematic representation of the SPeDE algorithm. See Materials and Methods for a detailed description of the algorithm. Briefly, all possible pairs of peak lists in a data set are compared (step 2.1). Peaks which are not shared by a pair of spectra are validated by estimating the Pearson product moment correlation (PPMC) between raw spectra in a local area surrounding the peak (step 2.2). Peaks with a PPMC below a threshold value are considered discriminating. The number of discriminating peaks or unique spectral features (USFs) between pairs of spectra is computed and tabulated (step 3). Pairs of spectra for which no USFs are found in at least one of the elements are matched and clustered into operational isolation units (OIUs; step 4). All spectra with a quality too poor to be considered for inclusion as a reference spectrum are matched to an OIU to give a reliable abundance estimate for each OIU. The output of SPeDE includes a table of representative spectra for each OIU and a USF distance matrix between all spectra which can be used to generate a dendrogram or a Krona plot.

The SPeDE algorithm relies on peak matching coupled to spectrum similarity in a focused area around the peaks to determine unique spectral features (USFs). For details of the algorithm, see the Materials and Methods section and Fig. 1. For each pair of spectra, peaks are matched if they fall within a predetermined peak accuracy window, calculated by the *m/z* difference between peak centroids. Peaks matched and peaks considered to be unique for one of the spectra are validated by calculating the Pearson product moment correlation (PPMC) between raw spectra in a local area around each peak. Spectral features are considered unique if the PPMC value is below a preset threshold. This step allows us to detect peaks which may have been missed by the peak-calling algorithm with high sensitivity, reducing the risk of erroneously identifying discriminating features. Both the size of the peak accuracy window and the PPMC value threshold are expected to affect the outcome of peak matching and, by extension, the outcome of the dereplication process.

To determine optimal values for the PPMC threshold and peak accuracy window, we applied SPeDE to a benchmark data set of 167 strains covering 132 genera and 143 species. These included several species of *Burkholderia* and *Lactobacillus*, which are notoriously difficult to discriminate by mass spectrometry (23–25). To account for closely related strains in the estimation of true- and false-positive spectral matches, we divided the data set into 149 OTUs, which we defined as groups of strains with an intragroup pairwise genome-wide average nucleotide identity (ANI) of >98%. This ANI threshold was empirically determined to give the widest precision range in response to SPeDE parameter changes. We obtained 19 to 32 spectra for each strain, for a total of 5,228 spectra. A quality assessment routine built in the SPeDE algorithm rejected 28 spectra as poor quality, as they contained less than 5 peaks with a signal-to-noise ratio (S/N) of >30. The total number of spectra used for benchmarking of the algorithm was thus 5,200.

Overall, varying the size of the peak accuracy window from 500 to 1,000 ppm had a negligible impact on the dereplication ratio (taken as the ratio of the number of OTUs/number of reference spectra) or on the precision, i.e., the ability of the algorithm to correctly match the spectra to an OTU (see Fig. S1 in the supplemental material). Varying the local PPMC threshold value had the most impact on overall performance. Increasing the local PPMC threshold values resulted in higher precision, but this came at the cost of lower dereplication ratios (Fig. S1). Gains in precision were incremental only for PPMC threshold values of >50%, rising from 95.3% to a maximum of 99.8%. In contrast, the dereplication ratio dipped rapidly from 70.5% to below 50% at local PPMC threshold values of 50% and 66%, respectively (Fig. S1A and B).

10.1128/mSystems.00437-19.1 FIG S1 Impact of local similarity parameters on SPeDE performance. For the calculation of precision (A and C), spectra of good quality matched to the reference spectrum of a strain with an ANI above the threshold were considered true positives; sample spectra of good quality matched to the reference spectrum from a strain with an ANI below the threshold were considered false positives (see the main text for details). The dereplication ratio was calculated as the number of OTUs in the sample (i.e., groups of strains with an ANI above the threshold) divided by the number of reference spectra. See the main text for details on the benchmarking set. (A) Precision as a function of the local PPMC threshold (in percent) for an ANI threshold of 98%. (B) Dereplication ratio as a function of the local PPMC threshold (ANI threshold = 98%). (C) Precision as a function of the local PPMC threshold (in percent) for an ANI threshold of 95%. (D) Dereplication ratio as a function of the local PPMC threshold (ANI threshold = 95%). (E) Precision as a function of the peak accuracy window (in parts per million). (F) Dereplication ratio as a function of the peak accuracy window (ANI threshold = 98%). The blue dashed lines indicate the default values of the SPeDE algorithm. Download FIG S1, PDF file, 0.2 MB. Copyright © 2019 Dumolin et al. 2019 Dumolin et al. This content is distributed under the terms of the Creative Commons Attribution 4.0 International license.

Therefore, we selected a local accuracy window of 700 ppm and a local PPMC threshold of 50% as the default parameter settings which offered the best compromise between precision (95.3%) and dereplication (70.5%).

We were also interested in determining optimal parameter values for the clustering of conspecific strains in a data set. We repeated our parameter search, this time defining an OTU_95%_ as a group of strains with an intragroup pairwise genome-wide average nucleotide identity (ANI) of >95%, a commonly accepted threshold for the circumscription of taxonomic species (26, 27) (Fig. S1C and D). With this definition, our benchmarking data set contained 150 OTU_95%_ values, each corresponding to a biological species. Precision values reached a plateau for local PPMC threshold values of 50 and above (precision > 99.5%), corresponding to dereplication ratios of <68.5%. In contrast, the dereplication ratio decreased rapidly at local PPMC threshold values of >20, with precision remaining >97.8%. We thus recommend setting the local PPMC threshold parameter value at 20 if dereplication at the species level is desired.

### Performance and taxonomic resolution of SPeDE.

**FIG 2:**
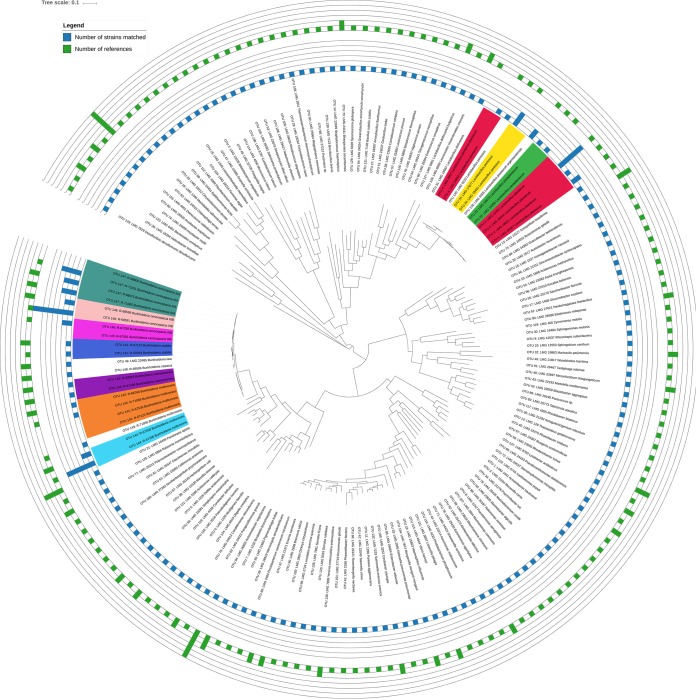
Approximate maximum likelihood phylogenetic tree of strains included in the benchmark data set based on 40 single-copy, conserved marker protein genes. OTUs were defined as groups of strains with an intragroup pairwise genome-wide ANI of >98%. OTU clusters containing more than one strain are highlighted. The number of the references obtained per strain are indicated by green bars, and the number of strains linked to each reference are indicated by blue bars.

**FIG 3:**
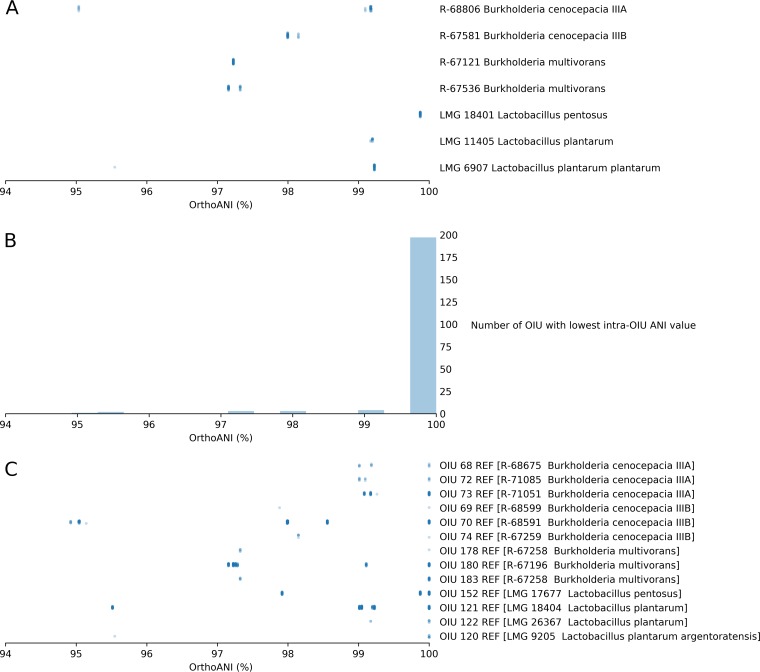
Accuracy of MALDI-TOF mass spectra matching by SPeDE. (A) Genomic similarity of the 7 strains of the benchmark data set for which no reference spectrum was retained to strains within the same OIU. Genomic similarities expressed by ANI values are shown. (B) Distribution of the lowest ANI values within 210 OIUs. Bins have a width of 0.36%. (C) Genomic similarity within OIUs composed of more than one strain. Each data point corresponds to the ANI value between a pair of strains contained within the OIU.

Our benchmark data set contained 5,200 MALDI-TOF mass spectra passing the SPeDE quality control. These mass spectra represented 167 strains. Dereplication analysis of these spectra with a PPMC threshold of 50% and an accuracy window width of 700 ppm resulted in 210 distinct clusters of spectra, or OIUs. A representative spectrum was picked for each cluster (see Materials and Methods for details), which we refer to here as the reference spectrum (Fig. 2; Table S1). This corresponded to a reduction of 96.0% of the spectra analyzed. Moreover, 160/167 (95.8%) of the strains were represented by reference spectra. In total, 123 (73.7%) of all strains were represented by a single reference spectrum, 31 (18.6%) were represented by two reference spectra, and 3 (1.8%) were represented by three reference spectra. Only seven strains did not yield any reference spectra. Furthermore, obtaining multiple reference spectra for a single strain did not seem to be dependent on the phylogenetic placement of the taxon (Fig. 2). The number of USFs between the spectra of distinct strains was always higher than the number of USFs between the reference spectra originating from the same strain (Fig. S2). To determine the resolution at which SPeDE was able to discriminate between strains, we calculated the genomic distance between distinct strains whose spectra were matched to a single reference (Fig. 3A). Overall, spectra from the seven strains which were not represented in the final set matched the reference spectra from strains with an average pairwise ANI of 98.4% ± 1.1%. Finally, over 93.8% of OIUs represented a single strain (Fig. 3B). Only 13/210 OIUs included multiple strains; of these, 10 had minimal intra-OIU ANI values of >97% and 3 had minimal intra-OIU ANI values of >94.9% (Fig. 3C). We were thus able to reduce a complex data set of over 5,000 experimentally acquired spectra to a final set of 210 OIUs without a significant loss of diversity.

10.1128/mSystems.00437-19.2 FIG S2 Dendrogram of references obtained from the benchmarking strain set. The dendrogram was derived from the unweighted pair group method with arithmetic mean (UPGMA), with levels of linkage being expressed as the relative distance in USFs observed. The distance metric was calculated as one minus the ratio of the number of USFs observed between samples and the maximal number of USFs observed between all references obtained. (Right) Percentage of spectra matched to the reference (abundance OIU). Download FIG S2, PDF file, 0.3 MB. Copyright © 2019 Dumolin et al. 2019 Dumolin et al. This content is distributed under the terms of the Creative Commons Attribution 4.0 International license.

10.1128/mSystems.00437-19.5 TABLE S1 Results of SPeDE analysis of the benchmarking data set. Download Table S1, PDF file, 0.6 MB. Copyright © 2019 Dumolin et al. 2019 Dumolin et al. This content is distributed under the terms of the Creative Commons Attribution 4.0 International license.

### Robustness of SPeDE dereplication to biological sample variation on a set of closely related strains.

MALDI-TOF MS measurements can be sensitive to various experimental factors, such as instrument calibration, but also to the physiological state of the cultures (28). To test the sensitivity of MALDI-TOF MS spectrum dereplication to experimental artifacts introduced during culturing in a high-throughput work flow, we generated MALDI-TOF MS spectra from at least 5 independent cultures for each of 25 strains of Lactobacillus brevis in our culture collection. Dereplication using randomly selected spectra from only one culture per strain (50 spectra in total) yielded on average 22.25 reference spectra, while adding spectra from 5 cultures (250 spectra) resulted in 31.29 reference spectra (Fig. S3). These data indicate that SPeDE is only moderately sensitive to biological or technical variation.

10.1128/mSystems.00437-19.3 FIG S3 Number of reference spectra obtained after dereplication when including spectra from 1, 2, 3, 4, or 5 independent cultures for 25 strains of Lactobacillus brevis. Samples were prepared from 5 or 6 cultures each of 25 strains of Lactobacillus brevis and analyzed by MALDI-TOF MS. Each sample was spotted 4 times, and MS spectra were acquired as detailed in Materials and Methods. A total of 548 spectra were processed by SPeDE using default settings (local PPMC threshold = 50%, peak accuracy window = 700 ppm). For each SPeDE run, spectra corresponding to *n* cultures were randomly sampled without replacement, with each culture being represented by 2 randomly chosen technical replicate spectra. For *n *equal to* *1, two spectra from a randomly chosen culture were selected for each strain and analyzed (50 spectra in total), for *n *equal to* *2, spectra from 2 randomly chosen cultures per strain were analyzed (100 spectra in total), etc. Each analysis was repeated 100 times for each value of *n*. Download FIG S3, PDF file, 0.06 MB. Copyright © 2019 Dumolin et al. 2019 Dumolin et al. This content is distributed under the terms of the Creative Commons Attribution 4.0 International license.

### Performance of SPeDE dereplication to sample variation.

To determine if dereplication analysis by SPeDE is robust under conditions representative of those of an isolation campaign which aims at dereplicating spectra of conspecific isolates, we generated MALDI-TOF mass spectra for 78 strains of the BCCM/LMG Bacteria Collection, representing 8 genera and 47 species, from samples taken before and after lyophilization and subculturing. Each measurement was done in duplicate (2 spots per extract), for a final set consisting of 312 spectra (Table S2). This set contained spectra from 4 species of *Enterococcus*, 4 species of *Klebsiella*, 13 species of *Lactobacillus*, 2 species of *Lactococcus*, 6 species of *Leuconostoc*, 6 species of *Streptococcus*, 3 species of *Weissella*, and 9 species of *Xanthomonas*. To account for intraspecies variability, this set also included spectra for several strains of most species (Table S5).

10.1128/mSystems.00437-19.6 TABLE S2 Results for the lyophilization data set analyzed by SPeDE. Download Table S2, PDF file, 0.3 MB. Copyright © 2019 Dumolin et al. 2019 Dumolin et al. This content is distributed under the terms of the Creative Commons Attribution 4.0 International license.

10.1128/mSystems.00437-19.9 TABLE S5 Strains included in the benchmark, lyophilization, and Lactobacillus brevis data set used in this study. Download Table S5, PDF file, 0.2 MB. Copyright © 2019 Dumolin et al. 2019 Dumolin et al. This content is distributed under the terms of the Creative Commons Attribution 4.0 International license.

Dereplication analysis by SPeDE using a PPMC threshold value of 50% yielded 97 reference spectra representing 72 strains. In total, 48 strains were represented by a single reference spectrum and 24 were represented by 2 reference spectra. Six strains did not yield reference spectra, but in all cases, the spectra were matched to reference spectra of the same species. These included 1 strain of Streptococcus salivarius, 2 strains of Lactobacillus rhamnosus, 2 strains of Weissella confusa, and 1 strain of Enterococcus faecalis. Of the 24 strains which yielded two reference spectra, 13 yielded spectra which clustered by condition (i.e., before or after lyophilization). For the remaining 11 cases, 3 spectra from either condition were matched to a single reference and the remaining spectrum was retained as a singleton reference. Hierarchical clustering of the reference spectra based on the number of USFs grouped all the spectra from a given strain together (Fig. S4), indicating that small spectral variations are responsible for the moderate decay in the dereplication ratio. Importantly, no spectra were matched to references outside species boundaries, highlighting the capacity of SPeDE to reliably discriminate spectra at the species level or lower.

10.1128/mSystems.00437-19.4 FIG S4 Dendrogram of references derived from the SPeDE analysis of the lyophilization data set. The dendrogram was derived from the unweighted pair group method with arithmetic mean (UPGMA), with the levels of linkage being expressed as the relative distance in USFs observed. The distance metric was calculated as one minus the ratio of the number of USFs observed between samples and the maximal number of USFs observed between all references obtained. (Right) Percentage of spectra matched to the reference (abundance OIU). Download FIG S4, PDF file, 0.2 MB. Copyright © 2019 Dumolin et al. 2019 Dumolin et al. This content is distributed under the terms of the Creative Commons Attribution 4.0 International license.

### Performance of SPeDE dereplication compared to other methods.

We compared the outcome of SPeDE dereplication for the benchmark data set to that of previously described methods based on (i) the global Pearson product moment correlation and the unweighted pair group method with arithmetic mean (UPGMA) clustering proposed by Ghyselinck and colleagues (21) and (ii) the cosine similarity/UPGMA clustering described by Strejcek and colleagues (22). The results are given in Table S3 and Table S4, respectively. The method of Ghyselinck et al. (21) returned the most OIUs, retrieved the least diversity from the benchmark data set, and had the overall longest processing time (Table 1). The method of Strejcek and colleagues (22), based on a proposed cosine similarity cutoff of 92%, was the best performing in terms of overall dereplication ratio, with a reduction of 97% of the spectra for the benchmark data set. However, the analysis also returned 15 OIUs which included multiple genetically distinct strains, with 1 OIU merging strains sharing less than 92% genome-wide ANI (Table 1). SPeDE was the second-best performer in terms of the dereplication ratio but was the most sensitive, retrieving the most diversity from the sample (149/149 OTUs) and returning the fewest OIUs containing multiple strains. SPeDE was also the fastest method in terms of both the run time and the manual data processing required before data analysis (Table 1).

**TABLE 1:** SPeDE accuracy compared to PPMC/UPGMA and cosine/UPGMA clustering^*a*^

Method	No. of OIUs	No. of strains retained	Reduction rate (%)	% of redundant references/cluster	OTU coverage	Lowest ANI within an OIU	No. of OIUs with multiple OTUs	Total analysis time (h)^*b*^
SPeDE	210	160	96	23.8	149	94.92	13	2
94.5% PPMC/UPGMA^*c*^	337	143	93.4	51	147	77.33	15	8
92% cosine/UPGMA^*d*^	152	152	97	0	145	91.89	5	2–3

a The benchmark data set consisted of 5,228 spectra, 167 strains, and 149 OTUs. PPMC/UPGMA, Pearson product moment correlation/unweighted pair group method with arithmetic mean; OIU, operational isolation unit; OTU, operational taxonomic unit (ANI > 98%).

b Including data import, data processing, and interpretation of results.

c Method as described in Ghyselinck et al. (21).

d Method as described in Strejcek et al. (22).

10.1128/mSystems.00437-19.7 TABLE S3 Results for the benchmark data set analyzed by the methods of Ghyselinck et al. (21). Download Table S3, PDF file, 3.0 MB. Copyright © 2019 Dumolin et al. 2019 Dumolin et al. This content is distributed under the terms of the Creative Commons Attribution 4.0 International license.

10.1128/mSystems.00437-19.8 TABLE S4 Results for the benchmark data set analyzed by the methods of Strejcek et al. (22). Download Table S4, PDF file, 2.9 MB. Copyright © 2019 Dumolin et al. 2019 Dumolin et al. This content is distributed under the terms of the Creative Commons Attribution 4.0 International license.

## DISCUSSION

We developed SPeDE, a fast, accurate, and low-memory program and algorithm for processing large spectral data sets. This is accomplished by identifying unique features of MALDI-TOF MS spectra instead of relying on global similarity measures. The algorithm groups spectra into OIUs and outputs a reference spectrum for each OIU. We optimized and validated the algorithm on a set of 5,228 spectra representing 167 strains belonging to 132 genera across six phyla and obtained 210 OIUs. SPeDE can accurately assign the MALDI-TOF mass spectra of bacterial strains sharing a minimum of 98% genome-wide average nucleotide identity. Dereplication using SPeDE yields a number of OIUs comparable to the number generated by previously published methods and is more accurate (21, 22). More relevant for an application in culturomics, SPeDE was also able to recover the most diversity from a sample.

One of the major limitations of MALDI-TOF MS analysis is the variability of measurements. We first tested SPeDE on a set of spectra obtained from multiple cultures of a large set of Lactobacillus brevis strains. SPeDE dereplication resulted in a small increase in the number of reference spectra with an increasing number of cultures included in the data set (Fig. S3). These results show that small artifacts introduced during culturing, sample preparation, and/or data acquisition have an impact on the efficiency of SPeDE. However, this impact is small: increasing the number of reference spectra from 22.5 on average when considering only spectra representing a single culture (50 spectra), to 31.29 for spectra representing 5 independent cultures (250 spectra).

To be useful in the frame of large-scale culturomics experiments, MALDI-TOF MS dereplication must also be robust to the sample variation introduced by the storage of cultures. We thus tested SPeDE on the spectra of bacterial cultures before and after lyophilization and subculturing. Experimental variability affects the dereplication ratio, introducing an excess of reference spectra but without sacrificing precision. In an analysis using global similarity to dereplicate mass spectra, Strejcek and colleagues also attributed the decreased overall performance of cluster-based dereplication to biological variation, a confounding factor inherent to MALDI-TOF MS measurements (22). Possible reasons for the variability in the mass spectra of strains grown on the same medium and analyzed using the same sample preparation method are not well described, but the total incubation time of cultures appears to be a critical factor affecting reproducibility (28, 29). For most culturomics experiments, isolates would be obtained from cultures incubated under identical conditions and processed using a linear work flow, thus minimizing sample variation. Rapid analysis and dereplication are crucial when performing large-scale culturomics experiments when long-term storage of cultures is limited or not feasible. We argue that the high-throughput capacity, speed, and low cost of a dereplication pipeline built around MALDI-TOF MS and SPeDE vastly outweigh the benefits of 16S rRNA gene sequence-based dereplication techniques.

In conclusion, SPeDE is a fully automated, scalable algorithm which can be run on a single workstation. SPeDE has been designed to deal with large quantities of data like those generated in culturomics experiments and can process more than 5,000 MALDI-TOF mass spectra in less than an hour on a Linux or Windows laptop computer. SPeDE can be run from a graphical user interface or from the command line and can be fully integrated in a culturomics pipeline designed to automatically retrieve the cultivable diversity from complex samples.

## MATERIALS AND METHODS

### MALDI-TOF MS data sets.

The strains used in the benchmark data set were derived from the BCCM/LMG Bacteria Collection and the research collection of LM-UGent. In total 167 different strains were used. The bacterial set consisted of 6 major bacterial phyla (*Actinobacteria*, *Bacteroidetes*, *Deinococcus*, *Firmicutes*, *Proteobacteria*, and *Epsilonbacteraeota*) representing 16 classes and 132 genera. The strains used in the lyophilization data set to estimate the effect of biological variability were derived from the BCCM/LMG Bacteria Collection. In total, 79 strains covering four major bacterial phyla were used. The 25 strains used in the Lactobacillus brevis study were obtained from the BCCM/LMG Bacteria Collection and the research collection of LM-UGent and were isolated from 22 different isolation sources. An overview of the cultures used in all sets is given in Table S5 in the supplemental material.

### MALDI-TOF MS sample preparation and data acquisition.

**(i) Preparation of MALDI-TOF-MS samples.** The strains included in the benchmark data set were subcultured 3 times, prior to harvesting of the cell material grown under the conditions stated by the BCCM/LMG Bacteria Collection catalogue (http://bccm.belspo.be/catalogues/lmg-catalogue-search). The strains included in the lyophilization study were subcultured 3 times before and after preservation according to the protocol described by Peiren et al. (30). For the preparation of cell extracts, a 1-μl loopful of bacterial cells was suspended in 300 μl of Milli-Q water and vortexed to a homogeneous suspension. Next, 900 μl of absolute ethanol (EtOH) was added, the components were mixed by inversion, and the mixture was centrifuged for 3 min at 4°C (14,000 rpm). Samples were stored at −20°C. At the time of analysis, samples were centrifuged as described above, supernatants were discarded, and centrifugation was repeated to remove the residual EtOH, followed by air drying for 5 min at room temperature. The pellet was suspended in 40 μl of 70% formic acid in water and mixed by vortexing. Finally, 40 μl of acetonitrile was added and the mixture was vortexed. The extract was centrifuged for 2 min at 4°C (14,000 rpm) to remove the cell debris, and the supernatant was transferred to a new tube.

The strains included in the Lactobacillus brevis study were passaged twice on MRS agar medium (Oxoid, UK) and incubated at 28°C for 3 days. For each strain, six colonies were transferred to a different well of a 96-well deep-well plate containing 1 ml of MRS broth (Oxoid, UK) and subcultured after 3 days of incubation at 28°C using a Viaflo 96/384 pipetting robot (Integra, UK). For the preparation of samples, the cultures were centrifuged for 10 min at 4°C (14,000 rpm). The cell pellets were suspended in 500 μl Milli-Q water, and this process was repeated twice to remove residual medium components from the cell suspension. After the second washing step, the cell pellets were suspended in 100 μl Milli-Q water. Sample preparation methods using solvent extraction tend to yield more consistent MALDI-TOF MS spectra, but whole-cell suspensions or smears yield spectra of equivalent quality for many Gram-positive bacteria, including lactic acid bacteria (31, 32). Whole-cell suspensions were used here instead of protein extracts because they significantly reduce the sample preparation time, are more likely to be adopted in a high-throughput isolation work flow (22), and tend to introduce more variability than protein extracts.

**(ii) MALDI-TOF MS data acquisition.** Bacterial cell extracts (1 μl) of the lyophilization study samples were spotted on a target plate (Bruker Daltonik, Bremen, Germany) in duplicate, and samples of the benchmark study were spotted seven to eight times and dried in air at room temperature. The sample spot was overlaid with 1 μl of matrix solution (10 mg/ml α-cyano-4-hydroxycinnamic acid in acetonitrile-water-trifluoroacetic acid [TFA] [50:47.5:2.5]). Each target plate comprised one spot of pure matrix solution, used as a negative control, and one spot of Bacterial Test Standard (Bruker Daltonik, Bremen, Germany), used for calibration. The target plate was measured automatically on a Bruker Microflex LT/SH (lyophilization study) or Bruker Microflex LT/SH s-Smart (benchmark study) platform (Bruker Daltonik, Bremen, Germany). The target plates of the benchmark study were measured 4 times, thus obtaining a total of 28 to 32 replicate spectra for each strain. The spectra were obtained in linear, positive ion mode using FlexControl (v3.4) software according to the manufacturer’s recommended settings (Bruker Daltonik, Bremen, Germany). Each final spectrum resulted from the sum of the spectra generated at random positions to a maximum of 240 shots per spectrum.

For Lactobacillus brevis cultures, bacterial cell suspensions were spotted (1 μl) on a target plate (Applied Biosystems, USA) in 4 replicates and dried in air at room temperature. The sample spot was overlaid with 1 μl of matrix solution (5 mg/ml α-cyano-4-hydroxycinnamic acid in acetonitrile-water-TFA [50:47.5:2.5]). Each target plate comprised one spot of pure matrix solution, used as a negative control, and one spot of Bacterial Test Standard (Bruker Daltonik, Bremen, Germany), used for calibration. The target plate was measured automatically on a 4800 Plus MALDI TOF/TOF analyzer (Applied Biosystems, USA). The spectra were obtained in the linear, positive ion mode using and covered a mass range of 2 to 20 kDa. Each final spectrum resulted from the sum of the spectra generated at random positions to a maximum of 2,000 shots per spectrum. The laser intensity was set to between 4,200 and 5,700 procedure defined units (pdu). The mass spectra were retrieved as t2dfiles from the 4800 Plus MALDI TOF/TOF analyzer via the 4000 series Explorer software. Data Explorer (v4.0) software (Applied Biosystems, USA) was used to convert the t2dfiles into text files.

**(iii) Bruker Biotyper identification.** The spectra were compared to those in the Bruker MBT 7712 MSP library using MBT Compass Explorer software according to the manufacturer’s settings (Bruker Daltonics, Bremen, Germany) to verify the authenticity of the strains. The scores obtained were reported to be of high-confidence identification (score, >2.0), low-confidence identification (score, 1.70 to 1.99), and no identification possible (score, <1.70).

**(iv) Mass spectrum preprocessing.** Mass spectra were converted to text format using the FlexAnalysis batch processing tool (Bruker Daltonics, Germany). Peak lists were generated using the continuous wavelet transformation (CWT) peak detection algorithm described by Du and colleagues with the following parameter settings: a signal-to-noise ratio of 3 and a relative amplitude threshold of 0.0001 (33). The R script used to generate the peak list is available at https://github.com/LM-UGent/SPeDE/tree/master/data_preprocessing/peak_calling. To control for small variations in *m/z* axis values between runs, raw spectra were normalized to a fixed *m/z* axis, referred to as “regridding.” The weighted average of the raw spectrum data was calculated for each *m/z* value of the fixed grid. The script used to regrid the spectra is available at https://github.com/LM-UGent/SPeDE/tree/master/data_preprocessing/regridding.

### Genome sequencing, assembly, and analysis.

**(i) DNA extraction.** Genomic DNA for genome sequencing was extracted using the procedure of Gevers et al. (34), Wilson (35), or Pitcher et al. (36) or using a Maxwell 16 tissue DNA purification kit (catalog number AS1030; Promega, Madison, WI, USA) and a Maxwell 16 instrument (catalog number AS2000; Promega). Gram-positive bacterial cultures were incubated with 5 mg of lysozyme (Serva) and 40 μl mutanolysin (5,000 U/ml; Sigma) dissolved in 110 μl of TE buffer (10 mM Tris Cl, 1 mM EDTA). DNA integrity and purity were evaluated on a 1.0% (wt/vol) agarose gel and by spectrophotometric measurements at 234, 260, and 280 nm. A Quantus fluorimeter and a QuantiFluor One double-stranded DNA system (Promega) were used to measure the DNA concentration.

**(ii) Genome sequencing, assembly, and ANI calculations.** Paired-end 2 × 150-bp libraries were prepared at the Wellcome Trust Human Genome Center (Oxford, UK) using a NEBNext DNA library kit for Illumina (New England Biolabs, Ipswich, MA, USA) and sequenced on an Illumina HiSeq 4000 instrument. Sequencing reads were prepared for assembly by adapter trimming and read filtering using the Trimmomatic tool (37). Reads with phred scores below 30 were removed, and nonpaired reads were discarded. Reads were assembled using the SPAdes (v3.10.1) program (38) and kmer lengths of 21, 33, 55, 77, and 99. Short contigs (<500 bp) or contigs with an average genome coverage of <50% were removed. To rule out possible contamination or mislabeling of samples, 16S rRNA gene sequences were extracted from the assembled genomes using the Barrnap (v0.6) program (https://github.com/tseemann/barrnap) and compared to available sequences for the strain.

Pairwise average nucleotide identity (ANI) values were calculated in two steps. First, genome distance values (corresponding approximately to 1 − ANI) were calculated between all possible genome pairs using Mash (v2.0) software (39). For genome pairs with Mash distance values of <0.1, genome distances were refined by calculating ANI values using OrthoANI (v0.90) software (40). Final genome distance values are given as percent ANI, with scores below 90% corresponding to [1 − (mash distance)] × 100. A matrix of ANI values is available in Data Set S1 in the supplemental material. OTUs were determined using the genome distance between pairs of strains in the benchmarking set as 1 − ANI and performing hierarchical clustering in R using the hclust function and the ward.D2 method. The dendrogram was cut using the R function cutree with a height parameter of 2, yielding groups of strains where all intragroup pairwise ANI values were >98%. As a reference, the commonly accepted threshold for species delineation is ANI values of 95 to 96% (26).

10.1128/mSystems.00437-19.10 DATA SET S1 Average nucleotide identity values between strains of the benchmark data set. Download Data Set S1, XLS file, 0.3 MB. Copyright © 2019 Dumolin et al. 2019 Dumolin et al. This content is distributed under the terms of the Creative Commons Attribution 4.0 International license.

**(iii) Phylogenetic analyses.** Forty single-copy, conserved marker protein sequences were extracted from assembled genomes using FetchMG software (41). FetchMG automatically extracts the sequences of 40 universal gene markers which were found in a single copy in bacterial genomes and which have been used to reconstruct robust phylogenies across the tree of life (42). Core protein sequences were aligned with the Muscle program (43). Each alignment was trimmed to remove poorly aligned regions using the Trimal program (44) and concatenated into a superalignment. This superalignment was used to create an approximate maximum likelihood phylogenetic tree using FastTree (v2) software with the JTT+CAT model (45). The resulting phylogenetic tree was annotated and visualized using the iTol web service (46).

### Description of the SPeDE algorithm.

**(i) Overview.** The objective of culturomics experiments is to isolate a maximal number of distinct or new taxa. To this purpose, SPeDE is based on unicity measures of MALDI-TOF mass spectra instead of global similarity measures. The algorithm relies on peak matching coupled to spectrum similarity in an area around the peaks to determine unique spectral features (USFs). Determination of the number of unique features, or unicity, allows for a higher resolution than standard matching algorithms to differentiate between profiles of different strains or taxa. This approach also circumvents the need for extensive manual preprocessing, minimizing the risk for technical errors. The most informative spectrum from all redundant profiles (i.e., the one containing the highest number of USFs overall) is selected as the reference profile to which all other profiles are matched. Subtle peak differences (e.g., *m/z* shifts) can therefore be easily detected and can improve the discrimination of otherwise similar profiles. The overall work flow of the SPeDE algorithm is presented in Fig. 1.

**(ii) Data input.** The SPeDE algorithm processes two data files per sample: (i) a file containing the one-dimensional raw spectrum of intensities observed to a fixed *m/z* axis and (ii) a file containing the list of peaks detected in the raw spectrum, containing for each detected peak the *m/z* value and the S/N ratio. Optionally, an existing set of reference profiles may also be added for incremental dereplication scenarios. A file containing metadata information which will be automatically parsed in the output (i.e., strain information and/or MALDI-TOF MS-based identification data) can be provided.

**(iii) Data processing.** *(a) Step 1: quality control.* The quality of the samples is assessed based on the peak signal strength. Only peaks with an S/N of >30 are taken into consideration for this step. Samples are considered to be of good quality if the spectrum contains five or more peaks with an S/N of >30 (green samples). Samples containing one to four peaks with an S/N of >30 are considered to be of low quality (orange). Samples containing no peaks with an S/N of >30 are considered to be of poor quality (red). Only good-quality samples are processed in the following steps. Low-quality samples are ignored for initial processing but are matched to reference spectra in the final step (step 5).

*(b) Step 2: USFs.* Each pair of good-quality samples is compared to determine the number of unique spectral features (USFs) for each spectrum. First, a peak-based comparison is carried out and peaks are matched if they fall within a predefined peak accuracy window, calculated as the *m/z* difference (in parts per million) between peaks. Second, peaks that match and peaks that are considered to be unique for one of the spectra are validated by calculating the Pearson product moment correlation (PPMC) in a local area around each peak. This local PPMC around a pair of matched peaks is efficient in finding peak shifts yet still robust to variations in peak intensities. Three scenarios are possible after these two steps: (i) peaks that are unmatched but that have a local PPMC above a predefined threshold are considered matched, (ii) peaks that are matched but that have a local PPMC below a threshold are considered unmatched and marked as a USF, and (iii) peaks that are unmatched and that have a local PPMC below the threshold are marked as a USF. The number of USFs between each pair of samples is stored in a USF matrix. Note that, in contrast to similarity matrices, USF matrices are not symmetric.

*(c) Step 3: reference spectra.* Once all pairs of good-quality samples are compared, the USF matrix is sorted on the basis of the sum of USFs per spectrum. Spectra containing the highest number of USFs have the lowest index number. Reference spectra are then selected by iteration over the sorted USF matrix by applying the following criterion: a spectrum is a reference spectrum if and only if it has at least one USF compared to all previously evaluated spectra. This approach results in the spectrum with the highest number of USFs in all matched spectra to be chosen as the reference spectrum of an operational isolation unit (OIU). OIUs are defined as clusters of spectra which cannot be distinguished from one another and which likely represent a single operational taxonomic unit.

*(d) Step 4: OIUs.* Spectra not marked as a reference are further matched by iterating over the index of the USF matrix: a spectrum is matched to the reference spectrum with the lowest index to which the spectrum has no USF. Spectra not marked as references are matched to existing reference spectra, and all spectra matched to a given reference are considered an OIU.

*(e) Step 5: match low-quality spectra to the obtained reference spectrum.* Low-quality spectra (i.e., spectra with <5 peaks with an S/N of >30) are matched to the set of references by Dice coefficient comparison. Peaks are matched if they fall within the peak accuracy window (700 ppm), and spectra are matched to all references resulting in a Dice coefficient of >70%.

**(iv) Output.** The default output format is a CSV table matching all samples to the references. Optionally, a USF matrix can be exported. Code to generate a dendrogram based on sample distance is available in a Jupyter notebook at https://github.com/LM-UGent/SPeDE/tree/master/output_dendrogram. Input files are a USF matrix and a table containing the percentage of samples matched to the references. A bar plot shows the abundance of each OIU in the set of samples. The dendrogram can be exported in PDF and Newick formats.

**(v) Implementation and availability.** SPeDE is implemented in Python (v3) software. A graphical user interface was developed for Microsoft Windows, but the software can be run as a command line tool under Windows, Linux, or MacOS. The data analysis performed for this study was done on a Windows computer with an Intel Core i5-4210 central processing unit and 8 GB of random-access memory. An installer for installation of all required modules for Windows computers is provided at https://github.com/LM-UGent/SPeDE. The SPeDE source code is freely distributed under the MIT license and is available at https://github.com/LM-UGent/SPeDE.

### Validation on a data set of 5,228 spectra representing 167 strains and parameter optimization.

To set the optimal threshold used for the peak accuracy window and the local PPMC used to determine a USF, the benchmark data set was analyzed using local PPMC threshold values (the −l flag in the command-line version of SPeDE) ranging from 1 to 100 in increments of 2. To determine the optimal value of the peak accuracy window (the −d flag), the values tested ranged from 500 to 1,000 in increments of 25.

We counted true positives as sample spectra matched to a reference spectrum originating from a strain within the same OTU. OTUs were defined as groups of strains sharing at least 98% genome-wide ANI (see above for details on ANI calculations). False positives were recovered as sample spectra matched to a reference spectrum outside of the expected OTU. Precision was calculated as number of samples with true-positive results/(number of samples with true-positive results + number of samples with false-positive results). The dereplication ratio was determined as the number of OTUs divided by the number of observed OIUs.

### Analysis of the Lactobacillus brevis data set.

Spectra were analyzed with SPeDE with default parameters (local PPMC threshold = 50 and PPMC window = 700), including for each run 2 technical replicates per culture, randomly selected without replacement with the random.choice function of the Numpy package (47), and one or more randomly selected cultures per strain. The analysis was repeated 100 times for each condition, and results were plotted using the R package ggplot (48).

### Comparison to other dereplication methods.

To compare the performance of the SPeDE algorithm with that of conventional clustering approaches, the spectra were imported in BioNumerics (v7.6.2) software (Applied Maths, Belgium). The similarity between spectra was expressed using PPMC. UPGMA was used as a hierarchical clustering algorithm to obtain OIUs. The dendrogram was further processed by grouping branches at 94.75% similarity, as proposed by Ghyselinck and colleagues (21). Subsequently, spectra were imported in R via the MALDIquant Foreign package and analyzed according to the method described by Strejcek and colleagues (22).

### Data availability.

All mass spectrometry data and genome assemblies used in this study are available at https://doi.org/10.5281/zenodo.3066838.
