# Supplementary figures and images for: Introducing SPeDE: High-Throughput Dereplication and Accurate Determination of Microbial Diversity from Matrix-Assisted Laser Desorption–Ionization Time of Flight Mass Spectrometry Data
Source: mSystems. 2019 Sep 10;4(5):e00437-19. doi: 10.1128/mSystems.00437-19 (PMC6739102; doi:10.1128/mSystems.00437-19)

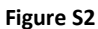

Supplement: FIG S2 [file mSystems.00437-19-sf002.pdf]

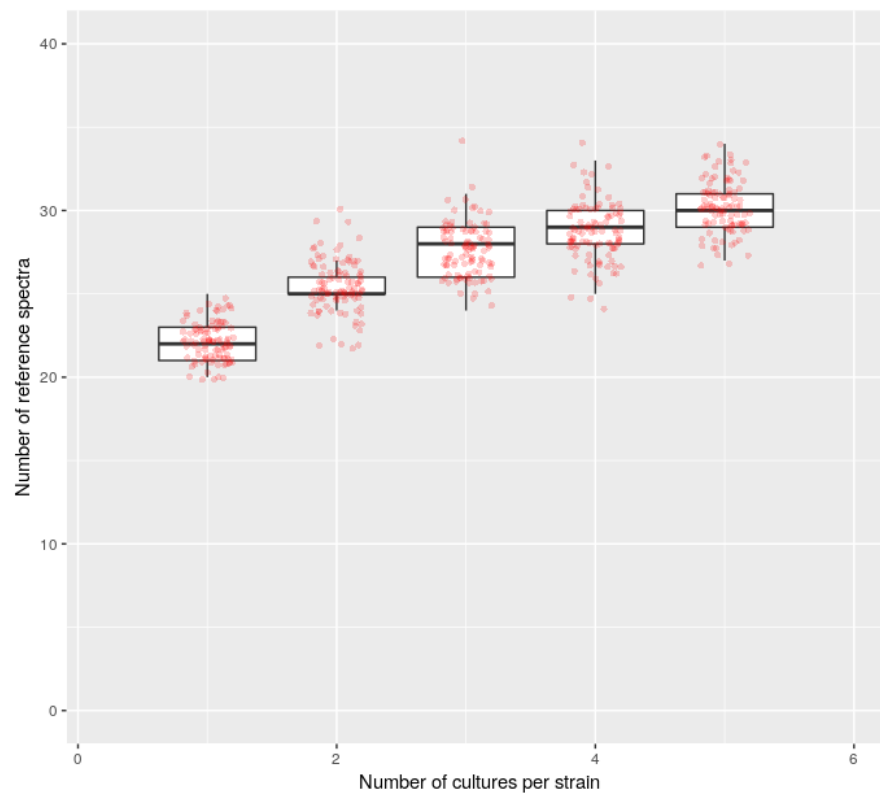

**Figure S3**

Supplement: FIG S3 [file mSystems.00437-19-sf003.pdf]

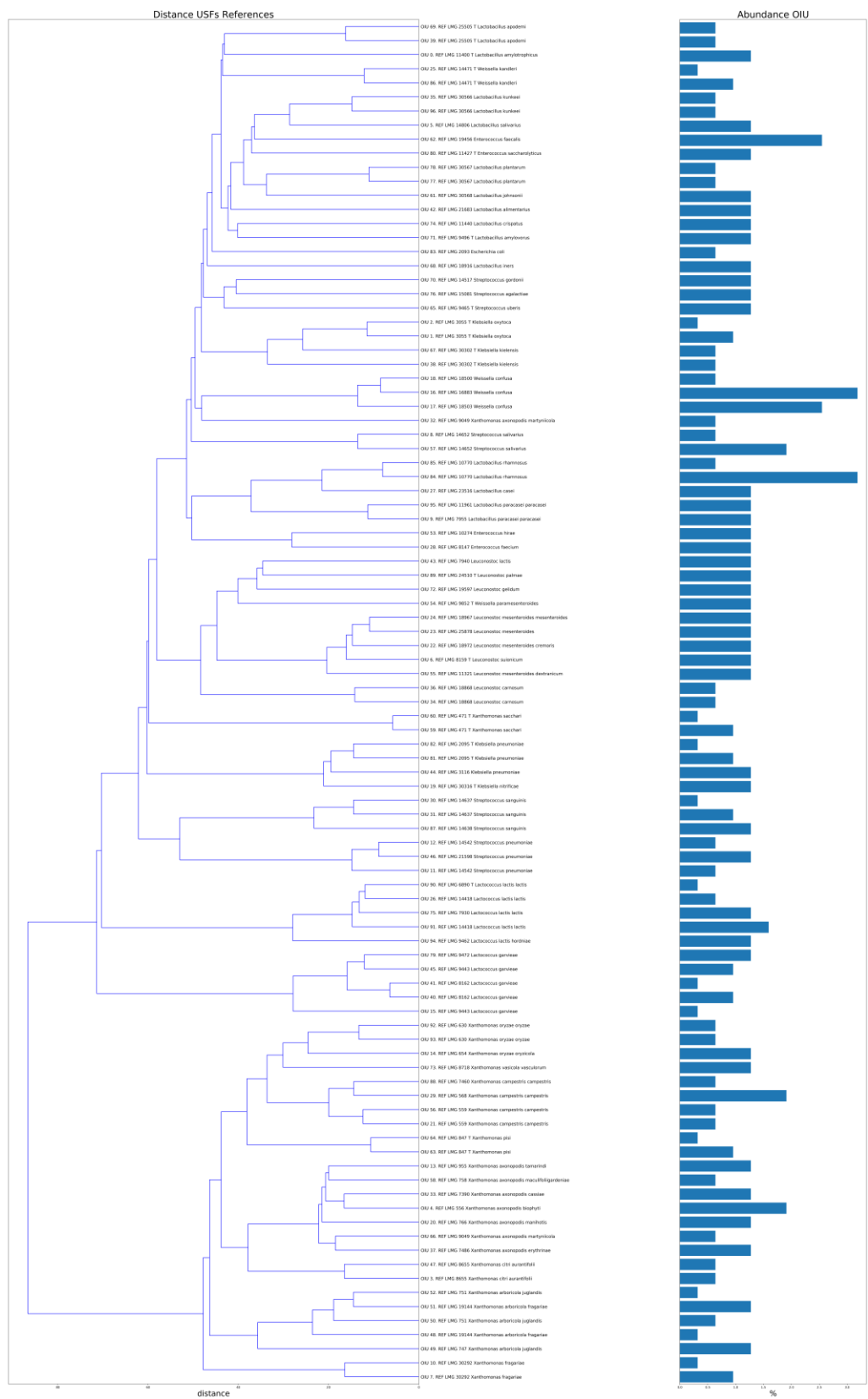

Figure S4

Supplement: FIG S4 [file mSystems.00437-19-sf004.pdf]
