# Supplementary material for: Introducing SPeDE: High-Throughput Dereplication and Accurate Determination of Microbial Diversity from Matrix-Assisted Laser Desorption–Ionization Time of Flight Mass Spectrometry Data
Source: mSystems. 2019 Sep 10;4(5):e00437-19. doi: 10.1128/mSystems.00437-19 (PMC6739102; doi:10.1128/mSystems.00437-19)
Supplement: TABLE S1 [file mSystems.00437-19-st001.pdf]

Table S1: Results of the benchmark study analyzed by SPeDE with default settings

Spectrum file	Quality	Reference	Reference number	OTU	Strain	Genus	Species	Subspecies
Z0012_RB_C10_2_C08_B	GREEN	Yes	0	5	LMG 1226	*Delftia*	*acidovorans*	
Z0012_LB_D06_1_C04_B	GREEN	No	0	5	LMG 1226	*Delftia*	*acidovorans*	
Z0012_RB_C11_2_C07_A	GREEN	No	0	5	LMG 1226	*Delftia*	*acidovorans*	
Z0012_LB_D05_1_C04_A	GREEN	No	0	5	LMG 1226	*Delftia*	*acidovorans*	
Z0012_LB_D07_1_C03_A	GREEN	No	0	5	LMG 1226	*Delftia*	*acidovorans*	
Z0012_LB_D10_1_C02_B	GREEN	Yes	1	5	LMG 1226	*Delftia*	*acidovorans*	
Z0012_RB_C06_2_C10_B	GREEN	No	1	5	LMG 1226	*Delftia*	*acidovorans*	
Z0012_RB_D01_1_C12_A	GREEN	No	1	5	LMG 1226	*Delftia*	*acidovorans*	
Z0012_LB_D01_1_C06_A	GREEN	No	1	5	LMG 1226	*Delftia*	*acidovorans*	
Z0012_LB_D08_1_C03_B	GREEN	No	1	5	LMG 1226	*Delftia*	*acidovorans*	
Z0012_RB_D02_1_C12_B	GREEN	No	1	5	LMG 1226	*Delftia*	*acidovorans*	
Z0012_LB_D12_1_C01_B	GREEN	No	1	5	LMG 1226	*Delftia*	*acidovorans*	
Z0012_LB_D09_1_C02_A	GREEN	No	1	5	LMG 1226	*Delftia*	*acidovorans*	
Z0012_LB_C12_2_C01_B	GREEN	No	1	5	LMG 1226	*Delftia*	*acidovorans*	
Z0012_RB_C08_2_C09_B	GREEN	No	1	5	LMG 1226	*Delftia*	*acidovorans*	
Z0012_LB_E03_2_B05_A	GREEN	No	1	5	LMG 1226	*Delftia*	*acidovorans*	
Z0012_LB_D02_1_C06_B	GREEN	No	1	5	LMG 1226	*Delftia*	*acidovorans*	
Z0012_LB_E05_2_B04_A	GREEN	No	1	5	LMG 1226	*Delftia*	*acidovorans*	
Z0012_RB_C12_2_C07_B	GREEN	No	1	5	LMG 1226	*Delftia*	*acidovorans*	
Z0012_LB_E04_2_B05_B	GREEN	No	1	5	LMG 1226	*Delftia*	*acidovorans*	
Z0012_RB_D06_1_C10_B	GREEN	No	1	5	LMG 1226	*Delftia*	*acidovorans*	
Z0012_LB_E01_2_B06_A	GREEN	No	1	5	LMG 1226	*Delftia*	*acidovorans*	
Z0012_LB_D04_1_C05_B	GREEN	No	1	5	LMG 1226	*Delftia*	*acidovorans*	
Z0012_LB_E02_2_B06_B	GREEN	No	1	5	LMG 1226	*Delftia*	*acidovorans*	
Z0012_RB_C07_2_C09_A	GREEN	No	1	5	LMG 1226	*Delftia*	*acidovorans*	
Z0012_RB_C09_2_C08_A	GREEN	No	1	5	LMG 1226	*Delftia*	*acidovorans*	
Z0012_RB_D04_1_C11_B	GREEN	No	1	5	LMG 1226	*Delftia*	*acidovorans*	
Z0012_LB_D11_1_C01_A	GREEN	No	1	5	LMG 1226	*Delftia*	*acidovorans*	
Z0012_LB_C11_2_C01_A	GREEN	No	1	5	LMG 1226	*Delftia*	*acidovorans*	
Z0012_LB_D03_1_C05_A	GREEN	No	1	5	LMG 1226	*Delftia*	*acidovorans*	
Z0012_RB_D05_1_C10_A	GREEN	No	1	5	LMG 1226	*Delftia*	*acidovorans*	
Z0012_RB_D03_1_C11_A	GREEN	No	1	5	LMG 1226	*Delftia*	*acidovorans*	
Z0017_LB_E10_2_B02_B	GREEN	Yes	2	59	LMG 23818	*Rheinheimera*	*chironomi*	
Z0017_RB_F03_1_B11_A	GREEN	No	2	59	LMG 23818	*Rheinheimera*	*chironomi*	
Z0017_RB_E09_2_B08_A	GREEN	No	2	59	LMG 23818	*Rheinheimera*	*chironomi*	
Z0017_RB_E08_2_B09_B	GREEN	No	2	59	LMG 23818	*Rheinheimera*	*chironomi*	
Z0017_LB_F03_1_B05_A	GREEN	No	2	59	LMG 23818	*Rheinheimera*	*chironomi*	
Z0017_RB_E12_2_B07_B	GREEN	No	2	59	LMG 23818	*Rheinheimera*	*chironomi*	
Z0017_RB_E10_2_B08_B	GREEN	No	2	59	LMG 23818	*Rheinheimera*	*chironomi*	
Z0017_RB_F05_1_B10_A	GREEN	No	2	59	LMG 23818	*Rheinheimera*	*chironomi*	
Z0017_RB_F01_1_B12_A	GREEN	No	2	59	LMG 23818	*Rheinheimera*	*chironomi*	
Z0017_LB_F09_1_B02_A	GREEN	No	2	59	LMG 23818	*Rheinheimera*	*chironomi*	
Z0017_RB_F02_1_B12_B	GREEN	No	2	59	LMG 23818	*Rheinheimera*	*chironomi*	
Z0017_LB_F05_1_B04_A	GREEN	No	2	59	LMG 23818	*Rheinheimera*	*chironomi*	
Z0017_RB_F09_1_B08_A	GREEN	No	2	59	LMG 23818	*Rheinheimera*	*chironomi*	
Z0017_RB_E11_2_B07_A	GREEN	No	2	59	LMG 23818	*Rheinheimera*	*chironomi*	
Z0017_RB_F06_1_B10_B	GREEN	No	2	59	LMG 23818	*Rheinheimera*	*chironomi*	
Z0017_RB_E06_2_B10_B	GREEN	No	2	59	LMG 23818	*Rheinheimera*	*chironomi*	
Z0017_LB_F07_1_B03_A	GREEN	No	2	59	LMG 23818	*Rheinheimera*	*chironomi*	
Z0017_RB_F08_1_B09_B	GREEN	No	2	59	LMG 23818	*Rheinheimera*	*chironomi*	
Z0017_RB_E05_2_B10_A	GREEN	No	2	59	LMG 23818	*Rheinheimera*	*chironomi*	
Z0017_LB_F01_1_B06_A	GREEN	No	2	59	LMG 23818	*Rheinheimera*	*chironomi*	
Z0017_LB_F02_1_B06_B	GREEN	No	2	59	LMG 23818	*Rheinheimera*	*chironomi*	
Z0017_LB_E12_2_B01_B	GREEN	No	2	59	LMG 23818	*Rheinheimera*	*chironomi*	
Z0017_LB_F04_1_B05_B	GREEN	No	2	59	LMG 23818	*Rheinheimera*	*chironomi*	
Z0017_RB_E07_2_B09_A	GREEN	No	2	59	LMG 23818	*Rheinheimera*	*chironomi*	
Z0017_LB_F10_1_B02_B	GREEN	No	2	59	LMG 23818	*Rheinheimera*	*chironomi*	
Z0017_RB_F10_1_B08_B	GREEN	No	2	59	LMG 23818	*Rheinheimera*	*chironomi*	
Z0017_LB_E09_2_B02_A	GREEN	No	2	59	LMG 23818	*Rheinheimera*	*chironomi*	
Z0017_RB_F04_1_B11_B	GREEN	No	2	59	LMG 23818	*Rheinheimera*	*chironomi*	
Z0017_LB_E11_2_B01_A	GREEN	No	2	59	LMG 23818	*Rheinheimera*	*chironomi*	
Z0017_RB_F07_1_B09_A	GREEN	No	2	59	LMG 23818	*Rheinheimera*	*chironomi*	
Z0017_LB_F08_1_B03_B	GREEN	No	2	59	LMG 23818	*Rheinheimera*	*chironomi*	
Z0017_LB_F06_1_B04_B	GREEN	No	2	59	LMG 23818	*Rheinheimera*	*chironomi*	
Z0023_LB_E10_2_B02_B	GREEN	Yes	3	118	LMG 4328	*Curvibacter*	*delicatus*	
Z0023_LB_E07_2_B03_A	GREEN	No	3	118	LMG 4328	*Curvibacter*	*delicatus*	

Z0023_LB_F04_1_B05_B	GREEN	No	3	118	LMG 4328	*Curvibacter*	*delicatus*	
Z0023_RB_E03_2_B11_A	GREEN	No	3	118	LMG 4328	*Curvibacter*	*delicatus*	
Z0023_RB_E01_2_B12_A	GREEN	No	3	118	LMG 4328	*Curvibacter*	*delicatus*	
Z0023_RB_E08_2_B09_B	GREEN	No	3	118	LMG 4328	*Curvibacter*	*delicatus*	
Z0023_RB_E02_2_B12_B	GREEN	No	3	118	LMG 4328	*Curvibacter*	*delicatus*	
Z0023_LB_F01_1_B06_A	GREEN	No	3	118	LMG 4328	*Curvibacter*	*delicatus*	
Z0023_RB_D05_1_C10_A	GREEN	No	3	118	LMG 4328	*Curvibacter*	*delicatus*	
Z0023_LB_E05_2_B04_A	GREEN	No	3	118	LMG 4328	*Curvibacter*	*delicatus*	
Z0023_RB_D10_1_C08_B	GREEN	No	3	118	LMG 4328	*Curvibacter*	*delicatus*	
Z0023_LB_E09_2_B02_A	GREEN	No	3	118	LMG 4328	*Curvibacter*	*delicatus*	
Z0023_RB_D07_1_C09_A	GREEN	No	3	118	LMG 4328	*Curvibacter*	*delicatus*	
Z0023_LB_E12_2_B01_B	GREEN	No	3	118	LMG 4328	*Curvibacter*	*delicatus*	
Z0023_LB_E03_2_B05_A	GREEN	No	3	118	LMG 4328	*Curvibacter*	*delicatus*	
Z0023_RB_D09_1_C08_A	GREEN	No	3	118	LMG 4328	*Curvibacter*	*delicatus*	
Z0023_RB_D08_1_C09_B	GREEN	No	3	118	LMG 4328	*Curvibacter*	*delicatus*	
Z0023_RB_D06_1_C10_B	GREEN	No	3	118	LMG 4328	*Curvibacter*	*delicatus*	
Z0023_RB_D04_1_C11_B	GREEN	No	3	118	LMG 4328	*Curvibacter*	*delicatus*	
Z0023_RB_E07_2_B09_A	GREEN	No	3	118	LMG 4328	*Curvibacter*	*delicatus*	
Z0023_LB_E04_2_B05_B	GREEN	No	3	118	LMG 4328	*Curvibacter*	*delicatus*	
Z0023_RB_E05_2_B10_A	GREEN	No	3	118	LMG 4328	*Curvibacter*	*delicatus*	
Z0023_RB_D12_1_C07_B	GREEN	No	3	118	LMG 4328	*Curvibacter*	*delicatus*	
Z0023_LB_F03_1_B05_A	GREEN	No	3	118	LMG 4328	*Curvibacter*	*delicatus*	
Z0023_LB_F02_1_B06_B	GREEN	No	3	118	LMG 4328	*Curvibacter*	*delicatus*	
Z0023_LB_F05_1_B04_A	GREEN	No	3	118	LMG 4328	*Curvibacter*	*delicatus*	
Z0023_RB_E04_2_B11_B	GREEN	No	3	118	LMG 4328	*Curvibacter*	*delicatus*	
Z0023_LB_E08_2_B03_B	GREEN	No	3	118	LMG 4328	*Curvibacter*	*delicatus*	
Z0023_RB_E06_2_B10_B	GREEN	No	3	118	LMG 4328	*Curvibacter*	*delicatus*	
Z0023_LB_E11_2_B01_A	GREEN	No	3	118	LMG 4328	*Curvibacter*	*delicatus*	
Z0023_RB_D11_1_C07_A	GREEN	No	3	118	LMG 4328	*Curvibacter*	*delicatus*	
Z0023_LB_E06_2_B04_B	GREEN	Yes	4	118	LMG 4328	*Curvibacter*	*delicatus*	
Z0022_RB_F02_1_B12_B	GREEN	Yes	5	108	LMG 29427	*Nakamurella*	*silvestris*	
Z0022_LB_F06_1_B04_B	GREEN	No	5	108	LMG 29427	*Nakamurella*	*silvestris*	
Z0022_LB_F12_1_B01_B	GREEN	No	5	108	LMG 29427	*Nakamurella*	*silvestris*	
Z0022_LB_F05_1_B04_A	GREEN	No	5	108	LMG 29427	*Nakamurella*	*silvestris*	
Z0022_RB_F08_1_B09_B	GREEN	No	5	108	LMG 29427	*Nakamurella*	*silvestris*	
Z0022_RB_E11_2_B07_A	GREEN	No	5	108	LMG 29427	*Nakamurella*	*silvestris*	
Z0022_LB_G04_2_A05_B	GREEN	No	5	108	LMG 29427	*Nakamurella*	*silvestris*	
Z0022_RB_F05_1_B10_A	GREEN	No	5	108	LMG 29427	*Nakamurella*	*silvestris*	
Z0022_LB_F02_1_B06_B	GREEN	No	5	108	LMG 29427	*Nakamurella*	*silvestris*	
Z0022_LB_F07_1_B03_A	GREEN	No	5	108	LMG 29427	*Nakamurella*	*silvestris*	
Z0022_RB_E10_2_B08_B	GREEN	No	5	108	LMG 29427	*Nakamurella*	*silvestris*	
Z0022_RB_F04_1_B11_B	GREEN	No	5	108	LMG 29427	*Nakamurella*	*silvestris*	
Z0022_RB_F06_1_B10_B	GREEN	No	5	108	LMG 29427	*Nakamurella*	*silvestris*	
Z0022_LB_G02_2_A06_B	GREEN	No	5	108	LMG 29427	*Nakamurella*	*silvestris*	
Z0022_LB_F09_1_B02_A	GREEN	No	5	108	LMG 29427	*Nakamurella*	*silvestris*	
Z0022_RB_F07_1_B09_A	GREEN	No	5	108	LMG 29427	*Nakamurella*	*silvestris*	
Z0022_LB_G03_2_A05_A	GREEN	No	5	108	LMG 29427	*Nakamurella*	*silvestris*	
Z0022_RB_E09_2_B08_A	GREEN	No	5	108	LMG 29427	*Nakamurella*	*silvestris*	
Z0022_RB_F12_1_B07_B	GREEN	No	5	108	LMG 29427	*Nakamurella*	*silvestris*	
Z0022_LB_F10_1_B02_B	GREEN	No	5	108	LMG 29427	*Nakamurella*	*silvestris*	
Z0022_RB_F09_1_B08_A	GREEN	No	5	108	LMG 29427	*Nakamurella*	*silvestris*	
Z0022_LB_F04_1_B05_B	GREEN	No	5	108	LMG 29427	*Nakamurella*	*silvestris*	
Z0022_RB_E12_2_B07_B	GREEN	No	5	108	LMG 29427	*Nakamurella*	*silvestris*	
Z0022_RB_F03_1_B11_A	GREEN	No	5	108	LMG 29427	*Nakamurella*	*silvestris*	
Z0022_LB_F11_1_B01_A	GREEN	No	5	108	LMG 29427	*Nakamurella*	*silvestris*	
Z0022_RB_G01_2_A12_A	GREEN	No	5	108	LMG 29427	*Nakamurella*	*silvestris*	
Z0022_RB_F10_1_B08_B	GREEN	No	5	108	LMG 29427	*Nakamurella*	*silvestris*	
Z0022_LB_G01_2_A06_A	GREEN	No	5	108	LMG 29427	*Nakamurella*	*silvestris*	
Z0022_RB_F11_1_B07_A	GREEN	No	5	108	LMG 29427	*Nakamurella*	*silvestris*	
Z0022_LB_F03_1_B05_A	GREEN	No	5	108	LMG 29427	*Nakamurella*	*silvestris*	
Z0022_LB_F08_1_B03_B	GREEN	No	5	108	LMG 29427	*Nakamurella*	*silvestris*	
Z0022_RB_F01_1_B12_A	GREEN	No	5	108	LMG 29427	*Nakamurella*	*silvestris*	
Z0021_LB_C10_2_C02_B	GREEN	Yes	6	99	LMG 2724	*Lonsdalea*	*quercina*	*quercina*
Z0021_RB_B10_1_D08_B	GREEN	No	6	99	LMG 2724	*Lonsdalea*	*quercina*	*quercina*
Z0021_LB_C09_2_C02_A	GREEN	No	6	99	LMG 2724	*Lonsdalea*	*quercina*	*quercina*
Z0021_RB_C11_2_C07_A	GREEN	No	6	99	LMG 2724	*Lonsdalea*	*quercina*	*quercina*
Z0021_RB_C02_2_C12_B	GREEN	No	6	99	LMG 2724	*Lonsdalea*	*quercina*	*quercina*
Z0021_RB_B11_1_D07_A	GREEN	No	6	99	LMG 2724	*Lonsdalea*	*quercina*	*quercina*
Z0021_LB_C08_2_C03_B	GREEN	No	6	99	LMG 2724	*Lonsdalea*	*quercina*	*quercina*
Z0021_RB_C01_2_C12_A	GREEN	No	6	99	LMG 2724	*Lonsdalea*	*quercina*	*quercina*

Z0021_RB_D02_1_C12_B	GREEN	No	6	99	LMG 2724	*Lonsdalea*	*quercina*	*quercina*
Z0021_RB_D01_1_C12_A	GREEN	No	6	99	LMG 2724	*Lonsdalea*	*quercina*	*quercina*
Z0021_RB_C04_2_C11_B	GREEN	No	6	99	LMG 2724	*Lonsdalea*	*quercina*	*quercina*
Z0021_LB_C07_2_C03_A	GREEN	No	6	99	LMG 2724	*Lonsdalea*	*quercina*	*quercina*
Z0021_RB_C03_2_C11_A	GREEN	No	6	99	LMG 2724	*Lonsdalea*	*quercina*	*quercina*
Z0021_RB_C07_2_C09_A	GREEN	No	6	99	LMG 2724	*Lonsdalea*	*quercina*	*quercina*
Z0021_RB_C10_2_C08_B	GREEN	No	6	99	LMG 2724	*Lonsdalea*	*quercina*	*quercina*
Z0021_RB_B12_1_D07_B	GREEN	No	6	99	LMG 2724	*Lonsdalea*	*quercina*	*quercina*
Z0021_LB_D01_1_C06_A	GREEN	No	6	99	LMG 2724	*Lonsdalea*	*quercina*	*quercina*
Z0021_LB_D02_1_C06_B	GREEN	No	6	99	LMG 2724	*Lonsdalea*	*quercina*	*quercina*
Z0021_LB_C12_2_C01_B	GREEN	No	6	99	LMG 2724	*Lonsdalea*	*quercina*	*quercina*
Z0021_LB_D04_1_C05_B	GREEN	No	6	99	LMG 2724	*Lonsdalea*	*quercina*	*quercina*
Z0021_LB_D05_1_C04_A	GREEN	No	6	99	LMG 2724	*Lonsdalea*	*quercina*	*quercina*
Z0021_RB_C09_2_C08_A	GREEN	No	6	99	LMG 2724	*Lonsdalea*	*quercina*	*quercina*
Z0021_RB_C06_2_C10_B	GREEN	No	6	99	LMG 2724	*Lonsdalea*	*quercina*	*quercina*
Z0021_RB_C12_2_C07_B	GREEN	No	6	99	LMG 2724	*Lonsdalea*	*quercina*	*quercina*
Z0021_RB_B09_1_D08_A	GREEN	No	6	99	LMG 2724	*Lonsdalea*	*quercina*	*quercina*
Z0021_LB_C06_2_C04_B	GREEN	No	6	99	LMG 2724	*Lonsdalea*	*quercina*	*quercina*
Z0021_LB_D06_1_C04_B	GREEN	No	6	99	LMG 2724	*Lonsdalea*	*quercina*	*quercina*
Z0021_RB_C05_2_C10_A	GREEN	No	6	99	LMG 2724	*Lonsdalea*	*quercina*	*quercina*
Z0021_RB_C08_2_C09_B	GREEN	No	6	99	LMG 2724	*Lonsdalea*	*quercina*	*quercina*
Z0021_RB_B08_1_D09_B	GREEN	No	6	99	LMG 2724	*Lonsdalea*	*quercina*	*quercina*
Z0021_LB_C11_2_C01_A	GREEN	No	6	99	LMG 2724	*Lonsdalea*	*quercina*	*quercina*
Z0021_LB_D03_1_C05_A	GREEN	No	6	99	LMG 2724	*Lonsdalea*	*quercina*	*quercina*
Z0024_RB_D04_1_C11_B	GREEN	Yes	7	125	LMG 6866	*Ralstonia*	*mannitolilytica*	
Z0024_RB_E06_2_B10_B	GREEN	No	7	125	LMG 6866	*Ralstonia*	*mannitolilytica*	
Z0024_LB_D12_1_C01_B	GREEN	No	7	125	LMG 6866	*Ralstonia*	*mannitolilytica*	
Z0024_LB_E01_2_B06_A	GREEN	No	7	125	LMG 6866	*Ralstonia*	*mannitolilytica*	
Z0024_LB_D11_1_C01_A	GREEN	No	7	125	LMG 6866	*Ralstonia*	*mannitolilytica*	
Z0024_LB_D05_1_C04_A	GREEN	No	7	125	LMG 6866	*Ralstonia*	*mannitolilytica*	
Z0024_LB_D10_1_C02_B	GREEN	No	7	125	LMG 6866	*Ralstonia*	*mannitolilytica*	
Z0024_RB_D08_1_C09_B	GREEN	No	7	125	LMG 6866	*Ralstonia*	*mannitolilytica*	
Z0024_RB_D06_1_C10_B	GREEN	No	7	125	LMG 6866	*Ralstonia*	*mannitolilytica*	
Z0024_LB_E02_2_B06_B	GREEN	No	7	125	LMG 6866	*Ralstonia*	*mannitolilytica*	
Z0024_LB_C11_2_C01_A	GREEN	No	7	125	LMG 6866	*Ralstonia*	*mannitolilytica*	
Z0024_LB_D04_1_C05_B	GREEN	No	7	125	LMG 6866	*Ralstonia*	*mannitolilytica*	
Z0024_RB_E03_2_B11_A	GREEN	No	7	125	LMG 6866	*Ralstonia*	*mannitolilytica*	
Z0024_RB_E02_2_B12_B	GREEN	No	7	125	LMG 6866	*Ralstonia*	*mannitolilytica*	
Z0024_RB_D10_1_C08_B	GREEN	No	7	125	LMG 6866	*Ralstonia*	*mannitolilytica*	
Z0024_LB_D01_1_C06_A	GREEN	No	7	125	LMG 6866	*Ralstonia*	*mannitolilytica*	
Z0024_LB_C10_2_C02_B	GREEN	No	7	125	LMG 6866	*Ralstonia*	*mannitolilytica*	
Z0024_RB_E05_2_B10_A	GREEN	No	7	125	LMG 6866	*Ralstonia*	*mannitolilytica*	
Z0024_LB_D09_1_C02_A	GREEN	No	7	125	LMG 6866	*Ralstonia*	*mannitolilytica*	
Z0024_RB_D11_1_C07_A	GREEN	No	7	125	LMG 6866	*Ralstonia*	*mannitolilytica*	
Z0024_RB_E01_2_B12_A	GREEN	No	7	125	LMG 6866	*Ralstonia*	*mannitolilytica*	
Z0024_LB_D03_1_C05_A	GREEN	No	7	125	LMG 6866	*Ralstonia*	*mannitolilytica*	
Z0024_LB_D08_1_C03_B	GREEN	No	7	125	LMG 6866	*Ralstonia*	*mannitolilytica*	
Z0024_RB_D12_1_C07_B	GREEN	No	7	125	LMG 6866	*Ralstonia*	*mannitolilytica*	
Z0024_RB_D05_1_C10_A	GREEN	No	7	125	LMG 6866	*Ralstonia*	*mannitolilytica*	
Z0024_LB_C12_2_C01_B	GREEN	No	7	125	LMG 6866	*Ralstonia*	*mannitolilytica*	
Z0024_RB_D07_1_C09_A	GREEN	No	7	125	LMG 6866	*Ralstonia*	*mannitolilytica*	
Z0024_LB_D06_1_C04_B	GREEN	No	7	125	LMG 6866	*Ralstonia*	*mannitolilytica*	
Z0024_LB_D02_1_C06_B	GREEN	No	7	125	LMG 6866	*Ralstonia*	*mannitolilytica*	
Z0024_RB_E04_2_B11_B	GREEN	No	7	125	LMG 6866	*Ralstonia*	*mannitolilytica*	
Z0024_RB_D03_1_C11_A	GREEN	No	7	125	LMG 6866	*Ralstonia*	*mannitolilytica*	
Z0024_RB_D09_1_C08_A	GREEN	No	7	125	LMG 6866	*Ralstonia*	*mannitolilytica*	
Z0016_LB_D04_1_C05_B	GREEN	Yes	8	45	LMG 22475	*Phaeobacter*	*inhibens*	
Z0016_RB_C10_2_C08_B	GREEN	No	8	45	LMG 22475	*Phaeobacter*	*inhibens*	
Z0016_RB_C12_2_C07_B	GREEN	No	8	45	LMG 22475	*Phaeobacter*	*inhibens*	
Z0016_LB_D06_1_C04_B	GREEN	No	8	45	LMG 22475	*Phaeobacter*	*inhibens*	
Z0016_RB_D01_1_C12_A	GREEN	No	8	45	LMG 22475	*Phaeobacter*	*inhibens*	
Z0016_RB_C11_2_C07_A	GREEN	No	8	45	LMG 22475	*Phaeobacter*	*inhibens*	
Z0016_LB_C11_2_C01_A	GREEN	No	8	45	LMG 22475	*Phaeobacter*	*inhibens*	
Z0016_LB_D03_1_C05_A	GREEN	No	8	45	LMG 22475	*Phaeobacter*	*inhibens*	
Z0016_LB_C12_2_C01_B	GREEN	No	8	45	LMG 22475	*Phaeobacter*	*inhibens*	
Z0016_RB_C09_2_C08_A	GREEN	No	8	45	LMG 22475	*Phaeobacter*	*inhibens*	
Z0016_RB_D03_1_C11_A	GREEN	No	8	45	LMG 22475	*Phaeobacter*	*inhibens*	
Z0016_LB_D02_1_C06_B	GREEN	No	8	45	LMG 22475	*Phaeobacter*	*inhibens*	
Z0016_LB_D01_1_C06_A	GREEN	No	8	45	LMG 22475	*Phaeobacter*	*inhibens*	
Z0016_LB_C06_2_C04_B	GREEN	No	8	45	LMG 22475	*Phaeobacter*	*inhibens*	

Z0016_RB_B12_1_D07_B	GREEN	No	8	45	LMG 22475	*Phaeobacter*	*inhibens*
Z0016_LB_C07_2_C03_A	GREEN	No	8	45	LMG 22475	*Phaeobacter*	*inhibens*
Z0016_RB_C08_2_C09_B	GREEN	No	8	45	LMG 22475	*Phaeobacter*	*inhibens*
Z0016_RB_B09_1_D08_A	GREEN	No	8	45	LMG 22475	*Phaeobacter*	*inhibens*
Z0016_RB_C05_2_C10_A	GREEN	No	8	45	LMG 22475	*Phaeobacter*	*inhibens*
Z0016_LB_D05_1_C04_A	GREEN	No	8	45	LMG 22475	*Phaeobacter*	*inhibens*
Z0016_RB_C06_2_C10_B	GREEN	No	8	45	LMG 22475	*Phaeobacter*	*inhibens*
Z0016_RB_B10_1_D08_B	GREEN	No	8	45	LMG 22475	*Phaeobacter*	*inhibens*
Z0016_RB_B11_1_D07_A	GREEN	No	8	45	LMG 22475	*Phaeobacter*	*inhibens*
Z0016_RB_C01_2_C12_A	GREEN	No	8	45	LMG 22475	*Phaeobacter*	*inhibens*
Z0016_RB_C02_2_C12_B	GREEN	No	8	45	LMG 22475	*Phaeobacter*	*inhibens*
Z0016_RB_C03_2_C11_A	GREEN	No	8	45	LMG 22475	*Phaeobacter*	*inhibens*
Z0016_RB_C07_2_C09_A	GREEN	No	8	45	LMG 22475	*Phaeobacter*	*inhibens*
Z0016_LB_C10_2_C02_B	GREEN	No	8	45	LMG 22475	*Phaeobacter*	*inhibens*
Z0016_LB_C09_2_C02_A	GREEN	No	8	45	LMG 22475	*Phaeobacter*	*inhibens*
Z0016_RB_D02_1_C12_B	GREEN	No	8	45	LMG 22475	*Phaeobacter*	*inhibens*
Z0016_RB_C04_2_C11_B	GREEN	No	8	45	LMG 22475	*Phaeobacter*	*inhibens*
Z0016_LB_C08_2_C03_B	GREEN	No	8	45	LMG 22475	*Phaeobacter*	*inhibens*
Z0015_RB_A04_2_D11_B	GREEN	Yes	9	34	LMG 21276	*Psychrobacter*	*luti*
Z0015_RB_A02_2_D12_B	GREEN	No	9	34	LMG 21276	*Psychrobacter*	*luti*
Z0015_RB_A05_2_D10_A	GREEN	No	9	34	LMG 21276	*Psychrobacter*	*luti*
Z0015_RB_A07_2_D09_A	GREEN	No	9	34	LMG 21276	*Psychrobacter*	*luti*
Z0015_RB_A06_2_D10_B	GREEN	No	9	34	LMG 21276	*Psychrobacter*	*luti*
Z0015_RB_A08_2_D09_B	GREEN	No	9	34	LMG 21276	*Psychrobacter*	*luti*
Z0015_LB_B11_1_D01_A	GREEN	No	9	34	LMG 21276	*Psychrobacter*	*luti*
Z0015_RB_B02_1_D12_B	GREEN	No	9	34	LMG 21276	*Psychrobacter*	*luti*
Z0015_RB_B01_1_D12_A	GREEN	No	9	34	LMG 21276	*Psychrobacter*	*luti*
Z0015_LB_C10_2_C02_B	GREEN	No	9	34	LMG 21276	*Psychrobacter*	*luti*
Z0015_RB_B03_1_D11_A	GREEN	No	9	34	LMG 21276	*Psychrobacter*	*luti*
Z0015_RB_A09_2_D08_A	GREEN	No	9	34	LMG 21276	*Psychrobacter*	*luti*
Z0015_LB_B12_1_D01_B	GREEN	No	9	34	LMG 21276	*Psychrobacter*	*luti*
Z0015_LB_C01_2_C06_A	GREEN	No	9	34	LMG 21276	*Psychrobacter*	*luti*
Z0015_RB_B04_1_D11_B	GREEN	No	9	34	LMG 21276	*Psychrobacter*	*luti*
Z0015_LB_C02_2_C06_B	GREEN	No	9	34	LMG 21276	*Psychrobacter*	*luti*
Z0015_RB_A03_2_D11_A	GREEN	No	9	34	LMG 21276	*Psychrobacter*	*luti*
Z0015_LO_H12_2_H06_B	GREEN	No	9	34	LMG 21276	*Psychrobacter*	*luti*
Z0015_LB_C05_2_C04_A	GREEN	No	9	34	LMG 21276	*Psychrobacter*	*luti*
Z0015_LB_B10_1_D02_B	GREEN	No	9	34	LMG 21276	*Psychrobacter*	*luti*
Z0015_LB_C04_2_C05_B	GREEN	No	9	34	LMG 21276	*Psychrobacter*	*luti*
Z0015_LB_C09_2_C02_A	GREEN	No	9	34	LMG 21276	*Psychrobacter*	*luti*
Z0015_LB_C08_2_C03_B	GREEN	No	9	34	LMG 21276	*Psychrobacter*	*luti*
Z0015_LB_B09_1_D02_A	GREEN	No	9	34	LMG 21276	*Psychrobacter*	*luti*
Z0015_LB_C06_2_C04_B	GREEN	No	9	34	LMG 21276	*Psychrobacter*	*luti*
Z0015_RB_A01_2_D12_A	GREEN	No	9	34	LMG 21276	*Psychrobacter*	*luti*
Z0015_LB_B08_1_D03_B	GREEN	No	9	34	LMG 21276	*Psychrobacter*	*luti*
Z0015_RB_A12_2_D07_B	GREEN	No	9	34	LMG 21276	*Psychrobacter*	*luti*
Z0015_RB_A10_2_D08_B	GREEN	No	9	34	LMG 21276	*Psychrobacter*	*luti*
Z0015_LB_C03_2_C05_A	GREEN	No	9	34	LMG 21276	*Psychrobacter*	*luti*
Z0015_LB_C07_2_C03_A	GREEN	No	9	34	LMG 21276	*Psychrobacter*	*luti*
Z0015_RB_A11_2_D07_A	GREEN	No	9	34	LMG 21276	*Psychrobacter*	*luti*
Z0018_LO_D02_2_F01_B	GREEN	Yes	10	74	LMG 24559	*Arcobacter*	*mytili*
Z0018_LO_D11_2_F06_A	GREEN	No	10	74	LMG 24559	*Arcobacter*	*mytili*
Z0018_LO_D12_2_F06_B	GREEN	No	10	74	LMG 24559	*Arcobacter*	*mytili*
Z0018_LO_E01_1_G01_A	GREEN	No	10	74	LMG 24559	*Arcobacter*	*mytili*
Z0018_LO_D04_2_F02_B	GREEN	No	10	74	LMG 24559	*Arcobacter*	*mytili*
Z0018_RO_E02_1_G07_B	GREEN	No	10	74	LMG 24559	*Arcobacter*	*mytili*
Z0018_LO_F09_2_G05_A	GREEN	No	10	74	LMG 24559	*Arcobacter*	*mytili*
Z0018_LO_D08_2_F04_B	GREEN	No	10	74	LMG 24559	*Arcobacter*	*mytili*
Z0018_RO_D03_2_F08_A	GREEN	No	10	74	LMG 24559	*Arcobacter*	*mytili*
Z0018_LO_F10_2_G05_B	GREEN	No	10	74	LMG 24559	*Arcobacter*	*mytili*
Z0018_RO_F05_2_G09_A	GREEN	No	10	74	LMG 24559	*Arcobacter*	*mytili*
Z0018_LO_D05_2_F03_A	GREEN	No	10	74	LMG 24559	*Arcobacter*	*mytili*
Z0018_RO_E04_1_G08_B	GREEN	No	10	74	LMG 24559	*Arcobacter*	*mytili*
Z0018_LO_F04_2_G02_B	GREEN	No	10	74	LMG 24559	*Arcobacter*	*mytili*
Z0018_RO_F04_2_G08_B	GREEN	No	10	74	LMG 24559	*Arcobacter*	*mytili*
Z0018_RO_F01_2_G07_A	GREEN	No	10	74	LMG 24559	*Arcobacter*	*mytili*
Z0018_LO_D07_2_F04_A	GREEN	No	10	74	LMG 24559	*Arcobacter*	*mytili*
Z0018_RO_E01_1_G07_A	GREEN	No	10	74	LMG 24559	*Arcobacter*	*mytili*
Z0018_LO_E04_1_G02_B	GREEN	No	10	74	LMG 24559	*Arcobacter*	*mytili*
Z0018_RO_C12_1_F12_B	GREEN	No	10	74	LMG 24559	*Arcobacter*	*mytili*

Z0018_LO_F03_2_G02_A	GREEN	No	10	74	LMG 24559	*Arcobacter*	*mytili*
Z0018_RO_F06_2_G09_B	GREEN	No	10	74	LMG 24559	*Arcobacter*	*mytili*
Z0018_LO_D09_2_F05_A	GREEN	No	10	74	LMG 24559	*Arcobacter*	*mytili*
Z0018_LO_E08_1_G04_B	GREEN	No	10	74	LMG 24559	*Arcobacter*	*mytili*
Z0018_LO_D10_2_F05_B	GREEN	No	10	74	LMG 24559	*Arcobacter*	*mytili*
Z0018_RO_E09_1_G11_A	GREEN	No	10	74	LMG 24559	*Arcobacter*	*mytili*
Z0018_LO_E09_1_G05_A	GREEN	No	10	74	LMG 24559	*Arcobacter*	*mytili*
Z0018_LO_E06_1_G03_B	GREEN	No	10	74	LMG 24559	*Arcobacter*	*mytili*
Z0018_RO_E06_1_G09_B	GREEN	No	10	74	LMG 24559	*Arcobacter*	*mytili*
Z0018_RO_E12_1_G12_B	GREEN	No	10	74	LMG 24559	*Arcobacter*	*mytili*
Z0018_RO_E10_1_G11_B	GREEN	No	10	74	LMG 24559	*Arcobacter*	*mytili*
Z0018_LO_E12_1_G06_B	GREEN	No	10	74	LMG 24559	*Arcobacter*	*mytili*
Z0012_RB_G03_2_A11_A	GREEN	Yes	11	7	LMG 1232	*Bordetella*	*bronchiseptica*
Z0012_LB_H02_1_A06_B	GREEN	No	11	7	LMG 1232	*Bordetella*	*bronchiseptica*
Z0012_RB_F03_1_B11_A	GREEN	No	11	7	LMG 1232	*Bordetella*	*bronchiseptica*
Z0012_RB_G01_2_A12_A	GREEN	No	11	7	LMG 1232	*Bordetella*	*bronchiseptica*
Z0012_LB_G09_2_A02_A	GREEN	No	11	7	LMG 1232	*Bordetella*	*bronchiseptica*
Z0012_LB_G06_2_A04_B	GREEN	No	11	7	LMG 1232	*Bordetella*	*bronchiseptica*
Z0012_LB_H06_1_A04_B	GREEN	No	11	7	LMG 1232	*Bordetella*	*bronchiseptica*
Z0012_RB_F04_1_B11_B	GREEN	No	11	7	LMG 1232	*Bordetella*	*bronchiseptica*
Z0012_LB_G12_2_A01_B	GREEN	No	11	7	LMG 1232	*Bordetella*	*bronchiseptica*
Z0012_LB_H11_1_A01_A	GREEN	No	11	7	LMG 1232	*Bordetella*	*bronchiseptica*
Z0012_LB_H07_1_A03_A	GREEN	No	11	7	LMG 1232	*Bordetella*	*bronchiseptica*
Z0012_LO_A01_1_E01_A	GREEN	No	11	7	LMG 1232	*Bordetella*	*bronchiseptica*
Z0012_LB_G01_2_A06_A	GREEN	No	11	7	LMG 1232	*Bordetella*	*bronchiseptica*
Z0012_LB_F12_1_B01_B	GREEN	No	11	7	LMG 1232	*Bordetella*	*bronchiseptica*
Z0012_RB_G10_2_A08_B	GREEN	No	11	7	LMG 1232	*Bordetella*	*bronchiseptica*
Z0012_RB_F02_1_B12_B	GREEN	No	11	7	LMG 1232	*Bordetella*	*bronchiseptica*
Z0012_RB_E11_2_B07_A	GREEN	No	11	7	LMG 1232	*Bordetella*	*bronchiseptica*
Z0012_RB_F01_1_B12_A	GREEN	No	11	7	LMG 1232	*Bordetella*	*bronchiseptica*
Z0012_RB_G11_2_A07_A	GREEN	No	11	7	LMG 1232	*Bordetella*	*bronchiseptica*
Z0012_LB_H09_1_A02_A	GREEN	No	11	7	LMG 1232	*Bordetella*	*bronchiseptica*
Z0012_RB_H01_1_A12_A	GREEN	No	11	7	LMG 1232	*Bordetella*	*bronchiseptica*
Z0012_LB_H01_1_A06_A	GREEN	No	11	7	LMG 1232	*Bordetella*	*bronchiseptica*
Z0012_RB_F06_1_B10_B	GREEN	No	11	7	LMG 1232	*Bordetella*	*bronchiseptica*
Z0012_RB_H04_1_A11_B	GREEN	No	11	7	LMG 1232	*Bordetella*	*bronchiseptica*
Z0012_LB_G07_2_A03_A	GREEN	No	11	7	LMG 1232	*Bordetella*	*bronchiseptica*
Z0012_RB_G06_2_A10_B	GREEN	No	11	7	LMG 1232	*Bordetella*	*bronchiseptica*
Z0012_LB_H04_1_A05_B	GREEN	No	11	7	LMG 1232	*Bordetella*	*bronchiseptica*
Z0012_LB_F10_1_B02_B	GREEN	No	11	7	LMG 1232	*Bordetella*	*bronchiseptica*
Z0012_RB_E12_2_B07_B	GREEN	No	11	7	LMG 1232	*Bordetella*	*bronchiseptica*
Z0012_LB_G11_2_A01_A	GREEN	Yes	12	7	LMG 1232	*Bordetella*	*bronchiseptica*
Z0012_RB_F09_1_B08_A	GREEN	No	12	7	LMG 1232	*Bordetella*	*bronchiseptica*
Z0012_RB_H03_1_A11_A	GREEN	Yes	13	7	LMG 1232	*Bordetella*	*bronchiseptica*
Z0010_RB_B06_1_D10_B	GREEN	Yes	14	148	R-69596	*Burkholderia*	*cepacia*
Z0010_LB_B10_1_D02_B	GREEN	No	14	148	R-69596	*Burkholderia*	*cepacia*
Z0010_RB_B07_1_D09_A	GREEN	No	14	148	R-69596	*Burkholderia*	*cepacia*
Z0010_RB_B05_1_D10_A	GREEN	No	14	148	R-69596	*Burkholderia*	*cepacia*
Z0010_LB_B06_1_D04_B	GREEN	No	14	148	R-69596	*Burkholderia*	*cepacia*
Z0010_LB_B07_1_D03_A	GREEN	No	14	148	R-69596	*Burkholderia*	*cepacia*
Z0010_LB_B08_1_D03_B	GREEN	No	14	148	R-69596	*Burkholderia*	*cepacia*
Z0010_RB_B03_1_D11_A	GREEN	No	14	148	R-69596	*Burkholderia*	*cepacia*
Z0010_LB_C06_2_C04_B	GREEN	No	14	148	R-69596	*Burkholderia*	*cepacia*
Z0010_LB_B12_1_D01_B	GREEN	No	14	148	R-69596	*Burkholderia*	*cepacia*
Z0010_LB_C04_2_C05_B	GREEN	No	14	148	R-69596	*Burkholderia*	*cepacia*
Z0010_LB_C02_2_C06_B	GREEN	No	14	148	R-69596	*Burkholderia*	*cepacia*
Z0010_LB_B02_1_D06_B	GREEN	No	14	148	R-69596	*Burkholderia*	*cepacia*
Z0010_RB_B01_1_D12_A	GREEN	No	14	148	R-69596	*Burkholderia*	*cepacia*
Z0010_LB_B11_1_D01_A	GREEN	No	14	148	R-69596	*Burkholderia*	*cepacia*
Z0010_RB_B10_1_D08_B	GREEN	No	14	148	R-69596	*Burkholderia*	*cepacia*
Z0010_LB_B05_1_D04_A	GREEN	No	14	148	R-69596	*Burkholderia*	*cepacia*
Z0010_RB_B12_1_D07_B	GREEN	No	14	148	R-69596	*Burkholderia*	*cepacia*
Z0010_RB_B11_1_D07_A	GREEN	No	14	148	R-69596	*Burkholderia*	*cepacia*
Z0010_RB_B08_1_D09_B	GREEN	No	14	148	R-69596	*Burkholderia*	*cepacia*
Z0010_LB_C05_2_C04_A	GREEN	No	14	148	R-69596	*Burkholderia*	*cepacia*
Z0010_RB_B04_1_D11_B	GREEN	No	14	148	R-69596	*Burkholderia*	*cepacia*
Z0010_LB_C03_2_C05_A	GREEN	No	14	148	R-69596	*Burkholderia*	*cepacia*
Z0010_LB_C07_2_C03_A	GREEN	No	14	148	R-69596	*Burkholderia*	*cepacia*
Z0010_RB_B09_1_D08_A	GREEN	No	14	148	R-69596	*Burkholderia*	*cepacia*
Z0010_RB_B02_1_D12_B	GREEN	No	14	148	R-69596	*Burkholderia*	*cepacia*

Z0010_LB_B03_1_D05_A	GREEN	No	14	148	R-69596	*Burkholderia*	*cepacia*
Z0010_LB_C01_2_C06_A	GREEN	No	14	148	R-69596	*Burkholderia*	*cepacia*
Z0010_LB_C08_2_C03_B	GREEN	No	14	148	R-69596	*Burkholderia*	*cepacia*
Z0010_LB_B04_1_D05_B	GREEN	No	14	148	R-69596	*Burkholderia*	*cepacia*
Z0010_RB_C01_2_C12_A	GREEN	No	14	148	R-69596	*Burkholderia*	*cepacia*
Z0010_LB_B09_1_D02_A	GREEN	Yes	15	148	R-69596	*Burkholderia*	*cepacia*
Z0018_RB_C03_2_C11_A	GREEN	Yes	16	69	LMG 24401	*Epilithonimonas*	*lactis*
Z0018_RB_C12_2_C07_B	GREEN	No	16	69	LMG 24401	*Epilithonimonas*	*lactis*
Z0018_LB_D01_1_C06_A	GREEN	No	16	69	LMG 24401	*Epilithonimonas*	*lactis*
Z0018_RB_D01_1_C12_A	GREEN	No	16	69	LMG 24401	*Epilithonimonas*	*lactis*
Z0018_LB_D02_1_C06_B	GREEN	No	16	69	LMG 24401	*Epilithonimonas*	*lactis*
Z0018_RB_C10_2_C08_B	GREEN	No	16	69	LMG 24401	*Epilithonimonas*	*lactis*
Z0018_LB_D07_1_C03_A	GREEN	No	16	69	LMG 24401	*Epilithonimonas*	*lactis*
Z0018_LB_D05_1_C04_A	GREEN	No	16	69	LMG 24401	*Epilithonimonas*	*lactis*
Z0018_LB_D06_1_C04_B	GREEN	No	16	69	LMG 24401	*Epilithonimonas*	*lactis*
Z0018_LB_C08_2_C03_B	GREEN	No	16	69	LMG 24401	*Epilithonimonas*	*lactis*
Z0018_RB_C02_2_C12_B	GREEN	No	16	69	LMG 24401	*Epilithonimonas*	*lactis*
Z0018_RB_C09_2_C08_A	GREEN	No	16	69	LMG 24401	*Epilithonimonas*	*lactis*
Z0018_RB_C11_2_C07_A	GREEN	No	16	69	LMG 24401	*Epilithonimonas*	*lactis*
Z0018_RB_C07_2_C09_A	GREEN	No	16	69	LMG 24401	*Epilithonimonas*	*lactis*
Z0018_LB_C12_2_C01_B	GREEN	No	16	69	LMG 24401	*Epilithonimonas*	*lactis*
Z0018_LB_D08_1_C03_B	GREEN	No	16	69	LMG 24401	*Epilithonimonas*	*lactis*
Z0018_LB_C09_2_C02_A	GREEN	No	16	69	LMG 24401	*Epilithonimonas*	*lactis*
Z0018_RB_B10_1_D08_B	GREEN	No	16	69	LMG 24401	*Epilithonimonas*	*lactis*
Z0018_RB_C04_2_C11_B	GREEN	No	16	69	LMG 24401	*Epilithonimonas*	*lactis*
Z0018_RB_C08_2_C09_B	GREEN	No	16	69	LMG 24401	*Epilithonimonas*	*lactis*
Z0018_RB_B11_1_D07_A	GREEN	No	16	69	LMG 24401	*Epilithonimonas*	*lactis*
Z0018_LB_D03_1_C05_A	GREEN	No	16	69	LMG 24401	*Epilithonimonas*	*lactis*
Z0018_RB_C06_2_C10_B	GREEN	No	16	69	LMG 24401	*Epilithonimonas*	*lactis*
Z0018_RB_B09_1_D08_A	GREEN	No	16	69	LMG 24401	*Epilithonimonas*	*lactis*
Z0018_RB_C05_2_C10_A	GREEN	No	16	69	LMG 24401	*Epilithonimonas*	*lactis*
Z0018_RB_B08_1_D09_B	GREEN	No	16	69	LMG 24401	*Epilithonimonas*	*lactis*
Z0018_LB_D04_1_C05_B	GREEN	No	16	69	LMG 24401	*Epilithonimonas*	*lactis*
Z0018_LB_C10_2_C02_B	GREEN	No	16	69	LMG 24401	*Epilithonimonas*	*lactis*
Z0018_RB_B12_1_D07_B	GREEN	No	16	69	LMG 24401	*Epilithonimonas*	*lactis*
Z0018_RB_C01_2_C12_A	GREEN	No	16	69	LMG 24401	*Epilithonimonas*	*lactis*
Z0018_LB_D09_1_C02_A	GREEN	No	16	69	LMG 24401	*Epilithonimonas*	*lactis*
Z0018_LB_C11_2_C01_A	GREEN	No	16	69	LMG 24401	*Epilithonimonas*	*lactis*
Z0019_LO_D10_2_F05_B	GREEN	Yes	17	85	LMG 26064	*Enterobacter*	*asburiae*
Z0019_LO_D06_2_F03_B	GREEN	No	17	85	LMG 26064	*Enterobacter*	*asburiae*
Z0019_RO_D02_2_F07_B	GREEN	No	17	85	LMG 26064	*Enterobacter*	*asburiae*
Z0019_RO_E10_1_G11_B	GREEN	No	17	85	LMG 26064	*Enterobacter*	*asburiae*
Z0019_LO_E04_1_G02_B	GREEN	No	17	85	LMG 26064	*Enterobacter*	*asburiae*
Z0019_RO_E06_1_G09_B	GREEN	No	17	85	LMG 26064	*Enterobacter*	*asburiae*
Z0019_RO_E12_1_G12_B	GREEN	No	17	85	LMG 26064	*Enterobacter*	*asburiae*
Z0019_LO_D09_2_F05_A	GREEN	No	17	85	LMG 26064	*Enterobacter*	*asburiae*
Z0019_RO_D01_2_F07_A	GREEN	No	17	85	LMG 26064	*Enterobacter*	*asburiae*
Z0019_RO_D08_2_F10_B	GREEN	No	17	85	LMG 26064	*Enterobacter*	*asburiae*
Z0019_RO_D07_2_F10_A	GREEN	No	17	85	LMG 26064	*Enterobacter*	*asburiae*
Z0019_RO_D03_2_F08_A	GREEN	No	17	85	LMG 26064	*Enterobacter*	*asburiae*
Z0019_RO_E01_1_G07_A	GREEN	No	17	85	LMG 26064	*Enterobacter*	*asburiae*
Z0019_RO_E04_1_G08_B	GREEN	No	17	85	LMG 26064	*Enterobacter*	*asburiae*
Z0019_RO_D06_2_F09_B	GREEN	No	17	85	LMG 26064	*Enterobacter*	*asburiae*
Z0019_LO_D05_2_F03_A	GREEN	No	17	85	LMG 26064	*Enterobacter*	*asburiae*
Z0019_RO_E08_1_G10_B	GREEN	No	17	85	LMG 26064	*Enterobacter*	*asburiae*
Z0019_LO_C12_1_F06_B	GREEN	No	17	85	LMG 26064	*Enterobacter*	*asburiae*
Z0019_LO_E02_1_G01_B	GREEN	No	17	85	LMG 26064	*Enterobacter*	*asburiae*
Z0019_RO_F08_2_G10_B	GREEN	No	17	85	LMG 26064	*Enterobacter*	*asburiae*
Z0019_RO_F07_2_G10_A	GREEN	No	17	85	LMG 26064	*Enterobacter*	*asburiae*
Z0019_RO_F05_2_G09_A	GREEN	No	17	85	LMG 26064	*Enterobacter*	*asburiae*
Z0019_RO_F06_2_G09_B	GREEN	No	17	85	LMG 26064	*Enterobacter*	*asburiae*
Z0019_LO_E06_1_G03_B	GREEN	No	17	85	LMG 26064	*Enterobacter*	*asburiae*
Z0019_LO_D12_2_F06_B	GREEN	No	17	85	LMG 26064	*Enterobacter*	*asburiae*
Z0019_RO_D09_2_F11_A	GREEN	No	17	85	LMG 26064	*Enterobacter*	*asburiae*
Z0019_RO_D12_2_F12_B	GREEN	No	17	85	LMG 26064	*Enterobacter*	*asburiae*
Z0019_RO_F01_2_G07_A	GREEN	No	17	85	LMG 26064	*Enterobacter*	*asburiae*
Z0019_RO_F04_2_G08_B	GREEN	No	17	85	LMG 26064	*Enterobacter*	*asburiae*
Z0019_RO_F02_2_G07_B	GREEN	No	17	85	LMG 26064	*Enterobacter*	*asburiae*
Z0019_LO_C11_1_F06_A	GREEN	No	17	85	LMG 26064	*Enterobacter*	*asburiae*
Z0019_RO_F03_2_G08_A	GREEN	No	17	85	LMG 26064	*Enterobacter*	*asburiae*

Z0023_LO_B06_2_E03_B	GREEN	Yes	18	121	LMG 5286	*Acidovorax*	*cattleyae*
Z0023_RO_A02_1_E07_B	GREEN	No	18	121	LMG 5286	*Acidovorax*	*cattleyae*
Z0023_RO_A08_1_E10_B	GREEN	No	18	121	LMG 5286	*Acidovorax*	*cattleyae*
Z0023_RB_H03_1_A11_A	GREEN	No	18	121	LMG 5286	*Acidovorax*	*cattleyae*
Z0023_LO_B01_2_E01_A	GREEN	No	18	121	LMG 5286	*Acidovorax*	*cattleyae*
Z0023_RB_H04_1_A11_B	GREEN	No	18	121	LMG 5286	*Acidovorax*	*cattleyae*
Z0023_LO_B09_2_E05_A	GREEN	No	18	121	LMG 5286	*Acidovorax*	*cattleyae*
Z0023_RO_A05_1_E09_A	GREEN	No	18	121	LMG 5286	*Acidovorax*	*cattleyae*
Z0023_LO_A09_1_E05_A	GREEN	No	18	121	LMG 5286	*Acidovorax*	*cattleyae*
Z0023_LO_B02_2_E01_B	GREEN	No	18	121	LMG 5286	*Acidovorax*	*cattleyae*
Z0023_RB_H12_1_A07_B	GREEN	No	18	121	LMG 5286	*Acidovorax*	*cattleyae*
Z0023_LO_A10_1_E05_B	GREEN	No	18	121	LMG 5286	*Acidovorax*	*cattleyae*
Z0023_RB_H09_1_A08_A	GREEN	No	18	121	LMG 5286	*Acidovorax*	*cattleyae*
Z0023_LO_B07_2_E04_A	GREEN	No	18	121	LMG 5286	*Acidovorax*	*cattleyae*
Z0023_LO_B05_2_E03_A	GREEN	No	18	121	LMG 5286	*Acidovorax*	*cattleyae*
Z0023_LO_A11_1_E06_A	GREEN	No	18	121	LMG 5286	*Acidovorax*	*cattleyae*
Z0023_RB_H07_1_A09_A	GREEN	No	18	121	LMG 5286	*Acidovorax*	*cattleyae*
Z0023_RO_A09_1_E11_A	GREEN	No	18	121	LMG 5286	*Acidovorax*	*cattleyae*
Z0023_RO_A03_1_E08_A	GREEN	No	18	121	LMG 5286	*Acidovorax*	*cattleyae*
Z0023_RB_H01_1_A12_A	GREEN	No	18	121	LMG 5286	*Acidovorax*	*cattleyae*
Z0023_RB_G12_2_A07_B	GREEN	No	18	121	LMG 5286	*Acidovorax*	*cattleyae*
Z0023_LO_A08_1_E04_B	GREEN	No	18	121	LMG 5286	*Acidovorax*	*cattleyae*
Z0023_LO_A07_1_E04_A	GREEN	No	18	121	LMG 5286	*Acidovorax*	*cattleyae*
Z0023_RB_H10_1_A08_B	GREEN	No	18	121	LMG 5286	*Acidovorax*	*cattleyae*
Z0023_LO_A12_1_E06_B	GREEN	No	18	121	LMG 5286	*Acidovorax*	*cattleyae*
Z0023_RB_H08_1_A09_B	GREEN	No	18	121	LMG 5286	*Acidovorax*	*cattleyae*
Z0023_LO_B04_2_E02_B	GREEN	No	18	121	LMG 5286	*Acidovorax*	*cattleyae*
Z0023_RB_H02_1_A12_B	GREEN	No	18	121	LMG 5286	*Acidovorax*	*cattleyae*
Z0017_LB_A06_2_D04_B	GREEN	Yes	19	55	LMG 23170	*Saccharibacter*	*floricola*
Z0017_LO_B10_2_E05_B	GREEN	No	19	55	LMG 23170	*Saccharibacter*	*floricola*
Z0017_LO_H05_2_H03_A	GREEN	No	19	55	LMG 23170	*Saccharibacter*	*floricola*
Z0017_LO_G12_1_H06_B	GREEN	No	19	55	LMG 23170	*Saccharibacter*	*floricola*
Z0017_LO_G10_1_H05_B	GREEN	No	19	55	LMG 23170	*Saccharibacter*	*floricola*
Z0017_RO_A06_1_E09_B	GREEN	No	19	55	LMG 23170	*Saccharibacter*	*floricola*
Z0017_LB_B01_1_D06_A	GREEN	No	19	55	LMG 23170	*Saccharibacter*	*floricola*
Z0017_LO_H06_2_H03_B	GREEN	No	19	55	LMG 23170	*Saccharibacter*	*floricola*
Z0017_LB_A05_2_D04_A	GREEN	No	19	55	LMG 23170	*Saccharibacter*	*floricola*
Z0017_LO_H09_2_H05_A	GREEN	No	19	55	LMG 23170	*Saccharibacter*	*floricola*
Z0017_LB_A07_2_D03_A	GREEN	No	19	55	LMG 23170	*Saccharibacter*	*floricola*
Z0017_LB_A08_2_D03_B	GREEN	No	19	55	LMG 23170	*Saccharibacter*	*floricola*
Z0017_LB_A09_2_D02_A	GREEN	No	19	55	LMG 23170	*Saccharibacter*	*floricola*
Z0017_LO_H07_2_H04_A	GREEN	No	19	55	LMG 23170	*Saccharibacter*	*floricola*
Z0017_LO_H11_2_H06_A	GREEN	No	19	55	LMG 23170	*Saccharibacter*	*floricola*
Z0017_RO_A12_1_E12_B	GREEN	No	19	55	LMG 23170	*Saccharibacter*	*floricola*
Z0017_RO_A10_1_E11_B	GREEN	No	19	55	LMG 23170	*Saccharibacter*	*floricola*
Z0017_LB_A02_2_D06_B	GREEN	No	19	55	LMG 23170	*Saccharibacter*	*floricola*
Z0017_RB_H10_1_A08_B	GREEN	No	19	55	LMG 23170	*Saccharibacter*	*floricola*
Z0017_LB_A12_2_D01_B	GREEN	No	19	55	LMG 23170	*Saccharibacter*	*floricola*
Z0017_LO_H08_2_H04_B	GREEN	No	19	55	LMG 23170	*Saccharibacter*	*floricola*
Z0017_LO_H01_2_H01_A	GREEN	No	19	55	LMG 23170	*Saccharibacter*	*floricola*
Z0017_LB_A01_2_D06_A	GREEN	No	19	55	LMG 23170	*Saccharibacter*	*floricola*
Z0017_LB_A11_2_D01_A	GREEN	No	19	55	LMG 23170	*Saccharibacter*	*floricola*
Z0017_LO_H02_2_H01_B	GREEN	No	19	55	LMG 23170	*Saccharibacter*	*floricola*
Z0017_LB_A10_2_D02_B	GREEN	No	19	55	LMG 23170	*Saccharibacter*	*floricola*
Z0017_LO_G11_1_H06_A	GREEN	No	19	55	LMG 23170	*Saccharibacter*	*floricola*
Z0017_LO_H10_2_H05_B	GREEN	No	19	55	LMG 23170	*Saccharibacter*	*floricola*
Z0022_LO_C10_1_F05_B	GREEN	Yes	20	110	LMG 3516	*Paenalcaligenes*	*hominis*
Z0022_LO_C08_1_F04_B	GREEN	No	20	110	LMG 3516	*Paenalcaligenes*	*hominis*
Z0022_LO_C12_1_F06_B	GREEN	No	20	110	LMG 3516	*Paenalcaligenes*	*hominis*
Z0022_LO_D05_2_F03_A	GREEN	No	20	110	LMG 3516	*Paenalcaligenes*	*hominis*
Z0022_RO_A12_1_E12_B	GREEN	No	20	110	LMG 3516	*Paenalcaligenes*	*hominis*
Z0022_RO_B02_2_E07_B	GREEN	No	20	110	LMG 3516	*Paenalcaligenes*	*hominis*
Z0022_LO_D01_2_F01_A	GREEN	No	20	110	LMG 3516	*Paenalcaligenes*	*hominis*
Z0022_LO_B06_2_E03_B	GREEN	No	20	110	LMG 3516	*Paenalcaligenes*	*hominis*
Z0022_LO_B07_2_E04_A	GREEN	No	20	110	LMG 3516	*Paenalcaligenes*	*hominis*
Z0022_LO_B05_2_E03_A	GREEN	No	20	110	LMG 3516	*Paenalcaligenes*	*hominis*
Z0022_LO_B12_2_E06_B	GREEN	No	20	110	LMG 3516	*Paenalcaligenes*	*hominis*
Z0022_LO_B03_2_E02_A	GREEN	No	20	110	LMG 3516	*Paenalcaligenes*	*hominis*
Z0022_LO_B11_2_E06_A	GREEN	No	20	110	LMG 3516	*Paenalcaligenes*	*hominis*
Z0022_RO_B05_2_E09_A	GREEN	No	20	110	LMG 3516	*Paenalcaligenes*	*hominis*

Z0022_LO_C06_1_F03_B	GREEN	No	20	110	LMG 3516	*Paenalcaligenes*	*hominis*
Z0022_LO_C05_1_F03_A	GREEN	No	20	110	LMG 3516	*Paenalcaligenes*	*hominis*
Z0022_LO_D04_2_F02_B	GREEN	No	20	110	LMG 3516	*Paenalcaligenes*	*hominis*
Z0022_LO_D02_2_F01_B	GREEN	No	20	110	LMG 3516	*Paenalcaligenes*	*hominis*
Z0022_LO_B04_2_E02_B	GREEN	No	20	110	LMG 3516	*Paenalcaligenes*	*hominis*
Z0022_LO_C07_1_F04_A	GREEN	No	20	110	LMG 3516	*Paenalcaligenes*	*hominis*
Z0022_RO_B06_2_E09_B	GREEN	No	20	110	LMG 3516	*Paenalcaligenes*	*hominis*
Z0022_LO_B09_2_E05_A	GREEN	No	20	110	LMG 3516	*Paenalcaligenes*	*hominis*
Z0022_RO_B08_2_E10_B	GREEN	No	20	110	LMG 3516	*Paenalcaligenes*	*hominis*
Z0022_RO_B07_2_E10_A	GREEN	No	20	110	LMG 3516	*Paenalcaligenes*	*hominis*
Z0022_LO_C11_1_F06_A	GREEN	No	20	110	LMG 3516	*Paenalcaligenes*	*hominis*
Z0022_LO_C09_1_F05_A	GREEN	No	20	110	LMG 3516	*Paenalcaligenes*	*hominis*
Z0022_LO_C04_1_F02_B	GREEN	No	20	110	LMG 3516	*Paenalcaligenes*	*hominis*
Z0022_LO_D03_2_F02_A	GREEN	No	20	110	LMG 3516	*Paenalcaligenes*	*hominis*
Z0012_RB_A09_2_D08_A	GREEN	Yes	21	3	LMG 11194	*Moraxella*	*canis*
Z0012_LB_A05_2_D04_A	GREEN	No	21	3	LMG 11194	*Moraxella*	*canis*
Z0012_LB_A08_2_D03_B	GREEN	No	21	3	LMG 11194	*Moraxella*	*canis*
Z0012_RB_B01_1_D12_A	GREEN	No	21	3	LMG 11194	*Moraxella*	*canis*
Z0012_LB_B03_1_D05_A	GREEN	No	21	3	LMG 11194	*Moraxella*	*canis*
Z0012_LB_B01_1_D06_A	GREEN	No	21	3	LMG 11194	*Moraxella*	*canis*
Z0012_LB_B04_1_D05_B	GREEN	No	21	3	LMG 11194	*Moraxella*	*canis*
Z0012_RB_B02_1_D12_B	GREEN	No	21	3	LMG 11194	*Moraxella*	*canis*
Z0012_RB_B03_1_D11_A	GREEN	No	21	3	LMG 11194	*Moraxella*	*canis*
Z0012_RB_A11_2_D07_A	GREEN	No	21	3	LMG 11194	*Moraxella*	*canis*
Z0012_RB_A10_2_D08_B	GREEN	No	21	3	LMG 11194	*Moraxella*	*canis*
Z0012_RB_A04_2_D11_B	GREEN	No	21	3	LMG 11194	*Moraxella*	*canis*
Z0012_LB_A11_2_D01_A	GREEN	No	21	3	LMG 11194	*Moraxella*	*canis*
Z0012_RB_A12_2_D07_B	GREEN	No	21	3	LMG 11194	*Moraxella*	*canis*
Z0012_LB_A04_2_D05_B	GREEN	No	21	3	LMG 11194	*Moraxella*	*canis*
Z0012_LB_B02_1_D06_B	GREEN	No	21	3	LMG 11194	*Moraxella*	*canis*
Z0012_LB_A09_2_D02_A	GREEN	No	21	3	LMG 11194	*Moraxella*	*canis*
Z0012_RB_A06_2_D10_B	GREEN	No	21	3	LMG 11194	*Moraxella*	*canis*
Z0012_LB_A12_2_D01_B	GREEN	No	21	3	LMG 11194	*Moraxella*	*canis*
Z0012_LB_A01_2_D06_A	GREEN	No	21	3	LMG 11194	*Moraxella*	*canis*
Z0012_LB_A07_2_D03_A	GREEN	No	21	3	LMG 11194	*Moraxella*	*canis*
Z0012_RB_F08_1_B09_B	GREEN	No	21	3	LMG 11194	*Moraxella*	*canis*
Z0012_LB_A06_2_D04_B	GREEN	No	21	3	LMG 11194	*Moraxella*	*canis*
Z0012_LB_A10_2_D02_B	GREEN	No	21	3	LMG 11194	*Moraxella*	*canis*
Z0012_LB_A03_2_D05_A	GREEN	No	21	3	LMG 11194	*Moraxella*	*canis*
Z0012_LB_A02_2_D06_B	GREEN	No	21	3	LMG 11194	*Moraxella*	*canis*
Z0012_RB_A07_2_D09_A	GREEN	No	21	3	LMG 11194	*Moraxella*	*canis*
Z0012_RB_A08_2_D09_B	GREEN	No	21	3	LMG 11194	*Moraxella*	*canis*
Z0012_RB_A05_2_D10_A	GREEN	No	21	3	LMG 11194	*Moraxella*	*canis*
Z0012_RB_F07_1_B09_A	GREEN	No	21	3	LMG 11194	*Moraxella*	*canis*
Z0012_LB_F11_1_B01_A	GREEN	No	21	3	LMG 11194	*Moraxella*	*canis*
Z0012_RB_F05_1_B10_A	GREEN	No	21	3	LMG 11194	*Moraxella*	*canis*
Z0012_LO_B10_2_E05_B	GREEN	Yes	22	12	LMG 129	*Thalassobius*	*gelatinovorus*
Z0012_LO_A12_1_E06_B	GREEN	No	22	12	LMG 129	*Thalassobius*	*gelatinovorus*
Z0012_RO_B12_2_E12_B	GREEN	No	22	12	LMG 129	*Thalassobius*	*gelatinovorus*
Z0012_RO_B06_2_E09_B	GREEN	No	22	12	LMG 129	*Thalassobius*	*gelatinovorus*
Z0012_RO_B02_2_E07_B	GREEN	No	22	12	LMG 129	*Thalassobius*	*gelatinovorus*
Z0012_LO_C12_1_F06_B	GREEN	No	22	12	LMG 129	*Thalassobius*	*gelatinovorus*
Z0012_RO_C04_1_F08_B	GREEN	No	22	12	LMG 129	*Thalassobius*	*gelatinovorus*
Z0012_LO_A03_1_E02_A	GREEN	No	22	12	LMG 129	*Thalassobius*	*gelatinovorus*
Z0012_LO_C07_1_F04_A	GREEN	No	22	12	LMG 129	*Thalassobius*	*gelatinovorus*
Z0012_RO_B04_2_E08_B	GREEN	No	22	12	LMG 129	*Thalassobius*	*gelatinovorus*
Z0012_LO_B07_2_E04_A	GREEN	No	22	12	LMG 129	*Thalassobius*	*gelatinovorus*
Z0012_LO_C04_1_F02_B	GREEN	No	22	12	LMG 129	*Thalassobius*	*gelatinovorus*
Z0012_LO_B11_2_E06_A	GREEN	No	22	12	LMG 129	*Thalassobius*	*gelatinovorus*
Z0012_RO_C08_1_F10_B	GREEN	No	22	12	LMG 129	*Thalassobius*	*gelatinovorus*
Z0012_LO_D01_2_F01_A	GREEN	No	22	12	LMG 129	*Thalassobius*	*gelatinovorus*
Z0012_LO_C02_1_F01_B	GREEN	No	22	12	LMG 129	*Thalassobius*	*gelatinovorus*
Z0012_LO_C01_1_F01_A	GREEN	No	22	12	LMG 129	*Thalassobius*	*gelatinovorus*
Z0012_LO_B05_2_E03_A	GREEN	No	22	12	LMG 129	*Thalassobius*	*gelatinovorus*
Z0012_LO_A04_1_E02_B	GREEN	No	22	12	LMG 129	*Thalassobius*	*gelatinovorus*
Z0012_RO_C09_1_F11_A	GREEN	No	22	12	LMG 129	*Thalassobius*	*gelatinovorus*
Z0012_RO_C05_1_F09_A	GREEN	No	22	12	LMG 129	*Thalassobius*	*gelatinovorus*
Z0012_RO_C02_1_F07_B	GREEN	No	22	12	LMG 129	*Thalassobius*	*gelatinovorus*
Z0012_LO_B04_2_E02_B	GREEN	No	22	12	LMG 129	*Thalassobius*	*gelatinovorus*
Z0012_RO_B11_2_E12_A	GREEN	No	22	12	LMG 129	*Thalassobius*	*gelatinovorus*

Z0012_LO_A10_1_E05_B	GREEN	No	22	12	LMG 129	*Thalassobius*	*gelatinovorus*	
Z0012_LO_A08_1_E04_B	GREEN	No	22	12	LMG 129	*Thalassobius*	*gelatinovorus*	
Z0012_LO_B09_2_E05_A	GREEN	No	22	12	LMG 129	*Thalassobius*	*gelatinovorus*	
Z0012_LO_A02_1_E01_B	GREEN	No	22	12	LMG 129	*Thalassobius*	*gelatinovorus*	
Z0012_LO_B12_2_E06_B	GREEN	No	22	12	LMG 129	*Thalassobius*	*gelatinovorus*	
Z0012_LO_A06_1_E03_B	GREEN	No	22	12	LMG 129	*Thalassobius*	*gelatinovorus*	
Z0012_LO_C10_1_F05_B	GREEN	No	22	12	LMG 129	*Thalassobius*	*gelatinovorus*	
Z0012_RO_B08_2_E10_B	GREEN	No	22	12	LMG 129	*Thalassobius*	*gelatinovorus*	
Z0024_RB_A06_2_D10_B	GREEN	Yes	23	131	LMG 7135	*Bacillus*	*subtilis*	*subtilis*
Z0024_RO_G04_1_H08_B	GREEN	No	23	131	LMG 7135	*Bacillus*	*subtilis*	*subtilis*
Z0024_LO_H08_2_H04_B	GREEN	No	23	131	LMG 7135	*Bacillus*	*subtilis*	*subtilis*
Z0024_RB_A11_2_D07_A	GREEN	No	23	131	LMG 7135	*Bacillus*	*subtilis*	*subtilis*
Z0024_LO_H07_2_H04_A	GREEN	No	23	131	LMG 7135	*Bacillus*	*subtilis*	*subtilis*
Z0024_RB_A09_2_D08_A	GREEN	No	23	131	LMG 7135	*Bacillus*	*subtilis*	*subtilis*
Z0024_RO_F12_2_G12_B	GREEN	No	23	131	LMG 7135	*Bacillus*	*subtilis*	*subtilis*
Z0024_RO_G03_1_H08_A	GREEN	No	23	131	LMG 7135	*Bacillus*	*subtilis*	*subtilis*
Z0024_RB_B02_1_D12_B	GREEN	No	23	131	LMG 7135	*Bacillus*	*subtilis*	*subtilis*
Z0024_RO_G11_1_H12_A	GREEN	No	23	131	LMG 7135	*Bacillus*	*subtilis*	*subtilis*
Z0024_RO_H03_2_H08_A	GREEN	No	23	131	LMG 7135	*Bacillus*	*subtilis*	*subtilis*
Z0024_RB_A08_2_D09_B	GREEN	No	23	131	LMG 7135	*Bacillus*	*subtilis*	*subtilis*
Z0024_RO_G01_1_H07_A	GREEN	No	23	131	LMG 7135	*Bacillus*	*subtilis*	*subtilis*
Z0024_LO_H09_2_H05_A	GREEN	No	23	131	LMG 7135	*Bacillus*	*subtilis*	*subtilis*
Z0024_RO_G06_1_H09_B	GREEN	No	23	131	LMG 7135	*Bacillus*	*subtilis*	*subtilis*
Z0024_LO_H12_2_H06_B	GREEN	No	23	131	LMG 7135	*Bacillus*	*subtilis*	*subtilis*
Z0024_RB_A10_2_D08_B	GREEN	No	23	131	LMG 7135	*Bacillus*	*subtilis*	*subtilis*
Z0024_RO_H04_2_H08_B	GREEN	No	23	131	LMG 7135	*Bacillus*	*subtilis*	*subtilis*
Z0024_RO_G08_1_H10_B	GREEN	No	23	131	LMG 7135	*Bacillus*	*subtilis*	*subtilis*
Z0024_RO_H02_2_H07_B	GREEN	No	23	131	LMG 7135	*Bacillus*	*subtilis*	*subtilis*
Z0024_RB_A05_2_D10_A	GREEN	No	23	131	LMG 7135	*Bacillus*	*subtilis*	*subtilis*
Z0024_RB_A01_2_D12_A	GREEN	No	23	131	LMG 7135	*Bacillus*	*subtilis*	*subtilis*
Z0024_RB_A12_2_D07_B	GREEN	No	23	131	LMG 7135	*Bacillus*	*subtilis*	*subtilis*
Z0024_RB_B01_1_D12_A	GREEN	No	23	131	LMG 7135	*Bacillus*	*subtilis*	*subtilis*
Z0024_RB_A02_2_D12_B	GREEN	No	23	131	LMG 7135	*Bacillus*	*subtilis*	*subtilis*
Z0024_RO_G10_1_H11_B	GREEN	No	23	131	LMG 7135	*Bacillus*	*subtilis*	*subtilis*
Z0024_RB_A03_2_D11_A	GREEN	No	23	131	LMG 7135	*Bacillus*	*subtilis*	*subtilis*
Z0024_RB_A07_2_D09_A	GREEN	No	23	131	LMG 7135	*Bacillus*	*subtilis*	*subtilis*
Z0024_RO_G07_1_H10_A	GREEN	No	23	131	LMG 7135	*Bacillus*	*subtilis*	*subtilis*
Z0024_RO_H01_2_H07_A	GREEN	No	23	131	LMG 7135	*Bacillus*	*subtilis*	*subtilis*
Z0024_RO_G12_1_H12_B	GREEN	No	23	131	LMG 7135	*Bacillus*	*subtilis*	*subtilis*
Z0024_RB_A04_2_D11_B	GREEN	No	23	131	LMG 7135	*Bacillus*	*subtilis*	*subtilis*
Z0013_RB_H10_1_A08_B	GREEN	Yes	24	14	LMG 13349	*Staphylococcus*	*haemolyticus*	
Z0013_LO_H09_2_H05_A	GREEN	No	24	14	LMG 13349	*Staphylococcus*	*haemolyticus*	
Z0013_LO_A02_1_E01_B	GREEN	No	24	14	LMG 13349	*Staphylococcus*	*haemolyticus*	
Z0013_LB_F03_1_B05_A	GREEN	No	24	14	LMG 13349	*Staphylococcus*	*haemolyticus*	
Z0013_LO_H11_2_H06_A	GREEN	No	24	14	LMG 13349	*Staphylococcus*	*haemolyticus*	
Z0013_LB_A05_2_D04_A	GREEN	No	24	14	LMG 13349	*Staphylococcus*	*haemolyticus*	
Z0013_LO_A05_1_E03_A	GREEN	No	24	14	LMG 13349	*Staphylococcus*	*haemolyticus*	
Z0013_LO_H10_2_H05_B	GREEN	No	24	14	LMG 13349	*Staphylococcus*	*haemolyticus*	
Z0013_LB_A04_2_D05_B	GREEN	No	24	14	LMG 13349	*Staphylococcus*	*haemolyticus*	
Z0013_RB_A01_2_D12_A	GREEN	No	24	14	LMG 13349	*Staphylococcus*	*haemolyticus*	
Z0013_LO_A01_1_E01_A	GREEN	No	24	14	LMG 13349	*Staphylococcus*	*haemolyticus*	
Z0013_LB_H12_1_A01_B	GREEN	No	24	14	LMG 13349	*Staphylococcus*	*haemolyticus*	
Z0013_LO_H12_2_H06_B	GREEN	No	24	14	LMG 13349	*Staphylococcus*	*haemolyticus*	
Z0013_RB_H07_1_A09_A	GREEN	No	24	14	LMG 13349	*Staphylococcus*	*haemolyticus*	
Z0013_LB_A01_2_D06_A	GREEN	No	24	14	LMG 13349	*Staphylococcus*	*haemolyticus*	
Z0013_LO_F02_2_G01_B	GREEN	No	24	14	LMG 13349	*Staphylococcus*	*haemolyticus*	
Z0013_LB_A06_2_D04_B	GREEN	No	24	14	LMG 13349	*Staphylococcus*	*haemolyticus*	
Z0013_RB_H11_1_A07_A	GREEN	No	24	14	LMG 13349	*Staphylococcus*	*haemolyticus*	
Z0013_RO_F05_2_G09_A	GREEN	No	24	14	LMG 13349	*Staphylococcus*	*haemolyticus*	
Z0013_LO_A03_1_E02_A	GREEN	No	24	14	LMG 13349	*Staphylococcus*	*haemolyticus*	
Z0013_LO_A04_1_E02_B	GREEN	No	24	14	LMG 13349	*Staphylococcus*	*haemolyticus*	
Z0013_LO_F03_2_G02_A	GREEN	No	24	14	LMG 13349	*Staphylococcus*	*haemolyticus*	
Z0013_LB_H09_1_A02_A	GREEN	No	24	14	LMG 13349	*Staphylococcus*	*haemolyticus*	
Z0013_RB_E08_2_B09_B	GREEN	No	24	14	LMG 13349	*Staphylococcus*	*haemolyticus*	
Z0013_RB_H09_1_A08_A	GREEN	No	24	14	LMG 13349	*Staphylococcus*	*haemolyticus*	
Z0013_RB_H08_1_A09_B	GREEN	No	24	14	LMG 13349	*Staphylococcus*	*haemolyticus*	
Z0013_LB_A03_2_D05_A	GREEN	No	24	14	LMG 13349	*Staphylococcus*	*haemolyticus*	
Z0013_LB_A02_2_D06_B	GREEN	No	24	14	LMG 13349	*Staphylococcus*	*haemolyticus*	
Z0013_RB_A02_2_D12_B	GREEN	No	24	14	LMG 13349	*Staphylococcus*	*haemolyticus*	
Z0013_LB_H10_1_A02_B	GREEN	No	24	14	LMG 13349	*Staphylococcus*	*haemolyticus*	

Z0013_RB_H12_1_A07_B	GREEN	No	24	14	LMG 13349	*Staphylococcus*	*haemolyticus*
Z0013_LB_H11_1_A01_A	GREEN	No	24	14	LMG 13349	*Staphylococcus*	*haemolyticus*
Z0024_LO_C08_1_F04_B	GREEN	Yes	25	129	LMG 6928	*Sporosarcina*	*globispora*
Z0024_LO_D05_2_F03_A	GREEN	No	25	129	LMG 6928	*Sporosarcina*	*globispora*
Z0024_RO_B05_2_E09_A	GREEN	No	25	129	LMG 6928	*Sporosarcina*	*globispora*
Z0024_RO_B03_2_E08_A	GREEN	No	25	129	LMG 6928	*Sporosarcina*	*globispora*
Z0024_LO_D01_2_F01_A	GREEN	No	25	129	LMG 6928	*Sporosarcina*	*globispora*
Z0024_RO_B10_2_E11_B	GREEN	No	25	129	LMG 6928	*Sporosarcina*	*globispora*
Z0024_RO_B06_2_E09_B	GREEN	No	25	129	LMG 6928	*Sporosarcina*	*globispora*
Z0024_RO_B12_2_E12_B	GREEN	No	25	129	LMG 6928	*Sporosarcina*	*globispora*
Z0024_LO_D07_2_F04_A	GREEN	No	25	129	LMG 6928	*Sporosarcina*	*globispora*
Z0024_RO_B08_2_E10_B	GREEN	No	25	129	LMG 6928	*Sporosarcina*	*globispora*
Z0024_RO_B01_2_E07_A	GREEN	No	25	129	LMG 6928	*Sporosarcina*	*globispora*
Z0024_RO_B02_2_E07_B	GREEN	No	25	129	LMG 6928	*Sporosarcina*	*globispora*
Z0024_LO_C10_1_F05_B	GREEN	No	25	129	LMG 6928	*Sporosarcina*	*globispora*
Z0024_LO_E09_1_G05_A	GREEN	No	25	129	LMG 6928	*Sporosarcina*	*globispora*
Z0024_RO_C08_1_F10_B	GREEN	No	25	129	LMG 6928	*Sporosarcina*	*globispora*
Z0024_LO_E04_1_G02_B	GREEN	No	25	129	LMG 6928	*Sporosarcina*	*globispora*
Z0024_LO_D12_2_F06_B	GREEN	No	25	129	LMG 6928	*Sporosarcina*	*globispora*
Z0024_LO_D10_2_F05_B	GREEN	No	25	129	LMG 6928	*Sporosarcina*	*globispora*
Z0024_RO_C11_1_F12_A	GREEN	No	25	129	LMG 6928	*Sporosarcina*	*globispora*
Z0024_LO_E01_1_G01_A	GREEN	No	25	129	LMG 6928	*Sporosarcina*	*globispora*
Z0024_RO_C01_1_F07_A	GREEN	No	25	129	LMG 6928	*Sporosarcina*	*globispora*
Z0024_RO_C06_1_F09_B	GREEN	No	25	129	LMG 6928	*Sporosarcina*	*globispora*
Z0024_LO_C12_1_F06_B	GREEN	No	25	129	LMG 6928	*Sporosarcina*	*globispora*
Z0024_RO_C10_1_F11_B	GREEN	No	25	129	LMG 6928	*Sporosarcina*	*globispora*
Z0024_LO_E06_1_G03_B	GREEN	No	25	129	LMG 6928	*Sporosarcina*	*globispora*
Z0024_LO_D03_2_F02_A	GREEN	No	25	129	LMG 6928	*Sporosarcina*	*globispora*
Z0024_LO_E02_1_G01_B	GREEN	No	25	129	LMG 6928	*Sporosarcina*	*globispora*
Z0024_RO_C03_1_F08_A	GREEN	No	25	129	LMG 6928	*Sporosarcina*	*globispora*
Z0025_LB_H01_1_A06_A	GREEN	Yes	26	137	LMG 8760	*Lysobacter*	*antibioticus*
Z0025_LB_H09_1_A02_A	GREEN	Yes	27	137	LMG 8760	*Lysobacter*	*antibioticus*
Z0025_RB_H06_1_A10_B	GREEN	No	27	137	LMG 8760	*Lysobacter*	*antibioticus*
Z0025_RB_G10_2_A08_B	GREEN	No	27	137	LMG 8760	*Lysobacter*	*antibioticus*
Z0025_RB_H03_1_A11_A	GREEN	No	27	137	LMG 8760	*Lysobacter*	*antibioticus*
Z0025_LB_H03_1_A05_A	GREEN	No	27	137	LMG 8760	*Lysobacter*	*antibioticus*
Z0025_LB_H07_1_A03_A	GREEN	No	27	137	LMG 8760	*Lysobacter*	*antibioticus*
Z0025_LB_H06_1_A04_B	GREEN	No	27	137	LMG 8760	*Lysobacter*	*antibioticus*
Z0025_LB_H04_1_A05_B	GREEN	No	27	137	LMG 8760	*Lysobacter*	*antibioticus*
Z0025_RB_H05_1_A10_A	GREEN	No	27	137	LMG 8760	*Lysobacter*	*antibioticus*
Z0025_RB_H07_1_A09_A	GREEN	No	27	137	LMG 8760	*Lysobacter*	*antibioticus*
Z0025_LB_G08_2_A03_B	GREEN	No	27	137	LMG 8760	*Lysobacter*	*antibioticus*
Z0025_RB_H10_1_A08_B	GREEN	No	27	137	LMG 8760	*Lysobacter*	*antibioticus*
Z0025_RB_H09_1_A08_A	GREEN	No	27	137	LMG 8760	*Lysobacter*	*antibioticus*
Z0025_RB_G11_2_A07_A	GREEN	No	27	137	LMG 8760	*Lysobacter*	*antibioticus*
Z0025_LB_H02_1_A06_B	GREEN	No	27	137	LMG 8760	*Lysobacter*	*antibioticus*
Z0025_RB_H12_1_A07_B	GREEN	No	27	137	LMG 8760	*Lysobacter*	*antibioticus*
Z0025_RB_H01_1_A12_A	GREEN	No	27	137	LMG 8760	*Lysobacter*	*antibioticus*
Z0025_LB_G11_2_A01_A	GREEN	No	27	137	LMG 8760	*Lysobacter*	*antibioticus*
Z0025_RB_G09_2_A08_A	GREEN	No	27	137	LMG 8760	*Lysobacter*	*antibioticus*
Z0025_LB_G10_2_A02_B	GREEN	No	27	137	LMG 8760	*Lysobacter*	*antibioticus*
Z0025_LB_H05_1_A04_A	GREEN	No	27	137	LMG 8760	*Lysobacter*	*antibioticus*
Z0025_RB_G12_2_A07_B	GREEN	No	27	137	LMG 8760	*Lysobacter*	*antibioticus*
Z0025_RB_G08_2_A09_B	GREEN	No	27	137	LMG 8760	*Lysobacter*	*antibioticus*
Z0025_LB_H11_1_A01_A	GREEN	No	27	137	LMG 8760	*Lysobacter*	*antibioticus*
Z0025_RB_H11_1_A07_A	GREEN	No	27	137	LMG 8760	*Lysobacter*	*antibioticus*
Z0025_RB_H04_1_A11_B	GREEN	No	27	137	LMG 8760	*Lysobacter*	*antibioticus*
Z0025_LB_G12_2_A01_B	GREEN	No	27	137	LMG 8760	*Lysobacter*	*antibioticus*
Z0025_LB_G09_2_A02_A	GREEN	No	27	137	LMG 8760	*Lysobacter*	*antibioticus*
Z0025_LB_H08_1_A03_B	GREEN	No	27	137	LMG 8760	*Lysobacter*	*antibioticus*
Z0025_RB_H02_1_A12_B	GREEN	No	27	137	LMG 8760	*Lysobacter*	*antibioticus*
Z0025_RB_H08_1_A09_B	GREEN	No	27	137	LMG 8760	*Lysobacter*	*antibioticus*
Z0024_RB_E11_2_B07_A	GREEN	Yes	28	126	LMG 6896	*Streptococcus*	*thermophilus*
Z0024_LB_F02_1_B06_B	GREEN	No	28	126	LMG 6896	*Streptococcus*	*thermophilus*
Z0024_LB_F03_1_B05_A	GREEN	No	28	126	LMG 6896	*Streptococcus*	*thermophilus*
Z0024_RB_F03_1_B11_A	GREEN	No	28	126	LMG 6896	*Streptococcus*	*thermophilus*
Z0024_LB_E08_2_B03_B	GREEN	No	28	126	LMG 6896	*Streptococcus*	*thermophilus*
Z0024_LB_F04_1_B05_B	GREEN	No	28	126	LMG 6896	*Streptococcus*	*thermophilus*
Z0024_RB_E12_2_B07_B	GREEN	No	28	126	LMG 6896	*Streptococcus*	*thermophilus*
Z0024_LB_F06_1_B04_B	GREEN	No	28	126	LMG 6896	*Streptococcus*	*thermophilus*

Z0024_LB_E07_2_B03_A	GREEN	No	28	126	LMG 6896	*Streptococcus*	*thermophilus*
Z0024_RB_F01_1_B12_A	GREEN	No	28	126	LMG 6896	*Streptococcus*	*thermophilus*
Z0024_LB_E12_2_B01_B	GREEN	No	28	126	LMG 6896	*Streptococcus*	*thermophilus*
Z0024_LB_E06_2_B04_B	GREEN	No	28	126	LMG 6896	*Streptococcus*	*thermophilus*
Z0024_RB_E08_2_B09_B	GREEN	No	28	126	LMG 6896	*Streptococcus*	*thermophilus*
Z0024_LB_F01_1_B06_A	GREEN	No	28	126	LMG 6896	*Streptococcus*	*thermophilus*
Z0024_RB_E10_2_B08_B	GREEN	No	28	126	LMG 6896	*Streptococcus*	*thermophilus*
Z0024_RB_F06_1_B10_B	GREEN	No	28	126	LMG 6896	*Streptococcus*	*thermophilus*
Z0024_LB_F07_1_B03_A	GREEN	No	28	126	LMG 6896	*Streptococcus*	*thermophilus*
Z0024_RB_F02_1_B12_B	GREEN	No	28	126	LMG 6896	*Streptococcus*	*thermophilus*
Z0024_LB_E05_2_B04_A	GREEN	No	28	126	LMG 6896	*Streptococcus*	*thermophilus*
Z0024_LB_F05_1_B04_A	GREEN	No	28	126	LMG 6896	*Streptococcus*	*thermophilus*
Z0024_RB_F08_1_B09_B	GREEN	No	28	126	LMG 6896	*Streptococcus*	*thermophilus*
Z0024_RB_E09_2_B08_A	GREEN	No	28	126	LMG 6896	*Streptococcus*	*thermophilus*
Z0024_RB_E07_2_B09_A	GREEN	No	28	126	LMG 6896	*Streptococcus*	*thermophilus*
Z0024_LB_E11_2_B01_A	GREEN	No	28	126	LMG 6896	*Streptococcus*	*thermophilus*
Z0024_RB_F07_1_B09_A	GREEN	No	28	126	LMG 6896	*Streptococcus*	*thermophilus*
Z0024_LB_E04_2_B05_B	GREEN	No	28	126	LMG 6896	*Streptococcus*	*thermophilus*
Z0024_LB_E03_2_B05_A	GREEN	No	28	126	LMG 6896	*Streptococcus*	*thermophilus*
Z0024_LB_D07_1_C03_A	GREEN	No	28	126	LMG 6896	*Streptococcus*	*thermophilus*
Z0024_LB_E10_2_B02_B	GREEN	No	28	126	LMG 6896	*Streptococcus*	*thermophilus*
Z0024_LB_E09_2_B02_A	GREEN	No	28	126	LMG 6896	*Streptococcus*	*thermophilus*
Z0024_RB_F05_1_B10_A	GREEN	No	28	126	LMG 6896	*Streptococcus*	*thermophilus*
Z0024_RB_F04_1_B11_B	GREEN	No	28	126	LMG 6896	*Streptococcus*	*thermophilus*
Z0010_RB_F01_1_B12_A	GREEN	Yes	29	143	R-69593	*Burkholderia*	*vietnamiensis*
Z0010_LB_F09_1_B02_A	GREEN	Yes	30	143	R-69593	*Burkholderia*	*vietnamiensis*
Z0010_LB_G03_2_A05_A	GREEN	No	30	143	R-69593	*Burkholderia*	*vietnamiensis*
Z0010_LB_F07_1_B03_A	GREEN	No	30	143	R-69593	*Burkholderia*	*vietnamiensis*
Z0010_LB_G10_2_A02_B	GREEN	No	30	143	R-69593	*Burkholderia*	*vietnamiensis*
Z0010_RB_E12_2_B07_B	GREEN	No	30	143	R-69593	*Burkholderia*	*vietnamiensis*
Z0010_LB_G05_2_A04_A	GREEN	No	30	143	R-69593	*Burkholderia*	*vietnamiensis*
Z0010_LB_G04_2_A05_B	GREEN	No	30	143	R-69593	*Burkholderia*	*vietnamiensis*
Z0010_LB_G06_2_A04_B	GREEN	No	30	143	R-69593	*Burkholderia*	*vietnamiensis*
Z0010_LB_G08_2_A03_B	GREEN	No	30	143	R-69593	*Burkholderia*	*vietnamiensis*
Z0010_RB_F09_1_B08_A	GREEN	No	30	143	R-69593	*Burkholderia*	*vietnamiensis*
Z0010_RB_E09_2_B08_A	GREEN	No	30	143	R-69593	*Burkholderia*	*vietnamiensis*
Z0010_RB_F08_1_B09_B	GREEN	No	30	143	R-69593	*Burkholderia*	*vietnamiensis*
Z0010_RB_F07_1_B09_A	GREEN	No	30	143	R-69593	*Burkholderia*	*vietnamiensis*
Z0010_RB_F02_1_B12_B	GREEN	No	30	143	R-69593	*Burkholderia*	*vietnamiensis*
Z0010_RB_E10_2_B08_B	GREEN	No	30	143	R-69593	*Burkholderia*	*vietnamiensis*
Z0010_RB_F04_1_B11_B	GREEN	No	30	143	R-69593	*Burkholderia*	*vietnamiensis*
Z0010_LB_F10_1_B02_B	GREEN	No	30	143	R-69593	*Burkholderia*	*vietnamiensis*
Z0010_RB_F06_1_B10_B	GREEN	No	30	143	R-69593	*Burkholderia*	*vietnamiensis*
Z0010_RB_F03_1_B11_A	GREEN	No	30	143	R-69593	*Burkholderia*	*vietnamiensis*
Z0010_RB_E11_2_B07_A	GREEN	No	30	143	R-69593	*Burkholderia*	*vietnamiensis*
Z0010_LB_G09_2_A02_A	GREEN	No	30	143	R-69593	*Burkholderia*	*vietnamiensis*
Z0010_RB_F05_1_B10_A	GREEN	No	30	143	R-69593	*Burkholderia*	*vietnamiensis*
Z0010_RB_F10_1_B08_B	GREEN	No	30	143	R-69593	*Burkholderia*	*vietnamiensis*
Z0010_LB_F11_1_B01_A	GREEN	No	30	143	R-69593	*Burkholderia*	*vietnamiensis*
Z0010_LB_F08_1_B03_B	GREEN	No	30	143	R-69593	*Burkholderia*	*vietnamiensis*
Z0010_LB_G02_2_A06_B	GREEN	No	30	143	R-69593	*Burkholderia*	*vietnamiensis*
Z0010_LB_G01_2_A06_A	GREEN	No	30	143	R-69593	*Burkholderia*	*vietnamiensis*
Z0010_LB_G11_2_A01_A	GREEN	No	30	143	R-69593	*Burkholderia*	*vietnamiensis*
Z0010_LB_G07_2_A03_A	GREEN	No	30	143	R-69593	*Burkholderia*	*vietnamiensis*
Z0010_LB_F12_1_B01_B	GREEN	No	30	143	R-69593	*Burkholderia*	*vietnamiensis*
Z0010_RB_E08_2_B09_B	GREEN	No	30	143	R-69593	*Burkholderia*	*vietnamiensis*
Z0017_LO_A03_1_E02_A	GREEN	Yes	31	66	LMG 24163	*Pseudoxanthomonas*	*dokdonensis*
Z0017_LO_C04_1_F02_B	GREEN	No	31	66	LMG 24163	*Pseudoxanthomonas*	*dokdonensis*
Z0017_LO_C07_1_F04_A	GREEN	No	31	66	LMG 24163	*Pseudoxanthomonas*	*dokdonensis*
Z0017_RO_A01_1_E07_A	GREEN	No	31	66	LMG 24163	*Pseudoxanthomonas*	*dokdonensis*
Z0017_RO_B06_2_E09_B	GREEN	No	31	66	LMG 24163	*Pseudoxanthomonas*	*dokdonensis*
Z0017_RO_C04_1_F08_B	GREEN	No	31	66	LMG 24163	*Pseudoxanthomonas*	*dokdonensis*
Z0017_LO_A04_1_E02_B	GREEN	No	31	66	LMG 24163	*Pseudoxanthomonas*	*dokdonensis*
Z0017_RB_H09_1_A08_A	GREEN	No	31	66	LMG 24163	*Pseudoxanthomonas*	*dokdonensis*
Z0017_LO_C09_1_F05_A	GREEN	No	31	66	LMG 24163	*Pseudoxanthomonas*	*dokdonensis*
Z0017_LO_D02_2_F01_B	GREEN	No	31	66	LMG 24163	*Pseudoxanthomonas*	*dokdonensis*
Z0017_LO_A01_1_E01_A	GREEN	No	31	66	LMG 24163	*Pseudoxanthomonas*	*dokdonensis*
Z0017_RO_C08_1_F10_B	GREEN	No	31	66	LMG 24163	*Pseudoxanthomonas*	*dokdonensis*
Z0017_LO_C01_1_F01_A	GREEN	No	31	66	LMG 24163	*Pseudoxanthomonas*	*dokdonensis*
Z0017_RO_C06_1_F09_B	GREEN	No	31	66	LMG 24163	*Pseudoxanthomonas*	*dokdonensis*

Z0017_RO_C11_1_F12_A	GREEN	No	31	66	LMG 24163	*Pseudoxanthomonas*	*dokdonensis*
Z0017_LO_B05_2_E03_A	GREEN	No	31	66	LMG 24163	*Pseudoxanthomonas*	*dokdonensis*
Z0017_RO_B05_2_E09_A	GREEN	No	31	66	LMG 24163	*Pseudoxanthomonas*	*dokdonensis*
Z0017_LO_B06_2_E03_B	GREEN	No	31	66	LMG 24163	*Pseudoxanthomonas*	*dokdonensis*
Z0017_RO_B03_2_E08_A	GREEN	No	31	66	LMG 24163	*Pseudoxanthomonas*	*dokdonensis*
Z0017_LO_B02_2_E01_B	GREEN	No	31	66	LMG 24163	*Pseudoxanthomonas*	*dokdonensis*
Z0017_RO_B10_2_E11_B	GREEN	No	31	66	LMG 24163	*Pseudoxanthomonas*	*dokdonensis*
Z0017_RO_C09_1_F11_A	GREEN	No	31	66	LMG 24163	*Pseudoxanthomonas*	*dokdonensis*
Z0017_RO_B04_2_E08_B	GREEN	No	31	66	LMG 24163	*Pseudoxanthomonas*	*dokdonensis*
Z0017_LO_B01_2_E01_A	GREEN	No	31	66	LMG 24163	*Pseudoxanthomonas*	*dokdonensis*
Z0017_LO_A07_1_E04_A	GREEN	No	31	66	LMG 24163	*Pseudoxanthomonas*	*dokdonensis*
Z0017_RB_H12_1_A07_B	GREEN	No	31	66	LMG 24163	*Pseudoxanthomonas*	*dokdonensis*
Z0017_LB_H12_1_A01_B	GREEN	No	31	66	LMG 24163	*Pseudoxanthomonas*	*dokdonensis*
Z0017_RO_C03_1_F08_A	GREEN	No	31	66	LMG 24163	*Pseudoxanthomonas*	*dokdonensis*
Z0017_LO_D05_2_F03_A	GREEN	No	31	66	LMG 24163	*Pseudoxanthomonas*	*dokdonensis*
Z0017_LO_C12_1_F06_B	GREEN	No	31	66	LMG 24163	*Pseudoxanthomonas*	*dokdonensis*
Z0017_LO_B04_2_E02_B	GREEN	No	31	66	LMG 24163	*Pseudoxanthomonas*	*dokdonensis*
Z0017_LO_C11_1_F06_A	GREEN	No	31	66	LMG 24163	*Pseudoxanthomonas*	*dokdonensis*
Z0020_LB_G02_2_A06_B	GREEN	Yes	32	91	LMG 26467	*Tardiphaga*	*robiniae*
Z0020_RB_F06_1_B10_B	GREEN	No	32	91	LMG 26467	*Tardiphaga*	*robiniae*
Z0020_RB_G07_2_A09_A	GREEN	No	32	91	LMG 26467	*Tardiphaga*	*robiniae*
Z0020_LB_H01_1_A06_A	GREEN	No	32	91	LMG 26467	*Tardiphaga*	*robiniae*
Z0020_LB_G08_2_A03_B	GREEN	No	32	91	LMG 26467	*Tardiphaga*	*robiniae*
Z0020_RB_F05_1_B10_A	GREEN	No	32	91	LMG 26467	*Tardiphaga*	*robiniae*
Z0020_LB_G03_2_A05_A	GREEN	No	32	91	LMG 26467	*Tardiphaga*	*robiniae*
Z0020_RB_G06_2_A10_B	GREEN	No	32	91	LMG 26467	*Tardiphaga*	*robiniae*
Z0020_RB_F11_1_B07_A	GREEN	No	32	91	LMG 26467	*Tardiphaga*	*robiniae*
Z0020_RB_F09_1_B08_A	GREEN	No	32	91	LMG 26467	*Tardiphaga*	*robiniae*
Z0020_LB_G04_2_A05_B	GREEN	No	32	91	LMG 26467	*Tardiphaga*	*robiniae*
Z0020_RB_F07_1_B09_A	GREEN	No	32	91	LMG 26467	*Tardiphaga*	*robiniae*
Z0020_LB_G07_2_A03_A	GREEN	No	32	91	LMG 26467	*Tardiphaga*	*robiniae*
Z0020_RB_G03_2_A11_A	GREEN	No	32	91	LMG 26467	*Tardiphaga*	*robiniae*
Z0020_LB_G06_2_A04_B	GREEN	No	32	91	LMG 26467	*Tardiphaga*	*robiniae*
Z0020_RB_G01_2_A12_A	GREEN	No	32	91	LMG 26467	*Tardiphaga*	*robiniae*
Z0020_RB_F10_1_B08_B	GREEN	No	32	91	LMG 26467	*Tardiphaga*	*robiniae*
Z0020_LB_G09_2_A02_A	GREEN	No	32	91	LMG 26467	*Tardiphaga*	*robiniae*
Z0020_RB_F12_1_B07_B	GREEN	No	32	91	LMG 26467	*Tardiphaga*	*robiniae*
Z0020_RB_G05_2_A10_A	GREEN	No	32	91	LMG 26467	*Tardiphaga*	*robiniae*
Z0020_LB_H02_1_A06_B	GREEN	No	32	91	LMG 26467	*Tardiphaga*	*robiniae*
Z0020_LB_G05_2_A04_A	GREEN	No	32	91	LMG 26467	*Tardiphaga*	*robiniae*
Z0020_RB_F08_1_B09_B	GREEN	No	32	91	LMG 26467	*Tardiphaga*	*robiniae*
Z0020_RB_G09_2_A08_A	GREEN	No	32	91	LMG 26467	*Tardiphaga*	*robiniae*
Z0020_RB_G11_2_A07_A	GREEN	No	32	91	LMG 26467	*Tardiphaga*	*robiniae*
Z0020_RB_G08_2_A09_B	GREEN	No	32	91	LMG 26467	*Tardiphaga*	*robiniae*
Z0020_RB_G10_2_A08_B	GREEN	No	32	91	LMG 26467	*Tardiphaga*	*robiniae*
Z0020_RB_G04_2_A11_B	GREEN	No	32	91	LMG 26467	*Tardiphaga*	*robiniae*
Z0020_LB_G12_2_A01_B	GREEN	No	32	91	LMG 26467	*Tardiphaga*	*robiniae*
Z0020_LB_G10_2_A02_B	GREEN	No	32	91	LMG 26467	*Tardiphaga*	*robiniae*
Z0020_RB_G02_2_A12_B	GREEN	No	32	91	LMG 26467	*Tardiphaga*	*robiniae*
Z0020_LB_G11_2_A01_A	GREEN	No	32	91	LMG 26467	*Tardiphaga*	*robiniae*
Z0019_LO_G05_1_H03_A	GREEN	Yes	33	86	LMG 26121	*Rosenbergiella*	*nectarea*
Z0019_LO_F07_2_G04_A	GREEN	No	33	86	LMG 26121	*Rosenbergiella*	*nectarea*
Z0019_LO_G02_1_H01_B	GREEN	No	33	86	LMG 26121	*Rosenbergiella*	*nectarea*
Z0019_RO_G11_1_H12_A	GREEN	No	33	86	LMG 26121	*Rosenbergiella*	*nectarea*
Z0019_LO_F10_2_G05_B	GREEN	No	33	86	LMG 26121	*Rosenbergiella*	*nectarea*
Z0019_LO_G01_1_H01_A	GREEN	No	33	86	LMG 26121	*Rosenbergiella*	*nectarea*
Z0019_LO_F06_2_G03_B	GREEN	No	33	86	LMG 26121	*Rosenbergiella*	*nectarea*
Z0019_RO_G10_1_H11_B	GREEN	No	33	86	LMG 26121	*Rosenbergiella*	*nectarea*
Z0019_RO_G04_1_H08_B	GREEN	No	33	86	LMG 26121	*Rosenbergiella*	*nectarea*
Z0019_LO_G07_1_H04_A	GREEN	No	33	86	LMG 26121	*Rosenbergiella*	*nectarea*
Z0019_LO_G03_1_H02_A	GREEN	No	33	86	LMG 26121	*Rosenbergiella*	*nectarea*
Z0019_RO_G05_1_H09_A	GREEN	No	33	86	LMG 26121	*Rosenbergiella*	*nectarea*
Z0019_LO_G09_1_H05_A	GREEN	No	33	86	LMG 26121	*Rosenbergiella*	*nectarea*
Z0019_RO_G09_1_H11_A	GREEN	No	33	86	LMG 26121	*Rosenbergiella*	*nectarea*
Z0019_LO_G12_1_H06_B	GREEN	No	33	86	LMG 26121	*Rosenbergiella*	*nectarea*
Z0019_LO_G08_1_H04_B	GREEN	No	33	86	LMG 26121	*Rosenbergiella*	*nectarea*
Z0019_RO_G03_1_H08_A	GREEN	No	33	86	LMG 26121	*Rosenbergiella*	*nectarea*
Z0019_LO_G11_1_H06_A	GREEN	No	33	86	LMG 26121	*Rosenbergiella*	*nectarea*
Z0019_LO_G06_1_H03_B	GREEN	No	33	86	LMG 26121	*Rosenbergiella*	*nectarea*
Z0019_LO_F11_2_G06_A	GREEN	No	33	86	LMG 26121	*Rosenbergiella*	*nectarea*

Z0019_RO_G08_1_H10_B	GREEN	No	33	86	LMG 26121	*Rosenbergiella*	*nectarea*
Z0019_LO_F08_2_G04_B	GREEN	No	33	86	LMG 26121	*Rosenbergiella*	*nectarea*
Z0019_RO_H04_2_H08_B	GREEN	No	33	86	LMG 26121	*Rosenbergiella*	*nectarea*
Z0019_RO_H01_2_H07_A	GREEN	No	33	86	LMG 26121	*Rosenbergiella*	*nectarea*
Z0019_RO_G07_1_H10_A	GREEN	No	33	86	LMG 26121	*Rosenbergiella*	*nectarea*
Z0019_RO_G12_1_H12_B	GREEN	No	33	86	LMG 26121	*Rosenbergiella*	*nectarea*
Z0019_LO_G10_1_H05_B	GREEN	No	33	86	LMG 26121	*Rosenbergiella*	*nectarea*
Z0019_RO_G06_1_H09_B	GREEN	No	33	86	LMG 26121	*Rosenbergiella*	*nectarea*
Z0019_LO_F09_2_G05_A	GREEN	No	33	86	LMG 26121	*Rosenbergiella*	*nectarea*
Z0019_RO_H03_2_H08_A	GREEN	No	33	86	LMG 26121	*Rosenbergiella*	*nectarea*
Z0019_RO_H02_2_H07_B	GREEN	No	33	86	LMG 26121	*Rosenbergiella*	*nectarea*
Z0019_LO_H01_2_H01_A	GREEN	No	33	86	LMG 26121	*Rosenbergiella*	*nectarea*
Z0018_RB_A06_2_D10_B	GREEN	Yes	34	67	LMG 24367	*Ruegeria*	*scottomollicae*
Z0018_RB_A02_2_D12_B	GREEN	No	34	67	LMG 24367	*Ruegeria*	*scottomollicae*
Z0018_RB_A05_2_D10_A	GREEN	No	34	67	LMG 24367	*Ruegeria*	*scottomollicae*
Z0018_LB_A11_2_D01_A	GREEN	No	34	67	LMG 24367	*Ruegeria*	*scottomollicae*
Z0018_LO_H12_2_H06_B	GREEN	No	34	67	LMG 24367	*Ruegeria*	*scottomollicae*
Z0018_LB_A12_2_D01_B	GREEN	No	34	67	LMG 24367	*Ruegeria*	*scottomollicae*
Z0018_RO_B08_2_E10_B	GREEN	Yes	35	67	LMG 24367	*Ruegeria*	*scottomollicae*
Z0018_LO_A12_1_E06_B	GREEN	No	35	67	LMG 24367	*Ruegeria*	*scottomollicae*
Z0018_LB_A04_2_D05_B	GREEN	No	35	67	LMG 24367	*Ruegeria*	*scottomollicae*
Z0018_LO_B01_2_E01_A	GREEN	No	35	67	LMG 24367	*Ruegeria*	*scottomollicae*
Z0018_LO_H08_2_H04_B	GREEN	No	35	67	LMG 24367	*Ruegeria*	*scottomollicae*
Z0018_RO_A01_1_E07_A	GREEN	No	35	67	LMG 24367	*Ruegeria*	*scottomollicae*
Z0018_LO_C02_1_F01_B	GREEN	No	35	67	LMG 24367	*Ruegeria*	*scottomollicae*
Z0018_RB_A04_2_D11_B	GREEN	No	35	67	LMG 24367	*Ruegeria*	*scottomollicae*
Z0018_LB_A01_2_D06_A	GREEN	No	35	67	LMG 24367	*Ruegeria*	*scottomollicae*
Z0018_LB_A10_2_D02_B	GREEN	No	35	67	LMG 24367	*Ruegeria*	*scottomollicae*
Z0018_LO_B02_2_E01_B	GREEN	No	35	67	LMG 24367	*Ruegeria*	*scottomollicae*
Z0018_LB_A06_2_D04_B	GREEN	No	35	67	LMG 24367	*Ruegeria*	*scottomollicae*
Z0018_RB_H10_1_A08_B	GREEN	No	35	67	LMG 24367	*Ruegeria*	*scottomollicae*
Z0018_LB_A02_2_D06_B	GREEN	No	35	67	LMG 24367	*Ruegeria*	*scottomollicae*
Z0018_LB_A09_2_D02_A	GREEN	No	35	67	LMG 24367	*Ruegeria*	*scottomollicae*
Z0018_LB_A07_2_D03_A	GREEN	No	35	67	LMG 24367	*Ruegeria*	*scottomollicae*
Z0018_LO_H07_2_H04_A	GREEN	No	35	67	LMG 24367	*Ruegeria*	*scottomollicae*
Z0018_RB_A01_2_D12_A	GREEN	No	35	67	LMG 24367	*Ruegeria*	*scottomollicae*
Z0018_LB_A03_2_D05_A	GREEN	No	35	67	LMG 24367	*Ruegeria*	*scottomollicae*
Z0018_LB_A08_2_D03_B	GREEN	No	35	67	LMG 24367	*Ruegeria*	*scottomollicae*
Z0018_RO_A03_1_E08_A	GREEN	No	35	67	LMG 24367	*Ruegeria*	*scottomollicae*
Z0018_LO_H09_2_H05_A	GREEN	No	35	67	LMG 24367	*Ruegeria*	*scottomollicae*
Z0018_RB_A03_2_D11_A	GREEN	No	35	67	LMG 24367	*Ruegeria*	*scottomollicae*
Z0018_LO_H10_2_H05_B	GREEN	No	35	67	LMG 24367	*Ruegeria*	*scottomollicae*
Z0018_LO_H11_2_H06_A	GREEN	No	35	67	LMG 24367	*Ruegeria*	*scottomollicae*
Z0018_LB_A05_2_D04_A	GREEN	Yes	36	67	LMG 24367	*Ruegeria*	*scottomollicae*
Z0024_RB_C08_2_C09_B	GREEN	Yes	37	124	LMG 6519	*Oligella*	*ureolytica*
Z0024_RB_D02_1_C12_B	GREEN	No	37	124	LMG 6519	*Oligella*	*ureolytica*
Z0024_LB_C08_2_C03_B	GREEN	No	37	124	LMG 6519	*Oligella*	*ureolytica*
Z0024_LB_B11_1_D01_A	GREEN	No	37	124	LMG 6519	*Oligella*	*ureolytica*
Z0024_RB_C02_2_C12_B	GREEN	No	37	124	LMG 6519	*Oligella*	*ureolytica*
Z0024_RB_C07_2_C09_A	GREEN	No	37	124	LMG 6519	*Oligella*	*ureolytica*
Z0024_RB_C04_2_C11_B	GREEN	No	37	124	LMG 6519	*Oligella*	*ureolytica*
Z0024_LB_C05_2_C04_A	GREEN	No	37	124	LMG 6519	*Oligella*	*ureolytica*
Z0024_LB_B12_1_D01_B	GREEN	No	37	124	LMG 6519	*Oligella*	*ureolytica*
Z0024_LB_C04_2_C05_B	GREEN	No	37	124	LMG 6519	*Oligella*	*ureolytica*
Z0024_LB_B04_1_D05_B	GREEN	No	37	124	LMG 6519	*Oligella*	*ureolytica*
Z0024_LB_B08_1_D03_B	GREEN	No	37	124	LMG 6519	*Oligella*	*ureolytica*
Z0024_LB_C09_2_C02_A	GREEN	No	37	124	LMG 6519	*Oligella*	*ureolytica*
Z0024_LB_C06_2_C04_B	GREEN	No	37	124	LMG 6519	*Oligella*	*ureolytica*
Z0024_LB_C03_2_C05_A	GREEN	No	37	124	LMG 6519	*Oligella*	*ureolytica*
Z0024_RB_C11_2_C07_A	GREEN	No	37	124	LMG 6519	*Oligella*	*ureolytica*
Z0024_RB_C10_2_C08_B	GREEN	No	37	124	LMG 6519	*Oligella*	*ureolytica*
Z0024_RB_D01_1_C12_A	GREEN	No	37	124	LMG 6519	*Oligella*	*ureolytica*
Z0024_RB_C05_2_C10_A	GREEN	No	37	124	LMG 6519	*Oligella*	*ureolytica*
Z0024_LB_B09_1_D02_A	GREEN	No	37	124	LMG 6519	*Oligella*	*ureolytica*
Z0024_LB_B06_1_D04_B	GREEN	No	37	124	LMG 6519	*Oligella*	*ureolytica*
Z0024_RB_C06_2_C10_B	GREEN	No	37	124	LMG 6519	*Oligella*	*ureolytica*
Z0024_LB_B05_1_D04_A	GREEN	No	37	124	LMG 6519	*Oligella*	*ureolytica*
Z0024_RB_C01_2_C12_A	GREEN	No	37	124	LMG 6519	*Oligella*	*ureolytica*
Z0024_RB_C12_2_C07_B	GREEN	No	37	124	LMG 6519	*Oligella*	*ureolytica*
Z0024_LB_B10_1_D02_B	GREEN	No	37	124	LMG 6519	*Oligella*	*ureolytica*

Z0024_LB_C02_2_C06_B	GREEN	No	37	124	LMG 6519	*Oligella*	*ureolytica*
Z0024_LB_C07_2_C03_A	GREEN	No	37	124	LMG 6519	*Oligella*	*ureolytica*
Z0024_LB_C01_2_C06_A	GREEN	No	37	124	LMG 6519	*Oligella*	*ureolytica*
Z0024_RB_C03_2_C11_A	GREEN	No	37	124	LMG 6519	*Oligella*	*ureolytica*
Z0024_LB_B07_1_D03_A	GREEN	No	37	124	LMG 6519	*Oligella*	*ureolytica*
Z0024_RB_C09_2_C08_A	GREEN	No	37	124	LMG 6519	*Oligella*	*ureolytica*
Z0014_RO_G06_1_H09_B	GREEN	Yes	38	30	LMG 19484	*Sphingomonas*	*melonis*
Z0014_RO_G12_1_H12_B	GREEN	No	38	30	LMG 19484	*Sphingomonas*	*melonis*
Z0014_RO_E11_1_G12_A	GREEN	No	38	30	LMG 19484	*Sphingomonas*	*melonis*
Z0014_RO_H02_2_H07_B	GREEN	No	38	30	LMG 19484	*Sphingomonas*	*melonis*
Z0014_RO_E12_1_G12_B	GREEN	No	38	30	LMG 19484	*Sphingomonas*	*melonis*
Z0014_RO_G11_1_H12_A	GREEN	No	38	30	LMG 19484	*Sphingomonas*	*melonis*
Z0014_RO_G10_1_H11_B	GREEN	No	38	30	LMG 19484	*Sphingomonas*	*melonis*
Z0014_LO_G10_1_H05_B	GREEN	No	38	30	LMG 19484	*Sphingomonas*	*melonis*
Z0014_RO_G07_1_H10_A	GREEN	No	38	30	LMG 19484	*Sphingomonas*	*melonis*
Z0014_LO_F09_2_G05_A	GREEN	No	38	30	LMG 19484	*Sphingomonas*	*melonis*
Z0014_LO_F08_2_G04_B	GREEN	No	38	30	LMG 19484	*Sphingomonas*	*melonis*
Z0014_RO_F09_2_G11_A	GREEN	No	38	30	LMG 19484	*Sphingomonas*	*melonis*
Z0014_LO_G12_1_H06_B	GREEN	No	38	30	LMG 19484	*Sphingomonas*	*melonis*
Z0014_LO_F05_2_G03_A	GREEN	No	38	30	LMG 19484	*Sphingomonas*	*melonis*
Z0014_LO_F12_2_G06_B	GREEN	No	38	30	LMG 19484	*Sphingomonas*	*melonis*
Z0014_LO_G08_1_H04_B	GREEN	No	38	30	LMG 19484	*Sphingomonas*	*melonis*
Z0014_LO_G07_1_H04_A	GREEN	No	38	30	LMG 19484	*Sphingomonas*	*melonis*
Z0014_RO_F04_2_G08_B	GREEN	No	38	30	LMG 19484	*Sphingomonas*	*melonis*
Z0014_LO_H03_2_H02_A	GREEN	No	38	30	LMG 19484	*Sphingomonas*	*melonis*
Z0014_LO_F04_2_G02_B	GREEN	No	38	30	LMG 19484	*Sphingomonas*	*melonis*
Z0014_LO_F02_2_G01_B	GREEN	No	38	30	LMG 19484	*Sphingomonas*	*melonis*
Z0014_LO_F10_2_G05_B	GREEN	No	38	30	LMG 19484	*Sphingomonas*	*melonis*
Z0014_LO_H04_2_H02_B	GREEN	No	38	30	LMG 19484	*Sphingomonas*	*melonis*
Z0014_LO_G11_1_H06_A	GREEN	No	38	30	LMG 19484	*Sphingomonas*	*melonis*
Z0014_LO_G06_1_H03_B	GREEN	No	38	30	LMG 19484	*Sphingomonas*	*melonis*
Z0014_LO_G03_1_H02_A	GREEN	No	38	30	LMG 19484	*Sphingomonas*	*melonis*
Z0014_LO_F03_2_G02_A	GREEN	No	38	30	LMG 19484	*Sphingomonas*	*melonis*
Z0014_LO_H08_2_H04_B	GREEN	No	38	30	LMG 19484	*Sphingomonas*	*melonis*
Z0014_LO_G05_1_H03_A	GREEN	No	38	30	LMG 19484	*Sphingomonas*	*melonis*
Z0014_LO_G02_1_H01_B	GREEN	No	38	30	LMG 19484	*Sphingomonas*	*melonis*
Z0014_LO_G01_1_H01_A	GREEN	No	38	30	LMG 19484	*Sphingomonas*	*melonis*
Z0014_LO_G09_1_H05_A	GREEN	No	38	30	LMG 19484	*Sphingomonas*	*melonis*
Z0012_LB_E11_2_B01_A	GREEN	Yes	39	6	LMG 1229	*Alcaligenes*	*faecalis*
Z0012_RB_E04_2_B11_B	GREEN	No	39	6	LMG 1229	*Alcaligenes*	*faecalis*
Z0012_RB_D12_1_C07_B	GREEN	No	39	6	LMG 1229	*Alcaligenes*	*faecalis*
Z0012_RB_E10_2_B08_B	GREEN	No	39	6	LMG 1229	*Alcaligenes*	*faecalis*
Z0012_RB_E06_2_B10_B	GREEN	No	39	6	LMG 1229	*Alcaligenes*	*faecalis*
Z0012_LB_E06_2_B04_B	GREEN	No	39	6	LMG 1229	*Alcaligenes*	*faecalis*
Z0012_RB_E07_2_B09_A	GREEN	No	39	6	LMG 1229	*Alcaligenes*	*faecalis*
Z0012_LB_E09_2_B02_A	GREEN	No	39	6	LMG 1229	*Alcaligenes*	*faecalis*
Z0012_RB_E08_2_B09_B	GREEN	No	39	6	LMG 1229	*Alcaligenes*	*faecalis*
Z0012_LB_F02_1_B06_B	GREEN	No	39	6	LMG 1229	*Alcaligenes*	*faecalis*
Z0012_RB_E09_2_B08_A	GREEN	No	39	6	LMG 1229	*Alcaligenes*	*faecalis*
Z0012_LB_E08_2_B03_B	GREEN	No	39	6	LMG 1229	*Alcaligenes*	*faecalis*
Z0012_RB_D08_1_C09_B	GREEN	No	39	6	LMG 1229	*Alcaligenes*	*faecalis*
Z0012_LB_E10_2_B02_B	GREEN	No	39	6	LMG 1229	*Alcaligenes*	*faecalis*
Z0012_RB_D11_1_C07_A	GREEN	No	39	6	LMG 1229	*Alcaligenes*	*faecalis*
Z0012_LB_F01_1_B06_A	GREEN	No	39	6	LMG 1229	*Alcaligenes*	*faecalis*
Z0012_LB_F07_1_B03_A	GREEN	No	39	6	LMG 1229	*Alcaligenes*	*faecalis*
Z0012_RB_E05_2_B10_A	GREEN	No	39	6	LMG 1229	*Alcaligenes*	*faecalis*
Z0012_LB_E12_2_B01_B	GREEN	No	39	6	LMG 1229	*Alcaligenes*	*faecalis*
Z0012_LB_F08_1_B03_B	GREEN	No	39	6	LMG 1229	*Alcaligenes*	*faecalis*
Z0012_RB_E01_2_B12_A	GREEN	No	39	6	LMG 1229	*Alcaligenes*	*faecalis*
Z0012_LB_F05_1_B04_A	GREEN	No	39	6	LMG 1229	*Alcaligenes*	*faecalis*
Z0012_LB_F06_1_B04_B	GREEN	No	39	6	LMG 1229	*Alcaligenes*	*faecalis*
Z0012_RB_D07_1_C09_A	GREEN	No	39	6	LMG 1229	*Alcaligenes*	*faecalis*
Z0012_LB_F09_1_B02_A	GREEN	No	39	6	LMG 1229	*Alcaligenes*	*faecalis*
Z0012_RB_D09_1_C08_A	GREEN	No	39	6	LMG 1229	*Alcaligenes*	*faecalis*
Z0012_LB_E07_2_B03_A	GREEN	No	39	6	LMG 1229	*Alcaligenes*	*faecalis*
Z0012_RB_E02_2_B12_B	GREEN	No	39	6	LMG 1229	*Alcaligenes*	*faecalis*
Z0012_RB_D10_1_C08_B	GREEN	No	39	6	LMG 1229	*Alcaligenes*	*faecalis*
Z0012_LB_F04_1_B05_B	GREEN	No	39	6	LMG 1229	*Alcaligenes*	*faecalis*
Z0012_RB_E03_2_B11_A	GREEN	No	39	6	LMG 1229	*Alcaligenes*	*faecalis*
Z0012_LB_F03_1_B05_A	GREEN	No	39	6	LMG 1229	*Alcaligenes*	*faecalis*

Z0016_LB_A08_2_D03_B	GREEN	Yes	40	44	LMG 22214	*Aeromonas*	*molluscorum*	
Z0016_RO_D02_2_F07_B	GREEN	No	40	44	LMG 22214	*Aeromonas*	*molluscorum*	
Z0016_RB_A04_2_D11_B	GREEN	No	40	44	LMG 22214	*Aeromonas*	*molluscorum*	
Z0016_LO_H06_2_H03_B	GREEN	No	40	44	LMG 22214	*Aeromonas*	*molluscorum*	
Z0016_LB_A09_2_D02_A	GREEN	No	40	44	LMG 22214	*Aeromonas*	*molluscorum*	
Z0016_LO_H09_2_H05_A	GREEN	No	40	44	LMG 22214	*Aeromonas*	*molluscorum*	
Z0016_LO_C06_1_F03_B	GREEN	No	40	44	LMG 22214	*Aeromonas*	*molluscorum*	
Z0016_LB_A06_2_D04_B	GREEN	No	40	44	LMG 22214	*Aeromonas*	*molluscorum*	
Z0016_LO_H10_2_H05_B	GREEN	No	40	44	LMG 22214	*Aeromonas*	*molluscorum*	
Z0016_RO_C05_1_F09_A	GREEN	No	40	44	LMG 22214	*Aeromonas*	*molluscorum*	
Z0016_RB_A02_2_D12_B	GREEN	No	40	44	LMG 22214	*Aeromonas*	*molluscorum*	
Z0016_RB_A06_2_D10_B	GREEN	No	40	44	LMG 22214	*Aeromonas*	*molluscorum*	
Z0016_RB_A01_2_D12_A	GREEN	No	40	44	LMG 22214	*Aeromonas*	*molluscorum*	
Z0016_RB_A05_2_D10_A	GREEN	No	40	44	LMG 22214	*Aeromonas*	*molluscorum*	
Z0016_LB_A03_2_D05_A	GREEN	No	40	44	LMG 22214	*Aeromonas*	*molluscorum*	
Z0016_LB_A04_2_D05_B	GREEN	No	40	44	LMG 22214	*Aeromonas*	*molluscorum*	
Z0016_LO_H08_2_H04_B	GREEN	No	40	44	LMG 22214	*Aeromonas*	*molluscorum*	
Z0016_RB_A03_2_D11_A	GREEN	No	40	44	LMG 22214	*Aeromonas*	*molluscorum*	
Z0016_LB_A11_2_D01_A	GREEN	No	40	44	LMG 22214	*Aeromonas*	*molluscorum*	
Z0016_LB_A10_2_D02_B	GREEN	No	40	44	LMG 22214	*Aeromonas*	*molluscorum*	
Z0016_RO_B07_2_E10_A	GREEN	No	40	44	LMG 22214	*Aeromonas*	*molluscorum*	
Z0016_LB_A01_2_D06_A	GREEN	No	40	44	LMG 22214	*Aeromonas*	*molluscorum*	
Z0016_LB_A07_2_D03_A	GREEN	No	40	44	LMG 22214	*Aeromonas*	*molluscorum*	
Z0016_LB_A02_2_D06_B	GREEN	No	40	44	LMG 22214	*Aeromonas*	*molluscorum*	
Z0016_RO_E06_1_G09_B	GREEN	No	40	44	LMG 22214	*Aeromonas*	*molluscorum*	
Z0016_RO_E12_1_G12_B	GREEN	No	40	44	LMG 22214	*Aeromonas*	*molluscorum*	
Z0016_LO_D04_2_F02_B	GREEN	No	40	44	LMG 22214	*Aeromonas*	*molluscorum*	
Z0016_LO_H07_2_H04_A	GREEN	No	40	44	LMG 22214	*Aeromonas*	*molluscorum*	
Z0016_LO_H11_2_H06_A	GREEN	No	40	44	LMG 22214	*Aeromonas*	*molluscorum*	
Z0016_LO_C08_1_F04_B	GREEN	No	40	44	LMG 22214	*Aeromonas*	*molluscorum*	
Z0016_LB_A05_2_D04_A	GREEN	No	40	44	LMG 22214	*Aeromonas*	*molluscorum*	
Z0016_LO_H12_2_H06_B	GREEN	No	40	44	LMG 22214	*Aeromonas*	*molluscorum*	
Z0014_LO_D11_2_F06_A	GREEN	Yes	41	32	LMG 19863	*Maricaulis*	*parjimensis*	
Z0014_RO_D01_2_F07_A	GREEN	No	41	32	LMG 19863	*Maricaulis*	*parjimensis*	
Z0014_RO_E08_1_G10_B	GREEN	Yes	42	32	LMG 19863	*Maricaulis*	*parjimensis*	
Z0014_RO_E09_1_G11_A	GREEN	No	42	32	LMG 19863	*Maricaulis*	*parjimensis*	
Z0014_RO_E02_1_G07_B	GREEN	No	42	32	LMG 19863	*Maricaulis*	*parjimensis*	
Z0014_LO_D03_2_F02_A	GREEN	No	42	32	LMG 19863	*Maricaulis*	*parjimensis*	
Z0014_LO_E03_1_G02_A	GREEN	No	42	32	LMG 19863	*Maricaulis*	*parjimensis*	
Z0014_RO_E07_1_G10_A	GREEN	No	42	32	LMG 19863	*Maricaulis*	*parjimensis*	
Z0014_LO_C03_1_F02_A	GREEN	No	42	32	LMG 19863	*Maricaulis*	*parjimensis*	
Z0014_RO_D02_2_F07_B	GREEN	No	42	32	LMG 19863	*Maricaulis*	*parjimensis*	
Z0014_RO_E05_1_G09_A	GREEN	No	42	32	LMG 19863	*Maricaulis*	*parjimensis*	
Z0014_LO_E04_1_G02_B	GREEN	No	42	32	LMG 19863	*Maricaulis*	*parjimensis*	
Z0014_RO_D10_2_F11_B	GREEN	No	42	32	LMG 19863	*Maricaulis*	*parjimensis*	
Z0014_RO_E04_1_G08_B	GREEN	No	42	32	LMG 19863	*Maricaulis*	*parjimensis*	
Z0014_LB_A01_2_D06_A	GREEN	No	42	32	LMG 19863	*Maricaulis*	*parjimensis*	
Z0014_RO_E10_1_G11_B	GREEN	No	42	32	LMG 19863	*Maricaulis*	*parjimensis*	
Z0014_RO_D06_2_F09_B	GREEN	No	42	32	LMG 19863	*Maricaulis*	*parjimensis*	
Z0014_RO_D05_2_F09_A	GREEN	No	42	32	LMG 19863	*Maricaulis*	*parjimensis*	
Z0014_RO_D12_2_F12_B	GREEN	No	42	32	LMG 19863	*Maricaulis*	*parjimensis*	
Z0014_LO_C07_1_F04_A	GREEN	No	42	32	LMG 19863	*Maricaulis*	*parjimensis*	
Z0014_LO_D02_2_F01_B	GREEN	No	42	32	LMG 19863	*Maricaulis*	*parjimensis*	
Z0014_LO_C10_1_F05_B	GREEN	No	42	32	LMG 19863	*Maricaulis*	*parjimensis*	
Z0014_RO_F03_2_G08_A	GREEN	No	42	32	LMG 19863	*Maricaulis*	*parjimensis*	
Z0014_LO_E02_1_G01_B	GREEN	No	42	32	LMG 19863	*Maricaulis*	*parjimensis*	
Z0014_RO_D03_2_F08_A	GREEN	No	42	32	LMG 19863	*Maricaulis*	*parjimensis*	
Z0014_LO_C08_1_F04_B	GREEN	No	42	32	LMG 19863	*Maricaulis*	*parjimensis*	
Z0014_RO_D11_2_F12_A	GREEN	No	42	32	LMG 19863	*Maricaulis*	*parjimensis*	
Z0014_LO_C12_1_F06_B	GREEN	No	42	32	LMG 19863	*Maricaulis*	*parjimensis*	
Z0014_RO_E01_1_G07_A	GREEN	No	42	32	LMG 19863	*Maricaulis*	*parjimensis*	
Z0014_LO_E10_1_G05_B	GREEN	No	42	32	LMG 19863	*Maricaulis*	*parjimensis*	
Z0014_LO_D01_2_F01_A	GREEN	No	42	32	LMG 19863	*Maricaulis*	*parjimensis*	
Z0014_LO_D05_2_F03_A	GREEN	No	42	32	LMG 19863	*Maricaulis*	*parjimensis*	
Z0024_RO_G09_1_H11_A	GREEN	Yes	43	132	LMG 7233	*Salmonella*	*enterica*	*enterica*
Z0024_RO_D11_2_F12_A	GREEN	No	43	132	LMG 7233	*Salmonella*	*enterica*	*enterica*
Z0024_RO_D10_2_F11_B	GREEN	No	43	132	LMG 7233	*Salmonella*	*enterica*	*enterica*
Z0024_LO_E08_1_G04_B	GREEN	No	43	132	LMG 7233	*Salmonella*	*enterica*	*enterica*
Z0024_LO_H11_2_H06_A	GREEN	No	43	132	LMG 7233	*Salmonella*	*enterica*	*enterica*
Z0024_LO_G10_1_H05_B	GREEN	No	43	132	LMG 7233	*Salmonella*	*enterica*	*enterica*

Z0024_LO_D11_2_F06_A	GREEN	No	43	132	LMG 7233	*Salmonella*	*enterica*	*enterica*
Z0024_LO_F06_2_G03_B	GREEN	No	43	132	LMG 7233	*Salmonella*	*enterica*	*enterica*
Z0024_LO_H10_2_H05_B	GREEN	No	43	132	LMG 7233	*Salmonella*	*enterica*	*enterica*
Z0024_LO_G12_1_H06_B	GREEN	No	43	132	LMG 7233	*Salmonella*	*enterica*	*enterica*
Z0024_RO_G05_1_H09_A	GREEN	No	43	132	LMG 7233	*Salmonella*	*enterica*	*enterica*
Z0024_RO_D12_2_F12_B	GREEN	No	43	132	LMG 7233	*Salmonella*	*enterica*	*enterica*
Z0024_LO_E03_1_G02_A	GREEN	No	43	132	LMG 7233	*Salmonella*	*enterica*	*enterica*
Z0024_RO_F05_2_G09_A	GREEN	No	43	132	LMG 7233	*Salmonella*	*enterica*	*enterica*
Z0024_RO_F03_2_G08_A	GREEN	No	43	132	LMG 7233	*Salmonella*	*enterica*	*enterica*
Z0024_LO_E05_1_G03_A	GREEN	No	43	132	LMG 7233	*Salmonella*	*enterica*	*enterica*
Z0024_LO_D08_2_F04_B	GREEN	No	43	132	LMG 7233	*Salmonella*	*enterica*	*enterica*
Z0024_RO_E02_1_G07_B	GREEN	No	43	132	LMG 7233	*Salmonella*	*enterica*	*enterica*
Z0024_LO_G11_1_H06_A	GREEN	No	43	132	LMG 7233	*Salmonella*	*enterica*	*enterica*
Z0024_LO_H02_2_H01_B	GREEN	No	43	132	LMG 7233	*Salmonella*	*enterica*	*enterica*
Z0024_RO_F11_2_G12_A	GREEN	No	43	132	LMG 7233	*Salmonella*	*enterica*	*enterica*
Z0024_LO_F05_2_G03_A	GREEN	No	43	132	LMG 7233	*Salmonella*	*enterica*	*enterica*
Z0024_LO_H05_2_H03_A	GREEN	No	43	132	LMG 7233	*Salmonella*	*enterica*	*enterica*
Z0024_RO_E01_1_G07_A	GREEN	No	43	132	LMG 7233	*Salmonella*	*enterica*	*enterica*
Z0024_RO_F09_2_G11_A	GREEN	No	43	132	LMG 7233	*Salmonella*	*enterica*	*enterica*
Z0024_RO_F10_2_G11_B	GREEN	No	43	132	LMG 7233	*Salmonella*	*enterica*	*enterica*
Z0024_LO_G09_1_H05_A	GREEN	No	43	132	LMG 7233	*Salmonella*	*enterica*	*enterica*
Z0024_RO_F08_2_G10_B	GREEN	No	43	132	LMG 7233	*Salmonella*	*enterica*	*enterica*
Z0024_RO_D09_2_F11_A	GREEN	No	43	132	LMG 7233	*Salmonella*	*enterica*	*enterica*
Z0024_LO_H04_2_H02_B	GREEN	No	43	132	LMG 7233	*Salmonella*	*enterica*	*enterica*
Z0024_RO_G02_1_H07_B	GREEN	No	43	132	LMG 7233	*Salmonella*	*enterica*	*enterica*
Z0024_LO_E07_1_G04_A	GREEN	No	43	132	LMG 7233	*Salmonella*	*enterica*	*enterica*
Z0019_RO_C01_1_F07_A	GREEN	Yes	44	84	LMG 26041	*Tetragenococcus*	*osmophilus*	
Z0019_LO_A09_1_E05_A	GREEN	No	44	84	LMG 26041	*Tetragenococcus*	*osmophilus*	
Z0019_RO_B07_2_E10_A	GREEN	No	44	84	LMG 26041	*Tetragenococcus*	*osmophilus*	
Z0019_RO_C11_1_F12_A	GREEN	No	44	84	LMG 26041	*Tetragenococcus*	*osmophilus*	
Z0019_LO_C04_1_F02_B	GREEN	No	44	84	LMG 26041	*Tetragenococcus*	*osmophilus*	
Z0019_LO_A12_1_E06_B	GREEN	No	44	84	LMG 26041	*Tetragenococcus*	*osmophilus*	
Z0019_LO_C03_1_F02_A	GREEN	No	44	84	LMG 26041	*Tetragenococcus*	*osmophilus*	
Z0019_RO_C05_1_F09_A	GREEN	No	44	84	LMG 26041	*Tetragenococcus*	*osmophilus*	
Z0019_RO_B02_2_E07_B	GREEN	No	44	84	LMG 26041	*Tetragenococcus*	*osmophilus*	
Z0019_LO_C06_1_F03_B	GREEN	No	44	84	LMG 26041	*Tetragenococcus*	*osmophilus*	
Z0019_RO_B05_2_E09_A	GREEN	No	44	84	LMG 26041	*Tetragenococcus*	*osmophilus*	
Z0019_RO_C04_1_F08_B	GREEN	No	44	84	LMG 26041	*Tetragenococcus*	*osmophilus*	
Z0019_RO_B01_2_E07_A	GREEN	No	44	84	LMG 26041	*Tetragenococcus*	*osmophilus*	
Z0019_RO_C03_1_F08_A	GREEN	No	44	84	LMG 26041	*Tetragenococcus*	*osmophilus*	
Z0019_RO_B11_2_E12_A	GREEN	No	44	84	LMG 26041	*Tetragenococcus*	*osmophilus*	
Z0019_RO_B10_2_E11_B	GREEN	No	44	84	LMG 26041	*Tetragenococcus*	*osmophilus*	
Z0019_RO_C10_1_F11_B	GREEN	No	44	84	LMG 26041	*Tetragenococcus*	*osmophilus*	
Z0019_LO_B01_2_E01_A	GREEN	No	44	84	LMG 26041	*Tetragenococcus*	*osmophilus*	
Z0019_LO_B03_2_E02_A	GREEN	No	44	84	LMG 26041	*Tetragenococcus*	*osmophilus*	
Z0019_RO_B08_2_E10_B	GREEN	No	44	84	LMG 26041	*Tetragenococcus*	*osmophilus*	
Z0019_LO_B05_2_E03_A	GREEN	No	44	84	LMG 26041	*Tetragenococcus*	*osmophilus*	
Z0019_RO_B03_2_E08_A	GREEN	No	44	84	LMG 26041	*Tetragenococcus*	*osmophilus*	
Z0019_LO_C05_1_F03_A	GREEN	No	44	84	LMG 26041	*Tetragenococcus*	*osmophilus*	
Z0019_RO_B06_2_E09_B	GREEN	No	44	84	LMG 26041	*Tetragenococcus*	*osmophilus*	
Z0019_LO_C08_1_F04_B	GREEN	No	44	84	LMG 26041	*Tetragenococcus*	*osmophilus*	
Z0019_LO_B07_2_E04_A	GREEN	No	44	84	LMG 26041	*Tetragenococcus*	*osmophilus*	
Z0019_RO_B12_2_E12_B	GREEN	No	44	84	LMG 26041	*Tetragenococcus*	*osmophilus*	
Z0019_RO_C02_1_F07_B	GREEN	No	44	84	LMG 26041	*Tetragenococcus*	*osmophilus*	
Z0017_LO_E02_1_G01_B	GREEN	Yes	45	63	LMG 24015	*Chitinophaga*	*terrae*	
Z0017_LO_E12_1_G06_B	GREEN	No	45	63	LMG 24015	*Chitinophaga*	*terrae*	
Z0017_LO_D11_2_F06_A	GREEN	Yes	46	63	LMG 24015	*Chitinophaga*	*terrae*	
Z0017_RO_D07_2_F10_A	GREEN	No	46	63	LMG 24015	*Chitinophaga*	*terrae*	
Z0017_RO_E08_1_G10_B	GREEN	No	46	63	LMG 24015	*Chitinophaga*	*terrae*	
Z0017_LO_D03_2_F02_A	GREEN	No	46	63	LMG 24015	*Chitinophaga*	*terrae*	
Z0017_LO_D12_2_F06_B	GREEN	No	46	63	LMG 24015	*Chitinophaga*	*terrae*	
Z0017_LO_D01_2_F01_A	GREEN	No	46	63	LMG 24015	*Chitinophaga*	*terrae*	
Z0017_RO_F03_2_G08_A	GREEN	No	46	63	LMG 24015	*Chitinophaga*	*terrae*	
Z0017_RO_D04_2_F08_B	GREEN	No	46	63	LMG 24015	*Chitinophaga*	*terrae*	
Z0017_LO_E05_1_G03_A	GREEN	No	46	63	LMG 24015	*Chitinophaga*	*terrae*	
Z0017_RO_D11_2_F12_A	GREEN	No	46	63	LMG 24015	*Chitinophaga*	*terrae*	
Z0017_RO_D09_2_F11_A	GREEN	No	46	63	LMG 24015	*Chitinophaga*	*terrae*	
Z0017_LO_D08_2_F04_B	GREEN	No	46	63	LMG 24015	*Chitinophaga*	*terrae*	
Z0017_LO_C10_1_F05_B	GREEN	No	46	63	LMG 24015	*Chitinophaga*	*terrae*	
Z0017_RO_D03_2_F08_A	GREEN	No	46	63	LMG 24015	*Chitinophaga*	*terrae*	

Z0017_LO_E03_1_G02_A	GREEN	No	46	63	LMG 24015	*Chitinophaga*	*terrae*
Z0017_RO_F01_2_G07_A	GREEN	No	46	63	LMG 24015	*Chitinophaga*	*terrae*
Z0017_LO_D06_2_F03_B	GREEN	No	46	63	LMG 24015	*Chitinophaga*	*terrae*
Z0017_LO_C08_1_F04_B	GREEN	No	46	63	LMG 24015	*Chitinophaga*	*terrae*
Z0017_RO_E02_1_G07_B	GREEN	No	46	63	LMG 24015	*Chitinophaga*	*terrae*
Z0017_RO_E05_1_G09_A	GREEN	No	46	63	LMG 24015	*Chitinophaga*	*terrae*
Z0017_RO_E11_1_G12_A	GREEN	No	46	63	LMG 24015	*Chitinophaga*	*terrae*
Z0017_RO_E04_1_G08_B	GREEN	Yes	47	63	LMG 24015	*Chitinophaga*	*terrae*
Z0017_LO_E07_1_G04_A	GREEN	Yes	48	63	LMG 24015	*Chitinophaga*	*terrae*
Z0017_RO_F04_2_G08_B	GREEN	No	48	63	LMG 24015	*Chitinophaga*	*terrae*
Z0017_RO_D06_2_F09_B	GREEN	Yes	49	63	LMG 24015	*Chitinophaga*	*terrae*
Z0017_RO_F05_2_G09_A	GREEN	Yes	50	63	LMG 24015	*Chitinophaga*	*terrae*
Z0017_RO_D12_2_F12_B	GREEN	No	50	63	LMG 24015	*Chitinophaga*	*terrae*
Z0017_LO_F02_2_G01_B	GREEN	No	50	63	LMG 24015	*Chitinophaga*	*terrae*
Z0017_LO_F01_2_G01_A	GREEN	No	50	63	LMG 24015	*Chitinophaga*	*terrae*
Z0017_RO_F10_2_G11_B	GREEN	No	50	63	LMG 24015	*Chitinophaga*	*terrae*
Z0020_RB_D09_1_C08_A	GREEN	Yes	51	90	LMG 26304	*Enterococcus*	*ureasiticus*
Z0020_RB_D07_1_C09_A	GREEN	No	51	90	LMG 26304	*Enterococcus*	*ureasiticus*
Z0020_LB_E03_2_B05_A	GREEN	No	51	90	LMG 26304	*Enterococcus*	*ureasiticus*
Z0020_RB_C09_2_C08_A	GREEN	Yes	52	90	LMG 26304	*Enterococcus*	*ureasiticus*
Z0020_LB_D10_1_C02_B	GREEN	No	52	90	LMG 26304	*Enterococcus*	*ureasiticus*
Z0020_RB_C12_2_C07_B	GREEN	No	52	90	LMG 26304	*Enterococcus*	*ureasiticus*
Z0020_LB_D06_1_C04_B	GREEN	No	52	90	LMG 26304	*Enterococcus*	*ureasiticus*
Z0020_RB_D05_1_C10_A	GREEN	No	52	90	LMG 26304	*Enterococcus*	*ureasiticus*
Z0020_LB_D11_1_C01_A	GREEN	No	52	90	LMG 26304	*Enterococcus*	*ureasiticus*
Z0020_LB_E04_2_B05_B	GREEN	No	52	90	LMG 26304	*Enterococcus*	*ureasiticus*
Z0020_RB_D10_1_C08_B	GREEN	No	52	90	LMG 26304	*Enterococcus*	*ureasiticus*
Z0020_LB_E06_2_B04_B	GREEN	No	52	90	LMG 26304	*Enterococcus*	*ureasiticus*
Z0020_RB_D08_1_C09_B	GREEN	No	52	90	LMG 26304	*Enterococcus*	*ureasiticus*
Z0020_RB_D03_1_C11_A	GREEN	No	52	90	LMG 26304	*Enterococcus*	*ureasiticus*
Z0020_LB_D08_1_C03_B	GREEN	No	52	90	LMG 26304	*Enterococcus*	*ureasiticus*
Z0020_RB_D12_1_C07_B	GREEN	No	52	90	LMG 26304	*Enterococcus*	*ureasiticus*
Z0020_RB_D06_1_C10_B	GREEN	No	52	90	LMG 26304	*Enterococcus*	*ureasiticus*
Z0020_RB_C10_2_C08_B	GREEN	No	52	90	LMG 26304	*Enterococcus*	*ureasiticus*
Z0020_LB_E05_2_B04_A	GREEN	No	52	90	LMG 26304	*Enterococcus*	*ureasiticus*
Z0020_RB_E02_2_B12_B	GREEN	No	52	90	LMG 26304	*Enterococcus*	*ureasiticus*
Z0020_RB_D11_1_C07_A	GREEN	No	52	90	LMG 26304	*Enterococcus*	*ureasiticus*
Z0020_RB_E01_2_B12_A	GREEN	No	52	90	LMG 26304	*Enterococcus*	*ureasiticus*
Z0020_RB_E03_2_B11_A	GREEN	No	52	90	LMG 26304	*Enterococcus*	*ureasiticus*
Z0020_LB_E02_2_B06_B	GREEN	No	52	90	LMG 26304	*Enterococcus*	*ureasiticus*
Z0020_LB_D12_1_C01_B	GREEN	No	52	90	LMG 26304	*Enterococcus*	*ureasiticus*
Z0020_LB_E01_2_B06_A	GREEN	No	52	90	LMG 26304	*Enterococcus*	*ureasiticus*
Z0020_RB_D04_1_C11_B	GREEN	No	52	90	LMG 26304	*Enterococcus*	*ureasiticus*
Z0020_RB_D01_1_C12_A	GREEN	No	52	90	LMG 26304	*Enterococcus*	*ureasiticus*
Z0020_RB_D02_1_C12_B	GREEN	No	52	90	LMG 26304	*Enterococcus*	*ureasiticus*
Z0020_LB_D09_1_C02_A	GREEN	No	52	90	LMG 26304	*Enterococcus*	*ureasiticus*
Z0020_RB_C11_2_C07_A	GREEN	No	52	90	LMG 26304	*Enterococcus*	*ureasiticus*
Z0020_LB_D07_1_C03_A	GREEN	No	52	90	LMG 26304	*Enterococcus*	*ureasiticus*
Z0025_LO_B03_2_E02_A	GREEN	Yes	53	138	LMG 8787	*Curtobacterium*	*luteum*
Z0025_RO_A07_1_E10_A	GREEN	No	53	138	LMG 8787	*Curtobacterium*	*luteum*
Z0025_RO_A03_1_E08_A	GREEN	No	53	138	LMG 8787	*Curtobacterium*	*luteum*
Z0025_RO_A11_1_E12_A	GREEN	No	53	138	LMG 8787	*Curtobacterium*	*luteum*
Z0025_LO_A03_1_E02_A	GREEN	No	53	138	LMG 8787	*Curtobacterium*	*luteum*
Z0025_RO_A08_1_E10_B	GREEN	No	53	138	LMG 8787	*Curtobacterium*	*luteum*
Z0025_RO_A01_1_E07_A	GREEN	No	53	138	LMG 8787	*Curtobacterium*	*luteum*
Z0025_LO_A01_1_E01_A	GREEN	No	53	138	LMG 8787	*Curtobacterium*	*luteum*
Z0025_RO_A02_1_E07_B	GREEN	No	53	138	LMG 8787	*Curtobacterium*	*luteum*
Z0025_LO_B11_2_E06_A	GREEN	No	53	138	LMG 8787	*Curtobacterium*	*luteum*
Z0025_LO_B08_2_E04_B	GREEN	No	53	138	LMG 8787	*Curtobacterium*	*luteum*
Z0025_RO_A06_1_E09_B	GREEN	No	53	138	LMG 8787	*Curtobacterium*	*luteum*
Z0025_LO_A04_1_E02_B	GREEN	No	53	138	LMG 8787	*Curtobacterium*	*luteum*
Z0025_RO_A05_1_E09_A	GREEN	No	53	138	LMG 8787	*Curtobacterium*	*luteum*
Z0025_LO_A07_1_E04_A	GREEN	No	53	138	LMG 8787	*Curtobacterium*	*luteum*
Z0025_LO_B10_2_E05_B	GREEN	No	53	138	LMG 8787	*Curtobacterium*	*luteum*
Z0025_LO_B09_2_E05_A	GREEN	No	53	138	LMG 8787	*Curtobacterium*	*luteum*
Z0025_RO_A04_1_E08_B	GREEN	No	53	138	LMG 8787	*Curtobacterium*	*luteum*
Z0025_LO_B01_2_E01_A	GREEN	No	53	138	LMG 8787	*Curtobacterium*	*luteum*
Z0025_RO_A09_1_E11_A	GREEN	No	53	138	LMG 8787	*Curtobacterium*	*luteum*
Z0025_RO_B03_2_E08_A	GREEN	No	53	138	LMG 8787	*Curtobacterium*	*luteum*
Z0025_RO_B02_2_E07_B	GREEN	No	53	138	LMG 8787	*Curtobacterium*	*luteum*

Z0025_RO_A10_1_E11_B	GREEN	No	53	138	LMG 8787	*Curtobacterium*	*luteum*
Z0025_LO_B06_2_E03_B	GREEN	No	53	138	LMG 8787	*Curtobacterium*	*luteum*
Z0025_RO_B01_2_E07_A	GREEN	No	53	138	LMG 8787	*Curtobacterium*	*luteum*
Z0025_RO_A12_1_E12_B	GREEN	No	53	138	LMG 8787	*Curtobacterium*	*luteum*
Z0025_RO_B04_2_E08_B	GREEN	No	53	138	LMG 8787	*Curtobacterium*	*luteum*
Z0025_LO_B07_2_E04_A	GREEN	No	53	138	LMG 8787	*Curtobacterium*	*luteum*
Z0021_LO_A04_1_E02_B	GREEN	Yes	54	103	LMG 2811	*hermoanaerobacteriu*	*ermosaccharolyticum*
Z0021_LO_A03_1_E02_A	GREEN	No	54	103	LMG 2811	*hermoanaerobacteriu*	*ermosaccharolyticum*
Z0021_RO_A07_1_E10_A	GREEN	No	54	103	LMG 2811	*hermoanaerobacteriu*	*ermosaccharolyticum*
Z0021_LB_H09_1_A02_A	GREEN	No	54	103	LMG 2811	*hermoanaerobacteriu*	*ermosaccharolyticum*
Z0021_LO_A05_1_E03_A	GREEN	No	54	103	LMG 2811	*hermoanaerobacteriu*	*ermosaccharolyticum*
Z0021_LB_H06_1_A04_B	GREEN	No	54	103	LMG 2811	*hermoanaerobacteriu*	*ermosaccharolyticum*
Z0021_RB_H05_1_A10_A	GREEN	No	54	103	LMG 2811	*hermoanaerobacteriu*	*ermosaccharolyticum*
Z0021_RO_A08_1_E10_B	GREEN	No	54	103	LMG 2811	*hermoanaerobacteriu*	*ermosaccharolyticum*
Z0021_LB_H05_1_A04_A	GREEN	No	54	103	LMG 2811	*hermoanaerobacteriu*	*ermosaccharolyticum*
Z0021_LB_H07_1_A03_A	GREEN	No	54	103	LMG 2811	*hermoanaerobacteriu*	*ermosaccharolyticum*
Z0021_RB_H10_1_A08_B	GREEN	No	54	103	LMG 2811	*hermoanaerobacteriu*	*ermosaccharolyticum*
Z0021_LO_A08_1_E04_B	GREEN	No	54	103	LMG 2811	*hermoanaerobacteriu*	*ermosaccharolyticum*
Z0021_RO_A05_1_E09_A	GREEN	No	54	103	LMG 2811	*hermoanaerobacteriu*	*ermosaccharolyticum*
Z0021_RB_H11_1_A07_A	GREEN	No	54	103	LMG 2811	*hermoanaerobacteriu*	*ermosaccharolyticum*
Z0021_LO_A07_1_E04_A	GREEN	No	54	103	LMG 2811	*hermoanaerobacteriu*	*ermosaccharolyticum*
Z0021_LO_A06_1_E03_B	GREEN	No	54	103	LMG 2811	*hermoanaerobacteriu*	*ermosaccharolyticum*
Z0021_RB_H08_1_A09_B	GREEN	No	54	103	LMG 2811	*hermoanaerobacteriu*	*ermosaccharolyticum*
Z0021_LB_H11_1_A01_A	GREEN	No	54	103	LMG 2811	*hermoanaerobacteriu*	*ermosaccharolyticum*
Z0021_LO_A01_1_E01_A	GREEN	No	54	103	LMG 2811	*hermoanaerobacteriu*	*ermosaccharolyticum*
Z0021_RB_H12_1_A07_B	GREEN	No	54	103	LMG 2811	*hermoanaerobacteriu*	*ermosaccharolyticum*
Z0021_RO_A04_1_E08_B	GREEN	No	54	103	LMG 2811	*hermoanaerobacteriu*	*ermosaccharolyticum*
Z0021_LB_H12_1_A01_B	GREEN	No	54	103	LMG 2811	*hermoanaerobacteriu*	*ermosaccharolyticum*
Z0021_RO_A01_1_E07_A	GREEN	No	54	103	LMG 2811	*hermoanaerobacteriu*	*ermosaccharolyticum*
Z0021_LB_H10_1_A02_B	GREEN	No	54	103	LMG 2811	*hermoanaerobacteriu*	*ermosaccharolyticum*
Z0021_RO_A06_1_E09_B	GREEN	No	54	103	LMG 2811	*hermoanaerobacteriu*	*ermosaccharolyticum*
Z0021_LO_A02_1_E01_B	GREEN	No	54	103	LMG 2811	*hermoanaerobacteriu*	*ermosaccharolyticum*
Z0021_RO_A03_1_E08_A	GREEN	No	54	103	LMG 2811	*hermoanaerobacteriu*	*ermosaccharolyticum*
Z0021_LB_H08_1_A03_B	GREEN	No	54	103	LMG 2811	*hermoanaerobacteriu*	*ermosaccharolyticum*
Z0023_LB_C02_2_C06_B	GREEN	Yes	55	116	LMG 4233	*Photobacterium*	*phosphoreum*
Z0023_LB_B09_1_D02_A	GREEN	No	55	116	LMG 4233	*Photobacterium*	*phosphoreum*
Z0023_LB_B12_1_D01_B	GREEN	No	55	116	LMG 4233	*Photobacterium*	*phosphoreum*
Z0023_RB_A10_2_D08_B	GREEN	No	55	116	LMG 4233	*Photobacterium*	*phosphoreum*
Z0023_RB_B11_1_D07_A	GREEN	No	55	116	LMG 4233	*Photobacterium*	*phosphoreum*
Z0023_LB_C03_2_C05_A	GREEN	No	55	116	LMG 4233	*Photobacterium*	*phosphoreum*
Z0023_RB_C01_2_C12_A	GREEN	No	55	116	LMG 4233	*Photobacterium*	*phosphoreum*
Z0023_RB_B06_1_D10_B	GREEN	No	55	116	LMG 4233	*Photobacterium*	*phosphoreum*
Z0023_RB_C02_2_C12_B	GREEN	No	55	116	LMG 4233	*Photobacterium*	*phosphoreum*
Z0023_LB_C07_2_C03_A	GREEN	No	55	116	LMG 4233	*Photobacterium*	*phosphoreum*
Z0023_LB_B10_1_D02_B	GREEN	No	55	116	LMG 4233	*Photobacterium*	*phosphoreum*
Z0023_LB_B11_1_D01_A	GREEN	No	55	116	LMG 4233	*Photobacterium*	*phosphoreum*
Z0023_LB_B08_1_D03_B	GREEN	No	55	116	LMG 4233	*Photobacterium*	*phosphoreum*
Z0023_LB_B06_1_D04_B	GREEN	No	55	116	LMG 4233	*Photobacterium*	*phosphoreum*
Z0023_RB_B02_1_D12_B	GREEN	No	55	116	LMG 4233	*Photobacterium*	*phosphoreum*
Z0023_LB_C05_2_C04_A	GREEN	No	55	116	LMG 4233	*Photobacterium*	*phosphoreum*
Z0023_RB_B10_1_D08_B	GREEN	No	55	116	LMG 4233	*Photobacterium*	*phosphoreum*
Z0023_LB_C04_2_C05_B	GREEN	No	55	116	LMG 4233	*Photobacterium*	*phosphoreum*
Z0023_LB_C01_2_C06_A	GREEN	No	55	116	LMG 4233	*Photobacterium*	*phosphoreum*
Z0023_RB_A11_2_D07_A	GREEN	No	55	116	LMG 4233	*Photobacterium*	*phosphoreum*
Z0023_RB_A12_2_D07_B	GREEN	No	55	116	LMG 4233	*Photobacterium*	*phosphoreum*
Z0023_LB_B07_1_D03_A	GREEN	No	55	116	LMG 4233	*Photobacterium*	*phosphoreum*
Z0023_LB_B05_1_D04_A	GREEN	No	55	116	LMG 4233	*Photobacterium*	*phosphoreum*
Z0023_RB_B12_1_D07_B	GREEN	No	55	116	LMG 4233	*Photobacterium*	*phosphoreum*
Z0023_RB_B09_1_D08_A	GREEN	No	55	116	LMG 4233	*Photobacterium*	*phosphoreum*
Z0023_LB_C06_2_C04_B	GREEN	No	55	116	LMG 4233	*Photobacterium*	*phosphoreum*
Z0023_RB_B01_1_D12_A	GREEN	No	55	116	LMG 4233	*Photobacterium*	*phosphoreum*
Z0023_RB_B03_1_D11_A	GREEN	No	55	116	LMG 4233	*Photobacterium*	*phosphoreum*
Z0023_RB_B04_1_D11_B	GREEN	No	55	116	LMG 4233	*Photobacterium*	*phosphoreum*
Z0023_RB_B08_1_D09_B	GREEN	No	55	116	LMG 4233	*Photobacterium*	*phosphoreum*
Z0023_RB_B05_1_D10_A	GREEN	No	55	116	LMG 4233	*Photobacterium*	*phosphoreum*
Z0023_RB_B07_1_D09_A	GREEN	No	55	116	LMG 4233	*Photobacterium*	*phosphoreum*
Z0016_RB_D10_1_C08_B	GREEN	Yes	56	46	LMG 22485	*Burkholderia*	*lata*
Z0016_LB_D10_1_C02_B	GREEN	No	56	46	LMG 22485	*Burkholderia*	*lata*
Z0016_RB_E03_2_B11_A	GREEN	No	56	46	LMG 22485	*Burkholderia*	*lata*
Z0016_RB_D04_1_C11_B	GREEN	Yes	57	46	LMG 22485	*Burkholderia*	*lata*

Z0016_LB_D09_1_C02_A	GREEN	No	57	46	LMG 22485	*Burkholderia*	*lata*
Z0016_RB_E02_2_B12_B	GREEN	No	57	46	LMG 22485	*Burkholderia*	*lata*
Z0016_LB_E05_2_B04_A	GREEN	No	57	46	LMG 22485	*Burkholderia*	*lata*
Z0016_RB_D12_1_C07_B	GREEN	No	57	46	LMG 22485	*Burkholderia*	*lata*
Z0016_RB_E07_2_B09_A	GREEN	No	57	46	LMG 22485	*Burkholderia*	*lata*
Z0016_RB_D07_1_C09_A	GREEN	No	57	46	LMG 22485	*Burkholderia*	*lata*
Z0016_LB_D07_1_C03_A	GREEN	No	57	46	LMG 22485	*Burkholderia*	*lata*
Z0016_RB_E08_2_B09_B	GREEN	No	57	46	LMG 22485	*Burkholderia*	*lata*
Z0016_LB_E07_2_B03_A	GREEN	No	57	46	LMG 22485	*Burkholderia*	*lata*
Z0016_LB_D11_1_C01_A	GREEN	No	57	46	LMG 22485	*Burkholderia*	*lata*
Z0016_RB_D08_1_C09_B	GREEN	No	57	46	LMG 22485	*Burkholderia*	*lata*
Z0016_RB_D05_1_C10_A	GREEN	No	57	46	LMG 22485	*Burkholderia*	*lata*
Z0016_LB_D12_1_C01_B	GREEN	No	57	46	LMG 22485	*Burkholderia*	*lata*
Z0016_LB_E02_2_B06_B	GREEN	No	57	46	LMG 22485	*Burkholderia*	*lata*
Z0016_RB_E01_2_B12_A	GREEN	No	57	46	LMG 22485	*Burkholderia*	*lata*
Z0016_LB_E03_2_B05_A	GREEN	No	57	46	LMG 22485	*Burkholderia*	*lata*
Z0016_RB_D06_1_C10_B	GREEN	No	57	46	LMG 22485	*Burkholderia*	*lata*
Z0016_LB_E04_2_B05_B	GREEN	No	57	46	LMG 22485	*Burkholderia*	*lata*
Z0016_LB_E06_2_B04_B	GREEN	No	57	46	LMG 22485	*Burkholderia*	*lata*
Z0016_LB_E01_2_B06_A	GREEN	No	57	46	LMG 22485	*Burkholderia*	*lata*
Z0016_RB_D11_1_C07_A	GREEN	No	57	46	LMG 22485	*Burkholderia*	*lata*
Z0016_RB_D09_1_C08_A	GREEN	No	57	46	LMG 22485	*Burkholderia*	*lata*
Z0016_LB_E09_2_B02_A	GREEN	No	57	46	LMG 22485	*Burkholderia*	*lata*
Z0016_LB_D08_1_C03_B	GREEN	No	57	46	LMG 22485	*Burkholderia*	*lata*
Z0016_RB_E05_2_B10_A	GREEN	No	57	46	LMG 22485	*Burkholderia*	*lata*
Z0016_LB_E08_2_B03_B	GREEN	No	57	46	LMG 22485	*Burkholderia*	*lata*
Z0016_RB_E04_2_B11_B	GREEN	No	57	46	LMG 22485	*Burkholderia*	*lata*
Z0016_RB_E06_2_B10_B	GREEN	No	57	46	LMG 22485	*Burkholderia*	*lata*
Z0023_LB_G02_2_A06_B	GREEN	Yes	58	119	LMG 460	*Zymomonas*	*mobilis*
Z0023_LB_G08_2_A03_B	GREEN	No	58	119	LMG 460	*Zymomonas*	*mobilis*
Z0023_RB_F02_1_B12_B	GREEN	No	58	119	LMG 460	*Zymomonas*	*mobilis*
Z0023_LB_G04_2_A05_B	GREEN	No	58	119	LMG 460	*Zymomonas*	*mobilis*
Z0023_LB_G07_2_A03_A	GREEN	No	58	119	LMG 460	*Zymomonas*	*mobilis*
Z0023_LB_G01_2_A06_A	GREEN	No	58	119	LMG 460	*Zymomonas*	*mobilis*
Z0023_LB_G05_2_A04_A	GREEN	No	58	119	LMG 460	*Zymomonas*	*mobilis*
Z0023_LB_F08_1_B03_B	GREEN	No	58	119	LMG 460	*Zymomonas*	*mobilis*
Z0023_LB_G03_2_A05_A	GREEN	No	58	119	LMG 460	*Zymomonas*	*mobilis*
Z0023_LB_F11_1_B01_A	GREEN	No	58	119	LMG 460	*Zymomonas*	*mobilis*
Z0023_LB_G06_2_A04_B	GREEN	No	58	119	LMG 460	*Zymomonas*	*mobilis*
Z0023_RB_F03_1_B11_A	GREEN	No	58	119	LMG 460	*Zymomonas*	*mobilis*
Z0023_RB_F05_1_B10_A	GREEN	No	58	119	LMG 460	*Zymomonas*	*mobilis*
Z0023_RB_F07_1_B09_A	GREEN	No	58	119	LMG 460	*Zymomonas*	*mobilis*
Z0023_RB_F08_1_B09_B	GREEN	No	58	119	LMG 460	*Zymomonas*	*mobilis*
Z0023_LB_G12_2_A01_B	GREEN	No	58	119	LMG 460	*Zymomonas*	*mobilis*
Z0023_RB_F04_1_B11_B	GREEN	No	58	119	LMG 460	*Zymomonas*	*mobilis*
Z0023_LB_F07_1_B03_A	GREEN	No	58	119	LMG 460	*Zymomonas*	*mobilis*
Z0023_RB_F01_1_B12_A	GREEN	No	58	119	LMG 460	*Zymomonas*	*mobilis*
Z0023_RB_E09_2_B08_A	GREEN	No	58	119	LMG 460	*Zymomonas*	*mobilis*
Z0023_RB_F09_1_B08_A	GREEN	No	58	119	LMG 460	*Zymomonas*	*mobilis*
Z0023_RB_F06_1_B10_B	GREEN	No	58	119	LMG 460	*Zymomonas*	*mobilis*
Z0023_LB_F12_1_B01_B	GREEN	No	58	119	LMG 460	*Zymomonas*	*mobilis*
Z0023_LB_G09_2_A02_A	GREEN	No	58	119	LMG 460	*Zymomonas*	*mobilis*
Z0023_RB_E10_2_B08_B	GREEN	No	58	119	LMG 460	*Zymomonas*	*mobilis*
Z0023_LB_F06_1_B04_B	GREEN	No	58	119	LMG 460	*Zymomonas*	*mobilis*
Z0023_LB_G11_2_A01_A	GREEN	No	58	119	LMG 460	*Zymomonas*	*mobilis*
Z0023_LB_F09_1_B02_A	GREEN	No	58	119	LMG 460	*Zymomonas*	*mobilis*
Z0023_LB_F10_1_B02_B	GREEN	No	58	119	LMG 460	*Zymomonas*	*mobilis*
Z0023_RB_E11_2_B07_A	GREEN	No	58	119	LMG 460	*Zymomonas*	*mobilis*
Z0023_RB_E12_2_B07_B	GREEN	No	58	119	LMG 460	*Zymomonas*	*mobilis*
Z0023_LB_G10_2_A02_B	GREEN	No	58	119	LMG 460	*Zymomonas*	*mobilis*
Z0021_LB_G03_2_A05_A	GREEN	Yes	59	102	LMG 2804	*Dickeya*	*chrysanthemi*
Z0021_LB_F12_1_B01_B	GREEN	No	59	102	LMG 2804	*Dickeya*	*chrysanthemi*
Z0021_RB_G08_2_A09_B	GREEN	No	59	102	LMG 2804	*Dickeya*	*chrysanthemi*
Z0021_LB_G05_2_A04_A	GREEN	No	59	102	LMG 2804	*Dickeya*	*chrysanthemi*
Z0021_LB_G01_2_A06_A	GREEN	No	59	102	LMG 2804	*Dickeya*	*chrysanthemi*
Z0021_LB_G02_2_A06_B	GREEN	No	59	102	LMG 2804	*Dickeya*	*chrysanthemi*
Z0021_RB_G02_2_A12_B	GREEN	No	59	102	LMG 2804	*Dickeya*	*chrysanthemi*
Z0021_RB_G03_2_A11_A	GREEN	No	59	102	LMG 2804	*Dickeya*	*chrysanthemi*
Z0021_RB_G07_2_A09_A	GREEN	No	59	102	LMG 2804	*Dickeya*	*chrysanthemi*
Z0021_RB_G12_2_A07_B	GREEN	No	59	102	LMG 2804	*Dickeya*	*chrysanthemi*

Z0021_RB_G05_2_A10_A	GREEN	No	59	102	LMG 2804	*Dickeya*	*chrysanthemi*
Z0021_LB_G07_2_A03_A	GREEN	No	59	102	LMG 2804	*Dickeya*	*chrysanthemi*
Z0021_RB_H01_1_A12_A	GREEN	No	59	102	LMG 2804	*Dickeya*	*chrysanthemi*
Z0021_RB_G04_2_A11_B	GREEN	No	59	102	LMG 2804	*Dickeya*	*chrysanthemi*
Z0021_RB_G06_2_A10_B	GREEN	No	59	102	LMG 2804	*Dickeya*	*chrysanthemi*
Z0021_LB_H03_1_A05_A	GREEN	No	59	102	LMG 2804	*Dickeya*	*chrysanthemi*
Z0021_LB_G06_2_A04_B	GREEN	No	59	102	LMG 2804	*Dickeya*	*chrysanthemi*
Z0021_LB_G11_2_A01_A	GREEN	No	59	102	LMG 2804	*Dickeya*	*chrysanthemi*
Z0021_LB_H01_1_A06_A	GREEN	No	59	102	LMG 2804	*Dickeya*	*chrysanthemi*
Z0021_RB_H02_1_A12_B	GREEN	No	59	102	LMG 2804	*Dickeya*	*chrysanthemi*
Z0021_LB_G12_2_A01_B	GREEN	No	59	102	LMG 2804	*Dickeya*	*chrysanthemi*
Z0021_RB_G11_2_A07_A	GREEN	No	59	102	LMG 2804	*Dickeya*	*chrysanthemi*
Z0021_LB_H02_1_A06_B	GREEN	No	59	102	LMG 2804	*Dickeya*	*chrysanthemi*
Z0021_LB_G04_2_A05_B	GREEN	No	59	102	LMG 2804	*Dickeya*	*chrysanthemi*
Z0021_RB_H04_1_A11_B	GREEN	No	59	102	LMG 2804	*Dickeya*	*chrysanthemi*
Z0021_LB_G09_2_A02_A	GREEN	No	59	102	LMG 2804	*Dickeya*	*chrysanthemi*
Z0021_LB_G08_2_A03_B	GREEN	No	59	102	LMG 2804	*Dickeya*	*chrysanthemi*
Z0021_RB_G10_2_A08_B	GREEN	No	59	102	LMG 2804	*Dickeya*	*chrysanthemi*
Z0021_LB_G10_2_A02_B	GREEN	No	59	102	LMG 2804	*Dickeya*	*chrysanthemi*
Z0021_LB_H04_1_A05_B	GREEN	No	59	102	LMG 2804	*Dickeya*	*chrysanthemi*
Z0021_RB_H03_1_A11_A	GREEN	No	59	102	LMG 2804	*Dickeya*	*chrysanthemi*
Z0021_RB_G09_2_A08_A	GREEN	No	59	102	LMG 2804	*Dickeya*	*chrysanthemi*
Z0019_RB_D07_1_C09_A	GREEN	Yes	60	80	LMG 25535	*Arcobacter*	*trophiarum*
Z0019_RB_D12_1_C07_B	GREEN	No	60	80	LMG 25535	*Arcobacter*	*trophiarum*
Z0019_RB_D01_1_C12_A	GREEN	No	60	80	LMG 25535	*Arcobacter*	*trophiarum*
Z0019_LB_E02_2_B06_B	GREEN	No	60	80	LMG 25535	*Arcobacter*	*trophiarum*
Z0019_RB_E03_2_B11_A	GREEN	No	60	80	LMG 25535	*Arcobacter*	*trophiarum*
Z0019_RB_D09_1_C08_A	GREEN	No	60	80	LMG 25535	*Arcobacter*	*trophiarum*
Z0019_LB_D11_1_C01_A	GREEN	No	60	80	LMG 25535	*Arcobacter*	*trophiarum*
Z0019_RB_E05_2_B10_A	GREEN	No	60	80	LMG 25535	*Arcobacter*	*trophiarum*
Z0019_LB_E03_2_B05_A	GREEN	No	60	80	LMG 25535	*Arcobacter*	*trophiarum*
Z0019_RB_D02_1_C12_B	GREEN	No	60	80	LMG 25535	*Arcobacter*	*trophiarum*
Z0019_LB_D10_1_C02_B	GREEN	No	60	80	LMG 25535	*Arcobacter*	*trophiarum*
Z0019_RB_D03_1_C11_A	GREEN	No	60	80	LMG 25535	*Arcobacter*	*trophiarum*
Z0019_RB_E01_2_B12_A	GREEN	No	60	80	LMG 25535	*Arcobacter*	*trophiarum*
Z0019_RB_E04_2_B11_B	GREEN	No	60	80	LMG 25535	*Arcobacter*	*trophiarum*
Z0019_RB_E06_2_B10_B	GREEN	No	60	80	LMG 25535	*Arcobacter*	*trophiarum*
Z0019_RB_D05_1_C10_A	GREEN	No	60	80	LMG 25535	*Arcobacter*	*trophiarum*
Z0019_LB_E05_2_B04_A	GREEN	No	60	80	LMG 25535	*Arcobacter*	*trophiarum*
Z0019_LB_E04_2_B05_B	GREEN	No	60	80	LMG 25535	*Arcobacter*	*trophiarum*
Z0019_RB_D04_1_C11_B	GREEN	No	60	80	LMG 25535	*Arcobacter*	*trophiarum*
Z0019_LB_E01_2_B06_A	GREEN	No	60	80	LMG 25535	*Arcobacter*	*trophiarum*
Z0019_LB_D09_1_C02_A	GREEN	No	60	80	LMG 25535	*Arcobacter*	*trophiarum*
Z0019_RB_C12_2_C07_B	GREEN	No	60	80	LMG 25535	*Arcobacter*	*trophiarum*
Z0019_RB_E02_2_B12_B	GREEN	No	60	80	LMG 25535	*Arcobacter*	*trophiarum*
Z0019_LB_D12_1_C01_B	GREEN	No	60	80	LMG 25535	*Arcobacter*	*trophiarum*
Z0019_LB_D07_1_C03_A	GREEN	No	60	80	LMG 25535	*Arcobacter*	*trophiarum*
Z0019_LB_E07_2_B03_A	GREEN	No	60	80	LMG 25535	*Arcobacter*	*trophiarum*
Z0019_RB_D06_1_C10_B	GREEN	No	60	80	LMG 25535	*Arcobacter*	*trophiarum*
Z0019_RB_D11_1_C07_A	GREEN	No	60	80	LMG 25535	*Arcobacter*	*trophiarum*
Z0019_LB_E06_2_B04_B	GREEN	No	60	80	LMG 25535	*Arcobacter*	*trophiarum*
Z0019_RB_D10_1_C08_B	GREEN	No	60	80	LMG 25535	*Arcobacter*	*trophiarum*
Z0019_LB_D08_1_C03_B	GREEN	No	60	80	LMG 25535	*Arcobacter*	*trophiarum*
Z0019_RB_D08_1_C09_B	GREEN	Yes	61	80	LMG 25535	*Arcobacter*	*trophiarum*
Z0012_LO_G01_1_H01_A	GREEN	Yes	62	11	LMG 1286	*Pantoea*	*agglomerans*
Z0012_LO_E09_1_G05_A	GREEN	No	62	11	LMG 1286	*Pantoea*	*agglomerans*
Z0012_LO_G03_1_H02_A	GREEN	No	62	11	LMG 1286	*Pantoea*	*agglomerans*
Z0012_RO_D06_2_F09_B	GREEN	No	62	11	LMG 1286	*Pantoea*	*agglomerans*
Z0012_RO_D08_2_F10_B	GREEN	No	62	11	LMG 1286	*Pantoea*	*agglomerans*
Z0012_LO_E08_1_G04_B	GREEN	No	62	11	LMG 1286	*Pantoea*	*agglomerans*
Z0012_RO_E03_1_G08_A	GREEN	No	62	11	LMG 1286	*Pantoea*	*agglomerans*
Z0012_RO_E06_1_G09_B	GREEN	No	62	11	LMG 1286	*Pantoea*	*agglomerans*
Z0012_LO_D03_2_F02_A	GREEN	No	62	11	LMG 1286	*Pantoea*	*agglomerans*
Z0012_LO_E01_1_G01_A	GREEN	No	62	11	LMG 1286	*Pantoea*	*agglomerans*
Z0012_RO_E04_1_G08_B	GREEN	No	62	11	LMG 1286	*Pantoea*	*agglomerans*
Z0012_LO_F05_2_G03_A	GREEN	No	62	11	LMG 1286	*Pantoea*	*agglomerans*
Z0012_RO_E12_1_G12_B	GREEN	No	62	11	LMG 1286	*Pantoea*	*agglomerans*
Z0012_RO_E05_1_G09_A	GREEN	No	62	11	LMG 1286	*Pantoea*	*agglomerans*
Z0012_LO_D07_2_F04_A	GREEN	No	62	11	LMG 1286	*Pantoea*	*agglomerans*
Z0012_RO_E11_1_G12_A	GREEN	No	62	11	LMG 1286	*Pantoea*	*agglomerans*

Z0012_RO_F02_2_G07_B	GREEN	No	62	11	LMG 1286	*Pantoea*	*agglomerans*
Z0012_LO_E06_1_G03_B	GREEN	No	62	11	LMG 1286	*Pantoea*	*agglomerans*
Z0012_LO_E10_1_G05_B	GREEN	No	62	11	LMG 1286	*Pantoea*	*agglomerans*
Z0012_LO_F12_2_G06_B	GREEN	No	62	11	LMG 1286	*Pantoea*	*agglomerans*
Z0012_LO_D04_2_F02_B	GREEN	No	62	11	LMG 1286	*Pantoea*	*agglomerans*
Z0012_RO_D04_2_F08_B	GREEN	No	62	11	LMG 1286	*Pantoea*	*agglomerans*
Z0012_LO_D09_2_F05_A	GREEN	No	62	11	LMG 1286	*Pantoea*	*agglomerans*
Z0012_RO_E02_1_G07_B	GREEN	No	62	11	LMG 1286	*Pantoea*	*agglomerans*
Z0012_LO_E02_1_G01_B	GREEN	No	62	11	LMG 1286	*Pantoea*	*agglomerans*
Z0012_RO_C11_1_F12_A	GREEN	No	62	11	LMG 1286	*Pantoea*	*agglomerans*
Z0012_RO_D01_2_F07_A	GREEN	No	62	11	LMG 1286	*Pantoea*	*agglomerans*
Z0012_RO_E01_1_G07_A	GREEN	No	62	11	LMG 1286	*Pantoea*	*agglomerans*
Z0012_LO_F06_2_G03_B	GREEN	No	62	11	LMG 1286	*Pantoea*	*agglomerans*
Z0012_LO_F04_2_G02_B	GREEN	No	62	11	LMG 1286	*Pantoea*	*agglomerans*
Z0012_LO_F02_2_G01_B	GREEN	No	62	11	LMG 1286	*Pantoea*	*agglomerans*
Z0012_LO_F07_2_G04_A	GREEN	No	62	11	LMG 1286	*Pantoea*	*agglomerans*
Z0016_RB_H09_1_A08_A	GREEN	Yes	63	49	LMG 22735	*Finegoldia*	*magna*
Z0016_LB_H09_1_A02_A	GREEN	No	63	49	LMG 22735	*Finegoldia*	*magna*
Z0016_RB_H07_1_A09_A	GREEN	No	63	49	LMG 22735	*Finegoldia*	*magna*
Z0016_LO_A04_1_E02_B	GREEN	No	63	49	LMG 22735	*Finegoldia*	*magna*
Z0016_LB_H10_1_A02_B	GREEN	No	63	49	LMG 22735	*Finegoldia*	*magna*
Z0016_RB_H10_1_A08_B	GREEN	No	63	49	LMG 22735	*Finegoldia*	*magna*
Z0016_LB_H07_1_A03_A	GREEN	No	63	49	LMG 22735	*Finegoldia*	*magna*
Z0016_LO_A09_1_E05_A	GREEN	No	63	49	LMG 22735	*Finegoldia*	*magna*
Z0016_RO_A04_1_E08_B	GREEN	No	63	49	LMG 22735	*Finegoldia*	*magna*
Z0016_LB_H11_1_A01_A	GREEN	No	63	49	LMG 22735	*Finegoldia*	*magna*
Z0016_RO_A03_1_E08_A	GREEN	No	63	49	LMG 22735	*Finegoldia*	*magna*
Z0016_LO_A08_1_E04_B	GREEN	No	63	49	LMG 22735	*Finegoldia*	*magna*
Z0016_RB_H12_1_A07_B	GREEN	No	63	49	LMG 22735	*Finegoldia*	*magna*
Z0016_RB_H04_1_A11_B	GREEN	No	63	49	LMG 22735	*Finegoldia*	*magna*
Z0016_RO_A01_1_E07_A	GREEN	No	63	49	LMG 22735	*Finegoldia*	*magna*
Z0016_RB_H08_1_A09_B	GREEN	No	63	49	LMG 22735	*Finegoldia*	*magna*
Z0016_LO_A02_1_E01_B	GREEN	No	63	49	LMG 22735	*Finegoldia*	*magna*
Z0016_LB_H08_1_A03_B	GREEN	No	63	49	LMG 22735	*Finegoldia*	*magna*
Z0016_LB_H12_1_A01_B	GREEN	No	63	49	LMG 22735	*Finegoldia*	*magna*
Z0016_RB_H11_1_A07_A	GREEN	No	63	49	LMG 22735	*Finegoldia*	*magna*
Z0016_RB_H06_1_A10_B	GREEN	No	63	49	LMG 22735	*Finegoldia*	*magna*
Z0016_LO_A03_1_E02_A	GREEN	No	63	49	LMG 22735	*Finegoldia*	*magna*
Z0016_LO_A01_1_E01_A	GREEN	No	63	49	LMG 22735	*Finegoldia*	*magna*
Z0016_LO_A07_1_E04_A	GREEN	No	63	49	LMG 22735	*Finegoldia*	*magna*
Z0016_LO_A06_1_E03_B	GREEN	No	63	49	LMG 22735	*Finegoldia*	*magna*
Z0016_LO_A05_1_E03_A	GREEN	No	63	49	LMG 22735	*Finegoldia*	*magna*
Z0016_RB_H05_1_A10_A	GREEN	No	63	49	LMG 22735	*Finegoldia*	*magna*
Z0016_RO_A02_1_E07_B	GREEN	No	63	49	LMG 22735	*Finegoldia*	*magna*
Z0013_LB_B01_1_D06_A	GREEN	Yes	64	16	LMG 1346	*Marinilabilia*	*salmonicolor*
Z0013_LB_B04_1_D05_B	GREEN	No	64	16	LMG 1346	*Marinilabilia*	*salmonicolor*
Z0013_LB_B08_1_D03_B	GREEN	No	64	16	LMG 1346	*Marinilabilia*	*salmonicolor*
Z0013_RB_B01_1_D12_A	GREEN	No	64	16	LMG 1346	*Marinilabilia*	*salmonicolor*
Z0013_LB_B03_1_D05_A	GREEN	No	64	16	LMG 1346	*Marinilabilia*	*salmonicolor*
Z0013_RB_A12_2_D07_B	GREEN	No	64	16	LMG 1346	*Marinilabilia*	*salmonicolor*
Z0013_RO_B07_2_E10_A	GREEN	No	64	16	LMG 1346	*Marinilabilia*	*salmonicolor*
Z0013_RB_B03_1_D11_A	GREEN	No	64	16	LMG 1346	*Marinilabilia*	*salmonicolor*
Z0013_RB_A11_2_D07_A	GREEN	No	64	16	LMG 1346	*Marinilabilia*	*salmonicolor*
Z0013_RB_B02_1_D12_B	GREEN	No	64	16	LMG 1346	*Marinilabilia*	*salmonicolor*
Z0013_LO_B07_2_E04_A	GREEN	No	64	16	LMG 1346	*Marinilabilia*	*salmonicolor*
Z0013_RB_A10_2_D08_B	GREEN	No	64	16	LMG 1346	*Marinilabilia*	*salmonicolor*
Z0013_RO_B02_2_E07_B	GREEN	No	64	16	LMG 1346	*Marinilabilia*	*salmonicolor*
Z0013_LO_B04_2_E02_B	GREEN	No	64	16	LMG 1346	*Marinilabilia*	*salmonicolor*
Z0013_LO_B08_2_E04_B	GREEN	No	64	16	LMG 1346	*Marinilabilia*	*salmonicolor*
Z0013_LO_B06_2_E03_B	GREEN	No	64	16	LMG 1346	*Marinilabilia*	*salmonicolor*
Z0013_RB_B04_1_D11_B	GREEN	No	64	16	LMG 1346	*Marinilabilia*	*salmonicolor*
Z0013_RO_B06_2_E09_B	GREEN	No	64	16	LMG 1346	*Marinilabilia*	*salmonicolor*
Z0013_RO_B04_2_E08_B	GREEN	No	64	16	LMG 1346	*Marinilabilia*	*salmonicolor*
Z0013_RO_B05_2_E09_A	GREEN	No	64	16	LMG 1346	*Marinilabilia*	*salmonicolor*
Z0013_LO_B03_2_E02_A	GREEN	No	64	16	LMG 1346	*Marinilabilia*	*salmonicolor*
Z0013_RO_A12_1_E12_B	GREEN	No	64	16	LMG 1346	*Marinilabilia*	*salmonicolor*
Z0013_LB_B06_1_D04_B	GREEN	No	64	16	LMG 1346	*Marinilabilia*	*salmonicolor*
Z0013_LB_B05_1_D04_A	GREEN	No	64	16	LMG 1346	*Marinilabilia*	*salmonicolor*
Z0013_RB_B06_1_D10_B	GREEN	No	64	16	LMG 1346	*Marinilabilia*	*salmonicolor*
Z0013_LO_B05_2_E03_A	GREEN	No	64	16	LMG 1346	*Marinilabilia*	*salmonicolor*

Z0013_RB_B05_1_D10_A	GREEN	No	64	16	LMG 1346	*Marinilabilia*	*salmonicolor*
Z0013_LB_B02_1_D06_B	GREEN	No	64	16	LMG 1346	*Marinilabilia*	*salmonicolor*
Z0013_LB_A12_2_D01_B	GREEN	No	64	16	LMG 1346	*Marinilabilia*	*salmonicolor*
Z0013_RO_B03_2_E08_A	GREEN	No	64	16	LMG 1346	*Marinilabilia*	*salmonicolor*
Z0013_RO_B01_2_E07_A	GREEN	No	64	16	LMG 1346	*Marinilabilia*	*salmonicolor*
Z0013_LB_B07_1_D03_A	GREEN	No	64	16	LMG 1346	*Marinilabilia*	*salmonicolor*
Z0016_LO_F10_2_G05_B	GREEN	Yes	65	54	LMG 23083	*Asaia*	*krungthepensis*
Z0016_RO_D12_2_F12_B	GREEN	No	65	54	LMG 23083	*Asaia*	*krungthepensis*
Z0016_RO_F02_2_G07_B	GREEN	No	65	54	LMG 23083	*Asaia*	*krungthepensis*
Z0016_LO_F08_2_G04_B	GREEN	No	65	54	LMG 23083	*Asaia*	*krungthepensis*
Z0016_LO_C12_1_F06_B	GREEN	No	65	54	LMG 23083	*Asaia*	*krungthepensis*
Z0016_LO_F04_2_G02_B	GREEN	No	65	54	LMG 23083	*Asaia*	*krungthepensis*
Z0016_LO_F11_2_G06_A	GREEN	No	65	54	LMG 23083	*Asaia*	*krungthepensis*
Z0016_RO_E01_1_G07_A	GREEN	No	65	54	LMG 23083	*Asaia*	*krungthepensis*
Z0016_RO_E02_1_G07_B	GREEN	No	65	54	LMG 23083	*Asaia*	*krungthepensis*
Z0016_LO_D08_2_F04_B	GREEN	No	65	54	LMG 23083	*Asaia*	*krungthepensis*
Z0016_RO_C02_1_F07_B	GREEN	No	65	54	LMG 23083	*Asaia*	*krungthepensis*
Z0016_RO_C01_1_F07_A	GREEN	No	65	54	LMG 23083	*Asaia*	*krungthepensis*
Z0016_LO_D09_2_F05_A	GREEN	No	65	54	LMG 23083	*Asaia*	*krungthepensis*
Z0016_RO_D05_2_F09_A	GREEN	No	65	54	LMG 23083	*Asaia*	*krungthepensis*
Z0016_LO_D02_2_F01_B	GREEN	No	65	54	LMG 23083	*Asaia*	*krungthepensis*
Z0016_LO_E09_1_G05_A	GREEN	No	65	54	LMG 23083	*Asaia*	*krungthepensis*
Z0016_RO_B02_2_E07_B	GREEN	No	65	54	LMG 23083	*Asaia*	*krungthepensis*
Z0016_LO_E12_1_G06_B	GREEN	No	65	54	LMG 23083	*Asaia*	*krungthepensis*
Z0016_LO_C11_1_F06_A	GREEN	No	65	54	LMG 23083	*Asaia*	*krungthepensis*
Z0016_LO_F05_2_G03_A	GREEN	No	65	54	LMG 23083	*Asaia*	*krungthepensis*
Z0016_RO_D10_2_F11_B	GREEN	No	65	54	LMG 23083	*Asaia*	*krungthepensis*
Z0016_RO_D04_2_F08_B	GREEN	No	65	54	LMG 23083	*Asaia*	*krungthepensis*
Z0016_LO_D01_2_F01_A	GREEN	No	65	54	LMG 23083	*Asaia*	*krungthepensis*
Z0016_LO_D10_2_F05_B	GREEN	No	65	54	LMG 23083	*Asaia*	*krungthepensis*
Z0016_RO_A10_1_E11_B	GREEN	No	65	54	LMG 23083	*Asaia*	*krungthepensis*
Z0016_RO_C07_1_F10_A	GREEN	No	65	54	LMG 23083	*Asaia*	*krungthepensis*
Z0016_RO_D11_2_F12_A	GREEN	No	65	54	LMG 23083	*Asaia*	*krungthepensis*
Z0016_LO_E07_1_G04_A	GREEN	No	65	54	LMG 23083	*Asaia*	*krungthepensis*
Z0016_LO_E06_1_G03_B	GREEN	No	65	54	LMG 23083	*Asaia*	*krungthepensis*
Z0016_RO_E09_1_G11_A	GREEN	No	65	54	LMG 23083	*Asaia*	*krungthepensis*
Z0016_LO_F02_2_G01_B	GREEN	No	65	54	LMG 23083	*Asaia*	*krungthepensis*
Z0016_RO_B01_2_E07_A	GREEN	No	65	54	LMG 23083	*Asaia*	*krungthepensis*
Z0016_RO_B10_2_E11_B	GREEN	Yes	66	51	LMG 23037	*Geobacillus*	*toebii*
Z0016_LO_C04_1_F02_B	GREEN	No	66	51	LMG 23037	*Geobacillus*	*toebii*
Z0016_LO_C03_1_F02_A	GREEN	No	66	51	LMG 23037	*Geobacillus*	*toebii*
Z0016_RO_D03_2_F08_A	GREEN	No	66	51	LMG 23037	*Geobacillus*	*toebii*
Z0016_RO_B11_2_E12_A	GREEN	No	66	51	LMG 23037	*Geobacillus*	*toebii*
Z0016_RO_D09_2_F11_A	GREEN	No	66	51	LMG 23037	*Geobacillus*	*toebii*
Z0016_LO_C10_1_F05_B	GREEN	No	66	51	LMG 23037	*Geobacillus*	*toebii*
Z0016_LO_D11_2_F06_A	GREEN	No	66	51	LMG 23037	*Geobacillus*	*toebii*
Z0016_RO_C08_1_F10_B	GREEN	No	66	51	LMG 23037	*Geobacillus*	*toebii*
Z0016_RO_B12_2_E12_B	GREEN	No	66	51	LMG 23037	*Geobacillus*	*toebii*
Z0016_RO_B09_2_E11_A	GREEN	No	66	51	LMG 23037	*Geobacillus*	*toebii*
Z0016_LO_D03_2_F02_A	GREEN	No	66	51	LMG 23037	*Geobacillus*	*toebii*
Z0016_RO_C06_1_F09_B	GREEN	No	66	51	LMG 23037	*Geobacillus*	*toebii*
Z0016_LO_C05_1_F03_A	GREEN	No	66	51	LMG 23037	*Geobacillus*	*toebii*
Z0016_LO_C09_1_F05_A	GREEN	No	66	51	LMG 23037	*Geobacillus*	*toebii*
Z0016_LO_D05_2_F03_A	GREEN	No	66	51	LMG 23037	*Geobacillus*	*toebii*
Z0016_LO_D07_2_F04_A	GREEN	No	66	51	LMG 23037	*Geobacillus*	*toebii*
Z0016_LO_D06_2_F03_B	GREEN	No	66	51	LMG 23037	*Geobacillus*	*toebii*
Z0016_RO_B08_2_E10_B	GREEN	No	66	51	LMG 23037	*Geobacillus*	*toebii*
Z0016_LO_C07_1_F04_A	GREEN	No	66	51	LMG 23037	*Geobacillus*	*toebii*
Z0016_RO_C09_1_F11_A	GREEN	No	66	51	LMG 23037	*Geobacillus*	*toebii*
Z0016_LO_E01_1_G01_A	GREEN	No	66	51	LMG 23037	*Geobacillus*	*toebii*
Z0016_RO_C10_1_F11_B	GREEN	No	66	51	LMG 23037	*Geobacillus*	*toebii*
Z0016_RO_D01_2_F07_A	GREEN	No	66	51	LMG 23037	*Geobacillus*	*toebii*
Z0016_RO_C11_1_F12_A	GREEN	No	66	51	LMG 23037	*Geobacillus*	*toebii*
Z0016_RO_D06_2_F09_B	GREEN	No	66	51	LMG 23037	*Geobacillus*	*toebii*
Z0016_RO_C04_1_F08_B	GREEN	No	66	51	LMG 23037	*Geobacillus*	*toebii*
Z0016_LO_D12_2_F06_B	GREEN	No	66	51	LMG 23037	*Geobacillus*	*toebii*
Z0016_RO_C03_1_F08_A	GREEN	No	66	51	LMG 23037	*Geobacillus*	*toebii*
Z0016_RO_D08_2_F10_B	GREEN	No	66	51	LMG 23037	*Geobacillus*	*toebii*
Z0016_RO_C12_1_F12_B	GREEN	No	66	51	LMG 23037	*Geobacillus*	*toebii*
Z0016_RO_D07_2_F10_A	GREEN	No	66	51	LMG 23037	*Geobacillus*	*toebii*

Z0024_LO_F02_2_G01_B	GREEN	Yes	67	130	LMG 7123	*Brevibacillus*	*brevis*	
Z0024_RO_F07_2_G10_A	GREEN	No	67	130	LMG 7123	*Brevibacillus*	*brevis*	
Z0024_LO_H01_2_H01_A	GREEN	No	67	130	LMG 7123	*Brevibacillus*	*brevis*	
Z0024_RO_D08_2_F10_B	GREEN	No	67	130	LMG 7123	*Brevibacillus*	*brevis*	
Z0024_RO_F04_2_G08_B	GREEN	No	67	130	LMG 7123	*Brevibacillus*	*brevis*	
Z0024_LO_E12_1_G06_B	GREEN	No	67	130	LMG 7123	*Brevibacillus*	*brevis*	
Z0024_LO_F11_2_G06_A	GREEN	No	67	130	LMG 7123	*Brevibacillus*	*brevis*	
Z0024_LO_G05_1_H03_A	GREEN	No	67	130	LMG 7123	*Brevibacillus*	*brevis*	
Z0024_LO_H06_2_H03_B	GREEN	No	67	130	LMG 7123	*Brevibacillus*	*brevis*	
Z0024_LO_H03_2_H02_A	GREEN	No	67	130	LMG 7123	*Brevibacillus*	*brevis*	
Z0024_LO_G06_1_H03_B	GREEN	No	67	130	LMG 7123	*Brevibacillus*	*brevis*	
Z0024_RO_F06_2_G09_B	GREEN	No	67	130	LMG 7123	*Brevibacillus*	*brevis*	
Z0024_LO_G03_1_H02_A	GREEN	No	67	130	LMG 7123	*Brevibacillus*	*brevis*	
Z0024_LO_E10_1_G05_B	GREEN	No	67	130	LMG 7123	*Brevibacillus*	*brevis*	
Z0024_LO_G01_1_H01_A	GREEN	No	67	130	LMG 7123	*Brevibacillus*	*brevis*	
Z0024_RO_F01_2_G07_A	GREEN	No	67	130	LMG 7123	*Brevibacillus*	*brevis*	
Z0024_LO_G02_1_H01_B	GREEN	No	67	130	LMG 7123	*Brevibacillus*	*brevis*	
Z0024_RO_D07_2_F10_A	GREEN	No	67	130	LMG 7123	*Brevibacillus*	*brevis*	
Z0024_LO_F08_2_G04_B	GREEN	No	67	130	LMG 7123	*Brevibacillus*	*brevis*	
Z0024_LO_F09_2_G05_A	GREEN	No	67	130	LMG 7123	*Brevibacillus*	*brevis*	
Z0024_LO_G07_1_H04_A	GREEN	No	67	130	LMG 7123	*Brevibacillus*	*brevis*	
Z0024_LO_E11_1_G06_A	GREEN	No	67	130	LMG 7123	*Brevibacillus*	*brevis*	
Z0024_LO_F03_2_G02_A	GREEN	No	67	130	LMG 7123	*Brevibacillus*	*brevis*	
Z0024_LO_F07_2_G04_A	GREEN	No	67	130	LMG 7123	*Brevibacillus*	*brevis*	
Z0024_RO_E12_1_G12_B	GREEN	No	67	130	LMG 7123	*Brevibacillus*	*brevis*	
Z0024_LO_F10_2_G05_B	GREEN	No	67	130	LMG 7123	*Brevibacillus*	*brevis*	
Z0024_LO_G04_1_H02_B	GREEN	No	67	130	LMG 7123	*Brevibacillus*	*brevis*	
Z0024_LO_F01_2_G01_A	GREEN	No	67	130	LMG 7123	*Brevibacillus*	*brevis*	
Z0024_RO_F02_2_G07_B	GREEN	No	67	130	LMG 7123	*Brevibacillus*	*brevis*	
Z0024_LO_G08_1_H04_B	GREEN	No	67	130	LMG 7123	*Brevibacillus*	*brevis*	
Z0024_LO_F12_2_G06_B	GREEN	No	67	130	LMG 7123	*Brevibacillus*	*brevis*	
Z0024_LO_F04_2_G02_B	GREEN	No	67	130	LMG 7123	*Brevibacillus*	*brevis*	
Z0011_LO_A05_1_E03_A	GREEN	Yes	68	147	R-68675	*Burkholderia*	*cenocepacia*	*IIIA*
Z0010_RB_A02_2_D12_B	GREEN	No	68	147	R-68806	*Burkholderia*	*cenocepacia*	*IIIA*
Z0010_RO_C08_1_F10_B	GREEN	No	68	147	R-68806	*Burkholderia*	*cenocepacia*	*IIIA*
Z0010_LB_A07_2_D03_A	GREEN	No	68	147	R-68806	*Burkholderia*	*cenocepacia*	*IIIA*
Z0010_LB_A05_2_D04_A	GREEN	No	68	147	R-68806	*Burkholderia*	*cenocepacia*	*IIIA*
Z0010_LB_H04_1_A05_B	GREEN	No	68	147	R-71085	*Burkholderia*	*cenocepacia*	*IIIA*
Z0010_RB_G08_2_A09_B	GREEN	No	68	147	R-71085	*Burkholderia*	*cenocepacia*	*IIIA*
Z0010_RB_H03_1_A11_A	GREEN	No	68	147	R-71085	*Burkholderia*	*cenocepacia*	*IIIA*
Z0010_LB_H05_1_A04_A	GREEN	No	68	147	R-71085	*Burkholderia*	*cenocepacia*	*IIIA*
Z0011_RB_H06_1_A10_B	GREEN	No	68	147	R-68675	*Burkholderia*	*cenocepacia*	*IIIA*
Z0011_LO_A07_1_E04_A	GREEN	No	68	147	R-68675	*Burkholderia*	*cenocepacia*	*IIIA*
Z0011_LO_B03_2_E02_A	GREEN	No	68	147	R-68675	*Burkholderia*	*cenocepacia*	*IIIA*
Z0011_LO_A03_1_E02_A	GREEN	Yes	69	146	R-68599	*Burkholderia*	*cenocepacia*	*IIIB*
Z0011_LB_C04_2_C05_B	GREEN	No	69	145	R-67259	*Burkholderia*	*cenocepacia*	*IIIB*
Z0011_LB_G10_2_A02_B	GREEN	Yes	70	146	R-68591	*Burkholderia*	*cenocepacia*	*IIIB*
Z0010_RO_C05_1_F09_A	GREEN	No	70	147	R-68806	*Burkholderia*	*cenocepacia*	*IIIA*
Z0010_LO_D04_2_F02_B	GREEN	No	70	147	R-68806	*Burkholderia*	*cenocepacia*	*IIIA*
Z0010_LB_A08_2_D03_B	GREEN	No	70	147	R-68806	*Burkholderia*	*cenocepacia*	*IIIA*
Z0010_LB_A01_2_D06_A	GREEN	No	70	147	R-68806	*Burkholderia*	*cenocepacia*	*IIIA*
Z0010_RB_A06_2_D10_B	GREEN	No	70	147	R-68806	*Burkholderia*	*cenocepacia*	*IIIA*
Z0010_LB_A02_2_D06_B	GREEN	No	70	147	R-68806	*Burkholderia*	*cenocepacia*	*IIIA*
Z0011_RB_D07_1_C09_A	GREEN	No	70	145	R-67581	*Burkholderia*	*cenocepacia*	*IIIB*
Z0011_RB_C11_2_C07_A	GREEN	No	70	145	R-67581	*Burkholderia*	*cenocepacia*	*IIIB*
Z0011_RB_D09_1_C08_A	GREEN	No	70	145	R-67581	*Burkholderia*	*cenocepacia*	*IIIB*
Z0011_LB_F06_1_B04_B	GREEN	No	70	145	R-67581	*Burkholderia*	*cenocepacia*	*IIIB*
Z0011_LB_F05_1_B04_A	GREEN	No	70	145	R-67581	*Burkholderia*	*cenocepacia*	*IIIB*
Z0011_LB_E07_2_B03_A	GREEN	No	70	145	R-67581	*Burkholderia*	*cenocepacia*	*IIIB*
Z0011_LB_F10_1_B02_B	GREEN	No	70	145	R-67581	*Burkholderia*	*cenocepacia*	*IIIB*
Z0011_RB_D01_1_C12_A	GREEN	No	70	145	R-67581	*Burkholderia*	*cenocepacia*	*IIIB*
Z0011_LB_F04_1_B05_B	GREEN	No	70	145	R-67581	*Burkholderia*	*cenocepacia*	*IIIB*
Z0011_RB_C07_2_C09_A	GREEN	No	70	145	R-67581	*Burkholderia*	*cenocepacia*	*IIIB*
Z0011_LB_E09_2_B02_A	GREEN	No	70	145	R-67581	*Burkholderia*	*cenocepacia*	*IIIB*
Z0011_LB_F09_1_B02_A	GREEN	No	70	145	R-67581	*Burkholderia*	*cenocepacia*	*IIIB*
Z0011_LB_E11_2_B01_A	GREEN	No	70	145	R-67581	*Burkholderia*	*cenocepacia*	*IIIB*
Z0011_LB_F08_1_B03_B	GREEN	No	70	145	R-67581	*Burkholderia*	*cenocepacia*	*IIIB*
Z0011_LB_E10_2_B02_B	GREEN	No	70	145	R-67581	*Burkholderia*	*cenocepacia*	*IIIB*
Z0011_RB_D08_1_C09_B	GREEN	No	70	145	R-67581	*Burkholderia*	*cenocepacia*	*IIIB*
Z0011_RB_D05_1_C10_A	GREEN	No	70	145	R-67581	*Burkholderia*	*cenocepacia*	*IIIB*

Z0011_RB_D06_1_C10_B	GREEN	No	70	145	R-67581	*Burkholderia*	*cenocepacia*	*IIIB*
Z0011_RB_D03_1_C11_A	GREEN	No	70	145	R-67581	*Burkholderia*	*cenocepacia*	*IIIB*
Z0011_RB_C12_2_C07_B	GREEN	No	70	145	R-67581	*Burkholderia*	*cenocepacia*	*IIIB*
Z0011_RB_C09_2_C08_A	GREEN	No	70	145	R-67581	*Burkholderia*	*cenocepacia*	*IIIB*
Z0011_LB_E12_2_B01_B	GREEN	No	70	145	R-67581	*Burkholderia*	*cenocepacia*	*IIIB*
Z0011_RB_D10_1_C08_B	GREEN	No	70	145	R-67581	*Burkholderia*	*cenocepacia*	*IIIB*
Z0011_LB_F07_1_B03_A	GREEN	No	70	145	R-67581	*Burkholderia*	*cenocepacia*	*IIIB*
Z0011_RB_C10_2_C08_B	GREEN	No	70	145	R-67581	*Burkholderia*	*cenocepacia*	*IIIB*
Z0011_RB_D02_1_C12_B	GREEN	No	70	145	R-67581	*Burkholderia*	*cenocepacia*	*IIIB*
Z0011_RB_C08_2_C09_B	GREEN	No	70	145	R-67581	*Burkholderia*	*cenocepacia*	*IIIB*
Z0010_RB_G09_2_A08_A	GREEN	No	70	147	R-71085	*Burkholderia*	*cenocepacia*	*IIIA*
Z0010_RB_H04_1_A11_B	GREEN	No	70	147	R-71085	*Burkholderia*	*cenocepacia*	*IIIA*
Z0010_RB_G10_2_A08_B	GREEN	No	70	147	R-71085	*Burkholderia*	*cenocepacia*	*IIIA*
Z0010_LO_A01_1_E01_A	GREEN	No	70	147	R-71085	*Burkholderia*	*cenocepacia*	*IIIA*
Z0010_LB_H02_1_A06_B	GREEN	No	70	147	R-71085	*Burkholderia*	*cenocepacia*	*IIIA*
Z0010_RB_G03_2_A11_A	GREEN	No	70	147	R-71085	*Burkholderia*	*cenocepacia*	*IIIA*
Z0011_RB_G09_2_A08_A	GREEN	No	70	147	R-68675	*Burkholderia*	*cenocepacia*	*IIIA*
Z0011_LO_A06_1_E03_B	GREEN	No	70	147	R-68675	*Burkholderia*	*cenocepacia*	*IIIA*
Z0011_RB_H01_1_A12_A	GREEN	No	70	147	R-68675	*Burkholderia*	*cenocepacia*	*IIIA*
Z0011_LO_A09_1_E05_A	GREEN	No	70	147	R-68675	*Burkholderia*	*cenocepacia*	*IIIA*
Z0011_RB_G12_2_A07_B	GREEN	No	70	147	R-68675	*Burkholderia*	*cenocepacia*	*IIIA*
Z0011_LO_A12_1_E06_B	GREEN	No	70	147	R-68675	*Burkholderia*	*cenocepacia*	*IIIA*
Z0011_LO_B05_2_E03_A	GREEN	No	70	147	R-68675	*Burkholderia*	*cenocepacia*	*IIIA*
Z0011_LO_A08_1_E04_B	GREEN	No	70	147	R-68675	*Burkholderia*	*cenocepacia*	*IIIA*
Z0011_LO_A11_1_E06_A	GREEN	No	70	147	R-68675	*Burkholderia*	*cenocepacia*	*IIIA*
Z0011_RB_H03_1_A11_A	GREEN	No	70	147	R-68675	*Burkholderia*	*cenocepacia*	*IIIA*
Z0011_RB_H08_1_A09_B	GREEN	No	70	147	R-68675	*Burkholderia*	*cenocepacia*	*IIIA*
Z0011_LO_B04_2_E02_B	GREEN	No	70	147	R-68675	*Burkholderia*	*cenocepacia*	*IIIA*
Z0011_LO_B06_2_E03_B	GREEN	No	70	147	R-68675	*Burkholderia*	*cenocepacia*	*IIIA*
Z0011_LO_A10_1_E05_B	GREEN	No	70	147	R-68675	*Burkholderia*	*cenocepacia*	*IIIA*
Z0010_RB_E06_2_B10_B	GREEN	No	70	147	R-71051	*Burkholderia*	*cenocepacia*	*IIIA*
Z0011_LB_B10_1_D02_B	GREEN	No	70	145	R-67259	*Burkholderia*	*cenocepacia*	*IIIB*
Z0011_LB_C05_2_C04_A	GREEN	No	70	145	R-67259	*Burkholderia*	*cenocepacia*	*IIIB*
Z0011_RB_A10_2_D08_B	GREEN	No	70	145	R-67259	*Burkholderia*	*cenocepacia*	*IIIB*
Z0011_LB_C08_2_C03_B	GREEN	No	70	145	R-67259	*Burkholderia*	*cenocepacia*	*IIIB*
Z0011_LB_C06_2_C04_B	GREEN	No	70	145	R-67259	*Burkholderia*	*cenocepacia*	*IIIB*
Z0011_RB_A04_2_D11_B	GREEN	No	70	145	R-67259	*Burkholderia*	*cenocepacia*	*IIIB*
Z0011_LB_C03_2_C05_A	GREEN	No	70	145	R-67259	*Burkholderia*	*cenocepacia*	*IIIB*
Z0011_LB_C07_2_C03_A	GREEN	No	70	145	R-67259	*Burkholderia*	*cenocepacia*	*IIIB*
Z0011_LB_B08_1_D03_B	GREEN	No	70	145	R-67259	*Burkholderia*	*cenocepacia*	*IIIB*
Z0011_RB_B03_1_D11_A	GREEN	No	70	145	R-67259	*Burkholderia*	*cenocepacia*	*IIIB*
Z0011_LB_B11_1_D01_A	GREEN	No	70	145	R-67259	*Burkholderia*	*cenocepacia*	*IIIB*
Z0011_RB_A11_2_D07_A	GREEN	No	70	145	R-67259	*Burkholderia*	*cenocepacia*	*IIIB*
Z0011_RB_A05_2_D10_A	GREEN	No	70	145	R-67259	*Burkholderia*	*cenocepacia*	*IIIB*
Z0011_RB_A12_2_D07_B	GREEN	No	70	145	R-67259	*Burkholderia*	*cenocepacia*	*IIIB*
Z0011_LB_C10_2_C02_B	GREEN	No	70	145	R-67259	*Burkholderia*	*cenocepacia*	*IIIB*
Z0011_LB_B06_1_D04_B	GREEN	No	70	145	R-67259	*Burkholderia*	*cenocepacia*	*IIIB*
Z0011_LB_B09_1_D02_A	GREEN	No	70	145	R-67259	*Burkholderia*	*cenocepacia*	*IIIB*
Z0011_LB_B07_1_D03_A	GREEN	No	70	145	R-67259	*Burkholderia*	*cenocepacia*	*IIIB*
Z0011_RB_A06_2_D10_B	GREEN	No	70	145	R-67259	*Burkholderia*	*cenocepacia*	*IIIB*
Z0011_RB_B04_1_D11_B	GREEN	No	70	145	R-67259	*Burkholderia*	*cenocepacia*	*IIIB*
Z0011_RB_B02_1_D12_B	GREEN	No	70	145	R-67259	*Burkholderia*	*cenocepacia*	*IIIB*
Z0011_LB_C09_2_C02_A	GREEN	No	70	145	R-67259	*Burkholderia*	*cenocepacia*	*IIIB*
Z0011_RB_B01_1_D12_A	GREEN	No	70	145	R-67259	*Burkholderia*	*cenocepacia*	*IIIB*
Z0011_LB_C12_2_C01_B	GREEN	No	70	145	R-67259	*Burkholderia*	*cenocepacia*	*IIIB*
Z0011_LB_C11_2_C01_A	GREEN	No	70	145	R-67259	*Burkholderia*	*cenocepacia*	*IIIB*
Z0011_LB_B12_1_D01_B	GREEN	No	70	145	R-67259	*Burkholderia*	*cenocepacia*	*IIIB*
Z0011_RB_A08_2_D09_B	GREEN	No	70	145	R-67259	*Burkholderia*	*cenocepacia*	*IIIB*
Z0011_LB_C02_2_C06_B	GREEN	No	70	145	R-67259	*Burkholderia*	*cenocepacia*	*IIIB*
Z0011_RB_A07_2_D09_A	GREEN	No	70	145	R-67259	*Burkholderia*	*cenocepacia*	*IIIB*
Z0011_LB_C01_2_C06_A	GREEN	No	70	145	R-67259	*Burkholderia*	*cenocepacia*	*IIIB*
Z0011_LB_H12_1_A01_B	GREEN	No	70	146	R-68599	*Burkholderia*	*cenocepacia*	*IIIB*
Z0011_RB_F06_1_B10_B	GREEN	No	70	146	R-68599	*Burkholderia*	*cenocepacia*	*IIIB*
Z0011_RB_G01_2_A12_A	GREEN	No	70	146	R-68599	*Burkholderia*	*cenocepacia*	*IIIB*
Z0011_LB_H10_1_A02_B	GREEN	No	70	146	R-68599	*Burkholderia*	*cenocepacia*	*IIIB*
Z0011_LB_H01_1_A06_A	GREEN	No	70	146	R-68599	*Burkholderia*	*cenocepacia*	*IIIB*
Z0011_RB_F05_1_B10_A	GREEN	No	70	146	R-68599	*Burkholderia*	*cenocepacia*	*IIIB*
Z0011_RB_F07_1_B09_A	GREEN	No	70	146	R-68599	*Burkholderia*	*cenocepacia*	*IIIB*
Z0011_LB_H05_1_A04_A	GREEN	No	70	146	R-68599	*Burkholderia*	*cenocepacia*	*IIIB*
Z0011_LO_A02_1_E01_B	GREEN	No	70	146	R-68599	*Burkholderia*	*cenocepacia*	*IIIB*

Z0011_LO_A04_1_E02_B	GREEN	No	70	146	R-68599	*Burkholderia*	*cenocepacia*	*IIIB*
Z0011_LB_H07_1_A03_A	GREEN	No	70	146	R-68599	*Burkholderia*	*cenocepacia*	*IIIB*
Z0011_RB_F10_1_B08_B	GREEN	No	70	146	R-68599	*Burkholderia*	*cenocepacia*	*IIIB*
Z0011_RB_F09_1_B08_A	GREEN	No	70	146	R-68599	*Burkholderia*	*cenocepacia*	*IIIB*
Z0011_LB_H04_1_A05_B	GREEN	No	70	146	R-68599	*Burkholderia*	*cenocepacia*	*IIIB*
Z0011_RB_F12_1_B07_B	GREEN	No	70	146	R-68599	*Burkholderia*	*cenocepacia*	*IIIB*
Z0011_LB_H02_1_A06_B	GREEN	No	70	146	R-68599	*Burkholderia*	*cenocepacia*	*IIIB*
Z0011_RB_F08_1_B09_B	GREEN	No	70	146	R-68599	*Burkholderia*	*cenocepacia*	*IIIB*
Z0011_RB_G04_2_A11_B	GREEN	No	70	146	R-68599	*Burkholderia*	*cenocepacia*	*IIIB*
Z0011_LB_H06_1_A04_B	GREEN	No	70	146	R-68599	*Burkholderia*	*cenocepacia*	*IIIB*
Z0011_LB_H03_1_A05_A	GREEN	No	70	146	R-68599	*Burkholderia*	*cenocepacia*	*IIIB*
Z0011_RB_G03_2_A11_A	GREEN	No	70	146	R-68599	*Burkholderia*	*cenocepacia*	*IIIB*
Z0011_RB_F11_1_B07_A	GREEN	No	70	146	R-68599	*Burkholderia*	*cenocepacia*	*IIIB*
Z0011_RB_G02_2_A12_B	GREEN	No	70	146	R-68599	*Burkholderia*	*cenocepacia*	*IIIB*
Z0011_RB_G06_2_A10_B	GREEN	No	70	146	R-68599	*Burkholderia*	*cenocepacia*	*IIIB*
Z0011_LO_A01_1_E01_A	GREEN	No	70	146	R-68599	*Burkholderia*	*cenocepacia*	*IIIB*
Z0011_LB_H08_1_A03_B	GREEN	No	70	146	R-68599	*Burkholderia*	*cenocepacia*	*IIIB*
Z0011_LB_H11_1_A01_A	GREEN	No	70	146	R-68599	*Burkholderia*	*cenocepacia*	*IIIB*
Z0011_RB_G05_2_A10_A	GREEN	No	70	146	R-68599	*Burkholderia*	*cenocepacia*	*IIIB*
Z0011_LB_G07_2_A03_A	GREEN	No	70	146	R-68591	*Burkholderia*	*cenocepacia*	*IIIB*
Z0011_RB_F04_1_B11_B	GREEN	No	70	146	R-68591	*Burkholderia*	*cenocepacia*	*IIIB*
Z0011_LB_G08_2_A03_B	GREEN	No	70	146	R-68591	*Burkholderia*	*cenocepacia*	*IIIB*
Z0011_RB_E05_2_B10_A	GREEN	No	70	146	R-68591	*Burkholderia*	*cenocepacia*	*IIIB*
Z0011_RB_E04_2_B11_B	GREEN	No	70	146	R-68591	*Burkholderia*	*cenocepacia*	*IIIB*
Z0011_RB_E07_2_B09_A	GREEN	No	70	146	R-68591	*Burkholderia*	*cenocepacia*	*IIIB*
Z0011_LB_G03_2_A05_A	GREEN	No	70	146	R-68591	*Burkholderia*	*cenocepacia*	*IIIB*
Z0011_RB_E06_2_B10_B	GREEN	No	70	146	R-68591	*Burkholderia*	*cenocepacia*	*IIIB*
Z0011_RB_E12_2_B07_B	GREEN	No	70	146	R-68591	*Burkholderia*	*cenocepacia*	*IIIB*
Z0011_RB_E11_2_B07_A	GREEN	No	70	146	R-68591	*Burkholderia*	*cenocepacia*	*IIIB*
Z0011_LB_G01_2_A06_A	GREEN	No	70	146	R-68591	*Burkholderia*	*cenocepacia*	*IIIB*
Z0011_RB_F02_1_B12_B	GREEN	No	70	146	R-68591	*Burkholderia*	*cenocepacia*	*IIIB*
Z0011_LB_G11_2_A01_A	GREEN	No	70	146	R-68591	*Burkholderia*	*cenocepacia*	*IIIB*
Z0011_RB_E09_2_B08_A	GREEN	No	70	146	R-68591	*Burkholderia*	*cenocepacia*	*IIIB*
Z0011_LB_F11_1_B01_A	GREEN	No	70	146	R-68591	*Burkholderia*	*cenocepacia*	*IIIB*
Z0011_LB_G09_2_A02_A	GREEN	No	70	146	R-68591	*Burkholderia*	*cenocepacia*	*IIIB*
Z0011_LB_F12_1_B01_B	GREEN	No	70	146	R-68591	*Burkholderia*	*cenocepacia*	*IIIB*
Z0011_RB_F01_1_B12_A	GREEN	No	70	146	R-68591	*Burkholderia*	*cenocepacia*	*IIIB*
Z0011_RB_E03_2_B11_A	GREEN	No	70	146	R-68591	*Burkholderia*	*cenocepacia*	*IIIB*
Z0011_LB_G04_2_A05_B	GREEN	No	70	146	R-68591	*Burkholderia*	*cenocepacia*	*IIIB*
Z0011_RB_E08_2_B09_B	GREEN	No	70	146	R-68591	*Burkholderia*	*cenocepacia*	*IIIB*
Z0011_RB_D11_1_C07_A	GREEN	No	70	146	R-68591	*Burkholderia*	*cenocepacia*	*IIIB*
Z0011_LB_G05_2_A04_A	GREEN	No	70	146	R-68591	*Burkholderia*	*cenocepacia*	*IIIB*
Z0011_RB_E02_2_B12_B	GREEN	No	70	146	R-68591	*Burkholderia*	*cenocepacia*	*IIIB*
Z0011_LB_G02_2_A06_B	GREEN	No	70	146	R-68591	*Burkholderia*	*cenocepacia*	*IIIB*
Z0011_RB_E10_2_B08_B	GREEN	No	70	146	R-68591	*Burkholderia*	*cenocepacia*	*IIIB*
Z0011_RB_F03_1_B11_A	GREEN	No	70	146	R-68591	*Burkholderia*	*cenocepacia*	*IIIB*
Z0011_LB_G06_2_A04_B	GREEN	No	70	146	R-68591	*Burkholderia*	*cenocepacia*	*IIIB*
Z0011_RB_D12_1_C07_B	GREEN	No	70	146	R-68591	*Burkholderia*	*cenocepacia*	*IIIB*
Z0011_RB_E01_2_B12_A	GREEN	No	70	146	R-68591	*Burkholderia*	*cenocepacia*	*IIIB*
Z0011_LB_G12_2_A01_B	GREEN	No	70	146	R-68591	*Burkholderia*	*cenocepacia*	*IIIB*
Z0011_RB_H05_1_A10_A	GREEN	Yes	71	147	R-68675	*Burkholderia*	*cenocepacia*	*IIIA*
Z0011_RB_G11_2_A07_A	GREEN	No	71	147	R-68675	*Burkholderia*	*cenocepacia*	*IIIA*
Z0011_LO_B02_2_E01_B	GREEN	No	71	147	R-68675	*Burkholderia*	*cenocepacia*	*IIIA*
Z0011_LO_B01_2_E01_A	GREEN	No	71	147	R-68675	*Burkholderia*	*cenocepacia*	*IIIA*
Z0010_LB_H01_1_A06_A	GREEN	Yes	72	147	R-71085	*Burkholderia*	*cenocepacia*	*IIIA*
Z0010_RO_C06_1_F09_B	GREEN	No	72	147	R-68806	*Burkholderia*	*cenocepacia*	*IIIA*
Z0010_RO_C10_1_F11_B	GREEN	No	72	147	R-68806	*Burkholderia*	*cenocepacia*	*IIIA*
Z0010_RB_A07_2_D09_A	GREEN	No	72	147	R-68806	*Burkholderia*	*cenocepacia*	*IIIA*
Z0011_RB_H10_1_A08_B	GREEN	No	72	147	R-68675	*Burkholderia*	*cenocepacia*	*IIIA*
Z0011_RB_H07_1_A09_A	GREEN	No	72	147	R-68675	*Burkholderia*	*cenocepacia*	*IIIA*
Z0011_RB_H04_1_A11_B	GREEN	No	72	147	R-68675	*Burkholderia*	*cenocepacia*	*IIIA*
Z0011_RB_H09_1_A08_A	GREEN	No	72	147	R-68675	*Burkholderia*	*cenocepacia*	*IIIA*
Z0011_RB_G10_2_A08_B	GREEN	No	72	147	R-68675	*Burkholderia*	*cenocepacia*	*IIIA*
Z0010_RB_G04_2_A11_B	GREEN	No	72	147	R-71085	*Burkholderia*	*cenocepacia*	*IIIA*
Z0010_RB_F12_1_B07_B	GREEN	No	72	147	R-71085	*Burkholderia*	*cenocepacia*	*IIIA*
Z0010_RB_G02_2_A12_B	GREEN	No	72	147	R-71085	*Burkholderia*	*cenocepacia*	*IIIA*
Z0010_RB_D11_1_C07_A	GREEN	Yes	73	147	R-71051	*Burkholderia*	*cenocepacia*	*IIIA*
Z0010_LB_A12_2_D01_B	GREEN	No	73	147	R-68806	*Burkholderia*	*cenocepacia*	*IIIA*
Z0010_RB_A11_2_D07_A	GREEN	No	73	147	R-68806	*Burkholderia*	*cenocepacia*	*IIIA*
Z0010_LB_B01_1_D06_A	GREEN	No	73	147	R-68806	*Burkholderia*	*cenocepacia*	*IIIA*

Z0010_LO_D06_2_F03_B	GREEN	No	73	147	R-68806	*Burkholderia*	*cenocepacia*	*IIIA*
Z0010_RB_A10_2_D08_B	GREEN	No	73	147	R-68806	*Burkholderia*	*cenocepacia*	*IIIA*
Z0010_RB_A04_2_D11_B	GREEN	No	73	147	R-68806	*Burkholderia*	*cenocepacia*	*IIIA*
Z0010_RB_A03_2_D11_A	GREEN	No	73	147	R-68806	*Burkholderia*	*cenocepacia*	*IIIA*
Z0010_RB_A12_2_D07_B	GREEN	No	73	147	R-68806	*Burkholderia*	*cenocepacia*	*IIIA*
Z0010_LB_A10_2_D02_B	GREEN	No	73	147	R-68806	*Burkholderia*	*cenocepacia*	*IIIA*
Z0010_RB_A08_2_D09_B	GREEN	No	73	147	R-68806	*Burkholderia*	*cenocepacia*	*IIIA*
Z0010_LB_A09_2_D02_A	GREEN	No	73	147	R-68806	*Burkholderia*	*cenocepacia*	*IIIA*
Z0010_LB_A11_2_D01_A	GREEN	No	73	147	R-68806	*Burkholderia*	*cenocepacia*	*IIIA*
Z0010_RO_C12_1_F12_B	GREEN	No	73	147	R-68806	*Burkholderia*	*cenocepacia*	*IIIA*
Z0010_RB_A05_2_D10_A	GREEN	No	73	147	R-68806	*Burkholderia*	*cenocepacia*	*IIIA*
Z0010_RB_A09_2_D08_A	GREEN	No	73	147	R-68806	*Burkholderia*	*cenocepacia*	*IIIA*
Z0010_LO_D01_2_F01_A	GREEN	No	73	147	R-68806	*Burkholderia*	*cenocepacia*	*IIIA*
Z0010_LB_A06_2_D04_B	GREEN	No	73	147	R-68806	*Burkholderia*	*cenocepacia*	*IIIA*
Z0010_LB_A03_2_D05_A	GREEN	No	73	147	R-68806	*Burkholderia*	*cenocepacia*	*IIIA*
Z0010_LB_A04_2_D05_B	GREEN	No	73	147	R-68806	*Burkholderia*	*cenocepacia*	*IIIA*
Z0010_RB_G11_2_A07_A	GREEN	No	73	147	R-71085	*Burkholderia*	*cenocepacia*	*IIIA*
Z0010_LB_H10_1_A02_B	GREEN	No	73	147	R-71085	*Burkholderia*	*cenocepacia*	*IIIA*
Z0010_LB_H03_1_A05_A	GREEN	No	73	147	R-71085	*Burkholderia*	*cenocepacia*	*IIIA*
Z0010_LB_G12_2_A01_B	GREEN	No	73	147	R-71085	*Burkholderia*	*cenocepacia*	*IIIA*
Z0010_RB_H02_1_A12_B	GREEN	No	73	147	R-71085	*Burkholderia*	*cenocepacia*	*IIIA*
Z0010_LB_H12_1_A01_B	GREEN	No	73	147	R-71085	*Burkholderia*	*cenocepacia*	*IIIA*
Z0010_RB_G05_2_A10_A	GREEN	No	73	147	R-71085	*Burkholderia*	*cenocepacia*	*IIIA*
Z0010_LB_H07_1_A03_A	GREEN	No	73	147	R-71085	*Burkholderia*	*cenocepacia*	*IIIA*
Z0010_RB_F11_1_B07_A	GREEN	No	73	147	R-71085	*Burkholderia*	*cenocepacia*	*IIIA*
Z0010_LB_H11_1_A01_A	GREEN	No	73	147	R-71085	*Burkholderia*	*cenocepacia*	*IIIA*
Z0010_LB_H09_1_A02_A	GREEN	No	73	147	R-71085	*Burkholderia*	*cenocepacia*	*IIIA*
Z0010_RB_G01_2_A12_A	GREEN	No	73	147	R-71085	*Burkholderia*	*cenocepacia*	*IIIA*
Z0010_RB_G07_2_A09_A	GREEN	No	73	147	R-71085	*Burkholderia*	*cenocepacia*	*IIIA*
Z0010_RB_G12_2_A07_B	GREEN	No	73	147	R-71085	*Burkholderia*	*cenocepacia*	*IIIA*
Z0011_RB_H02_1_A12_B	GREEN	No	73	147	R-68675	*Burkholderia*	*cenocepacia*	*IIIA*
Z0010_RB_E03_2_B11_A	GREEN	No	73	147	R-71051	*Burkholderia*	*cenocepacia*	*IIIA*
Z0010_LB_E11_2_B01_A	GREEN	No	73	147	R-71051	*Burkholderia*	*cenocepacia*	*IIIA*
Z0010_LB_E10_2_B02_B	GREEN	No	73	147	R-71051	*Burkholderia*	*cenocepacia*	*IIIA*
Z0010_LB_F02_1_B06_B	GREEN	No	73	147	R-71051	*Burkholderia*	*cenocepacia*	*IIIA*
Z0010_RB_E04_2_B11_B	GREEN	No	73	147	R-71051	*Burkholderia*	*cenocepacia*	*IIIA*
Z0010_RB_E01_2_B12_A	GREEN	No	73	147	R-71051	*Burkholderia*	*cenocepacia*	*IIIA*
Z0010_RB_D10_1_C08_B	GREEN	No	73	147	R-71051	*Burkholderia*	*cenocepacia*	*IIIA*
Z0010_RB_D04_1_C11_B	GREEN	No	73	147	R-71051	*Burkholderia*	*cenocepacia*	*IIIA*
Z0010_LB_F01_1_B06_A	GREEN	No	73	147	R-71051	*Burkholderia*	*cenocepacia*	*IIIA*
Z0010_RB_D09_1_C08_A	GREEN	No	73	147	R-71051	*Burkholderia*	*cenocepacia*	*IIIA*
Z0010_RB_D07_1_C09_A	GREEN	No	73	147	R-71051	*Burkholderia*	*cenocepacia*	*IIIA*
Z0010_RB_D08_1_C09_B	GREEN	No	73	147	R-71051	*Burkholderia*	*cenocepacia*	*IIIA*
Z0010_RB_D12_1_C07_B	GREEN	No	73	147	R-71051	*Burkholderia*	*cenocepacia*	*IIIA*
Z0010_RB_D06_1_C10_B	GREEN	No	73	147	R-71051	*Burkholderia*	*cenocepacia*	*IIIA*
Z0010_RB_E05_2_B10_A	GREEN	No	73	147	R-71051	*Burkholderia*	*cenocepacia*	*IIIA*
Z0010_LB_E12_2_B01_B	GREEN	No	73	147	R-71051	*Burkholderia*	*cenocepacia*	*IIIA*
Z0010_LB_E07_2_B03_A	GREEN	No	73	147	R-71051	*Burkholderia*	*cenocepacia*	*IIIA*
Z0010_LB_E09_2_B02_A	GREEN	No	73	147	R-71051	*Burkholderia*	*cenocepacia*	*IIIA*
Z0010_LB_E05_2_B04_A	GREEN	No	73	147	R-71051	*Burkholderia*	*cenocepacia*	*IIIA*
Z0010_LB_F05_1_B04_A	GREEN	No	73	147	R-71051	*Burkholderia*	*cenocepacia*	*IIIA*
Z0010_LB_F06_1_B04_B	GREEN	No	73	147	R-71051	*Burkholderia*	*cenocepacia*	*IIIA*
Z0010_RB_E07_2_B09_A	GREEN	No	73	147	R-71051	*Burkholderia*	*cenocepacia*	*IIIA*
Z0010_LB_E08_2_B03_B	GREEN	No	73	147	R-71051	*Burkholderia*	*cenocepacia*	*IIIA*
Z0010_RB_E02_2_B12_B	GREEN	No	73	147	R-71051	*Burkholderia*	*cenocepacia*	*IIIA*
Z0010_LB_F03_1_B05_A	GREEN	No	73	147	R-71051	*Burkholderia*	*cenocepacia*	*IIIA*
Z0010_LB_E06_2_B04_B	GREEN	No	73	147	R-71051	*Burkholderia*	*cenocepacia*	*IIIA*
Z0010_LB_F04_1_B05_B	GREEN	No	73	147	R-71051	*Burkholderia*	*cenocepacia*	*IIIA*
Z0010_RB_D05_1_C10_A	GREEN	No	73	147	R-71051	*Burkholderia*	*cenocepacia*	*IIIA*
Z0010_LB_E04_2_B05_B	GREEN	No	73	147	R-71051	*Burkholderia*	*cenocepacia*	*IIIA*
Z0010_LB_E03_2_B05_A	GREEN	No	73	147	R-71051	*Burkholderia*	*cenocepacia*	*IIIA*
Z0011_RB_A09_2_D08_A	GREEN	Yes	74	145	R-67259	*Burkholderia*	*cenocepacia*	*IIIB*
Z0011_LB_F02_1_B06_B	GREEN	No	74	145	R-67581	*Burkholderia*	*cenocepacia*	*IIIB*
Z0011_LB_F01_1_B06_A	GREEN	No	74	145	R-67581	*Burkholderia*	*cenocepacia*	*IIIB*
Z0011_LB_F03_1_B05_A	GREEN	No	74	145	R-67581	*Burkholderia*	*cenocepacia*	*IIIB*
Z0011_RB_D04_1_C11_B	GREEN	No	74	145	R-67581	*Burkholderia*	*cenocepacia*	*IIIB*
Z0011_LB_E08_2_B03_B	GREEN	No	74	145	R-67581	*Burkholderia*	*cenocepacia*	*IIIB*
Z0010_LB_H08_1_A03_B	GREEN	Yes	75	147	R-71085	*Burkholderia*	*cenocepacia*	*IIIA*
Z0010_RB_H01_1_A12_A	GREEN	No	75	147	R-71085	*Burkholderia*	*cenocepacia*	*IIIA*
Z0010_LB_H06_1_A04_B	GREEN	No	75	147	R-71085	*Burkholderia*	*cenocepacia*	*IIIA*

Z0010_RB_G06_2_A10_B	GREEN	No	75	147	R-71085	*Burkholderia*	*cenocepacia*	*IIIA*
Z0011_LB_H09_1_A02_A	GREEN	Yes	76	146	R-68599	*Burkholderia*	*cenocepacia*	*IIIB*
Z0011_RB_G08_2_A09_B	GREEN	No	76	146	R-68599	*Burkholderia*	*cenocepacia*	*IIIB*
Z0011_RB_G07_2_A09_A	GREEN	No	76	146	R-68599	*Burkholderia*	*cenocepacia*	*IIIB*
Z0025_LB_D07_1_C03_A	GREEN	Yes	77	135	LMG 7881	*Serratia*	*ficaria*	
Z0025_LB_C12_2_C01_B	GREEN	No	77	135	LMG 7881	*Serratia*	*ficaria*	
Z0025_RB_F05_1_B10_A	GREEN	No	77	135	LMG 7881	*Serratia*	*ficaria*	
Z0025_LB_E01_2_B06_A	GREEN	No	77	135	LMG 7881	*Serratia*	*ficaria*	
Z0025_RB_F04_1_B11_B	GREEN	No	77	135	LMG 7881	*Serratia*	*ficaria*	
Z0025_LB_D08_1_C03_B	GREEN	No	77	135	LMG 7881	*Serratia*	*ficaria*	
Z0025_LB_E07_2_B03_A	GREEN	No	77	135	LMG 7881	*Serratia*	*ficaria*	
Z0025_LB_C05_2_C04_A	GREEN	No	77	135	LMG 7881	*Serratia*	*ficaria*	
Z0025_RB_F07_1_B09_A	GREEN	No	77	135	LMG 7881	*Serratia*	*ficaria*	
Z0025_RB_F09_1_B08_A	GREEN	No	77	135	LMG 7881	*Serratia*	*ficaria*	
Z0025_LB_E05_2_B04_A	GREEN	No	77	135	LMG 7881	*Serratia*	*ficaria*	
Z0025_LB_D10_1_C02_B	GREEN	No	77	135	LMG 7881	*Serratia*	*ficaria*	
Z0025_LB_D02_1_C06_B	GREEN	No	77	135	LMG 7881	*Serratia*	*ficaria*	
Z0025_LB_D03_1_C05_A	GREEN	No	77	135	LMG 7881	*Serratia*	*ficaria*	
Z0025_LB_D09_1_C02_A	GREEN	No	77	135	LMG 7881	*Serratia*	*ficaria*	
Z0025_LB_D04_1_C05_B	GREEN	No	77	135	LMG 7881	*Serratia*	*ficaria*	
Z0025_LB_D01_1_C06_A	GREEN	No	77	135	LMG 7881	*Serratia*	*ficaria*	
Z0025_LB_E09_2_B02_A	GREEN	No	77	135	LMG 7881	*Serratia*	*ficaria*	
Z0025_LB_D06_1_C04_B	GREEN	No	77	135	LMG 7881	*Serratia*	*ficaria*	
Z0025_RB_F01_1_B12_A	GREEN	No	77	135	LMG 7881	*Serratia*	*ficaria*	
Z0025_RB_F06_1_B10_B	GREEN	No	77	135	LMG 7881	*Serratia*	*ficaria*	
Z0025_LB_E06_2_B04_B	GREEN	No	77	135	LMG 7881	*Serratia*	*ficaria*	
Z0025_RB_F02_1_B12_B	GREEN	No	77	135	LMG 7881	*Serratia*	*ficaria*	
Z0025_LB_E04_2_B05_B	GREEN	No	77	135	LMG 7881	*Serratia*	*ficaria*	
Z0025_RB_F03_1_B11_A	GREEN	No	77	135	LMG 7881	*Serratia*	*ficaria*	
Z0025_LB_D12_1_C01_B	GREEN	No	77	135	LMG 7881	*Serratia*	*ficaria*	
Z0025_LB_E08_2_B03_B	GREEN	No	77	135	LMG 7881	*Serratia*	*ficaria*	
Z0025_RB_F08_1_B09_B	GREEN	No	77	135	LMG 7881	*Serratia*	*ficaria*	
Z0025_LB_E03_2_B05_A	GREEN	No	77	135	LMG 7881	*Serratia*	*ficaria*	
Z0025_LB_E02_2_B06_B	GREEN	No	77	135	LMG 7881	*Serratia*	*ficaria*	
Z0025_LB_D05_1_C04_A	GREEN	No	77	135	LMG 7881	*Serratia*	*ficaria*	
Z0025_LB_D11_1_C01_A	GREEN	No	77	135	LMG 7881	*Serratia*	*ficaria*	
Z0025_RB_E11_2_B07_A	GREEN	Yes	78	134	LMG 7874	*Morganella*	*morganii*	*morganii*
Z0025_LB_C03_2_C05_A	GREEN	No	78	134	LMG 7874	*Morganella*	*morganii*	*morganii*
Z0025_RB_E12_2_B07_B	GREEN	No	78	134	LMG 7874	*Morganella*	*morganii*	*morganii*
Z0025_LB_B11_1_D01_A	GREEN	No	78	134	LMG 7874	*Morganella*	*morganii*	*morganii*
Z0025_RB_E06_2_B10_B	GREEN	No	78	134	LMG 7874	*Morganella*	*morganii*	*morganii*
Z0025_LB_C06_2_C04_B	GREEN	No	78	134	LMG 7874	*Morganella*	*morganii*	*morganii*
Z0025_RB_D09_1_C08_A	GREEN	No	78	134	LMG 7874	*Morganella*	*morganii*	*morganii*
Z0025_RB_D08_1_C09_B	GREEN	No	78	134	LMG 7874	*Morganella*	*morganii*	*morganii*
Z0025_LB_C04_2_C05_B	GREEN	No	78	134	LMG 7874	*Morganella*	*morganii*	*morganii*
Z0025_LB_B12_1_D01_B	GREEN	No	78	134	LMG 7874	*Morganella*	*morganii*	*morganii*
Z0025_LB_C10_2_C02_B	GREEN	No	78	134	LMG 7874	*Morganella*	*morganii*	*morganii*
Z0025_RB_E02_2_B12_B	GREEN	No	78	134	LMG 7874	*Morganella*	*morganii*	*morganii*
Z0025_RB_E05_2_B10_A	GREEN	No	78	134	LMG 7874	*Morganella*	*morganii*	*morganii*
Z0025_RB_E09_2_B08_A	GREEN	No	78	134	LMG 7874	*Morganella*	*morganii*	*morganii*
Z0025_RB_E07_2_B09_A	GREEN	No	78	134	LMG 7874	*Morganella*	*morganii*	*morganii*
Z0025_LB_B09_1_D02_A	GREEN	No	78	134	LMG 7874	*Morganella*	*morganii*	*morganii*
Z0025_RB_E08_2_B09_B	GREEN	No	78	134	LMG 7874	*Morganella*	*morganii*	*morganii*
Z0025_LB_C02_2_C06_B	GREEN	No	78	134	LMG 7874	*Morganella*	*morganii*	*morganii*
Z0025_LB_B10_1_D02_B	GREEN	No	78	134	LMG 7874	*Morganella*	*morganii*	*morganii*
Z0025_LB_B08_1_D03_B	GREEN	No	78	134	LMG 7874	*Morganella*	*morganii*	*morganii*
Z0025_LB_C07_2_C03_A	GREEN	No	78	134	LMG 7874	*Morganella*	*morganii*	*morganii*
Z0025_LB_C08_2_C03_B	GREEN	No	78	134	LMG 7874	*Morganella*	*morganii*	*morganii*
Z0025_LB_C01_2_C06_A	GREEN	No	78	134	LMG 7874	*Morganella*	*morganii*	*morganii*
Z0025_RB_E04_2_B11_B	GREEN	No	78	134	LMG 7874	*Morganella*	*morganii*	*morganii*
Z0025_RB_D10_1_C08_B	GREEN	No	78	134	LMG 7874	*Morganella*	*morganii*	*morganii*
Z0025_LB_C11_2_C01_A	GREEN	No	78	134	LMG 7874	*Morganella*	*morganii*	*morganii*
Z0025_RB_E10_2_B08_B	GREEN	No	78	134	LMG 7874	*Morganella*	*morganii*	*morganii*
Z0025_RB_E01_2_B12_A	GREEN	No	78	134	LMG 7874	*Morganella*	*morganii*	*morganii*
Z0025_RB_D11_1_C07_A	GREEN	No	78	134	LMG 7874	*Morganella*	*morganii*	*morganii*
Z0025_LB_C09_2_C02_A	GREEN	No	78	134	LMG 7874	*Morganella*	*morganii*	*morganii*
Z0025_RB_E03_2_B11_A	GREEN	Yes	79	134	LMG 7874	*Morganella*	*morganii*	*morganii*
Z0025_RB_D12_1_C07_B	GREEN	No	79	134	LMG 7874	*Morganella*	*morganii*	*morganii*
Z0024_RO_A12_1_E12_B	GREEN	Yes	80	128	LMG 6909	*Leuconostoc*	*mesenteroides*	*cremoris*
Z0024_LO_B06_2_E03_B	GREEN	No	80	128	LMG 6909	*Leuconostoc*	*mesenteroides*	*cremoris*

Z0024_LO_B05_2_E03_A	GREEN	No	80	128	LMG 6909	*Leuconostoc*	*mesenteroides*	*cremoris*
Z0024_LO_B10_2_E05_B	GREEN	No	80	128	LMG 6909	*Leuconostoc*	*mesenteroides*	*cremoris*
Z0024_RO_A05_1_E09_A	GREEN	No	80	128	LMG 6909	*Leuconostoc*	*mesenteroides*	*cremoris*
Z0024_RB_H11_1_A07_A	GREEN	No	80	128	LMG 6909	*Leuconostoc*	*mesenteroides*	*cremoris*
Z0024_RO_A09_1_E11_A	GREEN	No	80	128	LMG 6909	*Leuconostoc*	*mesenteroides*	*cremoris*
Z0024_RO_A11_1_E12_A	GREEN	No	80	128	LMG 6909	*Leuconostoc*	*mesenteroides*	*cremoris*
Z0024_RO_A03_1_E08_A	GREEN	No	80	128	LMG 6909	*Leuconostoc*	*mesenteroides*	*cremoris*
Z0024_RO_A01_1_E07_A	GREEN	No	80	128	LMG 6909	*Leuconostoc*	*mesenteroides*	*cremoris*
Z0024_RO_A08_1_E10_B	GREEN	No	80	128	LMG 6909	*Leuconostoc*	*mesenteroides*	*cremoris*
Z0024_LO_C05_1_F03_A	GREEN	No	80	128	LMG 6909	*Leuconostoc*	*mesenteroides*	*cremoris*
Z0024_RO_A06_1_E09_B	GREEN	No	80	128	LMG 6909	*Leuconostoc*	*mesenteroides*	*cremoris*
Z0024_LO_B03_2_E02_A	GREEN	No	80	128	LMG 6909	*Leuconostoc*	*mesenteroides*	*cremoris*
Z0024_LO_C04_1_F02_B	GREEN	No	80	128	LMG 6909	*Leuconostoc*	*mesenteroides*	*cremoris*
Z0024_LO_A12_1_E06_B	GREEN	No	80	128	LMG 6909	*Leuconostoc*	*mesenteroides*	*cremoris*
Z0024_LO_B09_2_E05_A	GREEN	No	80	128	LMG 6909	*Leuconostoc*	*mesenteroides*	*cremoris*
Z0024_RO_A07_1_E10_A	GREEN	No	80	128	LMG 6909	*Leuconostoc*	*mesenteroides*	*cremoris*
Z0024_LO_C06_1_F03_B	GREEN	No	80	128	LMG 6909	*Leuconostoc*	*mesenteroides*	*cremoris*
Z0024_RO_A04_1_E08_B	GREEN	No	80	128	LMG 6909	*Leuconostoc*	*mesenteroides*	*cremoris*
Z0024_RO_A02_1_E07_B	GREEN	No	80	128	LMG 6909	*Leuconostoc*	*mesenteroides*	*cremoris*
Z0024_LO_B12_2_E06_B	GREEN	No	80	128	LMG 6909	*Leuconostoc*	*mesenteroides*	*cremoris*
Z0024_LO_B11_2_E06_A	GREEN	Yes	81	128	LMG 6909	*Leuconostoc*	*mesenteroides*	*cremoris*
Z0024_LO_B08_2_E04_B	GREEN	No	81	128	LMG 6909	*Leuconostoc*	*mesenteroides*	*cremoris*
Z0024_RB_H12_1_A07_B	GREEN	No	81	128	LMG 6909	*Leuconostoc*	*mesenteroides*	*cremoris*
Z0024_LO_B07_2_E04_A	GREEN	No	81	128	LMG 6909	*Leuconostoc*	*mesenteroides*	*cremoris*
Z0024_RB_H10_1_A08_B	GREEN	No	81	128	LMG 6909	*Leuconostoc*	*mesenteroides*	*cremoris*
Z0024_RB_H09_1_A08_A	GREEN	No	81	128	LMG 6909	*Leuconostoc*	*mesenteroides*	*cremoris*
Z0020_LO_H03_2_H02_A	GREEN	Yes	82	89	LMG 26195	*Paracoccus*	*sp.*	
Z0020_LB_A03_2_D05_A	GREEN	No	82	89	LMG 26195	*Paracoccus*	*sp.*	
Z0020_LO_G11_1_H06_A	GREEN	No	82	89	LMG 26195	*Paracoccus*	*sp.*	
Z0020_RB_H08_1_A09_B	GREEN	No	82	89	LMG 26195	*Paracoccus*	*sp.*	
Z0020_LB_A09_2_D02_A	GREEN	No	82	89	LMG 26195	*Paracoccus*	*sp.*	
Z0020_LB_A01_2_D06_A	GREEN	No	82	89	LMG 26195	*Paracoccus*	*sp.*	
Z0020_LB_A07_2_D03_A	GREEN	No	82	89	LMG 26195	*Paracoccus*	*sp.*	
Z0020_LO_G10_1_H05_B	GREEN	No	82	89	LMG 26195	*Paracoccus*	*sp.*	
Z0020_RO_B05_2_E09_A	GREEN	No	82	89	LMG 26195	*Paracoccus*	*sp.*	
Z0020_RB_H04_1_A11_B	GREEN	No	82	89	LMG 26195	*Paracoccus*	*sp.*	
Z0020_LO_H05_2_H03_A	GREEN	No	82	89	LMG 26195	*Paracoccus*	*sp.*	
Z0020_RO_A01_1_E07_A	GREEN	No	82	89	LMG 26195	*Paracoccus*	*sp.*	
Z0020_LO_H08_2_H04_B	GREEN	No	82	89	LMG 26195	*Paracoccus*	*sp.*	
Z0020_LO_H02_2_H01_B	GREEN	No	82	89	LMG 26195	*Paracoccus*	*sp.*	
Z0020_LO_H01_2_H01_A	GREEN	No	82	89	LMG 26195	*Paracoccus*	*sp.*	
Z0020_LO_B09_2_E05_A	GREEN	No	82	89	LMG 26195	*Paracoccus*	*sp.*	
Z0020_LB_A02_2_D06_B	GREEN	No	82	89	LMG 26195	*Paracoccus*	*sp.*	
Z0020_LB_A04_2_D05_B	GREEN	No	82	89	LMG 26195	*Paracoccus*	*sp.*	
Z0020_LB_A08_2_D03_B	GREEN	No	82	89	LMG 26195	*Paracoccus*	*sp.*	
Z0020_LO_H09_2_H05_A	GREEN	No	82	89	LMG 26195	*Paracoccus*	*sp.*	
Z0020_LO_G12_1_H06_B	GREEN	No	82	89	LMG 26195	*Paracoccus*	*sp.*	
Z0020_LO_H04_2_H02_B	GREEN	No	82	89	LMG 26195	*Paracoccus*	*sp.*	
Z0020_LB_A05_2_D04_A	GREEN	No	82	89	LMG 26195	*Paracoccus*	*sp.*	
Z0020_LB_A10_2_D02_B	GREEN	No	82	89	LMG 26195	*Paracoccus*	*sp.*	
Z0020_RO_B07_2_E10_A	GREEN	No	82	89	LMG 26195	*Paracoccus*	*sp.*	
Z0020_LO_H07_2_H04_A	GREEN	No	82	89	LMG 26195	*Paracoccus*	*sp.*	
Z0020_LB_A06_2_D04_B	GREEN	No	82	89	LMG 26195	*Paracoccus*	*sp.*	
Z0020_LO_H06_2_H03_B	GREEN	No	82	89	LMG 26195	*Paracoccus*	*sp.*	
Z0020_LB_A11_2_D01_A	GREEN	No	82	89	LMG 26195	*Paracoccus*	*sp.*	
Z0020_LB_A12_2_D01_B	GREEN	No	82	89	LMG 26195	*Paracoccus*	*sp.*	
Z0020_RO_A06_1_E09_B	GREEN	No	82	89	LMG 26195	*Paracoccus*	*sp.*	
Z0020_RO_A04_1_E08_B	GREEN	No	82	89	LMG 26195	*Paracoccus*	*sp.*	
Z0022_RO_E06_1_G09_B	GREEN	Yes	83	111	LMG 3897	*Listonella*	*pelagia*	
Z0022_LO_D09_2_F05_A	GREEN	No	83	111	LMG 3897	*Listonella*	*pelagia*	
Z0022_RO_C10_1_F11_B	GREEN	No	83	111	LMG 3897	*Listonella*	*pelagia*	
Z0022_RO_D02_2_F07_B	GREEN	No	83	111	LMG 3897	*Listonella*	*pelagia*	
Z0022_RO_D08_2_F10_B	GREEN	No	83	111	LMG 3897	*Listonella*	*pelagia*	
Z0022_RO_D05_2_F09_A	GREEN	No	83	111	LMG 3897	*Listonella*	*pelagia*	
Z0022_LO_E03_1_G02_A	GREEN	No	83	111	LMG 3897	*Listonella*	*pelagia*	
Z0022_LO_E07_1_G04_A	GREEN	No	83	111	LMG 3897	*Listonella*	*pelagia*	
Z0022_LO_D12_2_F06_B	GREEN	No	83	111	LMG 3897	*Listonella*	*pelagia*	
Z0022_RO_E05_1_G09_A	GREEN	No	83	111	LMG 3897	*Listonella*	*pelagia*	
Z0022_RO_E07_1_G10_A	GREEN	No	83	111	LMG 3897	*Listonella*	*pelagia*	
Z0022_LO_G01_1_H01_A	GREEN	No	83	111	LMG 3897	*Listonella*	*pelagia*	

Z0022_RO_E01_1_G07_A	GREEN	No	83	111	LMG 3897	*Listonella*	*pelagia*
Z0022_RO_C12_1_F12_B	GREEN	No	83	111	LMG 3897	*Listonella*	*pelagia*
Z0022_LO_E06_1_G03_B	GREEN	No	83	111	LMG 3897	*Listonella*	*pelagia*
Z0022_RO_D12_2_F12_B	GREEN	No	83	111	LMG 3897	*Listonella*	*pelagia*
Z0022_RO_D06_2_F09_B	GREEN	No	83	111	LMG 3897	*Listonella*	*pelagia*
Z0022_RO_E02_1_G07_B	GREEN	No	83	111	LMG 3897	*Listonella*	*pelagia*
Z0022_RO_E03_1_G08_A	GREEN	No	83	111	LMG 3897	*Listonella*	*pelagia*
Z0022_LO_E12_1_G06_B	GREEN	No	83	111	LMG 3897	*Listonella*	*pelagia*
Z0022_RO_F01_2_G07_A	GREEN	No	83	111	LMG 3897	*Listonella*	*pelagia*
Z0022_LO_G02_1_H01_B	GREEN	No	83	111	LMG 3897	*Listonella*	*pelagia*
Z0022_RO_C09_1_F11_A	GREEN	No	83	111	LMG 3897	*Listonella*	*pelagia*
Z0022_LO_F12_2_G06_B	GREEN	No	83	111	LMG 3897	*Listonella*	*pelagia*
Z0022_RO_C11_1_F12_A	GREEN	No	83	111	LMG 3897	*Listonella*	*pelagia*
Z0022_RO_E10_1_G11_B	GREEN	No	83	111	LMG 3897	*Listonella*	*pelagia*
Z0022_LO_F10_2_G05_B	GREEN	No	83	111	LMG 3897	*Listonella*	*pelagia*
Z0022_RO_D09_2_F11_A	GREEN	No	83	111	LMG 3897	*Listonella*	*pelagia*
Z0022_RO_E08_1_G10_B	GREEN	No	83	111	LMG 3897	*Listonella*	*pelagia*
Z0022_RO_E11_1_G12_A	GREEN	No	83	111	LMG 3897	*Listonella*	*pelagia*
Z0022_RO_D03_2_F08_A	GREEN	No	83	111	LMG 3897	*Listonella*	*pelagia*
Z0022_RO_E09_1_G11_A	GREEN	No	83	111	LMG 3897	*Listonella*	*pelagia*
Z0021_RB_H09_1_A08_A	GREEN	Yes	84	106	LMG 28633	*Rhodococcus*	*degradans*
Z0021_LO_D09_2_F05_A	GREEN	No	84	106	LMG 28633	*Rhodococcus*	*degradans*
Z0021_RO_A02_1_E07_B	GREEN	No	84	106	LMG 28633	*Rhodococcus*	*degradans*
Z0021_RB_H07_1_A09_A	GREEN	No	84	106	LMG 28633	*Rhodococcus*	*degradans*
Z0021_LO_C11_1_F06_A	GREEN	No	84	106	LMG 28633	*Rhodococcus*	*degradans*
Z0021_LO_D10_2_F05_B	GREEN	No	84	106	LMG 28633	*Rhodococcus*	*degradans*
Z0021_LO_D01_2_F01_A	GREEN	No	84	106	LMG 28633	*Rhodococcus*	*degradans*
Z0021_LO_C10_1_F05_B	GREEN	No	84	106	LMG 28633	*Rhodococcus*	*degradans*
Z0021_RO_B08_2_E10_B	GREEN	No	84	106	LMG 28633	*Rhodococcus*	*degradans*
Z0021_LO_E03_1_G02_A	GREEN	No	84	106	LMG 28633	*Rhodococcus*	*degradans*
Z0021_RO_C11_1_F12_A	GREEN	No	84	106	LMG 28633	*Rhodococcus*	*degradans*
Z0021_RO_D04_2_F08_B	GREEN	No	84	106	LMG 28633	*Rhodococcus*	*degradans*
Z0021_RB_H06_1_A10_B	GREEN	No	84	106	LMG 28633	*Rhodococcus*	*degradans*
Z0021_LO_E07_1_G04_A	GREEN	No	84	106	LMG 28633	*Rhodococcus*	*degradans*
Z0021_LO_D06_2_F03_B	GREEN	No	84	106	LMG 28633	*Rhodococcus*	*degradans*
Z0021_LO_F06_2_G03_B	GREEN	No	84	106	LMG 28633	*Rhodococcus*	*degradans*
Z0021_LO_D12_2_F06_B	GREEN	No	84	106	LMG 28633	*Rhodococcus*	*degradans*
Z0021_LO_C07_1_F04_A	GREEN	No	84	106	LMG 28633	*Rhodococcus*	*degradans*
Z0021_LO_E08_1_G04_B	GREEN	No	84	106	LMG 28633	*Rhodococcus*	*degradans*
Z0021_RO_A09_1_E11_A	GREEN	No	84	106	LMG 28633	*Rhodococcus*	*degradans*
Z0021_LO_C09_1_F05_A	GREEN	No	84	106	LMG 28633	*Rhodococcus*	*degradans*
Z0021_LO_C02_1_F01_B	GREEN	No	84	106	LMG 28633	*Rhodococcus*	*degradans*
Z0021_RO_C09_1_F11_A	GREEN	No	84	106	LMG 28633	*Rhodococcus*	*degradans*
Z0021_LO_D05_2_F03_A	GREEN	No	84	106	LMG 28633	*Rhodococcus*	*degradans*
Z0021_LO_C06_1_F03_B	GREEN	No	84	106	LMG 28633	*Rhodococcus*	*degradans*
Z0021_LO_B07_2_E04_A	GREEN	No	84	106	LMG 28633	*Rhodococcus*	*degradans*
Z0021_RO_D01_2_F07_A	GREEN	No	84	106	LMG 28633	*Rhodococcus*	*degradans*
Z0021_LO_D08_2_F04_B	GREEN	No	84	106	LMG 28633	*Rhodococcus*	*degradans*
Z0021_RO_C12_1_F12_B	GREEN	No	84	106	LMG 28633	*Rhodococcus*	*degradans*
Z0021_RO_A11_1_E12_A	GREEN	No	84	106	LMG 28633	*Rhodococcus*	*degradans*
Z0021_LO_B11_2_E06_A	GREEN	No	84	106	LMG 28633	*Rhodococcus*	*degradans*
Z0021_RO_A10_1_E11_B	GREEN	No	84	106	LMG 28633	*Rhodococcus*	*degradans*
Z0018_LB_F04_1_B05_B	GREEN	Yes	85	71	LMG 24424	*Shewanella*	*vesiculosa*
Z0018_LB_G04_2_A05_B	GREEN	No	85	71	LMG 24424	*Shewanella*	*vesiculosa*
Z0018_LB_G06_2_A04_B	GREEN	No	85	71	LMG 24424	*Shewanella*	*vesiculosa*
Z0018_RB_E08_2_B09_B	GREEN	No	85	71	LMG 24424	*Shewanella*	*vesiculosa*
Z0018_RB_E07_2_B09_A	GREEN	No	85	71	LMG 24424	*Shewanella*	*vesiculosa*
Z0018_LB_G01_2_A06_A	GREEN	No	85	71	LMG 24424	*Shewanella*	*vesiculosa*
Z0018_RB_E10_2_B08_B	GREEN	No	85	71	LMG 24424	*Shewanella*	*vesiculosa*
Z0018_RB_F08_1_B09_B	GREEN	No	85	71	LMG 24424	*Shewanella*	*vesiculosa*
Z0018_RB_F06_1_B10_B	GREEN	No	85	71	LMG 24424	*Shewanella*	*vesiculosa*
Z0018_LB_G02_2_A06_B	GREEN	No	85	71	LMG 24424	*Shewanella*	*vesiculosa*
Z0018_RB_E04_2_B11_B	GREEN	No	85	71	LMG 24424	*Shewanella*	*vesiculosa*
Z0018_LB_F12_1_B01_B	GREEN	No	85	71	LMG 24424	*Shewanella*	*vesiculosa*
Z0018_RB_E11_2_B07_A	GREEN	No	85	71	LMG 24424	*Shewanella*	*vesiculosa*
Z0018_RB_E12_2_B07_B	GREEN	No	85	71	LMG 24424	*Shewanella*	*vesiculosa*
Z0018_LB_F05_1_B04_A	GREEN	No	85	71	LMG 24424	*Shewanella*	*vesiculosa*
Z0018_LB_F07_1_B03_A	GREEN	No	85	71	LMG 24424	*Shewanella*	*vesiculosa*
Z0018_RB_E06_2_B10_B	GREEN	No	85	71	LMG 24424	*Shewanella*	*vesiculosa*
Z0018_LB_G03_2_A05_A	GREEN	No	85	71	LMG 24424	*Shewanella*	*vesiculosa*

Z0018_RB_F07_1_B09_A	GREEN	No	85	71	LMG 24424	*Shewanella*	*vesiculosa*	
Z0018_LB_G05_2_A04_A	GREEN	No	85	71	LMG 24424	*Shewanella*	*vesiculosa*	
Z0018_LB_F06_1_B04_B	GREEN	No	85	71	LMG 24424	*Shewanella*	*vesiculosa*	
Z0018_RB_E09_2_B08_A	GREEN	No	85	71	LMG 24424	*Shewanella*	*vesiculosa*	
Z0018_RB_F05_1_B10_A	GREEN	No	85	71	LMG 24424	*Shewanella*	*vesiculosa*	
Z0018_LB_F11_1_B01_A	GREEN	No	85	71	LMG 24424	*Shewanella*	*vesiculosa*	
Z0018_RB_F02_1_B12_B	GREEN	No	85	71	LMG 24424	*Shewanella*	*vesiculosa*	
Z0018_RB_E05_2_B10_A	GREEN	No	85	71	LMG 24424	*Shewanella*	*vesiculosa*	
Z0018_LB_F08_1_B03_B	GREEN	No	85	71	LMG 24424	*Shewanella*	*vesiculosa*	
Z0018_LB_F09_1_B02_A	GREEN	No	85	71	LMG 24424	*Shewanella*	*vesiculosa*	
Z0018_RB_F03_1_B11_A	GREEN	No	85	71	LMG 24424	*Shewanella*	*vesiculosa*	
Z0018_RB_F01_1_B12_A	GREEN	No	85	71	LMG 24424	*Shewanella*	*vesiculosa*	
Z0018_LB_F10_1_B02_B	GREEN	No	85	71	LMG 24424	*Shewanella*	*vesiculosa*	
Z0018_RB_F04_1_B11_B	GREEN	No	85	71	LMG 24424	*Shewanella*	*vesiculosa*	
Z0016_LO_G10_1_H05_B	GREEN	Yes	86	53	LMG 23078	*Streptomyces*	*albus*	*albus*
Z0016_LO_G09_1_H05_A	GREEN	No	86	53	LMG 23078	*Streptomyces*	*albus*	*albus*
Z0016_RO_H01_2_H07_A	GREEN	No	86	53	LMG 23078	*Streptomyces*	*albus*	*albus*
Z0016_RO_G08_1_H10_B	GREEN	No	86	53	LMG 23078	*Streptomyces*	*albus*	*albus*
Z0016_RO_H03_2_H08_A	GREEN	No	86	53	LMG 23078	*Streptomyces*	*albus*	*albus*
Z0016_RO_G09_1_H11_A	GREEN	No	86	53	LMG 23078	*Streptomyces*	*albus*	*albus*
Z0016_RO_H04_2_H08_B	GREEN	No	86	53	LMG 23078	*Streptomyces*	*albus*	*albus*
Z0016_RO_F11_2_G12_A	GREEN	No	86	53	LMG 23078	*Streptomyces*	*albus*	*albus*
Z0016_RO_G03_1_H08_A	GREEN	No	86	53	LMG 23078	*Streptomyces*	*albus*	*albus*
Z0016_LO_G07_1_H04_A	GREEN	No	86	53	LMG 23078	*Streptomyces*	*albus*	*albus*
Z0016_LO_G11_1_H06_A	GREEN	No	86	53	LMG 23078	*Streptomyces*	*albus*	*albus*
Z0016_LO_H03_2_H02_A	GREEN	No	86	53	LMG 23078	*Streptomyces*	*albus*	*albus*
Z0016_LO_H02_2_H01_B	GREEN	No	86	53	LMG 23078	*Streptomyces*	*albus*	*albus*
Z0016_RO_G07_1_H10_A	GREEN	No	86	53	LMG 23078	*Streptomyces*	*albus*	*albus*
Z0016_LO_G12_1_H06_B	GREEN	No	86	53	LMG 23078	*Streptomyces*	*albus*	*albus*
Z0016_RO_G10_1_H11_B	GREEN	No	86	53	LMG 23078	*Streptomyces*	*albus*	*albus*
Z0016_RO_G06_1_H09_B	GREEN	No	86	53	LMG 23078	*Streptomyces*	*albus*	*albus*
Z0016_RO_G11_1_H12_A	GREEN	No	86	53	LMG 23078	*Streptomyces*	*albus*	*albus*
Z0016_LO_G08_1_H04_B	GREEN	No	86	53	LMG 23078	*Streptomyces*	*albus*	*albus*
Z0016_RO_F08_2_G10_B	GREEN	No	86	53	LMG 23078	*Streptomyces*	*albus*	*albus*
Z0016_RO_F12_2_G12_B	GREEN	No	86	53	LMG 23078	*Streptomyces*	*albus*	*albus*
Z0016_LO_H05_2_H03_A	GREEN	No	86	53	LMG 23078	*Streptomyces*	*albus*	*albus*
Z0016_RO_H02_2_H07_B	GREEN	No	86	53	LMG 23078	*Streptomyces*	*albus*	*albus*
Z0016_RO_F05_2_G09_A	GREEN	No	86	53	LMG 23078	*Streptomyces*	*albus*	*albus*
Z0016_RO_F06_2_G09_B	GREEN	No	86	53	LMG 23078	*Streptomyces*	*albus*	*albus*
Z0016_LO_G06_1_H03_B	GREEN	No	86	53	LMG 23078	*Streptomyces*	*albus*	*albus*
Z0016_RO_G12_1_H12_B	GREEN	No	86	53	LMG 23078	*Streptomyces*	*albus*	*albus*
Z0016_LO_H01_2_H01_A	GREEN	No	86	53	LMG 23078	*Streptomyces*	*albus*	*albus*
Z0016_LO_G05_1_H03_A	GREEN	No	86	53	LMG 23078	*Streptomyces*	*albus*	*albus*
Z0016_LO_G03_1_H02_A	GREEN	No	86	53	LMG 23078	*Streptomyces*	*albus*	*albus*
Z0016_LO_H04_2_H02_B	GREEN	No	86	53	LMG 23078	*Streptomyces*	*albus*	*albus*
Z0016_RO_G01_1_H07_A	GREEN	No	86	53	LMG 23078	*Streptomyces*	*albus*	*albus*
Z0018_RO_G02_1_H07_B	GREEN	Yes	87	75	LMG 24812	*Candidimonas*	*nitroreducens*	
Z0018_LO_H03_2_H02_A	GREEN	No	87	75	LMG 24812	*Candidimonas*	*nitroreducens*	
Z0018_LO_H02_2_H01_B	GREEN	No	87	75	LMG 24812	*Candidimonas*	*nitroreducens*	
Z0018_RO_G08_1_H10_B	GREEN	No	87	75	LMG 24812	*Candidimonas*	*nitroreducens*	
Z0018_LO_H04_2_H02_B	GREEN	No	87	75	LMG 24812	*Candidimonas*	*nitroreducens*	
Z0018_RO_H02_2_H07_B	GREEN	No	87	75	LMG 24812	*Candidimonas*	*nitroreducens*	
Z0018_RO_H01_2_H07_A	GREEN	No	87	75	LMG 24812	*Candidimonas*	*nitroreducens*	
Z0018_LO_H05_2_H03_A	GREEN	No	87	75	LMG 24812	*Candidimonas*	*nitroreducens*	
Z0018_RO_G04_1_H08_B	GREEN	No	87	75	LMG 24812	*Candidimonas*	*nitroreducens*	
Z0018_LO_G09_1_H05_A	GREEN	No	87	75	LMG 24812	*Candidimonas*	*nitroreducens*	
Z0018_RO_G10_1_H11_B	GREEN	No	87	75	LMG 24812	*Candidimonas*	*nitroreducens*	
Z0018_LO_G12_1_H06_B	GREEN	No	87	75	LMG 24812	*Candidimonas*	*nitroreducens*	
Z0018_RO_G07_1_H10_A	GREEN	No	87	75	LMG 24812	*Candidimonas*	*nitroreducens*	
Z0018_LO_G11_1_H06_A	GREEN	No	87	75	LMG 24812	*Candidimonas*	*nitroreducens*	
Z0018_RO_H04_2_H08_B	GREEN	No	87	75	LMG 24812	*Candidimonas*	*nitroreducens*	
Z0018_RO_H03_2_H08_A	GREEN	No	87	75	LMG 24812	*Candidimonas*	*nitroreducens*	
Z0018_RO_G11_1_H12_A	GREEN	No	87	75	LMG 24812	*Candidimonas*	*nitroreducens*	
Z0018_LO_G07_1_H04_A	GREEN	No	87	75	LMG 24812	*Candidimonas*	*nitroreducens*	
Z0018_RO_G03_1_H08_A	GREEN	No	87	75	LMG 24812	*Candidimonas*	*nitroreducens*	
Z0018_LO_H01_2_H01_A	GREEN	No	87	75	LMG 24812	*Candidimonas*	*nitroreducens*	
Z0018_RO_G06_1_H09_B	GREEN	No	87	75	LMG 24812	*Candidimonas*	*nitroreducens*	
Z0018_LO_H06_2_H03_B	GREEN	No	87	75	LMG 24812	*Candidimonas*	*nitroreducens*	
Z0018_LO_G04_1_H02_B	GREEN	No	87	75	LMG 24812	*Candidimonas*	*nitroreducens*	
Z0018_RO_F09_2_G11_A	GREEN	No	87	75	LMG 24812	*Candidimonas*	*nitroreducens*	

Z0018_RO_F11_2_G12_A	GREEN	No	87	75	LMG 24812	*Candidimonas*	*nitroreducens*
Z0018_RO_F10_2_G11_B	GREEN	No	87	75	LMG 24812	*Candidimonas*	*nitroreducens*
Z0018_RO_G09_1_H11_A	GREEN	No	87	75	LMG 24812	*Candidimonas*	*nitroreducens*
Z0018_RO_G12_1_H12_B	GREEN	No	87	75	LMG 24812	*Candidimonas*	*nitroreducens*
Z0018_RO_G05_1_H09_A	GREEN	No	87	75	LMG 24812	*Candidimonas*	*nitroreducens*
Z0018_LO_G08_1_H04_B	GREEN	Yes	88	75	LMG 24812	*Candidimonas*	*nitroreducens*
Z0018_RO_G01_1_H07_A	GREEN	No	88	75	LMG 24812	*Candidimonas*	*nitroreducens*
Z0018_RO_F12_2_G12_B	GREEN	No	88	75	LMG 24812	*Candidimonas*	*nitroreducens*
Z0012_RO_G05_1_H09_A	GREEN	Yes	89	9	LMG 12537	*Rhizorhapis*	*suberifaciens*
Z0012_LO_H01_2_H01_A	GREEN	No	89	9	LMG 12537	*Rhizorhapis*	*suberifaciens*
Z0012_RO_G04_1_H08_B	GREEN	No	89	9	LMG 12537	*Rhizorhapis*	*suberifaciens*
Z0012_LO_H02_2_H01_B	GREEN	No	89	9	LMG 12537	*Rhizorhapis*	*suberifaciens*
Z0012_RO_F05_2_G09_A	GREEN	No	89	9	LMG 12537	*Rhizorhapis*	*suberifaciens*
Z0012_RO_E08_1_G10_B	GREEN	No	89	9	LMG 12537	*Rhizorhapis*	*suberifaciens*
Z0012_RO_F11_2_G12_A	GREEN	No	89	9	LMG 12537	*Rhizorhapis*	*suberifaciens*
Z0012_LO_F11_2_G06_A	GREEN	No	89	9	LMG 12537	*Rhizorhapis*	*suberifaciens*
Z0012_RO_F10_2_G11_B	GREEN	No	89	9	LMG 12537	*Rhizorhapis*	*suberifaciens*
Z0012_RO_E09_1_G11_A	GREEN	No	89	9	LMG 12537	*Rhizorhapis*	*suberifaciens*
Z0012_LO_H06_2_H03_B	GREEN	No	89	9	LMG 12537	*Rhizorhapis*	*suberifaciens*
Z0012_RO_G06_1_H09_B	GREEN	No	89	9	LMG 12537	*Rhizorhapis*	*suberifaciens*
Z0012_RO_F09_2_G11_A	GREEN	No	89	9	LMG 12537	*Rhizorhapis*	*suberifaciens*
Z0012_RO_F03_2_G08_A	GREEN	No	89	9	LMG 12537	*Rhizorhapis*	*suberifaciens*
Z0012_RO_F04_2_G08_B	GREEN	No	89	9	LMG 12537	*Rhizorhapis*	*suberifaciens*
Z0012_RO_E10_1_G11_B	GREEN	No	89	9	LMG 12537	*Rhizorhapis*	*suberifaciens*
Z0012_LO_H07_2_H04_A	GREEN	No	89	9	LMG 12537	*Rhizorhapis*	*suberifaciens*
Z0012_LO_G09_1_H05_A	GREEN	No	89	9	LMG 12537	*Rhizorhapis*	*suberifaciens*
Z0012_LO_F09_2_G05_A	GREEN	No	89	9	LMG 12537	*Rhizorhapis*	*suberifaciens*
Z0012_RO_G03_1_H08_A	GREEN	No	89	9	LMG 12537	*Rhizorhapis*	*suberifaciens*
Z0012_LO_G08_1_H04_B	GREEN	No	89	9	LMG 12537	*Rhizorhapis*	*suberifaciens*
Z0012_RO_F08_2_G10_B	GREEN	No	89	9	LMG 12537	*Rhizorhapis*	*suberifaciens*
Z0012_LO_G04_1_H02_B	GREEN	No	89	9	LMG 12537	*Rhizorhapis*	*suberifaciens*
Z0012_LO_F08_2_G04_B	GREEN	No	89	9	LMG 12537	*Rhizorhapis*	*suberifaciens*
Z0012_LO_G02_1_H01_B	GREEN	No	89	9	LMG 12537	*Rhizorhapis*	*suberifaciens*
Z0012_LO_F10_2_G05_B	GREEN	No	89	9	LMG 12537	*Rhizorhapis*	*suberifaciens*
Z0012_RO_E07_1_G10_A	GREEN	No	89	9	LMG 12537	*Rhizorhapis*	*suberifaciens*
Z0012_RO_F01_2_G07_A	GREEN	No	89	9	LMG 12537	*Rhizorhapis*	*suberifaciens*
Z0021_LB_E04_2_B05_B	GREEN	Yes	90	100	LMG 27282	*Noviherbaspirillum*	*psychrotolerans*
Z0021_LB_E03_2_B05_A	GREEN	No	90	100	LMG 27282	*Noviherbaspirillum*	*psychrotolerans*
Z0021_LB_D12_1_C01_B	GREEN	No	90	100	LMG 27282	*Noviherbaspirillum*	*psychrotolerans*
Z0021_RB_D12_1_C07_B	GREEN	No	90	100	LMG 27282	*Noviherbaspirillum*	*psychrotolerans*
Z0021_LB_E09_2_B02_A	GREEN	No	90	100	LMG 27282	*Noviherbaspirillum*	*psychrotolerans*
Z0021_RB_D07_1_C09_A	GREEN	No	90	100	LMG 27282	*Noviherbaspirillum*	*psychrotolerans*
Z0021_RB_D09_1_C08_A	GREEN	No	90	100	LMG 27282	*Noviherbaspirillum*	*psychrotolerans*
Z0021_RB_D11_1_C07_A	GREEN	No	90	100	LMG 27282	*Noviherbaspirillum*	*psychrotolerans*
Z0021_RB_E02_2_B12_B	GREEN	No	90	100	LMG 27282	*Noviherbaspirillum*	*psychrotolerans*
Z0021_LB_D07_1_C03_A	GREEN	No	90	100	LMG 27282	*Noviherbaspirillum*	*psychrotolerans*
Z0021_LB_D08_1_C03_B	GREEN	No	90	100	LMG 27282	*Noviherbaspirillum*	*psychrotolerans*
Z0021_LB_E07_2_B03_A	GREEN	No	90	100	LMG 27282	*Noviherbaspirillum*	*psychrotolerans*
Z0021_LB_E10_2_B02_B	GREEN	No	90	100	LMG 27282	*Noviherbaspirillum*	*psychrotolerans*
Z0021_RB_E04_2_B11_B	GREEN	No	90	100	LMG 27282	*Noviherbaspirillum*	*psychrotolerans*
Z0021_RB_E05_2_B10_A	GREEN	No	90	100	LMG 27282	*Noviherbaspirillum*	*psychrotolerans*
Z0021_RB_D08_1_C09_B	GREEN	No	90	100	LMG 27282	*Noviherbaspirillum*	*psychrotolerans*
Z0021_LB_E08_2_B03_B	GREEN	No	90	100	LMG 27282	*Noviherbaspirillum*	*psychrotolerans*
Z0021_LB_E02_2_B06_B	GREEN	No	90	100	LMG 27282	*Noviherbaspirillum*	*psychrotolerans*
Z0021_RB_D06_1_C10_B	GREEN	No	90	100	LMG 27282	*Noviherbaspirillum*	*psychrotolerans*
Z0021_RB_D03_1_C11_A	GREEN	No	90	100	LMG 27282	*Noviherbaspirillum*	*psychrotolerans*
Z0021_LB_D09_1_C02_B	GREEN	No	90	100	LMG 27282	*Noviherbaspirillum*	*psychrotolerans*
Z0021_LB_D11_1_C01_A	GREEN	No	90	100	LMG 27282	*Noviherbaspirillum*	*psychrotolerans*
Z0021_RB_E03_2_B11_A	GREEN	No	90	100	LMG 27282	*Noviherbaspirillum*	*psychrotolerans*
Z0021_LB_E06_2_B04_B	GREEN	No	90	100	LMG 27282	*Noviherbaspirillum*	*psychrotolerans*
Z0021_RB_E01_2_B12_A	GREEN	No	90	100	LMG 27282	*Noviherbaspirillum*	*psychrotolerans*
Z0021_RB_D05_1_C10_A	GREEN	No	90	100	LMG 27282	*Noviherbaspirillum*	*psychrotolerans*
Z0021_LB_E01_2_B06_A	GREEN	No	90	100	LMG 27282	*Noviherbaspirillum*	*psychrotolerans*
Z0021_RB_D04_1_C11_B	GREEN	No	90	100	LMG 27282	*Noviherbaspirillum*	*psychrotolerans*
Z0021_RB_D10_1_C08_B	GREEN	No	90	100	LMG 27282	*Noviherbaspirillum*	*psychrotolerans*
Z0021_RB_E06_2_B10_B	GREEN	No	90	100	LMG 27282	*Noviherbaspirillum*	*psychrotolerans*
Z0021_LB_D10_1_C02_B	GREEN	No	90	100	LMG 27282	*Noviherbaspirillum*	*psychrotolerans*
Z0021_LB_E05_2_B04_A	GREEN	No	90	100	LMG 27282	*Noviherbaspirillum*	*psychrotolerans*
Z0010_RO_B11_2_E12_A	GREEN	Yes	91	141	R-67113	*Burkholderia*	*stabilis*
Z0010_LO_D07_2_F04_A	GREEN	No	91	141	R-67113	*Burkholderia*	*stabilis*

Z0010_RO_B09_2_E11_A	GREEN	No	91	141	R-67113	*Burkholderia*	*stabilis*
Z0010_LO_C10_1_F05_B	GREEN	No	91	141	R-67113	*Burkholderia*	*stabilis*
Z0010_LO_C08_1_F04_B	GREEN	Yes	92	141	R-67113	*Burkholderia*	*stabilis*
Z0010_RO_D08_2_F10_B	GREEN	No	92	141	R-67113	*Burkholderia*	*stabilis*
Z0010_LO_C09_1_F05_A	GREEN	No	92	141	R-67113	*Burkholderia*	*stabilis*
Z0010_LO_D11_2_F06_A	GREEN	No	92	141	R-67113	*Burkholderia*	*stabilis*
Z0010_LO_C06_1_F03_B	GREEN	No	92	141	R-67113	*Burkholderia*	*stabilis*
Z0010_RO_B10_2_E11_B	GREEN	No	92	141	R-67113	*Burkholderia*	*stabilis*
Z0010_RO_D06_2_F09_B	GREEN	No	92	141	R-67113	*Burkholderia*	*stabilis*
Z0010_RO_C01_1_F07_A	GREEN	No	92	141	R-67113	*Burkholderia*	*stabilis*
Z0010_RO_C04_1_F08_B	GREEN	No	92	141	R-67113	*Burkholderia*	*stabilis*
Z0010_LO_D09_2_F05_A	GREEN	No	92	141	R-67113	*Burkholderia*	*stabilis*
Z0010_RO_C02_1_F07_B	GREEN	No	92	141	R-67113	*Burkholderia*	*stabilis*
Z0010_RO_D01_2_F07_A	GREEN	No	92	141	R-67113	*Burkholderia*	*stabilis*
Z0010_RO_C03_1_F08_A	GREEN	No	92	141	R-67113	*Burkholderia*	*stabilis*
Z0010_RO_D07_2_F10_A	GREEN	No	92	141	R-67113	*Burkholderia*	*stabilis*
Z0010_LO_E06_1_G03_B	GREEN	No	92	141	R-67113	*Burkholderia*	*stabilis*
Z0010_LO_C07_1_F04_A	GREEN	No	92	141	R-67113	*Burkholderia*	*stabilis*
Z0010_RO_C09_1_F11_A	GREEN	No	92	141	R-67113	*Burkholderia*	*stabilis*
Z0010_RO_C11_1_F12_A	GREEN	No	92	141	R-67113	*Burkholderia*	*stabilis*
Z0010_LO_D03_2_F02_A	GREEN	No	92	141	R-67113	*Burkholderia*	*stabilis*
Z0010_LO_D05_2_F03_A	GREEN	No	92	141	R-67113	*Burkholderia*	*stabilis*
Z0010_LO_D12_2_F06_B	GREEN	No	92	141	R-67113	*Burkholderia*	*stabilis*
Z0010_RO_D04_2_F08_B	GREEN	No	92	141	R-67113	*Burkholderia*	*stabilis*
Z0010_LO_C11_1_F06_A	GREEN	No	92	141	R-67113	*Burkholderia*	*stabilis*
Z0010_LO_C12_1_F06_B	GREEN	No	92	141	R-67113	*Burkholderia*	*stabilis*
Z0010_LO_E02_1_G01_B	GREEN	No	92	141	R-67113	*Burkholderia*	*stabilis*
Z0010_RO_C07_1_F10_A	GREEN	No	92	141	R-67113	*Burkholderia*	*stabilis*
Z0010_RO_B12_2_E12_B	GREEN	No	92	141	R-67113	*Burkholderia*	*stabilis*
Z0010_LO_D02_2_F01_B	GREEN	No	92	141	R-67113	*Burkholderia*	*stabilis*
Z0012_RB_H10_1_A08_B	GREEN	Yes	93	13	LMG 13127	*Azospirillum*	*brasilense*
Z0012_RO_A05_1_E09_A	GREEN	No	93	13	LMG 13127	*Azospirillum*	*brasilense*
Z0012_LB_H10_1_A02_B	GREEN	No	93	13	LMG 13127	*Azospirillum*	*brasilense*
Z0012_LB_H08_1_A03_B	GREEN	No	93	13	LMG 13127	*Azospirillum*	*brasilense*
Z0012_RB_H12_1_A07_B	GREEN	No	93	13	LMG 13127	*Azospirillum*	*brasilense*
Z0012_RB_G09_2_A08_A	GREEN	No	93	13	LMG 13127	*Azospirillum*	*brasilense*
Z0012_RB_F11_1_B07_A	GREEN	No	93	13	LMG 13127	*Azospirillum*	*brasilense*
Z0012_RO_A01_1_E07_A	GREEN	No	93	13	LMG 13127	*Azospirillum*	*brasilense*
Z0012_RB_G07_2_A09_A	GREEN	No	93	13	LMG 13127	*Azospirillum*	*brasilense*
Z0012_RO_A02_1_E07_B	GREEN	No	93	13	LMG 13127	*Azospirillum*	*brasilense*
Z0012_RO_A07_1_E10_A	GREEN	No	93	13	LMG 13127	*Azospirillum*	*brasilense*
Z0012_RB_H02_1_A12_B	GREEN	No	93	13	LMG 13127	*Azospirillum*	*brasilense*
Z0012_LB_G10_2_A02_B	GREEN	No	93	13	LMG 13127	*Azospirillum*	*brasilense*
Z0012_LB_G03_2_A05_A	GREEN	No	93	13	LMG 13127	*Azospirillum*	*brasilense*
Z0012_LB_G08_2_A03_B	GREEN	No	93	13	LMG 13127	*Azospirillum*	*brasilense*
Z0012_RB_G08_2_A09_B	GREEN	No	93	13	LMG 13127	*Azospirillum*	*brasilense*
Z0012_LB_G05_2_A04_A	GREEN	No	93	13	LMG 13127	*Azospirillum*	*brasilense*
Z0012_LB_H05_1_A04_A	GREEN	No	93	13	LMG 13127	*Azospirillum*	*brasilense*
Z0012_LB_G02_2_A06_B	GREEN	No	93	13	LMG 13127	*Azospirillum*	*brasilense*
Z0012_RO_A12_1_E12_B	GREEN	No	93	13	LMG 13127	*Azospirillum*	*brasilense*
Z0012_LB_H03_1_A05_A	GREEN	No	93	13	LMG 13127	*Azospirillum*	*brasilense*
Z0012_LB_H12_1_A01_B	GREEN	No	93	13	LMG 13127	*Azospirillum*	*brasilense*
Z0012_RB_G04_2_A11_B	GREEN	No	93	13	LMG 13127	*Azospirillum*	*brasilense*
Z0012_RB_F12_1_B07_B	GREEN	No	93	13	LMG 13127	*Azospirillum*	*brasilense*
Z0012_RB_H08_1_A09_B	GREEN	No	93	13	LMG 13127	*Azospirillum*	*brasilense*
Z0012_RB_G02_2_A12_B	GREEN	No	93	13	LMG 13127	*Azospirillum*	*brasilense*
Z0012_RO_B03_2_E08_A	GREEN	No	93	13	LMG 13127	*Azospirillum*	*brasilense*
Z0012_RB_G12_2_A07_B	GREEN	No	93	13	LMG 13127	*Azospirillum*	*brasilense*
Z0012_RB_F10_1_B08_B	GREEN	No	93	13	LMG 13127	*Azospirillum*	*brasilense*
Z0012_RB_H05_1_A10_A	GREEN	No	93	13	LMG 13127	*Azospirillum*	*brasilense*
Z0012_LB_G04_2_A05_B	GREEN	No	93	13	LMG 13127	*Azospirillum*	*brasilense*
Z0012_RB_G05_2_A10_A	GREEN	No	93	13	LMG 13127	*Azospirillum*	*brasilense*
Z0017_LB_C11_2_C01_A	GREEN	Yes	94	57	LMG 23383	*Lactococcus*	*piscium*
Z0017_RB_B08_1_D09_B	GREEN	No	94	57	LMG 23383	*Lactococcus*	*piscium*
Z0017_LB_C10_2_C02_B	GREEN	No	94	57	LMG 23383	*Lactococcus*	*piscium*
Z0017_RB_C02_2_C12_B	GREEN	No	94	57	LMG 23383	*Lactococcus*	*piscium*
Z0017_LB_D04_1_C05_B	GREEN	No	94	57	LMG 23383	*Lactococcus*	*piscium*
Z0017_LB_C08_2_C03_B	GREEN	No	94	57	LMG 23383	*Lactococcus*	*piscium*
Z0017_LB_C07_2_C03_A	GREEN	No	94	57	LMG 23383	*Lactococcus*	*piscium*
Z0017_LB_D06_1_C04_B	GREEN	No	94	57	LMG 23383	*Lactococcus*	*piscium*

Z0017_RB_B09_1_D08_A	GREEN	No	94	57	LMG 23383	*Lactococcus*	*piscium*
Z0017_RB_C05_2_C10_A	GREEN	No	94	57	LMG 23383	*Lactococcus*	*piscium*
Z0017_RB_C06_2_C10_B	GREEN	No	94	57	LMG 23383	*Lactococcus*	*piscium*
Z0017_RB_C08_2_C09_B	GREEN	No	94	57	LMG 23383	*Lactococcus*	*piscium*
Z0017_RB_B10_1_D08_B	GREEN	No	94	57	LMG 23383	*Lactococcus*	*piscium*
Z0017_RB_B03_1_D11_A	GREEN	No	94	57	LMG 23383	*Lactococcus*	*piscium*
Z0017_LB_D05_1_C04_A	GREEN	No	94	57	LMG 23383	*Lactococcus*	*piscium*
Z0017_RB_B06_1_D10_B	GREEN	No	94	57	LMG 23383	*Lactococcus*	*piscium*
Z0017_RB_C03_2_C11_A	GREEN	No	94	57	LMG 23383	*Lactococcus*	*piscium*
Z0017_LB_C09_2_C02_A	GREEN	No	94	57	LMG 23383	*Lactococcus*	*piscium*
Z0017_LB_D07_1_C03_A	GREEN	No	94	57	LMG 23383	*Lactococcus*	*piscium*
Z0017_RB_B04_1_D11_B	GREEN	No	94	57	LMG 23383	*Lactococcus*	*piscium*
Z0017_RB_B05_1_D10_A	GREEN	No	94	57	LMG 23383	*Lactococcus*	*piscium*
Z0017_RB_C09_2_C08_A	GREEN	No	94	57	LMG 23383	*Lactococcus*	*piscium*
Z0017_RB_C07_2_C09_A	GREEN	No	94	57	LMG 23383	*Lactococcus*	*piscium*
Z0017_LB_D01_1_C06_A	GREEN	No	94	57	LMG 23383	*Lactococcus*	*piscium*
Z0017_LB_C12_2_C01_B	GREEN	No	94	57	LMG 23383	*Lactococcus*	*piscium*
Z0017_LB_D02_1_C06_B	GREEN	No	94	57	LMG 23383	*Lactococcus*	*piscium*
Z0017_RB_C01_2_C12_A	GREEN	No	94	57	LMG 23383	*Lactococcus*	*piscium*
Z0017_RB_B12_1_D07_B	GREEN	No	94	57	LMG 23383	*Lactococcus*	*piscium*
Z0017_RB_B07_1_D09_A	GREEN	No	94	57	LMG 23383	*Lactococcus*	*piscium*
Z0017_RB_C04_2_C11_B	GREEN	No	94	57	LMG 23383	*Lactococcus*	*piscium*
Z0017_RB_B11_1_D07_A	GREEN	No	94	57	LMG 23383	*Lactococcus*	*piscium*
Z0017_LB_D03_1_C05_A	GREEN	No	94	57	LMG 23383	*Lactococcus*	*piscium*
Z0019_RB_H01_1_A12_A	GREEN	Yes	95	82	LMG 25664	*Megasphaera*	*cerevisiae*
Z0019_RB_H02_1_A12_B	GREEN	No	95	82	LMG 25664	*Megasphaera*	*cerevisiae*
Z0019_RB_G12_2_A07_B	GREEN	No	95	82	LMG 25664	*Megasphaera*	*cerevisiae*
Z0019_LB_G09_2_A02_A	GREEN	No	95	82	LMG 25664	*Megasphaera*	*cerevisiae*
Z0019_LB_G11_2_A01_A	GREEN	No	95	82	LMG 25664	*Megasphaera*	*cerevisiae*
Z0019_RB_G04_2_A11_B	GREEN	No	95	82	LMG 25664	*Megasphaera*	*cerevisiae*
Z0019_RB_G09_2_A08_A	GREEN	No	95	82	LMG 25664	*Megasphaera*	*cerevisiae*
Z0019_RB_G05_2_A10_A	GREEN	No	95	82	LMG 25664	*Megasphaera*	*cerevisiae*
Z0019_LB_F11_1_B01_A	GREEN	No	95	82	LMG 25664	*Megasphaera*	*cerevisiae*
Z0019_RB_G10_2_A08_B	GREEN	No	95	82	LMG 25664	*Megasphaera*	*cerevisiae*
Z0019_RB_G02_2_A12_B	GREEN	No	95	82	LMG 25664	*Megasphaera*	*cerevisiae*
Z0019_LB_G04_2_A05_B	GREEN	No	95	82	LMG 25664	*Megasphaera*	*cerevisiae*
Z0019_LB_G05_2_A04_A	GREEN	No	95	82	LMG 25664	*Megasphaera*	*cerevisiae*
Z0019_LB_G10_2_A02_B	GREEN	No	95	82	LMG 25664	*Megasphaera*	*cerevisiae*
Z0019_RB_G11_2_A07_A	GREEN	No	95	82	LMG 25664	*Megasphaera*	*cerevisiae*
Z0019_LB_F09_1_B02_A	GREEN	No	95	82	LMG 25664	*Megasphaera*	*cerevisiae*
Z0019_LB_F10_1_B02_B	GREEN	No	95	82	LMG 25664	*Megasphaera*	*cerevisiae*
Z0019_LB_G08_2_A03_B	GREEN	No	95	82	LMG 25664	*Megasphaera*	*cerevisiae*
Z0019_RB_G08_2_A09_B	GREEN	No	95	82	LMG 25664	*Megasphaera*	*cerevisiae*
Z0019_LB_G12_2_A01_B	GREEN	No	95	82	LMG 25664	*Megasphaera*	*cerevisiae*
Z0019_LB_G03_2_A05_A	GREEN	No	95	82	LMG 25664	*Megasphaera*	*cerevisiae*
Z0019_RB_G07_2_A09_A	GREEN	No	95	82	LMG 25664	*Megasphaera*	*cerevisiae*
Z0019_LB_G07_2_A03_A	GREEN	No	95	82	LMG 25664	*Megasphaera*	*cerevisiae*
Z0019_LB_G01_2_A06_A	GREEN	No	95	82	LMG 25664	*Megasphaera*	*cerevisiae*
Z0019_LB_G06_2_A04_B	GREEN	No	95	82	LMG 25664	*Megasphaera*	*cerevisiae*
Z0019_RB_G06_2_A10_B	GREEN	No	95	82	LMG 25664	*Megasphaera*	*cerevisiae*
Z0019_LB_H02_1_A06_B	GREEN	No	95	82	LMG 25664	*Megasphaera*	*cerevisiae*
Z0019_LB_H01_1_A06_A	GREEN	No	95	82	LMG 25664	*Megasphaera*	*cerevisiae*
Z0019_LB_F12_1_B01_B	GREEN	No	95	82	LMG 25664	*Megasphaera*	*cerevisiae*
Z0019_RB_G03_2_A11_A	GREEN	No	95	82	LMG 25664	*Megasphaera*	*cerevisiae*
Z0019_LB_G02_2_A06_B	GREEN	No	95	82	LMG 25664	*Megasphaera*	*cerevisiae*
Z0019_RB_H03_1_A11_A	GREEN	No	95	82	LMG 25664	*Megasphaera*	*cerevisiae*
Z0013_LB_A09_2_D02_A	GREEN	Yes	96	15	LMG 1345	*Leeuwenhoekiella*	*marinoflava*
Z0013_RO_A03_1_E08_A	GREEN	No	96	15	LMG 1345	*Leeuwenhoekiella*	*marinoflava*
Z0013_RO_A08_1_E10_B	GREEN	No	96	15	LMG 1345	*Leeuwenhoekiella*	*marinoflava*
Z0013_RB_A05_2_D10_A	GREEN	No	96	15	LMG 1345	*Leeuwenhoekiella*	*marinoflava*
Z0013_RO_A01_1_E07_A	GREEN	No	96	15	LMG 1345	*Leeuwenhoekiella*	*marinoflava*
Z0013_LO_A09_1_E05_A	GREEN	No	96	15	LMG 1345	*Leeuwenhoekiella*	*marinoflava*
Z0013_RO_A10_1_E11_B	GREEN	No	96	15	LMG 1345	*Leeuwenhoekiella*	*marinoflava*
Z0013_RO_A02_1_E07_B	GREEN	No	96	15	LMG 1345	*Leeuwenhoekiella*	*marinoflava*
Z0013_RO_A05_1_E09_A	GREEN	No	96	15	LMG 1345	*Leeuwenhoekiella*	*marinoflava*
Z0013_RO_A04_1_E08_B	GREEN	No	96	15	LMG 1345	*Leeuwenhoekiella*	*marinoflava*
Z0013_LO_A08_1_E04_B	GREEN	No	96	15	LMG 1345	*Leeuwenhoekiella*	*marinoflava*
Z0013_RB_A04_2_D11_B	GREEN	No	96	15	LMG 1345	*Leeuwenhoekiella*	*marinoflava*
Z0013_LB_A10_2_D02_B	GREEN	No	96	15	LMG 1345	*Leeuwenhoekiella*	*marinoflava*
Z0013_LO_A12_1_E06_B	GREEN	No	96	15	LMG 1345	*Leeuwenhoekiella*	*marinoflava*

Z0013_LB_A11_2_D01_A	GREEN	No	96	15	LMG 1345	*Leeuwenhoekiella*	*marinoflava*
Z0013_RB_A07_2_D09_A	GREEN	No	96	15	LMG 1345	*Leeuwenhoekiella*	*marinoflava*
Z0013_RO_A11_1_E12_A	GREEN	No	96	15	LMG 1345	*Leeuwenhoekiella*	*marinoflava*
Z0013_RO_A06_1_E09_B	GREEN	No	96	15	LMG 1345	*Leeuwenhoekiella*	*marinoflava*
Z0013_RB_A08_2_D09_B	GREEN	No	96	15	LMG 1345	*Leeuwenhoekiella*	*marinoflava*
Z0013_LO_A10_1_E05_B	GREEN	No	96	15	LMG 1345	*Leeuwenhoekiella*	*marinoflava*
Z0013_RO_A09_1_E11_A	GREEN	No	96	15	LMG 1345	*Leeuwenhoekiella*	*marinoflava*
Z0013_LO_A07_1_E04_A	GREEN	No	96	15	LMG 1345	*Leeuwenhoekiella*	*marinoflava*
Z0013_RB_A03_2_D11_A	GREEN	No	96	15	LMG 1345	*Leeuwenhoekiella*	*marinoflava*
Z0013_RO_A07_1_E10_A	GREEN	No	96	15	LMG 1345	*Leeuwenhoekiella*	*marinoflava*
Z0013_LO_A11_1_E06_A	GREEN	No	96	15	LMG 1345	*Leeuwenhoekiella*	*marinoflava*
Z0013_RB_A09_2_D08_A	GREEN	No	96	15	LMG 1345	*Leeuwenhoekiella*	*marinoflava*
Z0013_LO_B02_2_E01_B	GREEN	No	96	15	LMG 1345	*Leeuwenhoekiella*	*marinoflava*
Z0013_LO_B01_2_E01_A	GREEN	No	96	15	LMG 1345	*Leeuwenhoekiella*	*marinoflava*
Z0013_LB_A07_2_D03_A	GREEN	No	96	15	LMG 1345	*Leeuwenhoekiella*	*marinoflava*
Z0013_LB_A08_2_D03_B	GREEN	No	96	15	LMG 1345	*Leeuwenhoekiella*	*marinoflava*
Z0013_LO_A06_1_E03_B	GREEN	No	96	15	LMG 1345	*Leeuwenhoekiella*	*marinoflava*
Z0013_RB_A06_2_D10_B	GREEN	No	96	15	LMG 1345	*Leeuwenhoekiella*	*marinoflava*
Z0016_LB_G12_2_A01_B	GREEN	Yes	97	48	LMG 22697	*Mesorhizobium*	*thiogangeticum*
Z0016_RB_G02_2_A12_B	GREEN	No	97	48	LMG 22697	*Mesorhizobium*	*thiogangeticum*
Z0016_LB_G10_2_A02_B	GREEN	No	97	48	LMG 22697	*Mesorhizobium*	*thiogangeticum*
Z0016_LB_H06_1_A04_B	GREEN	No	97	48	LMG 22697	*Mesorhizobium*	*thiogangeticum*
Z0016_LB_G04_2_A05_B	GREEN	No	97	48	LMG 22697	*Mesorhizobium*	*thiogangeticum*
Z0016_LB_H03_1_A05_A	GREEN	No	97	48	LMG 22697	*Mesorhizobium*	*thiogangeticum*
Z0016_LB_H02_1_A06_B	GREEN	No	97	48	LMG 22697	*Mesorhizobium*	*thiogangeticum*
Z0016_RB_G11_2_A07_A	GREEN	No	97	48	LMG 22697	*Mesorhizobium*	*thiogangeticum*
Z0016_RB_H01_1_A12_A	GREEN	No	97	48	LMG 22697	*Mesorhizobium*	*thiogangeticum*
Z0016_LB_H01_1_A06_A	GREEN	No	97	48	LMG 22697	*Mesorhizobium*	*thiogangeticum*
Z0016_LB_H05_1_A04_A	GREEN	No	97	48	LMG 22697	*Mesorhizobium*	*thiogangeticum*
Z0016_RB_G09_2_A08_A	GREEN	No	97	48	LMG 22697	*Mesorhizobium*	*thiogangeticum*
Z0016_LB_H04_1_A05_B	GREEN	No	97	48	LMG 22697	*Mesorhizobium*	*thiogangeticum*
Z0016_LB_G05_2_A04_A	GREEN	No	97	48	LMG 22697	*Mesorhizobium*	*thiogangeticum*
Z0016_RB_G01_2_A12_A	GREEN	No	97	48	LMG 22697	*Mesorhizobium*	*thiogangeticum*
Z0016_LB_G09_2_A02_A	GREEN	No	97	48	LMG 22697	*Mesorhizobium*	*thiogangeticum*
Z0016_LB_G11_2_A01_A	GREEN	No	97	48	LMG 22697	*Mesorhizobium*	*thiogangeticum*
Z0016_RB_G12_2_A07_B	GREEN	No	97	48	LMG 22697	*Mesorhizobium*	*thiogangeticum*
Z0016_LB_G03_2_A05_A	GREEN	No	97	48	LMG 22697	*Mesorhizobium*	*thiogangeticum*
Z0016_RB_G08_2_A09_B	GREEN	No	97	48	LMG 22697	*Mesorhizobium*	*thiogangeticum*
Z0016_RB_G10_2_A08_B	GREEN	No	97	48	LMG 22697	*Mesorhizobium*	*thiogangeticum*
Z0016_RB_H02_1_A12_B	GREEN	No	97	48	LMG 22697	*Mesorhizobium*	*thiogangeticum*
Z0016_LB_G06_2_A04_B	GREEN	No	97	48	LMG 22697	*Mesorhizobium*	*thiogangeticum*
Z0016_RB_G03_2_A11_A	GREEN	No	97	48	LMG 22697	*Mesorhizobium*	*thiogangeticum*
Z0016_LB_G07_2_A03_A	GREEN	No	97	48	LMG 22697	*Mesorhizobium*	*thiogangeticum*
Z0016_RB_G04_2_A11_B	GREEN	No	97	48	LMG 22697	*Mesorhizobium*	*thiogangeticum*
Z0016_RB_H03_1_A11_A	GREEN	No	97	48	LMG 22697	*Mesorhizobium*	*thiogangeticum*
Z0016_RB_G06_2_A10_B	GREEN	No	97	48	LMG 22697	*Mesorhizobium*	*thiogangeticum*
Z0016_RB_F12_1_B07_B	GREEN	No	97	48	LMG 22697	*Mesorhizobium*	*thiogangeticum*
Z0016_RB_G05_2_A10_A	GREEN	No	97	48	LMG 22697	*Mesorhizobium*	*thiogangeticum*
Z0016_LB_G08_2_A03_B	GREEN	No	97	48	LMG 22697	*Mesorhizobium*	*thiogangeticum*
Z0016_RB_G07_2_A09_A	GREEN	Yes	98	48	LMG 22697	*Mesorhizobium*	*thiogangeticum*
Z0018_LB_E02_2_B06_B	GREEN	Yes	99	70	LMG 24411	*Bhargavaea*	*cecembensis*
Z0023_LB_E02_2_B06_B	GREEN	Yes	100	117	LMG 4305	*Rhodobacter*	*blasticus*
Z0023_RB_D03_1_C11_A	GREEN	No	100	117	LMG 4305	*Rhodobacter*	*blasticus*
Z0023_LB_E01_2_B06_A	GREEN	No	100	117	LMG 4305	*Rhodobacter*	*blasticus*
Z0023_RB_C11_2_C07_A	GREEN	No	100	117	LMG 4305	*Rhodobacter*	*blasticus*
Z0023_LB_D11_1_C01_A	GREEN	No	100	117	LMG 4305	*Rhodobacter*	*blasticus*
Z0023_LB_D08_1_C03_B	GREEN	No	100	117	LMG 4305	*Rhodobacter*	*blasticus*
Z0023_LB_D12_1_C01_B	GREEN	No	100	117	LMG 4305	*Rhodobacter*	*blasticus*
Z0023_LB_D03_1_C05_A	GREEN	No	100	117	LMG 4305	*Rhodobacter*	*blasticus*
Z0023_LB_C10_2_C02_B	GREEN	No	100	117	LMG 4305	*Rhodobacter*	*blasticus*
Z0023_LB_C08_2_C03_B	GREEN	No	100	117	LMG 4305	*Rhodobacter*	*blasticus*
Z0023_LB_D06_1_C04_B	GREEN	No	100	117	LMG 4305	*Rhodobacter*	*blasticus*
Z0023_RB_C12_2_C07_B	GREEN	No	100	117	LMG 4305	*Rhodobacter*	*blasticus*
Z0023_LB_D09_1_C02_A	GREEN	No	100	117	LMG 4305	*Rhodobacter*	*blasticus*
Z0023_RB_C05_2_C10_A	GREEN	No	100	117	LMG 4305	*Rhodobacter*	*blasticus*
Z0023_RB_C04_2_C11_B	GREEN	No	100	117	LMG 4305	*Rhodobacter*	*blasticus*
Z0023_RB_D01_1_C12_A	GREEN	No	100	117	LMG 4305	*Rhodobacter*	*blasticus*
Z0023_LB_D05_1_C04_A	GREEN	No	100	117	LMG 4305	*Rhodobacter*	*blasticus*
Z0023_RB_C10_2_C08_B	GREEN	No	100	117	LMG 4305	*Rhodobacter*	*blasticus*
Z0023_LB_D10_1_C02_B	GREEN	No	100	117	LMG 4305	*Rhodobacter*	*blasticus*

Z0023_RB_C07_2_C09_A	GREEN	No	100	117	LMG 4305	*Rhodobacter*	*blasticus*
Z0023_RB_D02_1_C12_B	GREEN	No	100	117	LMG 4305	*Rhodobacter*	*blasticus*
Z0023_LB_D04_1_C05_B	GREEN	No	100	117	LMG 4305	*Rhodobacter*	*blasticus*
Z0023_LB_D01_1_C06_A	GREEN	No	100	117	LMG 4305	*Rhodobacter*	*blasticus*
Z0023_LB_C11_2_C01_A	GREEN	No	100	117	LMG 4305	*Rhodobacter*	*blasticus*
Z0023_LB_D02_1_C06_B	GREEN	No	100	117	LMG 4305	*Rhodobacter*	*blasticus*
Z0023_LB_C09_2_C02_A	GREEN	No	100	117	LMG 4305	*Rhodobacter*	*blasticus*
Z0023_RB_C06_2_C10_B	GREEN	No	100	117	LMG 4305	*Rhodobacter*	*blasticus*
Z0023_LB_C12_2_C01_B	GREEN	No	100	117	LMG 4305	*Rhodobacter*	*blasticus*
Z0023_RB_C09_2_C08_A	GREEN	No	100	117	LMG 4305	*Rhodobacter*	*blasticus*
Z0023_LB_D07_1_C03_A	GREEN	No	100	117	LMG 4305	*Rhodobacter*	*blasticus*
Z0023_RB_C08_2_C09_B	GREEN	No	100	117	LMG 4305	*Rhodobacter*	*blasticus*
Z0023_RB_C03_2_C11_A	GREEN	No	100	117	LMG 4305	*Rhodobacter*	*blasticus*
Z0020_LO_G08_1_H04_B	GREEN	Yes	101	94	LMG 26852	*Achromobacter*	*aegrifaciens*
Z0020_LO_F10_2_G05_B	GREEN	Yes	102	94	LMG 26852	*Achromobacter*	*aegrifaciens*
Z0020_LO_G05_1_H03_A	GREEN	No	102	94	LMG 26852	*Achromobacter*	*aegrifaciens*
Z0020_LO_G07_1_H04_A	GREEN	No	102	94	LMG 26852	*Achromobacter*	*aegrifaciens*
Z0020_RO_G01_1_H07_A	GREEN	No	102	94	LMG 26852	*Achromobacter*	*aegrifaciens*
Z0020_RO_G09_1_H11_A	GREEN	No	102	94	LMG 26852	*Achromobacter*	*aegrifaciens*
Z0020_RO_G02_1_H07_B	GREEN	No	102	94	LMG 26852	*Achromobacter*	*aegrifaciens*
Z0020_RO_G03_1_H08_A	GREEN	Yes	103	94	LMG 26852	*Achromobacter*	*aegrifaciens*
Z0020_LO_F06_2_G03_B	GREEN	No	103	94	LMG 26852	*Achromobacter*	*aegrifaciens*
Z0020_RO_G11_1_H12_A	GREEN	No	103	94	LMG 26852	*Achromobacter*	*aegrifaciens*
Z0020_RO_G08_1_H10_B	GREEN	No	103	94	LMG 26852	*Achromobacter*	*aegrifaciens*
Z0020_RO_G10_1_H11_B	GREEN	No	103	94	LMG 26852	*Achromobacter*	*aegrifaciens*
Z0020_LO_G06_1_H03_B	GREEN	No	103	94	LMG 26852	*Achromobacter*	*aegrifaciens*
Z0020_RO_G05_1_H09_A	GREEN	No	103	94	LMG 26852	*Achromobacter*	*aegrifaciens*
Z0020_LO_F12_2_G06_B	GREEN	No	103	94	LMG 26852	*Achromobacter*	*aegrifaciens*
Z0020_RO_G06_1_H09_B	GREEN	No	103	94	LMG 26852	*Achromobacter*	*aegrifaciens*
Z0020_RO_G04_1_H08_B	GREEN	No	103	94	LMG 26852	*Achromobacter*	*aegrifaciens*
Z0020_LO_F07_2_G04_A	GREEN	No	103	94	LMG 26852	*Achromobacter*	*aegrifaciens*
Z0020_RO_G07_1_H10_A	GREEN	No	103	94	LMG 26852	*Achromobacter*	*aegrifaciens*
Z0020_LO_G04_1_H02_B	GREEN	No	103	94	LMG 26852	*Achromobacter*	*aegrifaciens*
Z0020_LO_G02_1_H01_B	GREEN	No	103	94	LMG 26852	*Achromobacter*	*aegrifaciens*
Z0020_RO_F11_2_G12_A	GREEN	No	103	94	LMG 26852	*Achromobacter*	*aegrifaciens*
Z0020_RO_G12_1_H12_B	GREEN	No	103	94	LMG 26852	*Achromobacter*	*aegrifaciens*
Z0020_LO_G09_1_H05_A	GREEN	Yes	104	94	LMG 26852	*Achromobacter*	*aegrifaciens*
Z0020_LO_G03_1_H02_A	GREEN	No	104	94	LMG 26852	*Achromobacter*	*aegrifaciens*
Z0020_RO_F12_2_G12_B	GREEN	No	104	94	LMG 26852	*Achromobacter*	*aegrifaciens*
Z0020_LO_F02_2_G01_B	GREEN	No	104	94	LMG 26852	*Achromobacter*	*aegrifaciens*
Z0020_RO_H04_2_H08_B	GREEN	No	104	94	LMG 26852	*Achromobacter*	*aegrifaciens*
Z0020_RO_H01_2_H07_A	GREEN	No	104	94	LMG 26852	*Achromobacter*	*aegrifaciens*
Z0020_RO_H03_2_H08_A	GREEN	Yes	105	94	LMG 26852	*Achromobacter*	*aegrifaciens*
Z0020_RO_F10_2_G11_B	GREEN	Yes	106	94	LMG 26852	*Achromobacter*	*aegrifaciens*
Z0020_RO_H02_2_H07_B	GREEN	No	106	94	LMG 26852	*Achromobacter*	*aegrifaciens*
Z0017_LO_B08_2_E04_B	GREEN	Yes	107	62	LMG 24012	*Parapusillimonas*	*granuli*
Z0017_RO_B12_2_E12_B	GREEN	No	107	62	LMG 24012	*Parapusillimonas*	*granuli*
Z0017_RO_C02_1_F07_B	GREEN	No	107	62	LMG 24012	*Parapusillimonas*	*granuli*
Z0017_LO_B09_2_E05_A	GREEN	No	107	62	LMG 24012	*Parapusillimonas*	*granuli*
Z0017_LO_B11_2_E06_A	GREEN	No	107	62	LMG 24012	*Parapusillimonas*	*granuli*
Z0017_LO_B12_2_E06_B	GREEN	No	107	62	LMG 24012	*Parapusillimonas*	*granuli*
Z0017_LO_B03_2_E02_A	GREEN	No	107	62	LMG 24012	*Parapusillimonas*	*granuli*
Z0017_LO_C05_1_F03_A	GREEN	No	107	62	LMG 24012	*Parapusillimonas*	*granuli*
Z0017_RO_C01_1_F07_A	GREEN	No	107	62	LMG 24012	*Parapusillimonas*	*granuli*
Z0017_RO_C05_1_F09_A	GREEN	No	107	62	LMG 24012	*Parapusillimonas*	*granuli*
Z0017_LO_A11_1_E06_A	GREEN	No	107	62	LMG 24012	*Parapusillimonas*	*granuli*
Z0017_RO_B09_2_E11_A	GREEN	No	107	62	LMG 24012	*Parapusillimonas*	*granuli*
Z0017_RO_C10_1_F11_B	GREEN	No	107	62	LMG 24012	*Parapusillimonas*	*granuli*
Z0017_LO_C03_1_F02_A	GREEN	No	107	62	LMG 24012	*Parapusillimonas*	*granuli*
Z0017_RO_C12_1_F12_B	GREEN	No	107	62	LMG 24012	*Parapusillimonas*	*granuli*
Z0017_LO_C02_1_F01_B	GREEN	No	107	62	LMG 24012	*Parapusillimonas*	*granuli*
Z0017_LO_A08_1_E04_B	GREEN	No	107	62	LMG 24012	*Parapusillimonas*	*granuli*
Z0017_LO_C06_1_F03_B	GREEN	No	107	62	LMG 24012	*Parapusillimonas*	*granuli*
Z0017_LO_A12_1_E06_B	GREEN	No	107	62	LMG 24012	*Parapusillimonas*	*granuli*
Z0017_RO_B11_2_E12_A	GREEN	No	107	62	LMG 24012	*Parapusillimonas*	*granuli*
Z0017_RO_B08_2_E10_B	GREEN	No	107	62	LMG 24012	*Parapusillimonas*	*granuli*
Z0017_LO_B07_2_E04_A	GREEN	No	107	62	LMG 24012	*Parapusillimonas*	*granuli*
Z0017_RO_B07_2_E10_A	GREEN	No	107	62	LMG 24012	*Parapusillimonas*	*granuli*
Z0017_RO_C07_1_F10_A	GREEN	No	107	62	LMG 24012	*Parapusillimonas*	*granuli*
Z0017_RO_D01_2_F07_A	GREEN	No	107	62	LMG 24012	*Parapusillimonas*	*granuli*

Z0017_RO_B02_2_E07_B	GREEN	No	107	62	LMG 24012	*Parapusillimonas*	*granuli*
Z0017_LO_A09_1_E05_A	GREEN	No	107	62	LMG 24012	*Parapusillimonas*	*granuli*
Z0017_LO_A10_1_E05_B	GREEN	No	107	62	LMG 24012	*Parapusillimonas*	*granuli*
Z0020_LO_B03_2_E02_A	GREEN	Yes	108	96	LMG 27019	*Kozakia*	*baliensis*
Z0020_RO_D04_2_F08_B	GREEN	No	108	96	LMG 27019	*Kozakia*	*baliensis*
Z0020_RO_C09_1_F11_A	GREEN	No	108	96	LMG 27019	*Kozakia*	*baliensis*
Z0020_LO_D03_2_F02_A	GREEN	No	108	96	LMG 27019	*Kozakia*	*baliensis*
Z0020_LO_A10_1_E05_B	GREEN	No	108	96	LMG 27019	*Kozakia*	*baliensis*
Z0020_LO_B12_2_E06_B	GREEN	No	108	96	LMG 27019	*Kozakia*	*baliensis*
Z0020_LO_C06_1_F03_B	GREEN	No	108	96	LMG 27019	*Kozakia*	*baliensis*
Z0020_RO_C10_1_F11_B	GREEN	No	108	96	LMG 27019	*Kozakia*	*baliensis*
Z0020_LO_A04_1_E02_B	GREEN	No	108	96	LMG 27019	*Kozakia*	*baliensis*
Z0020_LB_H11_1_A01_A	GREEN	No	108	96	LMG 27019	*Kozakia*	*baliensis*
Z0020_LB_H05_1_A04_A	GREEN	No	108	96	LMG 27019	*Kozakia*	*baliensis*
Z0020_RO_A11_1_E12_A	GREEN	No	108	96	LMG 27019	*Kozakia*	*baliensis*
Z0020_RO_C11_1_F12_A	GREEN	No	108	96	LMG 27019	*Kozakia*	*baliensis*
Z0020_RO_A08_1_E10_B	GREEN	No	108	96	LMG 27019	*Kozakia*	*baliensis*
Z0020_LO_B02_2_E01_B	GREEN	No	108	96	LMG 27019	*Kozakia*	*baliensis*
Z0020_RO_D02_2_F07_B	GREEN	No	108	96	LMG 27019	*Kozakia*	*baliensis*
Z0020_RO_C12_1_F12_B	GREEN	No	108	96	LMG 27019	*Kozakia*	*baliensis*
Z0020_RO_C07_1_F10_A	GREEN	No	108	96	LMG 27019	*Kozakia*	*baliensis*
Z0020_LO_B01_2_E01_A	GREEN	No	108	96	LMG 27019	*Kozakia*	*baliensis*
Z0020_LB_H07_1_A03_A	GREEN	No	108	96	LMG 27019	*Kozakia*	*baliensis*
Z0020_LO_B06_2_E03_B	GREEN	No	108	96	LMG 27019	*Kozakia*	*baliensis*
Z0020_RO_B01_2_E07_A	GREEN	No	108	96	LMG 27019	*Kozakia*	*baliensis*
Z0020_RO_C01_1_F07_A	GREEN	No	108	96	LMG 27019	*Kozakia*	*baliensis*
Z0020_LB_H09_1_A02_A	GREEN	No	108	96	LMG 27019	*Kozakia*	*baliensis*
Z0020_LO_A09_1_E05_A	GREEN	No	108	96	LMG 27019	*Kozakia*	*baliensis*
Z0020_LO_C12_1_F06_B	GREEN	No	108	96	LMG 27019	*Kozakia*	*baliensis*
Z0020_RO_D06_2_F09_B	GREEN	No	108	96	LMG 27019	*Kozakia*	*baliensis*
Z0020_LO_C10_1_F05_B	GREEN	No	108	96	LMG 27019	*Kozakia*	*baliensis*
Z0020_RO_C06_1_F09_B	GREEN	No	108	96	LMG 27019	*Kozakia*	*baliensis*
Z0020_LB_H08_1_A03_B	GREEN	No	108	96	LMG 27019	*Kozakia*	*baliensis*
Z0020_RO_C04_1_F08_B	GREEN	No	108	96	LMG 27019	*Kozakia*	*baliensis*
Z0020_RO_D03_2_F08_A	GREEN	No	108	96	LMG 27019	*Kozakia*	*baliensis*
Z0020_LB_H06_1_A04_B	GREEN	Yes	109	92	LMG 26473	*Alishewanella*	*tabrizica*
Z0020_RO_A12_1_E12_B	GREEN	No	109	92	LMG 26473	*Alishewanella*	*tabrizica*
Z0020_RB_H01_1_A12_A	GREEN	No	109	92	LMG 26473	*Alishewanella*	*tabrizica*
Z0020_LO_A01_1_E01_A	GREEN	No	109	92	LMG 26473	*Alishewanella*	*tabrizica*
Z0020_LB_H04_1_A05_B	GREEN	No	109	92	LMG 26473	*Alishewanella*	*tabrizica*
Z0020_RO_A09_1_E11_A	GREEN	No	109	92	LMG 26473	*Alishewanella*	*tabrizica*
Z0020_RB_H09_1_A08_A	GREEN	No	109	92	LMG 26473	*Alishewanella*	*tabrizica*
Z0020_LB_H03_1_A05_A	GREEN	No	109	92	LMG 26473	*Alishewanella*	*tabrizica*
Z0020_RB_H12_1_A07_B	GREEN	No	109	92	LMG 26473	*Alishewanella*	*tabrizica*
Z0020_RB_H03_1_A11_A	GREEN	No	109	92	LMG 26473	*Alishewanella*	*tabrizica*
Z0020_RB_H11_1_A07_A	GREEN	No	109	92	LMG 26473	*Alishewanella*	*tabrizica*
Z0020_RB_G12_2_A07_B	GREEN	No	109	92	LMG 26473	*Alishewanella*	*tabrizica*
Z0020_RB_H02_1_A12_B	GREEN	No	109	92	LMG 26473	*Alishewanella*	*tabrizica*
Z0020_LO_A03_1_E02_A	GREEN	No	109	92	LMG 26473	*Alishewanella*	*tabrizica*
Z0020_RB_H07_1_A09_A	GREEN	No	109	92	LMG 26473	*Alishewanella*	*tabrizica*
Z0020_RO_A03_1_E08_A	GREEN	No	109	92	LMG 26473	*Alishewanella*	*tabrizica*
Z0020_RO_A07_1_E10_A	GREEN	No	109	92	LMG 26473	*Alishewanella*	*tabrizica*
Z0020_RB_H06_1_A10_B	GREEN	No	109	92	LMG 26473	*Alishewanella*	*tabrizica*
Z0020_RB_H05_1_A10_A	GREEN	No	109	92	LMG 26473	*Alishewanella*	*tabrizica*
Z0020_RO_A10_1_E11_B	GREEN	No	109	92	LMG 26473	*Alishewanella*	*tabrizica*
Z0020_LO_A05_1_E03_A	GREEN	No	109	92	LMG 26473	*Alishewanella*	*tabrizica*
Z0020_RO_A02_1_E07_B	GREEN	No	109	92	LMG 26473	*Alishewanella*	*tabrizica*
Z0020_RO_A05_1_E09_A	GREEN	No	109	92	LMG 26473	*Alishewanella*	*tabrizica*
Z0020_LB_H10_1_A02_B	GREEN	No	109	92	LMG 26473	*Alishewanella*	*tabrizica*
Z0020_LB_H12_1_A01_B	GREEN	No	109	92	LMG 26473	*Alishewanella*	*tabrizica*
Z0020_LO_A02_1_E01_B	GREEN	No	109	92	LMG 26473	*Alishewanella*	*tabrizica*
Z0020_RB_H10_1_A08_B	GREEN	No	109	92	LMG 26473	*Alishewanella*	*tabrizica*
Z0020_LO_A06_1_E03_B	GREEN	No	109	92	LMG 26473	*Alishewanella*	*tabrizica*
Z0013_RB_D08_1_C09_B	GREEN	Yes	110	20	LMG 1617	*Acetobacter*	*lovaniensis*
Z0013_RB_D07_1_C09_A	GREEN	No	110	20	LMG 1617	*Acetobacter*	*lovaniensis*
Z0013_RO_D12_2_F12_B	GREEN	No	110	20	LMG 1617	*Acetobacter*	*lovaniensis*
Z0013_RO_D11_2_F12_A	GREEN	No	110	20	LMG 1617	*Acetobacter*	*lovaniensis*
Z0013_LB_D12_1_C01_B	GREEN	No	110	20	LMG 1617	*Acetobacter*	*lovaniensis*
Z0013_LO_D07_2_F04_A	GREEN	No	110	20	LMG 1617	*Acetobacter*	*lovaniensis*
Z0013_LO_D08_2_F04_B	GREEN	No	110	20	LMG 1617	*Acetobacter*	*lovaniensis*

Z0013_LO_D09_2_F05_A	GREEN	No	110	20	LMG 1617	*Acetobacter*	*lovaniensis*
Z0013_LB_D11_1_C01_A	GREEN	No	110	20	LMG 1617	*Acetobacter*	*lovaniensis*
Z0013_LB_E02_2_B06_B	GREEN	No	110	20	LMG 1617	*Acetobacter*	*lovaniensis*
Z0013_RO_E03_1_G08_A	GREEN	No	110	20	LMG 1617	*Acetobacter*	*lovaniensis*
Z0013_LB_D10_1_C02_B	GREEN	No	110	20	LMG 1617	*Acetobacter*	*lovaniensis*
Z0013_LO_D12_2_F06_B	GREEN	No	110	20	LMG 1617	*Acetobacter*	*lovaniensis*
Z0013_RB_E02_2_B12_B	GREEN	No	110	20	LMG 1617	*Acetobacter*	*lovaniensis*
Z0013_LO_D10_2_F05_B	GREEN	No	110	20	LMG 1617	*Acetobacter*	*lovaniensis*
Z0013_LB_D09_1_C02_A	GREEN	No	110	20	LMG 1617	*Acetobacter*	*lovaniensis*
Z0013_RB_D10_1_C08_B	GREEN	No	110	20	LMG 1617	*Acetobacter*	*lovaniensis*
Z0013_RO_E02_1_G07_B	GREEN	No	110	20	LMG 1617	*Acetobacter*	*lovaniensis*
Z0013_LO_E01_1_G01_A	GREEN	No	110	20	LMG 1617	*Acetobacter*	*lovaniensis*
Z0013_LO_D11_2_F06_A	GREEN	No	110	20	LMG 1617	*Acetobacter*	*lovaniensis*
Z0013_RO_E04_1_G08_B	GREEN	No	110	20	LMG 1617	*Acetobacter*	*lovaniensis*
Z0013_RB_D09_1_C08_A	GREEN	No	110	20	LMG 1617	*Acetobacter*	*lovaniensis*
Z0013_RB_E01_2_B12_A	GREEN	No	110	20	LMG 1617	*Acetobacter*	*lovaniensis*
Z0013_RB_D12_1_C07_B	GREEN	No	110	20	LMG 1617	*Acetobacter*	*lovaniensis*
Z0013_RO_D10_2_F11_B	GREEN	No	110	20	LMG 1617	*Acetobacter*	*lovaniensis*
Z0013_LB_E01_2_B06_A	GREEN	No	110	20	LMG 1617	*Acetobacter*	*lovaniensis*
Z0013_RB_D11_1_C07_A	GREEN	No	110	20	LMG 1617	*Acetobacter*	*lovaniensis*
Z0013_RO_E01_1_G07_A	GREEN	No	110	20	LMG 1617	*Acetobacter*	*lovaniensis*
Z0021_RB_F04_1_B11_B	GREEN	Yes	111	101	LMG 27719	*Roseomonas*	*gilardii*
Z0021_RB_F08_1_B09_B	GREEN	No	111	101	LMG 27719	*Roseomonas*	*gilardii*
Z0021_RB_E09_2_B08_A	GREEN	No	111	101	LMG 27719	*Roseomonas*	*gilardii*
Z0021_LB_F05_1_B04_A	GREEN	No	111	101	LMG 27719	*Roseomonas*	*gilardii*
Z0021_LB_E11_2_B01_A	GREEN	No	111	101	LMG 27719	*Roseomonas*	*gilardii*
Z0021_RB_E07_2_B09_A	GREEN	No	111	101	LMG 27719	*Roseomonas*	*gilardii*
Z0021_RB_E10_2_B08_B	GREEN	No	111	101	LMG 27719	*Roseomonas*	*gilardii*
Z0021_LB_F07_1_B03_A	GREEN	No	111	101	LMG 27719	*Roseomonas*	*gilardii*
Z0021_LB_F10_1_B02_B	GREEN	No	111	101	LMG 27719	*Roseomonas*	*gilardii*
Z0021_LB_F08_1_B03_B	GREEN	No	111	101	LMG 27719	*Roseomonas*	*gilardii*
Z0021_RB_F03_1_B11_A	GREEN	No	111	101	LMG 27719	*Roseomonas*	*gilardii*
Z0021_LB_F04_1_B05_B	GREEN	No	111	101	LMG 27719	*Roseomonas*	*gilardii*
Z0021_LB_F06_1_B04_B	GREEN	No	111	101	LMG 27719	*Roseomonas*	*gilardii*
Z0021_LB_E12_2_B01_B	GREEN	No	111	101	LMG 27719	*Roseomonas*	*gilardii*
Z0021_RB_E08_2_B09_B	GREEN	No	111	101	LMG 27719	*Roseomonas*	*gilardii*
Z0021_RB_F09_1_B08_A	GREEN	No	111	101	LMG 27719	*Roseomonas*	*gilardii*
Z0021_RB_F06_1_B10_B	GREEN	No	111	101	LMG 27719	*Roseomonas*	*gilardii*
Z0021_RB_F07_1_B09_A	GREEN	No	111	101	LMG 27719	*Roseomonas*	*gilardii*
Z0021_LB_F11_1_B01_A	GREEN	No	111	101	LMG 27719	*Roseomonas*	*gilardii*
Z0021_LB_F01_1_B06_A	GREEN	No	111	101	LMG 27719	*Roseomonas*	*gilardii*
Z0021_RB_F01_1_B12_A	GREEN	No	111	101	LMG 27719	*Roseomonas*	*gilardii*
Z0021_RB_F05_1_B10_A	GREEN	No	111	101	LMG 27719	*Roseomonas*	*gilardii*
Z0021_LB_F03_1_B05_A	GREEN	No	111	101	LMG 27719	*Roseomonas*	*gilardii*
Z0021_RB_F11_1_B07_A	GREEN	No	111	101	LMG 27719	*Roseomonas*	*gilardii*
Z0021_LB_F09_1_B02_A	GREEN	No	111	101	LMG 27719	*Roseomonas*	*gilardii*
Z0021_RB_E11_2_B07_A	GREEN	No	111	101	LMG 27719	*Roseomonas*	*gilardii*
Z0021_RB_F10_1_B08_B	GREEN	No	111	101	LMG 27719	*Roseomonas*	*gilardii*
Z0021_RB_G01_2_A12_A	GREEN	No	111	101	LMG 27719	*Roseomonas*	*gilardii*
Z0021_LB_F02_1_B06_B	GREEN	No	111	101	LMG 27719	*Roseomonas*	*gilardii*
Z0021_RB_F12_1_B07_B	GREEN	No	111	101	LMG 27719	*Roseomonas*	*gilardii*
Z0021_RB_F02_1_B12_B	GREEN	No	111	101	LMG 27719	*Roseomonas*	*gilardii*
Z0021_RB_E12_2_B07_B	GREEN	No	111	101	LMG 27719	*Roseomonas*	*gilardii*
Z0022_RB_G12_2_A07_B	GREEN	Yes	112	109	LMG 3252	*Citrobacter*	*youngae*
Z0022_RB_G11_2_A07_A	GREEN	No	112	109	LMG 3252	*Citrobacter*	*youngae*
Z0022_RB_G10_2_A08_B	GREEN	Yes	113	109	LMG 3252	*Citrobacter*	*youngae*
Z0022_LB_H06_1_A04_B	GREEN	No	113	109	LMG 3252	*Citrobacter*	*youngae*
Z0022_RB_G09_2_A08_A	GREEN	No	113	109	LMG 3252	*Citrobacter*	*youngae*
Z0022_RB_H01_1_A12_A	GREEN	No	113	109	LMG 3252	*Citrobacter*	*youngae*
Z0022_LB_H08_1_A03_B	GREEN	No	113	109	LMG 3252	*Citrobacter*	*youngae*
Z0022_LB_G06_2_A04_B	GREEN	No	113	109	LMG 3252	*Citrobacter*	*youngae*
Z0022_LB_G10_2_A02_B	GREEN	No	113	109	LMG 3252	*Citrobacter*	*youngae*
Z0022_RB_H05_1_A10_A	GREEN	No	113	109	LMG 3252	*Citrobacter*	*youngae*
Z0022_LB_G07_2_A03_A	GREEN	No	113	109	LMG 3252	*Citrobacter*	*youngae*
Z0022_RB_G06_2_A10_B	GREEN	No	113	109	LMG 3252	*Citrobacter*	*youngae*
Z0022_LB_H05_1_A04_A	GREEN	No	113	109	LMG 3252	*Citrobacter*	*youngae*
Z0022_LB_G08_2_A03_B	GREEN	No	113	109	LMG 3252	*Citrobacter*	*youngae*
Z0022_LB_G12_2_A01_B	GREEN	No	113	109	LMG 3252	*Citrobacter*	*youngae*
Z0022_RB_G08_2_A09_B	GREEN	No	113	109	LMG 3252	*Citrobacter*	*youngae*
Z0022_RB_G03_2_A11_A	GREEN	No	113	109	LMG 3252	*Citrobacter*	*youngae*

Z0022_LB_G09_2_A02_A	GREEN	No	113	109	LMG 3252	*Citrobacter*	*youngae*
Z0022_LB_G11_2_A01_A	GREEN	No	113	109	LMG 3252	*Citrobacter*	*youngae*
Z0022_RB_H02_1_A12_B	GREEN	No	113	109	LMG 3252	*Citrobacter*	*youngae*
Z0022_LB_G05_2_A04_A	GREEN	No	113	109	LMG 3252	*Citrobacter*	*youngae*
Z0022_LB_H04_1_A05_B	GREEN	No	113	109	LMG 3252	*Citrobacter*	*youngae*
Z0022_RB_G07_2_A09_A	GREEN	No	113	109	LMG 3252	*Citrobacter*	*youngae*
Z0022_LB_H03_1_A05_A	GREEN	No	113	109	LMG 3252	*Citrobacter*	*youngae*
Z0022_RB_G05_2_A10_A	GREEN	No	113	109	LMG 3252	*Citrobacter*	*youngae*
Z0022_LB_H02_1_A06_B	GREEN	No	113	109	LMG 3252	*Citrobacter*	*youngae*
Z0022_LB_H07_1_A03_A	GREEN	No	113	109	LMG 3252	*Citrobacter*	*youngae*
Z0022_RB_G02_2_A12_B	GREEN	No	113	109	LMG 3252	*Citrobacter*	*youngae*
Z0022_LB_H01_1_A06_A	GREEN	No	113	109	LMG 3252	*Citrobacter*	*youngae*
Z0022_RB_G04_2_A11_B	GREEN	No	113	109	LMG 3252	*Citrobacter*	*youngae*
Z0022_RB_H04_1_A11_B	GREEN	No	113	109	LMG 3252	*Citrobacter*	*youngae*
Z0022_RB_H03_1_A11_A	GREEN	No	113	109	LMG 3252	*Citrobacter*	*youngae*
Z0019_RB_E09_2_B08_A	GREEN	Yes	114	81	LMG 25547	*Glaciimonas*	*immobilis*
Z0019_RB_F05_1_B10_A	GREEN	No	114	81	LMG 25547	*Glaciimonas*	*immobilis*
Z0019_LB_F07_1_B03_A	GREEN	No	114	81	LMG 25547	*Glaciimonas*	*immobilis*
Z0019_LB_E08_2_B03_B	GREEN	No	114	81	LMG 25547	*Glaciimonas*	*immobilis*
Z0019_RB_F03_1_B11_A	GREEN	No	114	81	LMG 25547	*Glaciimonas*	*immobilis*
Z0019_RB_F02_1_B12_B	GREEN	No	114	81	LMG 25547	*Glaciimonas*	*immobilis*
Z0019_RB_E12_2_B07_B	GREEN	No	114	81	LMG 25547	*Glaciimonas*	*immobilis*
Z0019_LB_E09_2_B02_A	GREEN	No	114	81	LMG 25547	*Glaciimonas*	*immobilis*
Z0019_RB_E11_2_B07_A	GREEN	No	114	81	LMG 25547	*Glaciimonas*	*immobilis*
Z0019_RB_E10_2_B08_B	GREEN	No	114	81	LMG 25547	*Glaciimonas*	*immobilis*
Z0019_RB_E08_2_B09_B	GREEN	No	114	81	LMG 25547	*Glaciimonas*	*immobilis*
Z0019_RB_F04_1_B11_B	GREEN	No	114	81	LMG 25547	*Glaciimonas*	*immobilis*
Z0019_RB_F10_1_B08_B	GREEN	No	114	81	LMG 25547	*Glaciimonas*	*immobilis*
Z0019_LB_E11_2_B01_A	GREEN	No	114	81	LMG 25547	*Glaciimonas*	*immobilis*
Z0019_RB_F08_1_B09_B	GREEN	No	114	81	LMG 25547	*Glaciimonas*	*immobilis*
Z0019_RB_F01_1_B12_A	GREEN	No	114	81	LMG 25547	*Glaciimonas*	*immobilis*
Z0019_LB_E10_2_B02_B	GREEN	No	114	81	LMG 25547	*Glaciimonas*	*immobilis*
Z0019_RB_E07_2_B09_A	GREEN	No	114	81	LMG 25547	*Glaciimonas*	*immobilis*
Z0019_RB_F06_1_B10_B	GREEN	No	114	81	LMG 25547	*Glaciimonas*	*immobilis*
Z0019_LB_F01_1_B06_A	GREEN	No	114	81	LMG 25547	*Glaciimonas*	*immobilis*
Z0019_RB_F11_1_B07_A	GREEN	No	114	81	LMG 25547	*Glaciimonas*	*immobilis*
Z0019_LB_E12_2_B01_B	GREEN	No	114	81	LMG 25547	*Glaciimonas*	*immobilis*
Z0019_RB_F07_1_B09_A	GREEN	No	114	81	LMG 25547	*Glaciimonas*	*immobilis*
Z0019_LB_F02_1_B06_B	GREEN	No	114	81	LMG 25547	*Glaciimonas*	*immobilis*
Z0019_RB_F12_1_B07_B	GREEN	No	114	81	LMG 25547	*Glaciimonas*	*immobilis*
Z0019_RB_G01_2_A12_A	GREEN	No	114	81	LMG 25547	*Glaciimonas*	*immobilis*
Z0019_LB_F06_1_B04_B	GREEN	No	114	81	LMG 25547	*Glaciimonas*	*immobilis*
Z0019_LB_F04_1_B05_B	GREEN	No	114	81	LMG 25547	*Glaciimonas*	*immobilis*
Z0019_RB_F09_1_B08_A	GREEN	No	114	81	LMG 25547	*Glaciimonas*	*immobilis*
Z0019_LB_F05_1_B04_A	GREEN	No	114	81	LMG 25547	*Glaciimonas*	*immobilis*
Z0019_LB_F03_1_B05_A	GREEN	No	114	81	LMG 25547	*Glaciimonas*	*immobilis*
Z0019_LB_F08_1_B03_B	GREEN	No	114	81	LMG 25547	*Glaciimonas*	*immobilis*
Z0022_LO_A03_1_E02_A	GREEN	Yes	115	114	LMG 4050	*Micrococcus*	*luteus*
Z0022_LO_E02_1_G01_B	GREEN	No	115	114	LMG 4050	*Micrococcus*	*luteus*
Z0022_LO_C03_1_F02_A	GREEN	No	115	114	LMG 4050	*Micrococcus*	*luteus*
Z0022_LO_A02_1_E01_B	GREEN	No	115	114	LMG 4050	*Micrococcus*	*luteus*
Z0022_LO_E05_1_G03_A	GREEN	No	115	114	LMG 4050	*Micrococcus*	*luteus*
Z0022_LO_C01_1_F01_A	GREEN	No	115	114	LMG 4050	*Micrococcus*	*luteus*
Z0022_LO_D06_2_F03_B	GREEN	No	115	114	LMG 4050	*Micrococcus*	*luteus*
Z0022_LO_C02_1_F01_B	GREEN	No	115	114	LMG 4050	*Micrococcus*	*luteus*
Z0022_LO_D11_2_F06_A	GREEN	No	115	114	LMG 4050	*Micrococcus*	*luteus*
Z0022_LO_E11_1_G06_A	GREEN	No	115	114	LMG 4050	*Micrococcus*	*luteus*
Z0022_RO_B10_2_E11_B	GREEN	No	115	114	LMG 4050	*Micrococcus*	*luteus*
Z0022_RO_C02_1_F07_B	GREEN	No	115	114	LMG 4050	*Micrococcus*	*luteus*
Z0022_LO_E09_1_G05_A	GREEN	No	115	114	LMG 4050	*Micrococcus*	*luteus*
Z0022_LO_D07_2_F04_A	GREEN	No	115	114	LMG 4050	*Micrococcus*	*luteus*
Z0022_RO_C05_1_F09_A	GREEN	No	115	114	LMG 4050	*Micrococcus*	*luteus*
Z0022_RO_C04_1_F08_B	GREEN	No	115	114	LMG 4050	*Micrococcus*	*luteus*
Z0022_LO_E10_1_G05_B	GREEN	No	115	114	LMG 4050	*Micrococcus*	*luteus*
Z0022_RO_C01_1_F07_A	GREEN	No	115	114	LMG 4050	*Micrococcus*	*luteus*
Z0022_RO_B11_2_E12_A	GREEN	No	115	114	LMG 4050	*Micrococcus*	*luteus*
Z0022_LO_E04_1_G02_B	GREEN	No	115	114	LMG 4050	*Micrococcus*	*luteus*
Z0022_LO_D08_2_F04_B	GREEN	No	115	114	LMG 4050	*Micrococcus*	*luteus*
Z0022_LO_A11_1_E06_A	GREEN	No	115	114	LMG 4050	*Micrococcus*	*luteus*
Z0022_RO_D11_2_F12_A	GREEN	No	115	114	LMG 4050	*Micrococcus*	*luteus*

Z0022_RO_B09_2_E11_A	GREEN	No	115	114	LMG 4050	*Micrococcus*	*luteus*	
Z0022_LO_E01_1_G01_A	GREEN	No	115	114	LMG 4050	*Micrococcus*	*luteus*	
Z0022_RO_D07_2_F10_A	GREEN	No	115	114	LMG 4050	*Micrococcus*	*luteus*	
Z0022_RO_B12_2_E12_B	GREEN	No	115	114	LMG 4050	*Micrococcus*	*luteus*	
Z0022_RO_C06_1_F09_B	GREEN	No	115	114	LMG 4050	*Micrococcus*	*luteus*	
Z0022_RO_C03_1_F08_A	GREEN	No	115	114	LMG 4050	*Micrococcus*	*luteus*	
Z0022_LO_D10_2_F05_B	GREEN	No	115	114	LMG 4050	*Micrococcus*	*luteus*	
Z0022_RO_D01_2_F07_A	GREEN	No	115	114	LMG 4050	*Micrococcus*	*luteus*	
Z0022_RO_D04_2_F08_B	GREEN	No	115	114	LMG 4050	*Micrococcus*	*luteus*	
Z0017_LO_G03_1_H02_A	GREEN	Yes	116	64	LMG 24024	*Oceanobacillus*	*oncorhynchi*	*oncorhynchi*
Z0017_LO_G02_1_H01_B	GREEN	No	116	64	LMG 24024	*Oceanobacillus*	*oncorhynchi*	*oncorhynchi*
Z0017_RO_G05_1_H09_A	GREEN	No	116	64	LMG 24024	*Oceanobacillus*	*oncorhynchi*	*oncorhynchi*
Z0017_RO_H01_2_H07_A	GREEN	No	116	64	LMG 24024	*Oceanobacillus*	*oncorhynchi*	*oncorhynchi*
Z0017_RO_G06_1_H09_B	GREEN	No	116	64	LMG 24024	*Oceanobacillus*	*oncorhynchi*	*oncorhynchi*
Z0017_LO_G01_1_H01_A	GREEN	No	116	64	LMG 24024	*Oceanobacillus*	*oncorhynchi*	*oncorhynchi*
Z0017_LO_F12_2_G06_B	GREEN	No	116	64	LMG 24024	*Oceanobacillus*	*oncorhynchi*	*oncorhynchi*
Z0017_LO_G09_1_H05_A	GREEN	No	116	64	LMG 24024	*Oceanobacillus*	*oncorhynchi*	*oncorhynchi*
Z0017_LO_G06_1_H03_B	GREEN	No	116	64	LMG 24024	*Oceanobacillus*	*oncorhynchi*	*oncorhynchi*
Z0017_RO_G08_1_H10_B	GREEN	No	116	64	LMG 24024	*Oceanobacillus*	*oncorhynchi*	*oncorhynchi*
Z0017_LO_G05_1_H03_A	GREEN	No	116	64	LMG 24024	*Oceanobacillus*	*oncorhynchi*	*oncorhynchi*
Z0017_LO_G07_1_H04_A	GREEN	No	116	64	LMG 24024	*Oceanobacillus*	*oncorhynchi*	*oncorhynchi*
Z0017_LO_F04_2_G02_B	GREEN	No	116	64	LMG 24024	*Oceanobacillus*	*oncorhynchi*	*oncorhynchi*
Z0017_LO_F10_2_G05_B	GREEN	No	116	64	LMG 24024	*Oceanobacillus*	*oncorhynchi*	*oncorhynchi*
Z0017_LO_F09_2_G05_A	GREEN	No	116	64	LMG 24024	*Oceanobacillus*	*oncorhynchi*	*oncorhynchi*
Z0017_LO_F11_2_G06_A	GREEN	No	116	64	LMG 24024	*Oceanobacillus*	*oncorhynchi*	*oncorhynchi*
Z0017_RO_G02_1_H07_B	GREEN	No	116	64	LMG 24024	*Oceanobacillus*	*oncorhynchi*	*oncorhynchi*
Z0017_RO_F12_2_G12_B	GREEN	No	116	64	LMG 24024	*Oceanobacillus*	*oncorhynchi*	*oncorhynchi*
Z0017_RO_G07_1_H10_A	GREEN	No	116	64	LMG 24024	*Oceanobacillus*	*oncorhynchi*	*oncorhynchi*
Z0017_LO_G04_1_H02_B	GREEN	No	116	64	LMG 24024	*Oceanobacillus*	*oncorhynchi*	*oncorhynchi*
Z0017_RO_H04_2_H08_B	GREEN	No	116	64	LMG 24024	*Oceanobacillus*	*oncorhynchi*	*oncorhynchi*
Z0017_RO_H02_2_H07_B	GREEN	No	116	64	LMG 24024	*Oceanobacillus*	*oncorhynchi*	*oncorhynchi*
Z0017_RO_G09_1_H11_A	GREEN	No	116	64	LMG 24024	*Oceanobacillus*	*oncorhynchi*	*oncorhynchi*
Z0017_RO_G11_1_H12_A	GREEN	No	116	64	LMG 24024	*Oceanobacillus*	*oncorhynchi*	*oncorhynchi*
Z0017_RO_G10_1_H11_B	GREEN	No	116	64	LMG 24024	*Oceanobacillus*	*oncorhynchi*	*oncorhynchi*
Z0017_RO_G12_1_H12_B	GREEN	No	116	64	LMG 24024	*Oceanobacillus*	*oncorhynchi*	*oncorhynchi*
Z0017_RO_G04_1_H08_B	GREEN	No	116	64	LMG 24024	*Oceanobacillus*	*oncorhynchi*	*oncorhynchi*
Z0017_RO_G03_1_H08_A	GREEN	No	116	64	LMG 24024	*Oceanobacillus*	*oncorhynchi*	*oncorhynchi*
Z0017_LO_G08_1_H04_B	GREEN	No	116	64	LMG 24024	*Oceanobacillus*	*oncorhynchi*	*oncorhynchi*
Z0017_RO_G01_1_H07_A	GREEN	No	116	64	LMG 24024	*Oceanobacillus*	*oncorhynchi*	*oncorhynchi*
Z0017_RO_H03_2_H08_A	GREEN	No	116	64	LMG 24024	*Oceanobacillus*	*oncorhynchi*	*oncorhynchi*
Z0017_LO_F07_2_G04_A	GREEN	No	116	64	LMG 24024	*Oceanobacillus*	*oncorhynchi*	*oncorhynchi*
Z0018_RO_E05_1_G09_A	GREEN	Yes	117	4	LMG 24832	*Lactobacillus*	*plantarum*	
Z0014_RB_A10_2_D08_B	GREEN	Yes	118	4	LMG 18021	*Lactobacillus*	*plantarum*	
Z0014_LB_A05_2_D04_A	GREEN	No	118	4	LMG 18021	*Lactobacillus*	*plantarum*	
Z0014_LB_A06_2_D04_B	GREEN	No	118	4	LMG 18021	*Lactobacillus*	*plantarum*	
Z0014_RB_A03_2_D11_A	GREEN	No	118	4	LMG 18021	*Lactobacillus*	*plantarum*	
Z0014_RB_A09_2_D08_A	GREEN	No	118	4	LMG 18021	*Lactobacillus*	*plantarum*	
Z0014_RB_A05_2_D10_A	GREEN	No	118	4	LMG 18021	*Lactobacillus*	*plantarum*	
Z0014_RB_A12_2_D07_B	GREEN	No	118	4	LMG 18021	*Lactobacillus*	*plantarum*	
Z0014_LB_B02_1_D06_B	GREEN	No	118	4	LMG 18021	*Lactobacillus*	*plantarum*	
Z0014_RB_B02_1_D12_B	GREEN	No	118	4	LMG 18021	*Lactobacillus*	*plantarum*	
Z0014_LB_A04_2_D05_B	GREEN	No	118	4	LMG 18021	*Lactobacillus*	*plantarum*	
Z0014_RB_A07_2_D09_A	GREEN	No	118	4	LMG 18021	*Lactobacillus*	*plantarum*	
Z0014_RB_A04_2_D11_B	GREEN	No	118	4	LMG 18021	*Lactobacillus*	*plantarum*	
Z0014_RB_B06_1_D10_B	GREEN	No	118	4	LMG 18021	*Lactobacillus*	*plantarum*	
Z0014_RB_A08_2_D09_B	GREEN	No	118	4	LMG 18021	*Lactobacillus*	*plantarum*	
Z0014_RB_B04_1_D11_B	GREEN	No	118	4	LMG 18021	*Lactobacillus*	*plantarum*	
Z0014_RB_A06_2_D10_B	GREEN	No	118	4	LMG 18021	*Lactobacillus*	*plantarum*	
Z0014_LB_A03_2_D05_A	GREEN	No	118	4	LMG 18021	*Lactobacillus*	*plantarum*	
Z0014_RB_B03_1_D11_A	GREEN	No	118	4	LMG 18021	*Lactobacillus*	*plantarum*	
Z0014_LB_A12_2_D01_B	GREEN	No	118	4	LMG 18021	*Lactobacillus*	*plantarum*	
Z0014_LO_H12_2_H06_B	GREEN	No	118	4	LMG 18021	*Lactobacillus*	*plantarum*	
Z0014_LB_B01_1_D06_A	GREEN	No	118	4	LMG 18021	*Lactobacillus*	*plantarum*	
Z0014_RB_A01_2_D12_A	GREEN	No	118	4	LMG 18021	*Lactobacillus*	*plantarum*	
Z0014_RB_B05_1_D10_A	GREEN	No	118	4	LMG 18021	*Lactobacillus*	*plantarum*	
Z0014_LB_A09_2_D02_A	GREEN	No	118	4	LMG 18021	*Lactobacillus*	*plantarum*	
Z0014_RB_A11_2_D07_A	GREEN	No	118	4	LMG 18021	*Lactobacillus*	*plantarum*	
Z0014_RB_B01_1_D12_A	GREEN	No	118	4	LMG 18021	*Lactobacillus*	*plantarum*	
Z0014_LB_A11_2_D01_A	GREEN	No	118	4	LMG 18021	*Lactobacillus*	*plantarum*	
Z0014_LB_A10_2_D02_B	GREEN	No	118	4	LMG 18021	*Lactobacillus*	*plantarum*	

Z0014_LB_A08_2_D03_B	GREEN	No	118	4	LMG 18021	*Lactobacillus*	*plantarum*	
Z0014_RB_A02_2_D12_B	GREEN	No	118	4	LMG 18021	*Lactobacillus*	*plantarum*	
Z0014_LB_A02_2_D06_B	GREEN	No	118	4	LMG 18021	*Lactobacillus*	*plantarum*	
Z0014_LB_A07_2_D03_A	GREEN	No	118	4	LMG 18021	*Lactobacillus*	*plantarum*	
Z0013_RB_F09_1_B08_A	GREEN	Yes	119	22	LMG 16673	*Lactobacillus*	*paraplantarum*	
Z0013_LB_E12_2_B01_B	GREEN	No	119	22	LMG 16673	*Lactobacillus*	*paraplantarum*	
Z0013_LB_F06_1_B04_B	GREEN	No	119	22	LMG 16673	*Lactobacillus*	*paraplantarum*	
Z0013_LO_F10_2_G05_B	GREEN	No	119	22	LMG 16673	*Lactobacillus*	*paraplantarum*	
Z0013_LB_F01_1_B06_A	GREEN	No	119	22	LMG 16673	*Lactobacillus*	*paraplantarum*	
Z0013_RB_F07_1_B09_A	GREEN	No	119	22	LMG 16673	*Lactobacillus*	*paraplantarum*	
Z0013_LO_F01_2_G01_A	GREEN	No	119	22	LMG 16673	*Lactobacillus*	*paraplantarum*	
Z0013_LB_E10_2_B02_B	GREEN	No	119	22	LMG 16673	*Lactobacillus*	*paraplantarum*	
Z0013_LO_F04_2_G02_B	GREEN	No	119	22	LMG 16673	*Lactobacillus*	*paraplantarum*	
Z0013_LB_E11_2_B01_A	GREEN	No	119	22	LMG 16673	*Lactobacillus*	*paraplantarum*	
Z0013_LO_E12_1_G06_B	GREEN	No	119	22	LMG 16673	*Lactobacillus*	*paraplantarum*	
Z0013_LO_F05_2_G03_A	GREEN	No	119	22	LMG 16673	*Lactobacillus*	*paraplantarum*	
Z0013_LB_F10_1_B02_B	GREEN	No	119	22	LMG 16673	*Lactobacillus*	*paraplantarum*	
Z0013_RB_F08_1_B09_B	GREEN	No	119	22	LMG 16673	*Lactobacillus*	*paraplantarum*	
Z0013_LB_F04_1_B05_B	GREEN	No	119	22	LMG 16673	*Lactobacillus*	*paraplantarum*	
Z0013_RO_F01_2_G07_A	GREEN	No	119	22	LMG 16673	*Lactobacillus*	*paraplantarum*	
Z0013_RB_E10_2_B08_B	GREEN	No	119	22	LMG 16673	*Lactobacillus*	*paraplantarum*	
Z0013_LB_F02_1_B06_B	GREEN	No	119	22	LMG 16673	*Lactobacillus*	*paraplantarum*	
Z0013_RB_E09_2_B08_A	GREEN	No	119	22	LMG 16673	*Lactobacillus*	*paraplantarum*	
Z0013_LB_E09_2_B02_A	GREEN	No	119	22	LMG 16673	*Lactobacillus*	*paraplantarum*	
Z0013_LB_F05_1_B04_A	GREEN	No	119	22	LMG 16673	*Lactobacillus*	*paraplantarum*	
Z0013_LB_F08_1_B03_B	GREEN	No	119	22	LMG 16673	*Lactobacillus*	*paraplantarum*	
Z0013_LB_F09_1_B02_A	GREEN	No	119	22	LMG 16673	*Lactobacillus*	*paraplantarum*	
Z0013_RB_E11_2_B07_A	GREEN	No	119	22	LMG 16673	*Lactobacillus*	*paraplantarum*	
Z0013_RO_E12_1_G12_B	GREEN	No	119	22	LMG 16673	*Lactobacillus*	*paraplantarum*	
Z0013_RB_F04_1_B11_B	GREEN	No	119	22	LMG 16673	*Lactobacillus*	*paraplantarum*	
Z0013_LO_F09_2_G05_A	GREEN	No	119	22	LMG 16673	*Lactobacillus*	*paraplantarum*	
Z0013_LO_F07_2_G04_A	GREEN	No	119	22	LMG 16673	*Lactobacillus*	*paraplantarum*	
Z0013_RB_F01_1_B12_A	GREEN	No	119	22	LMG 16673	*Lactobacillus*	*paraplantarum*	
Z0013_LO_F06_2_G03_B	GREEN	No	119	22	LMG 16673	*Lactobacillus*	*paraplantarum*	
Z0013_LB_F07_1_B03_A	GREEN	No	119	22	LMG 16673	*Lactobacillus*	*paraplantarum*	
Z0013_LB_G01_2_A06_A	GREEN	No	119	22	LMG 16673	*Lactobacillus*	*paraplantarum*	
Z0025_RO_B08_2_E10_B	GREEN	Yes	120	139	LMG 9205	*Lactobacillus*	*plantarum*	*argentoratensis*
Z0024_LO_A05_1_E03_A	GREEN	No	120	4	LMG 6907	*Lactobacillus*	*plantarum*	*plantarum*
Z0025_RO_B09_2_E11_A	GREEN	No	120	139	LMG 9205	*Lactobacillus*	*plantarum*	*argentoratensis*
Z0025_RO_B10_2_E11_B	GREEN	No	120	139	LMG 9205	*Lactobacillus*	*plantarum*	*argentoratensis*
Z0025_RO_C06_1_F09_B	GREEN	No	120	139	LMG 9205	*Lactobacillus*	*plantarum*	*argentoratensis*
Z0025_LO_D07_2_F04_A	GREEN	No	120	139	LMG 9205	*Lactobacillus*	*plantarum*	*argentoratensis*
Z0025_RO_C07_1_F10_A	GREEN	No	120	139	LMG 9205	*Lactobacillus*	*plantarum*	*argentoratensis*
Z0025_RO_C08_1_F10_B	GREEN	No	120	139	LMG 9205	*Lactobacillus*	*plantarum*	*argentoratensis*
Z0014_RO_A11_1_E12_A	GREEN	Yes	121	4	LMG 18404	*Lactobacillus*	*plantarum*	
Z0012_RB_C01_2_C12_A	GREEN	No	121	4	LMG 11405	*Lactobacillus*	*plantarum*	
Z0012_RB_B06_1_D10_B	GREEN	No	121	4	LMG 11405	*Lactobacillus*	*plantarum*	
Z0012_LB_B12_1_D01_B	GREEN	No	121	4	LMG 11405	*Lactobacillus*	*plantarum*	
Z0012_LB_B09_1_D02_A	GREEN	No	121	4	LMG 11405	*Lactobacillus*	*plantarum*	
Z0012_LB_B11_1_D01_A	GREEN	No	121	4	LMG 11405	*Lactobacillus*	*plantarum*	
Z0012_RB_B10_1_D08_B	GREEN	No	121	4	LMG 11405	*Lactobacillus*	*plantarum*	
Z0012_LB_C07_2_C03_A	GREEN	No	121	4	LMG 11405	*Lactobacillus*	*plantarum*	
Z0012_RB_B05_1_D10_A	GREEN	No	121	4	LMG 11405	*Lactobacillus*	*plantarum*	
Z0012_LB_C02_2_C06_B	GREEN	No	121	4	LMG 11405	*Lactobacillus*	*plantarum*	
Z0012_LB_B08_1_D03_B	GREEN	No	121	4	LMG 11405	*Lactobacillus*	*plantarum*	
Z0024_LB_H05_1_A04_A	GREEN	No	121	4	LMG 6907	*Lactobacillus*	*plantarum*	*plantarum*
Z0024_LO_A04_1_E02_B	GREEN	No	121	4	LMG 6907	*Lactobacillus*	*plantarum*	*plantarum*
Z0024_RB_H02_1_A12_B	GREEN	No	121	4	LMG 6907	*Lactobacillus*	*plantarum*	*plantarum*
Z0024_RB_H03_1_A11_A	GREEN	No	121	4	LMG 6907	*Lactobacillus*	*plantarum*	*plantarum*
Z0024_RB_H08_1_A09_B	GREEN	No	121	4	LMG 6907	*Lactobacillus*	*plantarum*	*plantarum*
Z0024_RB_H07_1_A09_A	GREEN	No	121	4	LMG 6907	*Lactobacillus*	*plantarum*	*plantarum*
Z0024_LB_H11_1_A01_A	GREEN	No	121	4	LMG 6907	*Lactobacillus*	*plantarum*	*plantarum*
Z0024_RB_G11_2_A07_A	GREEN	No	121	4	LMG 6907	*Lactobacillus*	*plantarum*	*plantarum*
Z0024_RB_G08_2_A09_B	GREEN	No	121	4	LMG 6907	*Lactobacillus*	*plantarum*	*plantarum*
Z0024_LO_A02_1_E01_B	GREEN	No	121	4	LMG 6907	*Lactobacillus*	*plantarum*	*plantarum*
Z0024_RB_G12_2_A07_B	GREEN	No	121	4	LMG 6907	*Lactobacillus*	*plantarum*	*plantarum*
Z0024_RB_H06_1_A10_B	GREEN	No	121	4	LMG 6907	*Lactobacillus*	*plantarum*	*plantarum*
Z0024_RB_H01_1_A12_A	GREEN	No	121	4	LMG 6907	*Lactobacillus*	*plantarum*	*plantarum*
Z0024_LB_H12_1_A01_B	GREEN	No	121	4	LMG 6907	*Lactobacillus*	*plantarum*	*plantarum*
Z0024_LO_A10_1_E05_B	GREEN	No	121	4	LMG 6907	*Lactobacillus*	*plantarum*	*plantarum*

Z0024_LB_H07_1_A03_A	GREEN	No	121	4	LMG 6907	*Lactobacillus*	*plantarum*	*plantarum*
Z0024_RB_G09_2_A08_A	GREEN	No	121	4	LMG 6907	*Lactobacillus*	*plantarum*	*plantarum*
Z0024_LB_H06_1_A04_B	GREEN	No	121	4	LMG 6907	*Lactobacillus*	*plantarum*	*plantarum*
Z0024_LO_A01_1_E01_A	GREEN	No	121	4	LMG 6907	*Lactobacillus*	*plantarum*	*plantarum*
Z0024_LB_H08_1_A03_B	GREEN	No	121	4	LMG 6907	*Lactobacillus*	*plantarum*	*plantarum*
Z0024_LO_A11_1_E06_A	GREEN	No	121	4	LMG 6907	*Lactobacillus*	*plantarum*	*plantarum*
Z0024_LB_H09_1_A02_A	GREEN	No	121	4	LMG 6907	*Lactobacillus*	*plantarum*	*plantarum*
Z0024_LO_A09_1_E05_A	GREEN	No	121	4	LMG 6907	*Lactobacillus*	*plantarum*	*plantarum*
Z0024_LB_H10_1_A02_B	GREEN	No	121	4	LMG 6907	*Lactobacillus*	*plantarum*	*plantarum*
Z0024_LO_A03_1_E02_A	GREEN	No	121	4	LMG 6907	*Lactobacillus*	*plantarum*	*plantarum*
Z0024_RB_H05_1_A10_A	GREEN	No	121	4	LMG 6907	*Lactobacillus*	*plantarum*	*plantarum*
Z0024_LO_A06_1_E03_B	GREEN	No	121	4	LMG 6907	*Lactobacillus*	*plantarum*	*plantarum*
Z0024_LO_A07_1_E04_A	GREEN	No	121	4	LMG 6907	*Lactobacillus*	*plantarum*	*plantarum*
Z0024_RB_G10_2_A08_B	GREEN	No	121	4	LMG 6907	*Lactobacillus*	*plantarum*	*plantarum*
Z0024_RB_H04_1_A11_B	GREEN	No	121	4	LMG 6907	*Lactobacillus*	*plantarum*	*plantarum*
Z0024_LO_A08_1_E04_B	GREEN	No	121	4	LMG 6907	*Lactobacillus*	*plantarum*	*plantarum*
Z0025_RO_B06_2_E09_B	GREEN	No	121	139	LMG 9205	*Lactobacillus*	*plantarum*	*argentoratensis*
Z0025_RO_C02_1_F07_B	GREEN	No	121	139	LMG 9205	*Lactobacillus*	*plantarum*	*argentoratensis*
Z0025_RO_B12_2_E12_B	GREEN	No	121	139	LMG 9205	*Lactobacillus*	*plantarum*	*argentoratensis*
Z0025_LO_D11_2_F06_A	GREEN	No	121	139	LMG 9205	*Lactobacillus*	*plantarum*	*argentoratensis*
Z0025_LO_D12_2_F06_B	GREEN	No	121	139	LMG 9205	*Lactobacillus*	*plantarum*	*argentoratensis*
Z0025_LO_D09_2_F05_A	GREEN	No	121	139	LMG 9205	*Lactobacillus*	*plantarum*	*argentoratensis*
Z0025_LO_D03_2_F02_A	GREEN	No	121	139	LMG 9205	*Lactobacillus*	*plantarum*	*argentoratensis*
Z0025_LO_D02_2_F01_B	GREEN	No	121	139	LMG 9205	*Lactobacillus*	*plantarum*	*argentoratensis*
Z0025_RO_C01_1_F07_A	GREEN	No	121	139	LMG 9205	*Lactobacillus*	*plantarum*	*argentoratensis*
Z0025_RO_B11_2_E12_A	GREEN	No	121	139	LMG 9205	*Lactobacillus*	*plantarum*	*argentoratensis*
Z0025_RO_C04_1_F08_B	GREEN	No	121	139	LMG 9205	*Lactobacillus*	*plantarum*	*argentoratensis*
Z0025_RO_C09_1_F11_A	GREEN	No	121	139	LMG 9205	*Lactobacillus*	*plantarum*	*argentoratensis*
Z0025_RO_C11_1_F12_A	GREEN	No	121	139	LMG 9205	*Lactobacillus*	*plantarum*	*argentoratensis*
Z0025_LO_C07_1_F04_A	GREEN	No	121	139	LMG 9205	*Lactobacillus*	*plantarum*	*argentoratensis*
Z0025_LO_C05_1_F03_A	GREEN	No	121	139	LMG 9205	*Lactobacillus*	*plantarum*	*argentoratensis*
Z0025_RO_B07_2_E10_A	GREEN	No	121	139	LMG 9205	*Lactobacillus*	*plantarum*	*argentoratensis*
Z0025_LO_C12_1_F06_B	GREEN	No	121	139	LMG 9205	*Lactobacillus*	*plantarum*	*argentoratensis*
Z0025_RO_B05_2_E09_A	GREEN	No	121	139	LMG 9205	*Lactobacillus*	*plantarum*	*argentoratensis*
Z0025_LO_C03_1_F02_A	GREEN	No	121	139	LMG 9205	*Lactobacillus*	*plantarum*	*argentoratensis*
Z0025_RO_C03_1_F08_A	GREEN	No	121	139	LMG 9205	*Lactobacillus*	*plantarum*	*argentoratensis*
Z0025_LO_C02_1_F01_B	GREEN	No	121	139	LMG 9205	*Lactobacillus*	*plantarum*	*argentoratensis*
Z0018_LO_G02_1_H01_B	GREEN	No	121	4	LMG 24832	*Lactobacillus*	*plantarum*	
Z0018_RO_D04_2_F08_B	GREEN	No	121	4	LMG 24832	*Lactobacillus*	*plantarum*	
Z0018_RO_E08_1_G10_B	GREEN	No	121	4	LMG 24832	*Lactobacillus*	*plantarum*	
Z0018_RO_F03_2_G08_A	GREEN	No	121	4	LMG 24832	*Lactobacillus*	*plantarum*	
Z0018_RO_D10_2_F11_B	GREEN	No	121	4	LMG 24832	*Lactobacillus*	*plantarum*	
Z0018_RO_D08_2_F10_B	GREEN	No	121	4	LMG 24832	*Lactobacillus*	*plantarum*	
Z0018_RO_D09_2_F11_A	GREEN	No	121	4	LMG 24832	*Lactobacillus*	*plantarum*	
Z0018_LO_F05_2_G03_A	GREEN	No	121	4	LMG 24832	*Lactobacillus*	*plantarum*	
Z0018_LO_G03_1_H02_A	GREEN	No	121	4	LMG 24832	*Lactobacillus*	*plantarum*	
Z0018_LO_G01_1_H01_A	GREEN	No	121	4	LMG 24832	*Lactobacillus*	*plantarum*	
Z0018_RO_F02_2_G07_B	GREEN	No	121	4	LMG 24832	*Lactobacillus*	*plantarum*	
Z0018_RO_E07_1_G10_A	GREEN	No	121	4	LMG 24832	*Lactobacillus*	*plantarum*	
Z0018_RO_E11_1_G12_A	GREEN	No	121	4	LMG 24832	*Lactobacillus*	*plantarum*	
Z0018_RO_D07_2_F10_A	GREEN	No	121	4	LMG 24832	*Lactobacillus*	*plantarum*	
Z0018_RO_D12_2_F12_B	GREEN	No	121	4	LMG 24832	*Lactobacillus*	*plantarum*	
Z0018_RO_D11_2_F12_A	GREEN	No	121	4	LMG 24832	*Lactobacillus*	*plantarum*	
Z0018_RO_D05_2_F09_A	GREEN	No	121	4	LMG 24832	*Lactobacillus*	*plantarum*	
Z0018_LO_F06_2_G03_B	GREEN	No	121	4	LMG 24832	*Lactobacillus*	*plantarum*	
Z0018_RO_D06_2_F09_B	GREEN	No	121	4	LMG 24832	*Lactobacillus*	*plantarum*	
Z0018_LO_G10_1_H05_B	GREEN	No	121	4	LMG 24832	*Lactobacillus*	*plantarum*	
Z0018_RO_F08_2_G10_B	GREEN	No	121	4	LMG 24832	*Lactobacillus*	*plantarum*	
Z0018_LO_F07_2_G04_A	GREEN	No	121	4	LMG 24832	*Lactobacillus*	*plantarum*	
Z0018_RO_F07_2_G10_A	GREEN	No	121	4	LMG 24832	*Lactobacillus*	*plantarum*	
Z0018_LO_F12_2_G06_B	GREEN	No	121	4	LMG 24832	*Lactobacillus*	*plantarum*	
Z0018_LO_G05_1_H03_A	GREEN	No	121	4	LMG 24832	*Lactobacillus*	*plantarum*	
Z0018_RO_E03_1_G08_A	GREEN	No	121	4	LMG 24832	*Lactobacillus*	*plantarum*	
Z0018_LO_G06_1_H03_B	GREEN	No	121	4	LMG 24832	*Lactobacillus*	*plantarum*	
Z0018_RO_D02_2_F07_B	GREEN	No	121	4	LMG 24832	*Lactobacillus*	*plantarum*	
Z0018_LO_F11_2_G06_A	GREEN	No	121	4	LMG 24832	*Lactobacillus*	*plantarum*	
Z0018_RO_C10_1_F11_B	GREEN	No	121	4	LMG 24832	*Lactobacillus*	*plantarum*	
Z0018_RO_D01_2_F07_A	GREEN	No	121	4	LMG 24832	*Lactobacillus*	*plantarum*	
Z0020_LB_F05_1_B04_A	GREEN	No	121	4	LMG 26367	*Lactobacillus*	*plantarum*	
Z0020_LB_F09_1_B02_A	GREEN	No	121	4	LMG 26367	*Lactobacillus*	*plantarum*	

Z0020_RB_E11_2_B07_A	GREEN	No	121	4	LMG 26367	*Lactobacillus*	*plantarum*
Z0020_RB_E09_2_B08_A	GREEN	No	121	4	LMG 26367	*Lactobacillus*	*plantarum*
Z0020_LB_E12_2_B01_B	GREEN	No	121	4	LMG 26367	*Lactobacillus*	*plantarum*
Z0020_LB_E11_2_B01_A	GREEN	No	121	4	LMG 26367	*Lactobacillus*	*plantarum*
Z0020_LB_F04_1_B05_B	GREEN	No	121	4	LMG 26367	*Lactobacillus*	*plantarum*
Z0020_RB_E05_2_B10_A	GREEN	No	121	4	LMG 26367	*Lactobacillus*	*plantarum*
Z0020_LB_F12_1_B01_B	GREEN	No	121	4	LMG 26367	*Lactobacillus*	*plantarum*
Z0020_LB_F01_1_B06_A	GREEN	No	121	4	LMG 26367	*Lactobacillus*	*plantarum*
Z0020_RB_E04_2_B11_B	GREEN	No	121	4	LMG 26367	*Lactobacillus*	*plantarum*
Z0020_RB_E07_2_B09_A	GREEN	No	121	4	LMG 26367	*Lactobacillus*	*plantarum*
Z0020_LB_E07_2_B03_A	GREEN	No	121	4	LMG 26367	*Lactobacillus*	*plantarum*
Z0020_LB_F07_1_B03_A	GREEN	No	121	4	LMG 26367	*Lactobacillus*	*plantarum*
Z0020_LB_E08_2_B03_B	GREEN	No	121	4	LMG 26367	*Lactobacillus*	*plantarum*
Z0020_LB_F11_1_B01_A	GREEN	No	121	4	LMG 26367	*Lactobacillus*	*plantarum*
Z0020_LB_F10_1_B02_B	GREEN	No	121	4	LMG 26367	*Lactobacillus*	*plantarum*
Z0020_RB_F02_1_B12_B	GREEN	No	121	4	LMG 26367	*Lactobacillus*	*plantarum*
Z0020_LB_F02_1_B06_B	GREEN	No	121	4	LMG 26367	*Lactobacillus*	*plantarum*
Z0020_RB_E10_2_B08_B	GREEN	No	121	4	LMG 26367	*Lactobacillus*	*plantarum*
Z0020_LB_G01_2_A06_A	GREEN	No	121	4	LMG 26367	*Lactobacillus*	*plantarum*
Z0020_RB_E08_2_B09_B	GREEN	No	121	4	LMG 26367	*Lactobacillus*	*plantarum*
Z0020_RB_F04_1_B11_B	GREEN	No	121	4	LMG 26367	*Lactobacillus*	*plantarum*
Z0020_LB_E09_2_B02_A	GREEN	No	121	4	LMG 26367	*Lactobacillus*	*plantarum*
Z0020_RB_E12_2_B07_B	GREEN	No	121	4	LMG 26367	*Lactobacillus*	*plantarum*
Z0020_LB_E10_2_B02_B	GREEN	No	121	4	LMG 26367	*Lactobacillus*	*plantarum*
Z0020_LB_F06_1_B04_B	GREEN	No	121	4	LMG 26367	*Lactobacillus*	*plantarum*
Z0014_LO_A01_1_E01_A	GREEN	No	121	4	LMG 18404	*Lactobacillus*	*plantarum*
Z0014_LO_A05_1_E03_A	GREEN	No	121	4	LMG 18404	*Lactobacillus*	*plantarum*
Z0014_LB_H11_1_A01_A	GREEN	No	121	4	LMG 18404	*Lactobacillus*	*plantarum*
Z0014_LB_H12_1_A01_B	GREEN	No	121	4	LMG 18404	*Lactobacillus*	*plantarum*
Z0014_RO_B05_2_E09_A	GREEN	No	121	4	LMG 18404	*Lactobacillus*	*plantarum*
Z0014_RO_B01_2_E07_A	GREEN	No	121	4	LMG 18404	*Lactobacillus*	*plantarum*
Z0014_LO_A06_1_E03_B	GREEN	No	121	4	LMG 18404	*Lactobacillus*	*plantarum*
Z0014_LO_A03_1_E02_A	GREEN	No	121	4	LMG 18404	*Lactobacillus*	*plantarum*
Z0014_RO_B03_2_E08_A	GREEN	No	121	4	LMG 18404	*Lactobacillus*	*plantarum*
Z0014_LB_H06_1_A04_B	GREEN	No	121	4	LMG 18404	*Lactobacillus*	*plantarum*
Z0014_LO_A04_1_E02_B	GREEN	No	121	4	LMG 18404	*Lactobacillus*	*plantarum*
Z0014_RO_A12_1_E12_B	GREEN	No	121	4	LMG 18404	*Lactobacillus*	*plantarum*
Z0014_RO_B04_2_E08_B	GREEN	No	121	4	LMG 18404	*Lactobacillus*	*plantarum*
Z0014_RO_B06_2_E09_B	GREEN	No	121	4	LMG 18404	*Lactobacillus*	*plantarum*
Z0014_LO_A08_1_E04_B	GREEN	No	121	4	LMG 18404	*Lactobacillus*	*plantarum*
Z0014_RO_B08_2_E10_B	GREEN	No	121	4	LMG 18404	*Lactobacillus*	*plantarum*
Z0014_RO_A08_1_E10_B	GREEN	No	121	4	LMG 18404	*Lactobacillus*	*plantarum*
Z0014_LO_A07_1_E04_A	GREEN	No	121	4	LMG 18404	*Lactobacillus*	*plantarum*
Z0014_LB_H07_1_A03_A	GREEN	No	121	4	LMG 18404	*Lactobacillus*	*plantarum*
Z0014_LB_H10_1_A02_B	GREEN	No	121	4	LMG 18404	*Lactobacillus*	*plantarum*
Z0014_LO_A02_1_E01_B	GREEN	No	121	4	LMG 18404	*Lactobacillus*	*plantarum*
Z0014_RO_B02_2_E07_B	GREEN	No	121	4	LMG 18404	*Lactobacillus*	*plantarum*
Z0014_LB_H09_1_A02_A	GREEN	No	121	4	LMG 18404	*Lactobacillus*	*plantarum*
Z0014_RO_A09_1_E11_A	GREEN	No	121	4	LMG 18404	*Lactobacillus*	*plantarum*
Z0014_LB_H08_1_A03_B	GREEN	No	121	4	LMG 18404	*Lactobacillus*	*plantarum*
Z0014_RO_A10_1_E11_B	GREEN	No	121	4	LMG 18404	*Lactobacillus*	*plantarum*
Z0014_RO_B07_2_E10_A	GREEN	No	121	4	LMG 18404	*Lactobacillus*	*plantarum*
Z0020_RB_F01_1_B12_A	GREEN	Yes	122	4	LMG 26367	*Lactobacillus*	*plantarum*
Z0012_LB_B10_1_D02_B	GREEN	No	122	4	LMG 11405	*Lactobacillus*	*plantarum*
Z0012_RB_B11_1_D07_A	GREEN	No	122	4	LMG 11405	*Lactobacillus*	*plantarum*
Z0020_RB_E06_2_B10_B	GREEN	No	122	4	LMG 26367	*Lactobacillus*	*plantarum*
Z0020_RB_F03_1_B11_A	GREEN	No	122	4	LMG 26367	*Lactobacillus*	*plantarum*
Z0020_LB_F03_1_B05_A	GREEN	No	122	4	LMG 26367	*Lactobacillus*	*plantarum*
Z0020_LB_F08_1_B03_B	GREEN	No	122	4	LMG 26367	*Lactobacillus*	*plantarum*
Z0013_RB_D03_1_C11_A	GREEN	Yes	123	19	LMG 15863	*Haemophilus*	*influenzae*
Z0013_RO_D07_2_F10_A	GREEN	No	123	19	LMG 15863	*Haemophilus*	*influenzae*
Z0013_LO_D01_2_F01_A	GREEN	No	123	19	LMG 15863	*Haemophilus*	*influenzae*
Z0013_RB_C11_2_C07_A	GREEN	No	123	19	LMG 15863	*Haemophilus*	*influenzae*
Z0013_RB_C12_2_C07_B	GREEN	No	123	19	LMG 15863	*Haemophilus*	*influenzae*
Z0013_RB_C09_2_C08_A	GREEN	No	123	19	LMG 15863	*Haemophilus*	*influenzae*
Z0013_RB_C10_2_C08_B	GREEN	No	123	19	LMG 15863	*Haemophilus*	*influenzae*
Z0013_LO_D05_2_F03_A	GREEN	No	123	19	LMG 15863	*Haemophilus*	*influenzae*
Z0013_LB_D07_1_C03_A	GREEN	No	123	19	LMG 15863	*Haemophilus*	*influenzae*
Z0013_RB_D04_1_C11_B	GREEN	No	123	19	LMG 15863	*Haemophilus*	*influenzae*
Z0013_LO_D02_2_F01_B	GREEN	No	123	19	LMG 15863	*Haemophilus*	*influenzae*

Z0013_RO_D09_2_F11_A	GREEN	No	123	19	LMG 15863	*Haemophilus*	*influenzae*
Z0013_RO_D08_2_F10_B	GREEN	No	123	19	LMG 15863	*Haemophilus*	*influenzae*
Z0013_LO_D06_2_F03_B	GREEN	No	123	19	LMG 15863	*Haemophilus*	*influenzae*
Z0013_LB_C11_2_C01_A	GREEN	No	123	19	LMG 15863	*Haemophilus*	*influenzae*
Z0013_RO_D05_2_F09_A	GREEN	No	123	19	LMG 15863	*Haemophilus*	*influenzae*
Z0013_RB_D01_1_C12_A	GREEN	No	123	19	LMG 15863	*Haemophilus*	*influenzae*
Z0013_LO_D03_2_F02_A	GREEN	No	123	19	LMG 15863	*Haemophilus*	*influenzae*
Z0013_LB_C12_2_C01_B	GREEN	No	123	19	LMG 15863	*Haemophilus*	*influenzae*
Z0013_LB_D01_1_C06_A	GREEN	No	123	19	LMG 15863	*Haemophilus*	*influenzae*
Z0013_LB_C10_2_C02_B	GREEN	No	123	19	LMG 15863	*Haemophilus*	*influenzae*
Z0013_LB_D03_1_C05_A	GREEN	No	123	19	LMG 15863	*Haemophilus*	*influenzae*
Z0013_LB_D06_1_C04_B	GREEN	No	123	19	LMG 15863	*Haemophilus*	*influenzae*
Z0013_RB_D06_1_C10_B	GREEN	No	123	19	LMG 15863	*Haemophilus*	*influenzae*
Z0013_LB_D02_1_C06_B	GREEN	No	123	19	LMG 15863	*Haemophilus*	*influenzae*
Z0014_LO_B01_2_E01_A	GREEN	Yes	124	28	LMG 18919	*Microvirgula*	*aerodenitrificans*
Z0014_RO_B10_2_E11_B	GREEN	No	124	28	LMG 18919	*Microvirgula*	*aerodenitrificans*
Z0014_LO_B09_2_E05_A	GREEN	No	124	28	LMG 18919	*Microvirgula*	*aerodenitrificans*
Z0014_LO_B05_2_E03_A	GREEN	No	124	28	LMG 18919	*Microvirgula*	*aerodenitrificans*
Z0014_LO_B10_2_E05_B	GREEN	No	124	28	LMG 18919	*Microvirgula*	*aerodenitrificans*
Z0014_LO_B08_2_E04_B	GREEN	No	124	28	LMG 18919	*Microvirgula*	*aerodenitrificans*
Z0014_LO_A12_1_E06_B	GREEN	No	124	28	LMG 18919	*Microvirgula*	*aerodenitrificans*
Z0014_RO_B11_2_E12_A	GREEN	No	124	28	LMG 18919	*Microvirgula*	*aerodenitrificans*
Z0014_LO_B11_2_E06_A	GREEN	No	124	28	LMG 18919	*Microvirgula*	*aerodenitrificans*
Z0014_RO_C03_1_F08_A	GREEN	No	124	28	LMG 18919	*Microvirgula*	*aerodenitrificans*
Z0014_LO_B02_2_E01_B	GREEN	No	124	28	LMG 18919	*Microvirgula*	*aerodenitrificans*
Z0014_RO_B12_2_E12_B	GREEN	No	124	28	LMG 18919	*Microvirgula*	*aerodenitrificans*
Z0014_LO_B03_2_E02_A	GREEN	No	124	28	LMG 18919	*Microvirgula*	*aerodenitrificans*
Z0014_RO_C04_1_F08_B	GREEN	No	124	28	LMG 18919	*Microvirgula*	*aerodenitrificans*
Z0014_LO_A11_1_E06_A	GREEN	No	124	28	LMG 18919	*Microvirgula*	*aerodenitrificans*
Z0014_LO_B04_2_E02_B	GREEN	No	124	28	LMG 18919	*Microvirgula*	*aerodenitrificans*
Z0014_LO_A10_1_E05_B	GREEN	No	124	28	LMG 18919	*Microvirgula*	*aerodenitrificans*
Z0014_LO_C02_1_F01_B	GREEN	No	124	28	LMG 18919	*Microvirgula*	*aerodenitrificans*
Z0014_LO_C01_1_F01_A	GREEN	No	124	28	LMG 18919	*Microvirgula*	*aerodenitrificans*
Z0014_RO_B09_2_E11_A	GREEN	No	124	28	LMG 18919	*Microvirgula*	*aerodenitrificans*
Z0014_LO_C05_1_F03_A	GREEN	No	124	28	LMG 18919	*Microvirgula*	*aerodenitrificans*
Z0014_RO_C01_1_F07_A	GREEN	No	124	28	LMG 18919	*Microvirgula*	*aerodenitrificans*
Z0014_LO_B12_2_E06_B	GREEN	No	124	28	LMG 18919	*Microvirgula*	*aerodenitrificans*
Z0014_LO_B07_2_E04_A	GREEN	No	124	28	LMG 18919	*Microvirgula*	*aerodenitrificans*
Z0014_LO_A09_1_E05_A	GREEN	No	124	28	LMG 18919	*Microvirgula*	*aerodenitrificans*
Z0014_LO_C04_1_F02_B	GREEN	No	124	28	LMG 18919	*Microvirgula*	*aerodenitrificans*
Z0014_RO_C02_1_F07_B	GREEN	No	124	28	LMG 18919	*Microvirgula*	*aerodenitrificans*
Z0014_LO_B06_2_E03_B	GREEN	No	124	28	LMG 18919	*Microvirgula*	*aerodenitrificans*
Z0023_LO_H11_2_H06_A	GREEN	Yes	125	122	LMG 5743	*Xanthomonas*	*populi*
Z0023_LO_H05_2_H03_A	GREEN	No	125	122	LMG 5743	*Xanthomonas*	*populi*
Z0023_RO_H03_2_H08_A	GREEN	No	125	122	LMG 5743	*Xanthomonas*	*populi*
Z0023_RO_F10_2_G11_B	GREEN	No	125	122	LMG 5743	*Xanthomonas*	*populi*
Z0023_RO_F11_2_G12_A	GREEN	No	125	122	LMG 5743	*Xanthomonas*	*populi*
Z0023_RO_G01_1_H07_A	GREEN	No	125	122	LMG 5743	*Xanthomonas*	*populi*
Z0023_RO_H02_2_H07_B	GREEN	No	125	122	LMG 5743	*Xanthomonas*	*populi*
Z0023_RO_G11_1_H12_A	GREEN	No	125	122	LMG 5743	*Xanthomonas*	*populi*
Z0023_LO_G10_1_H05_B	GREEN	No	125	122	LMG 5743	*Xanthomonas*	*populi*
Z0023_LO_H06_2_H03_B	GREEN	No	125	122	LMG 5743	*Xanthomonas*	*populi*
Z0023_RO_G06_1_H09_B	GREEN	No	125	122	LMG 5743	*Xanthomonas*	*populi*
Z0023_RO_G12_1_H12_B	GREEN	No	125	122	LMG 5743	*Xanthomonas*	*populi*
Z0023_LO_H09_2_H05_A	GREEN	No	125	122	LMG 5743	*Xanthomonas*	*populi*
Z0023_RO_G04_1_H08_B	GREEN	No	125	122	LMG 5743	*Xanthomonas*	*populi*
Z0023_RO_G02_1_H07_B	GREEN	No	125	122	LMG 5743	*Xanthomonas*	*populi*
Z0023_RO_G05_1_H09_A	GREEN	No	125	122	LMG 5743	*Xanthomonas*	*populi*
Z0023_RO_H04_2_H08_B	GREEN	No	125	122	LMG 5743	*Xanthomonas*	*populi*
Z0023_RO_F12_2_G12_B	GREEN	No	125	122	LMG 5743	*Xanthomonas*	*populi*
Z0023_LO_H08_2_H04_B	GREEN	No	125	122	LMG 5743	*Xanthomonas*	*populi*
Z0023_LO_H02_2_H01_B	GREEN	No	125	122	LMG 5743	*Xanthomonas*	*populi*
Z0023_LO_H01_2_H01_A	GREEN	No	125	122	LMG 5743	*Xanthomonas*	*populi*
Z0023_LO_G11_1_H06_A	GREEN	No	125	122	LMG 5743	*Xanthomonas*	*populi*
Z0023_LO_G12_1_H06_B	GREEN	No	125	122	LMG 5743	*Xanthomonas*	*populi*
Z0023_LO_H03_2_H02_A	GREEN	No	125	122	LMG 5743	*Xanthomonas*	*populi*
Z0023_LO_G04_1_H02_B	GREEN	No	125	122	LMG 5743	*Xanthomonas*	*populi*
Z0023_LO_H07_2_H04_A	GREEN	No	125	122	LMG 5743	*Xanthomonas*	*populi*
Z0023_LO_H04_2_H02_B	GREEN	No	125	122	LMG 5743	*Xanthomonas*	*populi*
Z0023_LO_G03_1_H02_A	GREEN	No	125	122	LMG 5743	*Xanthomonas*	*populi*

Z0023_RO_F09_2_G11_A	GREEN	No	125	122	LMG 5743	*Xanthomonas*	*populi*
Z0023_LO_H10_2_H05_B	GREEN	No	125	122	LMG 5743	*Xanthomonas*	*populi*
Z0023_RO_G10_1_H11_B	GREEN	No	125	122	LMG 5743	*Xanthomonas*	*populi*
Z0023_RO_H01_2_H07_A	GREEN	No	125	122	LMG 5743	*Xanthomonas*	*populi*
Z0011_LO_D02_2_F01_B	GREEN	Yes	126	2	LMG 11039	*Bifidobacterium*	*angulatum*
Z0011_RO_D01_2_F07_A	GREEN	No	126	2	LMG 11039	*Bifidobacterium*	*angulatum*
Z0011_RO_C12_1_F12_B	GREEN	No	126	2	LMG 11039	*Bifidobacterium*	*angulatum*
Z0011_LO_E02_1_G01_B	GREEN	No	126	2	LMG 11039	*Bifidobacterium*	*angulatum*
Z0011_RO_B10_2_E11_B	GREEN	No	126	2	LMG 11039	*Bifidobacterium*	*angulatum*
Z0011_LO_E04_1_G02_B	GREEN	No	126	2	LMG 11039	*Bifidobacterium*	*angulatum*
Z0011_RO_C04_1_F08_B	GREEN	No	126	2	LMG 11039	*Bifidobacterium*	*angulatum*
Z0011_RO_B06_2_E09_B	GREEN	No	126	2	LMG 11039	*Bifidobacterium*	*angulatum*
Z0011_LO_D09_2_F05_A	GREEN	No	126	2	LMG 11039	*Bifidobacterium*	*angulatum*
Z0011_LO_D08_2_F04_B	GREEN	No	126	2	LMG 11039	*Bifidobacterium*	*angulatum*
Z0011_RO_C05_1_F09_A	GREEN	No	126	2	LMG 11039	*Bifidobacterium*	*angulatum*
Z0011_RO_E01_1_G07_A	GREEN	No	126	2	LMG 11039	*Bifidobacterium*	*angulatum*
Z0011_RO_C09_1_F11_A	GREEN	No	126	2	LMG 11039	*Bifidobacterium*	*angulatum*
Z0011_LO_E12_1_G06_B	GREEN	No	126	2	LMG 11039	*Bifidobacterium*	*angulatum*
Z0011_RO_D11_2_F12_A	GREEN	No	126	2	LMG 11039	*Bifidobacterium*	*angulatum*
Z0011_RO_E04_1_G08_B	GREEN	No	126	2	LMG 11039	*Bifidobacterium*	*angulatum*
Z0011_LO_F06_2_G03_B	GREEN	No	126	2	LMG 11039	*Bifidobacterium*	*angulatum*
Z0011_RO_E09_1_G11_A	GREEN	No	126	2	LMG 11039	*Bifidobacterium*	*angulatum*
Z0011_RO_D06_2_F09_B	GREEN	No	126	2	LMG 11039	*Bifidobacterium*	*angulatum*
Z0011_LO_F02_2_G01_B	GREEN	No	126	2	LMG 11039	*Bifidobacterium*	*angulatum*
Z0011_LO_E11_1_G06_A	GREEN	No	126	2	LMG 11039	*Bifidobacterium*	*angulatum*
Z0011_RO_D07_2_F10_A	GREEN	No	126	2	LMG 11039	*Bifidobacterium*	*angulatum*
Z0011_RO_C08_1_F10_B	GREEN	No	126	2	LMG 11039	*Bifidobacterium*	*angulatum*
Z0011_LO_E01_1_G01_A	GREEN	No	126	2	LMG 11039	*Bifidobacterium*	*angulatum*
Z0011_LO_E05_1_G03_A	GREEN	No	126	2	LMG 11039	*Bifidobacterium*	*angulatum*
Z0011_LO_E06_1_G03_B	GREEN	No	126	2	LMG 11039	*Bifidobacterium*	*angulatum*
Z0011_RO_D08_2_F10_B	GREEN	No	126	2	LMG 11039	*Bifidobacterium*	*angulatum*
Z0011_RO_D12_2_F12_B	GREEN	No	126	2	LMG 11039	*Bifidobacterium*	*angulatum*
Z0011_RO_E08_1_G10_B	GREEN	No	126	2	LMG 11039	*Bifidobacterium*	*angulatum*
Z0011_LO_E09_1_G05_A	GREEN	No	126	2	LMG 11039	*Bifidobacterium*	*angulatum*
Z0011_RO_D05_2_F09_A	GREEN	No	126	2	LMG 11039	*Bifidobacterium*	*angulatum*
Z0011_RO_C03_1_F08_A	GREEN	No	126	2	LMG 11039	*Bifidobacterium*	*angulatum*
Z0016_RO_E03_1_G08_A	GREEN	Yes	127	52	LMG 23059	*Blastobacter*	*aggregatus*
Z0016_RO_G04_1_H08_B	GREEN	No	127	52	LMG 23059	*Blastobacter*	*aggregatus*
Z0016_RO_G05_1_H09_A	GREEN	No	127	52	LMG 23059	*Blastobacter*	*aggregatus*
Z0016_LO_F09_2_G05_A	GREEN	No	127	52	LMG 23059	*Blastobacter*	*aggregatus*
Z0016_RO_E10_1_G11_B	GREEN	No	127	52	LMG 23059	*Blastobacter*	*aggregatus*
Z0016_RO_F04_2_G08_B	GREEN	No	127	52	LMG 23059	*Blastobacter*	*aggregatus*
Z0016_RO_E05_1_G09_A	GREEN	No	127	52	LMG 23059	*Blastobacter*	*aggregatus*
Z0016_LO_F01_2_G01_A	GREEN	No	127	52	LMG 23059	*Blastobacter*	*aggregatus*
Z0016_RO_E11_1_G12_A	GREEN	No	127	52	LMG 23059	*Blastobacter*	*aggregatus*
Z0016_LO_E08_1_G04_B	GREEN	No	127	52	LMG 23059	*Blastobacter*	*aggregatus*
Z0016_RO_G02_1_H07_B	GREEN	No	127	52	LMG 23059	*Blastobacter*	*aggregatus*
Z0016_LO_F07_2_G04_A	GREEN	No	127	52	LMG 23059	*Blastobacter*	*aggregatus*
Z0016_LO_G04_1_H02_B	GREEN	No	127	52	LMG 23059	*Blastobacter*	*aggregatus*
Z0016_RO_F07_2_G10_A	GREEN	No	127	52	LMG 23059	*Blastobacter*	*aggregatus*
Z0016_RO_F09_2_G11_A	GREEN	No	127	52	LMG 23059	*Blastobacter*	*aggregatus*
Z0016_RO_F10_2_G11_B	GREEN	No	127	52	LMG 23059	*Blastobacter*	*aggregatus*
Z0016_LO_F12_2_G06_B	GREEN	No	127	52	LMG 23059	*Blastobacter*	*aggregatus*
Z0016_LO_G02_1_H01_B	GREEN	No	127	52	LMG 23059	*Blastobacter*	*aggregatus*
Z0016_RO_F03_2_G08_A	GREEN	No	127	52	LMG 23059	*Blastobacter*	*aggregatus*
Z0016_LO_E04_1_G02_B	GREEN	No	127	52	LMG 23059	*Blastobacter*	*aggregatus*
Z0016_RO_F01_2_G07_A	GREEN	No	127	52	LMG 23059	*Blastobacter*	*aggregatus*
Z0016_LO_G01_1_H01_A	GREEN	No	127	52	LMG 23059	*Blastobacter*	*aggregatus*
Z0016_RO_E04_1_G08_B	GREEN	No	127	52	LMG 23059	*Blastobacter*	*aggregatus*
Z0016_LO_E05_1_G03_A	GREEN	No	127	52	LMG 23059	*Blastobacter*	*aggregatus*
Z0016_LO_E03_1_G02_A	GREEN	No	127	52	LMG 23059	*Blastobacter*	*aggregatus*
Z0016_LO_F03_2_G02_A	GREEN	No	127	52	LMG 23059	*Blastobacter*	*aggregatus*
Z0016_LO_F06_2_G03_B	GREEN	No	127	52	LMG 23059	*Blastobacter*	*aggregatus*
Z0016_LO_E02_1_G01_B	GREEN	No	127	52	LMG 23059	*Blastobacter*	*aggregatus*
Z0016_LO_E10_1_G05_B	GREEN	No	127	52	LMG 23059	*Blastobacter*	*aggregatus*
Z0016_LO_E11_1_G06_A	GREEN	No	127	52	LMG 23059	*Blastobacter*	*aggregatus*
Z0016_RO_E07_1_G10_A	GREEN	No	127	52	LMG 23059	*Blastobacter*	*aggregatus*
Z0016_RO_E08_1_G10_B	GREEN	No	127	52	LMG 23059	*Blastobacter*	*aggregatus*
Z0022_RO_G08_1_H10_B	GREEN	Yes	128	112	LMG 4008	*Flavobacterium*	*aquatile*
Z0022_RO_G05_1_H09_A	GREEN	No	128	112	LMG 4008	*Flavobacterium*	*aquatile*

Z0022_RO_G06_1_H09_B	GREEN	No	128	112	LMG 4008	*Flavobacterium*	*aquatile*
Z0022_LO_G12_1_H06_B	GREEN	No	128	112	LMG 4008	*Flavobacterium*	*aquatile*
Z0022_LO_G08_1_H04_B	GREEN	No	128	112	LMG 4008	*Flavobacterium*	*aquatile*
Z0022_RO_G11_1_H12_A	GREEN	No	128	112	LMG 4008	*Flavobacterium*	*aquatile*
Z0022_RO_H04_2_H08_B	GREEN	No	128	112	LMG 4008	*Flavobacterium*	*aquatile*
Z0022_RO_G04_1_H08_B	GREEN	No	128	112	LMG 4008	*Flavobacterium*	*aquatile*
Z0022_LO_G07_1_H04_A	GREEN	No	128	112	LMG 4008	*Flavobacterium*	*aquatile*
Z0022_RO_G07_1_H10_A	GREEN	No	128	112	LMG 4008	*Flavobacterium*	*aquatile*
Z0022_LO_H06_2_H03_B	GREEN	No	128	112	LMG 4008	*Flavobacterium*	*aquatile*
Z0022_LO_G11_1_H06_A	GREEN	No	128	112	LMG 4008	*Flavobacterium*	*aquatile*
Z0022_LO_G05_1_H03_A	GREEN	No	128	112	LMG 4008	*Flavobacterium*	*aquatile*
Z0022_RO_F07_2_G10_A	GREEN	No	128	112	LMG 4008	*Flavobacterium*	*aquatile*
Z0022_LO_G03_1_H02_A	GREEN	No	128	112	LMG 4008	*Flavobacterium*	*aquatile*
Z0022_LO_H09_2_H05_A	GREEN	No	128	112	LMG 4008	*Flavobacterium*	*aquatile*
Z0022_RO_G02_1_H07_B	GREEN	No	128	112	LMG 4008	*Flavobacterium*	*aquatile*
Z0022_RB_A01_2_D12_A	GREEN	No	128	112	LMG 4008	*Flavobacterium*	*aquatile*
Z0022_RO_F12_2_G12_B	GREEN	No	128	112	LMG 4008	*Flavobacterium*	*aquatile*
Z0022_RO_G01_1_H07_A	GREEN	No	128	112	LMG 4008	*Flavobacterium*	*aquatile*
Z0022_RO_H03_2_H08_A	GREEN	No	128	112	LMG 4008	*Flavobacterium*	*aquatile*
Z0022_RO_H02_2_H07_B	GREEN	No	128	112	LMG 4008	*Flavobacterium*	*aquatile*
Z0022_RO_G12_1_H12_B	GREEN	No	128	112	LMG 4008	*Flavobacterium*	*aquatile*
Z0022_LO_H10_2_H05_B	GREEN	No	128	112	LMG 4008	*Flavobacterium*	*aquatile*
Z0022_LO_H07_2_H04_A	GREEN	No	128	112	LMG 4008	*Flavobacterium*	*aquatile*
Z0022_LO_H12_2_H06_B	GREEN	No	128	112	LMG 4008	*Flavobacterium*	*aquatile*
Z0022_LO_G06_1_H03_B	GREEN	No	128	112	LMG 4008	*Flavobacterium*	*aquatile*
Z0022_RO_H01_2_H07_A	GREEN	No	128	112	LMG 4008	*Flavobacterium*	*aquatile*
Z0022_RO_G10_1_H11_B	GREEN	No	128	112	LMG 4008	*Flavobacterium*	*aquatile*
Z0022_RO_G09_1_H11_A	GREEN	No	128	112	LMG 4008	*Flavobacterium*	*aquatile*
Z0022_LO_H11_2_H06_A	GREEN	No	128	112	LMG 4008	*Flavobacterium*	*aquatile*
Z0022_LO_H08_2_H04_B	GREEN	No	128	112	LMG 4008	*Flavobacterium*	*aquatile*
Z0019_RB_C06_2_C10_B	GREEN	Yes	129	79	LMG 25435	*Marinobacterium*	*coralli*
Z0019_RB_B08_1_D09_B	GREEN	No	129	79	LMG 25435	*Marinobacterium*	*coralli*
Z0019_RB_B07_1_D09_A	GREEN	No	129	79	LMG 25435	*Marinobacterium*	*coralli*
Z0019_LB_D03_1_C05_A	GREEN	No	129	79	LMG 25435	*Marinobacterium*	*coralli*
Z0019_RB_B06_1_D10_B	GREEN	No	129	79	LMG 25435	*Marinobacterium*	*coralli*
Z0019_RB_C10_2_C08_B	GREEN	No	129	79	LMG 25435	*Marinobacterium*	*coralli*
Z0019_RB_C01_2_C12_A	GREEN	No	129	79	LMG 25435	*Marinobacterium*	*coralli*
Z0019_LB_D02_1_C06_B	GREEN	No	129	79	LMG 25435	*Marinobacterium*	*coralli*
Z0019_RB_C09_2_C08_A	GREEN	No	129	79	LMG 25435	*Marinobacterium*	*coralli*
Z0019_RB_B11_1_D07_A	GREEN	No	129	79	LMG 25435	*Marinobacterium*	*coralli*
Z0019_RB_B12_1_D07_B	GREEN	No	129	79	LMG 25435	*Marinobacterium*	*coralli*
Z0019_LB_D06_1_C04_B	GREEN	No	129	79	LMG 25435	*Marinobacterium*	*coralli*
Z0019_RB_B10_1_D08_B	GREEN	No	129	79	LMG 25435	*Marinobacterium*	*coralli*
Z0019_LB_C08_2_C03_B	GREEN	No	129	79	LMG 25435	*Marinobacterium*	*coralli*
Z0019_RB_C05_2_C10_A	GREEN	No	129	79	LMG 25435	*Marinobacterium*	*coralli*
Z0019_RB_B09_1_D08_A	GREEN	No	129	79	LMG 25435	*Marinobacterium*	*coralli*
Z0019_LB_C06_2_C04_B	GREEN	No	129	79	LMG 25435	*Marinobacterium*	*coralli*
Z0019_RB_C08_2_C09_B	GREEN	No	129	79	LMG 25435	*Marinobacterium*	*coralli*
Z0019_LB_D01_1_C06_A	GREEN	No	129	79	LMG 25435	*Marinobacterium*	*coralli*
Z0019_RB_C02_2_C12_B	GREEN	No	129	79	LMG 25435	*Marinobacterium*	*coralli*
Z0019_RB_C07_2_C09_A	GREEN	No	129	79	LMG 25435	*Marinobacterium*	*coralli*
Z0019_LB_C05_2_C04_A	GREEN	No	129	79	LMG 25435	*Marinobacterium*	*coralli*
Z0019_LB_C10_2_C02_B	GREEN	No	129	79	LMG 25435	*Marinobacterium*	*coralli*
Z0019_RB_C11_2_C07_A	GREEN	No	129	79	LMG 25435	*Marinobacterium*	*coralli*
Z0019_LB_C12_2_C01_B	GREEN	No	129	79	LMG 25435	*Marinobacterium*	*coralli*
Z0019_LB_C11_2_C01_A	GREEN	No	129	79	LMG 25435	*Marinobacterium*	*coralli*
Z0019_LB_D05_1_C04_A	GREEN	No	129	79	LMG 25435	*Marinobacterium*	*coralli*
Z0019_LB_D04_1_C05_B	GREEN	No	129	79	LMG 25435	*Marinobacterium*	*coralli*
Z0019_RB_C03_2_C11_A	GREEN	No	129	79	LMG 25435	*Marinobacterium*	*coralli*
Z0019_LB_C09_2_C02_A	GREEN	No	129	79	LMG 25435	*Marinobacterium*	*coralli*
Z0019_RB_C04_2_C11_B	GREEN	No	129	79	LMG 25435	*Marinobacterium*	*coralli*
Z0019_LB_C07_2_C03_A	GREEN	No	129	79	LMG 25435	*Marinobacterium*	*coralli*
Z0016_LO_B09_2_E05_A	GREEN	Yes	130	50	LMG 23003	*Rhodanobacter*	*fulvus*
Z0016_RO_B06_2_E09_B	GREEN	No	130	50	LMG 23003	*Rhodanobacter*	*fulvus*
Z0016_LO_B10_2_E05_B	GREEN	No	130	50	LMG 23003	*Rhodanobacter*	*fulvus*
Z0016_LO_B02_2_E01_B	GREEN	No	130	50	LMG 23003	*Rhodanobacter*	*fulvus*
Z0016_LO_B05_2_E03_A	GREEN	No	130	50	LMG 23003	*Rhodanobacter*	*fulvus*
Z0016_RO_A12_1_E12_B	GREEN	No	130	50	LMG 23003	*Rhodanobacter*	*fulvus*
Z0016_RO_A08_1_E10_B	GREEN	No	130	50	LMG 23003	*Rhodanobacter*	*fulvus*
Z0016_LO_B06_2_E03_B	GREEN	No	130	50	LMG 23003	*Rhodanobacter*	*fulvus*

Z0016_LO_B01_2_E01_A	GREEN	No	130	50	LMG 23003	*Rhodanobacter*	*fulvus*
Z0016_LO_B07_2_E04_A	GREEN	No	130	50	LMG 23003	*Rhodanobacter*	*fulvus*
Z0016_RO_A11_1_E12_A	GREEN	No	130	50	LMG 23003	*Rhodanobacter*	*fulvus*
Z0016_LO_B08_2_E04_B	GREEN	No	130	50	LMG 23003	*Rhodanobacter*	*fulvus*
Z0016_RO_A05_1_E09_A	GREEN	No	130	50	LMG 23003	*Rhodanobacter*	*fulvus*
Z0016_RO_A09_1_E11_A	GREEN	No	130	50	LMG 23003	*Rhodanobacter*	*fulvus*
Z0016_RO_B04_2_E08_B	GREEN	No	130	50	LMG 23003	*Rhodanobacter*	*fulvus*
Z0016_LO_B04_2_E02_B	GREEN	No	130	50	LMG 23003	*Rhodanobacter*	*fulvus*
Z0016_LO_B03_2_E02_A	GREEN	No	130	50	LMG 23003	*Rhodanobacter*	*fulvus*
Z0016_LO_C02_1_F01_B	GREEN	No	130	50	LMG 23003	*Rhodanobacter*	*fulvus*
Z0016_RO_A07_1_E10_A	GREEN	No	130	50	LMG 23003	*Rhodanobacter*	*fulvus*
Z0016_LO_A11_1_E06_A	GREEN	No	130	50	LMG 23003	*Rhodanobacter*	*fulvus*
Z0016_LO_A12_1_E06_B	GREEN	No	130	50	LMG 23003	*Rhodanobacter*	*fulvus*
Z0016_LO_A10_1_E05_B	GREEN	No	130	50	LMG 23003	*Rhodanobacter*	*fulvus*
Z0016_RO_A06_1_E09_B	GREEN	No	130	50	LMG 23003	*Rhodanobacter*	*fulvus*
Z0016_RO_B03_2_E08_A	GREEN	No	130	50	LMG 23003	*Rhodanobacter*	*fulvus*
Z0016_LO_C01_1_F01_A	GREEN	No	130	50	LMG 23003	*Rhodanobacter*	*fulvus*
Z0016_LO_B11_2_E06_A	GREEN	No	130	50	LMG 23003	*Rhodanobacter*	*fulvus*
Z0016_LO_B12_2_E06_B	GREEN	No	130	50	LMG 23003	*Rhodanobacter*	*fulvus*
Z0016_RO_B05_2_E09_A	GREEN	No	130	50	LMG 23003	*Rhodanobacter*	*fulvus*
Z0016_LB_F10_1_B02_B	GREEN	Yes	131	47	LMG 22585	*Gramella*	*echinicola*
Z0016_LB_E11_2_B01_A	GREEN	No	131	47	LMG 22585	*Gramella*	*echinicola*
Z0016_LB_F04_1_B05_B	GREEN	No	131	47	LMG 22585	*Gramella*	*echinicola*
Z0016_LB_F03_1_B05_A	GREEN	No	131	47	LMG 22585	*Gramella*	*echinicola*
Z0016_RB_F09_1_B08_A	GREEN	No	131	47	LMG 22585	*Gramella*	*echinicola*
Z0016_LB_F02_1_B06_B	GREEN	No	131	47	LMG 22585	*Gramella*	*echinicola*
Z0016_RB_F01_1_B12_A	GREEN	No	131	47	LMG 22585	*Gramella*	*echinicola*
Z0016_LB_F12_1_B01_B	GREEN	No	131	47	LMG 22585	*Gramella*	*echinicola*
Z0016_LB_F11_1_B01_A	GREEN	No	131	47	LMG 22585	*Gramella*	*echinicola*
Z0016_RB_F07_1_B09_A	GREEN	No	131	47	LMG 22585	*Gramella*	*echinicola*
Z0016_LB_G02_2_A06_B	GREEN	No	131	47	LMG 22585	*Gramella*	*echinicola*
Z0016_RB_F06_1_B10_B	GREEN	No	131	47	LMG 22585	*Gramella*	*echinicola*
Z0016_LB_F06_1_B04_B	GREEN	No	131	47	LMG 22585	*Gramella*	*echinicola*
Z0016_RB_F03_1_B11_A	GREEN	No	131	47	LMG 22585	*Gramella*	*echinicola*
Z0016_LB_F05_1_B04_A	GREEN	No	131	47	LMG 22585	*Gramella*	*echinicola*
Z0016_RB_E10_2_B08_B	GREEN	No	131	47	LMG 22585	*Gramella*	*echinicola*
Z0016_RB_E12_2_B07_B	GREEN	No	131	47	LMG 22585	*Gramella*	*echinicola*
Z0016_RB_F10_1_B08_B	GREEN	No	131	47	LMG 22585	*Gramella*	*echinicola*
Z0016_LB_E10_2_B02_B	GREEN	No	131	47	LMG 22585	*Gramella*	*echinicola*
Z0016_LB_F01_1_B06_A	GREEN	No	131	47	LMG 22585	*Gramella*	*echinicola*
Z0016_RB_F11_1_B07_A	GREEN	No	131	47	LMG 22585	*Gramella*	*echinicola*
Z0016_RB_E11_2_B07_A	GREEN	No	131	47	LMG 22585	*Gramella*	*echinicola*
Z0016_LB_F08_1_B03_B	GREEN	No	131	47	LMG 22585	*Gramella*	*echinicola*
Z0016_RB_F08_1_B09_B	GREEN	No	131	47	LMG 22585	*Gramella*	*echinicola*
Z0016_RB_F02_1_B12_B	GREEN	No	131	47	LMG 22585	*Gramella*	*echinicola*
Z0016_RB_F04_1_B11_B	GREEN	No	131	47	LMG 22585	*Gramella*	*echinicola*
Z0016_LB_F09_1_B02_A	GREEN	No	131	47	LMG 22585	*Gramella*	*echinicola*
Z0016_RB_E09_2_B08_A	GREEN	No	131	47	LMG 22585	*Gramella*	*echinicola*
Z0016_LB_F07_1_B03_A	GREEN	No	131	47	LMG 22585	*Gramella*	*echinicola*
Z0016_RB_F05_1_B10_A	GREEN	No	131	47	LMG 22585	*Gramella*	*echinicola*
Z0016_LB_G01_2_A06_A	GREEN	No	131	47	LMG 22585	*Gramella*	*echinicola*
Z0016_LB_E12_2_B01_B	GREEN	No	131	47	LMG 22585	*Gramella*	*echinicola*
Z0010_LB_C10_2_C02_B	GREEN	Yes	132	149	R-71006	*Burkholderia*	*multivorans*
Z0010_LB_C12_2_C01_B	GREEN	No	132	149	R-71006	*Burkholderia*	*multivorans*
Z0010_RB_C06_2_C10_B	GREEN	No	132	149	R-71006	*Burkholderia*	*multivorans*
Z0010_LB_D04_1_C05_B	GREEN	No	132	149	R-71006	*Burkholderia*	*multivorans*
Z0010_LB_D09_1_C02_A	GREEN	No	132	149	R-71006	*Burkholderia*	*multivorans*
Z0010_RB_C05_2_C10_A	GREEN	No	132	149	R-71006	*Burkholderia*	*multivorans*
Z0010_LB_C11_2_C01_A	GREEN	No	132	149	R-71006	*Burkholderia*	*multivorans*
Z0010_RB_C09_2_C08_A	GREEN	No	132	149	R-71006	*Burkholderia*	*multivorans*
Z0010_LB_E01_2_B06_A	GREEN	No	132	149	R-71006	*Burkholderia*	*multivorans*
Z0010_RB_C10_2_C08_B	GREEN	No	132	149	R-71006	*Burkholderia*	*multivorans*
Z0010_LB_D05_1_C04_A	GREEN	No	132	149	R-71006	*Burkholderia*	*multivorans*
Z0010_LB_C09_2_C02_A	GREEN	No	132	149	R-71006	*Burkholderia*	*multivorans*
Z0010_LB_D01_1_C06_A	GREEN	No	132	149	R-71006	*Burkholderia*	*multivorans*
Z0010_LB_D11_1_C01_A	GREEN	No	132	149	R-71006	*Burkholderia*	*multivorans*
Z0010_RB_C07_2_C09_A	GREEN	No	132	149	R-71006	*Burkholderia*	*multivorans*
Z0010_LB_D02_1_C06_B	GREEN	No	132	149	R-71006	*Burkholderia*	*multivorans*
Z0010_LB_D10_1_C02_B	GREEN	No	132	149	R-71006	*Burkholderia*	*multivorans*
Z0010_RB_C04_2_C11_B	GREEN	No	132	149	R-71006	*Burkholderia*	*multivorans*

Z0010_LB_E02_2_B06_B	GREEN	No	132	149	R-71006	*Burkholderia*	*multivorans*
Z0010_LB_D12_1_C01_B	GREEN	No	132	149	R-71006	*Burkholderia*	*multivorans*
Z0010_RB_C03_2_C11_A	GREEN	No	132	149	R-71006	*Burkholderia*	*multivorans*
Z0010_LB_D07_1_C03_A	GREEN	No	132	149	R-71006	*Burkholderia*	*multivorans*
Z0010_LB_D03_1_C05_A	GREEN	No	132	149	R-71006	*Burkholderia*	*multivorans*
Z0010_RB_C02_2_C12_B	GREEN	No	132	149	R-71006	*Burkholderia*	*multivorans*
Z0010_RB_C11_2_C07_A	GREEN	No	132	149	R-71006	*Burkholderia*	*multivorans*
Z0010_LB_D08_1_C03_B	GREEN	No	132	149	R-71006	*Burkholderia*	*multivorans*
Z0010_RB_D02_1_C12_B	GREEN	No	132	149	R-71006	*Burkholderia*	*multivorans*
Z0010_RB_D01_1_C12_A	GREEN	No	132	149	R-71006	*Burkholderia*	*multivorans*
Z0010_LB_D06_1_C04_B	GREEN	No	132	149	R-71006	*Burkholderia*	*multivorans*
Z0010_RB_C12_2_C07_B	GREEN	No	132	149	R-71006	*Burkholderia*	*multivorans*
Z0010_RB_C08_2_C09_B	GREEN	No	132	149	R-71006	*Burkholderia*	*multivorans*
Z0010_RB_D03_1_C11_A	GREEN	No	132	149	R-71006	*Burkholderia*	*multivorans*
Z0023_LO_A04_1_E02_B	GREEN	Yes	133	120	LMG 5019	*Serratia*	*rubidaea*
Z0023_LB_H12_1_A01_B	GREEN	No	133	120	LMG 5019	*Serratia*	*rubidaea*
Z0023_RB_G09_2_A08_A	GREEN	No	133	120	LMG 5019	*Serratia*	*rubidaea*
Z0023_LB_H04_1_A05_B	GREEN	No	133	120	LMG 5019	*Serratia*	*rubidaea*
Z0023_LB_H05_1_A04_A	GREEN	No	133	120	LMG 5019	*Serratia*	*rubidaea*
Z0023_LB_H03_1_A05_A	GREEN	No	133	120	LMG 5019	*Serratia*	*rubidaea*
Z0023_LB_H06_1_A04_B	GREEN	No	133	120	LMG 5019	*Serratia*	*rubidaea*
Z0023_LO_A06_1_E03_B	GREEN	No	133	120	LMG 5019	*Serratia*	*rubidaea*
Z0023_LO_A03_1_E02_A	GREEN	No	133	120	LMG 5019	*Serratia*	*rubidaea*
Z0023_LB_H01_1_A06_A	GREEN	No	133	120	LMG 5019	*Serratia*	*rubidaea*
Z0023_RB_G10_2_A08_B	GREEN	No	133	120	LMG 5019	*Serratia*	*rubidaea*
Z0023_RB_G11_2_A07_A	GREEN	No	133	120	LMG 5019	*Serratia*	*rubidaea*
Z0023_RB_F11_1_B07_A	GREEN	No	133	120	LMG 5019	*Serratia*	*rubidaea*
Z0023_LO_A02_1_E01_B	GREEN	No	133	120	LMG 5019	*Serratia*	*rubidaea*
Z0023_LB_H02_1_A06_B	GREEN	No	133	120	LMG 5019	*Serratia*	*rubidaea*
Z0023_LO_A05_1_E03_A	GREEN	No	133	120	LMG 5019	*Serratia*	*rubidaea*
Z0023_RB_G07_2_A09_A	GREEN	No	133	120	LMG 5019	*Serratia*	*rubidaea*
Z0023_RB_G03_2_A11_A	GREEN	No	133	120	LMG 5019	*Serratia*	*rubidaea*
Z0023_RB_G02_2_A12_B	GREEN	No	133	120	LMG 5019	*Serratia*	*rubidaea*
Z0023_RB_F12_1_B07_B	GREEN	No	133	120	LMG 5019	*Serratia*	*rubidaea*
Z0023_RB_G08_2_A09_B	GREEN	No	133	120	LMG 5019	*Serratia*	*rubidaea*
Z0023_LO_A01_1_E01_A	GREEN	No	133	120	LMG 5019	*Serratia*	*rubidaea*
Z0023_RB_F10_1_B08_B	GREEN	No	133	120	LMG 5019	*Serratia*	*rubidaea*
Z0023_RB_G05_2_A10_A	GREEN	No	133	120	LMG 5019	*Serratia*	*rubidaea*
Z0023_LB_H11_1_A01_A	GREEN	No	133	120	LMG 5019	*Serratia*	*rubidaea*
Z0023_RB_G01_2_A12_A	GREEN	No	133	120	LMG 5019	*Serratia*	*rubidaea*
Z0023_LB_H09_1_A02_A	GREEN	No	133	120	LMG 5019	*Serratia*	*rubidaea*
Z0023_LB_H08_1_A03_B	GREEN	No	133	120	LMG 5019	*Serratia*	*rubidaea*
Z0023_RB_G04_2_A11_B	GREEN	No	133	120	LMG 5019	*Serratia*	*rubidaea*
Z0023_LB_H10_1_A02_B	GREEN	No	133	120	LMG 5019	*Serratia*	*rubidaea*
Z0023_LB_H07_1_A03_A	GREEN	No	133	120	LMG 5019	*Serratia*	*rubidaea*
Z0023_RB_G06_2_A10_B	GREEN	No	133	120	LMG 5019	*Serratia*	*rubidaea*
Z0021_LO_C08_1_F04_B	GREEN	Yes	134	104	LMG 28216	*Formosa*	*algae*
Z0021_LO_B12_2_E06_B	GREEN	No	134	104	LMG 28216	*Formosa*	*algae*
Z0021_RO_C04_1_F08_B	GREEN	No	134	104	LMG 28216	*Formosa*	*algae*
Z0021_RO_B12_2_E12_B	GREEN	No	134	104	LMG 28216	*Formosa*	*algae*
Z0021_LO_C01_1_F01_A	GREEN	No	134	104	LMG 28216	*Formosa*	*algae*
Z0021_LO_B01_2_E01_A	GREEN	No	134	104	LMG 28216	*Formosa*	*algae*
Z0021_RO_C06_1_F09_B	GREEN	No	134	104	LMG 28216	*Formosa*	*algae*
Z0021_RO_C07_1_F10_A	GREEN	No	134	104	LMG 28216	*Formosa*	*algae*
Z0021_LO_C03_1_F02_A	GREEN	No	134	104	LMG 28216	*Formosa*	*algae*
Z0021_RO_B02_2_E07_B	GREEN	No	134	104	LMG 28216	*Formosa*	*algae*
Z0021_RO_C02_1_F07_B	GREEN	No	134	104	LMG 28216	*Formosa*	*algae*
Z0021_LO_C05_1_F03_A	GREEN	No	134	104	LMG 28216	*Formosa*	*algae*
Z0021_LO_B10_2_E05_B	GREEN	No	134	104	LMG 28216	*Formosa*	*algae*
Z0021_LO_A12_1_E06_B	GREEN	No	134	104	LMG 28216	*Formosa*	*algae*
Z0021_LO_A11_1_E06_A	GREEN	No	134	104	LMG 28216	*Formosa*	*algae*
Z0021_RO_B06_2_E09_B	GREEN	No	134	104	LMG 28216	*Formosa*	*algae*
Z0021_RO_B03_2_E08_A	GREEN	No	134	104	LMG 28216	*Formosa*	*algae*
Z0021_RO_B07_2_E10_A	GREEN	No	134	104	LMG 28216	*Formosa*	*algae*
Z0021_LO_B08_2_E04_B	GREEN	No	134	104	LMG 28216	*Formosa*	*algae*
Z0021_RO_B10_2_E11_B	GREEN	No	134	104	LMG 28216	*Formosa*	*algae*
Z0021_RO_B01_2_E07_A	GREEN	No	134	104	LMG 28216	*Formosa*	*algae*
Z0021_RO_B04_2_E08_B	GREEN	No	134	104	LMG 28216	*Formosa*	*algae*
Z0021_RO_B09_2_E11_A	GREEN	No	134	104	LMG 28216	*Formosa*	*algae*
Z0021_RO_A12_1_E12_B	GREEN	No	134	104	LMG 28216	*Formosa*	*algae*

Z0021_RO_C03_1_F08_A	GREEN	No	134	104	LMG 28216	*Formosa*	*algae*	
Z0021_LO_B02_2_E01_B	GREEN	No	134	104	LMG 28216	*Formosa*	*algae*	
Z0021_LO_B04_2_E02_B	GREEN	No	134	104	LMG 28216	*Formosa*	*algae*	
Z0021_LO_B06_2_E03_B	GREEN	No	134	104	LMG 28216	*Formosa*	*algae*	
Z0021_RB_B06_1_D10_B	GREEN	Yes	135	98	LMG 27212	*Pectinatus*	*sp.*	
Z0021_LB_B06_1_D04_B	GREEN	No	135	98	LMG 27212	*Pectinatus*	*sp.*	
Z0021_RB_A08_2_D09_B	GREEN	No	135	98	LMG 27212	*Pectinatus*	*sp.*	
Z0021_RB_A09_2_D08_A	GREEN	No	135	98	LMG 27212	*Pectinatus*	*sp.*	
Z0021_RB_B04_1_D11_B	GREEN	No	135	98	LMG 27212	*Pectinatus*	*sp.*	
Z0021_LB_B05_1_D04_A	GREEN	No	135	98	LMG 27212	*Pectinatus*	*sp.*	
Z0021_RB_B05_1_D10_A	GREEN	No	135	98	LMG 27212	*Pectinatus*	*sp.*	
Z0021_LB_B07_1_D03_A	GREEN	No	135	98	LMG 27212	*Pectinatus*	*sp.*	
Z0021_LB_B08_1_D03_B	GREEN	No	135	98	LMG 27212	*Pectinatus*	*sp.*	
Z0021_RB_A07_2_D09_A	GREEN	No	135	98	LMG 27212	*Pectinatus*	*sp.*	
Z0021_RB_A12_2_D07_B	GREEN	No	135	98	LMG 27212	*Pectinatus*	*sp.*	
Z0021_LB_A12_2_D01_B	GREEN	No	135	98	LMG 27212	*Pectinatus*	*sp.*	
Z0021_LB_C05_2_C04_A	GREEN	No	135	98	LMG 27212	*Pectinatus*	*sp.*	
Z0021_LB_B01_1_D06_A	GREEN	No	135	98	LMG 27212	*Pectinatus*	*sp.*	
Z0021_RB_A10_2_D08_B	GREEN	No	135	98	LMG 27212	*Pectinatus*	*sp.*	
Z0021_LB_B09_1_D02_A	GREEN	No	135	98	LMG 27212	*Pectinatus*	*sp.*	
Z0021_RB_A11_2_D07_A	GREEN	No	135	98	LMG 27212	*Pectinatus*	*sp.*	
Z0021_LB_B11_1_D01_A	GREEN	No	135	98	LMG 27212	*Pectinatus*	*sp.*	
Z0021_LB_B12_1_D01_B	GREEN	No	135	98	LMG 27212	*Pectinatus*	*sp.*	
Z0021_RB_A06_2_D10_B	GREEN	No	135	98	LMG 27212	*Pectinatus*	*sp.*	
Z0021_LB_B02_1_D06_B	GREEN	No	135	98	LMG 27212	*Pectinatus*	*sp.*	
Z0021_RB_B07_1_D09_A	GREEN	No	135	98	LMG 27212	*Pectinatus*	*sp.*	
Z0021_LB_B10_1_D02_B	GREEN	No	135	98	LMG 27212	*Pectinatus*	*sp.*	
Z0021_LB_C02_2_C06_B	GREEN	No	135	98	LMG 27212	*Pectinatus*	*sp.*	
Z0021_LB_B04_1_D05_B	GREEN	No	135	98	LMG 27212	*Pectinatus*	*sp.*	
Z0021_LB_C01_2_C06_A	GREEN	No	135	98	LMG 27212	*Pectinatus*	*sp.*	
Z0021_LB_C04_2_C05_B	GREEN	No	135	98	LMG 27212	*Pectinatus*	*sp.*	
Z0021_RB_B03_1_D11_A	GREEN	No	135	98	LMG 27212	*Pectinatus*	*sp.*	
Z0021_RB_B02_1_D12_B	GREEN	No	135	98	LMG 27212	*Pectinatus*	*sp.*	
Z0021_RB_B01_1_D12_A	GREEN	No	135	98	LMG 27212	*Pectinatus*	*sp.*	
Z0021_LB_C03_2_C05_A	GREEN	No	135	98	LMG 27212	*Pectinatus*	*sp.*	
Z0021_LB_B03_1_D05_A	GREEN	No	135	98	LMG 27212	*Pectinatus*	*sp.*	
Z0024_RB_F11_1_B07_A	GREEN	Yes	136	127	LMG 6901	*Lactobacillus*	*delbrueckii*	*bulgaricus*
Z0024_LB_G04_2_A05_B	GREEN	No	136	127	LMG 6901	*Lactobacillus*	*delbrueckii*	*bulgaricus*
Z0024_LB_G12_2_A01_B	GREEN	No	136	127	LMG 6901	*Lactobacillus*	*delbrueckii*	*bulgaricus*
Z0024_LB_H02_1_A06_B	GREEN	No	136	127	LMG 6901	*Lactobacillus*	*delbrueckii*	*bulgaricus*
Z0024_LB_G02_2_A06_B	GREEN	No	136	127	LMG 6901	*Lactobacillus*	*delbrueckii*	*bulgaricus*
Z0024_LB_F10_1_B02_B	GREEN	No	136	127	LMG 6901	*Lactobacillus*	*delbrueckii*	*bulgaricus*
Z0024_LB_G06_2_A04_B	GREEN	No	136	127	LMG 6901	*Lactobacillus*	*delbrueckii*	*bulgaricus*
Z0024_LB_F12_1_B01_B	GREEN	No	136	127	LMG 6901	*Lactobacillus*	*delbrueckii*	*bulgaricus*
Z0024_LB_G03_2_A05_A	GREEN	No	136	127	LMG 6901	*Lactobacillus*	*delbrueckii*	*bulgaricus*
Z0024_RB_G02_2_A12_B	GREEN	No	136	127	LMG 6901	*Lactobacillus*	*delbrueckii*	*bulgaricus*
Z0024_RB_F12_1_B07_B	GREEN	No	136	127	LMG 6901	*Lactobacillus*	*delbrueckii*	*bulgaricus*
Z0024_LB_H03_1_A05_A	GREEN	No	136	127	LMG 6901	*Lactobacillus*	*delbrueckii*	*bulgaricus*
Z0024_LB_F09_1_B02_A	GREEN	No	136	127	LMG 6901	*Lactobacillus*	*delbrueckii*	*bulgaricus*
Z0024_LB_G10_2_A02_B	GREEN	No	136	127	LMG 6901	*Lactobacillus*	*delbrueckii*	*bulgaricus*
Z0024_RB_G05_2_A10_A	GREEN	No	136	127	LMG 6901	*Lactobacillus*	*delbrueckii*	*bulgaricus*
Z0024_RB_F09_1_B08_A	GREEN	No	136	127	LMG 6901	*Lactobacillus*	*delbrueckii*	*bulgaricus*
Z0024_LB_G11_2_A01_A	GREEN	No	136	127	LMG 6901	*Lactobacillus*	*delbrueckii*	*bulgaricus*
Z0024_LB_G01_2_A06_A	GREEN	No	136	127	LMG 6901	*Lactobacillus*	*delbrueckii*	*bulgaricus*
Z0024_LB_F11_1_B01_A	GREEN	No	136	127	LMG 6901	*Lactobacillus*	*delbrueckii*	*bulgaricus*
Z0024_LB_H01_1_A06_A	GREEN	No	136	127	LMG 6901	*Lactobacillus*	*delbrueckii*	*bulgaricus*
Z0024_RB_G07_2_A09_A	GREEN	No	136	127	LMG 6901	*Lactobacillus*	*delbrueckii*	*bulgaricus*
Z0024_RB_G01_2_A12_A	GREEN	No	136	127	LMG 6901	*Lactobacillus*	*delbrueckii*	*bulgaricus*
Z0024_LB_G09_2_A02_A	GREEN	No	136	127	LMG 6901	*Lactobacillus*	*delbrueckii*	*bulgaricus*
Z0024_LB_H04_1_A05_B	GREEN	No	136	127	LMG 6901	*Lactobacillus*	*delbrueckii*	*bulgaricus*
Z0024_RB_G04_2_A11_B	GREEN	No	136	127	LMG 6901	*Lactobacillus*	*delbrueckii*	*bulgaricus*
Z0024_LB_G05_2_A04_A	GREEN	No	136	127	LMG 6901	*Lactobacillus*	*delbrueckii*	*bulgaricus*
Z0024_RB_G03_2_A11_A	GREEN	No	136	127	LMG 6901	*Lactobacillus*	*delbrueckii*	*bulgaricus*
Z0024_LB_G08_2_A03_B	GREEN	No	136	127	LMG 6901	*Lactobacillus*	*delbrueckii*	*bulgaricus*
Z0024_RB_F10_1_B08_B	GREEN	No	136	127	LMG 6901	*Lactobacillus*	*delbrueckii*	*bulgaricus*
Z0024_LB_F08_1_B03_B	GREEN	No	136	127	LMG 6901	*Lactobacillus*	*delbrueckii*	*bulgaricus*
Z0024_RB_G06_2_A10_B	GREEN	No	136	127	LMG 6901	*Lactobacillus*	*delbrueckii*	*bulgaricus*
Z0024_LB_G07_2_A03_A	GREEN	No	136	127	LMG 6901	*Lactobacillus*	*delbrueckii*	*bulgaricus*
Z0021_LB_A07_2_D03_A	GREEN	Yes	137	97	LMG 27021	*Neokomagataea*	*thailandica*	
Z0021_RB_A04_2_D11_B	GREEN	No	137	97	LMG 27021	*Neokomagataea*	*thailandica*	

Z0021_RO_C05_1_F09_A	GREEN	No	137	97	LMG 27021	*Neokomagataea*	*thailandica*
Z0021_LO_H11_2_H06_A	GREEN	No	137	97	LMG 27021	*Neokomagataea*	*thailandica*
Z0021_RB_A02_2_D12_B	GREEN	No	137	97	LMG 27021	*Neokomagataea*	*thailandica*
Z0021_LO_A09_1_E05_A	GREEN	No	137	97	LMG 27021	*Neokomagataea*	*thailandica*
Z0021_LO_H12_2_H06_B	GREEN	No	137	97	LMG 27021	*Neokomagataea*	*thailandica*
Z0021_LO_A10_1_E05_B	GREEN	No	137	97	LMG 27021	*Neokomagataea*	*thailandica*
Z0021_LO_C04_1_F02_B	GREEN	No	137	97	LMG 27021	*Neokomagataea*	*thailandica*
Z0021_LB_A10_2_D02_B	GREEN	No	137	97	LMG 27021	*Neokomagataea*	*thailandica*
Z0021_LO_H10_2_H05_B	GREEN	No	137	97	LMG 27021	*Neokomagataea*	*thailandica*
Z0021_LB_A11_2_D01_A	GREEN	No	137	97	LMG 27021	*Neokomagataea*	*thailandica*
Z0021_RB_A01_2_D12_A	GREEN	No	137	97	LMG 27021	*Neokomagataea*	*thailandica*
Z0021_LB_A01_2_D06_A	GREEN	No	137	97	LMG 27021	*Neokomagataea*	*thailandica*
Z0021_LB_A06_2_D04_B	GREEN	No	137	97	LMG 27021	*Neokomagataea*	*thailandica*
Z0021_LB_A04_2_D05_B	GREEN	No	137	97	LMG 27021	*Neokomagataea*	*thailandica*
Z0021_LB_A09_2_D02_A	GREEN	No	137	97	LMG 27021	*Neokomagataea*	*thailandica*
Z0021_LO_H08_2_H04_B	GREEN	No	137	97	LMG 27021	*Neokomagataea*	*thailandica*
Z0021_LB_A08_2_D03_B	GREEN	No	137	97	LMG 27021	*Neokomagataea*	*thailandica*
Z0021_RB_A05_2_D10_A	GREEN	No	137	97	LMG 27021	*Neokomagataea*	*thailandica*
Z0021_LO_B05_2_E03_A	GREEN	No	137	97	LMG 27021	*Neokomagataea*	*thailandica*
Z0021_RO_B11_2_E12_A	GREEN	No	137	97	LMG 27021	*Neokomagataea*	*thailandica*
Z0021_RO_C01_1_F07_A	GREEN	No	137	97	LMG 27021	*Neokomagataea*	*thailandica*
Z0021_LB_A05_2_D04_A	GREEN	No	137	97	LMG 27021	*Neokomagataea*	*thailandica*
Z0021_LO_B03_2_E02_A	GREEN	No	137	97	LMG 27021	*Neokomagataea*	*thailandica*
Z0021_LO_H07_2_H04_A	GREEN	No	137	97	LMG 27021	*Neokomagataea*	*thailandica*
Z0021_LB_A02_2_D06_B	GREEN	No	137	97	LMG 27021	*Neokomagataea*	*thailandica*
Z0021_LB_A03_2_D05_A	GREEN	No	137	97	LMG 27021	*Neokomagataea*	*thailandica*
Z0021_LO_B09_2_E05_A	GREEN	No	137	97	LMG 27021	*Neokomagataea*	*thailandica*
Z0021_LO_H09_2_H05_A	GREEN	No	137	97	LMG 27021	*Neokomagataea*	*thailandica*
Z0021_RB_A03_2_D11_A	GREEN	No	137	97	LMG 27021	*Neokomagataea*	*thailandica*
Z0021_RO_B05_2_E09_A	GREEN	No	137	97	LMG 27021	*Neokomagataea*	*thailandica*
Z0014_LB_B11_1_D01_A	GREEN	Yes	138	25	LMG 18212	*Chryseobacterium*	*joostei*
Z0014_LB_B12_1_D01_B	GREEN	No	138	25	LMG 18212	*Chryseobacterium*	*joostei*
Z0014_LB_C01_2_C06_A	GREEN	No	138	25	LMG 18212	*Chryseobacterium*	*joostei*
Z0014_RB_C05_2_C10_A	GREEN	No	138	25	LMG 18212	*Chryseobacterium*	*joostei*
Z0014_LB_C02_2_C06_B	GREEN	No	138	25	LMG 18212	*Chryseobacterium*	*joostei*
Z0014_RB_B08_1_D09_B	GREEN	No	138	25	LMG 18212	*Chryseobacterium*	*joostei*
Z0014_LB_C03_2_C05_A	GREEN	No	138	25	LMG 18212	*Chryseobacterium*	*joostei*
Z0014_LB_B06_1_D04_B	GREEN	No	138	25	LMG 18212	*Chryseobacterium*	*joostei*
Z0014_RB_C06_2_C10_B	GREEN	No	138	25	LMG 18212	*Chryseobacterium*	*joostei*
Z0014_LB_C04_2_C05_B	GREEN	No	138	25	LMG 18212	*Chryseobacterium*	*joostei*
Z0014_LB_B04_1_D05_B	GREEN	No	138	25	LMG 18212	*Chryseobacterium*	*joostei*
Z0014_LB_B09_1_D02_A	GREEN	No	138	25	LMG 18212	*Chryseobacterium*	*joostei*
Z0014_LB_C06_2_C04_B	GREEN	No	138	25	LMG 18212	*Chryseobacterium*	*joostei*
Z0014_LB_B07_1_D03_A	GREEN	No	138	25	LMG 18212	*Chryseobacterium*	*joostei*
Z0014_RB_C03_2_C11_A	GREEN	No	138	25	LMG 18212	*Chryseobacterium*	*joostei*
Z0014_RB_B10_1_D08_B	GREEN	No	138	25	LMG 18212	*Chryseobacterium*	*joostei*
Z0014_RB_B12_1_D07_B	GREEN	No	138	25	LMG 18212	*Chryseobacterium*	*joostei*
Z0014_LB_B05_1_D04_A	GREEN	No	138	25	LMG 18212	*Chryseobacterium*	*joostei*
Z0014_RB_C04_2_C11_B	GREEN	No	138	25	LMG 18212	*Chryseobacterium*	*joostei*
Z0014_LB_B03_1_D05_A	GREEN	No	138	25	LMG 18212	*Chryseobacterium*	*joostei*
Z0014_LB_C05_2_C04_A	GREEN	No	138	25	LMG 18212	*Chryseobacterium*	*joostei*
Z0014_LB_B10_1_D02_B	GREEN	No	138	25	LMG 18212	*Chryseobacterium*	*joostei*
Z0014_RB_C01_2_C12_A	GREEN	No	138	25	LMG 18212	*Chryseobacterium*	*joostei*
Z0014_RB_C02_2_C12_B	GREEN	No	138	25	LMG 18212	*Chryseobacterium*	*joostei*
Z0014_RB_B11_1_D07_A	GREEN	No	138	25	LMG 18212	*Chryseobacterium*	*joostei*
Z0014_RB_B07_1_D09_A	GREEN	No	138	25	LMG 18212	*Chryseobacterium*	*joostei*
Z0014_LB_C07_2_C03_A	GREEN	No	138	25	LMG 18212	*Chryseobacterium*	*joostei*
Z0014_LB_B08_1_D03_B	GREEN	No	138	25	LMG 18212	*Chryseobacterium*	*joostei*
Z0014_RB_B09_1_D08_A	GREEN	No	138	25	LMG 18212	*Chryseobacterium*	*joostei*
Z0014_RB_C07_2_C09_A	GREEN	No	138	25	LMG 18212	*Chryseobacterium*	*joostei*
Z0014_LB_C08_2_C03_B	GREEN	No	138	25	LMG 18212	*Chryseobacterium*	*joostei*
Z0014_LB_C09_2_C02_A	GREEN	No	138	25	LMG 18212	*Chryseobacterium*	*joostei*
Z0014_LO_H05_2_H03_A	GREEN	Yes	139	31	LMG 19667	*Lactobacillus*	*diolivorans*
Z0014_RO_F05_2_G09_A	GREEN	No	139	31	LMG 19667	*Lactobacillus*	*diolivorans*
Z0014_RO_H04_2_H08_B	GREEN	No	139	31	LMG 19667	*Lactobacillus*	*diolivorans*
Z0014_LO_F07_2_G04_A	GREEN	No	139	31	LMG 19667	*Lactobacillus*	*diolivorans*
Z0014_RO_H03_2_H08_A	GREEN	No	139	31	LMG 19667	*Lactobacillus*	*diolivorans*
Z0014_LO_H06_2_H03_B	GREEN	No	139	31	LMG 19667	*Lactobacillus*	*diolivorans*
Z0014_LO_H09_2_H05_A	GREEN	No	139	31	LMG 19667	*Lactobacillus*	*diolivorans*
Z0014_RO_G04_1_H08_B	GREEN	No	139	31	LMG 19667	*Lactobacillus*	*diolivorans*

Z0014_RO_G01_1_H07_A	GREEN	No	139	31	LMG 19667	*Lactobacillus*	*diolivorans*
Z0014_RO_G08_1_H10_B	GREEN	No	139	31	LMG 19667	*Lactobacillus*	*diolivorans*
Z0014_LO_H01_2_H01_A	GREEN	No	139	31	LMG 19667	*Lactobacillus*	*diolivorans*
Z0014_LO_H11_2_H06_A	GREEN	No	139	31	LMG 19667	*Lactobacillus*	*diolivorans*
Z0014_RO_F11_2_G12_A	GREEN	No	139	31	LMG 19667	*Lactobacillus*	*diolivorans*
Z0014_LO_F11_2_G06_A	GREEN	No	139	31	LMG 19667	*Lactobacillus*	*diolivorans*
Z0014_RO_F12_2_G12_B	GREEN	No	139	31	LMG 19667	*Lactobacillus*	*diolivorans*
Z0014_RO_G09_1_H11_A	GREEN	No	139	31	LMG 19667	*Lactobacillus*	*diolivorans*
Z0014_LO_H07_2_H04_A	GREEN	No	139	31	LMG 19667	*Lactobacillus*	*diolivorans*
Z0014_RO_F01_2_G07_A	GREEN	No	139	31	LMG 19667	*Lactobacillus*	*diolivorans*
Z0014_LO_F06_2_G03_B	GREEN	No	139	31	LMG 19667	*Lactobacillus*	*diolivorans*
Z0014_RO_G03_1_H08_A	GREEN	No	139	31	LMG 19667	*Lactobacillus*	*diolivorans*
Z0014_LO_H10_2_H05_B	GREEN	No	139	31	LMG 19667	*Lactobacillus*	*diolivorans*
Z0014_RO_F08_2_G10_B	GREEN	No	139	31	LMG 19667	*Lactobacillus*	*diolivorans*
Z0014_RO_F06_2_G09_B	GREEN	No	139	31	LMG 19667	*Lactobacillus*	*diolivorans*
Z0014_RO_F10_2_G11_B	GREEN	No	139	31	LMG 19667	*Lactobacillus*	*diolivorans*
Z0014_LO_G04_1_H02_B	GREEN	No	139	31	LMG 19667	*Lactobacillus*	*diolivorans*
Z0014_RO_G02_1_H07_B	GREEN	No	139	31	LMG 19667	*Lactobacillus*	*diolivorans*
Z0014_RO_G05_1_H09_A	GREEN	No	139	31	LMG 19667	*Lactobacillus*	*diolivorans*
Z0014_RO_H01_2_H07_A	GREEN	No	139	31	LMG 19667	*Lactobacillus*	*diolivorans*
Z0014_LO_F01_2_G01_A	GREEN	No	139	31	LMG 19667	*Lactobacillus*	*diolivorans*
Z0014_RO_F02_2_G07_B	GREEN	No	139	31	LMG 19667	*Lactobacillus*	*diolivorans*
Z0014_RO_F07_2_G10_A	GREEN	No	139	31	LMG 19667	*Lactobacillus*	*diolivorans*
Z0014_LO_H02_2_H01_B	GREEN	No	139	31	LMG 19667	*Lactobacillus*	*diolivorans*
Z0020_RO_E06_1_G09_B	GREEN	Yes	140	95	LMG 2698	*Brenneria*	*salicis*
Z0020_RO_D11_2_F12_A	GREEN	No	140	95	LMG 2698	*Brenneria*	*salicis*
Z0020_RO_D12_2_F12_B	GREEN	No	140	95	LMG 2698	*Brenneria*	*salicis*
Z0020_RO_E03_1_G08_A	GREEN	No	140	95	LMG 2698	*Brenneria*	*salicis*
Z0020_LO_E05_1_G03_A	GREEN	Yes	141	95	LMG 2698	*Brenneria*	*salicis*
Z0020_RO_F07_2_G10_A	GREEN	No	141	95	LMG 2698	*Brenneria*	*salicis*
Z0020_LO_E02_1_G01_B	GREEN	No	141	95	LMG 2698	*Brenneria*	*salicis*
Z0020_RO_F02_2_G07_B	GREEN	No	141	95	LMG 2698	*Brenneria*	*salicis*
Z0020_LO_F09_2_G05_A	GREEN	No	141	95	LMG 2698	*Brenneria*	*salicis*
Z0020_LO_F03_2_G02_A	GREEN	No	141	95	LMG 2698	*Brenneria*	*salicis*
Z0020_LO_F05_2_G03_A	GREEN	No	141	95	LMG 2698	*Brenneria*	*salicis*
Z0020_RO_E11_1_G12_A	GREEN	No	141	95	LMG 2698	*Brenneria*	*salicis*
Z0020_LO_G01_1_H01_A	GREEN	No	141	95	LMG 2698	*Brenneria*	*salicis*
Z0020_RO_F09_2_G11_A	GREEN	No	141	95	LMG 2698	*Brenneria*	*salicis*
Z0020_RO_E05_1_G09_A	GREEN	No	141	95	LMG 2698	*Brenneria*	*salicis*
Z0020_RO_F04_2_G08_B	GREEN	No	141	95	LMG 2698	*Brenneria*	*salicis*
Z0020_LO_F08_2_G04_B	GREEN	No	141	95	LMG 2698	*Brenneria*	*salicis*
Z0020_LO_F04_2_G02_B	GREEN	No	141	95	LMG 2698	*Brenneria*	*salicis*
Z0020_RO_E08_1_G10_B	GREEN	No	141	95	LMG 2698	*Brenneria*	*salicis*
Z0020_LO_D06_2_F03_B	GREEN	No	141	95	LMG 2698	*Brenneria*	*salicis*
Z0020_LO_F11_2_G06_A	GREEN	No	141	95	LMG 2698	*Brenneria*	*salicis*
Z0020_LO_E03_1_G02_A	GREEN	No	141	95	LMG 2698	*Brenneria*	*salicis*
Z0020_RO_F05_2_G09_A	GREEN	No	141	95	LMG 2698	*Brenneria*	*salicis*
Z0020_RO_D09_2_F11_A	GREEN	No	141	95	LMG 2698	*Brenneria*	*salicis*
Z0020_LO_E08_1_G04_B	GREEN	No	141	95	LMG 2698	*Brenneria*	*salicis*
Z0020_LO_E11_1_G06_A	GREEN	No	141	95	LMG 2698	*Brenneria*	*salicis*
Z0020_RO_D08_2_F10_B	GREEN	No	141	95	LMG 2698	*Brenneria*	*salicis*
Z0020_LO_D12_2_F06_B	GREEN	No	141	95	LMG 2698	*Brenneria*	*salicis*
Z0020_LO_D07_2_F04_A	GREEN	No	141	95	LMG 2698	*Brenneria*	*salicis*
Z0020_LO_E06_1_G03_B	GREEN	No	141	95	LMG 2698	*Brenneria*	*salicis*
Z0020_LO_D09_2_F05_A	GREEN	No	141	95	LMG 2698	*Brenneria*	*salicis*
Z0018_RO_C11_1_F12_A	GREEN	Yes	142	76	LMG 24833	*Vagococcus*	*penaei*
Z0018_LO_E03_1_G02_A	GREEN	No	142	76	LMG 24833	*Vagococcus*	*penaei*
Z0018_LO_D06_2_F03_B	GREEN	No	142	76	LMG 24833	*Vagococcus*	*penaei*
Z0018_LO_E11_1_G06_A	GREEN	No	142	76	LMG 24833	*Vagococcus*	*penaei*
Z0018_LO_F08_2_G04_B	GREEN	No	142	76	LMG 24833	*Vagococcus*	*penaei*
Z0018_LO_E02_1_G01_B	GREEN	No	142	76	LMG 24833	*Vagococcus*	*penaei*
Z0018_LO_C09_1_F05_A	GREEN	No	142	76	LMG 24833	*Vagococcus*	*penaei*
Z0018_RO_C03_1_F08_A	GREEN	No	142	76	LMG 24833	*Vagococcus*	*penaei*
Z0018_LO_F01_2_G01_A	GREEN	No	142	76	LMG 24833	*Vagococcus*	*penaei*
Z0018_RB_H07_1_A09_A	GREEN	No	142	76	LMG 24833	*Vagococcus*	*penaei*
Z0018_LO_C12_1_F06_B	GREEN	No	142	76	LMG 24833	*Vagococcus*	*penaei*
Z0018_LO_B12_2_E06_B	GREEN	No	142	76	LMG 24833	*Vagococcus*	*penaei*
Z0018_RB_H08_1_A09_B	GREEN	No	142	76	LMG 24833	*Vagococcus*	*penaei*
Z0018_RO_C07_1_F10_A	GREEN	No	142	76	LMG 24833	*Vagococcus*	*penaei*
Z0018_LO_A11_1_E06_A	GREEN	No	142	76	LMG 24833	*Vagococcus*	*penaei*

Z0018_RO_C02_1_F07_B	GREEN	No	142	76	LMG 24833	*Vagococcus*	*penaei*
Z0018_RO_B10_2_E11_B	GREEN	No	142	76	LMG 24833	*Vagococcus*	*penaei*
Z0018_LO_D03_2_F02_A	GREEN	No	142	76	LMG 24833	*Vagococcus*	*penaei*
Z0018_LO_F02_2_G01_B	GREEN	No	142	76	LMG 24833	*Vagococcus*	*penaei*
Z0018_LO_C04_1_F02_B	GREEN	No	142	76	LMG 24833	*Vagococcus*	*penaei*
Z0018_RO_A12_1_E12_B	GREEN	No	142	76	LMG 24833	*Vagococcus*	*penaei*
Z0018_RO_B03_2_E08_A	GREEN	No	142	76	LMG 24833	*Vagococcus*	*penaei*
Z0018_RO_B02_2_E07_B	GREEN	No	142	76	LMG 24833	*Vagococcus*	*penaei*
Z0018_LO_E05_1_G03_A	GREEN	No	142	76	LMG 24833	*Vagococcus*	*penaei*
Z0018_RO_C06_1_F09_B	GREEN	No	142	76	LMG 24833	*Vagococcus*	*penaei*
Z0018_RO_C01_1_F07_A	GREEN	No	142	76	LMG 24833	*Vagococcus*	*penaei*
Z0018_LO_E07_1_G04_A	GREEN	No	142	76	LMG 24833	*Vagococcus*	*penaei*
Z0018_LO_E10_1_G05_B	GREEN	No	142	76	LMG 24833	*Vagococcus*	*penaei*
Z0018_RO_A11_1_E12_A	GREEN	No	142	76	LMG 24833	*Vagococcus*	*penaei*
Z0018_RO_B01_2_E07_A	GREEN	No	142	76	LMG 24833	*Vagococcus*	*penaei*
Z0018_LO_B10_2_E05_B	GREEN	No	142	76	LMG 24833	*Vagococcus*	*penaei*
Z0018_LO_B11_2_E06_A	GREEN	No	142	76	LMG 24833	*Vagococcus*	*penaei*
Z0015_LO_F02_2_G01_B	GREEN	Yes	143	41	LMG 2186	*Phaseolibacter*	*flectens*
Z0015_LO_E04_1_G02_B	GREEN	No	143	41	LMG 2186	*Phaseolibacter*	*flectens*
Z0015_RO_E07_1_G10_A	GREEN	No	143	41	LMG 2186	*Phaseolibacter*	*flectens*
Z0015_LO_G04_1_H02_B	GREEN	No	143	41	LMG 2186	*Phaseolibacter*	*flectens*
Z0015_LO_E06_1_G03_B	GREEN	No	143	41	LMG 2186	*Phaseolibacter*	*flectens*
Z0015_LO_E03_1_G02_A	GREEN	No	143	41	LMG 2186	*Phaseolibacter*	*flectens*
Z0015_RO_F04_2_G08_B	GREEN	No	143	41	LMG 2186	*Phaseolibacter*	*flectens*
Z0015_RO_E08_1_G10_B	GREEN	No	143	41	LMG 2186	*Phaseolibacter*	*flectens*
Z0015_LO_D12_2_F06_B	GREEN	No	143	41	LMG 2186	*Phaseolibacter*	*flectens*
Z0015_RO_G12_1_H12_B	GREEN	No	143	41	LMG 2186	*Phaseolibacter*	*flectens*
Z0015_RO_G11_1_H12_A	GREEN	No	143	41	LMG 2186	*Phaseolibacter*	*flectens*
Z0015_RO_F06_2_G09_B	GREEN	No	143	41	LMG 2186	*Phaseolibacter*	*flectens*
Z0015_RO_F07_2_G10_A	GREEN	No	143	41	LMG 2186	*Phaseolibacter*	*flectens*
Z0015_RO_H01_2_H07_A	GREEN	No	143	41	LMG 2186	*Phaseolibacter*	*flectens*
Z0015_RO_F09_2_G11_A	GREEN	No	143	41	LMG 2186	*Phaseolibacter*	*flectens*
Z0015_RO_F11_2_G12_A	GREEN	No	143	41	LMG 2186	*Phaseolibacter*	*flectens*
Z0015_RO_F05_2_G09_A	GREEN	No	143	41	LMG 2186	*Phaseolibacter*	*flectens*
Z0015_LO_G01_1_H01_A	GREEN	No	143	41	LMG 2186	*Phaseolibacter*	*flectens*
Z0015_RO_F02_2_G07_B	GREEN	No	143	41	LMG 2186	*Phaseolibacter*	*flectens*
Z0015_LO_E07_1_G04_A	GREEN	No	143	41	LMG 2186	*Phaseolibacter*	*flectens*
Z0015_LO_F12_2_G06_B	GREEN	No	143	41	LMG 2186	*Phaseolibacter*	*flectens*
Z0015_LO_E09_1_G05_A	GREEN	No	143	41	LMG 2186	*Phaseolibacter*	*flectens*
Z0015_RO_E11_1_G12_A	GREEN	No	143	41	LMG 2186	*Phaseolibacter*	*flectens*
Z0015_LO_G02_1_H01_B	GREEN	No	143	41	LMG 2186	*Phaseolibacter*	*flectens*
Z0015_LO_F04_2_G02_B	GREEN	No	143	41	LMG 2186	*Phaseolibacter*	*flectens*
Z0015_LO_F07_2_G04_A	GREEN	No	143	41	LMG 2186	*Phaseolibacter*	*flectens*
Z0015_RO_G03_1_H08_A	GREEN	No	143	41	LMG 2186	*Phaseolibacter*	*flectens*
Z0015_LO_G03_1_H02_A	GREEN	No	143	41	LMG 2186	*Phaseolibacter*	*flectens*
Z0015_RO_F12_2_G12_B	GREEN	No	143	41	LMG 2186	*Phaseolibacter*	*flectens*
Z0015_RO_G05_1_H09_A	GREEN	No	143	41	LMG 2186	*Phaseolibacter*	*flectens*
Z0015_RO_G04_1_H08_B	GREEN	No	143	41	LMG 2186	*Phaseolibacter*	*flectens*
Z0015_RO_G02_1_H07_B	GREEN	No	143	41	LMG 2186	*Phaseolibacter*	*flectens*
Z0015_LB_C12_2_C01_B	GREEN	Yes	144	35	LMG 21292	*Ketogulonicigenium*	*robustum*
Z0015_RB_B08_1_D09_B	GREEN	No	144	35	LMG 21292	*Ketogulonicigenium*	*robustum*
Z0015_LB_D01_1_C06_A	GREEN	No	144	35	LMG 21292	*Ketogulonicigenium*	*robustum*
Z0015_LB_D04_1_C05_B	GREEN	No	144	35	LMG 21292	*Ketogulonicigenium*	*robustum*
Z0015_LB_D09_1_C02_A	GREEN	No	144	35	LMG 21292	*Ketogulonicigenium*	*robustum*
Z0015_LB_D02_1_C06_B	GREEN	No	144	35	LMG 21292	*Ketogulonicigenium*	*robustum*
Z0015_RB_C01_2_C12_A	GREEN	No	144	35	LMG 21292	*Ketogulonicigenium*	*robustum*
Z0015_LB_E02_2_B06_B	GREEN	No	144	35	LMG 21292	*Ketogulonicigenium*	*robustum*
Z0015_LB_E03_2_B05_A	GREEN	No	144	35	LMG 21292	*Ketogulonicigenium*	*robustum*
Z0015_RB_C02_2_C12_B	GREEN	No	144	35	LMG 21292	*Ketogulonicigenium*	*robustum*
Z0015_LB_D07_1_C03_A	GREEN	No	144	35	LMG 21292	*Ketogulonicigenium*	*robustum*
Z0015_RB_C04_2_C11_B	GREEN	No	144	35	LMG 21292	*Ketogulonicigenium*	*robustum*
Z0015_LB_D11_1_C01_A	GREEN	No	144	35	LMG 21292	*Ketogulonicigenium*	*robustum*
Z0015_LB_C11_2_C01_A	GREEN	No	144	35	LMG 21292	*Ketogulonicigenium*	*robustum*
Z0015_LB_D10_1_C02_B	GREEN	No	144	35	LMG 21292	*Ketogulonicigenium*	*robustum*
Z0015_LB_D06_1_C04_B	GREEN	No	144	35	LMG 21292	*Ketogulonicigenium*	*robustum*
Z0015_RB_C06_2_C10_B	GREEN	No	144	35	LMG 21292	*Ketogulonicigenium*	*robustum*
Z0015_RB_B11_1_D07_A	GREEN	No	144	35	LMG 21292	*Ketogulonicigenium*	*robustum*
Z0015_RB_B10_1_D08_B	GREEN	No	144	35	LMG 21292	*Ketogulonicigenium*	*robustum*
Z0015_LB_D08_1_C03_B	GREEN	No	144	35	LMG 21292	*Ketogulonicigenium*	*robustum*
Z0015_RB_B12_1_D07_B	GREEN	No	144	35	LMG 21292	*Ketogulonicigenium*	*robustum*

Z0015_LB_D05_1_C04_A	GREEN	No	144	35	LMG 21292	*Ketogulonicigenium*	*robustum*
Z0015_RB_C07_2_C09_A	GREEN	No	144	35	LMG 21292	*Ketogulonicigenium*	*robustum*
Z0015_RB_C03_2_C11_A	GREEN	No	144	35	LMG 21292	*Ketogulonicigenium*	*robustum*
Z0015_LB_E01_2_B06_A	GREEN	No	144	35	LMG 21292	*Ketogulonicigenium*	*robustum*
Z0015_RB_C05_2_C10_A	GREEN	No	144	35	LMG 21292	*Ketogulonicigenium*	*robustum*
Z0015_LB_D12_1_C01_B	GREEN	No	144	35	LMG 21292	*Ketogulonicigenium*	*robustum*
Z0015_RB_B07_1_D09_A	GREEN	No	144	35	LMG 21292	*Ketogulonicigenium*	*robustum*
Z0015_RB_B05_1_D10_A	GREEN	No	144	35	LMG 21292	*Ketogulonicigenium*	*robustum*
Z0015_RB_B06_1_D10_B	GREEN	No	144	35	LMG 21292	*Ketogulonicigenium*	*robustum*
Z0015_LB_D03_1_C05_A	GREEN	No	144	35	LMG 21292	*Ketogulonicigenium*	*robustum*
Z0015_RB_B09_1_D08_A	GREEN	No	144	35	LMG 21292	*Ketogulonicigenium*	*robustum*
Z0015_RO_E03_1_G08_A	GREEN	Yes	145	43	LMG 22193	*Martelella*	*mediterranea*
Z0015_LO_C11_1_F06_A	GREEN	No	145	43	LMG 22193	*Martelella*	*mediterranea*
Z0015_LO_C01_1_F01_A	GREEN	No	145	43	LMG 22193	*Martelella*	*mediterranea*
Z0015_LO_C08_1_F04_B	GREEN	No	145	43	LMG 22193	*Martelella*	*mediterranea*
Z0015_LO_C06_1_F03_B	GREEN	No	145	43	LMG 22193	*Martelella*	*mediterranea*
Z0015_RO_C10_1_F11_B	GREEN	No	145	43	LMG 22193	*Martelella*	*mediterranea*
Z0015_LO_C05_1_F03_A	GREEN	No	145	43	LMG 22193	*Martelella*	*mediterranea*
Z0015_RO_C08_1_F10_B	GREEN	No	145	43	LMG 22193	*Martelella*	*mediterranea*
Z0015_LO_C10_1_F05_B	GREEN	No	145	43	LMG 22193	*Martelella*	*mediterranea*
Z0015_LO_D06_2_F03_B	GREEN	No	145	43	LMG 22193	*Martelella*	*mediterranea*
Z0015_RO_E06_1_G09_B	GREEN	No	145	43	LMG 22193	*Martelella*	*mediterranea*
Z0015_LO_B11_2_E06_A	GREEN	No	145	43	LMG 22193	*Martelella*	*mediterranea*
Z0015_RO_E09_1_G11_A	GREEN	No	145	43	LMG 22193	*Martelella*	*mediterranea*
Z0015_LO_D03_2_F02_A	GREEN	No	145	43	LMG 22193	*Martelella*	*mediterranea*
Z0015_RO_E10_1_G11_B	GREEN	No	145	43	LMG 22193	*Martelella*	*mediterranea*
Z0015_RO_E05_1_G09_A	GREEN	No	145	43	LMG 22193	*Martelella*	*mediterranea*
Z0015_LO_D10_2_F05_B	GREEN	No	145	43	LMG 22193	*Martelella*	*mediterranea*
Z0015_RO_C09_1_F11_A	GREEN	No	145	43	LMG 22193	*Martelella*	*mediterranea*
Z0015_LO_C07_1_F04_A	GREEN	No	145	43	LMG 22193	*Martelella*	*mediterranea*
Z0015_LO_B10_2_E05_B	GREEN	No	145	43	LMG 22193	*Martelella*	*mediterranea*
Z0015_RO_E12_1_G12_B	GREEN	No	145	43	LMG 22193	*Martelella*	*mediterranea*
Z0015_LO_C09_1_F05_A	GREEN	No	145	43	LMG 22193	*Martelella*	*mediterranea*
Z0015_RO_C06_1_F09_B	GREEN	No	145	43	LMG 22193	*Martelella*	*mediterranea*
Z0015_LO_D08_2_F04_B	GREEN	No	145	43	LMG 22193	*Martelella*	*mediterranea*
Z0015_RO_D07_2_F10_A	GREEN	No	145	43	LMG 22193	*Martelella*	*mediterranea*
Z0015_RO_F01_2_G07_A	GREEN	No	145	43	LMG 22193	*Martelella*	*mediterranea*
Z0015_RO_D10_2_F11_B	GREEN	No	145	43	LMG 22193	*Martelella*	*mediterranea*
Z0015_RO_D08_2_F10_B	GREEN	No	145	43	LMG 22193	*Martelella*	*mediterranea*
Z0015_LO_D07_2_F04_A	GREEN	No	145	43	LMG 22193	*Martelella*	*mediterranea*
Z0015_RO_F03_2_G08_A	GREEN	No	145	43	LMG 22193	*Martelella*	*mediterranea*
Z0015_LO_D05_2_F03_A	GREEN	No	145	43	LMG 22193	*Martelella*	*mediterranea*
Z0015_RO_D06_2_F09_B	GREEN	No	145	43	LMG 22193	*Martelella*	*mediterranea*
Z0013_LO_E09_1_G05_A	GREEN	Yes	146	21	LMG 16409	*Pandoraea*	*apista*
Z0013_LB_E06_2_B04_B	GREEN	No	146	21	LMG 16409	*Pandoraea*	*apista*
Z0013_LB_E07_2_B03_A	GREEN	No	146	21	LMG 16409	*Pandoraea*	*apista*
Z0013_RO_E07_1_G10_A	GREEN	No	146	21	LMG 16409	*Pandoraea*	*apista*
Z0013_RB_E07_2_B09_A	GREEN	No	146	21	LMG 16409	*Pandoraea*	*apista*
Z0013_LO_E10_1_G05_B	GREEN	Yes	147	21	LMG 16409	*Pandoraea*	*apista*
Z0013_RB_E06_2_B10_B	GREEN	No	147	21	LMG 16409	*Pandoraea*	*apista*
Z0013_LO_E08_1_G04_B	GREEN	No	147	21	LMG 16409	*Pandoraea*	*apista*
Z0013_LO_E04_1_G02_B	GREEN	Yes	148	21	LMG 16409	*Pandoraea*	*apista*
Z0013_LO_E07_1_G04_A	GREEN	No	148	21	LMG 16409	*Pandoraea*	*apista*
Z0013_RO_E08_1_G10_B	GREEN	No	148	21	LMG 16409	*Pandoraea*	*apista*
Z0013_LO_E02_1_G01_B	GREEN	No	148	21	LMG 16409	*Pandoraea*	*apista*
Z0013_LB_E03_2_B05_A	GREEN	No	148	21	LMG 16409	*Pandoraea*	*apista*
Z0013_RO_E06_1_G09_B	GREEN	No	148	21	LMG 16409	*Pandoraea*	*apista*
Z0013_LO_E03_1_G02_A	GREEN	No	148	21	LMG 16409	*Pandoraea*	*apista*
Z0013_LO_E05_1_G03_A	GREEN	No	148	21	LMG 16409	*Pandoraea*	*apista*
Z0013_LB_E08_2_B03_B	GREEN	No	148	21	LMG 16409	*Pandoraea*	*apista*
Z0013_RO_E11_1_G12_A	GREEN	No	148	21	LMG 16409	*Pandoraea*	*apista*
Z0013_RB_E03_2_B11_A	GREEN	No	148	21	LMG 16409	*Pandoraea*	*apista*
Z0013_RO_E10_1_G11_B	GREEN	No	148	21	LMG 16409	*Pandoraea*	*apista*
Z0013_RB_E05_2_B10_A	GREEN	No	148	21	LMG 16409	*Pandoraea*	*apista*
Z0013_LO_E11_1_G06_A	GREEN	No	148	21	LMG 16409	*Pandoraea*	*apista*
Z0013_LO_E06_1_G03_B	GREEN	No	148	21	LMG 16409	*Pandoraea*	*apista*
Z0013_RB_E04_2_B11_B	GREEN	No	148	21	LMG 16409	*Pandoraea*	*apista*
Z0013_LB_E04_2_B05_B	GREEN	No	148	21	LMG 16409	*Pandoraea*	*apista*
Z0013_RO_E05_1_G09_A	GREEN	No	148	21	LMG 16409	*Pandoraea*	*apista*
Z0013_RO_E09_1_G11_A	GREEN	Yes	149	21	LMG 16409	*Pandoraea*	*apista*

Z0013_LB_E05_2_B04_A	GREEN	No	149	21	LMG 16409	*Pandoraea*	*apista*
Z0012_RO_A06_1_E09_B	GREEN	Yes	150	8	LMG 1242	*Pseudomonas*	*aeruginosa*
Z0012_LO_B03_2_E02_A	GREEN	No	150	8	LMG 1242	*Pseudomonas*	*aeruginosa*
Z0012_RO_B09_2_E11_A	GREEN	No	150	8	LMG 1242	*Pseudomonas*	*aeruginosa*
Z0012_RO_A10_1_E11_B	GREEN	No	150	8	LMG 1242	*Pseudomonas*	*aeruginosa*
Z0012_LO_C08_1_F04_B	GREEN	No	150	8	LMG 1242	*Pseudomonas*	*aeruginosa*
Z0012_RO_B05_2_E09_A	GREEN	No	150	8	LMG 1242	*Pseudomonas*	*aeruginosa*
Z0012_RB_H11_1_A07_A	GREEN	No	150	8	LMG 1242	*Pseudomonas*	*aeruginosa*
Z0012_RB_H07_1_A09_A	GREEN	No	150	8	LMG 1242	*Pseudomonas*	*aeruginosa*
Z0012_RO_A11_1_E12_A	GREEN	No	150	8	LMG 1242	*Pseudomonas*	*aeruginosa*
Z0012_LO_A07_1_E04_A	GREEN	No	150	8	LMG 1242	*Pseudomonas*	*aeruginosa*
Z0012_RO_A08_1_E10_B	GREEN	No	150	8	LMG 1242	*Pseudomonas*	*aeruginosa*
Z0012_LO_C11_1_F06_A	GREEN	No	150	8	LMG 1242	*Pseudomonas*	*aeruginosa*
Z0012_LO_B08_2_E04_B	GREEN	No	150	8	LMG 1242	*Pseudomonas*	*aeruginosa*
Z0012_LO_B02_2_E01_B	GREEN	No	150	8	LMG 1242	*Pseudomonas*	*aeruginosa*
Z0012_RO_A03_1_E08_A	GREEN	No	150	8	LMG 1242	*Pseudomonas*	*aeruginosa*
Z0012_RO_B01_2_E07_A	GREEN	No	150	8	LMG 1242	*Pseudomonas*	*aeruginosa*
Z0012_LO_C09_1_F05_A	GREEN	No	150	8	LMG 1242	*Pseudomonas*	*aeruginosa*
Z0012_LO_C05_1_F03_A	GREEN	No	150	8	LMG 1242	*Pseudomonas*	*aeruginosa*
Z0012_LO_C06_1_F03_B	GREEN	No	150	8	LMG 1242	*Pseudomonas*	*aeruginosa*
Z0012_RB_H06_1_A10_B	GREEN	No	150	8	LMG 1242	*Pseudomonas*	*aeruginosa*
Z0012_RB_H09_1_A08_A	GREEN	No	150	8	LMG 1242	*Pseudomonas*	*aeruginosa*
Z0012_LO_B01_2_E01_A	GREEN	No	150	8	LMG 1242	*Pseudomonas*	*aeruginosa*
Z0012_LO_C03_1_F02_A	GREEN	No	150	8	LMG 1242	*Pseudomonas*	*aeruginosa*
Z0012_LO_B06_2_E03_B	GREEN	No	150	8	LMG 1242	*Pseudomonas*	*aeruginosa*
Z0012_LO_A09_1_E05_A	GREEN	No	150	8	LMG 1242	*Pseudomonas*	*aeruginosa*
Z0012_RO_B10_2_E11_B	GREEN	No	150	8	LMG 1242	*Pseudomonas*	*aeruginosa*
Z0012_LO_A11_1_E06_A	GREEN	No	150	8	LMG 1242	*Pseudomonas*	*aeruginosa*
Z0012_LO_A05_1_E03_A	GREEN	No	150	8	LMG 1242	*Pseudomonas*	*aeruginosa*
Z0012_RO_A09_1_E11_A	GREEN	No	150	8	LMG 1242	*Pseudomonas*	*aeruginosa*
Z0012_RO_A04_1_E08_B	GREEN	No	150	8	LMG 1242	*Pseudomonas*	*aeruginosa*
Z0012_RO_B07_2_E10_A	GREEN	No	150	8	LMG 1242	*Pseudomonas*	*aeruginosa*
Z0012_LO_D02_2_F01_B	GREEN	No	150	8	LMG 1242	*Pseudomonas*	*aeruginosa*
Z0018_LB_B05_1_D04_A	GREEN	Yes	151	68	LMG 24392	*Granulibacter*	*bethesdensis*
Z0018_RB_A08_2_D09_B	GREEN	No	151	68	LMG 24392	*Granulibacter*	*bethesdensis*
Z0018_RB_A11_2_D07_A	GREEN	No	151	68	LMG 24392	*Granulibacter*	*bethesdensis*
Z0018_LB_B06_1_D04_B	GREEN	No	151	68	LMG 24392	*Granulibacter*	*bethesdensis*
Z0018_RB_B02_1_D12_B	GREEN	No	151	68	LMG 24392	*Granulibacter*	*bethesdensis*
Z0018_RB_A07_2_D09_A	GREEN	No	151	68	LMG 24392	*Granulibacter*	*bethesdensis*
Z0018_LB_C06_2_C04_B	GREEN	No	151	68	LMG 24392	*Granulibacter*	*bethesdensis*
Z0018_LB_B08_1_D03_B	GREEN	No	151	68	LMG 24392	*Granulibacter*	*bethesdensis*
Z0018_LB_B07_1_D03_A	GREEN	No	151	68	LMG 24392	*Granulibacter*	*bethesdensis*
Z0018_LB_B09_1_D02_A	GREEN	No	151	68	LMG 24392	*Granulibacter*	*bethesdensis*
Z0018_RB_B01_1_D12_A	GREEN	No	151	68	LMG 24392	*Granulibacter*	*bethesdensis*
Z0018_LB_C07_2_C03_A	GREEN	No	151	68	LMG 24392	*Granulibacter*	*bethesdensis*
Z0018_RB_B07_1_D09_A	GREEN	No	151	68	LMG 24392	*Granulibacter*	*bethesdensis*
Z0018_LB_B04_1_D05_B	GREEN	No	151	68	LMG 24392	*Granulibacter*	*bethesdensis*
Z0018_RB_A10_2_D08_B	GREEN	No	151	68	LMG 24392	*Granulibacter*	*bethesdensis*
Z0018_LB_C05_2_C04_A	GREEN	No	151	68	LMG 24392	*Granulibacter*	*bethesdensis*
Z0018_RB_B04_1_D11_B	GREEN	No	151	68	LMG 24392	*Granulibacter*	*bethesdensis*
Z0018_LB_B11_1_D01_A	GREEN	No	151	68	LMG 24392	*Granulibacter*	*bethesdensis*
Z0018_LB_C03_2_C05_A	GREEN	No	151	68	LMG 24392	*Granulibacter*	*bethesdensis*
Z0018_LB_C01_2_C06_A	GREEN	No	151	68	LMG 24392	*Granulibacter*	*bethesdensis*
Z0018_LB_C02_2_C06_B	GREEN	No	151	68	LMG 24392	*Granulibacter*	*bethesdensis*
Z0018_RB_A12_2_D07_B	GREEN	No	151	68	LMG 24392	*Granulibacter*	*bethesdensis*
Z0018_LB_B02_1_D06_B	GREEN	No	151	68	LMG 24392	*Granulibacter*	*bethesdensis*
Z0018_LB_B01_1_D06_A	GREEN	No	151	68	LMG 24392	*Granulibacter*	*bethesdensis*
Z0018_LB_B10_1_D02_B	GREEN	No	151	68	LMG 24392	*Granulibacter*	*bethesdensis*
Z0018_LB_B12_1_D01_B	GREEN	No	151	68	LMG 24392	*Granulibacter*	*bethesdensis*
Z0018_RB_B06_1_D10_B	GREEN	No	151	68	LMG 24392	*Granulibacter*	*bethesdensis*
Z0018_LB_B03_1_D05_A	GREEN	No	151	68	LMG 24392	*Granulibacter*	*bethesdensis*
Z0018_RB_A09_2_D08_A	GREEN	No	151	68	LMG 24392	*Granulibacter*	*bethesdensis*
Z0018_RB_B05_1_D10_A	GREEN	No	151	68	LMG 24392	*Granulibacter*	*bethesdensis*
Z0018_LB_C04_2_C05_B	GREEN	No	151	68	LMG 24392	*Granulibacter*	*bethesdensis*
Z0018_RB_B03_1_D11_A	GREEN	No	151	68	LMG 24392	*Granulibacter*	*bethesdensis*
Z0013_RB_G03_2_A11_A	GREEN	Yes	152	24	LMG 17677	*Lactobacillus*	*pentosus*
Z0014_RB_G10_2_A08_B	GREEN	No	152	24	LMG 18401	*Lactobacillus*	*pentosus*
Z0014_RB_G09_2_A08_A	GREEN	No	152	24	LMG 18401	*Lactobacillus*	*pentosus*
Z0014_RB_G12_2_A07_B	GREEN	No	152	24	LMG 18401	*Lactobacillus*	*pentosus*
Z0014_RB_H04_1_A11_B	GREEN	No	152	24	LMG 18401	*Lactobacillus*	*pentosus*

Z0014_LB_H04_1_A05_B	GREEN	No	152	24	LMG 18401	*Lactobacillus*	*pentosus*
Z0014_RO_A05_1_E09_A	GREEN	No	152	24	LMG 18401	*Lactobacillus*	*pentosus*
Z0014_RB_G11_2_A07_A	GREEN	No	152	24	LMG 18401	*Lactobacillus*	*pentosus*
Z0014_RO_A06_1_E09_B	GREEN	No	152	24	LMG 18401	*Lactobacillus*	*pentosus*
Z0014_LB_G11_2_A01_A	GREEN	No	152	24	LMG 18401	*Lactobacillus*	*pentosus*
Z0014_RO_A02_1_E07_B	GREEN	No	152	24	LMG 18401	*Lactobacillus*	*pentosus*
Z0014_LB_H01_1_A06_A	GREEN	No	152	24	LMG 18401	*Lactobacillus*	*pentosus*
Z0014_RB_H05_1_A10_A	GREEN	No	152	24	LMG 18401	*Lactobacillus*	*pentosus*
Z0014_RB_H11_1_A07_A	GREEN	No	152	24	LMG 18401	*Lactobacillus*	*pentosus*
Z0014_LB_H05_1_A04_A	GREEN	No	152	24	LMG 18401	*Lactobacillus*	*pentosus*
Z0014_RB_H07_1_A09_A	GREEN	No	152	24	LMG 18401	*Lactobacillus*	*pentosus*
Z0014_RO_A03_1_E08_A	GREEN	No	152	24	LMG 18401	*Lactobacillus*	*pentosus*
Z0014_LB_G12_2_A01_B	GREEN	No	152	24	LMG 18401	*Lactobacillus*	*pentosus*
Z0014_LB_H02_1_A06_B	GREEN	No	152	24	LMG 18401	*Lactobacillus*	*pentosus*
Z0014_RB_H01_1_A12_A	GREEN	No	152	24	LMG 18401	*Lactobacillus*	*pentosus*
Z0014_RB_H03_1_A11_A	GREEN	No	152	24	LMG 18401	*Lactobacillus*	*pentosus*
Z0014_RB_H09_1_A08_A	GREEN	No	152	24	LMG 18401	*Lactobacillus*	*pentosus*
Z0014_RO_A07_1_E10_A	GREEN	No	152	24	LMG 18401	*Lactobacillus*	*pentosus*
Z0014_RB_H12_1_A07_B	GREEN	No	152	24	LMG 18401	*Lactobacillus*	*pentosus*
Z0014_RB_H08_1_A09_B	GREEN	No	152	24	LMG 18401	*Lactobacillus*	*pentosus*
Z0014_LB_G10_2_A02_B	GREEN	No	152	24	LMG 18401	*Lactobacillus*	*pentosus*
Z0014_RO_A04_1_E08_B	GREEN	No	152	24	LMG 18401	*Lactobacillus*	*pentosus*
Z0014_RB_H06_1_A10_B	GREEN	No	152	24	LMG 18401	*Lactobacillus*	*pentosus*
Z0014_RB_H10_1_A08_B	GREEN	No	152	24	LMG 18401	*Lactobacillus*	*pentosus*
Z0014_LB_H03_1_A05_A	GREEN	No	152	24	LMG 18401	*Lactobacillus*	*pentosus*
Z0014_RB_H02_1_A12_B	GREEN	No	152	24	LMG 18401	*Lactobacillus*	*pentosus*
Z0014_RO_A01_1_E07_A	GREEN	No	152	24	LMG 18401	*Lactobacillus*	*pentosus*
Z0014_LB_G09_2_A02_A	GREEN	No	152	24	LMG 18401	*Lactobacillus*	*pentosus*
Z0025_LO_G07_1_H04_A	GREEN	No	152	140	LMG 9210	*Lactobacillus*	*pentosus*
Z0025_LO_G06_1_H03_B	GREEN	No	152	140	LMG 9210	*Lactobacillus*	*pentosus*
Z0025_LO_G05_1_H03_A	GREEN	No	152	140	LMG 9210	*Lactobacillus*	*pentosus*
Z0025_RO_F02_2_G07_B	GREEN	No	152	140	LMG 9210	*Lactobacillus*	*pentosus*
Z0025_RO_E11_1_G12_A	GREEN	No	152	140	LMG 9210	*Lactobacillus*	*pentosus*
Z0025_LO_F02_2_G01_B	GREEN	No	152	140	LMG 9210	*Lactobacillus*	*pentosus*
Z0025_LO_E12_1_G06_B	GREEN	No	152	140	LMG 9210	*Lactobacillus*	*pentosus*
Z0025_LO_F06_2_G03_B	GREEN	No	152	140	LMG 9210	*Lactobacillus*	*pentosus*
Z0025_RO_E05_1_G09_A	GREEN	No	152	140	LMG 9210	*Lactobacillus*	*pentosus*
Z0025_LO_G03_1_H02_A	GREEN	No	152	140	LMG 9210	*Lactobacillus*	*pentosus*
Z0025_LO_G08_1_H04_B	GREEN	No	152	140	LMG 9210	*Lactobacillus*	*pentosus*
Z0025_LO_F03_2_G02_A	GREEN	No	152	140	LMG 9210	*Lactobacillus*	*pentosus*
Z0025_LO_F05_2_G03_A	GREEN	No	152	140	LMG 9210	*Lactobacillus*	*pentosus*
Z0025_LO_G09_1_H05_A	GREEN	No	152	140	LMG 9210	*Lactobacillus*	*pentosus*
Z0025_LO_G02_1_H01_B	GREEN	No	152	140	LMG 9210	*Lactobacillus*	*pentosus*
Z0025_LO_E02_1_G01_B	GREEN	No	152	140	LMG 9210	*Lactobacillus*	*pentosus*
Z0025_RO_E02_1_G07_B	GREEN	No	152	140	LMG 9210	*Lactobacillus*	*pentosus*
Z0025_LO_E03_1_G02_A	GREEN	No	152	140	LMG 9210	*Lactobacillus*	*pentosus*
Z0025_RO_D08_2_F10_B	GREEN	No	152	140	LMG 9210	*Lactobacillus*	*pentosus*
Z0025_LO_F01_2_G01_A	GREEN	No	152	140	LMG 9210	*Lactobacillus*	*pentosus*
Z0025_RO_D09_2_F11_A	GREEN	No	152	140	LMG 9210	*Lactobacillus*	*pentosus*
Z0025_RO_E10_1_G11_B	GREEN	No	152	140	LMG 9210	*Lactobacillus*	*pentosus*
Z0025_LO_E04_1_G02_B	GREEN	No	152	140	LMG 9210	*Lactobacillus*	*pentosus*
Z0025_LO_E01_1_G01_A	GREEN	No	152	140	LMG 9210	*Lactobacillus*	*pentosus*
Z0025_LO_F10_2_G05_B	GREEN	No	152	140	LMG 9210	*Lactobacillus*	*pentosus*
Z0025_LO_E09_1_G05_A	GREEN	No	152	140	LMG 9210	*Lactobacillus*	*pentosus*
Z0025_RO_D06_2_F09_B	GREEN	No	152	140	LMG 9210	*Lactobacillus*	*pentosus*
Z0025_LO_F08_2_G04_B	GREEN	No	152	140	LMG 9210	*Lactobacillus*	*pentosus*
Z0025_RO_D05_2_F09_A	GREEN	No	152	140	LMG 9210	*Lactobacillus*	*pentosus*
Z0025_LO_F09_2_G05_A	GREEN	No	152	140	LMG 9210	*Lactobacillus*	*pentosus*
Z0025_RO_D04_2_F08_B	GREEN	No	152	140	LMG 9210	*Lactobacillus*	*pentosus*
Z0013_RB_H06_1_A10_B	GREEN	No	152	24	LMG 17677	*Lactobacillus*	*pentosus*
Z0013_LB_G10_2_A02_B	GREEN	No	152	24	LMG 17677	*Lactobacillus*	*pentosus*
Z0013_RB_G05_2_A10_A	GREEN	No	152	24	LMG 17677	*Lactobacillus*	*pentosus*
Z0013_LB_H03_1_A05_A	GREEN	No	152	24	LMG 17677	*Lactobacillus*	*pentosus*
Z0013_RB_H03_1_A11_A	GREEN	No	152	24	LMG 17677	*Lactobacillus*	*pentosus*
Z0013_LB_H08_1_A03_B	GREEN	No	152	24	LMG 17677	*Lactobacillus*	*pentosus*
Z0013_LB_H05_1_A04_A	GREEN	No	152	24	LMG 17677	*Lactobacillus*	*pentosus*
Z0013_LO_H04_2_H02_B	GREEN	No	152	24	LMG 17677	*Lactobacillus*	*pentosus*
Z0013_RB_H01_1_A12_A	GREEN	No	152	24	LMG 17677	*Lactobacillus*	*pentosus*
Z0013_RB_G10_2_A08_B	GREEN	No	152	24	LMG 17677	*Lactobacillus*	*pentosus*
Z0013_LO_G11_1_H06_A	GREEN	No	152	24	LMG 17677	*Lactobacillus*	*pentosus*

Z0013_RB_H04_1_A11_B	GREEN	No	152	24	LMG 17677	*Lactobacillus*	*pentosus*	
Z0013_RO_H04_2_H08_B	GREEN	No	152	24	LMG 17677	*Lactobacillus*	*pentosus*	
Z0013_RO_H03_2_H08_A	GREEN	No	152	24	LMG 17677	*Lactobacillus*	*pentosus*	
Z0013_LO_H07_2_H04_A	GREEN	No	152	24	LMG 17677	*Lactobacillus*	*pentosus*	
Z0013_RO_G05_1_H09_A	GREEN	No	152	24	LMG 17677	*Lactobacillus*	*pentosus*	
Z0013_LO_H06_2_H03_B	GREEN	No	152	24	LMG 17677	*Lactobacillus*	*pentosus*	
Z0013_LB_G12_2_A01_B	GREEN	No	152	24	LMG 17677	*Lactobacillus*	*pentosus*	
Z0013_LB_H02_1_A06_B	GREEN	No	152	24	LMG 17677	*Lactobacillus*	*pentosus*	
Z0013_LO_H08_2_H04_B	GREEN	No	152	24	LMG 17677	*Lactobacillus*	*pentosus*	
Z0013_LO_H03_2_H02_A	GREEN	No	152	24	LMG 17677	*Lactobacillus*	*pentosus*	
Z0013_LO_H05_2_H03_A	GREEN	No	152	24	LMG 17677	*Lactobacillus*	*pentosus*	
Z0013_RB_G11_2_A07_A	GREEN	No	152	24	LMG 17677	*Lactobacillus*	*pentosus*	
Z0013_RO_G06_1_H09_B	GREEN	No	152	24	LMG 17677	*Lactobacillus*	*pentosus*	
Z0013_LO_H01_2_H01_A	GREEN	No	152	24	LMG 17677	*Lactobacillus*	*pentosus*	
Z0013_RB_H05_1_A10_A	GREEN	No	152	24	LMG 17677	*Lactobacillus*	*pentosus*	
Z0013_LB_H01_1_A06_A	GREEN	No	152	24	LMG 17677	*Lactobacillus*	*pentosus*	
Z0013_RO_H02_2_H07_B	GREEN	No	152	24	LMG 17677	*Lactobacillus*	*pentosus*	
Z0013_RO_H01_2_H07_A	GREEN	No	152	24	LMG 17677	*Lactobacillus*	*pentosus*	
Z0013_LB_H07_1_A03_A	GREEN	No	152	24	LMG 17677	*Lactobacillus*	*pentosus*	
Z0013_LB_H06_1_A04_B	GREEN	No	152	24	LMG 17677	*Lactobacillus*	*pentosus*	
Z0025_LO_F11_2_G06_A	GREEN	Yes	153	140	LMG 9210	*Lactobacillus*	*pentosus*	
Z0017_LO_F05_2_G03_A	GREEN	Yes	154	65	LMG 2404	*Pectobacterium*	*carotovorum*	*carotovorum*
Z0017_RO_F09_2_G11_A	GREEN	No	154	65	LMG 2404	*Pectobacterium*	*carotovorum*	*carotovorum*
Z0017_RO_F11_2_G12_A	GREEN	No	154	65	LMG 2404	*Pectobacterium*	*carotovorum*	*carotovorum*
Z0017_RO_F08_2_G10_B	GREEN	No	154	65	LMG 2404	*Pectobacterium*	*carotovorum*	*carotovorum*
Z0017_LO_E08_1_G04_B	GREEN	No	154	65	LMG 2404	*Pectobacterium*	*carotovorum*	*carotovorum*
Z0017_LO_F08_2_G04_B	GREEN	No	154	65	LMG 2404	*Pectobacterium*	*carotovorum*	*carotovorum*
Z0017_LO_E11_1_G06_A	GREEN	No	154	65	LMG 2404	*Pectobacterium*	*carotovorum*	*carotovorum*
Z0017_RO_E03_1_G08_A	GREEN	No	154	65	LMG 2404	*Pectobacterium*	*carotovorum*	*carotovorum*
Z0017_RO_F02_2_G07_B	GREEN	No	154	65	LMG 2404	*Pectobacterium*	*carotovorum*	*carotovorum*
Z0017_LO_F03_2_G02_A	GREEN	No	154	65	LMG 2404	*Pectobacterium*	*carotovorum*	*carotovorum*
Z0017_LO_F06_2_G03_B	GREEN	No	154	65	LMG 2404	*Pectobacterium*	*carotovorum*	*carotovorum*
Z0017_LO_D04_2_F02_B	GREEN	No	154	65	LMG 2404	*Pectobacterium*	*carotovorum*	*carotovorum*
Z0017_RO_E12_1_G12_B	GREEN	No	154	65	LMG 2404	*Pectobacterium*	*carotovorum*	*carotovorum*
Z0017_RO_D10_2_F11_B	GREEN	No	154	65	LMG 2404	*Pectobacterium*	*carotovorum*	*carotovorum*
Z0017_RO_F06_2_G09_B	GREEN	No	154	65	LMG 2404	*Pectobacterium*	*carotovorum*	*carotovorum*
Z0017_LO_E09_1_G05_A	GREEN	No	154	65	LMG 2404	*Pectobacterium*	*carotovorum*	*carotovorum*
Z0017_RO_E07_1_G10_A	GREEN	No	154	65	LMG 2404	*Pectobacterium*	*carotovorum*	*carotovorum*
Z0017_LO_D07_2_F04_A	GREEN	No	154	65	LMG 2404	*Pectobacterium*	*carotovorum*	*carotovorum*
Z0017_RO_F07_2_G10_A	GREEN	No	154	65	LMG 2404	*Pectobacterium*	*carotovorum*	*carotovorum*
Z0017_LO_D10_2_F05_B	GREEN	No	154	65	LMG 2404	*Pectobacterium*	*carotovorum*	*carotovorum*
Z0017_LO_E06_1_G03_B	GREEN	No	154	65	LMG 2404	*Pectobacterium*	*carotovorum*	*carotovorum*
Z0017_RO_D02_2_F07_B	GREEN	No	154	65	LMG 2404	*Pectobacterium*	*carotovorum*	*carotovorum*
Z0017_RO_D05_2_F09_A	GREEN	No	154	65	LMG 2404	*Pectobacterium*	*carotovorum*	*carotovorum*
Z0017_RO_E09_1_G11_A	GREEN	No	154	65	LMG 2404	*Pectobacterium*	*carotovorum*	*carotovorum*
Z0017_RO_E01_1_G07_A	GREEN	No	154	65	LMG 2404	*Pectobacterium*	*carotovorum*	*carotovorum*
Z0017_LO_E04_1_G02_B	GREEN	No	154	65	LMG 2404	*Pectobacterium*	*carotovorum*	*carotovorum*
Z0017_RO_E06_1_G09_B	GREEN	No	154	65	LMG 2404	*Pectobacterium*	*carotovorum*	*carotovorum*
Z0017_LO_E10_1_G05_B	GREEN	No	154	65	LMG 2404	*Pectobacterium*	*carotovorum*	*carotovorum*
Z0017_RO_E10_1_G11_B	GREEN	No	154	65	LMG 2404	*Pectobacterium*	*carotovorum*	*carotovorum*
Z0017_LO_D09_2_F05_A	GREEN	No	154	65	LMG 2404	*Pectobacterium*	*carotovorum*	*carotovorum*
Z0017_LO_E01_1_G01_A	GREEN	No	154	65	LMG 2404	*Pectobacterium*	*carotovorum*	*carotovorum*
Z0017_RO_D08_2_F10_B	GREEN	No	154	65	LMG 2404	*Pectobacterium*	*carotovorum*	*carotovorum*
Z0010_RO_F08_2_G10_B	GREEN	Yes	155	143	R-67189	*Burkholderia*	*vietnamiensis*	
Z0010_RO_H01_2_H07_A	GREEN	No	155	143	R-67189	*Burkholderia*	*vietnamiensis*	
Z0010_RO_F07_2_G10_A	GREEN	No	155	143	R-67189	*Burkholderia*	*vietnamiensis*	
Z0010_LO_H10_2_H05_B	GREEN	No	155	143	R-67189	*Burkholderia*	*vietnamiensis*	
Z0010_RO_G09_1_H11_A	GREEN	No	155	143	R-67189	*Burkholderia*	*vietnamiensis*	
Z0010_RO_G01_1_H07_A	GREEN	No	155	143	R-67189	*Burkholderia*	*vietnamiensis*	
Z0010_RO_F09_2_G11_A	GREEN	No	155	143	R-67189	*Burkholderia*	*vietnamiensis*	
Z0010_RB_A01_2_D12_A	GREEN	No	155	143	R-67189	*Burkholderia*	*vietnamiensis*	
Z0010_RO_H04_2_H08_B	GREEN	No	155	143	R-67189	*Burkholderia*	*vietnamiensis*	
Z0010_LO_H11_2_H06_A	GREEN	No	155	143	R-67189	*Burkholderia*	*vietnamiensis*	
Z0010_LO_H09_2_H05_A	GREEN	No	155	143	R-67189	*Burkholderia*	*vietnamiensis*	
Z0010_RO_H03_2_H08_A	GREEN	No	155	143	R-67189	*Burkholderia*	*vietnamiensis*	
Z0010_RO_G12_1_H12_B	GREEN	No	155	143	R-67189	*Burkholderia*	*vietnamiensis*	
Z0010_LO_H07_2_H04_A	GREEN	No	155	143	R-67189	*Burkholderia*	*vietnamiensis*	
Z0010_RO_G07_1_H10_A	GREEN	No	155	143	R-67189	*Burkholderia*	*vietnamiensis*	
Z0010_LO_H04_2_H02_B	GREEN	No	155	143	R-67189	*Burkholderia*	*vietnamiensis*	
Z0010_LO_H06_2_H03_B	GREEN	No	155	143	R-67189	*Burkholderia*	*vietnamiensis*	

Z0010_LO_H03_2_H02_A	GREEN	No	155	143	R-67189	*Burkholderia*	*vietnamiensis*
Z0010_LO_H12_2_H06_B	GREEN	No	155	143	R-67189	*Burkholderia*	*vietnamiensis*
Z0010_RO_G11_1_H12_A	GREEN	No	155	143	R-67189	*Burkholderia*	*vietnamiensis*
Z0010_RO_G08_1_H10_B	GREEN	No	155	143	R-67189	*Burkholderia*	*vietnamiensis*
Z0010_LO_G12_1_H06_B	GREEN	No	155	143	R-67189	*Burkholderia*	*vietnamiensis*
Z0010_RO_H02_2_H07_B	GREEN	No	155	143	R-67189	*Burkholderia*	*vietnamiensis*
Z0010_RO_G10_1_H11_B	GREEN	No	155	143	R-67189	*Burkholderia*	*vietnamiensis*
Z0010_LO_H08_2_H04_B	GREEN	No	155	143	R-67189	*Burkholderia*	*vietnamiensis*
Z0010_LO_G11_1_H06_A	GREEN	No	155	143	R-67189	*Burkholderia*	*vietnamiensis*
Z0010_LO_H01_2_H01_A	GREEN	No	155	143	R-67189	*Burkholderia*	*vietnamiensis*
Z0010_LO_H05_2_H03_A	GREEN	No	155	143	R-67189	*Burkholderia*	*vietnamiensis*
Z0010_RO_G06_1_H09_B	GREEN	No	155	143	R-67189	*Burkholderia*	*vietnamiensis*
Z0010_RO_F02_2_G07_B	GREEN	Yes	156	143	R-67189	*Burkholderia*	*vietnamiensis*
Z0010_LO_H02_2_H01_B	GREEN	No	156	143	R-67189	*Burkholderia*	*vietnamiensis*
Z0010_RO_F05_2_G09_A	GREEN	No	156	143	R-67189	*Burkholderia*	*vietnamiensis*
Z0018_LO_C11_1_F06_A	GREEN	Yes	157	73	LMG 24552	*Roseomonas*	*gilardii*
Z0018_RO_B06_2_E09_B	GREEN	Yes	158	73	LMG 24552	*Roseomonas*	*gilardii*
Z0018_RO_B12_2_E12_B	GREEN	No	158	73	LMG 24552	*Roseomonas*	*gilardii*
Z0018_LO_B09_2_E05_A	GREEN	No	158	73	LMG 24552	*Roseomonas*	*gilardii*
Z0018_RO_B04_2_E08_B	GREEN	No	158	73	LMG 24552	*Roseomonas*	*gilardii*
Z0018_RO_C05_1_F09_A	GREEN	No	158	73	LMG 24552	*Roseomonas*	*gilardii*
Z0018_LO_C06_1_F03_B	GREEN	No	158	73	LMG 24552	*Roseomonas*	*gilardii*
Z0018_LO_C07_1_F04_A	GREEN	No	158	73	LMG 24552	*Roseomonas*	*gilardii*
Z0018_RO_B07_2_E10_A	GREEN	No	158	73	LMG 24552	*Roseomonas*	*gilardii*
Z0018_LO_C10_1_F05_B	GREEN	No	158	73	LMG 24552	*Roseomonas*	*gilardii*
Z0018_RO_C04_1_F08_B	GREEN	No	158	73	LMG 24552	*Roseomonas*	*gilardii*
Z0018_LO_C08_1_F04_B	GREEN	No	158	73	LMG 24552	*Roseomonas*	*gilardii*
Z0018_LO_C01_1_F01_A	GREEN	No	158	73	LMG 24552	*Roseomonas*	*gilardii*
Z0018_LO_D01_2_F01_A	GREEN	No	158	73	LMG 24552	*Roseomonas*	*gilardii*
Z0018_LO_B07_2_E04_A	GREEN	No	158	73	LMG 24552	*Roseomonas*	*gilardii*
Z0018_RO_A10_1_E11_B	GREEN	No	158	73	LMG 24552	*Roseomonas*	*gilardii*
Z0018_RO_B05_2_E09_A	GREEN	No	158	73	LMG 24552	*Roseomonas*	*gilardii*
Z0018_LO_B06_2_E03_B	GREEN	No	158	73	LMG 24552	*Roseomonas*	*gilardii*
Z0018_RO_B09_2_E11_A	GREEN	No	158	73	LMG 24552	*Roseomonas*	*gilardii*
Z0018_LO_B03_2_E02_A	GREEN	No	158	73	LMG 24552	*Roseomonas*	*gilardii*
Z0018_LO_B05_2_E03_A	GREEN	No	158	73	LMG 24552	*Roseomonas*	*gilardii*
Z0018_LO_B04_2_E02_B	GREEN	No	158	73	LMG 24552	*Roseomonas*	*gilardii*
Z0018_RO_B11_2_E12_A	GREEN	No	158	73	LMG 24552	*Roseomonas*	*gilardii*
Z0018_LO_C03_1_F02_A	GREEN	No	158	73	LMG 24552	*Roseomonas*	*gilardii*
Z0018_RO_C09_1_F11_A	GREEN	No	158	73	LMG 24552	*Roseomonas*	*gilardii*
Z0018_RO_C08_1_F10_B	GREEN	No	158	73	LMG 24552	*Roseomonas*	*gilardii*
Z0018_LO_B08_2_E04_B	GREEN	No	158	73	LMG 24552	*Roseomonas*	*gilardii*
Z0018_LO_C05_1_F03_A	GREEN	Yes	159	73	LMG 24552	*Roseomonas*	*gilardii*
Z0013_RO_D04_2_F08_B	GREEN	Yes	160	18	LMG 1527	*Komagataeibacter*	*hansenii*
Z0013_LO_C12_1_F06_B	GREEN	No	160	18	LMG 1527	*Komagataeibacter*	*hansenii*
Z0013_LO_C08_1_F04_B	GREEN	No	160	18	LMG 1527	*Komagataeibacter*	*hansenii*
Z0013_RO_D03_2_F08_A	GREEN	No	160	18	LMG 1527	*Komagataeibacter*	*hansenii*
Z0013_RB_C08_2_C09_B	GREEN	No	160	18	LMG 1527	*Komagataeibacter*	*hansenii*
Z0013_RO_C08_1_F10_B	GREEN	No	160	18	LMG 1527	*Komagataeibacter*	*hansenii*
Z0013_RB_C04_2_C11_B	GREEN	No	160	18	LMG 1527	*Komagataeibacter*	*hansenii*
Z0013_RB_C07_2_C09_A	GREEN	No	160	18	LMG 1527	*Komagataeibacter*	*hansenii*
Z0013_LO_C10_1_F05_B	GREEN	No	160	18	LMG 1527	*Komagataeibacter*	*hansenii*
Z0013_LB_C06_2_C04_B	GREEN	No	160	18	LMG 1527	*Komagataeibacter*	*hansenii*
Z0013_RO_D02_2_F07_B	GREEN	No	160	18	LMG 1527	*Komagataeibacter*	*hansenii*
Z0013_LO_C09_1_F05_A	GREEN	No	160	18	LMG 1527	*Komagataeibacter*	*hansenii*
Z0013_LO_C07_1_F04_A	GREEN	No	160	18	LMG 1527	*Komagataeibacter*	*hansenii*
Z0013_RO_C12_1_F12_B	GREEN	No	160	18	LMG 1527	*Komagataeibacter*	*hansenii*
Z0013_RO_C09_1_F11_A	GREEN	No	160	18	LMG 1527	*Komagataeibacter*	*hansenii*
Z0013_RB_C03_2_C11_A	GREEN	No	160	18	LMG 1527	*Komagataeibacter*	*hansenii*
Z0013_RO_C10_1_F11_B	GREEN	No	160	18	LMG 1527	*Komagataeibacter*	*hansenii*
Z0013_LB_C02_2_C06_B	GREEN	No	160	18	LMG 1527	*Komagataeibacter*	*hansenii*
Z0013_RO_C11_1_F12_A	GREEN	No	160	18	LMG 1527	*Komagataeibacter*	*hansenii*
Z0013_LB_C05_2_C04_A	GREEN	No	160	18	LMG 1527	*Komagataeibacter*	*hansenii*
Z0013_LB_C08_2_C03_B	GREEN	No	160	18	LMG 1527	*Komagataeibacter*	*hansenii*
Z0013_RO_D01_2_F07_A	GREEN	No	160	18	LMG 1527	*Komagataeibacter*	*hansenii*
Z0013_LB_C03_2_C05_A	GREEN	No	160	18	LMG 1527	*Komagataeibacter*	*hansenii*
Z0013_LB_C01_2_C06_A	GREEN	No	160	18	LMG 1527	*Komagataeibacter*	*hansenii*
Z0013_LO_C06_1_F03_B	GREEN	No	160	18	LMG 1527	*Komagataeibacter*	*hansenii*
Z0013_LB_C04_2_C05_B	GREEN	No	160	18	LMG 1527	*Komagataeibacter*	*hansenii*
Z0013_LB_C09_2_C02_A	GREEN	No	160	18	LMG 1527	*Komagataeibacter*	*hansenii*

Z0013_LB_C07_2_C03_A	GREEN	No	160	18	LMG 1527	*Komagataeibacter*	*hansenii*
Z0013_LO_C11_1_F06_A	GREEN	No	160	18	LMG 1527	*Komagataeibacter*	*hansenii*
Z0013_RB_C02_2_C12_B	GREEN	No	160	18	LMG 1527	*Komagataeibacter*	*hansenii*
Z0013_RB_C05_2_C10_A	GREEN	No	160	18	LMG 1527	*Komagataeibacter*	*hansenii*
Z0013_RB_C06_2_C10_B	GREEN	No	160	18	LMG 1527	*Komagataeibacter*	*hansenii*
Z0015_LO_E02_1_G01_B	GREEN	Yes	161	42	LMG 22049	*Tatumella*	*citrea*
Z0015_LO_F06_2_G03_B	GREEN	No	161	42	LMG 22049	*Tatumella*	*citrea*
Z0015_RO_G06_1_H09_B	GREEN	No	161	42	LMG 22049	*Tatumella*	*citrea*
Z0015_RO_H02_2_H07_B	GREEN	No	161	42	LMG 22049	*Tatumella*	*citrea*
Z0015_RO_G10_1_H11_B	GREEN	No	161	42	LMG 22049	*Tatumella*	*citrea*
Z0015_RO_H03_2_H08_A	GREEN	No	161	42	LMG 22049	*Tatumella*	*citrea*
Z0015_LO_G10_1_H05_B	GREEN	No	161	42	LMG 22049	*Tatumella*	*citrea*
Z0015_RO_F08_2_G10_B	GREEN	No	161	42	LMG 22049	*Tatumella*	*citrea*
Z0015_LO_F01_2_G01_A	GREEN	No	161	42	LMG 22049	*Tatumella*	*citrea*
Z0015_LO_E11_1_G06_A	GREEN	No	161	42	LMG 22049	*Tatumella*	*citrea*
Z0015_LO_F03_2_G02_A	GREEN	No	161	42	LMG 22049	*Tatumella*	*citrea*
Z0015_LO_G07_1_H04_A	GREEN	No	161	42	LMG 22049	*Tatumella*	*citrea*
Z0015_LO_E05_1_G03_A	GREEN	No	161	42	LMG 22049	*Tatumella*	*citrea*
Z0015_LO_E01_1_G01_A	GREEN	No	161	42	LMG 22049	*Tatumella*	*citrea*
Z0015_RO_G01_1_H07_A	GREEN	No	161	42	LMG 22049	*Tatumella*	*citrea*
Z0015_LO_E10_1_G05_B	GREEN	No	161	42	LMG 22049	*Tatumella*	*citrea*
Z0015_LO_F05_2_G03_A	GREEN	No	161	42	LMG 22049	*Tatumella*	*citrea*
Z0015_LO_F09_2_G05_A	GREEN	No	161	42	LMG 22049	*Tatumella*	*citrea*
Z0015_RO_G07_1_H10_A	GREEN	No	161	42	LMG 22049	*Tatumella*	*citrea*
Z0015_LO_E12_1_G06_B	GREEN	No	161	42	LMG 22049	*Tatumella*	*citrea*
Z0015_RO_G08_1_H10_B	GREEN	No	161	42	LMG 22049	*Tatumella*	*citrea*
Z0015_LO_F11_2_G06_A	GREEN	No	161	42	LMG 22049	*Tatumella*	*citrea*
Z0015_LO_G05_1_H03_A	GREEN	No	161	42	LMG 22049	*Tatumella*	*citrea*
Z0015_LO_G08_1_H04_B	GREEN	No	161	42	LMG 22049	*Tatumella*	*citrea*
Z0015_RO_H04_2_H08_B	GREEN	No	161	42	LMG 22049	*Tatumella*	*citrea*
Z0015_LO_E08_1_G04_B	GREEN	No	161	42	LMG 22049	*Tatumella*	*citrea*
Z0015_RO_G09_1_H11_A	GREEN	No	161	42	LMG 22049	*Tatumella*	*citrea*
Z0015_LO_G06_1_H03_B	GREEN	No	161	42	LMG 22049	*Tatumella*	*citrea*
Z0015_LO_G09_1_H05_A	GREEN	No	161	42	LMG 22049	*Tatumella*	*citrea*
Z0015_RO_F10_2_G11_B	GREEN	No	161	42	LMG 22049	*Tatumella*	*citrea*
Z0015_LO_F08_2_G04_B	GREEN	No	161	42	LMG 22049	*Tatumella*	*citrea*
Z0015_LO_F10_2_G05_B	GREEN	No	161	42	LMG 22049	*Tatumella*	*citrea*
Z0015_RO_C11_1_F12_A	GREEN	Yes	162	40	LMG 21817	*Woodsholea*	*maritima*
Z0015_RO_E04_1_G08_B	GREEN	No	162	40	LMG 21817	*Woodsholea*	*maritima*
Z0015_LO_D11_2_F06_A	GREEN	No	162	40	LMG 21817	*Woodsholea*	*maritima*
Z0015_RO_C07_1_F10_A	GREEN	No	162	40	LMG 21817	*Woodsholea*	*maritima*
Z0015_LO_D09_2_F05_A	GREEN	No	162	40	LMG 21817	*Woodsholea*	*maritima*
Z0015_RO_C04_1_F08_B	GREEN	No	162	40	LMG 21817	*Woodsholea*	*maritima*
Z0015_RO_D12_2_F12_B	GREEN	No	162	40	LMG 21817	*Woodsholea*	*maritima*
Z0015_LO_C03_1_F02_A	GREEN	No	162	40	LMG 21817	*Woodsholea*	*maritima*
Z0015_LO_D04_2_F02_B	GREEN	No	162	40	LMG 21817	*Woodsholea*	*maritima*
Z0015_RO_C02_1_F07_B	GREEN	No	162	40	LMG 21817	*Woodsholea*	*maritima*
Z0015_RO_C03_1_F08_A	GREEN	No	162	40	LMG 21817	*Woodsholea*	*maritima*
Z0015_LO_C04_1_F02_B	GREEN	No	162	40	LMG 21817	*Woodsholea*	*maritima*
Z0015_LO_D01_2_F01_A	GREEN	No	162	40	LMG 21817	*Woodsholea*	*maritima*
Z0015_LO_B08_2_E04_B	GREEN	No	162	40	LMG 21817	*Woodsholea*	*maritima*
Z0015_RO_C05_1_F09_A	GREEN	No	162	40	LMG 21817	*Woodsholea*	*maritima*
Z0015_RO_D11_2_F12_A	GREEN	No	162	40	LMG 21817	*Woodsholea*	*maritima*
Z0015_RO_D02_2_F07_B	GREEN	No	162	40	LMG 21817	*Woodsholea*	*maritima*
Z0015_RO_D05_2_F09_A	GREEN	No	162	40	LMG 21817	*Woodsholea*	*maritima*
Z0015_RO_C01_1_F07_A	GREEN	No	162	40	LMG 21817	*Woodsholea*	*maritima*
Z0015_RO_D09_2_F11_A	GREEN	No	162	40	LMG 21817	*Woodsholea*	*maritima*
Z0015_RO_D03_2_F08_A	GREEN	No	162	40	LMG 21817	*Woodsholea*	*maritima*
Z0015_RO_E02_1_G07_B	GREEN	No	162	40	LMG 21817	*Woodsholea*	*maritima*
Z0015_LO_B09_2_E05_A	GREEN	No	162	40	LMG 21817	*Woodsholea*	*maritima*
Z0015_LO_C02_1_F01_B	GREEN	No	162	40	LMG 21817	*Woodsholea*	*maritima*
Z0015_RO_B12_2_E12_B	GREEN	No	162	40	LMG 21817	*Woodsholea*	*maritima*
Z0015_RO_E01_1_G07_A	GREEN	No	162	40	LMG 21817	*Woodsholea*	*maritima*
Z0015_RO_D04_2_F08_B	GREEN	No	162	40	LMG 21817	*Woodsholea*	*maritima*
Z0015_LO_B12_2_E06_B	GREEN	No	162	40	LMG 21817	*Woodsholea*	*maritima*
Z0015_RO_D01_2_F07_A	GREEN	No	162	40	LMG 21817	*Woodsholea*	*maritima*
Z0015_LO_D02_2_F01_B	GREEN	No	162	40	LMG 21817	*Woodsholea*	*maritima*
Z0015_RO_C12_1_F12_B	GREEN	No	162	40	LMG 21817	*Woodsholea*	*maritima*
Z0015_LO_C12_1_F06_B	GREEN	No	162	40	LMG 21817	*Woodsholea*	*maritima*
Z0014_RB_D04_1_C11_B	GREEN	Yes	163	26	LMG 18294	*Helicobacter*	*fennelliae*

Z0014_RB_D07_1_C09_A	GREEN	No	163	26	LMG 18294	*Helicobacter*	*fennelliae*
Z0014_RB_D09_1_C08_A	GREEN	No	163	26	LMG 18294	*Helicobacter*	*fennelliae*
Z0014_LB_D04_1_C05_B	GREEN	No	163	26	LMG 18294	*Helicobacter*	*fennelliae*
Z0014_RB_D12_1_C07_B	GREEN	No	163	26	LMG 18294	*Helicobacter*	*fennelliae*
Z0014_LB_D09_1_C02_A	GREEN	No	163	26	LMG 18294	*Helicobacter*	*fennelliae*
Z0014_LB_D05_1_C04_A	GREEN	No	163	26	LMG 18294	*Helicobacter*	*fennelliae*
Z0014_RB_E02_2_B12_B	GREEN	No	163	26	LMG 18294	*Helicobacter*	*fennelliae*
Z0014_LB_D01_1_C06_A	GREEN	No	163	26	LMG 18294	*Helicobacter*	*fennelliae*
Z0014_RB_C11_2_C07_A	GREEN	No	163	26	LMG 18294	*Helicobacter*	*fennelliae*
Z0014_RB_D02_1_C12_B	GREEN	No	163	26	LMG 18294	*Helicobacter*	*fennelliae*
Z0014_RB_E03_2_B11_A	GREEN	No	163	26	LMG 18294	*Helicobacter*	*fennelliae*
Z0014_LB_D08_1_C03_B	GREEN	No	163	26	LMG 18294	*Helicobacter*	*fennelliae*
Z0014_LB_D07_1_C03_A	GREEN	No	163	26	LMG 18294	*Helicobacter*	*fennelliae*
Z0014_RB_D10_1_C08_B	GREEN	No	163	26	LMG 18294	*Helicobacter*	*fennelliae*
Z0014_RB_D05_1_C10_A	GREEN	No	163	26	LMG 18294	*Helicobacter*	*fennelliae*
Z0014_RB_C12_2_C07_B	GREEN	No	163	26	LMG 18294	*Helicobacter*	*fennelliae*
Z0014_LB_C10_2_C02_B	GREEN	No	163	26	LMG 18294	*Helicobacter*	*fennelliae*
Z0014_RB_C09_2_C08_A	GREEN	No	163	26	LMG 18294	*Helicobacter*	*fennelliae*
Z0014_RB_C08_2_C09_B	GREEN	No	163	26	LMG 18294	*Helicobacter*	*fennelliae*
Z0014_LB_D02_1_C06_B	GREEN	No	163	26	LMG 18294	*Helicobacter*	*fennelliae*
Z0014_RB_E01_2_B12_A	GREEN	No	163	26	LMG 18294	*Helicobacter*	*fennelliae*
Z0014_LB_D03_1_C05_A	GREEN	No	163	26	LMG 18294	*Helicobacter*	*fennelliae*
Z0014_LB_D06_1_C04_B	GREEN	No	163	26	LMG 18294	*Helicobacter*	*fennelliae*
Z0014_RB_D06_1_C10_B	GREEN	No	163	26	LMG 18294	*Helicobacter*	*fennelliae*
Z0014_LB_C12_2_C01_B	GREEN	No	163	26	LMG 18294	*Helicobacter*	*fennelliae*
Z0014_RB_D03_1_C11_A	GREEN	No	163	26	LMG 18294	*Helicobacter*	*fennelliae*
Z0014_RB_D11_1_C07_A	GREEN	No	163	26	LMG 18294	*Helicobacter*	*fennelliae*
Z0014_RB_C10_2_C08_B	GREEN	No	163	26	LMG 18294	*Helicobacter*	*fennelliae*
Z0014_RB_D01_1_C12_A	GREEN	No	163	26	LMG 18294	*Helicobacter*	*fennelliae*
Z0014_RB_D08_1_C09_B	GREEN	No	163	26	LMG 18294	*Helicobacter*	*fennelliae*
Z0014_LB_C11_2_C01_A	GREEN	No	163	26	LMG 18294	*Helicobacter*	*fennelliae*
Z0017_RB_H02_1_A12_B	GREEN	Yes	164	60	LMG 23835	*Marinobacter*	*algicola*
Z0017_LB_F11_1_B01_A	GREEN	No	164	60	LMG 23835	*Marinobacter*	*algicola*
Z0017_RB_H03_1_A11_A	GREEN	No	164	60	LMG 23835	*Marinobacter*	*algicola*
Z0017_RB_G06_2_A10_B	GREEN	No	164	60	LMG 23835	*Marinobacter*	*algicola*
Z0017_LB_F12_1_B01_B	GREEN	No	164	60	LMG 23835	*Marinobacter*	*algicola*
Z0017_LB_G02_2_A06_B	GREEN	No	164	60	LMG 23835	*Marinobacter*	*algicola*
Z0017_RB_G01_2_A12_A	GREEN	No	164	60	LMG 23835	*Marinobacter*	*algicola*
Z0017_RB_F12_1_B07_B	GREEN	No	164	60	LMG 23835	*Marinobacter*	*algicola*
Z0017_RB_G03_2_A11_A	GREEN	No	164	60	LMG 23835	*Marinobacter*	*algicola*
Z0017_RB_G05_2_A10_A	GREEN	No	164	60	LMG 23835	*Marinobacter*	*algicola*
Z0017_RB_G07_2_A09_A	GREEN	No	164	60	LMG 23835	*Marinobacter*	*algicola*
Z0017_LB_H01_1_A06_A	GREEN	No	164	60	LMG 23835	*Marinobacter*	*algicola*
Z0017_LB_G01_2_A06_A	GREEN	No	164	60	LMG 23835	*Marinobacter*	*algicola*
Z0017_RB_G09_2_A08_A	GREEN	No	164	60	LMG 23835	*Marinobacter*	*algicola*
Z0017_RB_F11_1_B07_A	GREEN	No	164	60	LMG 23835	*Marinobacter*	*algicola*
Z0017_RB_G02_2_A12_B	GREEN	No	164	60	LMG 23835	*Marinobacter*	*algicola*
Z0017_RB_G08_2_A09_B	GREEN	No	164	60	LMG 23835	*Marinobacter*	*algicola*
Z0017_RB_G04_2_A11_B	GREEN	No	164	60	LMG 23835	*Marinobacter*	*algicola*
Z0017_LB_G07_2_A03_A	GREEN	No	164	60	LMG 23835	*Marinobacter*	*algicola*
Z0017_LB_G06_2_A04_B	GREEN	No	164	60	LMG 23835	*Marinobacter*	*algicola*
Z0017_LB_G10_2_A02_B	GREEN	No	164	60	LMG 23835	*Marinobacter*	*algicola*
Z0017_LB_G12_2_A01_B	GREEN	No	164	60	LMG 23835	*Marinobacter*	*algicola*
Z0017_LB_G11_2_A01_A	GREEN	No	164	60	LMG 23835	*Marinobacter*	*algicola*
Z0017_RB_H01_1_A12_A	GREEN	No	164	60	LMG 23835	*Marinobacter*	*algicola*
Z0017_RB_G12_2_A07_B	GREEN	No	164	60	LMG 23835	*Marinobacter*	*algicola*
Z0017_LB_G03_2_A05_A	GREEN	No	164	60	LMG 23835	*Marinobacter*	*algicola*
Z0017_LB_G05_2_A04_A	GREEN	No	164	60	LMG 23835	*Marinobacter*	*algicola*
Z0017_LB_G04_2_A05_B	GREEN	No	164	60	LMG 23835	*Marinobacter*	*algicola*
Z0017_LB_G09_2_A02_A	GREEN	No	164	60	LMG 23835	*Marinobacter*	*algicola*
Z0017_LB_G08_2_A03_B	GREEN	No	164	60	LMG 23835	*Marinobacter*	*algicola*
Z0017_RB_G11_2_A07_A	GREEN	No	164	60	LMG 23835	*Marinobacter*	*algicola*
Z0017_RB_G10_2_A08_B	GREEN	No	164	60	LMG 23835	*Marinobacter*	*algicola*
Z0019_RB_A01_2_D12_A	GREEN	Yes	165	77	LMG 25212	*Polynucleobacter*	*cosmopolitanus*
Z0019_LO_C02_1_F01_B	GREEN	Yes	166	77	LMG 25212	*Polynucleobacter*	*cosmopolitanus*
Z0019_LB_A11_2_D01_A	GREEN	No	166	77	LMG 25212	*Polynucleobacter*	*cosmopolitanus*
Z0019_LO_H07_2_H04_A	GREEN	No	166	77	LMG 25212	*Polynucleobacter*	*cosmopolitanus*
Z0019_LO_H04_2_H02_B	GREEN	No	166	77	LMG 25212	*Polynucleobacter*	*cosmopolitanus*
Z0019_LB_A04_2_D05_B	GREEN	No	166	77	LMG 25212	*Polynucleobacter*	*cosmopolitanus*
Z0019_LO_H06_2_H03_B	GREEN	No	166	77	LMG 25212	*Polynucleobacter*	*cosmopolitanus*

Z0019_LO_B02_2_E01_B	GREEN	No	166	77	LMG 25212	*Polynucleobacter*	*cosmopolitanus*
Z0019_LO_H03_2_H02_A	GREEN	No	166	77	LMG 25212	*Polynucleobacter*	*cosmopolitanus*
Z0019_LB_A05_2_D04_A	GREEN	No	166	77	LMG 25212	*Polynucleobacter*	*cosmopolitanus*
Z0019_LO_H02_2_H01_B	GREEN	No	166	77	LMG 25212	*Polynucleobacter*	*cosmopolitanus*
Z0019_RO_C09_1_F11_A	GREEN	No	166	77	LMG 25212	*Polynucleobacter*	*cosmopolitanus*
Z0019_LB_A06_2_D04_B	GREEN	No	166	77	LMG 25212	*Polynucleobacter*	*cosmopolitanus*
Z0019_LO_B04_2_E02_B	GREEN	No	166	77	LMG 25212	*Polynucleobacter*	*cosmopolitanus*
Z0019_LO_H05_2_H03_A	GREEN	No	166	77	LMG 25212	*Polynucleobacter*	*cosmopolitanus*
Z0019_LO_A11_1_E06_A	GREEN	No	166	77	LMG 25212	*Polynucleobacter*	*cosmopolitanus*
Z0019_LB_A09_2_D02_A	GREEN	No	166	77	LMG 25212	*Polynucleobacter*	*cosmopolitanus*
Z0019_LB_A08_2_D03_B	GREEN	No	166	77	LMG 25212	*Polynucleobacter*	*cosmopolitanus*
Z0019_LO_H08_2_H04_B	GREEN	No	166	77	LMG 25212	*Polynucleobacter*	*cosmopolitanus*
Z0019_LO_H10_2_H05_B	GREEN	No	166	77	LMG 25212	*Polynucleobacter*	*cosmopolitanus*
Z0019_RO_C08_1_F10_B	GREEN	No	166	77	LMG 25212	*Polynucleobacter*	*cosmopolitanus*
Z0019_LB_A07_2_D03_A	GREEN	No	166	77	LMG 25212	*Polynucleobacter*	*cosmopolitanus*
Z0019_LB_A02_2_D06_B	GREEN	No	166	77	LMG 25212	*Polynucleobacter*	*cosmopolitanus*
Z0019_LB_A03_2_D05_A	GREEN	No	166	77	LMG 25212	*Polynucleobacter*	*cosmopolitanus*
Z0019_LO_H11_2_H06_A	GREEN	No	166	77	LMG 25212	*Polynucleobacter*	*cosmopolitanus*
Z0019_LO_A07_1_E04_A	GREEN	No	166	77	LMG 25212	*Polynucleobacter*	*cosmopolitanus*
Z0019_LB_A12_2_D01_B	GREEN	No	166	77	LMG 25212	*Polynucleobacter*	*cosmopolitanus*
Z0019_LO_H12_2_H06_B	GREEN	No	166	77	LMG 25212	*Polynucleobacter*	*cosmopolitanus*
Z0019_LO_H09_2_H05_A	GREEN	No	166	77	LMG 25212	*Polynucleobacter*	*cosmopolitanus*
Z0019_LB_A10_2_D02_B	GREEN	No	166	77	LMG 25212	*Polynucleobacter*	*cosmopolitanus*
Z0019_RO_B09_2_E11_A	GREEN	No	166	77	LMG 25212	*Polynucleobacter*	*cosmopolitanus*
Z0019_LB_A01_2_D06_A	GREEN	No	166	77	LMG 25212	*Polynucleobacter*	*cosmopolitanus*
Z0014_LB_F10_1_B02_B	GREEN	Yes	167	22	LMG 18398	*Lactobacillus*	*paraplantarum*
Z0014_LB_G02_2_A06_B	GREEN	No	167	22	LMG 18398	*Lactobacillus*	*paraplantarum*
Z0014_LB_F04_1_B05_B	GREEN	No	167	22	LMG 18398	*Lactobacillus*	*paraplantarum*
Z0014_RB_G02_2_A12_B	GREEN	No	167	22	LMG 18398	*Lactobacillus*	*paraplantarum*
Z0014_RB_G07_2_A09_A	GREEN	No	167	22	LMG 18398	*Lactobacillus*	*paraplantarum*
Z0014_LB_F09_1_B02_A	GREEN	No	167	22	LMG 18398	*Lactobacillus*	*paraplantarum*
Z0014_LB_F05_1_B04_A	GREEN	No	167	22	LMG 18398	*Lactobacillus*	*paraplantarum*
Z0014_LB_F08_1_B03_B	GREEN	No	167	22	LMG 18398	*Lactobacillus*	*paraplantarum*
Z0014_RB_F12_1_B07_B	GREEN	No	167	22	LMG 18398	*Lactobacillus*	*paraplantarum*
Z0014_RB_F11_1_B07_A	GREEN	No	167	22	LMG 18398	*Lactobacillus*	*paraplantarum*
Z0014_LB_G06_2_A04_B	GREEN	No	167	22	LMG 18398	*Lactobacillus*	*paraplantarum*
Z0014_LB_G04_2_A05_B	GREEN	No	167	22	LMG 18398	*Lactobacillus*	*paraplantarum*
Z0014_RB_G01_2_A12_A	GREEN	No	167	22	LMG 18398	*Lactobacillus*	*paraplantarum*
Z0014_RB_F07_1_B09_A	GREEN	No	167	22	LMG 18398	*Lactobacillus*	*paraplantarum*
Z0014_RB_F06_1_B10_B	GREEN	No	167	22	LMG 18398	*Lactobacillus*	*paraplantarum*
Z0014_RB_G04_2_A11_B	GREEN	No	167	22	LMG 18398	*Lactobacillus*	*paraplantarum*
Z0014_LB_G03_2_A05_A	GREEN	No	167	22	LMG 18398	*Lactobacillus*	*paraplantarum*
Z0014_RB_G08_2_A09_B	GREEN	No	167	22	LMG 18398	*Lactobacillus*	*paraplantarum*
Z0014_LB_F06_1_B04_B	GREEN	No	167	22	LMG 18398	*Lactobacillus*	*paraplantarum*
Z0014_LB_G07_2_A03_A	GREEN	No	167	22	LMG 18398	*Lactobacillus*	*paraplantarum*
Z0014_LB_F07_1_B03_A	GREEN	No	167	22	LMG 18398	*Lactobacillus*	*paraplantarum*
Z0014_LB_G08_2_A03_B	GREEN	No	167	22	LMG 18398	*Lactobacillus*	*paraplantarum*
Z0014_RB_F08_1_B09_B	GREEN	No	167	22	LMG 18398	*Lactobacillus*	*paraplantarum*
Z0014_LB_F12_1_B01_B	GREEN	No	167	22	LMG 18398	*Lactobacillus*	*paraplantarum*
Z0014_RB_F10_1_B08_B	GREEN	No	167	22	LMG 18398	*Lactobacillus*	*paraplantarum*
Z0014_LB_G01_2_A06_A	GREEN	No	167	22	LMG 18398	*Lactobacillus*	*paraplantarum*
Z0014_RB_G03_2_A11_A	GREEN	No	167	22	LMG 18398	*Lactobacillus*	*paraplantarum*
Z0014_RB_G05_2_A10_A	GREEN	No	167	22	LMG 18398	*Lactobacillus*	*paraplantarum*
Z0014_RB_G06_2_A10_B	GREEN	No	167	22	LMG 18398	*Lactobacillus*	*paraplantarum*
Z0014_RB_F09_1_B08_A	GREEN	No	167	22	LMG 18398	*Lactobacillus*	*paraplantarum*
Z0014_LB_F11_1_B01_A	GREEN	No	167	22	LMG 18398	*Lactobacillus*	*paraplantarum*
Z0014_LB_G05_2_A04_A	GREEN	No	167	22	LMG 18398	*Lactobacillus*	*paraplantarum*
Z0018_LB_H11_1_A01_A	GREEN	Yes	168	72	LMG 24537	*Stenotrophomonas*	*rhizophila*
Z0018_RO_A08_1_E10_B	GREEN	Yes	169	72	LMG 24537	*Stenotrophomonas*	*rhizophila*
Z0018_RO_A09_1_E11_A	GREEN	No	169	72	LMG 24537	*Stenotrophomonas*	*rhizophila*
Z0018_LO_A06_1_E03_B	GREEN	No	169	72	LMG 24537	*Stenotrophomonas*	*rhizophila*
Z0018_RB_H06_1_A10_B	GREEN	No	169	72	LMG 24537	*Stenotrophomonas*	*rhizophila*
Z0018_LO_A07_1_E04_A	GREEN	No	169	72	LMG 24537	*Stenotrophomonas*	*rhizophila*
Z0018_RB_H09_1_A08_A	GREEN	No	169	72	LMG 24537	*Stenotrophomonas*	*rhizophila*
Z0018_RO_A06_1_E09_B	GREEN	No	169	72	LMG 24537	*Stenotrophomonas*	*rhizophila*
Z0018_RO_A07_1_E10_A	GREEN	No	169	72	LMG 24537	*Stenotrophomonas*	*rhizophila*
Z0018_RO_A04_1_E08_B	GREEN	No	169	72	LMG 24537	*Stenotrophomonas*	*rhizophila*
Z0018_LO_A05_1_E03_A	GREEN	No	169	72	LMG 24537	*Stenotrophomonas*	*rhizophila*
Z0018_RB_H12_1_A07_B	GREEN	No	169	72	LMG 24537	*Stenotrophomonas*	*rhizophila*
Z0018_LO_A09_1_E05_A	GREEN	No	169	72	LMG 24537	*Stenotrophomonas*	*rhizophila*

Z0018_LB_H12_1_A01_B	GREEN	No	169	72	LMG 24537	*Stenotrophomonas*	*rhizophila*
Z0018_RB_H02_1_A12_B	GREEN	No	169	72	LMG 24537	*Stenotrophomonas*	*rhizophila*
Z0018_RB_H05_1_A10_A	GREEN	No	169	72	LMG 24537	*Stenotrophomonas*	*rhizophila*
Z0018_RO_A05_1_E09_A	GREEN	No	169	72	LMG 24537	*Stenotrophomonas*	*rhizophila*
Z0018_LO_A10_1_E05_B	GREEN	No	169	72	LMG 24537	*Stenotrophomonas*	*rhizophila*
Z0018_LO_A03_1_E02_A	GREEN	No	169	72	LMG 24537	*Stenotrophomonas*	*rhizophila*
Z0018_RB_H04_1_A11_B	GREEN	No	169	72	LMG 24537	*Stenotrophomonas*	*rhizophila*
Z0018_RB_H03_1_A11_A	GREEN	No	169	72	LMG 24537	*Stenotrophomonas*	*rhizophila*
Z0018_RB_H01_1_A12_A	GREEN	No	169	72	LMG 24537	*Stenotrophomonas*	*rhizophila*
Z0018_LO_A04_1_E02_B	GREEN	No	169	72	LMG 24537	*Stenotrophomonas*	*rhizophila*
Z0018_LO_A01_1_E01_A	GREEN	No	169	72	LMG 24537	*Stenotrophomonas*	*rhizophila*
Z0018_LO_A02_1_E01_B	GREEN	No	169	72	LMG 24537	*Stenotrophomonas*	*rhizophila*
Z0018_RO_A02_1_E07_B	GREEN	No	169	72	LMG 24537	*Stenotrophomonas*	*rhizophila*
Z0018_RB_H11_1_A07_A	GREEN	No	169	72	LMG 24537	*Stenotrophomonas*	*rhizophila*
Z0018_LO_A08_1_E04_B	GREEN	No	169	72	LMG 24537	*Stenotrophomonas*	*rhizophila*
Z0017_LB_E04_2_B05_B	GREEN	Yes	170	58	LMG 23655	*Carnobacterium*	*inhibens*
Z0017_LB_D10_1_C02_B	GREEN	No	170	58	LMG 23655	*Carnobacterium*	*inhibens*
Z0017_LB_D12_1_C01_B	GREEN	No	170	58	LMG 23655	*Carnobacterium*	*inhibens*
Z0017_LB_D08_1_C03_B	GREEN	No	170	58	LMG 23655	*Carnobacterium*	*inhibens*
Z0017_LB_D11_1_C01_A	GREEN	No	170	58	LMG 23655	*Carnobacterium*	*inhibens*
Z0017_RB_D10_1_C08_B	GREEN	No	170	58	LMG 23655	*Carnobacterium*	*inhibens*
Z0017_LB_E02_2_B06_B	GREEN	No	170	58	LMG 23655	*Carnobacterium*	*inhibens*
Z0017_RB_D08_1_C09_B	GREEN	No	170	58	LMG 23655	*Carnobacterium*	*inhibens*
Z0017_RB_E04_2_B11_B	GREEN	No	170	58	LMG 23655	*Carnobacterium*	*inhibens*
Z0017_LB_E08_2_B03_B	GREEN	No	170	58	LMG 23655	*Carnobacterium*	*inhibens*
Z0017_RB_D06_1_C10_B	GREEN	No	170	58	LMG 23655	*Carnobacterium*	*inhibens*
Z0017_LB_D09_1_C02_A	GREEN	No	170	58	LMG 23655	*Carnobacterium*	*inhibens*
Z0017_RB_C11_2_C07_A	GREEN	No	170	58	LMG 23655	*Carnobacterium*	*inhibens*
Z0017_RB_C10_2_C08_B	GREEN	No	170	58	LMG 23655	*Carnobacterium*	*inhibens*
Z0017_LB_E03_2_B05_A	GREEN	No	170	58	LMG 23655	*Carnobacterium*	*inhibens*
Z0017_RB_D07_1_C09_A	GREEN	No	170	58	LMG 23655	*Carnobacterium*	*inhibens*
Z0017_RB_D05_1_C10_A	GREEN	No	170	58	LMG 23655	*Carnobacterium*	*inhibens*
Z0017_RB_D12_1_C07_B	GREEN	No	170	58	LMG 23655	*Carnobacterium*	*inhibens*
Z0017_RB_D01_1_C12_A	GREEN	No	170	58	LMG 23655	*Carnobacterium*	*inhibens*
Z0017_RB_D09_1_C08_A	GREEN	No	170	58	LMG 23655	*Carnobacterium*	*inhibens*
Z0017_RB_D02_1_C12_B	GREEN	No	170	58	LMG 23655	*Carnobacterium*	*inhibens*
Z0017_LB_E01_2_B06_A	GREEN	No	170	58	LMG 23655	*Carnobacterium*	*inhibens*
Z0017_RB_E02_2_B12_B	GREEN	No	170	58	LMG 23655	*Carnobacterium*	*inhibens*
Z0017_RB_E03_2_B11_A	GREEN	No	170	58	LMG 23655	*Carnobacterium*	*inhibens*
Z0017_LB_E07_2_B03_A	GREEN	No	170	58	LMG 23655	*Carnobacterium*	*inhibens*
Z0017_RB_D04_1_C11_B	GREEN	No	170	58	LMG 23655	*Carnobacterium*	*inhibens*
Z0017_LB_E05_2_B04_A	GREEN	No	170	58	LMG 23655	*Carnobacterium*	*inhibens*
Z0017_RB_C12_2_C07_B	GREEN	No	170	58	LMG 23655	*Carnobacterium*	*inhibens*
Z0017_RB_E01_2_B12_A	GREEN	No	170	58	LMG 23655	*Carnobacterium*	*inhibens*
Z0017_LB_E06_2_B04_B	GREEN	No	170	58	LMG 23655	*Carnobacterium*	*inhibens*
Z0017_RB_D03_1_C11_A	GREEN	No	170	58	LMG 23655	*Carnobacterium*	*inhibens*
Z0017_RB_D11_1_C07_A	GREEN	No	170	58	LMG 23655	*Carnobacterium*	*inhibens*
Z0014_LO_D04_2_F02_B	GREEN	Yes	171	29	LMG 19264	*Corynebacterium*	*casei*
Z0014_RO_C12_1_F12_B	GREEN	No	171	29	LMG 19264	*Corynebacterium*	*casei*
Z0014_LO_D10_2_F05_B	GREEN	No	171	29	LMG 19264	*Corynebacterium*	*casei*
Z0014_LO_C06_1_F03_B	GREEN	No	171	29	LMG 19264	*Corynebacterium*	*casei*
Z0014_RO_C10_1_F11_B	GREEN	No	171	29	LMG 19264	*Corynebacterium*	*casei*
Z0014_LO_E09_1_G05_A	GREEN	No	171	29	LMG 19264	*Corynebacterium*	*casei*
Z0014_LO_D12_2_F06_B	GREEN	No	171	29	LMG 19264	*Corynebacterium*	*casei*
Z0014_RO_C11_1_F12_A	GREEN	No	171	29	LMG 19264	*Corynebacterium*	*casei*
Z0014_RO_C05_1_F09_A	GREEN	No	171	29	LMG 19264	*Corynebacterium*	*casei*
Z0014_LO_D07_2_F04_A	GREEN	No	171	29	LMG 19264	*Corynebacterium*	*casei*
Z0014_LO_E01_1_G01_A	GREEN	No	171	29	LMG 19264	*Corynebacterium*	*casei*
Z0014_RO_D08_2_F10_B	GREEN	No	171	29	LMG 19264	*Corynebacterium*	*casei*
Z0014_RO_D04_2_F08_B	GREEN	No	171	29	LMG 19264	*Corynebacterium*	*casei*
Z0014_LO_E12_1_G06_B	GREEN	No	171	29	LMG 19264	*Corynebacterium*	*casei*
Z0014_LO_D09_2_F05_A	GREEN	No	171	29	LMG 19264	*Corynebacterium*	*casei*
Z0014_LO_D08_2_F04_B	GREEN	No	171	29	LMG 19264	*Corynebacterium*	*casei*
Z0014_LO_D06_2_F03_B	GREEN	No	171	29	LMG 19264	*Corynebacterium*	*casei*
Z0014_RO_D07_2_F10_A	GREEN	No	171	29	LMG 19264	*Corynebacterium*	*casei*
Z0014_RO_D09_2_F11_A	GREEN	No	171	29	LMG 19264	*Corynebacterium*	*casei*
Z0014_RO_C07_1_F10_A	GREEN	No	171	29	LMG 19264	*Corynebacterium*	*casei*
Z0014_LO_E07_1_G04_A	GREEN	No	171	29	LMG 19264	*Corynebacterium*	*casei*
Z0014_LO_E06_1_G03_B	GREEN	No	171	29	LMG 19264	*Corynebacterium*	*casei*
Z0014_LO_E08_1_G04_B	GREEN	No	171	29	LMG 19264	*Corynebacterium*	*casei*

Z0014_LO_C11_1_F06_A	GREEN	No	171	29	LMG 19264	*Corynebacterium*	*casei*	
Z0014_LO_E05_1_G03_A	GREEN	No	171	29	LMG 19264	*Corynebacterium*	*casei*	
Z0014_LO_E11_1_G06_A	GREEN	No	171	29	LMG 19264	*Corynebacterium*	*casei*	
Z0014_RO_E06_1_G09_B	GREEN	No	171	29	LMG 19264	*Corynebacterium*	*casei*	
Z0014_LO_C09_1_F05_A	GREEN	No	171	29	LMG 19264	*Corynebacterium*	*casei*	
Z0014_RO_C08_1_F10_B	GREEN	No	171	29	LMG 19264	*Corynebacterium*	*casei*	
Z0014_RO_C09_1_F11_A	GREEN	No	171	29	LMG 19264	*Corynebacterium*	*casei*	
Z0014_RO_E03_1_G08_A	GREEN	No	171	29	LMG 19264	*Corynebacterium*	*casei*	
Z0014_RO_C06_1_F09_B	GREEN	No	171	29	LMG 19264	*Corynebacterium*	*casei*	
Z0015_LB_B07_1_D03_A	GREEN	Yes	172	33	LMG 2095	*Klebsiella*	*pneumoniae*	*pneumoniae*
Z0015_LB_B04_1_D05_B	GREEN	No	172	33	LMG 2095	*Klebsiella*	*pneumoniae*	*pneumoniae*
Z0015_LO_H03_2_H02_A	GREEN	No	172	33	LMG 2095	*Klebsiella*	*pneumoniae*	*pneumoniae*
Z0015_LB_B02_1_D06_B	GREEN	No	172	33	LMG 2095	*Klebsiella*	*pneumoniae*	*pneumoniae*
Z0015_LO_H11_2_H06_A	GREEN	No	172	33	LMG 2095	*Klebsiella*	*pneumoniae*	*pneumoniae*
Z0015_LB_A10_2_D02_B	GREEN	No	172	33	LMG 2095	*Klebsiella*	*pneumoniae*	*pneumoniae*
Z0015_LO_H10_2_H05_B	GREEN	No	172	33	LMG 2095	*Klebsiella*	*pneumoniae*	*pneumoniae*
Z0015_LB_A04_2_D05_B	GREEN	No	172	33	LMG 2095	*Klebsiella*	*pneumoniae*	*pneumoniae*
Z0015_LB_A03_2_D05_A	GREEN	No	172	33	LMG 2095	*Klebsiella*	*pneumoniae*	*pneumoniae*
Z0015_LO_H02_2_H01_B	GREEN	No	172	33	LMG 2095	*Klebsiella*	*pneumoniae*	*pneumoniae*
Z0015_LO_H01_2_H01_A	GREEN	No	172	33	LMG 2095	*Klebsiella*	*pneumoniae*	*pneumoniae*
Z0015_LO_H07_2_H04_A	GREEN	No	172	33	LMG 2095	*Klebsiella*	*pneumoniae*	*pneumoniae*
Z0015_LB_A07_2_D03_A	GREEN	No	172	33	LMG 2095	*Klebsiella*	*pneumoniae*	*pneumoniae*
Z0015_LO_H08_2_H04_B	GREEN	No	172	33	LMG 2095	*Klebsiella*	*pneumoniae*	*pneumoniae*
Z0015_LB_B03_1_D05_A	GREEN	No	172	33	LMG 2095	*Klebsiella*	*pneumoniae*	*pneumoniae*
Z0015_LO_G11_1_H06_A	GREEN	No	172	33	LMG 2095	*Klebsiella*	*pneumoniae*	*pneumoniae*
Z0015_LO_H09_2_H05_A	GREEN	No	172	33	LMG 2095	*Klebsiella*	*pneumoniae*	*pneumoniae*
Z0015_LB_B05_1_D04_A	GREEN	No	172	33	LMG 2095	*Klebsiella*	*pneumoniae*	*pneumoniae*
Z0015_LB_A01_2_D06_A	GREEN	No	172	33	LMG 2095	*Klebsiella*	*pneumoniae*	*pneumoniae*
Z0015_LB_B06_1_D04_B	GREEN	No	172	33	LMG 2095	*Klebsiella*	*pneumoniae*	*pneumoniae*
Z0015_LO_H06_2_H03_B	GREEN	No	172	33	LMG 2095	*Klebsiella*	*pneumoniae*	*pneumoniae*
Z0015_LB_A02_2_D06_B	GREEN	No	172	33	LMG 2095	*Klebsiella*	*pneumoniae*	*pneumoniae*
Z0015_LO_G12_1_H06_B	GREEN	No	172	33	LMG 2095	*Klebsiella*	*pneumoniae*	*pneumoniae*
Z0015_LB_A11_2_D01_A	GREEN	No	172	33	LMG 2095	*Klebsiella*	*pneumoniae*	*pneumoniae*
Z0015_LB_A08_2_D03_B	GREEN	No	172	33	LMG 2095	*Klebsiella*	*pneumoniae*	*pneumoniae*
Z0015_LB_A06_2_D04_B	GREEN	No	172	33	LMG 2095	*Klebsiella*	*pneumoniae*	*pneumoniae*
Z0015_LO_H04_2_H02_B	GREEN	No	172	33	LMG 2095	*Klebsiella*	*pneumoniae*	*pneumoniae*
Z0015_LB_A05_2_D04_A	GREEN	No	172	33	LMG 2095	*Klebsiella*	*pneumoniae*	*pneumoniae*
Z0015_LB_A12_2_D01_B	GREEN	No	172	33	LMG 2095	*Klebsiella*	*pneumoniae*	*pneumoniae*
Z0015_LO_H05_2_H03_A	GREEN	No	172	33	LMG 2095	*Klebsiella*	*pneumoniae*	*pneumoniae*
Z0015_LB_A09_2_D02_A	GREEN	No	172	33	LMG 2095	*Klebsiella*	*pneumoniae*	*pneumoniae*
Z0015_LB_B01_1_D06_A	GREEN	No	172	33	LMG 2095	*Klebsiella*	*pneumoniae*	*pneumoniae*
Z0024_LO_C02_1_F01_B	GREEN	Yes	173	133	LMG 7529	*Desulfovibrio*	*desulfuricans*	*desulfuricans*
Z0024_LO_C07_1_F04_A	GREEN	No	173	133	LMG 7529	*Desulfovibrio*	*desulfuricans*	*desulfuricans*
Z0024_RO_E03_1_G08_A	GREEN	No	173	133	LMG 7529	*Desulfovibrio*	*desulfuricans*	*desulfuricans*
Z0024_RO_E07_1_G10_A	GREEN	No	173	133	LMG 7529	*Desulfovibrio*	*desulfuricans*	*desulfuricans*
Z0024_RO_C05_1_F09_A	GREEN	No	173	133	LMG 7529	*Desulfovibrio*	*desulfuricans*	*desulfuricans*
Z0024_LO_C01_1_F01_A	GREEN	No	173	133	LMG 7529	*Desulfovibrio*	*desulfuricans*	*desulfuricans*
Z0024_LO_D09_2_F05_A	GREEN	No	173	133	LMG 7529	*Desulfovibrio*	*desulfuricans*	*desulfuricans*
Z0024_LO_B04_2_E02_B	GREEN	No	173	133	LMG 7529	*Desulfovibrio*	*desulfuricans*	*desulfuricans*
Z0024_RO_B04_2_E08_B	GREEN	No	173	133	LMG 7529	*Desulfovibrio*	*desulfuricans*	*desulfuricans*
Z0024_RO_D02_2_F07_B	GREEN	No	173	133	LMG 7529	*Desulfovibrio*	*desulfuricans*	*desulfuricans*
Z0024_RO_D01_2_F07_A	GREEN	No	173	133	LMG 7529	*Desulfovibrio*	*desulfuricans*	*desulfuricans*
Z0024_RO_E05_1_G09_A	GREEN	No	173	133	LMG 7529	*Desulfovibrio*	*desulfuricans*	*desulfuricans*
Z0024_RO_D03_2_F08_A	GREEN	No	173	133	LMG 7529	*Desulfovibrio*	*desulfuricans*	*desulfuricans*
Z0024_LO_D04_2_F02_B	GREEN	No	173	133	LMG 7529	*Desulfovibrio*	*desulfuricans*	*desulfuricans*
Z0024_RO_A10_1_E11_B	GREEN	No	173	133	LMG 7529	*Desulfovibrio*	*desulfuricans*	*desulfuricans*
Z0024_LO_D06_2_F03_B	GREEN	No	173	133	LMG 7529	*Desulfovibrio*	*desulfuricans*	*desulfuricans*
Z0024_LO_C03_1_F02_A	GREEN	No	173	133	LMG 7529	*Desulfovibrio*	*desulfuricans*	*desulfuricans*
Z0024_RO_E08_1_G10_B	GREEN	No	173	133	LMG 7529	*Desulfovibrio*	*desulfuricans*	*desulfuricans*
Z0024_LO_B02_2_E01_B	GREEN	No	173	133	LMG 7529	*Desulfovibrio*	*desulfuricans*	*desulfuricans*
Z0024_LO_C11_1_F06_A	GREEN	No	173	133	LMG 7529	*Desulfovibrio*	*desulfuricans*	*desulfuricans*
Z0024_LO_B01_2_E01_A	GREEN	No	173	133	LMG 7529	*Desulfovibrio*	*desulfuricans*	*desulfuricans*
Z0024_RO_E09_1_G11_A	GREEN	No	173	133	LMG 7529	*Desulfovibrio*	*desulfuricans*	*desulfuricans*
Z0024_RO_E06_1_G09_B	GREEN	No	173	133	LMG 7529	*Desulfovibrio*	*desulfuricans*	*desulfuricans*
Z0024_RO_D04_2_F08_B	GREEN	No	173	133	LMG 7529	*Desulfovibrio*	*desulfuricans*	*desulfuricans*
Z0024_RO_C04_1_F08_B	GREEN	No	173	133	LMG 7529	*Desulfovibrio*	*desulfuricans*	*desulfuricans*
Z0024_LO_D02_2_F01_B	GREEN	No	173	133	LMG 7529	*Desulfovibrio*	*desulfuricans*	*desulfuricans*
Z0024_RO_E11_1_G12_A	GREEN	No	173	133	LMG 7529	*Desulfovibrio*	*desulfuricans*	*desulfuricans*
Z0024_RO_E10_1_G11_B	GREEN	No	173	133	LMG 7529	*Desulfovibrio*	*desulfuricans*	*desulfuricans*
Z0024_RO_C12_1_F12_B	GREEN	No	173	133	LMG 7529	*Desulfovibrio*	*desulfuricans*	*desulfuricans*

Z0024_RO_D06_2_F09_B	GREEN	No	173	133	LMG 7529	*Desulfovibrio*	*desulfuricans*	*desulfuricans*
Z0024_RO_D05_2_F09_A	GREEN	No	173	133	LMG 7529	*Desulfovibrio*	*desulfuricans*	*desulfuricans*
Z0024_RO_E04_1_G08_B	GREEN	No	173	133	LMG 7529	*Desulfovibrio*	*desulfuricans*	*desulfuricans*
Z0017_LB_B09_1_D02_A	GREEN	Yes	174	56	LMG 23381	*Simplicispira*	*metamorpha*	
Z0017_LB_B04_1_D05_B	GREEN	No	174	56	LMG 23381	*Simplicispira*	*metamorpha*	
Z0017_LB_B05_1_D04_A	GREEN	No	174	56	LMG 23381	*Simplicispira*	*metamorpha*	
Z0017_LB_C06_2_C04_B	GREEN	No	174	56	LMG 23381	*Simplicispira*	*metamorpha*	
Z0017_LB_C03_2_C05_A	GREEN	No	174	56	LMG 23381	*Simplicispira*	*metamorpha*	
Z0017_RB_A02_2_D12_B	GREEN	No	174	56	LMG 23381	*Simplicispira*	*metamorpha*	
Z0017_LO_H12_2_H06_B	GREEN	No	174	56	LMG 23381	*Simplicispira*	*metamorpha*	
Z0017_RB_A06_2_D10_B	GREEN	No	174	56	LMG 23381	*Simplicispira*	*metamorpha*	
Z0017_LB_B10_1_D02_B	GREEN	No	174	56	LMG 23381	*Simplicispira*	*metamorpha*	
Z0017_RB_A05_2_D10_A	GREEN	No	174	56	LMG 23381	*Simplicispira*	*metamorpha*	
Z0017_RB_A08_2_D09_B	GREEN	No	174	56	LMG 23381	*Simplicispira*	*metamorpha*	
Z0017_RB_A07_2_D09_A	GREEN	No	174	56	LMG 23381	*Simplicispira*	*metamorpha*	
Z0017_LB_B12_1_D01_B	GREEN	No	174	56	LMG 23381	*Simplicispira*	*metamorpha*	
Z0017_LB_B07_1_D03_A	GREEN	No	174	56	LMG 23381	*Simplicispira*	*metamorpha*	
Z0017_LB_B08_1_D03_B	GREEN	No	174	56	LMG 23381	*Simplicispira*	*metamorpha*	
Z0017_RB_A03_2_D11_A	GREEN	No	174	56	LMG 23381	*Simplicispira*	*metamorpha*	
Z0017_RB_A10_2_D08_B	GREEN	No	174	56	LMG 23381	*Simplicispira*	*metamorpha*	
Z0017_LB_B02_1_D06_B	GREEN	No	174	56	LMG 23381	*Simplicispira*	*metamorpha*	
Z0017_LB_C04_2_C05_B	GREEN	No	174	56	LMG 23381	*Simplicispira*	*metamorpha*	
Z0017_RB_A11_2_D07_A	GREEN	No	174	56	LMG 23381	*Simplicispira*	*metamorpha*	
Z0017_LB_B03_1_D05_A	GREEN	No	174	56	LMG 23381	*Simplicispira*	*metamorpha*	
Z0017_RB_A09_2_D08_A	GREEN	No	174	56	LMG 23381	*Simplicispira*	*metamorpha*	
Z0017_RB_A12_2_D07_B	GREEN	No	174	56	LMG 23381	*Simplicispira*	*metamorpha*	
Z0017_RB_A04_2_D11_B	GREEN	No	174	56	LMG 23381	*Simplicispira*	*metamorpha*	
Z0017_RB_A01_2_D12_A	GREEN	No	174	56	LMG 23381	*Simplicispira*	*metamorpha*	
Z0017_LB_C02_2_C06_B	GREEN	No	174	56	LMG 23381	*Simplicispira*	*metamorpha*	
Z0017_LB_B11_1_D01_A	GREEN	No	174	56	LMG 23381	*Simplicispira*	*metamorpha*	
Z0017_LB_C01_2_C06_A	GREEN	No	174	56	LMG 23381	*Simplicispira*	*metamorpha*	
Z0017_LB_B06_1_D04_B	GREEN	No	174	56	LMG 23381	*Simplicispira*	*metamorpha*	
Z0017_RB_B01_1_D12_A	GREEN	No	174	56	LMG 23381	*Simplicispira*	*metamorpha*	
Z0017_RB_B02_1_D12_B	GREEN	No	174	56	LMG 23381	*Simplicispira*	*metamorpha*	
Z0017_LB_C05_2_C04_A	GREEN	No	174	56	LMG 23381	*Simplicispira*	*metamorpha*	
Z0019_LO_B08_2_E04_B	GREEN	Yes	175	88	LMG 26187	*Halomonas*	*ventosae*	
Z0019_RB_H08_1_A09_B	GREEN	No	175	88	LMG 26187	*Halomonas*	*ventosae*	
Z0019_RO_B04_2_E08_B	GREEN	No	175	88	LMG 26187	*Halomonas*	*ventosae*	
Z0019_RO_A12_1_E12_B	GREEN	No	175	88	LMG 26187	*Halomonas*	*ventosae*	
Z0019_LO_D04_2_F02_B	GREEN	No	175	88	LMG 26187	*Halomonas*	*ventosae*	
Z0019_LO_D02_2_F01_B	GREEN	No	175	88	LMG 26187	*Halomonas*	*ventosae*	
Z0019_LO_D07_2_F04_A	GREEN	No	175	88	LMG 26187	*Halomonas*	*ventosae*	
Z0019_RB_H07_1_A09_A	GREEN	No	175	88	LMG 26187	*Halomonas*	*ventosae*	
Z0019_RO_A10_1_E11_B	GREEN	No	175	88	LMG 26187	*Halomonas*	*ventosae*	
Z0019_RB_H06_1_A10_B	GREEN	No	175	88	LMG 26187	*Halomonas*	*ventosae*	
Z0019_RO_C06_1_F09_B	GREEN	No	175	88	LMG 26187	*Halomonas*	*ventosae*	
Z0019_LO_B09_2_E05_A	GREEN	No	175	88	LMG 26187	*Halomonas*	*ventosae*	
Z0019_LO_D03_2_F02_A	GREEN	No	175	88	LMG 26187	*Halomonas*	*ventosae*	
Z0019_RB_H11_1_A07_A	GREEN	No	175	88	LMG 26187	*Halomonas*	*ventosae*	
Z0019_LO_A10_1_E05_B	GREEN	No	175	88	LMG 26187	*Halomonas*	*ventosae*	
Z0019_LO_C07_1_F04_A	GREEN	No	175	88	LMG 26187	*Halomonas*	*ventosae*	
Z0019_RO_D05_2_F09_A	GREEN	No	175	88	LMG 26187	*Halomonas*	*ventosae*	
Z0019_LO_D01_2_F01_A	GREEN	No	175	88	LMG 26187	*Halomonas*	*ventosae*	
Z0019_RB_H10_1_A08_B	GREEN	No	175	88	LMG 26187	*Halomonas*	*ventosae*	
Z0019_RO_A02_1_E07_B	GREEN	No	175	88	LMG 26187	*Halomonas*	*ventosae*	
Z0019_LO_C09_1_F05_A	GREEN	No	175	88	LMG 26187	*Halomonas*	*ventosae*	
Z0019_LO_D08_2_F04_B	GREEN	No	175	88	LMG 26187	*Halomonas*	*ventosae*	
Z0019_LO_B06_2_E03_B	GREEN	No	175	88	LMG 26187	*Halomonas*	*ventosae*	
Z0019_RO_A01_1_E07_A	GREEN	No	175	88	LMG 26187	*Halomonas*	*ventosae*	
Z0019_LO_B10_2_E05_B	GREEN	No	175	88	LMG 26187	*Halomonas*	*ventosae*	
Z0019_LO_B11_2_E06_A	GREEN	No	175	88	LMG 26187	*Halomonas*	*ventosae*	
Z0019_RO_C12_1_F12_B	GREEN	No	175	88	LMG 26187	*Halomonas*	*ventosae*	
Z0019_RO_C07_1_F10_A	GREEN	No	175	88	LMG 26187	*Halomonas*	*ventosae*	
Z0019_LO_B12_2_E06_B	GREEN	No	175	88	LMG 26187	*Halomonas*	*ventosae*	
Z0019_RB_H12_1_A07_B	GREEN	No	175	88	LMG 26187	*Halomonas*	*ventosae*	
Z0019_LO_C10_1_F05_B	GREEN	No	175	88	LMG 26187	*Halomonas*	*ventosae*	
Z0019_LO_C01_1_F01_A	GREEN	No	175	88	LMG 26187	*Halomonas*	*ventosae*	
Z0015_LB_G09_2_A02_A	GREEN	Yes	176	37	LMG 21371	*Erwinia*	*carotovora*	
Z0015_LB_F08_1_B03_B	GREEN	Yes	177	37	LMG 21371	*Erwinia*	*carotovora*	
Z0015_RB_F02_1_B12_B	GREEN	No	177	37	LMG 21371	*Erwinia*	*carotovora*	

Z0015_LB_F07_1_B03_A	GREEN	No	177	37	LMG 21371	*Erwinia*	*carotovora*
Z0015_LB_G03_2_A05_A	GREEN	No	177	37	LMG 21371	*Erwinia*	*carotovora*
Z0015_LB_G10_2_A02_B	GREEN	No	177	37	LMG 21371	*Erwinia*	*carotovora*
Z0015_LB_F10_1_B02_B	GREEN	No	177	37	LMG 21371	*Erwinia*	*carotovora*
Z0015_LB_G02_2_A06_B	GREEN	No	177	37	LMG 21371	*Erwinia*	*carotovora*
Z0015_RB_E03_2_B11_A	GREEN	No	177	37	LMG 21371	*Erwinia*	*carotovora*
Z0015_LB_F11_1_B01_A	GREEN	No	177	37	LMG 21371	*Erwinia*	*carotovora*
Z0015_RB_E07_2_B09_A	GREEN	No	177	37	LMG 21371	*Erwinia*	*carotovora*
Z0015_LB_G11_2_A01_A	GREEN	No	177	37	LMG 21371	*Erwinia*	*carotovora*
Z0015_RB_E09_2_B08_A	GREEN	No	177	37	LMG 21371	*Erwinia*	*carotovora*
Z0015_RB_E06_2_B10_B	GREEN	No	177	37	LMG 21371	*Erwinia*	*carotovora*
Z0015_LB_F09_1_B02_A	GREEN	No	177	37	LMG 21371	*Erwinia*	*carotovora*
Z0015_RB_F03_1_B11_A	GREEN	No	177	37	LMG 21371	*Erwinia*	*carotovora*
Z0015_LB_G01_2_A06_A	GREEN	No	177	37	LMG 21371	*Erwinia*	*carotovora*
Z0015_LB_F12_1_B01_B	GREEN	No	177	37	LMG 21371	*Erwinia*	*carotovora*
Z0015_RB_E10_2_B08_B	GREEN	No	177	37	LMG 21371	*Erwinia*	*carotovora*
Z0015_RB_E01_2_B12_A	GREEN	No	177	37	LMG 21371	*Erwinia*	*carotovora*
Z0015_LB_G06_2_A04_B	GREEN	No	177	37	LMG 21371	*Erwinia*	*carotovora*
Z0015_RB_E05_2_B10_A	GREEN	No	177	37	LMG 21371	*Erwinia*	*carotovora*
Z0015_RB_E04_2_B11_B	GREEN	No	177	37	LMG 21371	*Erwinia*	*carotovora*
Z0015_RB_E11_2_B07_A	GREEN	No	177	37	LMG 21371	*Erwinia*	*carotovora*
Z0015_RB_F01_1_B12_A	GREEN	No	177	37	LMG 21371	*Erwinia*	*carotovora*
Z0015_RB_E12_2_B07_B	GREEN	No	177	37	LMG 21371	*Erwinia*	*carotovora*
Z0015_RB_E02_2_B12_B	GREEN	No	177	37	LMG 21371	*Erwinia*	*carotovora*
Z0015_LB_G08_2_A03_B	GREEN	No	177	37	LMG 21371	*Erwinia*	*carotovora*
Z0015_LB_G04_2_A05_B	GREEN	No	177	37	LMG 21371	*Erwinia*	*carotovora*
Z0015_LB_G07_2_A03_A	GREEN	No	177	37	LMG 21371	*Erwinia*	*carotovora*
Z0015_RB_E08_2_B09_B	GREEN	No	177	37	LMG 21371	*Erwinia*	*carotovora*
Z0015_LB_G05_2_A04_A	GREEN	No	177	37	LMG 21371	*Erwinia*	*carotovora*
Z0011_RO_B07_2_E10_A	GREEN	Yes	178	144	R-67258	*Burkholderia*	*multivorans*
Z0011_LB_D07_1_C03_A	GREEN	No	178	142	R-67536	*Burkholderia*	*multivorans*
Z0011_LB_D08_1_C03_B	GREEN	No	178	142	R-67536	*Burkholderia*	*multivorans*
Z0011_LB_D10_1_C02_B	GREEN	No	178	142	R-67536	*Burkholderia*	*multivorans*
Z0011_LB_D09_1_C02_A	GREEN	No	178	142	R-67536	*Burkholderia*	*multivorans*
Z0011_LB_D06_1_C04_B	GREEN	No	178	142	R-67536	*Burkholderia*	*multivorans*
Z0011_RB_H12_1_A07_B	GREEN	Yes	179	142	R-68768	*Burkholderia*	*multivorans*
Z0010_LO_G03_1_H02_A	GREEN	Yes	180	144	R-67196	*Burkholderia*	*multivorans*
Z0011_RB_B08_1_D09_B	GREEN	No	180	142	R-67536	*Burkholderia*	*multivorans*
Z0011_RB_B05_1_D10_A	GREEN	No	180	142	R-67536	*Burkholderia*	*multivorans*
Z0011_RB_B06_1_D10_B	GREEN	No	180	142	R-67536	*Burkholderia*	*multivorans*
Z0011_LB_D11_1_C01_A	GREEN	No	180	142	R-67536	*Burkholderia*	*multivorans*
Z0011_RB_C02_2_C12_B	GREEN	No	180	142	R-67536	*Burkholderia*	*multivorans*
Z0011_LB_D04_1_C05_B	GREEN	No	180	142	R-67536	*Burkholderia*	*multivorans*
Z0011_LB_D02_1_C06_B	GREEN	No	180	142	R-67536	*Burkholderia*	*multivorans*
Z0011_LB_D01_1_C06_A	GREEN	No	180	142	R-67536	*Burkholderia*	*multivorans*
Z0011_LB_D05_1_C04_A	GREEN	No	180	142	R-67536	*Burkholderia*	*multivorans*
Z0011_LB_E04_2_B05_B	GREEN	No	180	142	R-67536	*Burkholderia*	*multivorans*
Z0011_RB_C01_2_C12_A	GREEN	No	180	142	R-67536	*Burkholderia*	*multivorans*
Z0011_LB_D03_1_C05_A	GREEN	No	180	142	R-67536	*Burkholderia*	*multivorans*
Z0011_RB_B07_1_D09_A	GREEN	No	180	142	R-67536	*Burkholderia*	*multivorans*
Z0011_RB_B11_1_D07_A	GREEN	No	180	142	R-67536	*Burkholderia*	*multivorans*
Z0011_LB_E01_2_B06_A	GREEN	No	180	142	R-67536	*Burkholderia*	*multivorans*
Z0011_LB_E03_2_B05_A	GREEN	No	180	142	R-67536	*Burkholderia*	*multivorans*
Z0011_LB_E05_2_B04_A	GREEN	No	180	142	R-67536	*Burkholderia*	*multivorans*
Z0011_LB_E02_2_B06_B	GREEN	No	180	142	R-67536	*Burkholderia*	*multivorans*
Z0011_LB_E06_2_B04_B	GREEN	No	180	142	R-67536	*Burkholderia*	*multivorans*
Z0011_LB_D12_1_C01_B	GREEN	No	180	142	R-67536	*Burkholderia*	*multivorans*
Z0011_RB_B12_1_D07_B	GREEN	No	180	142	R-67536	*Burkholderia*	*multivorans*
Z0011_RB_C03_2_C11_A	GREEN	No	180	142	R-67536	*Burkholderia*	*multivorans*
Z0010_LO_F03_2_G02_A	GREEN	No	180	142	R-67121	*Burkholderia*	*multivorans*
Z0010_LO_G06_1_H03_B	GREEN	No	180	142	R-67121	*Burkholderia*	*multivorans*
Z0010_LO_F06_2_G03_B	GREEN	No	180	142	R-67121	*Burkholderia*	*multivorans*
Z0010_RO_G02_1_H07_B	GREEN	No	180	142	R-67121	*Burkholderia*	*multivorans*
Z0010_RO_F12_2_G12_B	GREEN	No	180	142	R-67121	*Burkholderia*	*multivorans*
Z0010_RO_F04_2_G08_B	GREEN	No	180	142	R-67121	*Burkholderia*	*multivorans*
Z0010_LO_E07_1_G04_A	GREEN	No	180	142	R-67121	*Burkholderia*	*multivorans*
Z0010_RO_E08_1_G10_B	GREEN	No	180	142	R-67121	*Burkholderia*	*multivorans*
Z0010_LO_G10_1_H05_B	GREEN	No	180	142	R-67121	*Burkholderia*	*multivorans*
Z0010_RO_D12_2_F12_B	GREEN	No	180	142	R-67121	*Burkholderia*	*multivorans*
Z0010_RO_F11_2_G12_A	GREEN	No	180	142	R-67121	*Burkholderia*	*multivorans*

Z0010_RO_F06_2_G09_B	GREEN	No	180	142	R-67121	*Burkholderia*	*multivorans*
Z0010_LO_F04_2_G02_B	GREEN	No	180	142	R-67121	*Burkholderia*	*multivorans*
Z0010_LO_E10_1_G05_B	GREEN	No	180	142	R-67121	*Burkholderia*	*multivorans*
Z0010_LO_G04_1_H02_B	GREEN	No	180	142	R-67121	*Burkholderia*	*multivorans*
Z0010_LO_F12_2_G06_B	GREEN	No	180	142	R-67121	*Burkholderia*	*multivorans*
Z0010_LO_F01_2_G01_A	GREEN	No	180	142	R-67121	*Burkholderia*	*multivorans*
Z0010_RO_E03_1_G08_A	GREEN	No	180	142	R-67121	*Burkholderia*	*multivorans*
Z0010_LO_G05_1_H03_A	GREEN	No	180	142	R-67121	*Burkholderia*	*multivorans*
Z0010_RO_E05_1_G09_A	GREEN	No	180	142	R-67121	*Burkholderia*	*multivorans*
Z0010_RO_E10_1_G11_B	GREEN	No	180	142	R-67121	*Burkholderia*	*multivorans*
Z0010_RO_F01_2_G07_A	GREEN	No	180	142	R-67121	*Burkholderia*	*multivorans*
Z0010_RO_F10_2_G11_B	GREEN	No	180	142	R-67121	*Burkholderia*	*multivorans*
Z0010_RO_E02_1_G07_B	GREEN	No	180	142	R-67121	*Burkholderia*	*multivorans*
Z0010_LO_F11_2_G06_A	GREEN	No	180	142	R-67121	*Burkholderia*	*multivorans*
Z0010_LO_E08_1_G04_B	GREEN	No	180	142	R-67121	*Burkholderia*	*multivorans*
Z0010_LO_F09_2_G05_A	GREEN	No	180	142	R-67121	*Burkholderia*	*multivorans*
Z0010_RO_E11_1_G12_A	GREEN	No	180	142	R-67121	*Burkholderia*	*multivorans*
Z0010_LO_G02_1_H01_B	GREEN	No	180	142	R-67121	*Burkholderia*	*multivorans*
Z0010_RO_G03_1_H08_A	GREEN	No	180	142	R-67121	*Burkholderia*	*multivorans*
Z0010_RO_G05_1_H09_A	GREEN	No	180	142	R-67121	*Burkholderia*	*multivorans*
Z0010_RO_G04_1_H08_B	GREEN	No	180	142	R-67121	*Burkholderia*	*multivorans*
Z0010_RO_A03_1_E08_A	GREEN	No	180	142	R-71089	*Burkholderia*	*multivorans*
Z0010_RO_A07_1_E10_A	GREEN	No	180	142	R-71089	*Burkholderia*	*multivorans*
Z0010_RO_A06_1_E09_B	GREEN	No	180	142	R-71089	*Burkholderia*	*multivorans*
Z0010_LO_A06_1_E03_B	GREEN	No	180	142	R-71089	*Burkholderia*	*multivorans*
Z0010_RO_A04_1_E08_B	GREEN	No	180	142	R-71089	*Burkholderia*	*multivorans*
Z0010_LO_A10_1_E05_B	GREEN	No	180	142	R-71089	*Burkholderia*	*multivorans*
Z0010_LO_B01_2_E01_A	GREEN	No	180	142	R-71089	*Burkholderia*	*multivorans*
Z0010_RB_H12_1_A07_B	GREEN	No	180	142	R-71089	*Burkholderia*	*multivorans*
Z0010_LO_B02_2_E01_B	GREEN	No	180	142	R-71089	*Burkholderia*	*multivorans*
Z0010_LO_A05_1_E03_A	GREEN	No	180	142	R-71089	*Burkholderia*	*multivorans*
Z0010_RB_H08_1_A09_B	GREEN	No	180	142	R-71089	*Burkholderia*	*multivorans*
Z0010_LO_A08_1_E04_B	GREEN	No	180	142	R-71089	*Burkholderia*	*multivorans*
Z0010_LO_A03_1_E02_A	GREEN	No	180	142	R-71089	*Burkholderia*	*multivorans*
Z0010_RO_A02_1_E07_B	GREEN	No	180	142	R-71089	*Burkholderia*	*multivorans*
Z0010_RO_A01_1_E07_A	GREEN	No	180	142	R-71089	*Burkholderia*	*multivorans*
Z0010_RB_H05_1_A10_A	GREEN	No	180	142	R-71089	*Burkholderia*	*multivorans*
Z0010_LO_A09_1_E05_A	GREEN	No	180	142	R-71089	*Burkholderia*	*multivorans*
Z0010_RO_A05_1_E09_A	GREEN	No	180	142	R-71089	*Burkholderia*	*multivorans*
Z0010_LO_A12_1_E06_B	GREEN	No	180	142	R-71089	*Burkholderia*	*multivorans*
Z0010_LO_A07_1_E04_A	GREEN	No	180	142	R-71089	*Burkholderia*	*multivorans*
Z0010_LO_A04_1_E02_B	GREEN	No	180	142	R-71089	*Burkholderia*	*multivorans*
Z0010_RB_H11_1_A07_A	GREEN	No	180	142	R-71089	*Burkholderia*	*multivorans*
Z0010_RB_H07_1_A09_A	GREEN	No	180	142	R-71089	*Burkholderia*	*multivorans*
Z0010_RB_H06_1_A10_B	GREEN	No	180	142	R-71089	*Burkholderia*	*multivorans*
Z0010_LO_A02_1_E01_B	GREEN	No	180	142	R-71089	*Burkholderia*	*multivorans*
Z0010_RB_H09_1_A08_A	GREEN	No	180	142	R-71089	*Burkholderia*	*multivorans*
Z0011_RO_A09_1_E11_A	GREEN	No	180	142	R-68768	*Burkholderia*	*multivorans*
Z0011_RO_B04_2_E08_B	GREEN	No	180	142	R-68768	*Burkholderia*	*multivorans*
Z0011_LO_B10_2_E05_B	GREEN	No	180	142	R-68768	*Burkholderia*	*multivorans*
Z0011_RO_A12_1_E12_B	GREEN	No	180	142	R-68768	*Burkholderia*	*multivorans*
Z0011_RO_A05_1_E09_A	GREEN	No	180	142	R-68768	*Burkholderia*	*multivorans*
Z0011_RO_A01_1_E07_A	GREEN	No	180	142	R-68768	*Burkholderia*	*multivorans*
Z0011_RO_A08_1_E10_B	GREEN	No	180	142	R-68768	*Burkholderia*	*multivorans*
Z0011_RO_A02_1_E07_B	GREEN	No	180	142	R-68768	*Burkholderia*	*multivorans*
Z0011_LO_B07_2_E04_A	GREEN	No	180	142	R-68768	*Burkholderia*	*multivorans*
Z0011_RO_B02_2_E07_B	GREEN	No	180	142	R-68768	*Burkholderia*	*multivorans*
Z0011_LB_A07_2_D03_A	GREEN	No	180	144	R-67258	*Burkholderia*	*multivorans*
Z0011_LO_H07_2_H04_A	GREEN	No	180	144	R-67258	*Burkholderia*	*multivorans*
Z0011_LB_A05_2_D04_A	GREEN	No	180	144	R-67258	*Burkholderia*	*multivorans*
Z0011_LB_A06_2_D04_B	GREEN	No	180	144	R-67258	*Burkholderia*	*multivorans*
Z0011_LO_H08_2_H04_B	GREEN	No	180	144	R-67258	*Burkholderia*	*multivorans*
Z0011_LB_B03_1_D05_A	GREEN	No	180	144	R-67258	*Burkholderia*	*multivorans*
Z0011_LB_B05_1_D04_A	GREEN	No	180	144	R-67258	*Burkholderia*	*multivorans*
Z0011_LB_A08_2_D03_B	GREEN	No	180	144	R-67258	*Burkholderia*	*multivorans*
Z0011_RB_A03_2_D11_A	GREEN	No	180	144	R-67258	*Burkholderia*	*multivorans*
Z0011_LB_B04_1_D05_B	GREEN	No	180	144	R-67258	*Burkholderia*	*multivorans*
Z0011_RB_A02_2_D12_B	GREEN	No	180	144	R-67258	*Burkholderia*	*multivorans*
Z0011_RB_A01_2_D12_A	GREEN	No	180	144	R-67258	*Burkholderia*	*multivorans*
Z0011_LB_B02_1_D06_B	GREEN	No	180	144	R-67258	*Burkholderia*	*multivorans*

Z0011_LB_B01_1_D06_A	GREEN	No	180	144	R-67258	*Burkholderia*	*multivorans*
Z0010_LO_F05_2_G03_A	GREEN	No	180	144	R-67196	*Burkholderia*	*multivorans*
Z0010_LO_G07_1_H04_A	GREEN	No	180	144	R-67196	*Burkholderia*	*multivorans*
Z0010_LO_E11_1_G06_A	GREEN	No	180	144	R-67196	*Burkholderia*	*multivorans*
Z0010_RO_D09_2_F11_A	GREEN	No	180	144	R-67196	*Burkholderia*	*multivorans*
Z0010_LO_F02_2_G01_B	GREEN	No	180	144	R-67196	*Burkholderia*	*multivorans*
Z0010_RO_E09_1_G11_A	GREEN	No	180	144	R-67196	*Burkholderia*	*multivorans*
Z0010_RO_E12_1_G12_B	GREEN	No	180	144	R-67196	*Burkholderia*	*multivorans*
Z0010_LO_E03_1_G02_A	GREEN	No	180	144	R-67196	*Burkholderia*	*multivorans*
Z0010_RO_D10_2_F11_B	GREEN	No	180	144	R-67196	*Burkholderia*	*multivorans*
Z0010_RO_F03_2_G08_A	GREEN	No	180	144	R-67196	*Burkholderia*	*multivorans*
Z0010_RO_E06_1_G09_B	GREEN	No	180	144	R-67196	*Burkholderia*	*multivorans*
Z0010_LO_G09_1_H05_A	GREEN	No	180	144	R-67196	*Burkholderia*	*multivorans*
Z0010_LO_G08_1_H04_B	GREEN	No	180	144	R-67196	*Burkholderia*	*multivorans*
Z0010_LO_E09_1_G05_A	GREEN	No	180	144	R-67196	*Burkholderia*	*multivorans*
Z0010_RO_E07_1_G10_A	GREEN	No	180	144	R-67196	*Burkholderia*	*multivorans*
Z0010_LO_G01_1_H01_A	GREEN	No	180	144	R-67196	*Burkholderia*	*multivorans*
Z0010_LO_E12_1_G06_B	GREEN	No	180	144	R-67196	*Burkholderia*	*multivorans*
Z0010_RO_E01_1_G07_A	GREEN	No	180	144	R-67196	*Burkholderia*	*multivorans*
Z0010_LO_F10_2_G05_B	GREEN	No	180	144	R-67196	*Burkholderia*	*multivorans*
Z0010_RO_D05_2_F09_A	GREEN	No	180	144	R-67196	*Burkholderia*	*multivorans*
Z0010_RO_D02_2_F07_B	GREEN	No	180	144	R-67196	*Burkholderia*	*multivorans*
Z0010_LO_D08_2_F04_B	GREEN	No	180	144	R-67196	*Burkholderia*	*multivorans*
Z0010_RO_E04_1_G08_B	GREEN	No	180	144	R-67196	*Burkholderia*	*multivorans*
Z0010_LO_E01_1_G01_A	GREEN	No	180	144	R-67196	*Burkholderia*	*multivorans*
Z0010_LO_D10_2_F05_B	GREEN	No	180	144	R-67196	*Burkholderia*	*multivorans*
Z0010_LO_E05_1_G03_A	GREEN	No	180	144	R-67196	*Burkholderia*	*multivorans*
Z0010_RO_D11_2_F12_A	GREEN	No	180	144	R-67196	*Burkholderia*	*multivorans*
Z0010_LO_F07_2_G04_A	GREEN	No	180	144	R-67196	*Burkholderia*	*multivorans*
Z0010_LO_E04_1_G02_B	GREEN	No	180	144	R-67196	*Burkholderia*	*multivorans*
Z0010_RO_D03_2_F08_A	GREEN	No	180	144	R-67196	*Burkholderia*	*multivorans*
Z0010_LO_F08_2_G04_B	GREEN	No	180	144	R-67196	*Burkholderia*	*multivorans*
Z0010_LO_A11_1_E06_A	GREEN	Yes	181	142	R-71089	*Burkholderia*	*multivorans*
Z0010_RB_H10_1_A08_B	GREEN	No	181	142	R-71089	*Burkholderia*	*multivorans*
Z0011_LO_B09_2_E05_A	GREEN	Yes	182	142	R-68768	*Burkholderia*	*multivorans*
Z0011_RO_A11_1_E12_A	GREEN	No	182	142	R-68768	*Burkholderia*	*multivorans*
Z0011_LO_B12_2_E06_B	GREEN	No	182	142	R-68768	*Burkholderia*	*multivorans*
Z0011_RO_A07_1_E10_A	GREEN	No	182	142	R-68768	*Burkholderia*	*multivorans*
Z0011_LO_B08_2_E04_B	GREEN	No	182	142	R-68768	*Burkholderia*	*multivorans*
Z0011_RB_H11_1_A07_A	GREEN	No	182	142	R-68768	*Burkholderia*	*multivorans*
Z0011_RO_A03_1_E08_A	GREEN	No	182	142	R-68768	*Burkholderia*	*multivorans*
Z0011_LO_C03_1_F02_A	GREEN	No	182	142	R-68768	*Burkholderia*	*multivorans*
Z0011_RO_B05_2_E09_A	GREEN	No	182	142	R-68768	*Burkholderia*	*multivorans*
Z0011_LO_C04_1_F02_B	GREEN	No	182	142	R-68768	*Burkholderia*	*multivorans*
Z0011_LO_C01_1_F01_A	GREEN	No	182	142	R-68768	*Burkholderia*	*multivorans*
Z0011_LO_C02_1_F01_B	GREEN	No	182	142	R-68768	*Burkholderia*	*multivorans*
Z0011_RO_A10_1_E11_B	GREEN	No	182	142	R-68768	*Burkholderia*	*multivorans*
Z0011_LO_B11_2_E06_A	GREEN	No	182	142	R-68768	*Burkholderia*	*multivorans*
Z0011_RO_A06_1_E09_B	GREEN	No	182	142	R-68768	*Burkholderia*	*multivorans*
Z0011_RO_B01_2_E07_A	GREEN	No	182	142	R-68768	*Burkholderia*	*multivorans*
Z0011_RO_A04_1_E08_B	GREEN	No	182	142	R-68768	*Burkholderia*	*multivorans*
Z0011_LB_A12_2_D01_B	GREEN	Yes	183	144	R-67258	*Burkholderia*	*multivorans*
Z0011_RB_C06_2_C10_B	GREEN	No	183	142	R-67536	*Burkholderia*	*multivorans*
Z0011_RB_B10_1_D08_B	GREEN	No	183	142	R-67536	*Burkholderia*	*multivorans*
Z0011_RB_B09_1_D08_A	GREEN	No	183	142	R-67536	*Burkholderia*	*multivorans*
Z0011_RB_C04_2_C11_B	GREEN	No	183	142	R-67536	*Burkholderia*	*multivorans*
Z0011_RB_C05_2_C10_A	GREEN	No	183	142	R-67536	*Burkholderia*	*multivorans*
Z0011_LB_A09_2_D02_A	GREEN	No	183	144	R-67258	*Burkholderia*	*multivorans*
Z0011_LB_A02_2_D06_B	GREEN	No	183	144	R-67258	*Burkholderia*	*multivorans*
Z0011_LB_A03_2_D05_A	GREEN	No	183	144	R-67258	*Burkholderia*	*multivorans*
Z0011_LB_A04_2_D05_B	GREEN	No	183	144	R-67258	*Burkholderia*	*multivorans*
Z0011_LO_H05_2_H03_A	GREEN	No	183	144	R-67258	*Burkholderia*	*multivorans*
Z0011_LB_A10_2_D02_B	GREEN	No	183	144	R-67258	*Burkholderia*	*multivorans*
Z0011_LO_H11_2_H06_A	GREEN	No	183	144	R-67258	*Burkholderia*	*multivorans*
Z0011_LO_H12_2_H06_B	GREEN	No	183	144	R-67258	*Burkholderia*	*multivorans*
Z0011_RO_B03_2_E08_A	GREEN	No	183	144	R-67258	*Burkholderia*	*multivorans*
Z0011_LO_H09_2_H05_A	GREEN	No	183	144	R-67258	*Burkholderia*	*multivorans*
Z0011_LB_A11_2_D01_A	GREEN	No	183	144	R-67258	*Burkholderia*	*multivorans*
Z0011_LB_A01_2_D06_A	GREEN	No	183	144	R-67258	*Burkholderia*	*multivorans*
Z0011_LO_H10_2_H05_B	GREEN	No	183	144	R-67258	*Burkholderia*	*multivorans*

Z0011_LO_H04_2_H02_B	GREEN	No	183	144	R-67258	*Burkholderia*	*multivorans*
Z0011_LO_H06_2_H03_B	GREEN	No	183	144	R-67258	*Burkholderia*	*multivorans*
Z0011_LO_D03_2_F02_A	GREEN	No	183	144	R-67258	*Burkholderia*	*multivorans*
Z0023_RO_B01_2_E07_A	GREEN	Yes	184	123	LMG 6451	*Bacteroides*	*ureolyticus*
Z0023_LO_C12_1_F06_B	GREEN	No	184	123	LMG 6451	*Bacteroides*	*ureolyticus*
Z0023_RB_H11_1_A07_A	GREEN	Yes	185	123	LMG 6451	*Bacteroides*	*ureolyticus*
Z0023_LO_C07_1_F04_A	GREEN	No	185	123	LMG 6451	*Bacteroides*	*ureolyticus*
Z0023_LO_C05_1_F03_A	GREEN	No	185	123	LMG 6451	*Bacteroides*	*ureolyticus*
Z0023_RO_D12_2_F12_B	GREEN	No	185	123	LMG 6451	*Bacteroides*	*ureolyticus*
Z0023_LO_D03_2_F02_A	GREEN	No	185	123	LMG 6451	*Bacteroides*	*ureolyticus*
Z0023_RO_C08_1_F10_B	GREEN	No	185	123	LMG 6451	*Bacteroides*	*ureolyticus*
Z0023_RO_D08_2_F10_B	GREEN	No	185	123	LMG 6451	*Bacteroides*	*ureolyticus*
Z0023_LO_C10_1_F05_B	GREEN	No	185	123	LMG 6451	*Bacteroides*	*ureolyticus*
Z0023_LO_D10_2_F05_B	GREEN	No	185	123	LMG 6451	*Bacteroides*	*ureolyticus*
Z0023_RO_B02_2_E07_B	GREEN	No	185	123	LMG 6451	*Bacteroides*	*ureolyticus*
Z0023_LO_B08_2_E04_B	GREEN	No	185	123	LMG 6451	*Bacteroides*	*ureolyticus*
Z0023_LO_D04_2_F02_B	GREEN	No	185	123	LMG 6451	*Bacteroides*	*ureolyticus*
Z0023_LO_B11_2_E06_A	GREEN	No	185	123	LMG 6451	*Bacteroides*	*ureolyticus*
Z0023_LO_B03_2_E02_A	GREEN	No	185	123	LMG 6451	*Bacteroides*	*ureolyticus*
Z0023_RO_A06_1_E09_B	GREEN	No	185	123	LMG 6451	*Bacteroides*	*ureolyticus*
Z0023_LO_C11_1_F06_A	GREEN	No	185	123	LMG 6451	*Bacteroides*	*ureolyticus*
Z0023_RO_D09_2_F11_A	GREEN	No	185	123	LMG 6451	*Bacteroides*	*ureolyticus*
Z0023_RO_A10_1_E11_B	GREEN	No	185	123	LMG 6451	*Bacteroides*	*ureolyticus*
Z0023_RO_D05_2_F09_A	GREEN	No	185	123	LMG 6451	*Bacteroides*	*ureolyticus*
Z0023_RO_D03_2_F08_A	GREEN	No	185	123	LMG 6451	*Bacteroides*	*ureolyticus*
Z0023_RO_D04_2_F08_B	GREEN	No	185	123	LMG 6451	*Bacteroides*	*ureolyticus*
Z0023_RO_A07_1_E10_A	GREEN	No	185	123	LMG 6451	*Bacteroides*	*ureolyticus*
Z0023_RO_A01_1_E07_A	GREEN	No	185	123	LMG 6451	*Bacteroides*	*ureolyticus*
Z0023_LO_C09_1_F05_A	GREEN	No	185	123	LMG 6451	*Bacteroides*	*ureolyticus*
Z0023_RO_C09_1_F11_A	GREEN	No	185	123	LMG 6451	*Bacteroides*	*ureolyticus*
Z0023_LO_D11_2_F06_A	GREEN	No	185	123	LMG 6451	*Bacteroides*	*ureolyticus*
Z0023_RO_D07_2_F10_A	GREEN	No	185	123	LMG 6451	*Bacteroides*	*ureolyticus*
Z0023_LO_C06_1_F03_B	GREEN	No	185	123	LMG 6451	*Bacteroides*	*ureolyticus*
Z0023_RO_C05_1_F09_A	GREEN	No	185	123	LMG 6451	*Bacteroides*	*ureolyticus*
Z0023_RO_D06_2_F09_B	GREEN	No	185	123	LMG 6451	*Bacteroides*	*ureolyticus*
Z0013_RO_C02_1_F07_B	GREEN	Yes	186	17	LMG 1408	*Gluconobacter*	*oxydans*
Z0013_RB_B12_1_D07_B	GREEN	No	186	17	LMG 1408	*Gluconobacter*	*oxydans*
Z0013_RB_B11_1_D07_A	GREEN	No	186	17	LMG 1408	*Gluconobacter*	*oxydans*
Z0013_LO_C01_1_F01_A	GREEN	No	186	17	LMG 1408	*Gluconobacter*	*oxydans*
Z0013_RB_C01_2_C12_A	GREEN	No	186	17	LMG 1408	*Gluconobacter*	*oxydans*
Z0013_LO_B10_2_E05_B	GREEN	No	186	17	LMG 1408	*Gluconobacter*	*oxydans*
Z0013_LO_C03_1_F02_A	GREEN	No	186	17	LMG 1408	*Gluconobacter*	*oxydans*
Z0013_RO_C04_1_F08_B	GREEN	No	186	17	LMG 1408	*Gluconobacter*	*oxydans*
Z0013_LO_C02_1_F01_B	GREEN	No	186	17	LMG 1408	*Gluconobacter*	*oxydans*
Z0013_RB_B07_1_D09_A	GREEN	No	186	17	LMG 1408	*Gluconobacter*	*oxydans*
Z0013_RO_C06_1_F09_B	GREEN	No	186	17	LMG 1408	*Gluconobacter*	*oxydans*
Z0013_LB_B12_1_D01_B	GREEN	No	186	17	LMG 1408	*Gluconobacter*	*oxydans*
Z0013_RO_C05_1_F09_A	GREEN	No	186	17	LMG 1408	*Gluconobacter*	*oxydans*
Z0013_LO_C04_1_F02_B	GREEN	No	186	17	LMG 1408	*Gluconobacter*	*oxydans*
Z0013_LO_B11_2_E06_A	GREEN	No	186	17	LMG 1408	*Gluconobacter*	*oxydans*
Z0013_LB_B11_1_D01_A	GREEN	No	186	17	LMG 1408	*Gluconobacter*	*oxydans*
Z0013_LB_B09_1_D02_A	GREEN	No	186	17	LMG 1408	*Gluconobacter*	*oxydans*
Z0013_RO_C07_1_F10_A	GREEN	No	186	17	LMG 1408	*Gluconobacter*	*oxydans*
Z0013_RO_B12_2_E12_B	GREEN	No	186	17	LMG 1408	*Gluconobacter*	*oxydans*
Z0013_RO_B08_2_E10_B	GREEN	No	186	17	LMG 1408	*Gluconobacter*	*oxydans*
Z0013_RB_B08_1_D09_B	GREEN	No	186	17	LMG 1408	*Gluconobacter*	*oxydans*
Z0013_RO_B10_2_E11_B	GREEN	No	186	17	LMG 1408	*Gluconobacter*	*oxydans*
Z0013_RB_B09_1_D08_A	GREEN	No	186	17	LMG 1408	*Gluconobacter*	*oxydans*
Z0013_LB_B10_1_D02_B	GREEN	No	186	17	LMG 1408	*Gluconobacter*	*oxydans*
Z0013_LO_C05_1_F03_A	GREEN	No	186	17	LMG 1408	*Gluconobacter*	*oxydans*
Z0013_RO_B09_2_E11_A	GREEN	No	186	17	LMG 1408	*Gluconobacter*	*oxydans*
Z0013_RO_C01_1_F07_A	GREEN	No	186	17	LMG 1408	*Gluconobacter*	*oxydans*
Z0013_LO_B09_2_E05_A	GREEN	No	186	17	LMG 1408	*Gluconobacter*	*oxydans*
Z0013_LO_B12_2_E06_B	GREEN	No	186	17	LMG 1408	*Gluconobacter*	*oxydans*
Z0013_RB_B10_1_D08_B	GREEN	No	186	17	LMG 1408	*Gluconobacter*	*oxydans*
Z0013_RO_C03_1_F08_A	GREEN	No	186	17	LMG 1408	*Gluconobacter*	*oxydans*
Z0013_RO_B11_2_E12_A	GREEN	No	186	17	LMG 1408	*Gluconobacter*	*oxydans*
Z0019_LB_B07_1_D03_A	GREEN	Yes	187	78	LMG 25420	*Echinimonas*	*agarilytica*
Z0019_RB_A09_2_D08_A	GREEN	No	187	78	LMG 25420	*Echinimonas*	*agarilytica*
Z0019_RB_A07_2_D09_A	GREEN	No	187	78	LMG 25420	*Echinimonas*	*agarilytica*

Z0019_LB_B11_1_D01_A	GREEN	No	187	78	LMG 25420	*Echinimonas*	*agarilytica*
Z0019_RB_A12_2_D07_B	GREEN	No	187	78	LMG 25420	*Echinimonas*	*agarilytica*
Z0019_LB_B08_1_D03_B	GREEN	No	187	78	LMG 25420	*Echinimonas*	*agarilytica*
Z0019_RB_B03_1_D11_A	GREEN	No	187	78	LMG 25420	*Echinimonas*	*agarilytica*
Z0019_LB_B12_1_D01_B	GREEN	No	187	78	LMG 25420	*Echinimonas*	*agarilytica*
Z0019_LB_B04_1_D05_B	GREEN	No	187	78	LMG 25420	*Echinimonas*	*agarilytica*
Z0019_RB_B02_1_D12_B	GREEN	No	187	78	LMG 25420	*Echinimonas*	*agarilytica*
Z0019_LB_C02_2_C06_B	GREEN	No	187	78	LMG 25420	*Echinimonas*	*agarilytica*
Z0019_LB_B06_1_D04_B	GREEN	No	187	78	LMG 25420	*Echinimonas*	*agarilytica*
Z0019_LB_C04_2_C05_B	GREEN	No	187	78	LMG 25420	*Echinimonas*	*agarilytica*
Z0019_RB_A04_2_D11_B	GREEN	No	187	78	LMG 25420	*Echinimonas*	*agarilytica*
Z0019_RB_A03_2_D11_A	GREEN	No	187	78	LMG 25420	*Echinimonas*	*agarilytica*
Z0019_RB_B05_1_D10_A	GREEN	No	187	78	LMG 25420	*Echinimonas*	*agarilytica*
Z0019_RB_A06_2_D10_B	GREEN	No	187	78	LMG 25420	*Echinimonas*	*agarilytica*
Z0019_RB_B04_1_D11_B	GREEN	No	187	78	LMG 25420	*Echinimonas*	*agarilytica*
Z0019_LB_B05_1_D04_A	GREEN	No	187	78	LMG 25420	*Echinimonas*	*agarilytica*
Z0019_RB_A05_2_D10_A	GREEN	No	187	78	LMG 25420	*Echinimonas*	*agarilytica*
Z0019_LB_C01_2_C06_A	GREEN	No	187	78	LMG 25420	*Echinimonas*	*agarilytica*
Z0019_RB_B01_1_D12_A	GREEN	No	187	78	LMG 25420	*Echinimonas*	*agarilytica*
Z0019_LB_B02_1_D06_B	GREEN	No	187	78	LMG 25420	*Echinimonas*	*agarilytica*
Z0019_LB_B01_1_D06_A	GREEN	No	187	78	LMG 25420	*Echinimonas*	*agarilytica*
Z0019_RB_A11_2_D07_A	GREEN	No	187	78	LMG 25420	*Echinimonas*	*agarilytica*
Z0019_RB_A02_2_D12_B	GREEN	No	187	78	LMG 25420	*Echinimonas*	*agarilytica*
Z0019_LB_C03_2_C05_A	GREEN	No	187	78	LMG 25420	*Echinimonas*	*agarilytica*
Z0019_LB_B03_1_D05_A	GREEN	No	187	78	LMG 25420	*Echinimonas*	*agarilytica*
Z0019_LB_B10_1_D02_B	GREEN	No	187	78	LMG 25420	*Echinimonas*	*agarilytica*
Z0019_RB_A08_2_D09_B	GREEN	No	187	78	LMG 25420	*Echinimonas*	*agarilytica*
Z0019_RB_A10_2_D08_B	GREEN	No	187	78	LMG 25420	*Echinimonas*	*agarilytica*
Z0019_LB_B09_1_D02_A	GREEN	Yes	188	78	LMG 25420	*Echinimonas*	*agarilytica*
Z0018_LB_E07_2_B03_A	GREEN	Yes	189	70	LMG 24411	*Bhargavaea*	*cecembensis*
Z0018_RB_D07_1_C09_A	GREEN	No	189	70	LMG 24411	*Bhargavaea*	*cecembensis*
Z0018_LB_E09_2_B02_A	GREEN	No	189	70	LMG 24411	*Bhargavaea*	*cecembensis*
Z0018_LB_E08_2_B03_B	GREEN	No	189	70	LMG 24411	*Bhargavaea*	*cecembensis*
Z0018_RB_D02_1_C12_B	GREEN	No	189	70	LMG 24411	*Bhargavaea*	*cecembensis*
Z0018_RB_D12_1_C07_B	GREEN	No	189	70	LMG 24411	*Bhargavaea*	*cecembensis*
Z0018_RB_D06_1_C10_B	GREEN	No	189	70	LMG 24411	*Bhargavaea*	*cecembensis*
Z0018_LB_E10_2_B02_B	GREEN	No	189	70	LMG 24411	*Bhargavaea*	*cecembensis*
Z0018_RB_D08_1_C09_B	GREEN	No	189	70	LMG 24411	*Bhargavaea*	*cecembensis*
Z0018_RB_D03_1_C11_A	GREEN	No	189	70	LMG 24411	*Bhargavaea*	*cecembensis*
Z0018_LB_E04_2_B05_B	GREEN	No	189	70	LMG 24411	*Bhargavaea*	*cecembensis*
Z0018_RB_E02_2_B12_B	GREEN	No	189	70	LMG 24411	*Bhargavaea*	*cecembensis*
Z0018_LB_E11_2_B01_A	GREEN	No	189	70	LMG 24411	*Bhargavaea*	*cecembensis*
Z0018_RB_E01_2_B12_A	GREEN	No	189	70	LMG 24411	*Bhargavaea*	*cecembensis*
Z0018_RB_D09_1_C08_A	GREEN	No	189	70	LMG 24411	*Bhargavaea*	*cecembensis*
Z0018_RB_D11_1_C07_A	GREEN	No	189	70	LMG 24411	*Bhargavaea*	*cecembensis*
Z0018_RB_D10_1_C08_B	GREEN	No	189	70	LMG 24411	*Bhargavaea*	*cecembensis*
Z0018_LB_D11_1_C01_A	GREEN	No	189	70	LMG 24411	*Bhargavaea*	*cecembensis*
Z0018_LB_E06_2_B04_B	GREEN	No	189	70	LMG 24411	*Bhargavaea*	*cecembensis*
Z0018_LB_E03_2_B05_A	GREEN	No	189	70	LMG 24411	*Bhargavaea*	*cecembensis*
Z0018_RB_E03_2_B11_A	GREEN	No	189	70	LMG 24411	*Bhargavaea*	*cecembensis*
Z0018_RB_D04_1_C11_B	GREEN	No	189	70	LMG 24411	*Bhargavaea*	*cecembensis*
Z0018_LB_F03_1_B05_A	GREEN	No	189	70	LMG 24411	*Bhargavaea*	*cecembensis*
Z0018_LB_D12_1_C01_B	GREEN	No	189	70	LMG 24411	*Bhargavaea*	*cecembensis*
Z0018_LB_E12_2_B01_B	GREEN	No	189	70	LMG 24411	*Bhargavaea*	*cecembensis*
Z0018_LB_E05_2_B04_A	GREEN	No	189	70	LMG 24411	*Bhargavaea*	*cecembensis*
Z0018_LB_D10_1_C02_B	GREEN	No	189	70	LMG 24411	*Bhargavaea*	*cecembensis*
Z0018_RB_D05_1_C10_A	GREEN	No	189	70	LMG 24411	*Bhargavaea*	*cecembensis*
Z0018_LB_F02_1_B06_B	GREEN	No	189	70	LMG 24411	*Bhargavaea*	*cecembensis*
Z0018_LB_F01_1_B06_A	GREEN	No	189	70	LMG 24411	*Bhargavaea*	*cecembensis*
Z0018_LB_E01_2_B06_A	GREEN	No	189	70	LMG 24411	*Bhargavaea*	*cecembensis*
Z0011_LO_D01_2_F01_A	GREEN	Yes	190	1	LMG 1041	*Acinetobacter*	*baumannii*
Z0011_RO_B12_2_E12_B	GREEN	No	190	1	LMG 1041	*Acinetobacter*	*baumannii*
Z0011_RO_D02_2_F07_B	GREEN	No	190	1	LMG 1041	*Acinetobacter*	*baumannii*
Z0011_LO_C09_1_F05_A	GREEN	No	190	1	LMG 1041	*Acinetobacter*	*baumannii*
Z0011_RO_C06_1_F09_B	GREEN	No	190	1	LMG 1041	*Acinetobacter*	*baumannii*
Z0011_RO_E02_1_G07_B	GREEN	No	190	1	LMG 1041	*Acinetobacter*	*baumannii*
Z0011_RO_C01_1_F07_A	GREEN	No	190	1	LMG 1041	*Acinetobacter*	*baumannii*
Z0011_RO_B08_2_E10_B	GREEN	No	190	1	LMG 1041	*Acinetobacter*	*baumannii*
Z0011_LO_C07_1_F04_A	GREEN	No	190	1	LMG 1041	*Acinetobacter*	*baumannii*
Z0011_RO_C02_1_F07_B	GREEN	No	190	1	LMG 1041	*Acinetobacter*	*baumannii*

Z0011_LO_C06_1_F03_B	GREEN	No	190	1	LMG 1041	*Acinetobacter*	*baumannii*
Z0011_RO_B09_2_E11_A	GREEN	No	190	1	LMG 1041	*Acinetobacter*	*baumannii*
Z0011_RO_D09_2_F11_A	GREEN	No	190	1	LMG 1041	*Acinetobacter*	*baumannii*
Z0011_LO_D12_2_F06_B	GREEN	No	190	1	LMG 1041	*Acinetobacter*	*baumannii*
Z0011_LO_C08_1_F04_B	GREEN	No	190	1	LMG 1041	*Acinetobacter*	*baumannii*
Z0011_RO_D10_2_F11_B	GREEN	No	190	1	LMG 1041	*Acinetobacter*	*baumannii*
Z0011_LO_D11_2_F06_A	GREEN	No	190	1	LMG 1041	*Acinetobacter*	*baumannii*
Z0011_LO_C11_1_F06_A	GREEN	No	190	1	LMG 1041	*Acinetobacter*	*baumannii*
Z0011_LO_D10_2_F05_B	GREEN	No	190	1	LMG 1041	*Acinetobacter*	*baumannii*
Z0011_LO_D04_2_F02_B	GREEN	No	190	1	LMG 1041	*Acinetobacter*	*baumannii*
Z0011_RO_C10_1_F11_B	GREEN	No	190	1	LMG 1041	*Acinetobacter*	*baumannii*
Z0011_LO_C12_1_F06_B	GREEN	No	190	1	LMG 1041	*Acinetobacter*	*baumannii*
Z0011_RO_C11_1_F12_A	GREEN	No	190	1	LMG 1041	*Acinetobacter*	*baumannii*
Z0011_LO_D06_2_F03_B	GREEN	No	190	1	LMG 1041	*Acinetobacter*	*baumannii*
Z0011_RO_C07_1_F10_A	GREEN	No	190	1	LMG 1041	*Acinetobacter*	*baumannii*
Z0011_LO_D07_2_F04_A	GREEN	No	190	1	LMG 1041	*Acinetobacter*	*baumannii*
Z0011_RO_B11_2_E12_A	GREEN	No	190	1	LMG 1041	*Acinetobacter*	*baumannii*
Z0011_LO_C05_1_F03_A	GREEN	No	190	1	LMG 1041	*Acinetobacter*	*baumannii*
Z0011_LO_D05_2_F03_A	GREEN	No	190	1	LMG 1041	*Acinetobacter*	*baumannii*
Z0011_RO_D03_2_F08_A	GREEN	No	190	1	LMG 1041	*Acinetobacter*	*baumannii*
Z0011_LO_C10_1_F05_B	GREEN	No	190	1	LMG 1041	*Acinetobacter*	*baumannii*
Z0011_RO_D04_2_F08_B	GREEN	No	190	1	LMG 1041	*Acinetobacter*	*baumannii*
Z0012_RO_H02_2_H07_B	GREEN	Yes	191	10	LMG 12553	*Sphingobium*	*xanthum*
Z0012_RB_A03_2_D11_A	GREEN	No	191	10	LMG 12553	*Sphingobium*	*xanthum*
Z0012_LO_H12_2_H06_B	GREEN	No	191	10	LMG 12553	*Sphingobium*	*xanthum*
Z0012_RO_H04_2_H08_B	GREEN	No	191	10	LMG 12553	*Sphingobium*	*xanthum*
Z0012_LO_G06_1_H03_B	GREEN	No	191	10	LMG 12553	*Sphingobium*	*xanthum*
Z0012_RO_F06_2_G09_B	GREEN	No	191	10	LMG 12553	*Sphingobium*	*xanthum*
Z0012_LO_H11_2_H06_A	GREEN	No	191	10	LMG 12553	*Sphingobium*	*xanthum*
Z0012_RO_G11_1_H12_A	GREEN	No	191	10	LMG 12553	*Sphingobium*	*xanthum*
Z0012_RO_G12_1_H12_B	GREEN	No	191	10	LMG 12553	*Sphingobium*	*xanthum*
Z0012_LO_H04_2_H02_B	GREEN	No	191	10	LMG 12553	*Sphingobium*	*xanthum*
Z0012_RO_G07_1_H10_A	GREEN	No	191	10	LMG 12553	*Sphingobium*	*xanthum*
Z0012_RO_G09_1_H11_A	GREEN	No	191	10	LMG 12553	*Sphingobium*	*xanthum*
Z0012_RO_G08_1_H10_B	GREEN	No	191	10	LMG 12553	*Sphingobium*	*xanthum*
Z0012_LO_G05_1_H03_A	GREEN	No	191	10	LMG 12553	*Sphingobium*	*xanthum*
Z0012_LO_H08_2_H04_B	GREEN	No	191	10	LMG 12553	*Sphingobium*	*xanthum*
Z0012_LO_G10_1_H05_B	GREEN	No	191	10	LMG 12553	*Sphingobium*	*xanthum*
Z0012_RO_H01_2_H07_A	GREEN	No	191	10	LMG 12553	*Sphingobium*	*xanthum*
Z0012_RB_A01_2_D12_A	GREEN	No	191	10	LMG 12553	*Sphingobium*	*xanthum*
Z0012_LO_H05_2_H03_A	GREEN	No	191	10	LMG 12553	*Sphingobium*	*xanthum*
Z0012_LO_G12_1_H06_B	GREEN	No	191	10	LMG 12553	*Sphingobium*	*xanthum*
Z0012_RB_A02_2_D12_B	GREEN	No	191	10	LMG 12553	*Sphingobium*	*xanthum*
Z0012_RO_G01_1_H07_A	GREEN	No	191	10	LMG 12553	*Sphingobium*	*xanthum*
Z0012_LO_H10_2_H05_B	GREEN	No	191	10	LMG 12553	*Sphingobium*	*xanthum*
Z0012_RO_F12_2_G12_B	GREEN	No	191	10	LMG 12553	*Sphingobium*	*xanthum*
Z0012_RO_F07_2_G10_A	GREEN	No	191	10	LMG 12553	*Sphingobium*	*xanthum*
Z0012_RO_H03_2_H08_A	GREEN	No	191	10	LMG 12553	*Sphingobium*	*xanthum*
Z0012_LO_H03_2_H02_A	GREEN	No	191	10	LMG 12553	*Sphingobium*	*xanthum*
Z0012_LO_G07_1_H04_A	GREEN	No	191	10	LMG 12553	*Sphingobium*	*xanthum*
Z0012_LO_G11_1_H06_A	GREEN	No	191	10	LMG 12553	*Sphingobium*	*xanthum*
Z0012_RO_G10_1_H11_B	GREEN	No	191	10	LMG 12553	*Sphingobium*	*xanthum*
Z0012_RO_G02_1_H07_B	GREEN	No	191	10	LMG 12553	*Sphingobium*	*xanthum*
Z0012_LO_H09_2_H05_A	GREEN	No	191	10	LMG 12553	*Sphingobium*	*xanthum*
Z0023_LB_A12_2_D01_B	GREEN	Yes	192	115	LMG 4051	*Deinococcus*	*radiodurans*
Z0023_LB_A11_2_D01_A	GREEN	No	192	115	LMG 4051	*Deinococcus*	*radiodurans*
Z0023_RB_H05_1_A10_A	GREEN	No	192	115	LMG 4051	*Deinococcus*	*radiodurans*
Z0023_LB_A07_2_D03_A	GREEN	No	192	115	LMG 4051	*Deinococcus*	*radiodurans*
Z0023_LB_A01_2_D06_A	GREEN	No	192	115	LMG 4051	*Deinococcus*	*radiodurans*
Z0023_RB_A06_2_D10_B	GREEN	No	192	115	LMG 4051	*Deinococcus*	*radiodurans*
Z0023_RO_B04_2_E08_B	GREEN	No	192	115	LMG 4051	*Deinococcus*	*radiodurans*
Z0023_RB_A09_2_D08_A	GREEN	No	192	115	LMG 4051	*Deinococcus*	*radiodurans*
Z0023_RB_A01_2_D12_A	GREEN	No	192	115	LMG 4051	*Deinococcus*	*radiodurans*
Z0023_RB_A07_2_D09_A	GREEN	No	192	115	LMG 4051	*Deinococcus*	*radiodurans*
Z0023_LB_A06_2_D04_B	GREEN	No	192	115	LMG 4051	*Deinococcus*	*radiodurans*
Z0023_RB_A03_2_D11_A	GREEN	No	192	115	LMG 4051	*Deinococcus*	*radiodurans*
Z0023_RO_B11_2_E12_A	GREEN	No	192	115	LMG 4051	*Deinococcus*	*radiodurans*
Z0023_RB_A05_2_D10_A	GREEN	No	192	115	LMG 4051	*Deinococcus*	*radiodurans*
Z0023_LB_A10_2_D02_B	GREEN	No	192	115	LMG 4051	*Deinococcus*	*radiodurans*
Z0023_LB_B03_1_D05_A	GREEN	No	192	115	LMG 4051	*Deinococcus*	*radiodurans*

Z0023_RO_B06_2_E09_B	GREEN	No	192	115	LMG 4051	*Deinococcus*	*radiodurans*
Z0023_LB_A09_2_D02_A	GREEN	No	192	115	LMG 4051	*Deinococcus*	*radiodurans*
Z0023_LB_B02_1_D06_B	GREEN	No	192	115	LMG 4051	*Deinococcus*	*radiodurans*
Z0023_LB_B04_1_D05_B	GREEN	No	192	115	LMG 4051	*Deinococcus*	*radiodurans*
Z0023_LB_A02_2_D06_B	GREEN	No	192	115	LMG 4051	*Deinococcus*	*radiodurans*
Z0023_RB_H06_1_A10_B	GREEN	No	192	115	LMG 4051	*Deinococcus*	*radiodurans*
Z0023_LO_H12_2_H06_B	GREEN	No	192	115	LMG 4051	*Deinococcus*	*radiodurans*
Z0023_RO_A04_1_E08_B	GREEN	No	192	115	LMG 4051	*Deinococcus*	*radiodurans*
Z0023_LB_A08_2_D03_B	GREEN	No	192	115	LMG 4051	*Deinococcus*	*radiodurans*
Z0023_LB_B01_1_D06_A	GREEN	No	192	115	LMG 4051	*Deinococcus*	*radiodurans*
Z0023_RB_A08_2_D09_B	GREEN	No	192	115	LMG 4051	*Deinococcus*	*radiodurans*
Z0023_RB_A04_2_D11_B	GREEN	No	192	115	LMG 4051	*Deinococcus*	*radiodurans*
Z0023_RB_A02_2_D12_B	GREEN	No	192	115	LMG 4051	*Deinococcus*	*radiodurans*
Z0023_LB_A05_2_D04_A	GREEN	No	192	115	LMG 4051	*Deinococcus*	*radiodurans*
Z0023_LB_A03_2_D05_A	GREEN	No	192	115	LMG 4051	*Deinococcus*	*radiodurans*
Z0023_LB_A04_2_D05_B	GREEN	No	192	115	LMG 4051	*Deinococcus*	*radiodurans*
Z0015_RO_A08_1_E10_B	GREEN	Yes	193	39	LMG 21665	*Pigmentiphaga*	*kullae*
Z0015_RO_B05_2_E09_A	GREEN	No	193	39	LMG 21665	*Pigmentiphaga*	*kullae*
Z0015_LO_B03_2_E02_A	GREEN	No	193	39	LMG 21665	*Pigmentiphaga*	*kullae*
Z0015_LO_B02_2_E01_B	GREEN	No	193	39	LMG 21665	*Pigmentiphaga*	*kullae*
Z0015_RO_B07_2_E10_A	GREEN	No	193	39	LMG 21665	*Pigmentiphaga*	*kullae*
Z0015_LO_B05_2_E03_A	GREEN	No	193	39	LMG 21665	*Pigmentiphaga*	*kullae*
Z0015_RO_A04_1_E08_B	GREEN	No	193	39	LMG 21665	*Pigmentiphaga*	*kullae*
Z0015_RO_A11_1_E12_A	GREEN	No	193	39	LMG 21665	*Pigmentiphaga*	*kullae*
Z0015_RO_A09_1_E11_A	GREEN	No	193	39	LMG 21665	*Pigmentiphaga*	*kullae*
Z0015_RO_A03_1_E08_A	GREEN	No	193	39	LMG 21665	*Pigmentiphaga*	*kullae*
Z0015_RO_B08_2_E10_B	GREEN	No	193	39	LMG 21665	*Pigmentiphaga*	*kullae*
Z0015_LO_B04_2_E02_B	GREEN	No	193	39	LMG 21665	*Pigmentiphaga*	*kullae*
Z0015_RO_A10_1_E11_B	GREEN	No	193	39	LMG 21665	*Pigmentiphaga*	*kullae*
Z0015_RO_A05_1_E09_A	GREEN	No	193	39	LMG 21665	*Pigmentiphaga*	*kullae*
Z0015_RO_A07_1_E10_A	GREEN	No	193	39	LMG 21665	*Pigmentiphaga*	*kullae*
Z0015_RO_A06_1_E09_B	GREEN	No	193	39	LMG 21665	*Pigmentiphaga*	*kullae*
Z0015_RO_B01_2_E07_A	GREEN	No	193	39	LMG 21665	*Pigmentiphaga*	*kullae*
Z0015_LO_B01_2_E01_A	GREEN	No	193	39	LMG 21665	*Pigmentiphaga*	*kullae*
Z0015_RO_A12_1_E12_B	GREEN	No	193	39	LMG 21665	*Pigmentiphaga*	*kullae*
Z0015_RO_B03_2_E08_A	GREEN	No	193	39	LMG 21665	*Pigmentiphaga*	*kullae*
Z0015_RO_B04_2_E08_B	GREEN	No	193	39	LMG 21665	*Pigmentiphaga*	*kullae*
Z0015_RO_B11_2_E12_A	GREEN	No	193	39	LMG 21665	*Pigmentiphaga*	*kullae*
Z0015_RO_B10_2_E11_B	GREEN	No	193	39	LMG 21665	*Pigmentiphaga*	*kullae*
Z0015_RO_B09_2_E11_A	GREEN	No	193	39	LMG 21665	*Pigmentiphaga*	*kullae*
Z0015_LO_B06_2_E03_B	GREEN	No	193	39	LMG 21665	*Pigmentiphaga*	*kullae*
Z0015_LO_B07_2_E04_A	GREEN	No	193	39	LMG 21665	*Pigmentiphaga*	*kullae*
Z0015_RO_B02_2_E07_B	GREEN	No	193	39	LMG 21665	*Pigmentiphaga*	*kullae*
Z0015_RO_B06_2_E09_B	GREEN	No	193	39	LMG 21665	*Pigmentiphaga*	*kullae*
Z0020_LO_B10_2_E05_B	GREEN	Yes	194	93	LMG 26586	*Eilatimonas*	*milleporae*
Z0020_RO_B10_2_E11_B	GREEN	No	194	93	LMG 26586	*Eilatimonas*	*milleporae*
Z0020_LO_B08_2_E04_B	GREEN	Yes	195	93	LMG 26586	*Eilatimonas*	*milleporae*
Z0020_RO_C05_1_F09_A	GREEN	No	195	93	LMG 26586	*Eilatimonas*	*milleporae*
Z0020_RO_C02_1_F07_B	GREEN	No	195	93	LMG 26586	*Eilatimonas*	*milleporae*
Z0020_LO_B04_2_E02_B	GREEN	No	195	93	LMG 26586	*Eilatimonas*	*milleporae*
Z0020_RO_B08_2_E10_B	GREEN	No	195	93	LMG 26586	*Eilatimonas*	*milleporae*
Z0020_LO_A12_1_E06_B	GREEN	No	195	93	LMG 26586	*Eilatimonas*	*milleporae*
Z0020_LO_B05_2_E03_A	GREEN	No	195	93	LMG 26586	*Eilatimonas*	*milleporae*
Z0020_RO_C03_1_F08_A	GREEN	No	195	93	LMG 26586	*Eilatimonas*	*milleporae*
Z0020_LO_B07_2_E04_A	GREEN	No	195	93	LMG 26586	*Eilatimonas*	*milleporae*
Z0020_RO_B03_2_E08_A	GREEN	No	195	93	LMG 26586	*Eilatimonas*	*milleporae*
Z0020_LO_A11_1_E06_A	GREEN	No	195	93	LMG 26586	*Eilatimonas*	*milleporae*
Z0020_LO_A08_1_E04_B	GREEN	No	195	93	LMG 26586	*Eilatimonas*	*milleporae*
Z0020_LO_B11_2_E06_A	GREEN	No	195	93	LMG 26586	*Eilatimonas*	*milleporae*
Z0020_LO_A07_1_E04_A	GREEN	No	195	93	LMG 26586	*Eilatimonas*	*milleporae*
Z0020_RO_B06_2_E09_B	GREEN	No	195	93	LMG 26586	*Eilatimonas*	*milleporae*
Z0020_RO_B11_2_E12_A	GREEN	No	195	93	LMG 26586	*Eilatimonas*	*milleporae*
Z0020_RO_B02_2_E07_B	GREEN	No	195	93	LMG 26586	*Eilatimonas*	*milleporae*
Z0020_LO_C03_1_F02_A	GREEN	No	195	93	LMG 26586	*Eilatimonas*	*milleporae*
Z0020_RO_B04_2_E08_B	GREEN	No	195	93	LMG 26586	*Eilatimonas*	*milleporae*
Z0020_RO_B12_2_E12_B	GREEN	No	195	93	LMG 26586	*Eilatimonas*	*milleporae*
Z0020_LO_C05_1_F03_A	GREEN	No	195	93	LMG 26586	*Eilatimonas*	*milleporae*
Z0020_RO_C08_1_F10_B	GREEN	No	195	93	LMG 26586	*Eilatimonas*	*milleporae*
Z0020_LO_C04_1_F02_B	GREEN	No	195	93	LMG 26586	*Eilatimonas*	*milleporae*
Z0020_LO_C02_1_F01_B	GREEN	No	195	93	LMG 26586	*Eilatimonas*	*milleporae*

Z0020_LO_C01_1_F01_A	GREEN	No	195	93	LMG 26586	*Eilatimonas*	*milleporae*
Z0020_RO_B09_2_E11_A	GREEN	No	195	93	LMG 26586	*Eilatimonas*	*milleporae*
Z0017_RB_H08_1_A09_B	GREEN	Yes	196	61	LMG 23965	*Collimonas*	*pratensis*
Z0017_RO_A04_1_E08_B	GREEN	No	196	61	LMG 23965	*Collimonas*	*pratensis*
Z0017_LB_H05_1_A04_A	GREEN	No	196	61	LMG 23965	*Collimonas*	*pratensis*
Z0017_RO_A11_1_E12_A	GREEN	No	196	61	LMG 23965	*Collimonas*	*pratensis*
Z0017_LB_H08_1_A03_B	GREEN	No	196	61	LMG 23965	*Collimonas*	*pratensis*
Z0017_LB_H06_1_A04_B	GREEN	No	196	61	LMG 23965	*Collimonas*	*pratensis*
Z0017_RO_A05_1_E09_A	GREEN	No	196	61	LMG 23965	*Collimonas*	*pratensis*
Z0017_RO_A07_1_E10_A	GREEN	No	196	61	LMG 23965	*Collimonas*	*pratensis*
Z0017_LB_H03_1_A05_A	GREEN	No	196	61	LMG 23965	*Collimonas*	*pratensis*
Z0017_LO_A06_1_E03_B	GREEN	No	196	61	LMG 23965	*Collimonas*	*pratensis*
Z0017_RO_A08_1_E10_B	GREEN	No	196	61	LMG 23965	*Collimonas*	*pratensis*
Z0017_LB_H11_1_A01_A	GREEN	No	196	61	LMG 23965	*Collimonas*	*pratensis*
Z0017_LO_A05_1_E03_A	GREEN	No	196	61	LMG 23965	*Collimonas*	*pratensis*
Z0017_RO_B01_2_E07_A	GREEN	No	196	61	LMG 23965	*Collimonas*	*pratensis*
Z0017_RB_H07_1_A09_A	GREEN	No	196	61	LMG 23965	*Collimonas*	*pratensis*
Z0017_LB_H02_1_A06_B	GREEN	No	196	61	LMG 23965	*Collimonas*	*pratensis*
Z0017_RB_H05_1_A10_A	GREEN	No	196	61	LMG 23965	*Collimonas*	*pratensis*
Z0017_LB_H10_1_A02_B	GREEN	No	196	61	LMG 23965	*Collimonas*	*pratensis*
Z0017_LB_H07_1_A03_A	GREEN	No	196	61	LMG 23965	*Collimonas*	*pratensis*
Z0017_RB_H04_1_A11_B	GREEN	No	196	61	LMG 23965	*Collimonas*	*pratensis*
Z0017_LB_H09_1_A02_A	GREEN	No	196	61	LMG 23965	*Collimonas*	*pratensis*
Z0017_RB_H06_1_A10_B	GREEN	No	196	61	LMG 23965	*Collimonas*	*pratensis*
Z0017_RO_A03_1_E08_A	GREEN	No	196	61	LMG 23965	*Collimonas*	*pratensis*
Z0017_RO_A02_1_E07_B	GREEN	No	196	61	LMG 23965	*Collimonas*	*pratensis*
Z0017_RB_H11_1_A07_A	GREEN	No	196	61	LMG 23965	*Collimonas*	*pratensis*
Z0017_LB_H04_1_A05_B	GREEN	No	196	61	LMG 23965	*Collimonas*	*pratensis*
Z0017_RO_A09_1_E11_A	GREEN	No	196	61	LMG 23965	*Collimonas*	*pratensis*
Z0017_LO_A02_1_E01_B	GREEN	No	196	61	LMG 23965	*Collimonas*	*pratensis*
Z0019_LO_A03_1_E02_A	GREEN	Yes	197	83	LMG 25773	*Tabrizicola*	*aquatica*
Z0019_LB_H04_1_A05_B	GREEN	No	197	83	LMG 25773	*Tabrizicola*	*aquatica*
Z0019_RB_H04_1_A11_B	GREEN	No	197	83	LMG 25773	*Tabrizicola*	*aquatica*
Z0019_LB_H12_1_A01_B	GREEN	No	197	83	LMG 25773	*Tabrizicola*	*aquatica*
Z0019_LO_A01_1_E01_A	GREEN	No	197	83	LMG 25773	*Tabrizicola*	*aquatica*
Z0019_RO_A07_1_E10_A	GREEN	No	197	83	LMG 25773	*Tabrizicola*	*aquatica*
Z0019_RO_A06_1_E09_B	GREEN	No	197	83	LMG 25773	*Tabrizicola*	*aquatica*
Z0019_LO_A02_1_E01_B	GREEN	No	197	83	LMG 25773	*Tabrizicola*	*aquatica*
Z0019_RO_A08_1_E10_B	GREEN	No	197	83	LMG 25773	*Tabrizicola*	*aquatica*
Z0019_RO_A09_1_E11_A	GREEN	No	197	83	LMG 25773	*Tabrizicola*	*aquatica*
Z0019_RO_A04_1_E08_B	GREEN	No	197	83	LMG 25773	*Tabrizicola*	*aquatica*
Z0019_RO_A05_1_E09_A	GREEN	No	197	83	LMG 25773	*Tabrizicola*	*aquatica*
Z0019_LB_H03_1_A05_A	GREEN	No	197	83	LMG 25773	*Tabrizicola*	*aquatica*
Z0019_LB_H09_1_A02_A	GREEN	No	197	83	LMG 25773	*Tabrizicola*	*aquatica*
Z0019_LB_H06_1_A04_B	GREEN	No	197	83	LMG 25773	*Tabrizicola*	*aquatica*
Z0019_LB_H07_1_A03_A	GREEN	No	197	83	LMG 25773	*Tabrizicola*	*aquatica*
Z0019_LO_A08_1_E04_B	GREEN	No	197	83	LMG 25773	*Tabrizicola*	*aquatica*
Z0019_LB_H05_1_A04_A	GREEN	No	197	83	LMG 25773	*Tabrizicola*	*aquatica*
Z0019_RO_A11_1_E12_A	GREEN	No	197	83	LMG 25773	*Tabrizicola*	*aquatica*
Z0019_LB_H10_1_A02_B	GREEN	No	197	83	LMG 25773	*Tabrizicola*	*aquatica*
Z0019_LB_H11_1_A01_A	GREEN	No	197	83	LMG 25773	*Tabrizicola*	*aquatica*
Z0019_LO_A04_1_E02_B	GREEN	No	197	83	LMG 25773	*Tabrizicola*	*aquatica*
Z0019_LB_H08_1_A03_B	GREEN	No	197	83	LMG 25773	*Tabrizicola*	*aquatica*
Z0019_LO_A06_1_E03_B	GREEN	No	197	83	LMG 25773	*Tabrizicola*	*aquatica*
Z0019_LO_A05_1_E03_A	GREEN	No	197	83	LMG 25773	*Tabrizicola*	*aquatica*
Z0019_RO_A03_1_E08_A	GREEN	No	197	83	LMG 25773	*Tabrizicola*	*aquatica*
Z0019_RB_H05_1_A10_A	GREEN	No	197	83	LMG 25773	*Tabrizicola*	*aquatica*
Z0019_RB_H09_1_A08_A	GREEN	No	197	83	LMG 25773	*Tabrizicola*	*aquatica*
Z0022_RO_D10_2_F11_B	GREEN	Yes	198	113	LMG 4044	*Vibrio*	*harveyi*
Z0022_LO_F09_2_G05_A	GREEN	No	198	113	LMG 4044	*Vibrio*	*harveyi*
Z0022_RO_G03_1_H08_A	GREEN	Yes	199	113	LMG 4044	*Vibrio*	*harveyi*
Z0022_RO_F11_2_G12_A	GREEN	No	199	113	LMG 4044	*Vibrio*	*harveyi*
Z0022_LO_F06_2_G03_B	GREEN	No	199	113	LMG 4044	*Vibrio*	*harveyi*
Z0022_RO_F08_2_G10_B	GREEN	No	199	113	LMG 4044	*Vibrio*	*harveyi*
Z0022_RO_F06_2_G09_B	GREEN	No	199	113	LMG 4044	*Vibrio*	*harveyi*
Z0022_LO_H05_2_H03_A	GREEN	No	199	113	LMG 4044	*Vibrio*	*harveyi*
Z0022_LO_H04_2_H02_B	GREEN	No	199	113	LMG 4044	*Vibrio*	*harveyi*
Z0022_LO_F11_2_G06_A	GREEN	No	199	113	LMG 4044	*Vibrio*	*harveyi*
Z0022_LO_F05_2_G03_A	GREEN	No	199	113	LMG 4044	*Vibrio*	*harveyi*
Z0022_RO_F09_2_G11_A	GREEN	No	199	113	LMG 4044	*Vibrio*	*harveyi*

Z0022_LO_H03_2_H02_A	GREEN	No	199	113	LMG 4044	*Vibrio*	*harveyi*
Z0022_LO_G09_1_H05_A	GREEN	No	199	113	LMG 4044	*Vibrio*	*harveyi*
Z0022_RO_F03_2_G08_A	GREEN	No	199	113	LMG 4044	*Vibrio*	*harveyi*
Z0022_RO_F10_2_G11_B	GREEN	No	199	113	LMG 4044	*Vibrio*	*harveyi*
Z0022_LO_H02_2_H01_B	GREEN	No	199	113	LMG 4044	*Vibrio*	*harveyi*
Z0022_LO_G04_1_H02_B	GREEN	No	199	113	LMG 4044	*Vibrio*	*harveyi*
Z0022_RO_E12_1_G12_B	GREEN	No	199	113	LMG 4044	*Vibrio*	*harveyi*
Z0022_LO_F04_2_G02_B	GREEN	No	199	113	LMG 4044	*Vibrio*	*harveyi*
Z0022_LO_F07_2_G04_A	GREEN	No	199	113	LMG 4044	*Vibrio*	*harveyi*
Z0022_RO_F02_2_G07_B	GREEN	No	199	113	LMG 4044	*Vibrio*	*harveyi*
Z0022_RO_F04_2_G08_B	GREEN	No	199	113	LMG 4044	*Vibrio*	*harveyi*
Z0022_LO_H01_2_H01_A	GREEN	No	199	113	LMG 4044	*Vibrio*	*harveyi*
Z0022_LO_F02_2_G01_B	GREEN	No	199	113	LMG 4044	*Vibrio*	*harveyi*
Z0022_LO_F03_2_G02_A	GREEN	No	199	113	LMG 4044	*Vibrio*	*harveyi*
Z0022_LO_E08_1_G04_B	GREEN	No	199	113	LMG 4044	*Vibrio*	*harveyi*
Z0022_RO_F05_2_G09_A	GREEN	No	199	113	LMG 4044	*Vibrio*	*harveyi*
Z0022_LO_F08_2_G04_B	GREEN	No	199	113	LMG 4044	*Vibrio*	*harveyi*
Z0022_RO_E04_1_G08_B	GREEN	No	199	113	LMG 4044	*Vibrio*	*harveyi*
Z0022_LO_G10_1_H05_B	GREEN	No	199	113	LMG 4044	*Vibrio*	*harveyi*
Z0022_LO_F01_2_G01_A	GREEN	No	199	113	LMG 4044	*Vibrio*	*harveyi*
Z0022_RO_A10_1_E11_B	GREEN	Yes	200	107	LMG 2864	*Marinomonas*	*communis*
Z0022_RO_B03_2_E08_A	GREEN	No	200	107	LMG 2864	*Marinomonas*	*communis*
Z0022_RO_B04_2_E08_B	GREEN	No	200	107	LMG 2864	*Marinomonas*	*communis*
Z0022_RO_A11_1_E12_A	GREEN	No	200	107	LMG 2864	*Marinomonas*	*communis*
Z0022_LB_A01_2_D06_A	GREEN	No	200	107	LMG 2864	*Marinomonas*	*communis*
Z0022_LB_A03_2_D05_A	GREEN	No	200	107	LMG 2864	*Marinomonas*	*communis*
Z0022_RB_A07_2_D09_A	GREEN	No	200	107	LMG 2864	*Marinomonas*	*communis*
Z0022_LB_B01_1_D06_A	GREEN	No	200	107	LMG 2864	*Marinomonas*	*communis*
Z0022_RB_A05_2_D10_A	GREEN	No	200	107	LMG 2864	*Marinomonas*	*communis*
Z0022_RB_A06_2_D10_B	GREEN	No	200	107	LMG 2864	*Marinomonas*	*communis*
Z0022_RO_A08_1_E10_B	GREEN	No	200	107	LMG 2864	*Marinomonas*	*communis*
Z0022_RO_B01_2_E07_A	GREEN	No	200	107	LMG 2864	*Marinomonas*	*communis*
Z0022_LO_B08_2_E04_B	GREEN	No	200	107	LMG 2864	*Marinomonas*	*communis*
Z0022_RO_A09_1_E11_A	GREEN	No	200	107	LMG 2864	*Marinomonas*	*communis*
Z0022_RB_A04_2_D11_B	GREEN	No	200	107	LMG 2864	*Marinomonas*	*communis*
Z0022_LB_A11_2_D01_A	GREEN	No	200	107	LMG 2864	*Marinomonas*	*communis*
Z0022_LB_A06_2_D04_B	GREEN	No	200	107	LMG 2864	*Marinomonas*	*communis*
Z0022_RB_A02_2_D12_B	GREEN	No	200	107	LMG 2864	*Marinomonas*	*communis*
Z0022_RB_A03_2_D11_A	GREEN	No	200	107	LMG 2864	*Marinomonas*	*communis*
Z0022_LB_A08_2_D03_B	GREEN	No	200	107	LMG 2864	*Marinomonas*	*communis*
Z0022_LO_B10_2_E05_B	GREEN	No	200	107	LMG 2864	*Marinomonas*	*communis*
Z0022_RO_C07_1_F10_A	GREEN	No	200	107	LMG 2864	*Marinomonas*	*communis*
Z0022_LB_A07_2_D03_A	GREEN	No	200	107	LMG 2864	*Marinomonas*	*communis*
Z0022_LB_B02_1_D06_B	GREEN	No	200	107	LMG 2864	*Marinomonas*	*communis*
Z0022_LB_A12_2_D01_B	GREEN	No	200	107	LMG 2864	*Marinomonas*	*communis*
Z0022_LB_A02_2_D06_B	GREEN	No	200	107	LMG 2864	*Marinomonas*	*communis*
Z0022_LO_B01_2_E01_A	GREEN	No	200	107	LMG 2864	*Marinomonas*	*communis*
Z0022_LB_A10_2_D02_B	GREEN	No	200	107	LMG 2864	*Marinomonas*	*communis*
Z0022_LB_A05_2_D04_A	GREEN	No	200	107	LMG 2864	*Marinomonas*	*communis*
Z0022_LB_A09_2_D02_A	GREEN	No	200	107	LMG 2864	*Marinomonas*	*communis*
Z0022_RO_C08_1_F10_B	GREEN	No	200	107	LMG 2864	*Marinomonas*	*communis*
Z0022_LB_A04_2_D05_B	GREEN	No	200	107	LMG 2864	*Marinomonas*	*communis*
Z0015_RB_G03_2_A11_A	GREEN	Yes	201	38	LMG 21530	*Massilia*	*timonae*
Z0015_RB_G04_2_A11_B	GREEN	No	201	38	LMG 21530	*Massilia*	*timonae*
Z0015_LB_H06_1_A04_B	GREEN	No	201	38	LMG 21530	*Massilia*	*timonae*
Z0015_LB_H02_1_A06_B	GREEN	No	201	38	LMG 21530	*Massilia*	*timonae*
Z0015_RB_F10_1_B08_B	GREEN	No	201	38	LMG 21530	*Massilia*	*timonae*
Z0015_RB_F04_1_B11_B	GREEN	No	201	38	LMG 21530	*Massilia*	*timonae*
Z0015_LB_H10_1_A02_B	GREEN	No	201	38	LMG 21530	*Massilia*	*timonae*
Z0015_LB_H04_1_A05_B	GREEN	No	201	38	LMG 21530	*Massilia*	*timonae*
Z0015_LB_H03_1_A05_A	GREEN	No	201	38	LMG 21530	*Massilia*	*timonae*
Z0015_RB_G01_2_A12_A	GREEN	No	201	38	LMG 21530	*Massilia*	*timonae*
Z0015_RB_F09_1_B08_A	GREEN	No	201	38	LMG 21530	*Massilia*	*timonae*
Z0015_RB_F05_1_B10_A	GREEN	No	201	38	LMG 21530	*Massilia*	*timonae*
Z0015_LB_H01_1_A06_A	GREEN	No	201	38	LMG 21530	*Massilia*	*timonae*
Z0015_RB_F07_1_B09_A	GREEN	No	201	38	LMG 21530	*Massilia*	*timonae*
Z0015_LB_H09_1_A02_A	GREEN	No	201	38	LMG 21530	*Massilia*	*timonae*
Z0015_RB_G07_2_A09_A	GREEN	No	201	38	LMG 21530	*Massilia*	*timonae*
Z0015_RB_G12_2_A07_B	GREEN	No	201	38	LMG 21530	*Massilia*	*timonae*
Z0015_RB_F06_1_B10_B	GREEN	No	201	38	LMG 21530	*Massilia*	*timonae*

Z0015_RB_G05_2_A10_A	GREEN	No	201	38	LMG 21530	*Massilia*	*timonae*
Z0015_RB_F12_1_B07_B	GREEN	No	201	38	LMG 21530	*Massilia*	*timonae*
Z0015_RB_G06_2_A10_B	GREEN	No	201	38	LMG 21530	*Massilia*	*timonae*
Z0015_RB_G08_2_A09_B	GREEN	No	201	38	LMG 21530	*Massilia*	*timonae*
Z0015_RB_G09_2_A08_A	GREEN	No	201	38	LMG 21530	*Massilia*	*timonae*
Z0015_RB_F08_1_B09_B	GREEN	No	201	38	LMG 21530	*Massilia*	*timonae*
Z0015_LB_G12_2_A01_B	GREEN	No	201	38	LMG 21530	*Massilia*	*timonae*
Z0015_RB_G11_2_A07_A	GREEN	No	201	38	LMG 21530	*Massilia*	*timonae*
Z0015_RB_G02_2_A12_B	GREEN	No	201	38	LMG 21530	*Massilia*	*timonae*
Z0015_RB_F11_1_B07_A	GREEN	No	201	38	LMG 21530	*Massilia*	*timonae*
Z0015_RB_G10_2_A08_B	GREEN	No	201	38	LMG 21530	*Massilia*	*timonae*
Z0015_LB_H05_1_A04_A	GREEN	No	201	38	LMG 21530	*Massilia*	*timonae*
Z0015_LB_H08_1_A03_B	GREEN	No	201	38	LMG 21530	*Massilia*	*timonae*
Z0015_LB_H07_1_A03_A	GREEN	No	201	38	LMG 21530	*Massilia*	*timonae*
Z0010_LO_B08_2_E04_B	GREEN	Yes	202	141	R-50394	*Burkholderia*	*stabilis*
Z0010_RO_B06_2_E09_B	GREEN	No	202	141	R-50394	*Burkholderia*	*stabilis*
Z0010_RO_B02_2_E07_B	GREEN	No	202	141	R-50394	*Burkholderia*	*stabilis*
Z0010_LO_B04_2_E02_B	GREEN	No	202	141	R-50394	*Burkholderia*	*stabilis*
Z0010_LO_B10_2_E05_B	GREEN	No	202	141	R-50394	*Burkholderia*	*stabilis*
Z0010_LO_C03_1_F02_A	GREEN	No	202	141	R-50394	*Burkholderia*	*stabilis*
Z0010_LO_B06_2_E03_B	GREEN	No	202	141	R-50394	*Burkholderia*	*stabilis*
Z0010_LO_B07_2_E04_A	GREEN	No	202	141	R-50394	*Burkholderia*	*stabilis*
Z0010_LO_C02_1_F01_B	GREEN	No	202	141	R-50394	*Burkholderia*	*stabilis*
Z0010_LO_C01_1_F01_A	GREEN	No	202	141	R-50394	*Burkholderia*	*stabilis*
Z0010_RO_A09_1_E11_A	GREEN	No	202	141	R-50394	*Burkholderia*	*stabilis*
Z0010_RO_B07_2_E10_A	GREEN	No	202	141	R-50394	*Burkholderia*	*stabilis*
Z0010_RO_A12_1_E12_B	GREEN	No	202	141	R-50394	*Burkholderia*	*stabilis*
Z0010_LO_C05_1_F03_A	GREEN	No	202	141	R-50394	*Burkholderia*	*stabilis*
Z0010_RO_B01_2_E07_A	GREEN	No	202	141	R-50394	*Burkholderia*	*stabilis*
Z0010_LO_C04_1_F02_B	GREEN	No	202	141	R-50394	*Burkholderia*	*stabilis*
Z0010_LO_B09_2_E05_A	GREEN	No	202	141	R-50394	*Burkholderia*	*stabilis*
Z0010_RO_A10_1_E11_B	GREEN	No	202	141	R-50394	*Burkholderia*	*stabilis*
Z0010_RO_B04_2_E08_B	GREEN	No	202	141	R-50394	*Burkholderia*	*stabilis*
Z0010_RO_B03_2_E08_A	GREEN	No	202	141	R-50394	*Burkholderia*	*stabilis*
Z0010_LO_B12_2_E06_B	GREEN	No	202	141	R-50394	*Burkholderia*	*stabilis*
Z0010_LO_B11_2_E06_A	GREEN	No	202	141	R-50394	*Burkholderia*	*stabilis*
Z0010_LO_B05_2_E03_B	GREEN	No	202	141	R-50394	*Burkholderia*	*stabilis*
Z0010_RO_A11_1_E12_A	GREEN	No	202	141	R-50394	*Burkholderia*	*stabilis*
Z0010_RO_B08_2_E10_B	GREEN	No	202	141	R-50394	*Burkholderia*	*stabilis*
Z0010_RO_B05_2_E09_A	GREEN	No	202	141	R-50394	*Burkholderia*	*stabilis*
Z0010_RO_A08_1_E10_B	GREEN	No	202	141	R-50394	*Burkholderia*	*stabilis*
Z0010_LO_B03_2_E02_A	GREEN	No	202	141	R-50394	*Burkholderia*	*stabilis*
Z0019_RO_E07_1_G10_A	GREEN	Yes	203	87	LMG 26149	*Herbaspirillum*	*soli*
Z0019_RO_E02_1_G07_B	GREEN	No	203	87	LMG 26149	*Herbaspirillum*	*soli*
Z0019_RO_E11_1_G12_A	GREEN	No	203	87	LMG 26149	*Herbaspirillum*	*soli*
Z0019_RO_F12_2_G12_B	GREEN	No	203	87	LMG 26149	*Herbaspirillum*	*soli*
Z0019_LO_E05_1_G03_A	GREEN	No	203	87	LMG 26149	*Herbaspirillum*	*soli*
Z0019_LO_E09_1_G05_A	GREEN	No	203	87	LMG 26149	*Herbaspirillum*	*soli*
Z0019_RO_D04_2_F08_B	GREEN	No	203	87	LMG 26149	*Herbaspirillum*	*soli*
Z0019_RO_G02_1_H07_B	GREEN	No	203	87	LMG 26149	*Herbaspirillum*	*soli*
Z0019_LO_E03_1_G02_A	GREEN	No	203	87	LMG 26149	*Herbaspirillum*	*soli*
Z0019_RO_G01_1_H07_A	GREEN	No	203	87	LMG 26149	*Herbaspirillum*	*soli*
Z0019_RO_E09_1_G11_A	GREEN	No	203	87	LMG 26149	*Herbaspirillum*	*soli*
Z0019_RO_D11_2_F12_A	GREEN	No	203	87	LMG 26149	*Herbaspirillum*	*soli*
Z0019_LO_F04_2_G02_B	GREEN	No	203	87	LMG 26149	*Herbaspirillum*	*soli*
Z0019_LO_F02_2_G01_B	GREEN	No	203	87	LMG 26149	*Herbaspirillum*	*soli*
Z0019_LO_E11_1_G06_A	GREEN	No	203	87	LMG 26149	*Herbaspirillum*	*soli*
Z0019_LO_E01_1_G01_A	GREEN	No	203	87	LMG 26149	*Herbaspirillum*	*soli*
Z0019_LO_E10_1_G05_B	GREEN	No	203	87	LMG 26149	*Herbaspirillum*	*soli*
Z0019_RO_E05_1_G09_A	GREEN	No	203	87	LMG 26149	*Herbaspirillum*	*soli*
Z0019_LO_F03_2_G02_A	GREEN	No	203	87	LMG 26149	*Herbaspirillum*	*soli*
Z0019_LO_E12_1_G06_B	GREEN	No	203	87	LMG 26149	*Herbaspirillum*	*soli*
Z0019_LO_F05_2_G03_A	GREEN	No	203	87	LMG 26149	*Herbaspirillum*	*soli*
Z0019_RO_E03_1_G08_A	GREEN	No	203	87	LMG 26149	*Herbaspirillum*	*soli*
Z0019_RO_D10_2_F11_B	GREEN	No	203	87	LMG 26149	*Herbaspirillum*	*soli*
Z0019_LO_D11_2_F06_A	GREEN	No	203	87	LMG 26149	*Herbaspirillum*	*soli*
Z0019_LO_E07_1_G04_A	GREEN	No	203	87	LMG 26149	*Herbaspirillum*	*soli*
Z0019_LO_F12_2_G06_B	GREEN	No	203	87	LMG 26149	*Herbaspirillum*	*soli*
Z0019_LO_E08_1_G04_B	GREEN	No	203	87	LMG 26149	*Herbaspirillum*	*soli*
Z0019_LO_F01_2_G01_A	GREEN	No	203	87	LMG 26149	*Herbaspirillum*	*soli*

Z0019_LO_G04_1_H02_B	GREEN	No	203	87	LMG 26149	*Herbaspirillum*	*soli*	
Z0019_RO_F09_2_G11_A	GREEN	No	203	87	LMG 26149	*Herbaspirillum*	*soli*	
Z0019_RO_F10_2_G11_B	GREEN	No	203	87	LMG 26149	*Herbaspirillum*	*soli*	
Z0019_RO_F11_2_G12_A	GREEN	No	203	87	LMG 26149	*Herbaspirillum*	*soli*	
Z0014_LB_E11_2_B01_A	GREEN	Yes	204	27	LMG 18397	*Anoxybacillus*	*flavithermus*	
Z0014_LB_E08_2_B03_B	GREEN	No	204	27	LMG 18397	*Anoxybacillus*	*flavithermus*	
Z0014_LB_F03_1_B05_A	GREEN	No	204	27	LMG 18397	*Anoxybacillus*	*flavithermus*	
Z0014_RB_E07_2_B09_A	GREEN	No	204	27	LMG 18397	*Anoxybacillus*	*flavithermus*	
Z0014_LB_E05_2_B04_A	GREEN	No	204	27	LMG 18397	*Anoxybacillus*	*flavithermus*	
Z0014_RB_E05_2_B10_A	GREEN	No	204	27	LMG 18397	*Anoxybacillus*	*flavithermus*	
Z0014_LB_E10_2_B02_B	GREEN	No	204	27	LMG 18397	*Anoxybacillus*	*flavithermus*	
Z0014_LB_E07_2_B03_A	GREEN	No	204	27	LMG 18397	*Anoxybacillus*	*flavithermus*	
Z0014_RB_E08_2_B09_B	GREEN	No	204	27	LMG 18397	*Anoxybacillus*	*flavithermus*	
Z0014_LB_F01_1_B06_A	GREEN	No	204	27	LMG 18397	*Anoxybacillus*	*flavithermus*	
Z0014_LB_D11_1_C01_A	GREEN	No	204	27	LMG 18397	*Anoxybacillus*	*flavithermus*	
Z0014_RB_F01_1_B12_A	GREEN	No	204	27	LMG 18397	*Anoxybacillus*	*flavithermus*	
Z0014_RB_F05_1_B10_A	GREEN	No	204	27	LMG 18397	*Anoxybacillus*	*flavithermus*	
Z0014_LB_E01_2_B06_A	GREEN	No	204	27	LMG 18397	*Anoxybacillus*	*flavithermus*	
Z0014_RB_E06_2_B10_B	GREEN	No	204	27	LMG 18397	*Anoxybacillus*	*flavithermus*	
Z0014_LB_E09_2_B02_A	GREEN	No	204	27	LMG 18397	*Anoxybacillus*	*flavithermus*	
Z0014_LB_D10_1_C02_B	GREEN	No	204	27	LMG 18397	*Anoxybacillus*	*flavithermus*	
Z0014_RB_E12_2_B07_B	GREEN	No	204	27	LMG 18397	*Anoxybacillus*	*flavithermus*	
Z0014_RB_E04_2_B11_B	GREEN	No	204	27	LMG 18397	*Anoxybacillus*	*flavithermus*	
Z0014_LB_F02_1_B06_B	GREEN	No	204	27	LMG 18397	*Anoxybacillus*	*flavithermus*	
Z0014_LB_E06_2_B04_B	GREEN	No	204	27	LMG 18397	*Anoxybacillus*	*flavithermus*	
Z0014_RB_F03_1_B11_A	GREEN	No	204	27	LMG 18397	*Anoxybacillus*	*flavithermus*	
Z0014_LB_D12_1_C01_B	GREEN	No	204	27	LMG 18397	*Anoxybacillus*	*flavithermus*	
Z0014_LB_E03_2_B05_A	GREEN	No	204	27	LMG 18397	*Anoxybacillus*	*flavithermus*	
Z0014_LB_E12_2_B01_B	GREEN	No	204	27	LMG 18397	*Anoxybacillus*	*flavithermus*	
Z0014_RB_E10_2_B08_B	GREEN	No	204	27	LMG 18397	*Anoxybacillus*	*flavithermus*	
Z0014_RB_E09_2_B08_A	GREEN	No	204	27	LMG 18397	*Anoxybacillus*	*flavithermus*	
Z0014_RB_F02_1_B12_B	GREEN	No	204	27	LMG 18397	*Anoxybacillus*	*flavithermus*	
Z0014_LB_E04_2_B05_B	GREEN	No	204	27	LMG 18397	*Anoxybacillus*	*flavithermus*	
Z0014_RB_E11_2_B07_A	GREEN	No	204	27	LMG 18397	*Anoxybacillus*	*flavithermus*	
Z0014_LB_E02_2_B06_B	GREEN	No	204	27	LMG 18397	*Anoxybacillus*	*flavithermus*	
Z0014_RB_F04_1_B11_B	GREEN	No	204	27	LMG 18397	*Anoxybacillus*	*flavithermus*	
Z0013_RO_G11_1_H12_A	GREEN	Yes	205	23	LMG 1668	*Acidomonas*	*methanolica*	
Z0013_RB_F10_1_B08_B	GREEN	No	205	23	LMG 1668	*Acidomonas*	*methanolica*	
Z0013_RB_H02_1_A12_B	GREEN	No	205	23	LMG 1668	*Acidomonas*	*methanolica*	
Z0013_LO_G10_1_H05_B	GREEN	No	205	23	LMG 1668	*Acidomonas*	*methanolica*	
Z0013_RB_G06_2_A10_B	GREEN	No	205	23	LMG 1668	*Acidomonas*	*methanolica*	
Z0013_LO_F12_2_G06_B	GREEN	No	205	23	LMG 1668	*Acidomonas*	*methanolica*	
Z0013_RB_G01_2_A12_A	GREEN	No	205	23	LMG 1668	*Acidomonas*	*methanolica*	
Z0013_RB_G04_2_A11_B	GREEN	No	205	23	LMG 1668	*Acidomonas*	*methanolica*	
Z0013_LO_G01_1_H01_A	GREEN	No	205	23	LMG 1668	*Acidomonas*	*methanolica*	
Z0013_RO_F11_2_G12_A	GREEN	No	205	23	LMG 1668	*Acidomonas*	*methanolica*	
Z0013_RB_G09_2_A08_A	GREEN	No	205	23	LMG 1668	*Acidomonas*	*methanolica*	
Z0013_RB_G08_2_A09_B	GREEN	No	205	23	LMG 1668	*Acidomonas*	*methanolica*	
Z0013_LB_G11_2_A01_A	GREEN	No	205	23	LMG 1668	*Acidomonas*	*methanolica*	
Z0013_RO_G08_1_H10_B	GREEN	No	205	23	LMG 1668	*Acidomonas*	*methanolica*	
Z0013_RO_G04_1_H08_B	GREEN	No	205	23	LMG 1668	*Acidomonas*	*methanolica*	
Z0013_LB_G08_2_A03_B	GREEN	No	205	23	LMG 1668	*Acidomonas*	*methanolica*	
Z0013_RO_G12_1_H12_B	GREEN	No	205	23	LMG 1668	*Acidomonas*	*methanolica*	
Z0013_RO_G07_1_H10_A	GREEN	No	205	23	LMG 1668	*Acidomonas*	*methanolica*	
Z0013_RB_G07_2_A09_A	GREEN	No	205	23	LMG 1668	*Acidomonas*	*methanolica*	
Z0013_RO_G10_1_H11_B	GREEN	No	205	23	LMG 1668	*Acidomonas*	*methanolica*	
Z0013_LO_G08_1_H04_B	GREEN	No	205	23	LMG 1668	*Acidomonas*	*methanolica*	
Z0013_LB_H04_1_A05_B	GREEN	No	205	23	LMG 1668	*Acidomonas*	*methanolica*	
Z0013_LO_G04_1_H02_B	GREEN	No	205	23	LMG 1668	*Acidomonas*	*methanolica*	
Z0013_LO_H02_2_H01_B	GREEN	No	205	23	LMG 1668	*Acidomonas*	*methanolica*	
Z0013_RB_G12_2_A07_B	GREEN	No	205	23	LMG 1668	*Acidomonas*	*methanolica*	
Z0013_RO_G09_1_H11_A	GREEN	No	205	23	LMG 1668	*Acidomonas*	*methanolica*	
Z0013_LO_G03_1_H02_A	GREEN	No	205	23	LMG 1668	*Acidomonas*	*methanolica*	
Z0013_RO_G03_1_H08_A	GREEN	No	205	23	LMG 1668	*Acidomonas*	*methanolica*	
Z0013_RB_G02_2_A12_B	GREEN	No	205	23	LMG 1668	*Acidomonas*	*methanolica*	
Z0013_LB_G04_2_A05_B	GREEN	No	205	23	LMG 1668	*Acidomonas*	*methanolica*	
Z0013_LB_G05_2_A04_A	GREEN	No	205	23	LMG 1668	*Acidomonas*	*methanolica*	
Z0013_LO_G06_1_H03_B	GREEN	No	205	23	LMG 1668	*Acidomonas*	*methanolica*	
Z0025_RB_G04_2_A11_B	GREEN	Yes	206	136	LMG 7899	*Yersinia*	*enterocolitica*	*enterocolitica*
Z0025_RB_G07_2_A09_A	GREEN	No	206	136	LMG 7899	*Yersinia*	*enterocolitica*	*enterocolitica*

Z0025_RB_G06_2_A10_B	GREEN	Yes	207	136	LMG 7899	*Yersinia*	*enterocolitica*	*enterocolitica*
Z0025_LB_F06_1_B04_B	GREEN	No	207	136	LMG 7899	*Yersinia*	*enterocolitica*	*enterocolitica*
Z0025_LB_F11_1_B01_A	GREEN	No	207	136	LMG 7899	*Yersinia*	*enterocolitica*	*enterocolitica*
Z0025_RB_F10_1_B08_B	GREEN	No	207	136	LMG 7899	*Yersinia*	*enterocolitica*	*enterocolitica*
Z0025_LB_F04_1_B05_B	GREEN	No	207	136	LMG 7899	*Yersinia*	*enterocolitica*	*enterocolitica*
Z0025_LB_F03_1_B05_A	GREEN	No	207	136	LMG 7899	*Yersinia*	*enterocolitica*	*enterocolitica*
Z0025_RB_F12_1_B07_B	GREEN	No	207	136	LMG 7899	*Yersinia*	*enterocolitica*	*enterocolitica*
Z0025_RB_F11_1_B07_A	GREEN	No	207	136	LMG 7899	*Yersinia*	*enterocolitica*	*enterocolitica*
Z0025_LB_G03_2_A05_A	GREEN	No	207	136	LMG 7899	*Yersinia*	*enterocolitica*	*enterocolitica*
Z0025_LB_G06_2_A04_B	GREEN	No	207	136	LMG 7899	*Yersinia*	*enterocolitica*	*enterocolitica*
Z0025_LB_F12_1_B01_B	GREEN	No	207	136	LMG 7899	*Yersinia*	*enterocolitica*	*enterocolitica*
Z0025_LB_E12_2_B01_B	GREEN	No	207	136	LMG 7899	*Yersinia*	*enterocolitica*	*enterocolitica*
Z0025_LB_G07_2_A03_A	GREEN	No	207	136	LMG 7899	*Yersinia*	*enterocolitica*	*enterocolitica*
Z0025_LB_E10_2_B02_B	GREEN	No	207	136	LMG 7899	*Yersinia*	*enterocolitica*	*enterocolitica*
Z0025_RB_G05_2_A10_A	GREEN	No	207	136	LMG 7899	*Yersinia*	*enterocolitica*	*enterocolitica*
Z0025_LB_G01_2_A06_A	GREEN	No	207	136	LMG 7899	*Yersinia*	*enterocolitica*	*enterocolitica*
Z0025_LB_F09_1_B02_A	GREEN	No	207	136	LMG 7899	*Yersinia*	*enterocolitica*	*enterocolitica*
Z0025_LB_G05_2_A04_A	GREEN	No	207	136	LMG 7899	*Yersinia*	*enterocolitica*	*enterocolitica*
Z0025_LB_G04_2_A05_B	GREEN	No	207	136	LMG 7899	*Yersinia*	*enterocolitica*	*enterocolitica*
Z0025_LB_F10_1_B02_B	GREEN	No	207	136	LMG 7899	*Yersinia*	*enterocolitica*	*enterocolitica*
Z0025_LB_F07_1_B03_A	GREEN	No	207	136	LMG 7899	*Yersinia*	*enterocolitica*	*enterocolitica*
Z0025_LB_F02_1_B06_B	GREEN	No	207	136	LMG 7899	*Yersinia*	*enterocolitica*	*enterocolitica*
Z0025_RB_G02_2_A12_B	GREEN	No	207	136	LMG 7899	*Yersinia*	*enterocolitica*	*enterocolitica*
Z0025_LB_G02_2_A06_B	GREEN	No	207	136	LMG 7899	*Yersinia*	*enterocolitica*	*enterocolitica*
Z0025_LB_F05_1_B04_A	GREEN	No	207	136	LMG 7899	*Yersinia*	*enterocolitica*	*enterocolitica*
Z0025_RB_G01_2_A12_A	GREEN	No	207	136	LMG 7899	*Yersinia*	*enterocolitica*	*enterocolitica*
Z0025_LB_E11_2_B01_A	GREEN	No	207	136	LMG 7899	*Yersinia*	*enterocolitica*	*enterocolitica*
Z0025_LB_F08_1_B03_B	GREEN	No	207	136	LMG 7899	*Yersinia*	*enterocolitica*	*enterocolitica*
Z0025_RB_G03_2_A11_A	GREEN	No	207	136	LMG 7899	*Yersinia*	*enterocolitica*	*enterocolitica*
Z0025_LB_F01_1_B06_A	GREEN	No	207	136	LMG 7899	*Yersinia*	*enterocolitica*	*enterocolitica*
Z0015_LB_F06_1_B04_B	GREEN	Yes	208	36	LMG 21311	*Gluconacetobacter*	*azotocaptans*	
Z0015_RB_C10_2_C08_B	GREEN	No	208	36	LMG 21311	*Gluconacetobacter*	*azotocaptans*	
Z0015_LB_E10_2_B02_B	GREEN	No	208	36	LMG 21311	*Gluconacetobacter*	*azotocaptans*	
Z0015_RB_C12_2_C07_B	GREEN	No	208	36	LMG 21311	*Gluconacetobacter*	*azotocaptans*	
Z0015_LB_E08_2_B03_B	GREEN	No	208	36	LMG 21311	*Gluconacetobacter*	*azotocaptans*	
Z0015_LB_F01_1_B06_A	GREEN	No	208	36	LMG 21311	*Gluconacetobacter*	*azotocaptans*	
Z0015_LB_E07_2_B03_A	GREEN	No	208	36	LMG 21311	*Gluconacetobacter*	*azotocaptans*	
Z0015_LB_E12_2_B01_B	GREEN	No	208	36	LMG 21311	*Gluconacetobacter*	*azotocaptans*	
Z0015_RB_D08_1_C09_B	GREEN	No	208	36	LMG 21311	*Gluconacetobacter*	*azotocaptans*	
Z0015_RB_D06_1_C10_B	GREEN	No	208	36	LMG 21311	*Gluconacetobacter*	*azotocaptans*	
Z0015_RB_D01_1_C12_A	GREEN	No	208	36	LMG 21311	*Gluconacetobacter*	*azotocaptans*	
Z0015_RB_D11_1_C07_A	GREEN	No	208	36	LMG 21311	*Gluconacetobacter*	*azotocaptans*	
Z0015_RB_D07_1_C09_A	GREEN	No	208	36	LMG 21311	*Gluconacetobacter*	*azotocaptans*	
Z0015_RB_C11_2_C07_A	GREEN	No	208	36	LMG 21311	*Gluconacetobacter*	*azotocaptans*	
Z0015_RB_D02_1_C12_B	GREEN	No	208	36	LMG 21311	*Gluconacetobacter*	*azotocaptans*	
Z0015_RB_C09_2_C08_A	GREEN	No	208	36	LMG 21311	*Gluconacetobacter*	*azotocaptans*	
Z0015_RB_D05_1_C10_A	GREEN	No	208	36	LMG 21311	*Gluconacetobacter*	*azotocaptans*	
Z0015_LB_F05_1_B04_A	GREEN	No	208	36	LMG 21311	*Gluconacetobacter*	*azotocaptans*	
Z0015_RB_D10_1_C08_B	GREEN	No	208	36	LMG 21311	*Gluconacetobacter*	*azotocaptans*	
Z0015_RB_D12_1_C07_B	GREEN	No	208	36	LMG 21311	*Gluconacetobacter*	*azotocaptans*	
Z0015_RB_C08_2_C09_B	GREEN	No	208	36	LMG 21311	*Gluconacetobacter*	*azotocaptans*	
Z0015_RB_D03_1_C11_A	GREEN	No	208	36	LMG 21311	*Gluconacetobacter*	*azotocaptans*	
Z0015_RB_D09_1_C08_A	GREEN	No	208	36	LMG 21311	*Gluconacetobacter*	*azotocaptans*	
Z0015_LB_E04_2_B05_B	GREEN	No	208	36	LMG 21311	*Gluconacetobacter*	*azotocaptans*	
Z0015_LB_E09_2_B02_A	GREEN	No	208	36	LMG 21311	*Gluconacetobacter*	*azotocaptans*	
Z0015_LB_F02_1_B06_B	GREEN	No	208	36	LMG 21311	*Gluconacetobacter*	*azotocaptans*	
Z0015_LB_F04_1_B05_B	GREEN	No	208	36	LMG 21311	*Gluconacetobacter*	*azotocaptans*	
Z0015_RB_D04_1_C11_B	GREEN	No	208	36	LMG 21311	*Gluconacetobacter*	*azotocaptans*	
Z0015_LB_E05_2_B04_A	GREEN	No	208	36	LMG 21311	*Gluconacetobacter*	*azotocaptans*	
Z0015_LB_E06_2_B04_B	GREEN	No	208	36	LMG 21311	*Gluconacetobacter*	*azotocaptans*	
Z0015_LB_F03_1_B05_A	GREEN	No	208	36	LMG 21311	*Gluconacetobacter*	*azotocaptans*	
Z0015_LB_E11_2_B01_A	GREEN	No	208	36	LMG 21311	*Gluconacetobacter*	*azotocaptans*	
Z0021_RO_H03_2_H08_A	GREEN	Yes	209	105	LMG 28391	*Salininema*	*proteolyticum*	
Z0021_RO_G02_1_H07_B	GREEN	No	209	105	LMG 28391	*Salininema*	*proteolyticum*	
Z0021_LO_G09_1_H05_A	GREEN	No	209	105	LMG 28391	*Salininema*	*proteolyticum*	
Z0021_LO_G10_1_H05_B	GREEN	No	209	105	LMG 28391	*Salininema*	*proteolyticum*	
Z0021_RO_G05_1_H09_A	GREEN	No	209	105	LMG 28391	*Salininema*	*proteolyticum*	
Z0021_RO_H01_2_H07_A	GREEN	No	209	105	LMG 28391	*Salininema*	*proteolyticum*	
Z0021_LO_H04_2_H02_B	GREEN	No	209	105	LMG 28391	*Salininema*	*proteolyticum*	
Z0021_RO_F06_2_G09_B	GREEN	No	209	105	LMG 28391	*Salininema*	*proteolyticum*	

Z0021_RO_F11_2_G12_A	GREEN	No	209	105	LMG 28391	*Salininema*	*proteolyticum*
Z0021_LO_H06_2_H03_B	GREEN	No	209	105	LMG 28391	*Salininema*	*proteolyticum*
Z0021_RO_G07_1_H10_A	GREEN	No	209	105	LMG 28391	*Salininema*	*proteolyticum*
Z0021_RO_G06_1_H09_B	GREEN	No	209	105	LMG 28391	*Salininema*	*proteolyticum*
Z0021_RO_G03_1_H08_A	GREEN	No	209	105	LMG 28391	*Salininema*	*proteolyticum*
Z0021_LO_G06_1_H03_B	GREEN	No	209	105	LMG 28391	*Salininema*	*proteolyticum*
Z0021_RO_G08_1_H10_B	GREEN	No	209	105	LMG 28391	*Salininema*	*proteolyticum*
Z0021_RO_F12_2_G12_B	GREEN	No	209	105	LMG 28391	*Salininema*	*proteolyticum*
Z0021_LO_G08_1_H04_B	GREEN	No	209	105	LMG 28391	*Salininema*	*proteolyticum*
Z0021_LO_H01_2_H01_A	GREEN	No	209	105	LMG 28391	*Salininema*	*proteolyticum*
Z0021_RO_F10_2_G11_B	GREEN	No	209	105	LMG 28391	*Salininema*	*proteolyticum*
Z0021_RO_H04_2_H08_B	GREEN	No	209	105	LMG 28391	*Salininema*	*proteolyticum*
Z0021_LO_F09_2_G05_A	GREEN	No	209	105	LMG 28391	*Salininema*	*proteolyticum*
Z0021_LO_F11_2_G06_A	GREEN	No	209	105	LMG 28391	*Salininema*	*proteolyticum*
Z0021_LO_F10_2_G05_B	GREEN	No	209	105	LMG 28391	*Salininema*	*proteolyticum*
Z0021_RO_G11_1_H12_A	GREEN	No	209	105	LMG 28391	*Salininema*	*proteolyticum*
Z0021_RO_G01_1_H07_A	GREEN	No	209	105	LMG 28391	*Salininema*	*proteolyticum*
Z0021_LO_H05_2_H03_A	GREEN	No	209	105	LMG 28391	*Salininema*	*proteolyticum*
Z0021_LO_G11_1_H06_A	GREEN	No	209	105	LMG 28391	*Salininema*	*proteolyticum*
Z0021_LO_H03_2_H02_A	GREEN	No	209	105	LMG 28391	*Salininema*	*proteolyticum*
Z0021_RO_G12_1_H12_B	GREEN	No	209	105	LMG 28391	*Salininema*	*proteolyticum*
Z0021_LO_G12_1_H06_B	GREEN	No	209	105	LMG 28391	*Salininema*	*proteolyticum*
Z0021_RO_H02_2_H07_B	GREEN	No	209	105	LMG 28391	*Salininema*	*proteolyticum*
Z0021_RO_G09_1_H11_A	GREEN	No	209	105	LMG 28391	*Salininema*	*proteolyticum*
**Low quality samples:**							
Z0013_RO_D06_2_F09_B	ORANGE	No	0	19	LMG 15863	*Haemophilus*	*influenzae*
Z0013_LB_D04_1_C05_B	ORANGE	No	1	19	LMG 15863	*Haemophilus*	*influenzae*
Z0013_LO_D04_2_F02_B	ORANGE	No	1	19	LMG 15863	*Haemophilus*	*influenzae*
Z0013_RO_D06_2_F09_B	ORANGE	No	1	19	LMG 15863	*Haemophilus*	*influenzae*
Z0013_RO_D06_2_F09_B	ORANGE	No	16	19	LMG 15863	*Haemophilus*	*influenzae*
Z0013_LB_D05_1_C04_A	ORANGE	No	22	19	LMG 15863	*Haemophilus*	*influenzae*
Z0020_LO_F01_2_G01_A	ORANGE	No	22	95	LMG 2698	*Brenneria*	*salicis*
Z0020_LO_F01_2_G01_A	ORANGE	No	23	95	LMG 2698	*Brenneria*	*salicis*
Z0020_LO_F01_2_G01_A	ORANGE	No	24	95	LMG 2698	*Brenneria*	*salicis*
Z0013_LB_D05_1_C04_A	ORANGE	No	27	19	LMG 15863	*Haemophilus*	*influenzae*
Z0013_LB_D08_1_C03_B	ORANGE	No	27	19	LMG 15863	*Haemophilus*	*influenzae*
Z0013_RB_D02_1_C12_B	ORANGE	No	27	19	LMG 15863	*Haemophilus*	*influenzae*
Z0013_RB_D05_1_C10_A	ORANGE	No	27	19	LMG 15863	*Haemophilus*	*influenzae*
Z0013_LB_D05_1_C04_A	ORANGE	No	28	19	LMG 15863	*Haemophilus*	*influenzae*
Z0013_RB_D05_1_C10_A	ORANGE	No	28	19	LMG 15863	*Haemophilus*	*influenzae*
Z0013_LB_D05_1_C04_A	ORANGE	No	31	19	LMG 15863	*Haemophilus*	*influenzae*
Z0013_RO_D06_2_F09_B	ORANGE	No	31	19	LMG 15863	*Haemophilus*	*influenzae*
Z0013_LB_D08_1_C03_B	ORANGE	No	40	19	LMG 15863	*Haemophilus*	*influenzae*
Z0020_LO_F01_2_G01_A	ORANGE	No	40	95	LMG 2698	*Brenneria*	*salicis*
Z0013_LB_D05_1_C04_A	ORANGE	No	43	19	LMG 15863	*Haemophilus*	*influenzae*
Z0020_LO_F01_2_G01_A	ORANGE	No	43	95	LMG 2698	*Brenneria*	*salicis*
Z0013_LB_D05_1_C04_A	ORANGE	No	45	19	LMG 15863	*Haemophilus*	*influenzae*
Z0013_RB_D05_1_C10_A	ORANGE	No	45	19	LMG 15863	*Haemophilus*	*influenzae*
Z0013_LB_D05_1_C04_A	ORANGE	No	46	19	LMG 15863	*Haemophilus*	*influenzae*
Z0013_RB_D05_1_C10_A	ORANGE	No	46	19	LMG 15863	*Haemophilus*	*influenzae*
Z0013_LB_D05_1_C04_A	ORANGE	No	47	19	LMG 15863	*Haemophilus*	*influenzae*
Z0013_RB_D05_1_C10_A	ORANGE	No	47	19	LMG 15863	*Haemophilus*	*influenzae*
Z0013_LB_D05_1_C04_A	ORANGE	No	48	19	LMG 15863	*Haemophilus*	*influenzae*
Z0013_RB_D05_1_C10_A	ORANGE	No	48	19	LMG 15863	*Haemophilus*	*influenzae*
Z0013_LB_D05_1_C04_A	ORANGE	No	49	19	LMG 15863	*Haemophilus*	*influenzae*
Z0013_RB_D05_1_C10_A	ORANGE	No	49	19	LMG 15863	*Haemophilus*	*influenzae*
Z0013_LB_D05_1_C04_A	ORANGE	No	50	19	LMG 15863	*Haemophilus*	*influenzae*
Z0013_RB_D05_1_C10_A	ORANGE	No	50	19	LMG 15863	*Haemophilus*	*influenzae*
Z0013_LB_D05_1_C04_A	ORANGE	No	53	19	LMG 15863	*Haemophilus*	*influenzae*
Z0013_LB_D08_1_C03_B	ORANGE	No	53	19	LMG 15863	*Haemophilus*	*influenzae*
Z0013_RB_D02_1_C12_B	ORANGE	No	53	19	LMG 15863	*Haemophilus*	*influenzae*
Z0013_RB_D05_1_C10_A	ORANGE	No	53	19	LMG 15863	*Haemophilus*	*influenzae*
Z0020_LO_F01_2_G01_A	ORANGE	No	54	95	LMG 2698	*Brenneria*	*salicis*
Z0013_LB_D05_1_C04_A	ORANGE	No	59	19	LMG 15863	*Haemophilus*	*influenzae*
Z0020_LO_F01_2_G01_A	ORANGE	No	59	95	LMG 2698	*Brenneria*	*salicis*
Z0013_LB_D05_1_C04_A	ORANGE	No	65	19	LMG 15863	*Haemophilus*	*influenzae*
Z0013_LB_D08_1_C03_B	ORANGE	No	65	19	LMG 15863	*Haemophilus*	*influenzae*
Z0020_LO_F01_2_G01_A	ORANGE	No	65	95	LMG 2698	*Brenneria*	*salicis*
Z0013_LB_D04_1_C05_B	ORANGE	No	67	19	LMG 15863	*Haemophilus*	*influenzae*

Z0013_RB_D05_1_C10_A	ORANGE	No	67	19	LMG 15863	*Haemophilus*	*influenzae*
Z0013_LB_D05_1_C04_A	ORANGE	No	69	19	LMG 15863	*Haemophilus*	*influenzae*
Z0013_LB_D08_1_C03_B	ORANGE	No	69	19	LMG 15863	*Haemophilus*	*influenzae*
Z0020_LO_F01_2_G01_A	ORANGE	No	69	95	LMG 2698	*Brenneria*	*salicis*
Z0013_LB_D05_1_C04_A	ORANGE	No	75	19	LMG 15863	*Haemophilus*	*influenzae*
Z0013_LB_D08_1_C03_B	ORANGE	No	75	19	LMG 15863	*Haemophilus*	*influenzae*
Z0013_LB_D04_1_C05_B	ORANGE	No	82	19	LMG 15863	*Haemophilus*	*influenzae*
Z0013_LB_D05_1_C04_A	ORANGE	No	82	19	LMG 15863	*Haemophilus*	*influenzae*
Z0013_LB_D08_1_C03_B	ORANGE	No	82	19	LMG 15863	*Haemophilus*	*influenzae*
Z0013_LO_D04_2_F02_B	ORANGE	No	82	19	LMG 15863	*Haemophilus*	*influenzae*
Z0013_RB_D02_1_C12_B	ORANGE	No	82	19	LMG 15863	*Haemophilus*	*influenzae*
Z0013_RB_D05_1_C10_A	ORANGE	No	82	19	LMG 15863	*Haemophilus*	*influenzae*
Z0013_RO_D06_2_F09_B	ORANGE	No	82	19	LMG 15863	*Haemophilus*	*influenzae*
Z0013_LB_D04_1_C05_B	ORANGE	No	85	19	LMG 15863	*Haemophilus*	*influenzae*
Z0013_LB_D05_1_C04_A	ORANGE	No	85	19	LMG 15863	*Haemophilus*	*influenzae*
Z0013_LO_D04_2_F02_B	ORANGE	No	85	19	LMG 15863	*Haemophilus*	*influenzae*
Z0013_RB_D02_1_C12_B	ORANGE	No	85	19	LMG 15863	*Haemophilus*	*influenzae*
Z0013_RB_D05_1_C10_A	ORANGE	No	85	19	LMG 15863	*Haemophilus*	*influenzae*
Z0013_RO_D06_2_F09_B	ORANGE	No	85	19	LMG 15863	*Haemophilus*	*influenzae*
Z0013_RB_D02_1_C12_B	ORANGE	No	87	19	LMG 15863	*Haemophilus*	*influenzae*
Z0013_RB_D05_1_C10_A	ORANGE	No	87	19	LMG 15863	*Haemophilus*	*influenzae*
Z0013_RB_D02_1_C12_B	ORANGE	No	90	19	LMG 15863	*Haemophilus*	*influenzae*
Z0013_RB_D05_1_C10_A	ORANGE	No	90	19	LMG 15863	*Haemophilus*	*influenzae*
Z0013_LB_D05_1_C04_A	ORANGE	No	96	19	LMG 15863	*Haemophilus*	*influenzae*
Z0020_LO_F01_2_G01_A	ORANGE	No	96	95	LMG 2698	*Brenneria*	*salicis*
Z0013_LB_D04_1_C05_B	ORANGE	No	98	19	LMG 15863	*Haemophilus*	*influenzae*
Z0020_LO_F01_2_G01_A	ORANGE	No	100	95	LMG 2698	*Brenneria*	*salicis*
Z0020_LO_F01_2_G01_A	ORANGE	No	101	95	LMG 2698	*Brenneria*	*salicis*
Z0013_LB_D05_1_C04_A	ORANGE	No	102	19	LMG 15863	*Haemophilus*	*influenzae*
Z0020_LO_F01_2_G01_A	ORANGE	No	102	95	LMG 2698	*Brenneria*	*salicis*
Z0013_LB_D05_1_C04_A	ORANGE	No	103	19	LMG 15863	*Haemophilus*	*influenzae*
Z0020_LO_F01_2_G01_A	ORANGE	No	103	95	LMG 2698	*Brenneria*	*salicis*
Z0013_LB_D05_1_C04_A	ORANGE	No	104	19	LMG 15863	*Haemophilus*	*influenzae*
Z0020_LO_F01_2_G01_A	ORANGE	No	104	95	LMG 2698	*Brenneria*	*salicis*
Z0020_LO_F01_2_G01_A	ORANGE	No	105	95	LMG 2698	*Brenneria*	*salicis*
Z0013_LB_D05_1_C04_A	ORANGE	No	106	19	LMG 15863	*Haemophilus*	*influenzae*
Z0020_LO_F01_2_G01_A	ORANGE	No	106	95	LMG 2698	*Brenneria*	*salicis*
Z0013_RB_D02_1_C12_B	ORANGE	No	107	19	LMG 15863	*Haemophilus*	*influenzae*
Z0013_RB_D05_1_C10_A	ORANGE	No	107	19	LMG 15863	*Haemophilus*	*influenzae*
Z0013_LB_D08_1_C03_B	ORANGE	No	112	19	LMG 15863	*Haemophilus*	*influenzae*
Z0020_LO_F01_2_G01_A	ORANGE	No	112	95	LMG 2698	*Brenneria*	*salicis*
Z0013_LB_D04_1_C05_B	ORANGE	No	123	19	LMG 15863	*Haemophilus*	*influenzae*
Z0013_LB_D05_1_C04_A	ORANGE	No	123	19	LMG 15863	*Haemophilus*	*influenzae*
Z0013_LB_D08_1_C03_B	ORANGE	No	123	19	LMG 15863	*Haemophilus*	*influenzae*
Z0013_LO_D04_2_F02_B	ORANGE	No	123	19	LMG 15863	*Haemophilus*	*influenzae*
Z0013_RB_D02_1_C12_B	ORANGE	No	123	19	LMG 15863	*Haemophilus*	*influenzae*
Z0013_RB_D05_1_C10_A	ORANGE	No	123	19	LMG 15863	*Haemophilus*	*influenzae*
Z0013_RO_D06_2_F09_B	ORANGE	No	123	19	LMG 15863	*Haemophilus*	*influenzae*
Z0020_LO_F01_2_G01_A	ORANGE	No	125	95	LMG 2698	*Brenneria*	*salicis*
Z0013_LB_D05_1_C04_A	ORANGE	No	126	19	LMG 15863	*Haemophilus*	*influenzae*
Z0013_LB_D08_1_C03_B	ORANGE	No	126	19	LMG 15863	*Haemophilus*	*influenzae*
Z0013_LB_D05_1_C04_A	ORANGE	No	127	19	LMG 15863	*Haemophilus*	*influenzae*
Z0013_LB_D05_1_C04_A	ORANGE	No	131	19	LMG 15863	*Haemophilus*	*influenzae*
Z0020_LO_F01_2_G01_A	ORANGE	No	131	95	LMG 2698	*Brenneria*	*salicis*
Z0013_LB_D05_1_C04_A	ORANGE	No	132	19	LMG 15863	*Haemophilus*	*influenzae*
Z0013_LB_D08_1_C03_B	ORANGE	No	132	19	LMG 15863	*Haemophilus*	*influenzae*
Z0013_LB_D04_1_C05_B	ORANGE	No	134	19	LMG 15863	*Haemophilus*	*influenzae*
Z0013_LB_D05_1_C04_A	ORANGE	No	134	19	LMG 15863	*Haemophilus*	*influenzae*
Z0013_LB_D08_1_C03_B	ORANGE	No	134	19	LMG 15863	*Haemophilus*	*influenzae*
Z0013_LO_D04_2_F02_B	ORANGE	No	134	19	LMG 15863	*Haemophilus*	*influenzae*
Z0013_RB_D02_1_C12_B	ORANGE	No	134	19	LMG 15863	*Haemophilus*	*influenzae*
Z0013_RB_D05_1_C10_A	ORANGE	No	134	19	LMG 15863	*Haemophilus*	*influenzae*
Z0013_RO_D06_2_F09_B	ORANGE	No	134	19	LMG 15863	*Haemophilus*	*influenzae*
Z0020_LO_F01_2_G01_A	ORANGE	No	137	95	LMG 2698	*Brenneria*	*salicis*
Z0013_LB_D05_1_C04_A	ORANGE	No	138	19	LMG 15863	*Haemophilus*	*influenzae*
Z0020_LO_F01_2_G01_A	ORANGE	No	138	95	LMG 2698	*Brenneria*	*salicis*
Z0013_LB_D05_1_C04_A	ORANGE	No	140	19	LMG 15863	*Haemophilus*	*influenzae*
Z0020_LO_F01_2_G01_A	ORANGE	No	140	95	LMG 2698	*Brenneria*	*salicis*
Z0013_LB_D08_1_C03_B	ORANGE	No	142	19	LMG 15863	*Haemophilus*	*influenzae*
Z0020_LO_F01_2_G01_A	ORANGE	No	142	95	LMG 2698	*Brenneria*	*salicis*

Z0013_LB_D08_1_C03_B	ORANGE	No	144	19	LMG 15863	*Haemophilus*	*influenzae*
Z0013_RO_D06_2_F09_B	ORANGE	No	144	19	LMG 15863	*Haemophilus*	*influenzae*
Z0013_LB_D05_1_C04_A	ORANGE	No	148	19	LMG 15863	*Haemophilus*	*influenzae*
Z0013_LB_D08_1_C03_B	ORANGE	No	148	19	LMG 15863	*Haemophilus*	*influenzae*
Z0020_LO_F01_2_G01_A	ORANGE	No	151	95	LMG 2698	*Brenneria*	*salicis*
Z0013_LB_D05_1_C04_A	ORANGE	No	160	19	LMG 15863	*Haemophilus*	*influenzae*
Z0013_LB_D08_1_C03_B	ORANGE	No	160	19	LMG 15863	*Haemophilus*	*influenzae*
Z0020_LO_F01_2_G01_A	ORANGE	No	160	95	LMG 2698	*Brenneria*	*salicis*
Z0013_LB_D05_1_C04_A	ORANGE	No	161	19	LMG 15863	*Haemophilus*	*influenzae*
Z0013_LB_D08_1_C03_B	ORANGE	No	161	19	LMG 15863	*Haemophilus*	*influenzae*
Z0020_LO_F01_2_G01_A	ORANGE	No	161	95	LMG 2698	*Brenneria*	*salicis*
Z0013_LB_D05_1_C04_A	ORANGE	No	165	19	LMG 15863	*Haemophilus*	*influenzae*
Z0020_LO_F01_2_G01_A	ORANGE	No	165	95	LMG 2698	*Brenneria*	*salicis*
Z0013_LB_D05_1_C04_A	ORANGE	No	166	19	LMG 15863	*Haemophilus*	*influenzae*
Z0013_LB_D08_1_C03_B	ORANGE	No	170	19	LMG 15863	*Haemophilus*	*influenzae*
Z0013_RO_D06_2_F09_B	ORANGE	No	170	19	LMG 15863	*Haemophilus*	*influenzae*
Z0013_LB_D05_1_C04_A	ORANGE	No	171	19	LMG 15863	*Haemophilus*	*influenzae*
Z0020_LO_F01_2_G01_A	ORANGE	No	171	95	LMG 2698	*Brenneria*	*salicis*
Z0013_LB_D04_1_C05_B	ORANGE	No	173	19	LMG 15863	*Haemophilus*	*influenzae*
Z0013_LB_D04_1_C05_B	ORANGE	No	175	19	LMG 15863	*Haemophilus*	*influenzae*
Z0013_LB_D05_1_C04_A	ORANGE	No	175	19	LMG 15863	*Haemophilus*	*influenzae*
Z0013_LB_D08_1_C03_B	ORANGE	No	175	19	LMG 15863	*Haemophilus*	*influenzae*
Z0013_LO_D04_2_F02_B	ORANGE	No	175	19	LMG 15863	*Haemophilus*	*influenzae*
Z0013_RB_D02_1_C12_B	ORANGE	No	175	19	LMG 15863	*Haemophilus*	*influenzae*
Z0013_RB_D05_1_C10_A	ORANGE	No	175	19	LMG 15863	*Haemophilus*	*influenzae*
Z0013_RO_D06_2_F09_B	ORANGE	No	175	19	LMG 15863	*Haemophilus*	*influenzae*
Z0013_LB_D08_1_C03_B	ORANGE	No	178	19	LMG 15863	*Haemophilus*	*influenzae*
Z0013_LB_D05_1_C04_A	ORANGE	No	179	19	LMG 15863	*Haemophilus*	*influenzae*
Z0013_LB_D08_1_C03_B	ORANGE	No	179	19	LMG 15863	*Haemophilus*	*influenzae*
Z0013_RB_D02_1_C12_B	ORANGE	No	180	19	LMG 15863	*Haemophilus*	*influenzae*
Z0013_RB_D05_1_C10_A	ORANGE	No	180	19	LMG 15863	*Haemophilus*	*influenzae*
Z0013_LB_D05_1_C04_A	ORANGE	No	182	19	LMG 15863	*Haemophilus*	*influenzae*
Z0013_LB_D08_1_C03_B	ORANGE	No	182	19	LMG 15863	*Haemophilus*	*influenzae*
Z0013_LB_D04_1_C05_B	ORANGE	No	190	19	LMG 15863	*Haemophilus*	*influenzae*
Z0013_LB_D05_1_C04_A	ORANGE	No	190	19	LMG 15863	*Haemophilus*	*influenzae*
Z0013_LO_D04_2_F02_B	ORANGE	No	190	19	LMG 15863	*Haemophilus*	*influenzae*
Z0013_RB_D02_1_C12_B	ORANGE	No	190	19	LMG 15863	*Haemophilus*	*influenzae*
Z0013_RB_D05_1_C10_A	ORANGE	No	190	19	LMG 15863	*Haemophilus*	*influenzae*
Z0013_RO_D06_2_F09_B	ORANGE	No	190	19	LMG 15863	*Haemophilus*	*influenzae*
Z0020_LO_F01_2_G01_A	ORANGE	No	194	95	LMG 2698	*Brenneria*	*salicis*
Z0013_LB_D05_1_C04_A	ORANGE	No	195	19	LMG 15863	*Haemophilus*	*influenzae*
Z0013_LO_D04_2_F02_B	ORANGE	No	195	19	LMG 15863	*Haemophilus*	*influenzae*
Z0013_RB_D02_1_C12_B	ORANGE	No	195	19	LMG 15863	*Haemophilus*	*influenzae*
Z0013_RB_D05_1_C10_A	ORANGE	No	195	19	LMG 15863	*Haemophilus*	*influenzae*
Z0013_RO_D06_2_F09_B	ORANGE	No	195	19	LMG 15863	*Haemophilus*	*influenzae*
Z0013_LB_D04_1_C05_B	ORANGE	No	197	19	LMG 15863	*Haemophilus*	*influenzae*
Z0013_RB_D05_1_C10_A	ORANGE	No	197	19	LMG 15863	*Haemophilus*	*influenzae*
Z0013_LB_D05_1_C04_A	ORANGE	No	202	19	LMG 15863	*Haemophilus*	*influenzae*
Z0013_LB_D08_1_C03_B	ORANGE	No	202	19	LMG 15863	*Haemophilus*	*influenzae*
Z0013_RB_D02_1_C12_B	ORANGE	No	202	19	LMG 15863	*Haemophilus*	*influenzae*
Z0013_RB_D05_1_C10_A	ORANGE	No	202	19	LMG 15863	*Haemophilus*	*influenzae*
Z0013_LB_D05_1_C04_A	ORANGE	No	203	19	LMG 15863	*Haemophilus*	*influenzae*
Z0020_LO_F01_2_G01_A	ORANGE	No	203	95	LMG 2698	*Brenneria*	*salicis*
Z0013_LB_D04_1_C05_B	ORANGE	No	205	19	LMG 15863	*Haemophilus*	*influenzae*
Z0013_LB_D05_1_C04_A	ORANGE	No	205	19	LMG 15863	*Haemophilus*	*influenzae*
Z0013_LB_D08_1_C03_B	ORANGE	No	205	19	LMG 15863	*Haemophilus*	*influenzae*
Z0013_LO_D04_2_F02_B	ORANGE	No	205	19	LMG 15863	*Haemophilus*	*influenzae*
Z0013_RB_D02_1_C12_B	ORANGE	No	205	19	LMG 15863	*Haemophilus*	*influenzae*
Z0013_RB_D05_1_C10_A	ORANGE	No	205	19	LMG 15863	*Haemophilus*	*influenzae*
Z0013_RO_D06_2_F09_B	ORANGE	No	205	19	LMG 15863	*Haemophilus*	*influenzae*
Z0013_LB_D05_1_C04_A	ORANGE	No	206	19	LMG 15863	*Haemophilus*	*influenzae*
Z0013_LB_D08_1_C03_B	ORANGE	No	206	19	LMG 15863	*Haemophilus*	*influenzae*
Z0020_LO_F01_2_G01_A	ORANGE	No	206	95	LMG 2698	*Brenneria*	*salicis*
Z0013_LB_D05_1_C04_A	ORANGE	No	207	19	LMG 15863	*Haemophilus*	*influenzae*
Z0013_LO_D04_2_F02_B	ORANGE	No	207	19	LMG 15863	*Haemophilus*	*influenzae*
Z0013_RB_D02_1_C12_B	ORANGE	No	207	19	LMG 15863	*Haemophilus*	*influenzae*
Z0013_RB_D05_1_C10_A	ORANGE	No	207	19	LMG 15863	*Haemophilus*	*influenzae*
Z0013_RO_D06_2_F09_B	ORANGE	No	207	19	LMG 15863	*Haemophilus*	*influenzae*
Z0013_LB_D04_1_C05_B	ORANGE	No	208	19	LMG 15863	*Haemophilus*	*influenzae*
Z0013_LO_D04_2_F02_B	ORANGE	No	208	19	LMG 15863	*Haemophilus*	*influenzae*

Z0013_RB_D02_1_C12_B	ORANGE	No	208	19	LMG 15863	*Haemophilus*	*influenzae*
Z0013_RB_D05_1_C10_A	ORANGE	No	208	19	LMG 15863	*Haemophilus*	*influenzae*
Z0013_RO_D06_2_F09_B	ORANGE	No	208	19	LMG 15863	*Haemophilus*	*influenzae*

Not matched:

SOURCE_FILE	QUALITY						
Z0012_LB_B05_1_D04_A	ORANGE			4	LMG 11405	*Lactobacillus*	*plantarum*
Z0012_LB_B06_1_D04_B	ORANGE			4	LMG 11405	*Lactobacillus*	*plantarum*
Z0012_LB_B07_1_D03_A	ORANGE			4	LMG 11405	*Lactobacillus*	*plantarum*
Z0012_LB_C01_2_C06_A	ORANGE			4	LMG 11405	*Lactobacillus*	*plantarum*
Z0012_LB_C03_2_C05_A	ORANGE			4	LMG 11405	*Lactobacillus*	*plantarum*
Z0012_LB_C04_2_C05_B	ORANGE			4	LMG 11405	*Lactobacillus*	*plantarum*
Z0012_LB_C05_2_C04_A	ORANGE			4	LMG 11405	*Lactobacillus*	*plantarum*
Z0012_LB_C06_2_C04_B	ORANGE			4	LMG 11405	*Lactobacillus*	*plantarum*
Z0012_LB_C08_2_C03_B	ORANGE			4	LMG 11405	*Lactobacillus*	*plantarum*
Z0012_LB_C09_2_C02_A	ORANGE			4	LMG 11405	*Lactobacillus*	*plantarum*
Z0012_LB_C10_2_C02_B	ORANGE			4	LMG 11405	*Lactobacillus*	*plantarum*
Z0012_RB_B04_1_D11_B	ORANGE			4	LMG 11405	*Lactobacillus*	*plantarum*
Z0012_RB_B07_1_D09_A	ORANGE			4	LMG 11405	*Lactobacillus*	*plantarum*
Z0012_RB_B08_1_D09_B	ORANGE			4	LMG 11405	*Lactobacillus*	*plantarum*
Z0012_RB_B09_1_D08_A	ORANGE			4	LMG 11405	*Lactobacillus*	*plantarum*
Z0012_RB_B12_1_D07_B	ORANGE			4	LMG 11405	*Lactobacillus*	*plantarum*
Z0012_RB_C02_2_C12_B	ORANGE			4	LMG 11405	*Lactobacillus*	*plantarum*
Z0012_RB_C03_2_C11_A	ORANGE			4	LMG 11405	*Lactobacillus*	*plantarum*
Z0012_RB_C04_2_C11_B	ORANGE			4	LMG 11405	*Lactobacillus*	*plantarum*
Z0012_RB_C05_2_C10_A	ORANGE			4	LMG 11405	*Lactobacillus*	*plantarum*

Rejected spectra:

Z0017_LB_A03_2_D05_A	RED			55	LMG 23170	*Saccharibacter*	*floricola*
Z0017_LB_A04_2_D05_B	RED			55	LMG 23170	*Saccharibacter*	*floricola*
Z0017_LO_H03_2_H02_A	RED			55	LMG 23170	*Saccharibacter*	*floricola*
Z0017_LO_H04_2_H02_B	RED			55	LMG 23170	*Saccharibacter*	*floricola*
