# Supplementary material for: Introducing SPeDE: High-Throughput Dereplication and Accurate Determination of Microbial Diversity from Matrix-Assisted Laser Desorption–Ionization Time of Flight Mass Spectrometry Data
Source: mSystems. 2019 Sep 10;4(5):e00437-19. doi: 10.1128/mSystems.00437-19 (PMC6739102; doi:10.1128/mSystems.00437-19)
Supplement: TABLE S2 [file mSystems.00437-19-st002.pdf]

Table S2: Results of SPeDE dereplication of the lyophilization data set

Spectrum file	Quality	Reference	Reference number	Genus	Species	Strain	Before or after lyophilization
Q0408_RB_H07_1_A09_A	GREEN	No	65	*Enterococcus*	*hirae*	LMG10274	before
Q0408_RB_H06_1_A10_B	GREEN	No	65	*Enterococcus*	*hirae*	LMG10274	before
Q0408_LO_A01_1_E01_A	GREEN	Yes	65	*Enterococcus*	*hirae*	LMG10274	after
Q0408_LO_A02_1_E01_B	GREEN	No	65	*Enterococcus*	*hirae*	LMG10274	after
Q0412_RO_E10_1_G11_B	GREEN	Yes	3	*Lactobacillus*	*rhamnosus*	LMG10770	before
Q0412_RO_E11_1_G12_A	GREEN	No	3	*Lactobacillus*	*rhamnosus*	LMG10770	before
Q0413_LB_A12_2_D01_B	GREEN	Yes	4	*Lactobacillus*	*rhamnosus*	LMG10770	after
Q0413_LB_A07_2_D03_A	GREEN	No	4	*Lactobacillus*	*rhamnosus*	LMG10770	after
Q0414_RB_B06_1_D10_B	GREEN	Yes	37	*Leuconostoc*	*mesenteroides*	LMG11321	before
Q0414_RB_B07_1_D09_A	GREEN	No	37	*Leuconostoc*	*mesenteroides*	LMG11321	before
Q0414_RB_B08_1_D09_B	GREEN	No	37	*Leuconostoc*	*mesenteroides*	LMG11321	after
Q0414_RB_B09_1_D08_A	GREEN	No	37	*Leuconostoc*	*mesenteroides*	LMG11321	after
Q0409_RO_G03_1_H08_A	GREEN	Yes	5	*Lactobacillus*	*amylotrophicus*	LMG11400T	before
Q0409_RO_G02_1_H07_B	GREEN	No	5	*Lactobacillus*	*amylotrophicus*	LMG11400T	before
Q0409_RO_G04_1_H08_B	GREEN	No	5	*Lactobacillus*	*amylotrophicus*	LMG11400T	after
Q0409_RO_G05_1_H09_A	GREEN	No	5	*Lactobacillus*	*amylotrophicus*	LMG11400T	after
Q0408_LO_A10_1_E05_B	GREEN	No	43	*Enterococcus*	*saccharolyticus*	LMG11427T	before
Q0408_LO_A11_1_E06_A	GREEN	No	43	*Enterococcus*	*saccharolyticus*	LMG11427T	before
Q0408_LO_B01_2_E01_A	GREEN	Yes	43	*Enterococcus*	*saccharolyticus*	LMG11427T	after
Q0408_LO_A12_1_E06_B	GREEN	No	43	*Enterococcus*	*saccharolyticus*	LMG11427T	after
Q0410_LO_E11_1_G06_A	GREEN	No	20	*Lactobacillus*	*crispatus*	LMG11440	before
Q0410_LO_E10_1_G05_B	GREEN	No	20	*Lactobacillus*	*crispatus*	LMG11440	before
Q0410_LO_F08_2_G04_B	GREEN	Yes	20	*Lactobacillus*	*crispatus*	LMG11440	after
Q0410_LO_F09_2_G05_A	GREEN	No	20	*Lactobacillus*	*crispatus*	LMG11440	after
Q0412_LB_C03_2_C05_A	GREEN	Yes	41	*Lactobacillus*	*paracasei*	LMG11961	before
Q0412_LB_C02_2_C06_B	GREEN	No	41	*Lactobacillus*	*paracasei*	LMG11961	before
Q0412_LB_C04_2_C05_B	GREEN	No	41	*Lactobacillus*	*paracasei*	LMG11961	after
Q0412_LB_C05_2_C04_A	GREEN	No	41	*Lactobacillus*	*paracasei*	LMG11961	after
Q0419_RB_E03_2_B11_A	GREEN	No	38	*Streptococcus*	*salivarius*	LMG13103	before
Q0419_RB_E02_2_B12_B	GREEN	No	38	*Streptococcus*	*salivarius*	LMG13103	before
Q0419_RB_E04_2_B11_B	GREEN	No	38	*Streptococcus*	*salivarius*	LMG13103	after
Q0419_RB_E05_2_B10_A	GREEN	No	38	*Streptococcus*	*salivarius*	LMG13103	after
Q0413_RO_A02_1_E07_B	GREEN	Yes	40	*Lactococcus*	*lactis*	LMG14418	before
Q0413_RO_A03_1_E08_A	GREEN	No	40	*Lactococcus*	*lactis*	LMG14418	before
Q0413_RO_B08_2_E10_B	GREEN	No	6	*Lactococcus*	*lactis*	LMG14418	after
Q0413_RO_B09_2_E11_A	GREEN	Yes	6	*Lactococcus*	*lactis*	LMG14418	after
Q0420_RB_H07_1_A09_A	GREEN	Yes	60	*Weissella*	*kandleri*	LMG14471T	before
Q0420_RB_H06_1_A10_B	GREEN	No	57	*Weissella*	*kandleri*	LMG14471T	before
Q0420_LO_A01_1_E01_A	GREEN	Yes	57	*Weissella*	*kandleri*	LMG14471T	after
Q0420_LO_A02_1_E01_B	GREEN	No	57	*Weissella*	*kandleri*	LMG14471T	after
Q0419_LB_D07_1_C03_A	GREEN	No	47	*Streptococcus*	*gordonii*	LMG14517	before
Q0419_LB_D06_1_C04_B	GREEN	No	47	*Streptococcus*	*gordonii*	LMG14517	before
Q0419_LB_D09_1_C02_A	GREEN	Yes	47	*Streptococcus*	*gordonii*	LMG14517	after
Q0419_LB_D08_1_C03_B	GREEN	No	47	*Streptococcus*	*gordonii*	LMG14517	after
Q0419_RB_A11_2_D07_A	GREEN	Yes	52	*Streptococcus*	*pneumoniae*	LMG14542	before
Q0419_RB_A10_2_D08_B	GREEN	No	52	*Streptococcus*	*pneumoniae*	LMG14542	before
Q0419_RB_A12_2_D07_B	GREEN	Yes	51	*Streptococcus*	*pneumoniae*	LMG14542	after
Q0419_RB_B01_1_D12_A	GREEN	No	51	*Streptococcus*	*pneumoniae*	LMG14542	after
Q0419_RB_G11_2_A07_A	GREEN	Yes	88	*Streptococcus*	*sanguinis*	LMG14637	before
Q0419_RB_G10_2_A08_B	GREEN	No	88	*Streptococcus*	*sanguinis*	LMG14637	before
Q0419_LO_A02_1_E01_B	GREEN	Yes	87	*Streptococcus*	*sanguinis*	LMG14637	after
Q0419_LO_A01_1_E01_A	GREEN	No	88	*Streptococcus*	*sanguinis*	LMG14637	after
Q0419_RB_G05_2_A10_A	GREEN	No	72	*Streptococcus*	*sanguinis*	LMG14638	before
Q0419_RB_G06_2_A10_B	GREEN	No	72	*Streptococcus*	*sanguinis*	LMG14638	before
Q0419_RB_H01_1_A12_A	GREEN	Yes	72	*Streptococcus*	*sanguinis*	LMG14638	after
Q0419_RB_G12_2_A07_B	GREEN	No	72	*Streptococcus*	*sanguinis*	LMG14638	after
Q0419_RB_D10_1_C08_B	GREEN	Yes	38	*Streptococcus*	*salivarius*	LMG14652	before
Q0419_RB_D11_1_C07_A	GREEN	No	38	*Streptococcus*	*salivarius*	LMG14652	before
Q0419_RB_E12_2_B07_B	GREEN	Yes	90	*Streptococcus*	*salivarius*	LMG14652	after
Q0419_RB_F01_1_B12_A	GREEN	No	90	*Streptococcus*	*salivarius*	LMG14652	after
Q0413_LB_H07_1_A03_A	GREEN	No	27	*Lactobacillus*	*salivarius*	LMG14806	after
Q0413_LB_H06_1_A04_B	GREEN	No	27	*Lactobacillus*	*salivarius*	LMG14806	after
Q0413_LB_G04_2_A05_B	GREEN	Yes	27	*Lactobacillus*	*salivarius*	LMG14806	before
Q0413_LB_G05_2_A04_A	GREEN	No	27	*Lactobacillus*	*salivarius*	LMG14806	before
Q0419_LB_A11_2_D01_A	GREEN	No	42	*Streptococcus*	*agalactiae*	LMG15081	before

Q0419_LB_A10_2_D02_B	GREEN	No	42	*Streptococcus*	*agalactiae*	LMG15081	before
Q0419_LB_B01_1_D06_A	GREEN	Yes	42	*Streptococcus*	*agalactiae*	LMG15081	after
Q0419_LB_A12_2_D01_B	GREEN	No	42	*Streptococcus*	*agalactiae*	LMG15081	after
Q0420_RB_C05_2_C10_A	GREEN	No	80	*Weissella*	*confusa*	LMG16883	before
Q0420_RB_B12_1_D07_B	GREEN	No	80	*Weissella*	*confusa*	LMG16883	before
Q0420_RB_D03_1_C11_A	GREEN	Yes	80	*Weissella*	*confusa*	LMG16883	after
Q0420_RB_D01_1_C12_A	GREEN	No	80	*Weissella*	*confusa*	LMG16883	after
Q0412_RO_E03_1_G08_A	GREEN	No	4	*Lactobacillus*	*rhamnosus*	LMG18028	before
Q0412_RO_E02_1_G07_B	GREEN	No	4	*Lactobacillus*	*rhamnosus*	LMG18028	before
Q0413_LB_A08_2_D03_B	GREEN	No	4	*Lactobacillus*	*rhamnosus*	LMG18028	after
Q0413_LB_A04_2_D05_B	GREEN	No	4	*Lactobacillus*	*rhamnosus*	LMG18028	after
Q0420_RB_C04_2_C11_B	GREEN	No	82	*Weissella*	*confusa*	LMG18476	before
Q0420_RB_C06_2_C10_B	GREEN	No	82	*Weissella*	*confusa*	LMG18476	before
Q0420_RB_D05_1_C10_A	GREEN	No	82	*Weissella*	*confusa*	LMG18476	after
Q0420_RB_D08_1_C09_B	GREEN	No	82	*Weissella*	*confusa*	LMG18476	after
Q0420_RB_C01_2_C12_A	GREEN	Yes	81	*Weissella*	*confusa*	LMG18500	before
Q0420_RB_C08_2_C09_B	GREEN	No	81	*Weissella*	*confusa*	LMG18500	before
Q0420_RB_E04_2_B11_B	GREEN	No	80	*Weissella*	*confusa*	LMG18500	after
Q0420_RB_D07_1_C09_A	GREEN	No	80	*Weissella*	*confusa*	LMG18500	after
Q0420_RB_C10_2_C08_B	GREEN	No	82	*Weissella*	*confusa*	LMG18503	before
Q0420_RB_C02_2_C12_B	GREEN	No	82	*Weissella*	*confusa*	LMG18503	before
Q0420_RB_D11_1_C07_A	GREEN	Yes	82	*Weissella*	*confusa*	LMG18503	after
Q0420_RB_C12_2_C07_B	GREEN	No	82	*Weissella*	*confusa*	LMG18503	after
Q0420_RB_C11_2_C07_A	GREEN	No	80	*Weissella*	*confusa*	LMG18815	before
Q0420_RB_C09_2_C08_A	GREEN	No	80	*Weissella*	*confusa*	LMG18815	before
Q0420_RB_D04_1_C11_B	GREEN	No	80	*Weissella*	*confusa*	LMG18815	after
Q0420_RB_D02_1_C12_B	GREEN	No	80	*Weissella*	*confusa*	LMG18815	after
Q0414_LB_C03_2_C05_A	GREEN	Yes	84	*Leuconostoc*	*carnosum*	LMG18868	before
Q0414_LB_C02_2_C06_B	GREEN	No	84	*Leuconostoc*	*carnosum*	LMG18868	before
Q0414_LB_C05_2_C04_A	GREEN	Yes	46	*Leuconostoc*	*carnosum*	LMG18868	after
Q0414_LB_C04_2_C05_B	GREEN	No	46	*Leuconostoc*	*carnosum*	LMG18868	after
Q0411_LO_C10_1_F05_B	GREEN	Yes	89	*Lactobacillus*	*iners*	LMG18916	before
Q0411_LO_C11_1_F06_A	GREEN	No	89	*Lactobacillus*	*iners*	LMG18916	before
Q0411_LO_B08_2_E04_B	GREEN	No	89	*Lactobacillus*	*iners*	LMG18916	after
Q0411_LO_B09_2_E05_A	GREEN	No	89	*Lactobacillus*	*iners*	LMG18916	after
Q0414_RB_C10_2_C08_B	GREEN	Yes	55	*Leuconostoc*	*mesenteroides*	LMG18967	before
Q0414_RB_C11_2_C07_A	GREEN	No	55	*Leuconostoc*	*mesenteroides*	LMG18967	before
Q0414_RB_D01_1_C12_A	GREEN	No	55	*Leuconostoc*	*mesenteroides*	LMG18967	after
Q0414_RB_C12_2_C07_B	GREEN	No	55	*Leuconostoc*	*mesenteroides*	LMG18967	after
Q0414_LB_H12_1_A01_B	GREEN	Yes	53	*Leuconostoc*	*mesenteroides*	LMG18972	before
Q0414_LB_H11_1_A01_A	GREEN	No	53	*Leuconostoc*	*mesenteroides*	LMG18972	before
Q0414_RB_A04_2_D11_B	GREEN	No	53	*Leuconostoc*	*mesenteroides*	LMG18972	after
Q0414_RB_A05_2_D10_A	GREEN	No	53	*Leuconostoc*	*mesenteroides*	LMG18972	after
Q0420_LO_E11_1_G06_A	GREEN	Yes	34	*Xanthomonas*	*arboricola*	LMG19144	before
Q0420_LO_E10_1_G05_B	GREEN	No	32	*Xanthomonas*	*arboricola*	LMG19144	before
Q0420_LO_E12_1_G06_B	GREEN	Yes	32	*Xanthomonas*	*arboricola*	LMG19144	after
Q0420_LO_F01_2_G01_A	GREEN	No	32	*Xanthomonas*	*arboricola*	LMG19144	after
Q0408_RB_C12_2_C07_B	GREEN	No	59	*Enterococcus*	*faecalis*	LMG19456	before
Q0408_RB_D01_1_C12_A	GREEN	No	59	*Enterococcus*	*faecalis*	LMG19456	before
Q0408_RB_D09_1_C08_A	GREEN	Yes	59	*Enterococcus*	*faecalis*	LMG19456	after
Q0408_RB_D08_1_C09_B	GREEN	No	59	*Enterococcus*	*faecalis*	LMG19456	after
Q0414_LB_F07_1_B03_A	GREEN	No	7	*Leuconostoc*	*gelidum*	LMG19597	before
Q0414_LB_F06_1_B04_B	GREEN	No	7	*Leuconostoc*	*gelidum*	LMG19597	before
Q0414_LB_F09_1_B02_A	GREEN	No	7	*Leuconostoc*	*gelidum*	LMG19597	after
Q0414_LB_F08_1_B03_B	GREEN	Yes	7	*Leuconostoc*	*gelidum*	LMG19597	after
Q0409_LO_H07_2_H04_A	GREEN	Yes	66	*Klebsiella*	*pneumoniae*	LMG2095T	before
Q0409_LO_H06_2_H03_B	GREEN	No	67	*Klebsiella*	*pneumoniae*	LMG2095T	before
Q0409_LO_H09_2_H05_A	GREEN	Yes	67	*Klebsiella*	*pneumoniae*	LMG2095T	after
Q0409_LO_H08_2_H04_B	GREEN	No	67	*Klebsiella*	*pneumoniae*	LMG2095T	after
Q0419_RB_C02_2_C12_B	GREEN	No	95	*Streptococcus*	*pneumoniae*	LMG21598	before
Q0419_RB_C03_2_C11_A	GREEN	No	95	*Streptococcus*	*pneumoniae*	LMG21598	before
Q0419_RB_C04_2_C11_B	GREEN	Yes	95	*Streptococcus*	*pneumoniae*	LMG21598	after
Q0419_RB_C05_2_C10_A	GREEN	No	95	*Streptococcus*	*pneumoniae*	LMG21598	after
Q0409_RO_E10_1_G11_B	GREEN	No	14	*Lactobacillus*	*alimentarius*	LMG21683	before
Q0409_RO_E11_1_G12_A	GREEN	Yes	14	*Lactobacillus*	*alimentarius*	LMG21683	before
Q0409_RO_E12_1_G12_B	GREEN	No	14	*Lactobacillus*	*alimentarius*	LMG21683	after
Q0409_RO_F01_2_G07_A	GREEN	No	14	*Lactobacillus*	*alimentarius*	LMG21683	after
Q0410_RB_H12_1_A07_B	GREEN	No	2	*Lactobacillus*	*casei*	LMG23516	before
Q0410_RB_H11_1_A07_A	GREEN	Yes	2	*Lactobacillus*	*casei*	LMG23516	before
Q0410_LO_A05_1_E03_A	GREEN	No	2	*Lactobacillus*	*casei*	LMG23516	after

Q0410_LO_A04_1_E02_B	GREEN	No	2	*Lactobacillus*	*casei*	LMG23516	after
Q0412_RO_F06_2_G09_B	GREEN	No	4	*Lactobacillus*	*rhamnosus*	LMG23667	before
Q0412_RO_F07_2_G10_A	GREEN	No	4	*Lactobacillus*	*rhamnosus*	LMG23667	before
Q0413_LB_A06_2_D04_B	GREEN	No	4	*Lactobacillus*	*rhamnosus*	LMG23667	after
Q0413_LB_A05_2_D04_A	GREEN	No	4	*Lactobacillus*	*rhamnosus*	LMG23667	after
Q0414_RB_G02_2_A12_B	GREEN	No	8	*Leuconostoc*	*palmae*	LMG24510T	before
Q0414_RB_G03_2_A11_A	GREEN	No	8	*Leuconostoc*	*palmae*	LMG24510T	before
Q0414_RB_G04_2_A11_B	GREEN	No	8	*Leuconostoc*	*palmae*	LMG24510T	after
Q0414_RB_G05_2_A10_A	GREEN	Yes	8	*Leuconostoc*	*palmae*	LMG24510T	after
Q0410_LB_D06_1_C04_B	GREEN	Yes	58	*Lactobacillus*	*apodemi*	LMG25505T	after
Q0410_LB_D07_1_C03_A	GREEN	No	58	*Lactobacillus*	*apodemi*	LMG25505T	after
Q0410_LB_C05_2_C04_A	GREEN	No	25	*Lactobacillus*	*apodemi*	LMG25505T	before
Q0410_LB_C04_2_C05_B	GREEN	Yes	25	*Lactobacillus*	*apodemi*	LMG25505T	before
Q0414_RB_E10_2_B08_B	GREEN	No	54	*Leuconostoc*	*mesenteroides*	LMG25878	before
Q0414_RB_E11_2_B07_A	GREEN	No	54	*Leuconostoc*	*mesenteroides*	LMG25878	before
Q0414_RB_F01_1_B12_A	GREEN	Yes	54	*Leuconostoc*	*mesenteroides*	LMG25878	after
Q0414_RB_E12_2_B07_B	GREEN	No	54	*Leuconostoc*	*mesenteroides*	LMG25878	after
Q0421_RB_C08_2_C09_B	GREEN	Yes	61	*Xanthomonas*	*fragariae*	LMG30292	after
Q0421_RB_C07_2_C09_A	GREEN	No	44	*Xanthomonas*	*fragariae*	LMG30292	after
Q0421_RB_B12_1_D07_B	GREEN	Yes	44	*Xanthomonas*	*fragariae*	LMG30292	before
Q0421_RB_B09_1_D08_A	GREEN	No	44	*Xanthomonas*	*fragariae*	LMG30292	before
Q0409_LO_C03_1_F02_A	GREEN	Yes	74	*Klebsiella*	*kielensis*	LMG30302T	after
Q0409_LO_C02_1_F01_B	GREEN	No	74	*Klebsiella*	*kielensis*	LMG30302T	after
Q0409_LO_A04_1_E02_B	GREEN	Yes	62	*Klebsiella*	*kielensis*	LMG30302T	before
Q0409_LO_A05_1_E03_A	GREEN	No	62	*Klebsiella*	*kielensis*	LMG30302T	before
Q0409_LO_E02_1_G01_B	GREEN	No	28	*Klebsiella*	*nitrificae*	LMG30316T	after
Q0409_LO_E03_1_G02_A	GREEN	Yes	28	*Klebsiella*	*nitrificae*	LMG30316T	after
Q0409_LO_D02_2_F01_B	GREEN	No	28	*Klebsiella*	*nitrificae*	LMG30316T	before
Q0409_LO_D01_2_F01_A	GREEN	No	28	*Klebsiella*	*nitrificae*	LMG30316T	before
Q0409_LO_E10_1_G05_B	GREEN	No	21	*Klebsiella*	*oxytoca*	LMG3055T	before
Q0409_LO_E11_1_G06_A	GREEN	Yes	22	*Klebsiella*	*oxytoca*	LMG3055T	before
Q0409_LO_E12_1_G06_B	GREEN	No	21	*Klebsiella*	*oxytoca*	LMG3055T	after
Q0409_LO_F01_2_G01_A	GREEN	Yes	21	*Klebsiella*	*oxytoca*	LMG3055T	after
Q0411_RO_C02_1_F07_B	GREEN	Yes	93	*Lactobacillus*	*kunkeei*	LMG30566	after
Q0411_RO_C03_1_F08_A	GREEN	No	93	*Lactobacillus*	*kunkeei*	LMG30566	after
Q0411_RO_B01_2_E07_A	GREEN	Yes	79	*Lactobacillus*	*kunkeei*	LMG30566	before
Q0411_RO_A12_1_E12_B	GREEN	No	79	*Lactobacillus*	*kunkeei*	LMG30566	before
Q0412_LO_C02_1_F01_B	GREEN	Yes	70	*Lactobacillus*	*plantarum*	LMG30567	after
Q0412_LO_C03_1_F02_A	GREEN	No	70	*Lactobacillus*	*plantarum*	LMG30567	after
Q0412_LO_B01_2_E01_A	GREEN	Yes	69	*Lactobacillus*	*plantarum*	LMG30567	before
Q0412_LO_A12_1_E06_B	GREEN	No	69	*Lactobacillus*	*plantarum*	LMG30567	before
Q0411_LO_F06_2_G03_B	GREEN	Yes	56	*Lactobacillus*	*johnsonii*	LMG30568	after
Q0411_LO_F07_2_G04_A	GREEN	No	56	*Lactobacillus*	*johnsonii*	LMG30568	after
Q0411_LO_E05_1_G03_A	GREEN	No	56	*Lactobacillus*	*johnsonii*	LMG30568	before
Q0411_LO_E04_1_G02_B	GREEN	No	56	*Lactobacillus*	*johnsonii*	LMG30568	before
Q0409_LO_G03_1_H02_A	GREEN	Yes	83	*Klebsiella*	*pneumoniae*	LMG3116	before
Q0409_LO_G02_1_H01_B	GREEN	No	83	*Klebsiella*	*pneumoniae*	LMG3116	before
Q0409_LO_G04_1_H02_B	GREEN	No	83	*Klebsiella*	*pneumoniae*	LMG3116	after
Q0409_LO_G05_1_H03_A	GREEN	No	83	*Klebsiella*	*pneumoniae*	LMG3116	after
Q0421_LO_A06_1_E03_B	GREEN	No	48	*Xanthomonas*	*sacchari*	LMG471T	before
Q0421_LO_A04_1_E02_B	GREEN	No	48	*Xanthomonas*	*sacchari*	LMG471T	before
Q0421_LO_B04_2_E02_B	GREEN	Yes	48	*Xanthomonas*	*sacchari*	LMG471T	after
Q0421_LO_B07_2_E04_A	GREEN	Yes	49	*Xanthomonas*	*sacchari*	LMG471T	after
Q0420_RO_C03_1_F08_A	GREEN	No	17	*Xanthomonas*	*axonopodis*	LMG556	before
Q0420_RO_C02_1_F07_B	GREEN	Yes	17	*Xanthomonas*	*axonopodis*	LMG556	before
Q0420_RO_C05_1_F09_A	GREEN	No	17	*Xanthomonas*	*axonopodis*	LMG556	after
Q0420_RO_C04_1_F08_B	GREEN	No	17	*Xanthomonas*	*axonopodis*	LMG556	after
Q0421_LB_E06_2_B04_B	GREEN	Yes	77	*Xanthomonas*	*campestris*	LMG559	before
Q0421_LB_E07_2_B03_A	GREEN	No	77	*Xanthomonas*	*campestris*	LMG559	before
Q0421_LB_F06_1_B04_B	GREEN	No	45	*Xanthomonas*	*campestris*	LMG559	after
Q0421_LB_E12_2_B01_B	GREEN	Yes	45	*Xanthomonas*	*campestris*	LMG559	after
Q0421_LB_G01_2_A06_A	GREEN	No	73	*Xanthomonas*	*campestris*	LMG568	before
Q0421_LB_G03_2_A05_A	GREEN	No	73	*Xanthomonas*	*campestris*	LMG568	before
Q0421_LB_H04_1_A05_B	GREEN	Yes	73	*Xanthomonas*	*campestris*	LMG568	after
Q0421_LB_H01_1_A06_A	GREEN	No	73	*Xanthomonas*	*campestris*	LMG568	after
Q0421_RB_C12_2_C07_B	GREEN	No	15	*Xanthomonas*	*oryzae*	LMG630	before
Q0421_RB_D04_1_C11_B	GREEN	Yes	15	*Xanthomonas*	*oryzae*	LMG630	before
Q0421_RB_D09_1_C08_A	GREEN	No	16	*Xanthomonas*	*oryzae*	LMG630	after
Q0421_RB_D08_1_C09_B	GREEN	Yes	16	*Xanthomonas*	*oryzae*	LMG630	after
Q0421_RB_E07_2_B09_A	GREEN	No	13	*Xanthomonas*	*oryzae*	LMG654	before

Q0421_RB_E11_2_B07_A	GREEN	Yes	13	*Xanthomonas*	*oryzae*	LMG654	before
Q0421_RB_F03_1_B11_A	GREEN	No	13	*Xanthomonas*	*oryzae*	LMG654	after
Q0421_RB_F07_1_B09_A	GREEN	No	13	*Xanthomonas*	*oryzae*	LMG654	after
Q0413_RO_B07_2_E10_A	GREEN	No	40	*Lactococcus*	*lactis*	LMG6890T	before
Q0413_RO_B06_2_E09_B	GREEN	No	40	*Lactococcus*	*lactis*	LMG6890T	before
Q0413_RO_C05_1_F09_A	GREEN	No	40	*Lactococcus*	*lactis*	LMG6890T	after
Q0413_RO_C04_1_F08_B	GREEN	Yes	39	*Lactococcus*	*lactis*	LMG6890T	after
Q0420_RO_D06_2_F09_B	GREEN	No	19	*Xanthomonas*	*axonopodis*	LMG7390	before
Q0420_RO_D07_2_F10_A	GREEN	No	19	*Xanthomonas*	*axonopodis*	LMG7390	before
Q0420_RO_D08_2_F10_B	GREEN	No	19	*Xanthomonas*	*axonopodis*	LMG7390	after
Q0420_RO_D09_2_F11_A	GREEN	Yes	19	*Xanthomonas*	*axonopodis*	LMG7390	after
Q0421_LB_G08_2_A03_B	GREEN	Yes	71	*Xanthomonas*	*campestris*	LMG7460	before
Q0421_LB_G07_2_A03_A	GREEN	No	71	*Xanthomonas*	*campestris*	LMG7460	before
Q0421_RB_A02_2_D12_B	GREEN	No	73	*Xanthomonas*	*campestris*	LMG7460	after
Q0421_LB_H08_1_A03_B	GREEN	No	73	*Xanthomonas*	*campestris*	LMG7460	after
Q0420_RO_A03_1_E08_A	GREEN	No	31	*Xanthomonas*	*arboricola*	LMG747	before
Q0420_RO_A02_1_E07_B	GREEN	No	31	*Xanthomonas*	*arboricola*	LMG747	before
Q0420_RO_A05_1_E09_A	GREEN	No	31	*Xanthomonas*	*arboricola*	LMG747	after
Q0420_RO_A04_1_E08_B	GREEN	Yes	31	*Xanthomonas*	*arboricola*	LMG747	after
Q0420_RO_F06_2_G09_B	GREEN	No	50	*Xanthomonas*	*axonopodis*	LMG7486	before
Q0420_RO_F05_2_G09_A	GREEN	No	50	*Xanthomonas*	*axonopodis*	LMG7486	before
Q0420_RO_F08_2_G10_B	GREEN	No	50	*Xanthomonas*	*axonopodis*	LMG7486	after
Q0420_RO_F09_2_G11_A	GREEN	Yes	50	*Xanthomonas*	*axonopodis*	LMG7486	after
Q0420_LO_G11_1_H06_A	GREEN	No	32	*Xanthomonas*	*arboricola*	LMG751	before
Q0420_LO_G10_1_H05_B	GREEN	Yes	30	*Xanthomonas*	*arboricola*	LMG751	before
Q0420_LO_G12_1_H06_B	GREEN	No	30	*Xanthomonas*	*arboricola*	LMG751	after
Q0420_LO_H01_2_H01_A	GREEN	Yes	33	*Xanthomonas*	*arboricola*	LMG751	after
Q0420_RO_G10_1_H11_B	GREEN	No	17	*Xanthomonas*	*axonopodis*	LMG758	before
Q0420_RO_G11_1_H12_A	GREEN	No	17	*Xanthomonas*	*axonopodis*	LMG758	before
Q0420_RO_G12_1_H12_B	GREEN	Yes	94	*Xanthomonas*	*axonopodis*	LMG758	after
Q0420_RO_H01_2_H07_A	GREEN	No	94	*Xanthomonas*	*axonopodis*	LMG758	after
Q0421_LB_A02_2_D06_B	GREEN	No	91	*Xanthomonas*	*axonopodis*	LMG766	before
Q0421_LB_A01_2_D06_A	GREEN	No	91	*Xanthomonas*	*axonopodis*	LMG766	before
Q0421_LB_A09_2_D02_A	GREEN	Yes	91	*Xanthomonas*	*axonopodis*	LMG766	after
Q0421_LB_A08_2_D03_B	GREEN	No	91	*Xanthomonas*	*axonopodis*	LMG766	after
Q0413_LO_H07_2_H04_A	GREEN	No	35	*Lactococcus*	*lactis*	LMG7930	before
Q0413_LO_H06_2_H03_B	GREEN	No	35	*Lactococcus*	*lactis*	LMG7930	before
Q0413_RO_A04_1_E08_B	GREEN	No	35	*Lactococcus*	*lactis*	LMG7930	after
Q0413_RO_A05_1_E09_A	GREEN	Yes	35	*Lactococcus*	*lactis*	LMG7930	after
Q0408_RB_E04_2_B11_B	GREEN	No	59	*Enterococcus*	*faecalis*	LMG7937T	before
Q0408_RB_E05_2_B10_A	GREEN	No	59	*Enterococcus*	*faecalis*	LMG7937T	before
Q0408_RB_F01_1_B12_A	GREEN	No	59	*Enterococcus*	*faecalis*	LMG7937T	after
Q0408_RB_E12_2_B07_B	GREEN	No	59	*Enterococcus*	*faecalis*	LMG7937T	after
Q0414_LB_G10_2_A02_B	GREEN	No	76	*Leuconostoc*	*lactis*	LMG7940	before
Q0414_LB_G11_2_A01_A	GREEN	No	76	*Leuconostoc*	*lactis*	LMG7940	before
Q0414_LB_H01_1_A06_A	GREEN	Yes	76	*Leuconostoc*	*lactis*	LMG7940	after
Q0414_LB_G12_2_A01_B	GREEN	No	76	*Leuconostoc*	*lactis*	LMG7940	after
Q0411_RO_G11_1_H12_A	GREEN	Yes	92	*Lactobacillus*	*paracasei*	LMG7955	before
Q0411_RO_G10_1_H11_B	GREEN	No	92	*Lactobacillus*	*paracasei*	LMG7955	before
Q0411_RO_H12_2_H12_B	GREEN	No	92	*Lactobacillus*	*paracasei*	LMG7955	after
Q0411_RO_H08_2_H10_B	GREEN	No	92	*Lactobacillus*	*paracasei*	LMG7955	after
Q0408_RB_G03_2_A11_A	GREEN	No	9	*Enterococcus*	*faecium*	LMG8147	before
Q0408_RB_G02_2_A12_B	GREEN	No	9	*Enterococcus*	*faecium*	LMG8147	before
Q0408_RB_G05_2_A10_A	GREEN	No	9	*Enterococcus*	*faecium*	LMG8147	after
Q0408_RB_G04_2_A11_B	GREEN	Yes	9	*Enterococcus*	*faecium*	LMG8147	after
Q0414_RB_H11_1_A07_A	GREEN	No	85	*Leuconostoc*	*suionicum*	LMG8159T	before
Q0414_RB_H12_1_A07_B	GREEN	No	85	*Leuconostoc*	*suionicum*	LMG8159T	before
Q0414_LO_A05_1_E03_A	GREEN	Yes	85	*Leuconostoc*	*suionicum*	LMG8159T	after
Q0414_LO_A04_1_E02_B	GREEN	No	85	*Leuconostoc*	*suionicum*	LMG8159T	after
Q0413_LO_C02_1_F01_B	GREEN	No	11	*Lactococcus*	*garvieae*	LMG8162	before
Q0413_LO_C03_1_F02_A	GREEN	No	11	*Lactococcus*	*garvieae*	LMG8162	before
Q0413_LO_D10_2_F05_B	GREEN	Yes	11	*Lactococcus*	*garvieae*	LMG8162	after
Q0413_LO_E04_1_G02_B	GREEN	Yes	12	*Lactococcus*	*garvieae*	LMG8162	after
Q0421_RB_H07_1_A09_A	GREEN	Yes	23	*Xanthomonas*	*pisi*	LMG847T	before
Q0421_RB_H04_1_A11_B	GREEN	Yes	24	*Xanthomonas*	*pisi*	LMG847T	before
Q0421_LO_A02_1_E01_B	GREEN	No	23	*Xanthomonas*	*pisi*	LMG847T	after
Q0421_LO_A01_1_E01_A	GREEN	No	23	*Xanthomonas*	*pisi*	LMG847T	after
Q0421_RB_A05_2_D10_A	GREEN	No	1	*Xanthomonas*	*citri*	LMG8655	before
Q0421_RB_A04_2_D11_B	GREEN	Yes	1	*Xanthomonas*	*citri*	LMG8655	before
Q0421_RB_B04_1_D11_B	GREEN	No	18	*Xanthomonas*	*citri*	LMG8655	after

Q0421_RB_B01_1_D12_A	GREEN	Yes	18	*Xanthomonas*	*citri*	LMG8655	after
Q0421_LO_B12_2_E06_B	GREEN	No	64	*Xanthomonas*	*vasicola*	LMG8718	before
Q0421_LO_C01_1_F01_A	GREEN	No	64	*Xanthomonas*	*vasicola*	LMG8718	before
Q0421_LO_C10_1_F05_B	GREEN	No	64	*Xanthomonas*	*vasicola*	LMG8718	after
Q0421_LO_C09_1_F05_A	GREEN	Yes	64	*Xanthomonas*	*vasicola*	LMG8718	after
Q0421_LB_B02_1_D06_B	GREEN	No	68	*Xanthomonas*	*axonopodis*	LMG9049	before
Q0421_LB_B04_1_D05_B	GREEN	Yes	68	*Xanthomonas*	*axonopodis*	LMG9049	before
Q0421_LB_C07_2_C03_A	GREEN	No	26	*Xanthomonas*	*axonopodis*	LMG9049	after
Q0421_LB_C05_2_C04_A	GREEN	Yes	26	*Xanthomonas*	*axonopodis*	LMG9049	after
Q0413_LO_D04_2_F02_B	GREEN	No	86	*Lactococcus*	*garvieae*	LMG9443	before
Q0413_LO_D06_2_F03_B	GREEN	No	86	*Lactococcus*	*garvieae*	LMG9443	before
Q0413_LO_D09_2_F05_A	GREEN	Yes	78	*Lactococcus*	*garvieae*	LMG9443	after
Q0413_LO_D08_2_F04_B	GREEN	Yes	86	*Lactococcus*	*garvieae*	LMG9443	after
Q0413_LO_G02_1_H01_B	GREEN	No	36	*Lactococcus*	*lactis*	LMG9462	before
Q0413_LO_G03_1_H02_A	GREEN	No	36	*Lactococcus*	*lactis*	LMG9462	before
Q0413_LO_G04_1_H02_B	GREEN	No	36	*Lactococcus*	*lactis*	LMG9462	after
Q0413_LO_G05_1_H03_A	GREEN	Yes	36	*Lactococcus*	*lactis*	LMG9462	after
Q0419_LO_G02_1_H01_B	GREEN	No	10	*Streptococcus*	*uberis*	LMG9465T	before
Q0419_LO_G03_1_H02_A	GREEN	Yes	10	*Streptococcus*	*uberis*	LMG9465T	before
Q0419_LO_G04_1_H02_B	GREEN	No	10	*Streptococcus*	*uberis*	LMG9465T	after
Q0419_LO_G05_1_H03_A	GREEN	No	10	*Streptococcus*	*uberis*	LMG9465T	after
Q0413_LO_D05_2_F03_A	GREEN	No	75	*Lactococcus*	*garvieae*	LMG9472	before
Q0413_LO_D07_2_F04_A	GREEN	No	75	*Lactococcus*	*garvieae*	LMG9472	before
Q0413_LO_D11_2_F06_A	GREEN	No	75	*Lactococcus*	*garvieae*	LMG9472	after
Q0413_LO_D12_2_F06_B	GREEN	Yes	75	*Lactococcus*	*garvieae*	LMG9472	after
Q0410_LB_A03_2_D05_A	GREEN	No	0	*Lactobacillus*	*amylovorus*	LMG9496T	before
Q0410_LB_A02_2_D06_B	GREEN	No	0	*Lactobacillus*	*amylovorus*	LMG9496T	before
Q0410_LB_A05_2_D04_A	GREEN	No	0	*Lactobacillus*	*amylovorus*	LMG9496T	after
Q0410_LB_A04_2_D05_B	GREEN	Yes	0	*Lactobacillus*	*amylovorus*	LMG9496T	after
Q0421_LB_D07_1_C03_A	GREEN	No	63	*Xanthomonas*	*axonopodis*	LMG955	before
Q0421_LB_D05_1_C04_A	GREEN	No	63	*Xanthomonas*	*axonopodis*	LMG955	before
Q0421_LB_D09_1_C02_A	GREEN	No	63	*Xanthomonas*	*axonopodis*	LMG955	after
Q0421_LB_E01_2_B06_A	GREEN	Yes	63	*Xanthomonas*	*axonopodis*	LMG955	after
Q0420_LO_B07_2_E04_A	GREEN	No	29	*Weissella*	*paramesenteroides*	LMG9852T	before
Q0420_LO_B06_2_E03_B	GREEN	No	29	*Weissella*	*paramesenteroides*	LMG9852T	before
Q0420_LO_B09_2_E05_A	GREEN	No	29	*Weissella*	*paramesenteroides*	LMG9852T	after
Q0420_LO_B08_2_E04_B	GREEN	Yes	29	*Weissella*	*paramesenteroides*	LMG9852T	after
