# Supplementary material for: Introducing SPeDE: High-Throughput Dereplication and Accurate Determination of Microbial Diversity from Matrix-Assisted Laser Desorption–Ionization Time of Flight Mass Spectrometry Data
Source: mSystems. 2019 Sep 10;4(5):e00437-19. doi: 10.1128/mSystems.00437-19 (PMC6739102; doi:10.1128/mSystems.00437-19)
Supplement: TABLE S5 [file mSystems.00437-19-st005.pdf]

Table S5: List of strains included in the benchmark, lyophilization and *Lactobacillus brevis* dataset used in this study

	Benchmark set collection of strains		
Strain	Identification	Phylum	DNA extraction method
LMG 1617 T	*Acetobacter lovaniensis*	Proteobacteria	Wilson *et al.,* 1987
LMG 26852 T	*Achromobacter aegrifaciens*	Proteobacteria	Pitcher *et al.,* 1989
LMG 1668 T	*Acidomonas methanolica*	Proteobacteria	Wilson *et al.,* 1987
LMG 5286 T	*Acidovorax cattleyae*	Proteobacteria	Wilson *et al.,* 1987
LMG 1041 T	*Acinetobacter baumannii*	Proteobacteria	Wilson *et al.,* 1987
LMG 22214 T	*Aeromonas molluscorum*	Proteobacteria	Wilson *et al.,* 1987
LMG 1229 T	*Alcaligenes faecalis*	Proteobacteria	Wilson *et al.,* 1987
LMG 26473 T	*Alishewanella tabrizica*	Proteobacteria	Wilson *et al.,* 1987
LMG 18397 T	*Anoxybacillus flavithermus*	Firmicutes	Gevers *et al.,* 2001
LMG 24559 T	*Arcobacter mytili*	Epsilonbacteraeota	Wilson *et al.,* 1987
LMG 25535	*Arcobacter trophiarum*	Epsilonbacteraeota	Wilson *et al.,* 1987
LMG 23083 T	*Asaia krungthepensis*	Proteobacteria	Wilson *et al.,* 1987
LMG 13127 T	*Azospirillum brasilense*	Proteobacteria	Wilson *et al.,* 1987
LMG 7135 T	*Bacillus subtilis subtilis*	Firmicutes	Pitcher *et al.,* 1989
LMG 6451 T	*Bacteroides ureolyticus*	Bacteroidetes	Pitcher *et al.,* 1989
LMG 24411 T	*Bhargavaea cecembensis*	Firmicutes	Gevers *et al.,* 2001
LMG 11039 T	*Bifidobacterium angulatum*	Actinobacteria	Gevers *et al.,* 2001
LMG 23059 T	*Blastobacter aggregatus*	Proteobacteria	Wilson *et al.,* 1987
LMG 1232 T	*Bordetella bronchiseptica*	Proteobacteria	Wilson *et al.,* 1987
LMG 2698 T	*Brenneria salicis*	Proteobacteria	Wilson *et al.,* 1987
LMG 7123 T	*Brevibacillus brevis*	Firmicutes	Gevers *et al.,* 2001
R-68806	*Burkholderia cenocepacia IIIA*	Proteobacteria	Maxwell® 16 Tissue Purification kit
R-71051	*Burkholderia cenocepacia IIIA*	Proteobacteria	Maxwell® 16 Tissue Purification kit
R-71085	*Burkholderia cenocepacia IIIA*	Proteobacteria	Maxwell® 16 Tissue Purification kit
R-68675	*Burkholderia cenocepacia IIIA*	Proteobacteria	Maxwell® 16 Tissue Purification kit
R-67259	*Burkholderia cenocepacia IIIB*	Proteobacteria	Maxwell® 16 Tissue Purification kit
R-67581	*Burkholderia cenocepacia IIIB*	Proteobacteria	Maxwell® 16 Tissue Purification kit
R-68591	*Burkholderia cenocepacia IIIB*	Proteobacteria	Maxwell® 16 Tissue Purification kit
R-68599	*Burkholderia cenocepacia IIIB*	Proteobacteria	Maxwell® 16 Tissue Purification kit
R-69596	*Burkholderia cepacia*	Proteobacteria	Maxwell® 16 Tissue Purification kit
LMG 22485 T	*Burkholderia lata*	Proteobacteria	Wilson *et al.,* 1987
R-71006	*Burkholderia multivorans*	Proteobacteria	Maxwell® 16 Tissue Purification kit
R-71089	*Burkholderia multivorans*	Proteobacteria	Maxwell® 16 Tissue Purification kit
R-67121	*Burkholderia multivorans*	Proteobacteria	Maxwell® 16 Tissue Purification kit
R-67196	*Burkholderia multivorans*	Proteobacteria	Maxwell® 16 Tissue Purification kit
R-67258	*Burkholderia multivorans*	Proteobacteria	Maxwell® 16 Tissue Purification kit
R-67536	*Burkholderia multivorans*	Proteobacteria	Maxwell® 16 Tissue Purification kit
R-68768	*Burkholderia multivorans*	Proteobacteria	Maxwell® 16 Tissue Purification kit
R-50394	*Burkholderia stabilis*	Proteobacteria	Maxwell® 16 Tissue Purification kit
R-67113	*Burkholderia stabilis*	Proteobacteria	Maxwell® 16 Tissue Purification kit
R-69593	*Burkholderia vietnamiensis*	Proteobacteria	Maxwell® 16 Tissue Purification kit
R-67189	*Burkholderia vietnamiensis*	Proteobacteria	Maxwell® 16 Tissue Purification kit
LMG 24812 T	*Candidimonas nitroreducens*	Proteobacteria	Pitcher *et al.,* 1989
LMG 23655 T	*Carnobacterium inhibens*	Firmicutes	Gevers *et al.,* 2001
LMG 24015 T	*Chitinophaga terrae*	Bacteroidetes	Wilson *et al.,* 1987

LMG 18212 T	*Chryseobacterium joostei*	Bacteroidetes	Wilson *et al.,* 1987
LMG 3252 T	*Citrobacter youngae*	Proteobacteria	Wilson *et al.,* 1987
LMG 23965 T	*Collimonas pratensis*	Proteobacteria	Wilson *et al.,* 1987
LMG 19264 T	*Corynebacterium casei*	Actinobacteria	Gevers *et al.,* 2001
LMG 8787 T	*Curtobacterium luteum*	Actinobacteria	Gevers *et al.,* 2001
LMG 4328 T	*Curvibacter delicatus*	Proteobacteria	Wilson *et al.,* 1987
LMG 4051 T	*Deinococcus radiodurans*	Deinococcus-Thermus	Pitcher *et al.,* 1989
LMG 1226 T	*Delftia acidovorans*	Proteobacteria	Wilson *et al.,* 1987
LMG 7529 T	*Desulfovibrio desulfuricans desulfuricans*	Proteobacteria	Pitcher *et al.,* 1989
LMG 2804 T	*Dickeya chrysanthemi*	Proteobacteria	Wilson *et al.,* 1987
LMG 25420 T	*Echinimonas agarilytica*	Proteobacteria	Wilson *et al.,* 1987
LMG 26586 T	*Eilatimonas milleporae*	Proteobacteria	Wilson *et al.,* 1987
LMG 26064 T	*Enterobacter asburiae*	Proteobacteria	Wilson *et al.,* 1987
LMG 26304 T	*Enterococcus ureasiticus*	Firmicutes	Gevers *et al.,* 2001
LMG 24401 T	*Epilithonimonas lactis*	Bacteroidetes	Wilson *et al.,* 1987
LMG 21371 T	*Erwinia carotovora*	Proteobacteria	Wilson *et al.,* 1987
LMG 22735	*Finegoldia magna*	Firmicutes	Gevers *et al.,* 2001
LMG 4008 T	*Flavobacterium aquatile*	Bacteroidetes	Wilson *et al.,* 1987
LMG 28216 T	*Formosa algae*	Bacteroidetes	Wilson *et al.,* 1987
LMG 23037 T	*Geobacillus toebii*	Firmicutes	Gevers *et al.,* 2001
LMG 25547 T	*Glaciimonas immobilis*	Proteobacteria	Wilson *et al.,* 1987
LMG 21311 T	*Gluconacetobacter azotocaptans*	Proteobacteria	Wilson *et al.,* 1987
LMG 1408 T	*Gluconobacter oxydans*	Proteobacteria	Wilson *et al.,* 1987
LMG 22585 T	*Gramella echinicola*	Bacteroidetes	Wilson *et al.,* 1987
LMG 24392 T	*Granulibacter bethesdensis*	Proteobacteria	Wilson *et al.,* 1987
LMG 15863	*Haemophilus influenzae*	Proteobacteria	Wilson *et al.,* 1987
LMG 26187 T	*Halomonas ventosae*	Proteobacteria	Wilson *et al.,* 1987
LMG 18294 T	*Helicobacter fennelliae*	Epsilonbacteraeota	Maxwell® 16 Tissue Purification kit
LMG 26149 T	*Herbaspirillum soli*	Proteobacteria	Wilson *et al.,* 1987
LMG 21292	*Ketogulonicigenium robustum*	Proteobacteria	Wilson *et al.,* 1987
LMG 2095 T	*Klebsiella pneumoniae pneumoniae*	Proteobacteria	Wilson *et al.,* 1987
LMG 1527 T	*Komagataeibacter hansenii*	Proteobacteria	Wilson *et al.,* 1987
LMG 27019	*Kozakia baliensis*	Proteobacteria	Wilson *et al.,* 1987
LMG 6901 T	*Lactobacillus delbrueckii bulgaricus*	Firmicutes	Gevers *et al.,* 2001
LMG 19667 T	*Lactobacillus diolivorans*	Firmicutes	Gevers *et al.,* 2001
LMG 18398	*Lactobacillus paraplantarum*	Firmicutes	Gevers *et al.,* 2001
LMG 16673 T	*Lactobacillus paraplantarum*	Firmicutes	Pitcher *et al.,* 1989
LMG 17677	*Lactobacillus pentosus*	Firmicutes	Gevers *et al.,* 2001
LMG 18401	*Lactobacillus pentosus*	Firmicutes	Gevers *et al.,* 2001
LMG 9210	*Lactobacillus pentosus*	Firmicutes	Gevers *et al.,* 2001
LMG 18021	*Lactobacillus plantarum*	Firmicutes	Gevers *et al.,* 2001
LMG 18404	*Lactobacillus plantarum*	Firmicutes	Gevers *et al.,* 2001
LMG 24832	*Lactobacillus plantarum*	Firmicutes	Gevers *et al.,* 2001
LMG 26367	*Lactobacillus plantarum*	Firmicutes	Gevers *et al.,* 2001
LMG 11405	*Lactobacillus plantarum*	Firmicutes	Pitcher *et al.,* 1989
LMG 9205 T	*Lactobacillus plantarum argentoratensis*	Firmicutes	Pitcher *et al.,* 1989
LMG 6907 T	*Lactobacillus plantarum plantarum*	Firmicutes	Gevers *et al.,* 2001
LMG 23383 T	*Lactococcus piscium*	Firmicutes	Gevers *et al.,* 2001
LMG 1345 T	*Leeuwenhoekiella marinoflava*	Bacteroidetes	Wilson *et al.,* 1987
LMG 6909 T	*Leuconostoc mesenteroides cremoris*	Firmicutes	Gevers *et al.,* 2001

LMG 3897 T	*Listonella pelagia*	Proteobacteria	Pitcher *et al.*, 1989
LMG 2724 T	*Lonsdalea quercina quercina*	Proteobacteria	Wilson *et al.*, 1987
LMG 8760 T	*Lysobacter antibioticus*	Proteobacteria	Wilson *et al.*, 1987
LMG 19863 T	*Maricaulis parjimensis*	Proteobacteria	Pitcher *et al.*, 1989
LMG 1346 T	*Marinilabilia salmonicolor*	Bacteroidetes	Wilson *et al.*, 1987
LMG 23835 T	*Marinobacter algicola*	Proteobacteria	Wilson *et al.*, 1987
LMG 25435 T	*Marinobacterium coralli*	Proteobacteria	Wilson *et al.*, 1987
LMG 2864 T	*Marinomonas communis*	Proteobacteria	Wilson *et al.*, 1987
LMG 22193 T	*Martelella mediterranea*	Proteobacteria	Wilson *et al.*, 1987
LMG 21530 T	*Massilia timonae*	Proteobacteria	Wilson *et al.*, 1987
LMG 25664 T	*Megasphaera cerevisiae*	Firmicutes	Gevers *et al.*, 2001
LMG 22697 T	*Mesorhizobium thiogangeticum*	Proteobacteria	Wilson *et al.*, 1987
LMG 4050 T	*Micrococcus luteus*	Actinobacteria	Gevers *et al.*, 2001
LMG 18919 T	*Microvirgula aerodenitrificans*	Proteobacteria	Pitcher *et al.*, 1989
LMG 11194 T	*Moraxella canis*	Proteobacteria	Wilson *et al.*, 1987
LMG 7874 T	*Morganella morganii morganii*	Proteobacteria	Wilson *et al.*, 1987
LMG 29427 T	*Nakamurella silvestris*	Actinobacteria	Gevers *et al.*, 2001
LMG 27021 T	*Neokomagataea thailandica*	Proteobacteria	Wilson *et al.*, 1987
LMG 27282 T	*Noviherbaspirillum psychrotolerans*	Proteobacteria	Wilson *et al.*, 1987
LMG 24024 T	*Oceanobacillus oncorhynchi oncorhynchi*	Firmicutes	Pitcher *et al.*, 1989
LMG 6519 T	*Oligella ureolytica*	Proteobacteria	Pitcher *et al.*, 1989
LMG 3516	*Paenalcaligenes hominis*	Proteobacteria	Maxwell® 16 Tissue Purification kit
LMG 16409	*Pandoraea apista*	Proteobacteria	Maxwell® 16 Tissue Purification kit
LMG 1286 T	*Pantoea agglomerans*	Proteobacteria	Wilson *et al.*, 1987
LMG 26195 T	*Paracoccus sp.*	Proteobacteria	Wilson *et al.*, 1987
LMG 24012 T	*Parapusillimonas granuli*	Proteobacteria	Pitcher *et al.*, 1989
LMG 27212	*Pectinatus sp.*	Firmicutes	Wilson *et al.*, 1987
LMG 2404 T	*Pectobacterium carotovorum carotovorum*	Proteobacteria	Wilson *et al.*, 1987
LMG 22475 T	*Phaeobacter inhibens*	Proteobacteria	Wilson *et al.*, 1987
LMG 2186	*Phaseolibacter flectens*	Proteobacteria	Wilson *et al.*, 1987
LMG 4233 T	*Photobacterium phosphoreum*	Proteobacteria	Wilson *et al.*, 1987
LMG 21665 T	*Pigmentiphaga kullae*	Proteobacteria	Pitcher *et al.*, 1989
LMG 25212 T	*Polynucleobacter cosmopolitanus*	Proteobacteria	Pitcher *et al.*, 1989
LMG 6452 T	*Prevotella bivia*	Bacteroidetes	Gevers *et al.*, 2001
LMG 1242 T	*Pseudomonas aeruginosa*	Proteobacteria	Wilson *et al.*, 1987
LMG 24163 T	*Pseudoxanthomonas dokdonensis*	Proteobacteria	Pitcher *et al.*, 1989
LMG 21276 T	*Psychrobacter luti*	Proteobacteria	Wilson *et al.*, 1987
LMG 6866 T	*Ralstonia mannitolilytica*	Proteobacteria	Wilson *et al.*, 1987
LMG 23818 T	*Rheinheimera chironomi*	Proteobacteria	Wilson *et al.*, 1987
LMG 12537	*Rhizorhapis suberifaciens*	Proteobacteria	Wilson *et al.*, 1987
LMG 23003 T	*Rhodanobacter fulvus*	Proteobacteria	Wilson *et al.*, 1987
LMG 4305 T	*Rhodobacter blasticus*	Proteobacteria	Wilson *et al.*, 1987
LMG 28633 T	*Rhodococcus degradans*	Actinobacteria	Gevers *et al.*, 2001
LMG 26121 T	*Rosenbergiella nectarea*	Proteobacteria	Wilson *et al.*, 1987
LMG 24552 T	*Roseomonas gilardii*	Proteobacteria	Wilson *et al.*, 1987
LMG 27719 T	*Roseomonas gilardii*	Proteobacteria	Wilson *et al.*, 1987
LMG 24367 T	*Ruegeria scottomollicae*	Proteobacteria	Pitcher *et al.*, 1989
LMG 23170 T	*Saccharibacter floricola*	Proteobacteria	Wilson *et al.*, 1987
LMG 28391 T	*Salininema proteolyticum*	Actinobacteria	Gevers *et al.*, 2001

LMG 7233 T	*Salmonella enterica enterica*	Proteobacteria	Wilson *et al.,* 1987
LMG 7881 T	*Serratia ficaria*	Proteobacteria	Wilson *et al.,* 1987
LMG 5019 T	*Serratia rubidaea*	Proteobacteria	Wilson *et al.,* 1987
LMG 24424 T	*Shewanella vesiculosa*	Proteobacteria	Wilson *et al.,* 1987
LMG 23381 T	*Simplicispira metamorpha*	Proteobacteria	Pitcher *et al.,* 1989
LMG 12553	*Sphingobium xanthum*	Proteobacteria	Wilson *et al.,* 1987
LMG 19484 T	*Sphingomonas melonis*	Proteobacteria	Wilson *et al.,* 1987
LMG 6928 T	*Sporosarcina globispora*	Firmicutes	Pitcher *et al.,* 1989
LMG 13349 T	*Staphylococcus haemolyticus*	Firmicutes	Gevers *et al.,* 2001
LMG 24537 T	*Stenotrophomonas rhizophila*	Proteobacteria	Pitcher *et al.,* 1989
LMG 6896 T	*Streptococcus thermophilus*	Firmicutes	Gevers *et al.,* 2001
LMG 23078	*Streptomyces albus albus*	Actinobacteria	Gevers *et al.,* 2001
LMG 25773 T	*Tabrizicola aquatica*	Proteobacteria	Wilson *et al.,* 1987
LMG 26467 T	*Tardiphaga robiniae*	Proteobacteria	Wilson *et al.,* 1987
LMG 22049 T	*Tatumella citrea*	Proteobacteria	Wilson *et al.,* 1987
LMG 26041 T	*Tetragenococcus osmophilus*	Firmicutes	Gevers *et al.,* 2001
LMG 129 T	*Thalassobius gelatinovorus*	Proteobacteria	Wilson *et al.,* 1987
LMG 2811 T	*Thermoanaerobacterium thermosaccharolyticum*	Firmicutes	Gevers *et al.,* 2001
LMG 24833 T	*Vagococcus penaei*	Firmicutes	Gevers *et al.,* 2001
LMG 4044 T	*Vibrio harveyi*	Proteobacteria	Wilson *et al.,* 1987
LMG 21817 T	*Woodsholea maritima*	Proteobacteria	Maxwell® 16 Tissue Purification kit
LMG 5743 T	*Xanthomonas populi*	Proteobacteria	Wilson *et al.,* 1987
LMG 7899 T	*Yersinia enterocolitica enterocolitica*	Proteobacteria	Wilson *et al.,* 1987
LMG 460	*Zymomonas mobilis*	*Proteobacteria*	Wilson *et al.,* 1987

Strain	Lyophilization set collection of strains<br>Identification	Phylum
LMG 19456	*Enterococcus faecalis*	Firmicutes
LMG 7937 T	*Enterococcus faecalis*	Firmicutes
LMG 8147	*Enterococcus faecium*	Firmicutes
LMG 10274	*Enterococcus hirae*	Firmicutes
LMG 11427 T	*Enterococcus saccharolyticus*	Firmicutes
LMG 2093	*Escherichia coli*	Proteobacteria
LMG 30302 T	*Klebsiella kielensis*	Proteobacteria
LMG 30316 T	*Klebsiella nitrificae*	Proteobacteria
LMG 3055 T	*Klebsiella oxytoca*	Proteobacteria
LMG 2095 T	*Klebsiella pneumoniae*	Proteobacteria
LMG 3116	*Klebsiella pneumoniae*	Proteobacteria
LMG 21683	*Lactobacillus alimentarius*	Firmicutes
LMG 11400 T	*Lactobacillus amylotrophicus*	Firmicutes
LMG 9496 T	*Lactobacillus amylovorus*	Firmicutes
LMG 25505 T	*Lactobacillus apodemi*	Firmicutes
LMG 23516	*Lactobacillus casei*	Firmicutes
LMG 11440	*Lactobacillus crispatus*	Firmicutes
LMG 18916	*Lactobacillus iners*	Firmicutes
LMG 30568	*Lactobacillus johnsonii*	Firmicutes
LMG 30566	*Lactobacillus kunkeei*	Firmicutes
LMG 11961	*Lactobacillus paracasei paracasei*	Firmicutes

LMG 7955	*Lactobacillus paracasei paracasei*	Firmicutes
LMG 30567	*Lactobacillus plantarum*	Firmicutes
LMG 10770	*Lactobacillus rhamnosus*	Firmicutes
LMG 18028	*Lactobacillus rhamnosus*	Firmicutes
LMG 23667	*Lactobacillus rhamnosus*	Firmicutes
LMG 14806	*Lactobacillus salivarius*	Firmicutes
LMG 8162	*Lactococcus garvieae*	Firmicutes
LMG 9443	*Lactococcus garvieae*	Firmicutes
LMG 9472	*Lactococcus garvieae*	Firmicutes
LMG 9462	*Lactococcus lactis hordniae*	Firmicutes
LMG 14418	*Lactococcus lactis lactis*	Firmicutes
LMG 6890 T	*Lactococcus lactis lactis*	Firmicutes
LMG 7930	*Lactococcus lactis lactis*	Firmicutes
LMG 18868	*Leuconostoc carnosum*	Firmicutes
LMG 19597	*Leuconostoc gelidum*	Firmicutes
LMG 7940	*Leuconostoc lactis*	Firmicutes
LMG 25878	*Leuconostoc mesenteroides*	Firmicutes
LMG 18972	*Leuconostoc mesenteroides cremoris*	Firmicutes
LMG 11321	*Leuconostoc mesenteroides dextranicum*	Firmicutes
LMG 18967	*Leuconostoc mesenteroides mesenteroides*	Firmicutes
LMG 24510 T	*Leuconostoc palmae*	Firmicutes
LMG 8159 T	*Leuconostoc suionicum*	Firmicutes
LMG 15081	*Streptococcus agalactiae*	Firmicutes
LMG 14517	*Streptococcus gordonii*	Firmicutes
LMG 14542	*Streptococcus pneumoniae*	Firmicutes
LMG 21598	*Streptococcus pneumoniae*	Firmicutes
LMG 13103	*Streptococcus salivarius*	Firmicutes
LMG 14652	*Streptococcus salivarius*	Firmicutes
LMG 14637	*Streptococcus sanguinis*	Firmicutes
LMG 14638	*Streptococcus sanguinis*	Firmicutes
LMG 9465 T	*Streptococcus uberis*	Firmicutes
LMG 16883	*Weissella confusa*	Firmicutes
LMG 18476	*Weissella confusa*	Firmicutes
LMG 18500	*Weissella confusa*	Firmicutes
LMG 18503	*Weissella confusa*	Firmicutes
LMG 18815	*Weissella confusa*	Firmicutes
LMG 14471 T	*Weissella kandleri*	Firmicutes
LMG 9852 T	*Weissella paramesenteroides*	Firmicutes
LMG 19144	*Xanthomonas arboricola fragariae*	Proteobacteria
LMG 747	*Xanthomonas arboricola juglandis*	Proteobacteria
LMG 751	*Xanthomonas arboricola juglandis*	Proteobacteria
LMG 556	*Xanthomonas axonopodis biophyti*	Proteobacteria
LMG 7390	*Xanthomonas axonopodis cassiae*	Proteobacteria
LMG 7486	*Xanthomonas axonopodis erythrinae*	Proteobacteria
LMG 758	*Xanthomonas axonopodis maculifoliigardeniae*	Proteobacteria
LMG 766	*Xanthomonas axonopodis manihotis*	Proteobacteria
LMG 9049	*Xanthomonas axonopodis martyniicola*	Proteobacteria
LMG 955	*Xanthomonas axonopodis tamarindi*	Proteobacteria

LMG 559	*Xanthomonas campestris campestris*	Proteobacteria
LMG 568	*Xanthomonas campestris campestris*	Proteobacteria
LMG 7460	*Xanthomonas campestris campestris*	Proteobacteria
LMG 8655	*Xanthomonas citri aurantifolii*	Proteobacteria
LMG 30292	*Xanthomonas fragariae*	Proteobacteria
LMG 630	*Xanthomonas oryzae oryzae*	Proteobacteria
LMG 654	*Xanthomonas oryzae oryzicola*	Proteobacteria
LMG 847 T	*Xanthomonas pisi*	Proteobacteria
LMG 471 T	*Xanthomonas sacchari*	Proteobacteria
LMG 8718	*Xanthomonas vasicola vasculorum*	Proteobacteria

Lactobacillus brevis set collection of strains		
Strain	Identification	isolation source
LMG 6906 T	*Lactobacillus brevis*	human, faeces
LMG 7761	*Lactobacillus brevis*	green fermenting olives
LMG 11401	*Lactobacillus brevis*	beer
LMG 11434	*Lactobacillus brevis*	white Stilton cheese
LMG 11437	*Lactobacillus brevis*	silage
LMG 11438	*Lactobacillus brevis*	tomato pulp
LMG 11495	*Lactobacillus brevis*	wine
LMG 11969	*Lactobacillus brevis*	marinated fish
LMG 11988	*Lactobacillus brevis*	dried yeast
LMG 11993	*Lactobacillus brevis*	English hard cheese
LMG 11998	*Lactobacillus brevis*	starter from dairy
LMG 12023	*Lactobacillus brevis*	human, intestine
LMG 16322	*Lactobacillus brevis*	spoiled beer
LMG 18022	*Lactobacillus brevis*	Zabady (yoghurt)
R-33878	*Lactobacillus brevis*	sourdough
R-34116	*Lactobacillus brevis*	cocoa
R-42469	*Lactobacillus brevis*	Nem chua (fermented meat)
R-42874	*Lactobacillus brevis*	pasteurized milk (Beverse Kaasmakerij)
R-46486	*Lactobacillus brevis*	Dua chua (fermented plant)
R-47272	*Lactobacillus brevis*	cheese (Touloumotyri)
R-47313	*Lactobacillus brevis*	cheese (Anthotyro)
R-47325	*Lactobacillus brevis*	cheese (Kefalotyri)
R-47349	*Lactobacillus brevis*	cheese (Kefalotyri)
R-49154	*Lactobacillus brevis*	beer
R-53271	*Lactobacillus brevis*	beer
