# Supplementary material for: Introducing SPeDE: High-Throughput Dereplication and Accurate Determination of Microbial Diversity from Matrix-Assisted Laser Desorption–Ionization Time of Flight Mass Spectrometry Data
Source: mSystems. 2019 Sep 10;4(5):e00437-19. doi: 10.1128/mSystems.00437-19 (PMC6739102; doi:10.1128/mSystems.00437-19)
Supplement: TABLE S3 [file mSystems.00437-19-st003.pdf]

Table S3: Results of the benchmark study analyzed by methods of Ghyselinck et. al 2011

Spectrum	Cluster number	OTU	Strain	Genus	Species	Subspecies
Z0021_RO_G07_1_H10_A	1	105	LMG 28391	*Salininema*	*proteolyticum*	
Z0021_RO_F11_2_G12_A	1	105	LMG 28391	*Salininema*	*proteolyticum*	
Z0021_RO_G01_1_H07_A	1	105	LMG 28391	*Salininema*	*proteolyticum*	
Z0021_LO_F09_2_G05_A	1	105	LMG 28391	*Salininema*	*proteolyticum*	
Z0021_LO_F11_2_G06_A	1	105	LMG 28391	*Salininema*	*proteolyticum*	
Z0021_RO_F12_2_G12_B	1	105	LMG 28391	*Salininema*	*proteolyticum*	
Z0021_RO_F06_2_G09_B	1	105	LMG 28391	*Salininema*	*proteolyticum*	
Z0021_LO_F10_2_G05_B	1	105	LMG 28391	*Salininema*	*proteolyticum*	
Z0021_RO_H04_2_H08_B	1	105	LMG 28391	*Salininema*	*proteolyticum*	
Z0021_LO_G12_1_H06_B	1	105	LMG 28391	*Salininema*	*proteolyticum*	
Z0021_RO_G06_1_H09_B	1	105	LMG 28391	*Salininema*	*proteolyticum*	
Z0021_RO_H02_2_H07_B	1	105	LMG 28391	*Salininema*	*proteolyticum*	
Z0021_RO_F10_2_G11_B	1	105	LMG 28391	*Salininema*	*proteolyticum*	
Z0021_LO_H05_2_H03_A	1	105	LMG 28391	*Salininema*	*proteolyticum*	
Z0021_LO_H01_2_H01_A	1	105	LMG 28391	*Salininema*	*proteolyticum*	
Z0021_RO_G09_1_H11_A	1	105	LMG 28391	*Salininema*	*proteolyticum*	
Z0021_RO_G11_1_H12_A	1	105	LMG 28391	*Salininema*	*proteolyticum*	
Z0021_LO_H03_2_H02_A	1	105	LMG 28391	*Salininema*	*proteolyticum*	
Z0021_RO_G12_1_H12_B	1	105	LMG 28391	*Salininema*	*proteolyticum*	
Z0021_RO_H01_2_H07_A	1	105	LMG 28391	*Salininema*	*proteolyticum*	
Z0021_LO_G06_1_H03_B	1	105	LMG 28391	*Salininema*	*proteolyticum*	
Z0021_LO_G08_1_H04_B	1	105	LMG 28391	*Salininema*	*proteolyticum*	
Z0021_RO_G02_1_H07_B	1	105	LMG 28391	*Salininema*	*proteolyticum*	
Z0021_LO_G10_1_H05_B	1	105	LMG 28391	*Salininema*	*proteolyticum*	
Z0021_LO_G09_1_H05_A	1	105	LMG 28391	*Salininema*	*proteolyticum*	
Z0021_RO_G05_1_H09_A	1	105	LMG 28391	*Salininema*	*proteolyticum*	
Z0021_RO_G03_1_H08_A	1	105	LMG 28391	*Salininema*	*proteolyticum*	
Z0021_LO_H06_2_H03_B	1	105	LMG 28391	*Salininema*	*proteolyticum*	
Z0021_LO_H04_2_H02_B	1	105	LMG 28391	*Salininema*	*proteolyticum*	
Z0021_RO_G08_1_H10_B	1	105	LMG 28391	*Salininema*	*proteolyticum*	
Z0021_LO_G11_1_H06_A	1	105	LMG 28391	*Salininema*	*proteolyticum*	
Z0021_RO_H03_2_H08_A	1	105	LMG 28391	*Salininema*	*proteolyticum*	
Z0013_LO_D05_2_F03_A	1	19	LMG 15863	*Haemophilus*	*influenzae*	
Z0013_LB_D07_1_C03_A	1	19	LMG 15863	*Haemophilus*	*influenzae*	
Z0013_RB_D04_1_C11_B	1	19	LMG 15863	*Haemophilus*	*influenzae*	
Z0013_LO_D02_2_F01_B	1	19	LMG 15863	*Haemophilus*	*influenzae*	
Z0013_RB_C10_2_C08_B	1	19	LMG 15863	*Haemophilus*	*influenzae*	
Z0013_RB_C09_2_C08_A	1	19	LMG 15863	*Haemophilus*	*influenzae*	
Z0013_LB_C10_2_C02_B	1	19	LMG 15863	*Haemophilus*	*influenzae*	
Z0013_RB_C12_2_C07_B	1	19	LMG 15863	*Haemophilus*	*influenzae*	
Z0013_RB_C11_2_C07_A	1	19	LMG 15863	*Haemophilus*	*influenzae*	
Z0013_RB_D03_1_C11_A	1	19	LMG 15863	*Haemophilus*	*influenzae*	
Z0013_RO_D07_2_F10_A	1	19	LMG 15863	*Haemophilus*	*influenzae*	
Z0013_LO_D01_2_F01_A	1	19	LMG 15863	*Haemophilus*	*influenzae*	
Z0013_LB_D02_1_C06_B	1	19	LMG 15863	*Haemophilus*	*influenzae*	

Z0013_LB_D05_1_C04_A	1	19	LMG 15863	*Haemophilus*	*influenzae*
Z0013_RB_D02_1_C12_B	1	19	LMG 15863	*Haemophilus*	*influenzae*
Z0013_LB_D06_1_C04_B	1	19	LMG 15863	*Haemophilus*	*influenzae*
Z0013_LB_D03_1_C05_A	1	19	LMG 15863	*Haemophilus*	*influenzae*
Z0013_LB_D04_1_C05_B	1	19	LMG 15863	*Haemophilus*	*influenzae*
Z0013_LO_D04_2_F02_B	1	19	LMG 15863	*Haemophilus*	*influenzae*
Z0013_RO_D06_2_F09_B	1	19	LMG 15863	*Haemophilus*	*influenzae*
Z0013_LB_C12_2_C01_B	1	19	LMG 15863	*Haemophilus*	*influenzae*
Z0013_RO_D09_2_F11_A	1	19	LMG 15863	*Haemophilus*	*influenzae*
Z0013_LO_D03_2_F02_A	1	19	LMG 15863	*Haemophilus*	*influenzae*
Z0013_LO_D06_2_F03_B	1	19	LMG 15863	*Haemophilus*	*influenzae*
Z0013_RB_D05_1_C10_A	1	19	LMG 15863	*Haemophilus*	*influenzae*
Z0013_RB_D06_1_C10_B	1	19	LMG 15863	*Haemophilus*	*influenzae*
Z0013_LB_D08_1_C03_B	1	19	LMG 15863	*Haemophilus*	*influenzae*
Z0013_RO_D08_2_F10_B	1	19	LMG 15863	*Haemophilus*	*influenzae*
Z0013_RB_D01_1_C12_A	1	19	LMG 15863	*Haemophilus*	*influenzae*
Z0013_LB_D01_1_C06_A	1	19	LMG 15863	*Haemophilus*	*influenzae*
Z0013_RO_D05_2_F09_A	1	19	LMG 15863	*Haemophilus*	*influenzae*
Z0013_LB_C11_2_C01_A	1	19	LMG 15863	*Haemophilus*	*influenzae*
Z0012_RB_B11_1_D07_A	1	4	LMG 11405	*Lactobacillus*	*plantarum*
Z0012_LB_B10_1_D02_B	1	4	LMG 11405	*Lactobacillus*	*plantarum*
Z0012_LB_B07_1_D03_A	1	4	LMG 11405	*Lactobacillus*	*plantarum*
Z0012_RB_C02_2_C12_B	1	4	LMG 11405	*Lactobacillus*	*plantarum*
Z0012_RB_C01_2_C12_A	1	4	LMG 11405	*Lactobacillus*	*plantarum*
Z0012_RB_C03_2_C11_A	1	4	LMG 11405	*Lactobacillus*	*plantarum*
Z0012_LB_C05_2_C04_A	1	4	LMG 11405	*Lactobacillus*	*plantarum*
Z0012_LB_C08_2_C03_B	1	4	LMG 11405	*Lactobacillus*	*plantarum*
Z0012_LB_C06_2_C04_B	1	4	LMG 11405	*Lactobacillus*	*plantarum*
Z0012_RB_B08_1_D09_B	1	4	LMG 11405	*Lactobacillus*	*plantarum*
Z0012_LB_C04_2_C05_B	1	4	LMG 11405	*Lactobacillus*	*plantarum*
Z0012_RB_B09_1_D08_A	1	4	LMG 11405	*Lactobacillus*	*plantarum*
Z0012_LB_C10_2_C02_B	1	4	LMG 11405	*Lactobacillus*	*plantarum*
Z0012_LB_C09_2_C02_A	1	4	LMG 11405	*Lactobacillus*	*plantarum*
Z0012_LB_C03_2_C05_A	1	4	LMG 11405	*Lactobacillus*	*plantarum*
Z0012_RB_B07_1_D09_A	1	4	LMG 11405	*Lactobacillus*	*plantarum*
Z0012_RB_C05_2_C10_A	1	4	LMG 11405	*Lactobacillus*	*plantarum*
Z0012_LB_C07_2_C03_A	1	4	LMG 11405	*Lactobacillus*	*plantarum*
Z0012_LB_C02_2_C06_B	1	4	LMG 11405	*Lactobacillus*	*plantarum*
Z0012_LB_B05_1_D04_A	1	4	LMG 11405	*Lactobacillus*	*plantarum*
Z0012_RB_C04_2_C11_B	1	4	LMG 11405	*Lactobacillus*	*plantarum*
Z0012_RB_B10_1_D08_B	1	4	LMG 11405	*Lactobacillus*	*plantarum*
Z0012_LB_C01_2_C06_A	1	4	LMG 11405	*Lactobacillus*	*plantarum*
Z0012_RB_B12_1_D07_B	1	4	LMG 11405	*Lactobacillus*	*plantarum*
Z0012_RB_B05_1_D10_A	1	4	LMG 11405	*Lactobacillus*	*plantarum*
Z0012_RB_B04_1_D11_B	1	4	LMG 11405	*Lactobacillus*	*plantarum*
Z0012_LB_B06_1_D04_B	1	4	LMG 11405	*Lactobacillus*	*plantarum*
Z0012_LB_B08_1_D03_B	1	4	LMG 11405	*Lactobacillus*	*plantarum*
Z0012_LB_B12_1_D01_B	1	4	LMG 11405	*Lactobacillus*	*plantarum*
Z0012_LB_B11_1_D01_A	1	4	LMG 11405	*Lactobacillus*	*plantarum*
Z0012_LB_B09_1_D02_A	1	4	LMG 11405	*Lactobacillus*	*plantarum*

Z0012_RB_B06_1_D10_B	1	4	LMG 11405	*Lactobacillus*	*plantarum*
Z0018_RO_D01_2_F07_A	1	4	LMG 24832	*Lactobacillus*	*plantarum*
Z0018_LO_G02_1_H01_B	1	4	LMG 24832	*Lactobacillus*	*plantarum*
Z0018_RO_D09_2_F11_A	1	4	LMG 24832	*Lactobacillus*	*plantarum*
Z0018_LO_F05_2_G03_A	1	4	LMG 24832	*Lactobacillus*	*plantarum*
Z0018_RO_D05_2_F09_A	1	4	LMG 24832	*Lactobacillus*	*plantarum*
Z0018_RO_D06_2_F09_B	1	4	LMG 24832	*Lactobacillus*	*plantarum*
Z0018_LO_G03_1_H02_A	1	4	LMG 24832	*Lactobacillus*	*plantarum*
Z0018_RO_D07_2_F10_A	1	4	LMG 24832	*Lactobacillus*	*plantarum*
Z0018_RO_D10_2_F11_B	1	4	LMG 24832	*Lactobacillus*	*plantarum*
Z0018_RO_D11_2_F12_A	1	4	LMG 24832	*Lactobacillus*	*plantarum*
Z0018_RO_D12_2_F12_B	1	4	LMG 24832	*Lactobacillus*	*plantarum*
Z0018_RO_D08_2_F10_B	1	4	LMG 24832	*Lactobacillus*	*plantarum*
Z0018_RO_F03_2_G08_A	1	4	LMG 24832	*Lactobacillus*	*plantarum*
Z0018_RO_E11_1_G12_A	1	4	LMG 24832	*Lactobacillus*	*plantarum*
Z0018_RO_E08_1_G10_B	1	4	LMG 24832	*Lactobacillus*	*plantarum*
Z0018_RO_F02_2_G07_B	1	4	LMG 24832	*Lactobacillus*	*plantarum*
Z0018_LO_F12_2_G06_B	1	4	LMG 24832	*Lactobacillus*	*plantarum*
Z0018_LO_G01_1_H01_A	1	4	LMG 24832	*Lactobacillus*	*plantarum*
Z0018_LO_G06_1_H03_B	1	4	LMG 24832	*Lactobacillus*	*plantarum*
Z0018_RO_D04_2_F08_B	1	4	LMG 24832	*Lactobacillus*	*plantarum*
Z0018_LO_F07_2_G04_A	1	4	LMG 24832	*Lactobacillus*	*plantarum*
Z0018_RO_D02_2_F07_B	1	4	LMG 24832	*Lactobacillus*	*plantarum*
Z0018_RO_C10_1_F11_B	1	4	LMG 24832	*Lactobacillus*	*plantarum*
Z0018_LO_F11_2_G06_A	1	4	LMG 24832	*Lactobacillus*	*plantarum*
Z0018_LO_F06_2_G03_B	1	4	LMG 24832	*Lactobacillus*	*plantarum*
Z0018_RO_E07_1_G10_A	1	4	LMG 24832	*Lactobacillus*	*plantarum*
Z0018_RO_E05_1_G09_A	1	4	LMG 24832	*Lactobacillus*	*plantarum*
Z0018_RO_E03_1_G08_A	1	4	LMG 24832	*Lactobacillus*	*plantarum*
Z0018_RO_F08_2_G10_B	1	4	LMG 24832	*Lactobacillus*	*plantarum*
Z0018_RO_F07_2_G10_A	1	4	LMG 24832	*Lactobacillus*	*plantarum*
Z0018_LO_G10_1_H05_B	1	4	LMG 24832	*Lactobacillus*	*plantarum*
Z0018_LO_G05_1_H03_A	1	4	LMG 24832	*Lactobacillus*	*plantarum*
Z0020_RB_E09_2_B08_A	1	4	LMG 26367	*Lactobacillus*	*plantarum*
Z0020_LB_E10_2_B02_B	1	4	LMG 26367	*Lactobacillus*	*plantarum*
Z0020_RB_F04_1_B11_B	1	4	LMG 26367	*Lactobacillus*	*plantarum*
Z0020_RB_E06_2_B10_B	1	4	LMG 26367	*Lactobacillus*	*plantarum*
Z0020_RB_F01_1_B12_A	1	4	LMG 26367	*Lactobacillus*	*plantarum*
Z0020_LB_F02_1_B06_B	1	4	LMG 26367	*Lactobacillus*	*plantarum*
Z0020_LB_F03_1_B05_A	1	4	LMG 26367	*Lactobacillus*	*plantarum*
Z0020_LB_E07_2_B03_A	1	4	LMG 26367	*Lactobacillus*	*plantarum*
Z0020_LB_F11_1_B01_A	1	4	LMG 26367	*Lactobacillus*	*plantarum*
Z0020_LB_F12_1_B01_B	1	4	LMG 26367	*Lactobacillus*	*plantarum*
Z0020_RB_E05_2_B10_A	1	4	LMG 26367	*Lactobacillus*	*plantarum*
Z0020_RB_F02_1_B12_B	1	4	LMG 26367	*Lactobacillus*	*plantarum*
Z0020_RB_E10_2_B08_B	1	4	LMG 26367	*Lactobacillus*	*plantarum*
Z0020_LB_F06_1_B04_B	1	4	LMG 26367	*Lactobacillus*	*plantarum*
Z0020_LB_F10_1_B02_B	1	4	LMG 26367	*Lactobacillus*	*plantarum*
Z0020_LB_F08_1_B03_B	1	4	LMG 26367	*Lactobacillus*	*plantarum*
Z0020_RB_E11_2_B07_A	1	4	LMG 26367	*Lactobacillus*	*plantarum*

Z0020_LB_F04_1_B05_B	1	4	LMG 26367	*Lactobacillus*	*plantarum*
Z0020_RB_F03_1_B11_A	1	4	LMG 26367	*Lactobacillus*	*plantarum*
Z0020_LB_E12_2_B01_B	1	4	LMG 26367	*Lactobacillus*	*plantarum*
Z0020_LB_F01_1_B06_A	1	4	LMG 26367	*Lactobacillus*	*plantarum*
Z0020_LB_F09_1_B02_A	1	4	LMG 26367	*Lactobacillus*	*plantarum*
Z0020_RB_E08_2_B09_B	1	4	LMG 26367	*Lactobacillus*	*plantarum*
Z0020_LB_G01_2_A06_A	1	4	LMG 26367	*Lactobacillus*	*plantarum*
Z0020_RB_E07_2_B09_A	1	4	LMG 26367	*Lactobacillus*	*plantarum*
Z0020_LB_E08_2_B03_B	1	4	LMG 26367	*Lactobacillus*	*plantarum*
Z0020_LB_E11_2_B01_A	1	4	LMG 26367	*Lactobacillus*	*plantarum*
Z0020_LB_E09_2_B02_A	1	4	LMG 26367	*Lactobacillus*	*plantarum*
Z0020_RB_E12_2_B07_B	1	4	LMG 26367	*Lactobacillus*	*plantarum*
Z0020_LB_F05_1_B04_A	1	4	LMG 26367	*Lactobacillus*	*plantarum*
Z0020_LB_F07_1_B03_A	1	4	LMG 26367	*Lactobacillus*	*plantarum*
Z0020_RB_E04_2_B11_B	1	4	LMG 26367	*Lactobacillus*	*plantarum*
Z0013_LO_F10_2_G05_B	1	22	LMG 16673	*Lactobacillus*	*paraplantarum*
Z0013_LB_E12_2_B01_B	1	22	LMG 16673	*Lactobacillus*	*paraplantarum*
Z0013_LB_F07_1_B03_A	1	22	LMG 16673	*Lactobacillus*	*paraplantarum*
Z0013_LO_F07_2_G04_A	1	22	LMG 16673	*Lactobacillus*	*paraplantarum*
Z0013_RB_F07_1_B09_A	1	22	LMG 16673	*Lactobacillus*	*paraplantarum*
Z0013_RB_F09_1_B08_A	1	22	LMG 16673	*Lactobacillus*	*paraplantarum*
Z0013_LO_F01_2_G01_A	1	22	LMG 16673	*Lactobacillus*	*paraplantarum*
Z0013_LB_E11_2_B01_A	1	22	LMG 16673	*Lactobacillus*	*paraplantarum*
Z0013_LB_F08_1_B03_B	1	22	LMG 16673	*Lactobacillus*	*paraplantarum*
Z0013_RB_F01_1_B12_A	1	22	LMG 16673	*Lactobacillus*	*paraplantarum*
Z0013_LB_F06_1_B04_B	1	22	LMG 16673	*Lactobacillus*	*paraplantarum*
Z0013_LB_E10_2_B02_B	1	22	LMG 16673	*Lactobacillus*	*paraplantarum*
Z0013_LO_F09_2_G05_A	1	22	LMG 16673	*Lactobacillus*	*paraplantarum*
Z0013_LB_F02_1_B06_B	1	22	LMG 16673	*Lactobacillus*	*paraplantarum*
Z0013_LB_F09_1_B02_A	1	22	LMG 16673	*Lactobacillus*	*paraplantarum*
Z0013_LB_F10_1_B02_B	1	22	LMG 16673	*Lactobacillus*	*paraplantarum*
Z0013_RB_E10_2_B08_B	1	22	LMG 16673	*Lactobacillus*	*paraplantarum*
Z0013_RB_E11_2_B07_A	1	22	LMG 16673	*Lactobacillus*	*paraplantarum*
Z0013_RB_F08_1_B09_B	1	22	LMG 16673	*Lactobacillus*	*paraplantarum*
Z0013_LO_F05_2_G03_A	1	22	LMG 16673	*Lactobacillus*	*paraplantarum*
Z0013_LB_F04_1_B05_B	1	22	LMG 16673	*Lactobacillus*	*paraplantarum*
Z0013_RO_E12_1_G12_B	1	22	LMG 16673	*Lactobacillus*	*paraplantarum*
Z0013_LB_F05_1_B04_A	1	22	LMG 16673	*Lactobacillus*	*paraplantarum*
Z0013_RO_F01_2_G07_A	1	22	LMG 16673	*Lactobacillus*	*paraplantarum*
Z0013_LO_F06_2_G03_B	1	22	LMG 16673	*Lactobacillus*	*paraplantarum*
Z0013_RB_F04_1_B11_B	1	22	LMG 16673	*Lactobacillus*	*paraplantarum*
Z0013_LO_F04_2_G02_B	1	22	LMG 16673	*Lactobacillus*	*paraplantarum*
Z0013_LB_G01_2_A06_A	1	22	LMG 16673	*Lactobacillus*	*paraplantarum*
Z0013_LO_E12_1_G06_B	1	22	LMG 16673	*Lactobacillus*	*paraplantarum*
Z0013_LB_E09_2_B02_A	1	22	LMG 16673	*Lactobacillus*	*paraplantarum*
Z0013_RB_E09_2_B08_A	1	22	LMG 16673	*Lactobacillus*	*paraplantarum*
Z0013_LB_F01_1_B06_A	1	22	LMG 16673	*Lactobacillus*	*paraplantarum*
Z0014_LO_D09_2_F05_A	2	29	LMG 19264	*Corynebacterium*	*casei*
Z0014_LO_E07_1_G04_A	2	29	LMG 19264	*Corynebacterium*	*casei*
Z0014_LO_E09_1_G05_A	2	29	LMG 19264	*Corynebacterium*	*casei*

Z0014_LO_D12_2_F06_B	2	29	LMG 19264	*Corynebacterium*	*casei*	
Z0014_LO_D08_2_F04_B	2	29	LMG 19264	*Corynebacterium*	*casei*	
Z0014_RO_C11_1_F12_A	2	29	LMG 19264	*Corynebacterium*	*casei*	
Z0014_RO_C10_1_F11_B	2	29	LMG 19264	*Corynebacterium*	*casei*	
Z0014_RO_C05_1_F09_A	2	29	LMG 19264	*Corynebacterium*	*casei*	
Z0014_LO_C06_1_F03_B	2	29	LMG 19264	*Corynebacterium*	*casei*	
Z0014_RO_D04_2_F08_B	2	29	LMG 19264	*Corynebacterium*	*casei*	
Z0014_LO_D06_2_F03_B	2	29	LMG 19264	*Corynebacterium*	*casei*	
Z0014_RO_D09_2_F11_A	2	29	LMG 19264	*Corynebacterium*	*casei*	
Z0014_LO_E01_1_G01_A	2	29	LMG 19264	*Corynebacterium*	*casei*	
Z0014_RO_C12_1_F12_B	2	29	LMG 19264	*Corynebacterium*	*casei*	
Z0014_LO_D04_2_F02_B	2	29	LMG 19264	*Corynebacterium*	*casei*	
Z0014_LO_D07_2_F04_A	2	29	LMG 19264	*Corynebacterium*	*casei*	
Z0014_LO_D10_2_F05_B	2	29	LMG 19264	*Corynebacterium*	*casei*	
Z0014_RO_E03_1_G08_A	2	29	LMG 19264	*Corynebacterium*	*casei*	
Z0014_RO_E06_1_G09_B	2	29	LMG 19264	*Corynebacterium*	*casei*	
Z0014_LO_C09_1_F05_A	2	29	LMG 19264	*Corynebacterium*	*casei*	
Z0014_LO_E08_1_G04_B	2	29	LMG 19264	*Corynebacterium*	*casei*	
Z0014_RO_D08_2_F10_B	2	29	LMG 19264	*Corynebacterium*	*casei*	
Z0014_LO_E06_1_G03_B	2	29	LMG 19264	*Corynebacterium*	*casei*	
Z0014_LO_E12_1_G06_B	2	29	LMG 19264	*Corynebacterium*	*casei*	
Z0014_RO_C07_1_F10_A	2	29	LMG 19264	*Corynebacterium*	*casei*	
Z0014_LO_C11_1_F06_A	2	29	LMG 19264	*Corynebacterium*	*casei*	
Z0014_RO_D07_2_F10_A	2	29	LMG 19264	*Corynebacterium*	*casei*	
Z0014_LO_E05_1_G03_A	2	29	LMG 19264	*Corynebacterium*	*casei*	
Z0014_RO_C08_1_F10_B	2	29	LMG 19264	*Corynebacterium*	*casei*	
Z0014_LO_E11_1_G06_A	2	29	LMG 19264	*Corynebacterium*	*casei*	
Z0014_RO_C09_1_F11_A	2	29	LMG 19264	*Corynebacterium*	*casei*	
Z0014_RO_C06_1_F09_B	2	29	LMG 19264	*Corynebacterium*	*casei*	
Z0014_LB_A07_2_D03_A	3	4	LMG 18021	*Lactobacillus*	*plantarum*	
Z0014_LB_A08_2_D03_B	3	4	LMG 18021	*Lactobacillus*	*plantarum*	
Z0024_RO_G07_1_H10_A	4	131	LMG 7135	*Bacillus*	*subtilis*	*subtilis*
Z0024_RB_A07_2_D09_A	4	131	LMG 7135	*Bacillus*	*subtilis*	*subtilis*
Z0024_RB_A02_2_D12_B	4	131	LMG 7135	*Bacillus*	*subtilis*	*subtilis*
Z0024_RB_A05_2_D10_A	4	131	LMG 7135	*Bacillus*	*subtilis*	*subtilis*
Z0024_RB_A03_2_D11_A	4	131	LMG 7135	*Bacillus*	*subtilis*	*subtilis*
Z0024_RO_G12_1_H12_B	4	131	LMG 7135	*Bacillus*	*subtilis*	*subtilis*
Z0024_RO_G10_1_H11_B	4	131	LMG 7135	*Bacillus*	*subtilis*	*subtilis*
Z0024_RB_A01_2_D12_A	4	131	LMG 7135	*Bacillus*	*subtilis*	*subtilis*
Z0024_RO_G01_1_H07_A	4	131	LMG 7135	*Bacillus*	*subtilis*	*subtilis*
Z0024_RB_A12_2_D07_B	4	131	LMG 7135	*Bacillus*	*subtilis*	*subtilis*
Z0024_RO_G06_1_H09_B	4	131	LMG 7135	*Bacillus*	*subtilis*	*subtilis*
Z0024_RO_F12_2_G12_B	4	131	LMG 7135	*Bacillus*	*subtilis*	*subtilis*
Z0024_RB_A04_2_D11_B	4	131	LMG 7135	*Bacillus*	*subtilis*	*subtilis*
Z0024_LO_H12_2_H06_B	4	131	LMG 7135	*Bacillus*	*subtilis*	*subtilis*
Z0024_RB_B01_1_D12_A	4	131	LMG 7135	*Bacillus*	*subtilis*	*subtilis*
Z0024_RO_H02_2_H07_B	4	131	LMG 7135	*Bacillus*	*subtilis*	*subtilis*
Z0024_RO_H01_2_H07_A	4	131	LMG 7135	*Bacillus*	*subtilis*	*subtilis*
Z0024_RO_H04_2_H08_B	4	131	LMG 7135	*Bacillus*	*subtilis*	*subtilis*
Z0024_RO_G11_1_H12_A	4	131	LMG 7135	*Bacillus*	*subtilis*	*subtilis*

Z0024_RB_A09_2_D08_A	4	131	LMG 7135	*Bacillus*	*subtilis*	*subtilis*
Z0024_RB_A08_2_D09_B	4	131	LMG 7135	*Bacillus*	*subtilis*	*subtilis*
Z0024_LO_H09_2_H05_A	4	131	LMG 7135	*Bacillus*	*subtilis*	*subtilis*
Z0024_LO_H08_2_H04_B	4	131	LMG 7135	*Bacillus*	*subtilis*	*subtilis*
Z0024_RB_B02_1_D12_B	4	131	LMG 7135	*Bacillus*	*subtilis*	*subtilis*
Z0024_RO_G08_1_H10_B	4	131	LMG 7135	*Bacillus*	*subtilis*	*subtilis*
Z0024_RO_G04_1_H08_B	4	131	LMG 7135	*Bacillus*	*subtilis*	*subtilis*
Z0024_RB_A10_2_D08_B	4	131	LMG 7135	*Bacillus*	*subtilis*	*subtilis*
Z0024_RB_A11_2_D07_A	4	131	LMG 7135	*Bacillus*	*subtilis*	*subtilis*
Z0024_RB_A06_2_D10_B	4	131	LMG 7135	*Bacillus*	*subtilis*	*subtilis*
Z0024_RO_G03_1_H08_A	4	131	LMG 7135	*Bacillus*	*subtilis*	*subtilis*
Z0024_RO_H03_2_H08_A	4	131	LMG 7135	*Bacillus*	*subtilis*	*subtilis*
Z0024_LO_H07_2_H04_A	4	131	LMG 7135	*Bacillus*	*subtilis*	*subtilis*
Z0013_RB_H07_1_A09_A	5	14	LMG 13349	*Staphylococcus*	*haemolyticus*	
Z0013_LB_H09_1_A02_A	5	14	LMG 13349	*Staphylococcus*	*haemolyticus*	
Z0013_LO_A02_1_E01_B	5	14	LMG 13349	*Staphylococcus*	*haemolyticus*	
Z0013_LB_F03_1_B05_A	5	14	LMG 13349	*Staphylococcus*	*haemolyticus*	
Z0013_RO_F05_2_G09_A	5	14	LMG 13349	*Staphylococcus*	*haemolyticus*	
Z0013_LB_A03_2_D05_A	5	14	LMG 13349	*Staphylococcus*	*haemolyticus*	
Z0013_LO_F03_2_G02_A	5	14	LMG 13349	*Staphylococcus*	*haemolyticus*	
Z0013_RB_E08_2_B09_B	5	14	LMG 13349	*Staphylococcus*	*haemolyticus*	
Z0013_RB_H10_1_A08_B	5	14	LMG 13349	*Staphylococcus*	*haemolyticus*	
Z0013_LO_H09_2_H05_A	5	14	LMG 13349	*Staphylococcus*	*haemolyticus*	
Z0013_LO_H12_2_H06_B	5	14	LMG 13349	*Staphylococcus*	*haemolyticus*	
Z0013_LO_A01_1_E01_A	5	14	LMG 13349	*Staphylococcus*	*haemolyticus*	
Z0013_RB_H08_1_A09_B	5	14	LMG 13349	*Staphylococcus*	*haemolyticus*	
Z0013_LO_F02_2_G01_B	5	14	LMG 13349	*Staphylococcus*	*haemolyticus*	
Z0013_RB_H11_1_A07_A	5	14	LMG 13349	*Staphylococcus*	*haemolyticus*	
Z0013_LB_A05_2_D04_A	5	14	LMG 13349	*Staphylococcus*	*haemolyticus*	
Z0013_RB_A02_2_D12_B	5	14	LMG 13349	*Staphylococcus*	*haemolyticus*	
Z0013_RB_H12_1_A07_B	5	14	LMG 13349	*Staphylococcus*	*haemolyticus*	
Z0013_LB_A01_2_D06_A	5	14	LMG 13349	*Staphylococcus*	*haemolyticus*	
Z0013_LB_A02_2_D06_B	5	14	LMG 13349	*Staphylococcus*	*haemolyticus*	
Z0013_LB_H10_1_A02_B	5	14	LMG 13349	*Staphylococcus*	*haemolyticus*	
Z0013_LB_H11_1_A01_A	5	14	LMG 13349	*Staphylococcus*	*haemolyticus*	
Z0013_LO_A03_1_E02_A	5	14	LMG 13349	*Staphylococcus*	*haemolyticus*	
Z0013_LB_A04_2_D05_B	5	14	LMG 13349	*Staphylococcus*	*haemolyticus*	
Z0013_RB_H09_1_A08_A	5	14	LMG 13349	*Staphylococcus*	*haemolyticus*	
Z0013_LO_H11_2_H06_A	5	14	LMG 13349	*Staphylococcus*	*haemolyticus*	
Z0013_RB_A01_2_D12_A	5	14	LMG 13349	*Staphylococcus*	*haemolyticus*	
Z0013_LB_A06_2_D04_B	5	14	LMG 13349	*Staphylococcus*	*haemolyticus*	
Z0013_LO_H10_2_H05_B	5	14	LMG 13349	*Staphylococcus*	*haemolyticus*	
Z0013_LO_A04_1_E02_B	5	14	LMG 13349	*Staphylococcus*	*haemolyticus*	
Z0013_LO_A05_1_E03_A	5	14	LMG 13349	*Staphylococcus*	*haemolyticus*	
Z0013_LB_H12_1_A01_B	5	14	LMG 13349	*Staphylococcus*	*haemolyticus*	
Z0024_LB_D07_1_C03_A	6	126	LMG 6896	*Streptococcus*	*thermophilus*	
Z0024_LB_E03_2_B05_A	6	126	LMG 6896	*Streptococcus*	*thermophilus*	
Z0024_RB_E07_2_B09_A	6	126	LMG 6896	*Streptococcus*	*thermophilus*	
Z0024_LB_E04_2_B05_B	6	126	LMG 6896	*Streptococcus*	*thermophilus*	
Z0024_RB_F04_1_B11_B	6	126	LMG 6896	*Streptococcus*	*thermophilus*	

Z0024_LB_E09_2_B02_A	6	126	LMG 6896	*Streptococcus*	*thermophilus*
Z0024_RB_F05_1_B10_A	6	126	LMG 6896	*Streptococcus*	*thermophilus*
Z0024_LB_E10_2_B02_B	6	126	LMG 6896	*Streptococcus*	*thermophilus*
Z0016_RO_A01_1_E07_A	7	49	LMG 22735	*Finegoldia*	*magna*
Z0016_LO_A04_1_E02_B	7	49	LMG 22735	*Finegoldia*	*magna*
Z0016_LB_H07_1_A03_A	7	49	LMG 22735	*Finegoldia*	*magna*
Z0016_LO_A06_1_E03_B	7	49	LMG 22735	*Finegoldia*	*magna*
Z0016_LO_A07_1_E04_A	7	49	LMG 22735	*Finegoldia*	*magna*
Z0016_LO_A08_1_E04_B	7	49	LMG 22735	*Finegoldia*	*magna*
Z0016_RO_A04_1_E08_B	7	49	LMG 22735	*Finegoldia*	*magna*
Z0016_LO_A05_1_E03_A	7	49	LMG 22735	*Finegoldia*	*magna*
Z0016_RB_H09_1_A08_A	7	49	LMG 22735	*Finegoldia*	*magna*
Z0016_RB_H07_1_A09_A	7	49	LMG 22735	*Finegoldia*	*magna*
Z0016_RO_A03_1_E08_A	7	49	LMG 22735	*Finegoldia*	*magna*
Z0016_LO_A09_1_E05_A	7	49	LMG 22735	*Finegoldia*	*magna*
Z0016_RB_H10_1_A08_B	7	49	LMG 22735	*Finegoldia*	*magna*
Z0016_LB_H10_1_A02_B	7	49	LMG 22735	*Finegoldia*	*magna*
Z0016_LB_H09_1_A02_A	7	49	LMG 22735	*Finegoldia*	*magna*
Z0016_RB_H05_1_A10_A	7	49	LMG 22735	*Finegoldia*	*magna*
Z0016_RB_H04_1_A11_B	7	49	LMG 22735	*Finegoldia*	*magna*
Z0016_RB_H12_1_A07_B	7	49	LMG 22735	*Finegoldia*	*magna*
Z0016_LB_H12_1_A01_B	7	49	LMG 22735	*Finegoldia*	*magna*
Z0016_RB_H11_1_A07_A	7	49	LMG 22735	*Finegoldia*	*magna*
Z0016_LO_A02_1_E01_B	7	49	LMG 22735	*Finegoldia*	*magna*
Z0016_LO_A03_1_E02_A	7	49	LMG 22735	*Finegoldia*	*magna*
Z0016_LB_H08_1_A03_B	7	49	LMG 22735	*Finegoldia*	*magna*
Z0016_LB_H11_1_A01_A	7	49	LMG 22735	*Finegoldia*	*magna*
Z0016_RB_H08_1_A09_B	7	49	LMG 22735	*Finegoldia*	*magna*
Z0016_RB_H06_1_A10_B	7	49	LMG 22735	*Finegoldia*	*magna*
Z0016_LO_A01_1_E01_A	7	49	LMG 22735	*Finegoldia*	*magna*
Z0016_RO_A02_1_E07_B	7	49	LMG 22735	*Finegoldia*	*magna*
Z0025_RO_A02_1_E07_B	8	138	LMG 8787	*Curtobacterium*	*luteum*
Z0025_LO_A01_1_E01_A	8	138	LMG 8787	*Curtobacterium*	*luteum*
Z0025_LO_A03_1_E02_A	8	138	LMG 8787	*Curtobacterium*	*luteum*
Z0025_RO_A01_1_E07_A	8	138	LMG 8787	*Curtobacterium*	*luteum*
Z0025_RO_A10_1_E11_B	8	138	LMG 8787	*Curtobacterium*	*luteum*
Z0025_RO_A09_1_E11_A	8	138	LMG 8787	*Curtobacterium*	*luteum*
Z0025_RO_B01_2_E07_A	8	138	LMG 8787	*Curtobacterium*	*luteum*
Z0025_LO_B10_2_E05_B	8	138	LMG 8787	*Curtobacterium*	*luteum*
Z0025_LO_B09_2_E05_A	8	138	LMG 8787	*Curtobacterium*	*luteum*
Z0025_RO_A07_1_E10_A	8	138	LMG 8787	*Curtobacterium*	*luteum*
Z0025_RO_A06_1_E09_B	8	138	LMG 8787	*Curtobacterium*	*luteum*
Z0025_RO_A08_1_E10_B	8	138	LMG 8787	*Curtobacterium*	*luteum*
Z0025_LO_A07_1_E04_A	8	138	LMG 8787	*Curtobacterium*	*luteum*
Z0025_LO_B11_2_E06_A	8	138	LMG 8787	*Curtobacterium*	*luteum*
Z0025_LO_B08_2_E04_B	8	138	LMG 8787	*Curtobacterium*	*luteum*
Z0025_RO_A03_1_E08_A	8	138	LMG 8787	*Curtobacterium*	*luteum*
Z0025_LO_A04_1_E02_B	8	138	LMG 8787	*Curtobacterium*	*luteum*
Z0025_RO_A05_1_E09_A	8	138	LMG 8787	*Curtobacterium*	*luteum*
Z0025_RO_A04_1_E08_B	8	138	LMG 8787	*Curtobacterium*	*luteum*

Z0025_RO_A11_1_E12_A	8	138	LMG 8787	*Curtobacterium*	*luteum*
Z0025_RO_B02_2_E07_B	8	138	LMG 8787	*Curtobacterium*	*luteum*
Z0025_RO_A12_1_E12_B	8	138	LMG 8787	*Curtobacterium*	*luteum*
Z0025_LO_B03_2_E02_A	8	138	LMG 8787	*Curtobacterium*	*luteum*
Z0025_RO_B04_2_E08_B	8	138	LMG 8787	*Curtobacterium*	*luteum*
Z0025_RO_B03_2_E08_A	8	138	LMG 8787	*Curtobacterium*	*luteum*
Z0025_LO_B07_2_E04_A	8	138	LMG 8787	*Curtobacterium*	*luteum*
Z0025_LO_B01_2_E01_A	8	138	LMG 8787	*Curtobacterium*	*luteum*
Z0025_LO_B06_2_E03_B	8	138	LMG 8787	*Curtobacterium*	*luteum*
Z0012_RO_C04_1_F08_B	9	12	LMG 129	*Thalassobius*	*gelatinovorus*
Z0012_LO_B11_2_E06_A	9	12	LMG 129	*Thalassobius*	*gelatinovorus*
Z0012_LO_C02_1_F01_B	9	12	LMG 129	*Thalassobius*	*gelatinovorus*
Z0012_LO_D01_2_F01_A	9	12	LMG 129	*Thalassobius*	*gelatinovorus*
Z0012_RO_B12_2_E12_B	9	12	LMG 129	*Thalassobius*	*gelatinovorus*
Z0012_LO_B07_2_E04_A	9	12	LMG 129	*Thalassobius*	*gelatinovorus*
Z0012_LO_C07_1_F04_A	9	12	LMG 129	*Thalassobius*	*gelatinovorus*
Z0012_LO_B05_2_E03_A	9	12	LMG 129	*Thalassobius*	*gelatinovorus*
Z0012_LO_A03_1_E02_A	9	12	LMG 129	*Thalassobius*	*gelatinovorus*
Z0012_LO_C04_1_F02_B	9	12	LMG 129	*Thalassobius*	*gelatinovorus*
Z0012_LO_A12_1_E06_B	9	12	LMG 129	*Thalassobius*	*gelatinovorus*
Z0012_LO_B12_2_E06_B	9	12	LMG 129	*Thalassobius*	*gelatinovorus*
Z0012_LO_B04_2_E02_B	9	12	LMG 129	*Thalassobius*	*gelatinovorus*
Z0012_RO_B08_2_E10_B	9	12	LMG 129	*Thalassobius*	*gelatinovorus*
Z0012_LO_A02_1_E01_B	9	12	LMG 129	*Thalassobius*	*gelatinovorus*
Z0012_RO_B06_2_E09_B	9	12	LMG 129	*Thalassobius*	*gelatinovorus*
Z0012_LO_C12_1_F06_B	9	12	LMG 129	*Thalassobius*	*gelatinovorus*
Z0012_RO_B02_2_E07_B	9	12	LMG 129	*Thalassobius*	*gelatinovorus*
Z0012_LO_B10_2_E05_B	9	12	LMG 129	*Thalassobius*	*gelatinovorus*
Z0012_RO_C09_1_F11_A	9	12	LMG 129	*Thalassobius*	*gelatinovorus*
Z0012_LO_A08_1_E04_B	9	12	LMG 129	*Thalassobius*	*gelatinovorus*
Z0012_LO_B09_2_E05_A	9	12	LMG 129	*Thalassobius*	*gelatinovorus*
Z0012_RO_C08_1_F10_B	9	12	LMG 129	*Thalassobius*	*gelatinovorus*
Z0012_LO_C01_1_F01_A	9	12	LMG 129	*Thalassobius*	*gelatinovorus*
Z0012_RO_C05_1_F09_A	9	12	LMG 129	*Thalassobius*	*gelatinovorus*
Z0012_LO_A04_1_E02_B	9	12	LMG 129	*Thalassobius*	*gelatinovorus*
Z0012_LO_A10_1_E05_B	9	12	LMG 129	*Thalassobius*	*gelatinovorus*
Z0012_RO_B04_2_E08_B	9	12	LMG 129	*Thalassobius*	*gelatinovorus*
Z0012_RO_B11_2_E12_A	9	12	LMG 129	*Thalassobius*	*gelatinovorus*
Z0012_LO_C10_1_F05_B	9	12	LMG 129	*Thalassobius*	*gelatinovorus*
Z0012_LO_A06_1_E03_B	9	12	LMG 129	*Thalassobius*	*gelatinovorus*
Z0012_RO_C02_1_F07_B	9	12	LMG 129	*Thalassobius*	*gelatinovorus*
Z0011_RO_E04_1_G08_B	10	2	LMG 11039	*Bifidobacterium*	*angulatum*
Z0011_LO_F06_2_G03_B	10	2	LMG 11039	*Bifidobacterium*	*angulatum*
Z0011_RO_E08_1_G10_B	10	2	LMG 11039	*Bifidobacterium*	*angulatum*
Z0011_RO_E09_1_G11_A	10	2	LMG 11039	*Bifidobacterium*	*angulatum*
Z0011_RO_D06_2_F09_B	10	2	LMG 11039	*Bifidobacterium*	*angulatum*
Z0011_LO_F02_2_G01_B	10	2	LMG 11039	*Bifidobacterium*	*angulatum*
Z0011_LO_E11_1_G06_A	10	2	LMG 11039	*Bifidobacterium*	*angulatum*
Z0011_RO_C09_1_F11_A	10	2	LMG 11039	*Bifidobacterium*	*angulatum*
Z0011_LO_E09_1_G05_A	10	2	LMG 11039	*Bifidobacterium*	*angulatum*

Z0011_RO_C08_1_F10_B	10	2	LMG 11039	*Bifidobacterium*	*angulatum*	
Z0011_RO_C03_1_F08_A	10	2	LMG 11039	*Bifidobacterium*	*angulatum*	
Z0011_RO_C12_1_F12_B	10	2	LMG 11039	*Bifidobacterium*	*angulatum*	
Z0011_LO_E12_1_G06_B	10	2	LMG 11039	*Bifidobacterium*	*angulatum*	
Z0011_RO_C05_1_F09_A	10	2	LMG 11039	*Bifidobacterium*	*angulatum*	
Z0011_LO_E01_1_G01_A	10	2	LMG 11039	*Bifidobacterium*	*angulatum*	
Z0011_RO_D08_2_F10_B	10	2	LMG 11039	*Bifidobacterium*	*angulatum*	
Z0011_LO_E02_1_G01_B	10	2	LMG 11039	*Bifidobacterium*	*angulatum*	
Z0011_RO_D01_2_F07_A	10	2	LMG 11039	*Bifidobacterium*	*angulatum*	
Z0011_RO_B10_2_E11_B	10	2	LMG 11039	*Bifidobacterium*	*angulatum*	
Z0011_LO_D08_2_F04_B	10	2	LMG 11039	*Bifidobacterium*	*angulatum*	
Z0011_LO_D09_2_F05_A	10	2	LMG 11039	*Bifidobacterium*	*angulatum*	
Z0011_RO_B06_2_E09_B	10	2	LMG 11039	*Bifidobacterium*	*angulatum*	
Z0011_LO_D02_2_F01_B	10	2	LMG 11039	*Bifidobacterium*	*angulatum*	
Z0011_RO_D07_2_F10_A	10	2	LMG 11039	*Bifidobacterium*	*angulatum*	
Z0011_RO_D12_2_F12_B	10	2	LMG 11039	*Bifidobacterium*	*angulatum*	
Z0011_RO_D11_2_F12_A	10	2	LMG 11039	*Bifidobacterium*	*angulatum*	
Z0011_RO_E01_1_G07_A	10	2	LMG 11039	*Bifidobacterium*	*angulatum*	
Z0011_RO_D05_2_F09_A	10	2	LMG 11039	*Bifidobacterium*	*angulatum*	
Z0011_RO_C04_1_F08_B	10	2	LMG 11039	*Bifidobacterium*	*angulatum*	
Z0011_LO_E05_1_G03_A	10	2	LMG 11039	*Bifidobacterium*	*angulatum*	
Z0011_LO_E06_1_G03_B	10	2	LMG 11039	*Bifidobacterium*	*angulatum*	
Z0011_LO_E04_1_G02_B	10	2	LMG 11039	*Bifidobacterium*	*angulatum*	
Z0016_LO_G07_1_H04_A	11	53	LMG 23078	*Streptomyces*	*albus*	*albus*
Z0016_LO_G12_1_H06_B	11	53	LMG 23078	*Streptomyces*	*albus*	*albus*
Z0016_LO_H02_2_H01_B	11	53	LMG 23078	*Streptomyces*	*albus*	*albus*
Z0016_RO_G08_1_H10_B	11	53	LMG 23078	*Streptomyces*	*albus*	*albus*
Z0016_LO_G10_1_H05_B	11	53	LMG 23078	*Streptomyces*	*albus*	*albus*
Z0016_LO_G05_1_H03_A	11	53	LMG 23078	*Streptomyces*	*albus*	*albus*
Z0016_LO_G08_1_H04_B	11	53	LMG 23078	*Streptomyces*	*albus*	*albus*
Z0016_LO_G09_1_H05_A	11	53	LMG 23078	*Streptomyces*	*albus*	*albus*
Z0016_RO_F06_2_G09_B	11	53	LMG 23078	*Streptomyces*	*albus*	*albus*
Z0016_RO_G07_1_H10_A	11	53	LMG 23078	*Streptomyces*	*albus*	*albus*
Z0016_RO_H04_2_H08_B	11	53	LMG 23078	*Streptomyces*	*albus*	*albus*
Z0016_LO_H03_2_H02_A	11	53	LMG 23078	*Streptomyces*	*albus*	*albus*
Z0016_RO_H03_2_H08_A	11	53	LMG 23078	*Streptomyces*	*albus*	*albus*
Z0016_RO_G11_1_H12_A	11	53	LMG 23078	*Streptomyces*	*albus*	*albus*
Z0016_LO_G11_1_H06_A	11	53	LMG 23078	*Streptomyces*	*albus*	*albus*
Z0016_LO_G06_1_H03_B	11	53	LMG 23078	*Streptomyces*	*albus*	*albus*
Z0016_RO_H02_2_H07_B	11	53	LMG 23078	*Streptomyces*	*albus*	*albus*
Z0016_RO_G09_1_H11_A	11	53	LMG 23078	*Streptomyces*	*albus*	*albus*
Z0016_RO_G12_1_H12_B	11	53	LMG 23078	*Streptomyces*	*albus*	*albus*
Z0016_LO_H04_2_H02_B	11	53	LMG 23078	*Streptomyces*	*albus*	*albus*
Z0016_RO_G06_1_H09_B	11	53	LMG 23078	*Streptomyces*	*albus*	*albus*
Z0016_RO_G10_1_H11_B	11	53	LMG 23078	*Streptomyces*	*albus*	*albus*
Z0016_RO_F11_2_G12_A	11	53	LMG 23078	*Streptomyces*	*albus*	*albus*
Z0016_RO_G03_1_H08_A	11	53	LMG 23078	*Streptomyces*	*albus*	*albus*
Z0016_RO_G01_1_H07_A	11	53	LMG 23078	*Streptomyces*	*albus*	*albus*
Z0016_RO_F12_2_G12_B	11	53	LMG 23078	*Streptomyces*	*albus*	*albus*
Z0016_LO_H05_2_H03_A	11	53	LMG 23078	*Streptomyces*	*albus*	*albus*

Z0016_RO_F05_2_G09_A	11	53	LMG 23078	*Streptomyces*	*albus*	*albus*
Z0016_LO_G03_1_H02_A	11	53	LMG 23078	*Streptomyces*	*albus*	*albus*
Z0016_RO_H01_2_H07_A	11	53	LMG 23078	*Streptomyces*	*albus*	*albus*
Z0016_LO_H01_2_H01_A	11	53	LMG 23078	*Streptomyces*	*albus*	*albus*
Z0016_RO_F08_2_G10_B	11	53	LMG 23078	*Streptomyces*	*albus*	*albus*
Z0019_RO_B12_2_E12_B	12	84	LMG 26041	*Tetragenococcus*	*osmophilus*	
Z0019_RO_B01_2_E07_A	12	84	LMG 26041	*Tetragenococcus*	*osmophilus*	
Z0019_LO_B07_2_E04_A	12	84	LMG 26041	*Tetragenococcus*	*osmophilus*	
Z0019_RO_B11_2_E12_A	12	84	LMG 26041	*Tetragenococcus*	*osmophilus*	
Z0019_RO_C01_1_F07_A	12	84	LMG 26041	*Tetragenococcus*	*osmophilus*	
Z0019_LO_C04_1_F02_B	12	84	LMG 26041	*Tetragenococcus*	*osmophilus*	
Z0019_RO_C10_1_F11_B	12	84	LMG 26041	*Tetragenococcus*	*osmophilus*	
Z0019_RO_C03_1_F08_A	12	84	LMG 26041	*Tetragenococcus*	*osmophilus*	
Z0019_LO_C03_1_F02_A	12	84	LMG 26041	*Tetragenococcus*	*osmophilus*	
Z0019_LO_C06_1_F03_B	12	84	LMG 26041	*Tetragenococcus*	*osmophilus*	
Z0019_LO_C05_1_F03_A	12	84	LMG 26041	*Tetragenococcus*	*osmophilus*	
Z0019_RO_C04_1_F08_B	12	84	LMG 26041	*Tetragenococcus*	*osmophilus*	
Z0019_LO_C08_1_F04_B	12	84	LMG 26041	*Tetragenococcus*	*osmophilus*	
Z0019_LO_A09_1_E05_A	12	84	LMG 26041	*Tetragenococcus*	*osmophilus*	
Z0019_LO_B01_2_E01_A	12	84	LMG 26041	*Tetragenococcus*	*osmophilus*	
Z0019_LO_B03_2_E02_A	12	84	LMG 26041	*Tetragenococcus*	*osmophilus*	
Z0019_LO_A12_1_E06_B	12	84	LMG 26041	*Tetragenococcus*	*osmophilus*	
Z0019_RO_B10_2_E11_B	12	84	LMG 26041	*Tetragenococcus*	*osmophilus*	
Z0019_RO_C05_1_F09_A	12	84	LMG 26041	*Tetragenococcus*	*osmophilus*	
Z0019_RO_B03_2_E08_A	12	84	LMG 26041	*Tetragenococcus*	*osmophilus*	
Z0019_RO_B02_2_E07_B	12	84	LMG 26041	*Tetragenococcus*	*osmophilus*	
Z0019_LO_B05_2_E03_A	12	84	LMG 26041	*Tetragenococcus*	*osmophilus*	
Z0019_RO_B07_2_E10_A	12	84	LMG 26041	*Tetragenococcus*	*osmophilus*	
Z0019_RO_B05_2_E09_A	12	84	LMG 26041	*Tetragenococcus*	*osmophilus*	
Z0019_RO_B06_2_E09_B	12	84	LMG 26041	*Tetragenococcus*	*osmophilus*	
Z0019_RO_B08_2_E10_B	12	84	LMG 26041	*Tetragenococcus*	*osmophilus*	
Z0019_RO_C11_1_F12_A	12	84	LMG 26041	*Tetragenococcus*	*osmophilus*	
Z0019_RO_C02_1_F07_B	12	84	LMG 26041	*Tetragenococcus*	*osmophilus*	
Z0021_RO_C02_1_F07_B	13	104	LMG 28216	*Formosa*	*algae*	
Z0021_LO_C05_1_F03_A	13	104	LMG 28216	*Formosa*	*algae*	
Z0021_RO_B12_2_E12_B	13	104	LMG 28216	*Formosa*	*algae*	
Z0021_RO_C03_1_F08_A	13	104	LMG 28216	*Formosa*	*algae*	
Z0021_RO_C06_1_F09_B	13	104	LMG 28216	*Formosa*	*algae*	
Z0021_RO_B10_2_E11_B	13	104	LMG 28216	*Formosa*	*algae*	
Z0021_RO_B09_2_E11_A	13	104	LMG 28216	*Formosa*	*algae*	
Z0021_LO_C03_1_F02_A	13	104	LMG 28216	*Formosa*	*algae*	
Z0021_RO_B07_2_E10_A	13	104	LMG 28216	*Formosa*	*algae*	
Z0021_LO_C01_1_F01_A	13	104	LMG 28216	*Formosa*	*algae*	
Z0021_LO_B08_2_E04_B	13	104	LMG 28216	*Formosa*	*algae*	
Z0021_RO_C04_1_F08_B	13	104	LMG 28216	*Formosa*	*algae*	
Z0021_RO_C07_1_F10_A	13	104	LMG 28216	*Formosa*	*algae*	
Z0021_LO_C08_1_F04_B	13	104	LMG 28216	*Formosa*	*algae*	
Z0021_RO_B01_2_E07_A	13	104	LMG 28216	*Formosa*	*algae*	
Z0021_LO_B04_2_E02_B	13	104	LMG 28216	*Formosa*	*algae*	
Z0021_RO_B03_2_E08_A	13	104	LMG 28216	*Formosa*	*algae*	

Z0021_RO_A12_1_E12_B	13	104	LMG 28216	*Formosa*	*algae*
Z0021_RO_B04_2_E08_B	13	104	LMG 28216	*Formosa*	*algae*
Z0021_LO_B10_2_E05_B	13	104	LMG 28216	*Formosa*	*algae*
Z0021_LO_B06_2_E03_B	13	104	LMG 28216	*Formosa*	*algae*
Z0021_RO_B02_2_E07_B	13	104	LMG 28216	*Formosa*	*algae*
Z0021_LO_B12_2_E06_B	13	104	LMG 28216	*Formosa*	*algae*
Z0021_RO_B06_2_E09_B	14	104	LMG 28216	*Formosa*	*algae*
Z0021_LO_B02_2_E01_B	14	104	LMG 28216	*Formosa*	*algae*
Z0021_LO_A12_1_E06_B	14	104	LMG 28216	*Formosa*	*algae*
Z0021_LO_B01_2_E01_A	14	104	LMG 28216	*Formosa*	*algae*
Z0021_LO_A11_1_E06_A	14	104	LMG 28216	*Formosa*	*algae*
Z0021_RO_A09_1_E11_A	15	106	LMG 28633	*Rhodococcus*	*degradans*
Z0021_LO_C07_1_F04_A	15	106	LMG 28633	*Rhodococcus*	*degradans*
Z0021_LO_C02_1_F01_B	15	106	LMG 28633	*Rhodococcus*	*degradans*
Z0021_LO_C06_1_F03_B	15	106	LMG 28633	*Rhodococcus*	*degradans*
Z0021_LO_D09_2_F05_A	15	106	LMG 28633	*Rhodococcus*	*degradans*
Z0021_LO_C09_1_F05_A	15	106	LMG 28633	*Rhodococcus*	*degradans*
Z0021_LO_D06_2_F03_B	15	106	LMG 28633	*Rhodococcus*	*degradans*
Z0021_RO_B08_2_E10_B	15	106	LMG 28633	*Rhodococcus*	*degradans*
Z0021_LO_E03_1_G02_A	15	106	LMG 28633	*Rhodococcus*	*degradans*
Z0021_LO_C11_1_F06_A	15	106	LMG 28633	*Rhodococcus*	*degradans*
Z0021_LO_D01_2_F01_A	15	106	LMG 28633	*Rhodococcus*	*degradans*
Z0021_LO_D10_2_F05_B	15	106	LMG 28633	*Rhodococcus*	*degradans*
Z0021_LO_C10_1_F05_B	15	106	LMG 28633	*Rhodococcus*	*degradans*
Z0021_LO_E08_1_G04_B	15	106	LMG 28633	*Rhodococcus*	*degradans*
Z0021_LO_D12_2_F06_B	15	106	LMG 28633	*Rhodococcus*	*degradans*
Z0021_LO_D05_2_F03_A	15	106	LMG 28633	*Rhodococcus*	*degradans*
Z0021_RO_D01_2_F07_A	15	106	LMG 28633	*Rhodococcus*	*degradans*
Z0021_LO_D08_2_F04_B	15	106	LMG 28633	*Rhodococcus*	*degradans*
Z0021_RO_D04_2_F08_B	15	106	LMG 28633	*Rhodococcus*	*degradans*
Z0021_LO_E07_1_G04_A	15	106	LMG 28633	*Rhodococcus*	*degradans*
Z0021_RO_C11_1_F12_A	15	106	LMG 28633	*Rhodococcus*	*degradans*
Z0021_LO_F06_2_G03_B	15	106	LMG 28633	*Rhodococcus*	*degradans*
Z0021_RB_H06_1_A10_B	15	106	LMG 28633	*Rhodococcus*	*degradans*
Z0021_RO_A02_1_E07_B	15	106	LMG 28633	*Rhodococcus*	*degradans*
Z0021_RB_H09_1_A08_A	15	106	LMG 28633	*Rhodococcus*	*degradans*
Z0021_RB_H07_1_A09_A	15	106	LMG 28633	*Rhodococcus*	*degradans*
Z0021_RO_C09_1_F11_A	16	106	LMG 28633	*Rhodococcus*	*degradans*
Z0021_RO_C12_1_F12_B	16	106	LMG 28633	*Rhodococcus*	*degradans*
Z0021_LO_B11_2_E06_A	17	106	LMG 28633	*Rhodococcus*	*degradans*
Z0021_LO_B07_2_E04_A	17	106	LMG 28633	*Rhodococcus*	*degradans*
Z0021_RO_A11_1_E12_A	17	106	LMG 28633	*Rhodococcus*	*degradans*
Z0021_RO_A10_1_E11_B	17	106	LMG 28633	*Rhodococcus*	*degradans*
Z0015_RO_E12_1_G12_B	18	43	LMG 22193	*Martelella*	*mediterranea*
Z0015_LO_D06_2_F03_B	18	43	LMG 22193	*Martelella*	*mediterranea*
Z0015_RO_E09_1_G11_A	18	43	LMG 22193	*Martelella*	*mediterranea*
Z0015_RO_D08_2_F10_B	18	43	LMG 22193	*Martelella*	*mediterranea*
Z0015_RO_D07_2_F10_A	18	43	LMG 22193	*Martelella*	*mediterranea*
Z0015_LO_C10_1_F05_B	18	43	LMG 22193	*Martelella*	*mediterranea*
Z0015_LO_C09_1_F05_A	18	43	LMG 22193	*Martelella*	*mediterranea*

Z0015_LO_C08_1_F04_B	18	43	LMG 22193	*Martelella*	*mediterranea*
Z0015_LO_D07_2_F04_A	18	43	LMG 22193	*Martelella*	*mediterranea*
Z0015_LO_D10_2_F05_B	18	43	LMG 22193	*Martelella*	*mediterranea*
Z0015_RO_E10_1_G11_B	18	43	LMG 22193	*Martelella*	*mediterranea*
Z0015_LO_D03_2_F02_A	18	43	LMG 22193	*Martelella*	*mediterranea*
Z0015_LO_D08_2_F04_B	18	43	LMG 22193	*Martelella*	*mediterranea*
Z0015_RO_E03_1_G08_A	18	43	LMG 22193	*Martelella*	*mediterranea*
Z0015_LO_C06_1_F03_B	18	43	LMG 22193	*Martelella*	*mediterranea*
Z0015_LO_C01_1_F01_A	18	43	LMG 22193	*Martelella*	*mediterranea*
Z0015_LO_B11_2_E06_A	18	43	LMG 22193	*Martelella*	*mediterranea*
Z0015_LO_B10_2_E05_B	18	43	LMG 22193	*Martelella*	*mediterranea*
Z0015_RO_C09_1_F11_A	18	43	LMG 22193	*Martelella*	*mediterranea*
Z0015_RO_C06_1_F09_B	18	43	LMG 22193	*Martelella*	*mediterranea*
Z0015_RO_E06_1_G09_B	18	43	LMG 22193	*Martelella*	*mediterranea*
Z0015_LO_C05_1_F03_A	18	43	LMG 22193	*Martelella*	*mediterranea*
Z0015_RO_E05_1_G09_A	18	43	LMG 22193	*Martelella*	*mediterranea*
Z0015_LO_C07_1_F04_A	18	43	LMG 22193	*Martelella*	*mediterranea*
Z0015_RO_D10_2_F11_B	18	43	LMG 22193	*Martelella*	*mediterranea*
Z0015_RO_D06_2_F09_B	18	43	LMG 22193	*Martelella*	*mediterranea*
Z0015_RO_C08_1_F10_B	18	43	LMG 22193	*Martelella*	*mediterranea*
Z0015_RO_C10_1_F11_B	18	43	LMG 22193	*Martelella*	*mediterranea*
Z0015_LO_C11_1_F06_A	18	43	LMG 22193	*Martelella*	*mediterranea*
Z0015_RO_F01_2_G07_A	18	43	LMG 22193	*Martelella*	*mediterranea*
Z0015_LO_D05_2_F03_A	18	43	LMG 22193	*Martelella*	*mediterranea*
Z0015_RO_F03_2_G08_A	18	43	LMG 22193	*Martelella*	*mediterranea*
Z0022_LB_F09_1_B02_A	19	108	LMG 29427	*Nakamurella*	*silvestris*
Z0022_LB_F10_1_B02_B	19	108	LMG 29427	*Nakamurella*	*silvestris*
Z0022_LB_F06_1_B04_B	19	108	LMG 29427	*Nakamurella*	*silvestris*
Z0022_RB_F12_1_B07_B	19	108	LMG 29427	*Nakamurella*	*silvestris*
Z0022_LB_F11_1_B01_A	19	108	LMG 29427	*Nakamurella*	*silvestris*
Z0022_LB_F12_1_B01_B	19	108	LMG 29427	*Nakamurella*	*silvestris*
Z0022_RB_F08_1_B09_B	19	108	LMG 29427	*Nakamurella*	*silvestris*
Z0022_RB_F09_1_B08_A	19	108	LMG 29427	*Nakamurella*	*silvestris*
Z0022_RB_F02_1_B12_B	19	108	LMG 29427	*Nakamurella*	*silvestris*
Z0022_LB_F07_1_B03_A	19	108	LMG 29427	*Nakamurella*	*silvestris*
Z0022_LB_F08_1_B03_B	19	108	LMG 29427	*Nakamurella*	*silvestris*
Z0022_LB_G03_2_A05_A	19	108	LMG 29427	*Nakamurella*	*silvestris*
Z0022_LB_G04_2_A05_B	19	108	LMG 29427	*Nakamurella*	*silvestris*
Z0022_RB_F03_1_B11_A	19	108	LMG 29427	*Nakamurella*	*silvestris*
Z0022_RB_F11_1_B07_A	19	108	LMG 29427	*Nakamurella*	*silvestris*
Z0022_LB_G01_2_A06_A	19	108	LMG 29427	*Nakamurella*	*silvestris*
Z0022_LB_G02_2_A06_B	19	108	LMG 29427	*Nakamurella*	*silvestris*
Z0022_RB_F01_1_B12_A	19	108	LMG 29427	*Nakamurella*	*silvestris*
Z0022_LB_F03_1_B05_A	19	108	LMG 29427	*Nakamurella*	*silvestris*
Z0022_RB_E12_2_B07_B	19	108	LMG 29427	*Nakamurella*	*silvestris*
Z0022_LB_F04_1_B05_B	19	108	LMG 29427	*Nakamurella*	*silvestris*
Z0022_RB_E10_2_B08_B	19	108	LMG 29427	*Nakamurella*	*silvestris*
Z0022_LB_F02_1_B06_B	19	108	LMG 29427	*Nakamurella*	*silvestris*
Z0022_RB_E11_2_B07_A	19	108	LMG 29427	*Nakamurella*	*silvestris*
Z0022_LB_F05_1_B04_A	19	108	LMG 29427	*Nakamurella*	*silvestris*

Z0022_RB_E09_2_B08_A	19	108	LMG 29427	*Nakamurella*	*silvestris*
Z0022_RB_F10_1_B08_B	19	108	LMG 29427	*Nakamurella*	*silvestris*
Z0022_RB_F06_1_B10_B	19	108	LMG 29427	*Nakamurella*	*silvestris*
Z0022_RB_F04_1_B11_B	19	108	LMG 29427	*Nakamurella*	*silvestris*
Z0022_RB_F05_1_B10_A	19	108	LMG 29427	*Nakamurella*	*silvestris*
Z0022_RB_F07_1_B09_A	19	108	LMG 29427	*Nakamurella*	*silvestris*
Z0022_RB_G01_2_A12_A	19	108	LMG 29427	*Nakamurella*	*silvestris*
Z0018_LO_D09_2_F05_A	20	74	LMG 24559	*Arcobacter*	*mytili*
Z0018_LO_D07_2_F04_A	20	74	LMG 24559	*Arcobacter*	*mytili*
Z0018_RO_D03_2_F08_A	20	74	LMG 24559	*Arcobacter*	*mytili*
Z0018_LO_D12_2_F06_B	20	74	LMG 24559	*Arcobacter*	*mytili*
Z0018_LO_D10_2_F05_B	20	74	LMG 24559	*Arcobacter*	*mytili*
Z0018_LO_D11_2_F06_A	20	74	LMG 24559	*Arcobacter*	*mytili*
Z0018_RO_C12_1_F12_B	20	74	LMG 24559	*Arcobacter*	*mytili*
Z0018_LO_D05_2_F03_A	20	74	LMG 24559	*Arcobacter*	*mytili*
Z0018_LO_D04_2_F02_B	20	74	LMG 24559	*Arcobacter*	*mytili*
Z0018_LO_D08_2_F04_B	20	74	LMG 24559	*Arcobacter*	*mytili*
Z0018_LO_D02_2_F01_B	20	74	LMG 24559	*Arcobacter*	*mytili*
Z0018_RO_F05_2_G09_A	20	74	LMG 24559	*Arcobacter*	*mytili*
Z0018_LO_F09_2_G05_A	20	74	LMG 24559	*Arcobacter*	*mytili*
Z0018_RO_F06_2_G09_B	20	74	LMG 24559	*Arcobacter*	*mytili*
Z0018_LO_F10_2_G05_B	20	74	LMG 24559	*Arcobacter*	*mytili*
Z0018_RO_E01_1_G07_A	20	74	LMG 24559	*Arcobacter*	*mytili*
Z0018_LO_E04_1_G02_B	20	74	LMG 24559	*Arcobacter*	*mytili*
Z0018_RO_E02_1_G07_B	20	74	LMG 24559	*Arcobacter*	*mytili*
Z0018_LO_F03_2_G02_A	20	74	LMG 24559	*Arcobacter*	*mytili*
Z0018_LO_F04_2_G02_B	20	74	LMG 24559	*Arcobacter*	*mytili*
Z0018_LO_E01_1_G01_A	20	74	LMG 24559	*Arcobacter*	*mytili*
Z0018_RO_F01_2_G07_A	20	74	LMG 24559	*Arcobacter*	*mytili*
Z0018_RO_E04_1_G08_B	20	74	LMG 24559	*Arcobacter*	*mytili*
Z0018_RO_F04_2_G08_B	20	74	LMG 24559	*Arcobacter*	*mytili*
Z0018_RO_E12_1_G12_B	21	74	LMG 24559	*Arcobacter*	*mytili*
Z0018_RO_E09_1_G11_A	21	74	LMG 24559	*Arcobacter*	*mytili*
Z0018_RO_E10_1_G11_B	21	74	LMG 24559	*Arcobacter*	*mytili*
Z0018_LO_E12_1_G06_B	21	74	LMG 24559	*Arcobacter*	*mytili*
Z0018_LO_E09_1_G05_A	21	74	LMG 24559	*Arcobacter*	*mytili*
Z0018_LO_E08_1_G04_B	21	74	LMG 24559	*Arcobacter*	*mytili*
Z0018_LO_E06_1_G03_B	21	74	LMG 24559	*Arcobacter*	*mytili*
Z0018_RO_E06_1_G09_B	21	74	LMG 24559	*Arcobacter*	*mytili*
Z0022_RB_A03_2_D11_A	22	107	LMG 2864	*Marinomonas*	*communis*
Z0022_LB_A01_2_D06_A	22	107	LMG 2864	*Marinomonas*	*communis*
Z0022_RB_A04_2_D11_B	22	107	LMG 2864	*Marinomonas*	*communis*
Z0022_LB_A08_2_D03_B	22	107	LMG 2864	*Marinomonas*	*communis*
Z0022_LB_A09_2_D02_A	22	107	LMG 2864	*Marinomonas*	*communis*
Z0022_LB_A02_2_D06_B	22	107	LMG 2864	*Marinomonas*	*communis*
Z0022_RO_A08_1_E10_B	22	107	LMG 2864	*Marinomonas*	*communis*
Z0022_LB_A03_2_D05_A	22	107	LMG 2864	*Marinomonas*	*communis*
Z0022_RO_A11_1_E12_A	22	107	LMG 2864	*Marinomonas*	*communis*
Z0022_RO_B01_2_E07_A	22	107	LMG 2864	*Marinomonas*	*communis*
Z0022_RO_B04_2_E08_B	22	107	LMG 2864	*Marinomonas*	*communis*

Z0022_RO_B03_2_E08_A	22	107	LMG 2864	*Marinomonas*	*communis*
Z0022_RO_C07_1_F10_A	22	107	LMG 2864	*Marinomonas*	*communis*
Z0022_RO_C08_1_F10_B	22	107	LMG 2864	*Marinomonas*	*communis*
Z0022_LO_B10_2_E05_B	22	107	LMG 2864	*Marinomonas*	*communis*
Z0022_LB_A04_2_D05_B	22	107	LMG 2864	*Marinomonas*	*communis*
Z0022_LO_B08_2_E04_B	22	107	LMG 2864	*Marinomonas*	*communis*
Z0022_RB_A06_2_D10_B	22	107	LMG 2864	*Marinomonas*	*communis*
Z0022_LO_B01_2_E01_A	22	107	LMG 2864	*Marinomonas*	*communis*
Z0022_RB_A05_2_D10_A	22	107	LMG 2864	*Marinomonas*	*communis*
Z0022_LB_A11_2_D01_A	22	107	LMG 2864	*Marinomonas*	*communis*
Z0022_RO_A09_1_E11_A	22	107	LMG 2864	*Marinomonas*	*communis*
Z0022_RB_A02_2_D12_B	22	107	LMG 2864	*Marinomonas*	*communis*
Z0022_LB_A05_2_D04_A	22	107	LMG 2864	*Marinomonas*	*communis*
Z0022_LB_A12_2_D01_B	22	107	LMG 2864	*Marinomonas*	*communis*
Z0022_LB_A10_2_D02_B	22	107	LMG 2864	*Marinomonas*	*communis*
Z0022_RB_A07_2_D09_A	22	107	LMG 2864	*Marinomonas*	*communis*
Z0022_LB_B01_1_D06_A	22	107	LMG 2864	*Marinomonas*	*communis*
Z0022_LB_B02_1_D06_B	22	107	LMG 2864	*Marinomonas*	*communis*
Z0022_RO_A10_1_E11_B	22	107	LMG 2864	*Marinomonas*	*communis*
Z0022_LB_A06_2_D04_B	22	107	LMG 2864	*Marinomonas*	*communis*
Z0022_LB_A07_2_D03_A	22	107	LMG 2864	*Marinomonas*	*communis*
Z0020_LB_H04_1_A05_B	23	92	LMG 26473	*Alishewanella*	*tabrizica*
Z0020_LB_H06_1_A04_B	23	92	LMG 26473	*Alishewanella*	*tabrizica*
Z0020_LB_H03_1_A05_A	23	92	LMG 26473	*Alishewanella*	*tabrizica*
Z0020_RB_H01_1_A12_A	23	92	LMG 26473	*Alishewanella*	*tabrizica*
Z0020_RB_G12_2_A07_B	23	92	LMG 26473	*Alishewanella*	*tabrizica*
Z0020_RO_A09_1_E11_A	23	92	LMG 26473	*Alishewanella*	*tabrizica*
Z0020_LO_A03_1_E02_A	23	92	LMG 26473	*Alishewanella*	*tabrizica*
Z0020_LO_A01_1_E01_A	23	92	LMG 26473	*Alishewanella*	*tabrizica*
Z0020_RB_H03_1_A11_A	23	92	LMG 26473	*Alishewanella*	*tabrizica*
Z0020_RB_H05_1_A10_A	23	92	LMG 26473	*Alishewanella*	*tabrizica*
Z0020_RO_A03_1_E08_A	23	92	LMG 26473	*Alishewanella*	*tabrizica*
Z0020_RB_H07_1_A09_A	23	92	LMG 26473	*Alishewanella*	*tabrizica*
Z0020_RO_A07_1_E10_A	23	92	LMG 26473	*Alishewanella*	*tabrizica*
Z0020_RB_H06_1_A10_B	23	92	LMG 26473	*Alishewanella*	*tabrizica*
Z0020_LO_A06_1_E03_B	23	92	LMG 26473	*Alishewanella*	*tabrizica*
Z0020_RO_A10_1_E11_B	23	92	LMG 26473	*Alishewanella*	*tabrizica*
Z0020_RO_A02_1_E07_B	23	92	LMG 26473	*Alishewanella*	*tabrizica*
Z0020_LB_H10_1_A02_B	23	92	LMG 26473	*Alishewanella*	*tabrizica*
Z0020_LB_H12_1_A01_B	23	92	LMG 26473	*Alishewanella*	*tabrizica*
Z0020_RB_H12_1_A07_B	23	92	LMG 26473	*Alishewanella*	*tabrizica*
Z0020_RO_A05_1_E09_A	23	92	LMG 26473	*Alishewanella*	*tabrizica*
Z0020_RB_H10_1_A08_B	23	92	LMG 26473	*Alishewanella*	*tabrizica*
Z0020_RO_A12_1_E12_B	23	92	LMG 26473	*Alishewanella*	*tabrizica*
Z0020_RB_H09_1_A08_A	23	92	LMG 26473	*Alishewanella*	*tabrizica*
Z0020_RB_H02_1_A12_B	23	92	LMG 26473	*Alishewanella*	*tabrizica*
Z0020_RB_H11_1_A07_A	23	92	LMG 26473	*Alishewanella*	*tabrizica*
Z0020_LO_A05_1_E03_A	24	92	LMG 26473	*Alishewanella*	*tabrizica*
Z0020_LO_A02_1_E01_B	24	92	LMG 26473	*Alishewanella*	*tabrizica*
Z0015_RO_A12_1_E12_B	25	39	LMG 21665	*Pigmentiphaga*	*kullae*

Z0015_RO_A11_1_E12_A	25	39	LMG 21665	*Pigmentiphaga*	*kullae*
Z0015_RO_A05_1_E09_A	25	39	LMG 21665	*Pigmentiphaga*	*kullae*
Z0015_LO_B01_2_E01_A	25	39	LMG 21665	*Pigmentiphaga*	*kullae*
Z0015_RO_A06_1_E09_B	25	39	LMG 21665	*Pigmentiphaga*	*kullae*
Z0015_RO_A09_1_E11_A	25	39	LMG 21665	*Pigmentiphaga*	*kullae*
Z0015_RO_A07_1_E10_A	25	39	LMG 21665	*Pigmentiphaga*	*kullae*
Z0015_RO_A03_1_E08_A	25	39	LMG 21665	*Pigmentiphaga*	*kullae*
Z0015_RO_B02_2_E07_B	25	39	LMG 21665	*Pigmentiphaga*	*kullae*
Z0015_RO_B01_2_E07_A	25	39	LMG 21665	*Pigmentiphaga*	*kullae*
Z0015_RO_B10_2_E11_B	25	39	LMG 21665	*Pigmentiphaga*	*kullae*
Z0015_LO_B07_2_E04_A	25	39	LMG 21665	*Pigmentiphaga*	*kullae*
Z0015_RO_B09_2_E11_A	25	39	LMG 21665	*Pigmentiphaga*	*kullae*
Z0015_RO_B11_2_E12_A	25	39	LMG 21665	*Pigmentiphaga*	*kullae*
Z0015_RO_B06_2_E09_B	25	39	LMG 21665	*Pigmentiphaga*	*kullae*
Z0015_LO_B06_2_E03_B	25	39	LMG 21665	*Pigmentiphaga*	*kullae*
Z0015_RO_B07_2_E10_A	25	39	LMG 21665	*Pigmentiphaga*	*kullae*
Z0015_RO_B04_2_E08_B	25	39	LMG 21665	*Pigmentiphaga*	*kullae*
Z0015_RO_A10_1_E11_B	25	39	LMG 21665	*Pigmentiphaga*	*kullae*
Z0015_LO_B03_2_E02_A	25	39	LMG 21665	*Pigmentiphaga*	*kullae*
Z0015_LO_B02_2_E01_B	25	39	LMG 21665	*Pigmentiphaga*	*kullae*
Z0015_LO_B04_2_E02_B	25	39	LMG 21665	*Pigmentiphaga*	*kullae*
Z0015_RO_A08_1_E10_B	26	39	LMG 21665	*Pigmentiphaga*	*kullae*
Z0015_RO_A04_1_E08_B	26	39	LMG 21665	*Pigmentiphaga*	*kullae*
Z0015_RO_B05_2_E09_A	27	39	LMG 21665	*Pigmentiphaga*	*kullae*
Z0015_LO_B05_2_E03_A	27	39	LMG 21665	*Pigmentiphaga*	*kullae*
Z0015_RO_B03_2_E08_A	27	39	LMG 21665	*Pigmentiphaga*	*kullae*
Z0015_RO_B08_2_E10_B	28	39	LMG 21665	*Pigmentiphaga*	*kullae*
Z0020_LO_C03_1_F02_A	29	93	LMG 26586	*Eilatimonas*	*milleporae*
Z0020_LO_B04_2_E02_B	29	93	LMG 26586	*Eilatimonas*	*milleporae*
Z0020_RO_B08_2_E10_B	29	93	LMG 26586	*Eilatimonas*	*milleporae*
Z0020_LO_A11_1_E06_A	29	93	LMG 26586	*Eilatimonas*	*milleporae*
Z0020_RO_B06_2_E09_B	29	93	LMG 26586	*Eilatimonas*	*milleporae*
Z0020_LO_A12_1_E06_B	29	93	LMG 26586	*Eilatimonas*	*milleporae*
Z0020_RO_C02_1_F07_B	29	93	LMG 26586	*Eilatimonas*	*milleporae*
Z0020_RO_C05_1_F09_A	29	93	LMG 26586	*Eilatimonas*	*milleporae*
Z0020_RO_B12_2_E12_B	29	93	LMG 26586	*Eilatimonas*	*milleporae*
Z0020_LO_C04_1_F02_B	29	93	LMG 26586	*Eilatimonas*	*milleporae*
Z0020_RO_B11_2_E12_A	29	93	LMG 26586	*Eilatimonas*	*milleporae*
Z0020_RO_B10_2_E11_B	29	93	LMG 26586	*Eilatimonas*	*milleporae*
Z0020_LO_B08_2_E04_B	29	93	LMG 26586	*Eilatimonas*	*milleporae*
Z0020_RO_B02_2_E07_B	29	93	LMG 26586	*Eilatimonas*	*milleporae*
Z0020_LO_A08_1_E04_B	29	93	LMG 26586	*Eilatimonas*	*milleporae*
Z0020_LO_C05_1_F03_A	29	93	LMG 26586	*Eilatimonas*	*milleporae*
Z0020_LO_B07_2_E04_A	29	93	LMG 26586	*Eilatimonas*	*milleporae*
Z0020_LO_C02_1_F01_B	29	93	LMG 26586	*Eilatimonas*	*milleporae*
Z0020_LO_A07_1_E04_A	29	93	LMG 26586	*Eilatimonas*	*milleporae*
Z0020_RO_B04_2_E08_B	29	93	LMG 26586	*Eilatimonas*	*milleporae*
Z0020_RO_B03_2_E08_A	29	93	LMG 26586	*Eilatimonas*	*milleporae*
Z0020_RO_B09_2_E11_A	29	93	LMG 26586	*Eilatimonas*	*milleporae*
Z0020_LO_B05_2_E03_A	29	93	LMG 26586	*Eilatimonas*	*milleporae*

Z0020_RO_C03_1_F08_A	29	93	LMG 26586	*Eilatimonas*	*milleporae*
Z0020_RO_C08_1_F10_B	29	93	LMG 26586	*Eilatimonas*	*milleporae*
Z0020_LO_B11_2_E06_A	29	93	LMG 26586	*Eilatimonas*	*milleporae*
Z0020_LO_C01_1_F01_A	29	93	LMG 26586	*Eilatimonas*	*milleporae*
Z0020_LO_B10_2_E05_B	29	93	LMG 26586	*Eilatimonas*	*milleporae*
Z0016_LB_G01_2_A06_A	30	47	LMG 22585	*Gramella*	*echinicola*
Z0016_LB_F04_1_B05_B	30	47	LMG 22585	*Gramella*	*echinicola*
Z0016_RB_E12_2_B07_B	30	47	LMG 22585	*Gramella*	*echinicola*
Z0016_LB_F03_1_B05_A	30	47	LMG 22585	*Gramella*	*echinicola*
Z0016_LB_F12_1_B01_B	30	47	LMG 22585	*Gramella*	*echinicola*
Z0016_LB_F02_1_B06_B	30	47	LMG 22585	*Gramella*	*echinicola*
Z0016_RB_E11_2_B07_A	30	47	LMG 22585	*Gramella*	*echinicola*
Z0016_RB_E10_2_B08_B	30	47	LMG 22585	*Gramella*	*echinicola*
Z0016_LB_E11_2_B01_A	30	47	LMG 22585	*Gramella*	*echinicola*
Z0016_RB_F07_1_B09_A	30	47	LMG 22585	*Gramella*	*echinicola*
Z0016_LB_F10_1_B02_B	30	47	LMG 22585	*Gramella*	*echinicola*
Z0016_RB_F06_1_B10_B	30	47	LMG 22585	*Gramella*	*echinicola*
Z0016_LB_F01_1_B06_A	30	47	LMG 22585	*Gramella*	*echinicola*
Z0016_RB_F04_1_B11_B	30	47	LMG 22585	*Gramella*	*echinicola*
Z0016_RB_E09_2_B08_A	30	47	LMG 22585	*Gramella*	*echinicola*
Z0016_RB_F02_1_B12_B	30	47	LMG 22585	*Gramella*	*echinicola*
Z0016_RB_F03_1_B11_A	30	47	LMG 22585	*Gramella*	*echinicola*
Z0016_LB_E12_2_B01_B	30	47	LMG 22585	*Gramella*	*echinicola*
Z0016_RB_F01_1_B12_A	30	47	LMG 22585	*Gramella*	*echinicola*
Z0016_LB_F07_1_B03_A	30	47	LMG 22585	*Gramella*	*echinicola*
Z0016_RB_F11_1_B07_A	30	47	LMG 22585	*Gramella*	*echinicola*
Z0016_LB_G02_2_A06_B	30	47	LMG 22585	*Gramella*	*echinicola*
Z0016_RB_F05_1_B10_A	30	47	LMG 22585	*Gramella*	*echinicola*
Z0016_LB_E10_2_B02_B	30	47	LMG 22585	*Gramella*	*echinicola*
Z0016_LB_F06_1_B04_B	30	47	LMG 22585	*Gramella*	*echinicola*
Z0016_RB_F10_1_B08_B	30	47	LMG 22585	*Gramella*	*echinicola*
Z0016_RB_F09_1_B08_A	30	47	LMG 22585	*Gramella*	*echinicola*
Z0016_RB_F08_1_B09_B	30	47	LMG 22585	*Gramella*	*echinicola*
Z0016_LB_F11_1_B01_A	30	47	LMG 22585	*Gramella*	*echinicola*
Z0016_LB_F08_1_B03_B	30	47	LMG 22585	*Gramella*	*echinicola*
Z0016_LB_F09_1_B02_A	30	47	LMG 22585	*Gramella*	*echinicola*
Z0016_LB_F05_1_B04_A	30	47	LMG 22585	*Gramella*	*echinicola*
Z0021_RB_A11_2_D07_A	31	98	LMG 27212	*Pectinatus*	*sp.*
Z0021_RB_A10_2_D08_B	31	98	LMG 27212	*Pectinatus*	*sp.*
Z0021_RB_A06_2_D10_B	31	98	LMG 27212	*Pectinatus*	*sp.*
Z0021_LB_B09_1_D02_A	31	98	LMG 27212	*Pectinatus*	*sp.*
Z0021_LB_B08_1_D03_B	31	98	LMG 27212	*Pectinatus*	*sp.*
Z0021_RB_A09_2_D08_A	31	98	LMG 27212	*Pectinatus*	*sp.*
Z0021_LB_B07_1_D03_A	31	98	LMG 27212	*Pectinatus*	*sp.*
Z0021_RB_A08_2_D09_B	31	98	LMG 27212	*Pectinatus*	*sp.*
Z0021_LB_B06_1_D04_B	31	98	LMG 27212	*Pectinatus*	*sp.*
Z0021_RB_A07_2_D09_A	31	98	LMG 27212	*Pectinatus*	*sp.*
Z0021_LB_B05_1_D04_A	31	98	LMG 27212	*Pectinatus*	*sp.*
Z0021_LB_B04_1_D05_B	31	98	LMG 27212	*Pectinatus*	*sp.*
Z0021_RB_A12_2_D07_B	31	98	LMG 27212	*Pectinatus*	*sp.*

Z0021_LB_B10_1_D02_B	31	98	LMG 27212	*Pectinatus*	*sp.*
Z0021_LB_B11_1_D01_A	31	98	LMG 27212	*Pectinatus*	*sp.*
Z0021_RB_B02_1_D12_B	31	98	LMG 27212	*Pectinatus*	*sp.*
Z0021_RB_B04_1_D11_B	31	98	LMG 27212	*Pectinatus*	*sp.*
Z0021_RB_B03_1_D11_A	31	98	LMG 27212	*Pectinatus*	*sp.*
Z0021_LB_B12_1_D01_B	31	98	LMG 27212	*Pectinatus*	*sp.*
Z0021_RB_B05_1_D10_A	31	98	LMG 27212	*Pectinatus*	*sp.*
Z0021_LB_C05_2_C04_A	31	98	LMG 27212	*Pectinatus*	*sp.*
Z0021_RB_B07_1_D09_A	31	98	LMG 27212	*Pectinatus*	*sp.*
Z0021_LB_C02_2_C06_B	31	98	LMG 27212	*Pectinatus*	*sp.*
Z0021_LB_C01_2_C06_A	31	98	LMG 27212	*Pectinatus*	*sp.*
Z0021_RB_B06_1_D10_B	32	98	LMG 27212	*Pectinatus*	*sp.*
Z0021_LB_C04_2_C05_B	32	98	LMG 27212	*Pectinatus*	*sp.*
Z0021_RB_B01_1_D12_A	32	98	LMG 27212	*Pectinatus*	*sp.*
Z0021_LB_C03_2_C05_A	32	98	LMG 27212	*Pectinatus*	*sp.*
Z0021_LB_B03_1_D05_A	32	98	LMG 27212	*Pectinatus*	*sp.*
Z0021_LB_A12_2_D01_B	32	98	LMG 27212	*Pectinatus*	*sp.*
Z0021_LB_B02_1_D06_B	32	98	LMG 27212	*Pectinatus*	*sp.*
Z0021_LB_B01_1_D06_A	32	98	LMG 27212	*Pectinatus*	*sp.*
Z0015_RO_D09_2_F11_A	33	40	LMG 21817	*Woodsholea*	*maritima*
Z0015_LO_D01_2_F01_A	33	40	LMG 21817	*Woodsholea*	*maritima*
Z0015_RO_B12_2_E12_B	33	40	LMG 21817	*Woodsholea*	*maritima*
Z0015_LO_B09_2_E05_A	33	40	LMG 21817	*Woodsholea*	*maritima*
Z0015_RO_D05_2_F09_A	33	40	LMG 21817	*Woodsholea*	*maritima*
Z0015_RO_D04_2_F08_B	33	40	LMG 21817	*Woodsholea*	*maritima*
Z0015_LO_C12_1_F06_B	33	40	LMG 21817	*Woodsholea*	*maritima*
Z0015_RO_D02_2_F07_B	33	40	LMG 21817	*Woodsholea*	*maritima*
Z0015_RO_D03_2_F08_A	33	40	LMG 21817	*Woodsholea*	*maritima*
Z0015_RO_C05_1_F09_A	33	40	LMG 21817	*Woodsholea*	*maritima*
Z0015_LO_D02_2_F01_B	33	40	LMG 21817	*Woodsholea*	*maritima*
Z0015_RO_C01_1_F07_A	33	40	LMG 21817	*Woodsholea*	*maritima*
Z0015_LO_B08_2_E04_B	33	40	LMG 21817	*Woodsholea*	*maritima*
Z0015_RO_E02_1_G07_B	34	40	LMG 21817	*Woodsholea*	*maritima*
Z0015_RO_C07_1_F10_A	35	40	LMG 21817	*Woodsholea*	*maritima*
Z0015_RO_C03_1_F08_A	35	40	LMG 21817	*Woodsholea*	*maritima*
Z0015_RO_E04_1_G08_B	35	40	LMG 21817	*Woodsholea*	*maritima*
Z0015_LO_D11_2_F06_A	35	40	LMG 21817	*Woodsholea*	*maritima*
Z0015_LO_D09_2_F05_A	35	40	LMG 21817	*Woodsholea*	*maritima*
Z0015_LO_C04_1_F02_B	35	40	LMG 21817	*Woodsholea*	*maritima*
Z0015_RO_C12_1_F12_B	35	40	LMG 21817	*Woodsholea*	*maritima*
Z0015_RO_D01_2_F07_A	35	40	LMG 21817	*Woodsholea*	*maritima*
Z0015_LO_B12_2_E06_B	35	40	LMG 21817	*Woodsholea*	*maritima*
Z0015_LO_C02_1_F01_B	35	40	LMG 21817	*Woodsholea*	*maritima*
Z0015_RO_C02_1_F07_B	35	40	LMG 21817	*Woodsholea*	*maritima*
Z0015_RO_C04_1_F08_B	35	40	LMG 21817	*Woodsholea*	*maritima*
Z0015_LO_C03_1_F02_A	35	40	LMG 21817	*Woodsholea*	*maritima*
Z0015_RO_C11_1_F12_A	35	40	LMG 21817	*Woodsholea*	*maritima*
Z0015_LO_D04_2_F02_B	35	40	LMG 21817	*Woodsholea*	*maritima*
Z0015_RO_D12_2_F12_B	35	40	LMG 21817	*Woodsholea*	*maritima*
Z0015_RO_E01_1_G07_A	35	40	LMG 21817	*Woodsholea*	*maritima*

Z0015_RO_D11_2_F12_A	35	40	LMG 21817	*Woodsholea*	*maritima*	
Z0024_RB_G12_2_A07_B	36	4	LMG 6907	*Lactobacillus*	*plantarum*	*plantarum*
Z0024_RB_H08_1_A09_B	36	4	LMG 6907	*Lactobacillus*	*plantarum*	*plantarum*
Z0024_LO_A10_1_E05_B	36	4	LMG 6907	*Lactobacillus*	*plantarum*	*plantarum*
Z0024_RB_H02_1_A12_B	36	4	LMG 6907	*Lactobacillus*	*plantarum*	*plantarum*
Z0024_LO_A01_1_E01_A	36	4	LMG 6907	*Lactobacillus*	*plantarum*	*plantarum*
Z0024_RB_H01_1_A12_A	36	4	LMG 6907	*Lactobacillus*	*plantarum*	*plantarum*
Z0024_RB_H07_1_A09_A	36	4	LMG 6907	*Lactobacillus*	*plantarum*	*plantarum*
Z0024_LB_H12_1_A01_B	36	4	LMG 6907	*Lactobacillus*	*plantarum*	*plantarum*
Z0024_LO_A11_1_E06_A	36	4	LMG 6907	*Lactobacillus*	*plantarum*	*plantarum*
Z0024_RB_H03_1_A11_A	36	4	LMG 6907	*Lactobacillus*	*plantarum*	*plantarum*
Z0024_LO_A03_1_E02_A	36	4	LMG 6907	*Lactobacillus*	*plantarum*	*plantarum*
Z0024_LO_A02_1_E01_B	36	4	LMG 6907	*Lactobacillus*	*plantarum*	*plantarum*
Z0024_LB_H08_1_A03_B	36	4	LMG 6907	*Lactobacillus*	*plantarum*	*plantarum*
Z0024_LB_H10_1_A02_B	36	4	LMG 6907	*Lactobacillus*	*plantarum*	*plantarum*
Z0024_RB_G08_2_A09_B	36	4	LMG 6907	*Lactobacillus*	*plantarum*	*plantarum*
Z0024_LB_H06_1_A04_B	36	4	LMG 6907	*Lactobacillus*	*plantarum*	*plantarum*
Z0024_RB_G09_2_A08_A	36	4	LMG 6907	*Lactobacillus*	*plantarum*	*plantarum*
Z0024_LB_H05_1_A04_A	36	4	LMG 6907	*Lactobacillus*	*plantarum*	*plantarum*
Z0024_RB_G11_2_A07_A	36	4	LMG 6907	*Lactobacillus*	*plantarum*	*plantarum*
Z0024_LB_H07_1_A03_A	36	4	LMG 6907	*Lactobacillus*	*plantarum*	*plantarum*
Z0024_LO_A04_1_E02_B	36	4	LMG 6907	*Lactobacillus*	*plantarum*	*plantarum*
Z0024_LO_A07_1_E04_A	37	4	LMG 6907	*Lactobacillus*	*plantarum*	*plantarum*
Z0024_LO_A06_1_E03_B	37	4	LMG 6907	*Lactobacillus*	*plantarum*	*plantarum*
Z0024_LO_A05_1_E03_A	37	4	LMG 6907	*Lactobacillus*	*plantarum*	*plantarum*
Z0024_LB_H11_1_A01_A	37	4	LMG 6907	*Lactobacillus*	*plantarum*	*plantarum*
Z0024_LB_H09_1_A02_A	37	4	LMG 6907	*Lactobacillus*	*plantarum*	*plantarum*
Z0024_RB_H06_1_A10_B	37	4	LMG 6907	*Lactobacillus*	*plantarum*	*plantarum*
Z0024_RB_H04_1_A11_B	37	4	LMG 6907	*Lactobacillus*	*plantarum*	*plantarum*
Z0024_RB_G10_2_A08_B	37	4	LMG 6907	*Lactobacillus*	*plantarum*	*plantarum*
Z0024_RB_H05_1_A10_A	37	4	LMG 6907	*Lactobacillus*	*plantarum*	*plantarum*
Z0024_LO_A09_1_E05_A	37	4	LMG 6907	*Lactobacillus*	*plantarum*	*plantarum*
Z0024_LO_A08_1_E04_B	37	4	LMG 6907	*Lactobacillus*	*plantarum*	*plantarum*
Z0014_LO_A06_1_E03_B	38	4	LMG 18404	*Lactobacillus*	*plantarum*	
Z0014_LB_H11_1_A01_A	38	4	LMG 18404	*Lactobacillus*	*plantarum*	
Z0014_RO_A11_1_E12_A	38	4	LMG 18404	*Lactobacillus*	*plantarum*	
Z0014_LO_A01_1_E01_A	38	4	LMG 18404	*Lactobacillus*	*plantarum*	
Z0014_LB_H12_1_A01_B	38	4	LMG 18404	*Lactobacillus*	*plantarum*	
Z0014_RO_B03_2_E08_A	38	4	LMG 18404	*Lactobacillus*	*plantarum*	
Z0014_RO_B07_2_E10_A	38	4	LMG 18404	*Lactobacillus*	*plantarum*	
Z0014_LO_A03_1_E02_A	38	4	LMG 18404	*Lactobacillus*	*plantarum*	
Z0014_RO_A10_1_E11_B	38	4	LMG 18404	*Lactobacillus*	*plantarum*	
Z0014_RO_B02_2_E07_B	38	4	LMG 18404	*Lactobacillus*	*plantarum*	
Z0014_LO_A02_1_E01_B	38	4	LMG 18404	*Lactobacillus*	*plantarum*	
Z0014_RO_A08_1_E10_B	38	4	LMG 18404	*Lactobacillus*	*plantarum*	
Z0014_LB_H08_1_A03_B	38	4	LMG 18404	*Lactobacillus*	*plantarum*	
Z0014_RO_B01_2_E07_A	38	4	LMG 18404	*Lactobacillus*	*plantarum*	
Z0014_RO_A12_1_E12_B	38	4	LMG 18404	*Lactobacillus*	*plantarum*	
Z0014_LO_A04_1_E02_B	38	4	LMG 18404	*Lactobacillus*	*plantarum*	
Z0014_LO_A05_1_E03_A	38	4	LMG 18404	*Lactobacillus*	*plantarum*	

Z0014_LO_A07_1_E04_A	38	4	LMG 18404	*Lactobacillus*	*plantarum*	
Z0014_LB_H07_1_A03_A	38	4	LMG 18404	*Lactobacillus*	*plantarum*	
Z0014_RO_B05_2_E09_A	38	4	LMG 18404	*Lactobacillus*	*plantarum*	
Z0014_RO_B08_2_E10_B	38	4	LMG 18404	*Lactobacillus*	*plantarum*	
Z0014_RO_B06_2_E09_B	38	4	LMG 18404	*Lactobacillus*	*plantarum*	
Z0014_LO_A08_1_E04_B	38	4	LMG 18404	*Lactobacillus*	*plantarum*	
Z0014_RO_B04_2_E08_B	38	4	LMG 18404	*Lactobacillus*	*plantarum*	
Z0014_LB_H06_1_A04_B	38	4	LMG 18404	*Lactobacillus*	*plantarum*	
Z0014_RO_A09_1_E11_A	38	4	LMG 18404	*Lactobacillus*	*plantarum*	
Z0014_LB_H09_1_A02_A	38	4	LMG 18404	*Lactobacillus*	*plantarum*	
Z0014_LB_H10_1_A02_B	38	4	LMG 18404	*Lactobacillus*	*plantarum*	
Z0025_RO_B11_2_E12_A	39	139	LMG 9205	*Lactobacillus*	*plantarum*	*argentoratensis*
Z0025_RO_B10_2_E11_B	39	139	LMG 9205	*Lactobacillus*	*plantarum*	*argentoratensis*
Z0025_LO_C07_1_F04_A	39	139	LMG 9205	*Lactobacillus*	*plantarum*	*argentoratensis*
Z0025_RO_B12_2_E12_B	39	139	LMG 9205	*Lactobacillus*	*plantarum*	*argentoratensis*
Z0025_RO_C01_1_F07_A	39	139	LMG 9205	*Lactobacillus*	*plantarum*	*argentoratensis*
Z0025_RO_C04_1_F08_B	39	139	LMG 9205	*Lactobacillus*	*plantarum*	*argentoratensis*
Z0025_RO_C09_1_F11_A	39	139	LMG 9205	*Lactobacillus*	*plantarum*	*argentoratensis*
Z0025_RO_C02_1_F07_B	39	139	LMG 9205	*Lactobacillus*	*plantarum*	*argentoratensis*
Z0025_RO_C07_1_F10_A	39	139	LMG 9205	*Lactobacillus*	*plantarum*	*argentoratensis*
Z0025_RO_C08_1_F10_B	39	139	LMG 9205	*Lactobacillus*	*plantarum*	*argentoratensis*
Z0025_LO_D07_2_F04_A	39	139	LMG 9205	*Lactobacillus*	*plantarum*	*argentoratensis*
Z0025_RO_C06_1_F09_B	39	139	LMG 9205	*Lactobacillus*	*plantarum*	*argentoratensis*
Z0025_RO_B05_2_E09_A	39	139	LMG 9205	*Lactobacillus*	*plantarum*	*argentoratensis*
Z0025_RO_B06_2_E09_B	39	139	LMG 9205	*Lactobacillus*	*plantarum*	*argentoratensis*
Z0025_LO_D02_2_F01_B	39	139	LMG 9205	*Lactobacillus*	*plantarum*	*argentoratensis*
Z0025_RO_B09_2_E11_A	39	139	LMG 9205	*Lactobacillus*	*plantarum*	*argentoratensis*
Z0025_LO_C03_1_F02_A	39	139	LMG 9205	*Lactobacillus*	*plantarum*	*argentoratensis*
Z0025_RO_B08_2_E10_B	39	139	LMG 9205	*Lactobacillus*	*plantarum*	*argentoratensis*
Z0025_LO_C02_1_F01_B	39	139	LMG 9205	*Lactobacillus*	*plantarum*	*argentoratensis*
Z0025_LO_C12_1_F06_B	39	139	LMG 9205	*Lactobacillus*	*plantarum*	*argentoratensis*
Z0025_RO_C11_1_F12_A	39	139	LMG 9205	*Lactobacillus*	*plantarum*	*argentoratensis*
Z0025_RO_C03_1_F08_A	39	139	LMG 9205	*Lactobacillus*	*plantarum*	*argentoratensis*
Z0025_LO_D11_2_F06_A	39	139	LMG 9205	*Lactobacillus*	*plantarum*	*argentoratensis*
Z0025_LO_D12_2_F06_B	39	139	LMG 9205	*Lactobacillus*	*plantarum*	*argentoratensis*
Z0025_RO_B07_2_E10_A	39	139	LMG 9205	*Lactobacillus*	*plantarum*	*argentoratensis*
Z0025_LO_D03_2_F02_A	39	139	LMG 9205	*Lactobacillus*	*plantarum*	*argentoratensis*
Z0025_LO_C05_1_F03_A	39	139	LMG 9205	*Lactobacillus*	*plantarum*	*argentoratensis*
Z0025_LO_D09_2_F05_A	39	139	LMG 9205	*Lactobacillus*	*plantarum*	*argentoratensis*
Z0014_RB_A06_2_D10_B	40	4	LMG 18021	*Lactobacillus*	*plantarum*	
Z0014_RB_A05_2_D10_A	40	4	LMG 18021	*Lactobacillus*	*plantarum*	
Z0014_RB_A03_2_D11_A	40	4	LMG 18021	*Lactobacillus*	*plantarum*	
Z0014_RB_A07_2_D09_A	40	4	LMG 18021	*Lactobacillus*	*plantarum*	
Z0014_LB_A03_2_D05_A	40	4	LMG 18021	*Lactobacillus*	*plantarum*	
Z0014_RB_A08_2_D09_B	40	4	LMG 18021	*Lactobacillus*	*plantarum*	
Z0014_LB_A04_2_D05_B	40	4	LMG 18021	*Lactobacillus*	*plantarum*	
Z0014_RB_B06_1_D10_B	40	4	LMG 18021	*Lactobacillus*	*plantarum*	
Z0014_LB_B02_1_D06_B	40	4	LMG 18021	*Lactobacillus*	*plantarum*	
Z0014_RB_B04_1_D11_B	40	4	LMG 18021	*Lactobacillus*	*plantarum*	
Z0014_RB_B02_1_D12_B	40	4	LMG 18021	*Lactobacillus*	*plantarum*	

Z0014_RB_A10_2_D08_B	40	4	LMG 18021	*Lactobacillus*	*plantarum*
Z0014_RB_A09_2_D08_A	40	4	LMG 18021	*Lactobacillus*	*plantarum*
Z0014_LB_A06_2_D04_B	40	4	LMG 18021	*Lactobacillus*	*plantarum*
Z0014_LB_A05_2_D04_A	40	4	LMG 18021	*Lactobacillus*	*plantarum*
Z0014_RB_B05_1_D10_A	40	4	LMG 18021	*Lactobacillus*	*plantarum*
Z0014_LB_A10_2_D02_B	40	4	LMG 18021	*Lactobacillus*	*plantarum*
Z0014_LB_A12_2_D01_B	40	4	LMG 18021	*Lactobacillus*	*plantarum*
Z0014_RB_B03_1_D11_A	40	4	LMG 18021	*Lactobacillus*	*plantarum*
Z0014_LB_B01_1_D06_A	40	4	LMG 18021	*Lactobacillus*	*plantarum*
Z0014_RB_B01_1_D12_A	40	4	LMG 18021	*Lactobacillus*	*plantarum*
Z0014_LB_A09_2_D02_A	40	4	LMG 18021	*Lactobacillus*	*plantarum*
Z0014_RB_A11_2_D07_A	40	4	LMG 18021	*Lactobacillus*	*plantarum*
Z0014_RB_A12_2_D07_B	40	4	LMG 18021	*Lactobacillus*	*plantarum*
Z0014_RB_A04_2_D11_B	40	4	LMG 18021	*Lactobacillus*	*plantarum*
Z0014_LB_A02_2_D06_B	40	4	LMG 18021	*Lactobacillus*	*plantarum*
Z0014_LB_A11_2_D01_A	40	4	LMG 18021	*Lactobacillus*	*plantarum*
Z0014_RB_A01_2_D12_A	40	4	LMG 18021	*Lactobacillus*	*plantarum*
Z0014_LO_H12_2_H06_B	40	4	LMG 18021	*Lactobacillus*	*plantarum*
Z0014_RB_A02_2_D12_B	40	4	LMG 18021	*Lactobacillus*	*plantarum*
Z0014_RB_G06_2_A10_B	41	22	LMG 18398	*Lactobacillus*	*paraplantarum*
Z0014_LB_G05_2_A04_A	41	22	LMG 18398	*Lactobacillus*	*paraplantarum*
Z0014_RB_G03_2_A11_A	41	22	LMG 18398	*Lactobacillus*	*paraplantarum*
Z0014_RB_G05_2_A10_A	41	22	LMG 18398	*Lactobacillus*	*paraplantarum*
Z0014_RB_G07_2_A09_A	41	22	LMG 18398	*Lactobacillus*	*paraplantarum*
Z0014_RB_G08_2_A09_B	41	22	LMG 18398	*Lactobacillus*	*paraplantarum*
Z0014_RB_G04_2_A11_B	41	22	LMG 18398	*Lactobacillus*	*paraplantarum*
Z0014_LB_G04_2_A05_B	41	22	LMG 18398	*Lactobacillus*	*paraplantarum*
Z0014_LB_G08_2_A03_B	41	22	LMG 18398	*Lactobacillus*	*paraplantarum*
Z0014_LB_G02_2_A06_B	41	22	LMG 18398	*Lactobacillus*	*paraplantarum*
Z0014_LB_G03_2_A05_A	41	22	LMG 18398	*Lactobacillus*	*paraplantarum*
Z0014_LB_G01_2_A06_A	41	22	LMG 18398	*Lactobacillus*	*paraplantarum*
Z0014_LB_F12_1_B01_B	41	22	LMG 18398	*Lactobacillus*	*paraplantarum*
Z0014_LB_G07_2_A03_A	41	22	LMG 18398	*Lactobacillus*	*paraplantarum*
Z0014_LB_F11_1_B01_A	41	22	LMG 18398	*Lactobacillus*	*paraplantarum*
Z0014_RB_F06_1_B10_B	41	22	LMG 18398	*Lactobacillus*	*paraplantarum*
Z0014_RB_F12_1_B07_B	41	22	LMG 18398	*Lactobacillus*	*paraplantarum*
Z0014_RB_F08_1_B09_B	41	22	LMG 18398	*Lactobacillus*	*paraplantarum*
Z0014_RB_F07_1_B09_A	41	22	LMG 18398	*Lactobacillus*	*paraplantarum*
Z0014_LB_F04_1_B05_B	41	22	LMG 18398	*Lactobacillus*	*paraplantarum*
Z0014_RB_G02_2_A12_B	41	22	LMG 18398	*Lactobacillus*	*paraplantarum*
Z0014_LB_F09_1_B02_A	41	22	LMG 18398	*Lactobacillus*	*paraplantarum*
Z0014_LB_F08_1_B03_B	41	22	LMG 18398	*Lactobacillus*	*paraplantarum*
Z0014_LB_F10_1_B02_B	41	22	LMG 18398	*Lactobacillus*	*paraplantarum*
Z0014_RB_F10_1_B08_B	41	22	LMG 18398	*Lactobacillus*	*paraplantarum*
Z0014_RB_G01_2_A12_A	41	22	LMG 18398	*Lactobacillus*	*paraplantarum*
Z0014_RB_F11_1_B07_A	41	22	LMG 18398	*Lactobacillus*	*paraplantarum*
Z0014_LB_F05_1_B04_A	41	22	LMG 18398	*Lactobacillus*	*paraplantarum*
Z0014_LB_F07_1_B03_A	41	22	LMG 18398	*Lactobacillus*	*paraplantarum*
Z0014_RB_F09_1_B08_A	41	22	LMG 18398	*Lactobacillus*	*paraplantarum*
Z0014_LB_F06_1_B04_B	41	22	LMG 18398	*Lactobacillus*	*paraplantarum*

Z0014_LB_G06_2_A04_B	41	22	LMG 18398	*Lactobacillus*	*paraplantarum*
Z0013_RO_B02_2_E07_B	42	16	LMG 1346	*Marinilabilia*	*salmonicolor*
Z0013_RO_B03_2_E08_A	42	16	LMG 1346	*Marinilabilia*	*salmonicolor*
Z0013_RO_B04_2_E08_B	42	16	LMG 1346	*Marinilabilia*	*salmonicolor*
Z0013_RB_A10_2_D08_B	42	16	LMG 1346	*Marinilabilia*	*salmonicolor*
Z0013_LO_B04_2_E02_B	42	16	LMG 1346	*Marinilabilia*	*salmonicolor*
Z0013_LO_B03_2_E02_A	42	16	LMG 1346	*Marinilabilia*	*salmonicolor*
Z0013_LB_A12_2_D01_B	42	16	LMG 1346	*Marinilabilia*	*salmonicolor*
Z0013_LB_B07_1_D03_A	42	16	LMG 1346	*Marinilabilia*	*salmonicolor*
Z0013_RO_B05_2_E09_A	42	16	LMG 1346	*Marinilabilia*	*salmonicolor*
Z0013_RB_B04_1_D11_B	42	16	LMG 1346	*Marinilabilia*	*salmonicolor*
Z0013_RO_B06_2_E09_B	42	16	LMG 1346	*Marinilabilia*	*salmonicolor*
Z0013_LB_B02_1_D06_B	42	16	LMG 1346	*Marinilabilia*	*salmonicolor*
Z0013_RB_B05_1_D10_A	42	16	LMG 1346	*Marinilabilia*	*salmonicolor*
Z0013_LB_B03_1_D05_A	42	16	LMG 1346	*Marinilabilia*	*salmonicolor*
Z0013_RO_B01_2_E07_A	42	16	LMG 1346	*Marinilabilia*	*salmonicolor*
Z0013_LO_B08_2_E04_B	42	16	LMG 1346	*Marinilabilia*	*salmonicolor*
Z0013_LB_B08_1_D03_B	42	16	LMG 1346	*Marinilabilia*	*salmonicolor*
Z0013_RO_B07_2_E10_A	42	16	LMG 1346	*Marinilabilia*	*salmonicolor*
Z0013_RO_A12_1_E12_B	42	16	LMG 1346	*Marinilabilia*	*salmonicolor*
Z0013_LB_B06_1_D04_B	42	16	LMG 1346	*Marinilabilia*	*salmonicolor*
Z0013_LB_B05_1_D04_A	42	16	LMG 1346	*Marinilabilia*	*salmonicolor*
Z0013_RB_A11_2_D07_A	42	16	LMG 1346	*Marinilabilia*	*salmonicolor*
Z0013_RB_A12_2_D07_B	42	16	LMG 1346	*Marinilabilia*	*salmonicolor*
Z0013_LO_B05_2_E03_A	42	16	LMG 1346	*Marinilabilia*	*salmonicolor*
Z0013_RB_B06_1_D10_B	42	16	LMG 1346	*Marinilabilia*	*salmonicolor*
Z0013_RB_B01_1_D12_A	42	16	LMG 1346	*Marinilabilia*	*salmonicolor*
Z0013_LB_B01_1_D06_A	42	16	LMG 1346	*Marinilabilia*	*salmonicolor*
Z0013_LB_B04_1_D05_B	42	16	LMG 1346	*Marinilabilia*	*salmonicolor*
Z0013_RB_B02_1_D12_B	43	16	LMG 1346	*Marinilabilia*	*salmonicolor*
Z0013_LO_B06_2_E03_B	43	16	LMG 1346	*Marinilabilia*	*salmonicolor*
Z0013_RB_B03_1_D11_A	43	16	LMG 1346	*Marinilabilia*	*salmonicolor*
Z0013_LO_B07_2_E04_A	43	16	LMG 1346	*Marinilabilia*	*salmonicolor*
Z0023_RO_A06_1_E09_B	44	123	LMG 6451	*Bacteroides*	*ureolyticus*
Z0023_RB_H11_1_A07_A	44	123	LMG 6451	*Bacteroides*	*ureolyticus*
Z0023_RO_C09_1_F11_A	44	123	LMG 6451	*Bacteroides*	*ureolyticus*
Z0023_LO_C10_1_F05_B	44	123	LMG 6451	*Bacteroides*	*ureolyticus*
Z0023_RO_A10_1_E11_B	44	123	LMG 6451	*Bacteroides*	*ureolyticus*
Z0023_RO_A07_1_E10_A	44	123	LMG 6451	*Bacteroides*	*ureolyticus*
Z0023_RO_A01_1_E07_A	44	123	LMG 6451	*Bacteroides*	*ureolyticus*
Z0023_LO_B03_2_E02_A	44	123	LMG 6451	*Bacteroides*	*ureolyticus*
Z0023_RO_B01_2_E07_A	44	123	LMG 6451	*Bacteroides*	*ureolyticus*
Z0023_LO_C05_1_F03_A	44	123	LMG 6451	*Bacteroides*	*ureolyticus*
Z0023_LO_D03_2_F02_A	44	123	LMG 6451	*Bacteroides*	*ureolyticus*
Z0023_LO_C07_1_F04_A	44	123	LMG 6451	*Bacteroides*	*ureolyticus*
Z0023_RO_D08_2_F10_B	44	123	LMG 6451	*Bacteroides*	*ureolyticus*
Z0023_LO_C09_1_F05_A	44	123	LMG 6451	*Bacteroides*	*ureolyticus*
Z0023_LO_B08_2_E04_B	44	123	LMG 6451	*Bacteroides*	*ureolyticus*
Z0023_RO_C05_1_F09_A	44	123	LMG 6451	*Bacteroides*	*ureolyticus*
Z0023_LO_B11_2_E06_A	44	123	LMG 6451	*Bacteroides*	*ureolyticus*

Z0023_RO_D06_2_F09_B	44	123	LMG 6451	*Bacteroides*	*ureolyticus*
Z0023_RO_D09_2_F11_A	44	123	LMG 6451	*Bacteroides*	*ureolyticus*
Z0023_LO_C06_1_F03_B	44	123	LMG 6451	*Bacteroides*	*ureolyticus*
Z0023_LO_C11_1_F06_A	44	123	LMG 6451	*Bacteroides*	*ureolyticus*
Z0023_RO_D07_2_F10_A	44	123	LMG 6451	*Bacteroides*	*ureolyticus*
Z0023_LO_D11_2_F06_A	44	123	LMG 6451	*Bacteroides*	*ureolyticus*
Z0023_RO_C08_1_F10_B	44	123	LMG 6451	*Bacteroides*	*ureolyticus*
Z0023_LO_D04_2_F02_B	44	123	LMG 6451	*Bacteroides*	*ureolyticus*
Z0023_LO_D10_2_F05_B	44	123	LMG 6451	*Bacteroides*	*ureolyticus*
Z0023_RO_D04_2_F08_B	44	123	LMG 6451	*Bacteroides*	*ureolyticus*
Z0023_RO_D05_2_F09_A	44	123	LMG 6451	*Bacteroides*	*ureolyticus*
Z0023_RO_D03_2_F08_A	44	123	LMG 6451	*Bacteroides*	*ureolyticus*
Z0023_RO_D12_2_F12_B	44	123	LMG 6451	*Bacteroides*	*ureolyticus*
Z0023_LO_C12_1_F06_B	45	123	LMG 6451	*Bacteroides*	*ureolyticus*
Z0023_RO_B02_2_E07_B	46	123	LMG 6451	*Bacteroides*	*ureolyticus*
Z0017_LB_D11_1_C01_A	47	58	LMG 23655	*Carnobacterium*	*inhibens*
Z0017_LB_D10_1_C02_B	47	58	LMG 23655	*Carnobacterium*	*inhibens*
Z0017_LB_E02_2_B06_B	47	58	LMG 23655	*Carnobacterium*	*inhibens*
Z0017_RB_C12_2_C07_B	47	58	LMG 23655	*Carnobacterium*	*inhibens*
Z0017_RB_D09_1_C08_A	47	58	LMG 23655	*Carnobacterium*	*inhibens*
Z0017_RB_D08_1_C09_B	47	58	LMG 23655	*Carnobacterium*	*inhibens*
Z0017_RB_D12_1_C07_B	47	58	LMG 23655	*Carnobacterium*	*inhibens*
Z0017_RB_D01_1_C12_A	47	58	LMG 23655	*Carnobacterium*	*inhibens*
Z0017_LB_E01_2_B06_A	47	58	LMG 23655	*Carnobacterium*	*inhibens*
Z0017_RB_D02_1_C12_B	47	58	LMG 23655	*Carnobacterium*	*inhibens*
Z0017_RB_D03_1_C11_A	47	58	LMG 23655	*Carnobacterium*	*inhibens*
Z0017_RB_D07_1_C09_A	47	58	LMG 23655	*Carnobacterium*	*inhibens*
Z0017_RB_E01_2_B12_A	47	58	LMG 23655	*Carnobacterium*	*inhibens*
Z0017_RB_D05_1_C10_A	47	58	LMG 23655	*Carnobacterium*	*inhibens*
Z0017_RB_D04_1_C11_B	47	58	LMG 23655	*Carnobacterium*	*inhibens*
Z0017_LB_E08_2_B03_B	47	58	LMG 23655	*Carnobacterium*	*inhibens*
Z0017_RB_E02_2_B12_B	47	58	LMG 23655	*Carnobacterium*	*inhibens*
Z0017_LB_E05_2_B04_A	47	58	LMG 23655	*Carnobacterium*	*inhibens*
Z0017_LB_E06_2_B04_B	47	58	LMG 23655	*Carnobacterium*	*inhibens*
Z0017_RB_E03_2_B11_A	47	58	LMG 23655	*Carnobacterium*	*inhibens*
Z0017_RB_E04_2_B11_B	47	58	LMG 23655	*Carnobacterium*	*inhibens*
Z0017_RB_D06_1_C10_B	47	58	LMG 23655	*Carnobacterium*	*inhibens*
Z0017_RB_D10_1_C08_B	47	58	LMG 23655	*Carnobacterium*	*inhibens*
Z0017_RB_D11_1_C07_A	47	58	LMG 23655	*Carnobacterium*	*inhibens*
Z0017_LB_E04_2_B05_B	47	58	LMG 23655	*Carnobacterium*	*inhibens*
Z0017_LB_E03_2_B05_A	47	58	LMG 23655	*Carnobacterium*	*inhibens*
Z0017_RB_C11_2_C07_A	47	58	LMG 23655	*Carnobacterium*	*inhibens*
Z0017_LB_D09_1_C02_A	47	58	LMG 23655	*Carnobacterium*	*inhibens*
Z0017_RB_C10_2_C08_B	47	58	LMG 23655	*Carnobacterium*	*inhibens*
Z0017_LB_D08_1_C03_B	47	58	LMG 23655	*Carnobacterium*	*inhibens*
Z0017_LB_D12_1_C01_B	47	58	LMG 23655	*Carnobacterium*	*inhibens*
Z0017_LB_E07_2_B03_A	48	58	LMG 23655	*Carnobacterium*	*inhibens*
Z0019_RB_E02_2_B12_B	49	80	LMG 25535	*Arcobacter*	*trophiarum*
Z0019_RB_E04_2_B11_B	49	80	LMG 25535	*Arcobacter*	*trophiarum*
Z0019_RB_E05_2_B10_A	49	80	LMG 25535	*Arcobacter*	*trophiarum*

Z0019_RB_D05_1_C10_A	49	80	LMG 25535	*Arcobacter*	*trophiarum*
Z0019_LB_E03_2_B05_A	49	80	LMG 25535	*Arcobacter*	*trophiarum*
Z0019_RB_D04_1_C11_B	49	80	LMG 25535	*Arcobacter*	*trophiarum*
Z0019_RB_E06_2_B10_B	49	80	LMG 25535	*Arcobacter*	*trophiarum*
Z0019_LB_E05_2_B04_A	49	80	LMG 25535	*Arcobacter*	*trophiarum*
Z0019_LB_E06_2_B04_B	49	80	LMG 25535	*Arcobacter*	*trophiarum*
Z0019_RB_E03_2_B11_A	49	80	LMG 25535	*Arcobacter*	*trophiarum*
Z0019_RB_D06_1_C10_B	49	80	LMG 25535	*Arcobacter*	*trophiarum*
Z0019_LB_E01_2_B06_A	49	80	LMG 25535	*Arcobacter*	*trophiarum*
Z0019_LB_D12_1_C01_B	49	80	LMG 25535	*Arcobacter*	*trophiarum*
Z0019_LB_D11_1_C01_A	49	80	LMG 25535	*Arcobacter*	*trophiarum*
Z0019_LB_E02_2_B06_B	49	80	LMG 25535	*Arcobacter*	*trophiarum*
Z0019_LB_E07_2_B03_A	49	80	LMG 25535	*Arcobacter*	*trophiarum*
Z0019_RB_D03_1_C11_A	49	80	LMG 25535	*Arcobacter*	*trophiarum*
Z0019_LB_D10_1_C02_B	49	80	LMG 25535	*Arcobacter*	*trophiarum*
Z0019_RB_D02_1_C12_B	49	80	LMG 25535	*Arcobacter*	*trophiarum*
Z0019_RB_D01_1_C12_A	49	80	LMG 25535	*Arcobacter*	*trophiarum*
Z0019_RB_D07_1_C09_A	49	80	LMG 25535	*Arcobacter*	*trophiarum*
Z0019_LB_E04_2_B05_B	49	80	LMG 25535	*Arcobacter*	*trophiarum*
Z0019_RB_D09_1_C08_A	49	80	LMG 25535	*Arcobacter*	*trophiarum*
Z0019_RB_D12_1_C07_B	50	80	LMG 25535	*Arcobacter*	*trophiarum*
Z0019_RB_D11_1_C07_A	50	80	LMG 25535	*Arcobacter*	*trophiarum*
Z0019_RB_C12_2_C07_B	50	80	LMG 25535	*Arcobacter*	*trophiarum*
Z0019_LB_D09_1_C02_A	50	80	LMG 25535	*Arcobacter*	*trophiarum*
Z0019_RB_D10_1_C08_B	50	80	LMG 25535	*Arcobacter*	*trophiarum*
Z0019_RB_E01_2_B12_A	50	80	LMG 25535	*Arcobacter*	*trophiarum*
Z0019_LB_D08_1_C03_B	50	80	LMG 25535	*Arcobacter*	*trophiarum*
Z0019_LB_D07_1_C03_A	50	80	LMG 25535	*Arcobacter*	*trophiarum*
Z0019_RB_D08_1_C09_B	50	80	LMG 25535	*Arcobacter*	*trophiarum*
Z0023_RO_F12_2_G12_B	51	122	LMG 5743	*Xanthomonas*	*populi*
Z0023_RO_G04_1_H08_B	51	122	LMG 5743	*Xanthomonas*	*populi*
Z0023_LO_H09_2_H05_A	51	122	LMG 5743	*Xanthomonas*	*populi*
Z0023_LO_G10_1_H05_B	51	122	LMG 5743	*Xanthomonas*	*populi*
Z0023_RO_H04_2_H08_B	51	122	LMG 5743	*Xanthomonas*	*populi*
Z0023_LO_H10_2_H05_B	51	122	LMG 5743	*Xanthomonas*	*populi*
Z0023_RO_G02_1_H07_B	51	122	LMG 5743	*Xanthomonas*	*populi*
Z0023_RO_F09_2_G11_A	51	122	LMG 5743	*Xanthomonas*	*populi*
Z0023_RO_F11_2_G12_A	51	122	LMG 5743	*Xanthomonas*	*populi*
Z0023_LO_H08_2_H04_B	51	122	LMG 5743	*Xanthomonas*	*populi*
Z0023_RO_F10_2_G11_B	51	122	LMG 5743	*Xanthomonas*	*populi*
Z0023_RO_H02_2_H07_B	51	122	LMG 5743	*Xanthomonas*	*populi*
Z0023_RO_H03_2_H08_A	51	122	LMG 5743	*Xanthomonas*	*populi*
Z0023_LO_H11_2_H06_A	51	122	LMG 5743	*Xanthomonas*	*populi*
Z0023_RO_G11_1_H12_A	51	122	LMG 5743	*Xanthomonas*	*populi*
Z0023_RO_G01_1_H07_A	51	122	LMG 5743	*Xanthomonas*	*populi*
Z0023_LO_G11_1_H06_A	52	122	LMG 5743	*Xanthomonas*	*populi*
Z0023_LO_G04_1_H02_B	52	122	LMG 5743	*Xanthomonas*	*populi*
Z0023_LO_H04_2_H02_B	52	122	LMG 5743	*Xanthomonas*	*populi*
Z0023_LO_H03_2_H02_A	52	122	LMG 5743	*Xanthomonas*	*populi*
Z0023_RO_G10_1_H11_B	52	122	LMG 5743	*Xanthomonas*	*populi*

Z0023_LO_H06_2_H03_B	52	122	LMG 5743	*Xanthomonas*	*populi*
Z0023_LO_H07_2_H04_A	52	122	LMG 5743	*Xanthomonas*	*populi*
Z0023_RO_G12_1_H12_B	52	122	LMG 5743	*Xanthomonas*	*populi*
Z0023_RO_G05_1_H09_A	52	122	LMG 5743	*Xanthomonas*	*populi*
Z0023_LO_H05_2_H03_A	52	122	LMG 5743	*Xanthomonas*	*populi*
Z0023_RO_H01_2_H07_A	52	122	LMG 5743	*Xanthomonas*	*populi*
Z0023_LO_H02_2_H01_B	52	122	LMG 5743	*Xanthomonas*	*populi*
Z0023_RO_G06_1_H09_B	52	122	LMG 5743	*Xanthomonas*	*populi*
Z0023_LO_G03_1_H02_A	52	122	LMG 5743	*Xanthomonas*	*populi*
Z0023_LO_G12_1_H06_B	52	122	LMG 5743	*Xanthomonas*	*populi*
Z0023_LO_H01_2_H01_A	52	122	LMG 5743	*Xanthomonas*	*populi*
Z0021_LB_F07_1_B03_A	53	101	LMG 27719	*Roseomonas*	*gilardii*
Z0021_LB_F09_1_B02_A	53	101	LMG 27719	*Roseomonas*	*gilardii*
Z0021_RB_E11_2_B07_A	53	101	LMG 27719	*Roseomonas*	*gilardii*
Z0021_LB_F02_1_B06_B	53	101	LMG 27719	*Roseomonas*	*gilardii*
Z0021_LB_F01_1_B06_A	53	101	LMG 27719	*Roseomonas*	*gilardii*
Z0021_LB_E11_2_B01_A	53	101	LMG 27719	*Roseomonas*	*gilardii*
Z0021_RB_F11_1_B07_A	53	101	LMG 27719	*Roseomonas*	*gilardii*
Z0021_RB_F09_1_B08_A	53	101	LMG 27719	*Roseomonas*	*gilardii*
Z0021_RB_E10_2_B08_B	53	101	LMG 27719	*Roseomonas*	*gilardii*
Z0021_LB_F05_1_B04_A	53	101	LMG 27719	*Roseomonas*	*gilardii*
Z0021_LB_F08_1_B03_B	53	101	LMG 27719	*Roseomonas*	*gilardii*
Z0021_LB_F04_1_B05_B	54	101	LMG 27719	*Roseomonas*	*gilardii*
Z0021_LB_F06_1_B04_B	54	101	LMG 27719	*Roseomonas*	*gilardii*
Z0021_RB_E07_2_B09_A	54	101	LMG 27719	*Roseomonas*	*gilardii*
Z0021_RB_F06_1_B10_B	54	101	LMG 27719	*Roseomonas*	*gilardii*
Z0021_RB_E08_2_B09_B	54	101	LMG 27719	*Roseomonas*	*gilardii*
Z0021_RB_F05_1_B10_A	54	101	LMG 27719	*Roseomonas*	*gilardii*
Z0021_LB_F03_1_B05_A	54	101	LMG 27719	*Roseomonas*	*gilardii*
Z0021_RB_F04_1_B11_B	54	101	LMG 27719	*Roseomonas*	*gilardii*
Z0021_RB_F07_1_B09_A	54	101	LMG 27719	*Roseomonas*	*gilardii*
Z0021_RB_E12_2_B07_B	54	101	LMG 27719	*Roseomonas*	*gilardii*
Z0021_RB_F02_1_B12_B	54	101	LMG 27719	*Roseomonas*	*gilardii*
Z0021_RB_F01_1_B12_A	54	101	LMG 27719	*Roseomonas*	*gilardii*
Z0021_RB_F03_1_B11_A	54	101	LMG 27719	*Roseomonas*	*gilardii*
Z0021_RB_E09_2_B08_A	54	101	LMG 27719	*Roseomonas*	*gilardii*
Z0021_RB_F08_1_B09_B	54	101	LMG 27719	*Roseomonas*	*gilardii*
Z0021_LB_F11_1_B01_A	54	101	LMG 27719	*Roseomonas*	*gilardii*
Z0021_RB_F12_1_B07_B	54	101	LMG 27719	*Roseomonas*	*gilardii*
Z0021_RB_G01_2_A12_A	54	101	LMG 27719	*Roseomonas*	*gilardii*
Z0021_LB_F10_1_B02_B	54	101	LMG 27719	*Roseomonas*	*gilardii*
Z0021_RB_F10_1_B08_B	55	101	LMG 27719	*Roseomonas*	*gilardii*
Z0021_LB_E12_2_B01_B	55	101	LMG 27719	*Roseomonas*	*gilardii*
Z0022_RO_D11_2_F12_A	56	114	LMG 4050	*Micrococcus*	*luteus*
Z0022_LO_E09_1_G05_A	56	114	LMG 4050	*Micrococcus*	*luteus*
Z0022_RO_D07_2_F10_A	56	114	LMG 4050	*Micrococcus*	*luteus*
Z0022_LO_E11_1_G06_A	56	114	LMG 4050	*Micrococcus*	*luteus*
Z0022_LO_E05_1_G03_A	56	114	LMG 4050	*Micrococcus*	*luteus*
Z0022_RO_C02_1_F07_B	56	114	LMG 4050	*Micrococcus*	*luteus*
Z0022_LO_D06_2_F03_B	56	114	LMG 4050	*Micrococcus*	*luteus*

Z0022_LO_D08_2_F04_B	56	114	LMG 4050	*Micrococcus*	*luteus*	
Z0022_RO_B10_2_E11_B	56	114	LMG 4050	*Micrococcus*	*luteus*	
Z0022_LO_C01_1_F01_A	56	114	LMG 4050	*Micrococcus*	*luteus*	
Z0022_LO_E02_1_G01_B	56	114	LMG 4050	*Micrococcus*	*luteus*	
Z0022_LO_C02_1_F01_B	56	114	LMG 4050	*Micrococcus*	*luteus*	
Z0022_LO_A11_1_E06_A	56	114	LMG 4050	*Micrococcus*	*luteus*	
Z0022_RO_C03_1_F08_A	56	114	LMG 4050	*Micrococcus*	*luteus*	
Z0022_RO_C04_1_F08_B	56	114	LMG 4050	*Micrococcus*	*luteus*	
Z0022_RO_B12_2_E12_B	56	114	LMG 4050	*Micrococcus*	*luteus*	
Z0022_RO_C01_1_F07_A	56	114	LMG 4050	*Micrococcus*	*luteus*	
Z0022_LO_C03_1_F02_A	56	114	LMG 4050	*Micrococcus*	*luteus*	
Z0022_LO_D07_2_F04_A	56	114	LMG 4050	*Micrococcus*	*luteus*	
Z0022_LO_D11_2_F06_A	56	114	LMG 4050	*Micrococcus*	*luteus*	
Z0022_RO_C05_1_F09_A	56	114	LMG 4050	*Micrococcus*	*luteus*	
Z0022_LO_D10_2_F05_B	56	114	LMG 4050	*Micrococcus*	*luteus*	
Z0022_RO_B09_2_E11_A	56	114	LMG 4050	*Micrococcus*	*luteus*	
Z0022_RO_B11_2_E12_A	56	114	LMG 4050	*Micrococcus*	*luteus*	
Z0022_LO_E01_1_G01_A	56	114	LMG 4050	*Micrococcus*	*luteus*	
Z0022_LO_E10_1_G05_B	56	114	LMG 4050	*Micrococcus*	*luteus*	
Z0022_LO_E04_1_G02_B	56	114	LMG 4050	*Micrococcus*	*luteus*	
Z0022_LO_A03_1_E02_A	56	114	LMG 4050	*Micrococcus*	*luteus*	
Z0022_LO_A02_1_E01_B	56	114	LMG 4050	*Micrococcus*	*luteus*	
Z0022_RO_C06_1_F09_B	56	114	LMG 4050	*Micrococcus*	*luteus*	
Z0022_RO_D04_2_F08_B	56	114	LMG 4050	*Micrococcus*	*luteus*	
Z0022_RO_D01_2_F07_A	56	114	LMG 4050	*Micrococcus*	*luteus*	
Z0024_LB_F11_1_B01_A	57	127	LMG 6901	*Lactobacillus*	*delbrueckii*	*bulgaricus*
Z0024_LB_F12_1_B01_B	57	127	LMG 6901	*Lactobacillus*	*delbrueckii*	*bulgaricus*
Z0024_LB_G07_2_A03_A	57	127	LMG 6901	*Lactobacillus*	*delbrueckii*	*bulgaricus*
Z0024_LB_F09_1_B02_A	57	127	LMG 6901	*Lactobacillus*	*delbrueckii*	*bulgaricus*
Z0024_RB_G03_2_A11_A	57	127	LMG 6901	*Lactobacillus*	*delbrueckii*	*bulgaricus*
Z0024_LB_G10_2_A02_B	57	127	LMG 6901	*Lactobacillus*	*delbrueckii*	*bulgaricus*
Z0024_LB_H04_1_A05_B	57	127	LMG 6901	*Lactobacillus*	*delbrueckii*	*bulgaricus*
Z0024_LB_H03_1_A05_A	57	127	LMG 6901	*Lactobacillus*	*delbrueckii*	*bulgaricus*
Z0024_RB_F12_1_B07_B	57	127	LMG 6901	*Lactobacillus*	*delbrueckii*	*bulgaricus*
Z0024_LB_G06_2_A04_B	57	127	LMG 6901	*Lactobacillus*	*delbrueckii*	*bulgaricus*
Z0024_RB_F11_1_B07_A	57	127	LMG 6901	*Lactobacillus*	*delbrueckii*	*bulgaricus*
Z0024_RB_G06_2_A10_B	57	127	LMG 6901	*Lactobacillus*	*delbrueckii*	*bulgaricus*
Z0024_RB_G02_2_A12_B	57	127	LMG 6901	*Lactobacillus*	*delbrueckii*	*bulgaricus*
Z0024_RB_G05_2_A10_A	57	127	LMG 6901	*Lactobacillus*	*delbrueckii*	*bulgaricus*
Z0024_RB_G04_2_A11_B	57	127	LMG 6901	*Lactobacillus*	*delbrueckii*	*bulgaricus*
Z0024_RB_F10_1_B08_B	57	127	LMG 6901	*Lactobacillus*	*delbrueckii*	*bulgaricus*
Z0024_LB_H02_1_A06_B	57	127	LMG 6901	*Lactobacillus*	*delbrueckii*	*bulgaricus*
Z0024_LB_G01_2_A06_A	57	127	LMG 6901	*Lactobacillus*	*delbrueckii*	*bulgaricus*
Z0024_LB_G11_2_A01_A	57	127	LMG 6901	*Lactobacillus*	*delbrueckii*	*bulgaricus*
Z0024_LB_G12_2_A01_B	57	127	LMG 6901	*Lactobacillus*	*delbrueckii*	*bulgaricus*
Z0024_LB_H01_1_A06_A	57	127	LMG 6901	*Lactobacillus*	*delbrueckii*	*bulgaricus*
Z0024_LB_G08_2_A03_B	57	127	LMG 6901	*Lactobacillus*	*delbrueckii*	*bulgaricus*
Z0024_LB_F08_1_B03_B	57	127	LMG 6901	*Lactobacillus*	*delbrueckii*	*bulgaricus*
Z0024_LB_G04_2_A05_B	57	127	LMG 6901	*Lactobacillus*	*delbrueckii*	*bulgaricus*
Z0024_LB_G03_2_A05_A	57	127	LMG 6901	*Lactobacillus*	*delbrueckii*	*bulgaricus*

Z0024_LB_F10_1_B02_B	57	127	LMG 6901	*Lactobacillus*	*delbrueckii*	*bulgaricus*
Z0024_LB_G02_2_A06_B	57	127	LMG 6901	*Lactobacillus*	*delbrueckii*	*bulgaricus*
Z0024_RB_G07_2_A09_A	57	127	LMG 6901	*Lactobacillus*	*delbrueckii*	*bulgaricus*
Z0024_LB_G09_2_A02_A	57	127	LMG 6901	*Lactobacillus*	*delbrueckii*	*bulgaricus*
Z0024_RB_F09_1_B08_A	57	127	LMG 6901	*Lactobacillus*	*delbrueckii*	*bulgaricus*
Z0024_RB_G01_2_A12_A	57	127	LMG 6901	*Lactobacillus*	*delbrueckii*	*bulgaricus*
Z0024_LB_G05_2_A04_A	58	127	LMG 6901	*Lactobacillus*	*delbrueckii*	*bulgaricus*
Z0017_LO_G05_1_H03_A	59	64	LMG 24024	*Oceanobacillus*	*oncorhynchi*	*oncorhynchi*
Z0017_LO_G02_1_H01_B	59	64	LMG 24024	*Oceanobacillus*	*oncorhynchi*	*oncorhynchi*
Z0017_LO_F09_2_G05_A	59	64	LMG 24024	*Oceanobacillus*	*oncorhynchi*	*oncorhynchi*
Z0017_RO_G08_1_H10_B	59	64	LMG 24024	*Oceanobacillus*	*oncorhynchi*	*oncorhynchi*
Z0017_RO_G05_1_H09_A	59	64	LMG 24024	*Oceanobacillus*	*oncorhynchi*	*oncorhynchi*
Z0017_LO_F10_2_G05_B	59	64	LMG 24024	*Oceanobacillus*	*oncorhynchi*	*oncorhynchi*
Z0017_RO_G10_1_H11_B	59	64	LMG 24024	*Oceanobacillus*	*oncorhynchi*	*oncorhynchi*
Z0017_LO_G04_1_H02_B	59	64	LMG 24024	*Oceanobacillus*	*oncorhynchi*	*oncorhynchi*
Z0017_LO_G06_1_H03_B	59	64	LMG 24024	*Oceanobacillus*	*oncorhynchi*	*oncorhynchi*
Z0017_LO_G09_1_H05_A	59	64	LMG 24024	*Oceanobacillus*	*oncorhynchi*	*oncorhynchi*
Z0017_RO_G12_1_H12_B	59	64	LMG 24024	*Oceanobacillus*	*oncorhynchi*	*oncorhynchi*
Z0017_RO_G09_1_H11_A	59	64	LMG 24024	*Oceanobacillus*	*oncorhynchi*	*oncorhynchi*
Z0017_RO_G06_1_H09_B	59	64	LMG 24024	*Oceanobacillus*	*oncorhynchi*	*oncorhynchi*
Z0017_RO_G03_1_H08_A	59	64	LMG 24024	*Oceanobacillus*	*oncorhynchi*	*oncorhynchi*
Z0017_RO_H01_2_H07_A	60	64	LMG 24024	*Oceanobacillus*	*oncorhynchi*	*oncorhynchi*
Z0017_LO_F12_2_G06_B	60	64	LMG 24024	*Oceanobacillus*	*oncorhynchi*	*oncorhynchi*
Z0017_RO_H04_2_H08_B	61	64	LMG 24024	*Oceanobacillus*	*oncorhynchi*	*oncorhynchi*
Z0017_RO_G01_1_H07_A	61	64	LMG 24024	*Oceanobacillus*	*oncorhynchi*	*oncorhynchi*
Z0017_RO_H02_2_H07_B	61	64	LMG 24024	*Oceanobacillus*	*oncorhynchi*	*oncorhynchi*
Z0017_LO_G08_1_H04_B	61	64	LMG 24024	*Oceanobacillus*	*oncorhynchi*	*oncorhynchi*
Z0017_RO_H03_2_H08_A	61	64	LMG 24024	*Oceanobacillus*	*oncorhynchi*	*oncorhynchi*
Z0017_LO_F04_2_G02_B	61	64	LMG 24024	*Oceanobacillus*	*oncorhynchi*	*oncorhynchi*
Z0017_RO_F12_2_G12_B	61	64	LMG 24024	*Oceanobacillus*	*oncorhynchi*	*oncorhynchi*
Z0017_LO_F07_2_G04_A	61	64	LMG 24024	*Oceanobacillus*	*oncorhynchi*	*oncorhynchi*
Z0017_LO_G01_1_H01_A	62	64	LMG 24024	*Oceanobacillus*	*oncorhynchi*	*oncorhynchi*
Z0017_LO_G03_1_H02_A	62	64	LMG 24024	*Oceanobacillus*	*oncorhynchi*	*oncorhynchi*
Z0017_RO_G07_1_H10_A	62	64	LMG 24024	*Oceanobacillus*	*oncorhynchi*	*oncorhynchi*
Z0017_RO_G04_1_H08_B	62	64	LMG 24024	*Oceanobacillus*	*oncorhynchi*	*oncorhynchi*
Z0017_RO_G02_1_H07_B	62	64	LMG 24024	*Oceanobacillus*	*oncorhynchi*	*oncorhynchi*
Z0017_LO_F11_2_G06_A	62	64	LMG 24024	*Oceanobacillus*	*oncorhynchi*	*oncorhynchi*
Z0017_LO_G07_1_H04_A	62	64	LMG 24024	*Oceanobacillus*	*oncorhynchi*	*oncorhynchi*
Z0017_RO_G11_1_H12_A	62	64	LMG 24024	*Oceanobacillus*	*oncorhynchi*	*oncorhynchi*
Z0025_RB_H05_1_A10_A	63	137	LMG 8760	*Lysobacter*	*antibioticus*	
Z0025_LB_H04_1_A05_B	63	137	LMG 8760	*Lysobacter*	*antibioticus*	
Z0025_RB_G10_2_A08_B	63	137	LMG 8760	*Lysobacter*	*antibioticus*	
Z0025_RB_H07_1_A09_A	63	137	LMG 8760	*Lysobacter*	*antibioticus*	
Z0025_LB_H07_1_A03_A	63	137	LMG 8760	*Lysobacter*	*antibioticus*	
Z0025_RB_H09_1_A08_A	63	137	LMG 8760	*Lysobacter*	*antibioticus*	
Z0025_RB_H10_1_A08_B	63	137	LMG 8760	*Lysobacter*	*antibioticus*	
Z0025_RB_G11_2_A07_A	63	137	LMG 8760	*Lysobacter*	*antibioticus*	
Z0025_RB_G09_2_A08_A	63	137	LMG 8760	*Lysobacter*	*antibioticus*	
Z0025_RB_G08_2_A09_B	63	137	LMG 8760	*Lysobacter*	*antibioticus*	
Z0025_LB_H02_1_A06_B	63	137	LMG 8760	*Lysobacter*	*antibioticus*	

Z0025_LB_H03_1_A05_A	63	137	LMG 8760	*Lysobacter*	*antibioticus*
Z0025_LB_H01_1_A06_A	63	137	LMG 8760	*Lysobacter*	*antibioticus*
Z0025_RB_H11_1_A07_A	63	137	LMG 8760	*Lysobacter*	*antibioticus*
Z0025_RB_H12_1_A07_B	63	137	LMG 8760	*Lysobacter*	*antibioticus*
Z0025_RB_H03_1_A11_A	63	137	LMG 8760	*Lysobacter*	*antibioticus*
Z0025_LB_H11_1_A01_A	63	137	LMG 8760	*Lysobacter*	*antibioticus*
Z0025_LB_H09_1_A02_A	63	137	LMG 8760	*Lysobacter*	*antibioticus*
Z0025_RB_H02_1_A12_B	63	137	LMG 8760	*Lysobacter*	*antibioticus*
Z0025_LB_G12_2_A01_B	63	137	LMG 8760	*Lysobacter*	*antibioticus*
Z0025_LB_H08_1_A03_B	63	137	LMG 8760	*Lysobacter*	*antibioticus*
Z0025_LB_G10_2_A02_B	63	137	LMG 8760	*Lysobacter*	*antibioticus*
Z0025_RB_H08_1_A09_B	63	137	LMG 8760	*Lysobacter*	*antibioticus*
Z0025_RB_H01_1_A12_A	63	137	LMG 8760	*Lysobacter*	*antibioticus*
Z0025_LB_G11_2_A01_A	63	137	LMG 8760	*Lysobacter*	*antibioticus*
Z0025_RB_H06_1_A10_B	63	137	LMG 8760	*Lysobacter*	*antibioticus*
Z0025_LB_G09_2_A02_A	63	137	LMG 8760	*Lysobacter*	*antibioticus*
Z0025_LB_H06_1_A04_B	63	137	LMG 8760	*Lysobacter*	*antibioticus*
Z0025_RB_G12_2_A07_B	63	137	LMG 8760	*Lysobacter*	*antibioticus*
Z0025_RB_H04_1_A11_B	63	137	LMG 8760	*Lysobacter*	*antibioticus*
Z0025_LB_H05_1_A04_A	63	137	LMG 8760	*Lysobacter*	*antibioticus*
Z0025_LB_G08_2_A03_B	63	137	LMG 8760	*Lysobacter*	*antibioticus*
Z0024_RB_E10_2_B08_B	64	126	LMG 6896	*Streptococcus*	*thermophilus*
Z0024_LB_E12_2_B01_B	64	126	LMG 6896	*Streptococcus*	*thermophilus*
Z0024_RB_F08_1_B09_B	64	126	LMG 6896	*Streptococcus*	*thermophilus*
Z0024_RB_F01_1_B12_A	64	126	LMG 6896	*Streptococcus*	*thermophilus*
Z0024_RB_F07_1_B09_A	64	126	LMG 6896	*Streptococcus*	*thermophilus*
Z0024_RB_E09_2_B08_A	64	126	LMG 6896	*Streptococcus*	*thermophilus*
Z0024_RB_E08_2_B09_B	64	126	LMG 6896	*Streptococcus*	*thermophilus*
Z0024_LB_E06_2_B04_B	64	126	LMG 6896	*Streptococcus*	*thermophilus*
Z0024_RB_F02_1_B12_B	64	126	LMG 6896	*Streptococcus*	*thermophilus*
Z0024_LB_F05_1_B04_A	64	126	LMG 6896	*Streptococcus*	*thermophilus*
Z0024_LB_E07_2_B03_A	64	126	LMG 6896	*Streptococcus*	*thermophilus*
Z0024_LB_E11_2_B01_A	64	126	LMG 6896	*Streptococcus*	*thermophilus*
Z0024_LB_E05_2_B04_A	64	126	LMG 6896	*Streptococcus*	*thermophilus*
Z0024_RB_F06_1_B10_B	64	126	LMG 6896	*Streptococcus*	*thermophilus*
Z0024_LB_F01_1_B06_A	65	126	LMG 6896	*Streptococcus*	*thermophilus*
Z0024_LB_F02_1_B06_B	65	126	LMG 6896	*Streptococcus*	*thermophilus*
Z0024_RB_E12_2_B07_B	65	126	LMG 6896	*Streptococcus*	*thermophilus*
Z0024_LB_E08_2_B03_B	65	126	LMG 6896	*Streptococcus*	*thermophilus*
Z0024_LB_F04_1_B05_B	65	126	LMG 6896	*Streptococcus*	*thermophilus*
Z0024_LB_F06_1_B04_B	65	126	LMG 6896	*Streptococcus*	*thermophilus*
Z0024_LB_F07_1_B03_A	65	126	LMG 6896	*Streptococcus*	*thermophilus*
Z0024_RB_F03_1_B11_A	65	126	LMG 6896	*Streptococcus*	*thermophilus*
Z0024_RB_E11_2_B07_A	65	126	LMG 6896	*Streptococcus*	*thermophilus*
Z0024_LB_F03_1_B05_A	65	126	LMG 6896	*Streptococcus*	*thermophilus*
Z0022_RO_G03_1_H08_A	66	113	LMG 4044	*Vibrio*	*harveyi*
Z0022_LO_H02_2_H01_B	66	113	LMG 4044	*Vibrio*	*harveyi*
Z0022_RO_F05_2_G09_A	66	113	LMG 4044	*Vibrio*	*harveyi*
Z0022_LO_F01_2_G01_A	66	113	LMG 4044	*Vibrio*	*harveyi*
Z0022_RO_F04_2_G08_B	66	113	LMG 4044	*Vibrio*	*harveyi*

Z0022_LO_F02_2_G01_B	66	113	LMG 4044	*Vibrio*	*harveyi*
Z0022_RO_D10_2_F11_B	66	113	LMG 4044	*Vibrio*	*harveyi*
Z0022_LO_E08_1_G04_B	66	113	LMG 4044	*Vibrio*	*harveyi*
Z0022_LO_F09_2_G05_A	67	113	LMG 4044	*Vibrio*	*harveyi*
Z0022_RO_E04_1_G08_B	68	113	LMG 4044	*Vibrio*	*harveyi*
Z0022_RO_F08_2_G10_B	68	113	LMG 4044	*Vibrio*	*harveyi*
Z0022_RO_F02_2_G07_B	68	113	LMG 4044	*Vibrio*	*harveyi*
Z0022_LO_F11_2_G06_A	68	113	LMG 4044	*Vibrio*	*harveyi*
Z0022_LO_H01_2_H01_A	68	113	LMG 4044	*Vibrio*	*harveyi*
Z0022_RO_F03_2_G08_A	68	113	LMG 4044	*Vibrio*	*harveyi*
Z0022_LO_G04_1_H02_B	68	113	LMG 4044	*Vibrio*	*harveyi*
Z0022_LO_H05_2_H03_A	68	113	LMG 4044	*Vibrio*	*harveyi*
Z0022_RO_F10_2_G11_B	68	113	LMG 4044	*Vibrio*	*harveyi*
Z0022_LO_H04_2_H02_B	68	113	LMG 4044	*Vibrio*	*harveyi*
Z0022_LO_F04_2_G02_B	68	113	LMG 4044	*Vibrio*	*harveyi*
Z0022_LO_G10_1_H05_B	68	113	LMG 4044	*Vibrio*	*harveyi*
Z0022_LO_F08_2_G04_B	69	113	LMG 4044	*Vibrio*	*harveyi*
Z0022_LO_F05_2_G03_A	70	113	LMG 4044	*Vibrio*	*harveyi*
Z0022_LO_F07_2_G04_A	70	113	LMG 4044	*Vibrio*	*harveyi*
Z0022_LO_F03_2_G02_A	70	113	LMG 4044	*Vibrio*	*harveyi*
Z0022_RO_E12_1_G12_B	70	113	LMG 4044	*Vibrio*	*harveyi*
Z0022_RO_F06_2_G09_B	70	113	LMG 4044	*Vibrio*	*harveyi*
Z0022_RO_F11_2_G12_A	70	113	LMG 4044	*Vibrio*	*harveyi*
Z0022_LO_G09_1_H05_A	70	113	LMG 4044	*Vibrio*	*harveyi*
Z0022_RO_F09_2_G11_A	70	113	LMG 4044	*Vibrio*	*harveyi*
Z0022_LO_F06_2_G03_B	70	113	LMG 4044	*Vibrio*	*harveyi*
Z0022_LO_H03_2_H02_A	70	113	LMG 4044	*Vibrio*	*harveyi*
Z0013_RB_A06_2_D10_B	71	15	LMG 1345	*Leeuwenhoekiella*	*marinoflava*
Z0013_LO_A07_1_E04_A	71	15	LMG 1345	*Leeuwenhoekiella*	*marinoflava*
Z0013_RO_A03_1_E08_A	71	15	LMG 1345	*Leeuwenhoekiella*	*marinoflava*
Z0013_RO_A01_1_E07_A	72	15	LMG 1345	*Leeuwenhoekiella*	*marinoflava*
Z0013_RB_A03_2_D11_A	72	15	LMG 1345	*Leeuwenhoekiella*	*marinoflava*
Z0013_RO_A06_1_E09_B	72	15	LMG 1345	*Leeuwenhoekiella*	*marinoflava*
Z0013_LB_A07_2_D03_A	72	15	LMG 1345	*Leeuwenhoekiella*	*marinoflava*
Z0013_LB_A08_2_D03_B	72	15	LMG 1345	*Leeuwenhoekiella*	*marinoflava*
Z0013_LB_A10_2_D02_B	72	15	LMG 1345	*Leeuwenhoekiella*	*marinoflava*
Z0013_RO_A05_1_E09_A	72	15	LMG 1345	*Leeuwenhoekiella*	*marinoflava*
Z0013_RO_A02_1_E07_B	72	15	LMG 1345	*Leeuwenhoekiella*	*marinoflava*
Z0013_RB_A04_2_D11_B	72	15	LMG 1345	*Leeuwenhoekiella*	*marinoflava*
Z0013_LB_A09_2_D02_A	72	15	LMG 1345	*Leeuwenhoekiella*	*marinoflava*
Z0013_RB_A07_2_D09_A	72	15	LMG 1345	*Leeuwenhoekiella*	*marinoflava*
Z0013_LO_A06_1_E03_B	72	15	LMG 1345	*Leeuwenhoekiella*	*marinoflava*
Z0013_RO_A11_1_E12_A	72	15	LMG 1345	*Leeuwenhoekiella*	*marinoflava*
Z0013_LO_A08_1_E04_B	72	15	LMG 1345	*Leeuwenhoekiella*	*marinoflava*
Z0013_RB_A05_2_D10_A	72	15	LMG 1345	*Leeuwenhoekiella*	*marinoflava*
Z0013_RB_A09_2_D08_A	72	15	LMG 1345	*Leeuwenhoekiella*	*marinoflava*
Z0013_LO_B01_2_E01_A	72	15	LMG 1345	*Leeuwenhoekiella*	*marinoflava*
Z0013_RO_A04_1_E08_B	72	15	LMG 1345	*Leeuwenhoekiella*	*marinoflava*
Z0013_RO_A07_1_E10_A	72	15	LMG 1345	*Leeuwenhoekiella*	*marinoflava*
Z0013_RO_A08_1_E10_B	72	15	LMG 1345	*Leeuwenhoekiella*	*marinoflava*

Z0013_LO_B02_2_E01_B	72	15	LMG 1345	*Leeuwenhoekiella*	*marinoflava*
Z0013_RO_A10_1_E11_B	72	15	LMG 1345	*Leeuwenhoekiella*	*marinoflava*
Z0013_LO_A09_1_E05_A	72	15	LMG 1345	*Leeuwenhoekiella*	*marinoflava*
Z0013_RO_A09_1_E11_A	72	15	LMG 1345	*Leeuwenhoekiella*	*marinoflava*
Z0013_LO_A11_1_E06_A	72	15	LMG 1345	*Leeuwenhoekiella*	*marinoflava*
Z0013_RB_A08_2_D09_B	72	15	LMG 1345	*Leeuwenhoekiella*	*marinoflava*
Z0013_LO_A12_1_E06_B	72	15	LMG 1345	*Leeuwenhoekiella*	*marinoflava*
Z0013_LB_A11_2_D01_A	72	15	LMG 1345	*Leeuwenhoekiella*	*marinoflava*
Z0013_LO_A10_1_E05_B	72	15	LMG 1345	*Leeuwenhoekiella*	*marinoflava*
Z0014_LO_F11_2_G06_A	73	31	LMG 19667	*Lactobacillus*	*diolivorans*
Z0014_LO_G04_1_H02_B	73	31	LMG 19667	*Lactobacillus*	*diolivorans*
Z0014_LO_H07_2_H04_A	73	31	LMG 19667	*Lactobacillus*	*diolivorans*
Z0014_LO_H11_2_H06_A	73	31	LMG 19667	*Lactobacillus*	*diolivorans*
Z0014_RO_G04_1_H08_B	73	31	LMG 19667	*Lactobacillus*	*diolivorans*
Z0014_RO_G03_1_H08_A	73	31	LMG 19667	*Lactobacillus*	*diolivorans*
Z0014_RO_H01_2_H07_A	73	31	LMG 19667	*Lactobacillus*	*diolivorans*
Z0014_RO_G05_1_H09_A	73	31	LMG 19667	*Lactobacillus*	*diolivorans*
Z0014_RO_F02_2_G07_B	73	31	LMG 19667	*Lactobacillus*	*diolivorans*
Z0014_LO_F06_2_G03_B	73	31	LMG 19667	*Lactobacillus*	*diolivorans*
Z0014_RO_F01_2_G07_A	73	31	LMG 19667	*Lactobacillus*	*diolivorans*
Z0014_LO_H02_2_H01_B	73	31	LMG 19667	*Lactobacillus*	*diolivorans*
Z0014_RO_F07_2_G10_A	73	31	LMG 19667	*Lactobacillus*	*diolivorans*
Z0014_RO_F06_2_G09_B	73	31	LMG 19667	*Lactobacillus*	*diolivorans*
Z0014_RO_F08_2_G10_B	73	31	LMG 19667	*Lactobacillus*	*diolivorans*
Z0014_RO_F10_2_G11_B	73	31	LMG 19667	*Lactobacillus*	*diolivorans*
Z0014_RO_H03_2_H08_A	73	31	LMG 19667	*Lactobacillus*	*diolivorans*
Z0014_RO_G08_1_H10_B	73	31	LMG 19667	*Lactobacillus*	*diolivorans*
Z0014_LO_H05_2_H03_A	73	31	LMG 19667	*Lactobacillus*	*diolivorans*
Z0014_RO_F05_2_G09_A	73	31	LMG 19667	*Lactobacillus*	*diolivorans*
Z0014_RO_H04_2_H08_B	73	31	LMG 19667	*Lactobacillus*	*diolivorans*
Z0014_LO_H09_2_H05_A	73	31	LMG 19667	*Lactobacillus*	*diolivorans*
Z0014_RO_G09_1_H11_A	73	31	LMG 19667	*Lactobacillus*	*diolivorans*
Z0014_LO_H06_2_H03_B	73	31	LMG 19667	*Lactobacillus*	*diolivorans*
Z0014_LO_H10_2_H05_B	73	31	LMG 19667	*Lactobacillus*	*diolivorans*
Z0014_RO_F12_2_G12_B	73	31	LMG 19667	*Lactobacillus*	*diolivorans*
Z0014_LO_H01_2_H01_A	73	31	LMG 19667	*Lactobacillus*	*diolivorans*
Z0014_RO_G01_1_H07_A	73	31	LMG 19667	*Lactobacillus*	*diolivorans*
Z0014_RO_F11_2_G12_A	73	31	LMG 19667	*Lactobacillus*	*diolivorans*
Z0014_RO_G02_1_H07_B	73	31	LMG 19667	*Lactobacillus*	*diolivorans*
Z0014_LO_F01_2_G01_A	73	31	LMG 19667	*Lactobacillus*	*diolivorans*
Z0014_LO_F07_2_G04_A	73	31	LMG 19667	*Lactobacillus*	*diolivorans*
Z0023_LB_B02_1_D06_B	74	115	LMG 4051	*Deinococcus*	*radiodurans*
Z0023_LB_B04_1_D05_B	74	115	LMG 4051	*Deinococcus*	*radiodurans*
Z0023_LB_B03_1_D05_A	74	115	LMG 4051	*Deinococcus*	*radiodurans*
Z0023_LB_A04_2_D05_B	74	115	LMG 4051	*Deinococcus*	*radiodurans*
Z0023_LB_B01_1_D06_A	74	115	LMG 4051	*Deinococcus*	*radiodurans*
Z0023_RB_A04_2_D11_B	74	115	LMG 4051	*Deinococcus*	*radiodurans*
Z0023_LB_A03_2_D05_A	74	115	LMG 4051	*Deinococcus*	*radiodurans*
Z0023_RB_A08_2_D09_B	74	115	LMG 4051	*Deinococcus*	*radiodurans*
Z0023_RB_A02_2_D12_B	74	115	LMG 4051	*Deinococcus*	*radiodurans*

Z0023_LB_A01_2_D06_A	74	115	LMG 4051	*Deinococcus*	*radiodurans*
Z0023_RB_A05_2_D10_A	74	115	LMG 4051	*Deinococcus*	*radiodurans*
Z0023_LB_A02_2_D06_B	74	115	LMG 4051	*Deinococcus*	*radiodurans*
Z0023_RB_A07_2_D09_A	74	115	LMG 4051	*Deinococcus*	*radiodurans*
Z0023_RB_A03_2_D11_A	74	115	LMG 4051	*Deinococcus*	*radiodurans*
Z0023_LB_A10_2_D02_B	74	115	LMG 4051	*Deinococcus*	*radiodurans*
Z0023_RO_B11_2_E12_A	74	115	LMG 4051	*Deinococcus*	*radiodurans*
Z0023_RB_A06_2_D10_B	74	115	LMG 4051	*Deinococcus*	*radiodurans*
Z0023_LB_A11_2_D01_A	74	115	LMG 4051	*Deinococcus*	*radiodurans*
Z0023_RB_A09_2_D08_A	74	115	LMG 4051	*Deinococcus*	*radiodurans*
Z0023_LB_A12_2_D01_B	74	115	LMG 4051	*Deinococcus*	*radiodurans*
Z0023_RB_H05_1_A10_A	74	115	LMG 4051	*Deinococcus*	*radiodurans*
Z0023_LB_A08_2_D03_B	74	115	LMG 4051	*Deinococcus*	*radiodurans*
Z0023_LB_A09_2_D02_A	74	115	LMG 4051	*Deinococcus*	*radiodurans*
Z0023_RB_A01_2_D12_A	74	115	LMG 4051	*Deinococcus*	*radiodurans*
Z0023_LO_H12_2_H06_B	74	115	LMG 4051	*Deinococcus*	*radiodurans*
Z0023_RB_H06_1_A10_B	74	115	LMG 4051	*Deinococcus*	*radiodurans*
Z0023_RO_B06_2_E09_B	74	115	LMG 4051	*Deinococcus*	*radiodurans*
Z0023_LB_A06_2_D04_B	74	115	LMG 4051	*Deinococcus*	*radiodurans*
Z0023_LB_A07_2_D03_A	74	115	LMG 4051	*Deinococcus*	*radiodurans*
Z0023_RO_B04_2_E08_B	74	115	LMG 4051	*Deinococcus*	*radiodurans*
Z0023_LB_A05_2_D04_A	74	115	LMG 4051	*Deinococcus*	*radiodurans*
Z0023_RO_A04_1_E08_B	75	115	LMG 4051	*Deinococcus*	*radiodurans*
Z0022_RO_F07_2_G10_A	76	112	LMG 4008	*Flavobacterium*	*aquatile*
Z0022_LO_G03_1_H02_A	76	112	LMG 4008	*Flavobacterium*	*aquatile*
Z0022_LO_G06_1_H03_B	76	112	LMG 4008	*Flavobacterium*	*aquatile*
Z0022_RO_G11_1_H12_A	76	112	LMG 4008	*Flavobacterium*	*aquatile*
Z0022_RO_H02_2_H07_B	76	112	LMG 4008	*Flavobacterium*	*aquatile*
Z0022_RO_H03_2_H08_A	76	112	LMG 4008	*Flavobacterium*	*aquatile*
Z0022_RO_H04_2_H08_B	76	112	LMG 4008	*Flavobacterium*	*aquatile*
Z0022_RB_A01_2_D12_A	76	112	LMG 4008	*Flavobacterium*	*aquatile*
Z0022_RO_G09_1_H11_A	76	112	LMG 4008	*Flavobacterium*	*aquatile*
Z0022_RO_H01_2_H07_A	76	112	LMG 4008	*Flavobacterium*	*aquatile*
Z0022_LO_H11_2_H06_A	76	112	LMG 4008	*Flavobacterium*	*aquatile*
Z0022_LO_H12_2_H06_B	76	112	LMG 4008	*Flavobacterium*	*aquatile*
Z0022_LO_H08_2_H04_B	76	112	LMG 4008	*Flavobacterium*	*aquatile*
Z0022_RO_F12_2_G12_B	77	112	LMG 4008	*Flavobacterium*	*aquatile*
Z0022_LO_G07_1_H04_A	77	112	LMG 4008	*Flavobacterium*	*aquatile*
Z0022_LO_G11_1_H06_A	77	112	LMG 4008	*Flavobacterium*	*aquatile*
Z0022_LO_H06_2_H03_B	77	112	LMG 4008	*Flavobacterium*	*aquatile*
Z0022_LO_G12_1_H06_B	77	112	LMG 4008	*Flavobacterium*	*aquatile*
Z0022_RO_G06_1_H09_B	77	112	LMG 4008	*Flavobacterium*	*aquatile*
Z0022_RO_G01_1_H07_A	77	112	LMG 4008	*Flavobacterium*	*aquatile*
Z0022_LO_G08_1_H04_B	77	112	LMG 4008	*Flavobacterium*	*aquatile*
Z0022_RO_G08_1_H10_B	77	112	LMG 4008	*Flavobacterium*	*aquatile*
Z0022_RO_G04_1_H08_B	77	112	LMG 4008	*Flavobacterium*	*aquatile*
Z0022_RO_G05_1_H09_A	77	112	LMG 4008	*Flavobacterium*	*aquatile*
Z0022_RO_G07_1_H10_A	77	112	LMG 4008	*Flavobacterium*	*aquatile*
Z0022_LO_G05_1_H03_A	77	112	LMG 4008	*Flavobacterium*	*aquatile*
Z0022_LO_H10_2_H05_B	77	112	LMG 4008	*Flavobacterium*	*aquatile*

Z0022_LO_H09_2_H05_A	77	112	LMG 4008	*Flavobacterium*	*aquatile*
Z0022_RO_G10_1_H11_B	77	112	LMG 4008	*Flavobacterium*	*aquatile*
Z0022_RO_G02_1_H07_B	77	112	LMG 4008	*Flavobacterium*	*aquatile*
Z0022_RO_G12_1_H12_B	77	112	LMG 4008	*Flavobacterium*	*aquatile*
Z0022_LO_H07_2_H04_A	77	112	LMG 4008	*Flavobacterium*	*aquatile*
Z0025_RO_E10_1_G11_B	78	140	LMG 9210	*Lactobacillus*	*pentosus*
Z0025_LO_F11_2_G06_A	78	140	LMG 9210	*Lactobacillus*	*pentosus*
Z0025_RO_D09_2_F11_A	78	140	LMG 9210	*Lactobacillus*	*pentosus*
Z0025_LO_F01_2_G01_A	78	140	LMG 9210	*Lactobacillus*	*pentosus*
Z0025_LO_E12_1_G06_B	78	140	LMG 9210	*Lactobacillus*	*pentosus*
Z0025_LO_F06_2_G03_B	78	140	LMG 9210	*Lactobacillus*	*pentosus*
Z0025_LO_E01_1_G01_A	78	140	LMG 9210	*Lactobacillus*	*pentosus*
Z0025_RO_E11_1_G12_A	78	140	LMG 9210	*Lactobacillus*	*pentosus*
Z0025_LO_F10_2_G05_B	78	140	LMG 9210	*Lactobacillus*	*pentosus*
Z0025_RO_D08_2_F10_B	78	140	LMG 9210	*Lactobacillus*	*pentosus*
Z0025_LO_E03_1_G02_A	78	140	LMG 9210	*Lactobacillus*	*pentosus*
Z0025_RO_F02_2_G07_B	78	140	LMG 9210	*Lactobacillus*	*pentosus*
Z0025_LO_G03_1_H02_A	78	140	LMG 9210	*Lactobacillus*	*pentosus*
Z0025_LO_G05_1_H03_A	78	140	LMG 9210	*Lactobacillus*	*pentosus*
Z0025_LO_G02_1_H01_B	78	140	LMG 9210	*Lactobacillus*	*pentosus*
Z0025_RO_E05_1_G09_A	78	140	LMG 9210	*Lactobacillus*	*pentosus*
Z0025_LO_F08_2_G04_B	78	140	LMG 9210	*Lactobacillus*	*pentosus*
Z0025_LO_F09_2_G05_A	78	140	LMG 9210	*Lactobacillus*	*pentosus*
Z0025_RO_D06_2_F09_B	78	140	LMG 9210	*Lactobacillus*	*pentosus*
Z0025_RO_D05_2_F09_A	78	140	LMG 9210	*Lactobacillus*	*pentosus*
Z0025_RO_D04_2_F08_B	78	140	LMG 9210	*Lactobacillus*	*pentosus*
Z0025_LO_E09_1_G05_A	78	140	LMG 9210	*Lactobacillus*	*pentosus*
Z0025_LO_E02_1_G01_B	78	140	LMG 9210	*Lactobacillus*	*pentosus*
Z0025_LO_E04_1_G02_B	78	140	LMG 9210	*Lactobacillus*	*pentosus*
Z0025_LO_G07_1_H04_A	78	140	LMG 9210	*Lactobacillus*	*pentosus*
Z0025_LO_F02_2_G01_B	78	140	LMG 9210	*Lactobacillus*	*pentosus*
Z0025_LO_G09_1_H05_A	78	140	LMG 9210	*Lactobacillus*	*pentosus*
Z0025_LO_G08_1_H04_B	78	140	LMG 9210	*Lactobacillus*	*pentosus*
Z0025_LO_F03_2_G02_A	78	140	LMG 9210	*Lactobacillus*	*pentosus*
Z0025_RO_E02_1_G07_B	78	140	LMG 9210	*Lactobacillus*	*pentosus*
Z0025_LO_F05_2_G03_A	78	140	LMG 9210	*Lactobacillus*	*pentosus*
Z0014_RB_H07_1_A09_A	79	24	LMG 18401	*Lactobacillus*	*pentosus*
Z0014_RB_H09_1_A08_A	79	24	LMG 18401	*Lactobacillus*	*pentosus*
Z0014_RB_H08_1_A09_B	79	24	LMG 18401	*Lactobacillus*	*pentosus*
Z0014_RB_H06_1_A10_B	79	24	LMG 18401	*Lactobacillus*	*pentosus*
Z0014_LB_G10_2_A02_B	79	24	LMG 18401	*Lactobacillus*	*pentosus*
Z0014_LB_H05_1_A04_A	79	24	LMG 18401	*Lactobacillus*	*pentosus*
Z0014_RO_A01_1_E07_A	79	24	LMG 18401	*Lactobacillus*	*pentosus*
Z0014_LB_H03_1_A05_A	79	24	LMG 18401	*Lactobacillus*	*pentosus*
Z0014_RB_H11_1_A07_A	79	24	LMG 18401	*Lactobacillus*	*pentosus*
Z0014_RB_H12_1_A07_B	79	24	LMG 18401	*Lactobacillus*	*pentosus*
Z0014_RO_A04_1_E08_B	79	24	LMG 18401	*Lactobacillus*	*pentosus*
Z0014_RO_A05_1_E09_A	79	24	LMG 18401	*Lactobacillus*	*pentosus*
Z0014_RO_A06_1_E09_B	79	24	LMG 18401	*Lactobacillus*	*pentosus*
Z0014_RB_H04_1_A11_B	79	24	LMG 18401	*Lactobacillus*	*pentosus*

Z0014_LB_G11_2_A01_A	79	24	LMG 18401	*Lactobacillus*	*pentosus*
Z0014_RB_H05_1_A10_A	79	24	LMG 18401	*Lactobacillus*	*pentosus*
Z0014_RO_A07_1_E10_A	79	24	LMG 18401	*Lactobacillus*	*pentosus*
Z0014_RB_G09_2_A08_A	79	24	LMG 18401	*Lactobacillus*	*pentosus*
Z0014_RB_G11_2_A07_A	79	24	LMG 18401	*Lactobacillus*	*pentosus*
Z0014_RB_G10_2_A08_B	79	24	LMG 18401	*Lactobacillus*	*pentosus*
Z0014_RB_G12_2_A07_B	79	24	LMG 18401	*Lactobacillus*	*pentosus*
Z0014_LB_H04_1_A05_B	79	24	LMG 18401	*Lactobacillus*	*pentosus*
Z0014_RO_A02_1_E07_B	79	24	LMG 18401	*Lactobacillus*	*pentosus*
Z0014_RO_A03_1_E08_A	79	24	LMG 18401	*Lactobacillus*	*pentosus*
Z0014_RB_H01_1_A12_A	79	24	LMG 18401	*Lactobacillus*	*pentosus*
Z0014_LB_G09_2_A02_A	79	24	LMG 18401	*Lactobacillus*	*pentosus*
Z0014_RB_H02_1_A12_B	79	24	LMG 18401	*Lactobacillus*	*pentosus*
Z0014_RB_H03_1_A11_A	79	24	LMG 18401	*Lactobacillus*	*pentosus*
Z0014_RB_H10_1_A08_B	79	24	LMG 18401	*Lactobacillus*	*pentosus*
Z0014_LB_H02_1_A06_B	79	24	LMG 18401	*Lactobacillus*	*pentosus*
Z0014_LB_G12_2_A01_B	79	24	LMG 18401	*Lactobacillus*	*pentosus*
Z0014_LB_H01_1_A06_A	79	24	LMG 18401	*Lactobacillus*	*pentosus*
Z0025_LO_G06_1_H03_B	80	140	LMG 9210	*Lactobacillus*	*pentosus*
Z0013_RO_H04_2_H08_B	81	24	LMG 17677	*Lactobacillus*	*pentosus*
Z0013_RO_H03_2_H08_A	81	24	LMG 17677	*Lactobacillus*	*pentosus*
Z0013_RB_H05_1_A10_A	81	24	LMG 17677	*Lactobacillus*	*pentosus*
Z0013_LO_H07_2_H04_A	81	24	LMG 17677	*Lactobacillus*	*pentosus*
Z0013_RO_G06_1_H09_B	81	24	LMG 17677	*Lactobacillus*	*pentosus*
Z0013_RB_H01_1_A12_A	81	24	LMG 17677	*Lactobacillus*	*pentosus*
Z0013_LB_H02_1_A06_B	81	24	LMG 17677	*Lactobacillus*	*pentosus*
Z0013_LB_H03_1_A05_A	81	24	LMG 17677	*Lactobacillus*	*pentosus*
Z0013_RB_G10_2_A08_B	81	24	LMG 17677	*Lactobacillus*	*pentosus*
Z0013_RB_H04_1_A11_B	81	24	LMG 17677	*Lactobacillus*	*pentosus*
Z0013_LB_H08_1_A03_B	81	24	LMG 17677	*Lactobacillus*	*pentosus*
Z0013_LO_H03_2_H02_A	81	24	LMG 17677	*Lactobacillus*	*pentosus*
Z0013_LB_H05_1_A04_A	81	24	LMG 17677	*Lactobacillus*	*pentosus*
Z0013_LB_G12_2_A01_B	81	24	LMG 17677	*Lactobacillus*	*pentosus*
Z0013_LB_H07_1_A03_A	81	24	LMG 17677	*Lactobacillus*	*pentosus*
Z0013_RB_G11_2_A07_A	81	24	LMG 17677	*Lactobacillus*	*pentosus*
Z0013_LB_H01_1_A06_A	81	24	LMG 17677	*Lactobacillus*	*pentosus*
Z0013_RB_G05_2_A10_A	81	24	LMG 17677	*Lactobacillus*	*pentosus*
Z0013_LO_H01_2_H01_A	81	24	LMG 17677	*Lactobacillus*	*pentosus*
Z0013_LO_H05_2_H03_A	81	24	LMG 17677	*Lactobacillus*	*pentosus*
Z0013_LO_H06_2_H03_B	81	24	LMG 17677	*Lactobacillus*	*pentosus*
Z0013_RB_H03_1_A11_A	81	24	LMG 17677	*Lactobacillus*	*pentosus*
Z0013_LO_G11_1_H06_A	81	24	LMG 17677	*Lactobacillus*	*pentosus*
Z0013_LB_G10_2_A02_B	81	24	LMG 17677	*Lactobacillus*	*pentosus*
Z0013_RO_G05_1_H09_A	81	24	LMG 17677	*Lactobacillus*	*pentosus*
Z0013_RB_G03_2_A11_A	81	24	LMG 17677	*Lactobacillus*	*pentosus*
Z0013_RB_H06_1_A10_B	81	24	LMG 17677	*Lactobacillus*	*pentosus*
Z0013_RO_H01_2_H07_A	81	24	LMG 17677	*Lactobacillus*	*pentosus*
Z0013_LO_H08_2_H04_B	81	24	LMG 17677	*Lactobacillus*	*pentosus*
Z0013_LO_H04_2_H02_B	81	24	LMG 17677	*Lactobacillus*	*pentosus*
Z0013_LB_H06_1_A04_B	81	24	LMG 17677	*Lactobacillus*	*pentosus*

Z0013_RO_H02_2_H07_B	81	24	LMG 17677	*Lactobacillus*	*pentosus*
Z0015_RB_D12_1_C07_B	82	36	LMG 21311	*Gluconacetobacter*	*azotocaptans*
Z0015_RB_D09_1_C08_A	82	36	LMG 21311	*Gluconacetobacter*	*azotocaptans*
Z0015_RB_D10_1_C08_B	82	36	LMG 21311	*Gluconacetobacter*	*azotocaptans*
Z0015_RB_D11_1_C07_A	82	36	LMG 21311	*Gluconacetobacter*	*azotocaptans*
Z0015_RB_C08_2_C09_B	82	36	LMG 21311	*Gluconacetobacter*	*azotocaptans*
Z0015_LB_E05_2_B04_A	82	36	LMG 21311	*Gluconacetobacter*	*azotocaptans*
Z0015_RB_D04_1_C11_B	82	36	LMG 21311	*Gluconacetobacter*	*azotocaptans*
Z0015_LB_E06_2_B04_B	82	36	LMG 21311	*Gluconacetobacter*	*azotocaptans*
Z0015_LB_F02_1_B06_B	82	36	LMG 21311	*Gluconacetobacter*	*azotocaptans*
Z0015_LB_E04_2_B05_B	82	36	LMG 21311	*Gluconacetobacter*	*azotocaptans*
Z0015_RB_D03_1_C11_A	82	36	LMG 21311	*Gluconacetobacter*	*azotocaptans*
Z0015_LB_F01_1_B06_A	82	36	LMG 21311	*Gluconacetobacter*	*azotocaptans*
Z0015_RB_C11_2_C07_A	82	36	LMG 21311	*Gluconacetobacter*	*azotocaptans*
Z0015_LB_E11_2_B01_A	82	36	LMG 21311	*Gluconacetobacter*	*azotocaptans*
Z0015_RB_C09_2_C08_A	82	36	LMG 21311	*Gluconacetobacter*	*azotocaptans*
Z0015_RB_C12_2_C07_B	82	36	LMG 21311	*Gluconacetobacter*	*azotocaptans*
Z0015_LB_F03_1_B05_A	82	36	LMG 21311	*Gluconacetobacter*	*azotocaptans*
Z0015_LB_F04_1_B05_B	82	36	LMG 21311	*Gluconacetobacter*	*azotocaptans*
Z0015_RB_D01_1_C12_A	82	36	LMG 21311	*Gluconacetobacter*	*azotocaptans*
Z0015_RB_C10_2_C08_B	82	36	LMG 21311	*Gluconacetobacter*	*azotocaptans*
Z0015_LB_E10_2_B02_B	82	36	LMG 21311	*Gluconacetobacter*	*azotocaptans*
Z0015_LB_F05_1_B04_A	82	36	LMG 21311	*Gluconacetobacter*	*azotocaptans*
Z0015_RB_D06_1_C10_B	82	36	LMG 21311	*Gluconacetobacter*	*azotocaptans*
Z0015_LB_E07_2_B03_A	82	36	LMG 21311	*Gluconacetobacter*	*azotocaptans*
Z0015_RB_D08_1_C09_B	82	36	LMG 21311	*Gluconacetobacter*	*azotocaptans*
Z0015_LB_E08_2_B03_B	82	36	LMG 21311	*Gluconacetobacter*	*azotocaptans*
Z0015_RB_D07_1_C09_A	82	36	LMG 21311	*Gluconacetobacter*	*azotocaptans*
Z0015_RB_D02_1_C12_B	82	36	LMG 21311	*Gluconacetobacter*	*azotocaptans*
Z0015_LB_E09_2_B02_A	82	36	LMG 21311	*Gluconacetobacter*	*azotocaptans*
Z0015_RB_D05_1_C10_A	82	36	LMG 21311	*Gluconacetobacter*	*azotocaptans*
Z0015_LB_E12_2_B01_B	82	36	LMG 21311	*Gluconacetobacter*	*azotocaptans*
Z0015_LB_F06_1_B04_B	83	36	LMG 21311	*Gluconacetobacter*	*azotocaptans*
Z0022_RO_E11_1_G12_A	84	111	LMG 3897	*Listonella*	*pelagia*
Z0022_LO_F10_2_G05_B	84	111	LMG 3897	*Listonella*	*pelagia*
Z0022_RO_E09_1_G11_A	84	111	LMG 3897	*Listonella*	*pelagia*
Z0022_RO_C09_1_F11_A	84	111	LMG 3897	*Listonella*	*pelagia*
Z0022_RO_F01_2_G07_A	84	111	LMG 3897	*Listonella*	*pelagia*
Z0022_LO_F12_2_G06_B	84	111	LMG 3897	*Listonella*	*pelagia*
Z0022_LO_G01_1_H01_A	84	111	LMG 3897	*Listonella*	*pelagia*
Z0022_RO_E03_1_G08_A	84	111	LMG 3897	*Listonella*	*pelagia*
Z0022_RO_E02_1_G07_B	84	111	LMG 3897	*Listonella*	*pelagia*
Z0022_LO_E12_1_G06_B	84	111	LMG 3897	*Listonella*	*pelagia*
Z0022_RO_C12_1_F12_B	84	111	LMG 3897	*Listonella*	*pelagia*
Z0022_RO_E01_1_G07_A	84	111	LMG 3897	*Listonella*	*pelagia*
Z0022_RO_C11_1_F12_A	84	111	LMG 3897	*Listonella*	*pelagia*
Z0022_RO_C10_1_F11_B	84	111	LMG 3897	*Listonella*	*pelagia*
Z0022_RO_E10_1_G11_B	84	111	LMG 3897	*Listonella*	*pelagia*
Z0022_LO_G02_1_H01_B	84	111	LMG 3897	*Listonella*	*pelagia*
Z0022_RO_E07_1_G10_A	84	111	LMG 3897	*Listonella*	*pelagia*

Z0022_RO_D05_2_F09_A	84	111	LMG 3897	*Listonella*	*pelagia*
Z0022_LO_E03_1_G02_A	84	111	LMG 3897	*Listonella*	*pelagia*
Z0022_RO_D08_2_F10_B	84	111	LMG 3897	*Listonella*	*pelagia*
Z0022_RO_E06_1_G09_B	84	111	LMG 3897	*Listonella*	*pelagia*
Z0022_RO_D02_2_F07_B	84	111	LMG 3897	*Listonella*	*pelagia*
Z0022_LO_E07_1_G04_A	84	111	LMG 3897	*Listonella*	*pelagia*
Z0022_LO_D09_2_F05_A	84	111	LMG 3897	*Listonella*	*pelagia*
Z0022_RO_D06_2_F09_B	84	111	LMG 3897	*Listonella*	*pelagia*
Z0022_RO_E08_1_G10_B	84	111	LMG 3897	*Listonella*	*pelagia*
Z0022_RO_E05_1_G09_A	84	111	LMG 3897	*Listonella*	*pelagia*
Z0022_LO_D12_2_F06_B	84	111	LMG 3897	*Listonella*	*pelagia*
Z0022_RO_D09_2_F11_A	84	111	LMG 3897	*Listonella*	*pelagia*
Z0022_RO_D03_2_F08_A	84	111	LMG 3897	*Listonella*	*pelagia*
Z0022_LO_E06_1_G03_B	84	111	LMG 3897	*Listonella*	*pelagia*
Z0022_RO_D12_2_F12_B	84	111	LMG 3897	*Listonella*	*pelagia*
Z0018_RO_B05_2_E09_A	85	73	LMG 24552	*Roseomonas*	*gilardii*
Z0018_RO_B07_2_E10_A	85	73	LMG 24552	*Roseomonas*	*gilardii*
Z0018_LO_C08_1_F04_B	85	73	LMG 24552	*Roseomonas*	*gilardii*
Z0018_RO_B12_2_E12_B	85	73	LMG 24552	*Roseomonas*	*gilardii*
Z0018_LO_C05_1_F03_A	85	73	LMG 24552	*Roseomonas*	*gilardii*
Z0018_LO_C07_1_F04_A	85	73	LMG 24552	*Roseomonas*	*gilardii*
Z0018_LO_C06_1_F03_B	85	73	LMG 24552	*Roseomonas*	*gilardii*
Z0018_RO_C05_1_F09_A	85	73	LMG 24552	*Roseomonas*	*gilardii*
Z0018_LO_C10_1_F05_B	85	73	LMG 24552	*Roseomonas*	*gilardii*
Z0018_RO_C04_1_F08_B	85	73	LMG 24552	*Roseomonas*	*gilardii*
Z0018_RO_B06_2_E09_B	85	73	LMG 24552	*Roseomonas*	*gilardii*
Z0018_RO_B04_2_E08_B	85	73	LMG 24552	*Roseomonas*	*gilardii*
Z0018_LO_C01_1_F01_A	85	73	LMG 24552	*Roseomonas*	*gilardii*
Z0018_RO_A10_1_E11_B	85	73	LMG 24552	*Roseomonas*	*gilardii*
Z0018_LO_B04_2_E02_B	85	73	LMG 24552	*Roseomonas*	*gilardii*
Z0018_LO_B05_2_E03_A	85	73	LMG 24552	*Roseomonas*	*gilardii*
Z0018_LO_B03_2_E02_A	85	73	LMG 24552	*Roseomonas*	*gilardii*
Z0018_RO_B09_2_E11_A	85	73	LMG 24552	*Roseomonas*	*gilardii*
Z0018_RO_B11_2_E12_A	85	73	LMG 24552	*Roseomonas*	*gilardii*
Z0018_LO_B06_2_E03_B	85	73	LMG 24552	*Roseomonas*	*gilardii*
Z0018_LO_B09_2_E05_A	85	73	LMG 24552	*Roseomonas*	*gilardii*
Z0018_LO_B07_2_E04_A	85	73	LMG 24552	*Roseomonas*	*gilardii*
Z0018_LO_B08_2_E04_B	85	73	LMG 24552	*Roseomonas*	*gilardii*
Z0018_LO_C03_1_F02_A	85	73	LMG 24552	*Roseomonas*	*gilardii*
Z0018_RO_C09_1_F11_A	86	73	LMG 24552	*Roseomonas*	*gilardii*
Z0018_LO_D01_2_F01_A	86	73	LMG 24552	*Roseomonas*	*gilardii*
Z0018_RO_C08_1_F10_B	86	73	LMG 24552	*Roseomonas*	*gilardii*
Z0018_LO_C11_1_F06_A	86	73	LMG 24552	*Roseomonas*	*gilardii*
Z0014_RB_E05_2_B10_A	87	27	LMG 18397	*Anoxybacillus*	*flavithermus*
Z0014_LB_E10_2_B02_B	87	27	LMG 18397	*Anoxybacillus*	*flavithermus*
Z0014_LB_E08_2_B03_B	87	27	LMG 18397	*Anoxybacillus*	*flavithermus*
Z0014_LB_E11_2_B01_A	87	27	LMG 18397	*Anoxybacillus*	*flavithermus*
Z0014_RB_E12_2_B07_B	87	27	LMG 18397	*Anoxybacillus*	*flavithermus*
Z0014_LB_D10_1_C02_B	87	27	LMG 18397	*Anoxybacillus*	*flavithermus*
Z0014_RB_E04_2_B11_B	87	27	LMG 18397	*Anoxybacillus*	*flavithermus*

Z0014_LB_F02_1_B06_B	87	27	LMG 18397	*Anoxybacillus*	*flavithermus*
Z0014_LB_E06_2_B04_B	87	27	LMG 18397	*Anoxybacillus*	*flavithermus*
Z0014_RB_E10_2_B08_B	87	27	LMG 18397	*Anoxybacillus*	*flavithermus*
Z0014_LB_E12_2_B01_B	87	27	LMG 18397	*Anoxybacillus*	*flavithermus*
Z0014_RB_F05_1_B10_A	87	27	LMG 18397	*Anoxybacillus*	*flavithermus*
Z0014_RB_F02_1_B12_B	87	27	LMG 18397	*Anoxybacillus*	*flavithermus*
Z0014_LB_E03_2_B05_A	87	27	LMG 18397	*Anoxybacillus*	*flavithermus*
Z0014_RB_E06_2_B10_B	87	27	LMG 18397	*Anoxybacillus*	*flavithermus*
Z0014_LB_E04_2_B05_B	87	27	LMG 18397	*Anoxybacillus*	*flavithermus*
Z0014_LB_D12_1_C01_B	87	27	LMG 18397	*Anoxybacillus*	*flavithermus*
Z0014_LB_D11_1_C01_A	87	27	LMG 18397	*Anoxybacillus*	*flavithermus*
Z0014_RB_F04_1_B11_B	87	27	LMG 18397	*Anoxybacillus*	*flavithermus*
Z0014_RB_F01_1_B12_A	87	27	LMG 18397	*Anoxybacillus*	*flavithermus*
Z0014_RB_F03_1_B11_A	87	27	LMG 18397	*Anoxybacillus*	*flavithermus*
Z0014_LB_F01_1_B06_A	87	27	LMG 18397	*Anoxybacillus*	*flavithermus*
Z0014_LB_F03_1_B05_A	87	27	LMG 18397	*Anoxybacillus*	*flavithermus*
Z0014_LB_E09_2_B02_A	87	27	LMG 18397	*Anoxybacillus*	*flavithermus*
Z0014_RB_E07_2_B09_A	87	27	LMG 18397	*Anoxybacillus*	*flavithermus*
Z0014_LB_E05_2_B04_A	87	27	LMG 18397	*Anoxybacillus*	*flavithermus*
Z0014_LB_E01_2_B06_A	87	27	LMG 18397	*Anoxybacillus*	*flavithermus*
Z0014_RB_E09_2_B08_A	87	27	LMG 18397	*Anoxybacillus*	*flavithermus*
Z0014_LB_E02_2_B06_B	87	27	LMG 18397	*Anoxybacillus*	*flavithermus*
Z0014_RB_E08_2_B09_B	87	27	LMG 18397	*Anoxybacillus*	*flavithermus*
Z0014_RB_E11_2_B07_A	88	27	LMG 18397	*Anoxybacillus*	*flavithermus*
Z0014_LB_E07_2_B03_A	88	27	LMG 18397	*Anoxybacillus*	*flavithermus*
Z0019_RB_H12_1_A07_B	89	88	LMG 26187	*Halomonas*	*ventosae*
Z0019_LO_C09_1_F05_A	89	88	LMG 26187	*Halomonas*	*ventosae*
Z0019_LO_C01_1_F01_A	89	88	LMG 26187	*Halomonas*	*ventosae*
Z0019_RB_H11_1_A07_A	89	88	LMG 26187	*Halomonas*	*ventosae*
Z0019_LO_B12_2_E06_B	89	88	LMG 26187	*Halomonas*	*ventosae*
Z0019_RO_A02_1_E07_B	89	88	LMG 26187	*Halomonas*	*ventosae*
Z0019_LO_B11_2_E06_A	89	88	LMG 26187	*Halomonas*	*ventosae*
Z0019_RO_C07_1_F10_A	89	88	LMG 26187	*Halomonas*	*ventosae*
Z0019_RB_H10_1_A08_B	89	88	LMG 26187	*Halomonas*	*ventosae*
Z0019_RO_A01_1_E07_A	89	88	LMG 26187	*Halomonas*	*ventosae*
Z0019_RO_C12_1_F12_B	89	88	LMG 26187	*Halomonas*	*ventosae*
Z0019_LO_B08_2_E04_B	89	88	LMG 26187	*Halomonas*	*ventosae*
Z0019_LO_B09_2_E05_A	89	88	LMG 26187	*Halomonas*	*ventosae*
Z0019_LO_B10_2_E05_B	89	88	LMG 26187	*Halomonas*	*ventosae*
Z0019_LO_B06_2_E03_B	89	88	LMG 26187	*Halomonas*	*ventosae*
Z0019_RO_A12_1_E12_B	89	88	LMG 26187	*Halomonas*	*ventosae*
Z0019_RO_D05_2_F09_A	89	88	LMG 26187	*Halomonas*	*ventosae*
Z0019_LO_D04_2_F02_B	89	88	LMG 26187	*Halomonas*	*ventosae*
Z0019_RO_C06_1_F09_B	89	88	LMG 26187	*Halomonas*	*ventosae*
Z0019_LO_D03_2_F02_A	89	88	LMG 26187	*Halomonas*	*ventosae*
Z0019_LO_D02_2_F01_B	89	88	LMG 26187	*Halomonas*	*ventosae*
Z0019_LO_D01_2_F01_A	89	88	LMG 26187	*Halomonas*	*ventosae*
Z0019_LO_D08_2_F04_B	89	88	LMG 26187	*Halomonas*	*ventosae*
Z0019_LO_D07_2_F04_A	89	88	LMG 26187	*Halomonas*	*ventosae*
Z0019_RB_H08_1_A09_B	90	88	LMG 26187	*Halomonas*	*ventosae*

Z0019_RB_H07_1_A09_A	90	88	LMG 26187	*Halomonas*	*ventosae*
Z0019_RB_H06_1_A10_B	90	88	LMG 26187	*Halomonas*	*ventosae*
Z0019_LO_C10_1_F05_B	90	88	LMG 26187	*Halomonas*	*ventosae*
Z0019_LO_C07_1_F04_A	90	88	LMG 26187	*Halomonas*	*ventosae*
Z0019_LO_A10_1_E05_B	90	88	LMG 26187	*Halomonas*	*ventosae*
Z0019_RO_A10_1_E11_B	90	88	LMG 26187	*Halomonas*	*ventosae*
Z0019_RO_B04_2_E08_B	90	88	LMG 26187	*Halomonas*	*ventosae*
Z0019_RB_A06_2_D10_B	91	78	LMG 25420	*Echinimonas*	*agarilytica*
Z0019_LB_B02_1_D06_B	91	78	LMG 25420	*Echinimonas*	*agarilytica*
Z0019_LB_B03_1_D05_A	91	78	LMG 25420	*Echinimonas*	*agarilytica*
Z0019_LB_B10_1_D02_B	91	78	LMG 25420	*Echinimonas*	*agarilytica*
Z0019_LB_B09_1_D02_A	91	78	LMG 25420	*Echinimonas*	*agarilytica*
Z0019_LB_B12_1_D01_B	91	78	LMG 25420	*Echinimonas*	*agarilytica*
Z0019_RB_A09_2_D08_A	92	78	LMG 25420	*Echinimonas*	*agarilytica*
Z0019_LB_B04_1_D05_B	92	78	LMG 25420	*Echinimonas*	*agarilytica*
Z0019_LB_B08_1_D03_B	92	78	LMG 25420	*Echinimonas*	*agarilytica*
Z0019_RB_B02_1_D12_B	92	78	LMG 25420	*Echinimonas*	*agarilytica*
Z0019_RB_A04_2_D11_B	92	78	LMG 25420	*Echinimonas*	*agarilytica*
Z0019_LB_B07_1_D03_A	92	78	LMG 25420	*Echinimonas*	*agarilytica*
Z0019_RB_A08_2_D09_B	92	78	LMG 25420	*Echinimonas*	*agarilytica*
Z0019_LB_B05_1_D04_A	92	78	LMG 25420	*Echinimonas*	*agarilytica*
Z0019_RB_A07_2_D09_A	92	78	LMG 25420	*Echinimonas*	*agarilytica*
Z0019_RB_B05_1_D10_A	92	78	LMG 25420	*Echinimonas*	*agarilytica*
Z0019_LB_B01_1_D06_A	92	78	LMG 25420	*Echinimonas*	*agarilytica*
Z0019_RB_B04_1_D11_B	92	78	LMG 25420	*Echinimonas*	*agarilytica*
Z0019_LB_C04_2_C05_B	92	78	LMG 25420	*Echinimonas*	*agarilytica*
Z0019_LB_C03_2_C05_A	92	78	LMG 25420	*Echinimonas*	*agarilytica*
Z0019_RB_A12_2_D07_B	92	78	LMG 25420	*Echinimonas*	*agarilytica*
Z0019_RB_B03_1_D11_A	92	78	LMG 25420	*Echinimonas*	*agarilytica*
Z0019_RB_A03_2_D11_A	92	78	LMG 25420	*Echinimonas*	*agarilytica*
Z0019_LB_B06_1_D04_B	92	78	LMG 25420	*Echinimonas*	*agarilytica*
Z0019_RB_A10_2_D08_B	92	78	LMG 25420	*Echinimonas*	*agarilytica*
Z0019_RB_A11_2_D07_A	92	78	LMG 25420	*Echinimonas*	*agarilytica*
Z0019_RB_A02_2_D12_B	92	78	LMG 25420	*Echinimonas*	*agarilytica*
Z0019_RB_A05_2_D10_A	92	78	LMG 25420	*Echinimonas*	*agarilytica*
Z0019_LB_C01_2_C06_A	92	78	LMG 25420	*Echinimonas*	*agarilytica*
Z0019_LB_C02_2_C06_B	92	78	LMG 25420	*Echinimonas*	*agarilytica*
Z0019_LB_B11_1_D01_A	92	78	LMG 25420	*Echinimonas*	*agarilytica*
Z0019_RB_B01_1_D12_A	92	78	LMG 25420	*Echinimonas*	*agarilytica*
Z0020_RB_C10_2_C08_B	93	90	LMG 26304	*Enterococcus*	*ureasiticus*
Z0020_LB_D06_1_C04_B	93	90	LMG 26304	*Enterococcus*	*ureasiticus*
Z0020_RB_C09_2_C08_A	93	90	LMG 26304	*Enterococcus*	*ureasiticus*
Z0020_RB_D01_1_C12_A	93	90	LMG 26304	*Enterococcus*	*ureasiticus*
Z0020_RB_D03_1_C11_A	93	90	LMG 26304	*Enterococcus*	*ureasiticus*
Z0020_RB_D02_1_C12_B	93	90	LMG 26304	*Enterococcus*	*ureasiticus*
Z0020_RB_C12_2_C07_B	93	90	LMG 26304	*Enterococcus*	*ureasiticus*
Z0020_LB_D09_1_C02_A	93	90	LMG 26304	*Enterococcus*	*ureasiticus*
Z0020_RB_C11_2_C07_A	93	90	LMG 26304	*Enterococcus*	*ureasiticus*
Z0020_LB_D08_1_C03_B	93	90	LMG 26304	*Enterococcus*	*ureasiticus*
Z0020_LB_D07_1_C03_A	93	90	LMG 26304	*Enterococcus*	*ureasiticus*

Z0020_LB_E02_2_B06_B	93	90	LMG 26304	*Enterococcus*	*ureasiticus*	
Z0020_LB_D12_1_C01_B	93	90	LMG 26304	*Enterococcus*	*ureasiticus*	
Z0020_LB_E06_2_B04_B	93	90	LMG 26304	*Enterococcus*	*ureasiticus*	
Z0020_RB_D07_1_C09_A	93	90	LMG 26304	*Enterococcus*	*ureasiticus*	
Z0020_LB_E01_2_B06_A	93	90	LMG 26304	*Enterococcus*	*ureasiticus*	
Z0020_RB_D11_1_C07_A	93	90	LMG 26304	*Enterococcus*	*ureasiticus*	
Z0020_RB_D04_1_C11_B	94	90	LMG 26304	*Enterococcus*	*ureasiticus*	
Z0020_RB_D05_1_C10_A	95	90	LMG 26304	*Enterococcus*	*ureasiticus*	
Z0020_LB_D10_1_C02_B	95	90	LMG 26304	*Enterococcus*	*ureasiticus*	
Z0020_LB_D11_1_C01_A	95	90	LMG 26304	*Enterococcus*	*ureasiticus*	
Z0020_LB_E05_2_B04_A	95	90	LMG 26304	*Enterococcus*	*ureasiticus*	
Z0020_RB_E03_2_B11_A	95	90	LMG 26304	*Enterococcus*	*ureasiticus*	
Z0020_RB_D12_1_C07_B	95	90	LMG 26304	*Enterococcus*	*ureasiticus*	
Z0020_RB_D06_1_C10_B	95	90	LMG 26304	*Enterococcus*	*ureasiticus*	
Z0020_RB_E01_2_B12_A	95	90	LMG 26304	*Enterococcus*	*ureasiticus*	
Z0020_LB_E04_2_B05_B	95	90	LMG 26304	*Enterococcus*	*ureasiticus*	
Z0020_RB_E02_2_B12_B	95	90	LMG 26304	*Enterococcus*	*ureasiticus*	
Z0020_RB_D10_1_C08_B	95	90	LMG 26304	*Enterococcus*	*ureasiticus*	
Z0020_RB_D08_1_C09_B	95	90	LMG 26304	*Enterococcus*	*ureasiticus*	
Z0020_RB_D09_1_C08_A	95	90	LMG 26304	*Enterococcus*	*ureasiticus*	
Z0020_LB_E03_2_B05_A	95	90	LMG 26304	*Enterococcus*	*ureasiticus*	
Z0024_RO_A10_1_E11_B	96	133	LMG 7529	*Desulfovibrio*	*desulfuricans*	*desulfuricans*
Z0024_LO_B04_2_E02_B	96	133	LMG 7529	*Desulfovibrio*	*desulfuricans*	*desulfuricans*
Z0024_LO_B02_2_E01_B	96	133	LMG 7529	*Desulfovibrio*	*desulfuricans*	*desulfuricans*
Z0024_LO_B01_2_E01_A	96	133	LMG 7529	*Desulfovibrio*	*desulfuricans*	*desulfuricans*
Z0024_RO_E06_1_G09_B	96	133	LMG 7529	*Desulfovibrio*	*desulfuricans*	*desulfuricans*
Z0024_LO_D02_2_F01_B	96	133	LMG 7529	*Desulfovibrio*	*desulfuricans*	*desulfuricans*
Z0024_RO_E08_1_G10_B	96	133	LMG 7529	*Desulfovibrio*	*desulfuricans*	*desulfuricans*
Z0024_RO_E04_1_G08_B	96	133	LMG 7529	*Desulfovibrio*	*desulfuricans*	*desulfuricans*
Z0024_RO_E10_1_G11_B	96	133	LMG 7529	*Desulfovibrio*	*desulfuricans*	*desulfuricans*
Z0024_LO_C03_1_F02_A	96	133	LMG 7529	*Desulfovibrio*	*desulfuricans*	*desulfuricans*
Z0024_RO_D05_2_F09_A	96	133	LMG 7529	*Desulfovibrio*	*desulfuricans*	*desulfuricans*
Z0024_RO_D06_2_F09_B	96	133	LMG 7529	*Desulfovibrio*	*desulfuricans*	*desulfuricans*
Z0024_LO_C02_1_F01_B	96	133	LMG 7529	*Desulfovibrio*	*desulfuricans*	*desulfuricans*
Z0024_LO_C01_1_F01_A	96	133	LMG 7529	*Desulfovibrio*	*desulfuricans*	*desulfuricans*
Z0024_RO_D03_2_F08_A	96	133	LMG 7529	*Desulfovibrio*	*desulfuricans*	*desulfuricans*
Z0024_RO_D04_2_F08_B	96	133	LMG 7529	*Desulfovibrio*	*desulfuricans*	*desulfuricans*
Z0024_LO_D06_2_F03_B	96	133	LMG 7529	*Desulfovibrio*	*desulfuricans*	*desulfuricans*
Z0024_LO_D04_2_F02_B	96	133	LMG 7529	*Desulfovibrio*	*desulfuricans*	*desulfuricans*
Z0024_RO_E09_1_G11_A	96	133	LMG 7529	*Desulfovibrio*	*desulfuricans*	*desulfuricans*
Z0024_RO_E11_1_G12_A	96	133	LMG 7529	*Desulfovibrio*	*desulfuricans*	*desulfuricans*
Z0024_RO_E07_1_G10_A	96	133	LMG 7529	*Desulfovibrio*	*desulfuricans*	*desulfuricans*
Z0024_LO_D09_2_F05_A	96	133	LMG 7529	*Desulfovibrio*	*desulfuricans*	*desulfuricans*
Z0024_RO_E03_1_G08_A	96	133	LMG 7529	*Desulfovibrio*	*desulfuricans*	*desulfuricans*
Z0024_RO_E05_1_G09_A	96	133	LMG 7529	*Desulfovibrio*	*desulfuricans*	*desulfuricans*
Z0024_RO_C05_1_F09_A	96	133	LMG 7529	*Desulfovibrio*	*desulfuricans*	*desulfuricans*
Z0024_RO_C04_1_F08_B	96	133	LMG 7529	*Desulfovibrio*	*desulfuricans*	*desulfuricans*
Z0024_RO_D02_2_F07_B	96	133	LMG 7529	*Desulfovibrio*	*desulfuricans*	*desulfuricans*
Z0024_LO_C11_1_F06_A	96	133	LMG 7529	*Desulfovibrio*	*desulfuricans*	*desulfuricans*
Z0024_RO_D01_2_F07_A	96	133	LMG 7529	*Desulfovibrio*	*desulfuricans*	*desulfuricans*

Z0024_RO_B04_2_E08_B	96	133	LMG 7529	*Desulfovibrio*	*desulfuricans*	*desulfuricans*
Z0024_LO_C07_1_F04_A	96	133	LMG 7529	*Desulfovibrio*	*desulfuricans*	*desulfuricans*
Z0024_RO_C12_1_F12_B	96	133	LMG 7529	*Desulfovibrio*	*desulfuricans*	*desulfuricans*
Z0023_RB_B02_1_D12_B	97	116	LMG 4233	*Photobacterium*	*phosphoreum*	
Z0023_RB_B07_1_D09_A	97	116	LMG 4233	*Photobacterium*	*phosphoreum*	
Z0023_LB_B10_1_D02_B	97	116	LMG 4233	*Photobacterium*	*phosphoreum*	
Z0023_RB_B12_1_D07_B	97	116	LMG 4233	*Photobacterium*	*phosphoreum*	
Z0023_RB_B04_1_D11_B	97	116	LMG 4233	*Photobacterium*	*phosphoreum*	
Z0023_RB_B11_1_D07_A	97	116	LMG 4233	*Photobacterium*	*phosphoreum*	
Z0023_LB_C03_2_C05_A	97	116	LMG 4233	*Photobacterium*	*phosphoreum*	
Z0023_LB_B12_1_D01_B	97	116	LMG 4233	*Photobacterium*	*phosphoreum*	
Z0023_LB_B09_1_D02_A	97	116	LMG 4233	*Photobacterium*	*phosphoreum*	
Z0023_RB_C01_2_C12_A	97	116	LMG 4233	*Photobacterium*	*phosphoreum*	
Z0023_RB_B09_1_D08_A	97	116	LMG 4233	*Photobacterium*	*phosphoreum*	
Z0023_RB_B01_1_D12_A	97	116	LMG 4233	*Photobacterium*	*phosphoreum*	
Z0023_LB_C02_2_C06_B	97	116	LMG 4233	*Photobacterium*	*phosphoreum*	
Z0023_RB_B03_1_D11_A	98	116	LMG 4233	*Photobacterium*	*phosphoreum*	
Z0023_RB_A10_2_D08_B	98	116	LMG 4233	*Photobacterium*	*phosphoreum*	
Z0023_RB_A11_2_D07_A	98	116	LMG 4233	*Photobacterium*	*phosphoreum*	
Z0023_LB_B05_1_D04_A	98	116	LMG 4233	*Photobacterium*	*phosphoreum*	
Z0023_RB_A12_2_D07_B	98	116	LMG 4233	*Photobacterium*	*phosphoreum*	
Z0023_LB_B07_1_D03_A	98	116	LMG 4233	*Photobacterium*	*phosphoreum*	
Z0023_LB_B11_1_D01_A	98	116	LMG 4233	*Photobacterium*	*phosphoreum*	
Z0023_RB_B06_1_D10_B	98	116	LMG 4233	*Photobacterium*	*phosphoreum*	
Z0023_LB_B06_1_D04_B	98	116	LMG 4233	*Photobacterium*	*phosphoreum*	
Z0023_RB_B05_1_D10_A	98	116	LMG 4233	*Photobacterium*	*phosphoreum*	
Z0023_RB_B08_1_D09_B	98	116	LMG 4233	*Photobacterium*	*phosphoreum*	
Z0023_RB_B10_1_D08_B	98	116	LMG 4233	*Photobacterium*	*phosphoreum*	
Z0023_LB_B08_1_D03_B	98	116	LMG 4233	*Photobacterium*	*phosphoreum*	
Z0023_RB_C02_2_C12_B	98	116	LMG 4233	*Photobacterium*	*phosphoreum*	
Z0023_LB_C06_2_C04_B	98	116	LMG 4233	*Photobacterium*	*phosphoreum*	
Z0023_LB_C05_2_C04_A	98	116	LMG 4233	*Photobacterium*	*phosphoreum*	
Z0023_LB_C07_2_C03_A	98	116	LMG 4233	*Photobacterium*	*phosphoreum*	
Z0023_LB_C01_2_C06_A	98	116	LMG 4233	*Photobacterium*	*phosphoreum*	
Z0023_LB_C04_2_C05_B	98	116	LMG 4233	*Photobacterium*	*phosphoreum*	
Z0013_RO_G09_1_H11_A	99	23	LMG 1668	*Acidomonas*	*methanolica*	
Z0013_LO_G03_1_H02_A	99	23	LMG 1668	*Acidomonas*	*methanolica*	
Z0013_RB_G12_2_A07_B	99	23	LMG 1668	*Acidomonas*	*methanolica*	
Z0013_LO_H02_2_H01_B	99	23	LMG 1668	*Acidomonas*	*methanolica*	
Z0013_LO_G04_1_H02_B	99	23	LMG 1668	*Acidomonas*	*methanolica*	
Z0013_RO_G10_1_H11_B	99	23	LMG 1668	*Acidomonas*	*methanolica*	
Z0013_LB_G04_2_A05_B	99	23	LMG 1668	*Acidomonas*	*methanolica*	
Z0013_RO_G03_1_H08_A	99	23	LMG 1668	*Acidomonas*	*methanolica*	
Z0013_LB_G05_2_A04_A	99	23	LMG 1668	*Acidomonas*	*methanolica*	
Z0013_RB_G02_2_A12_B	99	23	LMG 1668	*Acidomonas*	*methanolica*	
Z0013_LO_G06_1_H03_B	99	23	LMG 1668	*Acidomonas*	*methanolica*	
Z0013_RB_G04_2_A11_B	99	23	LMG 1668	*Acidomonas*	*methanolica*	
Z0013_LB_G08_2_A03_B	99	23	LMG 1668	*Acidomonas*	*methanolica*	
Z0013_LO_G08_1_H04_B	99	23	LMG 1668	*Acidomonas*	*methanolica*	
Z0013_RO_G04_1_H08_B	99	23	LMG 1668	*Acidomonas*	*methanolica*	

Z0013_RO_G11_1_H12_A	99	23	LMG 1668	*Acidomonas*	*methanolica*
Z0013_RB_G01_2_A12_A	99	23	LMG 1668	*Acidomonas*	*methanolica*
Z0013_RO_G12_1_H12_B	99	23	LMG 1668	*Acidomonas*	*methanolica*
Z0013_RB_H02_1_A12_B	99	23	LMG 1668	*Acidomonas*	*methanolica*
Z0013_LB_H04_1_A05_B	99	23	LMG 1668	*Acidomonas*	*methanolica*
Z0013_RB_G06_2_A10_B	99	23	LMG 1668	*Acidomonas*	*methanolica*
Z0013_LO_G10_1_H05_B	99	23	LMG 1668	*Acidomonas*	*methanolica*
Z0013_RB_F10_1_B08_B	99	23	LMG 1668	*Acidomonas*	*methanolica*
Z0013_LO_G01_1_H01_A	99	23	LMG 1668	*Acidomonas*	*methanolica*
Z0013_LO_F12_2_G06_B	99	23	LMG 1668	*Acidomonas*	*methanolica*
Z0013_RO_F11_2_G12_A	99	23	LMG 1668	*Acidomonas*	*methanolica*
Z0013_RO_G08_1_H10_B	99	23	LMG 1668	*Acidomonas*	*methanolica*
Z0013_LB_G11_2_A01_A	99	23	LMG 1668	*Acidomonas*	*methanolica*
Z0013_RB_G08_2_A09_B	99	23	LMG 1668	*Acidomonas*	*methanolica*
Z0013_RB_G09_2_A08_A	99	23	LMG 1668	*Acidomonas*	*methanolica*
Z0013_RO_G07_1_H10_A	99	23	LMG 1668	*Acidomonas*	*methanolica*
Z0013_RB_G07_2_A09_A	99	23	LMG 1668	*Acidomonas*	*methanolica*
Z0021_LB_A03_2_D05_A	100	97	LMG 27021	*Neokomagataea*	*thailandica*
Z0021_LB_A04_2_D05_B	100	97	LMG 27021	*Neokomagataea*	*thailandica*
Z0021_LO_H11_2_H06_A	100	97	LMG 27021	*Neokomagataea*	*thailandica*
Z0021_LO_H12_2_H06_B	100	97	LMG 27021	*Neokomagataea*	*thailandica*
Z0021_RB_A01_2_D12_A	100	97	LMG 27021	*Neokomagataea*	*thailandica*
Z0021_LB_A06_2_D04_B	100	97	LMG 27021	*Neokomagataea*	*thailandica*
Z0021_RB_A02_2_D12_B	100	97	LMG 27021	*Neokomagataea*	*thailandica*
Z0021_LB_A05_2_D04_A	100	97	LMG 27021	*Neokomagataea*	*thailandica*
Z0021_LO_A09_1_E05_A	100	97	LMG 27021	*Neokomagataea*	*thailandica*
Z0021_LO_H07_2_H04_A	100	97	LMG 27021	*Neokomagataea*	*thailandica*
Z0021_LB_A01_2_D06_A	100	97	LMG 27021	*Neokomagataea*	*thailandica*
Z0021_LO_H10_2_H05_B	100	97	LMG 27021	*Neokomagataea*	*thailandica*
Z0021_LO_B05_2_E03_A	100	97	LMG 27021	*Neokomagataea*	*thailandica*
Z0021_LO_B09_2_E05_A	100	97	LMG 27021	*Neokomagataea*	*thailandica*
Z0021_LO_C04_1_F02_B	100	97	LMG 27021	*Neokomagataea*	*thailandica*
Z0021_LO_B03_2_E02_A	100	97	LMG 27021	*Neokomagataea*	*thailandica*
Z0021_RO_C01_1_F07_A	101	97	LMG 27021	*Neokomagataea*	*thailandica*
Z0021_RO_B11_2_E12_A	101	97	LMG 27021	*Neokomagataea*	*thailandica*
Z0021_RO_C05_1_F09_A	101	97	LMG 27021	*Neokomagataea*	*thailandica*
Z0021_RB_A04_2_D11_B	101	97	LMG 27021	*Neokomagataea*	*thailandica*
Z0021_LO_A10_1_E05_B	101	97	LMG 27021	*Neokomagataea*	*thailandica*
Z0021_LO_H09_2_H05_A	101	97	LMG 27021	*Neokomagataea*	*thailandica*
Z0021_LB_A02_2_D06_B	101	97	LMG 27021	*Neokomagataea*	*thailandica*
Z0021_LO_H08_2_H04_B	101	97	LMG 27021	*Neokomagataea*	*thailandica*
Z0021_LB_A07_2_D03_A	101	97	LMG 27021	*Neokomagataea*	*thailandica*
Z0021_LB_A11_2_D01_A	101	97	LMG 27021	*Neokomagataea*	*thailandica*
Z0021_LB_A09_2_D02_A	101	97	LMG 27021	*Neokomagataea*	*thailandica*
Z0021_LB_A08_2_D03_B	101	97	LMG 27021	*Neokomagataea*	*thailandica*
Z0021_LB_A10_2_D02_B	101	97	LMG 27021	*Neokomagataea*	*thailandica*
Z0021_RO_B05_2_E09_A	101	97	LMG 27021	*Neokomagataea*	*thailandica*
Z0021_RB_A05_2_D10_A	101	97	LMG 27021	*Neokomagataea*	*thailandica*
Z0021_RB_A03_2_D11_A	101	97	LMG 27021	*Neokomagataea*	*thailandica*
Z0012_LO_H04_2_H02_B	102	10	LMG 12553	*Sphingobium*	*xanthum*

Z0012_RB_A01_2_D12_A	102	10	LMG 12553	*Sphingobium*	*xanthum*
Z0012_LO_H09_2_H05_A	102	10	LMG 12553	*Sphingobium*	*xanthum*
Z0012_RO_G09_1_H11_A	102	10	LMG 12553	*Sphingobium*	*xanthum*
Z0012_RB_A02_2_D12_B	102	10	LMG 12553	*Sphingobium*	*xanthum*
Z0012_RO_G12_1_H12_B	102	10	LMG 12553	*Sphingobium*	*xanthum*
Z0012_RO_G11_1_H12_A	102	10	LMG 12553	*Sphingobium*	*xanthum*
Z0012_RO_H04_2_H08_B	102	10	LMG 12553	*Sphingobium*	*xanthum*
Z0012_RO_G07_1_H10_A	102	10	LMG 12553	*Sphingobium*	*xanthum*
Z0012_RO_G08_1_H10_B	102	10	LMG 12553	*Sphingobium*	*xanthum*
Z0012_RB_A03_2_D11_A	102	10	LMG 12553	*Sphingobium*	*xanthum*
Z0012_LO_H03_2_H02_A	102	10	LMG 12553	*Sphingobium*	*xanthum*
Z0012_RO_G01_1_H07_A	102	10	LMG 12553	*Sphingobium*	*xanthum*
Z0012_LO_H05_2_H03_A	102	10	LMG 12553	*Sphingobium*	*xanthum*
Z0012_RO_F12_2_G12_B	102	10	LMG 12553	*Sphingobium*	*xanthum*
Z0012_LO_G12_1_H06_B	102	10	LMG 12553	*Sphingobium*	*xanthum*
Z0012_RO_F06_2_G09_B	102	10	LMG 12553	*Sphingobium*	*xanthum*
Z0012_RO_F07_2_G10_A	102	10	LMG 12553	*Sphingobium*	*xanthum*
Z0012_LO_G10_1_H05_B	102	10	LMG 12553	*Sphingobium*	*xanthum*
Z0012_LO_G11_1_H06_A	102	10	LMG 12553	*Sphingobium*	*xanthum*
Z0012_RO_H03_2_H08_A	102	10	LMG 12553	*Sphingobium*	*xanthum*
Z0012_RO_G10_1_H11_B	102	10	LMG 12553	*Sphingobium*	*xanthum*
Z0012_RO_H02_2_H07_B	102	10	LMG 12553	*Sphingobium*	*xanthum*
Z0012_LO_G07_1_H04_A	102	10	LMG 12553	*Sphingobium*	*xanthum*
Z0012_LO_G06_1_H03_B	102	10	LMG 12553	*Sphingobium*	*xanthum*
Z0012_LO_G05_1_H03_A	102	10	LMG 12553	*Sphingobium*	*xanthum*
Z0012_RO_H01_2_H07_A	102	10	LMG 12553	*Sphingobium*	*xanthum*
Z0012_RO_G02_1_H07_B	102	10	LMG 12553	*Sphingobium*	*xanthum*
Z0012_LO_H11_2_H06_A	102	10	LMG 12553	*Sphingobium*	*xanthum*
Z0012_LO_H10_2_H05_B	102	10	LMG 12553	*Sphingobium*	*xanthum*
Z0012_LO_H08_2_H04_B	102	10	LMG 12553	*Sphingobium*	*xanthum*
Z0012_LO_H12_2_H06_B	102	10	LMG 12553	*Sphingobium*	*xanthum*
Z0017_LB_A08_2_D03_B	103	55	LMG 23170	*Saccharibacter*	*floricola*
Z0017_LB_A09_2_D02_A	103	55	LMG 23170	*Saccharibacter*	*floricola*
Z0017_LO_H06_2_H03_B	103	55	LMG 23170	*Saccharibacter*	*floricola*
Z0017_LO_H07_2_H04_A	103	55	LMG 23170	*Saccharibacter*	*floricola*
Z0017_LO_G10_1_H05_B	103	55	LMG 23170	*Saccharibacter*	*floricola*
Z0017_LO_H05_2_H03_A	103	55	LMG 23170	*Saccharibacter*	*floricola*
Z0017_LB_A07_2_D03_A	103	55	LMG 23170	*Saccharibacter*	*floricola*
Z0017_LB_A06_2_D04_B	103	55	LMG 23170	*Saccharibacter*	*floricola*
Z0017_LB_A05_2_D04_A	103	55	LMG 23170	*Saccharibacter*	*floricola*
Z0017_RB_H10_1_A08_B	103	55	LMG 23170	*Saccharibacter*	*floricola*
Z0017_LB_A01_2_D06_A	103	55	LMG 23170	*Saccharibacter*	*floricola*
Z0017_LB_A02_2_D06_B	103	55	LMG 23170	*Saccharibacter*	*floricola*
Z0017_LB_A10_2_D02_B	103	55	LMG 23170	*Saccharibacter*	*floricola*
Z0017_LB_A11_2_D01_A	103	55	LMG 23170	*Saccharibacter*	*floricola*
Z0017_LO_H10_2_H05_B	103	55	LMG 23170	*Saccharibacter*	*floricola*
Z0017_LO_H02_2_H01_B	103	55	LMG 23170	*Saccharibacter*	*floricola*
Z0017_LO_H01_2_H01_A	103	55	LMG 23170	*Saccharibacter*	*floricola*
Z0017_LO_G11_1_H06_A	103	55	LMG 23170	*Saccharibacter*	*floricola*
Z0017_LO_H09_2_H05_A	103	55	LMG 23170	*Saccharibacter*	*floricola*

Z0017_LO_H11_2_H06_A	103	55	LMG 23170	*Saccharibacter*	*floricola*
Z0017_LO_G12_1_H06_B	103	55	LMG 23170	*Saccharibacter*	*floricola*
Z0017_LO_H08_2_H04_B	103	55	LMG 23170	*Saccharibacter*	*floricola*
Z0017_LB_A12_2_D01_B	103	55	LMG 23170	*Saccharibacter*	*floricola*
Z0017_LB_B01_1_D06_A	104	55	LMG 23170	*Saccharibacter*	*floricola*
Z0017_RO_A12_1_E12_B	105	55	LMG 23170	*Saccharibacter*	*floricola*
Z0017_RO_A10_1_E11_B	105	55	LMG 23170	*Saccharibacter*	*floricola*
Z0017_LO_B10_2_E05_B	105	55	LMG 23170	*Saccharibacter*	*floricola*
Z0017_RO_A06_1_E09_B	105	55	LMG 23170	*Saccharibacter*	*floricola*
Z0012_RB_F11_1_B07_A	106	13	LMG 13127	*Azospirillum*	*brasilense*
Z0012_RB_G08_2_A09_B	106	13	LMG 13127	*Azospirillum*	*brasilense*
Z0012_RB_F10_1_B08_B	106	13	LMG 13127	*Azospirillum*	*brasilense*
Z0012_RB_G04_2_A11_B	106	13	LMG 13127	*Azospirillum*	*brasilense*
Z0012_RB_G07_2_A09_A	106	13	LMG 13127	*Azospirillum*	*brasilense*
Z0012_LB_G02_2_A06_B	106	13	LMG 13127	*Azospirillum*	*brasilense*
Z0012_LB_G03_2_A05_A	107	13	LMG 13127	*Azospirillum*	*brasilense*
Z0012_LB_G04_2_A05_B	107	13	LMG 13127	*Azospirillum*	*brasilense*
Z0012_LB_G05_2_A04_A	107	13	LMG 13127	*Azospirillum*	*brasilense*
Z0012_LB_G08_2_A03_B	107	13	LMG 13127	*Azospirillum*	*brasilense*
Z0012_LB_G10_2_A02_B	107	13	LMG 13127	*Azospirillum*	*brasilense*
Z0012_RB_G02_2_A12_B	107	13	LMG 13127	*Azospirillum*	*brasilense*
Z0012_RB_G05_2_A10_A	107	13	LMG 13127	*Azospirillum*	*brasilense*
Z0012_RB_F12_1_B07_B	107	13	LMG 13127	*Azospirillum*	*brasilense*
Z0012_RB_H08_1_A09_B	107	13	LMG 13127	*Azospirillum*	*brasilense*
Z0012_RO_A02_1_E07_B	107	13	LMG 13127	*Azospirillum*	*brasilense*
Z0012_LB_H03_1_A05_A	107	13	LMG 13127	*Azospirillum*	*brasilense*
Z0012_RO_A01_1_E07_A	107	13	LMG 13127	*Azospirillum*	*brasilense*
Z0012_RB_H02_1_A12_B	107	13	LMG 13127	*Azospirillum*	*brasilense*
Z0012_LB_H05_1_A04_A	107	13	LMG 13127	*Azospirillum*	*brasilense*
Z0012_RB_H05_1_A10_A	107	13	LMG 13127	*Azospirillum*	*brasilense*
Z0012_RB_G12_2_A07_B	107	13	LMG 13127	*Azospirillum*	*brasilense*
Z0012_RB_G09_2_A08_A	107	13	LMG 13127	*Azospirillum*	*brasilense*
Z0012_RO_A05_1_E09_A	107	13	LMG 13127	*Azospirillum*	*brasilense*
Z0012_LB_H08_1_A03_B	107	13	LMG 13127	*Azospirillum*	*brasilense*
Z0012_RB_H12_1_A07_B	107	13	LMG 13127	*Azospirillum*	*brasilense*
Z0012_LB_H12_1_A01_B	107	13	LMG 13127	*Azospirillum*	*brasilense*
Z0012_RO_A07_1_E10_A	107	13	LMG 13127	*Azospirillum*	*brasilense*
Z0012_RO_B03_2_E08_A	107	13	LMG 13127	*Azospirillum*	*brasilense*
Z0012_RO_A12_1_E12_B	107	13	LMG 13127	*Azospirillum*	*brasilense*
Z0012_LB_H10_1_A02_B	107	13	LMG 13127	*Azospirillum*	*brasilense*
Z0012_RB_H10_1_A08_B	107	13	LMG 13127	*Azospirillum*	*brasilense*
Z0013_RO_C01_1_F07_A	108	17	LMG 1408	*Gluconobacter*	*oxydans*
Z0013_LO_B12_2_E06_B	108	17	LMG 1408	*Gluconobacter*	*oxydans*
Z0013_RB_B10_1_D08_B	108	17	LMG 1408	*Gluconobacter*	*oxydans*
Z0013_RB_B07_1_D09_A	108	17	LMG 1408	*Gluconobacter*	*oxydans*
Z0013_RB_B09_1_D08_A	108	17	LMG 1408	*Gluconobacter*	*oxydans*
Z0013_RB_B08_1_D09_B	108	17	LMG 1408	*Gluconobacter*	*oxydans*
Z0013_RO_B11_2_E12_A	108	17	LMG 1408	*Gluconobacter*	*oxydans*
Z0013_RO_B12_2_E12_B	108	17	LMG 1408	*Gluconobacter*	*oxydans*
Z0013_RO_B10_2_E11_B	108	17	LMG 1408	*Gluconobacter*	*oxydans*

Z0013_LO_B11_2_E06_A	108	17	LMG 1408	*Gluconobacter*	*oxydans*
Z0013_LB_B09_1_D02_A	108	17	LMG 1408	*Gluconobacter*	*oxydans*
Z0013_LB_B10_1_D02_B	108	17	LMG 1408	*Gluconobacter*	*oxydans*
Z0013_LO_C02_1_F01_B	108	17	LMG 1408	*Gluconobacter*	*oxydans*
Z0013_LB_B11_1_D01_A	108	17	LMG 1408	*Gluconobacter*	*oxydans*
Z0013_RO_C04_1_F08_B	108	17	LMG 1408	*Gluconobacter*	*oxydans*
Z0013_RO_C06_1_F09_B	108	17	LMG 1408	*Gluconobacter*	*oxydans*
Z0013_LO_C03_1_F02_A	108	17	LMG 1408	*Gluconobacter*	*oxydans*
Z0013_RO_C05_1_F09_A	108	17	LMG 1408	*Gluconobacter*	*oxydans*
Z0013_LB_B12_1_D01_B	108	17	LMG 1408	*Gluconobacter*	*oxydans*
Z0013_RO_C03_1_F08_A	108	17	LMG 1408	*Gluconobacter*	*oxydans*
Z0013_RO_B09_2_E11_A	109	17	LMG 1408	*Gluconobacter*	*oxydans*
Z0013_LO_B10_2_E05_B	109	17	LMG 1408	*Gluconobacter*	*oxydans*
Z0013_RO_B08_2_E10_B	109	17	LMG 1408	*Gluconobacter*	*oxydans*
Z0013_LO_B09_2_E05_A	109	17	LMG 1408	*Gluconobacter*	*oxydans*
Z0013_RO_C02_1_F07_B	110	17	LMG 1408	*Gluconobacter*	*oxydans*
Z0013_RB_B12_1_D07_B	110	17	LMG 1408	*Gluconobacter*	*oxydans*
Z0013_RB_B11_1_D07_A	110	17	LMG 1408	*Gluconobacter*	*oxydans*
Z0013_LO_C01_1_F01_A	110	17	LMG 1408	*Gluconobacter*	*oxydans*
Z0013_LO_C04_1_F02_B	111	17	LMG 1408	*Gluconobacter*	*oxydans*
Z0013_LO_C05_1_F03_A	111	17	LMG 1408	*Gluconobacter*	*oxydans*
Z0013_RO_C07_1_F10_A	111	17	LMG 1408	*Gluconobacter*	*oxydans*
Z0013_RB_C01_2_C12_A	111	17	LMG 1408	*Gluconobacter*	*oxydans*
Z0017_LB_C07_2_C03_A	112	57	LMG 23383	*Lactococcus*	*piscium*
Z0017_LB_D07_1_C03_A	112	57	LMG 23383	*Lactococcus*	*piscium*
Z0017_LB_C08_2_C03_B	112	57	LMG 23383	*Lactococcus*	*piscium*
Z0017_RB_C02_2_C12_B	112	57	LMG 23383	*Lactococcus*	*piscium*
Z0017_LB_D04_1_C05_B	112	57	LMG 23383	*Lactococcus*	*piscium*
Z0017_RB_C03_2_C11_A	112	57	LMG 23383	*Lactococcus*	*piscium*
Z0017_LB_D05_1_C04_A	112	57	LMG 23383	*Lactococcus*	*piscium*
Z0017_RB_C05_2_C10_A	112	57	LMG 23383	*Lactococcus*	*piscium*
Z0017_LB_D06_1_C04_B	112	57	LMG 23383	*Lactococcus*	*piscium*
Z0017_RB_C04_2_C11_B	112	57	LMG 23383	*Lactococcus*	*piscium*
Z0017_RB_B03_1_D11_A	112	57	LMG 23383	*Lactococcus*	*piscium*
Z0017_LB_C09_2_C02_A	112	57	LMG 23383	*Lactococcus*	*piscium*
Z0017_RB_B12_1_D07_B	112	57	LMG 23383	*Lactococcus*	*piscium*
Z0017_LB_D03_1_C05_A	112	57	LMG 23383	*Lactococcus*	*piscium*
Z0017_RB_C01_2_C12_A	112	57	LMG 23383	*Lactococcus*	*piscium*
Z0017_LB_D02_1_C06_B	112	57	LMG 23383	*Lactococcus*	*piscium*
Z0017_RB_C08_2_C09_B	112	57	LMG 23383	*Lactococcus*	*piscium*
Z0017_RB_B09_1_D08_A	112	57	LMG 23383	*Lactococcus*	*piscium*
Z0017_RB_B08_1_D09_B	112	57	LMG 23383	*Lactococcus*	*piscium*
Z0017_RB_C09_2_C08_A	112	57	LMG 23383	*Lactococcus*	*piscium*
Z0017_RB_C06_2_C10_B	112	57	LMG 23383	*Lactococcus*	*piscium*
Z0017_LB_C12_2_C01_B	112	57	LMG 23383	*Lactococcus*	*piscium*
Z0017_RB_C07_2_C09_A	112	57	LMG 23383	*Lactococcus*	*piscium*
Z0017_RB_B10_1_D08_B	112	57	LMG 23383	*Lactococcus*	*piscium*
Z0017_LB_D01_1_C06_A	112	57	LMG 23383	*Lactococcus*	*piscium*
Z0017_RB_B04_1_D11_B	112	57	LMG 23383	*Lactococcus*	*piscium*
Z0017_RB_B05_1_D10_A	112	57	LMG 23383	*Lactococcus*	*piscium*

Z0017_RB_B06_1_D10_B	112	57	LMG 23383	*Lactococcus*	*piscium*
Z0017_RB_B07_1_D09_A	112	57	LMG 23383	*Lactococcus*	*piscium*
Z0017_LB_C11_2_C01_A	112	57	LMG 23383	*Lactococcus*	*piscium*
Z0017_LB_C10_2_C02_B	112	57	LMG 23383	*Lactococcus*	*piscium*
Z0017_RB_B11_1_D07_A	113	57	LMG 23383	*Lactococcus*	*piscium*
Z0018_RO_B01_2_E07_A	114	76	LMG 24833	*Vagococcus*	*penaei*
Z0018_RO_B03_2_E08_A	114	76	LMG 24833	*Vagococcus*	*penaei*
Z0018_RO_B02_2_E07_B	114	76	LMG 24833	*Vagococcus*	*penaei*
Z0018_LO_B11_2_E06_A	114	76	LMG 24833	*Vagococcus*	*penaei*
Z0018_RO_A12_1_E12_B	114	76	LMG 24833	*Vagococcus*	*penaei*
Z0018_LO_B10_2_E05_B	114	76	LMG 24833	*Vagococcus*	*penaei*
Z0018_RO_C01_1_F07_A	114	76	LMG 24833	*Vagococcus*	*penaei*
Z0018_LO_E10_1_G05_B	114	76	LMG 24833	*Vagococcus*	*penaei*
Z0018_RO_C06_1_F09_B	114	76	LMG 24833	*Vagococcus*	*penaei*
Z0018_LO_F01_2_G01_A	114	76	LMG 24833	*Vagococcus*	*penaei*
Z0018_LO_F08_2_G04_B	114	76	LMG 24833	*Vagococcus*	*penaei*
Z0018_LO_F02_2_G01_B	114	76	LMG 24833	*Vagococcus*	*penaei*
Z0018_RO_C07_1_F10_A	114	76	LMG 24833	*Vagococcus*	*penaei*
Z0018_RO_C11_1_F12_A	114	76	LMG 24833	*Vagococcus*	*penaei*
Z0018_RO_C03_1_F08_A	114	76	LMG 24833	*Vagococcus*	*penaei*
Z0018_LO_D03_2_F02_A	114	76	LMG 24833	*Vagococcus*	*penaei*
Z0018_RO_B10_2_E11_B	114	76	LMG 24833	*Vagococcus*	*penaei*
Z0018_LO_E07_1_G04_A	114	76	LMG 24833	*Vagococcus*	*penaei*
Z0018_RO_A11_1_E12_A	114	76	LMG 24833	*Vagococcus*	*penaei*
Z0018_LO_C04_1_F02_B	114	76	LMG 24833	*Vagococcus*	*penaei*
Z0018_LO_E11_1_G06_A	114	76	LMG 24833	*Vagococcus*	*penaei*
Z0018_LO_E02_1_G01_B	114	76	LMG 24833	*Vagococcus*	*penaei*
Z0018_RO_C02_1_F07_B	114	76	LMG 24833	*Vagococcus*	*penaei*
Z0018_LO_E05_1_G03_A	114	76	LMG 24833	*Vagococcus*	*penaei*
Z0018_LO_B12_2_E06_B	114	76	LMG 24833	*Vagococcus*	*penaei*
Z0018_LO_A11_1_E06_A	114	76	LMG 24833	*Vagococcus*	*penaei*
Z0018_RB_H07_1_A09_A	114	76	LMG 24833	*Vagococcus*	*penaei*
Z0018_RB_H08_1_A09_B	114	76	LMG 24833	*Vagococcus*	*penaei*
Z0018_LO_D06_2_F03_B	114	76	LMG 24833	*Vagococcus*	*penaei*
Z0018_LO_E03_1_G02_A	114	76	LMG 24833	*Vagococcus*	*penaei*
Z0018_LO_C12_1_F06_B	114	76	LMG 24833	*Vagococcus*	*penaei*
Z0018_LO_C09_1_F05_A	114	76	LMG 24833	*Vagococcus*	*penaei*
Z0015_RB_B09_1_D08_A	115	35	LMG 21292	*etogulonicigeniun*	*robustum*
Z0015_LB_D03_1_C05_A	115	35	LMG 21292	*etogulonicigeniun*	*robustum*
Z0015_LB_E03_2_B05_A	115	35	LMG 21292	*etogulonicigeniun*	*robustum*
Z0015_RB_C06_2_C10_B	115	35	LMG 21292	*etogulonicigeniun*	*robustum*
Z0015_RB_B11_1_D07_A	115	35	LMG 21292	*etogulonicigeniun*	*robustum*
Z0015_LB_D07_1_C03_A	115	35	LMG 21292	*etogulonicigeniun*	*robustum*
Z0015_LB_D06_1_C04_B	115	35	LMG 21292	*etogulonicigeniun*	*robustum*
Z0015_RB_B10_1_D08_B	115	35	LMG 21292	*etogulonicigeniun*	*robustum*
Z0015_LB_D04_1_C05_B	115	35	LMG 21292	*etogulonicigeniun*	*robustum*
Z0015_LB_E02_2_B06_B	115	35	LMG 21292	*etogulonicigeniun*	*robustum*
Z0015_LB_D05_1_C04_A	115	35	LMG 21292	*etogulonicigeniun*	*robustum*
Z0015_RB_C03_2_C11_A	115	35	LMG 21292	*etogulonicigeniun*	*robustum*
Z0015_LB_E01_2_B06_A	115	35	LMG 21292	*etogulonicigeniun*	*robustum*

Z0015_RB_C07_2_C09_A	115	35	LMG 21292	*:etogulonicigeniur*	*robustum*
Z0015_RB_B07_1_D09_A	116	35	LMG 21292	*:etogulonicigeniur*	*robustum*
Z0015_RB_B05_1_D10_A	116	35	LMG 21292	*:etogulonicigeniur*	*robustum*
Z0015_LB_C11_2_C01_A	116	35	LMG 21292	*:etogulonicigeniur*	*robustum*
Z0015_LB_D08_1_C03_B	116	35	LMG 21292	*:etogulonicigeniur*	*robustum*
Z0015_RB_C01_2_C12_A	116	35	LMG 21292	*:etogulonicigeniur*	*robustum*
Z0015_LB_D10_1_C02_B	116	35	LMG 21292	*:etogulonicigeniur*	*robustum*
Z0015_LB_D12_1_C01_B	116	35	LMG 21292	*:etogulonicigeniur*	*robustum*
Z0015_LB_D11_1_C01_A	116	35	LMG 21292	*:etogulonicigeniur*	*robustum*
Z0015_RB_C02_2_C12_B	116	35	LMG 21292	*:etogulonicigeniur*	*robustum*
Z0015_LB_D09_1_C02_A	116	35	LMG 21292	*:etogulonicigeniur*	*robustum*
Z0015_RB_B06_1_D10_B	116	35	LMG 21292	*:etogulonicigeniur*	*robustum*
Z0015_RB_C05_2_C10_A	117	35	LMG 21292	*:etogulonicigeniur*	*robustum*
Z0015_RB_C04_2_C11_B	117	35	LMG 21292	*:etogulonicigeniur*	*robustum*
Z0015_RB_B12_1_D07_B	117	35	LMG 21292	*:etogulonicigeniur*	*robustum*
Z0015_LB_D01_1_C06_A	117	35	LMG 21292	*:etogulonicigeniur*	*robustum*
Z0015_LB_D02_1_C06_B	117	35	LMG 21292	*:etogulonicigeniur*	*robustum*
Z0015_RB_B08_1_D09_B	117	35	LMG 21292	*:etogulonicigeniur*	*robustum*
Z0015_LB_C12_2_C01_B	117	35	LMG 21292	*:etogulonicigeniur*	*robustum*
Z0018_LO_H12_2_H06_B	118	67	LMG 24367	*Ruegeria*	*scottomollicae*
Z0018_LO_H10_2_H05_B	118	67	LMG 24367	*Ruegeria*	*scottomollicae*
Z0018_RB_A04_2_D11_B	118	67	LMG 24367	*Ruegeria*	*scottomollicae*
Z0018_LB_A10_2_D02_B	118	67	LMG 24367	*Ruegeria*	*scottomollicae*
Z0018_RB_A03_2_D11_A	118	67	LMG 24367	*Ruegeria*	*scottomollicae*
Z0018_LO_H11_2_H06_A	118	67	LMG 24367	*Ruegeria*	*scottomollicae*
Z0018_LO_H09_2_H05_A	118	67	LMG 24367	*Ruegeria*	*scottomollicae*
Z0018_LB_A03_2_D05_A	118	67	LMG 24367	*Ruegeria*	*scottomollicae*
Z0018_RB_A02_2_D12_B	118	67	LMG 24367	*Ruegeria*	*scottomollicae*
Z0018_RB_A06_2_D10_B	118	67	LMG 24367	*Ruegeria*	*scottomollicae*
Z0018_LB_A11_2_D01_A	118	67	LMG 24367	*Ruegeria*	*scottomollicae*
Z0018_RB_A05_2_D10_A	118	67	LMG 24367	*Ruegeria*	*scottomollicae*
Z0018_LB_A12_2_D01_B	118	67	LMG 24367	*Ruegeria*	*scottomollicae*
Z0018_RB_A01_2_D12_A	118	67	LMG 24367	*Ruegeria*	*scottomollicae*
Z0018_LB_A09_2_D02_A	118	67	LMG 24367	*Ruegeria*	*scottomollicae*
Z0018_LB_A08_2_D03_B	118	67	LMG 24367	*Ruegeria*	*scottomollicae*
Z0018_LO_C02_1_F01_B	119	67	LMG 24367	*Ruegeria*	*scottomollicae*
Z0018_LO_B02_2_E01_B	119	67	LMG 24367	*Ruegeria*	*scottomollicae*
Z0018_LO_B01_2_E01_A	119	67	LMG 24367	*Ruegeria*	*scottomollicae*
Z0018_RB_H10_1_A08_B	119	67	LMG 24367	*Ruegeria*	*scottomollicae*
Z0018_LB_A07_2_D03_A	119	67	LMG 24367	*Ruegeria*	*scottomollicae*
Z0018_LB_A06_2_D04_B	119	67	LMG 24367	*Ruegeria*	*scottomollicae*
Z0018_LB_A04_2_D05_B	119	67	LMG 24367	*Ruegeria*	*scottomollicae*
Z0018_LO_H07_2_H04_A	119	67	LMG 24367	*Ruegeria*	*scottomollicae*
Z0018_LB_A02_2_D06_B	119	67	LMG 24367	*Ruegeria*	*scottomollicae*
Z0018_LO_H08_2_H04_B	119	67	LMG 24367	*Ruegeria*	*scottomollicae*
Z0018_RO_A01_1_E07_A	119	67	LMG 24367	*Ruegeria*	*scottomollicae*
Z0018_LB_A01_2_D06_A	119	67	LMG 24367	*Ruegeria*	*scottomollicae*
Z0018_LB_A05_2_D04_A	119	67	LMG 24367	*Ruegeria*	*scottomollicae*
Z0018_RO_B08_2_E10_B	119	67	LMG 24367	*Ruegeria*	*scottomollicae*
Z0018_LO_A12_1_E06_B	119	67	LMG 24367	*Ruegeria*	*scottomollicae*

Z0018_RO_A03_1_E08_A	119	67	LMG 24367	*Ruegeria*	*scottomollicae*
Z0020_LO_G11_1_H06_A	120	89	LMG 26195	*Paracoccus*	*sp.*
Z0020_LO_G10_1_H05_B	120	89	LMG 26195	*Paracoccus*	*sp.*
Z0020_LB_A02_2_D06_B	120	89	LMG 26195	*Paracoccus*	*sp.*
Z0020_LO_H01_2_H01_A	120	89	LMG 26195	*Paracoccus*	*sp.*
Z0020_LO_H02_2_H01_B	120	89	LMG 26195	*Paracoccus*	*sp.*
Z0020_LO_G12_1_H06_B	120	89	LMG 26195	*Paracoccus*	*sp.*
Z0020_LB_A07_2_D03_A	120	89	LMG 26195	*Paracoccus*	*sp.*
Z0020_RB_H04_1_A11_B	120	89	LMG 26195	*Paracoccus*	*sp.*
Z0020_LB_A03_2_D05_A	120	89	LMG 26195	*Paracoccus*	*sp.*
Z0020_LB_A04_2_D05_B	120	89	LMG 26195	*Paracoccus*	*sp.*
Z0020_LO_H06_2_H03_B	120	89	LMG 26195	*Paracoccus*	*sp.*
Z0020_LO_H05_2_H03_A	120	89	LMG 26195	*Paracoccus*	*sp.*
Z0020_LB_A10_2_D02_B	120	89	LMG 26195	*Paracoccus*	*sp.*
Z0020_LB_A11_2_D01_A	120	89	LMG 26195	*Paracoccus*	*sp.*
Z0020_LO_H08_2_H04_B	120	89	LMG 26195	*Paracoccus*	*sp.*
Z0020_LO_H09_2_H05_A	120	89	LMG 26195	*Paracoccus*	*sp.*
Z0020_LO_H07_2_H04_A	120	89	LMG 26195	*Paracoccus*	*sp.*
Z0020_LB_A12_2_D01_B	120	89	LMG 26195	*Paracoccus*	*sp.*
Z0020_RO_A01_1_E07_A	120	89	LMG 26195	*Paracoccus*	*sp.*
Z0020_RO_A04_1_E08_B	120	89	LMG 26195	*Paracoccus*	*sp.*
Z0020_LB_A05_2_D04_A	120	89	LMG 26195	*Paracoccus*	*sp.*
Z0020_LB_A06_2_D04_B	120	89	LMG 26195	*Paracoccus*	*sp.*
Z0020_RB_H08_1_A09_B	120	89	LMG 26195	*Paracoccus*	*sp.*
Z0020_LO_H04_2_H02_B	120	89	LMG 26195	*Paracoccus*	*sp.*
Z0020_LO_H03_2_H02_A	120	89	LMG 26195	*Paracoccus*	*sp.*
Z0020_LB_A08_2_D03_B	120	89	LMG 26195	*Paracoccus*	*sp.*
Z0020_LB_A09_2_D02_A	120	89	LMG 26195	*Paracoccus*	*sp.*
Z0020_LB_A01_2_D06_A	120	89	LMG 26195	*Paracoccus*	*sp.*
Z0020_RO_B05_2_E09_A	121	89	LMG 26195	*Paracoccus*	*sp.*
Z0020_RO_B07_2_E10_A	121	89	LMG 26195	*Paracoccus*	*sp.*
Z0020_LO_B09_2_E05_A	121	89	LMG 26195	*Paracoccus*	*sp.*
Z0020_RO_A06_1_E09_B	121	89	LMG 26195	*Paracoccus*	*sp.*
Z0016_LB_C11_2_C01_A	122	45	LMG 22475	*Phaeobacter*	*inhibens*
Z0016_LB_D01_1_C06_A	122	45	LMG 22475	*Phaeobacter*	*inhibens*
Z0016_RB_C10_2_C08_B	122	45	LMG 22475	*Phaeobacter*	*inhibens*
Z0016_RB_B09_1_D08_A	122	45	LMG 22475	*Phaeobacter*	*inhibens*
Z0016_LB_C06_2_C04_B	122	45	LMG 22475	*Phaeobacter*	*inhibens*
Z0016_RB_C09_2_C08_A	122	45	LMG 22475	*Phaeobacter*	*inhibens*
Z0016_RB_C12_2_C07_B	122	45	LMG 22475	*Phaeobacter*	*inhibens*
Z0016_RB_C02_2_C12_B	122	45	LMG 22475	*Phaeobacter*	*inhibens*
Z0016_LB_C07_2_C03_A	122	45	LMG 22475	*Phaeobacter*	*inhibens*
Z0016_RB_B11_1_D07_A	122	45	LMG 22475	*Phaeobacter*	*inhibens*
Z0016_RB_C01_2_C12_A	122	45	LMG 22475	*Phaeobacter*	*inhibens*
Z0016_RB_B12_1_D07_B	122	45	LMG 22475	*Phaeobacter*	*inhibens*
Z0016_LB_C12_2_C01_B	122	45	LMG 22475	*Phaeobacter*	*inhibens*
Z0016_LB_D02_1_C06_B	122	45	LMG 22475	*Phaeobacter*	*inhibens*
Z0016_RB_C08_2_C09_B	122	45	LMG 22475	*Phaeobacter*	*inhibens*
Z0016_LB_D03_1_C05_A	122	45	LMG 22475	*Phaeobacter*	*inhibens*
Z0016_RB_B10_1_D08_B	122	45	LMG 22475	*Phaeobacter*	*inhibens*

Z0016_RB_D01_1_C12_A	122	45	LMG 22475	*Phaeobacter*	*inhibens*
Z0016_LB_D04_1_C05_B	122	45	LMG 22475	*Phaeobacter*	*inhibens*
Z0016_RB_C11_2_C07_A	122	45	LMG 22475	*Phaeobacter*	*inhibens*
Z0016_RB_C07_2_C09_A	122	45	LMG 22475	*Phaeobacter*	*inhibens*
Z0016_LB_C10_2_C02_B	122	45	LMG 22475	*Phaeobacter*	*inhibens*
Z0016_RB_C06_2_C10_B	122	45	LMG 22475	*Phaeobacter*	*inhibens*
Z0016_LB_D05_1_C04_A	122	45	LMG 22475	*Phaeobacter*	*inhibens*
Z0016_RB_D03_1_C11_A	122	45	LMG 22475	*Phaeobacter*	*inhibens*
Z0016_RB_C05_2_C10_A	122	45	LMG 22475	*Phaeobacter*	*inhibens*
Z0016_LB_C08_2_C03_B	122	45	LMG 22475	*Phaeobacter*	*inhibens*
Z0016_LB_D06_1_C04_B	122	45	LMG 22475	*Phaeobacter*	*inhibens*
Z0016_RB_C03_2_C11_A	122	45	LMG 22475	*Phaeobacter*	*inhibens*
Z0016_RB_D02_1_C12_B	122	45	LMG 22475	*Phaeobacter*	*inhibens*
Z0016_LB_C09_2_C02_A	122	45	LMG 22475	*Phaeobacter*	*inhibens*
Z0016_RB_C04_2_C11_B	123	45	LMG 22475	*Phaeobacter*	*inhibens*
Z0019_RO_A07_1_E10_A	124	83	LMG 25773	*Tabrizicola*	*aquatica*
Z0019_RO_A09_1_E11_A	124	83	LMG 25773	*Tabrizicola*	*aquatica*
Z0019_RO_A06_1_E09_B	124	83	LMG 25773	*Tabrizicola*	*aquatica*
Z0019_RO_A08_1_E10_B	124	83	LMG 25773	*Tabrizicola*	*aquatica*
Z0019_RO_A04_1_E08_B	124	83	LMG 25773	*Tabrizicola*	*aquatica*
Z0019_RO_A05_1_E09_A	124	83	LMG 25773	*Tabrizicola*	*aquatica*
Z0019_LO_A03_1_E02_A	124	83	LMG 25773	*Tabrizicola*	*aquatica*
Z0019_LO_A04_1_E02_B	124	83	LMG 25773	*Tabrizicola*	*aquatica*
Z0019_LB_H09_1_A02_A	124	83	LMG 25773	*Tabrizicola*	*aquatica*
Z0019_LB_H08_1_A03_B	124	83	LMG 25773	*Tabrizicola*	*aquatica*
Z0019_LB_H10_1_A02_B	124	83	LMG 25773	*Tabrizicola*	*aquatica*
Z0019_LB_H11_1_A01_A	124	83	LMG 25773	*Tabrizicola*	*aquatica*
Z0019_LO_A06_1_E03_B	125	83	LMG 25773	*Tabrizicola*	*aquatica*
Z0019_LO_A05_1_E03_A	125	83	LMG 25773	*Tabrizicola*	*aquatica*
Z0019_RO_A11_1_E12_A	125	83	LMG 25773	*Tabrizicola*	*aquatica*
Z0019_LO_A08_1_E04_B	125	83	LMG 25773	*Tabrizicola*	*aquatica*
Z0019_RB_H04_1_A11_B	126	83	LMG 25773	*Tabrizicola*	*aquatica*
Z0019_LB_H03_1_A05_A	126	83	LMG 25773	*Tabrizicola*	*aquatica*
Z0019_LB_H04_1_A05_B	126	83	LMG 25773	*Tabrizicola*	*aquatica*
Z0019_RB_H05_1_A10_A	126	83	LMG 25773	*Tabrizicola*	*aquatica*
Z0019_LO_A01_1_E01_A	126	83	LMG 25773	*Tabrizicola*	*aquatica*
Z0019_LO_A02_1_E01_B	126	83	LMG 25773	*Tabrizicola*	*aquatica*
Z0019_LB_H12_1_A01_B	126	83	LMG 25773	*Tabrizicola*	*aquatica*
Z0019_RO_A03_1_E08_A	126	83	LMG 25773	*Tabrizicola*	*aquatica*
Z0019_LB_H06_1_A04_B	127	83	LMG 25773	*Tabrizicola*	*aquatica*
Z0019_LB_H05_1_A04_A	127	83	LMG 25773	*Tabrizicola*	*aquatica*
Z0019_LB_H07_1_A03_A	127	83	LMG 25773	*Tabrizicola*	*aquatica*
Z0019_RB_H09_1_A08_A	127	83	LMG 25773	*Tabrizicola*	*aquatica*
Z0015_RO_G09_1_H11_A	128	42	LMG 22049	*Tatumella*	*citrea*
Z0015_LO_F03_2_G02_A	128	42	LMG 22049	*Tatumella*	*citrea*
Z0015_LO_F08_2_G04_B	128	42	LMG 22049	*Tatumella*	*citrea*
Z0015_LO_F10_2_G05_B	128	42	LMG 22049	*Tatumella*	*citrea*
Z0015_LO_G06_1_H03_B	128	42	LMG 22049	*Tatumella*	*citrea*
Z0015_LO_F09_2_G05_A	128	42	LMG 22049	*Tatumella*	*citrea*
Z0015_RO_H03_2_H08_A	128	42	LMG 22049	*Tatumella*	*citrea*

Z0015_LO_E11_1_G06_A	128	42	LMG 22049	*Tatumella*	*citrea*
Z0015_LO_F05_2_G03_A	128	42	LMG 22049	*Tatumella*	*citrea*
Z0015_LO_E12_1_G06_B	128	42	LMG 22049	*Tatumella*	*citrea*
Z0015_LO_E10_1_G05_B	128	42	LMG 22049	*Tatumella*	*citrea*
Z0015_LO_F01_2_G01_A	128	42	LMG 22049	*Tatumella*	*citrea*
Z0015_RO_G06_1_H09_B	128	42	LMG 22049	*Tatumella*	*citrea*
Z0015_LO_F11_2_G06_A	128	42	LMG 22049	*Tatumella*	*citrea*
Z0015_RO_H02_2_H07_B	128	42	LMG 22049	*Tatumella*	*citrea*
Z0015_LO_F06_2_G03_B	128	42	LMG 22049	*Tatumella*	*citrea*
Z0015_LO_G05_1_H03_A	128	42	LMG 22049	*Tatumella*	*citrea*
Z0015_LO_G09_1_H05_A	128	42	LMG 22049	*Tatumella*	*citrea*
Z0015_LO_G10_1_H05_B	128	42	LMG 22049	*Tatumella*	*citrea*
Z0015_LO_G08_1_H04_B	128	42	LMG 22049	*Tatumella*	*citrea*
Z0015_LO_G07_1_H04_A	128	42	LMG 22049	*Tatumella*	*citrea*
Z0015_RO_G10_1_H11_B	129	42	LMG 22049	*Tatumella*	*citrea*
Z0015_RO_F08_2_G10_B	130	42	LMG 22049	*Tatumella*	*citrea*
Z0015_LO_E02_1_G01_B	130	42	LMG 22049	*Tatumella*	*citrea*
Z0015_LO_E01_1_G01_A	130	42	LMG 22049	*Tatumella*	*citrea*
Z0015_RO_G01_1_H07_A	130	42	LMG 22049	*Tatumella*	*citrea*
Z0015_RO_F10_2_G11_B	130	42	LMG 22049	*Tatumella*	*citrea*
Z0015_RO_G07_1_H10_A	130	42	LMG 22049	*Tatumella*	*citrea*
Z0015_RO_H04_2_H08_B	130	42	LMG 22049	*Tatumella*	*citrea*
Z0015_RO_G08_1_H10_B	130	42	LMG 22049	*Tatumella*	*citrea*
Z0015_LO_E08_1_G04_B	130	42	LMG 22049	*Tatumella*	*citrea*
Z0015_LO_E05_1_G03_A	130	42	LMG 22049	*Tatumella*	*citrea*
Z0015_LO_E07_1_G04_A	131	41	LMG 2186	*Phaseolibacter*	*flectens*
Z0015_LO_G04_1_H02_B	131	41	LMG 2186	*Phaseolibacter*	*flectens*
Z0015_RO_E08_1_G10_B	131	41	LMG 2186	*Phaseolibacter*	*flectens*
Z0015_LO_E03_1_G02_A	131	41	LMG 2186	*Phaseolibacter*	*flectens*
Z0015_RO_F12_2_G12_B	131	41	LMG 2186	*Phaseolibacter*	*flectens*
Z0015_LO_F04_2_G02_B	131	41	LMG 2186	*Phaseolibacter*	*flectens*
Z0015_RO_F06_2_G09_B	131	41	LMG 2186	*Phaseolibacter*	*flectens*
Z0015_RO_F07_2_G10_A	131	41	LMG 2186	*Phaseolibacter*	*flectens*
Z0015_RO_F02_2_G07_B	131	41	LMG 2186	*Phaseolibacter*	*flectens*
Z0015_LO_F02_2_G01_B	131	41	LMG 2186	*Phaseolibacter*	*flectens*
Z0015_RO_F05_2_G09_A	131	41	LMG 2186	*Phaseolibacter*	*flectens*
Z0015_LO_E09_1_G05_A	131	41	LMG 2186	*Phaseolibacter*	*flectens*
Z0015_RO_G03_1_H08_A	131	41	LMG 2186	*Phaseolibacter*	*flectens*
Z0015_LO_G01_1_H01_A	131	41	LMG 2186	*Phaseolibacter*	*flectens*
Z0015_LO_F12_2_G06_B	131	41	LMG 2186	*Phaseolibacter*	*flectens*
Z0015_LO_G03_1_H02_A	131	41	LMG 2186	*Phaseolibacter*	*flectens*
Z0015_LO_G02_1_H01_B	131	41	LMG 2186	*Phaseolibacter*	*flectens*
Z0015_RO_G05_1_H09_A	131	41	LMG 2186	*Phaseolibacter*	*flectens*
Z0015_RO_G02_1_H07_B	131	41	LMG 2186	*Phaseolibacter*	*flectens*
Z0015_RO_G04_1_H08_B	131	41	LMG 2186	*Phaseolibacter*	*flectens*
Z0015_RO_F09_2_G11_A	131	41	LMG 2186	*Phaseolibacter*	*flectens*
Z0015_RO_F04_2_G08_B	131	41	LMG 2186	*Phaseolibacter*	*flectens*
Z0015_RO_H01_2_H07_A	131	41	LMG 2186	*Phaseolibacter*	*flectens*
Z0015_RO_E11_1_G12_A	132	41	LMG 2186	*Phaseolibacter*	*flectens*
Z0015_LO_E06_1_G03_B	132	41	LMG 2186	*Phaseolibacter*	*flectens*

Z0015_RO_G12_1_H12_B	132	41	LMG 2186	*Phaseolibacter*	*flectens*
Z0015_RO_F11_2_G12_A	132	41	LMG 2186	*Phaseolibacter*	*flectens*
Z0015_LO_F07_2_G04_A	132	41	LMG 2186	*Phaseolibacter*	*flectens*
Z0015_RO_G11_1_H12_A	132	41	LMG 2186	*Phaseolibacter*	*flectens*
Z0015_LO_E04_1_G02_B	132	41	LMG 2186	*Phaseolibacter*	*flectens*
Z0015_RO_E07_1_G10_A	133	41	LMG 2186	*Phaseolibacter*	*flectens*
Z0015_LO_D12_2_F06_B	133	41	LMG 2186	*Phaseolibacter*	*flectens*
Z0012_RO_E02_1_G07_B	134	11	LMG 1286	*Pantoea*	*agglomerans*
Z0012_RO_E01_1_G07_A	134	11	LMG 1286	*Pantoea*	*agglomerans*
Z0012_RO_E11_1_G12_A	134	11	LMG 1286	*Pantoea*	*agglomerans*
Z0012_RO_E12_1_G12_B	134	11	LMG 1286	*Pantoea*	*agglomerans*
Z0012_RO_F02_2_G07_B	134	11	LMG 1286	*Pantoea*	*agglomerans*
Z0012_LO_E10_1_G05_B	134	11	LMG 1286	*Pantoea*	*agglomerans*
Z0012_LO_E08_1_G04_B	134	11	LMG 1286	*Pantoea*	*agglomerans*
Z0012_LO_E06_1_G03_B	134	11	LMG 1286	*Pantoea*	*agglomerans*
Z0012_RO_D04_2_F08_B	134	11	LMG 1286	*Pantoea*	*agglomerans*
Z0012_LO_G03_1_H02_A	134	11	LMG 1286	*Pantoea*	*agglomerans*
Z0012_LO_G01_1_H01_A	134	11	LMG 1286	*Pantoea*	*agglomerans*
Z0012_RO_E06_1_G09_B	134	11	LMG 1286	*Pantoea*	*agglomerans*
Z0012_LO_E09_1_G05_A	134	11	LMG 1286	*Pantoea*	*agglomerans*
Z0012_RO_D06_2_F09_B	134	11	LMG 1286	*Pantoea*	*agglomerans*
Z0012_RO_D08_2_F10_B	134	11	LMG 1286	*Pantoea*	*agglomerans*
Z0012_RO_E04_1_G08_B	134	11	LMG 1286	*Pantoea*	*agglomerans*
Z0012_LO_D07_2_F04_A	134	11	LMG 1286	*Pantoea*	*agglomerans*
Z0012_LO_D03_2_F02_A	134	11	LMG 1286	*Pantoea*	*agglomerans*
Z0012_LO_D04_2_F02_B	134	11	LMG 1286	*Pantoea*	*agglomerans*
Z0012_LO_D09_2_F05_A	134	11	LMG 1286	*Pantoea*	*agglomerans*
Z0012_LO_F12_2_G06_B	134	11	LMG 1286	*Pantoea*	*agglomerans*
Z0012_RO_D01_2_F07_A	134	11	LMG 1286	*Pantoea*	*agglomerans*
Z0012_LO_E02_1_G01_B	134	11	LMG 1286	*Pantoea*	*agglomerans*
Z0012_RO_C11_1_F12_A	134	11	LMG 1286	*Pantoea*	*agglomerans*
Z0012_LO_E01_1_G01_A	134	11	LMG 1286	*Pantoea*	*agglomerans*
Z0012_RO_E05_1_G09_A	134	11	LMG 1286	*Pantoea*	*agglomerans*
Z0012_LO_F05_2_G03_A	134	11	LMG 1286	*Pantoea*	*agglomerans*
Z0012_RO_E03_1_G08_A	134	11	LMG 1286	*Pantoea*	*agglomerans*
Z0012_LO_F04_2_G02_B	135	11	LMG 1286	*Pantoea*	*agglomerans*
Z0012_LO_F06_2_G03_B	135	11	LMG 1286	*Pantoea*	*agglomerans*
Z0012_LO_F02_2_G01_B	135	11	LMG 1286	*Pantoea*	*agglomerans*
Z0012_LO_F07_2_G04_A	135	11	LMG 1286	*Pantoea*	*agglomerans*
Z0025_LB_E08_2_B03_B	136	135	LMG 7881	*Serratia*	*ficaria*
Z0025_LB_D04_1_C05_B	136	135	LMG 7881	*Serratia*	*ficaria*
Z0025_LB_E06_2_B04_B	136	135	LMG 7881	*Serratia*	*ficaria*
Z0025_LB_D03_1_C05_A	136	135	LMG 7881	*Serratia*	*ficaria*
Z0025_RB_F09_1_B08_A	136	135	LMG 7881	*Serratia*	*ficaria*
Z0025_LB_E04_2_B05_B	136	135	LMG 7881	*Serratia*	*ficaria*
Z0025_LB_D06_1_C04_B	136	135	LMG 7881	*Serratia*	*ficaria*
Z0025_LB_D05_1_C04_A	136	135	LMG 7881	*Serratia*	*ficaria*
Z0025_LB_E03_2_B05_A	136	135	LMG 7881	*Serratia*	*ficaria*
Z0025_RB_F06_1_B10_B	136	135	LMG 7881	*Serratia*	*ficaria*
Z0025_RB_F08_1_B09_B	136	135	LMG 7881	*Serratia*	*ficaria*

Z0025_LB_E09_2_B02_A	136	135	LMG 7881	*Serratia*	*ficaria*	
Z0025_LB_E02_2_B06_B	136	135	LMG 7881	*Serratia*	*ficaria*	
Z0025_RB_F05_1_B10_A	137	135	LMG 7881	*Serratia*	*ficaria*	
Z0025_LB_D09_1_C02_A	137	135	LMG 7881	*Serratia*	*ficaria*	
Z0025_LB_D08_1_C03_B	137	135	LMG 7881	*Serratia*	*ficaria*	
Z0025_LB_D07_1_C03_A	137	135	LMG 7881	*Serratia*	*ficaria*	
Z0025_RB_F07_1_B09_A	137	135	LMG 7881	*Serratia*	*ficaria*	
Z0025_LB_E05_2_B04_A	137	135	LMG 7881	*Serratia*	*ficaria*	
Z0025_LB_E07_2_B03_A	137	135	LMG 7881	*Serratia*	*ficaria*	
Z0025_RB_F04_1_B11_B	138	135	LMG 7881	*Serratia*	*ficaria*	
Z0025_LB_D01_1_C06_A	138	135	LMG 7881	*Serratia*	*ficaria*	
Z0025_LB_D02_1_C06_B	138	135	LMG 7881	*Serratia*	*ficaria*	
Z0025_LB_C12_2_C01_B	138	135	LMG 7881	*Serratia*	*ficaria*	
Z0025_LB_C05_2_C04_A	138	135	LMG 7881	*Serratia*	*ficaria*	
Z0025_RB_F03_1_B11_A	138	135	LMG 7881	*Serratia*	*ficaria*	
Z0025_RB_F02_1_B12_B	139	135	LMG 7881	*Serratia*	*ficaria*	
Z0025_RB_F01_1_B12_A	139	135	LMG 7881	*Serratia*	*ficaria*	
Z0025_LB_D11_1_C01_A	139	135	LMG 7881	*Serratia*	*ficaria*	
Z0025_LB_E01_2_B06_A	139	135	LMG 7881	*Serratia*	*ficaria*	
Z0025_LB_D10_1_C02_B	140	135	LMG 7881	*Serratia*	*ficaria*	
Z0025_LB_D12_1_C01_B	140	135	LMG 7881	*Serratia*	*ficaria*	
Z0021_RB_C03_2_C11_A	141	99	LMG 2724	*Lonsdalea*	*quercina*	*quercina*
Z0021_LB_C11_2_C01_A	141	99	LMG 2724	*Lonsdalea*	*quercina*	*quercina*
Z0021_RB_C04_2_C11_B	141	99	LMG 2724	*Lonsdalea*	*quercina*	*quercina*
Z0021_RB_C05_2_C10_A	141	99	LMG 2724	*Lonsdalea*	*quercina*	*quercina*
Z0021_RB_D02_1_C12_B	141	99	LMG 2724	*Lonsdalea*	*quercina*	*quercina*
Z0021_LB_D06_1_C04_B	141	99	LMG 2724	*Lonsdalea*	*quercina*	*quercina*
Z0021_RB_D01_1_C12_A	141	99	LMG 2724	*Lonsdalea*	*quercina*	*quercina*
Z0021_LB_D05_1_C04_A	141	99	LMG 2724	*Lonsdalea*	*quercina*	*quercina*
Z0021_RB_C02_2_C12_B	141	99	LMG 2724	*Lonsdalea*	*quercina*	*quercina*
Z0021_LB_C10_2_C02_B	141	99	LMG 2724	*Lonsdalea*	*quercina*	*quercina*
Z0021_RB_C01_2_C12_A	141	99	LMG 2724	*Lonsdalea*	*quercina*	*quercina*
Z0021_RB_B10_1_D08_B	142	99	LMG 2724	*Lonsdalea*	*quercina*	*quercina*
Z0021_RB_C11_2_C07_A	142	99	LMG 2724	*Lonsdalea*	*quercina*	*quercina*
Z0021_LB_C09_2_C02_A	142	99	LMG 2724	*Lonsdalea*	*quercina*	*quercina*
Z0021_RB_B12_1_D07_B	142	99	LMG 2724	*Lonsdalea*	*quercina*	*quercina*
Z0021_RB_B11_1_D07_A	142	99	LMG 2724	*Lonsdalea*	*quercina*	*quercina*
Z0021_LB_C08_2_C03_B	142	99	LMG 2724	*Lonsdalea*	*quercina*	*quercina*
Z0021_LB_D04_1_C05_B	142	99	LMG 2724	*Lonsdalea*	*quercina*	*quercina*
Z0021_RB_C12_2_C07_B	142	99	LMG 2724	*Lonsdalea*	*quercina*	*quercina*
Z0021_LB_D03_1_C05_A	142	99	LMG 2724	*Lonsdalea*	*quercina*	*quercina*
Z0021_RB_B08_1_D09_B	142	99	LMG 2724	*Lonsdalea*	*quercina*	*quercina*
Z0021_RB_C06_2_C10_B	142	99	LMG 2724	*Lonsdalea*	*quercina*	*quercina*
Z0021_LB_C07_2_C03_A	142	99	LMG 2724	*Lonsdalea*	*quercina*	*quercina*
Z0021_LB_C12_2_C01_B	142	99	LMG 2724	*Lonsdalea*	*quercina*	*quercina*
Z0021_RB_B09_1_D08_A	142	99	LMG 2724	*Lonsdalea*	*quercina*	*quercina*
Z0021_LB_C06_2_C04_B	142	99	LMG 2724	*Lonsdalea*	*quercina*	*quercina*
Z0021_RB_C07_2_C09_A	142	99	LMG 2724	*Lonsdalea*	*quercina*	*quercina*
Z0021_RB_C08_2_C09_B	142	99	LMG 2724	*Lonsdalea*	*quercina*	*quercina*
Z0021_RB_C10_2_C08_B	142	99	LMG 2724	*Lonsdalea*	*quercina*	*quercina*

Z0021_RB_C09_2_C08_A	142	99	LMG 2724	*Lonsdalea*	*quercina*	*quercina*
Z0021_LB_D02_1_C06_B	142	99	LMG 2724	*Lonsdalea*	*quercina*	*quercina*
Z0021_LB_D01_1_C06_A	142	99	LMG 2724	*Lonsdalea*	*quercina*	*quercina*
Z0021_LB_G12_2_A01_B	143	102	LMG 2804	*Dickeya*	*chrysanthemi*	
Z0021_LB_H01_1_A06_A	143	102	LMG 2804	*Dickeya*	*chrysanthemi*	
Z0021_LB_G11_2_A01_A	143	102	LMG 2804	*Dickeya*	*chrysanthemi*	
Z0021_RB_G02_2_A12_B	143	102	LMG 2804	*Dickeya*	*chrysanthemi*	
Z0021_LB_G05_2_A04_A	143	102	LMG 2804	*Dickeya*	*chrysanthemi*	
Z0021_RB_G03_2_A11_A	143	102	LMG 2804	*Dickeya*	*chrysanthemi*	
Z0021_RB_G12_2_A07_B	143	102	LMG 2804	*Dickeya*	*chrysanthemi*	
Z0021_LB_H02_1_A06_B	143	102	LMG 2804	*Dickeya*	*chrysanthemi*	
Z0021_RB_H01_1_A12_A	143	102	LMG 2804	*Dickeya*	*chrysanthemi*	
Z0021_RB_H02_1_A12_B	143	102	LMG 2804	*Dickeya*	*chrysanthemi*	
Z0021_LB_G04_2_A05_B	143	102	LMG 2804	*Dickeya*	*chrysanthemi*	
Z0021_LB_G03_2_A05_A	143	102	LMG 2804	*Dickeya*	*chrysanthemi*	
Z0021_LB_F12_1_B01_B	143	102	LMG 2804	*Dickeya*	*chrysanthemi*	
Z0021_LB_G02_2_A06_B	143	102	LMG 2804	*Dickeya*	*chrysanthemi*	
Z0021_LB_G01_2_A06_A	143	102	LMG 2804	*Dickeya*	*chrysanthemi*	
Z0021_RB_G09_2_A08_A	143	102	LMG 2804	*Dickeya*	*chrysanthemi*	
Z0021_LB_G10_2_A02_B	143	102	LMG 2804	*Dickeya*	*chrysanthemi*	
Z0021_RB_G10_2_A08_B	143	102	LMG 2804	*Dickeya*	*chrysanthemi*	
Z0021_LB_G06_2_A04_B	143	102	LMG 2804	*Dickeya*	*chrysanthemi*	
Z0021_RB_G11_2_A07_A	143	102	LMG 2804	*Dickeya*	*chrysanthemi*	
Z0021_RB_H04_1_A11_B	144	102	LMG 2804	*Dickeya*	*chrysanthemi*	
Z0021_RB_H03_1_A11_A	144	102	LMG 2804	*Dickeya*	*chrysanthemi*	
Z0021_LB_H04_1_A05_B	144	102	LMG 2804	*Dickeya*	*chrysanthemi*	
Z0021_LB_H03_1_A05_A	144	102	LMG 2804	*Dickeya*	*chrysanthemi*	
Z0021_RB_G05_2_A10_A	144	102	LMG 2804	*Dickeya*	*chrysanthemi*	
Z0021_LB_G07_2_A03_A	144	102	LMG 2804	*Dickeya*	*chrysanthemi*	
Z0021_RB_G06_2_A10_B	144	102	LMG 2804	*Dickeya*	*chrysanthemi*	
Z0021_RB_G04_2_A11_B	144	102	LMG 2804	*Dickeya*	*chrysanthemi*	
Z0021_RB_G08_2_A09_B	144	102	LMG 2804	*Dickeya*	*chrysanthemi*	
Z0021_RB_G07_2_A09_A	144	102	LMG 2804	*Dickeya*	*chrysanthemi*	
Z0021_LB_G08_2_A03_B	144	102	LMG 2804	*Dickeya*	*chrysanthemi*	
Z0021_LB_G09_2_A02_A	144	102	LMG 2804	*Dickeya*	*chrysanthemi*	
Z0023_RB_G11_2_A07_A	145	120	LMG 5019	*Serratia*	*rubidaea*	
Z0023_RB_G10_2_A08_B	145	120	LMG 5019	*Serratia*	*rubidaea*	
Z0023_LO_A06_1_E03_B	145	120	LMG 5019	*Serratia*	*rubidaea*	
Z0023_LB_H11_1_A01_A	145	120	LMG 5019	*Serratia*	*rubidaea*	
Z0023_LO_A05_1_E03_A	145	120	LMG 5019	*Serratia*	*rubidaea*	
Z0023_LB_H12_1_A01_B	145	120	LMG 5019	*Serratia*	*rubidaea*	
Z0023_RB_G02_2_A12_B	145	120	LMG 5019	*Serratia*	*rubidaea*	
Z0023_LB_H02_1_A06_B	146	120	LMG 5019	*Serratia*	*rubidaea*	
Z0023_LB_H06_1_A04_B	146	120	LMG 5019	*Serratia*	*rubidaea*	
Z0023_LB_H03_1_A05_A	146	120	LMG 5019	*Serratia*	*rubidaea*	
Z0023_RB_G09_2_A08_A	146	120	LMG 5019	*Serratia*	*rubidaea*	
Z0023_RB_F10_1_B08_B	146	120	LMG 5019	*Serratia*	*rubidaea*	
Z0023_LB_H01_1_A06_A	146	120	LMG 5019	*Serratia*	*rubidaea*	
Z0023_RB_G07_2_A09_A	146	120	LMG 5019	*Serratia*	*rubidaea*	
Z0023_RB_F11_1_B07_A	146	120	LMG 5019	*Serratia*	*rubidaea*	

Z0023_RB_F12_1_B07_B	146	120	LMG 5019	*Serratia*	*rubidaea*
Z0023_LO_A03_1_E02_A	147	120	LMG 5019	*Serratia*	*rubidaea*
Z0023_LB_H04_1_A05_B	147	120	LMG 5019	*Serratia*	*rubidaea*
Z0023_RB_G04_2_A11_B	148	120	LMG 5019	*Serratia*	*rubidaea*
Z0023_RB_G05_2_A10_A	148	120	LMG 5019	*Serratia*	*rubidaea*
Z0023_RB_G06_2_A10_B	148	120	LMG 5019	*Serratia*	*rubidaea*
Z0023_LO_A01_1_E01_A	148	120	LMG 5019	*Serratia*	*rubidaea*
Z0023_LB_H07_1_A03_A	148	120	LMG 5019	*Serratia*	*rubidaea*
Z0023_RB_G08_2_A09_B	148	120	LMG 5019	*Serratia*	*rubidaea*
Z0023_LB_H05_1_A04_A	148	120	LMG 5019	*Serratia*	*rubidaea*
Z0023_RB_G03_2_A11_A	148	120	LMG 5019	*Serratia*	*rubidaea*
Z0023_LO_A02_1_E01_B	148	120	LMG 5019	*Serratia*	*rubidaea*
Z0023_LO_A04_1_E02_B	148	120	LMG 5019	*Serratia*	*rubidaea*
Z0023_LB_H10_1_A02_B	148	120	LMG 5019	*Serratia*	*rubidaea*
Z0023_RB_G01_2_A12_A	149	120	LMG 5019	*Serratia*	*rubidaea*
Z0023_LB_H08_1_A03_B	149	120	LMG 5019	*Serratia*	*rubidaea*
Z0023_LB_H09_1_A02_A	149	120	LMG 5019	*Serratia*	*rubidaea*
Z0022_RB_G12_2_A07_B	150	109	LMG 3252	*Citrobacter*	*youngae*
Z0022_LB_H04_1_A05_B	150	109	LMG 3252	*Citrobacter*	*youngae*
Z0022_RB_H01_1_A12_A	150	109	LMG 3252	*Citrobacter*	*youngae*
Z0022_RB_G11_2_A07_A	150	109	LMG 3252	*Citrobacter*	*youngae*
Z0022_LB_G08_2_A03_B	151	109	LMG 3252	*Citrobacter*	*youngae*
Z0022_LB_G05_2_A04_A	151	109	LMG 3252	*Citrobacter*	*youngae*
Z0022_LB_G07_2_A03_A	151	109	LMG 3252	*Citrobacter*	*youngae*
Z0022_LB_G06_2_A04_B	151	109	LMG 3252	*Citrobacter*	*youngae*
Z0022_RB_G06_2_A10_B	152	109	LMG 3252	*Citrobacter*	*youngae*
Z0022_RB_G05_2_A10_A	152	109	LMG 3252	*Citrobacter*	*youngae*
Z0022_RB_G03_2_A11_A	152	109	LMG 3252	*Citrobacter*	*youngae*
Z0022_LB_H02_1_A06_B	152	109	LMG 3252	*Citrobacter*	*youngae*
Z0022_LB_H03_1_A05_A	152	109	LMG 3252	*Citrobacter*	*youngae*
Z0022_RB_G04_2_A11_B	152	109	LMG 3252	*Citrobacter*	*youngae*
Z0022_LB_H01_1_A06_A	152	109	LMG 3252	*Citrobacter*	*youngae*
Z0022_LB_G10_2_A02_B	152	109	LMG 3252	*Citrobacter*	*youngae*
Z0022_LB_G09_2_A02_A	152	109	LMG 3252	*Citrobacter*	*youngae*
Z0022_RB_G02_2_A12_B	152	109	LMG 3252	*Citrobacter*	*youngae*
Z0022_RB_G08_2_A09_B	153	109	LMG 3252	*Citrobacter*	*youngae*
Z0022_LB_G11_2_A01_A	153	109	LMG 3252	*Citrobacter*	*youngae*
Z0022_RB_H02_1_A12_B	153	109	LMG 3252	*Citrobacter*	*youngae*
Z0022_LB_H05_1_A04_A	153	109	LMG 3252	*Citrobacter*	*youngae*
Z0022_LB_H07_1_A03_A	153	109	LMG 3252	*Citrobacter*	*youngae*
Z0022_LB_H06_1_A04_B	153	109	LMG 3252	*Citrobacter*	*youngae*
Z0022_RB_H05_1_A10_A	153	109	LMG 3252	*Citrobacter*	*youngae*
Z0022_LB_H08_1_A03_B	153	109	LMG 3252	*Citrobacter*	*youngae*
Z0022_RB_H04_1_A11_B	153	109	LMG 3252	*Citrobacter*	*youngae*
Z0022_RB_G07_2_A09_A	153	109	LMG 3252	*Citrobacter*	*youngae*
Z0022_RB_H03_1_A11_A	153	109	LMG 3252	*Citrobacter*	*youngae*
Z0022_LB_G12_2_A01_B	153	109	LMG 3252	*Citrobacter*	*youngae*
Z0022_RB_G09_2_A08_A	153	109	LMG 3252	*Citrobacter*	*youngae*
Z0022_RB_G10_2_A08_B	153	109	LMG 3252	*Citrobacter*	*youngae*
Z0019_RO_E08_1_G10_B	154	85	LMG 26064	*Enterobacter*	*asburiae*

Z0019_RO_E06_1_G09_B	154	85	LMG 26064	*Enterobacter*	*asburiae*
Z0019_LO_D12_2_F06_B	154	85	LMG 26064	*Enterobacter*	*asburiae*
Z0019_LO_E02_1_G01_B	154	85	LMG 26064	*Enterobacter*	*asburiae*
Z0019_RO_D06_2_F09_B	154	85	LMG 26064	*Enterobacter*	*asburiae*
Z0019_LO_C12_1_F06_B	154	85	LMG 26064	*Enterobacter*	*asburiae*
Z0019_RO_D07_2_F10_A	154	85	LMG 26064	*Enterobacter*	*asburiae*
Z0019_RO_D08_2_F10_B	154	85	LMG 26064	*Enterobacter*	*asburiae*
Z0019_LO_E06_1_G03_B	154	85	LMG 26064	*Enterobacter*	*asburiae*
Z0019_RO_E10_1_G11_B	154	85	LMG 26064	*Enterobacter*	*asburiae*
Z0019_LO_E04_1_G02_B	154	85	LMG 26064	*Enterobacter*	*asburiae*
Z0019_RO_E12_1_G12_B	154	85	LMG 26064	*Enterobacter*	*asburiae*
Z0019_RO_D01_2_F07_A	154	85	LMG 26064	*Enterobacter*	*asburiae*
Z0019_RO_D02_2_F07_B	154	85	LMG 26064	*Enterobacter*	*asburiae*
Z0019_LO_C11_1_F06_A	154	85	LMG 26064	*Enterobacter*	*asburiae*
Z0019_RO_D03_2_F08_A	154	85	LMG 26064	*Enterobacter*	*asburiae*
Z0019_RO_D12_2_F12_B	154	85	LMG 26064	*Enterobacter*	*asburiae*
Z0019_LO_D06_2_F03_B	154	85	LMG 26064	*Enterobacter*	*asburiae*
Z0019_RO_D09_2_F11_A	154	85	LMG 26064	*Enterobacter*	*asburiae*
Z0019_LO_D05_2_F03_A	154	85	LMG 26064	*Enterobacter*	*asburiae*
Z0019_RO_F05_2_G09_A	155	85	LMG 26064	*Enterobacter*	*asburiae*
Z0019_RO_F04_2_G08_B	155	85	LMG 26064	*Enterobacter*	*asburiae*
Z0019_RO_F07_2_G10_A	155	85	LMG 26064	*Enterobacter*	*asburiae*
Z0019_RO_F06_2_G09_B	155	85	LMG 26064	*Enterobacter*	*asburiae*
Z0019_RO_F08_2_G10_B	155	85	LMG 26064	*Enterobacter*	*asburiae*
Z0019_RO_F02_2_G07_B	155	85	LMG 26064	*Enterobacter*	*asburiae*
Z0019_RO_F03_2_G08_A	155	85	LMG 26064	*Enterobacter*	*asburiae*
Z0019_RO_F01_2_G07_A	155	85	LMG 26064	*Enterobacter*	*asburiae*
Z0019_RO_E04_1_G08_B	155	85	LMG 26064	*Enterobacter*	*asburiae*
Z0019_LO_D10_2_F05_B	155	85	LMG 26064	*Enterobacter*	*asburiae*
Z0019_LO_D09_2_F05_A	155	85	LMG 26064	*Enterobacter*	*asburiae*
Z0019_RO_E01_1_G07_A	155	85	LMG 26064	*Enterobacter*	*asburiae*
Z0019_RO_G10_1_H11_B	156	86	LMG 26121	*Rosenbergiella*	*nectarea*
Z0019_RO_G09_1_H11_A	156	86	LMG 26121	*Rosenbergiella*	*nectarea*
Z0019_LO_G12_1_H06_B	156	86	LMG 26121	*Rosenbergiella*	*nectarea*
Z0019_LO_G08_1_H04_B	156	86	LMG 26121	*Rosenbergiella*	*nectarea*
Z0019_LO_G05_1_H03_A	156	86	LMG 26121	*Rosenbergiella*	*nectarea*
Z0019_RO_G05_1_H09_A	156	86	LMG 26121	*Rosenbergiella*	*nectarea*
Z0019_RO_G04_1_H08_B	156	86	LMG 26121	*Rosenbergiella*	*nectarea*
Z0019_LO_G03_1_H02_A	156	86	LMG 26121	*Rosenbergiella*	*nectarea*
Z0019_LO_G09_1_H05_A	156	86	LMG 26121	*Rosenbergiella*	*nectarea*
Z0019_RO_G03_1_H08_A	156	86	LMG 26121	*Rosenbergiella*	*nectarea*
Z0019_LO_G01_1_H01_A	156	86	LMG 26121	*Rosenbergiella*	*nectarea*
Z0019_LO_F10_2_G05_B	156	86	LMG 26121	*Rosenbergiella*	*nectarea*
Z0019_LO_F11_2_G06_A	156	86	LMG 26121	*Rosenbergiella*	*nectarea*
Z0019_RO_G12_1_H12_B	157	86	LMG 26121	*Rosenbergiella*	*nectarea*
Z0019_RO_G11_1_H12_A	157	86	LMG 26121	*Rosenbergiella*	*nectarea*
Z0019_LO_G06_1_H03_B	157	86	LMG 26121	*Rosenbergiella*	*nectarea*
Z0019_LO_G07_1_H04_A	157	86	LMG 26121	*Rosenbergiella*	*nectarea*
Z0019_RO_G07_1_H10_A	157	86	LMG 26121	*Rosenbergiella*	*nectarea*
Z0019_RO_G06_1_H09_B	157	86	LMG 26121	*Rosenbergiella*	*nectarea*

Z0019_LO_G02_1_H01_B	158	86	LMG 26121	*Rosenbergiella*	*nectarea*
Z0019_RO_G08_1_H10_B	159	86	LMG 26121	*Rosenbergiella*	*nectarea*
Z0019_LO_G11_1_H06_A	159	86	LMG 26121	*Rosenbergiella*	*nectarea*
Z0019_RO_H04_2_H08_B	159	86	LMG 26121	*Rosenbergiella*	*nectarea*
Z0019_RO_H01_2_H07_A	159	86	LMG 26121	*Rosenbergiella*	*nectarea*
Z0019_RO_H02_2_H07_B	159	86	LMG 26121	*Rosenbergiella*	*nectarea*
Z0019_LO_G10_1_H05_B	159	86	LMG 26121	*Rosenbergiella*	*nectarea*
Z0019_LO_H01_2_H01_A	159	86	LMG 26121	*Rosenbergiella*	*nectarea*
Z0019_RO_H03_2_H08_A	159	86	LMG 26121	*Rosenbergiella*	*nectarea*
Z0019_LO_F07_2_G04_A	160	86	LMG 26121	*Rosenbergiella*	*nectarea*
Z0019_LO_F06_2_G03_B	160	86	LMG 26121	*Rosenbergiella*	*nectarea*
Z0019_LO_F09_2_G05_A	160	86	LMG 26121	*Rosenbergiella*	*nectarea*
Z0019_LO_F08_2_G04_B	160	86	LMG 26121	*Rosenbergiella*	*nectarea*
Z0024_RB_C04_2_C11_B	161	124	LMG 6519	*Oligella*	*ureolytica*
Z0024_RB_C05_2_C10_A	161	124	LMG 6519	*Oligella*	*ureolytica*
Z0024_RB_C03_2_C11_A	161	124	LMG 6519	*Oligella*	*ureolytica*
Z0024_LB_C07_2_C03_A	161	124	LMG 6519	*Oligella*	*ureolytica*
Z0024_LB_C02_2_C06_B	161	124	LMG 6519	*Oligella*	*ureolytica*
Z0024_RB_C02_2_C12_B	161	124	LMG 6519	*Oligella*	*ureolytica*
Z0024_RB_C07_2_C09_A	161	124	LMG 6519	*Oligella*	*ureolytica*
Z0024_LB_B09_1_D02_A	161	124	LMG 6519	*Oligella*	*ureolytica*
Z0024_RB_C08_2_C09_B	161	124	LMG 6519	*Oligella*	*ureolytica*
Z0024_LB_B08_1_D03_B	161	124	LMG 6519	*Oligella*	*ureolytica*
Z0024_RB_C09_2_C08_A	162	124	LMG 6519	*Oligella*	*ureolytica*
Z0024_LB_B07_1_D03_A	162	124	LMG 6519	*Oligella*	*ureolytica*
Z0024_LB_B12_1_D01_B	163	124	LMG 6519	*Oligella*	*ureolytica*
Z0024_LB_C03_2_C05_A	163	124	LMG 6519	*Oligella*	*ureolytica*
Z0024_LB_C06_2_C04_B	163	124	LMG 6519	*Oligella*	*ureolytica*
Z0024_LB_B10_1_D02_B	163	124	LMG 6519	*Oligella*	*ureolytica*
Z0024_LB_C09_2_C02_A	163	124	LMG 6519	*Oligella*	*ureolytica*
Z0024_RB_D01_1_C12_A	163	124	LMG 6519	*Oligella*	*ureolytica*
Z0024_LB_B05_1_D04_A	163	124	LMG 6519	*Oligella*	*ureolytica*
Z0024_RB_C10_2_C08_B	163	124	LMG 6519	*Oligella*	*ureolytica*
Z0024_LB_C01_2_C06_A	163	124	LMG 6519	*Oligella*	*ureolytica*
Z0024_RB_C12_2_C07_B	163	124	LMG 6519	*Oligella*	*ureolytica*
Z0024_LB_B11_1_D01_A	163	124	LMG 6519	*Oligella*	*ureolytica*
Z0024_RB_C06_2_C10_B	163	124	LMG 6519	*Oligella*	*ureolytica*
Z0024_RB_C01_2_C12_A	163	124	LMG 6519	*Oligella*	*ureolytica*
Z0024_LB_B04_1_D05_B	163	124	LMG 6519	*Oligella*	*ureolytica*
Z0024_RB_C11_2_C07_A	164	124	LMG 6519	*Oligella*	*ureolytica*
Z0024_LB_C08_2_C03_B	164	124	LMG 6519	*Oligella*	*ureolytica*
Z0024_RB_D02_1_C12_B	164	124	LMG 6519	*Oligella*	*ureolytica*
Z0024_LB_C04_2_C05_B	164	124	LMG 6519	*Oligella*	*ureolytica*
Z0024_LB_C05_2_C04_A	164	124	LMG 6519	*Oligella*	*ureolytica*
Z0024_LB_B06_1_D04_B	165	124	LMG 6519	*Oligella*	*ureolytica*
Z0021_RB_E03_2_B11_A	166	100	LMG 27282	*Noviherbaspirillum*	*psychrotolerans*
Z0021_LB_E08_2_B03_B	166	100	LMG 27282	*Noviherbaspirillum*	*psychrotolerans*
Z0021_RB_E04_2_B11_B	166	100	LMG 27282	*Noviherbaspirillum*	*psychrotolerans*
Z0021_LB_E07_2_B03_A	166	100	LMG 27282	*Noviherbaspirillum*	*psychrotolerans*
Z0021_LB_E06_2_B04_B	166	100	LMG 27282	*Noviherbaspirillum*	*psychrotolerans*

Z0021_RB_D10_1_C08_B	166	100	LMG 27282	*Noviherbaspirillum*	*psychrotolerans*
Z0021_RB_D09_1_C08_A	166	100	LMG 27282	*Noviherbaspirillum*	*psychrotolerans*
Z0021_RB_D08_1_C09_B	166	100	LMG 27282	*Noviherbaspirillum*	*psychrotolerans*
Z0021_RB_E06_2_B10_B	166	100	LMG 27282	*Noviherbaspirillum*	*psychrotolerans*
Z0021_LB_E10_2_B02_B	166	100	LMG 27282	*Noviherbaspirillum*	*psychrotolerans*
Z0021_RB_E05_2_B10_A	166	100	LMG 27282	*Noviherbaspirillum*	*psychrotolerans*
Z0021_RB_D11_1_C07_A	166	100	LMG 27282	*Noviherbaspirillum*	*psychrotolerans*
Z0021_LB_E09_2_B02_A	166	100	LMG 27282	*Noviherbaspirillum*	*psychrotolerans*
Z0021_LB_D11_1_C01_A	166	100	LMG 27282	*Noviherbaspirillum*	*psychrotolerans*
Z0021_LB_D12_1_C01_B	166	100	LMG 27282	*Noviherbaspirillum*	*psychrotolerans*
Z0021_RB_D04_1_C11_B	166	100	LMG 27282	*Noviherbaspirillum*	*psychrotolerans*
Z0021_LB_D10_1_C02_B	166	100	LMG 27282	*Noviherbaspirillum*	*psychrotolerans*
Z0021_LB_E05_2_B04_A	166	100	LMG 27282	*Noviherbaspirillum*	*psychrotolerans*
Z0021_RB_E02_2_B12_B	166	100	LMG 27282	*Noviherbaspirillum*	*psychrotolerans*
Z0021_RB_E01_2_B12_A	166	100	LMG 27282	*Noviherbaspirillum*	*psychrotolerans*
Z0021_RB_D12_1_C07_B	166	100	LMG 27282	*Noviherbaspirillum*	*psychrotolerans*
Z0021_LB_D08_1_C03_B	167	100	LMG 27282	*Noviherbaspirillum*	*psychrotolerans*
Z0021_LB_D07_1_C03_A	167	100	LMG 27282	*Noviherbaspirillum*	*psychrotolerans*
Z0021_RB_D03_1_C11_A	167	100	LMG 27282	*Noviherbaspirillum*	*psychrotolerans*
Z0021_LB_D09_1_C02_A	167	100	LMG 27282	*Noviherbaspirillum*	*psychrotolerans*
Z0021_LB_E02_2_B06_B	167	100	LMG 27282	*Noviherbaspirillum*	*psychrotolerans*
Z0021_LB_E03_2_B05_A	168	100	LMG 27282	*Noviherbaspirillum*	*psychrotolerans*
Z0021_LB_E04_2_B05_B	168	100	LMG 27282	*Noviherbaspirillum*	*psychrotolerans*
Z0021_RB_D05_1_C10_A	169	100	LMG 27282	*Noviherbaspirillum*	*psychrotolerans*
Z0021_RB_D07_1_C09_A	169	100	LMG 27282	*Noviherbaspirillum*	*psychrotolerans*
Z0021_LB_E01_2_B06_A	169	100	LMG 27282	*Noviherbaspirillum*	*psychrotolerans*
Z0021_RB_D06_1_C10_B	169	100	LMG 27282	*Noviherbaspirillum*	*psychrotolerans*
Z0019_LO_E12_1_G06_B	170	87	LMG 26149	*Herbaspirillum*	*soli*
Z0019_LO_E07_1_G04_A	170	87	LMG 26149	*Herbaspirillum*	*soli*
Z0019_RO_D04_2_F08_B	170	87	LMG 26149	*Herbaspirillum*	*soli*
Z0019_RO_E09_1_G11_A	170	87	LMG 26149	*Herbaspirillum*	*soli*
Z0019_LO_E09_1_G05_A	170	87	LMG 26149	*Herbaspirillum*	*soli*
Z0019_LO_E11_1_G06_A	170	87	LMG 26149	*Herbaspirillum*	*soli*
Z0019_LO_E10_1_G05_B	170	87	LMG 26149	*Herbaspirillum*	*soli*
Z0019_RO_G02_1_H07_B	170	87	LMG 26149	*Herbaspirillum*	*soli*
Z0019_RO_E11_1_G12_A	170	87	LMG 26149	*Herbaspirillum*	*soli*
Z0019_RO_E07_1_G10_A	170	87	LMG 26149	*Herbaspirillum*	*soli*
Z0019_RO_G01_1_H07_A	170	87	LMG 26149	*Herbaspirillum*	*soli*
Z0019_RO_F12_2_G12_B	170	87	LMG 26149	*Herbaspirillum*	*soli*
Z0019_RO_E02_1_G07_B	170	87	LMG 26149	*Herbaspirillum*	*soli*
Z0019_LO_F04_2_G02_B	170	87	LMG 26149	*Herbaspirillum*	*soli*
Z0019_LO_F03_2_G02_A	170	87	LMG 26149	*Herbaspirillum*	*soli*
Z0019_LO_E01_1_G01_A	170	87	LMG 26149	*Herbaspirillum*	*soli*
Z0019_LO_E05_1_G03_A	170	87	LMG 26149	*Herbaspirillum*	*soli*
Z0019_RO_E05_1_G09_A	170	87	LMG 26149	*Herbaspirillum*	*soli*
Z0019_LO_F02_2_G01_B	170	87	LMG 26149	*Herbaspirillum*	*soli*
Z0019_RO_E03_1_G08_A	170	87	LMG 26149	*Herbaspirillum*	*soli*
Z0019_LO_E03_1_G02_A	170	87	LMG 26149	*Herbaspirillum*	*soli*
Z0019_RO_F09_2_G11_A	170	87	LMG 26149	*Herbaspirillum*	*soli*
Z0019_LO_G04_1_H02_B	170	87	LMG 26149	*Herbaspirillum*	*soli*

Z0019_LO_F05_2_G03_A	170	87	LMG 26149	*Herbaspirillum*	*soli*
Z0019_RO_D10_2_F11_B	170	87	LMG 26149	*Herbaspirillum*	*soli*
Z0019_LO_D11_2_F06_A	170	87	LMG 26149	*Herbaspirillum*	*soli*
Z0019_LO_F01_2_G01_A	170	87	LMG 26149	*Herbaspirillum*	*soli*
Z0019_RO_D11_2_F12_A	170	87	LMG 26149	*Herbaspirillum*	*soli*
Z0019_RO_F11_2_G12_A	170	87	LMG 26149	*Herbaspirillum*	*soli*
Z0019_RO_F10_2_G11_B	170	87	LMG 26149	*Herbaspirillum*	*soli*
Z0019_LO_E08_1_G04_B	170	87	LMG 26149	*Herbaspirillum*	*soli*
Z0019_LO_F12_2_G06_B	170	87	LMG 26149	*Herbaspirillum*	*soli*
Z0014_LO_B06_2_E03_B	171	28	LMG 18919	*Microvirgula*	*aerodenitrificans*
Z0014_LO_B07_2_E04_A	171	28	LMG 18919	*Microvirgula*	*aerodenitrificans*
Z0014_RO_C02_1_F07_B	171	28	LMG 18919	*Microvirgula*	*aerodenitrificans*
Z0014_RO_C01_1_F07_A	171	28	LMG 18919	*Microvirgula*	*aerodenitrificans*
Z0014_LO_C05_1_F03_A	171	28	LMG 18919	*Microvirgula*	*aerodenitrificans*
Z0014_LO_C02_1_F01_B	171	28	LMG 18919	*Microvirgula*	*aerodenitrificans*
Z0014_LO_C04_1_F02_B	171	28	LMG 18919	*Microvirgula*	*aerodenitrificans*
Z0014_LO_C01_1_F01_A	171	28	LMG 18919	*Microvirgula*	*aerodenitrificans*
Z0014_LO_B12_2_E06_B	171	28	LMG 18919	*Microvirgula*	*aerodenitrificans*
Z0014_RO_C03_1_F08_A	171	28	LMG 18919	*Microvirgula*	*aerodenitrificans*
Z0014_LO_A10_1_E05_B	171	28	LMG 18919	*Microvirgula*	*aerodenitrificans*
Z0014_RO_C04_1_F08_B	171	28	LMG 18919	*Microvirgula*	*aerodenitrificans*
Z0014_LO_B05_2_E03_A	171	28	LMG 18919	*Microvirgula*	*aerodenitrificans*
Z0014_LO_A11_1_E06_A	171	28	LMG 18919	*Microvirgula*	*aerodenitrificans*
Z0014_RO_B09_2_E11_A	171	28	LMG 18919	*Microvirgula*	*aerodenitrificans*
Z0014_LO_A09_1_E05_A	171	28	LMG 18919	*Microvirgula*	*aerodenitrificans*
Z0014_RO_B12_2_E12_B	171	28	LMG 18919	*Microvirgula*	*aerodenitrificans*
Z0014_LO_B08_2_E04_B	171	28	LMG 18919	*Microvirgula*	*aerodenitrificans*
Z0014_LO_B09_2_E05_A	171	28	LMG 18919	*Microvirgula*	*aerodenitrificans*
Z0014_RO_B10_2_E11_B	171	28	LMG 18919	*Microvirgula*	*aerodenitrificans*
Z0014_LO_B02_2_E01_B	171	28	LMG 18919	*Microvirgula*	*aerodenitrificans*
Z0014_LO_B11_2_E06_A	171	28	LMG 18919	*Microvirgula*	*aerodenitrificans*
Z0014_LO_B10_2_E05_B	171	28	LMG 18919	*Microvirgula*	*aerodenitrificans*
Z0014_RO_B11_2_E12_A	171	28	LMG 18919	*Microvirgula*	*aerodenitrificans*
Z0014_LO_B01_2_E01_A	171	28	LMG 18919	*Microvirgula*	*aerodenitrificans*
Z0014_LO_A12_1_E06_B	171	28	LMG 18919	*Microvirgula*	*aerodenitrificans*
Z0014_LO_B03_2_E02_A	171	28	LMG 18919	*Microvirgula*	*aerodenitrificans*
Z0014_LO_B04_2_E02_B	171	28	LMG 18919	*Microvirgula*	*aerodenitrificans*
Z0017_RO_B05_2_E09_A	172	66	LMG 24163	*seudoxanthomona*	*dokdonensis*
Z0017_LO_B02_2_E01_B	172	66	LMG 24163	*seudoxanthomona*	*dokdonensis*
Z0017_RO_B04_2_E08_B	172	66	LMG 24163	*seudoxanthomona*	*dokdonensis*
Z0017_RO_B06_2_E09_B	172	66	LMG 24163	*seudoxanthomona*	*dokdonensis*
Z0017_RO_C03_1_F08_A	172	66	LMG 24163	*seudoxanthomona*	*dokdonensis*
Z0017_LO_B06_2_E03_B	172	66	LMG 24163	*seudoxanthomona*	*dokdonensis*
Z0017_LO_B01_2_E01_A	172	66	LMG 24163	*seudoxanthomona*	*dokdonensis*
Z0017_RO_C04_1_F08_B	172	66	LMG 24163	*seudoxanthomona*	*dokdonensis*
Z0017_LO_B05_2_E03_A	172	66	LMG 24163	*seudoxanthomona*	*dokdonensis*
Z0017_RO_B03_2_E08_A	172	66	LMG 24163	*seudoxanthomona*	*dokdonensis*
Z0017_LO_B04_2_E02_B	172	66	LMG 24163	*seudoxanthomona*	*dokdonensis*
Z0017_LO_C04_1_F02_B	172	66	LMG 24163	*seudoxanthomona*	*dokdonensis*
Z0017_LO_C01_1_F01_A	172	66	LMG 24163	*seudoxanthomona*	*dokdonensis*

Z0017_RB_H12_1_A07_B	172	66	LMG 24163	*seudoxanthomona*	*dokdonensis*
Z0017_LO_A04_1_E02_B	172	66	LMG 24163	*seudoxanthomona*	*dokdonensis*
Z0017_LO_A07_1_E04_A	172	66	LMG 24163	*seudoxanthomona*	*dokdonensis*
Z0017_LB_H12_1_A01_B	173	66	LMG 24163	*seudoxanthomona*	*dokdonensis*
Z0017_LO_A01_1_E01_A	173	66	LMG 24163	*seudoxanthomona*	*dokdonensis*
Z0017_RO_A01_1_E07_A	173	66	LMG 24163	*seudoxanthomona*	*dokdonensis*
Z0017_RB_H09_1_A08_A	173	66	LMG 24163	*seudoxanthomona*	*dokdonensis*
Z0017_LO_A03_1_E02_A	174	66	LMG 24163	*seudoxanthomona*	*dokdonensis*
Z0017_RO_C11_1_F12_A	175	66	LMG 24163	*seudoxanthomona*	*dokdonensis*
Z0017_LO_C09_1_F05_A	175	66	LMG 24163	*seudoxanthomona*	*dokdonensis*
Z0017_RO_C08_1_F10_B	175	66	LMG 24163	*seudoxanthomona*	*dokdonensis*
Z0017_RO_B10_2_E11_B	175	66	LMG 24163	*seudoxanthomona*	*dokdonensis*
Z0017_LO_D05_2_F03_A	175	66	LMG 24163	*seudoxanthomona*	*dokdonensis*
Z0017_LO_C12_1_F06_B	175	66	LMG 24163	*seudoxanthomona*	*dokdonensis*
Z0017_RO_C06_1_F09_B	175	66	LMG 24163	*seudoxanthomona*	*dokdonensis*
Z0017_LO_C07_1_F04_A	175	66	LMG 24163	*seudoxanthomona*	*dokdonensis*
Z0017_LO_D02_2_F01_B	175	66	LMG 24163	*seudoxanthomona*	*dokdonensis*
Z0017_LO_C11_1_F06_A	175	66	LMG 24163	*seudoxanthomona*	*dokdonensis*
Z0017_RO_C09_1_F11_A	175	66	LMG 24163	*seudoxanthomona*	*dokdonensis*
Z0018_LB_D05_1_C04_A	176	69	LMG 24401	*Epilithonimonas*	*lactis*
Z0018_LB_D07_1_C03_A	176	69	LMG 24401	*Epilithonimonas*	*lactis*
Z0018_RB_C07_2_C09_A	176	69	LMG 24401	*Epilithonimonas*	*lactis*
Z0018_LB_C09_2_C02_A	176	69	LMG 24401	*Epilithonimonas*	*lactis*
Z0018_LB_D06_1_C04_B	176	69	LMG 24401	*Epilithonimonas*	*lactis*
Z0018_RB_C09_2_C08_A	176	69	LMG 24401	*Epilithonimonas*	*lactis*
Z0018_RB_C11_2_C07_A	176	69	LMG 24401	*Epilithonimonas*	*lactis*
Z0018_LB_D08_1_C03_B	176	69	LMG 24401	*Epilithonimonas*	*lactis*
Z0018_RB_C02_2_C12_B	176	69	LMG 24401	*Epilithonimonas*	*lactis*
Z0018_LB_C12_2_C01_B	176	69	LMG 24401	*Epilithonimonas*	*lactis*
Z0018_RB_C12_2_C07_B	176	69	LMG 24401	*Epilithonimonas*	*lactis*
Z0018_LB_C10_2_C02_B	176	69	LMG 24401	*Epilithonimonas*	*lactis*
Z0018_RB_C10_2_C08_B	176	69	LMG 24401	*Epilithonimonas*	*lactis*
Z0018_RB_C06_2_C10_B	176	69	LMG 24401	*Epilithonimonas*	*lactis*
Z0018_LB_D04_1_C05_B	176	69	LMG 24401	*Epilithonimonas*	*lactis*
Z0018_RB_C05_2_C10_A	176	69	LMG 24401	*Epilithonimonas*	*lactis*
Z0018_LB_D03_1_C05_A	176	69	LMG 24401	*Epilithonimonas*	*lactis*
Z0018_RB_B08_1_D09_B	176	69	LMG 24401	*Epilithonimonas*	*lactis*
Z0018_LB_C08_2_C03_B	176	69	LMG 24401	*Epilithonimonas*	*lactis*
Z0018_RB_B09_1_D08_A	176	69	LMG 24401	*Epilithonimonas*	*lactis*
Z0018_RB_B10_1_D08_B	176	69	LMG 24401	*Epilithonimonas*	*lactis*
Z0018_RB_B11_1_D07_A	176	69	LMG 24401	*Epilithonimonas*	*lactis*
Z0018_RB_B12_1_D07_B	176	69	LMG 24401	*Epilithonimonas*	*lactis*
Z0018_RB_C08_2_C09_B	176	69	LMG 24401	*Epilithonimonas*	*lactis*
Z0018_RB_C01_2_C12_A	176	69	LMG 24401	*Epilithonimonas*	*lactis*
Z0018_LB_C11_2_C01_A	176	69	LMG 24401	*Epilithonimonas*	*lactis*
Z0018_LB_D09_1_C02_A	176	69	LMG 24401	*Epilithonimonas*	*lactis*
Z0018_RB_C04_2_C11_B	176	69	LMG 24401	*Epilithonimonas*	*lactis*
Z0018_RB_C03_2_C11_A	176	69	LMG 24401	*Epilithonimonas*	*lactis*
Z0018_LB_D01_1_C06_A	176	69	LMG 24401	*Epilithonimonas*	*lactis*
Z0018_LB_D02_1_C06_B	176	69	LMG 24401	*Epilithonimonas*	*lactis*

Z0018_RB_D01_1_C12_A	176	69	LMG 24401	*Epilithonimonas*	*lactis*
Z0014_RB_B12_1_D07_B	177	25	LMG 18212	*Chryseobacterium*	*joostei*
Z0014_LB_B06_1_D04_B	177	25	LMG 18212	*Chryseobacterium*	*joostei*
Z0014_LB_B07_1_D03_A	177	25	LMG 18212	*Chryseobacterium*	*joostei*
Z0014_LB_B04_1_D05_B	177	25	LMG 18212	*Chryseobacterium*	*joostei*
Z0014_LB_B08_1_D03_B	177	25	LMG 18212	*Chryseobacterium*	*joostei*
Z0014_RB_C07_2_C09_A	177	25	LMG 18212	*Chryseobacterium*	*joostei*
Z0014_LB_C07_2_C03_A	177	25	LMG 18212	*Chryseobacterium*	*joostei*
Z0014_LB_C09_2_C02_A	177	25	LMG 18212	*Chryseobacterium*	*joostei*
Z0014_LB_C08_2_C03_B	177	25	LMG 18212	*Chryseobacterium*	*joostei*
Z0014_RB_B11_1_D07_A	177	25	LMG 18212	*Chryseobacterium*	*joostei*
Z0014_LB_B05_1_D04_A	177	25	LMG 18212	*Chryseobacterium*	*joostei*
Z0014_RB_B10_1_D08_B	177	25	LMG 18212	*Chryseobacterium*	*joostei*
Z0014_LB_B12_1_D01_B	177	25	LMG 18212	*Chryseobacterium*	*joostei*
Z0014_LB_B11_1_D01_A	177	25	LMG 18212	*Chryseobacterium*	*joostei*
Z0014_LB_C01_2_C06_A	177	25	LMG 18212	*Chryseobacterium*	*joostei*
Z0014_RB_C03_2_C11_A	177	25	LMG 18212	*Chryseobacterium*	*joostei*
Z0014_RB_C06_2_C10_B	177	25	LMG 18212	*Chryseobacterium*	*joostei*
Z0014_RB_C02_2_C12_B	177	25	LMG 18212	*Chryseobacterium*	*joostei*
Z0014_RB_C04_2_C11_B	177	25	LMG 18212	*Chryseobacterium*	*joostei*
Z0014_LB_C04_2_C05_B	177	25	LMG 18212	*Chryseobacterium*	*joostei*
Z0014_LB_C03_2_C05_A	177	25	LMG 18212	*Chryseobacterium*	*joostei*
Z0014_RB_C05_2_C10_A	177	25	LMG 18212	*Chryseobacterium*	*joostei*
Z0014_LB_C05_2_C04_A	177	25	LMG 18212	*Chryseobacterium*	*joostei*
Z0014_LB_C06_2_C04_B	177	25	LMG 18212	*Chryseobacterium*	*joostei*
Z0014_LB_C02_2_C06_B	177	25	LMG 18212	*Chryseobacterium*	*joostei*
Z0014_RB_B08_1_D09_B	177	25	LMG 18212	*Chryseobacterium*	*joostei*
Z0014_RB_B09_1_D08_A	177	25	LMG 18212	*Chryseobacterium*	*joostei*
Z0014_LB_B10_1_D02_B	177	25	LMG 18212	*Chryseobacterium*	*joostei*
Z0014_RB_B07_1_D09_A	177	25	LMG 18212	*Chryseobacterium*	*joostei*
Z0014_LB_B09_1_D02_A	177	25	LMG 18212	*Chryseobacterium*	*joostei*
Z0014_LB_B03_1_D05_A	177	25	LMG 18212	*Chryseobacterium*	*joostei*
Z0014_RB_C01_2_C12_A	177	25	LMG 18212	*Chryseobacterium*	*joostei*
Z0018_RB_E01_2_B12_A	178	70	LMG 24411	*Bhargavaea*	*cecembensis*
Z0018_RB_E02_2_B12_B	178	70	LMG 24411	*Bhargavaea*	*cecembensis*
Z0018_LB_E11_2_B01_A	178	70	LMG 24411	*Bhargavaea*	*cecembensis*
Z0018_LB_E12_2_B01_B	178	70	LMG 24411	*Bhargavaea*	*cecembensis*
Z0018_RB_D09_1_C08_A	178	70	LMG 24411	*Bhargavaea*	*cecembensis*
Z0018_LB_D12_1_C01_B	178	70	LMG 24411	*Bhargavaea*	*cecembensis*
Z0018_RB_D08_1_C09_B	178	70	LMG 24411	*Bhargavaea*	*cecembensis*
Z0018_RB_D11_1_C07_A	178	70	LMG 24411	*Bhargavaea*	*cecembensis*
Z0018_RB_D10_1_C08_B	178	70	LMG 24411	*Bhargavaea*	*cecembensis*
Z0018_LB_E03_2_B05_A	178	70	LMG 24411	*Bhargavaea*	*cecembensis*
Z0018_LB_F02_1_B06_B	178	70	LMG 24411	*Bhargavaea*	*cecembensis*
Z0018_LB_F01_1_B06_A	178	70	LMG 24411	*Bhargavaea*	*cecembensis*
Z0018_LB_F03_1_B05_A	178	70	LMG 24411	*Bhargavaea*	*cecembensis*
Z0018_RB_E03_2_B11_A	178	70	LMG 24411	*Bhargavaea*	*cecembensis*
Z0018_LB_E01_2_B06_A	178	70	LMG 24411	*Bhargavaea*	*cecembensis*
Z0018_LB_E04_2_B05_B	179	70	LMG 24411	*Bhargavaea*	*cecembensis*
Z0018_LB_E05_2_B04_A	179	70	LMG 24411	*Bhargavaea*	*cecembensis*

Z0018_RB_D12_1_C07_B	179	70	LMG 24411	*Bhargavaea*	*cecembensis*
Z0018_LB_E06_2_B04_B	179	70	LMG 24411	*Bhargavaea*	*cecembensis*
Z0018_RB_D03_1_C11_A	179	70	LMG 24411	*Bhargavaea*	*cecembensis*
Z0018_LB_D10_1_C02_B	179	70	LMG 24411	*Bhargavaea*	*cecembensis*
Z0018_LB_D11_1_C01_A	179	70	LMG 24411	*Bhargavaea*	*cecembensis*
Z0018_RB_D02_1_C12_B	179	70	LMG 24411	*Bhargavaea*	*cecembensis*
Z0018_RB_D04_1_C11_B	179	70	LMG 24411	*Bhargavaea*	*cecembensis*
Z0018_LB_E10_2_B02_B	179	70	LMG 24411	*Bhargavaea*	*cecembensis*
Z0018_RB_D07_1_C09_A	179	70	LMG 24411	*Bhargavaea*	*cecembensis*
Z0018_RB_D06_1_C10_B	179	70	LMG 24411	*Bhargavaea*	*cecembensis*
Z0018_RB_D05_1_C10_A	179	70	LMG 24411	*Bhargavaea*	*cecembensis*
Z0018_LB_E08_2_B03_B	179	70	LMG 24411	*Bhargavaea*	*cecembensis*
Z0018_LB_E09_2_B02_A	179	70	LMG 24411	*Bhargavaea*	*cecembensis*
Z0018_LB_E07_2_B03_A	179	70	LMG 24411	*Bhargavaea*	*cecembensis*
Z0018_LB_E02_2_B06_B	180	70	LMG 24411	*Bhargavaea*	*cecembensis*
Z0024_RO_C11_1_F12_A	181	129	LMG 6928	*Sporosarcina*	*globispora*
Z0024_LO_E09_1_G05_A	181	129	LMG 6928	*Sporosarcina*	*globispora*
Z0024_RO_B08_2_E10_B	181	129	LMG 6928	*Sporosarcina*	*globispora*
Z0024_LO_D05_2_F03_A	181	129	LMG 6928	*Sporosarcina*	*globispora*
Z0024_LO_E06_1_G03_B	181	129	LMG 6928	*Sporosarcina*	*globispora*
Z0024_LO_E02_1_G01_B	181	129	LMG 6928	*Sporosarcina*	*globispora*
Z0024_LO_D10_2_F05_B	181	129	LMG 6928	*Sporosarcina*	*globispora*
Z0024_LO_E04_1_G02_B	181	129	LMG 6928	*Sporosarcina*	*globispora*
Z0024_RO_C10_1_F11_B	181	129	LMG 6928	*Sporosarcina*	*globispora*
Z0024_RO_C03_1_F08_A	181	129	LMG 6928	*Sporosarcina*	*globispora*
Z0024_LO_E01_1_G01_A	181	129	LMG 6928	*Sporosarcina*	*globispora*
Z0024_RO_B10_2_E11_B	181	129	LMG 6928	*Sporosarcina*	*globispora*
Z0024_LO_D07_2_F04_A	181	129	LMG 6928	*Sporosarcina*	*globispora*
Z0024_RO_C01_1_F07_A	181	129	LMG 6928	*Sporosarcina*	*globispora*
Z0024_RO_B12_2_E12_B	181	129	LMG 6928	*Sporosarcina*	*globispora*
Z0024_LO_D12_2_F06_B	181	129	LMG 6928	*Sporosarcina*	*globispora*
Z0024_LO_D03_2_F02_A	181	129	LMG 6928	*Sporosarcina*	*globispora*
Z0024_RO_B06_2_E09_B	181	129	LMG 6928	*Sporosarcina*	*globispora*
Z0024_LO_C12_1_F06_B	181	129	LMG 6928	*Sporosarcina*	*globispora*
Z0024_LO_C10_1_F05_B	181	129	LMG 6928	*Sporosarcina*	*globispora*
Z0024_RO_B01_2_E07_A	181	129	LMG 6928	*Sporosarcina*	*globispora*
Z0024_LO_C08_1_F04_B	181	129	LMG 6928	*Sporosarcina*	*globispora*
Z0024_RO_B03_2_E08_A	181	129	LMG 6928	*Sporosarcina*	*globispora*
Z0024_RO_C08_1_F10_B	181	129	LMG 6928	*Sporosarcina*	*globispora*
Z0024_RO_C06_1_F09_B	181	129	LMG 6928	*Sporosarcina*	*globispora*
Z0024_RO_B02_2_E07_B	181	129	LMG 6928	*Sporosarcina*	*globispora*
Z0024_LO_D01_2_F01_A	181	129	LMG 6928	*Sporosarcina*	*globispora*
Z0024_RO_B05_2_E09_A	181	129	LMG 6928	*Sporosarcina*	*globispora*
Z0015_LO_H12_2_H06_B	182	34	LMG 21276	*Psychrobacter*	*luti*
Z0015_LB_B09_1_D02_A	182	34	LMG 21276	*Psychrobacter*	*luti*
Z0015_RB_A01_2_D12_A	182	34	LMG 21276	*Psychrobacter*	*luti*
Z0015_LB_B08_1_D03_B	182	34	LMG 21276	*Psychrobacter*	*luti*
Z0015_RB_B02_1_D12_B	182	34	LMG 21276	*Psychrobacter*	*luti*
Z0015_LB_C10_2_C02_B	182	34	LMG 21276	*Psychrobacter*	*luti*
Z0015_RB_B04_1_D11_B	182	34	LMG 21276	*Psychrobacter*	*luti*

Z0015_RB_B03_1_D11_A	182	34	LMG 21276	*Psychrobacter*	*luti*
Z0015_LB_B10_1_D02_B	182	34	LMG 21276	*Psychrobacter*	*luti*
Z0015_LB_C03_2_C05_A	182	34	LMG 21276	*Psychrobacter*	*luti*
Z0015_LB_C02_2_C06_B	182	34	LMG 21276	*Psychrobacter*	*luti*
Z0015_RB_A10_2_D08_B	182	34	LMG 21276	*Psychrobacter*	*luti*
Z0015_RB_A11_2_D07_A	182	34	LMG 21276	*Psychrobacter*	*luti*
Z0015_LB_C04_2_C05_B	182	34	LMG 21276	*Psychrobacter*	*luti*
Z0015_RB_A03_2_D11_A	182	34	LMG 21276	*Psychrobacter*	*luti*
Z0015_LB_C05_2_C04_A	182	34	LMG 21276	*Psychrobacter*	*luti*
Z0015_LB_C07_2_C03_A	182	34	LMG 21276	*Psychrobacter*	*luti*
Z0015_LB_C06_2_C04_B	182	34	LMG 21276	*Psychrobacter*	*luti*
Z0015_RB_B01_1_D12_A	182	34	LMG 21276	*Psychrobacter*	*luti*
Z0015_LB_C08_2_C03_B	182	34	LMG 21276	*Psychrobacter*	*luti*
Z0015_LB_C09_2_C02_A	182	34	LMG 21276	*Psychrobacter*	*luti*
Z0015_RB_A12_2_D07_B	182	34	LMG 21276	*Psychrobacter*	*luti*
Z0015_RB_A07_2_D09_A	182	34	LMG 21276	*Psychrobacter*	*luti*
Z0015_RB_A02_2_D12_B	182	34	LMG 21276	*Psychrobacter*	*luti*
Z0015_RB_A09_2_D08_A	182	34	LMG 21276	*Psychrobacter*	*luti*
Z0015_LB_C01_2_C06_A	182	34	LMG 21276	*Psychrobacter*	*luti*
Z0015_RB_A05_2_D10_A	182	34	LMG 21276	*Psychrobacter*	*luti*
Z0015_LB_B11_1_D01_A	182	34	LMG 21276	*Psychrobacter*	*luti*
Z0015_RB_A06_2_D10_B	182	34	LMG 21276	*Psychrobacter*	*luti*
Z0015_LB_B12_1_D01_B	182	34	LMG 21276	*Psychrobacter*	*luti*
Z0015_RB_A08_2_D09_B	182	34	LMG 21276	*Psychrobacter*	*luti*
Z0015_RB_A04_2_D11_B	183	34	LMG 21276	*Psychrobacter*	*luti*
Z0012_RB_A04_2_D11_B	184	3	LMG 11194	*Moraxella*	*canis*
Z0012_LB_A01_2_D06_A	184	3	LMG 11194	*Moraxella*	*canis*
Z0012_RB_A09_2_D08_A	184	3	LMG 11194	*Moraxella*	*canis*
Z0012_LB_A04_2_D05_B	184	3	LMG 11194	*Moraxella*	*canis*
Z0012_LB_A05_2_D04_A	184	3	LMG 11194	*Moraxella*	*canis*
Z0012_RB_A12_2_D07_B	184	3	LMG 11194	*Moraxella*	*canis*
Z0012_LB_A09_2_D02_A	184	3	LMG 11194	*Moraxella*	*canis*
Z0012_RB_B01_1_D12_A	184	3	LMG 11194	*Moraxella*	*canis*
Z0012_RB_F07_1_B09_A	184	3	LMG 11194	*Moraxella*	*canis*
Z0012_RB_F08_1_B09_B	184	3	LMG 11194	*Moraxella*	*canis*
Z0012_RB_F05_1_B10_A	185	3	LMG 11194	*Moraxella*	*canis*
Z0012_LB_F11_1_B01_A	185	3	LMG 11194	*Moraxella*	*canis*
Z0012_RB_A11_2_D07_A	185	3	LMG 11194	*Moraxella*	*canis*
Z0012_LB_A08_2_D03_B	185	3	LMG 11194	*Moraxella*	*canis*
Z0012_RB_B02_1_D12_B	185	3	LMG 11194	*Moraxella*	*canis*
Z0012_LB_B01_1_D06_A	185	3	LMG 11194	*Moraxella*	*canis*
Z0012_LB_A12_2_D01_B	185	3	LMG 11194	*Moraxella*	*canis*
Z0012_LB_A11_2_D01_A	185	3	LMG 11194	*Moraxella*	*canis*
Z0012_RB_A06_2_D10_B	185	3	LMG 11194	*Moraxella*	*canis*
Z0012_LB_B02_1_D06_B	185	3	LMG 11194	*Moraxella*	*canis*
Z0012_LB_B03_1_D05_A	185	3	LMG 11194	*Moraxella*	*canis*
Z0012_LB_A07_2_D03_A	185	3	LMG 11194	*Moraxella*	*canis*
Z0012_RB_A07_2_D09_A	185	3	LMG 11194	*Moraxella*	*canis*
Z0012_LB_A10_2_D02_B	185	3	LMG 11194	*Moraxella*	*canis*
Z0012_RB_A08_2_D09_B	185	3	LMG 11194	*Moraxella*	*canis*

Z0012_RB_B03_1_D11_A	185	3	LMG 11194	*Moraxella*	*canis*
Z0012_LB_B04_1_D05_B	185	3	LMG 11194	*Moraxella*	*canis*
Z0012_RB_A10_2_D08_B	186	3	LMG 11194	*Moraxella*	*canis*
Z0012_RB_A05_2_D10_A	186	3	LMG 11194	*Moraxella*	*canis*
Z0012_LB_A03_2_D05_A	186	3	LMG 11194	*Moraxella*	*canis*
Z0012_LB_A06_2_D04_B	186	3	LMG 11194	*Moraxella*	*canis*
Z0012_LB_A02_2_D06_B	186	3	LMG 11194	*Moraxella*	*canis*
Z0013_RB_E03_2_B11_A	187	21	LMG 16409	*Pandoraea*	*apista*
Z0013_LB_E04_2_B05_B	187	21	LMG 16409	*Pandoraea*	*apista*
Z0013_LO_E05_1_G03_A	187	21	LMG 16409	*Pandoraea*	*apista*
Z0013_RO_E05_1_G09_A	187	21	LMG 16409	*Pandoraea*	*apista*
Z0013_RB_E06_2_B10_B	187	21	LMG 16409	*Pandoraea*	*apista*
Z0013_LB_E05_2_B04_A	187	21	LMG 16409	*Pandoraea*	*apista*
Z0013_LB_E07_2_B03_A	187	21	LMG 16409	*Pandoraea*	*apista*
Z0013_RO_E09_1_G11_A	187	21	LMG 16409	*Pandoraea*	*apista*
Z0013_LO_E08_1_G04_B	187	21	LMG 16409	*Pandoraea*	*apista*
Z0013_LO_E07_1_G04_A	187	21	LMG 16409	*Pandoraea*	*apista*
Z0013_RB_E07_2_B09_A	187	21	LMG 16409	*Pandoraea*	*apista*
Z0013_RO_E06_1_G09_B	187	21	LMG 16409	*Pandoraea*	*apista*
Z0013_LO_E09_1_G05_A	187	21	LMG 16409	*Pandoraea*	*apista*
Z0013_RO_E07_1_G10_A	187	21	LMG 16409	*Pandoraea*	*apista*
Z0013_RO_E08_1_G10_B	187	21	LMG 16409	*Pandoraea*	*apista*
Z0013_LB_E08_2_B03_B	187	21	LMG 16409	*Pandoraea*	*apista*
Z0013_RO_E11_1_G12_A	187	21	LMG 16409	*Pandoraea*	*apista*
Z0013_RB_E04_2_B11_B	187	21	LMG 16409	*Pandoraea*	*apista*
Z0013_RO_E10_1_G11_B	187	21	LMG 16409	*Pandoraea*	*apista*
Z0013_LO_E11_1_G06_A	187	21	LMG 16409	*Pandoraea*	*apista*
Z0013_LO_E06_1_G03_B	187	21	LMG 16409	*Pandoraea*	*apista*
Z0013_RB_E05_2_B10_A	187	21	LMG 16409	*Pandoraea*	*apista*
Z0013_LO_E10_1_G05_B	187	21	LMG 16409	*Pandoraea*	*apista*
Z0013_LO_E04_1_G02_B	187	21	LMG 16409	*Pandoraea*	*apista*
Z0013_LB_E03_2_B05_A	187	21	LMG 16409	*Pandoraea*	*apista*
Z0013_LB_E06_2_B04_B	187	21	LMG 16409	*Pandoraea*	*apista*
Z0013_LO_E03_1_G02_A	187	21	LMG 16409	*Pandoraea*	*apista*
Z0013_LO_E02_1_G01_B	187	21	LMG 16409	*Pandoraea*	*apista*
Z0017_RO_B09_2_E11_A	188	62	LMG 24012	*Parapusillimonas*	*granuli*
Z0017_RO_B11_2_E12_A	188	62	LMG 24012	*Parapusillimonas*	*granuli*
Z0017_RO_B12_2_E12_B	188	62	LMG 24012	*Parapusillimonas*	*granuli*
Z0017_LO_B03_2_E02_A	188	62	LMG 24012	*Parapusillimonas*	*granuli*
Z0017_RO_B07_2_E10_A	188	62	LMG 24012	*Parapusillimonas*	*granuli*
Z0017_LO_A12_1_E06_B	188	62	LMG 24012	*Parapusillimonas*	*granuli*
Z0017_RO_C05_1_F09_A	188	62	LMG 24012	*Parapusillimonas*	*granuli*
Z0017_RO_B08_2_E10_B	188	62	LMG 24012	*Parapusillimonas*	*granuli*
Z0017_LO_A11_1_E06_A	188	62	LMG 24012	*Parapusillimonas*	*granuli*
Z0017_LO_B11_2_E06_A	188	62	LMG 24012	*Parapusillimonas*	*granuli*
Z0017_LO_B12_2_E06_B	188	62	LMG 24012	*Parapusillimonas*	*granuli*
Z0017_LO_B09_2_E05_A	188	62	LMG 24012	*Parapusillimonas*	*granuli*
Z0017_RO_C02_1_F07_B	188	62	LMG 24012	*Parapusillimonas*	*granuli*
Z0017_RO_C01_1_F07_A	188	62	LMG 24012	*Parapusillimonas*	*granuli*
Z0017_LO_B08_2_E04_B	188	62	LMG 24012	*Parapusillimonas*	*granuli*

Z0017_LO_B07_2_E04_A	188	62	LMG 24012	*Parapusillimonas*	*granuli*
Z0017_RO_B02_2_E07_B	188	62	LMG 24012	*Parapusillimonas*	*granuli*
Z0017_LO_A08_1_E04_B	188	62	LMG 24012	*Parapusillimonas*	*granuli*
Z0017_LO_C03_1_F02_A	188	62	LMG 24012	*Parapusillimonas*	*granuli*
Z0017_LO_A10_1_E05_B	188	62	LMG 24012	*Parapusillimonas*	*granuli*
Z0017_RO_C10_1_F11_B	188	62	LMG 24012	*Parapusillimonas*	*granuli*
Z0017_LO_C02_1_F01_B	188	62	LMG 24012	*Parapusillimonas*	*granuli*
Z0017_RO_C07_1_F10_A	188	62	LMG 24012	*Parapusillimonas*	*granuli*
Z0017_LO_C05_1_F03_A	188	62	LMG 24012	*Parapusillimonas*	*granuli*
Z0017_LO_C06_1_F03_B	188	62	LMG 24012	*Parapusillimonas*	*granuli*
Z0017_RO_D01_2_F07_A	188	62	LMG 24012	*Parapusillimonas*	*granuli*
Z0017_RO_C12_1_F12_B	188	62	LMG 24012	*Parapusillimonas*	*granuli*
Z0017_LO_A09_1_E05_A	188	62	LMG 24012	*Parapusillimonas*	*granuli*
Z0023_RB_H03_1_A11_A	189	121	LMG 5286	*Acidovorax*	*cattleyae*
Z0023_LO_A09_1_E05_A	189	121	LMG 5286	*Acidovorax*	*cattleyae*
Z0023_RB_H04_1_A11_B	189	121	LMG 5286	*Acidovorax*	*cattleyae*
Z0023_RO_A02_1_E07_B	189	121	LMG 5286	*Acidovorax*	*cattleyae*
Z0023_RO_A09_1_E11_A	189	121	LMG 5286	*Acidovorax*	*cattleyae*
Z0023_LO_B07_2_E04_A	189	121	LMG 5286	*Acidovorax*	*cattleyae*
Z0023_RO_A08_1_E10_B	189	121	LMG 5286	*Acidovorax*	*cattleyae*
Z0023_RO_A05_1_E09_A	189	121	LMG 5286	*Acidovorax*	*cattleyae*
Z0023_LO_B06_2_E03_B	189	121	LMG 5286	*Acidovorax*	*cattleyae*
Z0023_LO_B05_2_E03_A	189	121	LMG 5286	*Acidovorax*	*cattleyae*
Z0023_RB_H07_1_A09_A	190	121	LMG 5286	*Acidovorax*	*cattleyae*
Z0023_LO_A10_1_E05_B	190	121	LMG 5286	*Acidovorax*	*cattleyae*
Z0023_RB_H08_1_A09_B	190	121	LMG 5286	*Acidovorax*	*cattleyae*
Z0023_LO_B04_2_E02_B	190	121	LMG 5286	*Acidovorax*	*cattleyae*
Z0023_RB_H09_1_A08_A	190	121	LMG 5286	*Acidovorax*	*cattleyae*
Z0023_LO_B09_2_E05_A	190	121	LMG 5286	*Acidovorax*	*cattleyae*
Z0023_RB_G12_2_A07_B	191	121	LMG 5286	*Acidovorax*	*cattleyae*
Z0023_LO_A07_1_E04_A	191	121	LMG 5286	*Acidovorax*	*cattleyae*
Z0023_RB_H01_1_A12_A	191	121	LMG 5286	*Acidovorax*	*cattleyae*
Z0023_RB_H10_1_A08_B	191	121	LMG 5286	*Acidovorax*	*cattleyae*
Z0023_RB_H12_1_A07_B	191	121	LMG 5286	*Acidovorax*	*cattleyae*
Z0023_LO_A11_1_E06_A	191	121	LMG 5286	*Acidovorax*	*cattleyae*
Z0023_LO_A12_1_E06_B	191	121	LMG 5286	*Acidovorax*	*cattleyae*
Z0023_LO_B02_2_E01_B	191	121	LMG 5286	*Acidovorax*	*cattleyae*
Z0023_LO_B01_2_E01_A	191	121	LMG 5286	*Acidovorax*	*cattleyae*
Z0023_LO_A08_1_E04_B	191	121	LMG 5286	*Acidovorax*	*cattleyae*
Z0023_RO_A03_1_E08_A	191	121	LMG 5286	*Acidovorax*	*cattleyae*
Z0023_RB_H02_1_A12_B	191	121	LMG 5286	*Acidovorax*	*cattleyae*
Z0012_LB_E05_2_B04_A	192	5	LMG 1226	*Delftia*	*acidovorans*
Z0012_LB_E04_2_B05_B	192	5	LMG 1226	*Delftia*	*acidovorans*
Z0012_LB_C12_2_C01_B	192	5	LMG 1226	*Delftia*	*acidovorans*
Z0012_RB_D05_1_C10_A	192	5	LMG 1226	*Delftia*	*acidovorans*
Z0012_RB_D04_1_C11_B	192	5	LMG 1226	*Delftia*	*acidovorans*
Z0012_RB_D03_1_C11_A	192	5	LMG 1226	*Delftia*	*acidovorans*
Z0012_RB_C06_2_C10_B	192	5	LMG 1226	*Delftia*	*acidovorans*
Z0012_LB_D11_1_C01_A	192	5	LMG 1226	*Delftia*	*acidovorans*
Z0012_RB_D01_1_C12_A	192	5	LMG 1226	*Delftia*	*acidovorans*

Z0012_LB_D08_1_C03_B	192	5	LMG 1226	*Delftia*	*acidovorans*
Z0012_RB_D06_1_C10_B	192	5	LMG 1226	*Delftia*	*acidovorans*
Z0012_RB_C10_2_C08_B	192	5	LMG 1226	*Delftia*	*acidovorans*
Z0012_LB_D09_1_C02_A	192	5	LMG 1226	*Delftia*	*acidovorans*
Z0012_LB_D01_1_C06_A	192	5	LMG 1226	*Delftia*	*acidovorans*
Z0012_RB_D02_1_C12_B	192	5	LMG 1226	*Delftia*	*acidovorans*
Z0012_RB_C07_2_C09_A	192	5	LMG 1226	*Delftia*	*acidovorans*
Z0012_LB_D02_1_C06_B	192	5	LMG 1226	*Delftia*	*acidovorans*
Z0012_LB_D03_1_C05_A	192	5	LMG 1226	*Delftia*	*acidovorans*
Z0012_LB_C11_2_C01_A	192	5	LMG 1226	*Delftia*	*acidovorans*
Z0012_RB_C09_2_C08_A	192	5	LMG 1226	*Delftia*	*acidovorans*
Z0012_RB_C12_2_C07_B	192	5	LMG 1226	*Delftia*	*acidovorans*
Z0012_LB_D12_1_C01_B	192	5	LMG 1226	*Delftia*	*acidovorans*
Z0012_LB_D04_1_C05_B	192	5	LMG 1226	*Delftia*	*acidovorans*
Z0012_LB_E01_2_B06_A	192	5	LMG 1226	*Delftia*	*acidovorans*
Z0012_RB_C08_2_C09_B	193	5	LMG 1226	*Delftia*	*acidovorans*
Z0012_LB_E03_2_B05_A	193	5	LMG 1226	*Delftia*	*acidovorans*
Z0012_LB_E02_2_B06_B	193	5	LMG 1226	*Delftia*	*acidovorans*
Z0012_LB_D05_1_C04_A	193	5	LMG 1226	*Delftia*	*acidovorans*
Z0012_LB_D06_1_C04_B	193	5	LMG 1226	*Delftia*	*acidovorans*
Z0012_LB_D07_1_C03_A	193	5	LMG 1226	*Delftia*	*acidovorans*
Z0012_RB_C11_2_C07_A	193	5	LMG 1226	*Delftia*	*acidovorans*
Z0012_LB_D10_1_C02_B	194	5	LMG 1226	*Delftia*	*acidovorans*
Z0023_RB_F09_1_B08_A	195	119	LMG 460	*Zymomonas*	*mobilis*
Z0023_LB_G11_2_A01_A	195	119	LMG 460	*Zymomonas*	*mobilis*
Z0023_LB_G09_2_A02_A	195	119	LMG 460	*Zymomonas*	*mobilis*
Z0023_LB_G10_2_A02_B	195	119	LMG 460	*Zymomonas*	*mobilis*
Z0023_RB_E11_2_B07_A	195	119	LMG 460	*Zymomonas*	*mobilis*
Z0023_LB_F07_1_B03_A	195	119	LMG 460	*Zymomonas*	*mobilis*
Z0023_LB_F11_1_B01_A	195	119	LMG 460	*Zymomonas*	*mobilis*
Z0023_LB_F12_1_B01_B	195	119	LMG 460	*Zymomonas*	*mobilis*
Z0023_LB_F10_1_B02_B	195	119	LMG 460	*Zymomonas*	*mobilis*
Z0023_LB_G12_2_A01_B	195	119	LMG 460	*Zymomonas*	*mobilis*
Z0023_RB_F07_1_B09_A	195	119	LMG 460	*Zymomonas*	*mobilis*
Z0023_LB_F09_1_B02_A	195	119	LMG 460	*Zymomonas*	*mobilis*
Z0023_RB_F08_1_B09_B	195	119	LMG 460	*Zymomonas*	*mobilis*
Z0023_RB_E10_2_B08_B	195	119	LMG 460	*Zymomonas*	*mobilis*
Z0023_LB_F06_1_B04_B	195	119	LMG 460	*Zymomonas*	*mobilis*
Z0023_RB_E09_2_B08_A	195	119	LMG 460	*Zymomonas*	*mobilis*
Z0023_RB_F01_1_B12_A	195	119	LMG 460	*Zymomonas*	*mobilis*
Z0023_RB_E12_2_B07_B	195	119	LMG 460	*Zymomonas*	*mobilis*
Z0023_LB_F08_1_B03_B	195	119	LMG 460	*Zymomonas*	*mobilis*
Z0023_LB_G01_2_A06_A	195	119	LMG 460	*Zymomonas*	*mobilis*
Z0023_RB_F03_1_B11_A	195	119	LMG 460	*Zymomonas*	*mobilis*
Z0023_LB_G04_2_A05_B	195	119	LMG 460	*Zymomonas*	*mobilis*
Z0023_LB_G06_2_A04_B	195	119	LMG 460	*Zymomonas*	*mobilis*
Z0023_LB_G05_2_A04_A	195	119	LMG 460	*Zymomonas*	*mobilis*
Z0023_RB_F04_1_B11_B	195	119	LMG 460	*Zymomonas*	*mobilis*
Z0023_LB_G07_2_A03_A	195	119	LMG 460	*Zymomonas*	*mobilis*
Z0023_LB_G08_2_A03_B	195	119	LMG 460	*Zymomonas*	*mobilis*

Z0023_RB_F06_1_B10_B	195	119	LMG 460	*Zymomonas*	*mobilis*
Z0023_RB_F05_1_B10_A	195	119	LMG 460	*Zymomonas*	*mobilis*
Z0023_RB_F02_1_B12_B	196	119	LMG 460	*Zymomonas*	*mobilis*
Z0023_LB_G02_2_A06_B	196	119	LMG 460	*Zymomonas*	*mobilis*
Z0023_LB_G03_2_A05_A	196	119	LMG 460	*Zymomonas*	*mobilis*
Z0019_RB_B07_1_D09_A	197	79	LMG 25435	*Marinobacterium*	*coralli*
Z0019_RB_B08_1_D09_B	197	79	LMG 25435	*Marinobacterium*	*coralli*
Z0019_RB_B06_1_D10_B	197	79	LMG 25435	*Marinobacterium*	*coralli*
Z0019_RB_B09_1_D08_A	197	79	LMG 25435	*Marinobacterium*	*coralli*
Z0019_LB_D02_1_C06_B	197	79	LMG 25435	*Marinobacterium*	*coralli*
Z0019_LB_D03_1_C05_A	197	79	LMG 25435	*Marinobacterium*	*coralli*
Z0019_RB_B12_1_D07_B	197	79	LMG 25435	*Marinobacterium*	*coralli*
Z0019_LB_D04_1_C05_B	197	79	LMG 25435	*Marinobacterium*	*coralli*
Z0019_RB_C08_2_C09_B	197	79	LMG 25435	*Marinobacterium*	*coralli*
Z0019_RB_C01_2_C12_A	197	79	LMG 25435	*Marinobacterium*	*coralli*
Z0019_RB_C02_2_C12_B	197	79	LMG 25435	*Marinobacterium*	*coralli*
Z0019_RB_C05_2_C10_A	197	79	LMG 25435	*Marinobacterium*	*coralli*
Z0019_LB_C11_2_C01_A	197	79	LMG 25435	*Marinobacterium*	*coralli*
Z0019_LB_C12_2_C01_B	197	79	LMG 25435	*Marinobacterium*	*coralli*
Z0019_LB_C10_2_C02_B	197	79	LMG 25435	*Marinobacterium*	*coralli*
Z0019_RB_C06_2_C10_B	197	79	LMG 25435	*Marinobacterium*	*coralli*
Z0019_LB_D01_1_C06_A	197	79	LMG 25435	*Marinobacterium*	*coralli*
Z0019_RB_C10_2_C08_B	198	79	LMG 25435	*Marinobacterium*	*coralli*
Z0019_RB_C11_2_C07_A	198	79	LMG 25435	*Marinobacterium*	*coralli*
Z0019_LB_D06_1_C04_B	198	79	LMG 25435	*Marinobacterium*	*coralli*
Z0019_RB_C09_2_C08_A	198	79	LMG 25435	*Marinobacterium*	*coralli*
Z0019_RB_B11_1_D07_A	198	79	LMG 25435	*Marinobacterium*	*coralli*
Z0019_LB_C05_2_C04_A	198	79	LMG 25435	*Marinobacterium*	*coralli*
Z0019_LB_D05_1_C04_A	198	79	LMG 25435	*Marinobacterium*	*coralli*
Z0019_RB_B10_1_D08_B	198	79	LMG 25435	*Marinobacterium*	*coralli*
Z0019_RB_C07_2_C09_A	198	79	LMG 25435	*Marinobacterium*	*coralli*
Z0019_LB_C06_2_C04_B	198	79	LMG 25435	*Marinobacterium*	*coralli*
Z0019_RB_C03_2_C11_A	198	79	LMG 25435	*Marinobacterium*	*coralli*
Z0019_LB_C07_2_C03_A	198	79	LMG 25435	*Marinobacterium*	*coralli*
Z0019_LB_C09_2_C02_A	198	79	LMG 25435	*Marinobacterium*	*coralli*
Z0019_RB_C04_2_C11_B	198	79	LMG 25435	*Marinobacterium*	*coralli*
Z0019_LB_C08_2_C03_B	198	79	LMG 25435	*Marinobacterium*	*coralli*
Z0012_LB_F06_1_B04_B	199	6	LMG 1229	*Alcaligenes*	*faecalis*
Z0012_LB_F05_1_B04_A	199	6	LMG 1229	*Alcaligenes*	*faecalis*
Z0012_RB_E04_2_B11_B	199	6	LMG 1229	*Alcaligenes*	*faecalis*
Z0012_LB_E10_2_B02_B	199	6	LMG 1229	*Alcaligenes*	*faecalis*
Z0012_RB_E05_2_B10_A	199	6	LMG 1229	*Alcaligenes*	*faecalis*
Z0012_LB_E09_2_B02_A	199	6	LMG 1229	*Alcaligenes*	*faecalis*
Z0012_RB_D09_1_C08_A	199	6	LMG 1229	*Alcaligenes*	*faecalis*
Z0012_LB_E08_2_B03_B	199	6	LMG 1229	*Alcaligenes*	*faecalis*
Z0012_RB_D08_1_C09_B	199	6	LMG 1229	*Alcaligenes*	*faecalis*
Z0012_RB_D11_1_C07_A	199	6	LMG 1229	*Alcaligenes*	*faecalis*
Z0012_RB_E02_2_B12_B	199	6	LMG 1229	*Alcaligenes*	*faecalis*
Z0012_LB_F04_1_B05_B	199	6	LMG 1229	*Alcaligenes*	*faecalis*
Z0012_RB_E03_2_B11_A	199	6	LMG 1229	*Alcaligenes*	*faecalis*

Z0012_LB_F03_1_B05_A	199	6	LMG 1229	*Alcaligenes*	*faecalis*
Z0012_LB_E07_2_B03_A	199	6	LMG 1229	*Alcaligenes*	*faecalis*
Z0012_RB_D07_1_C09_A	199	6	LMG 1229	*Alcaligenes*	*faecalis*
Z0012_LB_E06_2_B04_B	199	6	LMG 1229	*Alcaligenes*	*faecalis*
Z0012_RB_E07_2_B09_A	200	6	LMG 1229	*Alcaligenes*	*faecalis*
Z0012_LB_F07_1_B03_A	200	6	LMG 1229	*Alcaligenes*	*faecalis*
Z0012_LB_E12_2_B01_B	200	6	LMG 1229	*Alcaligenes*	*faecalis*
Z0012_RB_E01_2_B12_A	200	6	LMG 1229	*Alcaligenes*	*faecalis*
Z0012_LB_F02_1_B06_B	200	6	LMG 1229	*Alcaligenes*	*faecalis*
Z0012_RB_E06_2_B10_B	200	6	LMG 1229	*Alcaligenes*	*faecalis*
Z0012_RB_E08_2_B09_B	200	6	LMG 1229	*Alcaligenes*	*faecalis*
Z0012_RB_D12_1_C07_B	200	6	LMG 1229	*Alcaligenes*	*faecalis*
Z0012_LB_E11_2_B01_A	200	6	LMG 1229	*Alcaligenes*	*faecalis*
Z0012_LB_F01_1_B06_A	200	6	LMG 1229	*Alcaligenes*	*faecalis*
Z0012_LB_F09_1_B02_A	200	6	LMG 1229	*Alcaligenes*	*faecalis*
Z0012_RB_E10_2_B08_B	200	6	LMG 1229	*Alcaligenes*	*faecalis*
Z0012_RB_E09_2_B08_A	200	6	LMG 1229	*Alcaligenes*	*faecalis*
Z0012_LB_F08_1_B03_B	200	6	LMG 1229	*Alcaligenes*	*faecalis*
Z0012_RB_D10_1_C08_B	201	6	LMG 1229	*Alcaligenes*	*faecalis*
Z0018_RO_F10_2_G11_B	202	75	LMG 24812	*Candidimonas*	*nitroreducens*
Z0018_RO_F09_2_G11_A	202	75	LMG 24812	*Candidimonas*	*nitroreducens*
Z0018_RO_F12_2_G12_B	202	75	LMG 24812	*Candidimonas*	*nitroreducens*
Z0018_RO_F11_2_G12_A	202	75	LMG 24812	*Candidimonas*	*nitroreducens*
Z0018_RO_G11_1_H12_A	202	75	LMG 24812	*Candidimonas*	*nitroreducens*
Z0018_LO_H03_2_H02_A	202	75	LMG 24812	*Candidimonas*	*nitroreducens*
Z0018_LO_H05_2_H03_A	202	75	LMG 24812	*Candidimonas*	*nitroreducens*
Z0018_LO_H04_2_H02_B	202	75	LMG 24812	*Candidimonas*	*nitroreducens*
Z0018_RO_G04_1_H08_B	202	75	LMG 24812	*Candidimonas*	*nitroreducens*
Z0018_RO_G03_1_H08_A	202	75	LMG 24812	*Candidimonas*	*nitroreducens*
Z0018_RO_G01_1_H07_A	202	75	LMG 24812	*Candidimonas*	*nitroreducens*
Z0018_LO_G04_1_H02_B	202	75	LMG 24812	*Candidimonas*	*nitroreducens*
Z0018_RO_G09_1_H11_A	202	75	LMG 24812	*Candidimonas*	*nitroreducens*
Z0018_LO_H06_2_H03_B	202	75	LMG 24812	*Candidimonas*	*nitroreducens*
Z0018_RO_G05_1_H09_A	202	75	LMG 24812	*Candidimonas*	*nitroreducens*
Z0018_RO_G12_1_H12_B	202	75	LMG 24812	*Candidimonas*	*nitroreducens*
Z0018_RO_H01_2_H07_A	203	75	LMG 24812	*Candidimonas*	*nitroreducens*
Z0018_RO_H03_2_H08_A	203	75	LMG 24812	*Candidimonas*	*nitroreducens*
Z0018_RO_H02_2_H07_B	203	75	LMG 24812	*Candidimonas*	*nitroreducens*
Z0018_RO_H04_2_H08_B	203	75	LMG 24812	*Candidimonas*	*nitroreducens*
Z0018_RO_G10_1_H11_B	204	75	LMG 24812	*Candidimonas*	*nitroreducens*
Z0018_LO_H02_2_H01_B	204	75	LMG 24812	*Candidimonas*	*nitroreducens*
Z0018_LO_H01_2_H01_A	204	75	LMG 24812	*Candidimonas*	*nitroreducens*
Z0018_RO_G08_1_H10_B	204	75	LMG 24812	*Candidimonas*	*nitroreducens*
Z0018_RO_G07_1_H10_A	204	75	LMG 24812	*Candidimonas*	*nitroreducens*
Z0018_LO_G07_1_H04_A	204	75	LMG 24812	*Candidimonas*	*nitroreducens*
Z0018_LO_G08_1_H04_B	204	75	LMG 24812	*Candidimonas*	*nitroreducens*
Z0018_LO_G11_1_H06_A	204	75	LMG 24812	*Candidimonas*	*nitroreducens*
Z0018_RO_G06_1_H09_B	204	75	LMG 24812	*Candidimonas*	*nitroreducens*
Z0018_LO_G09_1_H05_A	204	75	LMG 24812	*Candidimonas*	*nitroreducens*
Z0018_RO_G02_1_H07_B	204	75	LMG 24812	*Candidimonas*	*nitroreducens*

Z0018_LO_G12_1_H06_B	204	75	LMG 24812	*Candidimonas*	*nitroreducens*
Z0012_RB_H06_1_A10_B	205	8	LMG 1242	*Pseudomonas*	*aeruginosa*
Z0012_RB_H07_1_A09_A	205	8	LMG 1242	*Pseudomonas*	*aeruginosa*
Z0012_RB_H09_1_A08_A	205	8	LMG 1242	*Pseudomonas*	*aeruginosa*
Z0012_RB_H11_1_A07_A	205	8	LMG 1242	*Pseudomonas*	*aeruginosa*
Z0012_LO_C05_1_F03_A	205	8	LMG 1242	*Pseudomonas*	*aeruginosa*
Z0012_RO_B01_2_E07_A	205	8	LMG 1242	*Pseudomonas*	*aeruginosa*
Z0012_LO_C06_1_F03_B	205	8	LMG 1242	*Pseudomonas*	*aeruginosa*
Z0012_RO_A06_1_E09_B	205	8	LMG 1242	*Pseudomonas*	*aeruginosa*
Z0012_LO_C03_1_F02_A	205	8	LMG 1242	*Pseudomonas*	*aeruginosa*
Z0012_RO_A09_1_E11_A	205	8	LMG 1242	*Pseudomonas*	*aeruginosa*
Z0012_LO_A05_1_E03_A	205	8	LMG 1242	*Pseudomonas*	*aeruginosa*
Z0012_LO_A09_1_E05_A	205	8	LMG 1242	*Pseudomonas*	*aeruginosa*
Z0012_RO_A11_1_E12_A	205	8	LMG 1242	*Pseudomonas*	*aeruginosa*
Z0012_RO_A08_1_E10_B	205	8	LMG 1242	*Pseudomonas*	*aeruginosa*
Z0012_RO_A10_1_E11_B	205	8	LMG 1242	*Pseudomonas*	*aeruginosa*
Z0012_LO_A07_1_E04_A	205	8	LMG 1242	*Pseudomonas*	*aeruginosa*
Z0012_LO_A11_1_E06_A	205	8	LMG 1242	*Pseudomonas*	*aeruginosa*
Z0012_RO_B10_2_E11_B	205	8	LMG 1242	*Pseudomonas*	*aeruginosa*
Z0012_RO_B07_2_E10_A	205	8	LMG 1242	*Pseudomonas*	*aeruginosa*
Z0012_LO_B08_2_E04_B	205	8	LMG 1242	*Pseudomonas*	*aeruginosa*
Z0012_LO_C09_1_F05_A	205	8	LMG 1242	*Pseudomonas*	*aeruginosa*
Z0012_LO_C11_1_F06_A	205	8	LMG 1242	*Pseudomonas*	*aeruginosa*
Z0012_LO_C08_1_F04_B	205	8	LMG 1242	*Pseudomonas*	*aeruginosa*
Z0012_RO_B05_2_E09_A	205	8	LMG 1242	*Pseudomonas*	*aeruginosa*
Z0012_RO_A03_1_E08_A	205	8	LMG 1242	*Pseudomonas*	*aeruginosa*
Z0012_LO_B01_2_E01_A	205	8	LMG 1242	*Pseudomonas*	*aeruginosa*
Z0012_LO_B02_2_E01_B	205	8	LMG 1242	*Pseudomonas*	*aeruginosa*
Z0012_LO_B03_2_E02_A	205	8	LMG 1242	*Pseudomonas*	*aeruginosa*
Z0012_LO_B06_2_E03_B	205	8	LMG 1242	*Pseudomonas*	*aeruginosa*
Z0012_RO_B09_2_E11_A	205	8	LMG 1242	*Pseudomonas*	*aeruginosa*
Z0012_RO_A04_1_E08_B	205	8	LMG 1242	*Pseudomonas*	*aeruginosa*
Z0012_LO_D02_2_F01_B	205	8	LMG 1242	*Pseudomonas*	*aeruginosa*
Z0020_RO_G12_1_H12_B	206	94	LMG 26852	*Achromobacter*	*aegrifaciens*
Z0020_LO_G04_1_H02_B	206	94	LMG 26852	*Achromobacter*	*aegrifaciens*
Z0020_RO_G10_1_H11_B	206	94	LMG 26852	*Achromobacter*	*aegrifaciens*
Z0020_LO_G06_1_H03_B	206	94	LMG 26852	*Achromobacter*	*aegrifaciens*
Z0020_LO_G08_1_H04_B	206	94	LMG 26852	*Achromobacter*	*aegrifaciens*
Z0020_LO_G05_1_H03_A	206	94	LMG 26852	*Achromobacter*	*aegrifaciens*
Z0020_LO_G07_1_H04_A	206	94	LMG 26852	*Achromobacter*	*aegrifaciens*
Z0020_RO_G01_1_H07_A	206	94	LMG 26852	*Achromobacter*	*aegrifaciens*
Z0020_RO_G02_1_H07_B	206	94	LMG 26852	*Achromobacter*	*aegrifaciens*
Z0020_RO_G07_1_H10_A	206	94	LMG 26852	*Achromobacter*	*aegrifaciens*
Z0020_LO_G03_1_H02_A	206	94	LMG 26852	*Achromobacter*	*aegrifaciens*
Z0020_RO_G08_1_H10_B	206	94	LMG 26852	*Achromobacter*	*aegrifaciens*
Z0020_RO_G06_1_H09_B	206	94	LMG 26852	*Achromobacter*	*aegrifaciens*
Z0020_LO_G09_1_H05_A	206	94	LMG 26852	*Achromobacter*	*aegrifaciens*
Z0020_LO_F02_2_G01_B	206	94	LMG 26852	*Achromobacter*	*aegrifaciens*
Z0020_RO_H04_2_H08_B	206	94	LMG 26852	*Achromobacter*	*aegrifaciens*
Z0020_RO_G09_1_H11_A	206	94	LMG 26852	*Achromobacter*	*aegrifaciens*

Z0020_LO_F10_2_G05_B	206	94	LMG 26852	*Achromobacter*	*aegrifaciens*
Z0020_RO_F12_2_G12_B	206	94	LMG 26852	*Achromobacter*	*aegrifaciens*
Z0020_RO_F10_2_G11_B	206	94	LMG 26852	*Achromobacter*	*aegrifaciens*
Z0020_LO_F06_2_G03_B	206	94	LMG 26852	*Achromobacter*	*aegrifaciens*
Z0020_RO_H01_2_H07_A	206	94	LMG 26852	*Achromobacter*	*aegrifaciens*
Z0020_RO_H03_2_H08_A	206	94	LMG 26852	*Achromobacter*	*aegrifaciens*
Z0020_LO_G02_1_H01_B	206	94	LMG 26852	*Achromobacter*	*aegrifaciens*
Z0020_RO_G05_1_H09_A	206	94	LMG 26852	*Achromobacter*	*aegrifaciens*
Z0020_LO_F12_2_G06_B	206	94	LMG 26852	*Achromobacter*	*aegrifaciens*
Z0020_RO_G11_1_H12_A	207	94	LMG 26852	*Achromobacter*	*aegrifaciens*
Z0020_RO_H02_2_H07_B	207	94	LMG 26852	*Achromobacter*	*aegrifaciens*
Z0020_RO_F11_2_G12_A	207	94	LMG 26852	*Achromobacter*	*aegrifaciens*
Z0020_RO_G03_1_H08_A	207	94	LMG 26852	*Achromobacter*	*aegrifaciens*
Z0020_LO_F07_2_G04_A	207	94	LMG 26852	*Achromobacter*	*aegrifaciens*
Z0020_RO_G04_1_H08_B	207	94	LMG 26852	*Achromobacter*	*aegrifaciens*
Z0012_RB_G03_2_A11_A	208	7	LMG 1232	*Bordetella*	*bronchiseptica*
Z0012_LB_H02_1_A06_B	208	7	LMG 1232	*Bordetella*	*bronchiseptica*
Z0012_RB_F09_1_B08_A	208	7	LMG 1232	*Bordetella*	*bronchiseptica*
Z0012_LB_G09_2_A02_A	208	7	LMG 1232	*Bordetella*	*bronchiseptica*
Z0012_LB_H01_1_A06_A	208	7	LMG 1232	*Bordetella*	*bronchiseptica*
Z0012_LB_G07_2_A03_A	208	7	LMG 1232	*Bordetella*	*bronchiseptica*
Z0012_RB_E11_2_B07_A	208	7	LMG 1232	*Bordetella*	*bronchiseptica*
Z0012_LB_F10_1_B02_B	208	7	LMG 1232	*Bordetella*	*bronchiseptica*
Z0012_RB_F02_1_B12_B	208	7	LMG 1232	*Bordetella*	*bronchiseptica*
Z0012_LB_H04_1_A05_B	208	7	LMG 1232	*Bordetella*	*bronchiseptica*
Z0012_RB_H01_1_A12_A	208	7	LMG 1232	*Bordetella*	*bronchiseptica*
Z0012_RB_F06_1_B10_B	208	7	LMG 1232	*Bordetella*	*bronchiseptica*
Z0012_RB_G06_2_A10_B	208	7	LMG 1232	*Bordetella*	*bronchiseptica*
Z0012_LB_H09_1_A02_A	208	7	LMG 1232	*Bordetella*	*bronchiseptica*
Z0012_RB_F04_1_B11_B	208	7	LMG 1232	*Bordetella*	*bronchiseptica*
Z0012_RB_G11_2_A07_A	208	7	LMG 1232	*Bordetella*	*bronchiseptica*
Z0012_RB_H03_1_A11_A	208	7	LMG 1232	*Bordetella*	*bronchiseptica*
Z0012_RB_F01_1_B12_A	208	7	LMG 1232	*Bordetella*	*bronchiseptica*
Z0012_RB_E12_2_B07_B	208	7	LMG 1232	*Bordetella*	*bronchiseptica*
Z0012_LB_G01_2_A06_A	208	7	LMG 1232	*Bordetella*	*bronchiseptica*
Z0012_LB_F12_1_B01_B	208	7	LMG 1232	*Bordetella*	*bronchiseptica*
Z0012_RB_F03_1_B11_A	208	7	LMG 1232	*Bordetella*	*bronchiseptica*
Z0012_LB_H07_1_A03_A	208	7	LMG 1232	*Bordetella*	*bronchiseptica*
Z0012_LB_G11_2_A01_A	208	7	LMG 1232	*Bordetella*	*bronchiseptica*
Z0012_LB_G12_2_A01_B	208	7	LMG 1232	*Bordetella*	*bronchiseptica*
Z0012_LO_A01_1_E01_A	208	7	LMG 1232	*Bordetella*	*bronchiseptica*
Z0012_LB_H06_1_A04_B	208	7	LMG 1232	*Bordetella*	*bronchiseptica*
Z0012_LB_G06_2_A04_B	208	7	LMG 1232	*Bordetella*	*bronchiseptica*
Z0012_LB_H11_1_A01_A	208	7	LMG 1232	*Bordetella*	*bronchiseptica*
Z0012_RB_H04_1_A11_B	208	7	LMG 1232	*Bordetella*	*bronchiseptica*
Z0012_RB_G01_2_A12_A	208	7	LMG 1232	*Bordetella*	*bronchiseptica*
Z0012_RB_G10_2_A08_B	208	7	LMG 1232	*Bordetella*	*bronchiseptica*
Z0024_LB_D12_1_C01_B	209	125	LMG 6866	*Ralstonia*	*mannitolilytica*
Z0024_LB_D08_1_C03_B	209	125	LMG 6866	*Ralstonia*	*mannitolilytica*
Z0024_LB_E02_2_B06_B	209	125	LMG 6866	*Ralstonia*	*mannitolilytica*

Z0024_RB_D08_1_C09_B	209	125	LMG 6866	*Ralstonia*	*mannitolilytica*
Z0024_RB_D11_1_C07_A	209	125	LMG 6866	*Ralstonia*	*mannitolilytica*
Z0024_LB_D03_1_C05_A	209	125	LMG 6866	*Ralstonia*	*mannitolilytica*
Z0024_LB_C12_2_C01_B	209	125	LMG 6866	*Ralstonia*	*mannitolilytica*
Z0024_RB_E02_2_B12_B	209	125	LMG 6866	*Ralstonia*	*mannitolilytica*
Z0024_LB_D06_1_C04_B	209	125	LMG 6866	*Ralstonia*	*mannitolilytica*
Z0024_LB_D10_1_C02_B	209	125	LMG 6866	*Ralstonia*	*mannitolilytica*
Z0024_LB_D01_1_C06_A	209	125	LMG 6866	*Ralstonia*	*mannitolilytica*
Z0024_RB_E03_2_B11_A	209	125	LMG 6866	*Ralstonia*	*mannitolilytica*
Z0024_LB_D04_1_C05_B	209	125	LMG 6866	*Ralstonia*	*mannitolilytica*
Z0024_RB_D06_1_C10_B	209	125	LMG 6866	*Ralstonia*	*mannitolilytica*
Z0024_RB_E06_2_B10_B	209	125	LMG 6866	*Ralstonia*	*mannitolilytica*
Z0024_RB_D03_1_C11_A	209	125	LMG 6866	*Ralstonia*	*mannitolilytica*
Z0024_RB_E01_2_B12_A	209	125	LMG 6866	*Ralstonia*	*mannitolilytica*
Z0024_LB_D09_1_C02_A	209	125	LMG 6866	*Ralstonia*	*mannitolilytica*
Z0024_RB_D12_1_C07_B	209	125	LMG 6866	*Ralstonia*	*mannitolilytica*
Z0024_RB_E05_2_B10_A	209	125	LMG 6866	*Ralstonia*	*mannitolilytica*
Z0024_LB_D05_1_C04_A	209	125	LMG 6866	*Ralstonia*	*mannitolilytica*
Z0024_LB_C10_2_C02_B	209	125	LMG 6866	*Ralstonia*	*mannitolilytica*
Z0024_RB_D04_1_C11_B	209	125	LMG 6866	*Ralstonia*	*mannitolilytica*
Z0024_LB_E01_2_B06_A	209	125	LMG 6866	*Ralstonia*	*mannitolilytica*
Z0024_RB_D05_1_C10_A	209	125	LMG 6866	*Ralstonia*	*mannitolilytica*
Z0024_RB_E04_2_B11_B	210	125	LMG 6866	*Ralstonia*	*mannitolilytica*
Z0024_LB_C11_2_C01_A	210	125	LMG 6866	*Ralstonia*	*mannitolilytica*
Z0024_LB_D02_1_C06_B	210	125	LMG 6866	*Ralstonia*	*mannitolilytica*
Z0024_RB_D10_1_C08_B	210	125	LMG 6866	*Ralstonia*	*mannitolilytica*
Z0024_LB_D11_1_C01_A	210	125	LMG 6866	*Ralstonia*	*mannitolilytica*
Z0024_RB_D07_1_C09_A	210	125	LMG 6866	*Ralstonia*	*mannitolilytica*
Z0024_RB_D09_1_C08_A	210	125	LMG 6866	*Ralstonia*	*mannitolilytica*
Z0024_LO_G01_1_H01_A	211	130	LMG 7123	*Brevibacillus*	*brevis*
Z0024_LO_F12_2_G06_B	211	130	LMG 7123	*Brevibacillus*	*brevis*
Z0024_LO_F10_2_G05_B	211	130	LMG 7123	*Brevibacillus*	*brevis*
Z0024_RO_D08_2_F10_B	211	130	LMG 7123	*Brevibacillus*	*brevis*
Z0024_LO_F11_2_G06_A	211	130	LMG 7123	*Brevibacillus*	*brevis*
Z0024_RO_F01_2_G07_A	211	130	LMG 7123	*Brevibacillus*	*brevis*
Z0024_RO_F04_2_G08_B	211	130	LMG 7123	*Brevibacillus*	*brevis*
Z0024_LO_H01_2_H01_A	211	130	LMG 7123	*Brevibacillus*	*brevis*
Z0024_LO_F02_2_G01_B	211	130	LMG 7123	*Brevibacillus*	*brevis*
Z0024_LO_F07_2_G04_A	211	130	LMG 7123	*Brevibacillus*	*brevis*
Z0024_LO_F09_2_G05_A	211	130	LMG 7123	*Brevibacillus*	*brevis*
Z0024_RO_E12_1_G12_B	211	130	LMG 7123	*Brevibacillus*	*brevis*
Z0024_LO_G07_1_H04_A	211	130	LMG 7123	*Brevibacillus*	*brevis*
Z0024_LO_G08_1_H04_B	211	130	LMG 7123	*Brevibacillus*	*brevis*
Z0024_LO_H06_2_H03_B	211	130	LMG 7123	*Brevibacillus*	*brevis*
Z0024_LO_G06_1_H03_B	211	130	LMG 7123	*Brevibacillus*	*brevis*
Z0024_RO_F06_2_G09_B	211	130	LMG 7123	*Brevibacillus*	*brevis*
Z0024_RO_F07_2_G10_A	211	130	LMG 7123	*Brevibacillus*	*brevis*
Z0024_LO_H03_2_H02_A	211	130	LMG 7123	*Brevibacillus*	*brevis*
Z0024_RO_F02_2_G07_B	211	130	LMG 7123	*Brevibacillus*	*brevis*
Z0024_LO_G02_1_H01_B	212	130	LMG 7123	*Brevibacillus*	*brevis*

Z0024_LO_E11_1_G06_A	212	130	LMG 7123	*Brevibacillus*	*brevis*	
Z0024_LO_G03_1_H02_A	212	130	LMG 7123	*Brevibacillus*	*brevis*	
Z0024_LO_G05_1_H03_A	212	130	LMG 7123	*Brevibacillus*	*brevis*	
Z0024_LO_G04_1_H02_B	212	130	LMG 7123	*Brevibacillus*	*brevis*	
Z0024_LO_E10_1_G05_B	212	130	LMG 7123	*Brevibacillus*	*brevis*	
Z0024_LO_F03_2_G02_A	212	130	LMG 7123	*Brevibacillus*	*brevis*	
Z0024_RO_D07_2_F10_A	212	130	LMG 7123	*Brevibacillus*	*brevis*	
Z0024_LO_F08_2_G04_B	212	130	LMG 7123	*Brevibacillus*	*brevis*	
Z0024_LO_F04_2_G02_B	212	130	LMG 7123	*Brevibacillus*	*brevis*	
Z0024_LO_E12_1_G06_B	212	130	LMG 7123	*Brevibacillus*	*brevis*	
Z0024_LO_F01_2_G01_A	212	130	LMG 7123	*Brevibacillus*	*brevis*	
Z0022_RO_B02_2_E07_B	213	110	LMG 3516	*Paenalcaligenes*	*hominis*	
Z0022_LO_C08_1_F04_B	213	110	LMG 3516	*Paenalcaligenes*	*hominis*	
Z0022_LO_C10_1_F05_B	213	110	LMG 3516	*Paenalcaligenes*	*hominis*	
Z0022_LO_D01_2_F01_A	213	110	LMG 3516	*Paenalcaligenes*	*hominis*	
Z0022_LO_C11_1_F06_A	213	110	LMG 3516	*Paenalcaligenes*	*hominis*	
Z0022_LO_D02_2_F01_B	213	110	LMG 3516	*Paenalcaligenes*	*hominis*	
Z0022_LO_D04_2_F02_B	213	110	LMG 3516	*Paenalcaligenes*	*hominis*	
Z0022_LO_D03_2_F02_A	213	110	LMG 3516	*Paenalcaligenes*	*hominis*	
Z0022_LO_D05_2_F03_A	213	110	LMG 3516	*Paenalcaligenes*	*hominis*	
Z0022_LO_B11_2_E06_A	213	110	LMG 3516	*Paenalcaligenes*	*hominis*	
Z0022_LO_B07_2_E04_A	213	110	LMG 3516	*Paenalcaligenes*	*hominis*	
Z0022_RO_B05_2_E09_A	213	110	LMG 3516	*Paenalcaligenes*	*hominis*	
Z0022_LO_B12_2_E06_B	213	110	LMG 3516	*Paenalcaligenes*	*hominis*	
Z0022_LO_B03_2_E02_A	213	110	LMG 3516	*Paenalcaligenes*	*hominis*	
Z0022_LO_B05_2_E03_A	213	110	LMG 3516	*Paenalcaligenes*	*hominis*	
Z0022_LO_B04_2_E02_B	213	110	LMG 3516	*Paenalcaligenes*	*hominis*	
Z0022_RO_B08_2_E10_B	213	110	LMG 3516	*Paenalcaligenes*	*hominis*	
Z0022_RO_B07_2_E10_A	213	110	LMG 3516	*Paenalcaligenes*	*hominis*	
Z0022_LO_B06_2_E03_B	213	110	LMG 3516	*Paenalcaligenes*	*hominis*	
Z0022_LO_C04_1_F02_B	213	110	LMG 3516	*Paenalcaligenes*	*hominis*	
Z0022_LO_C05_1_F03_A	213	110	LMG 3516	*Paenalcaligenes*	*hominis*	
Z0022_LO_C07_1_F04_A	213	110	LMG 3516	*Paenalcaligenes*	*hominis*	
Z0022_LO_C09_1_F05_A	213	110	LMG 3516	*Paenalcaligenes*	*hominis*	
Z0022_LO_C06_1_F03_B	213	110	LMG 3516	*Paenalcaligenes*	*hominis*	
Z0022_RO_B06_2_E09_B	213	110	LMG 3516	*Paenalcaligenes*	*hominis*	
Z0022_LO_B09_2_E05_A	213	110	LMG 3516	*Paenalcaligenes*	*hominis*	
Z0022_RO_A12_1_E12_B	213	110	LMG 3516	*Paenalcaligenes*	*hominis*	
Z0022_LO_C12_1_F06_B	214	110	LMG 3516	*Paenalcaligenes*	*hominis*	
Z0024_RO_A01_1_E07_A	215	128	LMG 6909	*Leuconostoc*	*mesenteroides*	*cremoris*
Z0024_RO_A02_1_E07_B	215	128	LMG 6909	*Leuconostoc*	*mesenteroides*	*cremoris*
Z0024_RB_H12_1_A07_B	215	128	LMG 6909	*Leuconostoc*	*mesenteroides*	*cremoris*
Z0024_RO_A03_1_E08_A	216	128	LMG 6909	*Leuconostoc*	*mesenteroides*	*cremoris*
Z0024_RB_H10_1_A08_B	217	128	LMG 6909	*Leuconostoc*	*mesenteroides*	*cremoris*
Z0024_LO_B08_2_E04_B	217	128	LMG 6909	*Leuconostoc*	*mesenteroides*	*cremoris*
Z0024_RB_H09_1_A08_A	217	128	LMG 6909	*Leuconostoc*	*mesenteroides*	*cremoris*
Z0024_LO_B07_2_E04_A	217	128	LMG 6909	*Leuconostoc*	*mesenteroides*	*cremoris*
Z0024_LO_B09_2_E05_A	217	128	LMG 6909	*Leuconostoc*	*mesenteroides*	*cremoris*
Z0024_LO_B11_2_E06_A	217	128	LMG 6909	*Leuconostoc*	*mesenteroides*	*cremoris*
Z0024_RB_H11_1_A07_A	217	128	LMG 6909	*Leuconostoc*	*mesenteroides*	*cremoris*

Z0024_LO_B12_2_E06_B	217	128	LMG 6909	*Leuconostoc*	*mesenteroides*	*cremoris*
Z0024_LO_A12_1_E06_B	217	128	LMG 6909	*Leuconostoc*	*mesenteroides*	*cremoris*
Z0024_LO_B10_2_E05_B	217	128	LMG 6909	*Leuconostoc*	*mesenteroides*	*cremoris*
Z0024_RO_A08_1_E10_B	217	128	LMG 6909	*Leuconostoc*	*mesenteroides*	*cremoris*
Z0024_RO_A07_1_E10_A	217	128	LMG 6909	*Leuconostoc*	*mesenteroides*	*cremoris*
Z0024_RO_A06_1_E09_B	217	128	LMG 6909	*Leuconostoc*	*mesenteroides*	*cremoris*
Z0024_RO_A05_1_E09_A	217	128	LMG 6909	*Leuconostoc*	*mesenteroides*	*cremoris*
Z0024_LO_C04_1_F02_B	217	128	LMG 6909	*Leuconostoc*	*mesenteroides*	*cremoris*
Z0024_LO_C06_1_F03_B	217	128	LMG 6909	*Leuconostoc*	*mesenteroides*	*cremoris*
Z0024_LO_C05_1_F03_A	217	128	LMG 6909	*Leuconostoc*	*mesenteroides*	*cremoris*
Z0024_LO_B03_2_E02_A	217	128	LMG 6909	*Leuconostoc*	*mesenteroides*	*cremoris*
Z0024_LO_B06_2_E03_B	218	128	LMG 6909	*Leuconostoc*	*mesenteroides*	*cremoris*
Z0024_LO_B05_2_E03_A	218	128	LMG 6909	*Leuconostoc*	*mesenteroides*	*cremoris*
Z0024_RO_A09_1_E11_A	218	128	LMG 6909	*Leuconostoc*	*mesenteroides*	*cremoris*
Z0024_RO_A04_1_E08_B	218	128	LMG 6909	*Leuconostoc*	*mesenteroides*	*cremoris*
Z0024_RO_A12_1_E12_B	218	128	LMG 6909	*Leuconostoc*	*mesenteroides*	*cremoris*
Z0024_RO_A11_1_E12_A	218	128	LMG 6909	*Leuconostoc*	*mesenteroides*	*cremoris*
Z0023_RB_D01_1_C12_A	219	117	LMG 4305	*Rhodobacter*	*blasticus*	
Z0023_LB_D12_1_C01_B	219	117	LMG 4305	*Rhodobacter*	*blasticus*	
Z0023_RB_C10_2_C08_B	219	117	LMG 4305	*Rhodobacter*	*blasticus*	
Z0023_RB_C04_2_C11_B	219	117	LMG 4305	*Rhodobacter*	*blasticus*	
Z0023_LB_C11_2_C01_A	219	117	LMG 4305	*Rhodobacter*	*blasticus*	
Z0023_LB_D11_1_C01_A	219	117	LMG 4305	*Rhodobacter*	*blasticus*	
Z0023_LB_D01_1_C06_A	219	117	LMG 4305	*Rhodobacter*	*blasticus*	
Z0023_LB_C12_2_C01_B	219	117	LMG 4305	*Rhodobacter*	*blasticus*	
Z0023_LB_D03_1_C05_A	219	117	LMG 4305	*Rhodobacter*	*blasticus*	
Z0023_LB_E01_2_B06_A	219	117	LMG 4305	*Rhodobacter*	*blasticus*	
Z0023_LB_D04_1_C05_B	219	117	LMG 4305	*Rhodobacter*	*blasticus*	
Z0023_LB_D02_1_C06_B	219	117	LMG 4305	*Rhodobacter*	*blasticus*	
Z0023_RB_D02_1_C12_B	219	117	LMG 4305	*Rhodobacter*	*blasticus*	
Z0023_RB_D03_1_C11_A	219	117	LMG 4305	*Rhodobacter*	*blasticus*	
Z0023_LB_E02_2_B06_B	219	117	LMG 4305	*Rhodobacter*	*blasticus*	
Z0023_RB_C11_2_C07_A	219	117	LMG 4305	*Rhodobacter*	*blasticus*	
Z0023_LB_D08_1_C03_B	219	117	LMG 4305	*Rhodobacter*	*blasticus*	
Z0023_RB_C12_2_C07_B	219	117	LMG 4305	*Rhodobacter*	*blasticus*	
Z0023_LB_D09_1_C02_A	219	117	LMG 4305	*Rhodobacter*	*blasticus*	
Z0023_RB_C03_2_C11_A	219	117	LMG 4305	*Rhodobacter*	*blasticus*	
Z0023_LB_C09_2_C02_A	219	117	LMG 4305	*Rhodobacter*	*blasticus*	
Z0023_LB_D10_1_C02_B	219	117	LMG 4305	*Rhodobacter*	*blasticus*	
Z0023_LB_C08_2_C03_B	219	117	LMG 4305	*Rhodobacter*	*blasticus*	
Z0023_LB_C10_2_C02_B	219	117	LMG 4305	*Rhodobacter*	*blasticus*	
Z0023_RB_C09_2_C08_A	219	117	LMG 4305	*Rhodobacter*	*blasticus*	
Z0023_LB_D07_1_C03_A	219	117	LMG 4305	*Rhodobacter*	*blasticus*	
Z0023_RB_C08_2_C09_B	219	117	LMG 4305	*Rhodobacter*	*blasticus*	
Z0023_RB_C06_2_C10_B	220	117	LMG 4305	*Rhodobacter*	*blasticus*	
Z0023_LB_D05_1_C04_A	220	117	LMG 4305	*Rhodobacter*	*blasticus*	
Z0023_RB_C07_2_C09_A	220	117	LMG 4305	*Rhodobacter*	*blasticus*	
Z0023_LB_D06_1_C04_B	220	117	LMG 4305	*Rhodobacter*	*blasticus*	
Z0023_RB_C05_2_C10_A	221	117	LMG 4305	*Rhodobacter*	*blasticus*	
Z0018_LB_G03_2_A05_A	222	71	LMG 24424	*Shewanella*	*vesiculosa*	

Z0018_LB_G04_2_A05_B	222	71	LMG 24424	*Shewanella*	*vesiculosa*
Z0018_RB_F08_1_B09_B	222	71	LMG 24424	*Shewanella*	*vesiculosa*
Z0018_RB_F06_1_B10_B	222	71	LMG 24424	*Shewanella*	*vesiculosa*
Z0018_RB_F07_1_B09_A	222	71	LMG 24424	*Shewanella*	*vesiculosa*
Z0018_LB_G05_2_A04_A	222	71	LMG 24424	*Shewanella*	*vesiculosa*
Z0018_RB_F05_1_B10_A	222	71	LMG 24424	*Shewanella*	*vesiculosa*
Z0018_LB_G06_2_A04_B	222	71	LMG 24424	*Shewanella*	*vesiculosa*
Z0018_RB_E07_2_B09_A	222	71	LMG 24424	*Shewanella*	*vesiculosa*
Z0018_RB_E08_2_B09_B	222	71	LMG 24424	*Shewanella*	*vesiculosa*
Z0018_RB_E04_2_B11_B	222	71	LMG 24424	*Shewanella*	*vesiculosa*
Z0018_LB_F04_1_B05_B	222	71	LMG 24424	*Shewanella*	*vesiculosa*
Z0018_RB_E05_2_B10_A	222	71	LMG 24424	*Shewanella*	*vesiculosa*
Z0018_RB_E06_2_B10_B	222	71	LMG 24424	*Shewanella*	*vesiculosa*
Z0018_LB_F05_1_B04_A	222	71	LMG 24424	*Shewanella*	*vesiculosa*
Z0018_RB_E09_2_B08_A	222	71	LMG 24424	*Shewanella*	*vesiculosa*
Z0018_RB_E11_2_B07_A	222	71	LMG 24424	*Shewanella*	*vesiculosa*
Z0018_RB_E10_2_B08_B	222	71	LMG 24424	*Shewanella*	*vesiculosa*
Z0018_RB_F01_1_B12_A	222	71	LMG 24424	*Shewanella*	*vesiculosa*
Z0018_RB_E12_2_B07_B	222	71	LMG 24424	*Shewanella*	*vesiculosa*
Z0018_LB_G01_2_A06_A	222	71	LMG 24424	*Shewanella*	*vesiculosa*
Z0018_LB_G02_2_A06_B	222	71	LMG 24424	*Shewanella*	*vesiculosa*
Z0018_LB_F11_1_B01_A	222	71	LMG 24424	*Shewanella*	*vesiculosa*
Z0018_RB_F03_1_B11_A	222	71	LMG 24424	*Shewanella*	*vesiculosa*
Z0018_LB_F09_1_B02_A	222	71	LMG 24424	*Shewanella*	*vesiculosa*
Z0018_RB_F02_1_B12_B	222	71	LMG 24424	*Shewanella*	*vesiculosa*
Z0018_LB_F08_1_B03_B	222	71	LMG 24424	*Shewanella*	*vesiculosa*
Z0018_LB_F12_1_B01_B	222	71	LMG 24424	*Shewanella*	*vesiculosa*
Z0018_LB_F06_1_B04_B	222	71	LMG 24424	*Shewanella*	*vesiculosa*
Z0018_LB_F07_1_B03_A	222	71	LMG 24424	*Shewanella*	*vesiculosa*
Z0018_RB_F04_1_B11_B	222	71	LMG 24424	*Shewanella*	*vesiculosa*
Z0018_LB_F10_1_B02_B	222	71	LMG 24424	*Shewanella*	*vesiculosa*
Z0021_RB_H12_1_A07_B	223	103	LMG 2811	*rmoanaerobacteri*	*thermosaccharolyticum*
Z0021_RB_H11_1_A07_A	223	103	LMG 2811	*rmoanaerobacteri*	*thermosaccharolyticum*
Z0021_LB_H08_1_A03_B	223	103	LMG 2811	*rmoanaerobacteri*	*thermosaccharolyticum*
Z0021_RO_A01_1_E07_A	223	103	LMG 2811	*rmoanaerobacteri*	*thermosaccharolyticum*
Z0021_LO_A06_1_E03_B	223	103	LMG 2811	*rmoanaerobacteri*	*thermosaccharolyticum*
Z0021_RB_H08_1_A09_B	223	103	LMG 2811	*rmoanaerobacteri*	*thermosaccharolyticum*
Z0021_LO_A07_1_E04_A	223	103	LMG 2811	*rmoanaerobacteri*	*thermosaccharolyticum*
Z0021_RO_A04_1_E08_B	223	103	LMG 2811	*rmoanaerobacteri*	*thermosaccharolyticum*
Z0021_RO_A05_1_E09_A	223	103	LMG 2811	*rmoanaerobacteri*	*thermosaccharolyticum*
Z0021_RO_A03_1_E08_A	223	103	LMG 2811	*rmoanaerobacteri*	*thermosaccharolyticum*
Z0021_LO_A01_1_E01_A	223	103	LMG 2811	*rmoanaerobacteri*	*thermosaccharolyticum*
Z0021_LB_H11_1_A01_A	223	103	LMG 2811	*rmoanaerobacteri*	*thermosaccharolyticum*
Z0021_LB_H09_1_A02_A	223	103	LMG 2811	*rmoanaerobacteri*	*thermosaccharolyticum*
Z0021_LB_H10_1_A02_B	223	103	LMG 2811	*rmoanaerobacteri*	*thermosaccharolyticum*
Z0021_RO_A07_1_E10_A	223	103	LMG 2811	*rmoanaerobacteri*	*thermosaccharolyticum*
Z0021_RO_A08_1_E10_B	223	103	LMG 2811	*rmoanaerobacteri*	*thermosaccharolyticum*
Z0021_RO_A06_1_E09_B	223	103	LMG 2811	*rmoanaerobacteri*	*thermosaccharolyticum*
Z0021_LO_A08_1_E04_B	223	103	LMG 2811	*rmoanaerobacteri*	*thermosaccharolyticum*
Z0021_LO_A02_1_E01_B	223	103	LMG 2811	*rmoanaerobacteri*	*thermosaccharolyticum*

Z0021_LB_H12_1_A01_B	223	103	LMG 2811	*rmoanaerobacteri*	*thermosaccharolyticum*
Z0021_LO_A05_1_E03_A	223	103	LMG 2811	*rmoanaerobacteri*	*thermosaccharolyticum*
Z0021_LO_A04_1_E02_B	223	103	LMG 2811	*rmoanaerobacteri*	*thermosaccharolyticum*
Z0021_LO_A03_1_E02_A	223	103	LMG 2811	*rmoanaerobacteri*	*thermosaccharolyticum*
Z0021_RB_H10_1_A08_B	223	103	LMG 2811	*rmoanaerobacteri*	*thermosaccharolyticum*
Z0021_RB_H05_1_A10_A	223	103	LMG 2811	*rmoanaerobacteri*	*thermosaccharolyticum*
Z0021_LB_H06_1_A04_B	223	103	LMG 2811	*rmoanaerobacteri*	*thermosaccharolyticum*
Z0021_LB_H05_1_A04_A	223	103	LMG 2811	*rmoanaerobacteri*	*thermosaccharolyticum*
Z0021_LB_H07_1_A03_A	223	103	LMG 2811	*rmoanaerobacteri*	*thermosaccharolyticum*
Z0023_RB_E07_2_B09_A	224	118	LMG 4328	*Curvibacter*	*delicatus*
Z0023_RB_D09_1_C08_A	224	118	LMG 4328	*Curvibacter*	*delicatus*
Z0023_RB_D10_1_C08_B	224	118	LMG 4328	*Curvibacter*	*delicatus*
Z0023_LB_E11_2_B01_A	224	118	LMG 4328	*Curvibacter*	*delicatus*
Z0023_RB_E06_2_B10_B	224	118	LMG 4328	*Curvibacter*	*delicatus*
Z0023_RB_E08_2_B09_B	224	118	LMG 4328	*Curvibacter*	*delicatus*
Z0023_LB_E10_2_B02_B	224	118	LMG 4328	*Curvibacter*	*delicatus*
Z0023_RB_D08_1_C09_B	224	118	LMG 4328	*Curvibacter*	*delicatus*
Z0023_LB_E09_2_B02_A	224	118	LMG 4328	*Curvibacter*	*delicatus*
Z0023_RB_D07_1_C09_A	224	118	LMG 4328	*Curvibacter*	*delicatus*
Z0023_RB_E04_2_B11_B	224	118	LMG 4328	*Curvibacter*	*delicatus*
Z0023_RB_E05_2_B10_A	224	118	LMG 4328	*Curvibacter*	*delicatus*
Z0023_RB_E02_2_B12_B	224	118	LMG 4328	*Curvibacter*	*delicatus*
Z0023_LB_F04_1_B05_B	224	118	LMG 4328	*Curvibacter*	*delicatus*
Z0023_RB_E03_2_B11_A	224	118	LMG 4328	*Curvibacter*	*delicatus*
Z0023_LB_E07_2_B03_A	224	118	LMG 4328	*Curvibacter*	*delicatus*
Z0023_RB_E01_2_B12_A	224	118	LMG 4328	*Curvibacter*	*delicatus*
Z0023_LB_F03_1_B05_A	224	118	LMG 4328	*Curvibacter*	*delicatus*
Z0023_LB_F05_1_B04_A	224	118	LMG 4328	*Curvibacter*	*delicatus*
Z0023_LB_E08_2_B03_B	224	118	LMG 4328	*Curvibacter*	*delicatus*
Z0023_LB_E04_2_B05_B	225	118	LMG 4328	*Curvibacter*	*delicatus*
Z0023_LB_E05_2_B04_A	225	118	LMG 4328	*Curvibacter*	*delicatus*
Z0023_RB_D12_1_C07_B	225	118	LMG 4328	*Curvibacter*	*delicatus*
Z0023_LB_E06_2_B04_B	225	118	LMG 4328	*Curvibacter*	*delicatus*
Z0023_LB_F02_1_B06_B	225	118	LMG 4328	*Curvibacter*	*delicatus*
Z0023_LB_E12_2_B01_B	225	118	LMG 4328	*Curvibacter*	*delicatus*
Z0023_RB_D11_1_C07_A	225	118	LMG 4328	*Curvibacter*	*delicatus*
Z0023_LB_F01_1_B06_A	225	118	LMG 4328	*Curvibacter*	*delicatus*
Z0023_RB_D06_1_C10_B	225	118	LMG 4328	*Curvibacter*	*delicatus*
Z0023_RB_D05_1_C10_A	225	118	LMG 4328	*Curvibacter*	*delicatus*
Z0023_RB_D04_1_C11_B	225	118	LMG 4328	*Curvibacter*	*delicatus*
Z0023_LB_E03_2_B05_A	225	118	LMG 4328	*Curvibacter*	*delicatus*
Z0017_RB_F08_1_B09_B	226	59	LMG 23818	*Rheinheimera*	*chironomi*
Z0017_RB_F06_1_B10_B	226	59	LMG 23818	*Rheinheimera*	*chironomi*
Z0017_LB_F07_1_B03_A	226	59	LMG 23818	*Rheinheimera*	*chironomi*
Z0017_LB_F06_1_B04_B	226	59	LMG 23818	*Rheinheimera*	*chironomi*
Z0017_RB_F05_1_B10_A	226	59	LMG 23818	*Rheinheimera*	*chironomi*
Z0017_RB_F03_1_B11_A	226	59	LMG 23818	*Rheinheimera*	*chironomi*
Z0017_RB_E11_2_B07_A	226	59	LMG 23818	*Rheinheimera*	*chironomi*
Z0017_RB_E09_2_B08_A	226	59	LMG 23818	*Rheinheimera*	*chironomi*
Z0017_RB_E05_2_B10_A	226	59	LMG 23818	*Rheinheimera*	*chironomi*

Z0017_RB_F02_1_B12_B	226	59	LMG 23818	*Rheinheimera*	*chironomi*
Z0017_LB_F04_1_B05_B	226	59	LMG 23818	*Rheinheimera*	*chironomi*
Z0017_RB_E10_2_B08_B	226	59	LMG 23818	*Rheinheimera*	*chironomi*
Z0017_RB_E12_2_B07_B	226	59	LMG 23818	*Rheinheimera*	*chironomi*
Z0017_RB_E07_2_B09_A	226	59	LMG 23818	*Rheinheimera*	*chironomi*
Z0017_LB_E12_2_B01_B	226	59	LMG 23818	*Rheinheimera*	*chironomi*
Z0017_LB_E11_2_B01_A	226	59	LMG 23818	*Rheinheimera*	*chironomi*
Z0017_RB_F04_1_B11_B	226	59	LMG 23818	*Rheinheimera*	*chironomi*
Z0017_RB_E08_2_B09_B	226	59	LMG 23818	*Rheinheimera*	*chironomi*
Z0017_RB_F07_1_B09_A	226	59	LMG 23818	*Rheinheimera*	*chironomi*
Z0017_LB_F01_1_B06_A	226	59	LMG 23818	*Rheinheimera*	*chironomi*
Z0017_LB_E09_2_B02_A	226	59	LMG 23818	*Rheinheimera*	*chironomi*
Z0017_LB_E10_2_B02_B	226	59	LMG 23818	*Rheinheimera*	*chironomi*
Z0017_RB_F09_1_B08_A	226	59	LMG 23818	*Rheinheimera*	*chironomi*
Z0017_LB_F09_1_B02_A	226	59	LMG 23818	*Rheinheimera*	*chironomi*
Z0017_RB_F10_1_B08_B	226	59	LMG 23818	*Rheinheimera*	*chironomi*
Z0017_RB_F01_1_B12_A	226	59	LMG 23818	*Rheinheimera*	*chironomi*
Z0017_LB_F03_1_B05_A	226	59	LMG 23818	*Rheinheimera*	*chironomi*
Z0017_LB_F02_1_B06_B	226	59	LMG 23818	*Rheinheimera*	*chironomi*
Z0017_LB_F10_1_B02_B	226	59	LMG 23818	*Rheinheimera*	*chironomi*
Z0017_RB_E06_2_B10_B	226	59	LMG 23818	*Rheinheimera*	*chironomi*
Z0017_LB_F08_1_B03_B	226	59	LMG 23818	*Rheinheimera*	*chironomi*
Z0017_LB_F05_1_B04_A	226	59	LMG 23818	*Rheinheimera*	*chironomi*
Z0019_RB_G09_2_A08_A	227	82	LMG 25664	*Megasphaera*	*cerevisiae*
Z0019_RB_G11_2_A07_A	227	82	LMG 25664	*Megasphaera*	*cerevisiae*
Z0019_RB_G10_2_A08_B	227	82	LMG 25664	*Megasphaera*	*cerevisiae*
Z0019_LB_G10_2_A02_B	227	82	LMG 25664	*Megasphaera*	*cerevisiae*
Z0019_RB_G03_2_A11_A	227	82	LMG 25664	*Megasphaera*	*cerevisiae*
Z0019_LB_G01_2_A06_A	227	82	LMG 25664	*Megasphaera*	*cerevisiae*
Z0019_RB_G02_2_A12_B	227	82	LMG 25664	*Megasphaera*	*cerevisiae*
Z0019_LB_F09_1_B02_A	227	82	LMG 25664	*Megasphaera*	*cerevisiae*
Z0019_LB_F10_1_B02_B	227	82	LMG 25664	*Megasphaera*	*cerevisiae*
Z0019_LB_G07_2_A03_A	227	82	LMG 25664	*Megasphaera*	*cerevisiae*
Z0019_LB_F11_1_B01_A	227	82	LMG 25664	*Megasphaera*	*cerevisiae*
Z0019_LB_G09_2_A02_A	227	82	LMG 25664	*Megasphaera*	*cerevisiae*
Z0019_LB_H01_1_A06_A	228	82	LMG 25664	*Megasphaera*	*cerevisiae*
Z0019_LB_H02_1_A06_B	228	82	LMG 25664	*Megasphaera*	*cerevisiae*
Z0019_LB_G04_2_A05_B	228	82	LMG 25664	*Megasphaera*	*cerevisiae*
Z0019_RB_G06_2_A10_B	228	82	LMG 25664	*Megasphaera*	*cerevisiae*
Z0019_RB_G07_2_A09_A	228	82	LMG 25664	*Megasphaera*	*cerevisiae*
Z0019_LB_G06_2_A04_B	228	82	LMG 25664	*Megasphaera*	*cerevisiae*
Z0019_LB_G05_2_A04_A	228	82	LMG 25664	*Megasphaera*	*cerevisiae*
Z0019_LB_G12_2_A01_B	228	82	LMG 25664	*Megasphaera*	*cerevisiae*
Z0019_LB_F12_1_B01_B	229	82	LMG 25664	*Megasphaera*	*cerevisiae*
Z0019_LB_G02_2_A06_B	229	82	LMG 25664	*Megasphaera*	*cerevisiae*
Z0019_LB_G08_2_A03_B	229	82	LMG 25664	*Megasphaera*	*cerevisiae*
Z0019_RB_H03_1_A11_A	229	82	LMG 25664	*Megasphaera*	*cerevisiae*
Z0019_RB_G08_2_A09_B	229	82	LMG 25664	*Megasphaera*	*cerevisiae*
Z0019_RB_G04_2_A11_B	230	82	LMG 25664	*Megasphaera*	*cerevisiae*
Z0019_LB_G03_2_A05_A	230	82	LMG 25664	*Megasphaera*	*cerevisiae*

Z0019_RB_G05_2_A10_A	230	82	LMG 25664	*Megasphaera*	*cerevisiae*	
Z0019_RB_G12_2_A07_B	230	82	LMG 25664	*Megasphaera*	*cerevisiae*	
Z0019_LB_G11_2_A01_A	230	82	LMG 25664	*Megasphaera*	*cerevisiae*	
Z0019_RB_H02_1_A12_B	230	82	LMG 25664	*Megasphaera*	*cerevisiae*	
Z0019_RB_H01_1_A12_A	230	82	LMG 25664	*Megasphaera*	*cerevisiae*	
Z0020_RB_G07_2_A09_A	231	91	LMG 26467	*Tardiphaga*	*robiniae*	
Z0020_RB_F07_1_B09_A	231	91	LMG 26467	*Tardiphaga*	*robiniae*	
Z0020_LB_G09_2_A02_A	231	91	LMG 26467	*Tardiphaga*	*robiniae*	
Z0020_RB_G02_2_A12_B	231	91	LMG 26467	*Tardiphaga*	*robiniae*	
Z0020_RB_G11_2_A07_A	231	91	LMG 26467	*Tardiphaga*	*robiniae*	
Z0020_LB_G07_2_A03_A	231	91	LMG 26467	*Tardiphaga*	*robiniae*	
Z0020_RB_G08_2_A09_B	231	91	LMG 26467	*Tardiphaga*	*robiniae*	
Z0020_RB_G06_2_A10_B	231	91	LMG 26467	*Tardiphaga*	*robiniae*	
Z0020_LB_G08_2_A03_B	231	91	LMG 26467	*Tardiphaga*	*robiniae*	
Z0020_RB_F11_1_B07_A	231	91	LMG 26467	*Tardiphaga*	*robiniae*	
Z0020_RB_F12_1_B07_B	231	91	LMG 26467	*Tardiphaga*	*robiniae*	
Z0020_LB_G06_2_A04_B	231	91	LMG 26467	*Tardiphaga*	*robiniae*	
Z0020_LB_H01_1_A06_A	231	91	LMG 26467	*Tardiphaga*	*robiniae*	
Z0020_RB_F08_1_B09_B	231	91	LMG 26467	*Tardiphaga*	*robiniae*	
Z0020_RB_G10_2_A08_B	231	91	LMG 26467	*Tardiphaga*	*robiniae*	
Z0020_LB_G05_2_A04_A	231	91	LMG 26467	*Tardiphaga*	*robiniae*	
Z0020_LB_G04_2_A05_B	231	91	LMG 26467	*Tardiphaga*	*robiniae*	
Z0020_LB_G11_2_A01_A	231	91	LMG 26467	*Tardiphaga*	*robiniae*	
Z0020_RB_G09_2_A08_A	231	91	LMG 26467	*Tardiphaga*	*robiniae*	
Z0020_LB_H02_1_A06_B	231	91	LMG 26467	*Tardiphaga*	*robiniae*	
Z0020_RB_G05_2_A10_A	231	91	LMG 26467	*Tardiphaga*	*robiniae*	
Z0020_RB_F10_1_B08_B	231	91	LMG 26467	*Tardiphaga*	*robiniae*	
Z0020_RB_G03_2_A11_A	231	91	LMG 26467	*Tardiphaga*	*robiniae*	
Z0020_LB_G10_2_A02_B	231	91	LMG 26467	*Tardiphaga*	*robiniae*	
Z0020_RB_G04_2_A11_B	231	91	LMG 26467	*Tardiphaga*	*robiniae*	
Z0020_LB_G12_2_A01_B	231	91	LMG 26467	*Tardiphaga*	*robiniae*	
Z0020_RB_F09_1_B08_A	231	91	LMG 26467	*Tardiphaga*	*robiniae*	
Z0020_RB_G01_2_A12_A	231	91	LMG 26467	*Tardiphaga*	*robiniae*	
Z0020_RB_F06_1_B10_B	231	91	LMG 26467	*Tardiphaga*	*robiniae*	
Z0020_LB_G02_2_A06_B	231	91	LMG 26467	*Tardiphaga*	*robiniae*	
Z0020_RB_F05_1_B10_A	231	91	LMG 26467	*Tardiphaga*	*robiniae*	
Z0020_LB_G03_2_A05_A	231	91	LMG 26467	*Tardiphaga*	*robiniae*	
Z0011_RB_G12_2_A07_B	232	147	R-68675	*Burkholderia*	*cenocepacia*	*IIIA*
Z0011_LO_B02_2_E01_B	232	147	R-68675	*Burkholderia*	*cenocepacia*	*IIIA*
Z0011_LO_A07_1_E04_A	232	147	R-68675	*Burkholderia*	*cenocepacia*	*IIIA*
Z0011_RB_G09_2_A08_A	232	147	R-68675	*Burkholderia*	*cenocepacia*	*IIIA*
Z0011_LO_A06_1_E03_B	232	147	R-68675	*Burkholderia*	*cenocepacia*	*IIIA*
Z0011_RB_H01_1_A12_A	232	147	R-68675	*Burkholderia*	*cenocepacia*	*IIIA*
Z0011_RB_H02_1_A12_B	232	147	R-68675	*Burkholderia*	*cenocepacia*	*IIIA*
Z0011_RB_H04_1_A11_B	232	147	R-68675	*Burkholderia*	*cenocepacia*	*IIIA*
Z0011_RB_H03_1_A11_A	232	147	R-68675	*Burkholderia*	*cenocepacia*	*IIIA*
Z0011_LO_B04_2_E02_B	232	147	R-68675	*Burkholderia*	*cenocepacia*	*IIIA*
Z0011_RB_D04_1_C11_B	232	145	R-67581	*Burkholderia*	*cenocepacia*	*IIIB*
Z0011_LB_F02_1_B06_B	232	145	R-67581	*Burkholderia*	*cenocepacia*	*IIIB*
Z0011_LB_F03_1_B05_A	232	145	R-67581	*Burkholderia*	*cenocepacia*	*IIIB*

Z0011_RB_C07_2_C09_A	232	145	R-67581	*Burkholderia*	*cenocepacia*	*IIIB*
Z0011_LB_E08_2_B03_B	232	145	R-67581	*Burkholderia*	*cenocepacia*	*IIIB*
Z0011_LB_E07_2_B03_A	232	145	R-67581	*Burkholderia*	*cenocepacia*	*IIIB*
Z0011_LB_E09_2_B02_A	232	145	R-67581	*Burkholderia*	*cenocepacia*	*IIIB*
Z0011_RB_D05_1_C10_A	232	145	R-67581	*Burkholderia*	*cenocepacia*	*IIIB*
Z0011_LO_A05_1_E03_A	232	147	R-68675	*Burkholderia*	*cenocepacia*	*IIIA*
Z0011_LO_B03_2_E02_A	232	147	R-68675	*Burkholderia*	*cenocepacia*	*IIIA*
Z0010_LB_E07_2_B03_A	233	147	R-71051	*Burkholderia*	*cenocepacia*	*IIIA*
Z0010_LB_E08_2_B03_B	233	147	R-71051	*Burkholderia*	*cenocepacia*	*IIIA*
Z0010_RB_D06_1_C10_B	233	147	R-71051	*Burkholderia*	*cenocepacia*	*IIIA*
Z0010_RB_D05_1_C10_A	233	147	R-71051	*Burkholderia*	*cenocepacia*	*IIIA*
Z0010_LB_E06_2_B04_B	233	147	R-71051	*Burkholderia*	*cenocepacia*	*IIIA*
Z0010_LO_D04_2_F02_B	233	147	R-68806	*Burkholderia*	*cenocepacia*	*IIIA*
Z0010_LO_D01_2_F01_A	233	147	R-68806	*Burkholderia*	*cenocepacia*	*IIIA*
Z0010_RO_C06_1_F09_B	233	147	R-68806	*Burkholderia*	*cenocepacia*	*IIIA*
Z0010_RB_A06_2_D10_B	233	147	R-68806	*Burkholderia*	*cenocepacia*	*IIIA*
Z0010_RB_A09_2_D08_A	233	147	R-68806	*Burkholderia*	*cenocepacia*	*IIIA*
Z0010_LB_A01_2_D06_A	233	147	R-68806	*Burkholderia*	*cenocepacia*	*IIIA*
Z0010_LB_A11_2_D01_A	233	147	R-68806	*Burkholderia*	*cenocepacia*	*IIIA*
Z0010_RB_A07_2_D09_A	233	147	R-68806	*Burkholderia*	*cenocepacia*	*IIIA*
Z0010_RB_D12_1_C07_B	233	147	R-71051	*Burkholderia*	*cenocepacia*	*IIIA*
Z0010_LB_F01_1_B06_A	233	147	R-71051	*Burkholderia*	*cenocepacia*	*IIIA*
Z0010_LB_H01_1_A06_A	233	147	R-71085	*Burkholderia*	*cenocepacia*	*IIIA*
Z0010_LB_H02_1_A06_B	233	147	R-71085	*Burkholderia*	*cenocepacia*	*IIIA*
Z0010_RB_G02_2_A12_B	233	147	R-71085	*Burkholderia*	*cenocepacia*	*IIIA*
Z0010_RB_F11_1_B07_A	233	147	R-71085	*Burkholderia*	*cenocepacia*	*IIIA*
Z0010_RB_G03_2_A11_A	233	147	R-71085	*Burkholderia*	*cenocepacia*	*IIIA*
Z0010_LB_F05_1_B04_A	233	147	R-71051	*Burkholderia*	*cenocepacia*	*IIIA*
Z0010_LB_E12_2_B01_B	233	147	R-71051	*Burkholderia*	*cenocepacia*	*IIIA*
Z0010_RB_E05_2_B10_A	233	147	R-71051	*Burkholderia*	*cenocepacia*	*IIIA*
Z0010_RB_E07_2_B09_A	233	147	R-71051	*Burkholderia*	*cenocepacia*	*IIIA*
Z0010_LB_F06_1_B04_B	233	147	R-71051	*Burkholderia*	*cenocepacia*	*IIIA*
Z0010_RB_E02_2_B12_B	233	147	R-71051	*Burkholderia*	*cenocepacia*	*IIIA*
Z0010_RB_D09_1_C08_A	233	147	R-71051	*Burkholderia*	*cenocepacia*	*IIIA*
Z0010_RB_D08_1_C09_B	233	147	R-71051	*Burkholderia*	*cenocepacia*	*IIIA*
Z0010_LB_F03_1_B05_A	233	147	R-71051	*Burkholderia*	*cenocepacia*	*IIIA*
Z0010_RB_E06_2_B10_B	233	147	R-71051	*Burkholderia*	*cenocepacia*	*IIIA*
Z0010_LB_E09_2_B02_A	233	147	R-71051	*Burkholderia*	*cenocepacia*	*IIIA*
Z0010_LB_F04_1_B05_B	233	147	R-71051	*Burkholderia*	*cenocepacia*	*IIIA*
Z0010_RB_D07_1_C09_A	233	147	R-71051	*Burkholderia*	*cenocepacia*	*IIIA*
Z0010_RB_D11_1_C07_A	233	147	R-71051	*Burkholderia*	*cenocepacia*	*IIIA*
Z0010_RB_E01_2_B12_A	233	147	R-71051	*Burkholderia*	*cenocepacia*	*IIIA*
Z0010_RB_A11_2_D07_A	233	147	R-68806	*Burkholderia*	*cenocepacia*	*IIIA*
Z0010_LB_A09_2_D02_A	233	147	R-68806	*Burkholderia*	*cenocepacia*	*IIIA*
Z0010_LB_A02_2_D06_B	233	147	R-68806	*Burkholderia*	*cenocepacia*	*IIIA*
Z0010_RB_A12_2_D07_B	233	147	R-68806	*Burkholderia*	*cenocepacia*	*IIIA*
Z0010_LB_A07_2_D03_A	233	147	R-68806	*Burkholderia*	*cenocepacia*	*IIIA*
Z0010_LB_A08_2_D03_B	233	147	R-68806	*Burkholderia*	*cenocepacia*	*IIIA*
Z0010_LB_A10_2_D02_B	233	147	R-68806	*Burkholderia*	*cenocepacia*	*IIIA*
Z0010_RB_A02_2_D12_B	233	147	R-68806	*Burkholderia*	*cenocepacia*	*IIIA*

Z0010_RO_C10_1_F11_B	233	147	R-68806	*Burkholderia*	*cenocepacia*	*IIIA*
Z0010_LB_A03_2_D05_A	233	147	R-68806	*Burkholderia*	*cenocepacia*	*IIIA*
Z0010_RB_A10_2_D08_B	233	147	R-68806	*Burkholderia*	*cenocepacia*	*IIIA*
Z0010_LB_A06_2_D04_B	233	147	R-68806	*Burkholderia*	*cenocepacia*	*IIIA*
Z0010_RO_C05_1_F09_A	233	147	R-68806	*Burkholderia*	*cenocepacia*	*IIIA*
Z0010_RO_C08_1_F10_B	234	147	R-68806	*Burkholderia*	*cenocepacia*	*IIIA*
Z0010_RO_C12_1_F12_B	234	147	R-68806	*Burkholderia*	*cenocepacia*	*IIIA*
Z0010_RB_G07_2_A09_A	235	147	R-71085	*Burkholderia*	*cenocepacia*	*IIIA*
Z0010_LB_E04_2_B05_B	236	147	R-71051	*Burkholderia*	*cenocepacia*	*IIIA*
Z0010_LB_E05_2_B04_A	236	147	R-71051	*Burkholderia*	*cenocepacia*	*IIIA*
Z0010_LB_E03_2_B05_A	236	147	R-71051	*Burkholderia*	*cenocepacia*	*IIIA*
Z0011_RB_F02_1_B12_B	237	146	R-68591	*Burkholderia*	*cenocepacia*	*IIIB*
Z0011_RB_F04_1_B11_B	237	146	R-68591	*Burkholderia*	*cenocepacia*	*IIIB*
Z0011_RB_F03_1_B11_A	237	146	R-68591	*Burkholderia*	*cenocepacia*	*IIIB*
Z0011_RB_E09_2_B08_A	237	146	R-68591	*Burkholderia*	*cenocepacia*	*IIIB*
Z0011_LB_G06_2_A04_B	237	146	R-68591	*Burkholderia*	*cenocepacia*	*IIIB*
Z0011_RB_F01_1_B12_A	237	146	R-68591	*Burkholderia*	*cenocepacia*	*IIIB*
Z0011_LB_G11_2_A01_A	237	146	R-68591	*Burkholderia*	*cenocepacia*	*IIIB*
Z0011_RB_E08_2_B09_B	237	146	R-68591	*Burkholderia*	*cenocepacia*	*IIIB*
Z0011_RB_D11_1_C07_A	237	146	R-68591	*Burkholderia*	*cenocepacia*	*IIIB*
Z0011_RB_E01_2_B12_A	237	146	R-68591	*Burkholderia*	*cenocepacia*	*IIIB*
Z0011_LB_F11_1_B01_A	237	146	R-68591	*Burkholderia*	*cenocepacia*	*IIIB*
Z0011_RB_D12_1_C07_B	237	146	R-68591	*Burkholderia*	*cenocepacia*	*IIIB*
Z0011_RB_E07_2_B09_A	237	146	R-68591	*Burkholderia*	*cenocepacia*	*IIIB*
Z0011_LB_G04_2_A05_B	237	146	R-68591	*Burkholderia*	*cenocepacia*	*IIIB*
Z0011_RB_E06_2_B10_B	237	146	R-68591	*Burkholderia*	*cenocepacia*	*IIIB*
Z0011_LB_G05_2_A04_A	237	146	R-68591	*Burkholderia*	*cenocepacia*	*IIIB*
Z0011_LB_G01_2_A06_A	237	146	R-68591	*Burkholderia*	*cenocepacia*	*IIIB*
Z0011_LB_F12_1_B01_B	237	146	R-68591	*Burkholderia*	*cenocepacia*	*IIIB*
Z0011_RB_E02_2_B12_B	237	146	R-68591	*Burkholderia*	*cenocepacia*	*IIIB*
Z0011_RB_E03_2_B11_A	237	146	R-68591	*Burkholderia*	*cenocepacia*	*IIIB*
Z0011_RB_E10_2_B08_B	237	146	R-68591	*Burkholderia*	*cenocepacia*	*IIIB*
Z0011_LB_G03_2_A05_A	237	146	R-68591	*Burkholderia*	*cenocepacia*	*IIIB*
Z0011_RB_E04_2_B11_B	237	146	R-68591	*Burkholderia*	*cenocepacia*	*IIIB*
Z0011_RB_E05_2_B10_A	237	146	R-68591	*Burkholderia*	*cenocepacia*	*IIIB*
Z0011_LB_G02_2_A06_B	237	146	R-68591	*Burkholderia*	*cenocepacia*	*IIIB*
Z0011_LB_H03_1_A05_A	237	146	R-68599	*Burkholderia*	*cenocepacia*	*IIIB*
Z0011_LB_B08_1_D03_B	237	145	R-67259	*Burkholderia*	*cenocepacia*	*IIIB*
Z0011_RB_G11_2_A07_A	238	147	R-68675	*Burkholderia*	*cenocepacia*	*IIIA*
Z0011_LO_B01_2_E01_A	238	147	R-68675	*Burkholderia*	*cenocepacia*	*IIIA*
Z0011_LO_A08_1_E04_B	238	147	R-68675	*Burkholderia*	*cenocepacia*	*IIIA*
Z0011_LO_A10_1_E05_B	238	147	R-68675	*Burkholderia*	*cenocepacia*	*IIIA*
Z0011_LO_A12_1_E06_B	238	147	R-68675	*Burkholderia*	*cenocepacia*	*IIIA*
Z0011_RB_H08_1_A09_B	238	147	R-68675	*Burkholderia*	*cenocepacia*	*IIIA*
Z0011_RB_H10_1_A08_B	238	147	R-68675	*Burkholderia*	*cenocepacia*	*IIIA*
Z0011_LO_B06_2_E03_B	238	147	R-68675	*Burkholderia*	*cenocepacia*	*IIIA*
Z0011_RB_H05_1_A10_A	238	147	R-68675	*Burkholderia*	*cenocepacia*	*IIIA*
Z0011_RB_H09_1_A08_A	238	147	R-68675	*Burkholderia*	*cenocepacia*	*IIIA*
Z0011_RB_H07_1_A09_A	238	147	R-68675	*Burkholderia*	*cenocepacia*	*IIIA*
Z0011_LO_B05_2_E03_A	238	147	R-68675	*Burkholderia*	*cenocepacia*	*IIIA*

Z0011_LO_A09_1_E05_A	238	147	R-68675	*Burkholderia*	*cenocepacia*	*IIIA*
Z0011_RB_B01_1_D12_A	238	145	R-67259	*Burkholderia*	*cenocepacia*	*IIIB*
Z0011_LB_C10_2_C02_B	238	145	R-67259	*Burkholderia*	*cenocepacia*	*IIIB*
Z0011_RB_B02_1_D12_B	238	145	R-67259	*Burkholderia*	*cenocepacia*	*IIIB*
Z0011_LB_C09_2_C02_A	238	145	R-67259	*Burkholderia*	*cenocepacia*	*IIIB*
Z0011_LB_C12_2_C01_B	238	145	R-67259	*Burkholderia*	*cenocepacia*	*IIIB*
Z0011_LB_C11_2_C01_A	238	145	R-67259	*Burkholderia*	*cenocepacia*	*IIIB*
Z0011_RB_A10_2_D08_B	238	145	R-67259	*Burkholderia*	*cenocepacia*	*IIIB*
Z0011_RB_A11_2_D07_A	238	145	R-67259	*Burkholderia*	*cenocepacia*	*IIIB*
Z0011_RB_A12_2_D07_B	238	145	R-67259	*Burkholderia*	*cenocepacia*	*IIIB*
Z0011_LB_C07_2_C03_A	238	145	R-67259	*Burkholderia*	*cenocepacia*	*IIIB*
Z0011_LB_C01_2_C06_A	238	145	R-67259	*Burkholderia*	*cenocepacia*	*IIIB*
Z0011_RB_A07_2_D09_A	238	145	R-67259	*Burkholderia*	*cenocepacia*	*IIIB*
Z0011_LB_C03_2_C05_A	238	145	R-67259	*Burkholderia*	*cenocepacia*	*IIIB*
Z0011_LB_B09_1_D02_A	238	145	R-67259	*Burkholderia*	*cenocepacia*	*IIIB*
Z0011_LB_B07_1_D03_A	238	145	R-67259	*Burkholderia*	*cenocepacia*	*IIIB*
Z0011_LB_B06_1_D04_B	238	145	R-67259	*Burkholderia*	*cenocepacia*	*IIIB*
Z0011_LB_C02_2_C06_B	238	145	R-67259	*Burkholderia*	*cenocepacia*	*IIIB*
Z0011_LB_B12_1_D01_B	238	145	R-67259	*Burkholderia*	*cenocepacia*	*IIIB*
Z0011_RB_A09_2_D08_A	238	145	R-67259	*Burkholderia*	*cenocepacia*	*IIIB*
Z0011_LO_A01_1_E01_A	238	146	R-68599	*Burkholderia*	*cenocepacia*	*IIIB*
Z0011_RB_B04_1_D11_B	238	145	R-67259	*Burkholderia*	*cenocepacia*	*IIIB*
Z0011_RB_A06_2_D10_B	238	145	R-67259	*Burkholderia*	*cenocepacia*	*IIIB*
Z0011_RB_B03_1_D11_A	238	145	R-67259	*Burkholderia*	*cenocepacia*	*IIIB*
Z0011_LB_C08_2_C03_B	238	145	R-67259	*Burkholderia*	*cenocepacia*	*IIIB*
Z0011_RB_A08_2_D09_B	238	145	R-67259	*Burkholderia*	*cenocepacia*	*IIIB*
Z0011_LB_C04_2_C05_B	238	145	R-67259	*Burkholderia*	*cenocepacia*	*IIIB*
Z0011_RB_D09_1_C08_A	238	145	R-67581	*Burkholderia*	*cenocepacia*	*IIIB*
Z0011_LB_F10_1_B02_B	238	145	R-67581	*Burkholderia*	*cenocepacia*	*IIIB*
Z0011_LB_F09_1_B02_A	238	145	R-67581	*Burkholderia*	*cenocepacia*	*IIIB*
Z0011_RB_D10_1_C08_B	238	145	R-67581	*Burkholderia*	*cenocepacia*	*IIIB*
Z0011_LB_C06_2_C04_B	238	145	R-67259	*Burkholderia*	*cenocepacia*	*IIIB*
Z0011_LB_C05_2_C04_A	238	145	R-67259	*Burkholderia*	*cenocepacia*	*IIIB*
Z0011_LB_B10_1_D02_B	238	145	R-67259	*Burkholderia*	*cenocepacia*	*IIIB*
Z0011_RB_A05_2_D10_A	238	145	R-67259	*Burkholderia*	*cenocepacia*	*IIIB*
Z0011_RB_A04_2_D11_B	238	145	R-67259	*Burkholderia*	*cenocepacia*	*IIIB*
Z0011_LB_B11_1_D01_A	238	145	R-67259	*Burkholderia*	*cenocepacia*	*IIIB*
Z0011_RB_D07_1_C09_A	238	145	R-67581	*Burkholderia*	*cenocepacia*	*IIIB*
Z0011_RB_D02_1_C12_B	238	145	R-67581	*Burkholderia*	*cenocepacia*	*IIIB*
Z0011_RB_D03_1_C11_A	238	145	R-67581	*Burkholderia*	*cenocepacia*	*IIIB*
Z0011_RB_D01_1_C12_A	238	145	R-67581	*Burkholderia*	*cenocepacia*	*IIIB*
Z0011_RB_D08_1_C09_B	238	145	R-67581	*Burkholderia*	*cenocepacia*	*IIIB*
Z0011_LB_F08_1_B03_B	238	145	R-67581	*Burkholderia*	*cenocepacia*	*IIIB*
Z0011_RB_D06_1_C10_B	238	145	R-67581	*Burkholderia*	*cenocepacia*	*IIIB*
Z0011_LB_E12_2_B01_B	238	145	R-67581	*Burkholderia*	*cenocepacia*	*IIIB*
Z0011_LB_F01_1_B06_A	238	145	R-67581	*Burkholderia*	*cenocepacia*	*IIIB*
Z0011_LB_E11_2_B01_A	238	145	R-67581	*Burkholderia*	*cenocepacia*	*IIIB*
Z0011_LB_F06_1_B04_B	238	145	R-67581	*Burkholderia*	*cenocepacia*	*IIIB*
Z0011_LB_F05_1_B04_A	238	145	R-67581	*Burkholderia*	*cenocepacia*	*IIIB*
Z0011_RB_C11_2_C07_A	238	145	R-67581	*Burkholderia*	*cenocepacia*	*IIIB*

Z0011_RB_C12_2_C07_B	238	145	R-67581	*Burkholderia*	*cenocepacia*	*IIIB*
Z0011_LB_F07_1_B03_A	238	145	R-67581	*Burkholderia*	*cenocepacia*	*IIIB*
Z0011_LB_F04_1_B05_B	238	145	R-67581	*Burkholderia*	*cenocepacia*	*IIIB*
Z0011_RB_C10_2_C08_B	238	145	R-67581	*Burkholderia*	*cenocepacia*	*IIIB*
Z0011_LB_E10_2_B02_B	238	145	R-67581	*Burkholderia*	*cenocepacia*	*IIIB*
Z0011_RB_C08_2_C09_B	238	145	R-67581	*Burkholderia*	*cenocepacia*	*IIIB*
Z0011_RB_C09_2_C08_A	238	145	R-67581	*Burkholderia*	*cenocepacia*	*IIIB*
Z0011_RB_H06_1_A10_B	239	147	R-68675	*Burkholderia*	*cenocepacia*	*IIIA*
Z0011_LO_A11_1_E06_A	239	147	R-68675	*Burkholderia*	*cenocepacia*	*IIIA*
Z0011_RB_G10_2_A08_B	239	147	R-68675	*Burkholderia*	*cenocepacia*	*IIIA*
Z0011_RB_F12_1_B07_B	239	146	R-68599	*Burkholderia*	*cenocepacia*	*IIIB*
Z0011_LB_H08_1_A03_B	239	146	R-68599	*Burkholderia*	*cenocepacia*	*IIIB*
Z0011_LB_H07_1_A03_A	239	146	R-68599	*Burkholderia*	*cenocepacia*	*IIIB*
Z0011_RB_F11_1_B07_A	239	146	R-68599	*Burkholderia*	*cenocepacia*	*IIIB*
Z0011_RB_G07_2_A09_A	239	146	R-68599	*Burkholderia*	*cenocepacia*	*IIIB*
Z0011_RB_G08_2_A09_B	239	146	R-68599	*Burkholderia*	*cenocepacia*	*IIIB*
Z0011_LO_A04_1_E02_B	239	146	R-68599	*Burkholderia*	*cenocepacia*	*IIIB*
Z0011_LO_A03_1_E02_A	239	146	R-68599	*Burkholderia*	*cenocepacia*	*IIIB*
Z0011_LB_H06_1_A04_B	239	146	R-68599	*Burkholderia*	*cenocepacia*	*IIIB*
Z0011_RB_G06_2_A10_B	240	146	R-68599	*Burkholderia*	*cenocepacia*	*IIIB*
Z0011_LO_A02_1_E01_B	240	146	R-68599	*Burkholderia*	*cenocepacia*	*IIIB*
Z0011_RB_G01_2_A12_A	240	146	R-68599	*Burkholderia*	*cenocepacia*	*IIIB*
Z0011_LB_H11_1_A01_A	240	146	R-68599	*Burkholderia*	*cenocepacia*	*IIIB*
Z0011_LB_H01_1_A06_A	240	146	R-68599	*Burkholderia*	*cenocepacia*	*IIIB*
Z0011_LB_H04_1_A05_B	240	146	R-68599	*Burkholderia*	*cenocepacia*	*IIIB*
Z0011_LB_H02_1_A06_B	240	146	R-68599	*Burkholderia*	*cenocepacia*	*IIIB*
Z0011_RB_F05_1_B10_A	240	146	R-68599	*Burkholderia*	*cenocepacia*	*IIIB*
Z0011_LB_H05_1_A04_A	240	146	R-68599	*Burkholderia*	*cenocepacia*	*IIIB*
Z0011_RB_G03_2_A11_A	240	146	R-68599	*Burkholderia*	*cenocepacia*	*IIIB*
Z0011_RB_G05_2_A10_A	240	146	R-68599	*Burkholderia*	*cenocepacia*	*IIIB*
Z0011_RB_F09_1_B08_A	240	146	R-68599	*Burkholderia*	*cenocepacia*	*IIIB*
Z0011_RB_F07_1_B09_A	240	146	R-68599	*Burkholderia*	*cenocepacia*	*IIIB*
Z0011_RB_F06_1_B10_B	240	146	R-68599	*Burkholderia*	*cenocepacia*	*IIIB*
Z0011_RB_F10_1_B08_B	240	146	R-68599	*Burkholderia*	*cenocepacia*	*IIIB*
Z0011_RB_F08_1_B09_B	240	146	R-68599	*Burkholderia*	*cenocepacia*	*IIIB*
Z0011_RB_E12_2_B07_B	240	146	R-68591	*Burkholderia*	*cenocepacia*	*IIIB*
Z0011_RB_E11_2_B07_A	240	146	R-68591	*Burkholderia*	*cenocepacia*	*IIIB*
Z0011_LB_G08_2_A03_B	240	146	R-68591	*Burkholderia*	*cenocepacia*	*IIIB*
Z0011_LB_H12_1_A01_B	240	146	R-68599	*Burkholderia*	*cenocepacia*	*IIIB*
Z0011_LB_H10_1_A02_B	240	146	R-68599	*Burkholderia*	*cenocepacia*	*IIIB*
Z0011_RB_G04_2_A11_B	240	146	R-68599	*Burkholderia*	*cenocepacia*	*IIIB*
Z0011_RB_G02_2_A12_B	240	146	R-68599	*Burkholderia*	*cenocepacia*	*IIIB*
Z0010_RB_A03_2_D11_A	241	147	R-68806	*Burkholderia*	*cenocepacia*	*IIIA*
Z0010_LB_A04_2_D05_B	241	147	R-68806	*Burkholderia*	*cenocepacia*	*IIIA*
Z0010_RB_G12_2_A07_B	241	147	R-71085	*Burkholderia*	*cenocepacia*	*IIIA*
Z0010_LB_H07_1_A03_A	241	147	R-71085	*Burkholderia*	*cenocepacia*	*IIIA*
Z0010_RB_A08_2_D09_B	241	147	R-68806	*Burkholderia*	*cenocepacia*	*IIIA*
Z0010_LB_A05_2_D04_A	241	147	R-68806	*Burkholderia*	*cenocepacia*	*IIIA*
Z0010_RB_A04_2_D11_B	241	147	R-68806	*Burkholderia*	*cenocepacia*	*IIIA*
Z0010_LB_H05_1_A04_A	241	147	R-71085	*Burkholderia*	*cenocepacia*	*IIIA*

Z0010_LB_H04_1_A05_B	241	147	R-71085	*Burkholderia*	*cenocepacia*	*IIIA*
Z0010_RB_G08_2_A09_B	241	147	R-71085	*Burkholderia*	*cenocepacia*	*IIIA*
Z0010_RB_F12_1_B07_B	242	147	R-71085	*Burkholderia*	*cenocepacia*	*IIIA*
Z0010_LB_G12_2_A01_B	242	147	R-71085	*Burkholderia*	*cenocepacia*	*IIIA*
Z0010_LB_A12_2_D01_B	242	147	R-68806	*Burkholderia*	*cenocepacia*	*IIIA*
Z0010_RB_H02_1_A12_B	242	147	R-71085	*Burkholderia*	*cenocepacia*	*IIIA*
Z0010_LO_A01_1_E01_A	242	147	R-71085	*Burkholderia*	*cenocepacia*	*IIIA*
Z0010_RB_G01_2_A12_A	242	147	R-71085	*Burkholderia*	*cenocepacia*	*IIIA*
Z0010_RB_E04_2_B11_B	242	147	R-71051	*Burkholderia*	*cenocepacia*	*IIIA*
Z0010_LB_E11_2_B01_A	242	147	R-71051	*Burkholderia*	*cenocepacia*	*IIIA*
Z0010_RB_D10_1_C08_B	242	147	R-71051	*Burkholderia*	*cenocepacia*	*IIIA*
Z0010_RB_E03_2_B11_A	242	147	R-71051	*Burkholderia*	*cenocepacia*	*IIIA*
Z0010_LB_E10_2_B02_B	242	147	R-71051	*Burkholderia*	*cenocepacia*	*IIIA*
Z0010_LO_D06_2_F03_B	242	147	R-68806	*Burkholderia*	*cenocepacia*	*IIIA*
Z0010_LB_B01_1_D06_A	242	147	R-68806	*Burkholderia*	*cenocepacia*	*IIIA*
Z0010_RB_D04_1_C11_B	242	147	R-71051	*Burkholderia*	*cenocepacia*	*IIIA*
Z0010_RB_G09_2_A08_A	242	147	R-71085	*Burkholderia*	*cenocepacia*	*IIIA*
Z0010_RB_G11_2_A07_A	242	147	R-71085	*Burkholderia*	*cenocepacia*	*IIIA*
Z0010_RB_G04_2_A11_B	242	147	R-71085	*Burkholderia*	*cenocepacia*	*IIIA*
Z0010_LB_F02_1_B06_B	242	147	R-71051	*Burkholderia*	*cenocepacia*	*IIIA*
Z0010_RB_G05_2_A10_A	242	147	R-71085	*Burkholderia*	*cenocepacia*	*IIIA*
Z0010_LB_H10_1_A02_B	242	147	R-71085	*Burkholderia*	*cenocepacia*	*IIIA*
Z0010_RB_G10_2_A08_B	242	147	R-71085	*Burkholderia*	*cenocepacia*	*IIIA*
Z0010_RB_H03_1_A11_A	242	147	R-71085	*Burkholderia*	*cenocepacia*	*IIIA*
Z0010_LB_H12_1_A01_B	242	147	R-71085	*Burkholderia*	*cenocepacia*	*IIIA*
Z0010_RB_H04_1_A11_B	242	147	R-71085	*Burkholderia*	*cenocepacia*	*IIIA*
Z0010_RB_A05_2_D10_A	242	147	R-68806	*Burkholderia*	*cenocepacia*	*IIIA*
Z0010_LB_H11_1_A01_A	242	147	R-71085	*Burkholderia*	*cenocepacia*	*IIIA*
Z0010_LB_H03_1_A05_A	242	147	R-71085	*Burkholderia*	*cenocepacia*	*IIIA*
Z0010_LB_H09_1_A02_A	242	147	R-71085	*Burkholderia*	*cenocepacia*	*IIIA*
Z0011_LB_G09_2_A02_A	243	146	R-68591	*Burkholderia*	*cenocepacia*	*IIIB*
Z0011_LB_G07_2_A03_A	243	146	R-68591	*Burkholderia*	*cenocepacia*	*IIIB*
Z0011_LB_H09_1_A02_A	244	146	R-68599	*Burkholderia*	*cenocepacia*	*IIIB*
Z0011_LB_G10_2_A02_B	245	146	R-68591	*Burkholderia*	*cenocepacia*	*IIIB*
Z0011_LB_G12_2_A01_B	246	146	R-68591	*Burkholderia*	*cenocepacia*	*IIIB*
Z0010_RB_G06_2_A10_B	247	147	R-71085	*Burkholderia*	*cenocepacia*	*IIIA*
Z0010_LB_H06_1_A04_B	247	147	R-71085	*Burkholderia*	*cenocepacia*	*IIIA*
Z0010_RB_H01_1_A12_A	247	147	R-71085	*Burkholderia*	*cenocepacia*	*IIIA*
Z0010_LB_H08_1_A03_B	248	147	R-71085	*Burkholderia*	*cenocepacia*	*IIIA*
Z0010_LB_G07_2_A03_A	249	143	R-69593	*Burkholderia*	*vietnamiensis*	
Z0010_LB_F12_1_B01_B	249	143	R-69593	*Burkholderia*	*vietnamiensis*	
Z0010_RB_F02_1_B12_B	249	143	R-69593	*Burkholderia*	*vietnamiensis*	
Z0010_LB_G06_2_A04_B	249	143	R-69593	*Burkholderia*	*vietnamiensis*	
Z0010_LB_G09_2_A02_A	249	143	R-69593	*Burkholderia*	*vietnamiensis*	
Z0010_RB_E08_2_B09_B	249	143	R-69593	*Burkholderia*	*vietnamiensis*	
Z0010_RB_F03_1_B11_A	249	143	R-69593	*Burkholderia*	*vietnamiensis*	
Z0010_RB_F08_1_B09_B	249	143	R-69593	*Burkholderia*	*vietnamiensis*	
Z0010_RB_F09_1_B08_A	249	143	R-69593	*Burkholderia*	*vietnamiensis*	
Z0010_RB_F10_1_B08_B	249	143	R-69593	*Burkholderia*	*vietnamiensis*	
Z0010_LB_G01_2_A06_A	249	143	R-69593	*Burkholderia*	*vietnamiensis*	

Z0010_LB_G05_2_A04_A	249	143	R-69593	*Burkholderia*	*vietnamiensis*
Z0010_LB_F07_1_B03_A	249	143	R-69593	*Burkholderia*	*vietnamiensis*
Z0010_RB_F07_1_B09_A	249	143	R-69593	*Burkholderia*	*vietnamiensis*
Z0010_LB_G10_2_A02_B	249	143	R-69593	*Burkholderia*	*vietnamiensis*
Z0010_RB_F06_1_B10_B	249	143	R-69593	*Burkholderia*	*vietnamiensis*
Z0010_RB_F04_1_B11_B	249	143	R-69593	*Burkholderia*	*vietnamiensis*
Z0010_LB_F10_1_B02_B	249	143	R-69593	*Burkholderia*	*vietnamiensis*
Z0010_RB_F05_1_B10_A	249	143	R-69593	*Burkholderia*	*vietnamiensis*
Z0010_LB_F11_1_B01_A	249	143	R-69593	*Burkholderia*	*vietnamiensis*
Z0010_RB_E11_2_B07_A	249	143	R-69593	*Burkholderia*	*vietnamiensis*
Z0010_RB_E10_2_B08_B	249	143	R-69593	*Burkholderia*	*vietnamiensis*
Z0010_RB_E09_2_B08_A	249	143	R-69593	*Burkholderia*	*vietnamiensis*
Z0010_LB_F08_1_B03_B	249	143	R-69593	*Burkholderia*	*vietnamiensis*
Z0010_LB_G02_2_A06_B	249	143	R-69593	*Burkholderia*	*vietnamiensis*
Z0010_LB_G11_2_A01_A	249	143	R-69593	*Burkholderia*	*vietnamiensis*
Z0010_LB_G08_2_A03_B	249	143	R-69593	*Burkholderia*	*vietnamiensis*
Z0010_RB_E12_2_B07_B	249	143	R-69593	*Burkholderia*	*vietnamiensis*
Z0010_LB_F09_1_B02_A	250	143	R-69593	*Burkholderia*	*vietnamiensis*
Z0010_RB_F01_1_B12_A	251	143	R-69593	*Burkholderia*	*vietnamiensis*
Z0010_LB_G03_2_A05_A	251	143	R-69593	*Burkholderia*	*vietnamiensis*
Z0010_LB_G04_2_A05_B	251	143	R-69593	*Burkholderia*	*vietnamiensis*
Z0010_RO_G11_1_H12_A	252	143	R-67189	*Burkholderia*	*vietnamiensis*
Z0010_LO_H07_2_H04_A	252	143	R-67189	*Burkholderia*	*vietnamiensis*
Z0010_RO_G12_1_H12_B	252	143	R-67189	*Burkholderia*	*vietnamiensis*
Z0010_LO_H12_2_H06_B	252	143	R-67189	*Burkholderia*	*vietnamiensis*
Z0010_LO_H11_2_H06_A	252	143	R-67189	*Burkholderia*	*vietnamiensis*
Z0010_LO_H09_2_H05_A	252	143	R-67189	*Burkholderia*	*vietnamiensis*
Z0010_RO_G10_1_H11_B	252	143	R-67189	*Burkholderia*	*vietnamiensis*
Z0010_LO_H05_2_H03_A	252	143	R-67189	*Burkholderia*	*vietnamiensis*
Z0010_LO_H03_2_H02_A	252	143	R-67189	*Burkholderia*	*vietnamiensis*
Z0010_RO_G06_1_H09_B	252	143	R-67189	*Burkholderia*	*vietnamiensis*
Z0010_LO_H06_2_H03_B	252	143	R-67189	*Burkholderia*	*vietnamiensis*
Z0010_RO_G07_1_H10_A	252	143	R-67189	*Burkholderia*	*vietnamiensis*
Z0010_LO_H04_2_H02_B	252	143	R-67189	*Burkholderia*	*vietnamiensis*
Z0010_RO_H02_2_H07_B	252	143	R-67189	*Burkholderia*	*vietnamiensis*
Z0010_RO_H03_2_H08_A	252	143	R-67189	*Burkholderia*	*vietnamiensis*
Z0010_RB_A01_2_D12_A	252	143	R-67189	*Burkholderia*	*vietnamiensis*
Z0010_RO_F05_2_G09_A	252	143	R-67189	*Burkholderia*	*vietnamiensis*
Z0010_LO_H02_2_H01_B	252	143	R-67189	*Burkholderia*	*vietnamiensis*
Z0010_LO_H01_2_H01_A	252	143	R-67189	*Burkholderia*	*vietnamiensis*
Z0010_RO_G08_1_H10_B	252	143	R-67189	*Burkholderia*	*vietnamiensis*
Z0010_LO_H08_2_H04_B	252	143	R-67189	*Burkholderia*	*vietnamiensis*
Z0010_RO_F02_2_G07_B	252	143	R-67189	*Burkholderia*	*vietnamiensis*
Z0010_RO_H01_2_H07_A	252	143	R-67189	*Burkholderia*	*vietnamiensis*
Z0010_RO_F08_2_G10_B	253	143	R-67189	*Burkholderia*	*vietnamiensis*
Z0010_LO_H10_2_H05_B	253	143	R-67189	*Burkholderia*	*vietnamiensis*
Z0010_RO_F09_2_G11_A	253	143	R-67189	*Burkholderia*	*vietnamiensis*
Z0010_LO_G11_1_H06_A	253	143	R-67189	*Burkholderia*	*vietnamiensis*
Z0010_RO_G01_1_H07_A	253	143	R-67189	*Burkholderia*	*vietnamiensis*
Z0010_RO_F07_2_G10_A	253	143	R-67189	*Burkholderia*	*vietnamiensis*

Z0010_LO_G12_1_H06_B	253	143	R-67189	*Burkholderia*	*vietnamiensis*
Z0010_RO_G09_1_H11_A	253	143	R-67189	*Burkholderia*	*vietnamiensis*
Z0010_RO_H04_2_H08_B	253	143	R-67189	*Burkholderia*	*vietnamiensis*
Z0010_RO_A10_1_E11_B	254	141	R-50394	*Burkholderia*	*stabilis*
Z0010_LO_B12_2_E06_B	254	141	R-50394	*Burkholderia*	*stabilis*
Z0010_RO_A12_1_E12_B	254	141	R-50394	*Burkholderia*	*stabilis*
Z0010_RO_A11_1_E12_A	254	141	R-50394	*Burkholderia*	*stabilis*
Z0010_LO_B06_2_E03_B	254	141	R-50394	*Burkholderia*	*stabilis*
Z0010_RO_B03_2_E08_A	254	141	R-50394	*Burkholderia*	*stabilis*
Z0010_LO_B11_2_E06_A	254	141	R-50394	*Burkholderia*	*stabilis*
Z0010_RO_A08_1_E10_B	254	141	R-50394	*Burkholderia*	*stabilis*
Z0010_LO_B03_2_E02_A	254	141	R-50394	*Burkholderia*	*stabilis*
Z0010_RO_B05_2_E09_A	254	141	R-50394	*Burkholderia*	*stabilis*
Z0010_LO_C03_1_F02_A	254	141	R-50394	*Burkholderia*	*stabilis*
Z0010_LO_B05_2_E03_A	254	141	R-50394	*Burkholderia*	*stabilis*
Z0010_LO_B04_2_E02_B	254	141	R-50394	*Burkholderia*	*stabilis*
Z0010_RO_B01_2_E07_A	254	141	R-50394	*Burkholderia*	*stabilis*
Z0010_LO_C02_1_F01_B	254	141	R-50394	*Burkholderia*	*stabilis*
Z0010_RO_B02_2_E07_B	254	141	R-50394	*Burkholderia*	*stabilis*
Z0010_LO_B07_2_E04_A	254	141	R-50394	*Burkholderia*	*stabilis*
Z0010_LO_C01_1_F01_A	254	141	R-50394	*Burkholderia*	*stabilis*
Z0010_LO_C05_1_F03_A	254	141	R-50394	*Burkholderia*	*stabilis*
Z0010_RO_B07_2_E10_A	254	141	R-50394	*Burkholderia*	*stabilis*
Z0010_LO_C04_1_F02_B	254	141	R-50394	*Burkholderia*	*stabilis*
Z0010_LO_B09_2_E05_A	254	141	R-50394	*Burkholderia*	*stabilis*
Z0010_RO_B08_2_E10_B	254	141	R-50394	*Burkholderia*	*stabilis*
Z0010_RO_B04_2_E08_B	255	141	R-50394	*Burkholderia*	*stabilis*
Z0010_RO_B06_2_E09_B	255	141	R-50394	*Burkholderia*	*stabilis*
Z0010_RO_A09_1_E11_A	255	141	R-50394	*Burkholderia*	*stabilis*
Z0010_LO_B10_2_E05_B	255	141	R-50394	*Burkholderia*	*stabilis*
Z0010_LO_B08_2_E04_B	255	141	R-50394	*Burkholderia*	*stabilis*
Z0010_RB_B04_1_D11_B	256	148	R-69596	*Burkholderia*	*cepacia*
Z0010_LB_B04_1_D05_B	256	148	R-69596	*Burkholderia*	*cepacia*
Z0010_RB_B08_1_D09_B	256	148	R-69596	*Burkholderia*	*cepacia*
Z0010_LB_B05_1_D04_A	256	148	R-69596	*Burkholderia*	*cepacia*
Z0010_RB_B12_1_D07_B	256	148	R-69596	*Burkholderia*	*cepacia*
Z0010_RB_C01_2_C12_A	256	148	R-69596	*Burkholderia*	*cepacia*
Z0010_RB_B10_1_D08_B	256	148	R-69596	*Burkholderia*	*cepacia*
Z0010_LB_C03_2_C05_A	256	148	R-69596	*Burkholderia*	*cepacia*
Z0010_LB_C06_2_C04_B	256	148	R-69596	*Burkholderia*	*cepacia*
Z0010_LB_C07_2_C03_A	256	148	R-69596	*Burkholderia*	*cepacia*
Z0010_RB_B05_1_D10_A	256	148	R-69596	*Burkholderia*	*cepacia*
Z0010_RB_B09_1_D08_A	256	148	R-69596	*Burkholderia*	*cepacia*
Z0010_LB_B08_1_D03_B	256	148	R-69596	*Burkholderia*	*cepacia*
Z0010_LB_B12_1_D01_B	256	148	R-69596	*Burkholderia*	*cepacia*
Z0010_RB_B02_1_D12_B	256	148	R-69596	*Burkholderia*	*cepacia*
Z0010_RB_B03_1_D11_A	256	148	R-69596	*Burkholderia*	*cepacia*
Z0010_RB_B11_1_D07_A	256	148	R-69596	*Burkholderia*	*cepacia*
Z0010_LB_B09_1_D02_A	256	148	R-69596	*Burkholderia*	*cepacia*
Z0010_LB_C08_2_C03_B	256	148	R-69596	*Burkholderia*	*cepacia*

Z0010_LB_C05_2_C04_A	256	148	R-69596	*Burkholderia*	*cepacia*
Z0010_LB_B07_1_D03_A	256	148	R-69596	*Burkholderia*	*cepacia*
Z0010_LB_C01_2_C06_A	256	148	R-69596	*Burkholderia*	*cepacia*
Z0010_LB_B02_1_D06_B	256	148	R-69596	*Burkholderia*	*cepacia*
Z0010_LB_B03_1_D05_A	256	148	R-69596	*Burkholderia*	*cepacia*
Z0010_LB_C02_2_C06_B	256	148	R-69596	*Burkholderia*	*cepacia*
Z0010_LB_B06_1_D04_B	256	148	R-69596	*Burkholderia*	*cepacia*
Z0010_LB_C04_2_C05_B	256	148	R-69596	*Burkholderia*	*cepacia*
Z0010_LB_B11_1_D01_A	256	148	R-69596	*Burkholderia*	*cepacia*
Z0010_RB_B01_1_D12_A	256	148	R-69596	*Burkholderia*	*cepacia*
Z0010_RB_B06_1_D10_B	257	148	R-69596	*Burkholderia*	*cepacia*
Z0010_LB_B10_1_D02_B	257	148	R-69596	*Burkholderia*	*cepacia*
Z0010_RB_B07_1_D09_A	257	148	R-69596	*Burkholderia*	*cepacia*
Z0016_RB_D11_1_C07_A	258	46	LMG 22485	*Burkholderia*	*lata*
Z0016_RB_E01_2_B12_A	258	46	LMG 22485	*Burkholderia*	*lata*
Z0016_LB_E04_2_B05_B	258	46	LMG 22485	*Burkholderia*	*lata*
Z0016_RB_D12_1_C07_B	258	46	LMG 22485	*Burkholderia*	*lata*
Z0016_RB_D07_1_C09_A	258	46	LMG 22485	*Burkholderia*	*lata*
Z0016_LB_D09_1_C02_A	258	46	LMG 22485	*Burkholderia*	*lata*
Z0016_LB_D08_1_C03_B	258	46	LMG 22485	*Burkholderia*	*lata*
Z0016_RB_D08_1_C09_B	258	46	LMG 22485	*Burkholderia*	*lata*
Z0016_RB_E02_2_B12_B	258	46	LMG 22485	*Burkholderia*	*lata*
Z0016_LB_E07_2_B03_A	258	46	LMG 22485	*Burkholderia*	*lata*
Z0016_LB_E05_2_B04_A	258	46	LMG 22485	*Burkholderia*	*lata*
Z0016_LB_E06_2_B04_B	258	46	LMG 22485	*Burkholderia*	*lata*
Z0016_RB_E08_2_B09_B	258	46	LMG 22485	*Burkholderia*	*lata*
Z0016_LB_D10_1_C02_B	258	46	LMG 22485	*Burkholderia*	*lata*
Z0016_RB_E04_2_B11_B	258	46	LMG 22485	*Burkholderia*	*lata*
Z0016_LB_E03_2_B05_A	258	46	LMG 22485	*Burkholderia*	*lata*
Z0016_LB_D12_1_C01_B	258	46	LMG 22485	*Burkholderia*	*lata*
Z0016_LB_E02_2_B06_B	258	46	LMG 22485	*Burkholderia*	*lata*
Z0016_LB_E01_2_B06_A	258	46	LMG 22485	*Burkholderia*	*lata*
Z0016_RB_E07_2_B09_A	258	46	LMG 22485	*Burkholderia*	*lata*
Z0016_RB_D10_1_C08_B	258	46	LMG 22485	*Burkholderia*	*lata*
Z0016_LB_D11_1_C01_A	258	46	LMG 22485	*Burkholderia*	*lata*
Z0016_LB_E09_2_B02_A	258	46	LMG 22485	*Burkholderia*	*lata*
Z0016_LB_E08_2_B03_B	258	46	LMG 22485	*Burkholderia*	*lata*
Z0016_RB_E03_2_B11_A	258	46	LMG 22485	*Burkholderia*	*lata*
Z0016_RB_D09_1_C08_A	258	46	LMG 22485	*Burkholderia*	*lata*
Z0016_RB_E05_2_B10_A	258	46	LMG 22485	*Burkholderia*	*lata*
Z0016_RB_E06_2_B10_B	259	46	LMG 22485	*Burkholderia*	*lata*
Z0016_RB_D04_1_C11_B	260	46	LMG 22485	*Burkholderia*	*lata*
Z0016_RB_D05_1_C10_A	260	46	LMG 22485	*Burkholderia*	*lata*
Z0016_LB_D07_1_C03_A	260	46	LMG 22485	*Burkholderia*	*lata*
Z0016_RB_D06_1_C10_B	260	46	LMG 22485	*Burkholderia*	*lata*
Z0010_RO_C03_1_F08_A	261	141	R-67113	*Burkholderia*	*stabilis*
Z0010_LO_C12_1_F06_B	261	141	R-67113	*Burkholderia*	*stabilis*
Z0010_LO_C10_1_F05_B	261	141	R-67113	*Burkholderia*	*stabilis*
Z0010_LO_C09_1_F05_A	261	141	R-67113	*Burkholderia*	*stabilis*
Z0010_RO_B12_2_E12_B	261	141	R-67113	*Burkholderia*	*stabilis*

Z0010_LO_C07_1_F04_A	261	141	R-67113	*Burkholderia*	*stabilis*
Z0010_RO_D07_2_F10_A	261	141	R-67113	*Burkholderia*	*stabilis*
Z0010_LO_E06_1_G03_B	261	141	R-67113	*Burkholderia*	*stabilis*
Z0010_RO_C01_1_F07_A	261	141	R-67113	*Burkholderia*	*stabilis*
Z0010_LO_D02_2_F01_B	261	141	R-67113	*Burkholderia*	*stabilis*
Z0010_LO_C11_1_F06_A	261	141	R-67113	*Burkholderia*	*stabilis*
Z0010_RO_D04_2_F08_B	261	141	R-67113	*Burkholderia*	*stabilis*
Z0010_LO_D03_2_F02_A	261	141	R-67113	*Burkholderia*	*stabilis*
Z0010_LO_D05_2_F03_A	261	141	R-67113	*Burkholderia*	*stabilis*
Z0010_LO_E02_1_G01_B	261	141	R-67113	*Burkholderia*	*stabilis*
Z0010_LO_D12_2_F06_B	261	141	R-67113	*Burkholderia*	*stabilis*
Z0010_RO_C09_1_F11_A	262	141	R-67113	*Burkholderia*	*stabilis*
Z0010_RO_C04_1_F08_B	262	141	R-67113	*Burkholderia*	*stabilis*
Z0010_RO_C07_1_F10_A	262	141	R-67113	*Burkholderia*	*stabilis*
Z0010_LO_D11_2_F06_A	262	141	R-67113	*Burkholderia*	*stabilis*
Z0010_RO_D08_2_F10_B	263	141	R-67113	*Burkholderia*	*stabilis*
Z0010_LO_C08_1_F04_B	263	141	R-67113	*Burkholderia*	*stabilis*
Z0010_RO_C02_1_F07_B	263	141	R-67113	*Burkholderia*	*stabilis*
Z0010_LO_D09_2_F05_A	263	141	R-67113	*Burkholderia*	*stabilis*
Z0010_RO_C11_1_F12_A	264	141	R-67113	*Burkholderia*	*stabilis*
Z0010_LO_D07_2_F04_A	264	141	R-67113	*Burkholderia*	*stabilis*
Z0010_RO_D06_2_F09_B	264	141	R-67113	*Burkholderia*	*stabilis*
Z0010_RO_B10_2_E11_B	264	141	R-67113	*Burkholderia*	*stabilis*
Z0010_LO_C06_1_F03_B	264	141	R-67113	*Burkholderia*	*stabilis*
Z0010_RO_D01_2_F07_A	264	141	R-67113	*Burkholderia*	*stabilis*
Z0010_RO_B09_2_E11_A	264	141	R-67113	*Burkholderia*	*stabilis*
Z0010_RO_B11_2_E12_A	264	141	R-67113	*Burkholderia*	*stabilis*
Z0010_LO_A06_1_E03_B	265	142	R-71089	*Burkholderia*	*multivorans*
Z0010_LO_B02_2_E01_B	265	142	R-71089	*Burkholderia*	*multivorans*
Z0010_RO_E01_1_G07_A	265	144	R-67196	*Burkholderia*	*multivorans*
Z0010_RO_A06_1_E09_B	266	142	R-71089	*Burkholderia*	*multivorans*
Z0010_RO_A05_1_E09_A	266	142	R-71089	*Burkholderia*	*multivorans*
Z0010_LO_B01_2_E01_A	266	142	R-71089	*Burkholderia*	*multivorans*
Z0010_RO_A04_1_E08_B	266	142	R-71089	*Burkholderia*	*multivorans*
Z0010_RO_A07_1_E10_A	266	142	R-71089	*Burkholderia*	*multivorans*
Z0010_RB_H06_1_A10_B	266	142	R-71089	*Burkholderia*	*multivorans*
Z0010_LO_A02_1_E01_B	266	142	R-71089	*Burkholderia*	*multivorans*
Z0010_RO_E08_1_G10_B	266	142	R-67121	*Burkholderia*	*multivorans*
Z0010_LO_F04_2_G02_B	266	142	R-67121	*Burkholderia*	*multivorans*
Z0010_RO_E12_1_G12_B	266	144	R-67196	*Burkholderia*	*multivorans*
Z0010_LO_G03_1_H02_A	266	144	R-67196	*Burkholderia*	*multivorans*
Z0010_RO_E06_1_G09_B	266	144	R-67196	*Burkholderia*	*multivorans*
Z0010_RO_D02_2_F07_B	266	144	R-67196	*Burkholderia*	*multivorans*
Z0010_RB_H10_1_A08_B	266	142	R-71089	*Burkholderia*	*multivorans*
Z0010_RO_E07_1_G10_A	266	144	R-67196	*Burkholderia*	*multivorans*
Z0010_LO_E04_1_G02_B	266	144	R-67196	*Burkholderia*	*multivorans*
Z0010_LO_F08_2_G04_B	266	144	R-67196	*Burkholderia*	*multivorans*
Z0010_RO_D03_2_F08_A	266	144	R-67196	*Burkholderia*	*multivorans*
Z0010_LO_E03_1_G02_A	266	144	R-67196	*Burkholderia*	*multivorans*
Z0010_RO_F03_2_G08_A	266	144	R-67196	*Burkholderia*	*multivorans*

Z0010_LO_E12_1_G06_B	266	144	R-67196	*Burkholderia*	*multivorans*
Z0010_LO_F07_2_G04_A	266	144	R-67196	*Burkholderia*	*multivorans*
Z0010_RO_D05_2_F09_A	266	144	R-67196	*Burkholderia*	*multivorans*
Z0010_LO_E09_1_G05_A	266	144	R-67196	*Burkholderia*	*multivorans*
Z0010_LO_A11_1_E06_A	267	142	R-71089	*Burkholderia*	*multivorans*
Z0010_LO_A10_1_E05_B	267	142	R-71089	*Burkholderia*	*multivorans*
Z0010_LO_F10_2_G05_B	267	144	R-67196	*Burkholderia*	*multivorans*
Z0010_RO_A01_1_E07_A	268	142	R-71089	*Burkholderia*	*multivorans*
Z0010_LO_A07_1_E04_A	268	142	R-71089	*Burkholderia*	*multivorans*
Z0010_RB_H07_1_A09_A	268	142	R-71089	*Burkholderia*	*multivorans*
Z0010_LO_A08_1_E04_B	268	142	R-71089	*Burkholderia*	*multivorans*
Z0010_LO_A03_1_E02_A	268	142	R-71089	*Burkholderia*	*multivorans*
Z0010_LO_A04_1_E02_B	268	142	R-71089	*Burkholderia*	*multivorans*
Z0010_LO_A05_1_E03_A	268	142	R-71089	*Burkholderia*	*multivorans*
Z0010_RB_H11_1_A07_A	268	142	R-71089	*Burkholderia*	*multivorans*
Z0010_LO_D08_2_F04_B	268	144	R-67196	*Burkholderia*	*multivorans*
Z0010_LO_E05_1_G03_A	268	144	R-67196	*Burkholderia*	*multivorans*
Z0010_LO_D10_2_F05_B	268	144	R-67196	*Burkholderia*	*multivorans*
Z0010_LO_A12_1_E06_B	268	142	R-71089	*Burkholderia*	*multivorans*
Z0010_RO_F04_2_G08_B	268	142	R-67121	*Burkholderia*	*multivorans*
Z0010_LO_F12_2_G06_B	268	142	R-67121	*Burkholderia*	*multivorans*
Z0010_RO_F06_2_G09_B	268	142	R-67121	*Burkholderia*	*multivorans*
Z0010_LO_G07_1_H04_A	269	144	R-67196	*Burkholderia*	*multivorans*
Z0010_LO_G09_1_H05_A	269	144	R-67196	*Burkholderia*	*multivorans*
Z0010_LO_G08_1_H04_B	269	144	R-67196	*Burkholderia*	*multivorans*
Z0010_LO_F02_2_G01_B	269	144	R-67196	*Burkholderia*	*multivorans*
Z0010_LO_E01_1_G01_A	269	144	R-67196	*Burkholderia*	*multivorans*
Z0010_RO_E04_1_G08_B	269	144	R-67196	*Burkholderia*	*multivorans*
Z0010_LO_F05_2_G03_A	269	144	R-67196	*Burkholderia*	*multivorans*
Z0010_RO_D11_2_F12_A	269	144	R-67196	*Burkholderia*	*multivorans*
Z0010_RO_D09_2_F11_A	269	144	R-67196	*Burkholderia*	*multivorans*
Z0010_LO_E11_1_G06_A	269	144	R-67196	*Burkholderia*	*multivorans*
Z0010_RO_D10_2_F11_B	269	144	R-67196	*Burkholderia*	*multivorans*
Z0010_RO_E09_1_G11_A	269	144	R-67196	*Burkholderia*	*multivorans*
Z0010_RB_H12_1_A07_B	269	142	R-71089	*Burkholderia*	*multivorans*
Z0010_RB_H08_1_A09_B	269	142	R-71089	*Burkholderia*	*multivorans*
Z0010_RO_A03_1_E08_A	269	142	R-71089	*Burkholderia*	*multivorans*
Z0010_RO_A02_1_E07_B	269	142	R-71089	*Burkholderia*	*multivorans*
Z0010_RB_H09_1_A08_A	269	142	R-71089	*Burkholderia*	*multivorans*
Z0010_RB_H05_1_A10_A	269	142	R-71089	*Burkholderia*	*multivorans*
Z0010_LO_E07_1_G04_A	270	142	R-67121	*Burkholderia*	*multivorans*
Z0010_LO_E10_1_G05_B	270	142	R-67121	*Burkholderia*	*multivorans*
Z0010_LO_E08_1_G04_B	270	142	R-67121	*Burkholderia*	*multivorans*
Z0010_RO_F11_2_G12_A	270	142	R-67121	*Burkholderia*	*multivorans*
Z0010_LO_G04_1_H02_B	270	142	R-67121	*Burkholderia*	*multivorans*
Z0010_LO_G10_1_H05_B	270	142	R-67121	*Burkholderia*	*multivorans*
Z0010_LO_F03_2_G02_A	270	142	R-67121	*Burkholderia*	*multivorans*
Z0010_LO_F06_2_G03_B	270	142	R-67121	*Burkholderia*	*multivorans*
Z0010_LO_G05_1_H03_A	270	142	R-67121	*Burkholderia*	*multivorans*
Z0010_LO_G06_1_H03_B	270	142	R-67121	*Burkholderia*	*multivorans*

Z0010_LO_F11_2_G06_A	270	142	R-67121	*Burkholderia*	*multivorans*
Z0010_RO_F01_2_G07_A	270	142	R-67121	*Burkholderia*	*multivorans*
Z0010_RO_F10_2_G11_B	270	142	R-67121	*Burkholderia*	*multivorans*
Z0010_RO_F12_2_G12_B	270	142	R-67121	*Burkholderia*	*multivorans*
Z0010_RO_E03_1_G08_A	270	142	R-67121	*Burkholderia*	*multivorans*
Z0010_LO_F01_2_G01_A	270	142	R-67121	*Burkholderia*	*multivorans*
Z0010_RO_E10_1_G11_B	270	142	R-67121	*Burkholderia*	*multivorans*
Z0010_LO_G01_1_H01_A	271	144	R-67196	*Burkholderia*	*multivorans*
Z0011_LO_H09_2_H05_A	272	144	R-67258	*Burkholderia*	*multivorans*
Z0011_LB_A10_2_D02_B	272	144	R-67258	*Burkholderia*	*multivorans*
Z0011_LO_H10_2_H05_B	272	144	R-67258	*Burkholderia*	*multivorans*
Z0011_LB_A09_2_D02_A	272	144	R-67258	*Burkholderia*	*multivorans*
Z0011_LO_D03_2_F02_A	273	144	R-67258	*Burkholderia*	*multivorans*
Z0011_RB_B07_1_D09_A	274	142	R-67536	*Burkholderia*	*multivorans*
Z0011_LB_D04_1_C05_B	274	142	R-67536	*Burkholderia*	*multivorans*
Z0011_LB_D05_1_C04_A	274	142	R-67536	*Burkholderia*	*multivorans*
Z0011_LB_E04_2_B05_B	274	142	R-67536	*Burkholderia*	*multivorans*
Z0011_LB_E05_2_B04_A	274	142	R-67536	*Burkholderia*	*multivorans*
Z0011_RB_B05_1_D10_A	274	142	R-67536	*Burkholderia*	*multivorans*
Z0011_LB_D01_1_C06_A	274	142	R-67536	*Burkholderia*	*multivorans*
Z0011_LB_D03_1_C05_A	274	142	R-67536	*Burkholderia*	*multivorans*
Z0011_RB_B06_1_D10_B	274	142	R-67536	*Burkholderia*	*multivorans*
Z0011_LB_D02_1_C06_B	274	142	R-67536	*Burkholderia*	*multivorans*
Z0011_RB_C02_2_C12_B	274	142	R-67536	*Burkholderia*	*multivorans*
Z0011_LB_E01_2_B06_A	274	142	R-67536	*Burkholderia*	*multivorans*
Z0011_RB_C03_2_C11_A	274	142	R-67536	*Burkholderia*	*multivorans*
Z0011_LB_E03_2_B05_A	274	142	R-67536	*Burkholderia*	*multivorans*
Z0011_LB_D12_1_C01_B	274	142	R-67536	*Burkholderia*	*multivorans*
Z0011_LB_D11_1_C01_A	274	142	R-67536	*Burkholderia*	*multivorans*
Z0011_RB_B11_1_D07_A	274	142	R-67536	*Burkholderia*	*multivorans*
Z0011_RB_B12_1_D07_B	274	142	R-67536	*Burkholderia*	*multivorans*
Z0011_RB_C01_2_C12_A	274	142	R-67536	*Burkholderia*	*multivorans*
Z0011_LB_E02_2_B06_B	274	142	R-67536	*Burkholderia*	*multivorans*
Z0011_RB_C04_2_C11_B	274	142	R-67536	*Burkholderia*	*multivorans*
Z0011_LB_E06_2_B04_B	274	142	R-67536	*Burkholderia*	*multivorans*
Z0011_RB_C05_2_C10_A	274	142	R-67536	*Burkholderia*	*multivorans*
Z0011_RB_C06_2_C10_B	274	142	R-67536	*Burkholderia*	*multivorans*
Z0011_RB_B08_1_D09_B	274	142	R-67536	*Burkholderia*	*multivorans*
Z0011_RB_B09_1_D08_A	274	142	R-67536	*Burkholderia*	*multivorans*
Z0011_LB_D06_1_C04_B	274	142	R-67536	*Burkholderia*	*multivorans*
Z0011_LB_D07_1_C03_A	274	142	R-67536	*Burkholderia*	*multivorans*
Z0011_RB_B10_1_D08_B	274	142	R-67536	*Burkholderia*	*multivorans*
Z0011_LB_D09_1_C02_A	274	142	R-67536	*Burkholderia*	*multivorans*
Z0011_LB_D10_1_C02_B	274	142	R-67536	*Burkholderia*	*multivorans*
Z0011_LB_D08_1_C03_B	274	142	R-67536	*Burkholderia*	*multivorans*
Z0011_LO_H12_2_H06_B	275	144	R-67258	*Burkholderia*	*multivorans*
Z0011_LB_A11_2_D01_A	275	144	R-67258	*Burkholderia*	*multivorans*
Z0011_LO_H04_2_H02_B	275	144	R-67258	*Burkholderia*	*multivorans*
Z0011_RO_B07_2_E10_A	275	144	R-67258	*Burkholderia*	*multivorans*
Z0011_LB_A01_2_D06_A	275	144	R-67258	*Burkholderia*	*multivorans*

Z0011_RO_B03_2_E08_A	275	144	R-67258	*Burkholderia*	*multivorans*
Z0011_LO_H05_2_H03_A	275	144	R-67258	*Burkholderia*	*multivorans*
Z0011_LB_A02_2_D06_B	275	144	R-67258	*Burkholderia*	*multivorans*
Z0011_LB_A03_2_D05_A	275	144	R-67258	*Burkholderia*	*multivorans*
Z0011_LO_H11_2_H06_A	275	144	R-67258	*Burkholderia*	*multivorans*
Z0011_RB_A01_2_D12_A	275	144	R-67258	*Burkholderia*	*multivorans*
Z0011_LB_B01_1_D06_A	275	144	R-67258	*Burkholderia*	*multivorans*
Z0011_LB_B02_1_D06_B	275	144	R-67258	*Burkholderia*	*multivorans*
Z0011_RB_A02_2_D12_B	275	144	R-67258	*Burkholderia*	*multivorans*
Z0011_LB_A12_2_D01_B	275	144	R-67258	*Burkholderia*	*multivorans*
Z0011_RB_A03_2_D11_A	275	144	R-67258	*Burkholderia*	*multivorans*
Z0011_LB_B05_1_D04_A	275	144	R-67258	*Burkholderia*	*multivorans*
Z0011_LB_B04_1_D05_B	275	144	R-67258	*Burkholderia*	*multivorans*
Z0011_LB_B03_1_D05_A	275	144	R-67258	*Burkholderia*	*multivorans*
Z0011_LB_A07_2_D03_A	275	144	R-67258	*Burkholderia*	*multivorans*
Z0011_LB_A06_2_D04_B	275	144	R-67258	*Burkholderia*	*multivorans*
Z0011_LB_A05_2_D04_A	275	144	R-67258	*Burkholderia*	*multivorans*
Z0011_LO_H07_2_H04_A	275	144	R-67258	*Burkholderia*	*multivorans*
Z0011_LO_H08_2_H04_B	275	144	R-67258	*Burkholderia*	*multivorans*
Z0011_LB_A08_2_D03_B	275	144	R-67258	*Burkholderia*	*multivorans*
Z0011_LO_H06_2_H03_B	275	144	R-67258	*Burkholderia*	*multivorans*
Z0011_LB_A04_2_D05_B	275	144	R-67258	*Burkholderia*	*multivorans*
Z0010_RO_E11_1_G12_A	276	142	R-67121	*Burkholderia*	*multivorans*
Z0010_LO_F09_2_G05_A	276	142	R-67121	*Burkholderia*	*multivorans*
Z0010_RO_E05_1_G09_A	276	142	R-67121	*Burkholderia*	*multivorans*
Z0010_RO_E02_1_G07_B	276	142	R-67121	*Burkholderia*	*multivorans*
Z0010_LO_G02_1_H01_B	276	142	R-67121	*Burkholderia*	*multivorans*
Z0010_LO_A09_1_E05_A	276	142	R-71089	*Burkholderia*	*multivorans*
Z0010_RO_G04_1_H08_B	277	142	R-67121	*Burkholderia*	*multivorans*
Z0010_RO_G05_1_H09_A	277	142	R-67121	*Burkholderia*	*multivorans*
Z0010_RO_G03_1_H08_A	277	142	R-67121	*Burkholderia*	*multivorans*
Z0010_RO_G02_1_H07_B	278	142	R-67121	*Burkholderia*	*multivorans*
Z0010_RO_D12_2_F12_B	278	142	R-67121	*Burkholderia*	*multivorans*
Z0010_LB_D12_1_C01_B	279	149	R-71006	*Burkholderia*	*multivorans*
Z0010_LB_E01_2_B06_A	279	149	R-71006	*Burkholderia*	*multivorans*
Z0010_RB_D03_1_C11_A	279	149	R-71006	*Burkholderia*	*multivorans*
Z0010_RB_D01_1_C12_A	279	149	R-71006	*Burkholderia*	*multivorans*
Z0010_RB_D02_1_C12_B	279	149	R-71006	*Burkholderia*	*multivorans*
Z0010_LB_C10_2_C02_B	279	149	R-71006	*Burkholderia*	*multivorans*
Z0010_LB_C12_2_C01_B	279	149	R-71006	*Burkholderia*	*multivorans*
Z0010_LB_E02_2_B06_B	279	149	R-71006	*Burkholderia*	*multivorans*
Z0010_LB_D05_1_C04_A	280	149	R-71006	*Burkholderia*	*multivorans*
Z0010_RB_C05_2_C10_A	281	149	R-71006	*Burkholderia*	*multivorans*
Z0010_RB_C04_2_C11_B	281	149	R-71006	*Burkholderia*	*multivorans*
Z0010_RB_C06_2_C10_B	281	149	R-71006	*Burkholderia*	*multivorans*
Z0010_LB_D10_1_C02_B	281	149	R-71006	*Burkholderia*	*multivorans*
Z0010_LB_C09_2_C02_A	281	149	R-71006	*Burkholderia*	*multivorans*
Z0010_RB_C11_2_C07_A	281	149	R-71006	*Burkholderia*	*multivorans*
Z0010_LB_D09_1_C02_A	281	149	R-71006	*Burkholderia*	*multivorans*
Z0010_RB_C09_2_C08_A	281	149	R-71006	*Burkholderia*	*multivorans*

Z0010_LB_C11_2_C01_A	281	149	R-71006	*Burkholderia*	*multivorans*
Z0010_RB_C02_2_C12_B	281	149	R-71006	*Burkholderia*	*multivorans*
Z0010_LB_D01_1_C06_A	281	149	R-71006	*Burkholderia*	*multivorans*
Z0010_LB_D02_1_C06_B	281	149	R-71006	*Burkholderia*	*multivorans*
Z0010_LB_D08_1_C03_B	281	149	R-71006	*Burkholderia*	*multivorans*
Z0010_RB_C03_2_C11_A	281	149	R-71006	*Burkholderia*	*multivorans*
Z0010_RB_C07_2_C09_A	281	149	R-71006	*Burkholderia*	*multivorans*
Z0010_RB_C08_2_C09_B	281	149	R-71006	*Burkholderia*	*multivorans*
Z0010_LB_D04_1_C05_B	281	149	R-71006	*Burkholderia*	*multivorans*
Z0010_LB_D03_1_C05_A	281	149	R-71006	*Burkholderia*	*multivorans*
Z0010_LB_D06_1_C04_B	281	149	R-71006	*Burkholderia*	*multivorans*
Z0010_LB_D07_1_C03_A	281	149	R-71006	*Burkholderia*	*multivorans*
Z0010_LB_D11_1_C01_A	281	149	R-71006	*Burkholderia*	*multivorans*
Z0010_RB_C12_2_C07_B	281	149	R-71006	*Burkholderia*	*multivorans*
Z0010_RB_C10_2_C08_B	281	149	R-71006	*Burkholderia*	*multivorans*
Z0011_RO_B05_2_E09_A	282	142	R-68768	*Burkholderia*	*multivorans*
Z0011_LO_B10_2_E05_B	282	142	R-68768	*Burkholderia*	*multivorans*
Z0011_LO_C01_1_F01_A	282	142	R-68768	*Burkholderia*	*multivorans*
Z0011_LO_B07_2_E04_A	282	142	R-68768	*Burkholderia*	*multivorans*
Z0011_RO_A09_1_E11_A	282	142	R-68768	*Burkholderia*	*multivorans*
Z0011_RO_A11_1_E12_A	282	142	R-68768	*Burkholderia*	*multivorans*
Z0011_LO_B08_2_E04_B	282	142	R-68768	*Burkholderia*	*multivorans*
Z0011_RO_A03_1_E08_A	282	142	R-68768	*Burkholderia*	*multivorans*
Z0011_RO_A05_1_E09_A	282	142	R-68768	*Burkholderia*	*multivorans*
Z0011_LO_B11_2_E06_A	282	142	R-68768	*Burkholderia*	*multivorans*
Z0011_RO_B02_2_E07_B	282	142	R-68768	*Burkholderia*	*multivorans*
Z0011_LO_C02_1_F01_B	282	142	R-68768	*Burkholderia*	*multivorans*
Z0011_RO_A12_1_E12_B	282	142	R-68768	*Burkholderia*	*multivorans*
Z0011_RO_B01_2_E07_A	282	142	R-68768	*Burkholderia*	*multivorans*
Z0011_RO_A07_1_E10_A	282	142	R-68768	*Burkholderia*	*multivorans*
Z0011_RO_A10_1_E11_B	282	142	R-68768	*Burkholderia*	*multivorans*
Z0011_RO_A08_1_E10_B	282	142	R-68768	*Burkholderia*	*multivorans*
Z0011_RO_A02_1_E07_B	282	142	R-68768	*Burkholderia*	*multivorans*
Z0011_RO_A06_1_E09_B	282	142	R-68768	*Burkholderia*	*multivorans*
Z0011_RB_H11_1_A07_A	282	142	R-68768	*Burkholderia*	*multivorans*
Z0011_RO_A01_1_E07_A	282	142	R-68768	*Burkholderia*	*multivorans*
Z0011_LO_B09_2_E05_A	282	142	R-68768	*Burkholderia*	*multivorans*
Z0011_LO_B12_2_E06_B	282	142	R-68768	*Burkholderia*	*multivorans*
Z0011_LO_C04_1_F02_B	282	142	R-68768	*Burkholderia*	*multivorans*
Z0011_LO_C03_1_F02_A	282	142	R-68768	*Burkholderia*	*multivorans*
Z0011_RO_B04_2_E08_B	282	142	R-68768	*Burkholderia*	*multivorans*
Z0011_RO_A04_1_E08_B	282	142	R-68768	*Burkholderia*	*multivorans*
Z0011_RB_H12_1_A07_B	282	142	R-68768	*Burkholderia*	*multivorans*
Z0015_RB_F04_1_B11_B	283	38	LMG 21530	*Massilia*	*timonae*
Z0015_LB_H01_1_A06_A	283	38	LMG 21530	*Massilia*	*timonae*
Z0015_LB_H02_1_A06_B	283	38	LMG 21530	*Massilia*	*timonae*
Z0015_LB_G12_2_A01_B	283	38	LMG 21530	*Massilia*	*timonae*
Z0015_RB_G01_2_A12_A	283	38	LMG 21530	*Massilia*	*timonae*
Z0015_RB_G02_2_A12_B	283	38	LMG 21530	*Massilia*	*timonae*
Z0015_LB_H07_1_A03_A	283	38	LMG 21530	*Massilia*	*timonae*

Z0015_LB_H08_1_A03_B	283	38	LMG 21530	*Massilia*	*timonae*	
Z0015_RB_F12_1_B07_B	284	38	LMG 21530	*Massilia*	*timonae*	
Z0015_LB_H06_1_A04_B	284	38	LMG 21530	*Massilia*	*timonae*	
Z0015_RB_F10_1_B08_B	284	38	LMG 21530	*Massilia*	*timonae*	
Z0015_LB_H04_1_A05_B	284	38	LMG 21530	*Massilia*	*timonae*	
Z0015_RB_F09_1_B08_A	284	38	LMG 21530	*Massilia*	*timonae*	
Z0015_LB_H03_1_A05_A	284	38	LMG 21530	*Massilia*	*timonae*	
Z0015_RB_F06_1_B10_B	284	38	LMG 21530	*Massilia*	*timonae*	
Z0015_RB_F08_1_B09_B	284	38	LMG 21530	*Massilia*	*timonae*	
Z0015_RB_F07_1_B09_A	284	38	LMG 21530	*Massilia*	*timonae*	
Z0015_RB_F05_1_B10_A	284	38	LMG 21530	*Massilia*	*timonae*	
Z0015_RB_F11_1_B07_A	284	38	LMG 21530	*Massilia*	*timonae*	
Z0015_LB_H05_1_A04_A	284	38	LMG 21530	*Massilia*	*timonae*	
Z0015_RB_G09_2_A08_A	284	38	LMG 21530	*Massilia*	*timonae*	
Z0015_RB_G10_2_A08_B	284	38	LMG 21530	*Massilia*	*timonae*	
Z0015_RB_G11_2_A07_A	284	38	LMG 21530	*Massilia*	*timonae*	
Z0015_RB_G12_2_A07_B	284	38	LMG 21530	*Massilia*	*timonae*	
Z0015_RB_G06_2_A10_B	284	38	LMG 21530	*Massilia*	*timonae*	
Z0015_RB_G05_2_A10_A	284	38	LMG 21530	*Massilia*	*timonae*	
Z0015_RB_G07_2_A09_A	284	38	LMG 21530	*Massilia*	*timonae*	
Z0015_RB_G08_2_A09_B	284	38	LMG 21530	*Massilia*	*timonae*	
Z0015_LB_H09_1_A02_A	285	38	LMG 21530	*Massilia*	*timonae*	
Z0015_LB_H10_1_A02_B	285	38	LMG 21530	*Massilia*	*timonae*	
Z0015_RB_G04_2_A11_B	285	38	LMG 21530	*Massilia*	*timonae*	
Z0015_RB_G03_2_A11_A	285	38	LMG 21530	*Massilia*	*timonae*	
Z0025_RB_G07_2_A09_A	286	136	LMG 7899	*Yersinia*	*enterocolitica*	*enterocolitica*
Z0025_LB_F01_1_B06_A	286	136	LMG 7899	*Yersinia*	*enterocolitica*	*enterocolitica*
Z0025_RB_G03_2_A11_A	286	136	LMG 7899	*Yersinia*	*enterocolitica*	*enterocolitica*
Z0025_LB_F05_1_B04_A	286	136	LMG 7899	*Yersinia*	*enterocolitica*	*enterocolitica*
Z0025_LB_F06_1_B04_B	286	136	LMG 7899	*Yersinia*	*enterocolitica*	*enterocolitica*
Z0025_LB_F07_1_B03_A	286	136	LMG 7899	*Yersinia*	*enterocolitica*	*enterocolitica*
Z0025_LB_E11_2_B01_A	286	136	LMG 7899	*Yersinia*	*enterocolitica*	*enterocolitica*
Z0025_RB_G02_2_A12_B	286	136	LMG 7899	*Yersinia*	*enterocolitica*	*enterocolitica*
Z0025_LB_G04_2_A05_B	286	136	LMG 7899	*Yersinia*	*enterocolitica*	*enterocolitica*
Z0025_LB_F02_1_B06_B	286	136	LMG 7899	*Yersinia*	*enterocolitica*	*enterocolitica*
Z0025_LB_F09_1_B02_A	286	136	LMG 7899	*Yersinia*	*enterocolitica*	*enterocolitica*
Z0025_LB_G03_2_A05_A	286	136	LMG 7899	*Yersinia*	*enterocolitica*	*enterocolitica*
Z0025_LB_F12_1_B01_B	286	136	LMG 7899	*Yersinia*	*enterocolitica*	*enterocolitica*
Z0025_LB_G02_2_A06_B	286	136	LMG 7899	*Yersinia*	*enterocolitica*	*enterocolitica*
Z0025_LB_G07_2_A03_A	286	136	LMG 7899	*Yersinia*	*enterocolitica*	*enterocolitica*
Z0025_LB_F10_1_B02_B	286	136	LMG 7899	*Yersinia*	*enterocolitica*	*enterocolitica*
Z0025_LB_G06_2_A04_B	286	136	LMG 7899	*Yersinia*	*enterocolitica*	*enterocolitica*
Z0025_LB_G01_2_A06_A	286	136	LMG 7899	*Yersinia*	*enterocolitica*	*enterocolitica*
Z0025_RB_G01_2_A12_A	286	136	LMG 7899	*Yersinia*	*enterocolitica*	*enterocolitica*
Z0025_RB_G05_2_A10_A	286	136	LMG 7899	*Yersinia*	*enterocolitica*	*enterocolitica*
Z0025_LB_F08_1_B03_B	286	136	LMG 7899	*Yersinia*	*enterocolitica*	*enterocolitica*
Z0025_RB_F12_1_B07_B	286	136	LMG 7899	*Yersinia*	*enterocolitica*	*enterocolitica*
Z0025_RB_F10_1_B08_B	286	136	LMG 7899	*Yersinia*	*enterocolitica*	*enterocolitica*
Z0025_LB_E10_2_B02_B	286	136	LMG 7899	*Yersinia*	*enterocolitica*	*enterocolitica*
Z0025_RB_G04_2_A11_B	286	136	LMG 7899	*Yersinia*	*enterocolitica*	*enterocolitica*

Z0025_LB_E12_2_B01_B	286	136	LMG 7899	*Yersinia*	*enterocolitica*	*enterocolitica*
Z0025_RB_F11_1_B07_A	286	136	LMG 7899	*Yersinia*	*enterocolitica*	*enterocolitica*
Z0025_LB_F03_1_B05_A	286	136	LMG 7899	*Yersinia*	*enterocolitica*	*enterocolitica*
Z0025_LB_F11_1_B01_A	286	136	LMG 7899	*Yersinia*	*enterocolitica*	*enterocolitica*
Z0025_LB_F04_1_B05_B	286	136	LMG 7899	*Yersinia*	*enterocolitica*	*enterocolitica*
Z0025_LB_G05_2_A04_A	286	136	LMG 7899	*Yersinia*	*enterocolitica*	*enterocolitica*
Z0025_RB_G06_2_A10_B	287	136	LMG 7899	*Yersinia*	*enterocolitica*	*enterocolitica*
Z0017_RO_D08_2_F10_B	288	65	LMG 2404	*Pectobacterium*	*carotovorum*	*carotovorum*
Z0017_LO_E01_1_G01_A	288	65	LMG 2404	*Pectobacterium*	*carotovorum*	*carotovorum*
Z0017_LO_D09_2_F05_A	288	65	LMG 2404	*Pectobacterium*	*carotovorum*	*carotovorum*
Z0017_RO_F02_2_G07_B	289	65	LMG 2404	*Pectobacterium*	*carotovorum*	*carotovorum*
Z0017_LO_D10_2_F05_B	290	65	LMG 2404	*Pectobacterium*	*carotovorum*	*carotovorum*
Z0017_RO_D05_2_F09_A	291	65	LMG 2404	*Pectobacterium*	*carotovorum*	*carotovorum*
Z0017_RO_D02_2_F07_B	291	65	LMG 2404	*Pectobacterium*	*carotovorum*	*carotovorum*
Z0017_LO_D07_2_F04_A	291	65	LMG 2404	*Pectobacterium*	*carotovorum*	*carotovorum*
Z0017_RO_E06_1_G09_B	291	65	LMG 2404	*Pectobacterium*	*carotovorum*	*carotovorum*
Z0017_LO_E11_1_G06_A	291	65	LMG 2404	*Pectobacterium*	*carotovorum*	*carotovorum*
Z0017_RO_E07_1_G10_A	291	65	LMG 2404	*Pectobacterium*	*carotovorum*	*carotovorum*
Z0017_RO_E01_1_G07_A	291	65	LMG 2404	*Pectobacterium*	*carotovorum*	*carotovorum*
Z0017_LO_E06_1_G03_B	291	65	LMG 2404	*Pectobacterium*	*carotovorum*	*carotovorum*
Z0017_RO_D10_2_F11_B	291	65	LMG 2404	*Pectobacterium*	*carotovorum*	*carotovorum*
Z0017_LO_E04_1_G02_B	291	65	LMG 2404	*Pectobacterium*	*carotovorum*	*carotovorum*
Z0017_RO_E03_1_G08_A	291	65	LMG 2404	*Pectobacterium*	*carotovorum*	*carotovorum*
Z0017_LO_D04_2_F02_B	291	65	LMG 2404	*Pectobacterium*	*carotovorum*	*carotovorum*
Z0017_RO_E10_1_G11_B	291	65	LMG 2404	*Pectobacterium*	*carotovorum*	*carotovorum*
Z0017_LO_E10_1_G05_B	291	65	LMG 2404	*Pectobacterium*	*carotovorum*	*carotovorum*
Z0017_RO_F08_2_G10_B	291	65	LMG 2404	*Pectobacterium*	*carotovorum*	*carotovorum*
Z0017_RO_F07_2_G10_A	291	65	LMG 2404	*Pectobacterium*	*carotovorum*	*carotovorum*
Z0017_RO_F06_2_G09_B	291	65	LMG 2404	*Pectobacterium*	*carotovorum*	*carotovorum*
Z0017_RO_E12_1_G12_B	291	65	LMG 2404	*Pectobacterium*	*carotovorum*	*carotovorum*
Z0017_LO_F06_2_G03_B	291	65	LMG 2404	*Pectobacterium*	*carotovorum*	*carotovorum*
Z0017_RO_E09_1_G11_A	291	65	LMG 2404	*Pectobacterium*	*carotovorum*	*carotovorum*
Z0017_LO_E08_1_G04_B	291	65	LMG 2404	*Pectobacterium*	*carotovorum*	*carotovorum*
Z0017_RO_F11_2_G12_A	291	65	LMG 2404	*Pectobacterium*	*carotovorum*	*carotovorum*
Z0017_LO_F08_2_G04_B	291	65	LMG 2404	*Pectobacterium*	*carotovorum*	*carotovorum*
Z0017_LO_F03_2_G02_A	291	65	LMG 2404	*Pectobacterium*	*carotovorum*	*carotovorum*
Z0017_RO_F09_2_G11_A	291	65	LMG 2404	*Pectobacterium*	*carotovorum*	*carotovorum*
Z0017_LO_F05_2_G03_A	291	65	LMG 2404	*Pectobacterium*	*carotovorum*	*carotovorum*
Z0017_LO_E09_1_G05_A	291	65	LMG 2404	*Pectobacterium*	*carotovorum*	*carotovorum*
Z0015_RB_E01_2_B12_A	292	37	LMG 21371	*Erwinia*	*carotovora*	
Z0015_LB_F07_1_B03_A	292	37	LMG 21371	*Erwinia*	*carotovora*	
Z0015_LB_G11_2_A01_A	292	37	LMG 21371	*Erwinia*	*carotovora*	
Z0015_LB_F08_1_B03_B	292	37	LMG 21371	*Erwinia*	*carotovora*	
Z0015_LB_G05_2_A04_A	292	37	LMG 21371	*Erwinia*	*carotovora*	
Z0015_LB_G07_2_A03_A	292	37	LMG 21371	*Erwinia*	*carotovora*	
Z0015_LB_F11_1_B01_A	292	37	LMG 21371	*Erwinia*	*carotovora*	
Z0015_RB_E03_2_B11_A	292	37	LMG 21371	*Erwinia*	*carotovora*	
Z0015_LB_G01_2_A06_A	292	37	LMG 21371	*Erwinia*	*carotovora*	
Z0015_LB_F12_1_B01_B	292	37	LMG 21371	*Erwinia*	*carotovora*	
Z0015_LB_F09_1_B02_A	292	37	LMG 21371	*Erwinia*	*carotovora*	

Z0015_LB_G04_2_A05_B	292	37	LMG 21371	*Erwinia*	*carotovora*
Z0015_RB_E11_2_B07_A	292	37	LMG 21371	*Erwinia*	*carotovora*
Z0015_RB_F02_1_B12_B	292	37	LMG 21371	*Erwinia*	*carotovora*
Z0015_RB_F03_1_B11_A	292	37	LMG 21371	*Erwinia*	*carotovora*
Z0015_RB_E07_2_B09_A	292	37	LMG 21371	*Erwinia*	*carotovora*
Z0015_LB_G03_2_A05_A	292	37	LMG 21371	*Erwinia*	*carotovora*
Z0015_LB_G02_2_A06_B	292	37	LMG 21371	*Erwinia*	*carotovora*
Z0015_RB_E06_2_B10_B	292	37	LMG 21371	*Erwinia*	*carotovora*
Z0015_RB_E05_2_B10_A	292	37	LMG 21371	*Erwinia*	*carotovora*
Z0015_RB_E04_2_B11_B	292	37	LMG 21371	*Erwinia*	*carotovora*
Z0015_RB_F01_1_B12_A	292	37	LMG 21371	*Erwinia*	*carotovora*
Z0015_RB_E12_2_B07_B	292	37	LMG 21371	*Erwinia*	*carotovora*
Z0015_LB_G10_2_A02_B	292	37	LMG 21371	*Erwinia*	*carotovora*
Z0015_LB_F10_1_B02_B	292	37	LMG 21371	*Erwinia*	*carotovora*
Z0015_RB_E10_2_B08_B	292	37	LMG 21371	*Erwinia*	*carotovora*
Z0015_LB_G06_2_A04_B	292	37	LMG 21371	*Erwinia*	*carotovora*
Z0015_RB_E09_2_B08_A	293	37	LMG 21371	*Erwinia*	*carotovora*
Z0015_RB_E08_2_B09_B	293	37	LMG 21371	*Erwinia*	*carotovora*
Z0015_LB_G08_2_A03_B	293	37	LMG 21371	*Erwinia*	*carotovora*
Z0015_LB_G09_2_A02_A	293	37	LMG 21371	*Erwinia*	*carotovora*
Z0015_RB_E02_2_B12_B	294	37	LMG 21371	*Erwinia*	*carotovora*
Z0016_RO_D01_2_F07_A	295	51	LMG 23037	*Geobacillus*	*toebii*
Z0016_RO_C12_1_F12_B	295	51	LMG 23037	*Geobacillus*	*toebii*
Z0016_RO_C10_1_F11_B	295	51	LMG 23037	*Geobacillus*	*toebii*
Z0016_RO_C11_1_F12_A	295	51	LMG 23037	*Geobacillus*	*toebii*
Z0016_LO_D07_2_F04_A	295	51	LMG 23037	*Geobacillus*	*toebii*
Z0016_RO_C04_1_F08_B	295	51	LMG 23037	*Geobacillus*	*toebii*
Z0016_LO_C07_1_F04_A	295	51	LMG 23037	*Geobacillus*	*toebii*
Z0016_RO_C03_1_F08_A	295	51	LMG 23037	*Geobacillus*	*toebii*
Z0016_RO_C06_1_F09_B	295	51	LMG 23037	*Geobacillus*	*toebii*
Z0016_RO_D06_2_F09_B	295	51	LMG 23037	*Geobacillus*	*toebii*
Z0016_RO_D03_2_F08_A	295	51	LMG 23037	*Geobacillus*	*toebii*
Z0016_LO_D03_2_F02_A	295	51	LMG 23037	*Geobacillus*	*toebii*
Z0016_LO_D06_2_F03_B	295	51	LMG 23037	*Geobacillus*	*toebii*
Z0016_LO_D05_2_F03_A	295	51	LMG 23037	*Geobacillus*	*toebii*
Z0016_RO_B12_2_E12_B	295	51	LMG 23037	*Geobacillus*	*toebii*
Z0016_LO_C05_1_F03_A	295	51	LMG 23037	*Geobacillus*	*toebii*
Z0016_LO_C03_1_F02_A	295	51	LMG 23037	*Geobacillus*	*toebii*
Z0016_LO_C04_1_F02_B	295	51	LMG 23037	*Geobacillus*	*toebii*
Z0016_RO_B08_2_E10_B	295	51	LMG 23037	*Geobacillus*	*toebii*
Z0016_RO_B11_2_E12_A	295	51	LMG 23037	*Geobacillus*	*toebii*
Z0016_RO_B10_2_E11_B	295	51	LMG 23037	*Geobacillus*	*toebii*
Z0016_RO_D07_2_F10_A	295	51	LMG 23037	*Geobacillus*	*toebii*
Z0016_RO_D08_2_F10_B	295	51	LMG 23037	*Geobacillus*	*toebii*
Z0016_RO_B09_2_E11_A	295	51	LMG 23037	*Geobacillus*	*toebii*
Z0016_LO_E01_1_G01_A	295	51	LMG 23037	*Geobacillus*	*toebii*
Z0016_LO_D12_2_F06_B	295	51	LMG 23037	*Geobacillus*	*toebii*
Z0016_RO_D09_2_F11_A	295	51	LMG 23037	*Geobacillus*	*toebii*
Z0016_RO_C08_1_F10_B	295	51	LMG 23037	*Geobacillus*	*toebii*
Z0016_LO_C09_1_F05_A	295	51	LMG 23037	*Geobacillus*	*toebii*

Z0016_RO_C09_1_F11_A	295	51	LMG 23037	*Geobacillus*	*toebii*
Z0016_LO_C10_1_F05_B	295	51	LMG 23037	*Geobacillus*	*toebii*
Z0016_LO_D11_2_F06_A	295	51	LMG 23037	*Geobacillus*	*toebii*
Z0016_RO_B03_2_E08_A	296	50	LMG 23003	*Rhodanobacter*	*fulvus*
Z0016_LO_B11_2_E06_A	296	50	LMG 23003	*Rhodanobacter*	*fulvus*
Z0016_LO_C01_1_F01_A	296	50	LMG 23003	*Rhodanobacter*	*fulvus*
Z0016_RO_B04_2_E08_B	296	50	LMG 23003	*Rhodanobacter*	*fulvus*
Z0016_LO_B12_2_E06_B	296	50	LMG 23003	*Rhodanobacter*	*fulvus*
Z0016_RO_B06_2_E09_B	296	50	LMG 23003	*Rhodanobacter*	*fulvus*
Z0016_RO_A07_1_E10_A	296	50	LMG 23003	*Rhodanobacter*	*fulvus*
Z0016_LO_B03_2_E02_A	296	50	LMG 23003	*Rhodanobacter*	*fulvus*
Z0016_LO_A12_1_E06_B	296	50	LMG 23003	*Rhodanobacter*	*fulvus*
Z0016_RO_A09_1_E11_A	296	50	LMG 23003	*Rhodanobacter*	*fulvus*
Z0016_LO_B09_2_E05_A	296	50	LMG 23003	*Rhodanobacter*	*fulvus*
Z0016_RO_A08_1_E10_B	296	50	LMG 23003	*Rhodanobacter*	*fulvus*
Z0016_RO_A06_1_E09_B	296	50	LMG 23003	*Rhodanobacter*	*fulvus*
Z0016_LO_A10_1_E05_B	296	50	LMG 23003	*Rhodanobacter*	*fulvus*
Z0016_RO_A05_1_E09_A	296	50	LMG 23003	*Rhodanobacter*	*fulvus*
Z0016_LO_B06_2_E03_B	296	50	LMG 23003	*Rhodanobacter*	*fulvus*
Z0016_LO_B02_2_E01_B	296	50	LMG 23003	*Rhodanobacter*	*fulvus*
Z0016_LO_B05_2_E03_A	296	50	LMG 23003	*Rhodanobacter*	*fulvus*
Z0016_LO_A11_1_E06_A	296	50	LMG 23003	*Rhodanobacter*	*fulvus*
Z0016_RO_A12_1_E12_B	296	50	LMG 23003	*Rhodanobacter*	*fulvus*
Z0016_LO_B04_2_E02_B	296	50	LMG 23003	*Rhodanobacter*	*fulvus*
Z0016_RO_A11_1_E12_A	296	50	LMG 23003	*Rhodanobacter*	*fulvus*
Z0016_LO_B01_2_E01_A	296	50	LMG 23003	*Rhodanobacter*	*fulvus*
Z0016_LO_B08_2_E04_B	296	50	LMG 23003	*Rhodanobacter*	*fulvus*
Z0016_LO_B10_2_E05_B	296	50	LMG 23003	*Rhodanobacter*	*fulvus*
Z0016_LO_B07_2_E04_A	296	50	LMG 23003	*Rhodanobacter*	*fulvus*
Z0016_RO_B05_2_E09_A	296	50	LMG 23003	*Rhodanobacter*	*fulvus*
Z0016_LO_C02_1_F01_B	296	50	LMG 23003	*Rhodanobacter*	*fulvus*
Z0016_RO_F07_2_G10_A	297	52	LMG 23059	*Blastobacter*	*aggregatus*
Z0016_LO_F07_2_G04_A	297	52	LMG 23059	*Blastobacter*	*aggregatus*
Z0016_LO_G01_1_H01_A	297	52	LMG 23059	*Blastobacter*	*aggregatus*
Z0016_LO_G02_1_H01_B	297	52	LMG 23059	*Blastobacter*	*aggregatus*
Z0016_RO_F10_2_G11_B	297	52	LMG 23059	*Blastobacter*	*aggregatus*
Z0016_LO_F12_2_G06_B	297	52	LMG 23059	*Blastobacter*	*aggregatus*
Z0016_RO_F09_2_G11_A	297	52	LMG 23059	*Blastobacter*	*aggregatus*
Z0016_LO_F09_2_G05_A	297	52	LMG 23059	*Blastobacter*	*aggregatus*
Z0016_RO_E05_1_G09_A	297	52	LMG 23059	*Blastobacter*	*aggregatus*
Z0016_LO_E08_1_G04_B	297	52	LMG 23059	*Blastobacter*	*aggregatus*
Z0016_RO_E04_1_G08_B	297	52	LMG 23059	*Blastobacter*	*aggregatus*
Z0016_RO_E03_1_G08_A	297	52	LMG 23059	*Blastobacter*	*aggregatus*
Z0016_RO_E07_1_G10_A	297	52	LMG 23059	*Blastobacter*	*aggregatus*
Z0016_LO_E11_1_G06_A	297	52	LMG 23059	*Blastobacter*	*aggregatus*
Z0016_RO_E08_1_G10_B	297	52	LMG 23059	*Blastobacter*	*aggregatus*
Z0016_LO_E10_1_G05_B	297	52	LMG 23059	*Blastobacter*	*aggregatus*
Z0016_RO_G05_1_H09_A	297	52	LMG 23059	*Blastobacter*	*aggregatus*
Z0016_LO_G04_1_H02_B	297	52	LMG 23059	*Blastobacter*	*aggregatus*
Z0016_RO_G02_1_H07_B	297	52	LMG 23059	*Blastobacter*	*aggregatus*

Z0016_RO_G04_1_H08_B	297	52	LMG 23059	*Blastobacter*	*aggregatus*
Z0016_RO_E11_1_G12_A	297	52	LMG 23059	*Blastobacter*	*aggregatus*
Z0016_LO_F01_2_G01_A	297	52	LMG 23059	*Blastobacter*	*aggregatus*
Z0016_RO_F01_2_G07_A	297	52	LMG 23059	*Blastobacter*	*aggregatus*
Z0016_RO_E10_1_G11_B	297	52	LMG 23059	*Blastobacter*	*aggregatus*
Z0016_LO_F06_2_G03_B	298	52	LMG 23059	*Blastobacter*	*aggregatus*
Z0016_LO_F03_2_G02_A	298	52	LMG 23059	*Blastobacter*	*aggregatus*
Z0016_RO_F04_2_G08_B	298	52	LMG 23059	*Blastobacter*	*aggregatus*
Z0016_RO_F03_2_G08_A	298	52	LMG 23059	*Blastobacter*	*aggregatus*
Z0016_LO_E04_1_G02_B	298	52	LMG 23059	*Blastobacter*	*aggregatus*
Z0016_LO_E05_1_G03_A	298	52	LMG 23059	*Blastobacter*	*aggregatus*
Z0016_LO_E02_1_G01_B	298	52	LMG 23059	*Blastobacter*	*aggregatus*
Z0016_LO_E03_1_G02_A	298	52	LMG 23059	*Blastobacter*	*aggregatus*
Z0016_RB_G08_2_A09_B	299	48	LMG 22697	*Mesorhizobium*	*thiogangeticum*
Z0016_LB_G11_2_A01_A	299	48	LMG 22697	*Mesorhizobium*	*thiogangeticum*
Z0016_RB_G10_2_A08_B	299	48	LMG 22697	*Mesorhizobium*	*thiogangeticum*
Z0016_LB_G12_2_A01_B	299	48	LMG 22697	*Mesorhizobium*	*thiogangeticum*
Z0016_RB_G11_2_A07_A	299	48	LMG 22697	*Mesorhizobium*	*thiogangeticum*
Z0016_LB_H02_1_A06_B	299	48	LMG 22697	*Mesorhizobium*	*thiogangeticum*
Z0016_RB_F12_1_B07_B	299	48	LMG 22697	*Mesorhizobium*	*thiogangeticum*
Z0016_LB_G03_2_A05_A	299	48	LMG 22697	*Mesorhizobium*	*thiogangeticum*
Z0016_RB_G01_2_A12_A	299	48	LMG 22697	*Mesorhizobium*	*thiogangeticum*
Z0016_RB_G09_2_A08_A	299	48	LMG 22697	*Mesorhizobium*	*thiogangeticum*
Z0016_LB_H01_1_A06_A	299	48	LMG 22697	*Mesorhizobium*	*thiogangeticum*
Z0016_RB_G04_2_A11_B	299	48	LMG 22697	*Mesorhizobium*	*thiogangeticum*
Z0016_LB_G07_2_A03_A	299	48	LMG 22697	*Mesorhizobium*	*thiogangeticum*
Z0016_RB_G06_2_A10_B	299	48	LMG 22697	*Mesorhizobium*	*thiogangeticum*
Z0016_LB_H05_1_A04_A	299	48	LMG 22697	*Mesorhizobium*	*thiogangeticum*
Z0016_LB_H04_1_A05_B	299	48	LMG 22697	*Mesorhizobium*	*thiogangeticum*
Z0016_RB_G05_2_A10_A	299	48	LMG 22697	*Mesorhizobium*	*thiogangeticum*
Z0016_RB_G12_2_A07_B	299	48	LMG 22697	*Mesorhizobium*	*thiogangeticum*
Z0016_LB_H03_1_A05_A	299	48	LMG 22697	*Mesorhizobium*	*thiogangeticum*
Z0016_RB_G03_2_A11_A	299	48	LMG 22697	*Mesorhizobium*	*thiogangeticum*
Z0016_LB_G06_2_A04_B	299	48	LMG 22697	*Mesorhizobium*	*thiogangeticum*
Z0016_LB_G04_2_A05_B	299	48	LMG 22697	*Mesorhizobium*	*thiogangeticum*
Z0016_LB_G05_2_A04_A	299	48	LMG 22697	*Mesorhizobium*	*thiogangeticum*
Z0016_RB_H01_1_A12_A	299	48	LMG 22697	*Mesorhizobium*	*thiogangeticum*
Z0016_LB_H06_1_A04_B	299	48	LMG 22697	*Mesorhizobium*	*thiogangeticum*
Z0016_RB_H02_1_A12_B	299	48	LMG 22697	*Mesorhizobium*	*thiogangeticum*
Z0016_RB_H03_1_A11_A	299	48	LMG 22697	*Mesorhizobium*	*thiogangeticum*
Z0016_LB_G09_2_A02_A	299	48	LMG 22697	*Mesorhizobium*	*thiogangeticum*
Z0016_RB_G07_2_A09_A	299	48	LMG 22697	*Mesorhizobium*	*thiogangeticum*
Z0016_LB_G08_2_A03_B	299	48	LMG 22697	*Mesorhizobium*	*thiogangeticum*
Z0016_LB_G10_2_A02_B	299	48	LMG 22697	*Mesorhizobium*	*thiogangeticum*
Z0016_RB_G02_2_A12_B	300	48	LMG 22697	*Mesorhizobium*	*thiogangeticum*
Z0017_RB_G02_2_A12_B	301	60	LMG 23835	*Marinobacter*	*algicola*
Z0017_LB_G09_2_A02_A	301	60	LMG 23835	*Marinobacter*	*algicola*
Z0017_LB_G04_2_A05_B	301	60	LMG 23835	*Marinobacter*	*algicola*
Z0017_LB_G05_2_A04_A	301	60	LMG 23835	*Marinobacter*	*algicola*
Z0017_RB_G10_2_A08_B	302	60	LMG 23835	*Marinobacter*	*algicola*

Z0017_LB_G02_2_A06_B	303	60	LMG 23835	*Marinobacter*	*algicola*	
Z0017_LB_F12_1_B01_B	303	60	LMG 23835	*Marinobacter*	*algicola*	
Z0017_RB_G12_2_A07_B	303	60	LMG 23835	*Marinobacter*	*algicola*	
Z0017_LB_G11_2_A01_A	303	60	LMG 23835	*Marinobacter*	*algicola*	
Z0017_RB_H01_1_A12_A	303	60	LMG 23835	*Marinobacter*	*algicola*	
Z0017_RB_G11_2_A07_A	303	60	LMG 23835	*Marinobacter*	*algicola*	
Z0017_LB_G06_2_A04_B	303	60	LMG 23835	*Marinobacter*	*algicola*	
Z0017_LB_G08_2_A03_B	303	60	LMG 23835	*Marinobacter*	*algicola*	
Z0017_RB_F12_1_B07_B	303	60	LMG 23835	*Marinobacter*	*algicola*	
Z0017_RB_F11_1_B07_A	303	60	LMG 23835	*Marinobacter*	*algicola*	
Z0017_RB_G01_2_A12_A	303	60	LMG 23835	*Marinobacter*	*algicola*	
Z0017_LB_G03_2_A05_A	303	60	LMG 23835	*Marinobacter*	*algicola*	
Z0017_RB_G04_2_A11_B	303	60	LMG 23835	*Marinobacter*	*algicola*	
Z0017_LB_G07_2_A03_A	303	60	LMG 23835	*Marinobacter*	*algicola*	
Z0017_LB_F11_1_B01_A	303	60	LMG 23835	*Marinobacter*	*algicola*	
Z0017_RB_G05_2_A10_A	303	60	LMG 23835	*Marinobacter*	*algicola*	
Z0017_RB_G03_2_A11_A	303	60	LMG 23835	*Marinobacter*	*algicola*	
Z0017_LB_G01_2_A06_A	303	60	LMG 23835	*Marinobacter*	*algicola*	
Z0017_LB_G10_2_A02_B	303	60	LMG 23835	*Marinobacter*	*algicola*	
Z0017_RB_H03_1_A11_A	303	60	LMG 23835	*Marinobacter*	*algicola*	
Z0017_RB_H02_1_A12_B	303	60	LMG 23835	*Marinobacter*	*algicola*	
Z0017_LB_H01_1_A06_A	303	60	LMG 23835	*Marinobacter*	*algicola*	
Z0017_RB_G07_2_A09_A	303	60	LMG 23835	*Marinobacter*	*algicola*	
Z0017_RB_G09_2_A08_A	303	60	LMG 23835	*Marinobacter*	*algicola*	
Z0017_RB_G08_2_A09_B	303	60	LMG 23835	*Marinobacter*	*algicola*	
Z0017_LB_G12_2_A01_B	303	60	LMG 23835	*Marinobacter*	*algicola*	
Z0017_RB_G06_2_A10_B	303	60	LMG 23835	*Marinobacter*	*algicola*	
Z0025_LB_C11_2_C01_A	304	134	LMG 7874	*Morganella*	*morganii*	*morganii*
Z0025_LB_C03_2_C05_A	304	134	LMG 7874	*Morganella*	*morganii*	*morganii*
Z0025_RB_D12_1_C07_B	304	134	LMG 7874	*Morganella*	*morganii*	*morganii*
Z0025_RB_E01_2_B12_A	304	134	LMG 7874	*Morganella*	*morganii*	*morganii*
Z0025_LB_B12_1_D01_B	304	134	LMG 7874	*Morganella*	*morganii*	*morganii*
Z0025_LB_C01_2_C06_A	304	134	LMG 7874	*Morganella*	*morganii*	*morganii*
Z0025_RB_E02_2_B12_B	304	134	LMG 7874	*Morganella*	*morganii*	*morganii*
Z0025_LB_C10_2_C02_B	304	134	LMG 7874	*Morganella*	*morganii*	*morganii*
Z0025_LB_C02_2_C06_B	304	134	LMG 7874	*Morganella*	*morganii*	*morganii*
Z0025_RB_E03_2_B11_A	304	134	LMG 7874	*Morganella*	*morganii*	*morganii*
Z0025_RB_E10_2_B08_B	305	134	LMG 7874	*Morganella*	*morganii*	*morganii*
Z0025_LB_C07_2_C03_A	305	134	LMG 7874	*Morganella*	*morganii*	*morganii*
Z0025_RB_E12_2_B07_B	305	134	LMG 7874	*Morganella*	*morganii*	*morganii*
Z0025_LB_C08_2_C03_B	305	134	LMG 7874	*Morganella*	*morganii*	*morganii*
Z0025_LB_C09_2_C02_A	305	134	LMG 7874	*Morganella*	*morganii*	*morganii*
Z0025_RB_D08_1_C09_B	306	134	LMG 7874	*Morganella*	*morganii*	*morganii*
Z0025_RB_D10_1_C08_B	306	134	LMG 7874	*Morganella*	*morganii*	*morganii*
Z0025_RB_D11_1_C07_A	306	134	LMG 7874	*Morganella*	*morganii*	*morganii*
Z0025_RB_E07_2_B09_A	306	134	LMG 7874	*Morganella*	*morganii*	*morganii*
Z0025_LB_C06_2_C04_B	306	134	LMG 7874	*Morganella*	*morganii*	*morganii*
Z0025_LB_B11_1_D01_A	306	134	LMG 7874	*Morganella*	*morganii*	*morganii*
Z0025_LB_B09_1_D02_A	306	134	LMG 7874	*Morganella*	*morganii*	*morganii*
Z0025_LB_B10_1_D02_B	306	134	LMG 7874	*Morganella*	*morganii*	*morganii*

Z0025_RB_E11_2_B07_A	306	134	LMG 7874	*Morganella*	*morganii*	*morganii*
Z0025_RB_D09_1_C08_A	306	134	LMG 7874	*Morganella*	*morganii*	*morganii*
Z0025_LB_B08_1_D03_B	306	134	LMG 7874	*Morganella*	*morganii*	*morganii*
Z0025_RB_E06_2_B10_B	306	134	LMG 7874	*Morganella*	*morganii*	*morganii*
Z0025_RB_E04_2_B11_B	306	134	LMG 7874	*Morganella*	*morganii*	*morganii*
Z0025_LB_C04_2_C05_B	306	134	LMG 7874	*Morganella*	*morganii*	*morganii*
Z0025_RB_E05_2_B10_A	306	134	LMG 7874	*Morganella*	*morganii*	*morganii*
Z0025_RB_E09_2_B08_A	306	134	LMG 7874	*Morganella*	*morganii*	*morganii*
Z0025_RB_E08_2_B09_B	306	134	LMG 7874	*Morganella*	*morganii*	*morganii*
Z0017_LO_F02_2_G01_B	307	63	LMG 24015	*Chitinophaga*	*terrae*	
Z0017_LO_F01_2_G01_A	307	63	LMG 24015	*Chitinophaga*	*terrae*	
Z0017_LO_E12_1_G06_B	307	63	LMG 24015	*Chitinophaga*	*terrae*	
Z0017_RO_F10_2_G11_B	307	63	LMG 24015	*Chitinophaga*	*terrae*	
Z0017_RO_E05_1_G09_A	307	63	LMG 24015	*Chitinophaga*	*terrae*	
Z0017_RO_F05_2_G09_A	307	63	LMG 24015	*Chitinophaga*	*terrae*	
Z0017_LO_E07_1_G04_A	307	63	LMG 24015	*Chitinophaga*	*terrae*	
Z0017_RO_F04_2_G08_B	307	63	LMG 24015	*Chitinophaga*	*terrae*	
Z0017_RO_F03_2_G08_A	307	63	LMG 24015	*Chitinophaga*	*terrae*	
Z0017_RO_E04_1_G08_B	307	63	LMG 24015	*Chitinophaga*	*terrae*	
Z0017_RO_E02_1_G07_B	307	63	LMG 24015	*Chitinophaga*	*terrae*	
Z0017_LO_D12_2_F06_B	307	63	LMG 24015	*Chitinophaga*	*terrae*	
Z0017_LO_D11_2_F06_A	307	63	LMG 24015	*Chitinophaga*	*terrae*	
Z0017_RO_E08_1_G10_B	307	63	LMG 24015	*Chitinophaga*	*terrae*	
Z0017_RO_F01_2_G07_A	307	63	LMG 24015	*Chitinophaga*	*terrae*	
Z0017_LO_E03_1_G02_A	307	63	LMG 24015	*Chitinophaga*	*terrae*	
Z0017_RO_E11_1_G12_A	307	63	LMG 24015	*Chitinophaga*	*terrae*	
Z0017_LO_E05_1_G03_A	307	63	LMG 24015	*Chitinophaga*	*terrae*	
Z0017_RO_D07_2_F10_A	307	63	LMG 24015	*Chitinophaga*	*terrae*	
Z0017_RO_D09_2_F11_A	307	63	LMG 24015	*Chitinophaga*	*terrae*	
Z0017_LO_D01_2_F01_A	307	63	LMG 24015	*Chitinophaga*	*terrae*	
Z0017_LO_E02_1_G01_B	307	63	LMG 24015	*Chitinophaga*	*terrae*	
Z0017_LO_D06_2_F03_B	307	63	LMG 24015	*Chitinophaga*	*terrae*	
Z0017_RO_D12_2_F12_B	307	63	LMG 24015	*Chitinophaga*	*terrae*	
Z0017_RO_D11_2_F12_A	307	63	LMG 24015	*Chitinophaga*	*terrae*	
Z0017_RO_D06_2_F09_B	307	63	LMG 24015	*Chitinophaga*	*terrae*	
Z0017_LO_D03_2_F02_A	307	63	LMG 24015	*Chitinophaga*	*terrae*	
Z0017_RO_D03_2_F08_A	307	63	LMG 24015	*Chitinophaga*	*terrae*	
Z0017_LO_C10_1_F05_B	307	63	LMG 24015	*Chitinophaga*	*terrae*	
Z0017_RO_D04_2_F08_B	307	63	LMG 24015	*Chitinophaga*	*terrae*	
Z0017_LO_C08_1_F04_B	307	63	LMG 24015	*Chitinophaga*	*terrae*	
Z0017_LO_D08_2_F04_B	308	63	LMG 24015	*Chitinophaga*	*terrae*	
Z0012_RO_E10_1_G11_B	309	9	LMG 12537	*Rhizorhapis*	*suberifaciens*	
Z0012_LO_H01_2_H01_A	309	9	LMG 12537	*Rhizorhapis*	*suberifaciens*	
Z0012_RO_G05_1_H09_A	309	9	LMG 12537	*Rhizorhapis*	*suberifaciens*	
Z0012_RO_F08_2_G10_B	309	9	LMG 12537	*Rhizorhapis*	*suberifaciens*	
Z0012_RO_E09_1_G11_A	309	9	LMG 12537	*Rhizorhapis*	*suberifaciens*	
Z0012_RO_G06_1_H09_B	309	9	LMG 12537	*Rhizorhapis*	*suberifaciens*	
Z0012_RO_E07_1_G10_A	309	9	LMG 12537	*Rhizorhapis*	*suberifaciens*	
Z0012_RO_F01_2_G07_A	309	9	LMG 12537	*Rhizorhapis*	*suberifaciens*	
Z0012_RO_F03_2_G08_A	309	9	LMG 12537	*Rhizorhapis*	*suberifaciens*	

Z0012_LO_F08_2_G04_B	309	9	LMG 12537	*Rhizorhapis*	*suberifaciens*
Z0012_RO_G04_1_H08_B	309	9	LMG 12537	*Rhizorhapis*	*suberifaciens*
Z0012_RO_F10_2_G11_B	309	9	LMG 12537	*Rhizorhapis*	*suberifaciens*
Z0012_LO_H07_2_H04_A	309	9	LMG 12537	*Rhizorhapis*	*suberifaciens*
Z0012_RO_F09_2_G11_A	309	9	LMG 12537	*Rhizorhapis*	*suberifaciens*
Z0012_RO_G03_1_H08_A	309	9	LMG 12537	*Rhizorhapis*	*suberifaciens*
Z0012_RO_E08_1_G10_B	309	9	LMG 12537	*Rhizorhapis*	*suberifaciens*
Z0012_LO_F11_2_G06_A	309	9	LMG 12537	*Rhizorhapis*	*suberifaciens*
Z0012_LO_F09_2_G05_A	309	9	LMG 12537	*Rhizorhapis*	*suberifaciens*
Z0012_LO_F10_2_G05_B	309	9	LMG 12537	*Rhizorhapis*	*suberifaciens*
Z0012_LO_G02_1_H01_B	309	9	LMG 12537	*Rhizorhapis*	*suberifaciens*
Z0012_LO_G04_1_H02_B	309	9	LMG 12537	*Rhizorhapis*	*suberifaciens*
Z0012_LO_G08_1_H04_B	309	9	LMG 12537	*Rhizorhapis*	*suberifaciens*
Z0012_LO_G09_1_H05_A	309	9	LMG 12537	*Rhizorhapis*	*suberifaciens*
Z0012_RO_F05_2_G09_A	309	9	LMG 12537	*Rhizorhapis*	*suberifaciens*
Z0012_RO_F04_2_G08_B	309	9	LMG 12537	*Rhizorhapis*	*suberifaciens*
Z0012_RO_F11_2_G12_A	309	9	LMG 12537	*Rhizorhapis*	*suberifaciens*
Z0012_LO_H02_2_H01_B	309	9	LMG 12537	*Rhizorhapis*	*suberifaciens*
Z0012_LO_H06_2_H03_B	310	9	LMG 12537	*Rhizorhapis*	*suberifaciens*
Z0014_LO_C07_1_F04_A	311	32	LMG 19863	*Maricaulis*	*parjimensis*
Z0014_LO_C08_1_F04_B	311	32	LMG 19863	*Maricaulis*	*parjimensis*
Z0014_LO_E02_1_G01_B	311	32	LMG 19863	*Maricaulis*	*parjimensis*
Z0014_RO_D12_2_F12_B	311	32	LMG 19863	*Maricaulis*	*parjimensis*
Z0014_LO_D05_2_F03_A	311	32	LMG 19863	*Maricaulis*	*parjimensis*
Z0014_RO_E02_1_G07_B	311	32	LMG 19863	*Maricaulis*	*parjimensis*
Z0014_LO_E03_1_G02_A	311	32	LMG 19863	*Maricaulis*	*parjimensis*
Z0014_RO_E08_1_G10_B	311	32	LMG 19863	*Maricaulis*	*parjimensis*
Z0014_RO_E05_1_G09_A	311	32	LMG 19863	*Maricaulis*	*parjimensis*
Z0014_RO_D06_2_F09_B	311	32	LMG 19863	*Maricaulis*	*parjimensis*
Z0014_RO_E07_1_G10_A	311	32	LMG 19863	*Maricaulis*	*parjimensis*
Z0014_RO_E04_1_G08_B	311	32	LMG 19863	*Maricaulis*	*parjimensis*
Z0014_RO_F03_2_G08_A	311	32	LMG 19863	*Maricaulis*	*parjimensis*
Z0014_LO_E04_1_G02_B	311	32	LMG 19863	*Maricaulis*	*parjimensis*
Z0014_LO_D03_2_F02_A	311	32	LMG 19863	*Maricaulis*	*parjimensis*
Z0014_RO_D02_2_F07_B	311	32	LMG 19863	*Maricaulis*	*parjimensis*
Z0014_RO_D10_2_F11_B	311	32	LMG 19863	*Maricaulis*	*parjimensis*
Z0014_LO_C03_1_F02_A	311	32	LMG 19863	*Maricaulis*	*parjimensis*
Z0014_LB_A01_2_D06_A	311	32	LMG 19863	*Maricaulis*	*parjimensis*
Z0014_LO_C10_1_F05_B	311	32	LMG 19863	*Maricaulis*	*parjimensis*
Z0014_RO_D03_2_F08_A	311	32	LMG 19863	*Maricaulis*	*parjimensis*
Z0014_LO_D02_2_F01_B	311	32	LMG 19863	*Maricaulis*	*parjimensis*
Z0014_RO_E09_1_G11_A	311	32	LMG 19863	*Maricaulis*	*parjimensis*
Z0014_RO_D05_2_F09_A	311	32	LMG 19863	*Maricaulis*	*parjimensis*
Z0014_RO_E01_1_G07_A	311	32	LMG 19863	*Maricaulis*	*parjimensis*
Z0014_LO_D01_2_F01_A	311	32	LMG 19863	*Maricaulis*	*parjimensis*
Z0014_RO_E10_1_G11_B	311	32	LMG 19863	*Maricaulis*	*parjimensis*
Z0014_LO_C12_1_F06_B	311	32	LMG 19863	*Maricaulis*	*parjimensis*
Z0014_RO_D01_2_F07_A	311	32	LMG 19863	*Maricaulis*	*parjimensis*
Z0014_LO_E10_1_G05_B	311	32	LMG 19863	*Maricaulis*	*parjimensis*
Z0014_LO_D11_2_F06_A	311	32	LMG 19863	*Maricaulis*	*parjimensis*

Z0014_RO_D11_2_F12_A	311	32	LMG 19863	*Maricaulis*	*parjimensis*
Z0016_RO_D02_2_F07_B	312	44	LMG 22214	*Aeromonas*	*molluscorum*
Z0016_LO_H06_2_H03_B	312	44	LMG 22214	*Aeromonas*	*molluscorum*
Z0016_LB_A02_2_D06_B	312	44	LMG 22214	*Aeromonas*	*molluscorum*
Z0016_LB_A03_2_D05_A	312	44	LMG 22214	*Aeromonas*	*molluscorum*
Z0016_LB_A06_2_D04_B	312	44	LMG 22214	*Aeromonas*	*molluscorum*
Z0016_RB_A01_2_D12_A	312	44	LMG 22214	*Aeromonas*	*molluscorum*
Z0016_LB_A01_2_D06_A	312	44	LMG 22214	*Aeromonas*	*molluscorum*
Z0016_LB_A10_2_D02_B	312	44	LMG 22214	*Aeromonas*	*molluscorum*
Z0016_LB_A11_2_D01_A	312	44	LMG 22214	*Aeromonas*	*molluscorum*
Z0016_RB_A06_2_D10_B	312	44	LMG 22214	*Aeromonas*	*molluscorum*
Z0016_RB_A05_2_D10_A	312	44	LMG 22214	*Aeromonas*	*molluscorum*
Z0016_LO_H08_2_H04_B	312	44	LMG 22214	*Aeromonas*	*molluscorum*
Z0016_LB_A04_2_D05_B	312	44	LMG 22214	*Aeromonas*	*molluscorum*
Z0016_LO_H07_2_H04_A	312	44	LMG 22214	*Aeromonas*	*molluscorum*
Z0016_LO_H09_2_H05_A	312	44	LMG 22214	*Aeromonas*	*molluscorum*
Z0016_RB_A04_2_D11_B	312	44	LMG 22214	*Aeromonas*	*molluscorum*
Z0016_LB_A08_2_D03_B	312	44	LMG 22214	*Aeromonas*	*molluscorum*
Z0016_RB_A03_2_D11_A	312	44	LMG 22214	*Aeromonas*	*molluscorum*
Z0016_LB_A09_2_D02_A	312	44	LMG 22214	*Aeromonas*	*molluscorum*
Z0016_LO_H10_2_H05_B	313	44	LMG 22214	*Aeromonas*	*molluscorum*
Z0016_LO_C06_1_F03_B	313	44	LMG 22214	*Aeromonas*	*molluscorum*
Z0016_RO_C05_1_F09_A	313	44	LMG 22214	*Aeromonas*	*molluscorum*
Z0016_RO_B07_2_E10_A	313	44	LMG 22214	*Aeromonas*	*molluscorum*
Z0016_RB_A02_2_D12_B	313	44	LMG 22214	*Aeromonas*	*molluscorum*
Z0016_LB_A07_2_D03_A	313	44	LMG 22214	*Aeromonas*	*molluscorum*
Z0016_RO_E06_1_G09_B	313	44	LMG 22214	*Aeromonas*	*molluscorum*
Z0016_LO_H12_2_H06_B	313	44	LMG 22214	*Aeromonas*	*molluscorum*
Z0016_LB_A05_2_D04_A	313	44	LMG 22214	*Aeromonas*	*molluscorum*
Z0016_RO_E12_1_G12_B	313	44	LMG 22214	*Aeromonas*	*molluscorum*
Z0016_LO_H11_2_H06_A	313	44	LMG 22214	*Aeromonas*	*molluscorum*
Z0016_LO_C08_1_F04_B	313	44	LMG 22214	*Aeromonas*	*molluscorum*
Z0016_LO_D04_2_F02_B	313	44	LMG 22214	*Aeromonas*	*molluscorum*
Z0017_RO_A05_1_E09_A	314	61	LMG 23965	*Collimonas*	*pratensis*
Z0017_RO_A04_1_E08_B	314	61	LMG 23965	*Collimonas*	*pratensis*
Z0017_RO_A03_1_E08_A	314	61	LMG 23965	*Collimonas*	*pratensis*
Z0017_RO_A02_1_E07_B	314	61	LMG 23965	*Collimonas*	*pratensis*
Z0017_RO_A07_1_E10_A	315	61	LMG 23965	*Collimonas*	*pratensis*
Z0017_RO_A11_1_E12_A	316	61	LMG 23965	*Collimonas*	*pratensis*
Z0017_LO_A06_1_E03_B	316	61	LMG 23965	*Collimonas*	*pratensis*
Z0017_LO_A05_1_E03_A	316	61	LMG 23965	*Collimonas*	*pratensis*
Z0017_LB_H06_1_A04_B	316	61	LMG 23965	*Collimonas*	*pratensis*
Z0017_LB_H08_1_A03_B	316	61	LMG 23965	*Collimonas*	*pratensis*
Z0017_LB_H05_1_A04_A	316	61	LMG 23965	*Collimonas*	*pratensis*
Z0017_LB_H07_1_A03_A	316	61	LMG 23965	*Collimonas*	*pratensis*
Z0017_RO_B01_2_E07_A	316	61	LMG 23965	*Collimonas*	*pratensis*
Z0017_RO_A09_1_E11_A	316	61	LMG 23965	*Collimonas*	*pratensis*
Z0017_LO_A02_1_E01_B	316	61	LMG 23965	*Collimonas*	*pratensis*
Z0017_RO_A08_1_E10_B	316	61	LMG 23965	*Collimonas*	*pratensis*
Z0017_RB_H07_1_A09_A	316	61	LMG 23965	*Collimonas*	*pratensis*

Z0017_LB_H04_1_A05_B	316	61	LMG 23965	*Collimonas*	*pratensis*
Z0017_RB_H06_1_A10_B	316	61	LMG 23965	*Collimonas*	*pratensis*
Z0017_RB_H08_1_A09_B	316	61	LMG 23965	*Collimonas*	*pratensis*
Z0017_RB_H04_1_A11_B	317	61	LMG 23965	*Collimonas*	*pratensis*
Z0017_LB_H02_1_A06_B	317	61	LMG 23965	*Collimonas*	*pratensis*
Z0017_LB_H03_1_A05_A	317	61	LMG 23965	*Collimonas*	*pratensis*
Z0017_LB_H09_1_A02_A	317	61	LMG 23965	*Collimonas*	*pratensis*
Z0017_RB_H05_1_A10_A	317	61	LMG 23965	*Collimonas*	*pratensis*
Z0017_LB_H11_1_A01_A	317	61	LMG 23965	*Collimonas*	*pratensis*
Z0017_LB_H10_1_A02_B	317	61	LMG 23965	*Collimonas*	*pratensis*
Z0017_RB_H11_1_A07_A	317	61	LMG 23965	*Collimonas*	*pratensis*
Z0019_LB_F06_1_B04_B	318	81	LMG 25547	*Glaciimonas*	*immobilis*
Z0019_LB_F03_1_B05_A	318	81	LMG 25547	*Glaciimonas*	*immobilis*
Z0019_RB_F08_1_B09_B	318	81	LMG 25547	*Glaciimonas*	*immobilis*
Z0019_LB_F04_1_B05_B	318	81	LMG 25547	*Glaciimonas*	*immobilis*
Z0019_RB_G01_2_A12_A	318	81	LMG 25547	*Glaciimonas*	*immobilis*
Z0019_LB_F07_1_B03_A	318	81	LMG 25547	*Glaciimonas*	*immobilis*
Z0019_LB_F05_1_B04_A	318	81	LMG 25547	*Glaciimonas*	*immobilis*
Z0019_RB_F09_1_B08_A	318	81	LMG 25547	*Glaciimonas*	*immobilis*
Z0019_RB_F12_1_B07_B	318	81	LMG 25547	*Glaciimonas*	*immobilis*
Z0019_LB_F08_1_B03_B	318	81	LMG 25547	*Glaciimonas*	*immobilis*
Z0019_RB_F06_1_B10_B	318	81	LMG 25547	*Glaciimonas*	*immobilis*
Z0019_RB_F05_1_B10_A	318	81	LMG 25547	*Glaciimonas*	*immobilis*
Z0019_RB_F07_1_B09_A	318	81	LMG 25547	*Glaciimonas*	*immobilis*
Z0019_LB_F02_1_B06_B	318	81	LMG 25547	*Glaciimonas*	*immobilis*
Z0019_RB_F04_1_B11_B	318	81	LMG 25547	*Glaciimonas*	*immobilis*
Z0019_LB_F01_1_B06_A	318	81	LMG 25547	*Glaciimonas*	*immobilis*
Z0019_LB_E12_2_B01_B	318	81	LMG 25547	*Glaciimonas*	*immobilis*
Z0019_RB_F03_1_B11_A	318	81	LMG 25547	*Glaciimonas*	*immobilis*
Z0019_RB_E11_2_B07_A	318	81	LMG 25547	*Glaciimonas*	*immobilis*
Z0019_RB_E12_2_B07_B	318	81	LMG 25547	*Glaciimonas*	*immobilis*
Z0019_RB_F01_1_B12_A	318	81	LMG 25547	*Glaciimonas*	*immobilis*
Z0019_LB_E08_2_B03_B	318	81	LMG 25547	*Glaciimonas*	*immobilis*
Z0019_RB_F02_1_B12_B	318	81	LMG 25547	*Glaciimonas*	*immobilis*
Z0019_LB_E10_2_B02_B	318	81	LMG 25547	*Glaciimonas*	*immobilis*
Z0019_LB_E09_2_B02_A	318	81	LMG 25547	*Glaciimonas*	*immobilis*
Z0019_LB_E11_2_B01_A	318	81	LMG 25547	*Glaciimonas*	*immobilis*
Z0019_RB_E09_2_B08_A	318	81	LMG 25547	*Glaciimonas*	*immobilis*
Z0019_RB_E08_2_B09_B	318	81	LMG 25547	*Glaciimonas*	*immobilis*
Z0019_RB_E10_2_B08_B	318	81	LMG 25547	*Glaciimonas*	*immobilis*
Z0019_RB_E07_2_B09_A	318	81	LMG 25547	*Glaciimonas*	*immobilis*
Z0019_RB_F11_1_B07_A	318	81	LMG 25547	*Glaciimonas*	*immobilis*
Z0019_RB_F10_1_B08_B	318	81	LMG 25547	*Glaciimonas*	*immobilis*
Z0014_LO_F08_2_G04_B	319	30	LMG 19484	*Sphingomonas*	*melonis*
Z0014_LO_F09_2_G05_A	319	30	LMG 19484	*Sphingomonas*	*melonis*
Z0014_LO_H08_2_H04_B	319	30	LMG 19484	*Sphingomonas*	*melonis*
Z0014_LO_G12_1_H06_B	319	30	LMG 19484	*Sphingomonas*	*melonis*
Z0014_LO_F12_2_G06_B	319	30	LMG 19484	*Sphingomonas*	*melonis*
Z0014_RO_F04_2_G08_B	319	30	LMG 19484	*Sphingomonas*	*melonis*
Z0014_LO_G01_1_H01_A	319	30	LMG 19484	*Sphingomonas*	*melonis*

Z0014_RO_G11_1_H12_A	319	30	LMG 19484	*Sphingomonas*	*melonis*
Z0014_LO_H03_2_H02_A	319	30	LMG 19484	*Sphingomonas*	*melonis*
Z0014_RO_F09_2_G11_A	319	30	LMG 19484	*Sphingomonas*	*melonis*
Z0014_LO_G05_1_H03_A	319	30	LMG 19484	*Sphingomonas*	*melonis*
Z0014_RO_G10_1_H11_B	319	30	LMG 19484	*Sphingomonas*	*melonis*
Z0014_RO_E12_1_G12_B	319	30	LMG 19484	*Sphingomonas*	*melonis*
Z0014_LO_G10_1_H05_B	319	30	LMG 19484	*Sphingomonas*	*melonis*
Z0014_LO_G06_1_H03_B	319	30	LMG 19484	*Sphingomonas*	*melonis*
Z0014_RO_G12_1_H12_B	319	30	LMG 19484	*Sphingomonas*	*melonis*
Z0014_RO_E11_1_G12_A	319	30	LMG 19484	*Sphingomonas*	*melonis*
Z0014_RO_H02_2_H07_B	319	30	LMG 19484	*Sphingomonas*	*melonis*
Z0014_LO_F02_2_G01_B	319	30	LMG 19484	*Sphingomonas*	*melonis*
Z0014_LO_H04_2_H02_B	319	30	LMG 19484	*Sphingomonas*	*melonis*
Z0014_LO_F04_2_G02_B	319	30	LMG 19484	*Sphingomonas*	*melonis*
Z0014_LO_G11_1_H06_A	319	30	LMG 19484	*Sphingomonas*	*melonis*
Z0014_LO_G07_1_H04_A	319	30	LMG 19484	*Sphingomonas*	*melonis*
Z0014_LO_G09_1_H05_A	319	30	LMG 19484	*Sphingomonas*	*melonis*
Z0014_LO_G08_1_H04_B	319	30	LMG 19484	*Sphingomonas*	*melonis*
Z0014_RO_G06_1_H09_B	319	30	LMG 19484	*Sphingomonas*	*melonis*
Z0014_LO_F03_2_G02_A	319	30	LMG 19484	*Sphingomonas*	*melonis*
Z0014_LO_F05_2_G03_A	319	30	LMG 19484	*Sphingomonas*	*melonis*
Z0014_RO_G07_1_H10_A	319	30	LMG 19484	*Sphingomonas*	*melonis*
Z0014_LO_F10_2_G05_B	319	30	LMG 19484	*Sphingomonas*	*melonis*
Z0014_LO_G03_1_H02_A	319	30	LMG 19484	*Sphingomonas*	*melonis*
Z0014_LO_G02_1_H01_B	319	30	LMG 19484	*Sphingomonas*	*melonis*
Z0017_LB_B08_1_D03_B	320	56	LMG 23381	*Simplicispira*	*metamorpha*
Z0017_LB_B07_1_D03_A	320	56	LMG 23381	*Simplicispira*	*metamorpha*
Z0017_LB_B03_1_D05_A	320	56	LMG 23381	*Simplicispira*	*metamorpha*
Z0017_RB_A02_2_D12_B	320	56	LMG 23381	*Simplicispira*	*metamorpha*
Z0017_LB_B04_1_D05_B	320	56	LMG 23381	*Simplicispira*	*metamorpha*
Z0017_LB_B05_1_D04_A	320	56	LMG 23381	*Simplicispira*	*metamorpha*
Z0017_RB_A03_2_D11_A	320	56	LMG 23381	*Simplicispira*	*metamorpha*
Z0017_RB_A01_2_D12_A	320	56	LMG 23381	*Simplicispira*	*metamorpha*
Z0017_RB_A07_2_D09_A	320	56	LMG 23381	*Simplicispira*	*metamorpha*
Z0017_RB_A06_2_D10_B	320	56	LMG 23381	*Simplicispira*	*metamorpha*
Z0017_RB_A05_2_D10_A	320	56	LMG 23381	*Simplicispira*	*metamorpha*
Z0017_LB_B09_1_D02_A	320	56	LMG 23381	*Simplicispira*	*metamorpha*
Z0017_LB_B02_1_D06_B	320	56	LMG 23381	*Simplicispira*	*metamorpha*
Z0017_LO_H12_2_H06_B	320	56	LMG 23381	*Simplicispira*	*metamorpha*
Z0017_RB_A04_2_D11_B	320	56	LMG 23381	*Simplicispira*	*metamorpha*
Z0017_LB_B06_1_D04_B	320	56	LMG 23381	*Simplicispira*	*metamorpha*
Z0017_LB_C03_2_C05_A	320	56	LMG 23381	*Simplicispira*	*metamorpha*
Z0017_LB_C02_2_C06_B	320	56	LMG 23381	*Simplicispira*	*metamorpha*
Z0017_RB_A11_2_D07_A	320	56	LMG 23381	*Simplicispira*	*metamorpha*
Z0017_LB_C04_2_C05_B	320	56	LMG 23381	*Simplicispira*	*metamorpha*
Z0017_LB_B11_1_D01_A	320	56	LMG 23381	*Simplicispira*	*metamorpha*
Z0017_RB_A10_2_D08_B	320	56	LMG 23381	*Simplicispira*	*metamorpha*
Z0017_RB_B01_1_D12_A	320	56	LMG 23381	*Simplicispira*	*metamorpha*
Z0017_RB_A09_2_D08_A	320	56	LMG 23381	*Simplicispira*	*metamorpha*
Z0017_LB_C01_2_C06_A	320	56	LMG 23381	*Simplicispira*	*metamorpha*

Z0017_RB_A12_2_D07_B	320	56	LMG 23381	*Simplicispira*	*metamorpha*
Z0017_RB_A08_2_D09_B	320	56	LMG 23381	*Simplicispira*	*metamorpha*
Z0017_LB_B12_1_D01_B	320	56	LMG 23381	*Simplicispira*	*metamorpha*
Z0017_LB_B10_1_D02_B	320	56	LMG 23381	*Simplicispira*	*metamorpha*
Z0017_RB_B02_1_D12_B	320	56	LMG 23381	*Simplicispira*	*metamorpha*
Z0017_LB_C05_2_C04_A	320	56	LMG 23381	*Simplicispira*	*metamorpha*
Z0017_LB_C06_2_C04_B	320	56	LMG 23381	*Simplicispira*	*metamorpha*
Z0013_RO_D10_2_F11_B	321	20	LMG 1617	*Acetobacter*	*lovaniensis*
Z0013_LO_D12_2_F06_B	321	20	LMG 1617	*Acetobacter*	*lovaniensis*
Z0013_LB_D09_1_C02_A	321	20	LMG 1617	*Acetobacter*	*lovaniensis*
Z0013_RO_D11_2_F12_A	321	20	LMG 1617	*Acetobacter*	*lovaniensis*
Z0013_LB_D10_1_C02_B	321	20	LMG 1617	*Acetobacter*	*lovaniensis*
Z0013_RO_E01_1_G07_A	321	20	LMG 1617	*Acetobacter*	*lovaniensis*
Z0013_RB_E01_2_B12_A	321	20	LMG 1617	*Acetobacter*	*lovaniensis*
Z0013_LB_D11_1_C01_A	321	20	LMG 1617	*Acetobacter*	*lovaniensis*
Z0013_LO_D09_2_F05_A	321	20	LMG 1617	*Acetobacter*	*lovaniensis*
Z0013_LO_D10_2_F05_B	321	20	LMG 1617	*Acetobacter*	*lovaniensis*
Z0013_LO_D08_2_F04_B	321	20	LMG 1617	*Acetobacter*	*lovaniensis*
Z0013_RB_D12_1_C07_B	321	20	LMG 1617	*Acetobacter*	*lovaniensis*
Z0013_RO_E04_1_G08_B	321	20	LMG 1617	*Acetobacter*	*lovaniensis*
Z0013_RB_D09_1_C08_A	321	20	LMG 1617	*Acetobacter*	*lovaniensis*
Z0013_LB_E02_2_B06_B	321	20	LMG 1617	*Acetobacter*	*lovaniensis*
Z0013_RO_E03_1_G08_A	321	20	LMG 1617	*Acetobacter*	*lovaniensis*
Z0013_RB_E02_2_B12_B	321	20	LMG 1617	*Acetobacter*	*lovaniensis*
Z0013_LB_D12_1_C01_B	321	20	LMG 1617	*Acetobacter*	*lovaniensis*
Z0013_RO_E02_1_G07_B	321	20	LMG 1617	*Acetobacter*	*lovaniensis*
Z0013_LB_E01_2_B06_A	321	20	LMG 1617	*Acetobacter*	*lovaniensis*
Z0013_RB_D10_1_C08_B	321	20	LMG 1617	*Acetobacter*	*lovaniensis*
Z0013_RB_D11_1_C07_A	321	20	LMG 1617	*Acetobacter*	*lovaniensis*
Z0013_LO_E01_1_G01_A	321	20	LMG 1617	*Acetobacter*	*lovaniensis*
Z0013_LO_D11_2_F06_A	321	20	LMG 1617	*Acetobacter*	*lovaniensis*
Z0013_RB_D07_1_C09_A	321	20	LMG 1617	*Acetobacter*	*lovaniensis*
Z0013_RB_D08_1_C09_B	321	20	LMG 1617	*Acetobacter*	*lovaniensis*
Z0013_RO_D12_2_F12_B	321	20	LMG 1617	*Acetobacter*	*lovaniensis*
Z0013_LO_D07_2_F04_A	321	20	LMG 1617	*Acetobacter*	*lovaniensis*
Z0013_RO_D04_2_F08_B	322	18	LMG 1527	*Komagataeibacter*	*hansenii*
Z0013_LO_C11_1_F06_A	322	18	LMG 1527	*Komagataeibacter*	*hansenii*
Z0013_RO_D02_2_F07_B	322	18	LMG 1527	*Komagataeibacter*	*hansenii*
Z0013_LB_C09_2_C02_A	322	18	LMG 1527	*Komagataeibacter*	*hansenii*
Z0013_RO_D03_2_F08_A	322	18	LMG 1527	*Komagataeibacter*	*hansenii*
Z0013_RB_C08_2_C09_B	322	18	LMG 1527	*Komagataeibacter*	*hansenii*
Z0013_LO_C12_1_F06_B	322	18	LMG 1527	*Komagataeibacter*	*hansenii*
Z0013_LB_C06_2_C04_B	322	18	LMG 1527	*Komagataeibacter*	*hansenii*
Z0013_LB_C08_2_C03_B	322	18	LMG 1527	*Komagataeibacter*	*hansenii*
Z0013_RB_C07_2_C09_A	322	18	LMG 1527	*Komagataeibacter*	*hansenii*
Z0013_LO_C10_1_F05_B	322	18	LMG 1527	*Komagataeibacter*	*hansenii*
Z0013_RB_C06_2_C10_B	322	18	LMG 1527	*Komagataeibacter*	*hansenii*
Z0013_LB_C07_2_C03_A	322	18	LMG 1527	*Komagataeibacter*	*hansenii*
Z0013_RO_D01_2_F07_A	322	18	LMG 1527	*Komagataeibacter*	*hansenii*
Z0013_LB_C05_2_C04_A	322	18	LMG 1527	*Komagataeibacter*	*hansenii*

Z0013_RO_C09_1_F11_A	322	18	LMG 1527	*Komagataeibacter*	*hansenii*
Z0013_RB_C04_2_C11_B	322	18	LMG 1527	*Komagataeibacter*	*hansenii*
Z0013_RB_C02_2_C12_B	322	18	LMG 1527	*Komagataeibacter*	*hansenii*
Z0013_RB_C05_2_C10_A	322	18	LMG 1527	*Komagataeibacter*	*hansenii*
Z0013_RB_C03_2_C11_A	322	18	LMG 1527	*Komagataeibacter*	*hansenii*
Z0013_LB_C01_2_C06_A	322	18	LMG 1527	*Komagataeibacter*	*hansenii*
Z0013_RO_C12_1_F12_B	322	18	LMG 1527	*Komagataeibacter*	*hansenii*
Z0013_LB_C03_2_C05_A	322	18	LMG 1527	*Komagataeibacter*	*hansenii*
Z0013_RO_C11_1_F12_A	322	18	LMG 1527	*Komagataeibacter*	*hansenii*
Z0013_RO_C10_1_F11_B	322	18	LMG 1527	*Komagataeibacter*	*hansenii*
Z0013_LO_C06_1_F03_B	322	18	LMG 1527	*Komagataeibacter*	*hansenii*
Z0013_RO_C08_1_F10_B	322	18	LMG 1527	*Komagataeibacter*	*hansenii*
Z0013_LO_C08_1_F04_B	322	18	LMG 1527	*Komagataeibacter*	*hansenii*
Z0013_LO_C09_1_F05_A	322	18	LMG 1527	*Komagataeibacter*	*hansenii*
Z0013_LB_C04_2_C05_B	322	18	LMG 1527	*Komagataeibacter*	*hansenii*
Z0013_LO_C07_1_F04_A	322	18	LMG 1527	*Komagataeibacter*	*hansenii*
Z0013_LB_C02_2_C06_B	322	18	LMG 1527	*Komagataeibacter*	*hansenii*
Z0020_LO_B06_2_E03_B	323	96	LMG 27019	*Kozakia*	*baliensis*
Z0020_LO_A09_1_E05_A	323	96	LMG 27019	*Kozakia*	*baliensis*
Z0020_RO_C09_1_F11_A	323	96	LMG 27019	*Kozakia*	*baliensis*
Z0020_LO_B03_2_E02_A	323	96	LMG 27019	*Kozakia*	*baliensis*
Z0020_LO_B02_2_E01_B	323	96	LMG 27019	*Kozakia*	*baliensis*
Z0020_LO_A04_1_E02_B	323	96	LMG 27019	*Kozakia*	*baliensis*
Z0020_LO_A10_1_E05_B	323	96	LMG 27019	*Kozakia*	*baliensis*
Z0020_RO_C01_1_F07_A	323	96	LMG 27019	*Kozakia*	*baliensis*
Z0020_RO_C04_1_F08_B	323	96	LMG 27019	*Kozakia*	*baliensis*
Z0020_RO_C11_1_F12_A	323	96	LMG 27019	*Kozakia*	*baliensis*
Z0020_RO_C07_1_F10_A	323	96	LMG 27019	*Kozakia*	*baliensis*
Z0020_RO_C12_1_F12_B	323	96	LMG 27019	*Kozakia*	*baliensis*
Z0020_LO_C10_1_F05_B	323	96	LMG 27019	*Kozakia*	*baliensis*
Z0020_LO_C12_1_F06_B	323	96	LMG 27019	*Kozakia*	*baliensis*
Z0020_RO_D03_2_F08_A	323	96	LMG 27019	*Kozakia*	*baliensis*
Z0020_LO_B01_2_E01_A	323	96	LMG 27019	*Kozakia*	*baliensis*
Z0020_LB_H07_1_A03_A	323	96	LMG 27019	*Kozakia*	*baliensis*
Z0020_RO_B01_2_E07_A	323	96	LMG 27019	*Kozakia*	*baliensis*
Z0020_LB_H11_1_A01_A	323	96	LMG 27019	*Kozakia*	*baliensis*
Z0020_RO_D02_2_F07_B	323	96	LMG 27019	*Kozakia*	*baliensis*
Z0020_RO_D06_2_F09_B	323	96	LMG 27019	*Kozakia*	*baliensis*
Z0020_RO_C06_1_F09_B	323	96	LMG 27019	*Kozakia*	*baliensis*
Z0020_LO_C06_1_F03_B	323	96	LMG 27019	*Kozakia*	*baliensis*
Z0020_RO_C10_1_F11_B	323	96	LMG 27019	*Kozakia*	*baliensis*
Z0020_LO_D03_2_F02_A	323	96	LMG 27019	*Kozakia*	*baliensis*
Z0020_LO_B12_2_E06_B	323	96	LMG 27019	*Kozakia*	*baliensis*
Z0020_RO_D04_2_F08_B	323	96	LMG 27019	*Kozakia*	*baliensis*
Z0020_RO_A11_1_E12_A	324	96	LMG 27019	*Kozakia*	*baliensis*
Z0020_RO_A08_1_E10_B	324	96	LMG 27019	*Kozakia*	*baliensis*
Z0020_LB_H09_1_A02_A	324	96	LMG 27019	*Kozakia*	*baliensis*
Z0020_LB_H05_1_A04_A	324	96	LMG 27019	*Kozakia*	*baliensis*
Z0020_LB_H08_1_A03_B	324	96	LMG 27019	*Kozakia*	*baliensis*
Z0016_RO_C01_1_F07_A	325	54	LMG 23083	*Asaia*	*krungthepensis*

Z0016_RO_C02_1_F07_B	325	54	LMG 23083	*Asaia*	*krungthepensis*
Z0016_LO_D09_2_F05_A	325	54	LMG 23083	*Asaia*	*krungthepensis*
Z0016_RO_E01_1_G07_A	325	54	LMG 23083	*Asaia*	*krungthepensis*
Z0016_RO_E02_1_G07_B	325	54	LMG 23083	*Asaia*	*krungthepensis*
Z0016_LO_E09_1_G05_A	325	54	LMG 23083	*Asaia*	*krungthepensis*
Z0016_LO_E07_1_G04_A	325	54	LMG 23083	*Asaia*	*krungthepensis*
Z0016_RO_D12_2_F12_B	325	54	LMG 23083	*Asaia*	*krungthepensis*
Z0016_LO_D08_2_F04_B	325	54	LMG 23083	*Asaia*	*krungthepensis*
Z0016_RO_F02_2_G07_B	325	54	LMG 23083	*Asaia*	*krungthepensis*
Z0016_RO_D11_2_F12_A	325	54	LMG 23083	*Asaia*	*krungthepensis*
Z0016_RO_E09_1_G11_A	325	54	LMG 23083	*Asaia*	*krungthepensis*
Z0016_RO_D10_2_F11_B	325	54	LMG 23083	*Asaia*	*krungthepensis*
Z0016_LO_E12_1_G06_B	325	54	LMG 23083	*Asaia*	*krungthepensis*
Z0016_LO_F02_2_G01_B	325	54	LMG 23083	*Asaia*	*krungthepensis*
Z0016_LO_F05_2_G03_A	325	54	LMG 23083	*Asaia*	*krungthepensis*
Z0016_RO_D04_2_F08_B	325	54	LMG 23083	*Asaia*	*krungthepensis*
Z0016_LO_E06_1_G03_B	325	54	LMG 23083	*Asaia*	*krungthepensis*
Z0016_RO_D05_2_F09_A	325	54	LMG 23083	*Asaia*	*krungthepensis*
Z0016_LO_C12_1_F06_B	325	54	LMG 23083	*Asaia*	*krungthepensis*
Z0016_LO_C11_1_F06_A	325	54	LMG 23083	*Asaia*	*krungthepensis*
Z0016_LO_D01_2_F01_A	325	54	LMG 23083	*Asaia*	*krungthepensis*
Z0016_LO_D10_2_F05_B	325	54	LMG 23083	*Asaia*	*krungthepensis*
Z0016_RO_C07_1_F10_A	325	54	LMG 23083	*Asaia*	*krungthepensis*
Z0016_RO_A10_1_E11_B	325	54	LMG 23083	*Asaia*	*krungthepensis*
Z0016_RO_B02_2_E07_B	325	54	LMG 23083	*Asaia*	*krungthepensis*
Z0016_LO_D02_2_F01_B	325	54	LMG 23083	*Asaia*	*krungthepensis*
Z0016_RO_B01_2_E07_A	325	54	LMG 23083	*Asaia*	*krungthepensis*
Z0016_LO_F04_2_G02_B	326	54	LMG 23083	*Asaia*	*krungthepensis*
Z0016_LO_F08_2_G04_B	326	54	LMG 23083	*Asaia*	*krungthepensis*
Z0016_LO_F11_2_G06_A	326	54	LMG 23083	*Asaia*	*krungthepensis*
Z0016_LO_F10_2_G05_B	326	54	LMG 23083	*Asaia*	*krungthepensis*
Z0018_LO_A08_1_E04_B	327	72	LMG 24537	*Stenotrophomonas*	*rhizophila*
Z0018_LO_A09_1_E05_A	327	72	LMG 24537	*Stenotrophomonas*	*rhizophila*
Z0018_RB_H12_1_A07_B	327	72	LMG 24537	*Stenotrophomonas*	*rhizophila*
Z0018_LO_A07_1_E04_A	327	72	LMG 24537	*Stenotrophomonas*	*rhizophila*
Z0018_LO_A05_1_E03_A	327	72	LMG 24537	*Stenotrophomonas*	*rhizophila*
Z0018_LO_A06_1_E03_B	327	72	LMG 24537	*Stenotrophomonas*	*rhizophila*
Z0018_RB_H09_1_A08_A	327	72	LMG 24537	*Stenotrophomonas*	*rhizophila*
Z0018_RB_H01_1_A12_A	327	72	LMG 24537	*Stenotrophomonas*	*rhizophila*
Z0018_RB_H02_1_A12_B	327	72	LMG 24537	*Stenotrophomonas*	*rhizophila*
Z0018_LB_H11_1_A01_A	327	72	LMG 24537	*Stenotrophomonas*	*rhizophila*
Z0018_LB_H12_1_A01_B	327	72	LMG 24537	*Stenotrophomonas*	*rhizophila*
Z0018_RB_H04_1_A11_B	328	72	LMG 24537	*Stenotrophomonas*	*rhizophila*
Z0018_LO_A01_1_E01_A	328	72	LMG 24537	*Stenotrophomonas*	*rhizophila*
Z0018_RB_H03_1_A11_A	328	72	LMG 24537	*Stenotrophomonas*	*rhizophila*
Z0018_LO_A02_1_E01_B	328	72	LMG 24537	*Stenotrophomonas*	*rhizophila*
Z0018_RO_A05_1_E09_A	329	72	LMG 24537	*Stenotrophomonas*	*rhizophila*
Z0018_RO_A04_1_E08_B	329	72	LMG 24537	*Stenotrophomonas*	*rhizophila*
Z0018_RO_A02_1_E07_B	329	72	LMG 24537	*Stenotrophomonas*	*rhizophila*
Z0018_RB_H11_1_A07_A	329	72	LMG 24537	*Stenotrophomonas*	*rhizophila*

Z0018_LO_A03_1_E02_A	329	72	LMG 24537	*Stenotrophomonas*	*rhizophila*		
Z0018_LO_A04_1_E02_B	329	72	LMG 24537	*Stenotrophomonas*	*rhizophila*		
Z0018_RB_H06_1_A10_B	329	72	LMG 24537	*Stenotrophomonas*	*rhizophila*		
Z0018_RB_H05_1_A10_A	329	72	LMG 24537	*Stenotrophomonas*	*rhizophila*		
Z0018_LO_A10_1_E05_B	329	72	LMG 24537	*Stenotrophomonas*	*rhizophila*		
Z0018_RO_A09_1_E11_A	329	72	LMG 24537	*Stenotrophomonas*	*rhizophila*		
Z0018_RO_A08_1_E10_B	329	72	LMG 24537	*Stenotrophomonas*	*rhizophila*		
Z0018_RO_A07_1_E10_A	329	72	LMG 24537	*Stenotrophomonas*	*rhizophila*		
Z0018_RO_A06_1_E09_B	329	72	LMG 24537	*Stenotrophomonas*	*rhizophila*		
Z0011_LO_D04_2_F02_B	330	1	LMG 1041	*Acinetobacter*	*baumannii*		
Z0011_LO_D05_2_F03_A	330	1	LMG 1041	*Acinetobacter*	*baumannii*		
Z0011_RO_D04_2_F08_B	330	1	LMG 1041	*Acinetobacter*	*baumannii*		
Z0011_RO_D03_2_F08_A	330	1	LMG 1041	*Acinetobacter*	*baumannii*		
Z0011_RO_B08_2_E10_B	330	1	LMG 1041	*Acinetobacter*	*baumannii*		
Z0011_RO_B09_2_E11_A	330	1	LMG 1041	*Acinetobacter*	*baumannii*		
Z0011_LO_C05_1_F03_A	330	1	LMG 1041	*Acinetobacter*	*baumannii*		
Z0011_LO_C06_1_F03_B	330	1	LMG 1041	*Acinetobacter*	*baumannii*		
Z0011_RO_D02_2_F07_B	330	1	LMG 1041	*Acinetobacter*	*baumannii*		
Z0011_RO_C11_1_F12_A	330	1	LMG 1041	*Acinetobacter*	*baumannii*		
Z0011_LO_D01_2_F01_A	330	1	LMG 1041	*Acinetobacter*	*baumannii*		
Z0011_RO_C02_1_F07_B	330	1	LMG 1041	*Acinetobacter*	*baumannii*		
Z0011_LO_C12_1_F06_B	330	1	LMG 1041	*Acinetobacter*	*baumannii*		
Z0011_RO_B11_2_E12_A	330	1	LMG 1041	*Acinetobacter*	*baumannii*		
Z0011_RO_C06_1_F09_B	330	1	LMG 1041	*Acinetobacter*	*baumannii*		
Z0011_LO_C09_1_F05_A	330	1	LMG 1041	*Acinetobacter*	*baumannii*		
Z0011_RO_C01_1_F07_A	330	1	LMG 1041	*Acinetobacter*	*baumannii*		
Z0011_LO_C07_1_F04_A	330	1	LMG 1041	*Acinetobacter*	*baumannii*		
Z0011_LO_C08_1_F04_B	330	1	LMG 1041	*Acinetobacter*	*baumannii*		
Z0011_RO_C10_1_F11_B	330	1	LMG 1041	*Acinetobacter*	*baumannii*		
Z0011_LO_C10_1_F05_B	330	1	LMG 1041	*Acinetobacter*	*baumannii*		
Z0011_RO_C07_1_F10_A	330	1	LMG 1041	*Acinetobacter*	*baumannii*		
Z0011_LO_C11_1_F06_A	330	1	LMG 1041	*Acinetobacter*	*baumannii*		
Z0011_LO_D11_2_F06_A	330	1	LMG 1041	*Acinetobacter*	*baumannii*		
Z0011_LO_D12_2_F06_B	330	1	LMG 1041	*Acinetobacter*	*baumannii*		
Z0011_RO_E02_1_G07_B	330	1	LMG 1041	*Acinetobacter*	*baumannii*		
Z0011_LO_D10_2_F05_B	330	1	LMG 1041	*Acinetobacter*	*baumannii*		
Z0011_RO_D09_2_F11_A	330	1	LMG 1041	*Acinetobacter*	*baumannii*		
Z0011_RO_D10_2_F11_B	330	1	LMG 1041	*Acinetobacter*	*baumannii*		
Z0011_LO_D06_2_F03_B	330	1	LMG 1041	*Acinetobacter*	*baumannii*		
Z0011_LO_D07_2_F04_A	330	1	LMG 1041	*Acinetobacter*	*baumannii*		
Z0011_RO_B12_2_E12_B	330	1	LMG 1041	*Acinetobacter*	*baumannii*		
Z0024_RO_F08_2_G10_B	331	132	LMG 7233	*Salmonella*	*enterica*		*enterica*
Z0024_LO_H05_2_H03_A	331	132	LMG 7233	*Salmonella*	*enterica*		*enterica*
Z0024_LO_E05_1_G03_A	331	132	LMG 7233	*Salmonella*	*enterica*		*enterica*
Z0024_RO_D09_2_F11_A	331	132	LMG 7233	*Salmonella*	*enterica*		*enterica*
Z0024_LO_G11_1_H06_A	331	132	LMG 7233	*Salmonella*	*enterica*		*enterica*
Z0024_LO_H10_2_H05_B	331	132	LMG 7233	*Salmonella*	*enterica*		*enterica*
Z0024_LO_E08_1_G04_B	331	132	LMG 7233	*Salmonella*	*enterica*		*enterica*
Z0024_RO_E01_1_G07_A	331	132	LMG 7233	*Salmonella*	*enterica*		*enterica*
Z0024_LO_H11_2_H06_A	331	132	LMG 7233	*Salmonella*	*enterica*		*enterica*

Z0024_RO_G02_1_H07_B	331	132	LMG 7233	*Salmonella*	*enterica*	*enterica*
Z0024_LO_H04_2_H02_B	331	132	LMG 7233	*Salmonella*	*enterica*	*enterica*
Z0024_LO_F05_2_G03_A	331	132	LMG 7233	*Salmonella*	*enterica*	*enterica*
Z0024_RO_G05_1_H09_A	331	132	LMG 7233	*Salmonella*	*enterica*	*enterica*
Z0024_RO_F11_2_G12_A	331	132	LMG 7233	*Salmonella*	*enterica*	*enterica*
Z0024_RO_F03_2_G08_A	331	132	LMG 7233	*Salmonella*	*enterica*	*enterica*
Z0024_LO_G10_1_H05_B	331	132	LMG 7233	*Salmonella*	*enterica*	*enterica*
Z0024_RO_G09_1_H11_A	331	132	LMG 7233	*Salmonella*	*enterica*	*enterica*
Z0024_RO_F10_2_G11_B	331	132	LMG 7233	*Salmonella*	*enterica*	*enterica*
Z0024_LO_E07_1_G04_A	331	132	LMG 7233	*Salmonella*	*enterica*	*enterica*
Z0024_LO_G12_1_H06_B	331	132	LMG 7233	*Salmonella*	*enterica*	*enterica*
Z0024_LO_F06_2_G03_B	331	132	LMG 7233	*Salmonella*	*enterica*	*enterica*
Z0024_RO_D12_2_F12_B	331	132	LMG 7233	*Salmonella*	*enterica*	*enterica*
Z0024_LO_D08_2_F04_B	331	132	LMG 7233	*Salmonella*	*enterica*	*enterica*
Z0024_RO_E02_1_G07_B	331	132	LMG 7233	*Salmonella*	*enterica*	*enterica*
Z0024_LO_D11_2_F06_A	331	132	LMG 7233	*Salmonella*	*enterica*	*enterica*
Z0024_LO_E03_1_G02_A	331	132	LMG 7233	*Salmonella*	*enterica*	*enterica*
Z0024_RO_D11_2_F12_A	331	132	LMG 7233	*Salmonella*	*enterica*	*enterica*
Z0024_RO_D10_2_F11_B	331	132	LMG 7233	*Salmonella*	*enterica*	*enterica*
Z0024_RO_F09_2_G11_A	331	132	LMG 7233	*Salmonella*	*enterica*	*enterica*
Z0024_LO_G09_1_H05_A	331	132	LMG 7233	*Salmonella*	*enterica*	*enterica*
Z0024_LO_H02_2_H01_B	331	132	LMG 7233	*Salmonella*	*enterica*	*enterica*
Z0024_RO_F05_2_G09_A	331	132	LMG 7233	*Salmonella*	*enterica*	*enterica*
Z0019_LO_H06_2_H03_B	332	77	LMG 25212	*Polynucleobacter*	*cosmopolitanus*	
Z0019_LB_A09_2_D02_A	332	77	LMG 25212	*Polynucleobacter*	*cosmopolitanus*	
Z0019_LO_H08_2_H04_B	332	77	LMG 25212	*Polynucleobacter*	*cosmopolitanus*	
Z0019_LO_H07_2_H04_A	332	77	LMG 25212	*Polynucleobacter*	*cosmopolitanus*	
Z0019_LO_H04_2_H02_B	332	77	LMG 25212	*Polynucleobacter*	*cosmopolitanus*	
Z0019_LO_H05_2_H03_A	332	77	LMG 25212	*Polynucleobacter*	*cosmopolitanus*	
Z0019_LB_A07_2_D03_A	332	77	LMG 25212	*Polynucleobacter*	*cosmopolitanus*	
Z0019_LB_A08_2_D03_B	332	77	LMG 25212	*Polynucleobacter*	*cosmopolitanus*	
Z0019_LO_H03_2_H02_A	332	77	LMG 25212	*Polynucleobacter*	*cosmopolitanus*	
Z0019_LB_A06_2_D04_B	332	77	LMG 25212	*Polynucleobacter*	*cosmopolitanus*	
Z0019_LB_A04_2_D05_B	332	77	LMG 25212	*Polynucleobacter*	*cosmopolitanus*	
Z0019_LB_A05_2_D04_A	332	77	LMG 25212	*Polynucleobacter*	*cosmopolitanus*	
Z0019_LO_H09_2_H05_A	332	77	LMG 25212	*Polynucleobacter*	*cosmopolitanus*	
Z0019_LB_A10_2_D02_B	332	77	LMG 25212	*Polynucleobacter*	*cosmopolitanus*	
Z0019_LO_H10_2_H05_B	332	77	LMG 25212	*Polynucleobacter*	*cosmopolitanus*	
Z0019_LO_H11_2_H06_A	332	77	LMG 25212	*Polynucleobacter*	*cosmopolitanus*	
Z0019_LB_A01_2_D06_A	332	77	LMG 25212	*Polynucleobacter*	*cosmopolitanus*	
Z0019_RO_B09_2_E11_A	332	77	LMG 25212	*Polynucleobacter*	*cosmopolitanus*	
Z0019_LO_B02_2_E01_B	332	77	LMG 25212	*Polynucleobacter*	*cosmopolitanus*	
Z0019_LO_A11_1_E06_A	332	77	LMG 25212	*Polynucleobacter*	*cosmopolitanus*	
Z0019_LO_A07_1_E04_A	332	77	LMG 25212	*Polynucleobacter*	*cosmopolitanus*	
Z0019_LB_A02_2_D06_B	332	77	LMG 25212	*Polynucleobacter*	*cosmopolitanus*	
Z0019_LB_A03_2_D05_A	332	77	LMG 25212	*Polynucleobacter*	*cosmopolitanus*	
Z0019_LO_H02_2_H01_B	332	77	LMG 25212	*Polynucleobacter*	*cosmopolitanus*	
Z0019_RO_C09_1_F11_A	332	77	LMG 25212	*Polynucleobacter*	*cosmopolitanus*	
Z0019_LO_C02_1_F01_B	332	77	LMG 25212	*Polynucleobacter*	*cosmopolitanus*	
Z0019_RO_C08_1_F10_B	332	77	LMG 25212	*Polynucleobacter*	*cosmopolitanus*	

Z0019_LO_B04_2_E02_B	332	77	LMG 25212	*Polynucleobacter*	*cosmopolitanus*	
Z0019_RB_A01_2_D12_A	332	77	LMG 25212	*Polynucleobacter*	*cosmopolitanus*	
Z0019_LB_A11_2_D01_A	332	77	LMG 25212	*Polynucleobacter*	*cosmopolitanus*	
Z0019_LO_H12_2_H06_B	332	77	LMG 25212	*Polynucleobacter*	*cosmopolitanus*	
Z0019_LB_A12_2_D01_B	332	77	LMG 25212	*Polynucleobacter*	*cosmopolitanus*	
Z0014_RB_D09_1_C08_A	333	26	LMG 18294	*Helicobacter*	*fennelliae*	
Z0014_LB_D07_1_C03_A	333	26	LMG 18294	*Helicobacter*	*fennelliae*	
Z0014_LB_D05_1_C04_A	333	26	LMG 18294	*Helicobacter*	*fennelliae*	
Z0014_RB_E01_2_B12_A	333	26	LMG 18294	*Helicobacter*	*fennelliae*	
Z0014_RB_C09_2_C08_A	333	26	LMG 18294	*Helicobacter*	*fennelliae*	
Z0014_RB_C08_2_C09_B	333	26	LMG 18294	*Helicobacter*	*fennelliae*	
Z0014_RB_E03_2_B11_A	333	26	LMG 18294	*Helicobacter*	*fennelliae*	
Z0014_LB_D06_1_C04_B	333	26	LMG 18294	*Helicobacter*	*fennelliae*	
Z0014_RB_D12_1_C07_B	333	26	LMG 18294	*Helicobacter*	*fennelliae*	
Z0014_LB_D04_1_C05_B	333	26	LMG 18294	*Helicobacter*	*fennelliae*	
Z0014_LB_D08_1_C03_B	333	26	LMG 18294	*Helicobacter*	*fennelliae*	
Z0014_RB_D11_1_C07_A	333	26	LMG 18294	*Helicobacter*	*fennelliae*	
Z0014_RB_E02_2_B12_B	333	26	LMG 18294	*Helicobacter*	*fennelliae*	
Z0014_RB_D10_1_C08_B	333	26	LMG 18294	*Helicobacter*	*fennelliae*	
Z0014_LB_D09_1_C02_A	333	26	LMG 18294	*Helicobacter*	*fennelliae*	
Z0014_RB_D04_1_C11_B	333	26	LMG 18294	*Helicobacter*	*fennelliae*	
Z0014_RB_D01_1_C12_A	333	26	LMG 18294	*Helicobacter*	*fennelliae*	
Z0014_RB_D08_1_C09_B	333	26	LMG 18294	*Helicobacter*	*fennelliae*	
Z0014_LB_D03_1_C05_A	333	26	LMG 18294	*Helicobacter*	*fennelliae*	
Z0014_RB_C12_2_C07_B	333	26	LMG 18294	*Helicobacter*	*fennelliae*	
Z0014_LB_D02_1_C06_B	333	26	LMG 18294	*Helicobacter*	*fennelliae*	
Z0014_RB_D02_1_C12_B	333	26	LMG 18294	*Helicobacter*	*fennelliae*	
Z0014_LB_C11_2_C01_A	333	26	LMG 18294	*Helicobacter*	*fennelliae*	
Z0014_RB_C10_2_C08_B	333	26	LMG 18294	*Helicobacter*	*fennelliae*	
Z0014_LB_C10_2_C02_B	333	26	LMG 18294	*Helicobacter*	*fennelliae*	
Z0014_RB_D03_1_C11_A	333	26	LMG 18294	*Helicobacter*	*fennelliae*	
Z0014_LB_D01_1_C06_A	333	26	LMG 18294	*Helicobacter*	*fennelliae*	
Z0014_RB_C11_2_C07_A	333	26	LMG 18294	*Helicobacter*	*fennelliae*	
Z0014_LB_C12_2_C01_B	333	26	LMG 18294	*Helicobacter*	*fennelliae*	
Z0014_RB_D05_1_C10_A	333	26	LMG 18294	*Helicobacter*	*fennelliae*	
Z0014_RB_D06_1_C10_B	333	26	LMG 18294	*Helicobacter*	*fennelliae*	
Z0014_RB_D07_1_C09_A	333	26	LMG 18294	*Helicobacter*	*fennelliae*	
Z0015_LO_H08_2_H04_B	334	33	LMG 2095	*Klebsiella*	*pneumoniae*	*pneumoniae*
Z0015_LO_H01_2_H01_A	334	33	LMG 2095	*Klebsiella*	*pneumoniae*	*pneumoniae*
Z0015_LO_H09_2_H05_A	334	33	LMG 2095	*Klebsiella*	*pneumoniae*	*pneumoniae*
Z0015_LB_B04_1_D05_B	334	33	LMG 2095	*Klebsiella*	*pneumoniae*	*pneumoniae*
Z0015_LO_H10_2_H05_B	334	33	LMG 2095	*Klebsiella*	*pneumoniae*	*pneumoniae*
Z0015_LB_B03_1_D05_A	334	33	LMG 2095	*Klebsiella*	*pneumoniae*	*pneumoniae*
Z0015_LB_A07_2_D03_A	334	33	LMG 2095	*Klebsiella*	*pneumoniae*	*pneumoniae*
Z0015_LB_A08_2_D03_B	334	33	LMG 2095	*Klebsiella*	*pneumoniae*	*pneumoniae*
Z0015_LO_H02_2_H01_B	334	33	LMG 2095	*Klebsiella*	*pneumoniae*	*pneumoniae*
Z0015_LB_A11_2_D01_A	334	33	LMG 2095	*Klebsiella*	*pneumoniae*	*pneumoniae*
Z0015_LB_A09_2_D02_A	334	33	LMG 2095	*Klebsiella*	*pneumoniae*	*pneumoniae*
Z0015_LB_A10_2_D02_B	334	33	LMG 2095	*Klebsiella*	*pneumoniae*	*pneumoniae*
Z0015_LB_A12_2_D01_B	334	33	LMG 2095	*Klebsiella*	*pneumoniae*	*pneumoniae*

Z0015_LO_H04_2_H02_B	334	33	LMG 2095	*Klebsiella*	*pneumoniae*	*pneumoniae*
Z0015_LO_H05_2_H03_A	334	33	LMG 2095	*Klebsiella*	*pneumoniae*	*pneumoniae*
Z0015_LO_H03_2_H02_A	334	33	LMG 2095	*Klebsiella*	*pneumoniae*	*pneumoniae*
Z0015_LO_G11_1_H06_A	334	33	LMG 2095	*Klebsiella*	*pneumoniae*	*pneumoniae*
Z0015_LB_A01_2_D06_A	334	33	LMG 2095	*Klebsiella*	*pneumoniae*	*pneumoniae*
Z0015_LO_H07_2_H04_A	334	33	LMG 2095	*Klebsiella*	*pneumoniae*	*pneumoniae*
Z0015_LO_G12_1_H06_B	334	33	LMG 2095	*Klebsiella*	*pneumoniae*	*pneumoniae*
Z0015_LB_A06_2_D04_B	334	33	LMG 2095	*Klebsiella*	*pneumoniae*	*pneumoniae*
Z0015_LB_A03_2_D05_A	334	33	LMG 2095	*Klebsiella*	*pneumoniae*	*pneumoniae*
Z0015_LB_A04_2_D05_B	334	33	LMG 2095	*Klebsiella*	*pneumoniae*	*pneumoniae*
Z0015_LB_A05_2_D04_A	334	33	LMG 2095	*Klebsiella*	*pneumoniae*	*pneumoniae*
Z0015_LB_A02_2_D06_B	334	33	LMG 2095	*Klebsiella*	*pneumoniae*	*pneumoniae*
Z0015_LO_H11_2_H06_A	334	33	LMG 2095	*Klebsiella*	*pneumoniae*	*pneumoniae*
Z0015_LB_B07_1_D03_A	334	33	LMG 2095	*Klebsiella*	*pneumoniae*	*pneumoniae*
Z0015_LB_B06_1_D04_B	334	33	LMG 2095	*Klebsiella*	*pneumoniae*	*pneumoniae*
Z0015_LB_B05_1_D04_A	334	33	LMG 2095	*Klebsiella*	*pneumoniae*	*pneumoniae*
Z0015_LO_H06_2_H03_B	334	33	LMG 2095	*Klebsiella*	*pneumoniae*	*pneumoniae*
Z0015_LB_B02_1_D06_B	334	33	LMG 2095	*Klebsiella*	*pneumoniae*	*pneumoniae*
Z0015_LB_B01_1_D06_A	334	33	LMG 2095	*Klebsiella*	*pneumoniae*	*pneumoniae*
Z0020_LO_G01_1_H01_A	335	95	LMG 2698	*Brenneria*	*salicis*	
Z0020_LO_F08_2_G04_B	335	95	LMG 2698	*Brenneria*	*salicis*	
Z0020_LO_F11_2_G06_A	335	95	LMG 2698	*Brenneria*	*salicis*	
Z0020_LO_E06_1_G03_B	335	95	LMG 2698	*Brenneria*	*salicis*	
Z0020_LO_F01_2_G01_A	335	95	LMG 2698	*Brenneria*	*salicis*	
Z0020_LO_E11_1_G06_A	335	95	LMG 2698	*Brenneria*	*salicis*	
Z0020_RO_D09_2_F11_A	335	95	LMG 2698	*Brenneria*	*salicis*	
Z0020_LO_E08_1_G04_B	335	95	LMG 2698	*Brenneria*	*salicis*	
Z0020_RO_E05_1_G09_A	335	95	LMG 2698	*Brenneria*	*salicis*	
Z0020_LO_D07_2_F04_A	335	95	LMG 2698	*Brenneria*	*salicis*	
Z0020_LO_D06_2_F03_B	335	95	LMG 2698	*Brenneria*	*salicis*	
Z0020_RO_E11_1_G12_A	335	95	LMG 2698	*Brenneria*	*salicis*	
Z0020_RO_F02_2_G07_B	335	95	LMG 2698	*Brenneria*	*salicis*	
Z0020_LO_F03_2_G02_A	335	95	LMG 2698	*Brenneria*	*salicis*	
Z0020_RO_F07_2_G10_A	335	95	LMG 2698	*Brenneria*	*salicis*	
Z0020_LO_F05_2_G03_A	335	95	LMG 2698	*Brenneria*	*salicis*	
Z0020_LO_F04_2_G02_B	335	95	LMG 2698	*Brenneria*	*salicis*	
Z0020_RO_F05_2_G09_A	335	95	LMG 2698	*Brenneria*	*salicis*	
Z0020_RO_F09_2_G11_A	335	95	LMG 2698	*Brenneria*	*salicis*	
Z0020_RO_E06_1_G09_B	335	95	LMG 2698	*Brenneria*	*salicis*	
Z0020_LO_F09_2_G05_A	335	95	LMG 2698	*Brenneria*	*salicis*	
Z0020_RO_F04_2_G08_B	335	95	LMG 2698	*Brenneria*	*salicis*	
Z0020_LO_D09_2_F05_A	335	95	LMG 2698	*Brenneria*	*salicis*	
Z0020_LO_E03_1_G02_A	335	95	LMG 2698	*Brenneria*	*salicis*	
Z0020_RO_D12_2_F12_B	335	95	LMG 2698	*Brenneria*	*salicis*	
Z0020_RO_D11_2_F12_A	335	95	LMG 2698	*Brenneria*	*salicis*	
Z0020_RO_E03_1_G08_A	335	95	LMG 2698	*Brenneria*	*salicis*	
Z0020_RO_D08_2_F10_B	335	95	LMG 2698	*Brenneria*	*salicis*	
Z0020_LO_E05_1_G03_A	336	95	LMG 2698	*Brenneria*	*salicis*	
Z0020_LO_E02_1_G01_B	336	95	LMG 2698	*Brenneria*	*salicis*	
Z0020_LO_D12_2_F06_B	336	95	LMG 2698	*Brenneria*	*salicis*	

Z0020_RO_E08_1_G10_B	336	95	LMG 2698	*Brenneria*	*salicis*
Z0018_RB_A12_2_D07_B	337	68	LMG 24392	*Granulibacter*	*bethesdensis*
Z0018_RB_A11_2_D07_A	337	68	LMG 24392	*Granulibacter*	*bethesdensis*
Z0018_RB_B01_1_D12_A	337	68	LMG 24392	*Granulibacter*	*bethesdensis*
Z0018_LB_B09_1_D02_A	337	68	LMG 24392	*Granulibacter*	*bethesdensis*
Z0018_LB_B02_1_D06_B	337	68	LMG 24392	*Granulibacter*	*bethesdensis*
Z0018_LB_B03_1_D05_A	337	68	LMG 24392	*Granulibacter*	*bethesdensis*
Z0018_LB_B01_1_D06_A	337	68	LMG 24392	*Granulibacter*	*bethesdensis*
Z0018_RB_A08_2_D09_B	337	68	LMG 24392	*Granulibacter*	*bethesdensis*
Z0018_RB_A07_2_D09_A	337	68	LMG 24392	*Granulibacter*	*bethesdensis*
Z0018_LB_B06_1_D04_B	337	68	LMG 24392	*Granulibacter*	*bethesdensis*
Z0018_LB_B04_1_D05_B	337	68	LMG 24392	*Granulibacter*	*bethesdensis*
Z0018_RB_B03_1_D11_A	337	68	LMG 24392	*Granulibacter*	*bethesdensis*
Z0018_LB_B05_1_D04_A	337	68	LMG 24392	*Granulibacter*	*bethesdensis*
Z0018_RB_A09_2_D08_A	337	68	LMG 24392	*Granulibacter*	*bethesdensis*
Z0018_LB_C05_2_C04_A	337	68	LMG 24392	*Granulibacter*	*bethesdensis*
Z0018_LB_B08_1_D03_B	337	68	LMG 24392	*Granulibacter*	*bethesdensis*
Z0018_LB_B07_1_D03_A	337	68	LMG 24392	*Granulibacter*	*bethesdensis*
Z0018_RB_A10_2_D08_B	337	68	LMG 24392	*Granulibacter*	*bethesdensis*
Z0018_RB_B02_1_D12_B	337	68	LMG 24392	*Granulibacter*	*bethesdensis*
Z0018_LB_B11_1_D01_A	337	68	LMG 24392	*Granulibacter*	*bethesdensis*
Z0018_LB_B10_1_D02_B	337	68	LMG 24392	*Granulibacter*	*bethesdensis*
Z0018_LB_C06_2_C04_B	337	68	LMG 24392	*Granulibacter*	*bethesdensis*
Z0018_LB_C07_2_C03_A	337	68	LMG 24392	*Granulibacter*	*bethesdensis*
Z0018_RB_B07_1_D09_A	337	68	LMG 24392	*Granulibacter*	*bethesdensis*
Z0018_RB_B05_1_D10_A	337	68	LMG 24392	*Granulibacter*	*bethesdensis*
Z0018_LB_C04_2_C05_B	337	68	LMG 24392	*Granulibacter*	*bethesdensis*
Z0018_LB_C03_2_C05_A	337	68	LMG 24392	*Granulibacter*	*bethesdensis*
Z0018_LB_C01_2_C06_A	337	68	LMG 24392	*Granulibacter*	*bethesdensis*
Z0018_LB_C02_2_C06_B	337	68	LMG 24392	*Granulibacter*	*bethesdensis*
Z0018_RB_B06_1_D10_B	337	68	LMG 24392	*Granulibacter*	*bethesdensis*
Z0018_LB_B12_1_D01_B	337	68	LMG 24392	*Granulibacter*	*bethesdensis*
Z0018_RB_B04_1_D11_B	337	68	LMG 24392	*Granulibacter*	*bethesdensis*
