# Supplementary material for: Introducing SPeDE: High-Throughput Dereplication and Accurate Determination of Microbial Diversity from Matrix-Assisted Laser Desorption–Ionization Time of Flight Mass Spectrometry Data
Source: mSystems. 2019 Sep 10;4(5):e00437-19. doi: 10.1128/mSystems.00437-19 (PMC6739102; doi:10.1128/mSystems.00437-19)
Supplement: TABLE S4 [file mSystems.00437-19-st004.pdf]

Table S4: Results on the benchmark study analyzed by methods of Strejcek et. al 2018.

Spectrum	Cluster number	OTU	Strain	Genus	Species	Subspecies
Z0010_LB_A01_2_D06_A	1	147	R-68806	Burkholderia	cenocepacia	IIIA
Z0010_LB_A02_2_D06_B	1	147	R-68806	Burkholderia	cenocepacia	IIIA
Z0010_LB_A03_2_D05_A	1	147	R-68806	Burkholderia	cenocepacia	IIIA
Z0010_LB_A04_2_D05_B	1	147	R-68806	Burkholderia	cenocepacia	IIIA
Z0010_LB_A05_2_D04_A	1	147	R-68806	Burkholderia	cenocepacia	IIIA
Z0010_LB_A06_2_D04_B	1	147	R-68806	Burkholderia	cenocepacia	IIIA
Z0010_LB_A07_2_D03_A	1	147	R-68806	Burkholderia	cenocepacia	IIIA
Z0010_LB_A08_2_D03_B	1	147	R-68806	Burkholderia	cenocepacia	IIIA
Z0010_LB_A09_2_D02_A	1	147	R-68806	Burkholderia	cenocepacia	IIIA
Z0010_LB_A10_2_D02_B	1	147	R-68806	Burkholderia	cenocepacia	IIIA
Z0010_LB_A11_2_D01_A	1	147	R-68806	Burkholderia	cenocepacia	IIIA
Z0010_LB_A12_2_D01_B	1	147	R-68806	Burkholderia	cenocepacia	IIIA
Z0010_LB_B01_1_D06_A	1	147	R-68806	Burkholderia	cenocepacia	IIIA
Z0010_LB_E03_2_B05_A	1	147	R-71051	Burkholderia	cenocepacia	IIIA
Z0010_LB_E04_2_B05_B	1	147	R-71051	Burkholderia	cenocepacia	IIIA
Z0010_LB_E05_2_B04_A	1	147	R-71051	Burkholderia	cenocepacia	IIIA
Z0010_LB_E06_2_B04_B	1	147	R-71051	Burkholderia	cenocepacia	IIIA
Z0010_LB_E07_2_B03_A	1	147	R-71051	Burkholderia	cenocepacia	IIIA
Z0010_LB_E08_2_B03_B	1	147	R-71051	Burkholderia	cenocepacia	IIIA
Z0010_LB_E09_2_B02_A	1	147	R-71051	Burkholderia	cenocepacia	IIIA
Z0010_LB_E10_2_B02_B	1	147	R-71051	Burkholderia	cenocepacia	IIIA
Z0010_LB_E11_2_B01_A	1	147	R-71051	Burkholderia	cenocepacia	IIIA
Z0010_LB_E12_2_B01_B	1	147	R-71051	Burkholderia	cenocepacia	IIIA
Z0010_LB_F01_1_B06_A	1	147	R-71051	Burkholderia	cenocepacia	IIIA
Z0010_LB_F02_1_B06_B	1	147	R-71051	Burkholderia	cenocepacia	IIIA
Z0010_LB_F03_1_B05_A	1	147	R-71051	Burkholderia	cenocepacia	IIIA
Z0010_LB_F04_1_B05_B	1	147	R-71051	Burkholderia	cenocepacia	IIIA
Z0010_LB_F05_1_B04_A	1	147	R-71051	Burkholderia	cenocepacia	IIIA
Z0010_LB_F06_1_B04_B	1	147	R-71051	Burkholderia	cenocepacia	IIIA
Z0010_LB_G12_2_A01_B	1	147	R-71085	Burkholderia	cenocepacia	IIIA
Z0010_LB_H01_1_A06_A	1	147	R-71085	Burkholderia	cenocepacia	IIIA
Z0010_LB_H02_1_A06_B	1	147	R-71085	Burkholderia	cenocepacia	IIIA
Z0010_LB_H03_1_A05_A	1	147	R-71085	Burkholderia	cenocepacia	IIIA
Z0010_LB_H04_1_A05_B	1	147	R-71085	Burkholderia	cenocepacia	IIIA
Z0010_LB_H05_1_A04_A	1	147	R-71085	Burkholderia	cenocepacia	IIIA
Z0010_LB_H06_1_A04_B	1	147	R-71085	Burkholderia	cenocepacia	IIIA
Z0010_LB_H07_1_A03_A	1	147	R-71085	Burkholderia	cenocepacia	IIIA
Z0010_LB_H08_1_A03_B	1	147	R-71085	Burkholderia	cenocepacia	IIIA
Z0010_LB_H09_1_A02_A	1	147	R-71085	Burkholderia	cenocepacia	IIIA
Z0010_LB_H10_1_A02_B	1	147	R-71085	Burkholderia	cenocepacia	IIIA
Z0010_LB_H11_1_A01_A	1	147	R-71085	Burkholderia	cenocepacia	IIIA
Z0010_LB_H12_1_A01_B	1	147	R-71085	Burkholderia	cenocepacia	IIIA
Z0010_LO_A01_1_E01_A	1	147	R-71085	Burkholderia	cenocepacia	IIIA
Z0010_LO_D01_2_F01_A	1	147	R-68806	Burkholderia	cenocepacia	IIIA
Z0010_LO_D04_2_F02_B	1	147	R-68806	Burkholderia	cenocepacia	IIIA

Z0010_LO_D06_2_F03_B	1	147	R-68806	Burkholderia	cenocepacia	IIIA
Z0010_RB_A02_2_D12_B	1	147	R-68806	Burkholderia	cenocepacia	IIIA
Z0010_RB_A03_2_D11_A	1	147	R-68806	Burkholderia	cenocepacia	IIIA
Z0010_RB_A04_2_D11_B	1	147	R-68806	Burkholderia	cenocepacia	IIIA
Z0010_RB_A05_2_D10_A	1	147	R-68806	Burkholderia	cenocepacia	IIIA
Z0010_RB_A06_2_D10_B	1	147	R-68806	Burkholderia	cenocepacia	IIIA
Z0010_RB_A07_2_D09_A	1	147	R-68806	Burkholderia	cenocepacia	IIIA
Z0010_RB_A08_2_D09_B	1	147	R-68806	Burkholderia	cenocepacia	IIIA
Z0010_RB_A09_2_D08_A	1	147	R-68806	Burkholderia	cenocepacia	IIIA
Z0010_RB_A10_2_D08_B	1	147	R-68806	Burkholderia	cenocepacia	IIIA
Z0010_RB_A11_2_D07_A	1	147	R-68806	Burkholderia	cenocepacia	IIIA
Z0010_RB_A12_2_D07_B	1	147	R-68806	Burkholderia	cenocepacia	IIIA
Z0010_RB_D04_1_C11_B	1	147	R-71051	Burkholderia	cenocepacia	IIIA
Z0010_RB_D05_1_C10_A	1	147	R-71051	Burkholderia	cenocepacia	IIIA
Z0010_RB_D06_1_C10_B	1	147	R-71051	Burkholderia	cenocepacia	IIIA
Z0010_RB_D07_1_C09_A	1	147	R-71051	Burkholderia	cenocepacia	IIIA
Z0010_RB_D08_1_C09_B	1	147	R-71051	Burkholderia	cenocepacia	IIIA
Z0010_RB_D09_1_C08_A	1	147	R-71051	Burkholderia	cenocepacia	IIIA
Z0010_RB_D10_1_C08_B	1	147	R-71051	Burkholderia	cenocepacia	IIIA
Z0010_RB_D11_1_C07_A	1	147	R-71051	Burkholderia	cenocepacia	IIIA
Z0010_RB_D12_1_C07_B	1	147	R-71051	Burkholderia	cenocepacia	IIIA
Z0010_RB_E01_2_B12_A	1	147	R-71051	Burkholderia	cenocepacia	IIIA
Z0010_RB_E02_2_B12_B	1	147	R-71051	Burkholderia	cenocepacia	IIIA
Z0010_RB_E03_2_B11_A	1	147	R-71051	Burkholderia	cenocepacia	IIIA
Z0010_RB_E04_2_B11_B	1	147	R-71051	Burkholderia	cenocepacia	IIIA
Z0010_RB_E05_2_B10_A	1	147	R-71051	Burkholderia	cenocepacia	IIIA
Z0010_RB_E06_2_B10_B	1	147	R-71051	Burkholderia	cenocepacia	IIIA
Z0010_RB_E07_2_B09_A	1	147	R-71051	Burkholderia	cenocepacia	IIIA
Z0010_RB_F11_1_B07_A	1	147	R-71085	Burkholderia	cenocepacia	IIIA
Z0010_RB_F12_1_B07_B	1	147	R-71085	Burkholderia	cenocepacia	IIIA
Z0010_RB_G01_2_A12_A	1	147	R-71085	Burkholderia	cenocepacia	IIIA
Z0010_RB_G02_2_A12_B	1	147	R-71085	Burkholderia	cenocepacia	IIIA
Z0010_RB_G03_2_A11_A	1	147	R-71085	Burkholderia	cenocepacia	IIIA
Z0010_RB_G04_2_A11_B	1	147	R-71085	Burkholderia	cenocepacia	IIIA
Z0010_RB_G05_2_A10_A	1	147	R-71085	Burkholderia	cenocepacia	IIIA
Z0010_RB_G06_2_A10_B	1	147	R-71085	Burkholderia	cenocepacia	IIIA
Z0010_RB_G07_2_A09_A	1	147	R-71085	Burkholderia	cenocepacia	IIIA
Z0010_RB_G08_2_A09_B	1	147	R-71085	Burkholderia	cenocepacia	IIIA
Z0010_RB_G09_2_A08_A	1	147	R-71085	Burkholderia	cenocepacia	IIIA
Z0010_RB_G10_2_A08_B	1	147	R-71085	Burkholderia	cenocepacia	IIIA
Z0010_RB_G11_2_A07_A	1	147	R-71085	Burkholderia	cenocepacia	IIIA
Z0010_RB_G12_2_A07_B	1	147	R-71085	Burkholderia	cenocepacia	IIIA
Z0010_RB_H01_1_A12_A	1	147	R-71085	Burkholderia	cenocepacia	IIIA
Z0010_RB_H02_1_A12_B	1	147	R-71085	Burkholderia	cenocepacia	IIIA
Z0010_RB_H03_1_A11_A	1	147	R-71085	Burkholderia	cenocepacia	IIIA
Z0010_RB_H04_1_A11_B	1	147	R-71085	Burkholderia	cenocepacia	IIIA
Z0010_RO_C05_1_F09_A	1	147	R-68806	Burkholderia	cenocepacia	IIIA
Z0010_RO_C06_1_F09_B	1	147	R-68806	Burkholderia	cenocepacia	IIIA
Z0010_RO_C08_1_F10_B	1	147	R-68806	Burkholderia	cenocepacia	IIIA
Z0010_RO_C10_1_F11_B	1	147	R-68806	Burkholderia	cenocepacia	IIIA

Z0010_RO_C12_1_F12_B	1	147	R-68806	Burkholderia	cenocepacia	IIIA
Z0011_LB_B06_1_D04_B	1	145	R-67259	Burkholderia	cenocepacia	IIIB
Z0011_LB_B07_1_D03_A	1	145	R-67259	Burkholderia	cenocepacia	IIIB
Z0011_LB_B08_1_D03_B	1	145	R-67259	Burkholderia	cenocepacia	IIIB
Z0011_LB_B09_1_D02_A	1	145	R-67259	Burkholderia	cenocepacia	IIIB
Z0011_LB_B10_1_D02_B	1	145	R-67259	Burkholderia	cenocepacia	IIIB
Z0011_LB_B11_1_D01_A	1	145	R-67259	Burkholderia	cenocepacia	IIIB
Z0011_LB_B12_1_D01_B	1	145	R-67259	Burkholderia	cenocepacia	IIIB
Z0011_LB_C01_2_C06_A	1	145	R-67259	Burkholderia	cenocepacia	IIIB
Z0011_LB_C02_2_C06_B	1	145	R-67259	Burkholderia	cenocepacia	IIIB
Z0011_LB_C03_2_C05_A	1	145	R-67259	Burkholderia	cenocepacia	IIIB
Z0011_LB_C04_2_C05_B	1	145	R-67259	Burkholderia	cenocepacia	IIIB
Z0011_LB_C05_2_C04_A	1	145	R-67259	Burkholderia	cenocepacia	IIIB
Z0011_LB_C06_2_C04_B	1	145	R-67259	Burkholderia	cenocepacia	IIIB
Z0011_LB_C07_2_C03_A	1	145	R-67259	Burkholderia	cenocepacia	IIIB
Z0011_LB_C08_2_C03_B	1	145	R-67259	Burkholderia	cenocepacia	IIIB
Z0011_LB_C09_2_C02_A	1	145	R-67259	Burkholderia	cenocepacia	IIIB
Z0011_LB_C10_2_C02_B	1	145	R-67259	Burkholderia	cenocepacia	IIIB
Z0011_LB_C11_2_C01_A	1	145	R-67259	Burkholderia	cenocepacia	IIIB
Z0011_LB_C12_2_C01_B	1	145	R-67259	Burkholderia	cenocepacia	IIIB
Z0011_LB_E07_2_B03_A	1	145	R-67581	Burkholderia	cenocepacia	IIIB
Z0011_LB_E08_2_B03_B	1	145	R-67581	Burkholderia	cenocepacia	IIIB
Z0011_LB_E09_2_B02_A	1	145	R-67581	Burkholderia	cenocepacia	IIIB
Z0011_LB_E10_2_B02_B	1	145	R-67581	Burkholderia	cenocepacia	IIIB
Z0011_LB_E11_2_B01_A	1	145	R-67581	Burkholderia	cenocepacia	IIIB
Z0011_LB_E12_2_B01_B	1	145	R-67581	Burkholderia	cenocepacia	IIIB
Z0011_LB_F01_1_B06_A	1	145	R-67581	Burkholderia	cenocepacia	IIIB
Z0011_LB_F02_1_B06_B	1	145	R-67581	Burkholderia	cenocepacia	IIIB
Z0011_LB_F03_1_B05_A	1	145	R-67581	Burkholderia	cenocepacia	IIIB
Z0011_LB_F04_1_B05_B	1	145	R-67581	Burkholderia	cenocepacia	IIIB
Z0011_LB_F05_1_B04_A	1	145	R-67581	Burkholderia	cenocepacia	IIIB
Z0011_LB_F06_1_B04_B	1	145	R-67581	Burkholderia	cenocepacia	IIIB
Z0011_LB_F07_1_B03_A	1	145	R-67581	Burkholderia	cenocepacia	IIIB
Z0011_LB_F08_1_B03_B	1	145	R-67581	Burkholderia	cenocepacia	IIIB
Z0011_LB_F09_1_B02_A	1	145	R-67581	Burkholderia	cenocepacia	IIIB
Z0011_LB_F10_1_B02_B	1	145	R-67581	Burkholderia	cenocepacia	IIIB
Z0011_LB_F11_1_B01_A	1	146	R-68591	Burkholderia	cenocepacia	IIIB
Z0011_LB_F12_1_B01_B	1	146	R-68591	Burkholderia	cenocepacia	IIIB
Z0011_LB_G01_2_A06_A	1	146	R-68591	Burkholderia	cenocepacia	IIIB
Z0011_LB_G02_2_A06_B	1	146	R-68591	Burkholderia	cenocepacia	IIIB
Z0011_LB_G03_2_A05_A	1	146	R-68591	Burkholderia	cenocepacia	IIIB
Z0011_LB_G04_2_A05_B	1	146	R-68591	Burkholderia	cenocepacia	IIIB
Z0011_LB_G05_2_A04_A	1	146	R-68591	Burkholderia	cenocepacia	IIIB
Z0011_LB_G06_2_A04_B	1	146	R-68591	Burkholderia	cenocepacia	IIIB
Z0011_LB_G07_2_A03_A	1	146	R-68591	Burkholderia	cenocepacia	IIIB
Z0011_LB_G08_2_A03_B	1	146	R-68591	Burkholderia	cenocepacia	IIIB
Z0011_LB_G09_2_A02_A	1	146	R-68591	Burkholderia	cenocepacia	IIIB
Z0011_LB_G10_2_A02_B	1	146	R-68591	Burkholderia	cenocepacia	IIIB
Z0011_LB_G11_2_A01_A	1	146	R-68591	Burkholderia	cenocepacia	IIIB
Z0011_LB_G12_2_A01_B	1	146	R-68591	Burkholderia	cenocepacia	IIIB

Z0011_LB_H01_1_A06_A	1	146	R-68599	Burkholderia	cenocepacia	IIIB
Z0011_LB_H02_1_A06_B	1	146	R-68599	Burkholderia	cenocepacia	IIIB
Z0011_LB_H03_1_A05_A	1	146	R-68599	Burkholderia	cenocepacia	IIIB
Z0011_LB_H04_1_A05_B	1	146	R-68599	Burkholderia	cenocepacia	IIIB
Z0011_LB_H05_1_A04_A	1	146	R-68599	Burkholderia	cenocepacia	IIIB
Z0011_LB_H06_1_A04_B	1	146	R-68599	Burkholderia	cenocepacia	IIIB
Z0011_LB_H07_1_A03_A	1	146	R-68599	Burkholderia	cenocepacia	IIIB
Z0011_LB_H08_1_A03_B	1	146	R-68599	Burkholderia	cenocepacia	IIIB
Z0011_LB_H09_1_A02_A	1	146	R-68599	Burkholderia	cenocepacia	IIIB
Z0011_LB_H10_1_A02_B	1	146	R-68599	Burkholderia	cenocepacia	IIIB
Z0011_LB_H11_1_A01_A	1	146	R-68599	Burkholderia	cenocepacia	IIIB
Z0011_LB_H12_1_A01_B	1	146	R-68599	Burkholderia	cenocepacia	IIIB
Z0011_LO_A01_1_E01_A	1	146	R-68599	Burkholderia	cenocepacia	IIIB
Z0011_LO_A02_1_E01_B	1	146	R-68599	Burkholderia	cenocepacia	IIIB
Z0011_LO_A03_1_E02_A	1	146	R-68599	Burkholderia	cenocepacia	IIIB
Z0011_LO_A04_1_E02_B	1	146	R-68599	Burkholderia	cenocepacia	IIIB
Z0011_LO_A05_1_E03_A	1	147	R-68675	Burkholderia	cenocepacia	IIIA
Z0011_LO_A06_1_E03_B	1	147	R-68675	Burkholderia	cenocepacia	IIIA
Z0011_LO_A07_1_E04_A	1	147	R-68675	Burkholderia	cenocepacia	IIIA
Z0011_LO_A08_1_E04_B	1	147	R-68675	Burkholderia	cenocepacia	IIIA
Z0011_LO_A09_1_E05_A	1	147	R-68675	Burkholderia	cenocepacia	IIIA
Z0011_LO_A10_1_E05_B	1	147	R-68675	Burkholderia	cenocepacia	IIIA
Z0011_LO_A11_1_E06_A	1	147	R-68675	Burkholderia	cenocepacia	IIIA
Z0011_LO_A12_1_E06_B	1	147	R-68675	Burkholderia	cenocepacia	IIIA
Z0011_LO_B01_2_E01_A	1	147	R-68675	Burkholderia	cenocepacia	IIIA
Z0011_LO_B02_2_E01_B	1	147	R-68675	Burkholderia	cenocepacia	IIIA
Z0011_LO_B03_2_E02_A	1	147	R-68675	Burkholderia	cenocepacia	IIIA
Z0011_LO_B04_2_E02_B	1	147	R-68675	Burkholderia	cenocepacia	IIIA
Z0011_LO_B05_2_E03_A	1	147	R-68675	Burkholderia	cenocepacia	IIIA
Z0011_LO_B06_2_E03_B	1	147	R-68675	Burkholderia	cenocepacia	IIIA
Z0011_RB_A04_2_D11_B	1	145	R-67259	Burkholderia	cenocepacia	IIIB
Z0011_RB_A05_2_D10_A	1	145	R-67259	Burkholderia	cenocepacia	IIIB
Z0011_RB_A06_2_D10_B	1	145	R-67259	Burkholderia	cenocepacia	IIIB
Z0011_RB_A07_2_D09_A	1	145	R-67259	Burkholderia	cenocepacia	IIIB
Z0011_RB_A08_2_D09_B	1	145	R-67259	Burkholderia	cenocepacia	IIIB
Z0011_RB_A09_2_D08_A	1	145	R-67259	Burkholderia	cenocepacia	IIIB
Z0011_RB_A10_2_D08_B	1	145	R-67259	Burkholderia	cenocepacia	IIIB
Z0011_RB_A11_2_D07_A	1	145	R-67259	Burkholderia	cenocepacia	IIIB
Z0011_RB_A12_2_D07_B	1	145	R-67259	Burkholderia	cenocepacia	IIIB
Z0011_RB_B01_1_D12_A	1	145	R-67259	Burkholderia	cenocepacia	IIIB
Z0011_RB_B02_1_D12_B	1	145	R-67259	Burkholderia	cenocepacia	IIIB
Z0011_RB_B03_1_D11_A	1	145	R-67259	Burkholderia	cenocepacia	IIIB
Z0011_RB_B04_1_D11_B	1	145	R-67259	Burkholderia	cenocepacia	IIIB
Z0011_RB_C07_2_C09_A	1	145	R-67581	Burkholderia	cenocepacia	IIIB
Z0011_RB_C08_2_C09_B	1	145	R-67581	Burkholderia	cenocepacia	IIIB
Z0011_RB_C09_2_C08_A	1	145	R-67581	Burkholderia	cenocepacia	IIIB
Z0011_RB_C10_2_C08_B	1	145	R-67581	Burkholderia	cenocepacia	IIIB
Z0011_RB_C11_2_C07_A	1	145	R-67581	Burkholderia	cenocepacia	IIIB
Z0011_RB_C12_2_C07_B	1	145	R-67581	Burkholderia	cenocepacia	IIIB
Z0011_RB_D01_1_C12_A	1	145	R-67581	Burkholderia	cenocepacia	IIIB

Z0011_RB_D02_1_C12_B	1	145	R-67581	Burkholderia	cenocepacia	IIIB
Z0011_RB_D03_1_C11_A	1	145	R-67581	Burkholderia	cenocepacia	IIIB
Z0011_RB_D04_1_C11_B	1	145	R-67581	Burkholderia	cenocepacia	IIIB
Z0011_RB_D05_1_C10_A	1	145	R-67581	Burkholderia	cenocepacia	IIIB
Z0011_RB_D06_1_C10_B	1	145	R-67581	Burkholderia	cenocepacia	IIIB
Z0011_RB_D07_1_C09_A	1	145	R-67581	Burkholderia	cenocepacia	IIIB
Z0011_RB_D08_1_C09_B	1	145	R-67581	Burkholderia	cenocepacia	IIIB
Z0011_RB_D09_1_C08_A	1	145	R-67581	Burkholderia	cenocepacia	IIIB
Z0011_RB_D10_1_C08_B	1	145	R-67581	Burkholderia	cenocepacia	IIIB
Z0011_RB_D11_1_C07_A	1	146	R-68591	Burkholderia	cenocepacia	IIIB
Z0011_RB_D12_1_C07_B	1	146	R-68591	Burkholderia	cenocepacia	IIIB
Z0011_RB_E01_2_B12_A	1	146	R-68591	Burkholderia	cenocepacia	IIIB
Z0011_RB_E02_2_B12_B	1	146	R-68591	Burkholderia	cenocepacia	IIIB
Z0011_RB_E03_2_B11_A	1	146	R-68591	Burkholderia	cenocepacia	IIIB
Z0011_RB_E04_2_B11_B	1	146	R-68591	Burkholderia	cenocepacia	IIIB
Z0011_RB_E05_2_B10_A	1	146	R-68591	Burkholderia	cenocepacia	IIIB
Z0011_RB_E06_2_B10_B	1	146	R-68591	Burkholderia	cenocepacia	IIIB
Z0011_RB_E07_2_B09_A	1	146	R-68591	Burkholderia	cenocepacia	IIIB
Z0011_RB_E08_2_B09_B	1	146	R-68591	Burkholderia	cenocepacia	IIIB
Z0011_RB_E09_2_B08_A	1	146	R-68591	Burkholderia	cenocepacia	IIIB
Z0011_RB_E10_2_B08_B	1	146	R-68591	Burkholderia	cenocepacia	IIIB
Z0011_RB_E11_2_B07_A	1	146	R-68591	Burkholderia	cenocepacia	IIIB
Z0011_RB_E12_2_B07_B	1	146	R-68591	Burkholderia	cenocepacia	IIIB
Z0011_RB_F01_1_B12_A	1	146	R-68591	Burkholderia	cenocepacia	IIIB
Z0011_RB_F02_1_B12_B	1	146	R-68591	Burkholderia	cenocepacia	IIIB
Z0011_RB_F03_1_B11_A	1	146	R-68591	Burkholderia	cenocepacia	IIIB
Z0011_RB_F04_1_B11_B	1	146	R-68591	Burkholderia	cenocepacia	IIIB
Z0011_RB_F05_1_B10_A	1	146	R-68599	Burkholderia	cenocepacia	IIIB
Z0011_RB_F06_1_B10_B	1	146	R-68599	Burkholderia	cenocepacia	IIIB
Z0011_RB_F07_1_B09_A	1	146	R-68599	Burkholderia	cenocepacia	IIIB
Z0011_RB_F08_1_B09_B	1	146	R-68599	Burkholderia	cenocepacia	IIIB
Z0011_RB_F09_1_B08_A	1	146	R-68599	Burkholderia	cenocepacia	IIIB
Z0011_RB_F10_1_B08_B	1	146	R-68599	Burkholderia	cenocepacia	IIIB
Z0011_RB_F11_1_B07_A	1	146	R-68599	Burkholderia	cenocepacia	IIIB
Z0011_RB_F12_1_B07_B	1	146	R-68599	Burkholderia	cenocepacia	IIIB
Z0011_RB_G01_2_A12_A	1	146	R-68599	Burkholderia	cenocepacia	IIIB
Z0011_RB_G02_2_A12_B	1	146	R-68599	Burkholderia	cenocepacia	IIIB
Z0011_RB_G03_2_A11_A	1	146	R-68599	Burkholderia	cenocepacia	IIIB
Z0011_RB_G04_2_A11_B	1	146	R-68599	Burkholderia	cenocepacia	IIIB
Z0011_RB_G05_2_A10_A	1	146	R-68599	Burkholderia	cenocepacia	IIIB
Z0011_RB_G06_2_A10_B	1	146	R-68599	Burkholderia	cenocepacia	IIIB
Z0011_RB_G07_2_A09_A	1	146	R-68599	Burkholderia	cenocepacia	IIIB
Z0011_RB_G08_2_A09_B	1	146	R-68599	Burkholderia	cenocepacia	IIIB
Z0011_RB_G09_2_A08_A	1	147	R-68675	Burkholderia	cenocepacia	IIIA
Z0011_RB_G10_2_A08_B	1	147	R-68675	Burkholderia	cenocepacia	IIIA
Z0011_RB_G11_2_A07_A	1	147	R-68675	Burkholderia	cenocepacia	IIIA
Z0011_RB_G12_2_A07_B	1	147	R-68675	Burkholderia	cenocepacia	IIIA
Z0011_RB_H01_1_A12_A	1	147	R-68675	Burkholderia	cenocepacia	IIIA
Z0011_RB_H02_1_A12_B	1	147	R-68675	Burkholderia	cenocepacia	IIIA
Z0011_RB_H03_1_A11_A	1	147	R-68675	Burkholderia	cenocepacia	IIIA

Z0011_RB_H04_1_A11_B	1	147	R-68675	Burkholderia	cenocepacia	IIIA
Z0011_RB_H05_1_A10_A	1	147	R-68675	Burkholderia	cenocepacia	IIIA
Z0011_RB_H06_1_A10_B	1	147	R-68675	Burkholderia	cenocepacia	IIIA
Z0011_RB_H07_1_A09_A	1	147	R-68675	Burkholderia	cenocepacia	IIIA
Z0011_RB_H08_1_A09_B	1	147	R-68675	Burkholderia	cenocepacia	IIIA
Z0011_RB_H09_1_A08_A	1	147	R-68675	Burkholderia	cenocepacia	IIIA
Z0011_RB_H10_1_A08_B	1	147	R-68675	Burkholderia	cenocepacia	IIIA
Z0010_LB_B02_1_D06_B	2	148	R-69596	Burkholderia	cepacia	
Z0010_LB_B03_1_D05_A	2	148	R-69596	Burkholderia	cepacia	
Z0010_LB_B04_1_D05_B	2	148	R-69596	Burkholderia	cepacia	
Z0010_LB_B05_1_D04_A	2	148	R-69596	Burkholderia	cepacia	
Z0010_LB_B06_1_D04_B	2	148	R-69596	Burkholderia	cepacia	
Z0010_LB_B07_1_D03_A	2	148	R-69596	Burkholderia	cepacia	
Z0010_LB_B08_1_D03_B	2	148	R-69596	Burkholderia	cepacia	
Z0010_LB_B09_1_D02_A	2	148	R-69596	Burkholderia	cepacia	
Z0010_LB_B10_1_D02_B	2	148	R-69596	Burkholderia	cepacia	
Z0010_LB_B11_1_D01_A	2	148	R-69596	Burkholderia	cepacia	
Z0010_LB_B12_1_D01_B	2	148	R-69596	Burkholderia	cepacia	
Z0010_LB_C01_2_C06_A	2	148	R-69596	Burkholderia	cepacia	
Z0010_LB_C02_2_C06_B	2	148	R-69596	Burkholderia	cepacia	
Z0010_LB_C03_2_C05_A	2	148	R-69596	Burkholderia	cepacia	
Z0010_LB_C04_2_C05_B	2	148	R-69596	Burkholderia	cepacia	
Z0010_LB_C05_2_C04_A	2	148	R-69596	Burkholderia	cepacia	
Z0010_LB_C06_2_C04_B	2	148	R-69596	Burkholderia	cepacia	
Z0010_LB_C07_2_C03_A	2	148	R-69596	Burkholderia	cepacia	
Z0010_LB_C08_2_C03_B	2	148	R-69596	Burkholderia	cepacia	
Z0010_LO_B03_2_E02_A	2	141	R-50394	Burkholderia	stabilis	
Z0010_LO_B04_2_E02_B	2	141	R-50394	Burkholderia	stabilis	
Z0010_LO_B05_2_E03_A	2	141	R-50394	Burkholderia	stabilis	
Z0010_LO_B06_2_E03_B	2	141	R-50394	Burkholderia	stabilis	
Z0010_LO_B07_2_E04_A	2	141	R-50394	Burkholderia	stabilis	
Z0010_LO_B08_2_E04_B	2	141	R-50394	Burkholderia	stabilis	
Z0010_LO_B09_2_E05_A	2	141	R-50394	Burkholderia	stabilis	
Z0010_LO_B10_2_E05_B	2	141	R-50394	Burkholderia	stabilis	
Z0010_LO_B11_2_E06_A	2	141	R-50394	Burkholderia	stabilis	
Z0010_LO_B12_2_E06_B	2	141	R-50394	Burkholderia	stabilis	
Z0010_LO_C01_1_F01_A	2	141	R-50394	Burkholderia	stabilis	
Z0010_LO_C02_1_F01_B	2	141	R-50394	Burkholderia	stabilis	
Z0010_LO_C03_1_F02_A	2	141	R-50394	Burkholderia	stabilis	
Z0010_LO_C04_1_F02_B	2	141	R-50394	Burkholderia	stabilis	
Z0010_LO_C05_1_F03_A	2	141	R-50394	Burkholderia	stabilis	
Z0010_RB_B01_1_D12_A	2	148	R-69596	Burkholderia	cepacia	
Z0010_RB_B02_1_D12_B	2	148	R-69596	Burkholderia	cepacia	
Z0010_RB_B03_1_D11_A	2	148	R-69596	Burkholderia	cepacia	
Z0010_RB_B04_1_D11_B	2	148	R-69596	Burkholderia	cepacia	
Z0010_RB_B05_1_D10_A	2	148	R-69596	Burkholderia	cepacia	
Z0010_RB_B06_1_D10_B	2	148	R-69596	Burkholderia	cepacia	
Z0010_RB_B07_1_D09_A	2	148	R-69596	Burkholderia	cepacia	
Z0010_RB_B08_1_D09_B	2	148	R-69596	Burkholderia	cepacia	
Z0010_RB_B09_1_D08_A	2	148	R-69596	Burkholderia	cepacia	

Z0010_RB_B10_1_D08_B	2	148	R-69596	Burkholderia	cepacia
Z0010_RB_B11_1_D07_A	2	148	R-69596	Burkholderia	cepacia
Z0010_RB_B12_1_D07_B	2	148	R-69596	Burkholderia	cepacia
Z0010_RB_C01_2_C12_A	2	148	R-69596	Burkholderia	cepacia
Z0010_RO_A08_1_E10_B	2	141	R-50394	Burkholderia	stabilis
Z0010_RO_A09_1_E11_A	2	141	R-50394	Burkholderia	stabilis
Z0010_RO_A10_1_E11_B	2	141	R-50394	Burkholderia	stabilis
Z0010_RO_A11_1_E12_A	2	141	R-50394	Burkholderia	stabilis
Z0010_RO_A12_1_E12_B	2	141	R-50394	Burkholderia	stabilis
Z0010_RO_B01_2_E07_A	2	141	R-50394	Burkholderia	stabilis
Z0010_RO_B02_2_E07_B	2	141	R-50394	Burkholderia	stabilis
Z0010_RO_B03_2_E08_A	2	141	R-50394	Burkholderia	stabilis
Z0010_RO_B04_2_E08_B	2	141	R-50394	Burkholderia	stabilis
Z0010_RO_B05_2_E09_A	2	141	R-50394	Burkholderia	stabilis
Z0010_RO_B06_2_E09_B	2	141	R-50394	Burkholderia	stabilis
Z0010_RO_B07_2_E10_A	2	141	R-50394	Burkholderia	stabilis
Z0010_RO_B08_2_E10_B	2	141	R-50394	Burkholderia	stabilis
Z0010_LB_C09_2_C02_A	3	149	R-71006	Burkholderia	multivorans
Z0010_LB_C10_2_C02_B	3	149	R-71006	Burkholderia	multivorans
Z0010_LB_C11_2_C01_A	3	149	R-71006	Burkholderia	multivorans
Z0010_LB_C12_2_C01_B	3	149	R-71006	Burkholderia	multivorans
Z0010_LB_D01_1_C06_A	3	149	R-71006	Burkholderia	multivorans
Z0010_LB_D02_1_C06_B	3	149	R-71006	Burkholderia	multivorans
Z0010_LB_D03_1_C05_A	3	149	R-71006	Burkholderia	multivorans
Z0010_LB_D04_1_C05_B	3	149	R-71006	Burkholderia	multivorans
Z0010_LB_D05_1_C04_A	3	149	R-71006	Burkholderia	multivorans
Z0010_LB_D06_1_C04_B	3	149	R-71006	Burkholderia	multivorans
Z0010_LB_D07_1_C03_A	3	149	R-71006	Burkholderia	multivorans
Z0010_LB_D08_1_C03_B	3	149	R-71006	Burkholderia	multivorans
Z0010_LB_D09_1_C02_A	3	149	R-71006	Burkholderia	multivorans
Z0010_LB_D10_1_C02_B	3	149	R-71006	Burkholderia	multivorans
Z0010_LB_D11_1_C01_A	3	149	R-71006	Burkholderia	multivorans
Z0010_LB_D12_1_C01_B	3	149	R-71006	Burkholderia	multivorans
Z0010_LB_E01_2_B06_A	3	149	R-71006	Burkholderia	multivorans
Z0010_LB_E02_2_B06_B	3	149	R-71006	Burkholderia	multivorans
Z0010_RB_C02_2_C12_B	3	149	R-71006	Burkholderia	multivorans
Z0010_RB_C03_2_C11_A	3	149	R-71006	Burkholderia	multivorans
Z0010_RB_C04_2_C11_B	3	149	R-71006	Burkholderia	multivorans
Z0010_RB_C05_2_C10_A	3	149	R-71006	Burkholderia	multivorans
Z0010_RB_C06_2_C10_B	3	149	R-71006	Burkholderia	multivorans
Z0010_RB_C07_2_C09_A	3	149	R-71006	Burkholderia	multivorans
Z0010_RB_C08_2_C09_B	3	149	R-71006	Burkholderia	multivorans
Z0010_RB_C09_2_C08_A	3	149	R-71006	Burkholderia	multivorans
Z0010_RB_C10_2_C08_B	3	149	R-71006	Burkholderia	multivorans
Z0010_RB_C11_2_C07_A	3	149	R-71006	Burkholderia	multivorans
Z0010_RB_C12_2_C07_B	3	149	R-71006	Burkholderia	multivorans
Z0010_RB_D01_1_C12_A	3	149	R-71006	Burkholderia	multivorans
Z0010_RB_D02_1_C12_B	3	149	R-71006	Burkholderia	multivorans
Z0010_RB_D03_1_C11_A	3	149	R-71006	Burkholderia	multivorans
Z0010_LB_F07_1_B03_A	4	143	R-69593	Burkholderia	vietnamiensis

Z0010_LB_F08_1_B03_B	4	143	R-69593	Burkholderia	vietnamiensis
Z0010_LB_F09_1_B02_A	4	143	R-69593	Burkholderia	vietnamiensis
Z0010_LB_F10_1_B02_B	4	143	R-69593	Burkholderia	vietnamiensis
Z0010_LB_F11_1_B01_A	4	143	R-69593	Burkholderia	vietnamiensis
Z0010_LB_F12_1_B01_B	4	143	R-69593	Burkholderia	vietnamiensis
Z0010_LB_G01_2_A06_A	4	143	R-69593	Burkholderia	vietnamiensis
Z0010_LB_G02_2_A06_B	4	143	R-69593	Burkholderia	vietnamiensis
Z0010_LB_G03_2_A05_A	4	143	R-69593	Burkholderia	vietnamiensis
Z0010_LB_G04_2_A05_B	4	143	R-69593	Burkholderia	vietnamiensis
Z0010_LB_G05_2_A04_A	4	143	R-69593	Burkholderia	vietnamiensis
Z0010_LB_G06_2_A04_B	4	143	R-69593	Burkholderia	vietnamiensis
Z0010_LB_G07_2_A03_A	4	143	R-69593	Burkholderia	vietnamiensis
Z0010_LB_G08_2_A03_B	4	143	R-69593	Burkholderia	vietnamiensis
Z0010_LB_G09_2_A02_A	4	143	R-69593	Burkholderia	vietnamiensis
Z0010_LB_G10_2_A02_B	4	143	R-69593	Burkholderia	vietnamiensis
Z0010_LB_G11_2_A01_A	4	143	R-69593	Burkholderia	vietnamiensis
Z0010_RB_E08_2_B09_B	4	143	R-69593	Burkholderia	vietnamiensis
Z0010_RB_E09_2_B08_A	4	143	R-69593	Burkholderia	vietnamiensis
Z0010_RB_E10_2_B08_B	4	143	R-69593	Burkholderia	vietnamiensis
Z0010_RB_E11_2_B07_A	4	143	R-69593	Burkholderia	vietnamiensis
Z0010_RB_E12_2_B07_B	4	143	R-69593	Burkholderia	vietnamiensis
Z0010_RB_F01_1_B12_A	4	143	R-69593	Burkholderia	vietnamiensis
Z0010_RB_F02_1_B12_B	4	143	R-69593	Burkholderia	vietnamiensis
Z0010_RB_F03_1_B11_A	4	143	R-69593	Burkholderia	vietnamiensis
Z0010_RB_F04_1_B11_B	4	143	R-69593	Burkholderia	vietnamiensis
Z0010_RB_F05_1_B10_A	4	143	R-69593	Burkholderia	vietnamiensis
Z0010_RB_F06_1_B10_B	4	143	R-69593	Burkholderia	vietnamiensis
Z0010_RB_F07_1_B09_A	4	143	R-69593	Burkholderia	vietnamiensis
Z0010_RB_F08_1_B09_B	4	143	R-69593	Burkholderia	vietnamiensis
Z0010_RB_F09_1_B08_A	4	143	R-69593	Burkholderia	vietnamiensis
Z0010_RB_F10_1_B08_B	4	143	R-69593	Burkholderia	vietnamiensis
Z0010_LO_A02_1_E01_B	5	142	R-71089	Burkholderia	multivorans
Z0010_LO_A03_1_E02_A	5	142	R-71089	Burkholderia	multivorans
Z0010_LO_A04_1_E02_B	5	142	R-71089	Burkholderia	multivorans
Z0010_LO_A05_1_E03_A	5	142	R-71089	Burkholderia	multivorans
Z0010_LO_A06_1_E03_B	5	142	R-71089	Burkholderia	multivorans
Z0010_LO_A07_1_E04_A	5	142	R-71089	Burkholderia	multivorans
Z0010_LO_A08_1_E04_B	5	142	R-71089	Burkholderia	multivorans
Z0010_LO_A09_1_E05_A	5	142	R-71089	Burkholderia	multivorans
Z0010_LO_A10_1_E05_B	5	142	R-71089	Burkholderia	multivorans
Z0010_LO_A11_1_E06_A	5	142	R-71089	Burkholderia	multivorans
Z0010_LO_A12_1_E06_B	5	142	R-71089	Burkholderia	multivorans
Z0010_LO_B01_2_E01_A	5	142	R-71089	Burkholderia	multivorans
Z0010_LO_B02_2_E01_B	5	142	R-71089	Burkholderia	multivorans
Z0010_LO_D08_2_F04_B	5	144	R-67196	Burkholderia	multivorans
Z0010_LO_D10_2_F05_B	5	144	R-67196	Burkholderia	multivorans
Z0010_LO_E01_1_G01_A	5	144	R-67196	Burkholderia	multivorans
Z0010_LO_E03_1_G02_A	5	144	R-67196	Burkholderia	multivorans
Z0010_LO_E04_1_G02_B	5	144	R-67196	Burkholderia	multivorans
Z0010_LO_E05_1_G03_A	5	144	R-67196	Burkholderia	multivorans

Z0010_LO_E07_1_G04_A	5	142	R-67121	Burkholderia	multivorans
Z0010_LO_E08_1_G04_B	5	142	R-67121	Burkholderia	multivorans
Z0010_LO_E09_1_G05_A	5	144	R-67196	Burkholderia	multivorans
Z0010_LO_E10_1_G05_B	5	142	R-67121	Burkholderia	multivorans
Z0010_LO_E11_1_G06_A	5	144	R-67196	Burkholderia	multivorans
Z0010_LO_E12_1_G06_B	5	144	R-67196	Burkholderia	multivorans
Z0010_LO_F01_2_G01_A	5	142	R-67121	Burkholderia	multivorans
Z0010_LO_F02_2_G01_B	5	144	R-67196	Burkholderia	multivorans
Z0010_LO_F03_2_G02_A	5	142	R-67121	Burkholderia	multivorans
Z0010_LO_F04_2_G02_B	5	142	R-67121	Burkholderia	multivorans
Z0010_LO_F05_2_G03_A	5	144	R-67196	Burkholderia	multivorans
Z0010_LO_F06_2_G03_B	5	142	R-67121	Burkholderia	multivorans
Z0010_LO_F07_2_G04_A	5	144	R-67196	Burkholderia	multivorans
Z0010_LO_F08_2_G04_B	5	144	R-67196	Burkholderia	multivorans
Z0010_LO_F09_2_G05_A	5	142	R-67121	Burkholderia	multivorans
Z0010_LO_F10_2_G05_B	5	144	R-67196	Burkholderia	multivorans
Z0010_LO_F11_2_G06_A	5	142	R-67121	Burkholderia	multivorans
Z0010_LO_F12_2_G06_B	5	142	R-67121	Burkholderia	multivorans
Z0010_LO_G01_1_H01_A	5	144	R-67196	Burkholderia	multivorans
Z0010_LO_G02_1_H01_B	5	142	R-67121	Burkholderia	multivorans
Z0010_LO_G03_1_H02_A	5	144	R-67196	Burkholderia	multivorans
Z0010_LO_G04_1_H02_B	5	142	R-67121	Burkholderia	multivorans
Z0010_LO_G05_1_H03_A	5	142	R-67121	Burkholderia	multivorans
Z0010_LO_G06_1_H03_B	5	142	R-67121	Burkholderia	multivorans
Z0010_LO_G07_1_H04_A	5	144	R-67196	Burkholderia	multivorans
Z0010_LO_G08_1_H04_B	5	144	R-67196	Burkholderia	multivorans
Z0010_LO_G09_1_H05_A	5	144	R-67196	Burkholderia	multivorans
Z0010_LO_G10_1_H05_B	5	142	R-67121	Burkholderia	multivorans
Z0010_RB_H05_1_A10_A	5	142	R-71089	Burkholderia	multivorans
Z0010_RB_H06_1_A10_B	5	142	R-71089	Burkholderia	multivorans
Z0010_RB_H07_1_A09_A	5	142	R-71089	Burkholderia	multivorans
Z0010_RB_H08_1_A09_B	5	142	R-71089	Burkholderia	multivorans
Z0010_RB_H09_1_A08_A	5	142	R-71089	Burkholderia	multivorans
Z0010_RB_H10_1_A08_B	5	142	R-71089	Burkholderia	multivorans
Z0010_RB_H11_1_A07_A	5	142	R-71089	Burkholderia	multivorans
Z0010_RB_H12_1_A07_B	5	142	R-71089	Burkholderia	multivorans
Z0010_RO_A01_1_E07_A	5	142	R-71089	Burkholderia	multivorans
Z0010_RO_A02_1_E07_B	5	142	R-71089	Burkholderia	multivorans
Z0010_RO_A03_1_E08_A	5	142	R-71089	Burkholderia	multivorans
Z0010_RO_A04_1_E08_B	5	142	R-71089	Burkholderia	multivorans
Z0010_RO_A05_1_E09_A	5	142	R-71089	Burkholderia	multivorans
Z0010_RO_A06_1_E09_B	5	142	R-71089	Burkholderia	multivorans
Z0010_RO_A07_1_E10_A	5	142	R-71089	Burkholderia	multivorans
Z0010_RO_D02_2_F07_B	5	144	R-67196	Burkholderia	multivorans
Z0010_RO_D03_2_F08_A	5	144	R-67196	Burkholderia	multivorans
Z0010_RO_D05_2_F09_A	5	144	R-67196	Burkholderia	multivorans
Z0010_RO_D09_2_F11_A	5	144	R-67196	Burkholderia	multivorans
Z0010_RO_D10_2_F11_B	5	144	R-67196	Burkholderia	multivorans
Z0010_RO_D11_2_F12_A	5	144	R-67196	Burkholderia	multivorans
Z0010_RO_D12_2_F12_B	5	142	R-67121	Burkholderia	multivorans

Z0010_RO_E01_1_G07_A	5	144	R-67196	Burkholderia	multivorans
Z0010_RO_E02_1_G07_B	5	142	R-67121	Burkholderia	multivorans
Z0010_RO_E03_1_G08_A	5	142	R-67121	Burkholderia	multivorans
Z0010_RO_E04_1_G08_B	5	144	R-67196	Burkholderia	multivorans
Z0010_RO_E05_1_G09_A	5	142	R-67121	Burkholderia	multivorans
Z0010_RO_E06_1_G09_B	5	144	R-67196	Burkholderia	multivorans
Z0010_RO_E07_1_G10_A	5	144	R-67196	Burkholderia	multivorans
Z0010_RO_E08_1_G10_B	5	142	R-67121	Burkholderia	multivorans
Z0010_RO_E09_1_G11_A	5	144	R-67196	Burkholderia	multivorans
Z0010_RO_E10_1_G11_B	5	142	R-67121	Burkholderia	multivorans
Z0010_RO_E11_1_G12_A	5	142	R-67121	Burkholderia	multivorans
Z0010_RO_E12_1_G12_B	5	144	R-67196	Burkholderia	multivorans
Z0010_RO_F01_2_G07_A	5	142	R-67121	Burkholderia	multivorans
Z0010_RO_F03_2_G08_A	5	144	R-67196	Burkholderia	multivorans
Z0010_RO_F04_2_G08_B	5	142	R-67121	Burkholderia	multivorans
Z0010_RO_F06_2_G09_B	5	142	R-67121	Burkholderia	multivorans
Z0010_RO_F10_2_G11_B	5	142	R-67121	Burkholderia	multivorans
Z0010_RO_F11_2_G12_A	5	142	R-67121	Burkholderia	multivorans
Z0010_RO_F12_2_G12_B	5	142	R-67121	Burkholderia	multivorans
Z0010_RO_G02_1_H07_B	5	142	R-67121	Burkholderia	multivorans
Z0010_RO_G03_1_H08_A	5	142	R-67121	Burkholderia	multivorans
Z0010_RO_G04_1_H08_B	5	142	R-67121	Burkholderia	multivorans
Z0010_RO_G05_1_H09_A	5	142	R-67121	Burkholderia	multivorans
Z0011_LB_A01_2_D06_A	5	144	R-67258	Burkholderia	multivorans
Z0011_LB_A02_2_D06_B	5	144	R-67258	Burkholderia	multivorans
Z0011_LB_A03_2_D05_A	5	144	R-67258	Burkholderia	multivorans
Z0011_LB_A04_2_D05_B	5	144	R-67258	Burkholderia	multivorans
Z0011_LB_A05_2_D04_A	5	144	R-67258	Burkholderia	multivorans
Z0011_LB_A06_2_D04_B	5	144	R-67258	Burkholderia	multivorans
Z0011_LB_A07_2_D03_A	5	144	R-67258	Burkholderia	multivorans
Z0011_LB_A08_2_D03_B	5	144	R-67258	Burkholderia	multivorans
Z0011_LB_A09_2_D02_A	5	144	R-67258	Burkholderia	multivorans
Z0011_LB_A10_2_D02_B	5	144	R-67258	Burkholderia	multivorans
Z0011_LB_A11_2_D01_A	5	144	R-67258	Burkholderia	multivorans
Z0011_LB_A12_2_D01_B	5	144	R-67258	Burkholderia	multivorans
Z0011_LB_B01_1_D06_A	5	144	R-67258	Burkholderia	multivorans
Z0011_LB_B02_1_D06_B	5	144	R-67258	Burkholderia	multivorans
Z0011_LB_B03_1_D05_A	5	144	R-67258	Burkholderia	multivorans
Z0011_LB_B04_1_D05_B	5	144	R-67258	Burkholderia	multivorans
Z0011_LB_B05_1_D04_A	5	144	R-67258	Burkholderia	multivorans
Z0011_LB_D01_1_C06_A	5	142	R-67536	Burkholderia	multivorans
Z0011_LB_D02_1_C06_B	5	142	R-67536	Burkholderia	multivorans
Z0011_LB_D03_1_C05_A	5	142	R-67536	Burkholderia	multivorans
Z0011_LB_D04_1_C05_B	5	142	R-67536	Burkholderia	multivorans
Z0011_LB_D05_1_C04_A	5	142	R-67536	Burkholderia	multivorans
Z0011_LB_D06_1_C04_B	5	142	R-67536	Burkholderia	multivorans
Z0011_LB_D07_1_C03_A	5	142	R-67536	Burkholderia	multivorans
Z0011_LB_D08_1_C03_B	5	142	R-67536	Burkholderia	multivorans
Z0011_LB_D09_1_C02_A	5	142	R-67536	Burkholderia	multivorans
Z0011_LB_D10_1_C02_B	5	142	R-67536	Burkholderia	multivorans

Z0011_LB_D11_1_C01_A	5	142	R-67536	Burkholderia	multivorans
Z0011_LB_D12_1_C01_B	5	142	R-67536	Burkholderia	multivorans
Z0011_LB_E01_2_B06_A	5	142	R-67536	Burkholderia	multivorans
Z0011_LB_E02_2_B06_B	5	142	R-67536	Burkholderia	multivorans
Z0011_LB_E03_2_B05_A	5	142	R-67536	Burkholderia	multivorans
Z0011_LB_E04_2_B05_B	5	142	R-67536	Burkholderia	multivorans
Z0011_LB_E05_2_B04_A	5	142	R-67536	Burkholderia	multivorans
Z0011_LB_E06_2_B04_B	5	142	R-67536	Burkholderia	multivorans
Z0011_LO_D03_2_F02_A	5	144	R-67258	Burkholderia	multivorans
Z0011_LO_H04_2_H02_B	5	144	R-67258	Burkholderia	multivorans
Z0011_LO_H05_2_H03_A	5	144	R-67258	Burkholderia	multivorans
Z0011_LO_H06_2_H03_B	5	144	R-67258	Burkholderia	multivorans
Z0011_LO_H07_2_H04_A	5	144	R-67258	Burkholderia	multivorans
Z0011_LO_H08_2_H04_B	5	144	R-67258	Burkholderia	multivorans
Z0011_LO_H09_2_H05_A	5	144	R-67258	Burkholderia	multivorans
Z0011_LO_H10_2_H05_B	5	144	R-67258	Burkholderia	multivorans
Z0011_LO_H11_2_H06_A	5	144	R-67258	Burkholderia	multivorans
Z0011_LO_H12_2_H06_B	5	144	R-67258	Burkholderia	multivorans
Z0011_RB_A01_2_D12_A	5	144	R-67258	Burkholderia	multivorans
Z0011_RB_A02_2_D12_B	5	144	R-67258	Burkholderia	multivorans
Z0011_RB_A03_2_D11_A	5	144	R-67258	Burkholderia	multivorans
Z0011_RB_B05_1_D10_A	5	142	R-67536	Burkholderia	multivorans
Z0011_RB_B06_1_D10_B	5	142	R-67536	Burkholderia	multivorans
Z0011_RB_B07_1_D09_A	5	142	R-67536	Burkholderia	multivorans
Z0011_RB_B08_1_D09_B	5	142	R-67536	Burkholderia	multivorans
Z0011_RB_B09_1_D08_A	5	142	R-67536	Burkholderia	multivorans
Z0011_RB_B10_1_D08_B	5	142	R-67536	Burkholderia	multivorans
Z0011_RB_B11_1_D07_A	5	142	R-67536	Burkholderia	multivorans
Z0011_RB_B12_1_D07_B	5	142	R-67536	Burkholderia	multivorans
Z0011_RB_C01_2_C12_A	5	142	R-67536	Burkholderia	multivorans
Z0011_RB_C02_2_C12_B	5	142	R-67536	Burkholderia	multivorans
Z0011_RB_C03_2_C11_A	5	142	R-67536	Burkholderia	multivorans
Z0011_RB_C04_2_C11_B	5	142	R-67536	Burkholderia	multivorans
Z0011_RB_C05_2_C10_A	5	142	R-67536	Burkholderia	multivorans
Z0011_RB_C06_2_C10_B	5	142	R-67536	Burkholderia	multivorans
Z0011_RO_B03_2_E08_A	5	144	R-67258	Burkholderia	multivorans
Z0011_RO_B07_2_E10_A	5	144	R-67258	Burkholderia	multivorans
Z0010_LO_C06_1_F03_B	6	141	R-67113	Burkholderia	stabilis
Z0010_LO_C07_1_F04_A	6	141	R-67113	Burkholderia	stabilis
Z0010_LO_C08_1_F04_B	6	141	R-67113	Burkholderia	stabilis
Z0010_LO_C09_1_F05_A	6	141	R-67113	Burkholderia	stabilis
Z0010_LO_C10_1_F05_B	6	141	R-67113	Burkholderia	stabilis
Z0010_LO_C11_1_F06_A	6	141	R-67113	Burkholderia	stabilis
Z0010_LO_C12_1_F06_B	6	141	R-67113	Burkholderia	stabilis
Z0010_LO_D02_2_F01_B	6	141	R-67113	Burkholderia	stabilis
Z0010_LO_D03_2_F02_A	6	141	R-67113	Burkholderia	stabilis
Z0010_LO_D05_2_F03_A	6	141	R-67113	Burkholderia	stabilis
Z0010_LO_D07_2_F04_A	6	141	R-67113	Burkholderia	stabilis
Z0010_LO_D09_2_F05_A	6	141	R-67113	Burkholderia	stabilis
Z0010_LO_D11_2_F06_A	6	141	R-67113	Burkholderia	stabilis

Z0010_LO_D12_2_F06_B	6	141	R-67113	Burkholderia	stabilis
Z0010_LO_E02_1_G01_B	6	141	R-67113	Burkholderia	stabilis
Z0010_LO_E06_1_G03_B	6	141	R-67113	Burkholderia	stabilis
Z0010_RO_B09_2_E11_A	6	141	R-67113	Burkholderia	stabilis
Z0010_RO_B10_2_E11_B	6	141	R-67113	Burkholderia	stabilis
Z0010_RO_B11_2_E12_A	6	141	R-67113	Burkholderia	stabilis
Z0010_RO_B12_2_E12_B	6	141	R-67113	Burkholderia	stabilis
Z0010_RO_C01_1_F07_A	6	141	R-67113	Burkholderia	stabilis
Z0010_RO_C02_1_F07_B	6	141	R-67113	Burkholderia	stabilis
Z0010_RO_C03_1_F08_A	6	141	R-67113	Burkholderia	stabilis
Z0010_RO_C04_1_F08_B	6	141	R-67113	Burkholderia	stabilis
Z0010_RO_C07_1_F10_A	6	141	R-67113	Burkholderia	stabilis
Z0010_RO_C09_1_F11_A	6	141	R-67113	Burkholderia	stabilis
Z0010_RO_C11_1_F12_A	6	141	R-67113	Burkholderia	stabilis
Z0010_RO_D01_2_F07_A	6	141	R-67113	Burkholderia	stabilis
Z0010_RO_D04_2_F08_B	6	141	R-67113	Burkholderia	stabilis
Z0010_RO_D06_2_F09_B	6	141	R-67113	Burkholderia	stabilis
Z0010_RO_D07_2_F10_A	6	141	R-67113	Burkholderia	stabilis
Z0010_RO_D08_2_F10_B	6	141	R-67113	Burkholderia	stabilis
Z0010_LO_G11_1_H06_A	7	143	R-67189	Burkholderia	vietnamiensis
Z0010_LO_G12_1_H06_B	7	143	R-67189	Burkholderia	vietnamiensis
Z0010_LO_H01_2_H01_A	7	143	R-67189	Burkholderia	vietnamiensis
Z0010_LO_H02_2_H01_B	7	143	R-67189	Burkholderia	vietnamiensis
Z0010_LO_H03_2_H02_A	7	143	R-67189	Burkholderia	vietnamiensis
Z0010_LO_H04_2_H02_B	7	143	R-67189	Burkholderia	vietnamiensis
Z0010_LO_H05_2_H03_A	7	143	R-67189	Burkholderia	vietnamiensis
Z0010_LO_H06_2_H03_B	7	143	R-67189	Burkholderia	vietnamiensis
Z0010_LO_H07_2_H04_A	7	143	R-67189	Burkholderia	vietnamiensis
Z0010_LO_H08_2_H04_B	7	143	R-67189	Burkholderia	vietnamiensis
Z0010_LO_H09_2_H05_A	7	143	R-67189	Burkholderia	vietnamiensis
Z0010_LO_H10_2_H05_B	7	143	R-67189	Burkholderia	vietnamiensis
Z0010_LO_H11_2_H06_A	7	143	R-67189	Burkholderia	vietnamiensis
Z0010_LO_H12_2_H06_B	7	143	R-67189	Burkholderia	vietnamiensis
Z0010_RB_A01_2_D12_A	7	143	R-67189	Burkholderia	vietnamiensis
Z0010_RO_F02_2_G07_B	7	143	R-67189	Burkholderia	vietnamiensis
Z0010_RO_F05_2_G09_A	7	143	R-67189	Burkholderia	vietnamiensis
Z0010_RO_F07_2_G10_A	7	143	R-67189	Burkholderia	vietnamiensis
Z0010_RO_F08_2_G10_B	7	143	R-67189	Burkholderia	vietnamiensis
Z0010_RO_F09_2_G11_A	7	143	R-67189	Burkholderia	vietnamiensis
Z0010_RO_G01_1_H07_A	7	143	R-67189	Burkholderia	vietnamiensis
Z0010_RO_G06_1_H09_B	7	143	R-67189	Burkholderia	vietnamiensis
Z0010_RO_G07_1_H10_A	7	143	R-67189	Burkholderia	vietnamiensis
Z0010_RO_G08_1_H10_B	7	143	R-67189	Burkholderia	vietnamiensis
Z0010_RO_G09_1_H11_A	7	143	R-67189	Burkholderia	vietnamiensis
Z0010_RO_G10_1_H11_B	7	143	R-67189	Burkholderia	vietnamiensis
Z0010_RO_G11_1_H12_A	7	143	R-67189	Burkholderia	vietnamiensis
Z0010_RO_G12_1_H12_B	7	143	R-67189	Burkholderia	vietnamiensis
Z0010_RO_H01_2_H07_A	7	143	R-67189	Burkholderia	vietnamiensis
Z0010_RO_H02_2_H07_B	7	143	R-67189	Burkholderia	vietnamiensis
Z0010_RO_H03_2_H08_A	7	143	R-67189	Burkholderia	vietnamiensis

Z0010_RO_H04_2_H08_B	7	143	R-67189	Burkholderia	vietnamiensis
Z0011_LO_B07_2_E04_A	8	142	R-68768	Burkholderia	multivorans
Z0011_LO_B08_2_E04_B	8	142	R-68768	Burkholderia	multivorans
Z0011_LO_B09_2_E05_A	8	142	R-68768	Burkholderia	multivorans
Z0011_LO_B10_2_E05_B	8	142	R-68768	Burkholderia	multivorans
Z0011_LO_B11_2_E06_A	8	142	R-68768	Burkholderia	multivorans
Z0011_LO_B12_2_E06_B	8	142	R-68768	Burkholderia	multivorans
Z0011_LO_C01_1_F01_A	8	142	R-68768	Burkholderia	multivorans
Z0011_LO_C02_1_F01_B	8	142	R-68768	Burkholderia	multivorans
Z0011_LO_C03_1_F02_A	8	142	R-68768	Burkholderia	multivorans
Z0011_LO_C04_1_F02_B	8	142	R-68768	Burkholderia	multivorans
Z0011_RB_H11_1_A07_A	8	142	R-68768	Burkholderia	multivorans
Z0011_RB_H12_1_A07_B	8	142	R-68768	Burkholderia	multivorans
Z0011_RO_A01_1_E07_A	8	142	R-68768	Burkholderia	multivorans
Z0011_RO_A02_1_E07_B	8	142	R-68768	Burkholderia	multivorans
Z0011_RO_A03_1_E08_A	8	142	R-68768	Burkholderia	multivorans
Z0011_RO_A04_1_E08_B	8	142	R-68768	Burkholderia	multivorans
Z0011_RO_A05_1_E09_A	8	142	R-68768	Burkholderia	multivorans
Z0011_RO_A06_1_E09_B	8	142	R-68768	Burkholderia	multivorans
Z0011_RO_A07_1_E10_A	8	142	R-68768	Burkholderia	multivorans
Z0011_RO_A08_1_E10_B	8	142	R-68768	Burkholderia	multivorans
Z0011_RO_A09_1_E11_A	8	142	R-68768	Burkholderia	multivorans
Z0011_RO_A10_1_E11_B	8	142	R-68768	Burkholderia	multivorans
Z0011_RO_A11_1_E12_A	8	142	R-68768	Burkholderia	multivorans
Z0011_RO_A12_1_E12_B	8	142	R-68768	Burkholderia	multivorans
Z0011_RO_B01_2_E07_A	8	142	R-68768	Burkholderia	multivorans
Z0011_RO_B02_2_E07_B	8	142	R-68768	Burkholderia	multivorans
Z0011_RO_B04_2_E08_B	8	142	R-68768	Burkholderia	multivorans
Z0011_RO_B05_2_E09_A	8	142	R-68768	Burkholderia	multivorans
Z0011_LO_C05_1_F03_A	9	1	LMG 1041	Acinetobacter	baumannii
Z0011_LO_C06_1_F03_B	9	1	LMG 1041	Acinetobacter	baumannii
Z0011_LO_C07_1_F04_A	9	1	LMG 1041	Acinetobacter	baumannii
Z0011_LO_C08_1_F04_B	9	1	LMG 1041	Acinetobacter	baumannii
Z0011_LO_C09_1_F05_A	9	1	LMG 1041	Acinetobacter	baumannii
Z0011_LO_C10_1_F05_B	9	1	LMG 1041	Acinetobacter	baumannii
Z0011_LO_C11_1_F06_A	9	1	LMG 1041	Acinetobacter	baumannii
Z0011_LO_C12_1_F06_B	9	1	LMG 1041	Acinetobacter	baumannii
Z0011_LO_D01_2_F01_A	9	1	LMG 1041	Acinetobacter	baumannii
Z0011_LO_D04_2_F02_B	9	1	LMG 1041	Acinetobacter	baumannii
Z0011_LO_D05_2_F03_A	9	1	LMG 1041	Acinetobacter	baumannii
Z0011_LO_D06_2_F03_B	9	1	LMG 1041	Acinetobacter	baumannii
Z0011_LO_D07_2_F04_A	9	1	LMG 1041	Acinetobacter	baumannii
Z0011_LO_D10_2_F05_B	9	1	LMG 1041	Acinetobacter	baumannii
Z0011_LO_D11_2_F06_A	9	1	LMG 1041	Acinetobacter	baumannii
Z0011_LO_D12_2_F06_B	9	1	LMG 1041	Acinetobacter	baumannii
Z0011_RO_B08_2_E10_B	9	1	LMG 1041	Acinetobacter	baumannii
Z0011_RO_B09_2_E11_A	9	1	LMG 1041	Acinetobacter	baumannii
Z0011_RO_B11_2_E12_A	9	1	LMG 1041	Acinetobacter	baumannii
Z0011_RO_B12_2_E12_B	9	1	LMG 1041	Acinetobacter	baumannii
Z0011_RO_C01_1_F07_A	9	1	LMG 1041	Acinetobacter	baumannii

Z0011_RO_C02_1_F07_B	9	1	LMG 1041	Acinetobacter	baumannii
Z0011_RO_C06_1_F09_B	9	1	LMG 1041	Acinetobacter	baumannii
Z0011_RO_C07_1_F10_A	9	1	LMG 1041	Acinetobacter	baumannii
Z0011_RO_C10_1_F11_B	9	1	LMG 1041	Acinetobacter	baumannii
Z0011_RO_C11_1_F12_A	9	1	LMG 1041	Acinetobacter	baumannii
Z0011_RO_D02_2_F07_B	9	1	LMG 1041	Acinetobacter	baumannii
Z0011_RO_D03_2_F08_A	9	1	LMG 1041	Acinetobacter	baumannii
Z0011_RO_D04_2_F08_B	9	1	LMG 1041	Acinetobacter	baumannii
Z0011_RO_D09_2_F11_A	9	1	LMG 1041	Acinetobacter	baumannii
Z0011_RO_D10_2_F11_B	9	1	LMG 1041	Acinetobacter	baumannii
Z0011_RO_E02_1_G07_B	9	1	LMG 1041	Acinetobacter	baumannii
Z0011_LO_D02_2_F01_B	10	2	LMG 11039	Bifidobacterium	angulatum
Z0011_LO_D08_2_F04_B	10	2	LMG 11039	Bifidobacterium	angulatum
Z0011_LO_D09_2_F05_A	10	2	LMG 11039	Bifidobacterium	angulatum
Z0011_LO_E01_1_G01_A	10	2	LMG 11039	Bifidobacterium	angulatum
Z0011_LO_E02_1_G01_B	10	2	LMG 11039	Bifidobacterium	angulatum
Z0011_LO_E04_1_G02_B	10	2	LMG 11039	Bifidobacterium	angulatum
Z0011_LO_E05_1_G03_A	10	2	LMG 11039	Bifidobacterium	angulatum
Z0011_LO_E06_1_G03_B	10	2	LMG 11039	Bifidobacterium	angulatum
Z0011_LO_E09_1_G05_A	10	2	LMG 11039	Bifidobacterium	angulatum
Z0011_LO_E11_1_G06_A	10	2	LMG 11039	Bifidobacterium	angulatum
Z0011_LO_E12_1_G06_B	10	2	LMG 11039	Bifidobacterium	angulatum
Z0011_LO_F02_2_G01_B	10	2	LMG 11039	Bifidobacterium	angulatum
Z0011_LO_F06_2_G03_B	10	2	LMG 11039	Bifidobacterium	angulatum
Z0011_RO_B06_2_E09_B	10	2	LMG 11039	Bifidobacterium	angulatum
Z0011_RO_B10_2_E11_B	10	2	LMG 11039	Bifidobacterium	angulatum
Z0011_RO_C03_1_F08_A	10	2	LMG 11039	Bifidobacterium	angulatum
Z0011_RO_C04_1_F08_B	10	2	LMG 11039	Bifidobacterium	angulatum
Z0011_RO_C05_1_F09_A	10	2	LMG 11039	Bifidobacterium	angulatum
Z0011_RO_C08_1_F10_B	10	2	LMG 11039	Bifidobacterium	angulatum
Z0011_RO_C09_1_F11_A	10	2	LMG 11039	Bifidobacterium	angulatum
Z0011_RO_C12_1_F12_B	10	2	LMG 11039	Bifidobacterium	angulatum
Z0011_RO_D01_2_F07_A	10	2	LMG 11039	Bifidobacterium	angulatum
Z0011_RO_D05_2_F09_A	10	2	LMG 11039	Bifidobacterium	angulatum
Z0011_RO_D06_2_F09_B	10	2	LMG 11039	Bifidobacterium	angulatum
Z0011_RO_D07_2_F10_A	10	2	LMG 11039	Bifidobacterium	angulatum
Z0011_RO_D08_2_F10_B	10	2	LMG 11039	Bifidobacterium	angulatum
Z0011_RO_D11_2_F12_A	10	2	LMG 11039	Bifidobacterium	angulatum
Z0011_RO_D12_2_F12_B	10	2	LMG 11039	Bifidobacterium	angulatum
Z0011_RO_E01_1_G07_A	10	2	LMG 11039	Bifidobacterium	angulatum
Z0011_RO_E04_1_G08_B	10	2	LMG 11039	Bifidobacterium	angulatum
Z0011_RO_E08_1_G10_B	10	2	LMG 11039	Bifidobacterium	angulatum
Z0011_RO_E09_1_G11_A	10	2	LMG 11039	Bifidobacterium	angulatum
Z0012_LB_A01_2_D06_A	11	3	LMG 11194	Moraxella	canis
Z0012_LB_A02_2_D06_B	11	3	LMG 11194	Moraxella	canis
Z0012_LB_A03_2_D05_A	11	3	LMG 11194	Moraxella	canis
Z0012_LB_A04_2_D05_B	11	3	LMG 11194	Moraxella	canis
Z0012_LB_A05_2_D04_A	11	3	LMG 11194	Moraxella	canis
Z0012_LB_A06_2_D04_B	11	3	LMG 11194	Moraxella	canis
Z0012_LB_A07_2_D03_A	11	3	LMG 11194	Moraxella	canis

Z0012_LB_A08_2_D03_B	11	3	LMG 11194	Moraxella	canis
Z0012_LB_A09_2_D02_A	11	3	LMG 11194	Moraxella	canis
Z0012_LB_A10_2_D02_B	11	3	LMG 11194	Moraxella	canis
Z0012_LB_A11_2_D01_A	11	3	LMG 11194	Moraxella	canis
Z0012_LB_A12_2_D01_B	11	3	LMG 11194	Moraxella	canis
Z0012_LB_B01_1_D06_A	11	3	LMG 11194	Moraxella	canis
Z0012_LB_B02_1_D06_B	11	3	LMG 11194	Moraxella	canis
Z0012_LB_B03_1_D05_A	11	3	LMG 11194	Moraxella	canis
Z0012_LB_B04_1_D05_B	11	3	LMG 11194	Moraxella	canis
Z0012_LB_F11_1_B01_A	11	3	LMG 11194	Moraxella	canis
Z0012_RB_A04_2_D11_B	11	3	LMG 11194	Moraxella	canis
Z0012_RB_A05_2_D10_A	11	3	LMG 11194	Moraxella	canis
Z0012_RB_A06_2_D10_B	11	3	LMG 11194	Moraxella	canis
Z0012_RB_A07_2_D09_A	11	3	LMG 11194	Moraxella	canis
Z0012_RB_A08_2_D09_B	11	3	LMG 11194	Moraxella	canis
Z0012_RB_A09_2_D08_A	11	3	LMG 11194	Moraxella	canis
Z0012_RB_A10_2_D08_B	11	3	LMG 11194	Moraxella	canis
Z0012_RB_A11_2_D07_A	11	3	LMG 11194	Moraxella	canis
Z0012_RB_A12_2_D07_B	11	3	LMG 11194	Moraxella	canis
Z0012_RB_B01_1_D12_A	11	3	LMG 11194	Moraxella	canis
Z0012_RB_B02_1_D12_B	11	3	LMG 11194	Moraxella	canis
Z0012_RB_B03_1_D11_A	11	3	LMG 11194	Moraxella	canis
Z0012_RB_F05_1_B10_A	11	3	LMG 11194	Moraxella	canis
Z0012_RB_F07_1_B09_A	11	3	LMG 11194	Moraxella	canis
Z0012_RB_F08_1_B09_B	11	3	LMG 11194	Moraxella	canis
Z0012_LB_B08_1_D03_B	12	4	LMG 11405	Lactobacillus	plantarum
Z0012_LB_B09_1_D02_A	12	4	LMG 11405	Lactobacillus	plantarum
Z0012_LB_B10_1_D02_B	12	4	LMG 11405	Lactobacillus	plantarum
Z0012_LB_B11_1_D01_A	12	4	LMG 11405	Lactobacillus	plantarum
Z0012_LB_B12_1_D01_B	12	4	LMG 11405	Lactobacillus	plantarum
Z0012_LB_C02_2_C06_B	12	4	LMG 11405	Lactobacillus	plantarum
Z0012_LB_C07_2_C03_A	12	4	LMG 11405	Lactobacillus	plantarum
Z0012_RB_B05_1_D10_A	12	4	LMG 11405	Lactobacillus	plantarum
Z0012_RB_B06_1_D10_B	12	4	LMG 11405	Lactobacillus	plantarum
Z0012_RB_B10_1_D08_B	12	4	LMG 11405	Lactobacillus	plantarum
Z0012_RB_B11_1_D07_A	12	4	LMG 11405	Lactobacillus	plantarum
Z0012_RB_C01_2_C12_A	12	4	LMG 11405	Lactobacillus	plantarum
Z0012_LB_B05_1_D04_A	12	4	LMG 11405	Lactobacillus	plantarum
Z0012_LB_B06_1_D04_B	12	4	LMG 11405	Lactobacillus	plantarum
Z0012_LB_B07_1_D03_A	12	4	LMG 11405	Lactobacillus	plantarum
Z0012_LB_C01_2_C06_A	12	4	LMG 11405	Lactobacillus	plantarum
Z0012_LB_C03_2_C05_A	12	4	LMG 11405	Lactobacillus	plantarum
Z0012_LB_C04_2_C05_B	12	4	LMG 11405	Lactobacillus	plantarum
Z0012_LB_C05_2_C04_A	12	4	LMG 11405	Lactobacillus	plantarum
Z0012_LB_C06_2_C04_B	12	4	LMG 11405	Lactobacillus	plantarum
Z0012_LB_C08_2_C03_B	12	4	LMG 11405	Lactobacillus	plantarum
Z0012_LB_C09_2_C02_A	12	4	LMG 11405	Lactobacillus	plantarum
Z0012_LB_C10_2_C02_B	12	4	LMG 11405	Lactobacillus	plantarum
Z0012_RB_B04_1_D11_B	12	4	LMG 11405	Lactobacillus	plantarum
Z0012_RB_B07_1_D09_A	12	4	LMG 11405	Lactobacillus	plantarum

Z0012_RB_B08_1_D09_B	12	4	LMG 11405	Lactobacillus	plantarum
Z0012_RB_B09_1_D08_A	12	4	LMG 11405	Lactobacillus	plantarum
Z0012_RB_B12_1_D07_B	12	4	LMG 11405	Lactobacillus	plantarum
Z0012_RB_C02_2_C12_B	12	4	LMG 11405	Lactobacillus	plantarum
Z0012_RB_C03_2_C11_A	12	4	LMG 11405	Lactobacillus	plantarum
Z0012_RB_C04_2_C11_B	12	4	LMG 11405	Lactobacillus	plantarum
Z0012_RB_C05_2_C10_A	12	4	LMG 11405	Lactobacillus	plantarum
Z0012_LB_C11_2_C01_A	13	5	LMG 1226	Delftia	acidovorans
Z0012_LB_C12_2_C01_B	13	5	LMG 1226	Delftia	acidovorans
Z0012_LB_D01_1_C06_A	13	5	LMG 1226	Delftia	acidovorans
Z0012_LB_D02_1_C06_B	13	5	LMG 1226	Delftia	acidovorans
Z0012_LB_D03_1_C05_A	13	5	LMG 1226	Delftia	acidovorans
Z0012_LB_D04_1_C05_B	13	5	LMG 1226	Delftia	acidovorans
Z0012_LB_D05_1_C04_A	13	5	LMG 1226	Delftia	acidovorans
Z0012_LB_D06_1_C04_B	13	5	LMG 1226	Delftia	acidovorans
Z0012_LB_D07_1_C03_A	13	5	LMG 1226	Delftia	acidovorans
Z0012_LB_D08_1_C03_B	13	5	LMG 1226	Delftia	acidovorans
Z0012_LB_D09_1_C02_A	13	5	LMG 1226	Delftia	acidovorans
Z0012_LB_D10_1_C02_B	13	5	LMG 1226	Delftia	acidovorans
Z0012_LB_D11_1_C01_A	13	5	LMG 1226	Delftia	acidovorans
Z0012_LB_D12_1_C01_B	13	5	LMG 1226	Delftia	acidovorans
Z0012_LB_E01_2_B06_A	13	5	LMG 1226	Delftia	acidovorans
Z0012_LB_E02_2_B06_B	13	5	LMG 1226	Delftia	acidovorans
Z0012_LB_E03_2_B05_A	13	5	LMG 1226	Delftia	acidovorans
Z0012_LB_E04_2_B05_B	13	5	LMG 1226	Delftia	acidovorans
Z0012_LB_E05_2_B04_A	13	5	LMG 1226	Delftia	acidovorans
Z0012_RB_C06_2_C10_B	13	5	LMG 1226	Delftia	acidovorans
Z0012_RB_C07_2_C09_A	13	5	LMG 1226	Delftia	acidovorans
Z0012_RB_C08_2_C09_B	13	5	LMG 1226	Delftia	acidovorans
Z0012_RB_C09_2_C08_A	13	5	LMG 1226	Delftia	acidovorans
Z0012_RB_C10_2_C08_B	13	5	LMG 1226	Delftia	acidovorans
Z0012_RB_C11_2_C07_A	13	5	LMG 1226	Delftia	acidovorans
Z0012_RB_C12_2_C07_B	13	5	LMG 1226	Delftia	acidovorans
Z0012_RB_D01_1_C12_A	13	5	LMG 1226	Delftia	acidovorans
Z0012_RB_D02_1_C12_B	13	5	LMG 1226	Delftia	acidovorans
Z0012_RB_D03_1_C11_A	13	5	LMG 1226	Delftia	acidovorans
Z0012_RB_D04_1_C11_B	13	5	LMG 1226	Delftia	acidovorans
Z0012_RB_D05_1_C10_A	13	5	LMG 1226	Delftia	acidovorans
Z0012_RB_D06_1_C10_B	13	5	LMG 1226	Delftia	acidovorans
Z0012_LB_E06_2_B04_B	14	6	LMG 1229	Alcaligenes	faecalis
Z0012_LB_E07_2_B03_A	14	6	LMG 1229	Alcaligenes	faecalis
Z0012_LB_E08_2_B03_B	14	6	LMG 1229	Alcaligenes	faecalis
Z0012_LB_E09_2_B02_A	14	6	LMG 1229	Alcaligenes	faecalis
Z0012_LB_E10_2_B02_B	14	6	LMG 1229	Alcaligenes	faecalis
Z0012_LB_E11_2_B01_A	14	6	LMG 1229	Alcaligenes	faecalis
Z0012_LB_E12_2_B01_B	14	6	LMG 1229	Alcaligenes	faecalis
Z0012_LB_F01_1_B06_A	14	6	LMG 1229	Alcaligenes	faecalis
Z0012_LB_F02_1_B06_B	14	6	LMG 1229	Alcaligenes	faecalis
Z0012_LB_F03_1_B05_A	14	6	LMG 1229	Alcaligenes	faecalis
Z0012_LB_F04_1_B05_B	14	6	LMG 1229	Alcaligenes	faecalis

Z0012_LB_F05_1_B04_A	14	6	LMG 1229	Alcaligenes	faecalis
Z0012_LB_F06_1_B04_B	14	6	LMG 1229	Alcaligenes	faecalis
Z0012_LB_F07_1_B03_A	14	6	LMG 1229	Alcaligenes	faecalis
Z0012_LB_F08_1_B03_B	14	6	LMG 1229	Alcaligenes	faecalis
Z0012_LB_F09_1_B02_A	14	6	LMG 1229	Alcaligenes	faecalis
Z0012_RB_D07_1_C09_A	14	6	LMG 1229	Alcaligenes	faecalis
Z0012_RB_D08_1_C09_B	14	6	LMG 1229	Alcaligenes	faecalis
Z0012_RB_D09_1_C08_A	14	6	LMG 1229	Alcaligenes	faecalis
Z0012_RB_D10_1_C08_B	14	6	LMG 1229	Alcaligenes	faecalis
Z0012_RB_D11_1_C07_A	14	6	LMG 1229	Alcaligenes	faecalis
Z0012_RB_D12_1_C07_B	14	6	LMG 1229	Alcaligenes	faecalis
Z0012_RB_E01_2_B12_A	14	6	LMG 1229	Alcaligenes	faecalis
Z0012_RB_E02_2_B12_B	14	6	LMG 1229	Alcaligenes	faecalis
Z0012_RB_E03_2_B11_A	14	6	LMG 1229	Alcaligenes	faecalis
Z0012_RB_E04_2_B11_B	14	6	LMG 1229	Alcaligenes	faecalis
Z0012_RB_E05_2_B10_A	14	6	LMG 1229	Alcaligenes	faecalis
Z0012_RB_E06_2_B10_B	14	6	LMG 1229	Alcaligenes	faecalis
Z0012_RB_E07_2_B09_A	14	6	LMG 1229	Alcaligenes	faecalis
Z0012_RB_E08_2_B09_B	14	6	LMG 1229	Alcaligenes	faecalis
Z0012_RB_E09_2_B08_A	14	6	LMG 1229	Alcaligenes	faecalis
Z0012_RB_E10_2_B08_B	14	6	LMG 1229	Alcaligenes	faecalis
Z0012_LB_F10_1_B02_B	15	7	LMG 1232	Bordetella	bronchiseptica
Z0012_LB_F12_1_B01_B	15	7	LMG 1232	Bordetella	bronchiseptica
Z0012_LB_G01_2_A06_A	15	7	LMG 1232	Bordetella	bronchiseptica
Z0012_LB_G06_2_A04_B	15	7	LMG 1232	Bordetella	bronchiseptica
Z0012_LB_G07_2_A03_A	15	7	LMG 1232	Bordetella	bronchiseptica
Z0012_LB_G09_2_A02_A	15	7	LMG 1232	Bordetella	bronchiseptica
Z0012_LB_G11_2_A01_A	15	7	LMG 1232	Bordetella	bronchiseptica
Z0012_LB_G12_2_A01_B	15	7	LMG 1232	Bordetella	bronchiseptica
Z0012_LB_H01_1_A06_A	15	7	LMG 1232	Bordetella	bronchiseptica
Z0012_LB_H02_1_A06_B	15	7	LMG 1232	Bordetella	bronchiseptica
Z0012_LB_H04_1_A05_B	15	7	LMG 1232	Bordetella	bronchiseptica
Z0012_LB_H06_1_A04_B	15	7	LMG 1232	Bordetella	bronchiseptica
Z0012_LB_H07_1_A03_A	15	7	LMG 1232	Bordetella	bronchiseptica
Z0012_LB_H09_1_A02_A	15	7	LMG 1232	Bordetella	bronchiseptica
Z0012_LB_H11_1_A01_A	15	7	LMG 1232	Bordetella	bronchiseptica
Z0012_LO_A01_1_E01_A	15	7	LMG 1232	Bordetella	bronchiseptica
Z0012_RB_E11_2_B07_A	15	7	LMG 1232	Bordetella	bronchiseptica
Z0012_RB_E12_2_B07_B	15	7	LMG 1232	Bordetella	bronchiseptica
Z0012_RB_F01_1_B12_A	15	7	LMG 1232	Bordetella	bronchiseptica
Z0012_RB_F02_1_B12_B	15	7	LMG 1232	Bordetella	bronchiseptica
Z0012_RB_F03_1_B11_A	15	7	LMG 1232	Bordetella	bronchiseptica
Z0012_RB_F04_1_B11_B	15	7	LMG 1232	Bordetella	bronchiseptica
Z0012_RB_F06_1_B10_B	15	7	LMG 1232	Bordetella	bronchiseptica
Z0012_RB_F09_1_B08_A	15	7	LMG 1232	Bordetella	bronchiseptica
Z0012_RB_G01_2_A12_A	15	7	LMG 1232	Bordetella	bronchiseptica
Z0012_RB_G03_2_A11_A	15	7	LMG 1232	Bordetella	bronchiseptica
Z0012_RB_G06_2_A10_B	15	7	LMG 1232	Bordetella	bronchiseptica
Z0012_RB_G10_2_A08_B	15	7	LMG 1232	Bordetella	bronchiseptica
Z0012_RB_G11_2_A07_A	15	7	LMG 1232	Bordetella	bronchiseptica

Z0012_RB_H01_1_A12_A	15	7	LMG 1232	Bordetella	bronchiseptica
Z0012_RB_H03_1_A11_A	15	7	LMG 1232	Bordetella	bronchiseptica
Z0012_RB_H04_1_A11_B	15	7	LMG 1232	Bordetella	bronchiseptica
Z0012_LB_G02_2_A06_B	16	13	LMG 13127	Azospirillum	brasilense
Z0012_LB_G03_2_A05_A	16	13	LMG 13127	Azospirillum	brasilense
Z0012_LB_G04_2_A05_B	16	13	LMG 13127	Azospirillum	brasilense
Z0012_LB_G05_2_A04_A	16	13	LMG 13127	Azospirillum	brasilense
Z0012_LB_G08_2_A03_B	16	13	LMG 13127	Azospirillum	brasilense
Z0012_LB_G10_2_A02_B	16	13	LMG 13127	Azospirillum	brasilense
Z0012_LB_H03_1_A05_A	16	13	LMG 13127	Azospirillum	brasilense
Z0012_LB_H05_1_A04_A	16	13	LMG 13127	Azospirillum	brasilense
Z0012_LB_H08_1_A03_B	16	13	LMG 13127	Azospirillum	brasilense
Z0012_LB_H10_1_A02_B	16	13	LMG 13127	Azospirillum	brasilense
Z0012_LB_H12_1_A01_B	16	13	LMG 13127	Azospirillum	brasilense
Z0012_RB_F10_1_B08_B	16	13	LMG 13127	Azospirillum	brasilense
Z0012_RB_F11_1_B07_A	16	13	LMG 13127	Azospirillum	brasilense
Z0012_RB_F12_1_B07_B	16	13	LMG 13127	Azospirillum	brasilense
Z0012_RB_G02_2_A12_B	16	13	LMG 13127	Azospirillum	brasilense
Z0012_RB_G04_2_A11_B	16	13	LMG 13127	Azospirillum	brasilense
Z0012_RB_G05_2_A10_A	16	13	LMG 13127	Azospirillum	brasilense
Z0012_RB_G07_2_A09_A	16	13	LMG 13127	Azospirillum	brasilense
Z0012_RB_G08_2_A09_B	16	13	LMG 13127	Azospirillum	brasilense
Z0012_RB_G09_2_A08_A	16	13	LMG 13127	Azospirillum	brasilense
Z0012_RB_G12_2_A07_B	16	13	LMG 13127	Azospirillum	brasilense
Z0012_RB_H02_1_A12_B	16	13	LMG 13127	Azospirillum	brasilense
Z0012_RB_H05_1_A10_A	16	13	LMG 13127	Azospirillum	brasilense
Z0012_RB_H08_1_A09_B	16	13	LMG 13127	Azospirillum	brasilense
Z0012_RB_H10_1_A08_B	16	13	LMG 13127	Azospirillum	brasilense
Z0012_RB_H12_1_A07_B	16	13	LMG 13127	Azospirillum	brasilense
Z0012_RO_A01_1_E07_A	16	13	LMG 13127	Azospirillum	brasilense
Z0012_RO_A02_1_E07_B	16	13	LMG 13127	Azospirillum	brasilense
Z0012_RO_A05_1_E09_A	16	13	LMG 13127	Azospirillum	brasilense
Z0012_RO_A07_1_E10_A	16	13	LMG 13127	Azospirillum	brasilense
Z0012_RO_A12_1_E12_B	16	13	LMG 13127	Azospirillum	brasilense
Z0012_RO_B03_2_E08_A	16	13	LMG 13127	Azospirillum	brasilense
Z0012_LO_A02_1_E01_B	17	12	LMG 129	Thalassobius	gelatinovorus
Z0012_LO_A03_1_E02_A	17	12	LMG 129	Thalassobius	gelatinovorus
Z0012_LO_A04_1_E02_B	17	12	LMG 129	Thalassobius	gelatinovorus
Z0012_LO_A06_1_E03_B	17	12	LMG 129	Thalassobius	gelatinovorus
Z0012_LO_A08_1_E04_B	17	12	LMG 129	Thalassobius	gelatinovorus
Z0012_LO_A10_1_E05_B	17	12	LMG 129	Thalassobius	gelatinovorus
Z0012_LO_A12_1_E06_B	17	12	LMG 129	Thalassobius	gelatinovorus
Z0012_LO_B04_2_E02_B	17	12	LMG 129	Thalassobius	gelatinovorus
Z0012_LO_B05_2_E03_A	17	12	LMG 129	Thalassobius	gelatinovorus
Z0012_LO_B07_2_E04_A	17	12	LMG 129	Thalassobius	gelatinovorus
Z0012_LO_B09_2_E05_A	17	12	LMG 129	Thalassobius	gelatinovorus
Z0012_LO_B10_2_E05_B	17	12	LMG 129	Thalassobius	gelatinovorus
Z0012_LO_B11_2_E06_A	17	12	LMG 129	Thalassobius	gelatinovorus
Z0012_LO_B12_2_E06_B	17	12	LMG 129	Thalassobius	gelatinovorus
Z0012_LO_C01_1_F01_A	17	12	LMG 129	Thalassobius	gelatinovorus

Z0012_LO_C02_1_F01_B	17	12	LMG 129	Thalassobius	gelatinovorus
Z0012_LO_C04_1_F02_B	17	12	LMG 129	Thalassobius	gelatinovorus
Z0012_LO_C07_1_F04_A	17	12	LMG 129	Thalassobius	gelatinovorus
Z0012_LO_C10_1_F05_B	17	12	LMG 129	Thalassobius	gelatinovorus
Z0012_LO_C12_1_F06_B	17	12	LMG 129	Thalassobius	gelatinovorus
Z0012_LO_D01_2_F01_A	17	12	LMG 129	Thalassobius	gelatinovorus
Z0012_RO_B02_2_E07_B	17	12	LMG 129	Thalassobius	gelatinovorus
Z0012_RO_B04_2_E08_B	17	12	LMG 129	Thalassobius	gelatinovorus
Z0012_RO_B06_2_E09_B	17	12	LMG 129	Thalassobius	gelatinovorus
Z0012_RO_B08_2_E10_B	17	12	LMG 129	Thalassobius	gelatinovorus
Z0012_RO_B11_2_E12_A	17	12	LMG 129	Thalassobius	gelatinovorus
Z0012_RO_B12_2_E12_B	17	12	LMG 129	Thalassobius	gelatinovorus
Z0012_RO_C02_1_F07_B	17	12	LMG 129	Thalassobius	gelatinovorus
Z0012_RO_C04_1_F08_B	17	12	LMG 129	Thalassobius	gelatinovorus
Z0012_RO_C05_1_F09_A	17	12	LMG 129	Thalassobius	gelatinovorus
Z0012_RO_C08_1_F10_B	17	12	LMG 129	Thalassobius	gelatinovorus
Z0012_RO_C09_1_F11_A	17	12	LMG 129	Thalassobius	gelatinovorus
Z0012_LO_A05_1_E03_A	18	8	LMG 1242	Pseudomonas	aeruginosa
Z0012_LO_A07_1_E04_A	18	8	LMG 1242	Pseudomonas	aeruginosa
Z0012_LO_A09_1_E05_A	18	8	LMG 1242	Pseudomonas	aeruginosa
Z0012_LO_A11_1_E06_A	18	8	LMG 1242	Pseudomonas	aeruginosa
Z0012_LO_B01_2_E01_A	18	8	LMG 1242	Pseudomonas	aeruginosa
Z0012_LO_B02_2_E01_B	18	8	LMG 1242	Pseudomonas	aeruginosa
Z0012_LO_B03_2_E02_A	18	8	LMG 1242	Pseudomonas	aeruginosa
Z0012_LO_B06_2_E03_B	18	8	LMG 1242	Pseudomonas	aeruginosa
Z0012_LO_B08_2_E04_B	18	8	LMG 1242	Pseudomonas	aeruginosa
Z0012_LO_C03_1_F02_A	18	8	LMG 1242	Pseudomonas	aeruginosa
Z0012_LO_C05_1_F03_A	18	8	LMG 1242	Pseudomonas	aeruginosa
Z0012_LO_C06_1_F03_B	18	8	LMG 1242	Pseudomonas	aeruginosa
Z0012_LO_C08_1_F04_B	18	8	LMG 1242	Pseudomonas	aeruginosa
Z0012_LO_C09_1_F05_A	18	8	LMG 1242	Pseudomonas	aeruginosa
Z0012_LO_C11_1_F06_A	18	8	LMG 1242	Pseudomonas	aeruginosa
Z0012_LO_D02_2_F01_B	18	8	LMG 1242	Pseudomonas	aeruginosa
Z0012_RB_H06_1_A10_B	18	8	LMG 1242	Pseudomonas	aeruginosa
Z0012_RB_H07_1_A09_A	18	8	LMG 1242	Pseudomonas	aeruginosa
Z0012_RB_H09_1_A08_A	18	8	LMG 1242	Pseudomonas	aeruginosa
Z0012_RB_H11_1_A07_A	18	8	LMG 1242	Pseudomonas	aeruginosa
Z0012_RO_A03_1_E08_A	18	8	LMG 1242	Pseudomonas	aeruginosa
Z0012_RO_A04_1_E08_B	18	8	LMG 1242	Pseudomonas	aeruginosa
Z0012_RO_A06_1_E09_B	18	8	LMG 1242	Pseudomonas	aeruginosa
Z0012_RO_A08_1_E10_B	18	8	LMG 1242	Pseudomonas	aeruginosa
Z0012_RO_A09_1_E11_A	18	8	LMG 1242	Pseudomonas	aeruginosa
Z0012_RO_A10_1_E11_B	18	8	LMG 1242	Pseudomonas	aeruginosa
Z0012_RO_A11_1_E12_A	18	8	LMG 1242	Pseudomonas	aeruginosa
Z0012_RO_B01_2_E07_A	18	8	LMG 1242	Pseudomonas	aeruginosa
Z0012_RO_B05_2_E09_A	18	8	LMG 1242	Pseudomonas	aeruginosa
Z0012_RO_B07_2_E10_A	18	8	LMG 1242	Pseudomonas	aeruginosa
Z0012_RO_B09_2_E11_A	18	8	LMG 1242	Pseudomonas	aeruginosa
Z0012_RO_B10_2_E11_B	18	8	LMG 1242	Pseudomonas	aeruginosa
Z0012_LO_D03_2_F02_A	19	11	LMG 1286	Pantoea	agglomerans

Z0012_LO_D04_2_F02_B	19	11	LMG 1286	Pantoea	agglomerans
Z0012_LO_D07_2_F04_A	19	11	LMG 1286	Pantoea	agglomerans
Z0012_LO_D09_2_F05_A	19	11	LMG 1286	Pantoea	agglomerans
Z0012_LO_E01_1_G01_A	19	11	LMG 1286	Pantoea	agglomerans
Z0012_LO_E02_1_G01_B	19	11	LMG 1286	Pantoea	agglomerans
Z0012_LO_E06_1_G03_B	19	11	LMG 1286	Pantoea	agglomerans
Z0012_LO_E08_1_G04_B	19	11	LMG 1286	Pantoea	agglomerans
Z0012_LO_E09_1_G05_A	19	11	LMG 1286	Pantoea	agglomerans
Z0012_LO_E10_1_G05_B	19	11	LMG 1286	Pantoea	agglomerans
Z0012_LO_F02_2_G01_B	19	11	LMG 1286	Pantoea	agglomerans
Z0012_LO_F04_2_G02_B	19	11	LMG 1286	Pantoea	agglomerans
Z0012_LO_F05_2_G03_A	19	11	LMG 1286	Pantoea	agglomerans
Z0012_LO_F06_2_G03_B	19	11	LMG 1286	Pantoea	agglomerans
Z0012_LO_F07_2_G04_A	19	11	LMG 1286	Pantoea	agglomerans
Z0012_LO_F12_2_G06_B	19	11	LMG 1286	Pantoea	agglomerans
Z0012_LO_G01_1_H01_A	19	11	LMG 1286	Pantoea	agglomerans
Z0012_LO_G03_1_H02_A	19	11	LMG 1286	Pantoea	agglomerans
Z0012_RO_C11_1_F12_A	19	11	LMG 1286	Pantoea	agglomerans
Z0012_RO_D01_2_F07_A	19	11	LMG 1286	Pantoea	agglomerans
Z0012_RO_D04_2_F08_B	19	11	LMG 1286	Pantoea	agglomerans
Z0012_RO_D06_2_F09_B	19	11	LMG 1286	Pantoea	agglomerans
Z0012_RO_D08_2_F10_B	19	11	LMG 1286	Pantoea	agglomerans
Z0012_RO_E01_1_G07_A	19	11	LMG 1286	Pantoea	agglomerans
Z0012_RO_E02_1_G07_B	19	11	LMG 1286	Pantoea	agglomerans
Z0012_RO_E03_1_G08_A	19	11	LMG 1286	Pantoea	agglomerans
Z0012_RO_E04_1_G08_B	19	11	LMG 1286	Pantoea	agglomerans
Z0012_RO_E05_1_G09_A	19	11	LMG 1286	Pantoea	agglomerans
Z0012_RO_E06_1_G09_B	19	11	LMG 1286	Pantoea	agglomerans
Z0012_RO_E11_1_G12_A	19	11	LMG 1286	Pantoea	agglomerans
Z0012_RO_E12_1_G12_B	19	11	LMG 1286	Pantoea	agglomerans
Z0012_RO_F02_2_G07_B	19	11	LMG 1286	Pantoea	agglomerans
Z0012_LO_F08_2_G04_B	20	9	LMG 12537	Rhizorhapis	suberifaciens
Z0012_LO_F09_2_G05_A	20	9	LMG 12537	Rhizorhapis	suberifaciens
Z0012_LO_F10_2_G05_B	20	9	LMG 12537	Rhizorhapis	suberifaciens
Z0012_LO_F11_2_G06_A	20	9	LMG 12537	Rhizorhapis	suberifaciens
Z0012_LO_G02_1_H01_B	20	9	LMG 12537	Rhizorhapis	suberifaciens
Z0012_LO_G04_1_H02_B	20	9	LMG 12537	Rhizorhapis	suberifaciens
Z0012_LO_G08_1_H04_B	20	9	LMG 12537	Rhizorhapis	suberifaciens
Z0012_LO_G09_1_H05_A	20	9	LMG 12537	Rhizorhapis	suberifaciens
Z0012_LO_H01_2_H01_A	20	9	LMG 12537	Rhizorhapis	suberifaciens
Z0012_LO_H02_2_H01_B	20	9	LMG 12537	Rhizorhapis	suberifaciens
Z0012_LO_H06_2_H03_B	20	9	LMG 12537	Rhizorhapis	suberifaciens
Z0012_LO_H07_2_H04_A	20	9	LMG 12537	Rhizorhapis	suberifaciens
Z0012_RO_E07_1_G10_A	20	9	LMG 12537	Rhizorhapis	suberifaciens
Z0012_RO_E08_1_G10_B	20	9	LMG 12537	Rhizorhapis	suberifaciens
Z0012_RO_E09_1_G11_A	20	9	LMG 12537	Rhizorhapis	suberifaciens
Z0012_RO_E10_1_G11_B	20	9	LMG 12537	Rhizorhapis	suberifaciens
Z0012_RO_F01_2_G07_A	20	9	LMG 12537	Rhizorhapis	suberifaciens
Z0012_RO_F03_2_G08_A	20	9	LMG 12537	Rhizorhapis	suberifaciens
Z0012_RO_F04_2_G08_B	20	9	LMG 12537	Rhizorhapis	suberifaciens

Z0012_RO_F05_2_G09_A	20	9	LMG 12537	Rhizorhapis	suberifaciens
Z0012_RO_F08_2_G10_B	20	9	LMG 12537	Rhizorhapis	suberifaciens
Z0012_RO_F09_2_G11_A	20	9	LMG 12537	Rhizorhapis	suberifaciens
Z0012_RO_F10_2_G11_B	20	9	LMG 12537	Rhizorhapis	suberifaciens
Z0012_RO_F11_2_G12_A	20	9	LMG 12537	Rhizorhapis	suberifaciens
Z0012_RO_G03_1_H08_A	20	9	LMG 12537	Rhizorhapis	suberifaciens
Z0012_RO_G04_1_H08_B	20	9	LMG 12537	Rhizorhapis	suberifaciens
Z0012_RO_G05_1_H09_A	20	9	LMG 12537	Rhizorhapis	suberifaciens
Z0012_RO_G06_1_H09_B	20	9	LMG 12537	Rhizorhapis	suberifaciens
Z0012_LO_G05_1_H03_A	21	10	LMG 12553	Sphingobium	xanthum
Z0012_LO_G06_1_H03_B	21	10	LMG 12553	Sphingobium	xanthum
Z0012_LO_G07_1_H04_A	21	10	LMG 12553	Sphingobium	xanthum
Z0012_LO_G10_1_H05_B	21	10	LMG 12553	Sphingobium	xanthum
Z0012_LO_G11_1_H06_A	21	10	LMG 12553	Sphingobium	xanthum
Z0012_LO_G12_1_H06_B	21	10	LMG 12553	Sphingobium	xanthum
Z0012_LO_H03_2_H02_A	21	10	LMG 12553	Sphingobium	xanthum
Z0012_LO_H04_2_H02_B	21	10	LMG 12553	Sphingobium	xanthum
Z0012_LO_H05_2_H03_A	21	10	LMG 12553	Sphingobium	xanthum
Z0012_LO_H08_2_H04_B	21	10	LMG 12553	Sphingobium	xanthum
Z0012_LO_H09_2_H05_A	21	10	LMG 12553	Sphingobium	xanthum
Z0012_LO_H10_2_H05_B	21	10	LMG 12553	Sphingobium	xanthum
Z0012_LO_H11_2_H06_A	21	10	LMG 12553	Sphingobium	xanthum
Z0012_LO_H12_2_H06_B	21	10	LMG 12553	Sphingobium	xanthum
Z0012_RB_A01_2_D12_A	21	10	LMG 12553	Sphingobium	xanthum
Z0012_RB_A02_2_D12_B	21	10	LMG 12553	Sphingobium	xanthum
Z0012_RB_A03_2_D11_A	21	10	LMG 12553	Sphingobium	xanthum
Z0012_RO_F06_2_G09_B	21	10	LMG 12553	Sphingobium	xanthum
Z0012_RO_F07_2_G10_A	21	10	LMG 12553	Sphingobium	xanthum
Z0012_RO_F12_2_G12_B	21	10	LMG 12553	Sphingobium	xanthum
Z0012_RO_G01_1_H07_A	21	10	LMG 12553	Sphingobium	xanthum
Z0012_RO_G02_1_H07_B	21	10	LMG 12553	Sphingobium	xanthum
Z0012_RO_G07_1_H10_A	21	10	LMG 12553	Sphingobium	xanthum
Z0012_RO_G08_1_H10_B	21	10	LMG 12553	Sphingobium	xanthum
Z0012_RO_G09_1_H11_A	21	10	LMG 12553	Sphingobium	xanthum
Z0012_RO_G10_1_H11_B	21	10	LMG 12553	Sphingobium	xanthum
Z0012_RO_G11_1_H12_A	21	10	LMG 12553	Sphingobium	xanthum
Z0012_RO_G12_1_H12_B	21	10	LMG 12553	Sphingobium	xanthum
Z0012_RO_H01_2_H07_A	21	10	LMG 12553	Sphingobium	xanthum
Z0012_RO_H02_2_H07_B	21	10	LMG 12553	Sphingobium	xanthum
Z0012_RO_H03_2_H08_A	21	10	LMG 12553	Sphingobium	xanthum
Z0012_RO_H04_2_H08_B	21	10	LMG 12553	Sphingobium	xanthum
Z0013_LB_A01_2_D06_A	22	14	LMG 13349	Staphylococcus	haemolyticus
Z0013_LB_A02_2_D06_B	22	14	LMG 13349	Staphylococcus	haemolyticus
Z0013_LB_A03_2_D05_A	22	14	LMG 13349	Staphylococcus	haemolyticus
Z0013_LB_A04_2_D05_B	22	14	LMG 13349	Staphylococcus	haemolyticus
Z0013_LB_A05_2_D04_A	22	14	LMG 13349	Staphylococcus	haemolyticus
Z0013_LB_A06_2_D04_B	22	14	LMG 13349	Staphylococcus	haemolyticus
Z0013_LB_F03_1_B05_A	22	14	LMG 13349	Staphylococcus	haemolyticus
Z0013_LB_H09_1_A02_A	22	14	LMG 13349	Staphylococcus	haemolyticus
Z0013_LB_H10_1_A02_B	22	14	LMG 13349	Staphylococcus	haemolyticus

Z0013_LB_H11_1_A01_A	22	14	LMG 13349	Staphylococcus	haemolyticus
Z0013_LB_H12_1_A01_B	22	14	LMG 13349	Staphylococcus	haemolyticus
Z0013_LO_A01_1_E01_A	22	14	LMG 13349	Staphylococcus	haemolyticus
Z0013_LO_A02_1_E01_B	22	14	LMG 13349	Staphylococcus	haemolyticus
Z0013_LO_A03_1_E02_A	22	14	LMG 13349	Staphylococcus	haemolyticus
Z0013_LO_A04_1_E02_B	22	14	LMG 13349	Staphylococcus	haemolyticus
Z0013_LO_A05_1_E03_A	22	14	LMG 13349	Staphylococcus	haemolyticus
Z0013_LO_F02_2_G01_B	22	14	LMG 13349	Staphylococcus	haemolyticus
Z0013_LO_F03_2_G02_A	22	14	LMG 13349	Staphylococcus	haemolyticus
Z0013_LO_H09_2_H05_A	22	14	LMG 13349	Staphylococcus	haemolyticus
Z0013_LO_H10_2_H05_B	22	14	LMG 13349	Staphylococcus	haemolyticus
Z0013_LO_H11_2_H06_A	22	14	LMG 13349	Staphylococcus	haemolyticus
Z0013_LO_H12_2_H06_B	22	14	LMG 13349	Staphylococcus	haemolyticus
Z0013_RB_A01_2_D12_A	22	14	LMG 13349	Staphylococcus	haemolyticus
Z0013_RB_A02_2_D12_B	22	14	LMG 13349	Staphylococcus	haemolyticus
Z0013_RB_E08_2_B09_B	22	14	LMG 13349	Staphylococcus	haemolyticus
Z0013_RB_H07_1_A09_A	22	14	LMG 13349	Staphylococcus	haemolyticus
Z0013_RB_H08_1_A09_B	22	14	LMG 13349	Staphylococcus	haemolyticus
Z0013_RB_H09_1_A08_A	22	14	LMG 13349	Staphylococcus	haemolyticus
Z0013_RB_H10_1_A08_B	22	14	LMG 13349	Staphylococcus	haemolyticus
Z0013_RB_H11_1_A07_A	22	14	LMG 13349	Staphylococcus	haemolyticus
Z0013_RB_H12_1_A07_B	22	14	LMG 13349	Staphylococcus	haemolyticus
Z0013_RO_F05_2_G09_A	22	14	LMG 13349	Staphylococcus	haemolyticus
Z0013_LB_A07_2_D03_A	23	15	LMG 1345	Leeuwenhoekiella	marinoflava
Z0013_LB_A08_2_D03_B	23	15	LMG 1345	Leeuwenhoekiella	marinoflava
Z0013_LB_A09_2_D02_A	23	15	LMG 1345	Leeuwenhoekiella	marinoflava
Z0013_LB_A10_2_D02_B	23	15	LMG 1345	Leeuwenhoekiella	marinoflava
Z0013_LB_A11_2_D01_A	23	15	LMG 1345	Leeuwenhoekiella	marinoflava
Z0013_LO_A06_1_E03_B	23	15	LMG 1345	Leeuwenhoekiella	marinoflava
Z0013_LO_A07_1_E04_A	23	15	LMG 1345	Leeuwenhoekiella	marinoflava
Z0013_LO_A08_1_E04_B	23	15	LMG 1345	Leeuwenhoekiella	marinoflava
Z0013_LO_A09_1_E05_A	23	15	LMG 1345	Leeuwenhoekiella	marinoflava
Z0013_LO_A10_1_E05_B	23	15	LMG 1345	Leeuwenhoekiella	marinoflava
Z0013_LO_A11_1_E06_A	23	15	LMG 1345	Leeuwenhoekiella	marinoflava
Z0013_LO_A12_1_E06_B	23	15	LMG 1345	Leeuwenhoekiella	marinoflava
Z0013_LO_B01_2_E01_A	23	15	LMG 1345	Leeuwenhoekiella	marinoflava
Z0013_LO_B02_2_E01_B	23	15	LMG 1345	Leeuwenhoekiella	marinoflava
Z0013_RB_A03_2_D11_A	23	15	LMG 1345	Leeuwenhoekiella	marinoflava
Z0013_RB_A04_2_D11_B	23	15	LMG 1345	Leeuwenhoekiella	marinoflava
Z0013_RB_A05_2_D10_A	23	15	LMG 1345	Leeuwenhoekiella	marinoflava
Z0013_RB_A06_2_D10_B	23	15	LMG 1345	Leeuwenhoekiella	marinoflava
Z0013_RB_A07_2_D09_A	23	15	LMG 1345	Leeuwenhoekiella	marinoflava
Z0013_RB_A08_2_D09_B	23	15	LMG 1345	Leeuwenhoekiella	marinoflava
Z0013_RB_A09_2_D08_A	23	15	LMG 1345	Leeuwenhoekiella	marinoflava
Z0013_RO_A01_1_E07_A	23	15	LMG 1345	Leeuwenhoekiella	marinoflava
Z0013_RO_A02_1_E07_B	23	15	LMG 1345	Leeuwenhoekiella	marinoflava
Z0013_RO_A03_1_E08_A	23	15	LMG 1345	Leeuwenhoekiella	marinoflava
Z0013_RO_A04_1_E08_B	23	15	LMG 1345	Leeuwenhoekiella	marinoflava
Z0013_RO_A05_1_E09_A	23	15	LMG 1345	Leeuwenhoekiella	marinoflava
Z0013_RO_A06_1_E09_B	23	15	LMG 1345	Leeuwenhoekiella	marinoflava

Z0013_RO_A07_1_E10_A	23	15	LMG 1345	Leeuwenhoekiella	marinoflava
Z0013_RO_A08_1_E10_B	23	15	LMG 1345	Leeuwenhoekiella	marinoflava
Z0013_RO_A09_1_E11_A	23	15	LMG 1345	Leeuwenhoekiella	marinoflava
Z0013_RO_A10_1_E11_B	23	15	LMG 1345	Leeuwenhoekiella	marinoflava
Z0013_RO_A11_1_E12_A	23	15	LMG 1345	Leeuwenhoekiella	marinoflava
Z0013_LB_A12_2_D01_B	24	16	LMG 1346	Marinilabilia	salmonicolor
Z0013_LB_B01_1_D06_A	24	16	LMG 1346	Marinilabilia	salmonicolor
Z0013_LB_B02_1_D06_B	24	16	LMG 1346	Marinilabilia	salmonicolor
Z0013_LB_B03_1_D05_A	24	16	LMG 1346	Marinilabilia	salmonicolor
Z0013_LB_B04_1_D05_B	24	16	LMG 1346	Marinilabilia	salmonicolor
Z0013_LB_B05_1_D04_A	24	16	LMG 1346	Marinilabilia	salmonicolor
Z0013_LB_B06_1_D04_B	24	16	LMG 1346	Marinilabilia	salmonicolor
Z0013_LB_B07_1_D03_A	24	16	LMG 1346	Marinilabilia	salmonicolor
Z0013_LB_B08_1_D03_B	24	16	LMG 1346	Marinilabilia	salmonicolor
Z0013_LO_B03_2_E02_A	24	16	LMG 1346	Marinilabilia	salmonicolor
Z0013_LO_B04_2_E02_B	24	16	LMG 1346	Marinilabilia	salmonicolor
Z0013_LO_B05_2_E03_A	24	16	LMG 1346	Marinilabilia	salmonicolor
Z0013_LO_B06_2_E03_B	24	16	LMG 1346	Marinilabilia	salmonicolor
Z0013_LO_B07_2_E04_A	24	16	LMG 1346	Marinilabilia	salmonicolor
Z0013_LO_B08_2_E04_B	24	16	LMG 1346	Marinilabilia	salmonicolor
Z0013_RB_A10_2_D08_B	24	16	LMG 1346	Marinilabilia	salmonicolor
Z0013_RB_A11_2_D07_A	24	16	LMG 1346	Marinilabilia	salmonicolor
Z0013_RB_A12_2_D07_B	24	16	LMG 1346	Marinilabilia	salmonicolor
Z0013_RB_B01_1_D12_A	24	16	LMG 1346	Marinilabilia	salmonicolor
Z0013_RB_B02_1_D12_B	24	16	LMG 1346	Marinilabilia	salmonicolor
Z0013_RB_B03_1_D11_A	24	16	LMG 1346	Marinilabilia	salmonicolor
Z0013_RB_B04_1_D11_B	24	16	LMG 1346	Marinilabilia	salmonicolor
Z0013_RB_B05_1_D10_A	24	16	LMG 1346	Marinilabilia	salmonicolor
Z0013_RB_B06_1_D10_B	24	16	LMG 1346	Marinilabilia	salmonicolor
Z0013_RO_A12_1_E12_B	24	16	LMG 1346	Marinilabilia	salmonicolor
Z0013_RO_B01_2_E07_A	24	16	LMG 1346	Marinilabilia	salmonicolor
Z0013_RO_B02_2_E07_B	24	16	LMG 1346	Marinilabilia	salmonicolor
Z0013_RO_B03_2_E08_A	24	16	LMG 1346	Marinilabilia	salmonicolor
Z0013_RO_B04_2_E08_B	24	16	LMG 1346	Marinilabilia	salmonicolor
Z0013_RO_B05_2_E09_A	24	16	LMG 1346	Marinilabilia	salmonicolor
Z0013_RO_B06_2_E09_B	24	16	LMG 1346	Marinilabilia	salmonicolor
Z0013_RO_B07_2_E10_A	24	16	LMG 1346	Marinilabilia	salmonicolor
Z0013_LB_B09_1_D02_A	25	17	LMG 1408	Gluconobacter	oxydans
Z0013_LB_B10_1_D02_B	25	17	LMG 1408	Gluconobacter	oxydans
Z0013_LB_B11_1_D01_A	25	17	LMG 1408	Gluconobacter	oxydans
Z0013_LB_B12_1_D01_B	25	17	LMG 1408	Gluconobacter	oxydans
Z0013_LO_B09_2_E05_A	25	17	LMG 1408	Gluconobacter	oxydans
Z0013_LO_B10_2_E05_B	25	17	LMG 1408	Gluconobacter	oxydans
Z0013_LO_B11_2_E06_A	25	17	LMG 1408	Gluconobacter	oxydans
Z0013_LO_B12_2_E06_B	25	17	LMG 1408	Gluconobacter	oxydans
Z0013_LO_C01_1_F01_A	25	17	LMG 1408	Gluconobacter	oxydans
Z0013_LO_C02_1_F01_B	25	17	LMG 1408	Gluconobacter	oxydans
Z0013_LO_C03_1_F02_A	25	17	LMG 1408	Gluconobacter	oxydans
Z0013_LO_C04_1_F02_B	25	17	LMG 1408	Gluconobacter	oxydans
Z0013_LO_C05_1_F03_A	25	17	LMG 1408	Gluconobacter	oxydans

Z0013_RB_B07_1_D09_A	25	17	LMG 1408	Gluconobacter	oxydans
Z0013_RB_B08_1_D09_B	25	17	LMG 1408	Gluconobacter	oxydans
Z0013_RB_B09_1_D08_A	25	17	LMG 1408	Gluconobacter	oxydans
Z0013_RB_B10_1_D08_B	25	17	LMG 1408	Gluconobacter	oxydans
Z0013_RB_B11_1_D07_A	25	17	LMG 1408	Gluconobacter	oxydans
Z0013_RB_B12_1_D07_B	25	17	LMG 1408	Gluconobacter	oxydans
Z0013_RB_C01_2_C12_A	25	17	LMG 1408	Gluconobacter	oxydans
Z0013_RO_B08_2_E10_B	25	17	LMG 1408	Gluconobacter	oxydans
Z0013_RO_B09_2_E11_A	25	17	LMG 1408	Gluconobacter	oxydans
Z0013_RO_B10_2_E11_B	25	17	LMG 1408	Gluconobacter	oxydans
Z0013_RO_B11_2_E12_A	25	17	LMG 1408	Gluconobacter	oxydans
Z0013_RO_B12_2_E12_B	25	17	LMG 1408	Gluconobacter	oxydans
Z0013_RO_C01_1_F07_A	25	17	LMG 1408	Gluconobacter	oxydans
Z0013_RO_C02_1_F07_B	25	17	LMG 1408	Gluconobacter	oxydans
Z0013_RO_C03_1_F08_A	25	17	LMG 1408	Gluconobacter	oxydans
Z0013_RO_C04_1_F08_B	25	17	LMG 1408	Gluconobacter	oxydans
Z0013_RO_C05_1_F09_A	25	17	LMG 1408	Gluconobacter	oxydans
Z0013_RO_C06_1_F09_B	25	17	LMG 1408	Gluconobacter	oxydans
Z0013_RO_C07_1_F10_A	25	17	LMG 1408	Gluconobacter	oxydans
Z0013_LB_C01_2_C06_A	26	18	LMG 1527	Komagataeibacter	hansenii
Z0013_LB_C02_2_C06_B	26	18	LMG 1527	Komagataeibacter	hansenii
Z0013_LB_C03_2_C05_A	26	18	LMG 1527	Komagataeibacter	hansenii
Z0013_LB_C04_2_C05_B	26	18	LMG 1527	Komagataeibacter	hansenii
Z0013_LB_C05_2_C04_A	26	18	LMG 1527	Komagataeibacter	hansenii
Z0013_LB_C06_2_C04_B	26	18	LMG 1527	Komagataeibacter	hansenii
Z0013_LB_C07_2_C03_A	26	18	LMG 1527	Komagataeibacter	hansenii
Z0013_LB_C08_2_C03_B	26	18	LMG 1527	Komagataeibacter	hansenii
Z0013_LB_C09_2_C02_A	26	18	LMG 1527	Komagataeibacter	hansenii
Z0013_LO_C06_1_F03_B	26	18	LMG 1527	Komagataeibacter	hansenii
Z0013_LO_C07_1_F04_A	26	18	LMG 1527	Komagataeibacter	hansenii
Z0013_LO_C08_1_F04_B	26	18	LMG 1527	Komagataeibacter	hansenii
Z0013_LO_C09_1_F05_A	26	18	LMG 1527	Komagataeibacter	hansenii
Z0013_LO_C10_1_F05_B	26	18	LMG 1527	Komagataeibacter	hansenii
Z0013_LO_C11_1_F06_A	26	18	LMG 1527	Komagataeibacter	hansenii
Z0013_LO_C12_1_F06_B	26	18	LMG 1527	Komagataeibacter	hansenii
Z0013_RB_C02_2_C12_B	26	18	LMG 1527	Komagataeibacter	hansenii
Z0013_RB_C03_2_C11_A	26	18	LMG 1527	Komagataeibacter	hansenii
Z0013_RB_C04_2_C11_B	26	18	LMG 1527	Komagataeibacter	hansenii
Z0013_RB_C05_2_C10_A	26	18	LMG 1527	Komagataeibacter	hansenii
Z0013_RB_C06_2_C10_B	26	18	LMG 1527	Komagataeibacter	hansenii
Z0013_RB_C07_2_C09_A	26	18	LMG 1527	Komagataeibacter	hansenii
Z0013_RB_C08_2_C09_B	26	18	LMG 1527	Komagataeibacter	hansenii
Z0013_RO_C08_1_F10_B	26	18	LMG 1527	Komagataeibacter	hansenii
Z0013_RO_C09_1_F11_A	26	18	LMG 1527	Komagataeibacter	hansenii
Z0013_RO_C10_1_F11_B	26	18	LMG 1527	Komagataeibacter	hansenii
Z0013_RO_C11_1_F12_A	26	18	LMG 1527	Komagataeibacter	hansenii
Z0013_RO_C12_1_F12_B	26	18	LMG 1527	Komagataeibacter	hansenii
Z0013_RO_D01_2_F07_A	26	18	LMG 1527	Komagataeibacter	hansenii
Z0013_RO_D02_2_F07_B	26	18	LMG 1527	Komagataeibacter	hansenii
Z0013_RO_D03_2_F08_A	26	18	LMG 1527	Komagataeibacter	hansenii

Z0013_RO_D04_2_F08_B	26	18	LMG 1527	Komagataeibacter	hansenii
Z0013_LB_C10_2_C02_B	27	19	LMG 15863	Haemophilus	influenzae
Z0013_LB_C11_2_C01_A	27	19	LMG 15863	Haemophilus	influenzae
Z0013_LB_C12_2_C01_B	27	19	LMG 15863	Haemophilus	influenzae
Z0013_LB_D01_1_C06_A	27	19	LMG 15863	Haemophilus	influenzae
Z0013_LB_D02_1_C06_B	27	19	LMG 15863	Haemophilus	influenzae
Z0013_LB_D03_1_C05_A	27	19	LMG 15863	Haemophilus	influenzae
Z0013_LB_D06_1_C04_B	27	19	LMG 15863	Haemophilus	influenzae
Z0013_LB_D07_1_C03_A	27	19	LMG 15863	Haemophilus	influenzae
Z0013_LO_D01_2_F01_A	27	19	LMG 15863	Haemophilus	influenzae
Z0013_LO_D02_2_F01_B	27	19	LMG 15863	Haemophilus	influenzae
Z0013_LO_D03_2_F02_A	27	19	LMG 15863	Haemophilus	influenzae
Z0013_LO_D05_2_F03_A	27	19	LMG 15863	Haemophilus	influenzae
Z0013_LO_D06_2_F03_B	27	19	LMG 15863	Haemophilus	influenzae
Z0013_RB_C09_2_C08_A	27	19	LMG 15863	Haemophilus	influenzae
Z0013_RB_C10_2_C08_B	27	19	LMG 15863	Haemophilus	influenzae
Z0013_RB_C11_2_C07_A	27	19	LMG 15863	Haemophilus	influenzae
Z0013_RB_C12_2_C07_B	27	19	LMG 15863	Haemophilus	influenzae
Z0013_RB_D01_1_C12_A	27	19	LMG 15863	Haemophilus	influenzae
Z0013_RB_D03_1_C11_A	27	19	LMG 15863	Haemophilus	influenzae
Z0013_RB_D04_1_C11_B	27	19	LMG 15863	Haemophilus	influenzae
Z0013_RB_D06_1_C10_B	27	19	LMG 15863	Haemophilus	influenzae
Z0013_RO_D05_2_F09_A	27	19	LMG 15863	Haemophilus	influenzae
Z0013_RO_D07_2_F10_A	27	19	LMG 15863	Haemophilus	influenzae
Z0013_RO_D08_2_F10_B	27	19	LMG 15863	Haemophilus	influenzae
Z0013_RO_D09_2_F11_A	27	19	LMG 15863	Haemophilus	influenzae
Z0013_LB_D04_1_C05_B	27	19	LMG 15863	Haemophilus	influenzae
Z0013_LB_D05_1_C04_A	27	19	LMG 15863	Haemophilus	influenzae
Z0013_LB_D08_1_C03_B	27	19	LMG 15863	Haemophilus	influenzae
Z0013_LO_D04_2_F02_B	27	19	LMG 15863	Haemophilus	influenzae
Z0013_RB_D02_1_C12_B	27	19	LMG 15863	Haemophilus	influenzae
Z0013_RB_D05_1_C10_A	27	19	LMG 15863	Haemophilus	influenzae
Z0013_RO_D06_2_F09_B	27	19	LMG 15863	Haemophilus	influenzae
Z0013_LB_D09_1_C02_A	28	20	LMG 1617	Acetobacter	lovaniensis
Z0013_LB_D10_1_C02_B	28	20	LMG 1617	Acetobacter	lovaniensis
Z0013_LB_D11_1_C01_A	28	20	LMG 1617	Acetobacter	lovaniensis
Z0013_LB_D12_1_C01_B	28	20	LMG 1617	Acetobacter	lovaniensis
Z0013_LB_E01_2_B06_A	28	20	LMG 1617	Acetobacter	lovaniensis
Z0013_LB_E02_2_B06_B	28	20	LMG 1617	Acetobacter	lovaniensis
Z0013_LO_D07_2_F04_A	28	20	LMG 1617	Acetobacter	lovaniensis
Z0013_LO_D08_2_F04_B	28	20	LMG 1617	Acetobacter	lovaniensis
Z0013_LO_D09_2_F05_A	28	20	LMG 1617	Acetobacter	lovaniensis
Z0013_LO_D10_2_F05_B	28	20	LMG 1617	Acetobacter	lovaniensis
Z0013_LO_D11_2_F06_A	28	20	LMG 1617	Acetobacter	lovaniensis
Z0013_LO_D12_2_F06_B	28	20	LMG 1617	Acetobacter	lovaniensis
Z0013_LO_E01_1_G01_A	28	20	LMG 1617	Acetobacter	lovaniensis
Z0013_RB_D07_1_C09_A	28	20	LMG 1617	Acetobacter	lovaniensis
Z0013_RB_D08_1_C09_B	28	20	LMG 1617	Acetobacter	lovaniensis
Z0013_RB_D09_1_C08_A	28	20	LMG 1617	Acetobacter	lovaniensis
Z0013_RB_D10_1_C08_B	28	20	LMG 1617	Acetobacter	lovaniensis

Z0013_RB_D11_1_C07_A	28	20	LMG 1617	Acetobacter	lovaniensis
Z0013_RB_D12_1_C07_B	28	20	LMG 1617	Acetobacter	lovaniensis
Z0013_RB_E01_2_B12_A	28	20	LMG 1617	Acetobacter	lovaniensis
Z0013_RB_E02_2_B12_B	28	20	LMG 1617	Acetobacter	lovaniensis
Z0013_RO_D10_2_F11_B	28	20	LMG 1617	Acetobacter	lovaniensis
Z0013_RO_D11_2_F12_A	28	20	LMG 1617	Acetobacter	lovaniensis
Z0013_RO_D12_2_F12_B	28	20	LMG 1617	Acetobacter	lovaniensis
Z0013_RO_E01_1_G07_A	28	20	LMG 1617	Acetobacter	lovaniensis
Z0013_RO_E02_1_G07_B	28	20	LMG 1617	Acetobacter	lovaniensis
Z0013_RO_E03_1_G08_A	28	20	LMG 1617	Acetobacter	lovaniensis
Z0013_RO_E04_1_G08_B	28	20	LMG 1617	Acetobacter	lovaniensis
Z0013_LB_E03_2_B05_A	29	21	LMG 16409	Pandoraea	apista
Z0013_LB_E04_2_B05_B	29	21	LMG 16409	Pandoraea	apista
Z0013_LB_E05_2_B04_A	29	21	LMG 16409	Pandoraea	apista
Z0013_LB_E06_2_B04_B	29	21	LMG 16409	Pandoraea	apista
Z0013_LB_E07_2_B03_A	29	21	LMG 16409	Pandoraea	apista
Z0013_LB_E08_2_B03_B	29	21	LMG 16409	Pandoraea	apista
Z0013_LO_E02_1_G01_B	29	21	LMG 16409	Pandoraea	apista
Z0013_LO_E03_1_G02_A	29	21	LMG 16409	Pandoraea	apista
Z0013_LO_E04_1_G02_B	29	21	LMG 16409	Pandoraea	apista
Z0013_LO_E05_1_G03_A	29	21	LMG 16409	Pandoraea	apista
Z0013_LO_E06_1_G03_B	29	21	LMG 16409	Pandoraea	apista
Z0013_LO_E07_1_G04_A	29	21	LMG 16409	Pandoraea	apista
Z0013_LO_E08_1_G04_B	29	21	LMG 16409	Pandoraea	apista
Z0013_LO_E09_1_G05_A	29	21	LMG 16409	Pandoraea	apista
Z0013_LO_E10_1_G05_B	29	21	LMG 16409	Pandoraea	apista
Z0013_LO_E11_1_G06_A	29	21	LMG 16409	Pandoraea	apista
Z0013_RB_E03_2_B11_A	29	21	LMG 16409	Pandoraea	apista
Z0013_RB_E04_2_B11_B	29	21	LMG 16409	Pandoraea	apista
Z0013_RB_E05_2_B10_A	29	21	LMG 16409	Pandoraea	apista
Z0013_RB_E06_2_B10_B	29	21	LMG 16409	Pandoraea	apista
Z0013_RB_E07_2_B09_A	29	21	LMG 16409	Pandoraea	apista
Z0013_RO_E05_1_G09_A	29	21	LMG 16409	Pandoraea	apista
Z0013_RO_E06_1_G09_B	29	21	LMG 16409	Pandoraea	apista
Z0013_RO_E07_1_G10_A	29	21	LMG 16409	Pandoraea	apista
Z0013_RO_E08_1_G10_B	29	21	LMG 16409	Pandoraea	apista
Z0013_RO_E09_1_G11_A	29	21	LMG 16409	Pandoraea	apista
Z0013_RO_E10_1_G11_B	29	21	LMG 16409	Pandoraea	apista
Z0013_RO_E11_1_G12_A	29	21	LMG 16409	Pandoraea	apista
Z0013_LB_E09_2_B02_A	30	22	LMG 16673	Lactobacillus	paraplantarum
Z0013_LB_E10_2_B02_B	30	22	LMG 16673	Lactobacillus	paraplantarum
Z0013_LB_E11_2_B01_A	30	22	LMG 16673	Lactobacillus	paraplantarum
Z0013_LB_E12_2_B01_B	30	22	LMG 16673	Lactobacillus	paraplantarum
Z0013_LB_F01_1_B06_A	30	22	LMG 16673	Lactobacillus	paraplantarum
Z0013_LB_F02_1_B06_B	30	22	LMG 16673	Lactobacillus	paraplantarum
Z0013_LB_F04_1_B05_B	30	22	LMG 16673	Lactobacillus	paraplantarum
Z0013_LB_F05_1_B04_A	30	22	LMG 16673	Lactobacillus	paraplantarum
Z0013_LB_F06_1_B04_B	30	22	LMG 16673	Lactobacillus	paraplantarum
Z0013_LB_F07_1_B03_A	30	22	LMG 16673	Lactobacillus	paraplantarum
Z0013_LB_F08_1_B03_B	30	22	LMG 16673	Lactobacillus	paraplantarum

Z0013_LB_F09_1_B02_A	30	22	LMG 16673	Lactobacillus	paraplantarum
Z0013_LB_F10_1_B02_B	30	22	LMG 16673	Lactobacillus	paraplantarum
Z0013_LB_G01_2_A06_A	30	22	LMG 16673	Lactobacillus	paraplantarum
Z0013_LO_E12_1_G06_B	30	22	LMG 16673	Lactobacillus	paraplantarum
Z0013_LO_F01_2_G01_A	30	22	LMG 16673	Lactobacillus	paraplantarum
Z0013_LO_F04_2_G02_B	30	22	LMG 16673	Lactobacillus	paraplantarum
Z0013_LO_F05_2_G03_A	30	22	LMG 16673	Lactobacillus	paraplantarum
Z0013_LO_F06_2_G03_B	30	22	LMG 16673	Lactobacillus	paraplantarum
Z0013_LO_F07_2_G04_A	30	22	LMG 16673	Lactobacillus	paraplantarum
Z0013_LO_F09_2_G05_A	30	22	LMG 16673	Lactobacillus	paraplantarum
Z0013_LO_F10_2_G05_B	30	22	LMG 16673	Lactobacillus	paraplantarum
Z0013_RB_E09_2_B08_A	30	22	LMG 16673	Lactobacillus	paraplantarum
Z0013_RB_E10_2_B08_B	30	22	LMG 16673	Lactobacillus	paraplantarum
Z0013_RB_E11_2_B07_A	30	22	LMG 16673	Lactobacillus	paraplantarum
Z0013_RB_F01_1_B12_A	30	22	LMG 16673	Lactobacillus	paraplantarum
Z0013_RB_F04_1_B11_B	30	22	LMG 16673	Lactobacillus	paraplantarum
Z0013_RB_F07_1_B09_A	30	22	LMG 16673	Lactobacillus	paraplantarum
Z0013_RB_F08_1_B09_B	30	22	LMG 16673	Lactobacillus	paraplantarum
Z0013_RB_F09_1_B08_A	30	22	LMG 16673	Lactobacillus	paraplantarum
Z0013_RO_E12_1_G12_B	30	22	LMG 16673	Lactobacillus	paraplantarum
Z0013_RO_F01_2_G07_A	30	22	LMG 16673	Lactobacillus	paraplantarum
Z0013_LB_G04_2_A05_B	31	23	LMG 1668	Acidomonas	methanolica
Z0013_LB_G05_2_A04_A	31	23	LMG 1668	Acidomonas	methanolica
Z0013_LB_G08_2_A03_B	31	23	LMG 1668	Acidomonas	methanolica
Z0013_LB_G11_2_A01_A	31	23	LMG 1668	Acidomonas	methanolica
Z0013_LB_H04_1_A05_B	31	23	LMG 1668	Acidomonas	methanolica
Z0013_LO_F12_2_G06_B	31	23	LMG 1668	Acidomonas	methanolica
Z0013_LO_G01_1_H01_A	31	23	LMG 1668	Acidomonas	methanolica
Z0013_LO_G03_1_H02_A	31	23	LMG 1668	Acidomonas	methanolica
Z0013_LO_G04_1_H02_B	31	23	LMG 1668	Acidomonas	methanolica
Z0013_LO_G06_1_H03_B	31	23	LMG 1668	Acidomonas	methanolica
Z0013_LO_G08_1_H04_B	31	23	LMG 1668	Acidomonas	methanolica
Z0013_LO_G10_1_H05_B	31	23	LMG 1668	Acidomonas	methanolica
Z0013_LO_H02_2_H01_B	31	23	LMG 1668	Acidomonas	methanolica
Z0013_RB_F10_1_B08_B	31	23	LMG 1668	Acidomonas	methanolica
Z0013_RB_G01_2_A12_A	31	23	LMG 1668	Acidomonas	methanolica
Z0013_RB_G02_2_A12_B	31	23	LMG 1668	Acidomonas	methanolica
Z0013_RB_G04_2_A11_B	31	23	LMG 1668	Acidomonas	methanolica
Z0013_RB_G06_2_A10_B	31	23	LMG 1668	Acidomonas	methanolica
Z0013_RB_G07_2_A09_A	31	23	LMG 1668	Acidomonas	methanolica
Z0013_RB_G08_2_A09_B	31	23	LMG 1668	Acidomonas	methanolica
Z0013_RB_G09_2_A08_A	31	23	LMG 1668	Acidomonas	methanolica
Z0013_RB_G12_2_A07_B	31	23	LMG 1668	Acidomonas	methanolica
Z0013_RB_H02_1_A12_B	31	23	LMG 1668	Acidomonas	methanolica
Z0013_RO_F11_2_G12_A	31	23	LMG 1668	Acidomonas	methanolica
Z0013_RO_G03_1_H08_A	31	23	LMG 1668	Acidomonas	methanolica
Z0013_RO_G04_1_H08_B	31	23	LMG 1668	Acidomonas	methanolica
Z0013_RO_G07_1_H10_A	31	23	LMG 1668	Acidomonas	methanolica
Z0013_RO_G08_1_H10_B	31	23	LMG 1668	Acidomonas	methanolica
Z0013_RO_G09_1_H11_A	31	23	LMG 1668	Acidomonas	methanolica

Z0013_RO_G10_1_H11_B	31	23	LMG 1668	Acidomonas	methanolica
Z0013_RO_G11_1_H12_A	31	23	LMG 1668	Acidomonas	methanolica
Z0013_RO_G12_1_H12_B	31	23	LMG 1668	Acidomonas	methanolica
Z0013_LB_G10_2_A02_B	32	24	LMG 17677	Lactobacillus	pentosus
Z0013_LB_G12_2_A01_B	32	24	LMG 17677	Lactobacillus	pentosus
Z0013_LB_H01_1_A06_A	32	24	LMG 17677	Lactobacillus	pentosus
Z0013_LB_H02_1_A06_B	32	24	LMG 17677	Lactobacillus	pentosus
Z0013_LB_H03_1_A05_A	32	24	LMG 17677	Lactobacillus	pentosus
Z0013_LB_H05_1_A04_A	32	24	LMG 17677	Lactobacillus	pentosus
Z0013_LB_H06_1_A04_B	32	24	LMG 17677	Lactobacillus	pentosus
Z0013_LB_H07_1_A03_A	32	24	LMG 17677	Lactobacillus	pentosus
Z0013_LB_H08_1_A03_B	32	24	LMG 17677	Lactobacillus	pentosus
Z0013_LO_G11_1_H06_A	32	24	LMG 17677	Lactobacillus	pentosus
Z0013_LO_H01_2_H01_A	32	24	LMG 17677	Lactobacillus	pentosus
Z0013_LO_H03_2_H02_A	32	24	LMG 17677	Lactobacillus	pentosus
Z0013_LO_H04_2_H02_B	32	24	LMG 17677	Lactobacillus	pentosus
Z0013_LO_H05_2_H03_A	32	24	LMG 17677	Lactobacillus	pentosus
Z0013_LO_H06_2_H03_B	32	24	LMG 17677	Lactobacillus	pentosus
Z0013_LO_H07_2_H04_A	32	24	LMG 17677	Lactobacillus	pentosus
Z0013_LO_H08_2_H04_B	32	24	LMG 17677	Lactobacillus	pentosus
Z0013_RB_G03_2_A11_A	32	24	LMG 17677	Lactobacillus	pentosus
Z0013_RB_G05_2_A10_A	32	24	LMG 17677	Lactobacillus	pentosus
Z0013_RB_G10_2_A08_B	32	24	LMG 17677	Lactobacillus	pentosus
Z0013_RB_G11_2_A07_A	32	24	LMG 17677	Lactobacillus	pentosus
Z0013_RB_H01_1_A12_A	32	24	LMG 17677	Lactobacillus	pentosus
Z0013_RB_H03_1_A11_A	32	24	LMG 17677	Lactobacillus	pentosus
Z0013_RB_H04_1_A11_B	32	24	LMG 17677	Lactobacillus	pentosus
Z0013_RB_H05_1_A10_A	32	24	LMG 17677	Lactobacillus	pentosus
Z0013_RB_H06_1_A10_B	32	24	LMG 17677	Lactobacillus	pentosus
Z0013_RO_G05_1_H09_A	32	24	LMG 17677	Lactobacillus	pentosus
Z0013_RO_G06_1_H09_B	32	24	LMG 17677	Lactobacillus	pentosus
Z0013_RO_H01_2_H07_A	32	24	LMG 17677	Lactobacillus	pentosus
Z0013_RO_H02_2_H07_B	32	24	LMG 17677	Lactobacillus	pentosus
Z0013_RO_H03_2_H08_A	32	24	LMG 17677	Lactobacillus	pentosus
Z0013_RO_H04_2_H08_B	32	24	LMG 17677	Lactobacillus	pentosus
Z0014_LB_G09_2_A02_A	32	24	LMG 18401	Lactobacillus	pentosus
Z0014_LB_G10_2_A02_B	32	24	LMG 18401	Lactobacillus	pentosus
Z0014_LB_G11_2_A01_A	32	24	LMG 18401	Lactobacillus	pentosus
Z0014_LB_G12_2_A01_B	32	24	LMG 18401	Lactobacillus	pentosus
Z0014_LB_H01_1_A06_A	32	24	LMG 18401	Lactobacillus	pentosus
Z0014_LB_H02_1_A06_B	32	24	LMG 18401	Lactobacillus	pentosus
Z0014_LB_H03_1_A05_A	32	24	LMG 18401	Lactobacillus	pentosus
Z0014_LB_H04_1_A05_B	32	24	LMG 18401	Lactobacillus	pentosus
Z0014_LB_H05_1_A04_A	32	24	LMG 18401	Lactobacillus	pentosus
Z0014_RB_G09_2_A08_A	32	24	LMG 18401	Lactobacillus	pentosus
Z0014_RB_G10_2_A08_B	32	24	LMG 18401	Lactobacillus	pentosus
Z0014_RB_G11_2_A07_A	32	24	LMG 18401	Lactobacillus	pentosus
Z0014_RB_G12_2_A07_B	32	24	LMG 18401	Lactobacillus	pentosus
Z0014_RB_H01_1_A12_A	32	24	LMG 18401	Lactobacillus	pentosus
Z0014_RB_H02_1_A12_B	32	24	LMG 18401	Lactobacillus	pentosus

Z0014_RB_H03_1_A11_A	32	24	LMG 18401	Lactobacillus	pentosus
Z0014_RB_H04_1_A11_B	32	24	LMG 18401	Lactobacillus	pentosus
Z0014_RB_H05_1_A10_A	32	24	LMG 18401	Lactobacillus	pentosus
Z0014_RB_H06_1_A10_B	32	24	LMG 18401	Lactobacillus	pentosus
Z0014_RB_H07_1_A09_A	32	24	LMG 18401	Lactobacillus	pentosus
Z0014_RB_H08_1_A09_B	32	24	LMG 18401	Lactobacillus	pentosus
Z0014_RB_H09_1_A08_A	32	24	LMG 18401	Lactobacillus	pentosus
Z0014_RB_H10_1_A08_B	32	24	LMG 18401	Lactobacillus	pentosus
Z0014_RB_H11_1_A07_A	32	24	LMG 18401	Lactobacillus	pentosus
Z0014_RB_H12_1_A07_B	32	24	LMG 18401	Lactobacillus	pentosus
Z0014_RO_A01_1_E07_A	32	24	LMG 18401	Lactobacillus	pentosus
Z0014_RO_A02_1_E07_B	32	24	LMG 18401	Lactobacillus	pentosus
Z0014_RO_A03_1_E08_A	32	24	LMG 18401	Lactobacillus	pentosus
Z0014_RO_A04_1_E08_B	32	24	LMG 18401	Lactobacillus	pentosus
Z0014_RO_A05_1_E09_A	32	24	LMG 18401	Lactobacillus	pentosus
Z0014_RO_A06_1_E09_B	32	24	LMG 18401	Lactobacillus	pentosus
Z0014_RO_A07_1_E10_A	32	24	LMG 18401	Lactobacillus	pentosus
Z0025_LO_E01_1_G01_A	32	140	LMG 9210	Lactobacillus	pentosus
Z0025_LO_E02_1_G01_B	32	140	LMG 9210	Lactobacillus	pentosus
Z0025_LO_E03_1_G02_A	32	140	LMG 9210	Lactobacillus	pentosus
Z0025_LO_E04_1_G02_B	32	140	LMG 9210	Lactobacillus	pentosus
Z0025_LO_E09_1_G05_A	32	140	LMG 9210	Lactobacillus	pentosus
Z0025_LO_E12_1_G06_B	32	140	LMG 9210	Lactobacillus	pentosus
Z0025_LO_F01_2_G01_A	32	140	LMG 9210	Lactobacillus	pentosus
Z0025_LO_F02_2_G01_B	32	140	LMG 9210	Lactobacillus	pentosus
Z0025_LO_F03_2_G02_A	32	140	LMG 9210	Lactobacillus	pentosus
Z0025_LO_F05_2_G03_A	32	140	LMG 9210	Lactobacillus	pentosus
Z0025_LO_F06_2_G03_B	32	140	LMG 9210	Lactobacillus	pentosus
Z0025_LO_F08_2_G04_B	32	140	LMG 9210	Lactobacillus	pentosus
Z0025_LO_F09_2_G05_A	32	140	LMG 9210	Lactobacillus	pentosus
Z0025_LO_F10_2_G05_B	32	140	LMG 9210	Lactobacillus	pentosus
Z0025_LO_F11_2_G06_A	32	140	LMG 9210	Lactobacillus	pentosus
Z0025_LO_G02_1_H01_B	32	140	LMG 9210	Lactobacillus	pentosus
Z0025_LO_G03_1_H02_A	32	140	LMG 9210	Lactobacillus	pentosus
Z0025_LO_G05_1_H03_A	32	140	LMG 9210	Lactobacillus	pentosus
Z0025_LO_G06_1_H03_B	32	140	LMG 9210	Lactobacillus	pentosus
Z0025_LO_G07_1_H04_A	32	140	LMG 9210	Lactobacillus	pentosus
Z0025_LO_G08_1_H04_B	32	140	LMG 9210	Lactobacillus	pentosus
Z0025_LO_G09_1_H05_A	32	140	LMG 9210	Lactobacillus	pentosus
Z0025_RO_D04_2_F08_B	32	140	LMG 9210	Lactobacillus	pentosus
Z0025_RO_D05_2_F09_A	32	140	LMG 9210	Lactobacillus	pentosus
Z0025_RO_D06_2_F09_B	32	140	LMG 9210	Lactobacillus	pentosus
Z0025_RO_D08_2_F10_B	32	140	LMG 9210	Lactobacillus	pentosus
Z0025_RO_D09_2_F11_A	32	140	LMG 9210	Lactobacillus	pentosus
Z0025_RO_E02_1_G07_B	32	140	LMG 9210	Lactobacillus	pentosus
Z0025_RO_E05_1_G09_A	32	140	LMG 9210	Lactobacillus	pentosus
Z0025_RO_E10_1_G11_B	32	140	LMG 9210	Lactobacillus	pentosus
Z0025_RO_E11_1_G12_A	32	140	LMG 9210	Lactobacillus	pentosus
Z0025_RO_F02_2_G07_B	32	140	LMG 9210	Lactobacillus	pentosus
Z0014_LB_A01_2_D06_A	33	32	LMG 19863	Maricaulis	parjimensis

Z0014_LO_C03_1_F02_A	33	32	LMG 19863	Maricaulis	parjimensis
Z0014_LO_C07_1_F04_A	33	32	LMG 19863	Maricaulis	parjimensis
Z0014_LO_C08_1_F04_B	33	32	LMG 19863	Maricaulis	parjimensis
Z0014_LO_C10_1_F05_B	33	32	LMG 19863	Maricaulis	parjimensis
Z0014_LO_C12_1_F06_B	33	32	LMG 19863	Maricaulis	parjimensis
Z0014_LO_D01_2_F01_A	33	32	LMG 19863	Maricaulis	parjimensis
Z0014_LO_D02_2_F01_B	33	32	LMG 19863	Maricaulis	parjimensis
Z0014_LO_D03_2_F02_A	33	32	LMG 19863	Maricaulis	parjimensis
Z0014_LO_D05_2_F03_A	33	32	LMG 19863	Maricaulis	parjimensis
Z0014_LO_D11_2_F06_A	33	32	LMG 19863	Maricaulis	parjimensis
Z0014_LO_E02_1_G01_B	33	32	LMG 19863	Maricaulis	parjimensis
Z0014_LO_E03_1_G02_A	33	32	LMG 19863	Maricaulis	parjimensis
Z0014_LO_E04_1_G02_B	33	32	LMG 19863	Maricaulis	parjimensis
Z0014_LO_E10_1_G05_B	33	32	LMG 19863	Maricaulis	parjimensis
Z0014_RO_D01_2_F07_A	33	32	LMG 19863	Maricaulis	parjimensis
Z0014_RO_D02_2_F07_B	33	32	LMG 19863	Maricaulis	parjimensis
Z0014_RO_D03_2_F08_A	33	32	LMG 19863	Maricaulis	parjimensis
Z0014_RO_D05_2_F09_A	33	32	LMG 19863	Maricaulis	parjimensis
Z0014_RO_D06_2_F09_B	33	32	LMG 19863	Maricaulis	parjimensis
Z0014_RO_D10_2_F11_B	33	32	LMG 19863	Maricaulis	parjimensis
Z0014_RO_D11_2_F12_A	33	32	LMG 19863	Maricaulis	parjimensis
Z0014_RO_D12_2_F12_B	33	32	LMG 19863	Maricaulis	parjimensis
Z0014_RO_E01_1_G07_A	33	32	LMG 19863	Maricaulis	parjimensis
Z0014_RO_E02_1_G07_B	33	32	LMG 19863	Maricaulis	parjimensis
Z0014_RO_E04_1_G08_B	33	32	LMG 19863	Maricaulis	parjimensis
Z0014_RO_E05_1_G09_A	33	32	LMG 19863	Maricaulis	parjimensis
Z0014_RO_E07_1_G10_A	33	32	LMG 19863	Maricaulis	parjimensis
Z0014_RO_E08_1_G10_B	33	32	LMG 19863	Maricaulis	parjimensis
Z0014_RO_E09_1_G11_A	33	32	LMG 19863	Maricaulis	parjimensis
Z0014_RO_E10_1_G11_B	33	32	LMG 19863	Maricaulis	parjimensis
Z0014_RO_F03_2_G08_A	33	32	LMG 19863	Maricaulis	parjimensis
Z0014_LB_A02_2_D06_B	34	4	LMG 18021	Lactobacillus	plantarum
Z0014_LB_A03_2_D05_A	34	4	LMG 18021	Lactobacillus	plantarum
Z0014_LB_A04_2_D05_B	34	4	LMG 18021	Lactobacillus	plantarum
Z0014_LB_A05_2_D04_A	34	4	LMG 18021	Lactobacillus	plantarum
Z0014_LB_A06_2_D04_B	34	4	LMG 18021	Lactobacillus	plantarum
Z0014_LB_A07_2_D03_A	34	4	LMG 18021	Lactobacillus	plantarum
Z0014_LB_A08_2_D03_B	34	4	LMG 18021	Lactobacillus	plantarum
Z0014_LB_A09_2_D02_A	34	4	LMG 18021	Lactobacillus	plantarum
Z0014_LB_A10_2_D02_B	34	4	LMG 18021	Lactobacillus	plantarum
Z0014_LB_A11_2_D01_A	34	4	LMG 18021	Lactobacillus	plantarum
Z0014_LB_A12_2_D01_B	34	4	LMG 18021	Lactobacillus	plantarum
Z0014_LB_B01_1_D06_A	34	4	LMG 18021	Lactobacillus	plantarum
Z0014_LB_B02_1_D06_B	34	4	LMG 18021	Lactobacillus	plantarum
Z0014_LB_H06_1_A04_B	34	4	LMG 18404	Lactobacillus	plantarum
Z0014_LB_H07_1_A03_A	34	4	LMG 18404	Lactobacillus	plantarum
Z0014_LB_H08_1_A03_B	34	4	LMG 18404	Lactobacillus	plantarum
Z0014_LB_H09_1_A02_A	34	4	LMG 18404	Lactobacillus	plantarum
Z0014_LB_H10_1_A02_B	34	4	LMG 18404	Lactobacillus	plantarum
Z0014_LB_H11_1_A01_A	34	4	LMG 18404	Lactobacillus	plantarum

Z0014_LB_H12_1_A01_B	34	4	LMG 18404	Lactobacillus	plantarum
Z0014_LO_A01_1_E01_A	34	4	LMG 18404	Lactobacillus	plantarum
Z0014_LO_A02_1_E01_B	34	4	LMG 18404	Lactobacillus	plantarum
Z0014_LO_A03_1_E02_A	34	4	LMG 18404	Lactobacillus	plantarum
Z0014_LO_A04_1_E02_B	34	4	LMG 18404	Lactobacillus	plantarum
Z0014_LO_A05_1_E03_A	34	4	LMG 18404	Lactobacillus	plantarum
Z0014_LO_A06_1_E03_B	34	4	LMG 18404	Lactobacillus	plantarum
Z0014_LO_A07_1_E04_A	34	4	LMG 18404	Lactobacillus	plantarum
Z0014_LO_A08_1_E04_B	34	4	LMG 18404	Lactobacillus	plantarum
Z0014_LO_H12_2_H06_B	34	4	LMG 18021	Lactobacillus	plantarum
Z0014_RB_A01_2_D12_A	34	4	LMG 18021	Lactobacillus	plantarum
Z0014_RB_A02_2_D12_B	34	4	LMG 18021	Lactobacillus	plantarum
Z0014_RB_A03_2_D11_A	34	4	LMG 18021	Lactobacillus	plantarum
Z0014_RB_A04_2_D11_B	34	4	LMG 18021	Lactobacillus	plantarum
Z0014_RB_A05_2_D10_A	34	4	LMG 18021	Lactobacillus	plantarum
Z0014_RB_A06_2_D10_B	34	4	LMG 18021	Lactobacillus	plantarum
Z0014_RB_A07_2_D09_A	34	4	LMG 18021	Lactobacillus	plantarum
Z0014_RB_A08_2_D09_B	34	4	LMG 18021	Lactobacillus	plantarum
Z0014_RB_A09_2_D08_A	34	4	LMG 18021	Lactobacillus	plantarum
Z0014_RB_A10_2_D08_B	34	4	LMG 18021	Lactobacillus	plantarum
Z0014_RB_A11_2_D07_A	34	4	LMG 18021	Lactobacillus	plantarum
Z0014_RB_A12_2_D07_B	34	4	LMG 18021	Lactobacillus	plantarum
Z0014_RB_B01_1_D12_A	34	4	LMG 18021	Lactobacillus	plantarum
Z0014_RB_B02_1_D12_B	34	4	LMG 18021	Lactobacillus	plantarum
Z0014_RB_B03_1_D11_A	34	4	LMG 18021	Lactobacillus	plantarum
Z0014_RB_B04_1_D11_B	34	4	LMG 18021	Lactobacillus	plantarum
Z0014_RB_B05_1_D10_A	34	4	LMG 18021	Lactobacillus	plantarum
Z0014_RB_B06_1_D10_B	34	4	LMG 18021	Lactobacillus	plantarum
Z0014_RO_A08_1_E10_B	34	4	LMG 18404	Lactobacillus	plantarum
Z0014_RO_A09_1_E11_A	34	4	LMG 18404	Lactobacillus	plantarum
Z0014_RO_A10_1_E11_B	34	4	LMG 18404	Lactobacillus	plantarum
Z0014_RO_A11_1_E12_A	34	4	LMG 18404	Lactobacillus	plantarum
Z0014_RO_A12_1_E12_B	34	4	LMG 18404	Lactobacillus	plantarum
Z0014_RO_B01_2_E07_A	34	4	LMG 18404	Lactobacillus	plantarum
Z0014_RO_B02_2_E07_B	34	4	LMG 18404	Lactobacillus	plantarum
Z0014_RO_B03_2_E08_A	34	4	LMG 18404	Lactobacillus	plantarum
Z0014_RO_B04_2_E08_B	34	4	LMG 18404	Lactobacillus	plantarum
Z0014_RO_B05_2_E09_A	34	4	LMG 18404	Lactobacillus	plantarum
Z0014_RO_B06_2_E09_B	34	4	LMG 18404	Lactobacillus	plantarum
Z0014_RO_B07_2_E10_A	34	4	LMG 18404	Lactobacillus	plantarum
Z0014_RO_B08_2_E10_B	34	4	LMG 18404	Lactobacillus	plantarum
Z0014_LB_B03_1_D05_A	35	25	LMG 18212	Chryseobacterium	joostei
Z0014_LB_B04_1_D05_B	35	25	LMG 18212	Chryseobacterium	joostei
Z0014_LB_B05_1_D04_A	35	25	LMG 18212	Chryseobacterium	joostei
Z0014_LB_B06_1_D04_B	35	25	LMG 18212	Chryseobacterium	joostei
Z0014_LB_B07_1_D03_A	35	25	LMG 18212	Chryseobacterium	joostei
Z0014_LB_B08_1_D03_B	35	25	LMG 18212	Chryseobacterium	joostei
Z0014_LB_B09_1_D02_A	35	25	LMG 18212	Chryseobacterium	joostei
Z0014_LB_B10_1_D02_B	35	25	LMG 18212	Chryseobacterium	joostei
Z0014_LB_B11_1_D01_A	35	25	LMG 18212	Chryseobacterium	joostei

Z0014_LB_B12_1_D01_B	35	25	LMG 18212	Chryseobacterium	joostei
Z0014_LB_C01_2_C06_A	35	25	LMG 18212	Chryseobacterium	joostei
Z0014_LB_C02_2_C06_B	35	25	LMG 18212	Chryseobacterium	joostei
Z0014_LB_C03_2_C05_A	35	25	LMG 18212	Chryseobacterium	joostei
Z0014_LB_C04_2_C05_B	35	25	LMG 18212	Chryseobacterium	joostei
Z0014_LB_C05_2_C04_A	35	25	LMG 18212	Chryseobacterium	joostei
Z0014_LB_C06_2_C04_B	35	25	LMG 18212	Chryseobacterium	joostei
Z0014_LB_C07_2_C03_A	35	25	LMG 18212	Chryseobacterium	joostei
Z0014_LB_C08_2_C03_B	35	25	LMG 18212	Chryseobacterium	joostei
Z0014_LB_C09_2_C02_A	35	25	LMG 18212	Chryseobacterium	joostei
Z0014_RB_B07_1_D09_A	35	25	LMG 18212	Chryseobacterium	joostei
Z0014_RB_B08_1_D09_B	35	25	LMG 18212	Chryseobacterium	joostei
Z0014_RB_B09_1_D08_A	35	25	LMG 18212	Chryseobacterium	joostei
Z0014_RB_B10_1_D08_B	35	25	LMG 18212	Chryseobacterium	joostei
Z0014_RB_B11_1_D07_A	35	25	LMG 18212	Chryseobacterium	joostei
Z0014_RB_B12_1_D07_B	35	25	LMG 18212	Chryseobacterium	joostei
Z0014_RB_C01_2_C12_A	35	25	LMG 18212	Chryseobacterium	joostei
Z0014_RB_C02_2_C12_B	35	25	LMG 18212	Chryseobacterium	joostei
Z0014_RB_C03_2_C11_A	35	25	LMG 18212	Chryseobacterium	joostei
Z0014_RB_C04_2_C11_B	35	25	LMG 18212	Chryseobacterium	joostei
Z0014_RB_C05_2_C10_A	35	25	LMG 18212	Chryseobacterium	joostei
Z0014_RB_C06_2_C10_B	35	25	LMG 18212	Chryseobacterium	joostei
Z0014_RB_C07_2_C09_A	35	25	LMG 18212	Chryseobacterium	joostei
Z0014_LB_C10_2_C02_B	36	26	LMG 18294	Helicobacter	fennelliae
Z0014_LB_C11_2_C01_A	36	26	LMG 18294	Helicobacter	fennelliae
Z0014_LB_C12_2_C01_B	36	26	LMG 18294	Helicobacter	fennelliae
Z0014_LB_D01_1_C06_A	36	26	LMG 18294	Helicobacter	fennelliae
Z0014_LB_D02_1_C06_B	36	26	LMG 18294	Helicobacter	fennelliae
Z0014_LB_D03_1_C05_A	36	26	LMG 18294	Helicobacter	fennelliae
Z0014_LB_D04_1_C05_B	36	26	LMG 18294	Helicobacter	fennelliae
Z0014_LB_D05_1_C04_A	36	26	LMG 18294	Helicobacter	fennelliae
Z0014_LB_D06_1_C04_B	36	26	LMG 18294	Helicobacter	fennelliae
Z0014_LB_D07_1_C03_A	36	26	LMG 18294	Helicobacter	fennelliae
Z0014_LB_D08_1_C03_B	36	26	LMG 18294	Helicobacter	fennelliae
Z0014_LB_D09_1_C02_A	36	26	LMG 18294	Helicobacter	fennelliae
Z0014_RB_C08_2_C09_B	36	26	LMG 18294	Helicobacter	fennelliae
Z0014_RB_C09_2_C08_A	36	26	LMG 18294	Helicobacter	fennelliae
Z0014_RB_C10_2_C08_B	36	26	LMG 18294	Helicobacter	fennelliae
Z0014_RB_C11_2_C07_A	36	26	LMG 18294	Helicobacter	fennelliae
Z0014_RB_C12_2_C07_B	36	26	LMG 18294	Helicobacter	fennelliae
Z0014_RB_D01_1_C12_A	36	26	LMG 18294	Helicobacter	fennelliae
Z0014_RB_D02_1_C12_B	36	26	LMG 18294	Helicobacter	fennelliae
Z0014_RB_D03_1_C11_A	36	26	LMG 18294	Helicobacter	fennelliae
Z0014_RB_D04_1_C11_B	36	26	LMG 18294	Helicobacter	fennelliae
Z0014_RB_D05_1_C10_A	36	26	LMG 18294	Helicobacter	fennelliae
Z0014_RB_D06_1_C10_B	36	26	LMG 18294	Helicobacter	fennelliae
Z0014_RB_D07_1_C09_A	36	26	LMG 18294	Helicobacter	fennelliae
Z0014_RB_D08_1_C09_B	36	26	LMG 18294	Helicobacter	fennelliae
Z0014_RB_D09_1_C08_A	36	26	LMG 18294	Helicobacter	fennelliae
Z0014_RB_D10_1_C08_B	36	26	LMG 18294	Helicobacter	fennelliae

Z0014_RB_D11_1_C07_A	36	26	LMG 18294	Helicobacter	fennelliae
Z0014_RB_D12_1_C07_B	36	26	LMG 18294	Helicobacter	fennelliae
Z0014_RB_E01_2_B12_A	36	26	LMG 18294	Helicobacter	fennelliae
Z0014_RB_E02_2_B12_B	36	26	LMG 18294	Helicobacter	fennelliae
Z0014_RB_E03_2_B11_A	36	26	LMG 18294	Helicobacter	fennelliae
Z0014_LB_D10_1_C02_B	37	27	LMG 18397	Anoxybacillus	flavithermus
Z0014_LB_D11_1_C01_A	37	27	LMG 18397	Anoxybacillus	flavithermus
Z0014_LB_D12_1_C01_B	37	27	LMG 18397	Anoxybacillus	flavithermus
Z0014_LB_E01_2_B06_A	37	27	LMG 18397	Anoxybacillus	flavithermus
Z0014_LB_E02_2_B06_B	37	27	LMG 18397	Anoxybacillus	flavithermus
Z0014_LB_E03_2_B05_A	37	27	LMG 18397	Anoxybacillus	flavithermus
Z0014_LB_E04_2_B05_B	37	27	LMG 18397	Anoxybacillus	flavithermus
Z0014_LB_E05_2_B04_A	37	27	LMG 18397	Anoxybacillus	flavithermus
Z0014_LB_E06_2_B04_B	37	27	LMG 18397	Anoxybacillus	flavithermus
Z0014_LB_E07_2_B03_A	37	27	LMG 18397	Anoxybacillus	flavithermus
Z0014_LB_E08_2_B03_B	37	27	LMG 18397	Anoxybacillus	flavithermus
Z0014_LB_E09_2_B02_A	37	27	LMG 18397	Anoxybacillus	flavithermus
Z0014_LB_E10_2_B02_B	37	27	LMG 18397	Anoxybacillus	flavithermus
Z0014_LB_E11_2_B01_A	37	27	LMG 18397	Anoxybacillus	flavithermus
Z0014_LB_E12_2_B01_B	37	27	LMG 18397	Anoxybacillus	flavithermus
Z0014_LB_F01_1_B06_A	37	27	LMG 18397	Anoxybacillus	flavithermus
Z0014_LB_F02_1_B06_B	37	27	LMG 18397	Anoxybacillus	flavithermus
Z0014_LB_F03_1_B05_A	37	27	LMG 18397	Anoxybacillus	flavithermus
Z0014_RB_E04_2_B11_B	37	27	LMG 18397	Anoxybacillus	flavithermus
Z0014_RB_E05_2_B10_A	37	27	LMG 18397	Anoxybacillus	flavithermus
Z0014_RB_E06_2_B10_B	37	27	LMG 18397	Anoxybacillus	flavithermus
Z0014_RB_E07_2_B09_A	37	27	LMG 18397	Anoxybacillus	flavithermus
Z0014_RB_E08_2_B09_B	37	27	LMG 18397	Anoxybacillus	flavithermus
Z0014_RB_E09_2_B08_A	37	27	LMG 18397	Anoxybacillus	flavithermus
Z0014_RB_E10_2_B08_B	37	27	LMG 18397	Anoxybacillus	flavithermus
Z0014_RB_E11_2_B07_A	37	27	LMG 18397	Anoxybacillus	flavithermus
Z0014_RB_E12_2_B07_B	37	27	LMG 18397	Anoxybacillus	flavithermus
Z0014_RB_F01_1_B12_A	37	27	LMG 18397	Anoxybacillus	flavithermus
Z0014_RB_F02_1_B12_B	37	27	LMG 18397	Anoxybacillus	flavithermus
Z0014_RB_F03_1_B11_A	37	27	LMG 18397	Anoxybacillus	flavithermus
Z0014_RB_F04_1_B11_B	37	27	LMG 18397	Anoxybacillus	flavithermus
Z0014_RB_F05_1_B10_A	37	27	LMG 18397	Anoxybacillus	flavithermus
Z0014_LB_F04_1_B05_B	38	22	LMG 18398	Lactobacillus	paraplantarum
Z0014_LB_F05_1_B04_A	38	22	LMG 18398	Lactobacillus	paraplantarum
Z0014_LB_F06_1_B04_B	38	22	LMG 18398	Lactobacillus	paraplantarum
Z0014_LB_F07_1_B03_A	38	22	LMG 18398	Lactobacillus	paraplantarum
Z0014_LB_F08_1_B03_B	38	22	LMG 18398	Lactobacillus	paraplantarum
Z0014_LB_F09_1_B02_A	38	22	LMG 18398	Lactobacillus	paraplantarum
Z0014_LB_F10_1_B02_B	38	22	LMG 18398	Lactobacillus	paraplantarum
Z0014_LB_F11_1_B01_A	38	22	LMG 18398	Lactobacillus	paraplantarum
Z0014_LB_F12_1_B01_B	38	22	LMG 18398	Lactobacillus	paraplantarum
Z0014_LB_G01_2_A06_A	38	22	LMG 18398	Lactobacillus	paraplantarum
Z0014_LB_G02_2_A06_B	38	22	LMG 18398	Lactobacillus	paraplantarum
Z0014_LB_G03_2_A05_A	38	22	LMG 18398	Lactobacillus	paraplantarum
Z0014_LB_G04_2_A05_B	38	22	LMG 18398	Lactobacillus	paraplantarum

Z0014_LB_G05_2_A04_A	38	22	LMG 18398	Lactobacillus	paraplantarum
Z0014_LB_G06_2_A04_B	38	22	LMG 18398	Lactobacillus	paraplantarum
Z0014_LB_G07_2_A03_A	38	22	LMG 18398	Lactobacillus	paraplantarum
Z0014_LB_G08_2_A03_B	38	22	LMG 18398	Lactobacillus	paraplantarum
Z0014_RB_F06_1_B10_B	38	22	LMG 18398	Lactobacillus	paraplantarum
Z0014_RB_F07_1_B09_A	38	22	LMG 18398	Lactobacillus	paraplantarum
Z0014_RB_F08_1_B09_B	38	22	LMG 18398	Lactobacillus	paraplantarum
Z0014_RB_F09_1_B08_A	38	22	LMG 18398	Lactobacillus	paraplantarum
Z0014_RB_F10_1_B08_B	38	22	LMG 18398	Lactobacillus	paraplantarum
Z0014_RB_F11_1_B07_A	38	22	LMG 18398	Lactobacillus	paraplantarum
Z0014_RB_F12_1_B07_B	38	22	LMG 18398	Lactobacillus	paraplantarum
Z0014_RB_G01_2_A12_A	38	22	LMG 18398	Lactobacillus	paraplantarum
Z0014_RB_G02_2_A12_B	38	22	LMG 18398	Lactobacillus	paraplantarum
Z0014_RB_G03_2_A11_A	38	22	LMG 18398	Lactobacillus	paraplantarum
Z0014_RB_G04_2_A11_B	38	22	LMG 18398	Lactobacillus	paraplantarum
Z0014_RB_G05_2_A10_A	38	22	LMG 18398	Lactobacillus	paraplantarum
Z0014_RB_G06_2_A10_B	38	22	LMG 18398	Lactobacillus	paraplantarum
Z0014_RB_G07_2_A09_A	38	22	LMG 18398	Lactobacillus	paraplantarum
Z0014_RB_G08_2_A09_B	38	22	LMG 18398	Lactobacillus	paraplantarum
Z0014_LO_A09_1_E05_A	39	28	LMG 18919	Microvirgula	aerodenitrificans
Z0014_LO_A10_1_E05_B	39	28	LMG 18919	Microvirgula	aerodenitrificans
Z0014_LO_A11_1_E06_A	39	28	LMG 18919	Microvirgula	aerodenitrificans
Z0014_LO_A12_1_E06_B	39	28	LMG 18919	Microvirgula	aerodenitrificans
Z0014_LO_B01_2_E01_A	39	28	LMG 18919	Microvirgula	aerodenitrificans
Z0014_LO_B02_2_E01_B	39	28	LMG 18919	Microvirgula	aerodenitrificans
Z0014_LO_B03_2_E02_A	39	28	LMG 18919	Microvirgula	aerodenitrificans
Z0014_LO_B04_2_E02_B	39	28	LMG 18919	Microvirgula	aerodenitrificans
Z0014_LO_B05_2_E03_A	39	28	LMG 18919	Microvirgula	aerodenitrificans
Z0014_LO_B06_2_E03_B	39	28	LMG 18919	Microvirgula	aerodenitrificans
Z0014_LO_B07_2_E04_A	39	28	LMG 18919	Microvirgula	aerodenitrificans
Z0014_LO_B08_2_E04_B	39	28	LMG 18919	Microvirgula	aerodenitrificans
Z0014_LO_B09_2_E05_A	39	28	LMG 18919	Microvirgula	aerodenitrificans
Z0014_LO_B10_2_E05_B	39	28	LMG 18919	Microvirgula	aerodenitrificans
Z0014_LO_B11_2_E06_A	39	28	LMG 18919	Microvirgula	aerodenitrificans
Z0014_LO_B12_2_E06_B	39	28	LMG 18919	Microvirgula	aerodenitrificans
Z0014_LO_C01_1_F01_A	39	28	LMG 18919	Microvirgula	aerodenitrificans
Z0014_LO_C02_1_F01_B	39	28	LMG 18919	Microvirgula	aerodenitrificans
Z0014_LO_C04_1_F02_B	39	28	LMG 18919	Microvirgula	aerodenitrificans
Z0014_LO_C05_1_F03_A	39	28	LMG 18919	Microvirgula	aerodenitrificans
Z0014_RO_B09_2_E11_A	39	28	LMG 18919	Microvirgula	aerodenitrificans
Z0014_RO_B10_2_E11_B	39	28	LMG 18919	Microvirgula	aerodenitrificans
Z0014_RO_B11_2_E12_A	39	28	LMG 18919	Microvirgula	aerodenitrificans
Z0014_RO_B12_2_E12_B	39	28	LMG 18919	Microvirgula	aerodenitrificans
Z0014_RO_C01_1_F07_A	39	28	LMG 18919	Microvirgula	aerodenitrificans
Z0014_RO_C02_1_F07_B	39	28	LMG 18919	Microvirgula	aerodenitrificans
Z0014_RO_C03_1_F08_A	39	28	LMG 18919	Microvirgula	aerodenitrificans
Z0014_RO_C04_1_F08_B	39	28	LMG 18919	Microvirgula	aerodenitrificans
Z0014_LO_C06_1_F03_B	40	29	LMG 19264	Corynebacterium	casei
Z0014_LO_C09_1_F05_A	40	29	LMG 19264	Corynebacterium	casei
Z0014_LO_C11_1_F06_A	40	29	LMG 19264	Corynebacterium	casei

Z0014_LO_D04_2_F02_B	40	29	LMG 19264	Corynebacterium	casei
Z0014_LO_D06_2_F03_B	40	29	LMG 19264	Corynebacterium	casei
Z0014_LO_D07_2_F04_A	40	29	LMG 19264	Corynebacterium	casei
Z0014_LO_D08_2_F04_B	40	29	LMG 19264	Corynebacterium	casei
Z0014_LO_D09_2_F05_A	40	29	LMG 19264	Corynebacterium	casei
Z0014_LO_D10_2_F05_B	40	29	LMG 19264	Corynebacterium	casei
Z0014_LO_D12_2_F06_B	40	29	LMG 19264	Corynebacterium	casei
Z0014_LO_E01_1_G01_A	40	29	LMG 19264	Corynebacterium	casei
Z0014_LO_E05_1_G03_A	40	29	LMG 19264	Corynebacterium	casei
Z0014_LO_E06_1_G03_B	40	29	LMG 19264	Corynebacterium	casei
Z0014_LO_E07_1_G04_A	40	29	LMG 19264	Corynebacterium	casei
Z0014_LO_E08_1_G04_B	40	29	LMG 19264	Corynebacterium	casei
Z0014_LO_E09_1_G05_A	40	29	LMG 19264	Corynebacterium	casei
Z0014_LO_E11_1_G06_A	40	29	LMG 19264	Corynebacterium	casei
Z0014_LO_E12_1_G06_B	40	29	LMG 19264	Corynebacterium	casei
Z0014_RO_C05_1_F09_A	40	29	LMG 19264	Corynebacterium	casei
Z0014_RO_C06_1_F09_B	40	29	LMG 19264	Corynebacterium	casei
Z0014_RO_C07_1_F10_A	40	29	LMG 19264	Corynebacterium	casei
Z0014_RO_C08_1_F10_B	40	29	LMG 19264	Corynebacterium	casei
Z0014_RO_C09_1_F11_A	40	29	LMG 19264	Corynebacterium	casei
Z0014_RO_C10_1_F11_B	40	29	LMG 19264	Corynebacterium	casei
Z0014_RO_C11_1_F12_A	40	29	LMG 19264	Corynebacterium	casei
Z0014_RO_C12_1_F12_B	40	29	LMG 19264	Corynebacterium	casei
Z0014_RO_D04_2_F08_B	40	29	LMG 19264	Corynebacterium	casei
Z0014_RO_D07_2_F10_A	40	29	LMG 19264	Corynebacterium	casei
Z0014_RO_D08_2_F10_B	40	29	LMG 19264	Corynebacterium	casei
Z0014_RO_D09_2_F11_A	40	29	LMG 19264	Corynebacterium	casei
Z0014_RO_E03_1_G08_A	40	29	LMG 19264	Corynebacterium	casei
Z0014_RO_E06_1_G09_B	40	29	LMG 19264	Corynebacterium	casei
Z0014_LO_F01_2_G01_A	41	31	LMG 19667	Lactobacillus	diolivorans
Z0014_LO_F06_2_G03_B	41	31	LMG 19667	Lactobacillus	diolivorans
Z0014_LO_F07_2_G04_A	41	31	LMG 19667	Lactobacillus	diolivorans
Z0014_LO_F11_2_G06_A	41	31	LMG 19667	Lactobacillus	diolivorans
Z0014_LO_G04_1_H02_B	41	31	LMG 19667	Lactobacillus	diolivorans
Z0014_LO_H01_2_H01_A	41	31	LMG 19667	Lactobacillus	diolivorans
Z0014_LO_H02_2_H01_B	41	31	LMG 19667	Lactobacillus	diolivorans
Z0014_LO_H05_2_H03_A	41	31	LMG 19667	Lactobacillus	diolivorans
Z0014_LO_H06_2_H03_B	41	31	LMG 19667	Lactobacillus	diolivorans
Z0014_LO_H07_2_H04_A	41	31	LMG 19667	Lactobacillus	diolivorans
Z0014_LO_H09_2_H05_A	41	31	LMG 19667	Lactobacillus	diolivorans
Z0014_LO_H10_2_H05_B	41	31	LMG 19667	Lactobacillus	diolivorans
Z0014_LO_H11_2_H06_A	41	31	LMG 19667	Lactobacillus	diolivorans
Z0014_RO_F01_2_G07_A	41	31	LMG 19667	Lactobacillus	diolivorans
Z0014_RO_F02_2_G07_B	41	31	LMG 19667	Lactobacillus	diolivorans
Z0014_RO_F05_2_G09_A	41	31	LMG 19667	Lactobacillus	diolivorans
Z0014_RO_F06_2_G09_B	41	31	LMG 19667	Lactobacillus	diolivorans
Z0014_RO_F07_2_G10_A	41	31	LMG 19667	Lactobacillus	diolivorans
Z0014_RO_F08_2_G10_B	41	31	LMG 19667	Lactobacillus	diolivorans
Z0014_RO_F10_2_G11_B	41	31	LMG 19667	Lactobacillus	diolivorans
Z0014_RO_F11_2_G12_A	41	31	LMG 19667	Lactobacillus	diolivorans

Z0014_RO_F12_2_G12_B	41	31	LMG 19667	Lactobacillus	diolivorans	
Z0014_RO_G01_1_H07_A	41	31	LMG 19667	Lactobacillus	diolivorans	
Z0014_RO_G02_1_H07_B	41	31	LMG 19667	Lactobacillus	diolivorans	
Z0014_RO_G03_1_H08_A	41	31	LMG 19667	Lactobacillus	diolivorans	
Z0014_RO_G04_1_H08_B	41	31	LMG 19667	Lactobacillus	diolivorans	
Z0014_RO_G05_1_H09_A	41	31	LMG 19667	Lactobacillus	diolivorans	
Z0014_RO_G08_1_H10_B	41	31	LMG 19667	Lactobacillus	diolivorans	
Z0014_RO_G09_1_H11_A	41	31	LMG 19667	Lactobacillus	diolivorans	
Z0014_RO_H01_2_H07_A	41	31	LMG 19667	Lactobacillus	diolivorans	
Z0014_RO_H03_2_H08_A	41	31	LMG 19667	Lactobacillus	diolivorans	
Z0014_RO_H04_2_H08_B	41	31	LMG 19667	Lactobacillus	diolivorans	
Z0014_LO_F02_2_G01_B	42	30	LMG 19484	Sphingomonas	melonis	
Z0014_LO_F03_2_G02_A	42	30	LMG 19484	Sphingomonas	melonis	
Z0014_LO_F04_2_G02_B	42	30	LMG 19484	Sphingomonas	melonis	
Z0014_LO_F05_2_G03_A	42	30	LMG 19484	Sphingomonas	melonis	
Z0014_LO_F08_2_G04_B	42	30	LMG 19484	Sphingomonas	melonis	
Z0014_LO_F09_2_G05_A	42	30	LMG 19484	Sphingomonas	melonis	
Z0014_LO_F10_2_G05_B	42	30	LMG 19484	Sphingomonas	melonis	
Z0014_LO_F12_2_G06_B	42	30	LMG 19484	Sphingomonas	melonis	
Z0014_LO_G01_1_H01_A	42	30	LMG 19484	Sphingomonas	melonis	
Z0014_LO_G02_1_H01_B	42	30	LMG 19484	Sphingomonas	melonis	
Z0014_LO_G03_1_H02_A	42	30	LMG 19484	Sphingomonas	melonis	
Z0014_LO_G05_1_H03_A	42	30	LMG 19484	Sphingomonas	melonis	
Z0014_LO_G06_1_H03_B	42	30	LMG 19484	Sphingomonas	melonis	
Z0014_LO_G07_1_H04_A	42	30	LMG 19484	Sphingomonas	melonis	
Z0014_LO_G08_1_H04_B	42	30	LMG 19484	Sphingomonas	melonis	
Z0014_LO_G09_1_H05_A	42	30	LMG 19484	Sphingomonas	melonis	
Z0014_LO_G10_1_H05_B	42	30	LMG 19484	Sphingomonas	melonis	
Z0014_LO_G11_1_H06_A	42	30	LMG 19484	Sphingomonas	melonis	
Z0014_LO_G12_1_H06_B	42	30	LMG 19484	Sphingomonas	melonis	
Z0014_LO_H03_2_H02_A	42	30	LMG 19484	Sphingomonas	melonis	
Z0014_LO_H04_2_H02_B	42	30	LMG 19484	Sphingomonas	melonis	
Z0014_LO_H08_2_H04_B	42	30	LMG 19484	Sphingomonas	melonis	
Z0014_RO_E11_1_G12_A	42	30	LMG 19484	Sphingomonas	melonis	
Z0014_RO_E12_1_G12_B	42	30	LMG 19484	Sphingomonas	melonis	
Z0014_RO_F04_2_G08_B	42	30	LMG 19484	Sphingomonas	melonis	
Z0014_RO_F09_2_G11_A	42	30	LMG 19484	Sphingomonas	melonis	
Z0014_RO_G06_1_H09_B	42	30	LMG 19484	Sphingomonas	melonis	
Z0014_RO_G07_1_H10_A	42	30	LMG 19484	Sphingomonas	melonis	
Z0014_RO_G10_1_H11_B	42	30	LMG 19484	Sphingomonas	melonis	
Z0014_RO_G11_1_H12_A	42	30	LMG 19484	Sphingomonas	melonis	
Z0014_RO_G12_1_H12_B	42	30	LMG 19484	Sphingomonas	melonis	
Z0014_RO_H02_2_H07_B	42	30	LMG 19484	Sphingomonas	melonis	
Z0015_LB_A01_2_D06_A	43	33	LMG 2095	Klebsiella	pneumoniae	pneumoniae
Z0015_LB_A02_2_D06_B	43	33	LMG 2095	Klebsiella	pneumoniae	pneumoniae
Z0015_LB_A03_2_D05_A	43	33	LMG 2095	Klebsiella	pneumoniae	pneumoniae
Z0015_LB_A04_2_D05_B	43	33	LMG 2095	Klebsiella	pneumoniae	pneumoniae
Z0015_LB_A05_2_D04_A	43	33	LMG 2095	Klebsiella	pneumoniae	pneumoniae
Z0015_LB_A06_2_D04_B	43	33	LMG 2095	Klebsiella	pneumoniae	pneumoniae
Z0015_LB_A07_2_D03_A	43	33	LMG 2095	Klebsiella	pneumoniae	pneumoniae

Z0015_LB_A08_2_D03_B	43	33	LMG 2095	Klebsiella	pneumoniae	pneumoniae
Z0015_LB_A09_2_D02_A	43	33	LMG 2095	Klebsiella	pneumoniae	pneumoniae
Z0015_LB_A10_2_D02_B	43	33	LMG 2095	Klebsiella	pneumoniae	pneumoniae
Z0015_LB_A11_2_D01_A	43	33	LMG 2095	Klebsiella	pneumoniae	pneumoniae
Z0015_LB_A12_2_D01_B	43	33	LMG 2095	Klebsiella	pneumoniae	pneumoniae
Z0015_LB_B01_1_D06_A	43	33	LMG 2095	Klebsiella	pneumoniae	pneumoniae
Z0015_LB_B02_1_D06_B	43	33	LMG 2095	Klebsiella	pneumoniae	pneumoniae
Z0015_LB_B03_1_D05_A	43	33	LMG 2095	Klebsiella	pneumoniae	pneumoniae
Z0015_LB_B04_1_D05_B	43	33	LMG 2095	Klebsiella	pneumoniae	pneumoniae
Z0015_LB_B05_1_D04_A	43	33	LMG 2095	Klebsiella	pneumoniae	pneumoniae
Z0015_LB_B06_1_D04_B	43	33	LMG 2095	Klebsiella	pneumoniae	pneumoniae
Z0015_LB_B07_1_D03_A	43	33	LMG 2095	Klebsiella	pneumoniae	pneumoniae
Z0015_LO_G11_1_H06_A	43	33	LMG 2095	Klebsiella	pneumoniae	pneumoniae
Z0015_LO_G12_1_H06_B	43	33	LMG 2095	Klebsiella	pneumoniae	pneumoniae
Z0015_LO_H01_2_H01_A	43	33	LMG 2095	Klebsiella	pneumoniae	pneumoniae
Z0015_LO_H02_2_H01_B	43	33	LMG 2095	Klebsiella	pneumoniae	pneumoniae
Z0015_LO_H03_2_H02_A	43	33	LMG 2095	Klebsiella	pneumoniae	pneumoniae
Z0015_LO_H04_2_H02_B	43	33	LMG 2095	Klebsiella	pneumoniae	pneumoniae
Z0015_LO_H05_2_H03_A	43	33	LMG 2095	Klebsiella	pneumoniae	pneumoniae
Z0015_LO_H06_2_H03_B	43	33	LMG 2095	Klebsiella	pneumoniae	pneumoniae
Z0015_LO_H07_2_H04_A	43	33	LMG 2095	Klebsiella	pneumoniae	pneumoniae
Z0015_LO_H08_2_H04_B	43	33	LMG 2095	Klebsiella	pneumoniae	pneumoniae
Z0015_LO_H09_2_H05_A	43	33	LMG 2095	Klebsiella	pneumoniae	pneumoniae
Z0015_LO_H10_2_H05_B	43	33	LMG 2095	Klebsiella	pneumoniae	pneumoniae
Z0015_LO_H11_2_H06_A	43	33	LMG 2095	Klebsiella	pneumoniae	pneumoniae
Z0015_LB_B08_1_D03_B	44	34	LMG 21276	Psychrobacter	luti	
Z0015_LB_B09_1_D02_A	44	34	LMG 21276	Psychrobacter	luti	
Z0015_LB_B10_1_D02_B	44	34	LMG 21276	Psychrobacter	luti	
Z0015_LB_B11_1_D01_A	44	34	LMG 21276	Psychrobacter	luti	
Z0015_LB_B12_1_D01_B	44	34	LMG 21276	Psychrobacter	luti	
Z0015_LB_C01_2_C06_A	44	34	LMG 21276	Psychrobacter	luti	
Z0015_LB_C02_2_C06_B	44	34	LMG 21276	Psychrobacter	luti	
Z0015_LB_C03_2_C05_A	44	34	LMG 21276	Psychrobacter	luti	
Z0015_LB_C04_2_C05_B	44	34	LMG 21276	Psychrobacter	luti	
Z0015_LB_C05_2_C04_A	44	34	LMG 21276	Psychrobacter	luti	
Z0015_LB_C06_2_C04_B	44	34	LMG 21276	Psychrobacter	luti	
Z0015_LB_C07_2_C03_A	44	34	LMG 21276	Psychrobacter	luti	
Z0015_LB_C08_2_C03_B	44	34	LMG 21276	Psychrobacter	luti	
Z0015_LB_C09_2_C02_A	44	34	LMG 21276	Psychrobacter	luti	
Z0015_LB_C10_2_C02_B	44	34	LMG 21276	Psychrobacter	luti	
Z0015_LO_H12_2_H06_B	44	34	LMG 21276	Psychrobacter	luti	
Z0015_RB_A01_2_D12_A	44	34	LMG 21276	Psychrobacter	luti	
Z0015_RB_A02_2_D12_B	44	34	LMG 21276	Psychrobacter	luti	
Z0015_RB_A03_2_D11_A	44	34	LMG 21276	Psychrobacter	luti	
Z0015_RB_A04_2_D11_B	44	34	LMG 21276	Psychrobacter	luti	
Z0015_RB_A05_2_D10_A	44	34	LMG 21276	Psychrobacter	luti	
Z0015_RB_A06_2_D10_B	44	34	LMG 21276	Psychrobacter	luti	
Z0015_RB_A07_2_D09_A	44	34	LMG 21276	Psychrobacter	luti	
Z0015_RB_A08_2_D09_B	44	34	LMG 21276	Psychrobacter	luti	
Z0015_RB_A09_2_D08_A	44	34	LMG 21276	Psychrobacter	luti	

Z0015_RB_A10_2_D08_B	44	34	LMG 21276	Psychrobacter	luti
Z0015_RB_A11_2_D07_A	44	34	LMG 21276	Psychrobacter	luti
Z0015_RB_A12_2_D07_B	44	34	LMG 21276	Psychrobacter	luti
Z0015_RB_B01_1_D12_A	44	34	LMG 21276	Psychrobacter	luti
Z0015_RB_B02_1_D12_B	44	34	LMG 21276	Psychrobacter	luti
Z0015_RB_B03_1_D11_A	44	34	LMG 21276	Psychrobacter	luti
Z0015_RB_B04_1_D11_B	44	34	LMG 21276	Psychrobacter	luti
Z0015_LB_C11_2_C01_A	45	35	LMG 21292	Ketogulonicigenium	robustum
Z0015_LB_C12_2_C01_B	45	35	LMG 21292	Ketogulonicigenium	robustum
Z0015_LB_D01_1_C06_A	45	35	LMG 21292	Ketogulonicigenium	robustum
Z0015_LB_D02_1_C06_B	45	35	LMG 21292	Ketogulonicigenium	robustum
Z0015_LB_D03_1_C05_A	45	35	LMG 21292	Ketogulonicigenium	robustum
Z0015_LB_D04_1_C05_B	45	35	LMG 21292	Ketogulonicigenium	robustum
Z0015_LB_D05_1_C04_A	45	35	LMG 21292	Ketogulonicigenium	robustum
Z0015_LB_D06_1_C04_B	45	35	LMG 21292	Ketogulonicigenium	robustum
Z0015_LB_D07_1_C03_A	45	35	LMG 21292	Ketogulonicigenium	robustum
Z0015_LB_D08_1_C03_B	45	35	LMG 21292	Ketogulonicigenium	robustum
Z0015_LB_D09_1_C02_A	45	35	LMG 21292	Ketogulonicigenium	robustum
Z0015_LB_D10_1_C02_B	45	35	LMG 21292	Ketogulonicigenium	robustum
Z0015_LB_D11_1_C01_A	45	35	LMG 21292	Ketogulonicigenium	robustum
Z0015_LB_D12_1_C01_B	45	35	LMG 21292	Ketogulonicigenium	robustum
Z0015_LB_E01_2_B06_A	45	35	LMG 21292	Ketogulonicigenium	robustum
Z0015_LB_E02_2_B06_B	45	35	LMG 21292	Ketogulonicigenium	robustum
Z0015_LB_E03_2_B05_A	45	35	LMG 21292	Ketogulonicigenium	robustum
Z0015_RB_B05_1_D10_A	45	35	LMG 21292	Ketogulonicigenium	robustum
Z0015_RB_B06_1_D10_B	45	35	LMG 21292	Ketogulonicigenium	robustum
Z0015_RB_B07_1_D09_A	45	35	LMG 21292	Ketogulonicigenium	robustum
Z0015_RB_B08_1_D09_B	45	35	LMG 21292	Ketogulonicigenium	robustum
Z0015_RB_B09_1_D08_A	45	35	LMG 21292	Ketogulonicigenium	robustum
Z0015_RB_B10_1_D08_B	45	35	LMG 21292	Ketogulonicigenium	robustum
Z0015_RB_B11_1_D07_A	45	35	LMG 21292	Ketogulonicigenium	robustum
Z0015_RB_B12_1_D07_B	45	35	LMG 21292	Ketogulonicigenium	robustum
Z0015_RB_C01_2_C12_A	45	35	LMG 21292	Ketogulonicigenium	robustum
Z0015_RB_C02_2_C12_B	45	35	LMG 21292	Ketogulonicigenium	robustum
Z0015_RB_C03_2_C11_A	45	35	LMG 21292	Ketogulonicigenium	robustum
Z0015_RB_C04_2_C11_B	45	35	LMG 21292	Ketogulonicigenium	robustum
Z0015_RB_C05_2_C10_A	45	35	LMG 21292	Ketogulonicigenium	robustum
Z0015_RB_C06_2_C10_B	45	35	LMG 21292	Ketogulonicigenium	robustum
Z0015_RB_C07_2_C09_A	45	35	LMG 21292	Ketogulonicigenium	robustum
Z0015_LB_E04_2_B05_B	46	36	LMG 21311	Gluconacetobacter	azotocaptans
Z0015_LB_E05_2_B04_A	46	36	LMG 21311	Gluconacetobacter	azotocaptans
Z0015_LB_E06_2_B04_B	46	36	LMG 21311	Gluconacetobacter	azotocaptans
Z0015_LB_E07_2_B03_A	46	36	LMG 21311	Gluconacetobacter	azotocaptans
Z0015_LB_E08_2_B03_B	46	36	LMG 21311	Gluconacetobacter	azotocaptans
Z0015_LB_E09_2_B02_A	46	36	LMG 21311	Gluconacetobacter	azotocaptans
Z0015_LB_E10_2_B02_B	46	36	LMG 21311	Gluconacetobacter	azotocaptans
Z0015_LB_E11_2_B01_A	46	36	LMG 21311	Gluconacetobacter	azotocaptans
Z0015_LB_E12_2_B01_B	46	36	LMG 21311	Gluconacetobacter	azotocaptans
Z0015_LB_F01_1_B06_A	46	36	LMG 21311	Gluconacetobacter	azotocaptans
Z0015_LB_F02_1_B06_B	46	36	LMG 21311	Gluconacetobacter	azotocaptans

Z0015_LB_F03_1_B05_A	46	36	LMG 21311	Gluconacetobacter	azotocaptans
Z0015_LB_F04_1_B05_B	46	36	LMG 21311	Gluconacetobacter	azotocaptans
Z0015_LB_F05_1_B04_A	46	36	LMG 21311	Gluconacetobacter	azotocaptans
Z0015_LB_F06_1_B04_B	46	36	LMG 21311	Gluconacetobacter	azotocaptans
Z0015_RB_C08_2_C09_B	46	36	LMG 21311	Gluconacetobacter	azotocaptans
Z0015_RB_C09_2_C08_A	46	36	LMG 21311	Gluconacetobacter	azotocaptans
Z0015_RB_C10_2_C08_B	46	36	LMG 21311	Gluconacetobacter	azotocaptans
Z0015_RB_C11_2_C07_A	46	36	LMG 21311	Gluconacetobacter	azotocaptans
Z0015_RB_C12_2_C07_B	46	36	LMG 21311	Gluconacetobacter	azotocaptans
Z0015_RB_D01_1_C12_A	46	36	LMG 21311	Gluconacetobacter	azotocaptans
Z0015_RB_D02_1_C12_B	46	36	LMG 21311	Gluconacetobacter	azotocaptans
Z0015_RB_D03_1_C11_A	46	36	LMG 21311	Gluconacetobacter	azotocaptans
Z0015_RB_D04_1_C11_B	46	36	LMG 21311	Gluconacetobacter	azotocaptans
Z0015_RB_D05_1_C10_A	46	36	LMG 21311	Gluconacetobacter	azotocaptans
Z0015_RB_D06_1_C10_B	46	36	LMG 21311	Gluconacetobacter	azotocaptans
Z0015_RB_D07_1_C09_A	46	36	LMG 21311	Gluconacetobacter	azotocaptans
Z0015_RB_D08_1_C09_B	46	36	LMG 21311	Gluconacetobacter	azotocaptans
Z0015_RB_D09_1_C08_A	46	36	LMG 21311	Gluconacetobacter	azotocaptans
Z0015_RB_D10_1_C08_B	46	36	LMG 21311	Gluconacetobacter	azotocaptans
Z0015_RB_D11_1_C07_A	46	36	LMG 21311	Gluconacetobacter	azotocaptans
Z0015_RB_D12_1_C07_B	46	36	LMG 21311	Gluconacetobacter	azotocaptans
Z0015_LB_F07_1_B03_A	47	37	LMG 21371	Erwinia	carotovora
Z0015_LB_F08_1_B03_B	47	37	LMG 21371	Erwinia	carotovora
Z0015_LB_F09_1_B02_A	47	37	LMG 21371	Erwinia	carotovora
Z0015_LB_F10_1_B02_B	47	37	LMG 21371	Erwinia	carotovora
Z0015_LB_F11_1_B01_A	47	37	LMG 21371	Erwinia	carotovora
Z0015_LB_F12_1_B01_B	47	37	LMG 21371	Erwinia	carotovora
Z0015_LB_G01_2_A06_A	47	37	LMG 21371	Erwinia	carotovora
Z0015_LB_G02_2_A06_B	47	37	LMG 21371	Erwinia	carotovora
Z0015_LB_G03_2_A05_A	47	37	LMG 21371	Erwinia	carotovora
Z0015_LB_G04_2_A05_B	47	37	LMG 21371	Erwinia	carotovora
Z0015_LB_G05_2_A04_A	47	37	LMG 21371	Erwinia	carotovora
Z0015_LB_G06_2_A04_B	47	37	LMG 21371	Erwinia	carotovora
Z0015_LB_G07_2_A03_A	47	37	LMG 21371	Erwinia	carotovora
Z0015_LB_G08_2_A03_B	47	37	LMG 21371	Erwinia	carotovora
Z0015_LB_G09_2_A02_A	47	37	LMG 21371	Erwinia	carotovora
Z0015_LB_G10_2_A02_B	47	37	LMG 21371	Erwinia	carotovora
Z0015_LB_G11_2_A01_A	47	37	LMG 21371	Erwinia	carotovora
Z0015_RB_E01_2_B12_A	47	37	LMG 21371	Erwinia	carotovora
Z0015_RB_E02_2_B12_B	47	37	LMG 21371	Erwinia	carotovora
Z0015_RB_E03_2_B11_A	47	37	LMG 21371	Erwinia	carotovora
Z0015_RB_E04_2_B11_B	47	37	LMG 21371	Erwinia	carotovora
Z0015_RB_E05_2_B10_A	47	37	LMG 21371	Erwinia	carotovora
Z0015_RB_E06_2_B10_B	47	37	LMG 21371	Erwinia	carotovora
Z0015_RB_E07_2_B09_A	47	37	LMG 21371	Erwinia	carotovora
Z0015_RB_E08_2_B09_B	47	37	LMG 21371	Erwinia	carotovora
Z0015_RB_E09_2_B08_A	47	37	LMG 21371	Erwinia	carotovora
Z0015_RB_E10_2_B08_B	47	37	LMG 21371	Erwinia	carotovora
Z0015_RB_E11_2_B07_A	47	37	LMG 21371	Erwinia	carotovora
Z0015_RB_E12_2_B07_B	47	37	LMG 21371	Erwinia	carotovora

Z0015_RB_F01_1_B12_A	47	37	LMG 21371	Erwinia	carotovora
Z0015_RB_F02_1_B12_B	47	37	LMG 21371	Erwinia	carotovora
Z0015_RB_F03_1_B11_A	47	37	LMG 21371	Erwinia	carotovora
Z0015_LB_G12_2_A01_B	48	38	LMG 21530	Massilia	timonae
Z0015_LB_H01_1_A06_A	48	38	LMG 21530	Massilia	timonae
Z0015_LB_H02_1_A06_B	48	38	LMG 21530	Massilia	timonae
Z0015_LB_H03_1_A05_A	48	38	LMG 21530	Massilia	timonae
Z0015_LB_H04_1_A05_B	48	38	LMG 21530	Massilia	timonae
Z0015_LB_H05_1_A04_A	48	38	LMG 21530	Massilia	timonae
Z0015_LB_H06_1_A04_B	48	38	LMG 21530	Massilia	timonae
Z0015_LB_H07_1_A03_A	48	38	LMG 21530	Massilia	timonae
Z0015_LB_H08_1_A03_B	48	38	LMG 21530	Massilia	timonae
Z0015_LB_H09_1_A02_A	48	38	LMG 21530	Massilia	timonae
Z0015_LB_H10_1_A02_B	48	38	LMG 21530	Massilia	timonae
Z0015_RB_F04_1_B11_B	48	38	LMG 21530	Massilia	timonae
Z0015_RB_F05_1_B10_A	48	38	LMG 21530	Massilia	timonae
Z0015_RB_F06_1_B10_B	48	38	LMG 21530	Massilia	timonae
Z0015_RB_F07_1_B09_A	48	38	LMG 21530	Massilia	timonae
Z0015_RB_F08_1_B09_B	48	38	LMG 21530	Massilia	timonae
Z0015_RB_F09_1_B08_A	48	38	LMG 21530	Massilia	timonae
Z0015_RB_F10_1_B08_B	48	38	LMG 21530	Massilia	timonae
Z0015_RB_F11_1_B07_A	48	38	LMG 21530	Massilia	timonae
Z0015_RB_F12_1_B07_B	48	38	LMG 21530	Massilia	timonae
Z0015_RB_G01_2_A12_A	48	38	LMG 21530	Massilia	timonae
Z0015_RB_G02_2_A12_B	48	38	LMG 21530	Massilia	timonae
Z0015_RB_G03_2_A11_A	48	38	LMG 21530	Massilia	timonae
Z0015_RB_G04_2_A11_B	48	38	LMG 21530	Massilia	timonae
Z0015_RB_G05_2_A10_A	48	38	LMG 21530	Massilia	timonae
Z0015_RB_G06_2_A10_B	48	38	LMG 21530	Massilia	timonae
Z0015_RB_G07_2_A09_A	48	38	LMG 21530	Massilia	timonae
Z0015_RB_G08_2_A09_B	48	38	LMG 21530	Massilia	timonae
Z0015_RB_G09_2_A08_A	48	38	LMG 21530	Massilia	timonae
Z0015_RB_G10_2_A08_B	48	38	LMG 21530	Massilia	timonae
Z0015_RB_G11_2_A07_A	48	38	LMG 21530	Massilia	timonae
Z0015_RB_G12_2_A07_B	48	38	LMG 21530	Massilia	timonae
Z0015_LO_B01_2_E01_A	49	39	LMG 21665	Pigmentiphaga	kullae
Z0015_LO_B02_2_E01_B	49	39	LMG 21665	Pigmentiphaga	kullae
Z0015_LO_B03_2_E02_A	49	39	LMG 21665	Pigmentiphaga	kullae
Z0015_LO_B04_2_E02_B	49	39	LMG 21665	Pigmentiphaga	kullae
Z0015_LO_B05_2_E03_A	49	39	LMG 21665	Pigmentiphaga	kullae
Z0015_LO_B06_2_E03_B	49	39	LMG 21665	Pigmentiphaga	kullae
Z0015_LO_B07_2_E04_A	49	39	LMG 21665	Pigmentiphaga	kullae
Z0015_RO_A03_1_E08_A	49	39	LMG 21665	Pigmentiphaga	kullae
Z0015_RO_A04_1_E08_B	49	39	LMG 21665	Pigmentiphaga	kullae
Z0015_RO_A05_1_E09_A	49	39	LMG 21665	Pigmentiphaga	kullae
Z0015_RO_A06_1_E09_B	49	39	LMG 21665	Pigmentiphaga	kullae
Z0015_RO_A07_1_E10_A	49	39	LMG 21665	Pigmentiphaga	kullae
Z0015_RO_A08_1_E10_B	49	39	LMG 21665	Pigmentiphaga	kullae
Z0015_RO_A09_1_E11_A	49	39	LMG 21665	Pigmentiphaga	kullae
Z0015_RO_A10_1_E11_B	49	39	LMG 21665	Pigmentiphaga	kullae

Z0015_RO_A11_1_E12_A	49	39	LMG 21665	Pigmentiphaga	kullae
Z0015_RO_A12_1_E12_B	49	39	LMG 21665	Pigmentiphaga	kullae
Z0015_RO_B01_2_E07_A	49	39	LMG 21665	Pigmentiphaga	kullae
Z0015_RO_B02_2_E07_B	49	39	LMG 21665	Pigmentiphaga	kullae
Z0015_RO_B03_2_E08_A	49	39	LMG 21665	Pigmentiphaga	kullae
Z0015_RO_B04_2_E08_B	49	39	LMG 21665	Pigmentiphaga	kullae
Z0015_RO_B05_2_E09_A	49	39	LMG 21665	Pigmentiphaga	kullae
Z0015_RO_B06_2_E09_B	49	39	LMG 21665	Pigmentiphaga	kullae
Z0015_RO_B07_2_E10_A	49	39	LMG 21665	Pigmentiphaga	kullae
Z0015_RO_B08_2_E10_B	49	39	LMG 21665	Pigmentiphaga	kullae
Z0015_RO_B09_2_E11_A	49	39	LMG 21665	Pigmentiphaga	kullae
Z0015_RO_B10_2_E11_B	49	39	LMG 21665	Pigmentiphaga	kullae
Z0015_RO_B11_2_E12_A	49	39	LMG 21665	Pigmentiphaga	kullae
Z0015_LO_B08_2_E04_B	50	40	LMG 21817	Woodsholea	maritima
Z0015_LO_B09_2_E05_A	50	40	LMG 21817	Woodsholea	maritima
Z0015_LO_B12_2_E06_B	50	40	LMG 21817	Woodsholea	maritima
Z0015_LO_C02_1_F01_B	50	40	LMG 21817	Woodsholea	maritima
Z0015_LO_C03_1_F02_A	50	40	LMG 21817	Woodsholea	maritima
Z0015_LO_C04_1_F02_B	50	40	LMG 21817	Woodsholea	maritima
Z0015_LO_C12_1_F06_B	50	40	LMG 21817	Woodsholea	maritima
Z0015_LO_D01_2_F01_A	50	40	LMG 21817	Woodsholea	maritima
Z0015_LO_D02_2_F01_B	50	40	LMG 21817	Woodsholea	maritima
Z0015_LO_D04_2_F02_B	50	40	LMG 21817	Woodsholea	maritima
Z0015_LO_D09_2_F05_A	50	40	LMG 21817	Woodsholea	maritima
Z0015_LO_D11_2_F06_A	50	40	LMG 21817	Woodsholea	maritima
Z0015_RO_B12_2_E12_B	50	40	LMG 21817	Woodsholea	maritima
Z0015_RO_C01_1_F07_A	50	40	LMG 21817	Woodsholea	maritima
Z0015_RO_C02_1_F07_B	50	40	LMG 21817	Woodsholea	maritima
Z0015_RO_C03_1_F08_A	50	40	LMG 21817	Woodsholea	maritima
Z0015_RO_C04_1_F08_B	50	40	LMG 21817	Woodsholea	maritima
Z0015_RO_C05_1_F09_A	50	40	LMG 21817	Woodsholea	maritima
Z0015_RO_C07_1_F10_A	50	40	LMG 21817	Woodsholea	maritima
Z0015_RO_C11_1_F12_A	50	40	LMG 21817	Woodsholea	maritima
Z0015_RO_C12_1_F12_B	50	40	LMG 21817	Woodsholea	maritima
Z0015_RO_D01_2_F07_A	50	40	LMG 21817	Woodsholea	maritima
Z0015_RO_D02_2_F07_B	50	40	LMG 21817	Woodsholea	maritima
Z0015_RO_D03_2_F08_A	50	40	LMG 21817	Woodsholea	maritima
Z0015_RO_D04_2_F08_B	50	40	LMG 21817	Woodsholea	maritima
Z0015_RO_D05_2_F09_A	50	40	LMG 21817	Woodsholea	maritima
Z0015_RO_D09_2_F11_A	50	40	LMG 21817	Woodsholea	maritima
Z0015_RO_D11_2_F12_A	50	40	LMG 21817	Woodsholea	maritima
Z0015_RO_D12_2_F12_B	50	40	LMG 21817	Woodsholea	maritima
Z0015_RO_E01_1_G07_A	50	40	LMG 21817	Woodsholea	maritima
Z0015_RO_E02_1_G07_B	50	40	LMG 21817	Woodsholea	maritima
Z0015_RO_E04_1_G08_B	50	40	LMG 21817	Woodsholea	maritima
Z0015_LO_B10_2_E05_B	51	43	LMG 22193	Martelella	mediterranea
Z0015_LO_B11_2_E06_A	51	43	LMG 22193	Martelella	mediterranea
Z0015_LO_C01_1_F01_A	51	43	LMG 22193	Martelella	mediterranea
Z0015_LO_C05_1_F03_A	51	43	LMG 22193	Martelella	mediterranea
Z0015_LO_C06_1_F03_B	51	43	LMG 22193	Martelella	mediterranea

Z0015_LO_C07_1_F04_A	51	43	LMG 22193	Martelella	mediterranea
Z0015_LO_C08_1_F04_B	51	43	LMG 22193	Martelella	mediterranea
Z0015_LO_C09_1_F05_A	51	43	LMG 22193	Martelella	mediterranea
Z0015_LO_C10_1_F05_B	51	43	LMG 22193	Martelella	mediterranea
Z0015_LO_C11_1_F06_A	51	43	LMG 22193	Martelella	mediterranea
Z0015_LO_D03_2_F02_A	51	43	LMG 22193	Martelella	mediterranea
Z0015_LO_D05_2_F03_A	51	43	LMG 22193	Martelella	mediterranea
Z0015_LO_D06_2_F03_B	51	43	LMG 22193	Martelella	mediterranea
Z0015_LO_D07_2_F04_A	51	43	LMG 22193	Martelella	mediterranea
Z0015_LO_D08_2_F04_B	51	43	LMG 22193	Martelella	mediterranea
Z0015_LO_D10_2_F05_B	51	43	LMG 22193	Martelella	mediterranea
Z0015_RO_C06_1_F09_B	51	43	LMG 22193	Martelella	mediterranea
Z0015_RO_C08_1_F10_B	51	43	LMG 22193	Martelella	mediterranea
Z0015_RO_C09_1_F11_A	51	43	LMG 22193	Martelella	mediterranea
Z0015_RO_C10_1_F11_B	51	43	LMG 22193	Martelella	mediterranea
Z0015_RO_D06_2_F09_B	51	43	LMG 22193	Martelella	mediterranea
Z0015_RO_D07_2_F10_A	51	43	LMG 22193	Martelella	mediterranea
Z0015_RO_D08_2_F10_B	51	43	LMG 22193	Martelella	mediterranea
Z0015_RO_D10_2_F11_B	51	43	LMG 22193	Martelella	mediterranea
Z0015_RO_E03_1_G08_A	51	43	LMG 22193	Martelella	mediterranea
Z0015_RO_E05_1_G09_A	51	43	LMG 22193	Martelella	mediterranea
Z0015_RO_E06_1_G09_B	51	43	LMG 22193	Martelella	mediterranea
Z0015_RO_E09_1_G11_A	51	43	LMG 22193	Martelella	mediterranea
Z0015_RO_E10_1_G11_B	51	43	LMG 22193	Martelella	mediterranea
Z0015_RO_E12_1_G12_B	51	43	LMG 22193	Martelella	mediterranea
Z0015_RO_F01_2_G07_A	51	43	LMG 22193	Martelella	mediterranea
Z0015_RO_F03_2_G08_A	51	43	LMG 22193	Martelella	mediterranea
Z0015_LO_D12_2_F06_B	52	41	LMG 2186	Phaseolibacter	flectens
Z0015_LO_E03_1_G02_A	52	41	LMG 2186	Phaseolibacter	flectens
Z0015_LO_E04_1_G02_B	52	41	LMG 2186	Phaseolibacter	flectens
Z0015_LO_E06_1_G03_B	52	41	LMG 2186	Phaseolibacter	flectens
Z0015_LO_E07_1_G04_A	52	41	LMG 2186	Phaseolibacter	flectens
Z0015_LO_E09_1_G05_A	52	41	LMG 2186	Phaseolibacter	flectens
Z0015_LO_F02_2_G01_B	52	41	LMG 2186	Phaseolibacter	flectens
Z0015_LO_F04_2_G02_B	52	41	LMG 2186	Phaseolibacter	flectens
Z0015_LO_F07_2_G04_A	52	41	LMG 2186	Phaseolibacter	flectens
Z0015_LO_F12_2_G06_B	52	41	LMG 2186	Phaseolibacter	flectens
Z0015_LO_G01_1_H01_A	52	41	LMG 2186	Phaseolibacter	flectens
Z0015_LO_G02_1_H01_B	52	41	LMG 2186	Phaseolibacter	flectens
Z0015_LO_G03_1_H02_A	52	41	LMG 2186	Phaseolibacter	flectens
Z0015_LO_G04_1_H02_B	52	41	LMG 2186	Phaseolibacter	flectens
Z0015_RO_E07_1_G10_A	52	41	LMG 2186	Phaseolibacter	flectens
Z0015_RO_E08_1_G10_B	52	41	LMG 2186	Phaseolibacter	flectens
Z0015_RO_E11_1_G12_A	52	41	LMG 2186	Phaseolibacter	flectens
Z0015_RO_F02_2_G07_B	52	41	LMG 2186	Phaseolibacter	flectens
Z0015_RO_F04_2_G08_B	52	41	LMG 2186	Phaseolibacter	flectens
Z0015_RO_F05_2_G09_A	52	41	LMG 2186	Phaseolibacter	flectens
Z0015_RO_F06_2_G09_B	52	41	LMG 2186	Phaseolibacter	flectens
Z0015_RO_F07_2_G10_A	52	41	LMG 2186	Phaseolibacter	flectens
Z0015_RO_F09_2_G11_A	52	41	LMG 2186	Phaseolibacter	flectens

Z0015_RO_F11_2_G12_A	52	41	LMG 2186	Phaseolibacter	flectens
Z0015_RO_F12_2_G12_B	52	41	LMG 2186	Phaseolibacter	flectens
Z0015_RO_G02_1_H07_B	52	41	LMG 2186	Phaseolibacter	flectens
Z0015_RO_G03_1_H08_A	52	41	LMG 2186	Phaseolibacter	flectens
Z0015_RO_G04_1_H08_B	52	41	LMG 2186	Phaseolibacter	flectens
Z0015_RO_G05_1_H09_A	52	41	LMG 2186	Phaseolibacter	flectens
Z0015_RO_G11_1_H12_A	52	41	LMG 2186	Phaseolibacter	flectens
Z0015_RO_G12_1_H12_B	52	41	LMG 2186	Phaseolibacter	flectens
Z0015_RO_H01_2_H07_A	52	41	LMG 2186	Phaseolibacter	flectens
Z0015_LO_E01_1_G01_A	53	42	LMG 22049	Tatumella	citrea
Z0015_LO_E02_1_G01_B	53	42	LMG 22049	Tatumella	citrea
Z0015_LO_E05_1_G03_A	53	42	LMG 22049	Tatumella	citrea
Z0015_LO_E08_1_G04_B	53	42	LMG 22049	Tatumella	citrea
Z0015_LO_E10_1_G05_B	53	42	LMG 22049	Tatumella	citrea
Z0015_LO_E11_1_G06_A	53	42	LMG 22049	Tatumella	citrea
Z0015_LO_E12_1_G06_B	53	42	LMG 22049	Tatumella	citrea
Z0015_LO_F01_2_G01_A	53	42	LMG 22049	Tatumella	citrea
Z0015_LO_F03_2_G02_A	53	42	LMG 22049	Tatumella	citrea
Z0015_LO_F05_2_G03_A	53	42	LMG 22049	Tatumella	citrea
Z0015_LO_F06_2_G03_B	53	42	LMG 22049	Tatumella	citrea
Z0015_LO_F08_2_G04_B	53	42	LMG 22049	Tatumella	citrea
Z0015_LO_F09_2_G05_A	53	42	LMG 22049	Tatumella	citrea
Z0015_LO_F10_2_G05_B	53	42	LMG 22049	Tatumella	citrea
Z0015_LO_F11_2_G06_A	53	42	LMG 22049	Tatumella	citrea
Z0015_LO_G05_1_H03_A	53	42	LMG 22049	Tatumella	citrea
Z0015_LO_G06_1_H03_B	53	42	LMG 22049	Tatumella	citrea
Z0015_LO_G07_1_H04_A	53	42	LMG 22049	Tatumella	citrea
Z0015_LO_G08_1_H04_B	53	42	LMG 22049	Tatumella	citrea
Z0015_LO_G09_1_H05_A	53	42	LMG 22049	Tatumella	citrea
Z0015_LO_G10_1_H05_B	53	42	LMG 22049	Tatumella	citrea
Z0015_RO_F08_2_G10_B	53	42	LMG 22049	Tatumella	citrea
Z0015_RO_F10_2_G11_B	53	42	LMG 22049	Tatumella	citrea
Z0015_RO_G01_1_H07_A	53	42	LMG 22049	Tatumella	citrea
Z0015_RO_G06_1_H09_B	53	42	LMG 22049	Tatumella	citrea
Z0015_RO_G07_1_H10_A	53	42	LMG 22049	Tatumella	citrea
Z0015_RO_G08_1_H10_B	53	42	LMG 22049	Tatumella	citrea
Z0015_RO_G09_1_H11_A	53	42	LMG 22049	Tatumella	citrea
Z0015_RO_G10_1_H11_B	53	42	LMG 22049	Tatumella	citrea
Z0015_RO_H02_2_H07_B	53	42	LMG 22049	Tatumella	citrea
Z0015_RO_H03_2_H08_A	53	42	LMG 22049	Tatumella	citrea
Z0015_RO_H04_2_H08_B	53	42	LMG 22049	Tatumella	citrea
Z0016_LB_A01_2_D06_A	54	44	LMG 22214	Aeromonas	molluscorum
Z0016_LB_A02_2_D06_B	54	44	LMG 22214	Aeromonas	molluscorum
Z0016_LB_A03_2_D05_A	54	44	LMG 22214	Aeromonas	molluscorum
Z0016_LB_A04_2_D05_B	54	44	LMG 22214	Aeromonas	molluscorum
Z0016_LB_A05_2_D04_A	54	44	LMG 22214	Aeromonas	molluscorum
Z0016_LB_A06_2_D04_B	54	44	LMG 22214	Aeromonas	molluscorum
Z0016_LB_A07_2_D03_A	54	44	LMG 22214	Aeromonas	molluscorum
Z0016_LB_A08_2_D03_B	54	44	LMG 22214	Aeromonas	molluscorum
Z0016_LB_A09_2_D02_A	54	44	LMG 22214	Aeromonas	molluscorum

Z0016_LB_A10_2_D02_B	54	44	LMG 22214	Aeromonas	molluscorum
Z0016_LB_A11_2_D01_A	54	44	LMG 22214	Aeromonas	molluscorum
Z0016_LO_C06_1_F03_B	54	44	LMG 22214	Aeromonas	molluscorum
Z0016_LO_C08_1_F04_B	54	44	LMG 22214	Aeromonas	molluscorum
Z0016_LO_D04_2_F02_B	54	44	LMG 22214	Aeromonas	molluscorum
Z0016_LO_H06_2_H03_B	54	44	LMG 22214	Aeromonas	molluscorum
Z0016_LO_H07_2_H04_A	54	44	LMG 22214	Aeromonas	molluscorum
Z0016_LO_H08_2_H04_B	54	44	LMG 22214	Aeromonas	molluscorum
Z0016_LO_H09_2_H05_A	54	44	LMG 22214	Aeromonas	molluscorum
Z0016_LO_H10_2_H05_B	54	44	LMG 22214	Aeromonas	molluscorum
Z0016_LO_H11_2_H06_A	54	44	LMG 22214	Aeromonas	molluscorum
Z0016_LO_H12_2_H06_B	54	44	LMG 22214	Aeromonas	molluscorum
Z0016_RB_A01_2_D12_A	54	44	LMG 22214	Aeromonas	molluscorum
Z0016_RB_A02_2_D12_B	54	44	LMG 22214	Aeromonas	molluscorum
Z0016_RB_A03_2_D11_A	54	44	LMG 22214	Aeromonas	molluscorum
Z0016_RB_A04_2_D11_B	54	44	LMG 22214	Aeromonas	molluscorum
Z0016_RB_A05_2_D10_A	54	44	LMG 22214	Aeromonas	molluscorum
Z0016_RB_A06_2_D10_B	54	44	LMG 22214	Aeromonas	molluscorum
Z0016_RO_B07_2_E10_A	54	44	LMG 22214	Aeromonas	molluscorum
Z0016_RO_C05_1_F09_A	54	44	LMG 22214	Aeromonas	molluscorum
Z0016_RO_D02_2_F07_B	54	44	LMG 22214	Aeromonas	molluscorum
Z0016_RO_E06_1_G09_B	54	44	LMG 22214	Aeromonas	molluscorum
Z0016_RO_E12_1_G12_B	54	44	LMG 22214	Aeromonas	molluscorum
Z0016_LB_C06_2_C04_B	55	45	LMG 22475	Phaeobacter	inhibens
Z0016_LB_C07_2_C03_A	55	45	LMG 22475	Phaeobacter	inhibens
Z0016_LB_C08_2_C03_B	55	45	LMG 22475	Phaeobacter	inhibens
Z0016_LB_C09_2_C02_A	55	45	LMG 22475	Phaeobacter	inhibens
Z0016_LB_C10_2_C02_B	55	45	LMG 22475	Phaeobacter	inhibens
Z0016_LB_C11_2_C01_A	55	45	LMG 22475	Phaeobacter	inhibens
Z0016_LB_C12_2_C01_B	55	45	LMG 22475	Phaeobacter	inhibens
Z0016_LB_D01_1_C06_A	55	45	LMG 22475	Phaeobacter	inhibens
Z0016_LB_D02_1_C06_B	55	45	LMG 22475	Phaeobacter	inhibens
Z0016_LB_D03_1_C05_A	55	45	LMG 22475	Phaeobacter	inhibens
Z0016_LB_D04_1_C05_B	55	45	LMG 22475	Phaeobacter	inhibens
Z0016_LB_D05_1_C04_A	55	45	LMG 22475	Phaeobacter	inhibens
Z0016_LB_D06_1_C04_B	55	45	LMG 22475	Phaeobacter	inhibens
Z0016_RB_B09_1_D08_A	55	45	LMG 22475	Phaeobacter	inhibens
Z0016_RB_B10_1_D08_B	55	45	LMG 22475	Phaeobacter	inhibens
Z0016_RB_B11_1_D07_A	55	45	LMG 22475	Phaeobacter	inhibens
Z0016_RB_B12_1_D07_B	55	45	LMG 22475	Phaeobacter	inhibens
Z0016_RB_C01_2_C12_A	55	45	LMG 22475	Phaeobacter	inhibens
Z0016_RB_C02_2_C12_B	55	45	LMG 22475	Phaeobacter	inhibens
Z0016_RB_C03_2_C11_A	55	45	LMG 22475	Phaeobacter	inhibens
Z0016_RB_C04_2_C11_B	55	45	LMG 22475	Phaeobacter	inhibens
Z0016_RB_C05_2_C10_A	55	45	LMG 22475	Phaeobacter	inhibens
Z0016_RB_C06_2_C10_B	55	45	LMG 22475	Phaeobacter	inhibens
Z0016_RB_C07_2_C09_A	55	45	LMG 22475	Phaeobacter	inhibens
Z0016_RB_C08_2_C09_B	55	45	LMG 22475	Phaeobacter	inhibens
Z0016_RB_C09_2_C08_A	55	45	LMG 22475	Phaeobacter	inhibens
Z0016_RB_C10_2_C08_B	55	45	LMG 22475	Phaeobacter	inhibens

Z0016_RB_C11_2_C07_A	55	45	LMG 22475	Phaeobacter	inhibens
Z0016_RB_C12_2_C07_B	55	45	LMG 22475	Phaeobacter	inhibens
Z0016_RB_D01_1_C12_A	55	45	LMG 22475	Phaeobacter	inhibens
Z0016_RB_D02_1_C12_B	55	45	LMG 22475	Phaeobacter	inhibens
Z0016_RB_D03_1_C11_A	55	45	LMG 22475	Phaeobacter	inhibens
Z0016_LB_D07_1_C03_A	56	46	LMG 22485	Burkholderia	lata
Z0016_LB_D08_1_C03_B	56	46	LMG 22485	Burkholderia	lata
Z0016_LB_D09_1_C02_A	56	46	LMG 22485	Burkholderia	lata
Z0016_LB_D10_1_C02_B	56	46	LMG 22485	Burkholderia	lata
Z0016_LB_D11_1_C01_A	56	46	LMG 22485	Burkholderia	lata
Z0016_LB_D12_1_C01_B	56	46	LMG 22485	Burkholderia	lata
Z0016_LB_E01_2_B06_A	56	46	LMG 22485	Burkholderia	lata
Z0016_LB_E02_2_B06_B	56	46	LMG 22485	Burkholderia	lata
Z0016_LB_E03_2_B05_A	56	46	LMG 22485	Burkholderia	lata
Z0016_LB_E04_2_B05_B	56	46	LMG 22485	Burkholderia	lata
Z0016_LB_E05_2_B04_A	56	46	LMG 22485	Burkholderia	lata
Z0016_LB_E06_2_B04_B	56	46	LMG 22485	Burkholderia	lata
Z0016_LB_E07_2_B03_A	56	46	LMG 22485	Burkholderia	lata
Z0016_LB_E08_2_B03_B	56	46	LMG 22485	Burkholderia	lata
Z0016_LB_E09_2_B02_A	56	46	LMG 22485	Burkholderia	lata
Z0016_RB_D04_1_C11_B	56	46	LMG 22485	Burkholderia	lata
Z0016_RB_D05_1_C10_A	56	46	LMG 22485	Burkholderia	lata
Z0016_RB_D06_1_C10_B	56	46	LMG 22485	Burkholderia	lata
Z0016_RB_D07_1_C09_A	56	46	LMG 22485	Burkholderia	lata
Z0016_RB_D08_1_C09_B	56	46	LMG 22485	Burkholderia	lata
Z0016_RB_D09_1_C08_A	56	46	LMG 22485	Burkholderia	lata
Z0016_RB_D10_1_C08_B	56	46	LMG 22485	Burkholderia	lata
Z0016_RB_D11_1_C07_A	56	46	LMG 22485	Burkholderia	lata
Z0016_RB_D12_1_C07_B	56	46	LMG 22485	Burkholderia	lata
Z0016_RB_E01_2_B12_A	56	46	LMG 22485	Burkholderia	lata
Z0016_RB_E02_2_B12_B	56	46	LMG 22485	Burkholderia	lata
Z0016_RB_E03_2_B11_A	56	46	LMG 22485	Burkholderia	lata
Z0016_RB_E04_2_B11_B	56	46	LMG 22485	Burkholderia	lata
Z0016_RB_E05_2_B10_A	56	46	LMG 22485	Burkholderia	lata
Z0016_RB_E06_2_B10_B	56	46	LMG 22485	Burkholderia	lata
Z0016_RB_E07_2_B09_A	56	46	LMG 22485	Burkholderia	lata
Z0016_RB_E08_2_B09_B	56	46	LMG 22485	Burkholderia	lata
Z0016_LB_E10_2_B02_B	57	47	LMG 22585	Gramella	echinicola
Z0016_LB_E11_2_B01_A	57	47	LMG 22585	Gramella	echinicola
Z0016_LB_E12_2_B01_B	57	47	LMG 22585	Gramella	echinicola
Z0016_LB_F01_1_B06_A	57	47	LMG 22585	Gramella	echinicola
Z0016_LB_F02_1_B06_B	57	47	LMG 22585	Gramella	echinicola
Z0016_LB_F03_1_B05_A	57	47	LMG 22585	Gramella	echinicola
Z0016_LB_F04_1_B05_B	57	47	LMG 22585	Gramella	echinicola
Z0016_LB_F05_1_B04_A	57	47	LMG 22585	Gramella	echinicola
Z0016_LB_F06_1_B04_B	57	47	LMG 22585	Gramella	echinicola
Z0016_LB_F07_1_B03_A	57	47	LMG 22585	Gramella	echinicola
Z0016_LB_F08_1_B03_B	57	47	LMG 22585	Gramella	echinicola
Z0016_LB_F09_1_B02_A	57	47	LMG 22585	Gramella	echinicola
Z0016_LB_F10_1_B02_B	57	47	LMG 22585	Gramella	echinicola

Z0016_LB_F11_1_B01_A	57	47	LMG 22585	Gramella	echinicola
Z0016_LB_F12_1_B01_B	57	47	LMG 22585	Gramella	echinicola
Z0016_LB_G01_2_A06_A	57	47	LMG 22585	Gramella	echinicola
Z0016_LB_G02_2_A06_B	57	47	LMG 22585	Gramella	echinicola
Z0016_RB_E09_2_B08_A	57	47	LMG 22585	Gramella	echinicola
Z0016_RB_E10_2_B08_B	57	47	LMG 22585	Gramella	echinicola
Z0016_RB_E11_2_B07_A	57	47	LMG 22585	Gramella	echinicola
Z0016_RB_E12_2_B07_B	57	47	LMG 22585	Gramella	echinicola
Z0016_RB_F01_1_B12_A	57	47	LMG 22585	Gramella	echinicola
Z0016_RB_F02_1_B12_B	57	47	LMG 22585	Gramella	echinicola
Z0016_RB_F03_1_B11_A	57	47	LMG 22585	Gramella	echinicola
Z0016_RB_F04_1_B11_B	57	47	LMG 22585	Gramella	echinicola
Z0016_RB_F05_1_B10_A	57	47	LMG 22585	Gramella	echinicola
Z0016_RB_F06_1_B10_B	57	47	LMG 22585	Gramella	echinicola
Z0016_RB_F07_1_B09_A	57	47	LMG 22585	Gramella	echinicola
Z0016_RB_F08_1_B09_B	57	47	LMG 22585	Gramella	echinicola
Z0016_RB_F09_1_B08_A	57	47	LMG 22585	Gramella	echinicola
Z0016_RB_F10_1_B08_B	57	47	LMG 22585	Gramella	echinicola
Z0016_RB_F11_1_B07_A	57	47	LMG 22585	Gramella	echinicola
Z0016_LB_G03_2_A05_A	58	48	LMG 22697	Mesorhizobium	thiogangeticum
Z0016_LB_G04_2_A05_B	58	48	LMG 22697	Mesorhizobium	thiogangeticum
Z0016_LB_G05_2_A04_A	58	48	LMG 22697	Mesorhizobium	thiogangeticum
Z0016_LB_G06_2_A04_B	58	48	LMG 22697	Mesorhizobium	thiogangeticum
Z0016_LB_G07_2_A03_A	58	48	LMG 22697	Mesorhizobium	thiogangeticum
Z0016_LB_G08_2_A03_B	58	48	LMG 22697	Mesorhizobium	thiogangeticum
Z0016_LB_G09_2_A02_A	58	48	LMG 22697	Mesorhizobium	thiogangeticum
Z0016_LB_G10_2_A02_B	58	48	LMG 22697	Mesorhizobium	thiogangeticum
Z0016_LB_G11_2_A01_A	58	48	LMG 22697	Mesorhizobium	thiogangeticum
Z0016_LB_G12_2_A01_B	58	48	LMG 22697	Mesorhizobium	thiogangeticum
Z0016_LB_H01_1_A06_A	58	48	LMG 22697	Mesorhizobium	thiogangeticum
Z0016_LB_H02_1_A06_B	58	48	LMG 22697	Mesorhizobium	thiogangeticum
Z0016_LB_H03_1_A05_A	58	48	LMG 22697	Mesorhizobium	thiogangeticum
Z0016_LB_H04_1_A05_B	58	48	LMG 22697	Mesorhizobium	thiogangeticum
Z0016_LB_H05_1_A04_A	58	48	LMG 22697	Mesorhizobium	thiogangeticum
Z0016_LB_H06_1_A04_B	58	48	LMG 22697	Mesorhizobium	thiogangeticum
Z0016_RB_F12_1_B07_B	58	48	LMG 22697	Mesorhizobium	thiogangeticum
Z0016_RB_G01_2_A12_A	58	48	LMG 22697	Mesorhizobium	thiogangeticum
Z0016_RB_G02_2_A12_B	58	48	LMG 22697	Mesorhizobium	thiogangeticum
Z0016_RB_G03_2_A11_A	58	48	LMG 22697	Mesorhizobium	thiogangeticum
Z0016_RB_G04_2_A11_B	58	48	LMG 22697	Mesorhizobium	thiogangeticum
Z0016_RB_G05_2_A10_A	58	48	LMG 22697	Mesorhizobium	thiogangeticum
Z0016_RB_G06_2_A10_B	58	48	LMG 22697	Mesorhizobium	thiogangeticum
Z0016_RB_G07_2_A09_A	58	48	LMG 22697	Mesorhizobium	thiogangeticum
Z0016_RB_G08_2_A09_B	58	48	LMG 22697	Mesorhizobium	thiogangeticum
Z0016_RB_G09_2_A08_A	58	48	LMG 22697	Mesorhizobium	thiogangeticum
Z0016_RB_G10_2_A08_B	58	48	LMG 22697	Mesorhizobium	thiogangeticum
Z0016_RB_G11_2_A07_A	58	48	LMG 22697	Mesorhizobium	thiogangeticum
Z0016_RB_G12_2_A07_B	58	48	LMG 22697	Mesorhizobium	thiogangeticum
Z0016_RB_H01_1_A12_A	58	48	LMG 22697	Mesorhizobium	thiogangeticum
Z0016_RB_H02_1_A12_B	58	48	LMG 22697	Mesorhizobium	thiogangeticum

Z0016_RB_H03_1_A11_A	58	48	LMG 22697	Mesorhizobium	thiogangeticum
Z0016_LB_H07_1_A03_A	59	49	LMG 22735	Finegoldia	magna
Z0016_LB_H08_1_A03_B	59	49	LMG 22735	Finegoldia	magna
Z0016_LB_H09_1_A02_A	59	49	LMG 22735	Finegoldia	magna
Z0016_LB_H10_1_A02_B	59	49	LMG 22735	Finegoldia	magna
Z0016_LB_H11_1_A01_A	59	49	LMG 22735	Finegoldia	magna
Z0016_LB_H12_1_A01_B	59	49	LMG 22735	Finegoldia	magna
Z0016_LO_A01_1_E01_A	59	49	LMG 22735	Finegoldia	magna
Z0016_LO_A02_1_E01_B	59	49	LMG 22735	Finegoldia	magna
Z0016_LO_A03_1_E02_A	59	49	LMG 22735	Finegoldia	magna
Z0016_LO_A04_1_E02_B	59	49	LMG 22735	Finegoldia	magna
Z0016_LO_A05_1_E03_A	59	49	LMG 22735	Finegoldia	magna
Z0016_LO_A06_1_E03_B	59	49	LMG 22735	Finegoldia	magna
Z0016_LO_A07_1_E04_A	59	49	LMG 22735	Finegoldia	magna
Z0016_LO_A08_1_E04_B	59	49	LMG 22735	Finegoldia	magna
Z0016_LO_A09_1_E05_A	59	49	LMG 22735	Finegoldia	magna
Z0016_RB_H04_1_A11_B	59	49	LMG 22735	Finegoldia	magna
Z0016_RB_H05_1_A10_A	59	49	LMG 22735	Finegoldia	magna
Z0016_RB_H06_1_A10_B	59	49	LMG 22735	Finegoldia	magna
Z0016_RB_H07_1_A09_A	59	49	LMG 22735	Finegoldia	magna
Z0016_RB_H08_1_A09_B	59	49	LMG 22735	Finegoldia	magna
Z0016_RB_H09_1_A08_A	59	49	LMG 22735	Finegoldia	magna
Z0016_RB_H10_1_A08_B	59	49	LMG 22735	Finegoldia	magna
Z0016_RB_H11_1_A07_A	59	49	LMG 22735	Finegoldia	magna
Z0016_RB_H12_1_A07_B	59	49	LMG 22735	Finegoldia	magna
Z0016_RO_A01_1_E07_A	59	49	LMG 22735	Finegoldia	magna
Z0016_RO_A02_1_E07_B	59	49	LMG 22735	Finegoldia	magna
Z0016_RO_A03_1_E08_A	59	49	LMG 22735	Finegoldia	magna
Z0016_RO_A04_1_E08_B	59	49	LMG 22735	Finegoldia	magna
Z0016_LO_A10_1_E05_B	60	50	LMG 23003	Rhodanobacter	fulvus
Z0016_LO_A11_1_E06_A	60	50	LMG 23003	Rhodanobacter	fulvus
Z0016_LO_A12_1_E06_B	60	50	LMG 23003	Rhodanobacter	fulvus
Z0016_LO_B01_2_E01_A	60	50	LMG 23003	Rhodanobacter	fulvus
Z0016_LO_B02_2_E01_B	60	50	LMG 23003	Rhodanobacter	fulvus
Z0016_LO_B03_2_E02_A	60	50	LMG 23003	Rhodanobacter	fulvus
Z0016_LO_B04_2_E02_B	60	50	LMG 23003	Rhodanobacter	fulvus
Z0016_LO_B05_2_E03_A	60	50	LMG 23003	Rhodanobacter	fulvus
Z0016_LO_B06_2_E03_B	60	50	LMG 23003	Rhodanobacter	fulvus
Z0016_LO_B07_2_E04_A	60	50	LMG 23003	Rhodanobacter	fulvus
Z0016_LO_B08_2_E04_B	60	50	LMG 23003	Rhodanobacter	fulvus
Z0016_LO_B09_2_E05_A	60	50	LMG 23003	Rhodanobacter	fulvus
Z0016_LO_B10_2_E05_B	60	50	LMG 23003	Rhodanobacter	fulvus
Z0016_LO_B11_2_E06_A	60	50	LMG 23003	Rhodanobacter	fulvus
Z0016_LO_B12_2_E06_B	60	50	LMG 23003	Rhodanobacter	fulvus
Z0016_LO_C01_1_F01_A	60	50	LMG 23003	Rhodanobacter	fulvus
Z0016_LO_C02_1_F01_B	60	50	LMG 23003	Rhodanobacter	fulvus
Z0016_RO_A05_1_E09_A	60	50	LMG 23003	Rhodanobacter	fulvus
Z0016_RO_A06_1_E09_B	60	50	LMG 23003	Rhodanobacter	fulvus
Z0016_RO_A07_1_E10_A	60	50	LMG 23003	Rhodanobacter	fulvus
Z0016_RO_A08_1_E10_B	60	50	LMG 23003	Rhodanobacter	fulvus

Z0016_RO_A09_1_E11_A	60	50	LMG 23003	Rhodanobacter	fulvus
Z0016_RO_A11_1_E12_A	60	50	LMG 23003	Rhodanobacter	fulvus
Z0016_RO_A12_1_E12_B	60	50	LMG 23003	Rhodanobacter	fulvus
Z0016_RO_B03_2_E08_A	60	50	LMG 23003	Rhodanobacter	fulvus
Z0016_RO_B04_2_E08_B	60	50	LMG 23003	Rhodanobacter	fulvus
Z0016_RO_B05_2_E09_A	60	50	LMG 23003	Rhodanobacter	fulvus
Z0016_RO_B06_2_E09_B	60	50	LMG 23003	Rhodanobacter	fulvus
Z0016_LO_C03_1_F02_A	61	51	LMG 23037	Geobacillus	toebii
Z0016_LO_C04_1_F02_B	61	51	LMG 23037	Geobacillus	toebii
Z0016_LO_C05_1_F03_A	61	51	LMG 23037	Geobacillus	toebii
Z0016_LO_C07_1_F04_A	61	51	LMG 23037	Geobacillus	toebii
Z0016_LO_C09_1_F05_A	61	51	LMG 23037	Geobacillus	toebii
Z0016_LO_C10_1_F05_B	61	51	LMG 23037	Geobacillus	toebii
Z0016_LO_D03_2_F02_A	61	51	LMG 23037	Geobacillus	toebii
Z0016_LO_D05_2_F03_A	61	51	LMG 23037	Geobacillus	toebii
Z0016_LO_D06_2_F03_B	61	51	LMG 23037	Geobacillus	toebii
Z0016_LO_D07_2_F04_A	61	51	LMG 23037	Geobacillus	toebii
Z0016_LO_D11_2_F06_A	61	51	LMG 23037	Geobacillus	toebii
Z0016_LO_D12_2_F06_B	61	51	LMG 23037	Geobacillus	toebii
Z0016_LO_E01_1_G01_A	61	51	LMG 23037	Geobacillus	toebii
Z0016_RO_B08_2_E10_B	61	51	LMG 23037	Geobacillus	toebii
Z0016_RO_B09_2_E11_A	61	51	LMG 23037	Geobacillus	toebii
Z0016_RO_B10_2_E11_B	61	51	LMG 23037	Geobacillus	toebii
Z0016_RO_B11_2_E12_A	61	51	LMG 23037	Geobacillus	toebii
Z0016_RO_B12_2_E12_B	61	51	LMG 23037	Geobacillus	toebii
Z0016_RO_C03_1_F08_A	61	51	LMG 23037	Geobacillus	toebii
Z0016_RO_C04_1_F08_B	61	51	LMG 23037	Geobacillus	toebii
Z0016_RO_C06_1_F09_B	61	51	LMG 23037	Geobacillus	toebii
Z0016_RO_C08_1_F10_B	61	51	LMG 23037	Geobacillus	toebii
Z0016_RO_C09_1_F11_A	61	51	LMG 23037	Geobacillus	toebii
Z0016_RO_C10_1_F11_B	61	51	LMG 23037	Geobacillus	toebii
Z0016_RO_C11_1_F12_A	61	51	LMG 23037	Geobacillus	toebii
Z0016_RO_C12_1_F12_B	61	51	LMG 23037	Geobacillus	toebii
Z0016_RO_D01_2_F07_A	61	51	LMG 23037	Geobacillus	toebii
Z0016_RO_D03_2_F08_A	61	51	LMG 23037	Geobacillus	toebii
Z0016_RO_D06_2_F09_B	61	51	LMG 23037	Geobacillus	toebii
Z0016_RO_D07_2_F10_A	61	51	LMG 23037	Geobacillus	toebii
Z0016_RO_D08_2_F10_B	61	51	LMG 23037	Geobacillus	toebii
Z0016_RO_D09_2_F11_A	61	51	LMG 23037	Geobacillus	toebii
Z0016_LO_C11_1_F06_A	62	54	LMG 23083	Asaia	krungthepensis
Z0016_LO_C12_1_F06_B	62	54	LMG 23083	Asaia	krungthepensis
Z0016_LO_D01_2_F01_A	62	54	LMG 23083	Asaia	krungthepensis
Z0016_LO_D02_2_F01_B	62	54	LMG 23083	Asaia	krungthepensis
Z0016_LO_D08_2_F04_B	62	54	LMG 23083	Asaia	krungthepensis
Z0016_LO_D09_2_F05_A	62	54	LMG 23083	Asaia	krungthepensis
Z0016_LO_D10_2_F05_B	62	54	LMG 23083	Asaia	krungthepensis
Z0016_LO_E06_1_G03_B	62	54	LMG 23083	Asaia	krungthepensis
Z0016_LO_E07_1_G04_A	62	54	LMG 23083	Asaia	krungthepensis
Z0016_LO_E09_1_G05_A	62	54	LMG 23083	Asaia	krungthepensis
Z0016_LO_E12_1_G06_B	62	54	LMG 23083	Asaia	krungthepensis

Z0016_LO_F02_2_G01_B	62	54	LMG 23083	Asaia	krungthepensis
Z0016_LO_F04_2_G02_B	62	54	LMG 23083	Asaia	krungthepensis
Z0016_LO_F05_2_G03_A	62	54	LMG 23083	Asaia	krungthepensis
Z0016_LO_F08_2_G04_B	62	54	LMG 23083	Asaia	krungthepensis
Z0016_LO_F10_2_G05_B	62	54	LMG 23083	Asaia	krungthepensis
Z0016_LO_F11_2_G06_A	62	54	LMG 23083	Asaia	krungthepensis
Z0016_RO_A10_1_E11_B	62	54	LMG 23083	Asaia	krungthepensis
Z0016_RO_B01_2_E07_A	62	54	LMG 23083	Asaia	krungthepensis
Z0016_RO_B02_2_E07_B	62	54	LMG 23083	Asaia	krungthepensis
Z0016_RO_C01_1_F07_A	62	54	LMG 23083	Asaia	krungthepensis
Z0016_RO_C02_1_F07_B	62	54	LMG 23083	Asaia	krungthepensis
Z0016_RO_C07_1_F10_A	62	54	LMG 23083	Asaia	krungthepensis
Z0016_RO_D04_2_F08_B	62	54	LMG 23083	Asaia	krungthepensis
Z0016_RO_D05_2_F09_A	62	54	LMG 23083	Asaia	krungthepensis
Z0016_RO_D10_2_F11_B	62	54	LMG 23083	Asaia	krungthepensis
Z0016_RO_D11_2_F12_A	62	54	LMG 23083	Asaia	krungthepensis
Z0016_RO_D12_2_F12_B	62	54	LMG 23083	Asaia	krungthepensis
Z0016_RO_E01_1_G07_A	62	54	LMG 23083	Asaia	krungthepensis
Z0016_RO_E02_1_G07_B	62	54	LMG 23083	Asaia	krungthepensis
Z0016_RO_E09_1_G11_A	62	54	LMG 23083	Asaia	krungthepensis
Z0016_RO_F02_2_G07_B	62	54	LMG 23083	Asaia	krungthepensis
Z0016_LO_E02_1_G01_B	63	52	LMG 23059	Blastobacter	aggregatus
Z0016_LO_E03_1_G02_A	63	52	LMG 23059	Blastobacter	aggregatus
Z0016_LO_E04_1_G02_B	63	52	LMG 23059	Blastobacter	aggregatus
Z0016_LO_E05_1_G03_A	63	52	LMG 23059	Blastobacter	aggregatus
Z0016_LO_E08_1_G04_B	63	52	LMG 23059	Blastobacter	aggregatus
Z0016_LO_E10_1_G05_B	63	52	LMG 23059	Blastobacter	aggregatus
Z0016_LO_E11_1_G06_A	63	52	LMG 23059	Blastobacter	aggregatus
Z0016_LO_F01_2_G01_A	63	52	LMG 23059	Blastobacter	aggregatus
Z0016_LO_F03_2_G02_A	63	52	LMG 23059	Blastobacter	aggregatus
Z0016_LO_F06_2_G03_B	63	52	LMG 23059	Blastobacter	aggregatus
Z0016_LO_F07_2_G04_A	63	52	LMG 23059	Blastobacter	aggregatus
Z0016_LO_F09_2_G05_A	63	52	LMG 23059	Blastobacter	aggregatus
Z0016_LO_F12_2_G06_B	63	52	LMG 23059	Blastobacter	aggregatus
Z0016_LO_G01_1_H01_A	63	52	LMG 23059	Blastobacter	aggregatus
Z0016_LO_G02_1_H01_B	63	52	LMG 23059	Blastobacter	aggregatus
Z0016_LO_G04_1_H02_B	63	52	LMG 23059	Blastobacter	aggregatus
Z0016_RO_E03_1_G08_A	63	52	LMG 23059	Blastobacter	aggregatus
Z0016_RO_E04_1_G08_B	63	52	LMG 23059	Blastobacter	aggregatus
Z0016_RO_E05_1_G09_A	63	52	LMG 23059	Blastobacter	aggregatus
Z0016_RO_E07_1_G10_A	63	52	LMG 23059	Blastobacter	aggregatus
Z0016_RO_E08_1_G10_B	63	52	LMG 23059	Blastobacter	aggregatus
Z0016_RO_E10_1_G11_B	63	52	LMG 23059	Blastobacter	aggregatus
Z0016_RO_E11_1_G12_A	63	52	LMG 23059	Blastobacter	aggregatus
Z0016_RO_F01_2_G07_A	63	52	LMG 23059	Blastobacter	aggregatus
Z0016_RO_F03_2_G08_A	63	52	LMG 23059	Blastobacter	aggregatus
Z0016_RO_F04_2_G08_B	63	52	LMG 23059	Blastobacter	aggregatus
Z0016_RO_F07_2_G10_A	63	52	LMG 23059	Blastobacter	aggregatus
Z0016_RO_F09_2_G11_A	63	52	LMG 23059	Blastobacter	aggregatus
Z0016_RO_F10_2_G11_B	63	52	LMG 23059	Blastobacter	aggregatus

Z0016_RO_G02_1_H07_B	63	52	LMG 23059	Blastobacter	aggregatus	
Z0016_RO_G04_1_H08_B	63	52	LMG 23059	Blastobacter	aggregatus	
Z0016_RO_G05_1_H09_A	63	52	LMG 23059	Blastobacter	aggregatus	
Z0016_LO_G03_1_H02_A	64	53	LMG 23078	Streptomyces	albus	albus
Z0016_LO_G05_1_H03_A	64	53	LMG 23078	Streptomyces	albus	albus
Z0016_LO_G06_1_H03_B	64	53	LMG 23078	Streptomyces	albus	albus
Z0016_LO_G07_1_H04_A	64	53	LMG 23078	Streptomyces	albus	albus
Z0016_LO_G08_1_H04_B	64	53	LMG 23078	Streptomyces	albus	albus
Z0016_LO_G09_1_H05_A	64	53	LMG 23078	Streptomyces	albus	albus
Z0016_LO_G10_1_H05_B	64	53	LMG 23078	Streptomyces	albus	albus
Z0016_LO_G11_1_H06_A	64	53	LMG 23078	Streptomyces	albus	albus
Z0016_LO_G12_1_H06_B	64	53	LMG 23078	Streptomyces	albus	albus
Z0016_LO_H01_2_H01_A	64	53	LMG 23078	Streptomyces	albus	albus
Z0016_LO_H02_2_H01_B	64	53	LMG 23078	Streptomyces	albus	albus
Z0016_LO_H03_2_H02_A	64	53	LMG 23078	Streptomyces	albus	albus
Z0016_LO_H04_2_H02_B	64	53	LMG 23078	Streptomyces	albus	albus
Z0016_LO_H05_2_H03_A	64	53	LMG 23078	Streptomyces	albus	albus
Z0016_RO_F05_2_G09_A	64	53	LMG 23078	Streptomyces	albus	albus
Z0016_RO_F06_2_G09_B	64	53	LMG 23078	Streptomyces	albus	albus
Z0016_RO_F08_2_G10_B	64	53	LMG 23078	Streptomyces	albus	albus
Z0016_RO_F11_2_G12_A	64	53	LMG 23078	Streptomyces	albus	albus
Z0016_RO_F12_2_G12_B	64	53	LMG 23078	Streptomyces	albus	albus
Z0016_RO_G01_1_H07_A	64	53	LMG 23078	Streptomyces	albus	albus
Z0016_RO_G03_1_H08_A	64	53	LMG 23078	Streptomyces	albus	albus
Z0016_RO_G06_1_H09_B	64	53	LMG 23078	Streptomyces	albus	albus
Z0016_RO_G07_1_H10_A	64	53	LMG 23078	Streptomyces	albus	albus
Z0016_RO_G08_1_H10_B	64	53	LMG 23078	Streptomyces	albus	albus
Z0016_RO_G09_1_H11_A	64	53	LMG 23078	Streptomyces	albus	albus
Z0016_RO_G10_1_H11_B	64	53	LMG 23078	Streptomyces	albus	albus
Z0016_RO_G11_1_H12_A	64	53	LMG 23078	Streptomyces	albus	albus
Z0016_RO_G12_1_H12_B	64	53	LMG 23078	Streptomyces	albus	albus
Z0016_RO_H01_2_H07_A	64	53	LMG 23078	Streptomyces	albus	albus
Z0016_RO_H02_2_H07_B	64	53	LMG 23078	Streptomyces	albus	albus
Z0016_RO_H03_2_H08_A	64	53	LMG 23078	Streptomyces	albus	albus
Z0016_RO_H04_2_H08_B	64	53	LMG 23078	Streptomyces	albus	albus
Z0017_LB_A01_2_D06_A	65	55	LMG 23170	Saccharibacter	floricola	
Z0017_LB_A02_2_D06_B	65	55	LMG 23170	Saccharibacter	floricola	
Z0017_LB_A05_2_D04_A	65	55	LMG 23170	Saccharibacter	floricola	
Z0017_LB_A06_2_D04_B	65	55	LMG 23170	Saccharibacter	floricola	
Z0017_LB_A07_2_D03_A	65	55	LMG 23170	Saccharibacter	floricola	
Z0017_LB_A08_2_D03_B	65	55	LMG 23170	Saccharibacter	floricola	
Z0017_LB_A09_2_D02_A	65	55	LMG 23170	Saccharibacter	floricola	
Z0017_LB_A10_2_D02_B	65	55	LMG 23170	Saccharibacter	floricola	
Z0017_LB_A11_2_D01_A	65	55	LMG 23170	Saccharibacter	floricola	
Z0017_LB_A12_2_D01_B	65	55	LMG 23170	Saccharibacter	floricola	
Z0017_LB_B01_1_D06_A	65	55	LMG 23170	Saccharibacter	floricola	
Z0017_LO_B10_2_E05_B	65	55	LMG 23170	Saccharibacter	floricola	
Z0017_LO_G10_1_H05_B	65	55	LMG 23170	Saccharibacter	floricola	
Z0017_LO_G11_1_H06_A	65	55	LMG 23170	Saccharibacter	floricola	
Z0017_LO_G12_1_H06_B	65	55	LMG 23170	Saccharibacter	floricola	

Z0017_LO_H01_2_H01_A	65	55	LMG 23170	Saccharibacter	floricola
Z0017_LO_H02_2_H01_B	65	55	LMG 23170	Saccharibacter	floricola
Z0017_LO_H05_2_H03_A	65	55	LMG 23170	Saccharibacter	floricola
Z0017_LO_H06_2_H03_B	65	55	LMG 23170	Saccharibacter	floricola
Z0017_LO_H07_2_H04_A	65	55	LMG 23170	Saccharibacter	floricola
Z0017_LO_H08_2_H04_B	65	55	LMG 23170	Saccharibacter	floricola
Z0017_LO_H09_2_H05_A	65	55	LMG 23170	Saccharibacter	floricola
Z0017_LO_H10_2_H05_B	65	55	LMG 23170	Saccharibacter	floricola
Z0017_LO_H11_2_H06_A	65	55	LMG 23170	Saccharibacter	floricola
Z0017_RB_H10_1_A08_B	65	55	LMG 23170	Saccharibacter	floricola
Z0017_RO_A06_1_E09_B	65	55	LMG 23170	Saccharibacter	floricola
Z0017_RO_A10_1_E11_B	65	55	LMG 23170	Saccharibacter	floricola
Z0017_RO_A12_1_E12_B	65	55	LMG 23170	Saccharibacter	floricola
Z0017_LB_B02_1_D06_B	66	56	LMG 23381	Simplicispira	metamorpha
Z0017_LB_B03_1_D05_A	66	56	LMG 23381	Simplicispira	metamorpha
Z0017_LB_B04_1_D05_B	66	56	LMG 23381	Simplicispira	metamorpha
Z0017_LB_B05_1_D04_A	66	56	LMG 23381	Simplicispira	metamorpha
Z0017_LB_B06_1_D04_B	66	56	LMG 23381	Simplicispira	metamorpha
Z0017_LB_B07_1_D03_A	66	56	LMG 23381	Simplicispira	metamorpha
Z0017_LB_B08_1_D03_B	66	56	LMG 23381	Simplicispira	metamorpha
Z0017_LB_B09_1_D02_A	66	56	LMG 23381	Simplicispira	metamorpha
Z0017_LB_B10_1_D02_B	66	56	LMG 23381	Simplicispira	metamorpha
Z0017_LB_B11_1_D01_A	66	56	LMG 23381	Simplicispira	metamorpha
Z0017_LB_B12_1_D01_B	66	56	LMG 23381	Simplicispira	metamorpha
Z0017_LB_C01_2_C06_A	66	56	LMG 23381	Simplicispira	metamorpha
Z0017_LB_C02_2_C06_B	66	56	LMG 23381	Simplicispira	metamorpha
Z0017_LB_C03_2_C05_A	66	56	LMG 23381	Simplicispira	metamorpha
Z0017_LB_C04_2_C05_B	66	56	LMG 23381	Simplicispira	metamorpha
Z0017_LB_C05_2_C04_A	66	56	LMG 23381	Simplicispira	metamorpha
Z0017_LB_C06_2_C04_B	66	56	LMG 23381	Simplicispira	metamorpha
Z0017_LO_H12_2_H06_B	66	56	LMG 23381	Simplicispira	metamorpha
Z0017_RB_A01_2_D12_A	66	56	LMG 23381	Simplicispira	metamorpha
Z0017_RB_A02_2_D12_B	66	56	LMG 23381	Simplicispira	metamorpha
Z0017_RB_A03_2_D11_A	66	56	LMG 23381	Simplicispira	metamorpha
Z0017_RB_A04_2_D11_B	66	56	LMG 23381	Simplicispira	metamorpha
Z0017_RB_A05_2_D10_A	66	56	LMG 23381	Simplicispira	metamorpha
Z0017_RB_A06_2_D10_B	66	56	LMG 23381	Simplicispira	metamorpha
Z0017_RB_A07_2_D09_A	66	56	LMG 23381	Simplicispira	metamorpha
Z0017_RB_A08_2_D09_B	66	56	LMG 23381	Simplicispira	metamorpha
Z0017_RB_A09_2_D08_A	66	56	LMG 23381	Simplicispira	metamorpha
Z0017_RB_A10_2_D08_B	66	56	LMG 23381	Simplicispira	metamorpha
Z0017_RB_A11_2_D07_A	66	56	LMG 23381	Simplicispira	metamorpha
Z0017_RB_A12_2_D07_B	66	56	LMG 23381	Simplicispira	metamorpha
Z0017_RB_B01_1_D12_A	66	56	LMG 23381	Simplicispira	metamorpha
Z0017_RB_B02_1_D12_B	66	56	LMG 23381	Simplicispira	metamorpha
Z0017_LB_C07_2_C03_A	67	57	LMG 23383	Lactococcus	piscium
Z0017_LB_C08_2_C03_B	67	57	LMG 23383	Lactococcus	piscium
Z0017_LB_C09_2_C02_A	67	57	LMG 23383	Lactococcus	piscium
Z0017_LB_C10_2_C02_B	67	57	LMG 23383	Lactococcus	piscium
Z0017_LB_C11_2_C01_A	67	57	LMG 23383	Lactococcus	piscium

Z0017_LB_C12_2_C01_B	67	57	LMG 23383	Lactococcus	piscium
Z0017_LB_D01_1_C06_A	67	57	LMG 23383	Lactococcus	piscium
Z0017_LB_D02_1_C06_B	67	57	LMG 23383	Lactococcus	piscium
Z0017_LB_D03_1_C05_A	67	57	LMG 23383	Lactococcus	piscium
Z0017_LB_D04_1_C05_B	67	57	LMG 23383	Lactococcus	piscium
Z0017_LB_D05_1_C04_A	67	57	LMG 23383	Lactococcus	piscium
Z0017_LB_D06_1_C04_B	67	57	LMG 23383	Lactococcus	piscium
Z0017_LB_D07_1_C03_A	67	57	LMG 23383	Lactococcus	piscium
Z0017_RB_B03_1_D11_A	67	57	LMG 23383	Lactococcus	piscium
Z0017_RB_B04_1_D11_B	67	57	LMG 23383	Lactococcus	piscium
Z0017_RB_B05_1_D10_A	67	57	LMG 23383	Lactococcus	piscium
Z0017_RB_B06_1_D10_B	67	57	LMG 23383	Lactococcus	piscium
Z0017_RB_B07_1_D09_A	67	57	LMG 23383	Lactococcus	piscium
Z0017_RB_B08_1_D09_B	67	57	LMG 23383	Lactococcus	piscium
Z0017_RB_B09_1_D08_A	67	57	LMG 23383	Lactococcus	piscium
Z0017_RB_B10_1_D08_B	67	57	LMG 23383	Lactococcus	piscium
Z0017_RB_B11_1_D07_A	67	57	LMG 23383	Lactococcus	piscium
Z0017_RB_B12_1_D07_B	67	57	LMG 23383	Lactococcus	piscium
Z0017_RB_C01_2_C12_A	67	57	LMG 23383	Lactococcus	piscium
Z0017_RB_C02_2_C12_B	67	57	LMG 23383	Lactococcus	piscium
Z0017_RB_C03_2_C11_A	67	57	LMG 23383	Lactococcus	piscium
Z0017_RB_C04_2_C11_B	67	57	LMG 23383	Lactococcus	piscium
Z0017_RB_C05_2_C10_A	67	57	LMG 23383	Lactococcus	piscium
Z0017_RB_C06_2_C10_B	67	57	LMG 23383	Lactococcus	piscium
Z0017_RB_C07_2_C09_A	67	57	LMG 23383	Lactococcus	piscium
Z0017_RB_C08_2_C09_B	67	57	LMG 23383	Lactococcus	piscium
Z0017_RB_C09_2_C08_A	67	57	LMG 23383	Lactococcus	piscium
Z0017_LB_D08_1_C03_B	68	58	LMG 23655	Carnobacterium	inhibens
Z0017_LB_D09_1_C02_A	68	58	LMG 23655	Carnobacterium	inhibens
Z0017_LB_D10_1_C02_B	68	58	LMG 23655	Carnobacterium	inhibens
Z0017_LB_D11_1_C01_A	68	58	LMG 23655	Carnobacterium	inhibens
Z0017_LB_D12_1_C01_B	68	58	LMG 23655	Carnobacterium	inhibens
Z0017_LB_E01_2_B06_A	68	58	LMG 23655	Carnobacterium	inhibens
Z0017_LB_E02_2_B06_B	68	58	LMG 23655	Carnobacterium	inhibens
Z0017_LB_E03_2_B05_A	68	58	LMG 23655	Carnobacterium	inhibens
Z0017_LB_E04_2_B05_B	68	58	LMG 23655	Carnobacterium	inhibens
Z0017_LB_E05_2_B04_A	68	58	LMG 23655	Carnobacterium	inhibens
Z0017_LB_E06_2_B04_B	68	58	LMG 23655	Carnobacterium	inhibens
Z0017_LB_E07_2_B03_A	68	58	LMG 23655	Carnobacterium	inhibens
Z0017_LB_E08_2_B03_B	68	58	LMG 23655	Carnobacterium	inhibens
Z0017_RB_C10_2_C08_B	68	58	LMG 23655	Carnobacterium	inhibens
Z0017_RB_C11_2_C07_A	68	58	LMG 23655	Carnobacterium	inhibens
Z0017_RB_C12_2_C07_B	68	58	LMG 23655	Carnobacterium	inhibens
Z0017_RB_D01_1_C12_A	68	58	LMG 23655	Carnobacterium	inhibens
Z0017_RB_D02_1_C12_B	68	58	LMG 23655	Carnobacterium	inhibens
Z0017_RB_D03_1_C11_A	68	58	LMG 23655	Carnobacterium	inhibens
Z0017_RB_D04_1_C11_B	68	58	LMG 23655	Carnobacterium	inhibens
Z0017_RB_D05_1_C10_A	68	58	LMG 23655	Carnobacterium	inhibens
Z0017_RB_D06_1_C10_B	68	58	LMG 23655	Carnobacterium	inhibens
Z0017_RB_D07_1_C09_A	68	58	LMG 23655	Carnobacterium	inhibens

Z0017_RB_D08_1_C09_B	68	58	LMG 23655	Carnobacterium	inhibens
Z0017_RB_D09_1_C08_A	68	58	LMG 23655	Carnobacterium	inhibens
Z0017_RB_D10_1_C08_B	68	58	LMG 23655	Carnobacterium	inhibens
Z0017_RB_D11_1_C07_A	68	58	LMG 23655	Carnobacterium	inhibens
Z0017_RB_D12_1_C07_B	68	58	LMG 23655	Carnobacterium	inhibens
Z0017_RB_E01_2_B12_A	68	58	LMG 23655	Carnobacterium	inhibens
Z0017_RB_E02_2_B12_B	68	58	LMG 23655	Carnobacterium	inhibens
Z0017_RB_E03_2_B11_A	68	58	LMG 23655	Carnobacterium	inhibens
Z0017_RB_E04_2_B11_B	68	58	LMG 23655	Carnobacterium	inhibens
Z0017_LB_E09_2_B02_A	69	59	LMG 23818	Rheinheimera	chironomi
Z0017_LB_E10_2_B02_B	69	59	LMG 23818	Rheinheimera	chironomi
Z0017_LB_E11_2_B01_A	69	59	LMG 23818	Rheinheimera	chironomi
Z0017_LB_E12_2_B01_B	69	59	LMG 23818	Rheinheimera	chironomi
Z0017_LB_F01_1_B06_A	69	59	LMG 23818	Rheinheimera	chironomi
Z0017_LB_F02_1_B06_B	69	59	LMG 23818	Rheinheimera	chironomi
Z0017_LB_F03_1_B05_A	69	59	LMG 23818	Rheinheimera	chironomi
Z0017_LB_F04_1_B05_B	69	59	LMG 23818	Rheinheimera	chironomi
Z0017_LB_F05_1_B04_A	69	59	LMG 23818	Rheinheimera	chironomi
Z0017_LB_F06_1_B04_B	69	59	LMG 23818	Rheinheimera	chironomi
Z0017_LB_F07_1_B03_A	69	59	LMG 23818	Rheinheimera	chironomi
Z0017_LB_F08_1_B03_B	69	59	LMG 23818	Rheinheimera	chironomi
Z0017_LB_F09_1_B02_A	69	59	LMG 23818	Rheinheimera	chironomi
Z0017_LB_F10_1_B02_B	69	59	LMG 23818	Rheinheimera	chironomi
Z0017_RB_E05_2_B10_A	69	59	LMG 23818	Rheinheimera	chironomi
Z0017_RB_E06_2_B10_B	69	59	LMG 23818	Rheinheimera	chironomi
Z0017_RB_E07_2_B09_A	69	59	LMG 23818	Rheinheimera	chironomi
Z0017_RB_E08_2_B09_B	69	59	LMG 23818	Rheinheimera	chironomi
Z0017_RB_E09_2_B08_A	69	59	LMG 23818	Rheinheimera	chironomi
Z0017_RB_E10_2_B08_B	69	59	LMG 23818	Rheinheimera	chironomi
Z0017_RB_E11_2_B07_A	69	59	LMG 23818	Rheinheimera	chironomi
Z0017_RB_E12_2_B07_B	69	59	LMG 23818	Rheinheimera	chironomi
Z0017_RB_F01_1_B12_A	69	59	LMG 23818	Rheinheimera	chironomi
Z0017_RB_F02_1_B12_B	69	59	LMG 23818	Rheinheimera	chironomi
Z0017_RB_F03_1_B11_A	69	59	LMG 23818	Rheinheimera	chironomi
Z0017_RB_F04_1_B11_B	69	59	LMG 23818	Rheinheimera	chironomi
Z0017_RB_F05_1_B10_A	69	59	LMG 23818	Rheinheimera	chironomi
Z0017_RB_F06_1_B10_B	69	59	LMG 23818	Rheinheimera	chironomi
Z0017_RB_F07_1_B09_A	69	59	LMG 23818	Rheinheimera	chironomi
Z0017_RB_F08_1_B09_B	69	59	LMG 23818	Rheinheimera	chironomi
Z0017_RB_F09_1_B08_A	69	59	LMG 23818	Rheinheimera	chironomi
Z0017_RB_F10_1_B08_B	69	59	LMG 23818	Rheinheimera	chironomi
Z0017_LB_F11_1_B01_A	70	60	LMG 23835	Marinobacter	algicola
Z0017_LB_F12_1_B01_B	70	60	LMG 23835	Marinobacter	algicola
Z0017_LB_G01_2_A06_A	70	60	LMG 23835	Marinobacter	algicola
Z0017_LB_G02_2_A06_B	70	60	LMG 23835	Marinobacter	algicola
Z0017_LB_G03_2_A05_A	70	60	LMG 23835	Marinobacter	algicola
Z0017_LB_G04_2_A05_B	70	60	LMG 23835	Marinobacter	algicola
Z0017_LB_G05_2_A04_A	70	60	LMG 23835	Marinobacter	algicola
Z0017_LB_G06_2_A04_B	70	60	LMG 23835	Marinobacter	algicola
Z0017_LB_G07_2_A03_A	70	60	LMG 23835	Marinobacter	algicola

Z0017_LB_G08_2_A03_B	70	60	LMG 23835	Marinobacter	algicola
Z0017_LB_G09_2_A02_A	70	60	LMG 23835	Marinobacter	algicola
Z0017_LB_G10_2_A02_B	70	60	LMG 23835	Marinobacter	algicola
Z0017_LB_G11_2_A01_A	70	60	LMG 23835	Marinobacter	algicola
Z0017_LB_G12_2_A01_B	70	60	LMG 23835	Marinobacter	algicola
Z0017_LB_H01_1_A06_A	70	60	LMG 23835	Marinobacter	algicola
Z0017_RB_F11_1_B07_A	70	60	LMG 23835	Marinobacter	algicola
Z0017_RB_F12_1_B07_B	70	60	LMG 23835	Marinobacter	algicola
Z0017_RB_G01_2_A12_A	70	60	LMG 23835	Marinobacter	algicola
Z0017_RB_G02_2_A12_B	70	60	LMG 23835	Marinobacter	algicola
Z0017_RB_G03_2_A11_A	70	60	LMG 23835	Marinobacter	algicola
Z0017_RB_G04_2_A11_B	70	60	LMG 23835	Marinobacter	algicola
Z0017_RB_G05_2_A10_A	70	60	LMG 23835	Marinobacter	algicola
Z0017_RB_G06_2_A10_B	70	60	LMG 23835	Marinobacter	algicola
Z0017_RB_G07_2_A09_A	70	60	LMG 23835	Marinobacter	algicola
Z0017_RB_G08_2_A09_B	70	60	LMG 23835	Marinobacter	algicola
Z0017_RB_G09_2_A08_A	70	60	LMG 23835	Marinobacter	algicola
Z0017_RB_G10_2_A08_B	70	60	LMG 23835	Marinobacter	algicola
Z0017_RB_G11_2_A07_A	70	60	LMG 23835	Marinobacter	algicola
Z0017_RB_G12_2_A07_B	70	60	LMG 23835	Marinobacter	algicola
Z0017_RB_H01_1_A12_A	70	60	LMG 23835	Marinobacter	algicola
Z0017_RB_H02_1_A12_B	70	60	LMG 23835	Marinobacter	algicola
Z0017_RB_H03_1_A11_A	70	60	LMG 23835	Marinobacter	algicola
Z0017_LB_H02_1_A06_B	71	61	LMG 23965	Collimonas	pratensis
Z0017_LB_H03_1_A05_A	71	61	LMG 23965	Collimonas	pratensis
Z0017_LB_H04_1_A05_B	71	61	LMG 23965	Collimonas	pratensis
Z0017_LB_H05_1_A04_A	71	61	LMG 23965	Collimonas	pratensis
Z0017_LB_H06_1_A04_B	71	61	LMG 23965	Collimonas	pratensis
Z0017_LB_H07_1_A03_A	71	61	LMG 23965	Collimonas	pratensis
Z0017_LB_H08_1_A03_B	71	61	LMG 23965	Collimonas	pratensis
Z0017_LB_H09_1_A02_A	71	61	LMG 23965	Collimonas	pratensis
Z0017_LB_H10_1_A02_B	71	61	LMG 23965	Collimonas	pratensis
Z0017_LB_H11_1_A01_A	71	61	LMG 23965	Collimonas	pratensis
Z0017_LO_A02_1_E01_B	71	61	LMG 23965	Collimonas	pratensis
Z0017_LO_A05_1_E03_A	71	61	LMG 23965	Collimonas	pratensis
Z0017_LO_A06_1_E03_B	71	61	LMG 23965	Collimonas	pratensis
Z0017_RB_H04_1_A11_B	71	61	LMG 23965	Collimonas	pratensis
Z0017_RB_H05_1_A10_A	71	61	LMG 23965	Collimonas	pratensis
Z0017_RB_H06_1_A10_B	71	61	LMG 23965	Collimonas	pratensis
Z0017_RB_H07_1_A09_A	71	61	LMG 23965	Collimonas	pratensis
Z0017_RB_H08_1_A09_B	71	61	LMG 23965	Collimonas	pratensis
Z0017_RB_H11_1_A07_A	71	61	LMG 23965	Collimonas	pratensis
Z0017_RO_A02_1_E07_B	71	61	LMG 23965	Collimonas	pratensis
Z0017_RO_A03_1_E08_A	71	61	LMG 23965	Collimonas	pratensis
Z0017_RO_A04_1_E08_B	71	61	LMG 23965	Collimonas	pratensis
Z0017_RO_A05_1_E09_A	71	61	LMG 23965	Collimonas	pratensis
Z0017_RO_A07_1_E10_A	71	61	LMG 23965	Collimonas	pratensis
Z0017_RO_A08_1_E10_B	71	61	LMG 23965	Collimonas	pratensis
Z0017_RO_A09_1_E11_A	71	61	LMG 23965	Collimonas	pratensis
Z0017_RO_A11_1_E12_A	71	61	LMG 23965	Collimonas	pratensis

Z0017_RO_B01_2_E07_A	71	61	LMG 23965	Collimonas	pratensis
Z0017_LB_H12_1_A01_B	72	66	LMG 24163	Pseudoxanthomonas	dokdonensis
Z0017_LO_A01_1_E01_A	72	66	LMG 24163	Pseudoxanthomonas	dokdonensis
Z0017_LO_A03_1_E02_A	72	66	LMG 24163	Pseudoxanthomonas	dokdonensis
Z0017_LO_A04_1_E02_B	72	66	LMG 24163	Pseudoxanthomonas	dokdonensis
Z0017_LO_A07_1_E04_A	72	66	LMG 24163	Pseudoxanthomonas	dokdonensis
Z0017_LO_B01_2_E01_A	72	66	LMG 24163	Pseudoxanthomonas	dokdonensis
Z0017_LO_B02_2_E01_B	72	66	LMG 24163	Pseudoxanthomonas	dokdonensis
Z0017_LO_B04_2_E02_B	72	66	LMG 24163	Pseudoxanthomonas	dokdonensis
Z0017_LO_B05_2_E03_A	72	66	LMG 24163	Pseudoxanthomonas	dokdonensis
Z0017_LO_B06_2_E03_B	72	66	LMG 24163	Pseudoxanthomonas	dokdonensis
Z0017_LO_C01_1_F01_A	72	66	LMG 24163	Pseudoxanthomonas	dokdonensis
Z0017_LO_C04_1_F02_B	72	66	LMG 24163	Pseudoxanthomonas	dokdonensis
Z0017_LO_C07_1_F04_A	72	66	LMG 24163	Pseudoxanthomonas	dokdonensis
Z0017_LO_C09_1_F05_A	72	66	LMG 24163	Pseudoxanthomonas	dokdonensis
Z0017_LO_C11_1_F06_A	72	66	LMG 24163	Pseudoxanthomonas	dokdonensis
Z0017_LO_C12_1_F06_B	72	66	LMG 24163	Pseudoxanthomonas	dokdonensis
Z0017_LO_D02_2_F01_B	72	66	LMG 24163	Pseudoxanthomonas	dokdonensis
Z0017_LO_D05_2_F03_A	72	66	LMG 24163	Pseudoxanthomonas	dokdonensis
Z0017_RB_H09_1_A08_A	72	66	LMG 24163	Pseudoxanthomonas	dokdonensis
Z0017_RB_H12_1_A07_B	72	66	LMG 24163	Pseudoxanthomonas	dokdonensis
Z0017_RO_A01_1_E07_A	72	66	LMG 24163	Pseudoxanthomonas	dokdonensis
Z0017_RO_B03_2_E08_A	72	66	LMG 24163	Pseudoxanthomonas	dokdonensis
Z0017_RO_B04_2_E08_B	72	66	LMG 24163	Pseudoxanthomonas	dokdonensis
Z0017_RO_B05_2_E09_A	72	66	LMG 24163	Pseudoxanthomonas	dokdonensis
Z0017_RO_B06_2_E09_B	72	66	LMG 24163	Pseudoxanthomonas	dokdonensis
Z0017_RO_B10_2_E11_B	72	66	LMG 24163	Pseudoxanthomonas	dokdonensis
Z0017_RO_C03_1_F08_A	72	66	LMG 24163	Pseudoxanthomonas	dokdonensis
Z0017_RO_C04_1_F08_B	72	66	LMG 24163	Pseudoxanthomonas	dokdonensis
Z0017_RO_C06_1_F09_B	72	66	LMG 24163	Pseudoxanthomonas	dokdonensis
Z0017_RO_C08_1_F10_B	72	66	LMG 24163	Pseudoxanthomonas	dokdonensis
Z0017_RO_C09_1_F11_A	72	66	LMG 24163	Pseudoxanthomonas	dokdonensis
Z0017_RO_C11_1_F12_A	72	66	LMG 24163	Pseudoxanthomonas	dokdonensis
Z0017_LO_A08_1_E04_B	73	62	LMG 24012	Parapusillimonas	granuli
Z0017_LO_A09_1_E05_A	73	62	LMG 24012	Parapusillimonas	granuli
Z0017_LO_A10_1_E05_B	73	62	LMG 24012	Parapusillimonas	granuli
Z0017_LO_A11_1_E06_A	73	62	LMG 24012	Parapusillimonas	granuli
Z0017_LO_A12_1_E06_B	73	62	LMG 24012	Parapusillimonas	granuli
Z0017_LO_B03_2_E02_A	73	62	LMG 24012	Parapusillimonas	granuli
Z0017_LO_B07_2_E04_A	73	62	LMG 24012	Parapusillimonas	granuli
Z0017_LO_B08_2_E04_B	73	62	LMG 24012	Parapusillimonas	granuli
Z0017_LO_B09_2_E05_A	73	62	LMG 24012	Parapusillimonas	granuli
Z0017_LO_B11_2_E06_A	73	62	LMG 24012	Parapusillimonas	granuli
Z0017_LO_B12_2_E06_B	73	62	LMG 24012	Parapusillimonas	granuli
Z0017_LO_C02_1_F01_B	73	62	LMG 24012	Parapusillimonas	granuli
Z0017_LO_C03_1_F02_A	73	62	LMG 24012	Parapusillimonas	granuli
Z0017_LO_C05_1_F03_A	73	62	LMG 24012	Parapusillimonas	granuli
Z0017_LO_C06_1_F03_B	73	62	LMG 24012	Parapusillimonas	granuli
Z0017_RO_B02_2_E07_B	73	62	LMG 24012	Parapusillimonas	granuli
Z0017_RO_B07_2_E10_A	73	62	LMG 24012	Parapusillimonas	granuli

Z0017_RO_B08_2_E10_B	73	62	LMG 24012	Parapusillimonas	granuli	
Z0017_RO_B09_2_E11_A	73	62	LMG 24012	Parapusillimonas	granuli	
Z0017_RO_B11_2_E12_A	73	62	LMG 24012	Parapusillimonas	granuli	
Z0017_RO_B12_2_E12_B	73	62	LMG 24012	Parapusillimonas	granuli	
Z0017_RO_C01_1_F07_A	73	62	LMG 24012	Parapusillimonas	granuli	
Z0017_RO_C02_1_F07_B	73	62	LMG 24012	Parapusillimonas	granuli	
Z0017_RO_C05_1_F09_A	73	62	LMG 24012	Parapusillimonas	granuli	
Z0017_RO_C07_1_F10_A	73	62	LMG 24012	Parapusillimonas	granuli	
Z0017_RO_C10_1_F11_B	73	62	LMG 24012	Parapusillimonas	granuli	
Z0017_RO_C12_1_F12_B	73	62	LMG 24012	Parapusillimonas	granuli	
Z0017_RO_D01_2_F07_A	73	62	LMG 24012	Parapusillimonas	granuli	
Z0017_LO_C08_1_F04_B	74	63	LMG 24015	Chitinophaga	terrae	
Z0017_LO_C10_1_F05_B	74	63	LMG 24015	Chitinophaga	terrae	
Z0017_LO_D01_2_F01_A	74	63	LMG 24015	Chitinophaga	terrae	
Z0017_LO_D03_2_F02_A	74	63	LMG 24015	Chitinophaga	terrae	
Z0017_LO_D06_2_F03_B	74	63	LMG 24015	Chitinophaga	terrae	
Z0017_LO_D08_2_F04_B	74	63	LMG 24015	Chitinophaga	terrae	
Z0017_LO_D11_2_F06_A	74	63	LMG 24015	Chitinophaga	terrae	
Z0017_LO_D12_2_F06_B	74	63	LMG 24015	Chitinophaga	terrae	
Z0017_LO_E02_1_G01_B	74	63	LMG 24015	Chitinophaga	terrae	
Z0017_LO_E03_1_G02_A	74	63	LMG 24015	Chitinophaga	terrae	
Z0017_LO_E05_1_G03_A	74	63	LMG 24015	Chitinophaga	terrae	
Z0017_LO_E07_1_G04_A	74	63	LMG 24015	Chitinophaga	terrae	
Z0017_LO_E12_1_G06_B	74	63	LMG 24015	Chitinophaga	terrae	
Z0017_LO_F01_2_G01_A	74	63	LMG 24015	Chitinophaga	terrae	
Z0017_LO_F02_2_G01_B	74	63	LMG 24015	Chitinophaga	terrae	
Z0017_RO_D03_2_F08_A	74	63	LMG 24015	Chitinophaga	terrae	
Z0017_RO_D04_2_F08_B	74	63	LMG 24015	Chitinophaga	terrae	
Z0017_RO_D06_2_F09_B	74	63	LMG 24015	Chitinophaga	terrae	
Z0017_RO_D07_2_F10_A	74	63	LMG 24015	Chitinophaga	terrae	
Z0017_RO_D09_2_F11_A	74	63	LMG 24015	Chitinophaga	terrae	
Z0017_RO_D11_2_F12_A	74	63	LMG 24015	Chitinophaga	terrae	
Z0017_RO_D12_2_F12_B	74	63	LMG 24015	Chitinophaga	terrae	
Z0017_RO_E02_1_G07_B	74	63	LMG 24015	Chitinophaga	terrae	
Z0017_RO_E04_1_G08_B	74	63	LMG 24015	Chitinophaga	terrae	
Z0017_RO_E05_1_G09_A	74	63	LMG 24015	Chitinophaga	terrae	
Z0017_RO_E08_1_G10_B	74	63	LMG 24015	Chitinophaga	terrae	
Z0017_RO_E11_1_G12_A	74	63	LMG 24015	Chitinophaga	terrae	
Z0017_RO_F01_2_G07_A	74	63	LMG 24015	Chitinophaga	terrae	
Z0017_RO_F03_2_G08_A	74	63	LMG 24015	Chitinophaga	terrae	
Z0017_RO_F04_2_G08_B	74	63	LMG 24015	Chitinophaga	terrae	
Z0017_RO_F05_2_G09_A	74	63	LMG 24015	Chitinophaga	terrae	
Z0017_RO_F10_2_G11_B	74	63	LMG 24015	Chitinophaga	terrae	
Z0017_LO_D04_2_F02_B	75	65	LMG 2404	Pectobacterium	carotovorum	carotovorum
Z0017_LO_D07_2_F04_A	75	65	LMG 2404	Pectobacterium	carotovorum	carotovorum
Z0017_LO_D09_2_F05_A	75	65	LMG 2404	Pectobacterium	carotovorum	carotovorum
Z0017_LO_D10_2_F05_B	75	65	LMG 2404	Pectobacterium	carotovorum	carotovorum
Z0017_LO_E01_1_G01_A	75	65	LMG 2404	Pectobacterium	carotovorum	carotovorum
Z0017_LO_E04_1_G02_B	75	65	LMG 2404	Pectobacterium	carotovorum	carotovorum
Z0017_LO_E06_1_G03_B	75	65	LMG 2404	Pectobacterium	carotovorum	carotovorum

Z0017_LO_E08_1_G04_B	75	65	LMG 2404	Pectobacterium	carotovorum	carotovorum
Z0017_LO_E09_1_G05_A	75	65	LMG 2404	Pectobacterium	carotovorum	carotovorum
Z0017_LO_E10_1_G05_B	75	65	LMG 2404	Pectobacterium	carotovorum	carotovorum
Z0017_LO_E11_1_G06_A	75	65	LMG 2404	Pectobacterium	carotovorum	carotovorum
Z0017_LO_F03_2_G02_A	75	65	LMG 2404	Pectobacterium	carotovorum	carotovorum
Z0017_LO_F05_2_G03_A	75	65	LMG 2404	Pectobacterium	carotovorum	carotovorum
Z0017_LO_F06_2_G03_B	75	65	LMG 2404	Pectobacterium	carotovorum	carotovorum
Z0017_LO_F08_2_G04_B	75	65	LMG 2404	Pectobacterium	carotovorum	carotovorum
Z0017_RO_D02_2_F07_B	75	65	LMG 2404	Pectobacterium	carotovorum	carotovorum
Z0017_RO_D05_2_F09_A	75	65	LMG 2404	Pectobacterium	carotovorum	carotovorum
Z0017_RO_D08_2_F10_B	75	65	LMG 2404	Pectobacterium	carotovorum	carotovorum
Z0017_RO_D10_2_F11_B	75	65	LMG 2404	Pectobacterium	carotovorum	carotovorum
Z0017_RO_E01_1_G07_A	75	65	LMG 2404	Pectobacterium	carotovorum	carotovorum
Z0017_RO_E03_1_G08_A	75	65	LMG 2404	Pectobacterium	carotovorum	carotovorum
Z0017_RO_E06_1_G09_B	75	65	LMG 2404	Pectobacterium	carotovorum	carotovorum
Z0017_RO_E07_1_G10_A	75	65	LMG 2404	Pectobacterium	carotovorum	carotovorum
Z0017_RO_E09_1_G11_A	75	65	LMG 2404	Pectobacterium	carotovorum	carotovorum
Z0017_RO_E10_1_G11_B	75	65	LMG 2404	Pectobacterium	carotovorum	carotovorum
Z0017_RO_E12_1_G12_B	75	65	LMG 2404	Pectobacterium	carotovorum	carotovorum
Z0017_RO_F02_2_G07_B	75	65	LMG 2404	Pectobacterium	carotovorum	carotovorum
Z0017_RO_F06_2_G09_B	75	65	LMG 2404	Pectobacterium	carotovorum	carotovorum
Z0017_RO_F07_2_G10_A	75	65	LMG 2404	Pectobacterium	carotovorum	carotovorum
Z0017_RO_F08_2_G10_B	75	65	LMG 2404	Pectobacterium	carotovorum	carotovorum
Z0017_RO_F09_2_G11_A	75	65	LMG 2404	Pectobacterium	carotovorum	carotovorum
Z0017_RO_F11_2_G12_A	75	65	LMG 2404	Pectobacterium	carotovorum	carotovorum
Z0017_LO_F04_2_G02_B	76	64	LMG 24024	Oceanobacillus	oncorhynchi	oncorhynchi
Z0017_LO_F07_2_G04_A	76	64	LMG 24024	Oceanobacillus	oncorhynchi	oncorhynchi
Z0017_LO_F09_2_G05_A	76	64	LMG 24024	Oceanobacillus	oncorhynchi	oncorhynchi
Z0017_LO_F10_2_G05_B	76	64	LMG 24024	Oceanobacillus	oncorhynchi	oncorhynchi
Z0017_LO_F11_2_G06_A	76	64	LMG 24024	Oceanobacillus	oncorhynchi	oncorhynchi
Z0017_LO_F12_2_G06_B	76	64	LMG 24024	Oceanobacillus	oncorhynchi	oncorhynchi
Z0017_LO_G01_1_H01_A	76	64	LMG 24024	Oceanobacillus	oncorhynchi	oncorhynchi
Z0017_LO_G02_1_H01_B	76	64	LMG 24024	Oceanobacillus	oncorhynchi	oncorhynchi
Z0017_LO_G03_1_H02_A	76	64	LMG 24024	Oceanobacillus	oncorhynchi	oncorhynchi
Z0017_LO_G04_1_H02_B	76	64	LMG 24024	Oceanobacillus	oncorhynchi	oncorhynchi
Z0017_LO_G05_1_H03_A	76	64	LMG 24024	Oceanobacillus	oncorhynchi	oncorhynchi
Z0017_LO_G06_1_H03_B	76	64	LMG 24024	Oceanobacillus	oncorhynchi	oncorhynchi
Z0017_LO_G07_1_H04_A	76	64	LMG 24024	Oceanobacillus	oncorhynchi	oncorhynchi
Z0017_LO_G08_1_H04_B	76	64	LMG 24024	Oceanobacillus	oncorhynchi	oncorhynchi
Z0017_LO_G09_1_H05_A	76	64	LMG 24024	Oceanobacillus	oncorhynchi	oncorhynchi
Z0017_RO_F12_2_G12_B	76	64	LMG 24024	Oceanobacillus	oncorhynchi	oncorhynchi
Z0017_RO_G01_1_H07_A	76	64	LMG 24024	Oceanobacillus	oncorhynchi	oncorhynchi
Z0017_RO_G02_1_H07_B	76	64	LMG 24024	Oceanobacillus	oncorhynchi	oncorhynchi
Z0017_RO_G03_1_H08_A	76	64	LMG 24024	Oceanobacillus	oncorhynchi	oncorhynchi
Z0017_RO_G04_1_H08_B	76	64	LMG 24024	Oceanobacillus	oncorhynchi	oncorhynchi
Z0017_RO_G05_1_H09_A	76	64	LMG 24024	Oceanobacillus	oncorhynchi	oncorhynchi
Z0017_RO_G06_1_H09_B	76	64	LMG 24024	Oceanobacillus	oncorhynchi	oncorhynchi
Z0017_RO_G07_1_H10_A	76	64	LMG 24024	Oceanobacillus	oncorhynchi	oncorhynchi
Z0017_RO_G08_1_H10_B	76	64	LMG 24024	Oceanobacillus	oncorhynchi	oncorhynchi
Z0017_RO_G09_1_H11_A	76	64	LMG 24024	Oceanobacillus	oncorhynchi	oncorhynchi

Z0017_RO_G10_1_H11_B	76	64	LMG 24024	Oceanobacillus	oncorhynchi	oncorhynchi
Z0017_RO_G11_1_H12_A	76	64	LMG 24024	Oceanobacillus	oncorhynchi	oncorhynchi
Z0017_RO_G12_1_H12_B	76	64	LMG 24024	Oceanobacillus	oncorhynchi	oncorhynchi
Z0017_RO_H01_2_H07_A	76	64	LMG 24024	Oceanobacillus	oncorhynchi	oncorhynchi
Z0017_RO_H02_2_H07_B	76	64	LMG 24024	Oceanobacillus	oncorhynchi	oncorhynchi
Z0017_RO_H03_2_H08_A	76	64	LMG 24024	Oceanobacillus	oncorhynchi	oncorhynchi
Z0017_RO_H04_2_H08_B	76	64	LMG 24024	Oceanobacillus	oncorhynchi	oncorhynchi
Z0018_LB_A01_2_D06_A	77	67	LMG 24367	Ruegeria	scottomollicae	
Z0018_LB_A02_2_D06_B	77	67	LMG 24367	Ruegeria	scottomollicae	
Z0018_LB_A03_2_D05_A	77	67	LMG 24367	Ruegeria	scottomollicae	
Z0018_LB_A04_2_D05_B	77	67	LMG 24367	Ruegeria	scottomollicae	
Z0018_LB_A05_2_D04_A	77	67	LMG 24367	Ruegeria	scottomollicae	
Z0018_LB_A06_2_D04_B	77	67	LMG 24367	Ruegeria	scottomollicae	
Z0018_LB_A07_2_D03_A	77	67	LMG 24367	Ruegeria	scottomollicae	
Z0018_LB_A08_2_D03_B	77	67	LMG 24367	Ruegeria	scottomollicae	
Z0018_LB_A09_2_D02_A	77	67	LMG 24367	Ruegeria	scottomollicae	
Z0018_LB_A10_2_D02_B	77	67	LMG 24367	Ruegeria	scottomollicae	
Z0018_LB_A11_2_D01_A	77	67	LMG 24367	Ruegeria	scottomollicae	
Z0018_LB_A12_2_D01_B	77	67	LMG 24367	Ruegeria	scottomollicae	
Z0018_LO_A12_1_E06_B	77	67	LMG 24367	Ruegeria	scottomollicae	
Z0018_LO_B01_2_E01_A	77	67	LMG 24367	Ruegeria	scottomollicae	
Z0018_LO_B02_2_E01_B	77	67	LMG 24367	Ruegeria	scottomollicae	
Z0018_LO_C02_1_F01_B	77	67	LMG 24367	Ruegeria	scottomollicae	
Z0018_LO_H07_2_H04_A	77	67	LMG 24367	Ruegeria	scottomollicae	
Z0018_LO_H08_2_H04_B	77	67	LMG 24367	Ruegeria	scottomollicae	
Z0018_LO_H09_2_H05_A	77	67	LMG 24367	Ruegeria	scottomollicae	
Z0018_LO_H10_2_H05_B	77	67	LMG 24367	Ruegeria	scottomollicae	
Z0018_LO_H11_2_H06_A	77	67	LMG 24367	Ruegeria	scottomollicae	
Z0018_LO_H12_2_H06_B	77	67	LMG 24367	Ruegeria	scottomollicae	
Z0018_RB_A01_2_D12_A	77	67	LMG 24367	Ruegeria	scottomollicae	
Z0018_RB_A02_2_D12_B	77	67	LMG 24367	Ruegeria	scottomollicae	
Z0018_RB_A03_2_D11_A	77	67	LMG 24367	Ruegeria	scottomollicae	
Z0018_RB_A04_2_D11_B	77	67	LMG 24367	Ruegeria	scottomollicae	
Z0018_RB_A05_2_D10_A	77	67	LMG 24367	Ruegeria	scottomollicae	
Z0018_RB_A06_2_D10_B	77	67	LMG 24367	Ruegeria	scottomollicae	
Z0018_RB_H10_1_A08_B	77	67	LMG 24367	Ruegeria	scottomollicae	
Z0018_RO_A01_1_E07_A	77	67	LMG 24367	Ruegeria	scottomollicae	
Z0018_RO_A03_1_E08_A	77	67	LMG 24367	Ruegeria	scottomollicae	
Z0018_RO_B08_2_E10_B	77	67	LMG 24367	Ruegeria	scottomollicae	
Z0018_LB_B01_1_D06_A	78	68	LMG 24392	Granulibacter	bethesdensis	
Z0018_LB_B02_1_D06_B	78	68	LMG 24392	Granulibacter	bethesdensis	
Z0018_LB_B03_1_D05_A	78	68	LMG 24392	Granulibacter	bethesdensis	
Z0018_LB_B04_1_D05_B	78	68	LMG 24392	Granulibacter	bethesdensis	
Z0018_LB_B05_1_D04_A	78	68	LMG 24392	Granulibacter	bethesdensis	
Z0018_LB_B06_1_D04_B	78	68	LMG 24392	Granulibacter	bethesdensis	
Z0018_LB_B07_1_D03_A	78	68	LMG 24392	Granulibacter	bethesdensis	
Z0018_LB_B08_1_D03_B	78	68	LMG 24392	Granulibacter	bethesdensis	
Z0018_LB_B09_1_D02_A	78	68	LMG 24392	Granulibacter	bethesdensis	
Z0018_LB_B10_1_D02_B	78	68	LMG 24392	Granulibacter	bethesdensis	
Z0018_LB_B11_1_D01_A	78	68	LMG 24392	Granulibacter	bethesdensis	

Z0018_LB_B12_1_D01_B	78	68	LMG 24392	Granulibacter	bethesdensis
Z0018_LB_C01_2_C06_A	78	68	LMG 24392	Granulibacter	bethesdensis
Z0018_LB_C02_2_C06_B	78	68	LMG 24392	Granulibacter	bethesdensis
Z0018_LB_C03_2_C05_A	78	68	LMG 24392	Granulibacter	bethesdensis
Z0018_LB_C04_2_C05_B	78	68	LMG 24392	Granulibacter	bethesdensis
Z0018_LB_C05_2_C04_A	78	68	LMG 24392	Granulibacter	bethesdensis
Z0018_LB_C06_2_C04_B	78	68	LMG 24392	Granulibacter	bethesdensis
Z0018_LB_C07_2_C03_A	78	68	LMG 24392	Granulibacter	bethesdensis
Z0018_RB_A07_2_D09_A	78	68	LMG 24392	Granulibacter	bethesdensis
Z0018_RB_A08_2_D09_B	78	68	LMG 24392	Granulibacter	bethesdensis
Z0018_RB_A09_2_D08_A	78	68	LMG 24392	Granulibacter	bethesdensis
Z0018_RB_A10_2_D08_B	78	68	LMG 24392	Granulibacter	bethesdensis
Z0018_RB_A11_2_D07_A	78	68	LMG 24392	Granulibacter	bethesdensis
Z0018_RB_A12_2_D07_B	78	68	LMG 24392	Granulibacter	bethesdensis
Z0018_RB_B01_1_D12_A	78	68	LMG 24392	Granulibacter	bethesdensis
Z0018_RB_B02_1_D12_B	78	68	LMG 24392	Granulibacter	bethesdensis
Z0018_RB_B03_1_D11_A	78	68	LMG 24392	Granulibacter	bethesdensis
Z0018_RB_B04_1_D11_B	78	68	LMG 24392	Granulibacter	bethesdensis
Z0018_RB_B05_1_D10_A	78	68	LMG 24392	Granulibacter	bethesdensis
Z0018_RB_B06_1_D10_B	78	68	LMG 24392	Granulibacter	bethesdensis
Z0018_RB_B07_1_D09_A	78	68	LMG 24392	Granulibacter	bethesdensis
Z0018_LB_C08_2_C03_B	79	69	LMG 24401	Epilithonimonas	lactis
Z0018_LB_C09_2_C02_A	79	69	LMG 24401	Epilithonimonas	lactis
Z0018_LB_C10_2_C02_B	79	69	LMG 24401	Epilithonimonas	lactis
Z0018_LB_C11_2_C01_A	79	69	LMG 24401	Epilithonimonas	lactis
Z0018_LB_C12_2_C01_B	79	69	LMG 24401	Epilithonimonas	lactis
Z0018_LB_D01_1_C06_A	79	69	LMG 24401	Epilithonimonas	lactis
Z0018_LB_D02_1_C06_B	79	69	LMG 24401	Epilithonimonas	lactis
Z0018_LB_D03_1_C05_A	79	69	LMG 24401	Epilithonimonas	lactis
Z0018_LB_D04_1_C05_B	79	69	LMG 24401	Epilithonimonas	lactis
Z0018_LB_D05_1_C04_A	79	69	LMG 24401	Epilithonimonas	lactis
Z0018_LB_D06_1_C04_B	79	69	LMG 24401	Epilithonimonas	lactis
Z0018_LB_D07_1_C03_A	79	69	LMG 24401	Epilithonimonas	lactis
Z0018_LB_D08_1_C03_B	79	69	LMG 24401	Epilithonimonas	lactis
Z0018_LB_D09_1_C02_A	79	69	LMG 24401	Epilithonimonas	lactis
Z0018_RB_B08_1_D09_B	79	69	LMG 24401	Epilithonimonas	lactis
Z0018_RB_B09_1_D08_A	79	69	LMG 24401	Epilithonimonas	lactis
Z0018_RB_B10_1_D08_B	79	69	LMG 24401	Epilithonimonas	lactis
Z0018_RB_B11_1_D07_A	79	69	LMG 24401	Epilithonimonas	lactis
Z0018_RB_B12_1_D07_B	79	69	LMG 24401	Epilithonimonas	lactis
Z0018_RB_C01_2_C12_A	79	69	LMG 24401	Epilithonimonas	lactis
Z0018_RB_C02_2_C12_B	79	69	LMG 24401	Epilithonimonas	lactis
Z0018_RB_C03_2_C11_A	79	69	LMG 24401	Epilithonimonas	lactis
Z0018_RB_C04_2_C11_B	79	69	LMG 24401	Epilithonimonas	lactis
Z0018_RB_C05_2_C10_A	79	69	LMG 24401	Epilithonimonas	lactis
Z0018_RB_C06_2_C10_B	79	69	LMG 24401	Epilithonimonas	lactis
Z0018_RB_C07_2_C09_A	79	69	LMG 24401	Epilithonimonas	lactis
Z0018_RB_C08_2_C09_B	79	69	LMG 24401	Epilithonimonas	lactis
Z0018_RB_C09_2_C08_A	79	69	LMG 24401	Epilithonimonas	lactis
Z0018_RB_C10_2_C08_B	79	69	LMG 24401	Epilithonimonas	lactis

Z0018_RB_C11_2_C07_A	79	69	LMG 24401	Epilithonimonas	lactis
Z0018_RB_C12_2_C07_B	79	69	LMG 24401	Epilithonimonas	lactis
Z0018_RB_D01_1_C12_A	79	69	LMG 24401	Epilithonimonas	lactis
Z0018_LB_D10_1_C02_B	80	70	LMG 24411	Bhargavaea	cecembensis
Z0018_LB_D11_1_C01_A	80	70	LMG 24411	Bhargavaea	cecembensis
Z0018_LB_D12_1_C01_B	80	70	LMG 24411	Bhargavaea	cecembensis
Z0018_LB_E01_2_B06_A	80	70	LMG 24411	Bhargavaea	cecembensis
Z0018_LB_E02_2_B06_B	80	70	LMG 24411	Bhargavaea	cecembensis
Z0018_LB_E03_2_B05_A	80	70	LMG 24411	Bhargavaea	cecembensis
Z0018_LB_E04_2_B05_B	80	70	LMG 24411	Bhargavaea	cecembensis
Z0018_LB_E05_2_B04_A	80	70	LMG 24411	Bhargavaea	cecembensis
Z0018_LB_E06_2_B04_B	80	70	LMG 24411	Bhargavaea	cecembensis
Z0018_LB_E07_2_B03_A	80	70	LMG 24411	Bhargavaea	cecembensis
Z0018_LB_E08_2_B03_B	80	70	LMG 24411	Bhargavaea	cecembensis
Z0018_LB_E09_2_B02_A	80	70	LMG 24411	Bhargavaea	cecembensis
Z0018_LB_E10_2_B02_B	80	70	LMG 24411	Bhargavaea	cecembensis
Z0018_LB_E11_2_B01_A	80	70	LMG 24411	Bhargavaea	cecembensis
Z0018_LB_E12_2_B01_B	80	70	LMG 24411	Bhargavaea	cecembensis
Z0018_LB_F01_1_B06_A	80	70	LMG 24411	Bhargavaea	cecembensis
Z0018_LB_F02_1_B06_B	80	70	LMG 24411	Bhargavaea	cecembensis
Z0018_LB_F03_1_B05_A	80	70	LMG 24411	Bhargavaea	cecembensis
Z0018_RB_D02_1_C12_B	80	70	LMG 24411	Bhargavaea	cecembensis
Z0018_RB_D03_1_C11_A	80	70	LMG 24411	Bhargavaea	cecembensis
Z0018_RB_D04_1_C11_B	80	70	LMG 24411	Bhargavaea	cecembensis
Z0018_RB_D05_1_C10_A	80	70	LMG 24411	Bhargavaea	cecembensis
Z0018_RB_D06_1_C10_B	80	70	LMG 24411	Bhargavaea	cecembensis
Z0018_RB_D07_1_C09_A	80	70	LMG 24411	Bhargavaea	cecembensis
Z0018_RB_D08_1_C09_B	80	70	LMG 24411	Bhargavaea	cecembensis
Z0018_RB_D09_1_C08_A	80	70	LMG 24411	Bhargavaea	cecembensis
Z0018_RB_D10_1_C08_B	80	70	LMG 24411	Bhargavaea	cecembensis
Z0018_RB_D11_1_C07_A	80	70	LMG 24411	Bhargavaea	cecembensis
Z0018_RB_D12_1_C07_B	80	70	LMG 24411	Bhargavaea	cecembensis
Z0018_RB_E01_2_B12_A	80	70	LMG 24411	Bhargavaea	cecembensis
Z0018_RB_E02_2_B12_B	80	70	LMG 24411	Bhargavaea	cecembensis
Z0018_RB_E03_2_B11_A	80	70	LMG 24411	Bhargavaea	cecembensis
Z0018_LB_F04_1_B05_B	81	71	LMG 24424	Shewanella	vesiculosa
Z0018_LB_F05_1_B04_A	81	71	LMG 24424	Shewanella	vesiculosa
Z0018_LB_F06_1_B04_B	81	71	LMG 24424	Shewanella	vesiculosa
Z0018_LB_F07_1_B03_A	81	71	LMG 24424	Shewanella	vesiculosa
Z0018_LB_F08_1_B03_B	81	71	LMG 24424	Shewanella	vesiculosa
Z0018_LB_F09_1_B02_A	81	71	LMG 24424	Shewanella	vesiculosa
Z0018_LB_F10_1_B02_B	81	71	LMG 24424	Shewanella	vesiculosa
Z0018_LB_F11_1_B01_A	81	71	LMG 24424	Shewanella	vesiculosa
Z0018_LB_F12_1_B01_B	81	71	LMG 24424	Shewanella	vesiculosa
Z0018_LB_G01_2_A06_A	81	71	LMG 24424	Shewanella	vesiculosa
Z0018_LB_G02_2_A06_B	81	71	LMG 24424	Shewanella	vesiculosa
Z0018_LB_G03_2_A05_A	81	71	LMG 24424	Shewanella	vesiculosa
Z0018_LB_G04_2_A05_B	81	71	LMG 24424	Shewanella	vesiculosa
Z0018_LB_G05_2_A04_A	81	71	LMG 24424	Shewanella	vesiculosa
Z0018_LB_G06_2_A04_B	81	71	LMG 24424	Shewanella	vesiculosa

Z0018_RB_E04_2_B11_B	81	71	LMG 24424	Shewanella	vesiculosa
Z0018_RB_E05_2_B10_A	81	71	LMG 24424	Shewanella	vesiculosa
Z0018_RB_E06_2_B10_B	81	71	LMG 24424	Shewanella	vesiculosa
Z0018_RB_E07_2_B09_A	81	71	LMG 24424	Shewanella	vesiculosa
Z0018_RB_E08_2_B09_B	81	71	LMG 24424	Shewanella	vesiculosa
Z0018_RB_E09_2_B08_A	81	71	LMG 24424	Shewanella	vesiculosa
Z0018_RB_E10_2_B08_B	81	71	LMG 24424	Shewanella	vesiculosa
Z0018_RB_E11_2_B07_A	81	71	LMG 24424	Shewanella	vesiculosa
Z0018_RB_E12_2_B07_B	81	71	LMG 24424	Shewanella	vesiculosa
Z0018_RB_F01_1_B12_A	81	71	LMG 24424	Shewanella	vesiculosa
Z0018_RB_F02_1_B12_B	81	71	LMG 24424	Shewanella	vesiculosa
Z0018_RB_F03_1_B11_A	81	71	LMG 24424	Shewanella	vesiculosa
Z0018_RB_F04_1_B11_B	81	71	LMG 24424	Shewanella	vesiculosa
Z0018_RB_F05_1_B10_A	81	71	LMG 24424	Shewanella	vesiculosa
Z0018_RB_F06_1_B10_B	81	71	LMG 24424	Shewanella	vesiculosa
Z0018_RB_F07_1_B09_A	81	71	LMG 24424	Shewanella	vesiculosa
Z0018_RB_F08_1_B09_B	81	71	LMG 24424	Shewanella	vesiculosa
Z0018_LB_H11_1_A01_A	82	72	LMG 24537	Stenotrophomonas	rhizophila
Z0018_LB_H12_1_A01_B	82	72	LMG 24537	Stenotrophomonas	rhizophila
Z0018_LO_A01_1_E01_A	82	72	LMG 24537	Stenotrophomonas	rhizophila
Z0018_LO_A02_1_E01_B	82	72	LMG 24537	Stenotrophomonas	rhizophila
Z0018_LO_A03_1_E02_A	82	72	LMG 24537	Stenotrophomonas	rhizophila
Z0018_LO_A04_1_E02_B	82	72	LMG 24537	Stenotrophomonas	rhizophila
Z0018_LO_A05_1_E03_A	82	72	LMG 24537	Stenotrophomonas	rhizophila
Z0018_LO_A06_1_E03_B	82	72	LMG 24537	Stenotrophomonas	rhizophila
Z0018_LO_A07_1_E04_A	82	72	LMG 24537	Stenotrophomonas	rhizophila
Z0018_LO_A08_1_E04_B	82	72	LMG 24537	Stenotrophomonas	rhizophila
Z0018_LO_A09_1_E05_A	82	72	LMG 24537	Stenotrophomonas	rhizophila
Z0018_LO_A10_1_E05_B	82	72	LMG 24537	Stenotrophomonas	rhizophila
Z0018_RB_H01_1_A12_A	82	72	LMG 24537	Stenotrophomonas	rhizophila
Z0018_RB_H02_1_A12_B	82	72	LMG 24537	Stenotrophomonas	rhizophila
Z0018_RB_H03_1_A11_A	82	72	LMG 24537	Stenotrophomonas	rhizophila
Z0018_RB_H04_1_A11_B	82	72	LMG 24537	Stenotrophomonas	rhizophila
Z0018_RB_H05_1_A10_A	82	72	LMG 24537	Stenotrophomonas	rhizophila
Z0018_RB_H06_1_A10_B	82	72	LMG 24537	Stenotrophomonas	rhizophila
Z0018_RB_H09_1_A08_A	82	72	LMG 24537	Stenotrophomonas	rhizophila
Z0018_RB_H11_1_A07_A	82	72	LMG 24537	Stenotrophomonas	rhizophila
Z0018_RB_H12_1_A07_B	82	72	LMG 24537	Stenotrophomonas	rhizophila
Z0018_RO_A02_1_E07_B	82	72	LMG 24537	Stenotrophomonas	rhizophila
Z0018_RO_A04_1_E08_B	82	72	LMG 24537	Stenotrophomonas	rhizophila
Z0018_RO_A05_1_E09_A	82	72	LMG 24537	Stenotrophomonas	rhizophila
Z0018_RO_A06_1_E09_B	82	72	LMG 24537	Stenotrophomonas	rhizophila
Z0018_RO_A07_1_E10_A	82	72	LMG 24537	Stenotrophomonas	rhizophila
Z0018_RO_A08_1_E10_B	82	72	LMG 24537	Stenotrophomonas	rhizophila
Z0018_RO_A09_1_E11_A	82	72	LMG 24537	Stenotrophomonas	rhizophila
Z0018_LO_A11_1_E06_A	83	76	LMG 24833	Vagococcus	penaei
Z0018_LO_B10_2_E05_B	83	76	LMG 24833	Vagococcus	penaei
Z0018_LO_B11_2_E06_A	83	76	LMG 24833	Vagococcus	penaei
Z0018_LO_B12_2_E06_B	83	76	LMG 24833	Vagococcus	penaei
Z0018_LO_C04_1_F02_B	83	76	LMG 24833	Vagococcus	penaei

Z0018_LO_C09_1_F05_A	83	76	LMG 24833	Vagococcus	penaei
Z0018_LO_C12_1_F06_B	83	76	LMG 24833	Vagococcus	penaei
Z0018_LO_D03_2_F02_A	83	76	LMG 24833	Vagococcus	penaei
Z0018_LO_D06_2_F03_B	83	76	LMG 24833	Vagococcus	penaei
Z0018_LO_E02_1_G01_B	83	76	LMG 24833	Vagococcus	penaei
Z0018_LO_E03_1_G02_A	83	76	LMG 24833	Vagococcus	penaei
Z0018_LO_E05_1_G03_A	83	76	LMG 24833	Vagococcus	penaei
Z0018_LO_E07_1_G04_A	83	76	LMG 24833	Vagococcus	penaei
Z0018_LO_E10_1_G05_B	83	76	LMG 24833	Vagococcus	penaei
Z0018_LO_E11_1_G06_A	83	76	LMG 24833	Vagococcus	penaei
Z0018_LO_F01_2_G01_A	83	76	LMG 24833	Vagococcus	penaei
Z0018_LO_F02_2_G01_B	83	76	LMG 24833	Vagococcus	penaei
Z0018_LO_F08_2_G04_B	83	76	LMG 24833	Vagococcus	penaei
Z0018_RB_H07_1_A09_A	83	76	LMG 24833	Vagococcus	penaei
Z0018_RB_H08_1_A09_B	83	76	LMG 24833	Vagococcus	penaei
Z0018_RO_A11_1_E12_A	83	76	LMG 24833	Vagococcus	penaei
Z0018_RO_A12_1_E12_B	83	76	LMG 24833	Vagococcus	penaei
Z0018_RO_B01_2_E07_A	83	76	LMG 24833	Vagococcus	penaei
Z0018_RO_B02_2_E07_B	83	76	LMG 24833	Vagococcus	penaei
Z0018_RO_B03_2_E08_A	83	76	LMG 24833	Vagococcus	penaei
Z0018_RO_B10_2_E11_B	83	76	LMG 24833	Vagococcus	penaei
Z0018_RO_C01_1_F07_A	83	76	LMG 24833	Vagococcus	penaei
Z0018_RO_C02_1_F07_B	83	76	LMG 24833	Vagococcus	penaei
Z0018_RO_C03_1_F08_A	83	76	LMG 24833	Vagococcus	penaei
Z0018_RO_C06_1_F09_B	83	76	LMG 24833	Vagococcus	penaei
Z0018_RO_C07_1_F10_A	83	76	LMG 24833	Vagococcus	penaei
Z0018_RO_C11_1_F12_A	83	76	LMG 24833	Vagococcus	penaei
Z0018_LO_B03_2_E02_A	84	73	LMG 24552	Roseomonas	gilardii
Z0018_LO_B04_2_E02_B	84	73	LMG 24552	Roseomonas	gilardii
Z0018_LO_B05_2_E03_A	84	73	LMG 24552	Roseomonas	gilardii
Z0018_LO_B06_2_E03_B	84	73	LMG 24552	Roseomonas	gilardii
Z0018_LO_B07_2_E04_A	84	73	LMG 24552	Roseomonas	gilardii
Z0018_LO_B08_2_E04_B	84	73	LMG 24552	Roseomonas	gilardii
Z0018_LO_B09_2_E05_A	84	73	LMG 24552	Roseomonas	gilardii
Z0018_LO_C01_1_F01_A	84	73	LMG 24552	Roseomonas	gilardii
Z0018_LO_C03_1_F02_A	84	73	LMG 24552	Roseomonas	gilardii
Z0018_LO_C05_1_F03_A	84	73	LMG 24552	Roseomonas	gilardii
Z0018_LO_C06_1_F03_B	84	73	LMG 24552	Roseomonas	gilardii
Z0018_LO_C07_1_F04_A	84	73	LMG 24552	Roseomonas	gilardii
Z0018_LO_C08_1_F04_B	84	73	LMG 24552	Roseomonas	gilardii
Z0018_LO_C10_1_F05_B	84	73	LMG 24552	Roseomonas	gilardii
Z0018_LO_C11_1_F06_A	84	73	LMG 24552	Roseomonas	gilardii
Z0018_LO_D01_2_F01_A	84	73	LMG 24552	Roseomonas	gilardii
Z0018_RO_A10_1_E11_B	84	73	LMG 24552	Roseomonas	gilardii
Z0018_RO_B04_2_E08_B	84	73	LMG 24552	Roseomonas	gilardii
Z0018_RO_B05_2_E09_A	84	73	LMG 24552	Roseomonas	gilardii
Z0018_RO_B06_2_E09_B	84	73	LMG 24552	Roseomonas	gilardii
Z0018_RO_B07_2_E10_A	84	73	LMG 24552	Roseomonas	gilardii
Z0018_RO_B09_2_E11_A	84	73	LMG 24552	Roseomonas	gilardii
Z0018_RO_B11_2_E12_A	84	73	LMG 24552	Roseomonas	gilardii

Z0018_RO_B12_2_E12_B	84	73	LMG 24552	Roseomonas	gilardii
Z0018_RO_C04_1_F08_B	84	73	LMG 24552	Roseomonas	gilardii
Z0018_RO_C05_1_F09_A	84	73	LMG 24552	Roseomonas	gilardii
Z0018_RO_C08_1_F10_B	84	73	LMG 24552	Roseomonas	gilardii
Z0018_RO_C09_1_F11_A	84	73	LMG 24552	Roseomonas	gilardii
Z0018_LO_D02_2_F01_B	85	74	LMG 24559	Arcobacter	mytili
Z0018_LO_D04_2_F02_B	85	74	LMG 24559	Arcobacter	mytili
Z0018_LO_D05_2_F03_A	85	74	LMG 24559	Arcobacter	mytili
Z0018_LO_D07_2_F04_A	85	74	LMG 24559	Arcobacter	mytili
Z0018_LO_D08_2_F04_B	85	74	LMG 24559	Arcobacter	mytili
Z0018_LO_D09_2_F05_A	85	74	LMG 24559	Arcobacter	mytili
Z0018_LO_D10_2_F05_B	85	74	LMG 24559	Arcobacter	mytili
Z0018_LO_D11_2_F06_A	85	74	LMG 24559	Arcobacter	mytili
Z0018_LO_D12_2_F06_B	85	74	LMG 24559	Arcobacter	mytili
Z0018_LO_E01_1_G01_A	85	74	LMG 24559	Arcobacter	mytili
Z0018_LO_E04_1_G02_B	85	74	LMG 24559	Arcobacter	mytili
Z0018_LO_E06_1_G03_B	85	74	LMG 24559	Arcobacter	mytili
Z0018_LO_E08_1_G04_B	85	74	LMG 24559	Arcobacter	mytili
Z0018_LO_E09_1_G05_A	85	74	LMG 24559	Arcobacter	mytili
Z0018_LO_E12_1_G06_B	85	74	LMG 24559	Arcobacter	mytili
Z0018_LO_F03_2_G02_A	85	74	LMG 24559	Arcobacter	mytili
Z0018_LO_F04_2_G02_B	85	74	LMG 24559	Arcobacter	mytili
Z0018_LO_F09_2_G05_A	85	74	LMG 24559	Arcobacter	mytili
Z0018_LO_F10_2_G05_B	85	74	LMG 24559	Arcobacter	mytili
Z0018_RO_C12_1_F12_B	85	74	LMG 24559	Arcobacter	mytili
Z0018_RO_D03_2_F08_A	85	74	LMG 24559	Arcobacter	mytili
Z0018_RO_E01_1_G07_A	85	74	LMG 24559	Arcobacter	mytili
Z0018_RO_E02_1_G07_B	85	74	LMG 24559	Arcobacter	mytili
Z0018_RO_E04_1_G08_B	85	74	LMG 24559	Arcobacter	mytili
Z0018_RO_E06_1_G09_B	85	74	LMG 24559	Arcobacter	mytili
Z0018_RO_E09_1_G11_A	85	74	LMG 24559	Arcobacter	mytili
Z0018_RO_E10_1_G11_B	85	74	LMG 24559	Arcobacter	mytili
Z0018_RO_E12_1_G12_B	85	74	LMG 24559	Arcobacter	mytili
Z0018_RO_F01_2_G07_A	85	74	LMG 24559	Arcobacter	mytili
Z0018_RO_F04_2_G08_B	85	74	LMG 24559	Arcobacter	mytili
Z0018_RO_F05_2_G09_A	85	74	LMG 24559	Arcobacter	mytili
Z0018_RO_F06_2_G09_B	85	74	LMG 24559	Arcobacter	mytili
Z0018_LO_F05_2_G03_A	86	4	LMG 24832	Lactobacillus	plantarum
Z0018_LO_F06_2_G03_B	86	4	LMG 24832	Lactobacillus	plantarum
Z0018_LO_F07_2_G04_A	86	4	LMG 24832	Lactobacillus	plantarum
Z0018_LO_F11_2_G06_A	86	4	LMG 24832	Lactobacillus	plantarum
Z0018_LO_F12_2_G06_B	86	4	LMG 24832	Lactobacillus	plantarum
Z0018_LO_G01_1_H01_A	86	4	LMG 24832	Lactobacillus	plantarum
Z0018_LO_G02_1_H01_B	86	4	LMG 24832	Lactobacillus	plantarum
Z0018_LO_G03_1_H02_A	86	4	LMG 24832	Lactobacillus	plantarum
Z0018_LO_G05_1_H03_A	86	4	LMG 24832	Lactobacillus	plantarum
Z0018_LO_G06_1_H03_B	86	4	LMG 24832	Lactobacillus	plantarum
Z0018_LO_G10_1_H05_B	86	4	LMG 24832	Lactobacillus	plantarum
Z0018_RO_C10_1_F11_B	86	4	LMG 24832	Lactobacillus	plantarum
Z0018_RO_D01_2_F07_A	86	4	LMG 24832	Lactobacillus	plantarum

Z0018_RO_D02_2_F07_B	86	4	LMG 24832	Lactobacillus	plantarum
Z0018_RO_D04_2_F08_B	86	4	LMG 24832	Lactobacillus	plantarum
Z0018_RO_D05_2_F09_A	86	4	LMG 24832	Lactobacillus	plantarum
Z0018_RO_D06_2_F09_B	86	4	LMG 24832	Lactobacillus	plantarum
Z0018_RO_D07_2_F10_A	86	4	LMG 24832	Lactobacillus	plantarum
Z0018_RO_D08_2_F10_B	86	4	LMG 24832	Lactobacillus	plantarum
Z0018_RO_D09_2_F11_A	86	4	LMG 24832	Lactobacillus	plantarum
Z0018_RO_D10_2_F11_B	86	4	LMG 24832	Lactobacillus	plantarum
Z0018_RO_D11_2_F12_A	86	4	LMG 24832	Lactobacillus	plantarum
Z0018_RO_D12_2_F12_B	86	4	LMG 24832	Lactobacillus	plantarum
Z0018_RO_E03_1_G08_A	86	4	LMG 24832	Lactobacillus	plantarum
Z0018_RO_E05_1_G09_A	86	4	LMG 24832	Lactobacillus	plantarum
Z0018_RO_E07_1_G10_A	86	4	LMG 24832	Lactobacillus	plantarum
Z0018_RO_E08_1_G10_B	86	4	LMG 24832	Lactobacillus	plantarum
Z0018_RO_E11_1_G12_A	86	4	LMG 24832	Lactobacillus	plantarum
Z0018_RO_F02_2_G07_B	86	4	LMG 24832	Lactobacillus	plantarum
Z0018_RO_F03_2_G08_A	86	4	LMG 24832	Lactobacillus	plantarum
Z0018_RO_F07_2_G10_A	86	4	LMG 24832	Lactobacillus	plantarum
Z0018_RO_F08_2_G10_B	86	4	LMG 24832	Lactobacillus	plantarum
Z0018_LO_G04_1_H02_B	87	75	LMG 24812	Candidimonas	nitroreducens
Z0018_LO_G07_1_H04_A	87	75	LMG 24812	Candidimonas	nitroreducens
Z0018_LO_G08_1_H04_B	87	75	LMG 24812	Candidimonas	nitroreducens
Z0018_LO_G09_1_H05_A	87	75	LMG 24812	Candidimonas	nitroreducens
Z0018_LO_G11_1_H06_A	87	75	LMG 24812	Candidimonas	nitroreducens
Z0018_LO_G12_1_H06_B	87	75	LMG 24812	Candidimonas	nitroreducens
Z0018_LO_H01_2_H01_A	87	75	LMG 24812	Candidimonas	nitroreducens
Z0018_LO_H02_2_H01_B	87	75	LMG 24812	Candidimonas	nitroreducens
Z0018_LO_H03_2_H02_A	87	75	LMG 24812	Candidimonas	nitroreducens
Z0018_LO_H04_2_H02_B	87	75	LMG 24812	Candidimonas	nitroreducens
Z0018_LO_H05_2_H03_A	87	75	LMG 24812	Candidimonas	nitroreducens
Z0018_LO_H06_2_H03_B	87	75	LMG 24812	Candidimonas	nitroreducens
Z0018_RO_F09_2_G11_A	87	75	LMG 24812	Candidimonas	nitroreducens
Z0018_RO_F10_2_G11_B	87	75	LMG 24812	Candidimonas	nitroreducens
Z0018_RO_F11_2_G12_A	87	75	LMG 24812	Candidimonas	nitroreducens
Z0018_RO_F12_2_G12_B	87	75	LMG 24812	Candidimonas	nitroreducens
Z0018_RO_G01_1_H07_A	87	75	LMG 24812	Candidimonas	nitroreducens
Z0018_RO_G02_1_H07_B	87	75	LMG 24812	Candidimonas	nitroreducens
Z0018_RO_G03_1_H08_A	87	75	LMG 24812	Candidimonas	nitroreducens
Z0018_RO_G04_1_H08_B	87	75	LMG 24812	Candidimonas	nitroreducens
Z0018_RO_G05_1_H09_A	87	75	LMG 24812	Candidimonas	nitroreducens
Z0018_RO_G06_1_H09_B	87	75	LMG 24812	Candidimonas	nitroreducens
Z0018_RO_G07_1_H10_A	87	75	LMG 24812	Candidimonas	nitroreducens
Z0018_RO_G08_1_H10_B	87	75	LMG 24812	Candidimonas	nitroreducens
Z0018_RO_G09_1_H11_A	87	75	LMG 24812	Candidimonas	nitroreducens
Z0018_RO_G10_1_H11_B	87	75	LMG 24812	Candidimonas	nitroreducens
Z0018_RO_G11_1_H12_A	87	75	LMG 24812	Candidimonas	nitroreducens
Z0018_RO_G12_1_H12_B	87	75	LMG 24812	Candidimonas	nitroreducens
Z0018_RO_H01_2_H07_A	87	75	LMG 24812	Candidimonas	nitroreducens
Z0018_RO_H02_2_H07_B	87	75	LMG 24812	Candidimonas	nitroreducens
Z0018_RO_H03_2_H08_A	87	75	LMG 24812	Candidimonas	nitroreducens

Z0018_RO_H04_2_H08_B	87	75	LMG 24812	Candidimonas	nitroreducens
Z0019_LB_A01_2_D06_A	88	77	LMG 25212	Polynucleobacter	cosmopolitanus
Z0019_LB_A02_2_D06_B	88	77	LMG 25212	Polynucleobacter	cosmopolitanus
Z0019_LB_A03_2_D05_A	88	77	LMG 25212	Polynucleobacter	cosmopolitanus
Z0019_LB_A04_2_D05_B	88	77	LMG 25212	Polynucleobacter	cosmopolitanus
Z0019_LB_A05_2_D04_A	88	77	LMG 25212	Polynucleobacter	cosmopolitanus
Z0019_LB_A06_2_D04_B	88	77	LMG 25212	Polynucleobacter	cosmopolitanus
Z0019_LB_A07_2_D03_A	88	77	LMG 25212	Polynucleobacter	cosmopolitanus
Z0019_LB_A08_2_D03_B	88	77	LMG 25212	Polynucleobacter	cosmopolitanus
Z0019_LB_A09_2_D02_A	88	77	LMG 25212	Polynucleobacter	cosmopolitanus
Z0019_LB_A10_2_D02_B	88	77	LMG 25212	Polynucleobacter	cosmopolitanus
Z0019_LB_A11_2_D01_A	88	77	LMG 25212	Polynucleobacter	cosmopolitanus
Z0019_LB_A12_2_D01_B	88	77	LMG 25212	Polynucleobacter	cosmopolitanus
Z0019_LO_A07_1_E04_A	88	77	LMG 25212	Polynucleobacter	cosmopolitanus
Z0019_LO_A11_1_E06_A	88	77	LMG 25212	Polynucleobacter	cosmopolitanus
Z0019_LO_B02_2_E01_B	88	77	LMG 25212	Polynucleobacter	cosmopolitanus
Z0019_LO_B04_2_E02_B	88	77	LMG 25212	Polynucleobacter	cosmopolitanus
Z0019_LO_C02_1_F01_B	88	77	LMG 25212	Polynucleobacter	cosmopolitanus
Z0019_LO_H02_2_H01_B	88	77	LMG 25212	Polynucleobacter	cosmopolitanus
Z0019_LO_H03_2_H02_A	88	77	LMG 25212	Polynucleobacter	cosmopolitanus
Z0019_LO_H04_2_H02_B	88	77	LMG 25212	Polynucleobacter	cosmopolitanus
Z0019_LO_H05_2_H03_A	88	77	LMG 25212	Polynucleobacter	cosmopolitanus
Z0019_LO_H06_2_H03_B	88	77	LMG 25212	Polynucleobacter	cosmopolitanus
Z0019_LO_H07_2_H04_A	88	77	LMG 25212	Polynucleobacter	cosmopolitanus
Z0019_LO_H08_2_H04_B	88	77	LMG 25212	Polynucleobacter	cosmopolitanus
Z0019_LO_H09_2_H05_A	88	77	LMG 25212	Polynucleobacter	cosmopolitanus
Z0019_LO_H10_2_H05_B	88	77	LMG 25212	Polynucleobacter	cosmopolitanus
Z0019_LO_H11_2_H06_A	88	77	LMG 25212	Polynucleobacter	cosmopolitanus
Z0019_LO_H12_2_H06_B	88	77	LMG 25212	Polynucleobacter	cosmopolitanus
Z0019_RB_A01_2_D12_A	88	77	LMG 25212	Polynucleobacter	cosmopolitanus
Z0019_RO_B09_2_E11_A	88	77	LMG 25212	Polynucleobacter	cosmopolitanus
Z0019_RO_C08_1_F10_B	88	77	LMG 25212	Polynucleobacter	cosmopolitanus
Z0019_RO_C09_1_F11_A	88	77	LMG 25212	Polynucleobacter	cosmopolitanus
Z0019_LB_B01_1_D06_A	89	78	LMG 25420	Echinimonas	agarilytica
Z0019_LB_B02_1_D06_B	89	78	LMG 25420	Echinimonas	agarilytica
Z0019_LB_B03_1_D05_A	89	78	LMG 25420	Echinimonas	agarilytica
Z0019_LB_B04_1_D05_B	89	78	LMG 25420	Echinimonas	agarilytica
Z0019_LB_B05_1_D04_A	89	78	LMG 25420	Echinimonas	agarilytica
Z0019_LB_B06_1_D04_B	89	78	LMG 25420	Echinimonas	agarilytica
Z0019_LB_B07_1_D03_A	89	78	LMG 25420	Echinimonas	agarilytica
Z0019_LB_B08_1_D03_B	89	78	LMG 25420	Echinimonas	agarilytica
Z0019_LB_B09_1_D02_A	89	78	LMG 25420	Echinimonas	agarilytica
Z0019_LB_B10_1_D02_B	89	78	LMG 25420	Echinimonas	agarilytica
Z0019_LB_B11_1_D01_A	89	78	LMG 25420	Echinimonas	agarilytica
Z0019_LB_B12_1_D01_B	89	78	LMG 25420	Echinimonas	agarilytica
Z0019_LB_C01_2_C06_A	89	78	LMG 25420	Echinimonas	agarilytica
Z0019_LB_C02_2_C06_B	89	78	LMG 25420	Echinimonas	agarilytica
Z0019_LB_C03_2_C05_A	89	78	LMG 25420	Echinimonas	agarilytica
Z0019_LB_C04_2_C05_B	89	78	LMG 25420	Echinimonas	agarilytica
Z0019_RB_A02_2_D12_B	89	78	LMG 25420	Echinimonas	agarilytica

Z0019_RB_A03_2_D11_A	89	78	LMG 25420	Echinimonas	agarilytica
Z0019_RB_A04_2_D11_B	89	78	LMG 25420	Echinimonas	agarilytica
Z0019_RB_A05_2_D10_A	89	78	LMG 25420	Echinimonas	agarilytica
Z0019_RB_A06_2_D10_B	89	78	LMG 25420	Echinimonas	agarilytica
Z0019_RB_A07_2_D09_A	89	78	LMG 25420	Echinimonas	agarilytica
Z0019_RB_A08_2_D09_B	89	78	LMG 25420	Echinimonas	agarilytica
Z0019_RB_A09_2_D08_A	89	78	LMG 25420	Echinimonas	agarilytica
Z0019_RB_A10_2_D08_B	89	78	LMG 25420	Echinimonas	agarilytica
Z0019_RB_A11_2_D07_A	89	78	LMG 25420	Echinimonas	agarilytica
Z0019_RB_A12_2_D07_B	89	78	LMG 25420	Echinimonas	agarilytica
Z0019_RB_B01_1_D12_A	89	78	LMG 25420	Echinimonas	agarilytica
Z0019_RB_B02_1_D12_B	89	78	LMG 25420	Echinimonas	agarilytica
Z0019_RB_B03_1_D11_A	89	78	LMG 25420	Echinimonas	agarilytica
Z0019_RB_B04_1_D11_B	89	78	LMG 25420	Echinimonas	agarilytica
Z0019_RB_B05_1_D10_A	89	78	LMG 25420	Echinimonas	agarilytica
Z0019_LB_C05_2_C04_A	90	79	LMG 25435	Marinobacterium	coralli
Z0019_LB_C06_2_C04_B	90	79	LMG 25435	Marinobacterium	coralli
Z0019_LB_C07_2_C03_A	90	79	LMG 25435	Marinobacterium	coralli
Z0019_LB_C08_2_C03_B	90	79	LMG 25435	Marinobacterium	coralli
Z0019_LB_C09_2_C02_A	90	79	LMG 25435	Marinobacterium	coralli
Z0019_LB_C10_2_C02_B	90	79	LMG 25435	Marinobacterium	coralli
Z0019_LB_C11_2_C01_A	90	79	LMG 25435	Marinobacterium	coralli
Z0019_LB_C12_2_C01_B	90	79	LMG 25435	Marinobacterium	coralli
Z0019_LB_D01_1_C06_A	90	79	LMG 25435	Marinobacterium	coralli
Z0019_LB_D02_1_C06_B	90	79	LMG 25435	Marinobacterium	coralli
Z0019_LB_D03_1_C05_A	90	79	LMG 25435	Marinobacterium	coralli
Z0019_LB_D04_1_C05_B	90	79	LMG 25435	Marinobacterium	coralli
Z0019_LB_D05_1_C04_A	90	79	LMG 25435	Marinobacterium	coralli
Z0019_LB_D06_1_C04_B	90	79	LMG 25435	Marinobacterium	coralli
Z0019_RB_B06_1_D10_B	90	79	LMG 25435	Marinobacterium	coralli
Z0019_RB_B07_1_D09_A	90	79	LMG 25435	Marinobacterium	coralli
Z0019_RB_B08_1_D09_B	90	79	LMG 25435	Marinobacterium	coralli
Z0019_RB_B09_1_D08_A	90	79	LMG 25435	Marinobacterium	coralli
Z0019_RB_B10_1_D08_B	90	79	LMG 25435	Marinobacterium	coralli
Z0019_RB_B11_1_D07_A	90	79	LMG 25435	Marinobacterium	coralli
Z0019_RB_B12_1_D07_B	90	79	LMG 25435	Marinobacterium	coralli
Z0019_RB_C01_2_C12_A	90	79	LMG 25435	Marinobacterium	coralli
Z0019_RB_C02_2_C12_B	90	79	LMG 25435	Marinobacterium	coralli
Z0019_RB_C03_2_C11_A	90	79	LMG 25435	Marinobacterium	coralli
Z0019_RB_C04_2_C11_B	90	79	LMG 25435	Marinobacterium	coralli
Z0019_RB_C05_2_C10_A	90	79	LMG 25435	Marinobacterium	coralli
Z0019_RB_C06_2_C10_B	90	79	LMG 25435	Marinobacterium	coralli
Z0019_RB_C07_2_C09_A	90	79	LMG 25435	Marinobacterium	coralli
Z0019_RB_C08_2_C09_B	90	79	LMG 25435	Marinobacterium	coralli
Z0019_RB_C09_2_C08_A	90	79	LMG 25435	Marinobacterium	coralli
Z0019_RB_C10_2_C08_B	90	79	LMG 25435	Marinobacterium	coralli
Z0019_RB_C11_2_C07_A	90	79	LMG 25435	Marinobacterium	coralli
Z0019_LB_D07_1_C03_A	91	80	LMG 25535	Arcobacter	trophiarum
Z0019_LB_D08_1_C03_B	91	80	LMG 25535	Arcobacter	trophiarum
Z0019_LB_D09_1_C02_A	91	80	LMG 25535	Arcobacter	trophiarum

Z0019_LB_D10_1_C02_B	91	80	LMG 25535	Arcobacter	trophiarum
Z0019_LB_D11_1_C01_A	91	80	LMG 25535	Arcobacter	trophiarum
Z0019_LB_D12_1_C01_B	91	80	LMG 25535	Arcobacter	trophiarum
Z0019_LB_E01_2_B06_A	91	80	LMG 25535	Arcobacter	trophiarum
Z0019_LB_E02_2_B06_B	91	80	LMG 25535	Arcobacter	trophiarum
Z0019_LB_E03_2_B05_A	91	80	LMG 25535	Arcobacter	trophiarum
Z0019_LB_E04_2_B05_B	91	80	LMG 25535	Arcobacter	trophiarum
Z0019_LB_E05_2_B04_A	91	80	LMG 25535	Arcobacter	trophiarum
Z0019_LB_E06_2_B04_B	91	80	LMG 25535	Arcobacter	trophiarum
Z0019_LB_E07_2_B03_A	91	80	LMG 25535	Arcobacter	trophiarum
Z0019_RB_C12_2_C07_B	91	80	LMG 25535	Arcobacter	trophiarum
Z0019_RB_D01_1_C12_A	91	80	LMG 25535	Arcobacter	trophiarum
Z0019_RB_D02_1_C12_B	91	80	LMG 25535	Arcobacter	trophiarum
Z0019_RB_D03_1_C11_A	91	80	LMG 25535	Arcobacter	trophiarum
Z0019_RB_D04_1_C11_B	91	80	LMG 25535	Arcobacter	trophiarum
Z0019_RB_D05_1_C10_A	91	80	LMG 25535	Arcobacter	trophiarum
Z0019_RB_D06_1_C10_B	91	80	LMG 25535	Arcobacter	trophiarum
Z0019_RB_D07_1_C09_A	91	80	LMG 25535	Arcobacter	trophiarum
Z0019_RB_D08_1_C09_B	91	80	LMG 25535	Arcobacter	trophiarum
Z0019_RB_D09_1_C08_A	91	80	LMG 25535	Arcobacter	trophiarum
Z0019_RB_D10_1_C08_B	91	80	LMG 25535	Arcobacter	trophiarum
Z0019_RB_D11_1_C07_A	91	80	LMG 25535	Arcobacter	trophiarum
Z0019_RB_D12_1_C07_B	91	80	LMG 25535	Arcobacter	trophiarum
Z0019_RB_E01_2_B12_A	91	80	LMG 25535	Arcobacter	trophiarum
Z0019_RB_E02_2_B12_B	91	80	LMG 25535	Arcobacter	trophiarum
Z0019_RB_E03_2_B11_A	91	80	LMG 25535	Arcobacter	trophiarum
Z0019_RB_E04_2_B11_B	91	80	LMG 25535	Arcobacter	trophiarum
Z0019_RB_E05_2_B10_A	91	80	LMG 25535	Arcobacter	trophiarum
Z0019_RB_E06_2_B10_B	91	80	LMG 25535	Arcobacter	trophiarum
Z0019_LB_E08_2_B03_B	92	81	LMG 25547	Glaciimonas	immobilis
Z0019_LB_E09_2_B02_A	92	81	LMG 25547	Glaciimonas	immobilis
Z0019_LB_E10_2_B02_B	92	81	LMG 25547	Glaciimonas	immobilis
Z0019_LB_E11_2_B01_A	92	81	LMG 25547	Glaciimonas	immobilis
Z0019_LB_E12_2_B01_B	92	81	LMG 25547	Glaciimonas	immobilis
Z0019_LB_F01_1_B06_A	92	81	LMG 25547	Glaciimonas	immobilis
Z0019_LB_F02_1_B06_B	92	81	LMG 25547	Glaciimonas	immobilis
Z0019_LB_F03_1_B05_A	92	81	LMG 25547	Glaciimonas	immobilis
Z0019_LB_F04_1_B05_B	92	81	LMG 25547	Glaciimonas	immobilis
Z0019_LB_F05_1_B04_A	92	81	LMG 25547	Glaciimonas	immobilis
Z0019_LB_F06_1_B04_B	92	81	LMG 25547	Glaciimonas	immobilis
Z0019_LB_F07_1_B03_A	92	81	LMG 25547	Glaciimonas	immobilis
Z0019_LB_F08_1_B03_B	92	81	LMG 25547	Glaciimonas	immobilis
Z0019_RB_E07_2_B09_A	92	81	LMG 25547	Glaciimonas	immobilis
Z0019_RB_E08_2_B09_B	92	81	LMG 25547	Glaciimonas	immobilis
Z0019_RB_E09_2_B08_A	92	81	LMG 25547	Glaciimonas	immobilis
Z0019_RB_E10_2_B08_B	92	81	LMG 25547	Glaciimonas	immobilis
Z0019_RB_E11_2_B07_A	92	81	LMG 25547	Glaciimonas	immobilis
Z0019_RB_E12_2_B07_B	92	81	LMG 25547	Glaciimonas	immobilis
Z0019_RB_F01_1_B12_A	92	81	LMG 25547	Glaciimonas	immobilis
Z0019_RB_F02_1_B12_B	92	81	LMG 25547	Glaciimonas	immobilis

Z0019_RB_F03_1_B11_A	92	81	LMG 25547	Glaciimonas	immobilis
Z0019_RB_F04_1_B11_B	92	81	LMG 25547	Glaciimonas	immobilis
Z0019_RB_F05_1_B10_A	92	81	LMG 25547	Glaciimonas	immobilis
Z0019_RB_F06_1_B10_B	92	81	LMG 25547	Glaciimonas	immobilis
Z0019_RB_F07_1_B09_A	92	81	LMG 25547	Glaciimonas	immobilis
Z0019_RB_F08_1_B09_B	92	81	LMG 25547	Glaciimonas	immobilis
Z0019_RB_F09_1_B08_A	92	81	LMG 25547	Glaciimonas	immobilis
Z0019_RB_F10_1_B08_B	92	81	LMG 25547	Glaciimonas	immobilis
Z0019_RB_F11_1_B07_A	92	81	LMG 25547	Glaciimonas	immobilis
Z0019_RB_F12_1_B07_B	92	81	LMG 25547	Glaciimonas	immobilis
Z0019_RB_G01_2_A12_A	92	81	LMG 25547	Glaciimonas	immobilis
Z0019_LB_F09_1_B02_A	93	82	LMG 25664	Megasphaera	cerevisiae
Z0019_LB_F10_1_B02_B	93	82	LMG 25664	Megasphaera	cerevisiae
Z0019_LB_F11_1_B01_A	93	82	LMG 25664	Megasphaera	cerevisiae
Z0019_LB_F12_1_B01_B	93	82	LMG 25664	Megasphaera	cerevisiae
Z0019_LB_G01_2_A06_A	93	82	LMG 25664	Megasphaera	cerevisiae
Z0019_LB_G02_2_A06_B	93	82	LMG 25664	Megasphaera	cerevisiae
Z0019_LB_G03_2_A05_A	93	82	LMG 25664	Megasphaera	cerevisiae
Z0019_LB_G04_2_A05_B	93	82	LMG 25664	Megasphaera	cerevisiae
Z0019_LB_G05_2_A04_A	93	82	LMG 25664	Megasphaera	cerevisiae
Z0019_LB_G06_2_A04_B	93	82	LMG 25664	Megasphaera	cerevisiae
Z0019_LB_G07_2_A03_A	93	82	LMG 25664	Megasphaera	cerevisiae
Z0019_LB_G08_2_A03_B	93	82	LMG 25664	Megasphaera	cerevisiae
Z0019_LB_G09_2_A02_A	93	82	LMG 25664	Megasphaera	cerevisiae
Z0019_LB_G10_2_A02_B	93	82	LMG 25664	Megasphaera	cerevisiae
Z0019_LB_G11_2_A01_A	93	82	LMG 25664	Megasphaera	cerevisiae
Z0019_LB_G12_2_A01_B	93	82	LMG 25664	Megasphaera	cerevisiae
Z0019_LB_H01_1_A06_A	93	82	LMG 25664	Megasphaera	cerevisiae
Z0019_LB_H02_1_A06_B	93	82	LMG 25664	Megasphaera	cerevisiae
Z0019_RB_G02_2_A12_B	93	82	LMG 25664	Megasphaera	cerevisiae
Z0019_RB_G03_2_A11_A	93	82	LMG 25664	Megasphaera	cerevisiae
Z0019_RB_G04_2_A11_B	93	82	LMG 25664	Megasphaera	cerevisiae
Z0019_RB_G05_2_A10_A	93	82	LMG 25664	Megasphaera	cerevisiae
Z0019_RB_G06_2_A10_B	93	82	LMG 25664	Megasphaera	cerevisiae
Z0019_RB_G07_2_A09_A	93	82	LMG 25664	Megasphaera	cerevisiae
Z0019_RB_G08_2_A09_B	93	82	LMG 25664	Megasphaera	cerevisiae
Z0019_RB_G09_2_A08_A	93	82	LMG 25664	Megasphaera	cerevisiae
Z0019_RB_G10_2_A08_B	93	82	LMG 25664	Megasphaera	cerevisiae
Z0019_RB_G11_2_A07_A	93	82	LMG 25664	Megasphaera	cerevisiae
Z0019_RB_G12_2_A07_B	93	82	LMG 25664	Megasphaera	cerevisiae
Z0019_RB_H01_1_A12_A	93	82	LMG 25664	Megasphaera	cerevisiae
Z0019_RB_H02_1_A12_B	93	82	LMG 25664	Megasphaera	cerevisiae
Z0019_RB_H03_1_A11_A	93	82	LMG 25664	Megasphaera	cerevisiae
Z0019_LB_H03_1_A05_A	94	83	LMG 25773	Tabrizicola	aquatica
Z0019_LB_H04_1_A05_B	94	83	LMG 25773	Tabrizicola	aquatica
Z0019_LB_H05_1_A04_A	94	83	LMG 25773	Tabrizicola	aquatica
Z0019_LB_H06_1_A04_B	94	83	LMG 25773	Tabrizicola	aquatica
Z0019_LB_H07_1_A03_A	94	83	LMG 25773	Tabrizicola	aquatica
Z0019_LB_H08_1_A03_B	94	83	LMG 25773	Tabrizicola	aquatica
Z0019_LB_H09_1_A02_A	94	83	LMG 25773	Tabrizicola	aquatica

Z0019_LB_H10_1_A02_B	94	83	LMG 25773	Tabrizicola	aquatica
Z0019_LB_H11_1_A01_A	94	83	LMG 25773	Tabrizicola	aquatica
Z0019_LB_H12_1_A01_B	94	83	LMG 25773	Tabrizicola	aquatica
Z0019_LO_A01_1_E01_A	94	83	LMG 25773	Tabrizicola	aquatica
Z0019_LO_A02_1_E01_B	94	83	LMG 25773	Tabrizicola	aquatica
Z0019_LO_A03_1_E02_A	94	83	LMG 25773	Tabrizicola	aquatica
Z0019_LO_A04_1_E02_B	94	83	LMG 25773	Tabrizicola	aquatica
Z0019_LO_A05_1_E03_A	94	83	LMG 25773	Tabrizicola	aquatica
Z0019_LO_A06_1_E03_B	94	83	LMG 25773	Tabrizicola	aquatica
Z0019_LO_A08_1_E04_B	94	83	LMG 25773	Tabrizicola	aquatica
Z0019_RB_H04_1_A11_B	94	83	LMG 25773	Tabrizicola	aquatica
Z0019_RB_H05_1_A10_A	94	83	LMG 25773	Tabrizicola	aquatica
Z0019_RB_H09_1_A08_A	94	83	LMG 25773	Tabrizicola	aquatica
Z0019_RO_A03_1_E08_A	94	83	LMG 25773	Tabrizicola	aquatica
Z0019_RO_A04_1_E08_B	94	83	LMG 25773	Tabrizicola	aquatica
Z0019_RO_A05_1_E09_A	94	83	LMG 25773	Tabrizicola	aquatica
Z0019_RO_A06_1_E09_B	94	83	LMG 25773	Tabrizicola	aquatica
Z0019_RO_A07_1_E10_A	94	83	LMG 25773	Tabrizicola	aquatica
Z0019_RO_A08_1_E10_B	94	83	LMG 25773	Tabrizicola	aquatica
Z0019_RO_A09_1_E11_A	94	83	LMG 25773	Tabrizicola	aquatica
Z0019_RO_A11_1_E12_A	94	83	LMG 25773	Tabrizicola	aquatica
Z0019_LO_A09_1_E05_A	95	84	LMG 26041	Tetragenococcus	osmophilus
Z0019_LO_A12_1_E06_B	95	84	LMG 26041	Tetragenococcus	osmophilus
Z0019_LO_B01_2_E01_A	95	84	LMG 26041	Tetragenococcus	osmophilus
Z0019_LO_B03_2_E02_A	95	84	LMG 26041	Tetragenococcus	osmophilus
Z0019_LO_B05_2_E03_A	95	84	LMG 26041	Tetragenococcus	osmophilus
Z0019_LO_B07_2_E04_A	95	84	LMG 26041	Tetragenococcus	osmophilus
Z0019_LO_C03_1_F02_A	95	84	LMG 26041	Tetragenococcus	osmophilus
Z0019_LO_C04_1_F02_B	95	84	LMG 26041	Tetragenococcus	osmophilus
Z0019_LO_C05_1_F03_A	95	84	LMG 26041	Tetragenococcus	osmophilus
Z0019_LO_C06_1_F03_B	95	84	LMG 26041	Tetragenococcus	osmophilus
Z0019_LO_C08_1_F04_B	95	84	LMG 26041	Tetragenococcus	osmophilus
Z0019_RO_B01_2_E07_A	95	84	LMG 26041	Tetragenococcus	osmophilus
Z0019_RO_B02_2_E07_B	95	84	LMG 26041	Tetragenococcus	osmophilus
Z0019_RO_B03_2_E08_A	95	84	LMG 26041	Tetragenococcus	osmophilus
Z0019_RO_B05_2_E09_A	95	84	LMG 26041	Tetragenococcus	osmophilus
Z0019_RO_B06_2_E09_B	95	84	LMG 26041	Tetragenococcus	osmophilus
Z0019_RO_B07_2_E10_A	95	84	LMG 26041	Tetragenococcus	osmophilus
Z0019_RO_B08_2_E10_B	95	84	LMG 26041	Tetragenococcus	osmophilus
Z0019_RO_B10_2_E11_B	95	84	LMG 26041	Tetragenococcus	osmophilus
Z0019_RO_B11_2_E12_A	95	84	LMG 26041	Tetragenococcus	osmophilus
Z0019_RO_B12_2_E12_B	95	84	LMG 26041	Tetragenococcus	osmophilus
Z0019_RO_C01_1_F07_A	95	84	LMG 26041	Tetragenococcus	osmophilus
Z0019_RO_C02_1_F07_B	95	84	LMG 26041	Tetragenococcus	osmophilus
Z0019_RO_C03_1_F08_A	95	84	LMG 26041	Tetragenococcus	osmophilus
Z0019_RO_C04_1_F08_B	95	84	LMG 26041	Tetragenococcus	osmophilus
Z0019_RO_C05_1_F09_A	95	84	LMG 26041	Tetragenococcus	osmophilus
Z0019_RO_C10_1_F11_B	95	84	LMG 26041	Tetragenococcus	osmophilus
Z0019_RO_C11_1_F12_A	95	84	LMG 26041	Tetragenococcus	osmophilus
Z0019_LO_A10_1_E05_B	96	88	LMG 26187	Halomonas	ventosae

Z0019_LO_B06_2_E03_B	96	88	LMG 26187	Halomonas	ventosae
Z0019_LO_B08_2_E04_B	96	88	LMG 26187	Halomonas	ventosae
Z0019_LO_B09_2_E05_A	96	88	LMG 26187	Halomonas	ventosae
Z0019_LO_B10_2_E05_B	96	88	LMG 26187	Halomonas	ventosae
Z0019_LO_B11_2_E06_A	96	88	LMG 26187	Halomonas	ventosae
Z0019_LO_B12_2_E06_B	96	88	LMG 26187	Halomonas	ventosae
Z0019_LO_C01_1_F01_A	96	88	LMG 26187	Halomonas	ventosae
Z0019_LO_C07_1_F04_A	96	88	LMG 26187	Halomonas	ventosae
Z0019_LO_C09_1_F05_A	96	88	LMG 26187	Halomonas	ventosae
Z0019_LO_C10_1_F05_B	96	88	LMG 26187	Halomonas	ventosae
Z0019_LO_D01_2_F01_A	96	88	LMG 26187	Halomonas	ventosae
Z0019_LO_D02_2_F01_B	96	88	LMG 26187	Halomonas	ventosae
Z0019_LO_D03_2_F02_A	96	88	LMG 26187	Halomonas	ventosae
Z0019_LO_D04_2_F02_B	96	88	LMG 26187	Halomonas	ventosae
Z0019_LO_D07_2_F04_A	96	88	LMG 26187	Halomonas	ventosae
Z0019_LO_D08_2_F04_B	96	88	LMG 26187	Halomonas	ventosae
Z0019_RB_H06_1_A10_B	96	88	LMG 26187	Halomonas	ventosae
Z0019_RB_H07_1_A09_A	96	88	LMG 26187	Halomonas	ventosae
Z0019_RB_H08_1_A09_B	96	88	LMG 26187	Halomonas	ventosae
Z0019_RB_H10_1_A08_B	96	88	LMG 26187	Halomonas	ventosae
Z0019_RB_H11_1_A07_A	96	88	LMG 26187	Halomonas	ventosae
Z0019_RB_H12_1_A07_B	96	88	LMG 26187	Halomonas	ventosae
Z0019_RO_A01_1_E07_A	96	88	LMG 26187	Halomonas	ventosae
Z0019_RO_A02_1_E07_B	96	88	LMG 26187	Halomonas	ventosae
Z0019_RO_A10_1_E11_B	96	88	LMG 26187	Halomonas	ventosae
Z0019_RO_A12_1_E12_B	96	88	LMG 26187	Halomonas	ventosae
Z0019_RO_B04_2_E08_B	96	88	LMG 26187	Halomonas	ventosae
Z0019_RO_C06_1_F09_B	96	88	LMG 26187	Halomonas	ventosae
Z0019_RO_C07_1_F10_A	96	88	LMG 26187	Halomonas	ventosae
Z0019_RO_C12_1_F12_B	96	88	LMG 26187	Halomonas	ventosae
Z0019_RO_D05_2_F09_A	96	88	LMG 26187	Halomonas	ventosae
Z0019_LO_C11_1_F06_A	97	85	LMG 26064	Enterobacter	asburiae
Z0019_LO_C12_1_F06_B	97	85	LMG 26064	Enterobacter	asburiae
Z0019_LO_D05_2_F03_A	97	85	LMG 26064	Enterobacter	asburiae
Z0019_LO_D06_2_F03_B	97	85	LMG 26064	Enterobacter	asburiae
Z0019_LO_D09_2_F05_A	97	85	LMG 26064	Enterobacter	asburiae
Z0019_LO_D10_2_F05_B	97	85	LMG 26064	Enterobacter	asburiae
Z0019_LO_D12_2_F06_B	97	85	LMG 26064	Enterobacter	asburiae
Z0019_LO_E02_1_G01_B	97	85	LMG 26064	Enterobacter	asburiae
Z0019_LO_E04_1_G02_B	97	85	LMG 26064	Enterobacter	asburiae
Z0019_LO_E06_1_G03_B	97	85	LMG 26064	Enterobacter	asburiae
Z0019_RO_D01_2_F07_A	97	85	LMG 26064	Enterobacter	asburiae
Z0019_RO_D02_2_F07_B	97	85	LMG 26064	Enterobacter	asburiae
Z0019_RO_D03_2_F08_A	97	85	LMG 26064	Enterobacter	asburiae
Z0019_RO_D06_2_F09_B	97	85	LMG 26064	Enterobacter	asburiae
Z0019_RO_D07_2_F10_A	97	85	LMG 26064	Enterobacter	asburiae
Z0019_RO_D08_2_F10_B	97	85	LMG 26064	Enterobacter	asburiae
Z0019_RO_D09_2_F11_A	97	85	LMG 26064	Enterobacter	asburiae
Z0019_RO_D12_2_F12_B	97	85	LMG 26064	Enterobacter	asburiae
Z0019_RO_E01_1_G07_A	97	85	LMG 26064	Enterobacter	asburiae

Z0019_RO_E04_1_G08_B	97	85	LMG 26064	Enterobacter	asburiae
Z0019_RO_E06_1_G09_B	97	85	LMG 26064	Enterobacter	asburiae
Z0019_RO_E08_1_G10_B	97	85	LMG 26064	Enterobacter	asburiae
Z0019_RO_E10_1_G11_B	97	85	LMG 26064	Enterobacter	asburiae
Z0019_RO_E12_1_G12_B	97	85	LMG 26064	Enterobacter	asburiae
Z0019_RO_F01_2_G07_A	97	85	LMG 26064	Enterobacter	asburiae
Z0019_RO_F02_2_G07_B	97	85	LMG 26064	Enterobacter	asburiae
Z0019_RO_F03_2_G08_A	97	85	LMG 26064	Enterobacter	asburiae
Z0019_RO_F04_2_G08_B	97	85	LMG 26064	Enterobacter	asburiae
Z0019_RO_F05_2_G09_A	97	85	LMG 26064	Enterobacter	asburiae
Z0019_RO_F06_2_G09_B	97	85	LMG 26064	Enterobacter	asburiae
Z0019_RO_F07_2_G10_A	97	85	LMG 26064	Enterobacter	asburiae
Z0019_RO_F08_2_G10_B	97	85	LMG 26064	Enterobacter	asburiae
Z0019_LO_D11_2_F06_A	98	87	LMG 26149	Herbaspirillum	soli
Z0019_LO_E01_1_G01_A	98	87	LMG 26149	Herbaspirillum	soli
Z0019_LO_E03_1_G02_A	98	87	LMG 26149	Herbaspirillum	soli
Z0019_LO_E05_1_G03_A	98	87	LMG 26149	Herbaspirillum	soli
Z0019_LO_E07_1_G04_A	98	87	LMG 26149	Herbaspirillum	soli
Z0019_LO_E08_1_G04_B	98	87	LMG 26149	Herbaspirillum	soli
Z0019_LO_E09_1_G05_A	98	87	LMG 26149	Herbaspirillum	soli
Z0019_LO_E10_1_G05_B	98	87	LMG 26149	Herbaspirillum	soli
Z0019_LO_E11_1_G06_A	98	87	LMG 26149	Herbaspirillum	soli
Z0019_LO_E12_1_G06_B	98	87	LMG 26149	Herbaspirillum	soli
Z0019_LO_F01_2_G01_A	98	87	LMG 26149	Herbaspirillum	soli
Z0019_LO_F02_2_G01_B	98	87	LMG 26149	Herbaspirillum	soli
Z0019_LO_F03_2_G02_A	98	87	LMG 26149	Herbaspirillum	soli
Z0019_LO_F04_2_G02_B	98	87	LMG 26149	Herbaspirillum	soli
Z0019_LO_F05_2_G03_A	98	87	LMG 26149	Herbaspirillum	soli
Z0019_LO_F12_2_G06_B	98	87	LMG 26149	Herbaspirillum	soli
Z0019_LO_G04_1_H02_B	98	87	LMG 26149	Herbaspirillum	soli
Z0019_RO_D04_2_F08_B	98	87	LMG 26149	Herbaspirillum	soli
Z0019_RO_D10_2_F11_B	98	87	LMG 26149	Herbaspirillum	soli
Z0019_RO_D11_2_F12_A	98	87	LMG 26149	Herbaspirillum	soli
Z0019_RO_E02_1_G07_B	98	87	LMG 26149	Herbaspirillum	soli
Z0019_RO_E03_1_G08_A	98	87	LMG 26149	Herbaspirillum	soli
Z0019_RO_E05_1_G09_A	98	87	LMG 26149	Herbaspirillum	soli
Z0019_RO_E07_1_G10_A	98	87	LMG 26149	Herbaspirillum	soli
Z0019_RO_E09_1_G11_A	98	87	LMG 26149	Herbaspirillum	soli
Z0019_RO_E11_1_G12_A	98	87	LMG 26149	Herbaspirillum	soli
Z0019_RO_F09_2_G11_A	98	87	LMG 26149	Herbaspirillum	soli
Z0019_RO_F10_2_G11_B	98	87	LMG 26149	Herbaspirillum	soli
Z0019_RO_F11_2_G12_A	98	87	LMG 26149	Herbaspirillum	soli
Z0019_RO_F12_2_G12_B	98	87	LMG 26149	Herbaspirillum	soli
Z0019_RO_G01_1_H07_A	98	87	LMG 26149	Herbaspirillum	soli
Z0019_RO_G02_1_H07_B	98	87	LMG 26149	Herbaspirillum	soli
Z0019_LO_F06_2_G03_B	99	86	LMG 26121	Rosenbergiella	nectarea
Z0019_LO_F07_2_G04_A	99	86	LMG 26121	Rosenbergiella	nectarea
Z0019_LO_F08_2_G04_B	99	86	LMG 26121	Rosenbergiella	nectarea
Z0019_LO_F09_2_G05_A	99	86	LMG 26121	Rosenbergiella	nectarea
Z0019_LO_F10_2_G05_B	99	86	LMG 26121	Rosenbergiella	nectarea

Z0019_LO_F11_2_G06_A	99	86	LMG 26121	Rosenbergiella	nectarea
Z0019_LO_G01_1_H01_A	99	86	LMG 26121	Rosenbergiella	nectarea
Z0019_LO_G02_1_H01_B	99	86	LMG 26121	Rosenbergiella	nectarea
Z0019_LO_G03_1_H02_A	99	86	LMG 26121	Rosenbergiella	nectarea
Z0019_LO_G05_1_H03_A	99	86	LMG 26121	Rosenbergiella	nectarea
Z0019_LO_G06_1_H03_B	99	86	LMG 26121	Rosenbergiella	nectarea
Z0019_LO_G07_1_H04_A	99	86	LMG 26121	Rosenbergiella	nectarea
Z0019_LO_G08_1_H04_B	99	86	LMG 26121	Rosenbergiella	nectarea
Z0019_LO_G09_1_H05_A	99	86	LMG 26121	Rosenbergiella	nectarea
Z0019_LO_G10_1_H05_B	99	86	LMG 26121	Rosenbergiella	nectarea
Z0019_LO_G11_1_H06_A	99	86	LMG 26121	Rosenbergiella	nectarea
Z0019_LO_G12_1_H06_B	99	86	LMG 26121	Rosenbergiella	nectarea
Z0019_LO_H01_2_H01_A	99	86	LMG 26121	Rosenbergiella	nectarea
Z0019_RO_G03_1_H08_A	99	86	LMG 26121	Rosenbergiella	nectarea
Z0019_RO_G04_1_H08_B	99	86	LMG 26121	Rosenbergiella	nectarea
Z0019_RO_G05_1_H09_A	99	86	LMG 26121	Rosenbergiella	nectarea
Z0019_RO_G06_1_H09_B	99	86	LMG 26121	Rosenbergiella	nectarea
Z0019_RO_G07_1_H10_A	99	86	LMG 26121	Rosenbergiella	nectarea
Z0019_RO_G08_1_H10_B	99	86	LMG 26121	Rosenbergiella	nectarea
Z0019_RO_G09_1_H11_A	99	86	LMG 26121	Rosenbergiella	nectarea
Z0019_RO_G10_1_H11_B	99	86	LMG 26121	Rosenbergiella	nectarea
Z0019_RO_G11_1_H12_A	99	86	LMG 26121	Rosenbergiella	nectarea
Z0019_RO_G12_1_H12_B	99	86	LMG 26121	Rosenbergiella	nectarea
Z0019_RO_H01_2_H07_A	99	86	LMG 26121	Rosenbergiella	nectarea
Z0019_RO_H02_2_H07_B	99	86	LMG 26121	Rosenbergiella	nectarea
Z0019_RO_H03_2_H08_A	99	86	LMG 26121	Rosenbergiella	nectarea
Z0019_RO_H04_2_H08_B	99	86	LMG 26121	Rosenbergiella	nectarea
Z0020_LB_A01_2_D06_A	100	89	LMG 26195	Paracoccus	sp.
Z0020_LB_A02_2_D06_B	100	89	LMG 26195	Paracoccus	sp.
Z0020_LB_A03_2_D05_A	100	89	LMG 26195	Paracoccus	sp.
Z0020_LB_A04_2_D05_B	100	89	LMG 26195	Paracoccus	sp.
Z0020_LB_A05_2_D04_A	100	89	LMG 26195	Paracoccus	sp.
Z0020_LB_A06_2_D04_B	100	89	LMG 26195	Paracoccus	sp.
Z0020_LB_A07_2_D03_A	100	89	LMG 26195	Paracoccus	sp.
Z0020_LB_A08_2_D03_B	100	89	LMG 26195	Paracoccus	sp.
Z0020_LB_A09_2_D02_A	100	89	LMG 26195	Paracoccus	sp.
Z0020_LB_A10_2_D02_B	100	89	LMG 26195	Paracoccus	sp.
Z0020_LB_A11_2_D01_A	100	89	LMG 26195	Paracoccus	sp.
Z0020_LB_A12_2_D01_B	100	89	LMG 26195	Paracoccus	sp.
Z0020_LO_B09_2_E05_A	100	89	LMG 26195	Paracoccus	sp.
Z0020_LO_G10_1_H05_B	100	89	LMG 26195	Paracoccus	sp.
Z0020_LO_G11_1_H06_A	100	89	LMG 26195	Paracoccus	sp.
Z0020_LO_G12_1_H06_B	100	89	LMG 26195	Paracoccus	sp.
Z0020_LO_H01_2_H01_A	100	89	LMG 26195	Paracoccus	sp.
Z0020_LO_H02_2_H01_B	100	89	LMG 26195	Paracoccus	sp.
Z0020_LO_H03_2_H02_A	100	89	LMG 26195	Paracoccus	sp.
Z0020_LO_H04_2_H02_B	100	89	LMG 26195	Paracoccus	sp.
Z0020_LO_H05_2_H03_A	100	89	LMG 26195	Paracoccus	sp.
Z0020_LO_H06_2_H03_B	100	89	LMG 26195	Paracoccus	sp.
Z0020_LO_H07_2_H04_A	100	89	LMG 26195	Paracoccus	sp.

Z0020_LO_H08_2_H04_B	100	89	LMG 26195	Paracoccus	sp.
Z0020_LO_H09_2_H05_A	100	89	LMG 26195	Paracoccus	sp.
Z0020_RB_H04_1_A11_B	100	89	LMG 26195	Paracoccus	sp.
Z0020_RB_H08_1_A09_B	100	89	LMG 26195	Paracoccus	sp.
Z0020_RO_A01_1_E07_A	100	89	LMG 26195	Paracoccus	sp.
Z0020_RO_A04_1_E08_B	100	89	LMG 26195	Paracoccus	sp.
Z0020_RO_A06_1_E09_B	100	89	LMG 26195	Paracoccus	sp.
Z0020_RO_B05_2_E09_A	100	89	LMG 26195	Paracoccus	sp.
Z0020_RO_B07_2_E10_A	100	89	LMG 26195	Paracoccus	sp.
Z0020_LB_D06_1_C04_B	101	90	LMG 26304	Enterococcus	ureasiticus
Z0020_LB_D07_1_C03_A	101	90	LMG 26304	Enterococcus	ureasiticus
Z0020_LB_D08_1_C03_B	101	90	LMG 26304	Enterococcus	ureasiticus
Z0020_LB_D09_1_C02_A	101	90	LMG 26304	Enterococcus	ureasiticus
Z0020_LB_D10_1_C02_B	101	90	LMG 26304	Enterococcus	ureasiticus
Z0020_LB_D11_1_C01_A	101	90	LMG 26304	Enterococcus	ureasiticus
Z0020_LB_D12_1_C01_B	101	90	LMG 26304	Enterococcus	ureasiticus
Z0020_LB_E01_2_B06_A	101	90	LMG 26304	Enterococcus	ureasiticus
Z0020_LB_E02_2_B06_B	101	90	LMG 26304	Enterococcus	ureasiticus
Z0020_LB_E03_2_B05_A	101	90	LMG 26304	Enterococcus	ureasiticus
Z0020_LB_E04_2_B05_B	101	90	LMG 26304	Enterococcus	ureasiticus
Z0020_LB_E05_2_B04_A	101	90	LMG 26304	Enterococcus	ureasiticus
Z0020_LB_E06_2_B04_B	101	90	LMG 26304	Enterococcus	ureasiticus
Z0020_RB_C09_2_C08_A	101	90	LMG 26304	Enterococcus	ureasiticus
Z0020_RB_C10_2_C08_B	101	90	LMG 26304	Enterococcus	ureasiticus
Z0020_RB_C11_2_C07_A	101	90	LMG 26304	Enterococcus	ureasiticus
Z0020_RB_C12_2_C07_B	101	90	LMG 26304	Enterococcus	ureasiticus
Z0020_RB_D01_1_C12_A	101	90	LMG 26304	Enterococcus	ureasiticus
Z0020_RB_D02_1_C12_B	101	90	LMG 26304	Enterococcus	ureasiticus
Z0020_RB_D03_1_C11_A	101	90	LMG 26304	Enterococcus	ureasiticus
Z0020_RB_D04_1_C11_B	101	90	LMG 26304	Enterococcus	ureasiticus
Z0020_RB_D05_1_C10_A	101	90	LMG 26304	Enterococcus	ureasiticus
Z0020_RB_D06_1_C10_B	101	90	LMG 26304	Enterococcus	ureasiticus
Z0020_RB_D07_1_C09_A	101	90	LMG 26304	Enterococcus	ureasiticus
Z0020_RB_D08_1_C09_B	101	90	LMG 26304	Enterococcus	ureasiticus
Z0020_RB_D09_1_C08_A	101	90	LMG 26304	Enterococcus	ureasiticus
Z0020_RB_D10_1_C08_B	101	90	LMG 26304	Enterococcus	ureasiticus
Z0020_RB_D11_1_C07_A	101	90	LMG 26304	Enterococcus	ureasiticus
Z0020_RB_D12_1_C07_B	101	90	LMG 26304	Enterococcus	ureasiticus
Z0020_RB_E01_2_B12_A	101	90	LMG 26304	Enterococcus	ureasiticus
Z0020_RB_E02_2_B12_B	101	90	LMG 26304	Enterococcus	ureasiticus
Z0020_RB_E03_2_B11_A	101	90	LMG 26304	Enterococcus	ureasiticus
Z0020_LB_E07_2_B03_A	102	4	LMG 26367	Lactobacillus	plantarum
Z0020_LB_E08_2_B03_B	102	4	LMG 26367	Lactobacillus	plantarum
Z0020_LB_E09_2_B02_A	102	4	LMG 26367	Lactobacillus	plantarum
Z0020_LB_E10_2_B02_B	102	4	LMG 26367	Lactobacillus	plantarum
Z0020_LB_E11_2_B01_A	102	4	LMG 26367	Lactobacillus	plantarum
Z0020_LB_E12_2_B01_B	102	4	LMG 26367	Lactobacillus	plantarum
Z0020_LB_F01_1_B06_A	102	4	LMG 26367	Lactobacillus	plantarum
Z0020_LB_F02_1_B06_B	102	4	LMG 26367	Lactobacillus	plantarum
Z0020_LB_F03_1_B05_A	102	4	LMG 26367	Lactobacillus	plantarum

Z0020_LB_F04_1_B05_B	102	4	LMG 26367	Lactobacillus	plantarum
Z0020_LB_F05_1_B04_A	102	4	LMG 26367	Lactobacillus	plantarum
Z0020_LB_F06_1_B04_B	102	4	LMG 26367	Lactobacillus	plantarum
Z0020_LB_F07_1_B03_A	102	4	LMG 26367	Lactobacillus	plantarum
Z0020_LB_F08_1_B03_B	102	4	LMG 26367	Lactobacillus	plantarum
Z0020_LB_F09_1_B02_A	102	4	LMG 26367	Lactobacillus	plantarum
Z0020_LB_F10_1_B02_B	102	4	LMG 26367	Lactobacillus	plantarum
Z0020_LB_F11_1_B01_A	102	4	LMG 26367	Lactobacillus	plantarum
Z0020_LB_F12_1_B01_B	102	4	LMG 26367	Lactobacillus	plantarum
Z0020_LB_G01_2_A06_A	102	4	LMG 26367	Lactobacillus	plantarum
Z0020_RB_E04_2_B11_B	102	4	LMG 26367	Lactobacillus	plantarum
Z0020_RB_E05_2_B10_A	102	4	LMG 26367	Lactobacillus	plantarum
Z0020_RB_E06_2_B10_B	102	4	LMG 26367	Lactobacillus	plantarum
Z0020_RB_E07_2_B09_A	102	4	LMG 26367	Lactobacillus	plantarum
Z0020_RB_E08_2_B09_B	102	4	LMG 26367	Lactobacillus	plantarum
Z0020_RB_E09_2_B08_A	102	4	LMG 26367	Lactobacillus	plantarum
Z0020_RB_E10_2_B08_B	102	4	LMG 26367	Lactobacillus	plantarum
Z0020_RB_E11_2_B07_A	102	4	LMG 26367	Lactobacillus	plantarum
Z0020_RB_E12_2_B07_B	102	4	LMG 26367	Lactobacillus	plantarum
Z0020_RB_F01_1_B12_A	102	4	LMG 26367	Lactobacillus	plantarum
Z0020_RB_F02_1_B12_B	102	4	LMG 26367	Lactobacillus	plantarum
Z0020_RB_F03_1_B11_A	102	4	LMG 26367	Lactobacillus	plantarum
Z0020_RB_F04_1_B11_B	102	4	LMG 26367	Lactobacillus	plantarum
Z0020_LB_G02_2_A06_B	103	91	LMG 26467	Tardiphaga	robiniae
Z0020_LB_G03_2_A05_A	103	91	LMG 26467	Tardiphaga	robiniae
Z0020_LB_G04_2_A05_B	103	91	LMG 26467	Tardiphaga	robiniae
Z0020_LB_G05_2_A04_A	103	91	LMG 26467	Tardiphaga	robiniae
Z0020_LB_G06_2_A04_B	103	91	LMG 26467	Tardiphaga	robiniae
Z0020_LB_G07_2_A03_A	103	91	LMG 26467	Tardiphaga	robiniae
Z0020_LB_G08_2_A03_B	103	91	LMG 26467	Tardiphaga	robiniae
Z0020_LB_G09_2_A02_A	103	91	LMG 26467	Tardiphaga	robiniae
Z0020_LB_G10_2_A02_B	103	91	LMG 26467	Tardiphaga	robiniae
Z0020_LB_G11_2_A01_A	103	91	LMG 26467	Tardiphaga	robiniae
Z0020_LB_G12_2_A01_B	103	91	LMG 26467	Tardiphaga	robiniae
Z0020_LB_H01_1_A06_A	103	91	LMG 26467	Tardiphaga	robiniae
Z0020_LB_H02_1_A06_B	103	91	LMG 26467	Tardiphaga	robiniae
Z0020_RB_F05_1_B10_A	103	91	LMG 26467	Tardiphaga	robiniae
Z0020_RB_F06_1_B10_B	103	91	LMG 26467	Tardiphaga	robiniae
Z0020_RB_F07_1_B09_A	103	91	LMG 26467	Tardiphaga	robiniae
Z0020_RB_F08_1_B09_B	103	91	LMG 26467	Tardiphaga	robiniae
Z0020_RB_F09_1_B08_A	103	91	LMG 26467	Tardiphaga	robiniae
Z0020_RB_F10_1_B08_B	103	91	LMG 26467	Tardiphaga	robiniae
Z0020_RB_F11_1_B07_A	103	91	LMG 26467	Tardiphaga	robiniae
Z0020_RB_F12_1_B07_B	103	91	LMG 26467	Tardiphaga	robiniae
Z0020_RB_G01_2_A12_A	103	91	LMG 26467	Tardiphaga	robiniae
Z0020_RB_G02_2_A12_B	103	91	LMG 26467	Tardiphaga	robiniae
Z0020_RB_G03_2_A11_A	103	91	LMG 26467	Tardiphaga	robiniae
Z0020_RB_G04_2_A11_B	103	91	LMG 26467	Tardiphaga	robiniae
Z0020_RB_G05_2_A10_A	103	91	LMG 26467	Tardiphaga	robiniae
Z0020_RB_G06_2_A10_B	103	91	LMG 26467	Tardiphaga	robiniae

Z0020_RB_G07_2_A09_A	103	91	LMG 26467	Tardiphaga	robiniae
Z0020_RB_G08_2_A09_B	103	91	LMG 26467	Tardiphaga	robiniae
Z0020_RB_G09_2_A08_A	103	91	LMG 26467	Tardiphaga	robiniae
Z0020_RB_G10_2_A08_B	103	91	LMG 26467	Tardiphaga	robiniae
Z0020_RB_G11_2_A07_A	103	91	LMG 26467	Tardiphaga	robiniae
Z0020_LB_H03_1_A05_A	104	92	LMG 26473	Alishewanella	tabrizica
Z0020_LB_H04_1_A05_B	104	92	LMG 26473	Alishewanella	tabrizica
Z0020_LB_H06_1_A04_B	104	92	LMG 26473	Alishewanella	tabrizica
Z0020_LB_H10_1_A02_B	104	92	LMG 26473	Alishewanella	tabrizica
Z0020_LB_H12_1_A01_B	104	92	LMG 26473	Alishewanella	tabrizica
Z0020_LO_A01_1_E01_A	104	92	LMG 26473	Alishewanella	tabrizica
Z0020_LO_A02_1_E01_B	104	92	LMG 26473	Alishewanella	tabrizica
Z0020_LO_A03_1_E02_A	104	92	LMG 26473	Alishewanella	tabrizica
Z0020_LO_A05_1_E03_A	104	92	LMG 26473	Alishewanella	tabrizica
Z0020_LO_A06_1_E03_B	104	92	LMG 26473	Alishewanella	tabrizica
Z0020_RB_G12_2_A07_B	104	92	LMG 26473	Alishewanella	tabrizica
Z0020_RB_H01_1_A12_A	104	92	LMG 26473	Alishewanella	tabrizica
Z0020_RB_H02_1_A12_B	104	92	LMG 26473	Alishewanella	tabrizica
Z0020_RB_H03_1_A11_A	104	92	LMG 26473	Alishewanella	tabrizica
Z0020_RB_H05_1_A10_A	104	92	LMG 26473	Alishewanella	tabrizica
Z0020_RB_H06_1_A10_B	104	92	LMG 26473	Alishewanella	tabrizica
Z0020_RB_H07_1_A09_A	104	92	LMG 26473	Alishewanella	tabrizica
Z0020_RB_H09_1_A08_A	104	92	LMG 26473	Alishewanella	tabrizica
Z0020_RB_H10_1_A08_B	104	92	LMG 26473	Alishewanella	tabrizica
Z0020_RB_H11_1_A07_A	104	92	LMG 26473	Alishewanella	tabrizica
Z0020_RB_H12_1_A07_B	104	92	LMG 26473	Alishewanella	tabrizica
Z0020_RO_A02_1_E07_B	104	92	LMG 26473	Alishewanella	tabrizica
Z0020_RO_A03_1_E08_A	104	92	LMG 26473	Alishewanella	tabrizica
Z0020_RO_A05_1_E09_A	104	92	LMG 26473	Alishewanella	tabrizica
Z0020_RO_A07_1_E10_A	104	92	LMG 26473	Alishewanella	tabrizica
Z0020_RO_A09_1_E11_A	104	92	LMG 26473	Alishewanella	tabrizica
Z0020_RO_A10_1_E11_B	104	92	LMG 26473	Alishewanella	tabrizica
Z0020_RO_A12_1_E12_B	104	92	LMG 26473	Alishewanella	tabrizica
Z0020_LB_H05_1_A04_A	105	96	LMG 27019	Kozakia	baliensis
Z0020_LB_H07_1_A03_A	105	96	LMG 27019	Kozakia	baliensis
Z0020_LB_H08_1_A03_B	105	96	LMG 27019	Kozakia	baliensis
Z0020_LB_H09_1_A02_A	105	96	LMG 27019	Kozakia	baliensis
Z0020_LB_H11_1_A01_A	105	96	LMG 27019	Kozakia	baliensis
Z0020_LO_A04_1_E02_B	105	96	LMG 27019	Kozakia	baliensis
Z0020_LO_A09_1_E05_A	105	96	LMG 27019	Kozakia	baliensis
Z0020_LO_A10_1_E05_B	105	96	LMG 27019	Kozakia	baliensis
Z0020_LO_B01_2_E01_A	105	96	LMG 27019	Kozakia	baliensis
Z0020_LO_B02_2_E01_B	105	96	LMG 27019	Kozakia	baliensis
Z0020_LO_B03_2_E02_A	105	96	LMG 27019	Kozakia	baliensis
Z0020_LO_B06_2_E03_B	105	96	LMG 27019	Kozakia	baliensis
Z0020_LO_B12_2_E06_B	105	96	LMG 27019	Kozakia	baliensis
Z0020_LO_C06_1_F03_B	105	96	LMG 27019	Kozakia	baliensis
Z0020_LO_C10_1_F05_B	105	96	LMG 27019	Kozakia	baliensis
Z0020_LO_C12_1_F06_B	105	96	LMG 27019	Kozakia	baliensis
Z0020_LO_D03_2_F02_A	105	96	LMG 27019	Kozakia	baliensis

Z0020_RO_A08_1_E10_B	105	96	LMG 27019	Kozakia	baliensis
Z0020_RO_A11_1_E12_A	105	96	LMG 27019	Kozakia	baliensis
Z0020_RO_B01_2_E07_A	105	96	LMG 27019	Kozakia	baliensis
Z0020_RO_C01_1_F07_A	105	96	LMG 27019	Kozakia	baliensis
Z0020_RO_C04_1_F08_B	105	96	LMG 27019	Kozakia	baliensis
Z0020_RO_C06_1_F09_B	105	96	LMG 27019	Kozakia	baliensis
Z0020_RO_C07_1_F10_A	105	96	LMG 27019	Kozakia	baliensis
Z0020_RO_C09_1_F11_A	105	96	LMG 27019	Kozakia	baliensis
Z0020_RO_C10_1_F11_B	105	96	LMG 27019	Kozakia	baliensis
Z0020_RO_C11_1_F12_A	105	96	LMG 27019	Kozakia	baliensis
Z0020_RO_C12_1_F12_B	105	96	LMG 27019	Kozakia	baliensis
Z0020_RO_D02_2_F07_B	105	96	LMG 27019	Kozakia	baliensis
Z0020_RO_D03_2_F08_A	105	96	LMG 27019	Kozakia	baliensis
Z0020_RO_D04_2_F08_B	105	96	LMG 27019	Kozakia	baliensis
Z0020_RO_D06_2_F09_B	105	96	LMG 27019	Kozakia	baliensis
Z0020_LO_A07_1_E04_A	106	93	LMG 26586	Eilatimonas	milleporae
Z0020_LO_A08_1_E04_B	106	93	LMG 26586	Eilatimonas	milleporae
Z0020_LO_A11_1_E06_A	106	93	LMG 26586	Eilatimonas	milleporae
Z0020_LO_A12_1_E06_B	106	93	LMG 26586	Eilatimonas	milleporae
Z0020_LO_B04_2_E02_B	106	93	LMG 26586	Eilatimonas	milleporae
Z0020_LO_B05_2_E03_A	106	93	LMG 26586	Eilatimonas	milleporae
Z0020_LO_B07_2_E04_A	106	93	LMG 26586	Eilatimonas	milleporae
Z0020_LO_B08_2_E04_B	106	93	LMG 26586	Eilatimonas	milleporae
Z0020_LO_B10_2_E05_B	106	93	LMG 26586	Eilatimonas	milleporae
Z0020_LO_B11_2_E06_A	106	93	LMG 26586	Eilatimonas	milleporae
Z0020_LO_C01_1_F01_A	106	93	LMG 26586	Eilatimonas	milleporae
Z0020_LO_C02_1_F01_B	106	93	LMG 26586	Eilatimonas	milleporae
Z0020_LO_C03_1_F02_A	106	93	LMG 26586	Eilatimonas	milleporae
Z0020_LO_C04_1_F02_B	106	93	LMG 26586	Eilatimonas	milleporae
Z0020_LO_C05_1_F03_A	106	93	LMG 26586	Eilatimonas	milleporae
Z0020_RO_B02_2_E07_B	106	93	LMG 26586	Eilatimonas	milleporae
Z0020_RO_B03_2_E08_A	106	93	LMG 26586	Eilatimonas	milleporae
Z0020_RO_B04_2_E08_B	106	93	LMG 26586	Eilatimonas	milleporae
Z0020_RO_B06_2_E09_B	106	93	LMG 26586	Eilatimonas	milleporae
Z0020_RO_B08_2_E10_B	106	93	LMG 26586	Eilatimonas	milleporae
Z0020_RO_B09_2_E11_A	106	93	LMG 26586	Eilatimonas	milleporae
Z0020_RO_B10_2_E11_B	106	93	LMG 26586	Eilatimonas	milleporae
Z0020_RO_B11_2_E12_A	106	93	LMG 26586	Eilatimonas	milleporae
Z0020_RO_B12_2_E12_B	106	93	LMG 26586	Eilatimonas	milleporae
Z0020_RO_C02_1_F07_B	106	93	LMG 26586	Eilatimonas	milleporae
Z0020_RO_C03_1_F08_A	106	93	LMG 26586	Eilatimonas	milleporae
Z0020_RO_C05_1_F09_A	106	93	LMG 26586	Eilatimonas	milleporae
Z0020_RO_C08_1_F10_B	106	93	LMG 26586	Eilatimonas	milleporae
Z0020_LO_D06_2_F03_B	107	95	LMG 2698	Brenneria	salicis
Z0020_LO_D07_2_F04_A	107	95	LMG 2698	Brenneria	salicis
Z0020_LO_D09_2_F05_A	107	95	LMG 2698	Brenneria	salicis
Z0020_LO_D12_2_F06_B	107	95	LMG 2698	Brenneria	salicis
Z0020_LO_E02_1_G01_B	107	95	LMG 2698	Brenneria	salicis
Z0020_LO_E03_1_G02_A	107	95	LMG 2698	Brenneria	salicis
Z0020_LO_E05_1_G03_A	107	95	LMG 2698	Brenneria	salicis

Z0020_LO_E06_1_G03_B	107	95	LMG 2698	Brenneria	salicis
Z0020_LO_E08_1_G04_B	107	95	LMG 2698	Brenneria	salicis
Z0020_LO_E11_1_G06_A	107	95	LMG 2698	Brenneria	salicis
Z0020_LO_F03_2_G02_A	107	95	LMG 2698	Brenneria	salicis
Z0020_LO_F04_2_G02_B	107	95	LMG 2698	Brenneria	salicis
Z0020_LO_F05_2_G03_A	107	95	LMG 2698	Brenneria	salicis
Z0020_LO_F08_2_G04_B	107	95	LMG 2698	Brenneria	salicis
Z0020_LO_F09_2_G05_A	107	95	LMG 2698	Brenneria	salicis
Z0020_LO_F11_2_G06_A	107	95	LMG 2698	Brenneria	salicis
Z0020_LO_G01_1_H01_A	107	95	LMG 2698	Brenneria	salicis
Z0020_RO_D08_2_F10_B	107	95	LMG 2698	Brenneria	salicis
Z0020_RO_D09_2_F11_A	107	95	LMG 2698	Brenneria	salicis
Z0020_RO_D11_2_F12_A	107	95	LMG 2698	Brenneria	salicis
Z0020_RO_D12_2_F12_B	107	95	LMG 2698	Brenneria	salicis
Z0020_RO_E03_1_G08_A	107	95	LMG 2698	Brenneria	salicis
Z0020_RO_E05_1_G09_A	107	95	LMG 2698	Brenneria	salicis
Z0020_RO_E06_1_G09_B	107	95	LMG 2698	Brenneria	salicis
Z0020_RO_E08_1_G10_B	107	95	LMG 2698	Brenneria	salicis
Z0020_RO_E11_1_G12_A	107	95	LMG 2698	Brenneria	salicis
Z0020_RO_F02_2_G07_B	107	95	LMG 2698	Brenneria	salicis
Z0020_RO_F04_2_G08_B	107	95	LMG 2698	Brenneria	salicis
Z0020_RO_F05_2_G09_A	107	95	LMG 2698	Brenneria	salicis
Z0020_RO_F07_2_G10_A	107	95	LMG 2698	Brenneria	salicis
Z0020_RO_F09_2_G11_A	107	95	LMG 2698	Brenneria	salicis
Z0020_LO_F01_2_G01_A	107	95	LMG 2698	Brenneria	salicis
Z0020_LO_F02_2_G01_B	108	94	LMG 26852	Achromobacter	aegrifaciens
Z0020_LO_F06_2_G03_B	108	94	LMG 26852	Achromobacter	aegrifaciens
Z0020_LO_F07_2_G04_A	108	94	LMG 26852	Achromobacter	aegrifaciens
Z0020_LO_F10_2_G05_B	108	94	LMG 26852	Achromobacter	aegrifaciens
Z0020_LO_F12_2_G06_B	108	94	LMG 26852	Achromobacter	aegrifaciens
Z0020_LO_G02_1_H01_B	108	94	LMG 26852	Achromobacter	aegrifaciens
Z0020_LO_G03_1_H02_A	108	94	LMG 26852	Achromobacter	aegrifaciens
Z0020_LO_G04_1_H02_B	108	94	LMG 26852	Achromobacter	aegrifaciens
Z0020_LO_G05_1_H03_A	108	94	LMG 26852	Achromobacter	aegrifaciens
Z0020_LO_G06_1_H03_B	108	94	LMG 26852	Achromobacter	aegrifaciens
Z0020_LO_G07_1_H04_A	108	94	LMG 26852	Achromobacter	aegrifaciens
Z0020_LO_G08_1_H04_B	108	94	LMG 26852	Achromobacter	aegrifaciens
Z0020_LO_G09_1_H05_A	108	94	LMG 26852	Achromobacter	aegrifaciens
Z0020_RO_F10_2_G11_B	108	94	LMG 26852	Achromobacter	aegrifaciens
Z0020_RO_F11_2_G12_A	108	94	LMG 26852	Achromobacter	aegrifaciens
Z0020_RO_F12_2_G12_B	108	94	LMG 26852	Achromobacter	aegrifaciens
Z0020_RO_G01_1_H07_A	108	94	LMG 26852	Achromobacter	aegrifaciens
Z0020_RO_G02_1_H07_B	108	94	LMG 26852	Achromobacter	aegrifaciens
Z0020_RO_G03_1_H08_A	108	94	LMG 26852	Achromobacter	aegrifaciens
Z0020_RO_G04_1_H08_B	108	94	LMG 26852	Achromobacter	aegrifaciens
Z0020_RO_G05_1_H09_A	108	94	LMG 26852	Achromobacter	aegrifaciens
Z0020_RO_G06_1_H09_B	108	94	LMG 26852	Achromobacter	aegrifaciens
Z0020_RO_G07_1_H10_A	108	94	LMG 26852	Achromobacter	aegrifaciens
Z0020_RO_G08_1_H10_B	108	94	LMG 26852	Achromobacter	aegrifaciens
Z0020_RO_G09_1_H11_A	108	94	LMG 26852	Achromobacter	aegrifaciens

Z0020_RO_G10_1_H11_B	108	94	LMG 26852	Achromobacter	aegrifaciens
Z0020_RO_G11_1_H12_A	108	94	LMG 26852	Achromobacter	aegrifaciens
Z0020_RO_G12_1_H12_B	108	94	LMG 26852	Achromobacter	aegrifaciens
Z0020_RO_H01_2_H07_A	108	94	LMG 26852	Achromobacter	aegrifaciens
Z0020_RO_H02_2_H07_B	108	94	LMG 26852	Achromobacter	aegrifaciens
Z0020_RO_H03_2_H08_A	108	94	LMG 26852	Achromobacter	aegrifaciens
Z0020_RO_H04_2_H08_B	108	94	LMG 26852	Achromobacter	aegrifaciens
Z0021_LB_A01_2_D06_A	109	97	LMG 27021	Neokomagataea	thailandica
Z0021_LB_A02_2_D06_B	109	97	LMG 27021	Neokomagataea	thailandica
Z0021_LB_A03_2_D05_A	109	97	LMG 27021	Neokomagataea	thailandica
Z0021_LB_A04_2_D05_B	109	97	LMG 27021	Neokomagataea	thailandica
Z0021_LB_A05_2_D04_A	109	97	LMG 27021	Neokomagataea	thailandica
Z0021_LB_A06_2_D04_B	109	97	LMG 27021	Neokomagataea	thailandica
Z0021_LB_A07_2_D03_A	109	97	LMG 27021	Neokomagataea	thailandica
Z0021_LB_A08_2_D03_B	109	97	LMG 27021	Neokomagataea	thailandica
Z0021_LB_A09_2_D02_A	109	97	LMG 27021	Neokomagataea	thailandica
Z0021_LB_A10_2_D02_B	109	97	LMG 27021	Neokomagataea	thailandica
Z0021_LB_A11_2_D01_A	109	97	LMG 27021	Neokomagataea	thailandica
Z0021_LO_A09_1_E05_A	109	97	LMG 27021	Neokomagataea	thailandica
Z0021_LO_A10_1_E05_B	109	97	LMG 27021	Neokomagataea	thailandica
Z0021_LO_B03_2_E02_A	109	97	LMG 27021	Neokomagataea	thailandica
Z0021_LO_B05_2_E03_A	109	97	LMG 27021	Neokomagataea	thailandica
Z0021_LO_B09_2_E05_A	109	97	LMG 27021	Neokomagataea	thailandica
Z0021_LO_C04_1_F02_B	109	97	LMG 27021	Neokomagataea	thailandica
Z0021_LO_H07_2_H04_A	109	97	LMG 27021	Neokomagataea	thailandica
Z0021_LO_H08_2_H04_B	109	97	LMG 27021	Neokomagataea	thailandica
Z0021_LO_H09_2_H05_A	109	97	LMG 27021	Neokomagataea	thailandica
Z0021_LO_H10_2_H05_B	109	97	LMG 27021	Neokomagataea	thailandica
Z0021_LO_H11_2_H06_A	109	97	LMG 27021	Neokomagataea	thailandica
Z0021_LO_H12_2_H06_B	109	97	LMG 27021	Neokomagataea	thailandica
Z0021_RB_A01_2_D12_A	109	97	LMG 27021	Neokomagataea	thailandica
Z0021_RB_A02_2_D12_B	109	97	LMG 27021	Neokomagataea	thailandica
Z0021_RB_A03_2_D11_A	109	97	LMG 27021	Neokomagataea	thailandica
Z0021_RB_A04_2_D11_B	109	97	LMG 27021	Neokomagataea	thailandica
Z0021_RB_A05_2_D10_A	109	97	LMG 27021	Neokomagataea	thailandica
Z0021_RO_B05_2_E09_A	109	97	LMG 27021	Neokomagataea	thailandica
Z0021_RO_B11_2_E12_A	109	97	LMG 27021	Neokomagataea	thailandica
Z0021_RO_C01_1_F07_A	109	97	LMG 27021	Neokomagataea	thailandica
Z0021_RO_C05_1_F09_A	109	97	LMG 27021	Neokomagataea	thailandica
Z0021_LB_A12_2_D01_B	110	98	LMG 27212	Pectinatus	sp.
Z0021_LB_B01_1_D06_A	110	98	LMG 27212	Pectinatus	sp.
Z0021_LB_B02_1_D06_B	110	98	LMG 27212	Pectinatus	sp.
Z0021_LB_B03_1_D05_A	110	98	LMG 27212	Pectinatus	sp.
Z0021_LB_B04_1_D05_B	110	98	LMG 27212	Pectinatus	sp.
Z0021_LB_B05_1_D04_A	110	98	LMG 27212	Pectinatus	sp.
Z0021_LB_B06_1_D04_B	110	98	LMG 27212	Pectinatus	sp.
Z0021_LB_B07_1_D03_A	110	98	LMG 27212	Pectinatus	sp.
Z0021_LB_B08_1_D03_B	110	98	LMG 27212	Pectinatus	sp.
Z0021_LB_B09_1_D02_A	110	98	LMG 27212	Pectinatus	sp.
Z0021_LB_B10_1_D02_B	110	98	LMG 27212	Pectinatus	sp.

Z0021_LB_B11_1_D01_A	110	98	LMG 27212	Pectinatus	sp.	
Z0021_LB_B12_1_D01_B	110	98	LMG 27212	Pectinatus	sp.	
Z0021_LB_C01_2_C06_A	110	98	LMG 27212	Pectinatus	sp.	
Z0021_LB_C02_2_C06_B	110	98	LMG 27212	Pectinatus	sp.	
Z0021_LB_C03_2_C05_A	110	98	LMG 27212	Pectinatus	sp.	
Z0021_LB_C04_2_C05_B	110	98	LMG 27212	Pectinatus	sp.	
Z0021_LB_C05_2_C04_A	110	98	LMG 27212	Pectinatus	sp.	
Z0021_RB_A06_2_D10_B	110	98	LMG 27212	Pectinatus	sp.	
Z0021_RB_A07_2_D09_A	110	98	LMG 27212	Pectinatus	sp.	
Z0021_RB_A08_2_D09_B	110	98	LMG 27212	Pectinatus	sp.	
Z0021_RB_A09_2_D08_A	110	98	LMG 27212	Pectinatus	sp.	
Z0021_RB_A10_2_D08_B	110	98	LMG 27212	Pectinatus	sp.	
Z0021_RB_A11_2_D07_A	110	98	LMG 27212	Pectinatus	sp.	
Z0021_RB_A12_2_D07_B	110	98	LMG 27212	Pectinatus	sp.	
Z0021_RB_B01_1_D12_A	110	98	LMG 27212	Pectinatus	sp.	
Z0021_RB_B02_1_D12_B	110	98	LMG 27212	Pectinatus	sp.	
Z0021_RB_B03_1_D11_A	110	98	LMG 27212	Pectinatus	sp.	
Z0021_RB_B04_1_D11_B	110	98	LMG 27212	Pectinatus	sp.	
Z0021_RB_B05_1_D10_A	110	98	LMG 27212	Pectinatus	sp.	
Z0021_RB_B06_1_D10_B	110	98	LMG 27212	Pectinatus	sp.	
Z0021_RB_B07_1_D09_A	110	98	LMG 27212	Pectinatus	sp.	
Z0021_LB_C06_2_C04_B	111	99	LMG 2724	Lonsdalea	quercina	quercina
Z0021_LB_C07_2_C03_A	111	99	LMG 2724	Lonsdalea	quercina	quercina
Z0021_LB_C08_2_C03_B	111	99	LMG 2724	Lonsdalea	quercina	quercina
Z0021_LB_C09_2_C02_A	111	99	LMG 2724	Lonsdalea	quercina	quercina
Z0021_LB_C10_2_C02_B	111	99	LMG 2724	Lonsdalea	quercina	quercina
Z0021_LB_C11_2_C01_A	111	99	LMG 2724	Lonsdalea	quercina	quercina
Z0021_LB_C12_2_C01_B	111	99	LMG 2724	Lonsdalea	quercina	quercina
Z0021_LB_D01_1_C06_A	111	99	LMG 2724	Lonsdalea	quercina	quercina
Z0021_LB_D02_1_C06_B	111	99	LMG 2724	Lonsdalea	quercina	quercina
Z0021_LB_D03_1_C05_A	111	99	LMG 2724	Lonsdalea	quercina	quercina
Z0021_LB_D04_1_C05_B	111	99	LMG 2724	Lonsdalea	quercina	quercina
Z0021_LB_D05_1_C04_A	111	99	LMG 2724	Lonsdalea	quercina	quercina
Z0021_LB_D06_1_C04_B	111	99	LMG 2724	Lonsdalea	quercina	quercina
Z0021_RB_B08_1_D09_B	111	99	LMG 2724	Lonsdalea	quercina	quercina
Z0021_RB_B09_1_D08_A	111	99	LMG 2724	Lonsdalea	quercina	quercina
Z0021_RB_B10_1_D08_B	111	99	LMG 2724	Lonsdalea	quercina	quercina
Z0021_RB_B11_1_D07_A	111	99	LMG 2724	Lonsdalea	quercina	quercina
Z0021_RB_B12_1_D07_B	111	99	LMG 2724	Lonsdalea	quercina	quercina
Z0021_RB_C01_2_C12_A	111	99	LMG 2724	Lonsdalea	quercina	quercina
Z0021_RB_C02_2_C12_B	111	99	LMG 2724	Lonsdalea	quercina	quercina
Z0021_RB_C03_2_C11_A	111	99	LMG 2724	Lonsdalea	quercina	quercina
Z0021_RB_C04_2_C11_B	111	99	LMG 2724	Lonsdalea	quercina	quercina
Z0021_RB_C05_2_C10_A	111	99	LMG 2724	Lonsdalea	quercina	quercina
Z0021_RB_C06_2_C10_B	111	99	LMG 2724	Lonsdalea	quercina	quercina
Z0021_RB_C07_2_C09_A	111	99	LMG 2724	Lonsdalea	quercina	quercina
Z0021_RB_C08_2_C09_B	111	99	LMG 2724	Lonsdalea	quercina	quercina
Z0021_RB_C09_2_C08_A	111	99	LMG 2724	Lonsdalea	quercina	quercina
Z0021_RB_C10_2_C08_B	111	99	LMG 2724	Lonsdalea	quercina	quercina
Z0021_RB_C11_2_C07_A	111	99	LMG 2724	Lonsdalea	quercina	quercina

Z0021_RB_C12_2_C07_B	111	99	LMG 2724	Lonsdalea	quercina	quercina
Z0021_RB_D01_1_C12_A	111	99	LMG 2724	Lonsdalea	quercina	quercina
Z0021_RB_D02_1_C12_B	111	99	LMG 2724	Lonsdalea	quercina	quercina
Z0021_LB_D07_1_C03_A	112	100	LMG 27282	Noviherbaspirillum	psychrotolerans	
Z0021_LB_D08_1_C03_B	112	100	LMG 27282	Noviherbaspirillum	psychrotolerans	
Z0021_LB_D09_1_C02_A	112	100	LMG 27282	Noviherbaspirillum	psychrotolerans	
Z0021_LB_D10_1_C02_B	112	100	LMG 27282	Noviherbaspirillum	psychrotolerans	
Z0021_LB_D11_1_C01_A	112	100	LMG 27282	Noviherbaspirillum	psychrotolerans	
Z0021_LB_D12_1_C01_B	112	100	LMG 27282	Noviherbaspirillum	psychrotolerans	
Z0021_LB_E01_2_B06_A	112	100	LMG 27282	Noviherbaspirillum	psychrotolerans	
Z0021_LB_E02_2_B06_B	112	100	LMG 27282	Noviherbaspirillum	psychrotolerans	
Z0021_LB_E03_2_B05_A	112	100	LMG 27282	Noviherbaspirillum	psychrotolerans	
Z0021_LB_E04_2_B05_B	112	100	LMG 27282	Noviherbaspirillum	psychrotolerans	
Z0021_LB_E05_2_B04_A	112	100	LMG 27282	Noviherbaspirillum	psychrotolerans	
Z0021_LB_E06_2_B04_B	112	100	LMG 27282	Noviherbaspirillum	psychrotolerans	
Z0021_LB_E07_2_B03_A	112	100	LMG 27282	Noviherbaspirillum	psychrotolerans	
Z0021_LB_E08_2_B03_B	112	100	LMG 27282	Noviherbaspirillum	psychrotolerans	
Z0021_LB_E09_2_B02_A	112	100	LMG 27282	Noviherbaspirillum	psychrotolerans	
Z0021_LB_E10_2_B02_B	112	100	LMG 27282	Noviherbaspirillum	psychrotolerans	
Z0021_RB_D03_1_C11_A	112	100	LMG 27282	Noviherbaspirillum	psychrotolerans	
Z0021_RB_D04_1_C11_B	112	100	LMG 27282	Noviherbaspirillum	psychrotolerans	
Z0021_RB_D05_1_C10_A	112	100	LMG 27282	Noviherbaspirillum	psychrotolerans	
Z0021_RB_D06_1_C10_B	112	100	LMG 27282	Noviherbaspirillum	psychrotolerans	
Z0021_RB_D07_1_C09_A	112	100	LMG 27282	Noviherbaspirillum	psychrotolerans	
Z0021_RB_D08_1_C09_B	112	100	LMG 27282	Noviherbaspirillum	psychrotolerans	
Z0021_RB_D09_1_C08_A	112	100	LMG 27282	Noviherbaspirillum	psychrotolerans	
Z0021_RB_D10_1_C08_B	112	100	LMG 27282	Noviherbaspirillum	psychrotolerans	
Z0021_RB_D11_1_C07_A	112	100	LMG 27282	Noviherbaspirillum	psychrotolerans	
Z0021_RB_D12_1_C07_B	112	100	LMG 27282	Noviherbaspirillum	psychrotolerans	
Z0021_RB_E01_2_B12_A	112	100	LMG 27282	Noviherbaspirillum	psychrotolerans	
Z0021_RB_E02_2_B12_B	112	100	LMG 27282	Noviherbaspirillum	psychrotolerans	
Z0021_RB_E03_2_B11_A	112	100	LMG 27282	Noviherbaspirillum	psychrotolerans	
Z0021_RB_E04_2_B11_B	112	100	LMG 27282	Noviherbaspirillum	psychrotolerans	
Z0021_RB_E05_2_B10_A	112	100	LMG 27282	Noviherbaspirillum	psychrotolerans	
Z0021_RB_E06_2_B10_B	112	100	LMG 27282	Noviherbaspirillum	psychrotolerans	
Z0021_LB_E11_2_B01_A	113	101	LMG 27719	Roseomonas	gilardii	
Z0021_LB_E12_2_B01_B	113	101	LMG 27719	Roseomonas	gilardii	
Z0021_LB_F01_1_B06_A	113	101	LMG 27719	Roseomonas	gilardii	
Z0021_LB_F02_1_B06_B	113	101	LMG 27719	Roseomonas	gilardii	
Z0021_LB_F03_1_B05_A	113	101	LMG 27719	Roseomonas	gilardii	
Z0021_LB_F04_1_B05_B	113	101	LMG 27719	Roseomonas	gilardii	
Z0021_LB_F05_1_B04_A	113	101	LMG 27719	Roseomonas	gilardii	
Z0021_LB_F06_1_B04_B	113	101	LMG 27719	Roseomonas	gilardii	
Z0021_LB_F07_1_B03_A	113	101	LMG 27719	Roseomonas	gilardii	
Z0021_LB_F08_1_B03_B	113	101	LMG 27719	Roseomonas	gilardii	
Z0021_LB_F09_1_B02_A	113	101	LMG 27719	Roseomonas	gilardii	
Z0021_LB_F10_1_B02_B	113	101	LMG 27719	Roseomonas	gilardii	
Z0021_LB_F11_1_B01_A	113	101	LMG 27719	Roseomonas	gilardii	
Z0021_RB_E07_2_B09_A	113	101	LMG 27719	Roseomonas	gilardii	
Z0021_RB_E08_2_B09_B	113	101	LMG 27719	Roseomonas	gilardii	

Z0021_RB_E09_2_B08_A	113	101	LMG 27719	Roseomonas	gilardii
Z0021_RB_E10_2_B08_B	113	101	LMG 27719	Roseomonas	gilardii
Z0021_RB_E11_2_B07_A	113	101	LMG 27719	Roseomonas	gilardii
Z0021_RB_E12_2_B07_B	113	101	LMG 27719	Roseomonas	gilardii
Z0021_RB_F01_1_B12_A	113	101	LMG 27719	Roseomonas	gilardii
Z0021_RB_F02_1_B12_B	113	101	LMG 27719	Roseomonas	gilardii
Z0021_RB_F03_1_B11_A	113	101	LMG 27719	Roseomonas	gilardii
Z0021_RB_F04_1_B11_B	113	101	LMG 27719	Roseomonas	gilardii
Z0021_RB_F05_1_B10_A	113	101	LMG 27719	Roseomonas	gilardii
Z0021_RB_F06_1_B10_B	113	101	LMG 27719	Roseomonas	gilardii
Z0021_RB_F07_1_B09_A	113	101	LMG 27719	Roseomonas	gilardii
Z0021_RB_F08_1_B09_B	113	101	LMG 27719	Roseomonas	gilardii
Z0021_RB_F09_1_B08_A	113	101	LMG 27719	Roseomonas	gilardii
Z0021_RB_F10_1_B08_B	113	101	LMG 27719	Roseomonas	gilardii
Z0021_RB_F11_1_B07_A	113	101	LMG 27719	Roseomonas	gilardii
Z0021_RB_F12_1_B07_B	113	101	LMG 27719	Roseomonas	gilardii
Z0021_RB_G01_2_A12_A	113	101	LMG 27719	Roseomonas	gilardii
Z0021_LB_F12_1_B01_B	114	102	LMG 2804	Dickeya	chrysanthemi
Z0021_LB_G01_2_A06_A	114	102	LMG 2804	Dickeya	chrysanthemi
Z0021_LB_G02_2_A06_B	114	102	LMG 2804	Dickeya	chrysanthemi
Z0021_LB_G03_2_A05_A	114	102	LMG 2804	Dickeya	chrysanthemi
Z0021_LB_G04_2_A05_B	114	102	LMG 2804	Dickeya	chrysanthemi
Z0021_LB_G05_2_A04_A	114	102	LMG 2804	Dickeya	chrysanthemi
Z0021_LB_G06_2_A04_B	114	102	LMG 2804	Dickeya	chrysanthemi
Z0021_LB_G07_2_A03_A	114	102	LMG 2804	Dickeya	chrysanthemi
Z0021_LB_G08_2_A03_B	114	102	LMG 2804	Dickeya	chrysanthemi
Z0021_LB_G09_2_A02_A	114	102	LMG 2804	Dickeya	chrysanthemi
Z0021_LB_G10_2_A02_B	114	102	LMG 2804	Dickeya	chrysanthemi
Z0021_LB_G11_2_A01_A	114	102	LMG 2804	Dickeya	chrysanthemi
Z0021_LB_G12_2_A01_B	114	102	LMG 2804	Dickeya	chrysanthemi
Z0021_LB_H01_1_A06_A	114	102	LMG 2804	Dickeya	chrysanthemi
Z0021_LB_H02_1_A06_B	114	102	LMG 2804	Dickeya	chrysanthemi
Z0021_LB_H03_1_A05_A	114	102	LMG 2804	Dickeya	chrysanthemi
Z0021_LB_H04_1_A05_B	114	102	LMG 2804	Dickeya	chrysanthemi
Z0021_RB_G02_2_A12_B	114	102	LMG 2804	Dickeya	chrysanthemi
Z0021_RB_G03_2_A11_A	114	102	LMG 2804	Dickeya	chrysanthemi
Z0021_RB_G04_2_A11_B	114	102	LMG 2804	Dickeya	chrysanthemi
Z0021_RB_G05_2_A10_A	114	102	LMG 2804	Dickeya	chrysanthemi
Z0021_RB_G06_2_A10_B	114	102	LMG 2804	Dickeya	chrysanthemi
Z0021_RB_G07_2_A09_A	114	102	LMG 2804	Dickeya	chrysanthemi
Z0021_RB_G08_2_A09_B	114	102	LMG 2804	Dickeya	chrysanthemi
Z0021_RB_G09_2_A08_A	114	102	LMG 2804	Dickeya	chrysanthemi
Z0021_RB_G10_2_A08_B	114	102	LMG 2804	Dickeya	chrysanthemi
Z0021_RB_G11_2_A07_A	114	102	LMG 2804	Dickeya	chrysanthemi
Z0021_RB_G12_2_A07_B	114	102	LMG 2804	Dickeya	chrysanthemi
Z0021_RB_H01_1_A12_A	114	102	LMG 2804	Dickeya	chrysanthemi
Z0021_RB_H02_1_A12_B	114	102	LMG 2804	Dickeya	chrysanthemi
Z0021_RB_H03_1_A11_A	114	102	LMG 2804	Dickeya	chrysanthemi
Z0021_RB_H04_1_A11_B	114	102	LMG 2804	Dickeya	chrysanthemi
Z0021_LB_H05_1_A04_A	115	103	LMG 2811	hermoanaerobacteriun	hermosaccharolyticum

Z0021_LB_H06_1_A04_B	115	103	LMG 2811	hermoanaerobacteriun	hermosaccharolyticum
Z0021_LB_H07_1_A03_A	115	103	LMG 2811	hermoanaerobacteriun	hermosaccharolyticum
Z0021_LB_H08_1_A03_B	115	103	LMG 2811	hermoanaerobacteriun	hermosaccharolyticum
Z0021_LB_H09_1_A02_A	115	103	LMG 2811	hermoanaerobacteriun	hermosaccharolyticum
Z0021_LB_H10_1_A02_B	115	103	LMG 2811	hermoanaerobacteriun	hermosaccharolyticum
Z0021_LB_H11_1_A01_A	115	103	LMG 2811	hermoanaerobacteriun	hermosaccharolyticum
Z0021_LB_H12_1_A01_B	115	103	LMG 2811	hermoanaerobacteriun	hermosaccharolyticum
Z0021_LO_A01_1_E01_A	115	103	LMG 2811	hermoanaerobacteriun	hermosaccharolyticum
Z0021_LO_A02_1_E01_B	115	103	LMG 2811	hermoanaerobacteriun	hermosaccharolyticum
Z0021_LO_A03_1_E02_A	115	103	LMG 2811	hermoanaerobacteriun	hermosaccharolyticum
Z0021_LO_A04_1_E02_B	115	103	LMG 2811	hermoanaerobacteriun	hermosaccharolyticum
Z0021_LO_A05_1_E03_A	115	103	LMG 2811	hermoanaerobacteriun	hermosaccharolyticum
Z0021_LO_A06_1_E03_B	115	103	LMG 2811	hermoanaerobacteriun	hermosaccharolyticum
Z0021_LO_A07_1_E04_A	115	103	LMG 2811	hermoanaerobacteriun	hermosaccharolyticum
Z0021_LO_A08_1_E04_B	115	103	LMG 2811	hermoanaerobacteriun	hermosaccharolyticum
Z0021_RB_H05_1_A10_A	115	103	LMG 2811	hermoanaerobacteriun	hermosaccharolyticum
Z0021_RB_H08_1_A09_B	115	103	LMG 2811	hermoanaerobacteriun	hermosaccharolyticum
Z0021_RB_H10_1_A08_B	115	103	LMG 2811	hermoanaerobacteriun	hermosaccharolyticum
Z0021_RB_H11_1_A07_A	115	103	LMG 2811	hermoanaerobacteriun	hermosaccharolyticum
Z0021_RB_H12_1_A07_B	115	103	LMG 2811	hermoanaerobacteriun	hermosaccharolyticum
Z0021_RO_A01_1_E07_A	115	103	LMG 2811	hermoanaerobacteriun	hermosaccharolyticum
Z0021_RO_A03_1_E08_A	115	103	LMG 2811	hermoanaerobacteriun	hermosaccharolyticum
Z0021_RO_A04_1_E08_B	115	103	LMG 2811	hermoanaerobacteriun	hermosaccharolyticum
Z0021_RO_A05_1_E09_A	115	103	LMG 2811	hermoanaerobacteriun	hermosaccharolyticum
Z0021_RO_A06_1_E09_B	115	103	LMG 2811	hermoanaerobacteriun	hermosaccharolyticum
Z0021_RO_A07_1_E10_A	115	103	LMG 2811	hermoanaerobacteriun	hermosaccharolyticum
Z0021_RO_A08_1_E10_B	115	103	LMG 2811	hermoanaerobacteriun	hermosaccharolyticum
Z0021_LO_A11_1_E06_A	116	104	LMG 28216	Formosa	algae
Z0021_LO_A12_1_E06_B	116	104	LMG 28216	Formosa	algae
Z0021_LO_B01_2_E01_A	116	104	LMG 28216	Formosa	algae
Z0021_LO_B02_2_E01_B	116	104	LMG 28216	Formosa	algae
Z0021_LO_B04_2_E02_B	116	104	LMG 28216	Formosa	algae
Z0021_LO_B06_2_E03_B	116	104	LMG 28216	Formosa	algae
Z0021_LO_B08_2_E04_B	116	104	LMG 28216	Formosa	algae
Z0021_LO_B10_2_E05_B	116	104	LMG 28216	Formosa	algae
Z0021_LO_B12_2_E06_B	116	104	LMG 28216	Formosa	algae
Z0021_LO_C01_1_F01_A	116	104	LMG 28216	Formosa	algae
Z0021_LO_C03_1_F02_A	116	104	LMG 28216	Formosa	algae
Z0021_LO_C05_1_F03_A	116	104	LMG 28216	Formosa	algae
Z0021_LO_C08_1_F04_B	116	104	LMG 28216	Formosa	algae
Z0021_RO_A12_1_E12_B	116	104	LMG 28216	Formosa	algae
Z0021_RO_B01_2_E07_A	116	104	LMG 28216	Formosa	algae
Z0021_RO_B02_2_E07_B	116	104	LMG 28216	Formosa	algae
Z0021_RO_B03_2_E08_A	116	104	LMG 28216	Formosa	algae
Z0021_RO_B04_2_E08_B	116	104	LMG 28216	Formosa	algae
Z0021_RO_B06_2_E09_B	116	104	LMG 28216	Formosa	algae
Z0021_RO_B07_2_E10_A	116	104	LMG 28216	Formosa	algae
Z0021_RO_B09_2_E11_A	116	104	LMG 28216	Formosa	algae
Z0021_RO_B10_2_E11_B	116	104	LMG 28216	Formosa	algae
Z0021_RO_B12_2_E12_B	116	104	LMG 28216	Formosa	algae

Z0021_RO_C02_1_F07_B	116	104	LMG 28216	Formosa	algae
Z0021_RO_C03_1_F08_A	116	104	LMG 28216	Formosa	algae
Z0021_RO_C04_1_F08_B	116	104	LMG 28216	Formosa	algae
Z0021_RO_C06_1_F09_B	116	104	LMG 28216	Formosa	algae
Z0021_RO_C07_1_F10_A	116	104	LMG 28216	Formosa	algae
Z0021_LO_B07_2_E04_A	117	106	LMG 28633	Rhodococcus	degradans
Z0021_LO_B11_2_E06_A	117	106	LMG 28633	Rhodococcus	degradans
Z0021_LO_C02_1_F01_B	117	106	LMG 28633	Rhodococcus	degradans
Z0021_LO_C06_1_F03_B	117	106	LMG 28633	Rhodococcus	degradans
Z0021_LO_C07_1_F04_A	117	106	LMG 28633	Rhodococcus	degradans
Z0021_LO_C09_1_F05_A	117	106	LMG 28633	Rhodococcus	degradans
Z0021_LO_C10_1_F05_B	117	106	LMG 28633	Rhodococcus	degradans
Z0021_LO_C11_1_F06_A	117	106	LMG 28633	Rhodococcus	degradans
Z0021_LO_D01_2_F01_A	117	106	LMG 28633	Rhodococcus	degradans
Z0021_LO_D05_2_F03_A	117	106	LMG 28633	Rhodococcus	degradans
Z0021_LO_D06_2_F03_B	117	106	LMG 28633	Rhodococcus	degradans
Z0021_LO_D08_2_F04_B	117	106	LMG 28633	Rhodococcus	degradans
Z0021_LO_D09_2_F05_A	117	106	LMG 28633	Rhodococcus	degradans
Z0021_LO_D10_2_F05_B	117	106	LMG 28633	Rhodococcus	degradans
Z0021_LO_D12_2_F06_B	117	106	LMG 28633	Rhodococcus	degradans
Z0021_LO_E03_1_G02_A	117	106	LMG 28633	Rhodococcus	degradans
Z0021_LO_E07_1_G04_A	117	106	LMG 28633	Rhodococcus	degradans
Z0021_LO_E08_1_G04_B	117	106	LMG 28633	Rhodococcus	degradans
Z0021_LO_F06_2_G03_B	117	106	LMG 28633	Rhodococcus	degradans
Z0021_RB_H06_1_A10_B	117	106	LMG 28633	Rhodococcus	degradans
Z0021_RB_H07_1_A09_A	117	106	LMG 28633	Rhodococcus	degradans
Z0021_RB_H09_1_A08_A	117	106	LMG 28633	Rhodococcus	degradans
Z0021_RO_A02_1_E07_B	117	106	LMG 28633	Rhodococcus	degradans
Z0021_RO_A09_1_E11_A	117	106	LMG 28633	Rhodococcus	degradans
Z0021_RO_A10_1_E11_B	117	106	LMG 28633	Rhodococcus	degradans
Z0021_RO_A11_1_E12_A	117	106	LMG 28633	Rhodococcus	degradans
Z0021_RO_B08_2_E10_B	117	106	LMG 28633	Rhodococcus	degradans
Z0021_RO_C09_1_F11_A	117	106	LMG 28633	Rhodococcus	degradans
Z0021_RO_C11_1_F12_A	117	106	LMG 28633	Rhodococcus	degradans
Z0021_RO_C12_1_F12_B	117	106	LMG 28633	Rhodococcus	degradans
Z0021_RO_D01_2_F07_A	117	106	LMG 28633	Rhodococcus	degradans
Z0021_RO_D04_2_F08_B	117	106	LMG 28633	Rhodococcus	degradans
Z0021_LO_F09_2_G05_A	118	105	LMG 28391	Salininema	proteolyticum
Z0021_LO_F10_2_G05_B	118	105	LMG 28391	Salininema	proteolyticum
Z0021_LO_F11_2_G06_A	118	105	LMG 28391	Salininema	proteolyticum
Z0021_LO_G06_1_H03_B	118	105	LMG 28391	Salininema	proteolyticum
Z0021_LO_G08_1_H04_B	118	105	LMG 28391	Salininema	proteolyticum
Z0021_LO_G09_1_H05_A	118	105	LMG 28391	Salininema	proteolyticum
Z0021_LO_G10_1_H05_B	118	105	LMG 28391	Salininema	proteolyticum
Z0021_LO_G11_1_H06_A	118	105	LMG 28391	Salininema	proteolyticum
Z0021_LO_G12_1_H06_B	118	105	LMG 28391	Salininema	proteolyticum
Z0021_LO_H01_2_H01_A	118	105	LMG 28391	Salininema	proteolyticum
Z0021_LO_H03_2_H02_A	118	105	LMG 28391	Salininema	proteolyticum
Z0021_LO_H04_2_H02_B	118	105	LMG 28391	Salininema	proteolyticum
Z0021_LO_H05_2_H03_A	118	105	LMG 28391	Salininema	proteolyticum

Z0021_LO_H06_2_H03_B	118	105	LMG 28391	Salininema	proteolyticum
Z0021_RO_F06_2_G09_B	118	105	LMG 28391	Salininema	proteolyticum
Z0021_RO_F10_2_G11_B	118	105	LMG 28391	Salininema	proteolyticum
Z0021_RO_F11_2_G12_A	118	105	LMG 28391	Salininema	proteolyticum
Z0021_RO_F12_2_G12_B	118	105	LMG 28391	Salininema	proteolyticum
Z0021_RO_G01_1_H07_A	118	105	LMG 28391	Salininema	proteolyticum
Z0021_RO_G02_1_H07_B	118	105	LMG 28391	Salininema	proteolyticum
Z0021_RO_G03_1_H08_A	118	105	LMG 28391	Salininema	proteolyticum
Z0021_RO_G05_1_H09_A	118	105	LMG 28391	Salininema	proteolyticum
Z0021_RO_G06_1_H09_B	118	105	LMG 28391	Salininema	proteolyticum
Z0021_RO_G07_1_H10_A	118	105	LMG 28391	Salininema	proteolyticum
Z0021_RO_G08_1_H10_B	118	105	LMG 28391	Salininema	proteolyticum
Z0021_RO_G09_1_H11_A	118	105	LMG 28391	Salininema	proteolyticum
Z0021_RO_G11_1_H12_A	118	105	LMG 28391	Salininema	proteolyticum
Z0021_RO_G12_1_H12_B	118	105	LMG 28391	Salininema	proteolyticum
Z0021_RO_H01_2_H07_A	118	105	LMG 28391	Salininema	proteolyticum
Z0021_RO_H02_2_H07_B	118	105	LMG 28391	Salininema	proteolyticum
Z0021_RO_H03_2_H08_A	118	105	LMG 28391	Salininema	proteolyticum
Z0021_RO_H04_2_H08_B	118	105	LMG 28391	Salininema	proteolyticum
Z0022_LB_A01_2_D06_A	119	107	LMG 2864	Marinomonas	communis
Z0022_LB_A02_2_D06_B	119	107	LMG 2864	Marinomonas	communis
Z0022_LB_A03_2_D05_A	119	107	LMG 2864	Marinomonas	communis
Z0022_LB_A04_2_D05_B	119	107	LMG 2864	Marinomonas	communis
Z0022_LB_A05_2_D04_A	119	107	LMG 2864	Marinomonas	communis
Z0022_LB_A06_2_D04_B	119	107	LMG 2864	Marinomonas	communis
Z0022_LB_A07_2_D03_A	119	107	LMG 2864	Marinomonas	communis
Z0022_LB_A08_2_D03_B	119	107	LMG 2864	Marinomonas	communis
Z0022_LB_A09_2_D02_A	119	107	LMG 2864	Marinomonas	communis
Z0022_LB_A10_2_D02_B	119	107	LMG 2864	Marinomonas	communis
Z0022_LB_A11_2_D01_A	119	107	LMG 2864	Marinomonas	communis
Z0022_LB_A12_2_D01_B	119	107	LMG 2864	Marinomonas	communis
Z0022_LB_B01_1_D06_A	119	107	LMG 2864	Marinomonas	communis
Z0022_LB_B02_1_D06_B	119	107	LMG 2864	Marinomonas	communis
Z0022_LO_B01_2_E01_A	119	107	LMG 2864	Marinomonas	communis
Z0022_LO_B08_2_E04_B	119	107	LMG 2864	Marinomonas	communis
Z0022_LO_B10_2_E05_B	119	107	LMG 2864	Marinomonas	communis
Z0022_RB_A02_2_D12_B	119	107	LMG 2864	Marinomonas	communis
Z0022_RB_A03_2_D11_A	119	107	LMG 2864	Marinomonas	communis
Z0022_RB_A04_2_D11_B	119	107	LMG 2864	Marinomonas	communis
Z0022_RB_A05_2_D10_A	119	107	LMG 2864	Marinomonas	communis
Z0022_RB_A06_2_D10_B	119	107	LMG 2864	Marinomonas	communis
Z0022_RB_A07_2_D09_A	119	107	LMG 2864	Marinomonas	communis
Z0022_RO_A08_1_E10_B	119	107	LMG 2864	Marinomonas	communis
Z0022_RO_A09_1_E11_A	119	107	LMG 2864	Marinomonas	communis
Z0022_RO_A10_1_E11_B	119	107	LMG 2864	Marinomonas	communis
Z0022_RO_A11_1_E12_A	119	107	LMG 2864	Marinomonas	communis
Z0022_RO_B01_2_E07_A	119	107	LMG 2864	Marinomonas	communis
Z0022_RO_B03_2_E08_A	119	107	LMG 2864	Marinomonas	communis
Z0022_RO_B04_2_E08_B	119	107	LMG 2864	Marinomonas	communis
Z0022_RO_C07_1_F10_A	119	107	LMG 2864	Marinomonas	communis

Z0022_RO_C08_1_F10_B	119	107	LMG 2864	Marinomonas	communis
Z0022_LB_F02_1_B06_B	120	108	LMG 29427	Nakamurella	silvestris
Z0022_LB_F03_1_B05_A	120	108	LMG 29427	Nakamurella	silvestris
Z0022_LB_F04_1_B05_B	120	108	LMG 29427	Nakamurella	silvestris
Z0022_LB_F05_1_B04_A	120	108	LMG 29427	Nakamurella	silvestris
Z0022_LB_F06_1_B04_B	120	108	LMG 29427	Nakamurella	silvestris
Z0022_LB_F07_1_B03_A	120	108	LMG 29427	Nakamurella	silvestris
Z0022_LB_F08_1_B03_B	120	108	LMG 29427	Nakamurella	silvestris
Z0022_LB_F09_1_B02_A	120	108	LMG 29427	Nakamurella	silvestris
Z0022_LB_F10_1_B02_B	120	108	LMG 29427	Nakamurella	silvestris
Z0022_LB_F11_1_B01_A	120	108	LMG 29427	Nakamurella	silvestris
Z0022_LB_F12_1_B01_B	120	108	LMG 29427	Nakamurella	silvestris
Z0022_LB_G01_2_A06_A	120	108	LMG 29427	Nakamurella	silvestris
Z0022_LB_G02_2_A06_B	120	108	LMG 29427	Nakamurella	silvestris
Z0022_LB_G03_2_A05_A	120	108	LMG 29427	Nakamurella	silvestris
Z0022_LB_G04_2_A05_B	120	108	LMG 29427	Nakamurella	silvestris
Z0022_RB_E09_2_B08_A	120	108	LMG 29427	Nakamurella	silvestris
Z0022_RB_E10_2_B08_B	120	108	LMG 29427	Nakamurella	silvestris
Z0022_RB_E11_2_B07_A	120	108	LMG 29427	Nakamurella	silvestris
Z0022_RB_E12_2_B07_B	120	108	LMG 29427	Nakamurella	silvestris
Z0022_RB_F01_1_B12_A	120	108	LMG 29427	Nakamurella	silvestris
Z0022_RB_F02_1_B12_B	120	108	LMG 29427	Nakamurella	silvestris
Z0022_RB_F03_1_B11_A	120	108	LMG 29427	Nakamurella	silvestris
Z0022_RB_F04_1_B11_B	120	108	LMG 29427	Nakamurella	silvestris
Z0022_RB_F05_1_B10_A	120	108	LMG 29427	Nakamurella	silvestris
Z0022_RB_F06_1_B10_B	120	108	LMG 29427	Nakamurella	silvestris
Z0022_RB_F07_1_B09_A	120	108	LMG 29427	Nakamurella	silvestris
Z0022_RB_F08_1_B09_B	120	108	LMG 29427	Nakamurella	silvestris
Z0022_RB_F09_1_B08_A	120	108	LMG 29427	Nakamurella	silvestris
Z0022_RB_F10_1_B08_B	120	108	LMG 29427	Nakamurella	silvestris
Z0022_RB_F11_1_B07_A	120	108	LMG 29427	Nakamurella	silvestris
Z0022_RB_F12_1_B07_B	120	108	LMG 29427	Nakamurella	silvestris
Z0022_RB_G01_2_A12_A	120	108	LMG 29427	Nakamurella	silvestris
Z0022_LB_G05_2_A04_A	121	109	LMG 3252	Citrobacter	youngae
Z0022_LB_G06_2_A04_B	121	109	LMG 3252	Citrobacter	youngae
Z0022_LB_G07_2_A03_A	121	109	LMG 3252	Citrobacter	youngae
Z0022_LB_G08_2_A03_B	121	109	LMG 3252	Citrobacter	youngae
Z0022_LB_G09_2_A02_A	121	109	LMG 3252	Citrobacter	youngae
Z0022_LB_G10_2_A02_B	121	109	LMG 3252	Citrobacter	youngae
Z0022_LB_G11_2_A01_A	121	109	LMG 3252	Citrobacter	youngae
Z0022_LB_G12_2_A01_B	121	109	LMG 3252	Citrobacter	youngae
Z0022_LB_H01_1_A06_A	121	109	LMG 3252	Citrobacter	youngae
Z0022_LB_H02_1_A06_B	121	109	LMG 3252	Citrobacter	youngae
Z0022_LB_H03_1_A05_A	121	109	LMG 3252	Citrobacter	youngae
Z0022_LB_H04_1_A05_B	121	109	LMG 3252	Citrobacter	youngae
Z0022_LB_H05_1_A04_A	121	109	LMG 3252	Citrobacter	youngae
Z0022_LB_H06_1_A04_B	121	109	LMG 3252	Citrobacter	youngae
Z0022_LB_H07_1_A03_A	121	109	LMG 3252	Citrobacter	youngae
Z0022_LB_H08_1_A03_B	121	109	LMG 3252	Citrobacter	youngae
Z0022_RB_G02_2_A12_B	121	109	LMG 3252	Citrobacter	youngae

Z0022_RB_G03_2_A11_A	121	109	LMG 3252	Citrobacter	youngae
Z0022_RB_G04_2_A11_B	121	109	LMG 3252	Citrobacter	youngae
Z0022_RB_G05_2_A10_A	121	109	LMG 3252	Citrobacter	youngae
Z0022_RB_G06_2_A10_B	121	109	LMG 3252	Citrobacter	youngae
Z0022_RB_G07_2_A09_A	121	109	LMG 3252	Citrobacter	youngae
Z0022_RB_G08_2_A09_B	121	109	LMG 3252	Citrobacter	youngae
Z0022_RB_G09_2_A08_A	121	109	LMG 3252	Citrobacter	youngae
Z0022_RB_G10_2_A08_B	121	109	LMG 3252	Citrobacter	youngae
Z0022_RB_G11_2_A07_A	121	109	LMG 3252	Citrobacter	youngae
Z0022_RB_G12_2_A07_B	121	109	LMG 3252	Citrobacter	youngae
Z0022_RB_H01_1_A12_A	121	109	LMG 3252	Citrobacter	youngae
Z0022_RB_H02_1_A12_B	121	109	LMG 3252	Citrobacter	youngae
Z0022_RB_H03_1_A11_A	121	109	LMG 3252	Citrobacter	youngae
Z0022_RB_H04_1_A11_B	121	109	LMG 3252	Citrobacter	youngae
Z0022_RB_H05_1_A10_A	121	109	LMG 3252	Citrobacter	youngae
Z0022_LO_A02_1_E01_B	122	114	LMG 4050	Micrococcus	luteus
Z0022_LO_A03_1_E02_A	122	114	LMG 4050	Micrococcus	luteus
Z0022_LO_A11_1_E06_A	122	114	LMG 4050	Micrococcus	luteus
Z0022_LO_C01_1_F01_A	122	114	LMG 4050	Micrococcus	luteus
Z0022_LO_C02_1_F01_B	122	114	LMG 4050	Micrococcus	luteus
Z0022_LO_C03_1_F02_A	122	114	LMG 4050	Micrococcus	luteus
Z0022_LO_D06_2_F03_B	122	114	LMG 4050	Micrococcus	luteus
Z0022_LO_D07_2_F04_A	122	114	LMG 4050	Micrococcus	luteus
Z0022_LO_D08_2_F04_B	122	114	LMG 4050	Micrococcus	luteus
Z0022_LO_D10_2_F05_B	122	114	LMG 4050	Micrococcus	luteus
Z0022_LO_D11_2_F06_A	122	114	LMG 4050	Micrococcus	luteus
Z0022_LO_E01_1_G01_A	122	114	LMG 4050	Micrococcus	luteus
Z0022_LO_E02_1_G01_B	122	114	LMG 4050	Micrococcus	luteus
Z0022_LO_E04_1_G02_B	122	114	LMG 4050	Micrococcus	luteus
Z0022_LO_E05_1_G03_A	122	114	LMG 4050	Micrococcus	luteus
Z0022_LO_E09_1_G05_A	122	114	LMG 4050	Micrococcus	luteus
Z0022_LO_E10_1_G05_B	122	114	LMG 4050	Micrococcus	luteus
Z0022_LO_E11_1_G06_A	122	114	LMG 4050	Micrococcus	luteus
Z0022_RO_B09_2_E11_A	122	114	LMG 4050	Micrococcus	luteus
Z0022_RO_B10_2_E11_B	122	114	LMG 4050	Micrococcus	luteus
Z0022_RO_B11_2_E12_A	122	114	LMG 4050	Micrococcus	luteus
Z0022_RO_B12_2_E12_B	122	114	LMG 4050	Micrococcus	luteus
Z0022_RO_C01_1_F07_A	122	114	LMG 4050	Micrococcus	luteus
Z0022_RO_C02_1_F07_B	122	114	LMG 4050	Micrococcus	luteus
Z0022_RO_C03_1_F08_A	122	114	LMG 4050	Micrococcus	luteus
Z0022_RO_C04_1_F08_B	122	114	LMG 4050	Micrococcus	luteus
Z0022_RO_C05_1_F09_A	122	114	LMG 4050	Micrococcus	luteus
Z0022_RO_C06_1_F09_B	122	114	LMG 4050	Micrococcus	luteus
Z0022_RO_D01_2_F07_A	122	114	LMG 4050	Micrococcus	luteus
Z0022_RO_D04_2_F08_B	122	114	LMG 4050	Micrococcus	luteus
Z0022_RO_D07_2_F10_A	122	114	LMG 4050	Micrococcus	luteus
Z0022_RO_D11_2_F12_A	122	114	LMG 4050	Micrococcus	luteus
Z0022_LO_B03_2_E02_A	123	110	LMG 3516	Paenalcaligenes	hominis
Z0022_LO_B04_2_E02_B	123	110	LMG 3516	Paenalcaligenes	hominis
Z0022_LO_B05_2_E03_A	123	110	LMG 3516	Paenalcaligenes	hominis

Z0022_LO_B06_2_E03_B	123	110	LMG 3516	Paenalcaligenes	hominis
Z0022_LO_B07_2_E04_A	123	110	LMG 3516	Paenalcaligenes	hominis
Z0022_LO_B09_2_E05_A	123	110	LMG 3516	Paenalcaligenes	hominis
Z0022_LO_B11_2_E06_A	123	110	LMG 3516	Paenalcaligenes	hominis
Z0022_LO_B12_2_E06_B	123	110	LMG 3516	Paenalcaligenes	hominis
Z0022_LO_C04_1_F02_B	123	110	LMG 3516	Paenalcaligenes	hominis
Z0022_LO_C05_1_F03_A	123	110	LMG 3516	Paenalcaligenes	hominis
Z0022_LO_C06_1_F03_B	123	110	LMG 3516	Paenalcaligenes	hominis
Z0022_LO_C07_1_F04_A	123	110	LMG 3516	Paenalcaligenes	hominis
Z0022_LO_C08_1_F04_B	123	110	LMG 3516	Paenalcaligenes	hominis
Z0022_LO_C09_1_F05_A	123	110	LMG 3516	Paenalcaligenes	hominis
Z0022_LO_C10_1_F05_B	123	110	LMG 3516	Paenalcaligenes	hominis
Z0022_LO_C11_1_F06_A	123	110	LMG 3516	Paenalcaligenes	hominis
Z0022_LO_C12_1_F06_B	123	110	LMG 3516	Paenalcaligenes	hominis
Z0022_LO_D01_2_F01_A	123	110	LMG 3516	Paenalcaligenes	hominis
Z0022_LO_D02_2_F01_B	123	110	LMG 3516	Paenalcaligenes	hominis
Z0022_LO_D03_2_F02_A	123	110	LMG 3516	Paenalcaligenes	hominis
Z0022_LO_D04_2_F02_B	123	110	LMG 3516	Paenalcaligenes	hominis
Z0022_LO_D05_2_F03_A	123	110	LMG 3516	Paenalcaligenes	hominis
Z0022_RO_A12_1_E12_B	123	110	LMG 3516	Paenalcaligenes	hominis
Z0022_RO_B02_2_E07_B	123	110	LMG 3516	Paenalcaligenes	hominis
Z0022_RO_B05_2_E09_A	123	110	LMG 3516	Paenalcaligenes	hominis
Z0022_RO_B06_2_E09_B	123	110	LMG 3516	Paenalcaligenes	hominis
Z0022_RO_B07_2_E10_A	123	110	LMG 3516	Paenalcaligenes	hominis
Z0022_RO_B08_2_E10_B	123	110	LMG 3516	Paenalcaligenes	hominis
Z0022_LO_D09_2_F05_A	124	111	LMG 3897	Listonella	pelagia
Z0022_LO_D12_2_F06_B	124	111	LMG 3897	Listonella	pelagia
Z0022_LO_E03_1_G02_A	124	111	LMG 3897	Listonella	pelagia
Z0022_LO_E06_1_G03_B	124	111	LMG 3897	Listonella	pelagia
Z0022_LO_E07_1_G04_A	124	111	LMG 3897	Listonella	pelagia
Z0022_LO_E12_1_G06_B	124	111	LMG 3897	Listonella	pelagia
Z0022_LO_F10_2_G05_B	124	111	LMG 3897	Listonella	pelagia
Z0022_LO_F12_2_G06_B	124	111	LMG 3897	Listonella	pelagia
Z0022_LO_G01_1_H01_A	124	111	LMG 3897	Listonella	pelagia
Z0022_LO_G02_1_H01_B	124	111	LMG 3897	Listonella	pelagia
Z0022_RO_C09_1_F11_A	124	111	LMG 3897	Listonella	pelagia
Z0022_RO_C10_1_F11_B	124	111	LMG 3897	Listonella	pelagia
Z0022_RO_C11_1_F12_A	124	111	LMG 3897	Listonella	pelagia
Z0022_RO_C12_1_F12_B	124	111	LMG 3897	Listonella	pelagia
Z0022_RO_D02_2_F07_B	124	111	LMG 3897	Listonella	pelagia
Z0022_RO_D03_2_F08_A	124	111	LMG 3897	Listonella	pelagia
Z0022_RO_D05_2_F09_A	124	111	LMG 3897	Listonella	pelagia
Z0022_RO_D06_2_F09_B	124	111	LMG 3897	Listonella	pelagia
Z0022_RO_D08_2_F10_B	124	111	LMG 3897	Listonella	pelagia
Z0022_RO_D09_2_F11_A	124	111	LMG 3897	Listonella	pelagia
Z0022_RO_D12_2_F12_B	124	111	LMG 3897	Listonella	pelagia
Z0022_RO_E01_1_G07_A	124	111	LMG 3897	Listonella	pelagia
Z0022_RO_E02_1_G07_B	124	111	LMG 3897	Listonella	pelagia
Z0022_RO_E03_1_G08_A	124	111	LMG 3897	Listonella	pelagia
Z0022_RO_E05_1_G09_A	124	111	LMG 3897	Listonella	pelagia

Z0022_RO_E06_1_G09_B	124	111	LMG 3897	Listonella	pelagia
Z0022_RO_E07_1_G10_A	124	111	LMG 3897	Listonella	pelagia
Z0022_RO_E08_1_G10_B	124	111	LMG 3897	Listonella	pelagia
Z0022_RO_E09_1_G11_A	124	111	LMG 3897	Listonella	pelagia
Z0022_RO_E10_1_G11_B	124	111	LMG 3897	Listonella	pelagia
Z0022_RO_E11_1_G12_A	124	111	LMG 3897	Listonella	pelagia
Z0022_RO_F01_2_G07_A	124	111	LMG 3897	Listonella	pelagia
Z0022_LO_E08_1_G04_B	125	113	LMG 4044	Vibrio	harveyi
Z0022_LO_F01_2_G01_A	125	113	LMG 4044	Vibrio	harveyi
Z0022_LO_F02_2_G01_B	125	113	LMG 4044	Vibrio	harveyi
Z0022_LO_F03_2_G02_A	125	113	LMG 4044	Vibrio	harveyi
Z0022_LO_F04_2_G02_B	125	113	LMG 4044	Vibrio	harveyi
Z0022_LO_F05_2_G03_A	125	113	LMG 4044	Vibrio	harveyi
Z0022_LO_F06_2_G03_B	125	113	LMG 4044	Vibrio	harveyi
Z0022_LO_F07_2_G04_A	125	113	LMG 4044	Vibrio	harveyi
Z0022_LO_F08_2_G04_B	125	113	LMG 4044	Vibrio	harveyi
Z0022_LO_F09_2_G05_A	125	113	LMG 4044	Vibrio	harveyi
Z0022_LO_F11_2_G06_A	125	113	LMG 4044	Vibrio	harveyi
Z0022_LO_G04_1_H02_B	125	113	LMG 4044	Vibrio	harveyi
Z0022_LO_G09_1_H05_A	125	113	LMG 4044	Vibrio	harveyi
Z0022_LO_G10_1_H05_B	125	113	LMG 4044	Vibrio	harveyi
Z0022_LO_H01_2_H01_A	125	113	LMG 4044	Vibrio	harveyi
Z0022_LO_H02_2_H01_B	125	113	LMG 4044	Vibrio	harveyi
Z0022_LO_H03_2_H02_A	125	113	LMG 4044	Vibrio	harveyi
Z0022_LO_H04_2_H02_B	125	113	LMG 4044	Vibrio	harveyi
Z0022_LO_H05_2_H03_A	125	113	LMG 4044	Vibrio	harveyi
Z0022_RO_D10_2_F11_B	125	113	LMG 4044	Vibrio	harveyi
Z0022_RO_E04_1_G08_B	125	113	LMG 4044	Vibrio	harveyi
Z0022_RO_E12_1_G12_B	125	113	LMG 4044	Vibrio	harveyi
Z0022_RO_F02_2_G07_B	125	113	LMG 4044	Vibrio	harveyi
Z0022_RO_F03_2_G08_A	125	113	LMG 4044	Vibrio	harveyi
Z0022_RO_F04_2_G08_B	125	113	LMG 4044	Vibrio	harveyi
Z0022_RO_F05_2_G09_A	125	113	LMG 4044	Vibrio	harveyi
Z0022_RO_F06_2_G09_B	125	113	LMG 4044	Vibrio	harveyi
Z0022_RO_F08_2_G10_B	125	113	LMG 4044	Vibrio	harveyi
Z0022_RO_F09_2_G11_A	125	113	LMG 4044	Vibrio	harveyi
Z0022_RO_F10_2_G11_B	125	113	LMG 4044	Vibrio	harveyi
Z0022_RO_F11_2_G12_A	125	113	LMG 4044	Vibrio	harveyi
Z0022_RO_G03_1_H08_A	125	113	LMG 4044	Vibrio	harveyi
Z0022_LO_G03_1_H02_A	126	112	LMG 4008	Flavobacterium	aquatile
Z0022_LO_G05_1_H03_A	126	112	LMG 4008	Flavobacterium	aquatile
Z0022_LO_G06_1_H03_B	126	112	LMG 4008	Flavobacterium	aquatile
Z0022_LO_G07_1_H04_A	126	112	LMG 4008	Flavobacterium	aquatile
Z0022_LO_G08_1_H04_B	126	112	LMG 4008	Flavobacterium	aquatile
Z0022_LO_G11_1_H06_A	126	112	LMG 4008	Flavobacterium	aquatile
Z0022_LO_G12_1_H06_B	126	112	LMG 4008	Flavobacterium	aquatile
Z0022_LO_H06_2_H03_B	126	112	LMG 4008	Flavobacterium	aquatile
Z0022_LO_H07_2_H04_A	126	112	LMG 4008	Flavobacterium	aquatile
Z0022_LO_H08_2_H04_B	126	112	LMG 4008	Flavobacterium	aquatile
Z0022_LO_H09_2_H05_A	126	112	LMG 4008	Flavobacterium	aquatile

Z0022_LO_H10_2_H05_B	126	112	LMG 4008	Flavobacterium	aquatile
Z0022_LO_H11_2_H06_A	126	112	LMG 4008	Flavobacterium	aquatile
Z0022_LO_H12_2_H06_B	126	112	LMG 4008	Flavobacterium	aquatile
Z0022_RB_A01_2_D12_A	126	112	LMG 4008	Flavobacterium	aquatile
Z0022_RO_F07_2_G10_A	126	112	LMG 4008	Flavobacterium	aquatile
Z0022_RO_F12_2_G12_B	126	112	LMG 4008	Flavobacterium	aquatile
Z0022_RO_G01_1_H07_A	126	112	LMG 4008	Flavobacterium	aquatile
Z0022_RO_G02_1_H07_B	126	112	LMG 4008	Flavobacterium	aquatile
Z0022_RO_G04_1_H08_B	126	112	LMG 4008	Flavobacterium	aquatile
Z0022_RO_G05_1_H09_A	126	112	LMG 4008	Flavobacterium	aquatile
Z0022_RO_G06_1_H09_B	126	112	LMG 4008	Flavobacterium	aquatile
Z0022_RO_G07_1_H10_A	126	112	LMG 4008	Flavobacterium	aquatile
Z0022_RO_G08_1_H10_B	126	112	LMG 4008	Flavobacterium	aquatile
Z0022_RO_G09_1_H11_A	126	112	LMG 4008	Flavobacterium	aquatile
Z0022_RO_G10_1_H11_B	126	112	LMG 4008	Flavobacterium	aquatile
Z0022_RO_G11_1_H12_A	126	112	LMG 4008	Flavobacterium	aquatile
Z0022_RO_G12_1_H12_B	126	112	LMG 4008	Flavobacterium	aquatile
Z0022_RO_H01_2_H07_A	126	112	LMG 4008	Flavobacterium	aquatile
Z0022_RO_H02_2_H07_B	126	112	LMG 4008	Flavobacterium	aquatile
Z0022_RO_H03_2_H08_A	126	112	LMG 4008	Flavobacterium	aquatile
Z0022_RO_H04_2_H08_B	126	112	LMG 4008	Flavobacterium	aquatile
Z0023_LB_A01_2_D06_A	127	115	LMG 4051	Deinococcus	radiodurans
Z0023_LB_A02_2_D06_B	127	115	LMG 4051	Deinococcus	radiodurans
Z0023_LB_A03_2_D05_A	127	115	LMG 4051	Deinococcus	radiodurans
Z0023_LB_A04_2_D05_B	127	115	LMG 4051	Deinococcus	radiodurans
Z0023_LB_A05_2_D04_A	127	115	LMG 4051	Deinococcus	radiodurans
Z0023_LB_A06_2_D04_B	127	115	LMG 4051	Deinococcus	radiodurans
Z0023_LB_A07_2_D03_A	127	115	LMG 4051	Deinococcus	radiodurans
Z0023_LB_A08_2_D03_B	127	115	LMG 4051	Deinococcus	radiodurans
Z0023_LB_A09_2_D02_A	127	115	LMG 4051	Deinococcus	radiodurans
Z0023_LB_A10_2_D02_B	127	115	LMG 4051	Deinococcus	radiodurans
Z0023_LB_A11_2_D01_A	127	115	LMG 4051	Deinococcus	radiodurans
Z0023_LB_A12_2_D01_B	127	115	LMG 4051	Deinococcus	radiodurans
Z0023_LB_B01_1_D06_A	127	115	LMG 4051	Deinococcus	radiodurans
Z0023_LB_B02_1_D06_B	127	115	LMG 4051	Deinococcus	radiodurans
Z0023_LB_B03_1_D05_A	127	115	LMG 4051	Deinococcus	radiodurans
Z0023_LB_B04_1_D05_B	127	115	LMG 4051	Deinococcus	radiodurans
Z0023_LO_H12_2_H06_B	127	115	LMG 4051	Deinococcus	radiodurans
Z0023_RB_A01_2_D12_A	127	115	LMG 4051	Deinococcus	radiodurans
Z0023_RB_A02_2_D12_B	127	115	LMG 4051	Deinococcus	radiodurans
Z0023_RB_A03_2_D11_A	127	115	LMG 4051	Deinococcus	radiodurans
Z0023_RB_A04_2_D11_B	127	115	LMG 4051	Deinococcus	radiodurans
Z0023_RB_A05_2_D10_A	127	115	LMG 4051	Deinococcus	radiodurans
Z0023_RB_A06_2_D10_B	127	115	LMG 4051	Deinococcus	radiodurans
Z0023_RB_A07_2_D09_A	127	115	LMG 4051	Deinococcus	radiodurans
Z0023_RB_A08_2_D09_B	127	115	LMG 4051	Deinococcus	radiodurans
Z0023_RB_A09_2_D08_A	127	115	LMG 4051	Deinococcus	radiodurans
Z0023_RB_H05_1_A10_A	127	115	LMG 4051	Deinococcus	radiodurans
Z0023_RB_H06_1_A10_B	127	115	LMG 4051	Deinococcus	radiodurans
Z0023_RO_A04_1_E08_B	127	115	LMG 4051	Deinococcus	radiodurans

Z0023_RO_B04_2_E08_B	127	115	LMG 4051	Deinococcus	radiodurans
Z0023_RO_B06_2_E09_B	127	115	LMG 4051	Deinococcus	radiodurans
Z0023_RO_B11_2_E12_A	127	115	LMG 4051	Deinococcus	radiodurans
Z0023_LB_B05_1_D04_A	128	116	LMG 4233	Photobacterium	phosphoreum
Z0023_LB_B06_1_D04_B	128	116	LMG 4233	Photobacterium	phosphoreum
Z0023_LB_B07_1_D03_A	128	116	LMG 4233	Photobacterium	phosphoreum
Z0023_LB_B08_1_D03_B	128	116	LMG 4233	Photobacterium	phosphoreum
Z0023_LB_B09_1_D02_A	128	116	LMG 4233	Photobacterium	phosphoreum
Z0023_LB_B10_1_D02_B	128	116	LMG 4233	Photobacterium	phosphoreum
Z0023_LB_B11_1_D01_A	128	116	LMG 4233	Photobacterium	phosphoreum
Z0023_LB_B12_1_D01_B	128	116	LMG 4233	Photobacterium	phosphoreum
Z0023_LB_C01_2_C06_A	128	116	LMG 4233	Photobacterium	phosphoreum
Z0023_LB_C02_2_C06_B	128	116	LMG 4233	Photobacterium	phosphoreum
Z0023_LB_C03_2_C05_A	128	116	LMG 4233	Photobacterium	phosphoreum
Z0023_LB_C04_2_C05_B	128	116	LMG 4233	Photobacterium	phosphoreum
Z0023_LB_C05_2_C04_A	128	116	LMG 4233	Photobacterium	phosphoreum
Z0023_LB_C06_2_C04_B	128	116	LMG 4233	Photobacterium	phosphoreum
Z0023_LB_C07_2_C03_A	128	116	LMG 4233	Photobacterium	phosphoreum
Z0023_RB_A10_2_D08_B	128	116	LMG 4233	Photobacterium	phosphoreum
Z0023_RB_A11_2_D07_A	128	116	LMG 4233	Photobacterium	phosphoreum
Z0023_RB_A12_2_D07_B	128	116	LMG 4233	Photobacterium	phosphoreum
Z0023_RB_B01_1_D12_A	128	116	LMG 4233	Photobacterium	phosphoreum
Z0023_RB_B02_1_D12_B	128	116	LMG 4233	Photobacterium	phosphoreum
Z0023_RB_B03_1_D11_A	128	116	LMG 4233	Photobacterium	phosphoreum
Z0023_RB_B04_1_D11_B	128	116	LMG 4233	Photobacterium	phosphoreum
Z0023_RB_B05_1_D10_A	128	116	LMG 4233	Photobacterium	phosphoreum
Z0023_RB_B06_1_D10_B	128	116	LMG 4233	Photobacterium	phosphoreum
Z0023_RB_B07_1_D09_A	128	116	LMG 4233	Photobacterium	phosphoreum
Z0023_RB_B08_1_D09_B	128	116	LMG 4233	Photobacterium	phosphoreum
Z0023_RB_B09_1_D08_A	128	116	LMG 4233	Photobacterium	phosphoreum
Z0023_RB_B10_1_D08_B	128	116	LMG 4233	Photobacterium	phosphoreum
Z0023_RB_B11_1_D07_A	128	116	LMG 4233	Photobacterium	phosphoreum
Z0023_RB_B12_1_D07_B	128	116	LMG 4233	Photobacterium	phosphoreum
Z0023_RB_C01_2_C12_A	128	116	LMG 4233	Photobacterium	phosphoreum
Z0023_RB_C02_2_C12_B	128	116	LMG 4233	Photobacterium	phosphoreum
Z0023_LB_C08_2_C03_B	129	117	LMG 4305	Rhodobacter	blasticus
Z0023_LB_C09_2_C02_A	129	117	LMG 4305	Rhodobacter	blasticus
Z0023_LB_C10_2_C02_B	129	117	LMG 4305	Rhodobacter	blasticus
Z0023_LB_C11_2_C01_A	129	117	LMG 4305	Rhodobacter	blasticus
Z0023_LB_C12_2_C01_B	129	117	LMG 4305	Rhodobacter	blasticus
Z0023_LB_D01_1_C06_A	129	117	LMG 4305	Rhodobacter	blasticus
Z0023_LB_D02_1_C06_B	129	117	LMG 4305	Rhodobacter	blasticus
Z0023_LB_D03_1_C05_A	129	117	LMG 4305	Rhodobacter	blasticus
Z0023_LB_D04_1_C05_B	129	117	LMG 4305	Rhodobacter	blasticus
Z0023_LB_D05_1_C04_A	129	117	LMG 4305	Rhodobacter	blasticus
Z0023_LB_D06_1_C04_B	129	117	LMG 4305	Rhodobacter	blasticus
Z0023_LB_D07_1_C03_A	129	117	LMG 4305	Rhodobacter	blasticus
Z0023_LB_D08_1_C03_B	129	117	LMG 4305	Rhodobacter	blasticus
Z0023_LB_D09_1_C02_A	129	117	LMG 4305	Rhodobacter	blasticus
Z0023_LB_D10_1_C02_B	129	117	LMG 4305	Rhodobacter	blasticus

Z0023_LB_D11_1_C01_A	129	117	LMG 4305	Rhodobacter	blasticus
Z0023_LB_D12_1_C01_B	129	117	LMG 4305	Rhodobacter	blasticus
Z0023_LB_E01_2_B06_A	129	117	LMG 4305	Rhodobacter	blasticus
Z0023_LB_E02_2_B06_B	129	117	LMG 4305	Rhodobacter	blasticus
Z0023_RB_C03_2_C11_A	129	117	LMG 4305	Rhodobacter	blasticus
Z0023_RB_C04_2_C11_B	129	117	LMG 4305	Rhodobacter	blasticus
Z0023_RB_C05_2_C10_A	129	117	LMG 4305	Rhodobacter	blasticus
Z0023_RB_C06_2_C10_B	129	117	LMG 4305	Rhodobacter	blasticus
Z0023_RB_C07_2_C09_A	129	117	LMG 4305	Rhodobacter	blasticus
Z0023_RB_C08_2_C09_B	129	117	LMG 4305	Rhodobacter	blasticus
Z0023_RB_C09_2_C08_A	129	117	LMG 4305	Rhodobacter	blasticus
Z0023_RB_C10_2_C08_B	129	117	LMG 4305	Rhodobacter	blasticus
Z0023_RB_C11_2_C07_A	129	117	LMG 4305	Rhodobacter	blasticus
Z0023_RB_C12_2_C07_B	129	117	LMG 4305	Rhodobacter	blasticus
Z0023_RB_D01_1_C12_A	129	117	LMG 4305	Rhodobacter	blasticus
Z0023_RB_D02_1_C12_B	129	117	LMG 4305	Rhodobacter	blasticus
Z0023_RB_D03_1_C11_A	129	117	LMG 4305	Rhodobacter	blasticus
Z0023_LB_E03_2_B05_A	130	118	LMG 4328	Curvibacter	delicatus
Z0023_LB_E04_2_B05_B	130	118	LMG 4328	Curvibacter	delicatus
Z0023_LB_E05_2_B04_A	130	118	LMG 4328	Curvibacter	delicatus
Z0023_LB_E06_2_B04_B	130	118	LMG 4328	Curvibacter	delicatus
Z0023_LB_E07_2_B03_A	130	118	LMG 4328	Curvibacter	delicatus
Z0023_LB_E08_2_B03_B	130	118	LMG 4328	Curvibacter	delicatus
Z0023_LB_E09_2_B02_A	130	118	LMG 4328	Curvibacter	delicatus
Z0023_LB_E10_2_B02_B	130	118	LMG 4328	Curvibacter	delicatus
Z0023_LB_E11_2_B01_A	130	118	LMG 4328	Curvibacter	delicatus
Z0023_LB_E12_2_B01_B	130	118	LMG 4328	Curvibacter	delicatus
Z0023_LB_F01_1_B06_A	130	118	LMG 4328	Curvibacter	delicatus
Z0023_LB_F02_1_B06_B	130	118	LMG 4328	Curvibacter	delicatus
Z0023_LB_F03_1_B05_A	130	118	LMG 4328	Curvibacter	delicatus
Z0023_LB_F04_1_B05_B	130	118	LMG 4328	Curvibacter	delicatus
Z0023_LB_F05_1_B04_A	130	118	LMG 4328	Curvibacter	delicatus
Z0023_RB_D04_1_C11_B	130	118	LMG 4328	Curvibacter	delicatus
Z0023_RB_D05_1_C10_A	130	118	LMG 4328	Curvibacter	delicatus
Z0023_RB_D06_1_C10_B	130	118	LMG 4328	Curvibacter	delicatus
Z0023_RB_D07_1_C09_A	130	118	LMG 4328	Curvibacter	delicatus
Z0023_RB_D08_1_C09_B	130	118	LMG 4328	Curvibacter	delicatus
Z0023_RB_D09_1_C08_A	130	118	LMG 4328	Curvibacter	delicatus
Z0023_RB_D10_1_C08_B	130	118	LMG 4328	Curvibacter	delicatus
Z0023_RB_D11_1_C07_A	130	118	LMG 4328	Curvibacter	delicatus
Z0023_RB_D12_1_C07_B	130	118	LMG 4328	Curvibacter	delicatus
Z0023_RB_E01_2_B12_A	130	118	LMG 4328	Curvibacter	delicatus
Z0023_RB_E02_2_B12_B	130	118	LMG 4328	Curvibacter	delicatus
Z0023_RB_E03_2_B11_A	130	118	LMG 4328	Curvibacter	delicatus
Z0023_RB_E04_2_B11_B	130	118	LMG 4328	Curvibacter	delicatus
Z0023_RB_E05_2_B10_A	130	118	LMG 4328	Curvibacter	delicatus
Z0023_RB_E06_2_B10_B	130	118	LMG 4328	Curvibacter	delicatus
Z0023_RB_E07_2_B09_A	130	118	LMG 4328	Curvibacter	delicatus
Z0023_RB_E08_2_B09_B	130	118	LMG 4328	Curvibacter	delicatus
Z0023_LB_F06_1_B04_B	131	119	LMG 460	Zymomonas	mobilis

Z0023_LB_F07_1_B03_A	131	119	LMG 460	Zymomonas	mobilis
Z0023_LB_F08_1_B03_B	131	119	LMG 460	Zymomonas	mobilis
Z0023_LB_F09_1_B02_A	131	119	LMG 460	Zymomonas	mobilis
Z0023_LB_F10_1_B02_B	131	119	LMG 460	Zymomonas	mobilis
Z0023_LB_F11_1_B01_A	131	119	LMG 460	Zymomonas	mobilis
Z0023_LB_F12_1_B01_B	131	119	LMG 460	Zymomonas	mobilis
Z0023_LB_G01_2_A06_A	131	119	LMG 460	Zymomonas	mobilis
Z0023_LB_G02_2_A06_B	131	119	LMG 460	Zymomonas	mobilis
Z0023_LB_G03_2_A05_A	131	119	LMG 460	Zymomonas	mobilis
Z0023_LB_G04_2_A05_B	131	119	LMG 460	Zymomonas	mobilis
Z0023_LB_G05_2_A04_A	131	119	LMG 460	Zymomonas	mobilis
Z0023_LB_G06_2_A04_B	131	119	LMG 460	Zymomonas	mobilis
Z0023_LB_G07_2_A03_A	131	119	LMG 460	Zymomonas	mobilis
Z0023_LB_G08_2_A03_B	131	119	LMG 460	Zymomonas	mobilis
Z0023_LB_G09_2_A02_A	131	119	LMG 460	Zymomonas	mobilis
Z0023_LB_G10_2_A02_B	131	119	LMG 460	Zymomonas	mobilis
Z0023_LB_G11_2_A01_A	131	119	LMG 460	Zymomonas	mobilis
Z0023_LB_G12_2_A01_B	131	119	LMG 460	Zymomonas	mobilis
Z0023_RB_E09_2_B08_A	131	119	LMG 460	Zymomonas	mobilis
Z0023_RB_E10_2_B08_B	131	119	LMG 460	Zymomonas	mobilis
Z0023_RB_E11_2_B07_A	131	119	LMG 460	Zymomonas	mobilis
Z0023_RB_E12_2_B07_B	131	119	LMG 460	Zymomonas	mobilis
Z0023_RB_F01_1_B12_A	131	119	LMG 460	Zymomonas	mobilis
Z0023_RB_F02_1_B12_B	131	119	LMG 460	Zymomonas	mobilis
Z0023_RB_F03_1_B11_A	131	119	LMG 460	Zymomonas	mobilis
Z0023_RB_F04_1_B11_B	131	119	LMG 460	Zymomonas	mobilis
Z0023_RB_F05_1_B10_A	131	119	LMG 460	Zymomonas	mobilis
Z0023_RB_F06_1_B10_B	131	119	LMG 460	Zymomonas	mobilis
Z0023_RB_F07_1_B09_A	131	119	LMG 460	Zymomonas	mobilis
Z0023_RB_F08_1_B09_B	131	119	LMG 460	Zymomonas	mobilis
Z0023_RB_F09_1_B08_A	131	119	LMG 460	Zymomonas	mobilis
Z0023_LB_H01_1_A06_A	132	120	LMG 5019	Serratia	rubidaea
Z0023_LB_H02_1_A06_B	132	120	LMG 5019	Serratia	rubidaea
Z0023_LB_H03_1_A05_A	132	120	LMG 5019	Serratia	rubidaea
Z0023_LB_H04_1_A05_B	132	120	LMG 5019	Serratia	rubidaea
Z0023_LB_H05_1_A04_A	132	120	LMG 5019	Serratia	rubidaea
Z0023_LB_H06_1_A04_B	132	120	LMG 5019	Serratia	rubidaea
Z0023_LB_H07_1_A03_A	132	120	LMG 5019	Serratia	rubidaea
Z0023_LB_H08_1_A03_B	132	120	LMG 5019	Serratia	rubidaea
Z0023_LB_H09_1_A02_A	132	120	LMG 5019	Serratia	rubidaea
Z0023_LB_H10_1_A02_B	132	120	LMG 5019	Serratia	rubidaea
Z0023_LB_H11_1_A01_A	132	120	LMG 5019	Serratia	rubidaea
Z0023_LB_H12_1_A01_B	132	120	LMG 5019	Serratia	rubidaea
Z0023_LO_A01_1_E01_A	132	120	LMG 5019	Serratia	rubidaea
Z0023_LO_A02_1_E01_B	132	120	LMG 5019	Serratia	rubidaea
Z0023_LO_A03_1_E02_A	132	120	LMG 5019	Serratia	rubidaea
Z0023_LO_A04_1_E02_B	132	120	LMG 5019	Serratia	rubidaea
Z0023_LO_A05_1_E03_A	132	120	LMG 5019	Serratia	rubidaea
Z0023_LO_A06_1_E03_B	132	120	LMG 5019	Serratia	rubidaea
Z0023_RB_F10_1_B08_B	132	120	LMG 5019	Serratia	rubidaea

Z0023_RB_F11_1_B07_A	132	120	LMG 5019	Serratia	rubidaea
Z0023_RB_F12_1_B07_B	132	120	LMG 5019	Serratia	rubidaea
Z0023_RB_G01_2_A12_A	132	120	LMG 5019	Serratia	rubidaea
Z0023_RB_G02_2_A12_B	132	120	LMG 5019	Serratia	rubidaea
Z0023_RB_G03_2_A11_A	132	120	LMG 5019	Serratia	rubidaea
Z0023_RB_G04_2_A11_B	132	120	LMG 5019	Serratia	rubidaea
Z0023_RB_G05_2_A10_A	132	120	LMG 5019	Serratia	rubidaea
Z0023_RB_G06_2_A10_B	132	120	LMG 5019	Serratia	rubidaea
Z0023_RB_G07_2_A09_A	132	120	LMG 5019	Serratia	rubidaea
Z0023_RB_G08_2_A09_B	132	120	LMG 5019	Serratia	rubidaea
Z0023_RB_G09_2_A08_A	132	120	LMG 5019	Serratia	rubidaea
Z0023_RB_G10_2_A08_B	132	120	LMG 5019	Serratia	rubidaea
Z0023_RB_G11_2_A07_A	132	120	LMG 5019	Serratia	rubidaea
Z0023_LO_A07_1_E04_A	133	121	LMG 5286	Acidovorax	cattleyae
Z0023_LO_A08_1_E04_B	133	121	LMG 5286	Acidovorax	cattleyae
Z0023_LO_A09_1_E05_A	133	121	LMG 5286	Acidovorax	cattleyae
Z0023_LO_A10_1_E05_B	133	121	LMG 5286	Acidovorax	cattleyae
Z0023_LO_A11_1_E06_A	133	121	LMG 5286	Acidovorax	cattleyae
Z0023_LO_A12_1_E06_B	133	121	LMG 5286	Acidovorax	cattleyae
Z0023_LO_B01_2_E01_A	133	121	LMG 5286	Acidovorax	cattleyae
Z0023_LO_B02_2_E01_B	133	121	LMG 5286	Acidovorax	cattleyae
Z0023_LO_B04_2_E02_B	133	121	LMG 5286	Acidovorax	cattleyae
Z0023_LO_B05_2_E03_A	133	121	LMG 5286	Acidovorax	cattleyae
Z0023_LO_B06_2_E03_B	133	121	LMG 5286	Acidovorax	cattleyae
Z0023_LO_B07_2_E04_A	133	121	LMG 5286	Acidovorax	cattleyae
Z0023_LO_B09_2_E05_A	133	121	LMG 5286	Acidovorax	cattleyae
Z0023_RB_G12_2_A07_B	133	121	LMG 5286	Acidovorax	cattleyae
Z0023_RB_H01_1_A12_A	133	121	LMG 5286	Acidovorax	cattleyae
Z0023_RB_H02_1_A12_B	133	121	LMG 5286	Acidovorax	cattleyae
Z0023_RB_H03_1_A11_A	133	121	LMG 5286	Acidovorax	cattleyae
Z0023_RB_H04_1_A11_B	133	121	LMG 5286	Acidovorax	cattleyae
Z0023_RB_H07_1_A09_A	133	121	LMG 5286	Acidovorax	cattleyae
Z0023_RB_H08_1_A09_B	133	121	LMG 5286	Acidovorax	cattleyae
Z0023_RB_H09_1_A08_A	133	121	LMG 5286	Acidovorax	cattleyae
Z0023_RB_H10_1_A08_B	133	121	LMG 5286	Acidovorax	cattleyae
Z0023_RB_H12_1_A07_B	133	121	LMG 5286	Acidovorax	cattleyae
Z0023_RO_A02_1_E07_B	133	121	LMG 5286	Acidovorax	cattleyae
Z0023_RO_A03_1_E08_A	133	121	LMG 5286	Acidovorax	cattleyae
Z0023_RO_A05_1_E09_A	133	121	LMG 5286	Acidovorax	cattleyae
Z0023_RO_A08_1_E10_B	133	121	LMG 5286	Acidovorax	cattleyae
Z0023_RO_A09_1_E11_A	133	121	LMG 5286	Acidovorax	cattleyae
Z0023_LO_B03_2_E02_A	134	123	LMG 6451	Bacteroides	ureolyticus
Z0023_LO_B08_2_E04_B	134	123	LMG 6451	Bacteroides	ureolyticus
Z0023_LO_B11_2_E06_A	134	123	LMG 6451	Bacteroides	ureolyticus
Z0023_LO_C05_1_F03_A	134	123	LMG 6451	Bacteroides	ureolyticus
Z0023_LO_C06_1_F03_B	134	123	LMG 6451	Bacteroides	ureolyticus
Z0023_LO_C07_1_F04_A	134	123	LMG 6451	Bacteroides	ureolyticus
Z0023_LO_C09_1_F05_A	134	123	LMG 6451	Bacteroides	ureolyticus
Z0023_LO_C10_1_F05_B	134	123	LMG 6451	Bacteroides	ureolyticus
Z0023_LO_C11_1_F06_A	134	123	LMG 6451	Bacteroides	ureolyticus

Z0023_LO_C12_1_F06_B	134	123	LMG 6451	Bacteroides	ureolyticus
Z0023_LO_D03_2_F02_A	134	123	LMG 6451	Bacteroides	ureolyticus
Z0023_LO_D04_2_F02_B	134	123	LMG 6451	Bacteroides	ureolyticus
Z0023_LO_D10_2_F05_B	134	123	LMG 6451	Bacteroides	ureolyticus
Z0023_LO_D11_2_F06_A	134	123	LMG 6451	Bacteroides	ureolyticus
Z0023_RB_H11_1_A07_A	134	123	LMG 6451	Bacteroides	ureolyticus
Z0023_RO_A01_1_E07_A	134	123	LMG 6451	Bacteroides	ureolyticus
Z0023_RO_A06_1_E09_B	134	123	LMG 6451	Bacteroides	ureolyticus
Z0023_RO_A07_1_E10_A	134	123	LMG 6451	Bacteroides	ureolyticus
Z0023_RO_A10_1_E11_B	134	123	LMG 6451	Bacteroides	ureolyticus
Z0023_RO_B01_2_E07_A	134	123	LMG 6451	Bacteroides	ureolyticus
Z0023_RO_B02_2_E07_B	134	123	LMG 6451	Bacteroides	ureolyticus
Z0023_RO_C05_1_F09_A	134	123	LMG 6451	Bacteroides	ureolyticus
Z0023_RO_C08_1_F10_B	134	123	LMG 6451	Bacteroides	ureolyticus
Z0023_RO_C09_1_F11_A	134	123	LMG 6451	Bacteroides	ureolyticus
Z0023_RO_D03_2_F08_A	134	123	LMG 6451	Bacteroides	ureolyticus
Z0023_RO_D04_2_F08_B	134	123	LMG 6451	Bacteroides	ureolyticus
Z0023_RO_D05_2_F09_A	134	123	LMG 6451	Bacteroides	ureolyticus
Z0023_RO_D06_2_F09_B	134	123	LMG 6451	Bacteroides	ureolyticus
Z0023_RO_D07_2_F10_A	134	123	LMG 6451	Bacteroides	ureolyticus
Z0023_RO_D08_2_F10_B	134	123	LMG 6451	Bacteroides	ureolyticus
Z0023_RO_D09_2_F11_A	134	123	LMG 6451	Bacteroides	ureolyticus
Z0023_RO_D12_2_F12_B	134	123	LMG 6451	Bacteroides	ureolyticus
Z0023_LO_G03_1_H02_A	135	122	LMG 5743	Xanthomonas	populi
Z0023_LO_G04_1_H02_B	135	122	LMG 5743	Xanthomonas	populi
Z0023_LO_G10_1_H05_B	135	122	LMG 5743	Xanthomonas	populi
Z0023_LO_G11_1_H06_A	135	122	LMG 5743	Xanthomonas	populi
Z0023_LO_G12_1_H06_B	135	122	LMG 5743	Xanthomonas	populi
Z0023_LO_H01_2_H01_A	135	122	LMG 5743	Xanthomonas	populi
Z0023_LO_H02_2_H01_B	135	122	LMG 5743	Xanthomonas	populi
Z0023_LO_H03_2_H02_A	135	122	LMG 5743	Xanthomonas	populi
Z0023_LO_H04_2_H02_B	135	122	LMG 5743	Xanthomonas	populi
Z0023_LO_H05_2_H03_A	135	122	LMG 5743	Xanthomonas	populi
Z0023_LO_H06_2_H03_B	135	122	LMG 5743	Xanthomonas	populi
Z0023_LO_H07_2_H04_A	135	122	LMG 5743	Xanthomonas	populi
Z0023_LO_H08_2_H04_B	135	122	LMG 5743	Xanthomonas	populi
Z0023_LO_H09_2_H05_A	135	122	LMG 5743	Xanthomonas	populi
Z0023_LO_H10_2_H05_B	135	122	LMG 5743	Xanthomonas	populi
Z0023_LO_H11_2_H06_A	135	122	LMG 5743	Xanthomonas	populi
Z0023_RO_F09_2_G11_A	135	122	LMG 5743	Xanthomonas	populi
Z0023_RO_F10_2_G11_B	135	122	LMG 5743	Xanthomonas	populi
Z0023_RO_F11_2_G12_A	135	122	LMG 5743	Xanthomonas	populi
Z0023_RO_F12_2_G12_B	135	122	LMG 5743	Xanthomonas	populi
Z0023_RO_G01_1_H07_A	135	122	LMG 5743	Xanthomonas	populi
Z0023_RO_G02_1_H07_B	135	122	LMG 5743	Xanthomonas	populi
Z0023_RO_G04_1_H08_B	135	122	LMG 5743	Xanthomonas	populi
Z0023_RO_G05_1_H09_A	135	122	LMG 5743	Xanthomonas	populi
Z0023_RO_G06_1_H09_B	135	122	LMG 5743	Xanthomonas	populi
Z0023_RO_G10_1_H11_B	135	122	LMG 5743	Xanthomonas	populi
Z0023_RO_G11_1_H12_A	135	122	LMG 5743	Xanthomonas	populi

Z0023_RO_G12_1_H12_B	135	122	LMG 5743	Xanthomonas	populi
Z0023_RO_H01_2_H07_A	135	122	LMG 5743	Xanthomonas	populi
Z0023_RO_H02_2_H07_B	135	122	LMG 5743	Xanthomonas	populi
Z0023_RO_H03_2_H08_A	135	122	LMG 5743	Xanthomonas	populi
Z0023_RO_H04_2_H08_B	135	122	LMG 5743	Xanthomonas	populi
Z0024_LB_B04_1_D05_B	136	124	LMG 6519	Oligella	ureolytica
Z0024_LB_B05_1_D04_A	136	124	LMG 6519	Oligella	ureolytica
Z0024_LB_B06_1_D04_B	136	124	LMG 6519	Oligella	ureolytica
Z0024_LB_B07_1_D03_A	136	124	LMG 6519	Oligella	ureolytica
Z0024_LB_B08_1_D03_B	136	124	LMG 6519	Oligella	ureolytica
Z0024_LB_B09_1_D02_A	136	124	LMG 6519	Oligella	ureolytica
Z0024_LB_B10_1_D02_B	136	124	LMG 6519	Oligella	ureolytica
Z0024_LB_B11_1_D01_A	136	124	LMG 6519	Oligella	ureolytica
Z0024_LB_B12_1_D01_B	136	124	LMG 6519	Oligella	ureolytica
Z0024_LB_C01_2_C06_A	136	124	LMG 6519	Oligella	ureolytica
Z0024_LB_C02_2_C06_B	136	124	LMG 6519	Oligella	ureolytica
Z0024_LB_C03_2_C05_A	136	124	LMG 6519	Oligella	ureolytica
Z0024_LB_C04_2_C05_B	136	124	LMG 6519	Oligella	ureolytica
Z0024_LB_C05_2_C04_A	136	124	LMG 6519	Oligella	ureolytica
Z0024_LB_C06_2_C04_B	136	124	LMG 6519	Oligella	ureolytica
Z0024_LB_C07_2_C03_A	136	124	LMG 6519	Oligella	ureolytica
Z0024_LB_C08_2_C03_B	136	124	LMG 6519	Oligella	ureolytica
Z0024_LB_C09_2_C02_A	136	124	LMG 6519	Oligella	ureolytica
Z0024_RB_C01_2_C12_A	136	124	LMG 6519	Oligella	ureolytica
Z0024_RB_C02_2_C12_B	136	124	LMG 6519	Oligella	ureolytica
Z0024_RB_C03_2_C11_A	136	124	LMG 6519	Oligella	ureolytica
Z0024_RB_C04_2_C11_B	136	124	LMG 6519	Oligella	ureolytica
Z0024_RB_C05_2_C10_A	136	124	LMG 6519	Oligella	ureolytica
Z0024_RB_C06_2_C10_B	136	124	LMG 6519	Oligella	ureolytica
Z0024_RB_C07_2_C09_A	136	124	LMG 6519	Oligella	ureolytica
Z0024_RB_C08_2_C09_B	136	124	LMG 6519	Oligella	ureolytica
Z0024_RB_C09_2_C08_A	136	124	LMG 6519	Oligella	ureolytica
Z0024_RB_C10_2_C08_B	136	124	LMG 6519	Oligella	ureolytica
Z0024_RB_C11_2_C07_A	136	124	LMG 6519	Oligella	ureolytica
Z0024_RB_C12_2_C07_B	136	124	LMG 6519	Oligella	ureolytica
Z0024_RB_D01_1_C12_A	136	124	LMG 6519	Oligella	ureolytica
Z0024_RB_D02_1_C12_B	136	124	LMG 6519	Oligella	ureolytica
Z0024_LB_C10_2_C02_B	137	125	LMG 6866	Ralstonia	mannitolilytica
Z0024_LB_C11_2_C01_A	137	125	LMG 6866	Ralstonia	mannitolilytica
Z0024_LB_C12_2_C01_B	137	125	LMG 6866	Ralstonia	mannitolilytica
Z0024_LB_D01_1_C06_A	137	125	LMG 6866	Ralstonia	mannitolilytica
Z0024_LB_D02_1_C06_B	137	125	LMG 6866	Ralstonia	mannitolilytica
Z0024_LB_D03_1_C05_A	137	125	LMG 6866	Ralstonia	mannitolilytica
Z0024_LB_D04_1_C05_B	137	125	LMG 6866	Ralstonia	mannitolilytica
Z0024_LB_D05_1_C04_A	137	125	LMG 6866	Ralstonia	mannitolilytica
Z0024_LB_D06_1_C04_B	137	125	LMG 6866	Ralstonia	mannitolilytica
Z0024_LB_D08_1_C03_B	137	125	LMG 6866	Ralstonia	mannitolilytica
Z0024_LB_D09_1_C02_A	137	125	LMG 6866	Ralstonia	mannitolilytica
Z0024_LB_D10_1_C02_B	137	125	LMG 6866	Ralstonia	mannitolilytica
Z0024_LB_D11_1_C01_A	137	125	LMG 6866	Ralstonia	mannitolilytica

Z0024_LB_D12_1_C01_B	137	125	LMG 6866	Ralstonia	mannitolilytica
Z0024_LB_E01_2_B06_A	137	125	LMG 6866	Ralstonia	mannitolilytica
Z0024_LB_E02_2_B06_B	137	125	LMG 6866	Ralstonia	mannitolilytica
Z0024_RB_D03_1_C11_A	137	125	LMG 6866	Ralstonia	mannitolilytica
Z0024_RB_D04_1_C11_B	137	125	LMG 6866	Ralstonia	mannitolilytica
Z0024_RB_D05_1_C10_A	137	125	LMG 6866	Ralstonia	mannitolilytica
Z0024_RB_D06_1_C10_B	137	125	LMG 6866	Ralstonia	mannitolilytica
Z0024_RB_D07_1_C09_A	137	125	LMG 6866	Ralstonia	mannitolilytica
Z0024_RB_D08_1_C09_B	137	125	LMG 6866	Ralstonia	mannitolilytica
Z0024_RB_D09_1_C08_A	137	125	LMG 6866	Ralstonia	mannitolilytica
Z0024_RB_D10_1_C08_B	137	125	LMG 6866	Ralstonia	mannitolilytica
Z0024_RB_D11_1_C07_A	137	125	LMG 6866	Ralstonia	mannitolilytica
Z0024_RB_D12_1_C07_B	137	125	LMG 6866	Ralstonia	mannitolilytica
Z0024_RB_E01_2_B12_A	137	125	LMG 6866	Ralstonia	mannitolilytica
Z0024_RB_E02_2_B12_B	137	125	LMG 6866	Ralstonia	mannitolilytica
Z0024_RB_E03_2_B11_A	137	125	LMG 6866	Ralstonia	mannitolilytica
Z0024_RB_E04_2_B11_B	137	125	LMG 6866	Ralstonia	mannitolilytica
Z0024_RB_E05_2_B10_A	137	125	LMG 6866	Ralstonia	mannitolilytica
Z0024_RB_E06_2_B10_B	137	125	LMG 6866	Ralstonia	mannitolilytica
Z0024_LB_D07_1_C03_A	138	126	LMG 6896	Streptococcus	thermophilus
Z0024_LB_E03_2_B05_A	138	126	LMG 6896	Streptococcus	thermophilus
Z0024_LB_E04_2_B05_B	138	126	LMG 6896	Streptococcus	thermophilus
Z0024_LB_E05_2_B04_A	138	126	LMG 6896	Streptococcus	thermophilus
Z0024_LB_E06_2_B04_B	138	126	LMG 6896	Streptococcus	thermophilus
Z0024_LB_E07_2_B03_A	138	126	LMG 6896	Streptococcus	thermophilus
Z0024_LB_E08_2_B03_B	138	126	LMG 6896	Streptococcus	thermophilus
Z0024_LB_E09_2_B02_A	138	126	LMG 6896	Streptococcus	thermophilus
Z0024_LB_E10_2_B02_B	138	126	LMG 6896	Streptococcus	thermophilus
Z0024_LB_E11_2_B01_A	138	126	LMG 6896	Streptococcus	thermophilus
Z0024_LB_E12_2_B01_B	138	126	LMG 6896	Streptococcus	thermophilus
Z0024_LB_F01_1_B06_A	138	126	LMG 6896	Streptococcus	thermophilus
Z0024_LB_F02_1_B06_B	138	126	LMG 6896	Streptococcus	thermophilus
Z0024_LB_F03_1_B05_A	138	126	LMG 6896	Streptococcus	thermophilus
Z0024_LB_F04_1_B05_B	138	126	LMG 6896	Streptococcus	thermophilus
Z0024_LB_F05_1_B04_A	138	126	LMG 6896	Streptococcus	thermophilus
Z0024_LB_F06_1_B04_B	138	126	LMG 6896	Streptococcus	thermophilus
Z0024_LB_F07_1_B03_A	138	126	LMG 6896	Streptococcus	thermophilus
Z0024_RB_E07_2_B09_A	138	126	LMG 6896	Streptococcus	thermophilus
Z0024_RB_E08_2_B09_B	138	126	LMG 6896	Streptococcus	thermophilus
Z0024_RB_E09_2_B08_A	138	126	LMG 6896	Streptococcus	thermophilus
Z0024_RB_E10_2_B08_B	138	126	LMG 6896	Streptococcus	thermophilus
Z0024_RB_E11_2_B07_A	138	126	LMG 6896	Streptococcus	thermophilus
Z0024_RB_E12_2_B07_B	138	126	LMG 6896	Streptococcus	thermophilus
Z0024_RB_F01_1_B12_A	138	126	LMG 6896	Streptococcus	thermophilus
Z0024_RB_F02_1_B12_B	138	126	LMG 6896	Streptococcus	thermophilus
Z0024_RB_F03_1_B11_A	138	126	LMG 6896	Streptococcus	thermophilus
Z0024_RB_F04_1_B11_B	138	126	LMG 6896	Streptococcus	thermophilus
Z0024_RB_F05_1_B10_A	138	126	LMG 6896	Streptococcus	thermophilus
Z0024_RB_F06_1_B10_B	138	126	LMG 6896	Streptococcus	thermophilus
Z0024_RB_F07_1_B09_A	138	126	LMG 6896	Streptococcus	thermophilus

Z0024_RB_F08_1_B09_B	138	126	LMG 6896	Streptococcus	thermophilus	
Z0024_LB_F08_1_B03_B	139	127	LMG 6901	Lactobacillus	delbrueckii	bulgaricus
Z0024_LB_F09_1_B02_A	139	127	LMG 6901	Lactobacillus	delbrueckii	bulgaricus
Z0024_LB_F10_1_B02_B	139	127	LMG 6901	Lactobacillus	delbrueckii	bulgaricus
Z0024_LB_F11_1_B01_A	139	127	LMG 6901	Lactobacillus	delbrueckii	bulgaricus
Z0024_LB_F12_1_B01_B	139	127	LMG 6901	Lactobacillus	delbrueckii	bulgaricus
Z0024_LB_G01_2_A06_A	139	127	LMG 6901	Lactobacillus	delbrueckii	bulgaricus
Z0024_LB_G02_2_A06_B	139	127	LMG 6901	Lactobacillus	delbrueckii	bulgaricus
Z0024_LB_G03_2_A05_A	139	127	LMG 6901	Lactobacillus	delbrueckii	bulgaricus
Z0024_LB_G04_2_A05_B	139	127	LMG 6901	Lactobacillus	delbrueckii	bulgaricus
Z0024_LB_G05_2_A04_A	139	127	LMG 6901	Lactobacillus	delbrueckii	bulgaricus
Z0024_LB_G06_2_A04_B	139	127	LMG 6901	Lactobacillus	delbrueckii	bulgaricus
Z0024_LB_G07_2_A03_A	139	127	LMG 6901	Lactobacillus	delbrueckii	bulgaricus
Z0024_LB_G08_2_A03_B	139	127	LMG 6901	Lactobacillus	delbrueckii	bulgaricus
Z0024_LB_G09_2_A02_A	139	127	LMG 6901	Lactobacillus	delbrueckii	bulgaricus
Z0024_LB_G10_2_A02_B	139	127	LMG 6901	Lactobacillus	delbrueckii	bulgaricus
Z0024_LB_G11_2_A01_A	139	127	LMG 6901	Lactobacillus	delbrueckii	bulgaricus
Z0024_LB_G12_2_A01_B	139	127	LMG 6901	Lactobacillus	delbrueckii	bulgaricus
Z0024_LB_H01_1_A06_A	139	127	LMG 6901	Lactobacillus	delbrueckii	bulgaricus
Z0024_LB_H02_1_A06_B	139	127	LMG 6901	Lactobacillus	delbrueckii	bulgaricus
Z0024_LB_H03_1_A05_A	139	127	LMG 6901	Lactobacillus	delbrueckii	bulgaricus
Z0024_LB_H04_1_A05_B	139	127	LMG 6901	Lactobacillus	delbrueckii	bulgaricus
Z0024_RB_F09_1_B08_A	139	127	LMG 6901	Lactobacillus	delbrueckii	bulgaricus
Z0024_RB_F10_1_B08_B	139	127	LMG 6901	Lactobacillus	delbrueckii	bulgaricus
Z0024_RB_F11_1_B07_A	139	127	LMG 6901	Lactobacillus	delbrueckii	bulgaricus
Z0024_RB_F12_1_B07_B	139	127	LMG 6901	Lactobacillus	delbrueckii	bulgaricus
Z0024_RB_G01_2_A12_A	139	127	LMG 6901	Lactobacillus	delbrueckii	bulgaricus
Z0024_RB_G02_2_A12_B	139	127	LMG 6901	Lactobacillus	delbrueckii	bulgaricus
Z0024_RB_G03_2_A11_A	139	127	LMG 6901	Lactobacillus	delbrueckii	bulgaricus
Z0024_RB_G04_2_A11_B	139	127	LMG 6901	Lactobacillus	delbrueckii	bulgaricus
Z0024_RB_G05_2_A10_A	139	127	LMG 6901	Lactobacillus	delbrueckii	bulgaricus
Z0024_RB_G06_2_A10_B	139	127	LMG 6901	Lactobacillus	delbrueckii	bulgaricus
Z0024_RB_G07_2_A09_A	139	127	LMG 6901	Lactobacillus	delbrueckii	bulgaricus
Z0024_LB_H05_1_A04_A	140	4	LMG 6907	Lactobacillus	plantarum	plantarum
Z0024_LB_H06_1_A04_B	140	4	LMG 6907	Lactobacillus	plantarum	plantarum
Z0024_LB_H07_1_A03_A	140	4	LMG 6907	Lactobacillus	plantarum	plantarum
Z0024_LB_H08_1_A03_B	140	4	LMG 6907	Lactobacillus	plantarum	plantarum
Z0024_LB_H09_1_A02_A	140	4	LMG 6907	Lactobacillus	plantarum	plantarum
Z0024_LB_H10_1_A02_B	140	4	LMG 6907	Lactobacillus	plantarum	plantarum
Z0024_LB_H11_1_A01_A	140	4	LMG 6907	Lactobacillus	plantarum	plantarum
Z0024_LB_H12_1_A01_B	140	4	LMG 6907	Lactobacillus	plantarum	plantarum
Z0024_LO_A01_1_E01_A	140	4	LMG 6907	Lactobacillus	plantarum	plantarum
Z0024_LO_A02_1_E01_B	140	4	LMG 6907	Lactobacillus	plantarum	plantarum
Z0024_LO_A03_1_E02_A	140	4	LMG 6907	Lactobacillus	plantarum	plantarum
Z0024_LO_A04_1_E02_B	140	4	LMG 6907	Lactobacillus	plantarum	plantarum
Z0024_LO_A05_1_E03_A	140	4	LMG 6907	Lactobacillus	plantarum	plantarum
Z0024_LO_A06_1_E03_B	140	4	LMG 6907	Lactobacillus	plantarum	plantarum
Z0024_LO_A07_1_E04_A	140	4	LMG 6907	Lactobacillus	plantarum	plantarum
Z0024_LO_A08_1_E04_B	140	4	LMG 6907	Lactobacillus	plantarum	plantarum
Z0024_LO_A09_1_E05_A	140	4	LMG 6907	Lactobacillus	plantarum	plantarum

Z0024_LO_A10_1_E05_B	140	4	LMG 6907	Lactobacillus	plantarum	plantarum
Z0024_LO_A11_1_E06_A	140	4	LMG 6907	Lactobacillus	plantarum	plantarum
Z0024_RB_G08_2_A09_B	140	4	LMG 6907	Lactobacillus	plantarum	plantarum
Z0024_RB_G09_2_A08_A	140	4	LMG 6907	Lactobacillus	plantarum	plantarum
Z0024_RB_G10_2_A08_B	140	4	LMG 6907	Lactobacillus	plantarum	plantarum
Z0024_RB_G11_2_A07_A	140	4	LMG 6907	Lactobacillus	plantarum	plantarum
Z0024_RB_G12_2_A07_B	140	4	LMG 6907	Lactobacillus	plantarum	plantarum
Z0024_RB_H01_1_A12_A	140	4	LMG 6907	Lactobacillus	plantarum	plantarum
Z0024_RB_H02_1_A12_B	140	4	LMG 6907	Lactobacillus	plantarum	plantarum
Z0024_RB_H03_1_A11_A	140	4	LMG 6907	Lactobacillus	plantarum	plantarum
Z0024_RB_H04_1_A11_B	140	4	LMG 6907	Lactobacillus	plantarum	plantarum
Z0024_RB_H05_1_A10_A	140	4	LMG 6907	Lactobacillus	plantarum	plantarum
Z0024_RB_H06_1_A10_B	140	4	LMG 6907	Lactobacillus	plantarum	plantarum
Z0024_RB_H07_1_A09_A	140	4	LMG 6907	Lactobacillus	plantarum	plantarum
Z0024_RB_H08_1_A09_B	140	4	LMG 6907	Lactobacillus	plantarum	plantarum
Z0024_LO_A12_1_E06_B	141	128	LMG 6909	Leuconostoc	mesenteroides	cremoris
Z0024_LO_B03_2_E02_A	141	128	LMG 6909	Leuconostoc	mesenteroides	cremoris
Z0024_LO_B05_2_E03_A	141	128	LMG 6909	Leuconostoc	mesenteroides	cremoris
Z0024_LO_B06_2_E03_B	141	128	LMG 6909	Leuconostoc	mesenteroides	cremoris
Z0024_LO_B07_2_E04_A	141	128	LMG 6909	Leuconostoc	mesenteroides	cremoris
Z0024_LO_B08_2_E04_B	141	128	LMG 6909	Leuconostoc	mesenteroides	cremoris
Z0024_LO_B09_2_E05_A	141	128	LMG 6909	Leuconostoc	mesenteroides	cremoris
Z0024_LO_B10_2_E05_B	141	128	LMG 6909	Leuconostoc	mesenteroides	cremoris
Z0024_LO_B11_2_E06_A	141	128	LMG 6909	Leuconostoc	mesenteroides	cremoris
Z0024_LO_B12_2_E06_B	141	128	LMG 6909	Leuconostoc	mesenteroides	cremoris
Z0024_LO_C04_1_F02_B	141	128	LMG 6909	Leuconostoc	mesenteroides	cremoris
Z0024_LO_C05_1_F03_A	141	128	LMG 6909	Leuconostoc	mesenteroides	cremoris
Z0024_LO_C06_1_F03_B	141	128	LMG 6909	Leuconostoc	mesenteroides	cremoris
Z0024_RB_H09_1_A08_A	141	128	LMG 6909	Leuconostoc	mesenteroides	cremoris
Z0024_RB_H10_1_A08_B	141	128	LMG 6909	Leuconostoc	mesenteroides	cremoris
Z0024_RB_H11_1_A07_A	141	128	LMG 6909	Leuconostoc	mesenteroides	cremoris
Z0024_RB_H12_1_A07_B	141	128	LMG 6909	Leuconostoc	mesenteroides	cremoris
Z0024_RO_A01_1_E07_A	141	128	LMG 6909	Leuconostoc	mesenteroides	cremoris
Z0024_RO_A02_1_E07_B	141	128	LMG 6909	Leuconostoc	mesenteroides	cremoris
Z0024_RO_A03_1_E08_A	141	128	LMG 6909	Leuconostoc	mesenteroides	cremoris
Z0024_RO_A04_1_E08_B	141	128	LMG 6909	Leuconostoc	mesenteroides	cremoris
Z0024_RO_A05_1_E09_A	141	128	LMG 6909	Leuconostoc	mesenteroides	cremoris
Z0024_RO_A06_1_E09_B	141	128	LMG 6909	Leuconostoc	mesenteroides	cremoris
Z0024_RO_A07_1_E10_A	141	128	LMG 6909	Leuconostoc	mesenteroides	cremoris
Z0024_RO_A08_1_E10_B	141	128	LMG 6909	Leuconostoc	mesenteroides	cremoris
Z0024_RO_A09_1_E11_A	141	128	LMG 6909	Leuconostoc	mesenteroides	cremoris
Z0024_RO_A11_1_E12_A	141	128	LMG 6909	Leuconostoc	mesenteroides	cremoris
Z0024_RO_A12_1_E12_B	141	128	LMG 6909	Leuconostoc	mesenteroides	cremoris
Z0024_LO_B01_2_E01_A	142	133	LMG 7529	Desulfovibrio	desulfuricans	desulfuricans
Z0024_LO_B02_2_E01_B	142	133	LMG 7529	Desulfovibrio	desulfuricans	desulfuricans
Z0024_LO_B04_2_E02_B	142	133	LMG 7529	Desulfovibrio	desulfuricans	desulfuricans
Z0024_LO_C01_1_F01_A	142	133	LMG 7529	Desulfovibrio	desulfuricans	desulfuricans
Z0024_LO_C02_1_F01_B	142	133	LMG 7529	Desulfovibrio	desulfuricans	desulfuricans
Z0024_LO_C03_1_F02_A	142	133	LMG 7529	Desulfovibrio	desulfuricans	desulfuricans
Z0024_LO_C07_1_F04_A	142	133	LMG 7529	Desulfovibrio	desulfuricans	desulfuricans

Z0024_LO_C11_1_F06_A	142	133	LMG 7529	Desulfovibrio	desulfuricans	desulfuricans
Z0024_LO_D02_2_F01_B	142	133	LMG 7529	Desulfovibrio	desulfuricans	desulfuricans
Z0024_LO_D04_2_F02_B	142	133	LMG 7529	Desulfovibrio	desulfuricans	desulfuricans
Z0024_LO_D06_2_F03_B	142	133	LMG 7529	Desulfovibrio	desulfuricans	desulfuricans
Z0024_LO_D09_2_F05_A	142	133	LMG 7529	Desulfovibrio	desulfuricans	desulfuricans
Z0024_RO_A10_1_E11_B	142	133	LMG 7529	Desulfovibrio	desulfuricans	desulfuricans
Z0024_RO_B04_2_E08_B	142	133	LMG 7529	Desulfovibrio	desulfuricans	desulfuricans
Z0024_RO_C04_1_F08_B	142	133	LMG 7529	Desulfovibrio	desulfuricans	desulfuricans
Z0024_RO_C05_1_F09_A	142	133	LMG 7529	Desulfovibrio	desulfuricans	desulfuricans
Z0024_RO_C12_1_F12_B	142	133	LMG 7529	Desulfovibrio	desulfuricans	desulfuricans
Z0024_RO_D01_2_F07_A	142	133	LMG 7529	Desulfovibrio	desulfuricans	desulfuricans
Z0024_RO_D02_2_F07_B	142	133	LMG 7529	Desulfovibrio	desulfuricans	desulfuricans
Z0024_RO_D03_2_F08_A	142	133	LMG 7529	Desulfovibrio	desulfuricans	desulfuricans
Z0024_RO_D04_2_F08_B	142	133	LMG 7529	Desulfovibrio	desulfuricans	desulfuricans
Z0024_RO_D05_2_F09_A	142	133	LMG 7529	Desulfovibrio	desulfuricans	desulfuricans
Z0024_RO_D06_2_F09_B	142	133	LMG 7529	Desulfovibrio	desulfuricans	desulfuricans
Z0024_RO_E03_1_G08_A	142	133	LMG 7529	Desulfovibrio	desulfuricans	desulfuricans
Z0024_RO_E04_1_G08_B	142	133	LMG 7529	Desulfovibrio	desulfuricans	desulfuricans
Z0024_RO_E05_1_G09_A	142	133	LMG 7529	Desulfovibrio	desulfuricans	desulfuricans
Z0024_RO_E06_1_G09_B	142	133	LMG 7529	Desulfovibrio	desulfuricans	desulfuricans
Z0024_RO_E07_1_G10_A	142	133	LMG 7529	Desulfovibrio	desulfuricans	desulfuricans
Z0024_RO_E08_1_G10_B	142	133	LMG 7529	Desulfovibrio	desulfuricans	desulfuricans
Z0024_RO_E09_1_G11_A	142	133	LMG 7529	Desulfovibrio	desulfuricans	desulfuricans
Z0024_RO_E10_1_G11_B	142	133	LMG 7529	Desulfovibrio	desulfuricans	desulfuricans
Z0024_RO_E11_1_G12_A	142	133	LMG 7529	Desulfovibrio	desulfuricans	desulfuricans
Z0024_LO_C08_1_F04_B	143	129	LMG 6928	Sporosarcina	globispora	
Z0024_LO_C10_1_F05_B	143	129	LMG 6928	Sporosarcina	globispora	
Z0024_LO_C12_1_F06_B	143	129	LMG 6928	Sporosarcina	globispora	
Z0024_LO_D01_2_F01_A	143	129	LMG 6928	Sporosarcina	globispora	
Z0024_LO_D03_2_F02_A	143	129	LMG 6928	Sporosarcina	globispora	
Z0024_LO_D05_2_F03_A	143	129	LMG 6928	Sporosarcina	globispora	
Z0024_LO_D07_2_F04_A	143	129	LMG 6928	Sporosarcina	globispora	
Z0024_LO_D10_2_F05_B	143	129	LMG 6928	Sporosarcina	globispora	
Z0024_LO_D12_2_F06_B	143	129	LMG 6928	Sporosarcina	globispora	
Z0024_LO_E01_1_G01_A	143	129	LMG 6928	Sporosarcina	globispora	
Z0024_LO_E02_1_G01_B	143	129	LMG 6928	Sporosarcina	globispora	
Z0024_LO_E04_1_G02_B	143	129	LMG 6928	Sporosarcina	globispora	
Z0024_LO_E06_1_G03_B	143	129	LMG 6928	Sporosarcina	globispora	
Z0024_LO_E09_1_G05_A	143	129	LMG 6928	Sporosarcina	globispora	
Z0024_RO_B01_2_E07_A	143	129	LMG 6928	Sporosarcina	globispora	
Z0024_RO_B02_2_E07_B	143	129	LMG 6928	Sporosarcina	globispora	
Z0024_RO_B03_2_E08_A	143	129	LMG 6928	Sporosarcina	globispora	
Z0024_RO_B05_2_E09_A	143	129	LMG 6928	Sporosarcina	globispora	
Z0024_RO_B06_2_E09_B	143	129	LMG 6928	Sporosarcina	globispora	
Z0024_RO_B08_2_E10_B	143	129	LMG 6928	Sporosarcina	globispora	
Z0024_RO_B10_2_E11_B	143	129	LMG 6928	Sporosarcina	globispora	
Z0024_RO_B12_2_E12_B	143	129	LMG 6928	Sporosarcina	globispora	
Z0024_RO_C01_1_F07_A	143	129	LMG 6928	Sporosarcina	globispora	
Z0024_RO_C03_1_F08_A	143	129	LMG 6928	Sporosarcina	globispora	
Z0024_RO_C06_1_F09_B	143	129	LMG 6928	Sporosarcina	globispora	

Z0024_RO_C08_1_F10_B	143	129	LMG 6928	Sporosarcina	globispora	
Z0024_RO_C10_1_F11_B	143	129	LMG 6928	Sporosarcina	globispora	
Z0024_RO_C11_1_F12_A	143	129	LMG 6928	Sporosarcina	globispora	
Z0024_LO_D08_2_F04_B	144	132	LMG 7233	Salmonella	enterica	enterica
Z0024_LO_D11_2_F06_A	144	132	LMG 7233	Salmonella	enterica	enterica
Z0024_LO_E03_1_G02_A	144	132	LMG 7233	Salmonella	enterica	enterica
Z0024_LO_E05_1_G03_A	144	132	LMG 7233	Salmonella	enterica	enterica
Z0024_LO_E07_1_G04_A	144	132	LMG 7233	Salmonella	enterica	enterica
Z0024_LO_E08_1_G04_B	144	132	LMG 7233	Salmonella	enterica	enterica
Z0024_LO_F05_2_G03_A	144	132	LMG 7233	Salmonella	enterica	enterica
Z0024_LO_F06_2_G03_B	144	132	LMG 7233	Salmonella	enterica	enterica
Z0024_LO_G09_1_H05_A	144	132	LMG 7233	Salmonella	enterica	enterica
Z0024_LO_G10_1_H05_B	144	132	LMG 7233	Salmonella	enterica	enterica
Z0024_LO_G11_1_H06_A	144	132	LMG 7233	Salmonella	enterica	enterica
Z0024_LO_G12_1_H06_B	144	132	LMG 7233	Salmonella	enterica	enterica
Z0024_LO_H02_2_H01_B	144	132	LMG 7233	Salmonella	enterica	enterica
Z0024_LO_H04_2_H02_B	144	132	LMG 7233	Salmonella	enterica	enterica
Z0024_LO_H05_2_H03_A	144	132	LMG 7233	Salmonella	enterica	enterica
Z0024_LO_H10_2_H05_B	144	132	LMG 7233	Salmonella	enterica	enterica
Z0024_LO_H11_2_H06_A	144	132	LMG 7233	Salmonella	enterica	enterica
Z0024_RO_D09_2_F11_A	144	132	LMG 7233	Salmonella	enterica	enterica
Z0024_RO_D10_2_F11_B	144	132	LMG 7233	Salmonella	enterica	enterica
Z0024_RO_D11_2_F12_A	144	132	LMG 7233	Salmonella	enterica	enterica
Z0024_RO_D12_2_F12_B	144	132	LMG 7233	Salmonella	enterica	enterica
Z0024_RO_E01_1_G07_A	144	132	LMG 7233	Salmonella	enterica	enterica
Z0024_RO_E02_1_G07_B	144	132	LMG 7233	Salmonella	enterica	enterica
Z0024_RO_F03_2_G08_A	144	132	LMG 7233	Salmonella	enterica	enterica
Z0024_RO_F05_2_G09_A	144	132	LMG 7233	Salmonella	enterica	enterica
Z0024_RO_F08_2_G10_B	144	132	LMG 7233	Salmonella	enterica	enterica
Z0024_RO_F09_2_G11_A	144	132	LMG 7233	Salmonella	enterica	enterica
Z0024_RO_F10_2_G11_B	144	132	LMG 7233	Salmonella	enterica	enterica
Z0024_RO_F11_2_G12_A	144	132	LMG 7233	Salmonella	enterica	enterica
Z0024_RO_G02_1_H07_B	144	132	LMG 7233	Salmonella	enterica	enterica
Z0024_RO_G05_1_H09_A	144	132	LMG 7233	Salmonella	enterica	enterica
Z0024_RO_G09_1_H11_A	144	132	LMG 7233	Salmonella	enterica	enterica
Z0024_LO_E10_1_G05_B	145	130	LMG 7123	Brevibacillus	brevis	
Z0024_LO_E11_1_G06_A	145	130	LMG 7123	Brevibacillus	brevis	
Z0024_LO_E12_1_G06_B	145	130	LMG 7123	Brevibacillus	brevis	
Z0024_LO_F01_2_G01_A	145	130	LMG 7123	Brevibacillus	brevis	
Z0024_LO_F02_2_G01_B	145	130	LMG 7123	Brevibacillus	brevis	
Z0024_LO_F03_2_G02_A	145	130	LMG 7123	Brevibacillus	brevis	
Z0024_LO_F04_2_G02_B	145	130	LMG 7123	Brevibacillus	brevis	
Z0024_LO_F07_2_G04_A	145	130	LMG 7123	Brevibacillus	brevis	
Z0024_LO_F08_2_G04_B	145	130	LMG 7123	Brevibacillus	brevis	
Z0024_LO_F09_2_G05_A	145	130	LMG 7123	Brevibacillus	brevis	
Z0024_LO_F10_2_G05_B	145	130	LMG 7123	Brevibacillus	brevis	
Z0024_LO_F11_2_G06_A	145	130	LMG 7123	Brevibacillus	brevis	
Z0024_LO_F12_2_G06_B	145	130	LMG 7123	Brevibacillus	brevis	
Z0024_LO_G01_1_H01_A	145	130	LMG 7123	Brevibacillus	brevis	
Z0024_LO_G02_1_H01_B	145	130	LMG 7123	Brevibacillus	brevis	

Z0024_LO_G03_1_H02_A	145	130	LMG 7123	Brevibacillus	brevis	
Z0024_LO_G04_1_H02_B	145	130	LMG 7123	Brevibacillus	brevis	
Z0024_LO_G05_1_H03_A	145	130	LMG 7123	Brevibacillus	brevis	
Z0024_LO_G06_1_H03_B	145	130	LMG 7123	Brevibacillus	brevis	
Z0024_LO_G07_1_H04_A	145	130	LMG 7123	Brevibacillus	brevis	
Z0024_LO_G08_1_H04_B	145	130	LMG 7123	Brevibacillus	brevis	
Z0024_LO_H01_2_H01_A	145	130	LMG 7123	Brevibacillus	brevis	
Z0024_LO_H03_2_H02_A	145	130	LMG 7123	Brevibacillus	brevis	
Z0024_LO_H06_2_H03_B	145	130	LMG 7123	Brevibacillus	brevis	
Z0024_RO_D07_2_F10_A	145	130	LMG 7123	Brevibacillus	brevis	
Z0024_RO_D08_2_F10_B	145	130	LMG 7123	Brevibacillus	brevis	
Z0024_RO_E12_1_G12_B	145	130	LMG 7123	Brevibacillus	brevis	
Z0024_RO_F01_2_G07_A	145	130	LMG 7123	Brevibacillus	brevis	
Z0024_RO_F02_2_G07_B	145	130	LMG 7123	Brevibacillus	brevis	
Z0024_RO_F04_2_G08_B	145	130	LMG 7123	Brevibacillus	brevis	
Z0024_RO_F06_2_G09_B	145	130	LMG 7123	Brevibacillus	brevis	
Z0024_RO_F07_2_G10_A	145	130	LMG 7123	Brevibacillus	brevis	
Z0024_LO_H07_2_H04_A	146	131	LMG 7135	Bacillus	subtilis	subtilis
Z0024_LO_H08_2_H04_B	146	131	LMG 7135	Bacillus	subtilis	subtilis
Z0024_LO_H09_2_H05_A	146	131	LMG 7135	Bacillus	subtilis	subtilis
Z0024_LO_H12_2_H06_B	146	131	LMG 7135	Bacillus	subtilis	subtilis
Z0024_RB_A01_2_D12_A	146	131	LMG 7135	Bacillus	subtilis	subtilis
Z0024_RB_A02_2_D12_B	146	131	LMG 7135	Bacillus	subtilis	subtilis
Z0024_RB_A03_2_D11_A	146	131	LMG 7135	Bacillus	subtilis	subtilis
Z0024_RB_A04_2_D11_B	146	131	LMG 7135	Bacillus	subtilis	subtilis
Z0024_RB_A05_2_D10_A	146	131	LMG 7135	Bacillus	subtilis	subtilis
Z0024_RB_A06_2_D10_B	146	131	LMG 7135	Bacillus	subtilis	subtilis
Z0024_RB_A07_2_D09_A	146	131	LMG 7135	Bacillus	subtilis	subtilis
Z0024_RB_A08_2_D09_B	146	131	LMG 7135	Bacillus	subtilis	subtilis
Z0024_RB_A09_2_D08_A	146	131	LMG 7135	Bacillus	subtilis	subtilis
Z0024_RB_A10_2_D08_B	146	131	LMG 7135	Bacillus	subtilis	subtilis
Z0024_RB_A11_2_D07_A	146	131	LMG 7135	Bacillus	subtilis	subtilis
Z0024_RB_A12_2_D07_B	146	131	LMG 7135	Bacillus	subtilis	subtilis
Z0024_RB_B01_1_D12_A	146	131	LMG 7135	Bacillus	subtilis	subtilis
Z0024_RB_B02_1_D12_B	146	131	LMG 7135	Bacillus	subtilis	subtilis
Z0024_RO_F12_2_G12_B	146	131	LMG 7135	Bacillus	subtilis	subtilis
Z0024_RO_G01_1_H07_A	146	131	LMG 7135	Bacillus	subtilis	subtilis
Z0024_RO_G03_1_H08_A	146	131	LMG 7135	Bacillus	subtilis	subtilis
Z0024_RO_G04_1_H08_B	146	131	LMG 7135	Bacillus	subtilis	subtilis
Z0024_RO_G06_1_H09_B	146	131	LMG 7135	Bacillus	subtilis	subtilis
Z0024_RO_G07_1_H10_A	146	131	LMG 7135	Bacillus	subtilis	subtilis
Z0024_RO_G08_1_H10_B	146	131	LMG 7135	Bacillus	subtilis	subtilis
Z0024_RO_G10_1_H11_B	146	131	LMG 7135	Bacillus	subtilis	subtilis
Z0024_RO_G11_1_H12_A	146	131	LMG 7135	Bacillus	subtilis	subtilis
Z0024_RO_G12_1_H12_B	146	131	LMG 7135	Bacillus	subtilis	subtilis
Z0024_RO_H01_2_H07_A	146	131	LMG 7135	Bacillus	subtilis	subtilis
Z0024_RO_H02_2_H07_B	146	131	LMG 7135	Bacillus	subtilis	subtilis
Z0024_RO_H03_2_H08_A	146	131	LMG 7135	Bacillus	subtilis	subtilis
Z0024_RO_H04_2_H08_B	146	131	LMG 7135	Bacillus	subtilis	subtilis
Z0025_LB_B08_1_D03_B	147	134	LMG 7874	Morganella	morganii	morganii

Z0025_LB_B09_1_D02_A	147	134	LMG 7874	Morganella	morganii	morganii
Z0025_LB_B10_1_D02_B	147	134	LMG 7874	Morganella	morganii	morganii
Z0025_LB_B11_1_D01_A	147	134	LMG 7874	Morganella	morganii	morganii
Z0025_LB_B12_1_D01_B	147	134	LMG 7874	Morganella	morganii	morganii
Z0025_LB_C01_2_C06_A	147	134	LMG 7874	Morganella	morganii	morganii
Z0025_LB_C02_2_C06_B	147	134	LMG 7874	Morganella	morganii	morganii
Z0025_LB_C03_2_C05_A	147	134	LMG 7874	Morganella	morganii	morganii
Z0025_LB_C04_2_C05_B	147	134	LMG 7874	Morganella	morganii	morganii
Z0025_LB_C06_2_C04_B	147	134	LMG 7874	Morganella	morganii	morganii
Z0025_LB_C07_2_C03_A	147	134	LMG 7874	Morganella	morganii	morganii
Z0025_LB_C08_2_C03_B	147	134	LMG 7874	Morganella	morganii	morganii
Z0025_LB_C09_2_C02_A	147	134	LMG 7874	Morganella	morganii	morganii
Z0025_LB_C10_2_C02_B	147	134	LMG 7874	Morganella	morganii	morganii
Z0025_LB_C11_2_C01_A	147	134	LMG 7874	Morganella	morganii	morganii
Z0025_RB_D08_1_C09_B	147	134	LMG 7874	Morganella	morganii	morganii
Z0025_RB_D09_1_C08_A	147	134	LMG 7874	Morganella	morganii	morganii
Z0025_RB_D10_1_C08_B	147	134	LMG 7874	Morganella	morganii	morganii
Z0025_RB_D11_1_C07_A	147	134	LMG 7874	Morganella	morganii	morganii
Z0025_RB_D12_1_C07_B	147	134	LMG 7874	Morganella	morganii	morganii
Z0025_RB_E01_2_B12_A	147	134	LMG 7874	Morganella	morganii	morganii
Z0025_RB_E02_2_B12_B	147	134	LMG 7874	Morganella	morganii	morganii
Z0025_RB_E03_2_B11_A	147	134	LMG 7874	Morganella	morganii	morganii
Z0025_RB_E04_2_B11_B	147	134	LMG 7874	Morganella	morganii	morganii
Z0025_RB_E05_2_B10_A	147	134	LMG 7874	Morganella	morganii	morganii
Z0025_RB_E06_2_B10_B	147	134	LMG 7874	Morganella	morganii	morganii
Z0025_RB_E07_2_B09_A	147	134	LMG 7874	Morganella	morganii	morganii
Z0025_RB_E08_2_B09_B	147	134	LMG 7874	Morganella	morganii	morganii
Z0025_RB_E09_2_B08_A	147	134	LMG 7874	Morganella	morganii	morganii
Z0025_RB_E10_2_B08_B	147	134	LMG 7874	Morganella	morganii	morganii
Z0025_RB_E11_2_B07_A	147	134	LMG 7874	Morganella	morganii	morganii
Z0025_RB_E12_2_B07_B	147	134	LMG 7874	Morganella	morganii	morganii
Z0025_LB_C05_2_C04_A	148	135	LMG 7881	Serratia	ficaria	
Z0025_LB_C12_2_C01_B	148	135	LMG 7881	Serratia	ficaria	
Z0025_LB_D01_1_C06_A	148	135	LMG 7881	Serratia	ficaria	
Z0025_LB_D02_1_C06_B	148	135	LMG 7881	Serratia	ficaria	
Z0025_LB_D03_1_C05_A	148	135	LMG 7881	Serratia	ficaria	
Z0025_LB_D04_1_C05_B	148	135	LMG 7881	Serratia	ficaria	
Z0025_LB_D05_1_C04_A	148	135	LMG 7881	Serratia	ficaria	
Z0025_LB_D06_1_C04_B	148	135	LMG 7881	Serratia	ficaria	
Z0025_LB_D07_1_C03_A	148	135	LMG 7881	Serratia	ficaria	
Z0025_LB_D08_1_C03_B	148	135	LMG 7881	Serratia	ficaria	
Z0025_LB_D09_1_C02_A	148	135	LMG 7881	Serratia	ficaria	
Z0025_LB_D10_1_C02_B	148	135	LMG 7881	Serratia	ficaria	
Z0025_LB_D11_1_C01_A	148	135	LMG 7881	Serratia	ficaria	
Z0025_LB_D12_1_C01_B	148	135	LMG 7881	Serratia	ficaria	
Z0025_LB_E01_2_B06_A	148	135	LMG 7881	Serratia	ficaria	
Z0025_LB_E02_2_B06_B	148	135	LMG 7881	Serratia	ficaria	
Z0025_LB_E03_2_B05_A	148	135	LMG 7881	Serratia	ficaria	
Z0025_LB_E04_2_B05_B	148	135	LMG 7881	Serratia	ficaria	
Z0025_LB_E05_2_B04_A	148	135	LMG 7881	Serratia	ficaria	

Z0025_LB_E06_2_B04_B	148	135	LMG 7881	Serratia	ficaria	
Z0025_LB_E07_2_B03_A	148	135	LMG 7881	Serratia	ficaria	
Z0025_LB_E08_2_B03_B	148	135	LMG 7881	Serratia	ficaria	
Z0025_LB_E09_2_B02_A	148	135	LMG 7881	Serratia	ficaria	
Z0025_RB_F01_1_B12_A	148	135	LMG 7881	Serratia	ficaria	
Z0025_RB_F02_1_B12_B	148	135	LMG 7881	Serratia	ficaria	
Z0025_RB_F03_1_B11_A	148	135	LMG 7881	Serratia	ficaria	
Z0025_RB_F04_1_B11_B	148	135	LMG 7881	Serratia	ficaria	
Z0025_RB_F05_1_B10_A	148	135	LMG 7881	Serratia	ficaria	
Z0025_RB_F06_1_B10_B	148	135	LMG 7881	Serratia	ficaria	
Z0025_RB_F07_1_B09_A	148	135	LMG 7881	Serratia	ficaria	
Z0025_RB_F08_1_B09_B	148	135	LMG 7881	Serratia	ficaria	
Z0025_RB_F09_1_B08_A	148	135	LMG 7881	Serratia	ficaria	
Z0025_LB_E10_2_B02_B	149	136	LMG 7899	Yersinia	enterocolitica	enterocolitica
Z0025_LB_E11_2_B01_A	149	136	LMG 7899	Yersinia	enterocolitica	enterocolitica
Z0025_LB_E12_2_B01_B	149	136	LMG 7899	Yersinia	enterocolitica	enterocolitica
Z0025_LB_F01_1_B06_A	149	136	LMG 7899	Yersinia	enterocolitica	enterocolitica
Z0025_LB_F02_1_B06_B	149	136	LMG 7899	Yersinia	enterocolitica	enterocolitica
Z0025_LB_F03_1_B05_A	149	136	LMG 7899	Yersinia	enterocolitica	enterocolitica
Z0025_LB_F04_1_B05_B	149	136	LMG 7899	Yersinia	enterocolitica	enterocolitica
Z0025_LB_F05_1_B04_A	149	136	LMG 7899	Yersinia	enterocolitica	enterocolitica
Z0025_LB_F06_1_B04_B	149	136	LMG 7899	Yersinia	enterocolitica	enterocolitica
Z0025_LB_F07_1_B03_A	149	136	LMG 7899	Yersinia	enterocolitica	enterocolitica
Z0025_LB_F08_1_B03_B	149	136	LMG 7899	Yersinia	enterocolitica	enterocolitica
Z0025_LB_F09_1_B02_A	149	136	LMG 7899	Yersinia	enterocolitica	enterocolitica
Z0025_LB_F10_1_B02_B	149	136	LMG 7899	Yersinia	enterocolitica	enterocolitica
Z0025_LB_F11_1_B01_A	149	136	LMG 7899	Yersinia	enterocolitica	enterocolitica
Z0025_LB_F12_1_B01_B	149	136	LMG 7899	Yersinia	enterocolitica	enterocolitica
Z0025_LB_G01_2_A06_A	149	136	LMG 7899	Yersinia	enterocolitica	enterocolitica
Z0025_LB_G02_2_A06_B	149	136	LMG 7899	Yersinia	enterocolitica	enterocolitica
Z0025_LB_G03_2_A05_A	149	136	LMG 7899	Yersinia	enterocolitica	enterocolitica
Z0025_LB_G04_2_A05_B	149	136	LMG 7899	Yersinia	enterocolitica	enterocolitica
Z0025_LB_G05_2_A04_A	149	136	LMG 7899	Yersinia	enterocolitica	enterocolitica
Z0025_LB_G06_2_A04_B	149	136	LMG 7899	Yersinia	enterocolitica	enterocolitica
Z0025_LB_G07_2_A03_A	149	136	LMG 7899	Yersinia	enterocolitica	enterocolitica
Z0025_RB_F10_1_B08_B	149	136	LMG 7899	Yersinia	enterocolitica	enterocolitica
Z0025_RB_F11_1_B07_A	149	136	LMG 7899	Yersinia	enterocolitica	enterocolitica
Z0025_RB_F12_1_B07_B	149	136	LMG 7899	Yersinia	enterocolitica	enterocolitica
Z0025_RB_G01_2_A12_A	149	136	LMG 7899	Yersinia	enterocolitica	enterocolitica
Z0025_RB_G02_2_A12_B	149	136	LMG 7899	Yersinia	enterocolitica	enterocolitica
Z0025_RB_G03_2_A11_A	149	136	LMG 7899	Yersinia	enterocolitica	enterocolitica
Z0025_RB_G04_2_A11_B	149	136	LMG 7899	Yersinia	enterocolitica	enterocolitica
Z0025_RB_G05_2_A10_A	149	136	LMG 7899	Yersinia	enterocolitica	enterocolitica
Z0025_RB_G06_2_A10_B	149	136	LMG 7899	Yersinia	enterocolitica	enterocolitica
Z0025_RB_G07_2_A09_A	149	136	LMG 7899	Yersinia	enterocolitica	enterocolitica
Z0025_LB_G08_2_A03_B	150	137	LMG 8760	Lysobacter	antibioticus	
Z0025_LB_G09_2_A02_A	150	137	LMG 8760	Lysobacter	antibioticus	
Z0025_LB_G10_2_A02_B	150	137	LMG 8760	Lysobacter	antibioticus	
Z0025_LB_G11_2_A01_A	150	137	LMG 8760	Lysobacter	antibioticus	
Z0025_LB_G12_2_A01_B	150	137	LMG 8760	Lysobacter	antibioticus	

Z0025_LB_H01_1_A06_A	150	137	LMG 8760	Lysobacter	antibioticus
Z0025_LB_H02_1_A06_B	150	137	LMG 8760	Lysobacter	antibioticus
Z0025_LB_H03_1_A05_A	150	137	LMG 8760	Lysobacter	antibioticus
Z0025_LB_H04_1_A05_B	150	137	LMG 8760	Lysobacter	antibioticus
Z0025_LB_H05_1_A04_A	150	137	LMG 8760	Lysobacter	antibioticus
Z0025_LB_H06_1_A04_B	150	137	LMG 8760	Lysobacter	antibioticus
Z0025_LB_H07_1_A03_A	150	137	LMG 8760	Lysobacter	antibioticus
Z0025_LB_H08_1_A03_B	150	137	LMG 8760	Lysobacter	antibioticus
Z0025_LB_H09_1_A02_A	150	137	LMG 8760	Lysobacter	antibioticus
Z0025_LB_H11_1_A01_A	150	137	LMG 8760	Lysobacter	antibioticus
Z0025_RB_G08_2_A09_B	150	137	LMG 8760	Lysobacter	antibioticus
Z0025_RB_G09_2_A08_A	150	137	LMG 8760	Lysobacter	antibioticus
Z0025_RB_G10_2_A08_B	150	137	LMG 8760	Lysobacter	antibioticus
Z0025_RB_G11_2_A07_A	150	137	LMG 8760	Lysobacter	antibioticus
Z0025_RB_G12_2_A07_B	150	137	LMG 8760	Lysobacter	antibioticus
Z0025_RB_H01_1_A12_A	150	137	LMG 8760	Lysobacter	antibioticus
Z0025_RB_H02_1_A12_B	150	137	LMG 8760	Lysobacter	antibioticus
Z0025_RB_H03_1_A11_A	150	137	LMG 8760	Lysobacter	antibioticus
Z0025_RB_H04_1_A11_B	150	137	LMG 8760	Lysobacter	antibioticus
Z0025_RB_H05_1_A10_A	150	137	LMG 8760	Lysobacter	antibioticus
Z0025_RB_H06_1_A10_B	150	137	LMG 8760	Lysobacter	antibioticus
Z0025_RB_H07_1_A09_A	150	137	LMG 8760	Lysobacter	antibioticus
Z0025_RB_H08_1_A09_B	150	137	LMG 8760	Lysobacter	antibioticus
Z0025_RB_H09_1_A08_A	150	137	LMG 8760	Lysobacter	antibioticus
Z0025_RB_H10_1_A08_B	150	137	LMG 8760	Lysobacter	antibioticus
Z0025_RB_H11_1_A07_A	150	137	LMG 8760	Lysobacter	antibioticus
Z0025_RB_H12_1_A07_B	150	137	LMG 8760	Lysobacter	antibioticus
Z0025_LO_A01_1_E01_A	151	138	LMG 8787	Curtobacterium	luteum
Z0025_LO_A03_1_E02_A	151	138	LMG 8787	Curtobacterium	luteum
Z0025_LO_A04_1_E02_B	151	138	LMG 8787	Curtobacterium	luteum
Z0025_LO_A07_1_E04_A	151	138	LMG 8787	Curtobacterium	luteum
Z0025_LO_B01_2_E01_A	151	138	LMG 8787	Curtobacterium	luteum
Z0025_LO_B03_2_E02_A	151	138	LMG 8787	Curtobacterium	luteum
Z0025_LO_B06_2_E03_B	151	138	LMG 8787	Curtobacterium	luteum
Z0025_LO_B07_2_E04_A	151	138	LMG 8787	Curtobacterium	luteum
Z0025_LO_B08_2_E04_B	151	138	LMG 8787	Curtobacterium	luteum
Z0025_LO_B09_2_E05_A	151	138	LMG 8787	Curtobacterium	luteum
Z0025_LO_B10_2_E05_B	151	138	LMG 8787	Curtobacterium	luteum
Z0025_LO_B11_2_E06_A	151	138	LMG 8787	Curtobacterium	luteum
Z0025_RO_A01_1_E07_A	151	138	LMG 8787	Curtobacterium	luteum
Z0025_RO_A02_1_E07_B	151	138	LMG 8787	Curtobacterium	luteum
Z0025_RO_A03_1_E08_A	151	138	LMG 8787	Curtobacterium	luteum
Z0025_RO_A04_1_E08_B	151	138	LMG 8787	Curtobacterium	luteum
Z0025_RO_A05_1_E09_A	151	138	LMG 8787	Curtobacterium	luteum
Z0025_RO_A06_1_E09_B	151	138	LMG 8787	Curtobacterium	luteum
Z0025_RO_A07_1_E10_A	151	138	LMG 8787	Curtobacterium	luteum
Z0025_RO_A08_1_E10_B	151	138	LMG 8787	Curtobacterium	luteum
Z0025_RO_A09_1_E11_A	151	138	LMG 8787	Curtobacterium	luteum
Z0025_RO_A10_1_E11_B	151	138	LMG 8787	Curtobacterium	luteum
Z0025_RO_A11_1_E12_A	151	138	LMG 8787	Curtobacterium	luteum

Z0025_RO_A12_1_E12_B	151	138	LMG 8787	Curtobacterium	luteum	
Z0025_RO_B01_2_E07_A	151	138	LMG 8787	Curtobacterium	luteum	
Z0025_RO_B02_2_E07_B	151	138	LMG 8787	Curtobacterium	luteum	
Z0025_RO_B03_2_E08_A	151	138	LMG 8787	Curtobacterium	luteum	
Z0025_RO_B04_2_E08_B	151	138	LMG 8787	Curtobacterium	luteum	
Z0025_LO_C02_1_F01_B	152	139	LMG 9205	Lactobacillus	plantarum	argentoratensis
Z0025_LO_C03_1_F02_A	152	139	LMG 9205	Lactobacillus	plantarum	argentoratensis
Z0025_LO_C05_1_F03_A	152	139	LMG 9205	Lactobacillus	plantarum	argentoratensis
Z0025_LO_C07_1_F04_A	152	139	LMG 9205	Lactobacillus	plantarum	argentoratensis
Z0025_LO_C12_1_F06_B	152	139	LMG 9205	Lactobacillus	plantarum	argentoratensis
Z0025_LO_D02_2_F01_B	152	139	LMG 9205	Lactobacillus	plantarum	argentoratensis
Z0025_LO_D03_2_F02_A	152	139	LMG 9205	Lactobacillus	plantarum	argentoratensis
Z0025_LO_D07_2_F04_A	152	139	LMG 9205	Lactobacillus	plantarum	argentoratensis
Z0025_LO_D09_2_F05_A	152	139	LMG 9205	Lactobacillus	plantarum	argentoratensis
Z0025_LO_D11_2_F06_A	152	139	LMG 9205	Lactobacillus	plantarum	argentoratensis
Z0025_LO_D12_2_F06_B	152	139	LMG 9205	Lactobacillus	plantarum	argentoratensis
Z0025_RO_B05_2_E09_A	152	139	LMG 9205	Lactobacillus	plantarum	argentoratensis
Z0025_RO_B06_2_E09_B	152	139	LMG 9205	Lactobacillus	plantarum	argentoratensis
Z0025_RO_B07_2_E10_A	152	139	LMG 9205	Lactobacillus	plantarum	argentoratensis
Z0025_RO_B08_2_E10_B	152	139	LMG 9205	Lactobacillus	plantarum	argentoratensis
Z0025_RO_B09_2_E11_A	152	139	LMG 9205	Lactobacillus	plantarum	argentoratensis
Z0025_RO_B10_2_E11_B	152	139	LMG 9205	Lactobacillus	plantarum	argentoratensis
Z0025_RO_B11_2_E12_A	152	139	LMG 9205	Lactobacillus	plantarum	argentoratensis
Z0025_RO_B12_2_E12_B	152	139	LMG 9205	Lactobacillus	plantarum	argentoratensis
Z0025_RO_C01_1_F07_A	152	139	LMG 9205	Lactobacillus	plantarum	argentoratensis
Z0025_RO_C02_1_F07_B	152	139	LMG 9205	Lactobacillus	plantarum	argentoratensis
Z0025_RO_C03_1_F08_A	152	139	LMG 9205	Lactobacillus	plantarum	argentoratensis
Z0025_RO_C04_1_F08_B	152	139	LMG 9205	Lactobacillus	plantarum	argentoratensis
Z0025_RO_C06_1_F09_B	152	139	LMG 9205	Lactobacillus	plantarum	argentoratensis
Z0025_RO_C07_1_F10_A	152	139	LMG 9205	Lactobacillus	plantarum	argentoratensis
Z0025_RO_C08_1_F10_B	152	139	LMG 9205	Lactobacillus	plantarum	argentoratensis
Z0025_RO_C09_1_F11_A	152	139	LMG 9205	Lactobacillus	plantarum	argentoratensis
Z0025_RO_C11_1_F12_A	152	139	LMG 9205	Lactobacillus	plantarum	argentoratensis
