# Supplementary material for: Introducing SPeDE: High-Throughput Dereplication and Accurate Determination of Microbial Diversity from Matrix-Assisted Laser Desorption–Ionization Time of Flight Mass Spectrometry Data
Source: mSystems. 2019 Sep 10;4(5):e00437-19. doi: 10.1128/mSystems.00437-19 (PMC6739102; doi:10.1128/mSystems.00437-19)
Supplement: FIG S1 [file mSystems.00437-19-sf001.pdf]

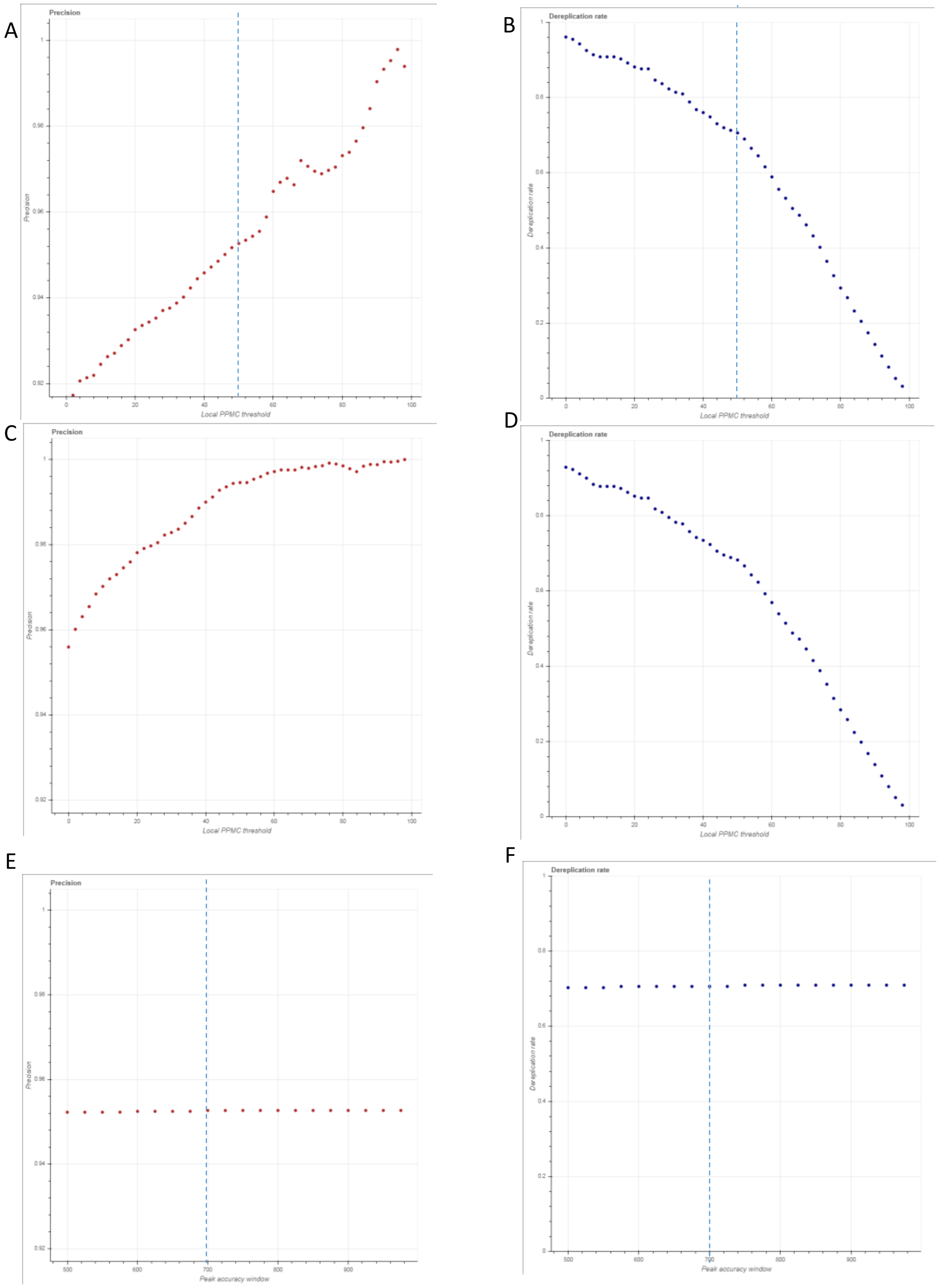
A
Precision
Local PPMC threshold
B
Dereplication rate
Local PPMC threshold
C
Precision
Local PPMC threshold
D
Dereplication rate
Local PPMC threshold
E
Precision
Peak accuracy window
F
Dereplication rate
Peak accuracy window

Figure S1
